# Phenotypic divergence between broiler and layer chicken lines is regulated at the molecular level during development

**DOI:** 10.1186/s12864-024-10083-x

**Published:** 2024-02-12

**Authors:** Renata Erbert Contriciani, Carla Vermeulen Carvalho Grade, Igor Buzzatto-Leite, Fernanda Cristina da Veiga, Mônica Corrêa Ledur, Antonio Reverter, Pamela Almeida Alexandre, Aline Silva Mello Cesar, Luiz Lehmann Coutinho, Lúcia Elvira Alvares

**Affiliations:** 1 Department of Biochemistry and Tissue Biology, Institute of Biology, University of Campinas (UNICAMP), Campinas, Brazil; 2 Instituto Latino-Americano de Ciências da Vida e da Natureza, Universidade Federal da Integração Latino-Americana (UNILA), Foz do Iguaçu, Brazil; 3 Department of Animal Science, Luiz de Queiroz College of Agriculture, University of São Paulo (USP), Piracicaba, Brazil; 4 Embrapa Suínos e Aves, Concórdia, Santa Catarina Brazil; 5 Commonwealth Scientific and Industrial Research Organisation (CSIRO), Agriculture and Food, Brisbane, QLD Australia; 6 Department of Agri-Food Industry, Food and Nutrition, Luiz de Queiroz College of Agriculture, University of São Paulo (USP), Piracicaba, Brazil

**Keywords:** RNA-Seq, Transcriptome, Broiler and layer chicken lines, Differentially expressed genes, Gene ontology, Regulatory network, Breeding programs

## Abstract

**Background:**

Understanding the molecular underpinnings of phenotypic variations is critical for enhancing poultry breeding programs. The Brazilian broiler (TT) and laying hen (CC) lines exhibit striking differences in body weight, growth potential, and muscle mass. Our work aimed to compare the global transcriptome of wing and pectoral tissues during the early development (days 2.5 to 3.5) of these chicken lines, unveiling disparities in gene expression and regulation.

**Results:**

Different and bona-fide transcriptomic profiles were identified for the compared lines. A similar number of up- and downregulated differentially expressed genes (DEGs) were identified, considering the broiler line as a reference. Upregulated DEGs displayed an enrichment of protease-encoding genes, whereas downregulated DEGs exhibited a prevalence of receptors and ligands. Gene Ontology analysis revealed that upregulated DEGs were mainly associated with hormone response, mitotic cell cycle, and different metabolic and biosynthetic processes. In contrast, downregulated DEGs were primarily linked to communication, signal transduction, cell differentiation, and nervous system development. Regulatory networks were constructed for the *mitotic cell cycle* and *cell differentiation* biological processes, as their contrasting roles may impact the development of distinct postnatal traits. Within the mitotic cell cycle network, key upregulated DEGs included *CCND1* and *HSP90*, with central regulators being *NF-κB* subunits (*RELA* and *REL*) and *NFATC2*. The cell differentiation network comprises numerous DEGs encoding transcription factors (e.g., *HOX* genes), receptors, ligands, and histones, while the main regulatory hubs are *CREB*, *AR* and epigenetic modifiers. Clustering analyses highlighted *PIK3CD* as a central player within the differentiation network.

**Conclusions:**

Our study revealed distinct developmental transcriptomes between Brazilian broiler and layer lines. The gene expression profile of broiler embryos seems to favour increased cell proliferation and delayed differentiation, which may contribute to the subsequent enlargement of pectoral tissues during foetal and postnatal development. Our findings pave the way for future functional studies and improvement of targeted traits of economic interest in poultry.

**Supplementary Information:**

The online version contains supplementary material available at 10.1186/s12864-024-10083-x.

## Background

The domestic chicken *Gallus gallus domesticus* is omnipresent in human societies and is currently the world’s most common livestock species [1, 2]. Every year billions of chickens are raised to meet the demand for 120 million tons of chicken meat and 1.2 trillion eggs per year [3]. This demand is expected to rise as human populations increase, become more urban, and gain access to improved living standards. It is estimated that by 2050 the demand for livestock products will double [4].

Poultry breeding programs have contributed to the poultry industry, optimising broiler chickens’ production traits and layer hens’ reproductive attributes. Intensive directional genetic selection is the primary strategy in breeding programs to generate chicken strains with desired features. For this purpose, chickens with particular traits are systematically selected, and breeding is performed over several generations [5]. Due to this directional selection, broilers’ body weight and muscle yield have significantly increased, and feed conversion rate improved over the last 50 years [6]. Notably, broiler chickens weigh approximately five times as much as layer hens at 42 days of age [7]. Regarding layer hens, most commercial strains lay approximately 300 eggs annually, while female broiler strains from a purebred line lay an average of 132.4 eggs in the same period [8]. Therefore, the genomes of broilers and laying hens have been driven toward selecting alleles of genes related to body weight and increased egg production, respectively [5].

Understanding the molecular basis underlying phenotypic variations in chickens is essential to comprehend how distinct features arise among different lines and has significant applications for breeding improvement programmes. Hence, studies focused on analysing developmental differences are crucial, given that changes in early development establish the conditions that give rise to distinct phenotypes displayed by juvenile and adult birds [9]. Since thousands of genes are expressed in the embryo’s body to regulate multiple developmental processes [10], mRNA sequencing methodologies have become powerful tools for comparing vast gene repertoires between conditions and identifying differentially expressed representatives potentially involved in morphological diversification in chickens.

The Brazilian broiler (TT) and laying hen (CC) lines show remarkable phenotypic differences concerning body weight, growth potential and muscle mass. For example, broilers’ breast muscle weight is more than four times higher than that of layer lines [6]. Therefore, this work aimed to identify key differentially expressed genes (DEGs) during early development (days 2.5 to 3.5) between these lines, which may contribute to distinct postnatal body traits in these chickens. For that, a global transcriptome analysis was performed using embryonic tissues of the broiler and layer chicken lines to identify DEGs. A flow of bioinformatics analysis was initially conducted with the broilers up- and downregulated DEGs. Further analyses were conducted on the *mitotic cell cycle* and *cell differentiation* biological processes, given that these processes may impact postnatal body structure development.

## Results

### Summary of sequencing data

The present study employed RNA sequencing to investigate gene expression profiles in embryonic tissues from a Brazilian broiler (TT) and a layer (CC) chicken lines. The sequencing yielded an average of 13,426,155 raw paired-end reads of 100 bp per sample. Following quality control, a mean of 12,604,966 reads per sample was retained, with 93.8% of these uniquely mapped to the chicken reference genome (GRCg6a). In total, 16,455 transcripts were expressed in at least half of the samples, as outlined in Table S1. Principal component analysis (PCA) indicated that sex and developmental stage variables contributed to data bias, as depicted in Fig. S1. Consequently, we accounted for these variables as fixed effects during the differential gene expression analysis to ensure an accurate interpretation of the results.

### Data analysis outline

Our study aimed to identify DEGs during the development of the Brazilian broiler (TT) and layer (CC) chicken lines, which may contribute to their distinct postnatal body traits. RNA-sequencing data were generated from tissue samples from embryos of these strains. We categorised DEGs as up- and downregulated in TT compared to CC and conducted protein function and Gene Ontology (GO) term enrichment analyses to detect potential biological processes and pathways associated with these genes.

**Fig. 1:**
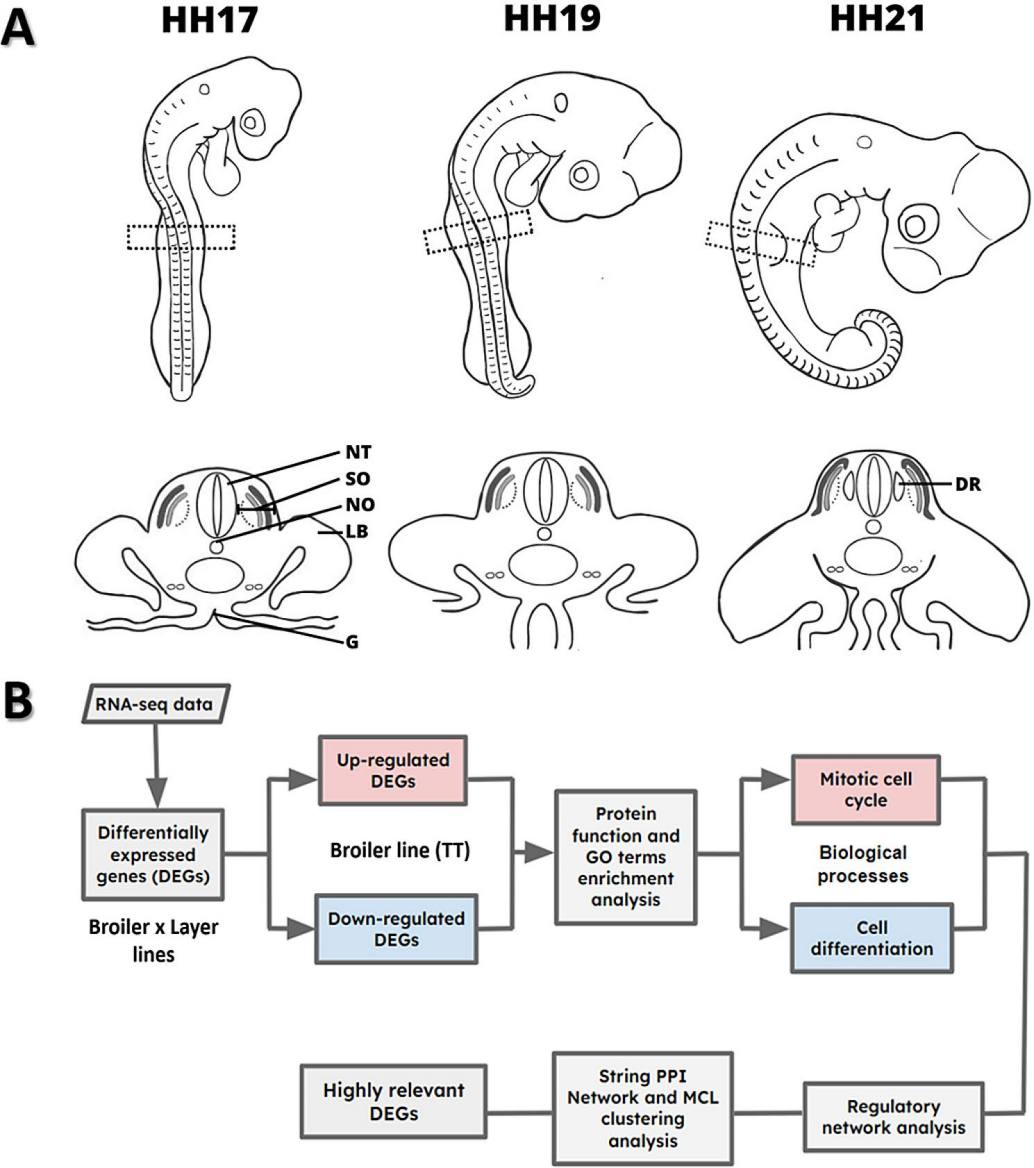
Overview of sample collection and RNA-seq data analysis. (**A**) This picture shows chicken embryos at developmental stages HH17, 19 and 21. The precursor region of chicken wings and breast, which was collected for RNA-seq analysis, is indicated by the dotted rectangle. A cross-section of the embryo body is also provided to better understand the structures developing at this axial level. (NT) neural tube, (NO) notochord, (G) midgut, (LB) limb bud, (SO) somite and (DR) dorsal root ganglion. (**B**) The flowchart illustrates the key steps involved in RNA-seq data analysis. Boxes in the flowchart represent specific analysis stages, while arrows depict the flow of data between them

Further analyses were focused on the *mitotic cell cycle* and *cell differentiation* biological processes, which were enriched in the up- and downregulated broiler gene sets and are likely to impact postnatal body structure development. We constructed regulatory networks to identify primary regulators, their target genes, and possible genetic interactions driving the expression of specific DEGs. We also built a STRING protein–protein interaction network and applied MCL clustering analysis to reduce network complexity and extract functional modules. Critical genes for network functionality were identified based on the connectors of different clusters and their first interactors. A flowchart of our analyses is presented in Fig. 1B.

### Differentially expressed genes between the TT and CC lines

**Fig. 2:**
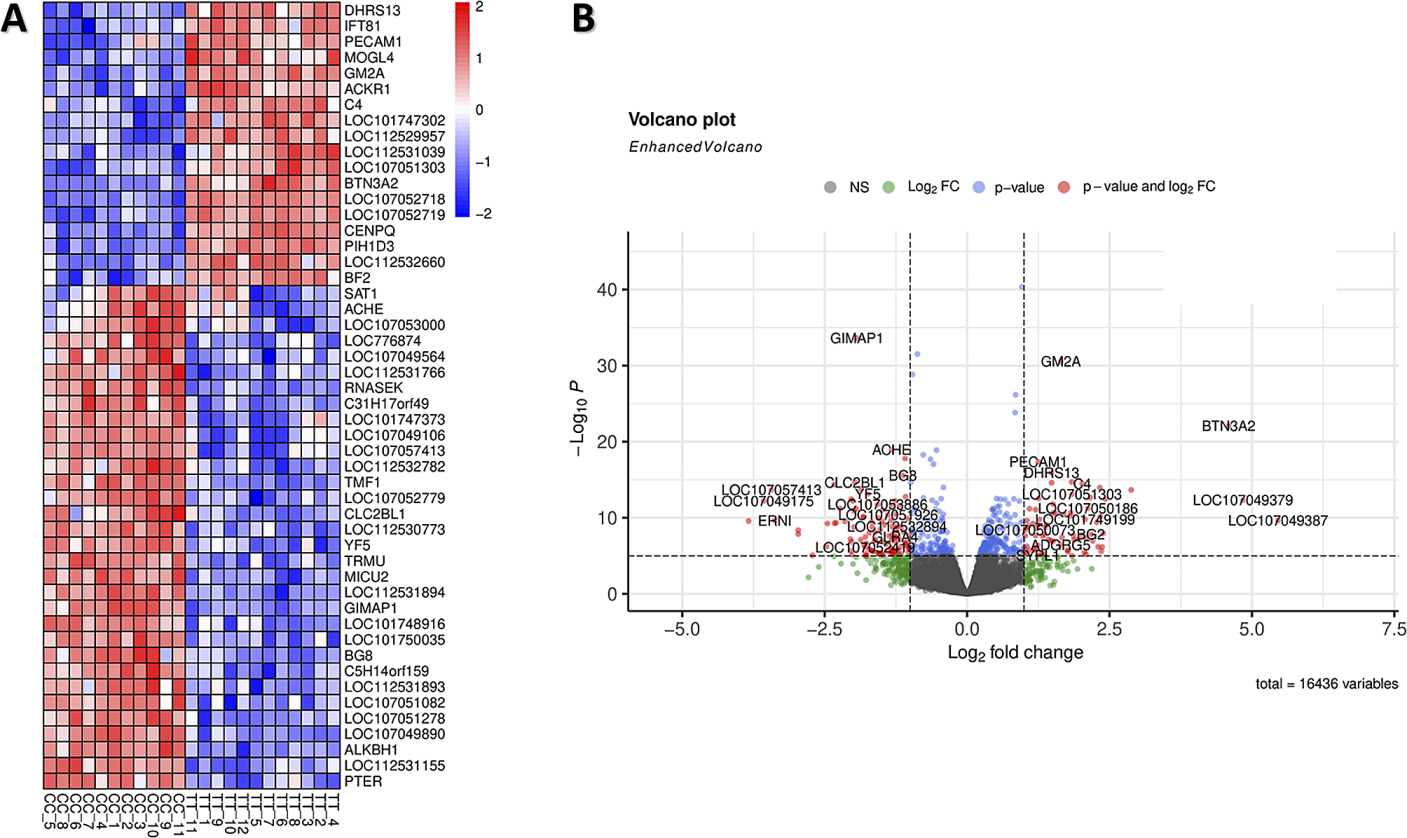
Differential gene expression profiles between embryonic tissues of the Brazilian broiler (TT) and layer (CC) lines. **(A)** Heatmap built based on the 50 most significantly up- and downregulated DEGs between broilers and layers. Genes and samples were clustered into rows and columns, respectively. Upregulated DEGs (red); downregulated DEGs (blue). **(B)** Volcano plot generated from the set of expressed genes (16.436 genes). The X-axis displays the relative expression as log2-fold change, and the Y-axis shows the log10 FDR corrected *p*-value. DEGs are denoted as red dots

Our study identified 2,421 DEGs between embryonic tissues of the Brazilian broiler (TT) and layer (CC) chicken lines based on FDR of < 0.05. A heatmap analysis of the top 50 up- and downregulated DEGs confirmed distinct expression patterns between the TT and CC lines and consistent gene expression profiles within each strain (Fig. 2A). Table S2 provides detailed information about the identified DEGs, including gene name, ID, description, and position in chicken chromosomes.

The differential expression between the TT and CC chicken lines ranged from a 5.46 to -3.83 log2-fold change, as pictured in a volcano plot (Fig. 2B), with a similar number of upregulated (1,199) and downregulated (1,222) DEGs identified in TT compared to CC. Moreover, the distribution of DEGs in the chicken genome analysis revealed that chromosome 1 had the highest number of upregulated DEGs (133 DEGs), while chromosomes 2 to 5 had between 79 and 63 upregulated DEGs each. Chromosome 1 also had the most downregulated DEGs (159 genes), followed by chromosome 2 (111 genes), and chromosomes 3, 4, and 5, with 88, 80, and 60 downregulated DEGs, respectively.

Finally, our analysis revealed that approximately 16% of the upregulated DEGs in the broiler line and approximately 4% of the downregulated DEGs have LOC IDs. This means that so far there is limited information about the annotation of these DEGs.

### Protein function and Gene Ontology (GO) enrichment analysis

To characterise the subsets of up- and downregulated DEGs, we performed an overrepresentation analysis of protein function and biological processes using the Metacore™ and GeneGO databases. To ensure the reliability of the results, we excluded the DEGs with LOC IDs, given the lack of information about them. The main findings of the analysis are summarised in Table S3.

Regarding protein function, a summary of enriched protein classes in broiler up- and downregulated DEGs is presented in Table 1. Upregulated DEGs were enriched in proteases, while downregulated DEGs were enriched in receptors and ligands. In addition, kinases, enzymes, and transcription factors were enriched in both DEG subsets.

**Fig. 3:**
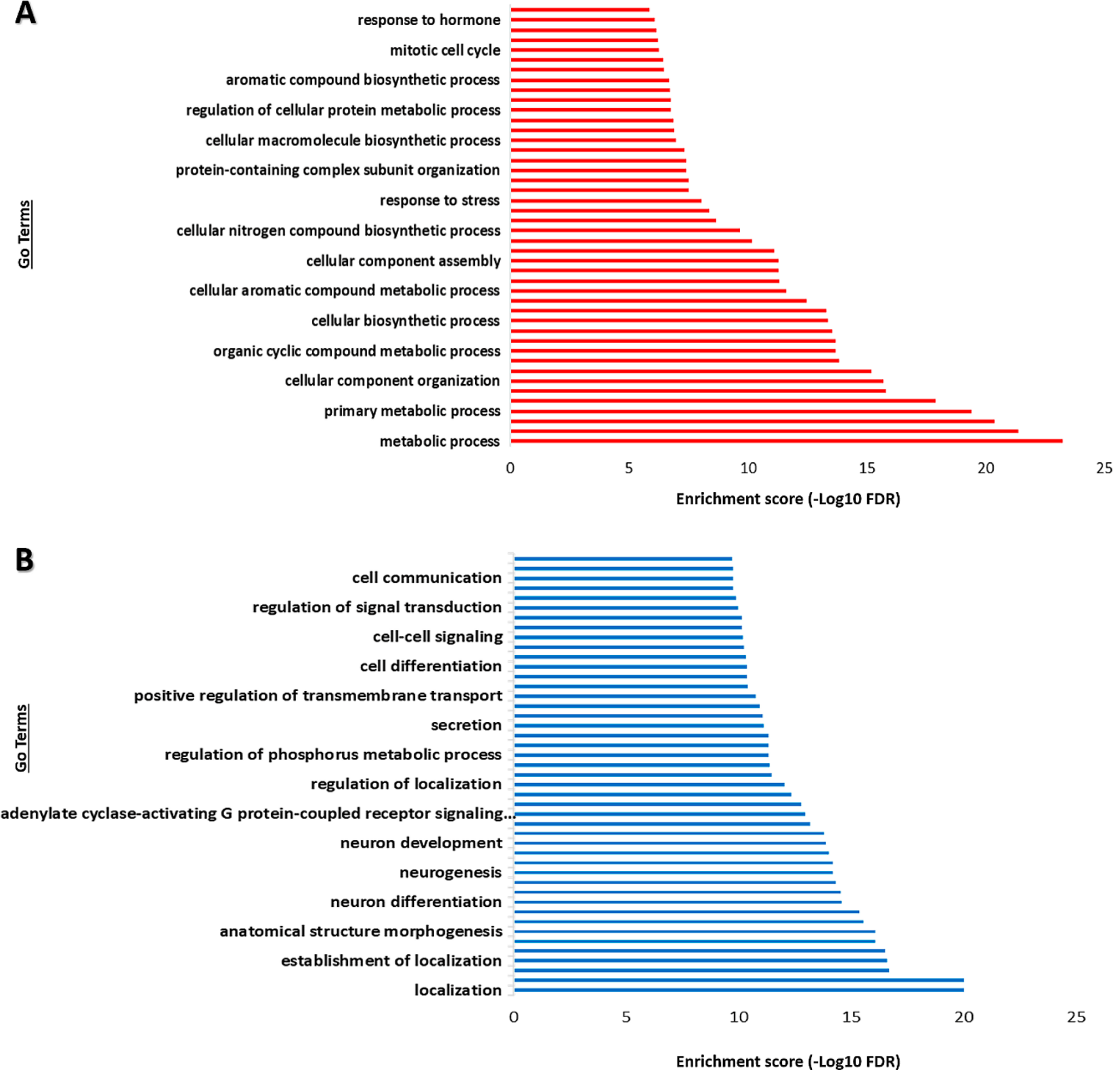
Enriched GO terms in up- and downregulated gene sets of the TT broiler embryonic tissues. **(A)** Upregulated DEGs; **(B)** Downregulated DEGs. Significantly enriched GO terms were selected based on a False Discovery Rate (FDR) of less than 0.05

The GO analysis revealed that upregulated DEGs were significantly enriched in various biological processes, including the response to hormones, the mitotic cell cycle, and different types of metabolic and biosynthetic processes (Fig. 3A). In contrast, downregulated DEGs were enriched in cell communication, signal transduction, and cell differentiation (Fig. 3B). Notably, the downregulated DEGs were also enriched in a range of biological processes associated with nervous system development, such as neuron development, neurogenesis, and neuron differentiation, as well as with the regulation and establishment of localization (Fig. 3B).

**Table 1:** Enrichment of functional annotation in up- and downregulated DEGs in broiler embryonic tissues

Protein classes	Up-regulated *p*-value	Down-regulated *p*-value
Kinases	2.21.e-15	2.44.e-10
Receptors	-	5.86.e-15
Enzymes	2.99.e-45	1.24.e-22
Ligands	-	7.10.e-5
Transcription factors	1.89.e-11	8.23.e-8
Proteases	4.45.e-5	-

- Statistically nonsignificant

### Integrated analysis of regulatory networks, protein–protein interactions, and clustering to identify key genes in broiler development

In broilers, the DEGs were significantly enriched in the *mitotic cell cycle* (upregulated DEGs, *p*-value 2.79 e-12) and *cell differentiation* (downregulated DEGs, *p*-value 2.058e-13) biological processes. Given that the interplay between cell proliferation and differentiation during development plays a crucial role in determining postnatal growth potential, we investigated the regulatory networks associated with these processes. This decision was founded on the idea that gene regulation is the basis for genetic information manifesting as morphological attributes [11, 12].

**Fig. 4:**
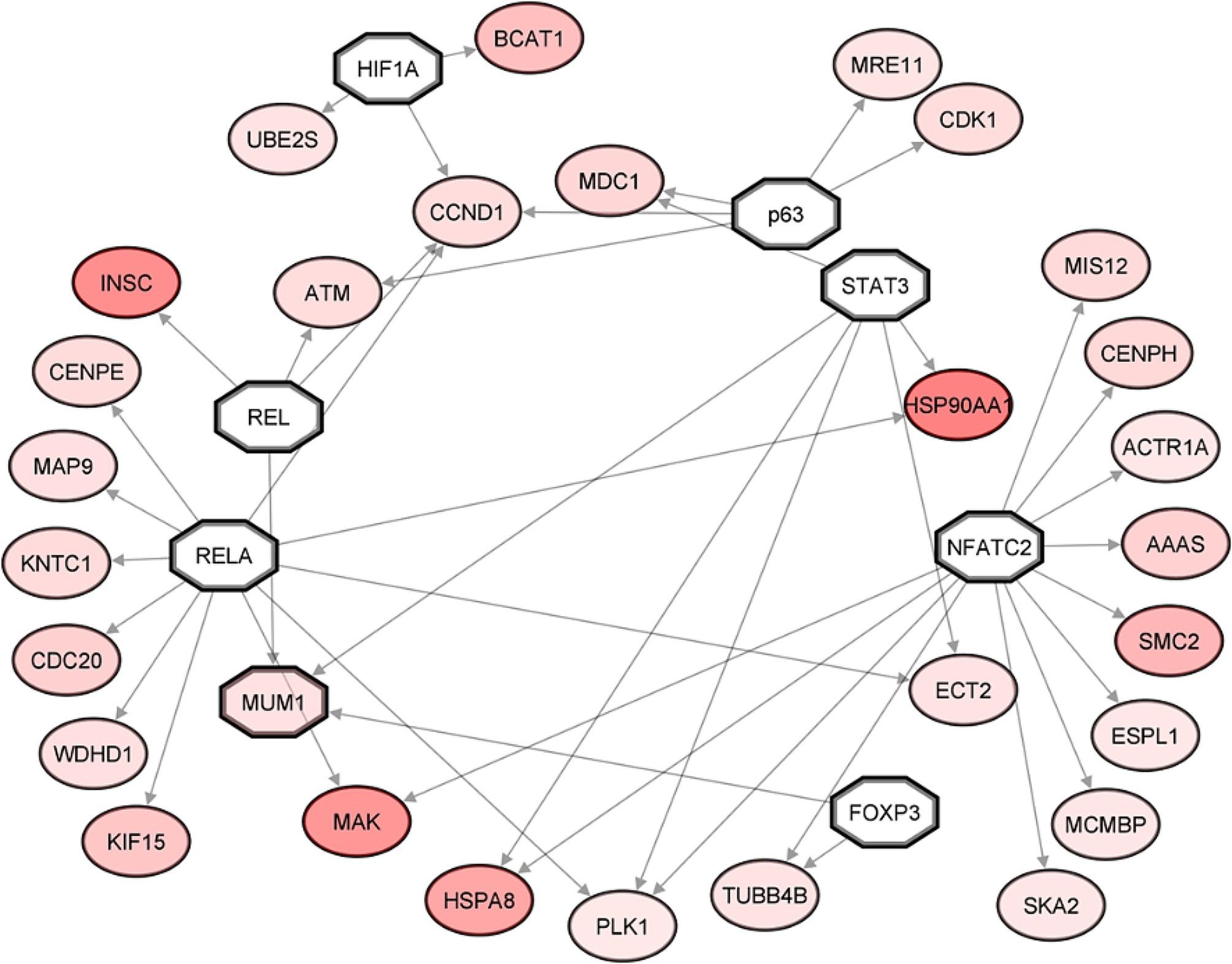
*Mitotic cell cycle* regulatory network generated from broilers’ upregulated DEGs. A gradient of red was used to indicate upregulated DEGs; darker red indicates higher expression levels. Transcription factors are represented as bold octagons. Arrows indicate the interaction between the regulator and the target gene. White geometric shapes denote genes that are not DEGs

The *mitotic cell cycle* regulatory network constructed using broilers’ upregulated DEGs is shown in Fig. 4. Among the DEGs, those with the highest expression levels were *HSP90AA1* (log2FC 0.83), *INSC* (log2FC 0.76), *MAK* (log2FC 0.69), *HSPA8* (log2FC 0.59), *SMC2* (log2FC 0.48), and *BCAT1* (log2FC 0.43). The regulatory hubs that Metacore™ identified as potential regulators of these DEGs include the transcription factors *NFATC2, RELA, REL, STAT3, p63, HIF1A*, and *FOXP3*, although they were not differentially expressed themselves. In this network, the outer-located DEGs are regulated by a single transcription factor, while the others are coregulated. *CCND1, PLK1*, and *MUM1* are the most regulated genes in this network (Fig. 4). Table S4 summarises the mechanisms and interaction effects among transcription factors and DEGs of the generated network.

**Fig. 5:**
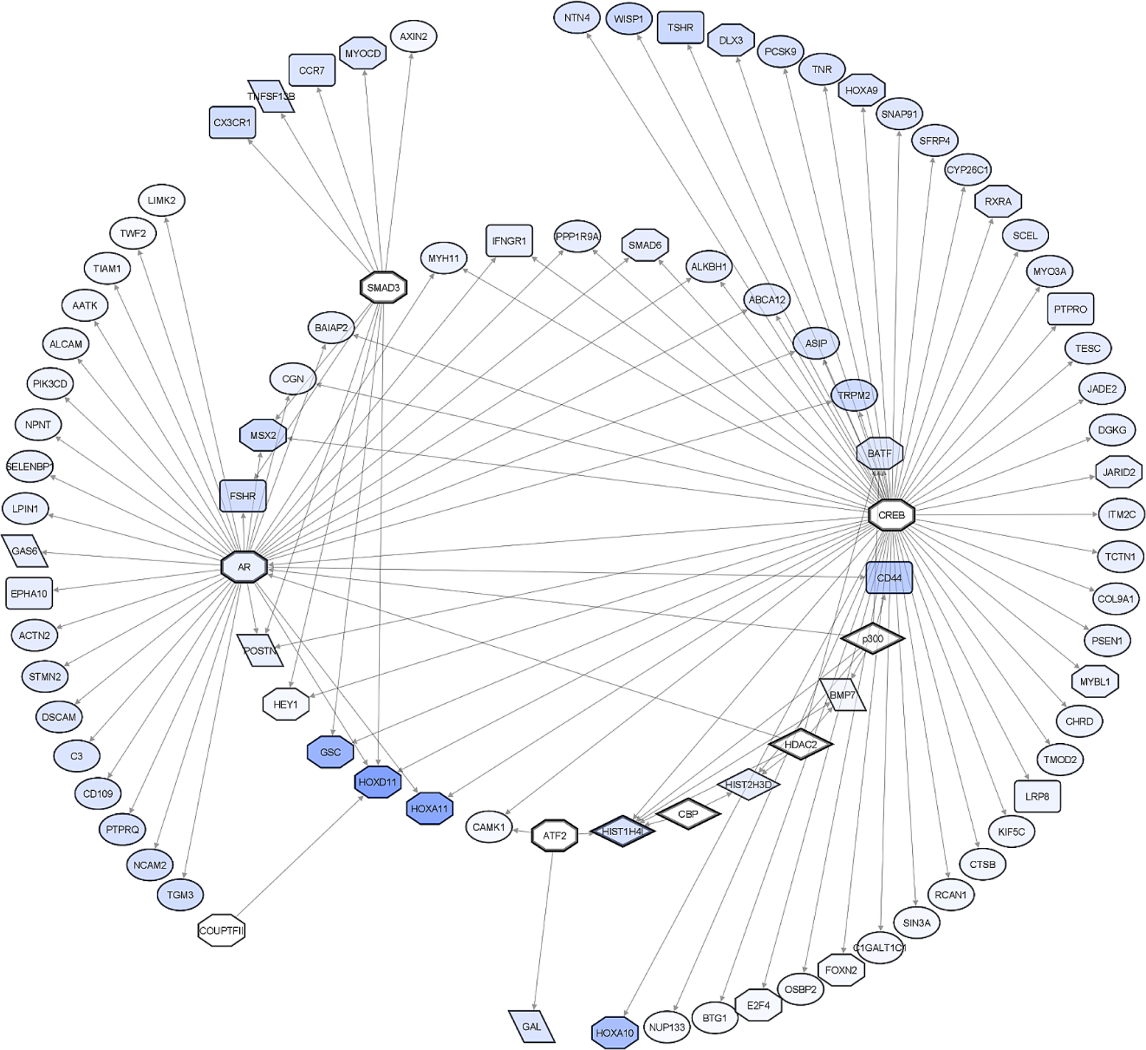
*Cell differentiation* regulatory network generated from broilers’ downregulated DEGs. A gradient of blue was used to indicate downregulated DEGs; darker blue indicates lower expression levels. DEGs representing ligands, receptors, and transcription factors are represented as parallelograms, round rectangles, and octagons, respectively. DEGs encoding other protein classes are shown as ellipses. Regulatory genes are represented either as bold octagons (transcription factors) or diamonds (proteins involved in epigenetic modifications). Arrows indicate the interaction between the regulator and the target gene. White geometric shapes denote genes that are not DEGs

The *cell differentiation* regulatory network generated from broilers’ downregulated DEGs is presented in Fig. 5. Among the genes with the most significant reduction in expression levels are the transcription factors *HOXD11* (log2FC -2.78), *HOXA11* (log2FC -2.6), *GSC* (log2FC -2.21), *HOXA10* (log2FC -1.93), and the receptor *CD44* (log2FC -1.49). *HOX* genes and the homeobox *Goosecoid* (*GSC*) are pivotal components of the developmental genetic toolkit [13, 14], and they were all downregulated in the TT compared to the CC line. In addition, many other transcription factors, receptors, and ligands were downregulated in the *cell differentiation* network. Notably, genes encoding histones *HIST1H4I* (log2FC -0.92) and *HIST2H3D* (log2FC -0.52) were also among the downregulated DEGs.

Regarding the regulators of the *cell differentiation* network, *CREB* (non DEG, 114 connections) and *AR* (log2FC -0.44, 62 connections) are the main hubs, followed by the transcription factors SMAD3, ATF2 and COUPTFII. Additionally, genes encoding epigenetic modifiers such as *CBP*, *HDAC2*, and *p300* are putative regulators of the network, despite not being DEGs themselves (Fig. 5). Notably, the DEGs in the network were found to interact primarily with either *CREB* or *AR* (see outer-located DEGs, Fig. 5). However, genes encoding histones *HIST1H4I*, *HIST2H3D*, and *BMP7* are linked to several putative regulators, most of which are chromatin-modifying enzymes (Fig. 5). Table S5 summarises the mechanisms and interaction effects among transcription factors and DEGs of the *cell differentiation* network.

After regulatory analysis, the genes related to *cell differentiation* and their regulators were employed to create a protein–protein interaction (PPI) network, followed by MCL clustering analysis. Clustering analysis helps the interpretation of complex networks. This approach was used to identify highly connected genes and genes that bridge different clusters, which are particularly relevant since they can profoundly affect gene expression and specific biological processes during development. These molecules were subsequently highlighted as pertinent to broiler (TT) development control.

The PPI network built by STRING was highly significant (*p*-value of 7.7e-14) and was enriched in nervous, skeletal, and immune system development (GO:0007399, GO:0001501, GO:0002376). In addition, it was significantly enriched in the TGF-beta (gga04350) and Notch (gga04330) KEGG signalling pathways.

**Fig. 6:**
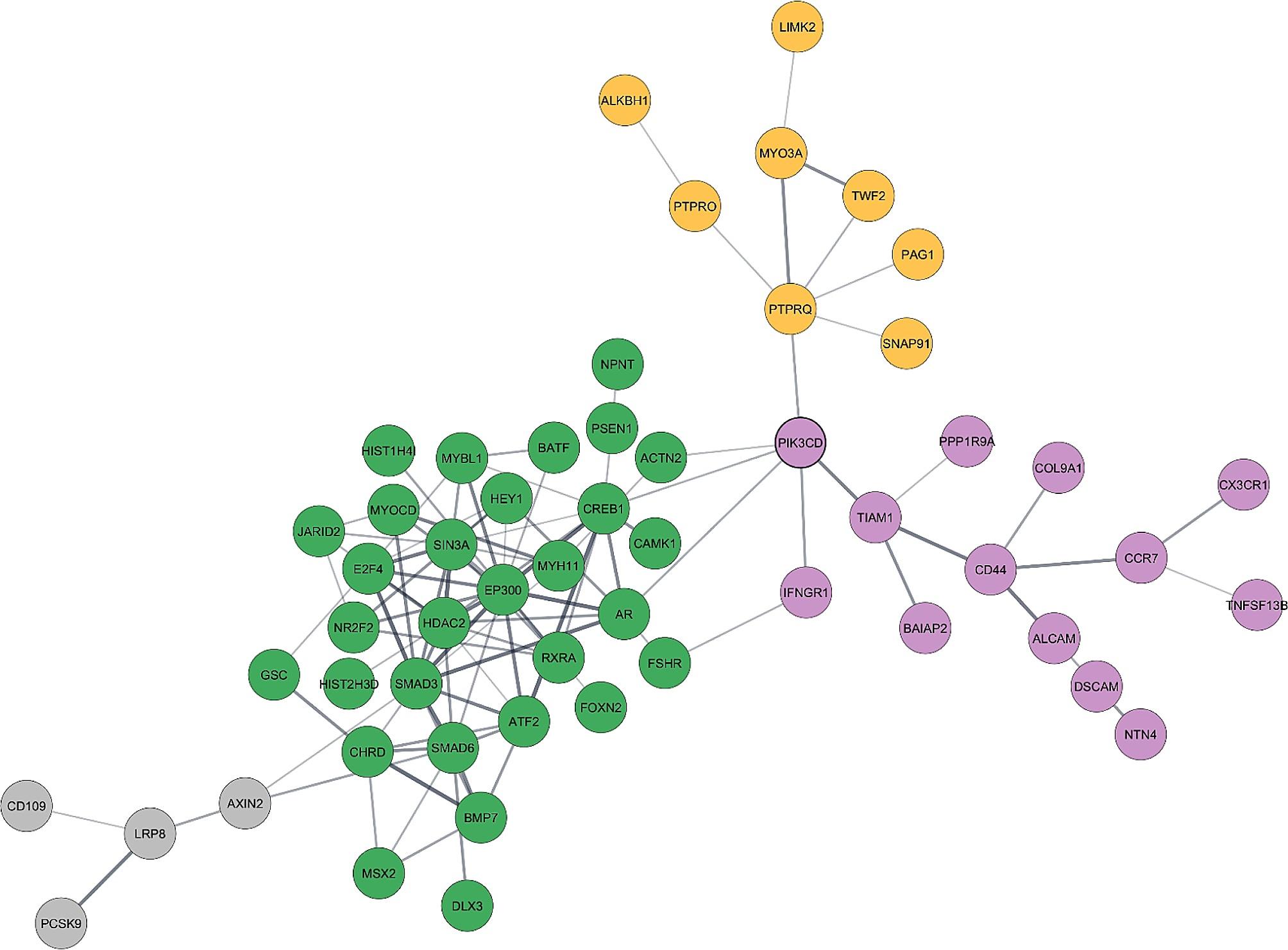
MCL clustering analysis in STRING reveals different functional clusters in the protein–protein interaction network generated from cell differentiation downregulated DEGs and their putative regulators. Cytoscape software was used for better visualization of this network. The nodes represent genes and the line thickness denotes the degree of confidence prediction of node interaction. Each colour represents a different cluster. Green and purple clusters were considered “main clusters” as they contain most of the genes. Interaction score > 0.4

MCL clustering analysis (Fig. 6) revealed two main protein clusters in the *cell differentiation* PPI network. The MCL algorithm employs a Markov chain to simulate random walks on the network, with clusters determined by the steady-state probabilities of these walks. These resulting clusters reflect sets of proteins that exhibit strong interconnectivity and likely possess functional relevance [15]. To assess the biological significance of these clusters, we subjected them to further analysis using established tools for gene ontology (GO) and pathway enrichment analysis. The first cluster, which contained 32 proteins (clustering coefficient 0.512), was enriched in the GO term “Regulation of Transcription, DNA-templated” (GO:0006355), with 24 proteins involved in this biological process. Transcription factors and chromatin modification proteins constitute most of this cluster. Therefore, it is a cluster involved in the modulation of gene expression at different levels. The second larger cluster comprised 11 proteins (clustering coefficient 0.545) and was associated with biological processes such as “Regulation of anatomical structure morphogenesis” (GO:0022603), with 8 proteins. This cluster mainly comprises proteins related to morphogenesis, cell adhesion and migration, which are located in the cell plasma membrane; thus it is a cluster of proteins involved in signal transduction.

Cytoscape software was used for better visualisation of clusters after the PPI network and MCL analyses from the *cell differentiation* regulatory network (Fig. 6). The green cluster represents “regulation of transcription, DNA-templated”, and the purple cluster represents “regulation of anatomical structure morphogenesis”. Connecting these two clusters is the *PIK3CD* lipid kinase (FC -0.34), which has as its first partners *ACTN2* (FC -0.56), *CREB1* (regulator) and *AR* (FC -0.44) in the first cluster, while in the second cluster, it connects with *TIAM1* (FC -0.28) and *IFNGR1* (FC -0.47). The MCL clustering analysis is summarised in Table S6.

## Discussion

The postnatal characteristics of chickens are significantly influenced by events occurring during embryonic development. The Brazilian chicken lines TT (broiler) and CC (layer) exhibit notable differences in their morphological and growth performance traits [16]. Our study compared the transcriptomes of wings and breast-level tissues in TT and CC embryos during early development to better understand the genetic mechanisms underlying the phenotypic variations between these lines. Specifically, we aimed to identify dissimilarities in gene expression, regulation, enriched biological processes, and pathways that may contribute to the observed phenotypic differences.

Comparing the developmental transcriptomes of TT and CC lines revealed that chromosomes (Chr) 1 and 2 have the highest number of differentially expressed genes (DEGs), followed by Chr3 to 5. Importantly, among the chromosomes with the highest number of DEGs, Chr1 and 4, along with Chr27, have been associated with quantitative trait genes (QTGs) or quantitative trait loci (QTLs) that have the strongest influence on chicken growth [17, 18]. Therefore, some DEGs identified in our work may be within these QTGs or QTLs regions. To confirm that, future investigations could be conducted addressing the overlap between the DEGs identified in our study to the distribution of known QTLs on chromosomes 1 and 4.

Another important finding of our RNA-seq data analysis is that many DEGs are named LOCs, which infers that their orthologues are currently unknown. Among the LOCs are the top upregulated genes in broilers - LOC107049387 (log2FC 5.45) and LOC107052718 (log2FC 4.01) - predicted to encode a coiled-coil domain-containing protein 81-like and endogenous retrovirus group K member 11 Pol protein, respectively. To fully understand the roles of these genes during development and their potential in genetics and poultry breeding programs, it will be essential to characterise their expression patterns and perform functional studies.

In the functional enrichment analysis of proteins encoded by DEGs, the upregulated DEGs showed enrichment only in proteases. Proteases play a critical role in breaking complex proteins into amino acids present in egg white or yolk. Therefore, genetic selection may have contributed to the increased or advanced expression of these proteins during breeding [19, 20]. On the other hand, receptors and ligands were enriched among the downregulated DEGs in broilers. This enrichment implies a potential reduction in signal transduction within molecular signalling pathways, considering the crucial role of these protein classes in activating molecular pathways.

The enrichment analysis of DEGs revealed that the subset of broilers’ upregulated DEGs was significantly enriched in biosynthetic and metabolic biological processes, which is consistent with the enrichment of proteases in this gene set. This finding suggests that these metabolic alterations are intrinsic to the TT broiler line and may be related to increased nutrient uptake by the embryos during development, providing higher energy levels necessary to sustain a higher growth rate in this line compared to the CC line. Other studies have also indicated that broiler embryos have higher growth capacity than layer embryos, likely due to their enhanced nutrient uptake [21–23]. This difference could promote better utilisation of nutrients from the egg in broiler embryos. Furthermore, the subset of downregulated DEGs in TT was significantly enriched in biological processes related to cell interaction and communication, which are essential for tissue differentiation at the cellular and molecular levels [24]. This finding is consistent with the significant reduction in the classes of proteins that encode receptors and ligands in this gene set, as previously discussed. This result suggests a possible delay in the start of these genetic programs during broiler embryo development.

We selected the *mitotic cell cycle* and *cell differentiation* for deeper analysis among the enriched biological processes in broilers due to their impact on the postnatal growth potential of chickens. During development, proliferation and differentiation are fine-tuned by a switch-on/off type mechanism, where progenitor cells proliferate and then withdraw from the mitotic cell cycle to only then activate specific differentiation programs [25]. Therefore, more extended periods of cell proliferation could lead to greater growth potential in postnatal life.

The analysis of the *mitotic cell cycle* regulatory network indicates that the *CCND1* and *HSP90* are central molecules in broiler cell cycle regulation, as they are the most coregulated gene and the most upregulated gene, respectively. In addition, *CDK1* (Cyclin Dependent Kinase), which encodes an essential protein involved in G1/S and G2/M phase transitions [26], was upregulated in TT. On the other hand, a critical CDK1 inhibitor, *CDKN1A* (Cyclin Dependent Kinase Inhibitor), which encodes the p21 protein that arrests cell cycle progression, was strongly downregulated in TT [25, 27]. Therefore, reduced *p21/CDKN1A* in broilers may be related to increased mitosis as *CDK1* is free to promote the cell cycle. Regarding gene regulation in the *mitosis cell cycle* network, *CCND1* is among the most coregulated genes with *PLK1* and *MUM1*. *PLK1* encodes a kinase highly expressed during mitosis, while *MUM1* encodes a protein involved in DNA repair and chromatin organisation [28, 29]. The main regulators of these genes and other DEGs in the regulatory network include *NF-κB* subunits (*RELA* and *REL*) and the transcription factors *STAT3, NFATC2* and *FOXP3*. These transcription factors have been associated with controlling the cell cycle [30–32], suggesting that the *NF-κB JAK-STAT* and *FOXP* signalling pathways might orchestrate the expression of multiple genes, most likely enhancing the mitotic potential of undifferentiated mesenchymal cells.

The *cell differentiation* regulatory network analysis revealed a significant downregulation of genes that constitute the developmental genetic toolkit. The toolkit encompasses a hierarchical network of evolutionarily conserved genes, mainly encoding transcription factors and signalling molecules that drive the expression of numerous other genes to regulate cell proliferation, differentiation, migration, and apoptosis in the developing embryo [33]. Examples of genes in the genetic toolkit are the transcription factors *HOXA9, A10, A11* and *D11*, which were all strongly downregulated in TT compared to CC and are known to be expressed in the developing wings of chickens during the stages evaluated in our study [13, 34]. In addition to the *HOX* genes, the homeobox transcription factor GSC is also a key gene in the developmental genetic toolkit and was found to be strongly downregulated in broiler chickens. This gene is well known for regulating dorsoventral axis patterning during gastrulation and it has also been shown to regulate *HOX* genes during limb development [35, 36]. Concerning the regulation of the *cell differentiation* network, *CREB* and *AR* have been identified as the primary regulatory hubs (Fig. 5). Notably, *CREB* regulates essential *HOX* genes in the network and coregulates *GSC*s along with *SMAD3*.

In addition to transcription factors, histone genes were also downregulated in TT, suggesting that the chromatin-state is an additional layer in the regulation of cell differentiation. These findings indicate that a repressive influence on gene expression, particularly on differentiation-related genes in broilers, can be achieved through the coordinated suppression of foundational toolkit genes such as *HOX* genes and *GSC*, as well as a reduction in the expression of chromatin structural proteins. Furthermore, proteins involved in the epigenetic control of transcription, including *p300, HDAC2*, and *CBP*, were predicted to be regulators of the cell differentiation network. Although proteins involved in epigenetic changes are not direct regulators of transcription, they influence the binding of transcription factors to gene promoters [37]. This combined regulatory action probably delays the activation of differentiation-related genes in broilers, potentially extending the proliferative phase of progenitor cells during development. Consequently, these synergistic regulatory mechanisms may influence the growth rate capacity of broilers by affording additional time for progenitor cells to undergo proliferation before committing to specific cell fates. Regarding the PPI network and MCL analyses of the *cell differentiation* biological process, one might hypothesise that *PIK3CD* and its first connectors in the aforementioned networks are pivotal in controlling the notable downregulation of genes that contribute to different aspects of cell differentiation in the TT line. However, further research is needed to fully understand the mechanisms underlying these clusters’ conjoint roles in differentiation.

## Conclusions

**Fig. 7:**
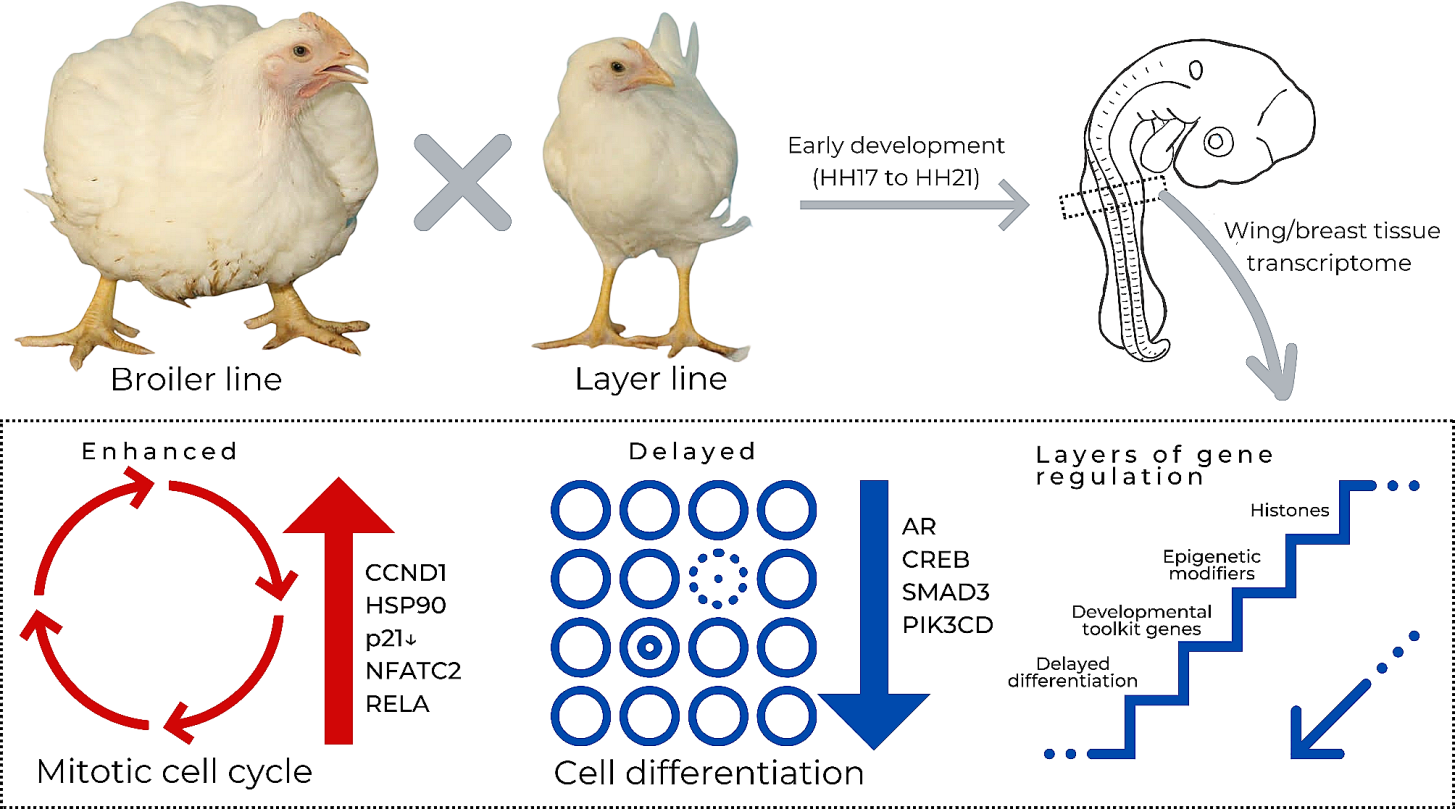
Distinct transcriptomic profiles between broiler and layer lines in pectoral and wing development. The biological processes *Mitosis cell cycle* and *Cell differentiation* were highlighted, together with DEGs and regulators that may contribute to the phenotypic divergence of the compared lines

Our study reveals distinct transcriptomic profiles in early embryonic development for the TT broiler and CC layer Brazilian lines. Notably, our findings suggest heightened cell proliferation and postponed differentiation in broiler embryos, likely influencing their increased postnatal growth rates. In addition, our work points to specific molecules and regulators as playing a critical role in the phenotypic divergence of the compared strains (Fig. 7). This study paves the way for in vivo functional assays to uncover the impact of modulating gene expression on specific morphological traits of chicken. Additionally, they may allow future genetic interventions in chickens (e.g., gene editing or RNAi use) to refine poultry growth traits.

## Methods

### Ethics statement

The procedures involving animals were evaluated and approved by the Ethics Committee for the Use of Animals (CEUA, number 015/2016) from the Brazilian Agricultural Research Corporation – Embrapa Suínos e Aves. All procedures followed the guidelines by the Brazilian Council of Animal Experimentation and the ethical principles in animal research, according to FASS [38], the Guide for the Care and Use of Agricultural Animals in Agricultural Research and Teaching. This study was carried out in compliance with the ARRIVE guidelines.

### Egg incubation and embryo tissue harvesting

Fertilised eggs from broilers (TT) and layers (CC) were supplied by Embrapa Suínos e Aves National Research Center, Concórdia-SC, Brazil. Egg incubation was performed in a humidified atmosphere at 37.8^o^C until the embryos reached HH17 (52–64 h), HH19 (68–72 h), and HH21 (3.5 days) Hamburger & Hamilton developmental stages [39].

After embryo harvesting, a tissue strip at the level of somite 17 to 18 was dissected using a sharpened tungsten needle (100 μm), as illustrated in Fig. 1A. The removed tissue section, including somites and all adjacent structures, was immediately processed for RNA extraction. Four samples (*n* = 4) were prepared for each developmental stage and chicken line, totalling 24 independent samples.

### RNA extraction, quality analysis, library preparation, and sequencing

Total RNA of samples from the collected tissue section was extracted immediately after collection using TRIzol Reagent (Life Technologies, Carlsbad, CA) extraction protocol. The integrity of RNA samples was evaluated with a Bioanalyzer 2100 (Agilent, Santa Clara, CA, USA), and only samples with RIN (RNA Integrity Number) values > 7 were used for subsequent analyses. RNA libraries were prepared with 2 µg of total RNA using the TruSeq RNA Sample Preparation kit v2 (Illumina, San Diego, CA) according to the protocol provided by the manufacturers. Average library sizes were estimated using an Agilent Bioanalyzer 2100 (Agilent, Santa Clara, CA, USA) and quantification was performed by quantitative PCR with the KAPA Library Quantification kit (KAPA Biosystems, Foster City, CA, USA). One lane of a sequencing flow cell, using the TruSeq PE Cluster kit v3-cBot-HS kit (Illumina, San Diego, CA, USA), was clustered and sequenced with TruSeq SBS Kit v3-HS (200 cycles) equipment, according to the manufacturer’s instructions. Sequencing was performed using HiSeq 2500 (Illumina, San Diego, CA, USA), using a paired-end (2 × 100 bp) protocol. All sequencing steps were performed at the ESALQ-USP Animal Genomics Center, located in the Animal Biotechnology Laboratory of ESALQ-USP, Piracicaba, São Paulo, Brazil.

### Quality control and read alignment

Sequencing adaptors and low-complexity reads were removed in an initial data-filtering step. Quality control and read statistics were estimated with FASTQC version 0.11.8 software [http://www.bioinformatics.bbsrc.ac.uk/projects/fastqc/]. Sequencing data were aligned and uniquely mapped against the reference chicken genome GRCg6a with STAR v.2.5.4 software [40]. The same software was used to quantify the paired-end reads, which provided the expression matrix with 24,106 genes. From this matrix, only the genes presenting at least one count per million (CPM) in at least 12 samples (half the samples) were kept for further analysis. Data were normalised for library sizes and transcripts with zero counts (unexpressed) or low expression were removed from this matrix, using TMM (trimmed means of m values) and transformed into log2CPM using the EdgeR v3.26.8 package [41]. Finally, the expression matrix was normalised using the ARSyNseq package with the NOISeq v2.28.0 function to decrease batch effects concerning sex and developmental stage variation in the mathematical model.

### Identification of differentially expressed genes

After data filtering and normalisation, expressed genes were analysed for differential expression. This analysis was carried out using the EdgeR package [41, 42], from a R environment and adjusted *p*-values (q-value) were calculated using Benjamini and Hochberg’s (BH) approach for controlling the false discovery rate (FDR) at 5% [43]. Differentially expressed genes received transcript annotation from the Biomart database, a tool of Ensembl Release 106 (Mar 2022) [https://www.ensembl.org/biomart/martview/a6b1284a9436076779cf1bb5057d78ca]. Information about transcripts that were not annotated in Ensembl was obtained from the Gene database of The National Center for Biotechnology Information (NCBI) [https://www.ncbi.nlm.nih.gov/].

### Functional enrichment and regulatory network analyses

To further investigate the DEGs, we conducted an enrichment analysis using the GeneGo Metacore™ software (Clarivate Analytics, London, UK, v.21.4, build 70,700). [https://portal.genego.com/]. The Gene Ontologies (GO) terms were corrected for multiple tests with a FDR threshold of less than 0.05. The regulatory networks generated by Metacore™ were illustrated and simplified using Cytoscape software [https://cytoscape.org/] [44] for better visualisation of the interplay among the regulators, DEGs, and their associated pathways.

### Protein–protein interaction analysis

To explore protein–protein interactions related to DEGs involved in the *cell differentiation* regulatory network, we employed the Search Tool for the Retrieval of Interacting Genes/Proteins (STRING) version 11.5 [45, 46]. We applied a filter for interactions with at least medium confidence (interaction score > 0.4) to generate a PPI network [45]. We then applied the Markov Cluster Algorithm (MCL) in STRING to identify clusters of functionally related proteins [15]. An inflation parameter of 1.4 was used to generate the clusters. Cytoscape was also used to simplify the MCL cluster network, and only the interconnected nodes within the clusters were considered for the analysis; the other nodes were manually removed [44].

### Electronic supplementary material

Below is the link to the electronic supplementary material.

Supplementary Material 1

Supplementary Material 2

Supplementary Material 3

Supplementary Material 4

Supplementary Material 5

Supplementary Material 6

Supplementary Material 7

## Data Availability

The dataset supporting the conclusions of this article is available in the European Nucleotide Archive (ENA) repository (EMBL-EBI), under accession PRJEB65907 [http://www.ebi.ac.uk/ena/data/view/]. The original contributions presented in the study are included in the article, further inquiries can be directed to the corresponding author.
