# Supplementary material for: Phenotypic divergence between broiler and layer chicken lines is regulated at the molecular level during development
Source: BMC Genomics. 2024 Feb 12;25:168. doi: 10.1186/s12864-024-10083-x (PMC10863267; doi:10.1186/s12864-024-10083-x)
Supplement: Supplementary file 1 — Supplementary Material 1 [file 12864_2024_10083_MOESM1_ESM.pdf]

Table S1 - Readings uniquely mapped against the chicken reference genome (GRCg6a). These 16,455 transcripts were expressed in at least 1 CPM of the 50% sequenced samples.

Gene name	logFC	logCPM	LR	P Value	FDR
KBTBD13	0.046206314	-1.089449347	0.011445051	0.914803622	0.961017703
LOC107054734	0.526966374	-1.088795175	1.668194662	0.196500519	0.421566629
LOC107052357	0.30940896	-1.053617412	0.646626625	0.421321337	0.650673858
CLDN15	-0.826844871	-1.043426796	4.201616096	0.040385474	0.15741623
LOC107054386	0.425108486	-1.043395934	1.253175541	0.262946832	0.498076449
SPP2	-0.759722106	-1.042763609	4.349744289	0.037014503	0.148301352
LOC112532389	-0.632555393	-1.042408725	2.605927086	0.106464882	0.29042149
LOC107055083	-0.37922814	-1.0395478	0.957718708	0.327762318	0.565526033
LOC112531639	0.310597721	-1.038681927	0.557263628	0.455364874	0.678040811
LOC112532486	0.459145324	-1.03017425	1.602518046	0.205546732	0.432404944
RASAL1	0.10967708	-1.028140355	0.084222368	0.771654896	0.886527969
LOC107050050	-0.500507044	-1.020831286	1.906310343	0.167373688	0.385402706
LOC112532620	-0.310452108	-1.014968979	0.526242528	0.468190877	0.687680164
GMNC	0.166286589	-1.001990703	0.204081823	0.651446095	0.813994258
CAPN14	-0.805796345	-0.996991846	4.498111764	0.033932303	0.140290465
LOC107053323	-0.760323523	-0.992454689	2.381023641	0.122817046	0.317579619
LOC112533400	1.449733114	-0.991126145	12.94308588	0.000321105	0.005185267
LOC107053239	-0.584024325	-0.989514326	2.207825877	0.137312066	0.341718161
AGBL3	-0.060018175	-0.989399621	0.021766129	0.882710953	0.947489154
CAPN9	-0.803178916	-0.980759419	4.345677565	0.037103001	0.148475165
BFSP1	-0.833210157	-0.978760003	5.584803476	0.018116966	0.092928514
C5H15ORF62	0.123330403	-0.978732346	0.102168295	0.749242949	0.873205803
LSMEM2	0.190657708	-0.975943176	0.206096218	0.649844508	0.813173579
LOC107055493	0.591959718	-0.970208774	1.612186752	0.204184684	0.430972163
LOC107053578	0.039301221	-0.968684453	0.012625841	0.910534188	0.95894146
LOC107054244	0.186616202	-0.965800061	0.243791476	0.621481023	0.795958144
LOC107053997	-0.102117933	-0.96016329	0.071298864	0.789454939	0.896499422
LOC112531077	0.331719798	-0.958225977	0.51608013	0.472518594	0.691022409
MIR30B	0.107287546	-0.955723187	0.087216406	0.767746202	0.884179034

ASZ1	0.465541708	-0.955646638	1.530984898	0.215964689	0.444824003
LOC100859484	0.089868818	-0.955099915	0.042955911	0.835808312	0.922298307
TMEM45B	0.544465452	-0.954858618	2.164327978	0.14124636	0.348196083
LOC112532651	-1.429940497	-0.954739046	14.41856303	0.000146353	0.002784082
PRR15L	0.68972788	-0.954555949	2.895440111	0.088830513	0.259268143
LOC107052351	-0.206338615	-0.954138373	0.344707848	0.557124286	0.753115441
LOC107054342	-0.198766131	-0.953801689	0.289789814	0.590355254	0.776026179
LOC107054341	0.003966563	-0.953100022	0.00012919	0.990931291	0.995986157
MC3R	1.117686439	-0.952748111	9.209619449	0.002407467	0.022701934
LOC112530324	0.880102035	-0.952214886	5.898455102	0.015154171	0.08262488
CERS4L	-1.399980269	-0.94793211	15.18765279	9.73E-05	0.002024897
CNTN6	-1.032082788	-0.944821067	6.369219514	0.011611666	0.069095879
LOC112532298	0.253852995	-0.943810926	0.538770153	0.462942862	0.684057976
LOC112531764	-0.235362661	-0.943047404	0.45838542	0.498379518	0.709783189
FOXN1	0.4464774	-0.94253676	1.567605916	0.210555034	0.438234642
LOC112530100	0.665951296	-0.942409646	2.861031434	0.090749291	0.262808798
LOC107054716	-1.065185519	-0.941636747	9.235789276	0.002373301	0.022547728
LOC107052698	0.587337996	-0.941207829	2.912934423	0.08787186	0.257054481
LOC112530099	0.063360916	-0.936323354	0.034492644	0.852662812	0.931394748
CXCL13L2	-0.606981863	-0.93605867	2.308523796	0.128666193	0.32784178
LOC112531567	-0.299049604	-0.934331768	0.677074688	0.410595869	0.640898787
KCTD4	-0.404739182	-0.933641631	1.266463267	0.260431219	0.495135263
LOC107051860	0.308132729	-0.932509504	0.686485461	0.407362384	0.637865574
CLDN22	0.037994021	-0.932023088	0.011962065	0.912908055	0.960095348
TRNAV-UAC	0.192096945	-0.931663761	0.249413024	0.617488689	0.793398483
LOC107052525	-0.135723686	-0.931628587	0.140979848	0.707308847	0.848492169
LOC112532054	0.66520973	-0.931592313	3.582798952	0.058380708	0.19889328
LOC112532487	-0.892793795	-0.929741776	6.02119071	0.014135107	0.079032682
MIR22	0.548754989	-0.92910105	1.489518075	0.222291248	0.451915307
SLC25A40	-0.271286462	-0.923175778	0.51993818	0.470868054	0.689581152
TEKT5	0.266910973	-0.923060367	0.552833717	0.457162145	0.679732818
IFIT5	0.312331793	-0.922981148	0.783968777	0.375930088	0.610473661

RGR	0.052576257	-0.922584304	0.020895804	0.88506317	0.9486918
MCHR2	-0.276953001	-0.922552855	0.418538334	0.517667289	0.723314877
LOC107052584	-0.432994286	-0.922343714	1.420450247	0.233329309	0.464849933
LOC107052610	0.158591974	-0.922319383	0.147916036	0.700534877	0.844482927
LOC107054484	-0.287566094	-0.921350903	0.513912512	0.473450047	0.691577499
LOC107053142	0.003314095	-0.919915123	4.89E-05	0.994422045	0.997544036
LOC101749318	0.36263208	-0.917398478	0.909694401	0.340195516	0.577342947
LOC112531879	-0.88649377	-0.917070399	5.378029926	0.020391893	0.100433581
LOC112532878	0.351167667	-0.912918237	0.879401438	0.348365673	0.584934403
DMBX1	0.426599986	-0.911603383	1.524162426	0.216990679	0.446105303
LOC112533028	0.173647144	-0.909484289	0.281460877	0.595745301	0.779071694
LOC107052473	0.007529483	-0.908927887	0.000435174	0.983356674	0.992585822
LOC112531946	-1.742878515	-0.908684615	17.59674044	2.73E-05	0.000727045
LOC107052147	0.171115382	-0.908029528	0.202811882	0.652460685	0.814647589
LOC107052087	-0.478133891	-0.906459012	1.327672477	0.249219709	0.483462139
LOC107053915	-0.819720486	-0.904306885	3.825479299	0.050478909	0.181004672
ENDOU	0.384094941	-0.899483136	1.13449476	0.286818625	0.523179301
ZPBP2	-0.792116592	-0.898427644	4.740943013	0.029452923	0.127807975
LOC107052214	0.450511452	-0.898263057	1.489245832	0.22233351	0.451945387
PLEKHG7	-0.496383888	-0.897802937	1.826657622	0.176522841	0.39680191
LOC112530794	0.048665311	-0.896987853	0.018543024	0.891684698	0.951008202
GPR139	0.579110062	-0.896433887	1.882540648	0.170045667	0.388944472
LOC112530928	0.819244589	-0.895136802	4.474999588	0.034394211	0.141595381
MIR1748	1.073801483	-0.894826003	8.378007123	0.003797886	0.031320685
COL10A1	0.508391619	-0.894619133	1.950302117	0.162554298	0.378860143
LOC107054414	0.031983883	-0.893207014	0.006680107	0.934859893	0.971447144
USH1G	-0.986335573	-0.889165421	7.76674057	0.005321699	0.039858242
LOC112531830	-0.07825147	-0.888014774	0.052410074	0.818921407	0.914637328
LOC112531558	-0.041775015	-0.887787375	0.013622708	0.907084829	0.957413782
LOC112532161	0.184152713	-0.887637123	0.323382733	0.569581803	0.762361198
NSUN7	0.212238131	-0.884242393	0.284795081	0.593575436	0.777649985
LOC112532930	0.819568696	-0.884241552	5.677043318	0.017188309	0.090236788

BDH1	0.34758896	-0.884059558	0.929268158	0.335053166	0.572334667
LOC101750952	-0.979463265	-0.884041128	4.982839656	0.02559993	0.116850721
LOC107051047	0.62777928	-0.883322119	3.517783948	0.060713575	0.204136061
MAMDC4	-0.202465516	-0.880819323	0.211574141	0.645536396	0.810805381
LOC112532490	0.553083144	-0.878905791	2.325628027	0.12725885	0.325162171
LOC112533197	0.377139984	-0.876421576	0.894078324	0.344374508	0.581257825
MIR1780	-0.133040723	-0.876307807	0.15120289	0.697388474	0.842611597
ADAMTSL5	0.020726257	-0.87626307	0.001528008	0.968818842	0.98723768
LOC107051653	-0.419601869	-0.87624454	1.277932122	0.258283877	0.492703592
LOC112530225	-0.829602152	-0.875836814	5.854761109	0.015534967	0.084088117
CYP2AB1	-0.30935075	-0.875706959	0.633168949	0.4261953	0.654384964
LOC101749244	0.316802132	-0.875306856	0.868013845	0.351505748	0.587969672
LOC107054987	-0.088267769	-0.875202524	0.043664757	0.834478577	0.921720365
MIR6665	-0.423514711	-0.874578831	1.408409579	0.235320592	0.467544112
MMP27	-0.949570364	-0.873436441	5.114462909	0.02372721	0.111297388
LOC107052608	-0.677989948	-0.873411649	2.899393634	0.08861288	0.25880812
LOC101749389	-0.012391043	-0.873133751	0.001315093	0.971071682	0.988443637
LOC107053514	0.777680596	-0.872986381	4.104352199	0.042772974	0.163008754
LOC107049544	-0.390276242	-0.871801173	1.346811947	0.245836231	0.479463693
LOC107050082	0.844648786	-0.87177902	5.451918033	0.019546855	0.097853209
LOC101748959	-0.031569477	-0.870430182	0.005541086	0.940661497	0.974411302
LOC101752128	0.788702236	-0.870298754	4.316147347	0.037752304	0.150160544
LOC112532135	-0.356280549	-0.868218663	0.848082849	0.357095257	0.593652052
LOC112532678	-0.30153224	-0.865313495	0.802916996	0.370222664	0.604786453
PADI1	-0.884608159	-0.865261563	6.764968698	0.009296455	0.058903802
LINC00954	0.187114829	-0.864397675	0.279801181	0.59683156	0.779990732
DNAJC22	0.490655425	-0.8636482	1.725535688	0.188982037	0.412574387
LRRC72	-0.601968969	-0.863560461	3.028716882	0.081802684	0.245631964
MIR6637-2	-0.621091491	-0.863208399	3.811697982	0.050895831	0.181747156
LOC112532656	-0.621355233	-0.86313297	2.736307048	0.098091217	0.274842017
LOC107054377	-0.30327532	-0.863072109	0.5495988	0.45848167	0.68090502
A2ML2	-0.509149502	-0.862813198	2.0606514	0.151145546	0.362762538

LOC112530895	-0.005644142	-0.862607789	0.000305002	0.986066199	0.993435858
LOC107054122	0.701083615	-0.862162268	4.331569063	0.037411743	0.149238846
OVST	-0.161473185	-0.861197229	0.179976268	0.671393636	0.826344165
LOC107051856	-1.37832749	-0.860383474	11.10917861	0.000859015	0.010732799
LOC112530453	-0.379698089	-0.859581404	1.153960807	0.282721546	0.518406847
LOC107053274	-0.341121313	-0.858782164	0.67073358	0.412795965	0.643087944
PPP1R3A	-0.615627862	-0.857231417	2.45871316	0.116874497	0.308299111
MROH2BL2	-1.23269618	-0.854554698	9.985361108	0.001577896	0.016805359
LOC101749620	0.21086389	-0.853914933	0.325753223	0.56817051	0.761029364
CGREF1	-0.8791469	-0.853525885	6.093916081	0.013564811	0.076843124
LOC112532949	0.321933043	-0.851833605	0.729875067	0.392923687	0.626150638
GTF2A1L	0.336853678	-0.851813107	1.006220506	0.315809994	0.553298466
LOC100858040	0.027568921	-0.85002419	0.005226827	0.94236571	0.975259608
LOC112533228	0.025875333	-0.849829026	0.004333847	0.947511613	0.977802274
LOC107052575	-0.210246718	-0.849810941	0.334549069	0.56299263	0.757485178
LOC107054681	-0.111530621	-0.848276954	0.055473071	0.813799556	0.911019232
G0S2	-0.428146681	-0.848055817	1.194165907	0.274490851	0.509412101
LOC100859611	0.043353383	-0.846695896	0.01479068	0.903202396	0.955693331
LOC112532350	0.243358869	-0.843570364	0.4485335	0.503032196	0.713753107
LOC107053858	-1.259242437	-0.840529734	11.07237061	0.000876236	0.010890076
C4A	-0.094966518	-0.840403024	0.078694581	0.779074331	0.890625824
LOC101750481	0.075889151	-0.839840161	0.038280444	0.844881079	0.927205426
LOC107052079	-0.476420875	-0.839482226	1.59290369	0.206911768	0.434352468
C26H6ORF222	-0.103481363	-0.839258765	0.080753794	0.77627848	0.889259549
CA14	-0.18843713	-0.838632493	0.327835053	0.566936677	0.760490993
LOC100858177	0.084004497	-0.837672666	0.049891038	0.823252983	0.916737351
CFAP77	0.111955886	-0.835333885	0.102931361	0.748339847	0.873080841
LOC101749058	-0.139020548	-0.833867566	0.122785569	0.726032734	0.858930811
LOC107051635	-0.147356262	-0.832754991	0.126634788	0.721947046	0.85661686
CHUNK-1	-0.529290262	-0.832614603	2.054083413	0.151798579	0.363587427
LCTL	0.060783011	-0.832463835	0.029668878	0.863243693	0.936366182
LOC112533277	-0.268576838	-0.832114823	0.578232661	0.447005896	0.670631111

C4H4ORF47	-0.399053673	-0.831232431	1.515906156	0.218240067	0.447323462
LOC112529921	0.108125614	-0.831196871	0.098233938	0.753959359	0.875422686
LOC112530013	-0.259364429	-0.830356071	0.57512221	0.448230636	0.671427866
NEUROG3	0.483554124	-0.82989819	1.407477161	0.235475651	0.467739236
LOC107052410	0.170828681	-0.829572579	0.235259204	0.627651333	0.800097516
AKAIN1	-0.573114691	-0.829446033	2.13311183	0.144147858	0.352809999
MIR6516	0.348247284	-0.829416638	1.157686802	0.281945795	0.517706338
MYCBP	0.211676107	-0.828425276	0.219530256	0.639398073	0.806971567
LOC101751992	-0.057957453	-0.827261919	0.029285782	0.864120847	0.937070551
LOC112532677	-1.014451482	-0.826723474	6.350068304	0.011737678	0.069426128
AREG	0.135200305	-0.826067668	0.168387622	0.681548772	0.83319824
RLN3	0.681179227	-0.823481706	3.643996097	0.056271719	0.193787499
LOC107055394	0.732397885	-0.822143674	3.025335161	0.081973382	0.245915347
C20H20orf204	0.247594879	-0.820866592	0.456914161	0.499069686	0.710212894
PEBP4	-0.06975297	-0.820662698	0.040488073	0.840529304	0.924773315
LOC112531509	0.231571661	-0.820563988	0.460951668	0.497179546	0.708810381
LOC107053612	1.320002965	-0.819586206	12.30254263	0.000452342	0.006717763
LOC101748625	0.138220415	-0.819491703	0.12897995	0.719492014	0.855189331
LOC107054738	-0.646869293	-0.819359292	2.675791591	0.101884655	0.281728453
FBXO43	-0.199492057	-0.819276355	0.287161578	0.592045226	0.776980894
MEIOC	0.144693791	-0.818449782	0.182151871	0.669530442	0.825005498
LOC112532483	0.465506219	-0.81693634	1.983994728	0.158970202	0.37396064
LOC107054633	0.10154159	-0.815395203	0.075182295	0.783935615	0.893823486
LOC107056765	-0.737519804	-0.812493338	2.957476191	0.085481272	0.252704255
CCDC69	-0.373964042	-0.810789445	1.011493628	0.314545278	0.552148767
LOC112532217	-0.179256652	-0.810658354	0.288301465	0.591311053	0.776786155
LOC107053940	-0.024032824	-0.809932386	0.005192938	0.942552534	0.975391607
ADMP	-0.97389252	-0.809835532	7.870783592	0.005023988	0.038292634
S100A4	0.471141481	-0.809236238	1.526928959	0.216573939	0.445576915
LOC112532213	0.032801491	-0.808774806	0.008665657	0.925832452	0.967012404
CYSP1L	0.472575051	-0.808623684	2.074107674	0.149817536	0.361155518
LOC101749929	1.056260124	-0.808487894	6.777732621	0.009230207	0.058593149

LOC112532891	0.224489589	-0.808221227	0.531371097	0.466030992	0.686100024
PPP1R42	-0.247197113	-0.807805786	0.566663297	0.451587888	0.674958899
LOC107052075	0.378579077	-0.807427674	1.193294554	0.274666012	0.509655979
LOC112533011	-0.728003027	-0.804688935	3.909245415	0.048021154	0.174743055
LOC107051625	0.039357656	-0.802864398	0.013897673	0.90615609	0.957047398
SERTM2	0.662981975	-0.800198511	2.78257686	0.095294732	0.270386182
LOC112530894	1.034221866	-0.798804271	4.109887152	0.042633207	0.162654631
IL23A	-0.472878839	-0.7986081	1.814203772	0.17800477	0.398874371
LOC107054560	0.121492779	-0.798069448	0.125829966	0.722795472	0.857066913
LOC107053654	-0.197899977	-0.797231893	0.237347051	0.626128815	0.799103476
LOC107054930	0.282272267	-0.796986539	0.613665863	0.433410877	0.659982971
PI16	-0.444907512	-0.796744052	1.581558721	0.208536315	0.436184704
MIR1744	-0.6757682	-0.79639217	3.580935173	0.05844624	0.199034123
LOC107053745	-0.301678185	-0.796345783	0.825587248	0.363551634	0.599363003
FAM194A	-0.463850617	-0.796306272	1.245112775	0.264487968	0.499779071
FFAR4	-1.206911533	-0.796291251	12.62165917	0.000381303	0.00590248
ADRA1A	-0.521504109	-0.792991222	1.87951293	0.170389517	0.389194822
MIR1680	0.182882743	-0.791829495	0.234650044	0.628097123	0.800200405
LOC112532824	-0.446611826	-0.791575959	1.399993272	0.236724697	0.469065191
LOC101748467	-1.691342866	-0.790421616	15.19931613	9.67E-05	0.00201753
LOC112532471	0.120651359	-0.789869771	0.118557225	0.730604594	0.862046364
LOC112532584	-0.069023987	-0.788625898	0.024595889	0.875378225	0.94349569
LOC101747663	0.430864286	-0.787925155	1.584472044	0.208117706	0.43586316
LOC112532995	0.011090291	-0.787328417	0.001016336	0.97456773	0.989871494
LOC107054170	0.198049209	-0.787041249	0.347211981	0.555695619	0.752912164
LOC112530157	0.463357996	-0.786702414	1.945369255	0.163086733	0.379467221
LOC101749505	-0.249795009	-0.786565756	0.579459588	0.446524223	0.670621395
LOC112530208	0.057750464	-0.785837078	0.020088384	0.887290515	0.949185114
LOC107051753	-0.171157843	-0.785065693	0.20220899	0.652943691	0.814861243
NMS	0.662724742	-0.783558292	3.495704759	0.061528214	0.206078422
MYH1A	-0.58235963	-0.783119778	1.784632088	0.18158156	0.403693205
REN	0.282804191	-0.782699146	0.420500374	0.516687473	0.722783697

LOC112532354	0.187642483	-0.782672097	0.267965595	0.604699745	0.785222088
MIR7452	0.659658742	-0.780487083	3.612232164	0.057356088	0.196541945
LOC112532646	-0.273572722	-0.779834549	0.420858733	0.516508864	0.722717913
TMEM125	0.15139027	-0.778459652	0.142261055	0.706043484	0.847788218
LOC101748032	-0.375021481	-0.778347081	1.37287808	0.241318005	0.474135853
LOC112530785	-0.047606862	-0.777887313	0.018431823	0.892007968	0.951008202
MIR125B2	0.123246859	-0.777236843	0.140067664	0.708213755	0.848944796
LOC107051792	0.904902998	-0.775618731	5.811503098	0.01592168	0.085786262
LOC112532384	-0.281362351	-0.775583617	0.501081005	0.479025523	0.695155214
SERPIND1	0.051517499	-0.775135265	0.020546522	0.88602121	0.948791653
LOC771190	0.004611293	-0.774984069	0.00019222	0.988938213	0.994924082
LOC101750379	-0.154610779	-0.774705903	0.231513529	0.630403833	0.801583732
AQP12A	0.58593376	-0.77457858	2.870483296	0.090217774	0.262036199
LOC100859643	0.250611882	-0.774123445	0.578078879	0.447066326	0.670660625
LOC112532131	-1.104518592	-0.772887023	3.981527648	0.046001825	0.17002696
TMEM182L	-0.131296956	-0.772846875	0.132676482	0.715672953	0.852929561
LOC112530348	0.216671476	-0.772567067	0.322216128	0.570278867	0.762549874
LOC112533451	-1.00931959	-0.769182804	5.777294276	0.016234514	0.086959286
LOC107054203	0.581261771	-0.768332862	2.388772518	0.122209554	0.316736212
LOC107053458	-0.312255786	-0.766474714	0.516051102	0.472531048	0.691022409
ADH1L	-0.071214655	-0.765935181	0.033349835	0.855096683	0.932526733
LOC107055318	0.937792244	-0.764627448	6.611164061	0.010134139	0.062526159
LOC112533231	0.434862292	-0.763732031	1.238358859	0.265787576	0.500977614
LOC107054829	0.214091703	-0.763581583	0.378300027	0.53851444	0.739658146
LOC112530956	-0.165209595	-0.75673609	0.198266355	0.656123848	0.816485578
TACSTD2	1.052263191	-0.756277725	5.171460468	0.022960904	0.108735465
LOC112530405	0.031760121	-0.756125633	0.009109902	0.923960733	0.965809545
LOC770277	0.33381281	-0.755706946	0.854735266	0.355216196	0.591907089
SPDEF	-0.388910496	-0.754558788	1.12476746	0.288894207	0.525392814
NR2E1	0.477313101	-0.754110964	1.999137442	0.15738875	0.371941961
LOC100857439	-1.114057278	-0.753793015	11.32038687	0.000766607	0.00987825
PCDHGA2	-0.149277257	-0.752783999	0.191650374	0.661546561	0.820458761

LOC112531728	0.14864473	-0.752523408	0.121442266	0.72747547	0.859710489
YJEFN3	-0.001402872	-0.752433045	3.73E-05	0.995128312	0.99763501
S1PR4	-0.448857793	-0.752351376	1.707630912	0.191293036	0.415364966
LOC107053649	0.114624576	-0.752047089	0.128039879	0.720473079	0.855792151
CHRM5	-0.46525741	-0.751712721	1.390595437	0.23830457	0.470995489
IL17B	-0.340402641	-0.748833097	0.564721359	0.452364166	0.67549848
LOC107056935	0.829475746	-0.747608032	4.411979681	0.035687403	0.144925028
LOC107053930	-0.359939787	-0.746889511	1.146521716	0.284278462	0.520218204
LOC107052561	-0.210308431	-0.74670564	0.416731537	0.518572466	0.724065331
OVSTL	-0.289383913	-0.746528444	0.565621209	0.452004197	0.675359036
TOR1BL	-1.3055788	-0.746296691	14.13188718	0.00017043	0.003151042
MROH2B1	1.586959819	-0.745654855	19.18099925	1.19E-05	0.000373695
LOC107054931	0.44413283	-0.745264264	1.764617801	0.184049574	0.406733244
CCR9	-0.600054605	-0.744830352	3.361206584	0.066749145	0.216817351
LOC107054472	0.710534605	-0.743885525	4.737418016	0.029513334	0.127899143
LOC112530333	0.182566336	-0.743734429	0.271899257	0.602060571	0.783443547
LOC107055039	-0.646356229	-0.743657653	2.726689904	0.098683623	0.27564743
HSD11B1b	0.508291208	-0.743194741	1.856292599	0.173053298	0.392392451
KHK	-0.040097065	-0.742877876	0.015175166	0.901958604	0.955177611
LOC107053695	0.753016905	-0.742753356	4.30664914	0.03796367	0.15049741
LOC107054557	-0.673263654	-0.740647942	3.321011522	0.068399733	0.219956537
LOC107054070	-0.464895688	-0.737799436	1.771887418	0.183148669	0.405613909
LOC112532106	-0.554908292	-0.737648918	3.236984163	0.071993047	0.226683044
P2RY13	0.014855677	-0.737541028	0.001861532	0.965585566	0.985815656
LOC107051881	-0.268169736	-0.737288623	0.526092859	0.468254151	0.687705188
LOC107055056	0.27052738	-0.736436693	0.651107284	0.419717075	0.649223958
MIR124-3	-0.217424733	-0.736157472	0.279993863	0.596705239	0.779887586
KMO	-0.018397565	-0.735773807	0.002650872	0.958937766	0.982278445
LOC112531598	0.188189662	-0.731289236	0.283712652	0.594278075	0.778013025
LOC107053816	-0.019752041	-0.731103831	0.003633447	0.95193416	0.979652938
LAMP3	0.117305622	-0.730677637	0.108073183	0.742348544	0.869233992
LOC112530344	0.028927672	-0.730171802	0.008828004	0.925142949	0.966492745

LOC107053136	-1.053440475	-0.729309795	7.861239727	0.005050575	0.038439969
GPR83	0.204110008	-0.727766797	0.295092503	0.586975465	0.773482167
LOC107052983	-0.881274332	-0.726862336	5.947903099	0.014734804	0.0810907
LOC101751607	0.12483998	-0.726619832	0.124188508	0.724535378	0.857961258
SSTR3	-0.733382526	-0.726254962	3.559233537	0.059215075	0.200531809
LOC112533341	-0.349073414	-0.725962022	1.067499395	0.301510957	0.539337189
GRB14	-0.381513524	-0.725327682	1.436504414	0.230705841	0.462170028
DUSP13L	0.213389147	-0.724357271	0.392271855	0.531107629	0.733723117
LOC101749793	-0.419858902	-0.724247504	1.467017751	0.225816815	0.4557507
HGFAC	0.531096797	-0.723897899	2.164999089	0.141184707	0.348096246
LOC107055575	0.226354099	-0.72364998	0.352998439	0.552420674	0.750843039
LOC112532528	0.518808718	-0.722400779	1.748080357	0.18611824	0.408888604
LOC107052206	0.114045215	-0.722332104	0.099792706	0.752078537	0.874404884
LOC112532190	0.005235167	-0.722149851	0.000534252	0.981559423	0.991828264
LOC107052058	0.399634843	-0.720686125	1.577357987	0.209141665	0.43679089
LOC107052939	-0.140340227	-0.719839487	0.180826569	0.67066386	0.825783734
LOC107053496	-0.308954875	-0.719383392	0.787781978	0.37477165	0.60943448
CALB1	-0.391099799	-0.718875331	0.915621135	0.338627352	0.57576162
C6H10orf107	0.626482006	-0.717271296	2.369286573	0.123743592	0.318853868
CFAP161	1.076484608	-0.71654484	9.036249739	0.002646781	0.02416913
LOC112530650	-0.655520985	-0.716517658	3.934556149	0.047303622	0.17308897
MIR6575	0.339451499	-0.716297246	1.022596787	0.311903835	0.549771677
LOC107054420	-0.001076209	-0.715945558	9.33E-06	0.997563347	0.998889311
LIPML1	-0.905855807	-0.715544257	5.997966262	0.014322379	0.079566087
PLA2G2A	0.733916242	-0.715367334	5.483974344	0.019191576	0.096574125
LOC107052316	0.224947868	-0.71462348	0.458596191	0.498280777	0.709765425
FNDC9	0.429393576	-0.714556264	1.129735267	0.287831814	0.524620348
LOC423719	-0.141308187	-0.714455351	0.197908071	0.656414716	0.816610278
LOC112530207	-0.162594679	-0.714410011	0.172334546	0.678045303	0.830532564
LOC101748858	-0.450866153	-0.7143856	1.567931541	0.210507659	0.438234642
LOC112530474	-0.235433069	-0.714192741	0.487288992	0.485139122	0.699830301
CASP18	-0.117054185	-0.713356016	0.100044927	0.751775726	0.874361717

LOC107056428	-0.167495142	-0.712652891	0.205039815	0.650683241	0.813613209
LOC107053162	-0.605490235	-0.712513676	3.106723152	0.077969682	0.238280562
LOC112531786	0.006424298	-0.712114015	0.000334538	0.985407196	0.993236215
LOC107052349	0.541255181	-0.71177242	2.015611246	0.15568856	0.369116269
SLC5A11	-0.076878113	-0.710455154	0.037723358	0.845999693	0.927917895
COL6A6	-0.344160105	-0.710284596	0.894095089	0.344369985	0.581257825
TROJANZ	-0.425209084	-0.709432674	1.550147132	0.213113722	0.441463576
LOC101751009	-0.916960296	-0.709022102	5.506537263	0.01894552	0.095716465
SI	0.145635936	-0.708087275	0.164600951	0.68495538	0.835563601
LOC107054022	-0.392344085	-0.707383675	1.19510404	0.274302423	0.509383408
LOC107052992	-0.291244976	-0.707148408	0.822801551	0.36436233	0.600046653
LOC101748878	0.772566074	-0.706683969	5.294691208	0.021390523	0.103862114
LOC112532140	0.278309189	-0.705400747	0.609970649	0.434798862	0.661222386
CD1B	-0.689924379	-0.70470316	2.072167574	0.15000819	0.361281572
LOC107053503	1.081571563	-0.704229392	5.747596374	0.016511233	0.087983271
LOC101747644	-0.920691393	-0.703839543	8.810324683	0.002995307	0.026399454
BLA	-0.193596065	-0.702403572	0.264989601	0.606712769	0.786666377
LOC107054732	-0.65086281	-0.702206732	2.099414426	0.147355563	0.35717587
TMEM238	-0.094503374	-0.701690807	0.096346888	0.756258352	0.87704156
LOC107054882	-0.374032559	-0.701395285	0.905309337	0.341362061	0.578399406
LOC107052648	-0.375360738	-0.700108992	0.914955777	0.338802916	0.575810987
LOC101750880	-0.783406466	-0.699025644	5.454633763	0.019516495	0.097760706
ARHGEF33	-0.158808642	-0.698474671	0.258392272	0.611226843	0.788965932
LOC112529999	0.149929682	-0.69579019	0.213280116	0.644208541	0.810184313
LOC112530146	-0.091131247	-0.695560944	0.091466764	0.762320685	0.880756463
LOC107053839	-0.656476455	-0.694987446	2.997021414	0.083417749	0.248846819
LOC107050695	0.167790009	-0.694468536	0.238231753	0.625486163	0.798796471
THT2L	-0.915560875	-0.694371344	6.009533159	0.014228791	0.079236332
LOC112530199	-0.286247938	-0.693847687	0.816723582	0.366139858	0.60134059
LOC112531245	-0.745664084	-0.693485997	2.828421882	0.092609322	0.265995181
LOC101750610	0.22766081	-0.693286684	0.339146983	0.560322014	0.755498094
GC1	-1.186783932	-0.69295522	6.664790742	0.00983362	0.061408812

LOC112532215	0.675236044	-0.692315825	3.108802761	0.077870181	0.238215389
HAPLN2	0.051057888	-0.692073026	0.020842551	0.885208705	0.9486918
LOC107053509	-0.75616436	-0.691978393	3.379264775	0.0660215	0.215466837
RGSL1	0.200853966	-0.691575594	0.326708432	0.567603741	0.760792527
LOC107052507	0.006708108	-0.691529805	0.000184874	0.989151624	0.995057477
OLFR1019L2	-0.76478842	-0.690556159	4.453138866	0.034837158	0.142552294
LOC112532435	-0.389306906	-0.688373163	1.401917297	0.236402816	0.468732178
LOC112532629	-0.76915356	-0.688305701	3.015948347	0.082449216	0.246807686
LOC107053332	-0.134063931	-0.686007118	0.157598659	0.691377065	0.839044886
HEATR7B2L1	0.67670232	-0.685800225	2.681097005	0.101545755	0.281117424
FMO5	-0.97307043	-0.685717279	6.891471588	0.008660794	0.056173972
GDF9	0.225992158	-0.683522954	0.435677771	0.509216023	0.717892027
IFNAL4	0.714061893	-0.683262201	2.975426015	0.084537867	0.251095778
RHBDL1	0.410561779	-0.68291427	1.808110765	0.178735028	0.399712541
LOC107054411	0.091155785	-0.682702381	0.081966991	0.774649294	0.888778004
LOC112531855	-0.406077821	-0.682191454	1.274272112	0.258966762	0.493434237
LOC101750974	1.769949685	-0.681639431	21.9225482	2.84E-06	0.000113932
LOC101751606	-1.121047041	-0.681613099	9.834818345	0.001712392	0.017845098
LOC112531489	0.336918588	-0.681555556	0.800741984	0.370871621	0.60536579
LOC107054407	0.636662822	-0.680897968	2.269645054	0.131930115	0.333300054
LOC112532918	-0.557977568	-0.680296881	2.298106316	0.129531836	0.329180906
MMR1L3	-1.078304594	-0.67973163	4.83011032	0.027966841	0.124008184
LOC107050519	-0.380742296	-0.678737769	1.099526377	0.294370068	0.531297924
LOC771995	-0.37982784	-0.6765039	1.028421917	0.310529602	0.548336682
LOC112530148	0.799217837	-0.675561616	4.804847721	0.028379775	0.124896816
KCNJ1	-0.379231658	-0.674524219	1.000211679	0.317259293	0.555139665
LOC101747803	-0.296657918	-0.674498123	0.501416612	0.478878336	0.695002912
LOC101749989	-0.821365244	-0.674364966	7.024011987	0.008042383	0.053336266
LOC107052573	-0.35629888	-0.673893885	1.256556529	0.262303901	0.497574203
LOC112532206	-0.887171069	-0.673880297	6.011643734	0.014211783	0.079236332
LOC107053448	0.094512942	-0.673773834	0.084323685	0.771521405	0.88652336
ABCA13	-0.954170524	-0.673185584	8.954640635	0.002767656	0.024940736

LOC101751313	-0.743930525	-0.672178519	4.688359971	0.030367619	0.130469757
LOC107054031	-0.238527673	-0.670610172	0.297172335	0.585660567	0.772343452
GIMAP7L6	-0.184508407	-0.669781339	0.283976443	0.594106681	0.777946576
KRT23	0.021660362	-0.669410142	0.003257882	0.954483171	0.980523198
LOC101749231	-0.237861277	-0.668927862	0.491771908	0.48313796	0.697948254
CDHR1	1.275414095	-0.665778985	10.56603041	0.001151848	0.013292521
HOPX	-0.052388275	-0.665721075	0.026968864	0.869556387	0.939867994
LOC112532643	-0.898711401	-0.665576482	7.366381472	0.006645453	0.046651417
IGFBP1	0.069675268	-0.665023326	0.042139858	0.837353407	0.923303231
SPERT	0.806452548	-0.664137387	4.504522858	0.033805327	0.140046991
LOC112531858	-0.198756532	-0.663909639	0.362004145	0.547395286	0.746757539
LOC100858984	0.290425946	-0.662598751	0.81739849	0.365941886	0.60110042
LOC101749095	0.319774124	-0.661180113	0.775291819	0.378584948	0.613271837
LOC112531578	-0.435293226	-0.661055756	1.727359636	0.188748448	0.412354715
CAPN13	-1.194497377	-0.660732253	10.54511294	0.001164957	0.01338643
LOC101748554	-1.309224052	-0.659648131	15.01385002	0.000106725	0.002155577
LOC112530016	-0.056140089	-0.65869863	0.03371635	0.854311475	0.932178863
NCF2	-0.096180522	-0.6582746	0.077886391	0.780182407	0.891500492
LOC101747446	-0.573102169	-0.657831045	3.327605302	0.068125998	0.219439538
RANBP3L	-0.250067846	-0.657661062	0.529602921	0.466773848	0.686608642
RUNX3	0.401868828	-0.657402368	1.506116674	0.219732605	0.448987957
LOC101747501	0.677632825	-0.656849436	4.540407849	0.033103704	0.138394676
LOC101747354	-0.929714941	-0.656632157	8.341748235	0.00387443	0.031749872
NKX1-1	-0.038036106	-0.656314668	0.015621881	0.900533428	0.95453991
LOC107051908	-0.055624388	-0.656126692	0.031841891	0.858375112	0.933836045
LOC112530999	0.021355401	-0.655580101	0.004734473	0.945142844	0.976598148
MIR1464	-0.581497395	-0.652173101	3.065356232	0.079977631	0.242273917
LOC101749621	1.163147255	-0.651835985	10.34655493	0.001297166	0.014472241
SH2D2A	-0.497262943	-0.651805881	1.894093421	0.168740946	0.387094976
GABRA5	0.104293155	-0.650758307	0.061056012	0.804834589	0.90566365
CLDN34	0.413374451	-0.648302855	1.836405596	0.175372818	0.395038977
LOC107049724	1.839777611	-0.648231744	19.55386835	9.78E-06	0.000318681

LOC107052300	0.131660644	-0.647896656	0.158766235	0.690294964	0.838473731
LOC416951	-1.079760069	-0.647787597	8.465222419	0.00362001	0.030345014
RBP4	0.763134574	-0.647725829	4.097070998	0.042957572	0.163356737
LOC101752108	-0.227632654	-0.647254772	0.399515334	0.527339665	0.731218858
LOC107052695	-0.916726247	-0.64690169	5.132740207	0.023478633	0.110383115
PTPN22	-0.450724102	-0.64675077	1.829469784	0.176190185	0.396283419
BCL2L14	-0.270933491	-0.646674007	0.653435666	0.418887011	0.648610687
CFAP100	-0.339475048	-0.646421137	1.369149922	0.241957993	0.474826329
QRFPR	0.174188839	-0.646287943	0.200070588	0.654663876	0.815727251
SLC22A18	0.282551278	-0.64625927	0.819780546	0.365244337	0.60086122
LOC112533582	-0.177415453	-0.645842445	0.2705744	0.60294672	0.784121416
GIF	0.38725779	-0.645472794	1.488412041	0.222463004	0.452027755
LOC100859449	-0.400874181	-0.645341867	1.755302956	0.185211463	0.407836809
LOC107054094	-1.354515009	-0.645230154	10.84852501	0.000988745	0.011928003
LOC428505	2.202832121	-0.645183771	28.24581338	1.07E-07	7.17E-06
GRM1	-0.684462367	-0.644687485	4.088150584	0.04318487	0.163790274
LOC112532291	-0.226627118	-0.644457628	0.575300264	0.448160386	0.671427866
NRSN2	-0.960491436	-0.644066634	8.06801412	0.004505347	0.03543761
LOC112530782	0.030756895	-0.643442384	0.008389128	0.92702206	0.96741172
LOC419545	-0.179175365	-0.642881769	0.245316583	0.620392294	0.795058816
CCLI7	0.024036906	-0.642526321	0.002710675	0.958477581	0.982173907
VSIG1	1.575920969	-0.642504952	14.28824294	0.000156841	0.002932757
CLC2LL	-0.405908589	-0.642397443	1.264844002	0.260736175	0.495543284
MIR3594	0.009931035	-0.642014957	0.000943191	0.975499688	0.990018742
LOC112532273	0.224613379	-0.641607694	0.480959282	0.487988122	0.702056988
LOC112533343	-0.636253531	-0.641052147	3.18061098	0.074516862	0.232142173
FAM180B	-0.648512703	-0.639936329	3.338292553	0.067684812	0.218726154
SLC5A10	0.303994269	-0.639138716	0.844244246	0.358185729	0.59444742
CPLX3	-0.618868733	-0.638192531	4.067727139	0.043710051	0.164914059
LOC107054939	0.698293518	-0.637569023	4.35499173	0.036900638	0.148097562
AQP10	-0.365332901	-0.636733237	1.492978641	0.221754881	0.451179047
LOC112532518	-0.205182027	-0.636704937	0.317885574	0.572881117	0.764662458

LOC112531693	-0.07654745	-0.636212033	0.048090901	0.826419184	0.917837275
GABRQ	-0.54495217	-0.635551066	2.395858717	0.12165693	0.315701747
SLC2A11L1	-0.482776764	-0.6354526	1.960176849	0.161494406	0.37728194
KBTBD12	0.126821507	-0.635213985	0.144351846	0.703992453	0.846674156
C3H2ORF70	0.395239111	-0.634833145	1.384505518	0.239335182	0.471874002
PRLH	-0.908320135	-0.634662811	4.45083965	0.034884091	0.142648538
SELPLG	-0.225333913	-0.634068879	0.429217543	0.512373138	0.719929979
LOC112533272	-0.096183093	-0.634020262	0.097031878	0.755420996	0.876495029
LOC431648	-0.535508569	-0.633741684	3.37335694	0.066258617	0.216067281
LOC107053964	0.425797092	-0.633563717	1.227425994	0.267908206	0.503073094
LOC112530188	-0.325140074	-0.632938705	1.121972157	0.289494194	0.526135073
C2orf50	1.76237836	-0.632899381	22.80234937	1.80E-06	7.73E-05
LOC107053254	-0.11668453	-0.630378201	0.072538601	0.787675817	0.895442461
MIR7444	0.19415979	-0.630111417	0.372695378	0.54153871	0.742118605
CCDC92B	0.306985739	-0.629332066	0.668856224	0.413450664	0.643645286
LOC112530191	-0.962204574	-0.628988441	5.958822744	0.014643816	0.08076448
LOC107055347	0.200667085	-0.627752683	0.379882853	0.537665938	0.738813613
LOC107053204	0.277026983	-0.627284663	0.664514063	0.414970818	0.645405182
LOC112530198	-0.406019561	-0.627154679	1.959024731	0.16161766	0.377383085
MIP	-0.406892843	-0.627096953	1.469856499	0.225368336	0.455246253
LOC426820	-0.921315276	-0.625742166	5.538719008	0.018600193	0.094552418
LOC112530519	-0.917408814	-0.625472394	8.379656228	0.003794442	0.031312709
LOC107052166	-1.305444453	-0.625394227	15.51800653	8.17E-05	0.00178113
SULT1B	0.009764914	-0.625065	0.001009613	0.974651956	0.989871494
LOC112532675	0.112966478	-0.623975747	0.085159604	0.770423347	0.885411103
LOC112531534	0.597110642	-0.623746881	3.100544727	0.078266102	0.238715238
CNR2	-0.604806945	-0.623641008	3.337244662	0.067727935	0.218779578
LOC107053720	0.329672285	-0.623439035	0.922885883	0.336718373	0.573918636
LOC107057254	0.327263189	-0.623402403	0.720166702	0.396089233	0.62893451
HIST1H2A4L2	-0.056032544	-0.621051538	0.023806921	0.877377206	0.944660206
LOC107052244	-0.453339137	-0.620491707	1.770817291	0.183280965	0.40570577
TLR3	-0.805769099	-0.618954586	6.181899111	0.012906368	0.074256743

LOC415414	-0.583223052	-0.618568542	2.608048883	0.106322503	0.290287555
LOC112532125	-0.269458632	-0.616856439	0.524261423	0.469029519	0.688269654
CACNG1	-0.278409649	-0.616001596	0.748221967	0.387039751	0.621219187
LOC107054145	-0.901853399	-0.61587017	4.182936205	0.0408329	0.158588622
LOC112533525	-0.3523495	-0.615670759	1.127231555	0.288366624	0.525060454
LOC107050668	2.085805259	-0.615166586	20.40741534	6.26E-06	0.000222433
ALDH3B1L2	-0.106975659	-0.614974736	0.138203883	0.710073165	0.849641566
FGF6	-0.547844291	-0.614547912	2.438945122	0.118355794	0.31056364
LOC101751481	-1.197364056	-0.613504167	11.34528085	0.000756399	0.009797586
LOC107051996	0.68911692	-0.613372538	3.026252048	0.081927063	0.245826006
LOC107052164	-1.64387803	-0.613134025	22.05470618	2.65E-06	0.000107399
LOC107052456	-0.21003114	-0.613088228	0.268528302	0.604320712	0.785137502
STOML3	0.040301701	-0.612732911	0.014551392	0.903984777	0.956164575
LOC112532205	0.42486699	-0.612382737	1.696822544	0.192704044	0.41714129
LOC107052665	-0.07625829	-0.612131854	0.035511484	0.850527897	0.930485775
AGPAT1	-0.925855352	-0.61205649	6.045802461	0.013939396	0.078337692
MGST2	0.138229383	-0.611184477	0.145235419	0.703130791	0.846380187
PRLHR	-0.023103255	-0.610837238	0.003472855	0.953007121	0.979802073
GABRE	-0.238630678	-0.609321758	0.50586685	0.47693356	0.693600685
LOC107055073	-0.313693541	-0.608588658	0.877854765	0.348789916	0.5854078
LOC101751717	0.111450337	-0.607937252	0.126589711	0.721994484	0.85661686
LOC112531817	-0.961794892	-0.607833633	6.18781366	0.012863301	0.074164548
LOC107054193	-0.126113105	-0.607664777	0.135163667	0.713137124	0.850955139
LOC107055072	-1.683842023	-0.607223469	22.58981152	2.01E-06	8.48E-05
GSC2	0.619311534	-0.607112383	4.005479312	0.0453526	0.168535916
MIR6642	0.565567413	-0.607107758	2.939766579	0.086423221	0.254776398
LOC112533411	-1.478270836	-0.60698907	15.29212441	9.21E-05	0.001942949
SLC15A1	-0.362835225	-0.606697483	1.390014044	0.238402728	0.470995489
IL17F	0.4421771	-0.60655744	1.66040472	0.197548688	0.423263498
LOC107054249	-0.227525551	-0.606506155	0.433323472	0.510362661	0.718341124
LOC101747538	0.226078253	-0.605721581	0.44359755	0.505391163	0.715440088
LOC101751730	0.8264115	-0.604597378	4.254338522	0.039150192	0.154045053

CRCBL	-0.926211179	-0.604219094	5.042151964	0.024737741	0.114127827
LOC107052640	-0.706563309	-0.602487121	4.597612175	0.032016519	0.135293225
LOC101749898	-0.534452023	-0.601460696	2.11752548	0.145621657	0.354782998
TESPA1	0.098053935	-0.601371823	0.09648505	0.756089196	0.877026984
ACTBL2L	-0.139261175	-0.601129733	0.170163426	0.679966607	0.832132271
MHCIA3	-0.811654289	-0.600423158	6.183121939	0.012897452	0.074256743
LOC107051587	0.795570608	-0.600239954	3.459311918	0.062896483	0.208973648
LOC107052858	-0.154197727	-0.599436303	0.226846871	0.633871747	0.80403489
IL17D	0.265841439	-0.599358036	0.650591295	0.419901356	0.649386918
LOC112532128	0.171342177	-0.599087338	0.297022699	0.585754969	0.772385449
LOC112530304	-0.337503934	-0.598612089	0.85265834	0.355801392	0.592239624
LOC107051969	-0.703328884	-0.597626013	4.873923478	0.027265443	0.121841837
POU2F3	0.021976458	-0.597221174	0.003565014	0.952388409	0.979802073
LOC101748744	0.05282103	-0.597045583	0.026975662	0.869540093	0.939867994
LOC107054903	0.220555981	-0.596859005	0.422413352	0.515735278	0.722248851
IL18BP	0.636985752	-0.596432796	4.3685377	0.036608395	0.147383256
FER1L5	0.006258384	-0.594800305	0.00031907	0.98574852	0.993345211
P2RX7	0.537715303	-0.594782572	2.605716042	0.106479055	0.29042149
CCDC166	0.406917467	-0.594719396	1.11135872	0.291786784	0.52883963
LOC112532321	-0.149711153	-0.594522316	0.123997738	0.724738427	0.858016463
ENTPD8	-0.642100285	-0.594277682	3.426170138	0.064170752	0.211681257
LOC112531968	-0.223873608	-0.59416486	0.211940779	0.645250476	0.81068921
LOC107054729	-0.010469027	-0.594000935	0.001285023	0.971404178	0.988647684
LOC112532063	0.621878536	-0.593099603	3.118799943	0.077393762	0.237684649
LOC107054905	-0.498317612	-0.592336171	1.815081869	0.177899812	0.398766028
LOC112530826	-0.414056302	-0.592250147	1.158785879	0.281717481	0.517430645
LOC112530346	0.294312462	-0.591745866	0.72512971	0.394466409	0.627445382
MSS51	0.698590033	-0.591692584	4.553192134	0.032857435	0.137679931
LOC107055020	0.152592659	-0.589766788	0.199694637	0.65496744	0.815920147
XG	0.651556005	-0.589731593	2.525226331	0.112038704	0.29977185
LOC107053884	-0.344982738	-0.589500894	1.24385584	0.264729231	0.499885477
LOC107054189	-1.325888385	-0.588646364	12.9259851	0.000324052	0.005214537

LOC107052654	-0.67598056	-0.587209367	2.949116832	0.085924503	0.253566659
LOC100859304	-0.072896682	-0.586558005	0.026664219	0.870288673	0.940243379
LOC107052897	-0.609990334	-0.585132229	3.088473304	0.078848755	0.239970995
ABCB1	0.184253219	-0.585128529	0.255728588	0.613069977	0.790261455
ADGRG5	1.639816871	-0.583246273	25.77582172	3.83E-07	2.16E-05
ACE2	0.659367277	-0.582734917	2.466601375	0.11628913	0.307120817
HIST1H111L	-0.606344626	-0.582711758	3.105854128	0.078011302	0.238335679
LOC107052464	0.306764854	-0.582645918	0.952640793	0.329048016	0.566653518
RBP1	0.043807485	-0.582237713	0.02140307	0.883686239	0.947979469
LOC107053831	-0.239522267	-0.581519574	0.452302818	0.501243407	0.712013144
LOC112530087	0.79437466	-0.581411849	4.160044494	0.041388317	0.159710841
LOC107052196	0.125816336	-0.580592432	0.160035775	0.689123582	0.83810791
CCNA1	0.139257409	-0.580192603	0.20682834	0.649264758	0.81275402
ANGPTL5	-0.902010137	-0.580054008	4.361670289	0.03675625	0.147662131
LOC112530777	-0.163686913	-0.579837919	0.244666867	0.620855591	0.795404761
CLDN20	0.438372053	-0.579283342	1.369486437	0.241900141	0.474797763
UGT2A1	0.527881078	-0.57886958	2.87024866	0.090230928	0.262036199
LOC107052007	0.118895685	-0.578534227	0.127883542	0.720636627	0.855792151
LOC107052895	-0.422553642	-0.578502491	1.302064729	0.253836421	0.488454682
GLRA1	-0.150510446	-0.578078246	0.1622795	0.687066449	0.837023649
LOC101750779	0.390883273	-0.577245527	0.803114229	0.370163894	0.604750484
LOC101750184	0.205605138	-0.575801681	0.345650286	0.556585786	0.75311398
LPAR3	0.802380631	-0.57442595	4.519974009	0.03350134	0.139436135
LOC112533185	-0.217312155	-0.573915468	0.515700481	0.47268152	0.691022409
MIR181B2	-0.232808045	-0.572931926	0.548448593	0.458952292	0.681397294
LOC112529974	-0.314078788	-0.571562975	0.63875798	0.424160909	0.65309226
LOC101750827	0.164597646	-0.571486001	0.266229835	0.605872118	0.786214274
LOC112532884	0.799023187	-0.571192218	5.465233364	0.019398463	0.097406688
GZMA	-0.206775027	-0.569415898	0.308910417	0.578349568	0.767850745
PCDHB14L2	0.518537076	-0.569313015	1.503976788	0.220060481	0.449434679
LOC101751578	-0.13186681	-0.569292871	0.146534694	0.701869171	0.845541929
LOC101751418	0.206112579	-0.568720048	0.371755854	0.54204873	0.742418166

TEX12	0.24504926	-0.568561869	0.687397874	0.407050873	0.637801433
LOC112532537	-0.147216185	-0.567294154	0.254988943	0.613583911	0.790643951
LOC107054723	0.120384429	-0.565688107	0.125307813	0.72334755	0.857066913
LOC107054462	-0.123084816	-0.565675421	0.112073157	0.737796109	0.866307619
LOC107053498	0.177498448	-0.564719013	0.248131825	0.6183936	0.793919535
LACC1	0.139576711	-0.563936207	0.184064677	0.667903143	0.824166458
LOC112532267	-0.404383216	-0.563348785	1.591900265	0.207054849	0.434352468
ANKRD34C	0.125077688	-0.562649437	0.138411656	0.709865175	0.849641566
AANAT	-0.291844263	-0.560890489	0.846098815	0.357658312	0.59400061
LOC107054526	0.902628954	-0.560380512	9.034371582	0.002649501	0.024180556
TACR3	0.13587146	-0.559389131	0.135736696	0.712556647	0.850454908
LOC107052959	0.234061203	-0.55763708	0.361185969	0.547848315	0.747189725
FGF4	-0.006109565	-0.557247937	0.000415959	0.983728212	0.992657841
LOC107053833	-0.971980663	-0.556741298	5.768115865	0.016319522	0.087272584
SLC17A8	-0.263171949	-0.554996084	0.55689899	0.455512392	0.678137737
LOC107053531	-0.117162561	-0.554725702	0.132236583	0.716124252	0.853085451
GJA9	-0.081820828	-0.55354247	0.08007947	0.777189744	0.889536847
LIME1	-0.290262983	-0.553454974	0.887611031	0.346125518	0.582540186
LOC112533361	-0.425223754	-0.552776435	1.684610762	0.194312916	0.418892604
LOC426064	0.28828941	-0.552538213	0.768424449	0.380704903	0.615373201
LOC101747264	-0.044002434	-0.552368014	0.01647425	0.897870372	0.95334891
LOC107051652	0.31997185	-0.5520287	1.031835873	0.309727864	0.547547486
CCDC33	0.451397149	-0.550980753	1.904321118	0.167595445	0.385434388
LOC112532657	0.529374957	-0.550800561	2.322099957	0.127547733	0.325528727
LOC112529985	-0.169717705	-0.550532036	0.253611	0.61454386	0.791137476
LOC112533325	-0.314264218	-0.549158376	0.838364379	0.359864979	0.596332147
LOC107054803	-1.174010037	-0.549134633	8.93542426	0.002796925	0.025139403
LOC107055022	-1.152657567	-0.547929482	9.465376767	0.002093864	0.020835945
LOC112532533	-0.021381775	-0.547520806	0.004667883	0.945529385	0.97671734
LOC112531971	0.136468246	-0.545510707	0.198516476	0.655920978	0.816485578
BTLA	0.183490123	-0.544719913	0.317318059	0.573223869	0.764747751
LOC100859420	0.000916379	-0.544528382	1.12E-05	0.997329986	0.998885697

ZC3H12D	-0.478915574	-0.54443087	2.238073823	0.134648619	0.33764752
LITAF	-0.208649329	-0.544359925	0.472975715	0.491621377	0.704487482
FAM135B	-1.153557582	-0.544242735	8.42344772	0.003704127	0.030877111
LOC112533550	-1.013461514	-0.544217557	8.302383076	0.003959309	0.032220766
LOC100859213	-0.346585977	-0.543215416	1.516310528	0.218178675	0.447286229
LOC101748722	0.385994147	-0.542665182	1.64977541	0.198989502	0.424801797
LOC112530094	-0.448612645	-0.542329983	1.459554701	0.227000986	0.456782792
LOC112533246	1.295550379	-0.542151877	9.013462067	0.002679983	0.024377623
LOC107053938	0.261472974	-0.542038356	0.520137042	0.47078323	0.689579099
LOC112530092	0.009857757	-0.542014683	0.001071127	0.97389143	0.989715351
LOC112531292	-0.744564953	-0.541945208	5.038657579	0.024787692	0.114316554
LOC112531800	-0.770552236	-0.541306236	4.976424845	0.02569503	0.117089927
LOC107052980	0.198986248	-0.54116625	0.416771134	0.518552598	0.724065331
BTN3A2	4.588603341	-0.541043496	96.64485822	8.29E-23	8.53E-20
LOC112532365	-0.703225991	-0.541029576	3.631681948	0.056689498	0.194907166
LOC107053194	-0.459877235	-0.540969343	2.092825248	0.147992164	0.358171945
AQP9	-0.391887757	-0.540616559	1.139673337	0.285721364	0.521987903
KCNK17	0.811945625	-0.537066137	5.910666982	0.015049473	0.082217488
LOC112533473	-1.227329532	-0.536521726	14.62770433	0.000130975	0.002547508
LOC112533261	-0.917979079	-0.536030904	7.912936121	0.004908254	0.037776111
SP9	-0.879535087	-0.535852723	7.33974679	0.006744634	0.047114718
LOC112532325	0.026783947	-0.535728233	0.007979591	0.928820798	0.968392732
LOC101749139	-0.114342527	-0.535110091	0.127734688	0.720792453	0.855792151
UPK3B	0.719258441	-0.534962048	4.309056695	0.037909977	0.15049741
LOC396477	0.371802166	-0.53304638	1.269867202	0.259791594	0.494318997
LOC112530547	0.117170874	-0.532525992	0.101360078	0.750203552	0.873555895
C2H8ORF22	-0.115790375	-0.53244671	0.138593379	0.709683407	0.849639454
LOC112530232	0.162975133	-0.532347119	0.251138438	0.616274607	0.792126125
HTR2A	0.079405934	-0.531945158	0.029866483	0.862793527	0.936001285
LOC107051715	-1.339731768	-0.530914667	16.21992048	5.64E-05	0.001321977
MYO7B	-0.279925998	-0.530625442	0.74174728	0.389101736	0.623009543
MEIG1	-0.189785498	-0.530032982	0.34531174	0.556779114	0.753115441

LOC107050315	0.160149113	-0.529702989	0.300496958	0.58357104	0.770936649
LOC107054397	0.292710884	-0.529474246	0.858776899	0.354081194	0.590853468
TMEM40L	-0.498193665	-0.527602368	3.062120384	0.080137032	0.242578156
LOC107052966	-0.17505442	-0.527419639	0.211371212	0.645694779	0.81093784
LOC107054800	0.481500457	-0.52735485	2.444628184	0.117927826	0.310122056
LOC112531019	-0.05717069	-0.527281125	0.021422524	0.883633767	0.947979469
TNMD	-0.525855322	-0.527277995	2.063514961	0.150861828	0.362345843
LOC112532583	-0.370019932	-0.525010618	1.760378121	0.184577359	0.407134108
XAF1	-0.514024801	-0.524892734	3.366196295	0.066547235	0.216452808
GRXCR2	0.586372428	-0.524498744	3.890956966	0.048546754	0.175924057
LOC112530061	-0.575036375	-0.524468096	3.29178306	0.069627372	0.222469593
ALPP	1.368387244	-0.52398683	7.16814305	0.007420955	0.050355389
LOC107054089	-0.192175594	-0.523658013	0.380797738	0.537176609	0.738339554
TGM4	0.405297295	-0.523362192	1.084738912	0.297639843	0.534694288
ODF3L6	0.473249417	-0.522965403	1.453459644	0.227973634	0.458314739
RD3L	-0.334243764	-0.522397725	0.882479888	0.347523357	0.584085637
LOC112532357	-0.235235178	-0.521925267	0.395375264	0.529487381	0.733084969
LOC112531448	-0.454635224	-0.520955	1.938823711	0.163796311	0.380418954
LOC771515	-0.255550662	-0.51974997	0.651120986	0.419712183	0.649223958
LOC107051727	-0.447503426	-0.519290026	2.036967925	0.153515381	0.366153877
LOC428250	-0.27188519	-0.518005568	0.554588729	0.45644878	0.678978907
LOC427618	0.805287591	-0.517856236	4.653424907	0.030991654	0.13235142
LOC107049860	1.413133263	-0.517156727	14.34271701	0.000152368	0.002862918
LOC101752170	-0.316401936	-0.517045531	0.819366716	0.365365388	0.600872977
CWH43	-0.557876724	-0.516364793	3.685731764	0.054879885	0.190718017
LOC107053690	-0.31499487	-0.516331703	0.496071804	0.481231242	0.696758476
SPATA4	-0.62104464	-0.51586051	3.106837235	0.07796422	0.238280562
FGF3	-0.093534531	-0.515826968	0.079978973	0.777325907	0.889554058
LOC107053653	0.226177779	-0.513821567	0.588632282	0.442948495	0.667648391
LOC107052476	0.301844763	-0.512738714	0.861503993	0.353318152	0.590030781
LOC107049052	-0.655581659	-0.511977255	1.332874875	0.248294418	0.482485197
LOC112533394	1.701754778	-0.51149049	14.10427608	0.00017295	0.003190465

LOC107051704	-0.233711186	-0.511127171	0.583045193	0.445121172	0.669924475
DYDC1	0.306598896	-0.509880846	0.745451863	0.387920029	0.621932256
LOC112530177	-0.940973458	-0.509704385	8.750605326	0.003094993	0.027019761
LOC101749862	-0.033121731	-0.50927178	0.006730443	0.93461548	0.971393237
GBE	0.009766959	-0.508811826	0.000857181	0.976643154	0.990307746
HTR1F	0.671580149	-0.508220782	2.741762522	0.097756891	0.274222577
TSPAN32	0.257378925	-0.508109257	0.739490623	0.389824099	0.623763967
LOC101748556	-0.267367929	-0.507822342	0.70331663	0.401671498	0.634066049
EXPH5	-0.274923104	-0.507529748	0.701061667	0.402427189	0.634650123
LOC107054058	0.443075723	-0.507469788	1.463657646	0.226349047	0.456254766
LOC112530783	0.571538604	-0.50702945	2.625144261	0.105182945	0.288176051
LOC107051685	-0.037034636	-0.50567522	0.011369912	0.915082686	0.961017703
LOC101747873	0.538434359	-0.503733886	2.049018973	0.152304301	0.364427407
LOC112532290	0.944830192	-0.502092733	6.411913214	0.011335721	0.067868823
LOC107051988	-1.655910787	-0.501353842	18.1207911	2.07E-05	0.00057828
HTR1A	-0.43832564	-0.5013538	1.781786873	0.181930062	0.404111659
LOC107052570	-0.011258708	-0.500389804	0.000550693	0.981277874	0.991828264
MAL2	-0.291588673	-0.500295485	0.957560289	0.327802328	0.565526033
LOC112532480	0.324633993	-0.499249166	1.315931638	0.251323502	0.48584683
LOC107054990	-0.601132393	-0.499039299	2.114544684	0.145905442	0.355031785
LOC107055549	-0.363254842	-0.498324867	1.256108529	0.26238898	0.497588898
HMGCS2	-0.034822294	-0.497364973	0.010679189	0.917693003	0.96231374
LOC101749453	-0.114197389	-0.497317017	0.119268619	0.729829089	0.861397607
LOC107054664	-0.723494132	-0.496583944	5.080873852	0.024191175	0.112607009
FAM20CL	0.063161372	-0.496220185	0.046816424	0.828698543	0.918759906
LOC112532495	0.208388384	-0.495678654	0.340981297	0.559263345	0.754689056
LOC101747681	0.885067013	-0.495024033	4.97989128	0.025643595	0.116920296
WNT11B	1.196540169	-0.494018467	10.28950173	0.001337892	0.014804986
C14orf180	-1.452942727	-0.492542336	9.237685551	0.002370844	0.022537402
LOC107052750	-0.766541566	-0.491083806	3.993400545	0.045678787	0.169327426
FAM172BP	-0.221265954	-0.490537222	0.616529716	0.432339796	0.659328206
PLN	0.691789785	-0.488947895	5.457270057	0.019487069	0.09771631

LOC107049609	2.08616041	-0.488424526	29.97033048	4.39E-08	3.30E-06
LOC107052042	-0.15591592	-0.488152532	0.280955037	0.596075934	0.779387029
LOC112532303	-0.424759334	-0.487779174	1.19650568	0.2740212	0.508957412
LOC112530007	0.550735697	-0.487392474	3.668092714	0.055463619	0.192071733
ZC2HC1B	-0.578258307	-0.487230841	3.641866157	0.056343746	0.19392101
ZPBP	0.835232614	-0.486616572	4.76471934	0.029048795	0.126716076
TRPM6	0.177413154	-0.485939824	0.254541856	0.613895019	0.790936713
SOWAHB	-0.048305354	-0.485643352	0.027172324	0.869069684	0.939650546
IGSF5	-0.9926959	-0.48488705	10.49204785	0.001198894	0.013652459
TRNP1	1.084493552	-0.483609302	12.13907673	0.000493762	0.007202887
HMX2	-0.021341595	-0.482799584	0.004231911	0.948131689	0.978142017
LOC107054337	0.304168336	-0.481964555	0.594626477	0.440635645	0.6651417
FUT6	0.297571387	-0.481889463	0.711574245	0.398921696	0.631258824
LOC101748714	0.155063509	-0.481432982	0.285458794	0.593145448	0.777327412
LOC107052916	-0.193265423	-0.481328297	0.270087615	0.603273006	0.784359775
HGD	-0.365951992	-0.479779264	1.580442285	0.208696997	0.436268268
LOC416655	-0.207599637	-0.4753676	0.474407203	0.490966606	0.704284195
LOC415472	0.095967106	-0.474673639	0.089037002	0.765404996	0.882540762
SERPINB5	-0.089438808	-0.474073266	0.065078592	0.798641956	0.901588892
LOC112529994	-0.302352239	-0.473866608	0.962530624	0.326550111	0.564254007
TUBA8A	-0.608340363	-0.473634301	3.898781199	0.048321151	0.175408016
ADCY1	0.356115729	-0.473537093	1.444994652	0.229332804	0.460259945
LOC107055363	0.85955312	-0.473078096	8.008817629	0.004655012	0.036336919
CLCN2	-0.21765892	-0.472210778	0.407254536	0.523366294	0.727919042
LOC100858995	-0.032051568	-0.471848895	0.009484511	0.922417912	0.964657225
HIST1H2A4L3	-1.612986014	-0.471223006	28.84638392	7.84E-08	5.49E-06
CHRNA6	-0.313230552	-0.468621665	0.907241093	0.340847499	0.577972547
TRDNL	-0.115061295	-0.467908283	0.121662936	0.727237853	0.859614889
F7	-0.892140457	-0.467450656	8.98860004	0.00271669	0.02460272
POU2AF1	0.077237648	-0.467174252	0.034719466	0.852184726	0.931269647
LOC101750475	0.487031747	-0.467130427	2.202127296	0.137820425	0.342448818
LOC112530626	-0.231902535	-0.46564785	0.454805994	0.500061456	0.710749352

LOC107054351	-0.738507693	-0.465038951	5.940048364	0.014800613	0.081289746
LOC107056949	-0.442763657	-0.464785386	1.645837442	0.199526426	0.425451255
MLANA	0.768323556	-0.462590588	2.597337983	0.107043375	0.291188417
AK9	0.340456597	-0.461635926	1.328478299	0.24907611	0.483290069
NIPAL1	-0.373362063	-0.461329831	1.426788832	0.232289219	0.463704852
ANXA13	-1.136183484	-0.46085273	10.54855456	0.00116279	0.013379263
LOC112533310	0.113096767	-0.45988734	0.144773823	0.703580565	0.846550028
LOC112531824	1.002222084	-0.459780513	5.028819293	0.024928888	0.114806842
LOC107049103	-1.712442315	-0.458512529	23.45004174	1.28E-06	5.84E-05
LOC107054302	-0.635532197	-0.458425782	3.510636309	0.060976032	0.20464218
LOC101748561	-0.62738681	-0.458001659	4.636467197	0.031299371	0.133151797
LOC107053795	0.09817713	-0.457484935	0.091436508	0.762358815	0.880756463
LOC107054872	-0.096591475	-0.457102862	0.101383862	0.750175224	0.873555895
SIGLEC1	-0.464801019	-0.456704627	1.312141412	0.252007307	0.486796086
LOC107052149	-0.40459379	-0.455990139	1.610553309	0.204414041	0.431179727
LOC112532581	0.163375985	-0.455492782	0.245327621	0.62038443	0.795058816
LOC112532239	0.078215724	-0.455135691	0.053128353	0.817706461	0.913838618
PLA2G12B	-0.007948596	-0.454979652	0.000799622	0.977440754	0.990525896
SH3BP1	0.421516083	-0.454948137	1.723139739	0.189289393	0.412769276
HCN1	0.218729922	-0.454629917	0.474982897	0.49070369	0.703969418
LOC427665	-0.104893789	-0.454483123	0.118307013	0.730877974	0.862250839
LOC112530293	0.461527759	-0.454195477	1.082458357	0.298148255	0.534894621
SSPO	-0.665906839	-0.453099057	3.041499441	0.081160918	0.244534238
LOC101747613	-0.3426386	-0.451877975	1.158211946	0.281836676	0.517591797
LOC107055145	-0.199923038	-0.451290213	0.439288972	0.507465838	0.716891343
LOC107052001	-0.495122575	-0.450393887	1.762574178	0.184303759	0.406903552
LOC112530001	0.682150133	-0.449463976	4.781915655	0.02876011	0.126044495
LOC771085	-0.176598087	-0.448557177	0.316700104	0.573597543	0.764869254
LOC112532877	0.110853854	-0.448011649	0.121532232	0.727378566	0.859661471
LOC107052706	-0.729005598	-0.447901475	3.498239162	0.061434116	0.205927557
LOC112532963	0.268442749	-0.447674084	0.719728843	0.396232868	0.629052616
LOC112531865	-0.891755318	-0.447566702	7.899973798	0.004943551	0.037959

LOC112530238	-0.473749192	-0.446772398	2.502460405	0.113668591	0.302558503
LOC112533422	1.105967826	-0.445087341	5.648942691	0.0174659	0.091093941
LOC107053822	0.106588877	-0.444408137	0.110229247	0.739883293	0.867829466
LOC107051624	-0.155707878	-0.443671761	0.260404826	0.60984217	0.787717486
ZNF366	0.036362502	-0.4431985	0.013707908	0.906796045	0.957377047
NR1H4	0.773135601	-0.443045339	6.220382037	0.012628786	0.073296899
LOC112532100	0.646566422	-0.44278974	2.489129451	0.114635117	0.304098155
LOC112532811	-0.184350027	-0.442611994	0.277501429	0.598343565	0.781222197
GVINP1	0.904458943	-0.441251634	5.487319483	0.019154889	0.096494854
LOC112530098	-0.147179483	-0.440476458	0.163325896	0.686112723	0.836197732
TBC1D24L	-0.12517253	-0.440099605	0.160831186	0.688392414	0.837770666
TACR1	-0.919383241	-0.440009594	10.0425599	0.001529645	0.016386918
LOC107054691	0.050929441	-0.439743103	0.03374726	0.854245457	0.932178863
LOC112533541	-0.281934708	-0.439586749	0.549091897	0.458688982	0.681079878
COLCA2	-0.000116223	-0.438736899	5.05E-07	0.999433134	0.999676142
MYH1B	-0.944980559	-0.438539866	5.247788673	0.02197468	0.105482311
LOC101750462	-0.088639751	-0.438531939	0.063754702	0.800656984	0.90281715
RPL3L	-0.02844401	-0.437610524	0.011946596	0.912964162	0.960095348
LOC107054815	-0.098836172	-0.437202463	0.096341355	0.756265129	0.87704156
HIST2H4B	-0.60348395	-0.437120198	3.805892391	0.051072554	0.182071677
LOC107049551	-0.754238288	-0.436595927	3.787190629	0.051646264	0.183378723
LOC101747645	0.987728981	-0.435793326	9.011446052	0.002682941	0.024391043
FUT7	0.040103574	-0.434838997	0.015888894	0.899691431	0.954302681
NR4A1	-0.1166471	-0.434519592	0.122197692	0.726663031	0.859204948
TRNAC-GCA	-0.083514513	-0.434165896	0.070204308	0.791039541	0.897322187
LOC418544	-0.361778513	-0.433721692	0.955267354	0.328382152	0.565814483
LOC101749758	0.661425974	-0.433352353	3.928154692	0.047484021	0.173440526
OVAL	-0.30599215	-0.431612219	0.464952025	0.495318686	0.707178212
C9ORF152	-0.791899132	-0.431112175	5.931963571	0.014868665	0.081581822
LOC101749594	-0.685005729	-0.43044928	4.445069773	0.03500216	0.143024718
LOC112531026	0.030720881	-0.429432934	0.007650443	0.930300466	0.969480315
LOC769175	0.241098122	-0.429354211	0.656223814	0.417896253	0.647686055

TRBV19	-1.786144842	-0.42859861	19.56405863	9.73E-06	0.000317615
LOC112532328	-0.316266495	-0.42799379	0.812793813	0.367295546	0.602336876
LOC107053048	-0.624980302	-0.427988565	2.758223054	0.096755653	0.272657543
LOC100859665	-1.140207941	-0.425585743	11.14014884	0.000844791	0.010627705
LOC769384	0.635067622	-0.424512892	3.704726684	0.054258557	0.189579921
PLA2G4F	-0.776136602	-0.42391707	5.91223037	0.015036123	0.082201308
LOC107049206	-0.728965394	-0.422634966	7.005606686	0.008125483	0.053718291
HS3ST1L	0.250401422	-0.4225102	0.711536829	0.398934094	0.631258824
LOC112532456	-0.439729598	-0.421639709	2.025632448	0.154664522	0.367509705
LRRC9	-0.589051001	-0.420304095	3.350880947	0.067169058	0.217628083
LOC112532925	-0.827660507	-0.419728246	7.272937072	0.007000124	0.048276213
LOC107054488	-0.027460542	-0.419524169	0.004250075	0.948020655	0.978142017
LOC112530257	-0.579597904	-0.417000335	3.658259557	0.055791885	0.19262599
LOC112532820	-0.728432242	-0.416783543	5.075512088	0.024266103	0.112764397
LOC112531585	0.235655846	-0.415667134	0.529714059	0.4667271	0.686608642
FAM83F	-0.142567397	-0.414640562	0.17613498	0.674716036	0.828603058
LOC101750758	0.70876934	-0.414605551	4.067116977	0.043725844	0.164914059
LOC107053818	-0.049502183	-0.413976506	0.034041822	0.853617885	0.932007319
G6PC	-0.649494373	-0.41280262	3.00409056	0.083054575	0.248033218
AGMO	-0.613901097	-0.411966204	3.311434579	0.068799408	0.220941268
LOC107054519	-0.81673933	-0.411821335	4.476240255	0.034369249	0.141562306
MIR1634	0.226560645	-0.410573157	0.494563058	0.481898859	0.697173002
LOC112533467	0.581139042	-0.409611075	3.344929817	0.067412353	0.217974116
LOC107053497	0.618798087	-0.408949888	4.770769012	0.028946893	0.126479321
HCK	-0.815384592	-0.408810218	4.501933998	0.033856541	0.140118054
TMIGD2	-0.141286656	-0.408223464	0.222434228	0.637191386	0.805669606
MYO1G	0.426345467	-0.40797832	1.598123723	0.206169309	0.43321619
MIR3528	-0.368351783	-0.407966303	1.474945401	0.224567039	0.454129363
LOC112531516	0.458764237	-0.407872226	1.646730746	0.199404479	0.425301451
SPATA22	-0.114816	-0.407803941	0.168040059	0.681859575	0.833288668
LOC101749127	-1.550598534	-0.407433099	20.79141769	5.12E-06	0.000188945
LOC112530345	-0.403525312	-0.407319724	1.434008384	0.23111138	0.462544585

WNT3	-0.050526643	-0.407158676	0.026084081	0.871695147	0.941005291
LOC101747852	-0.502212598	-0.406736233	3.110734488	0.077777879	0.238215389
LOC112532096	-0.218835019	-0.40640056	0.536865305	0.463734757	0.684929547
LOC101751461	-0.38852092	-0.406134907	1.038629423	0.308140446	0.546031773
LOC112531635	-0.059655161	-0.406116721	0.026352849	0.871041576	0.940731028
PLA2G10L	0.150474519	-0.406026605	0.167542451	0.682305209	0.83346248
LOC101749173	-0.641480977	-0.405655504	3.873065466	0.049066826	0.177215675
LOC112533210	-0.333247534	-0.40511878	0.854291	0.355341262	0.59199604
LOC107053584	-1.558259047	-0.403916649	21.13805673	4.27E-06	0.000161659
LOC107049917	-0.723978273	-0.40350369	4.037459329	0.04450077	0.166612097
LOC107055322	-0.268584446	-0.402486867	0.60949732	0.434977141	0.661252025
LOC107051661	-0.396419961	-0.400390381	1.647661586	0.199277501	0.425111187
SLC38A4	-0.513697191	-0.399632161	3.442559644	0.063537186	0.210363058
NXNL1	0.036315048	-0.399398803	0.013451455	0.907668068	0.957538022
SAMD10	0.118021148	-0.398997633	0.136307936	0.711979366	0.850375297
LOC112532385	-0.410063347	-0.398328534	1.396674325	0.237281192	0.469624971
LOC101748799	-0.276171939	-0.398090896	0.941455854	0.331903714	0.569378192
LOC101747771	0.228567326	-0.396078165	0.671245799	0.412617602	0.642956689
LOC112531707	-0.067222675	-0.39600396	0.036231448	0.849038321	0.929677837
DRD4	0.240399344	-0.395882562	0.557583991	0.455235331	0.677909264
LOC112531790	1.754867581	-0.395839607	15.17031187	9.82E-05	0.002038424
GIMAP8L1	1.078532086	-0.394833829	8.957741693	0.002762962	0.024912079
LOC424300	-0.29304104	-0.392301717	0.906443121	0.341059928	0.57809447
LOC112532759	0.356508825	-0.392011243	1.269977048	0.259770986	0.494318997
LOC107054132	-0.028339132	-0.391771369	0.007501854	0.93097894	0.969780408
LOC107051713	0.381700809	-0.391663754	1.426871973	0.232275613	0.463704852
LOC112532806	0.143678786	-0.391445593	0.251437218	0.616064901	0.792054659
CYP1A2	-0.135703797	-0.39140042	0.180908844	0.670593355	0.825783734
DMP1	-0.774869019	-0.391346285	5.292103609	0.021422328	0.103862114
LOC112531997	-0.244526278	-0.390606164	0.573580764	0.448839514	0.672156372
LOC107054329	-0.383130864	-0.388541258	1.684646041	0.194308245	0.418892604
LOC112532457	0.258131324	-0.388396694	0.610053898	0.434767517	0.661222386

DMRT3	0.039213052	-0.387576532	0.010920515	0.916771561	0.961993117
LOC101747366	0.101575252	-0.387508903	0.066027337	0.797211311	0.901033555
ACVR1C	0.429526782	-0.387468852	2.179483122	0.139861426	0.345818148
CTLA4	-0.565618768	-0.387059426	3.407044199	0.064918632	0.213178225
SCN1A	-0.266726646	-0.386949185	0.578698859	0.446822779	0.670621395
CRP	1.219971351	-0.385625799	16.73408144	4.30E-05	0.001059267
LOC112532177	-0.032397535	-0.384441751	0.013552806	0.907322443	0.957480331
ATP10B	-0.582386146	-0.383723468	3.583565664	0.058353772	0.19889328
P2RY4	0.025623944	-0.383523958	0.006014626	0.93818282	0.973257994
LOC422643	-0.65133155	-0.383243979	3.691458375	0.054691776	0.190379083
LOC101749388	0.265243484	-0.38301883	0.532160716	0.465699864	0.686047844
CYP2AB2	-0.294954427	-0.382933292	0.743257016	0.388619533	0.622600956
ABRA	-0.296164655	-0.382750019	1.162025732	0.281045817	0.516716081
LOC112530231	-0.257803884	-0.381423959	0.661365511	0.41607828	0.646383861
MIR1454	-0.196163385	-0.380006077	0.335512286	0.562431141	0.756921375
DLGAP1L	-0.929726033	-0.379275878	9.929112342	0.00162685	0.017182164
LOC107054452	-0.631900013	-0.378867641	4.352402536	0.036956776	0.148216938
LOC101751264	1.03440388	-0.376976466	7.435561124	0.006394742	0.04559163
ZPLD1	-0.257999886	-0.376550857	0.338245408	0.560843761	0.755874706
SLC1A7	-0.618189245	-0.375982521	3.531283971	0.060221136	0.202853387
DMBT1	-0.201309644	-0.375865817	0.50927053	0.475454809	0.692572543
CDKN2C	-0.283086756	-0.374925951	0.79672001	0.372075845	0.606489157
OR52B2	0.780644023	-0.373941878	5.347593016	0.020750898	0.101623817
CD36	0.097747919	-0.372787535	0.079733762	0.777658533	0.889865642
LOC112530300	0.143265992	-0.371801333	0.173518209	0.677003806	0.830079538
LOC100857119	-0.717459268	-0.371023629	5.939442435	0.014805702	0.081290565
LOC112532722	0.142769924	-0.370772744	0.214961511	0.642906107	0.809288555
LOC112530456	-0.779593513	-0.370400327	4.157313715	0.041455101	0.159753578
LOC107052133	-0.15856181	-0.370021735	0.307935099	0.578950064	0.768307327
LOC101747293	1.150674235	-0.3690309	9.571522174	0.001976189	0.019986591
LOC107051936	0.037013848	-0.368995272	0.01717474	0.895733829	0.952335734
LOC107054046	0.707030611	-0.368715787	5.504506518	0.018967532	0.095739139

TSHR	-1.131455105	-0.368432607	11.44426014	0.000717152	0.009440587
CSF2RA	0.415032467	-0.3683963	1.555349917	0.212347383	0.440294378
LOC112531510	0.24683579	-0.36821265	0.421989568	0.515945954	0.722359455
HTR2C	0.419850351	-0.36736898	2.247644478	0.133817963	0.336179326
LOC112532315	-0.025888822	-0.367070159	0.008950885	0.92462531	0.966229608
GHRH	0.579514283	-0.366872943	2.330283152	0.126878796	0.324543851
LOC107057093	-1.201872859	-0.36639529	17.34890131	3.11E-05	0.000808656
LOC107055286	0.327897615	-0.366238184	1.308170629	0.25272614	0.487280718
LOC112530548	0.224917538	-0.366163036	0.530339523	0.466464152	0.686492051
LOC107052254	0.098432682	-0.365770783	0.106967676	0.743623161	0.869767312
LOC112533494	0.092720143	-0.364772478	0.108445849	0.741920499	0.868980128
BCO1	-0.847612867	-0.364476308	3.69388223	0.054612363	0.190222373
MUC4	1.213671846	-0.363908227	7.962807922	0.004774828	0.037061226
LOC107055287	-0.167858264	-0.363460595	0.317538927	0.573090427	0.764693721
LOC100858253	0.11303482	-0.362511064	0.144100904	0.704237722	0.846723552
LOC112530787	-0.447577932	-0.362069213	2.039817537	0.153228025	0.365839691
DMRT1	-0.089421118	-0.36081949	0.069931678	0.791436282	0.897586603
LOC101750607	0.373111645	-0.360510635	0.872968633	0.350134775	0.586707507
LOC107053783	-0.015147048	-0.360034066	0.003090799	0.955664486	0.981041173
LOC112532312	-0.460765939	-0.359787962	1.954466276	0.162106379	0.378333331
LOC107054831	-0.021937638	-0.359301977	0.003314594	0.954089144	0.980523198
LOC112532240	-0.242503937	-0.357809417	0.780550451	0.376972845	0.611748295
KIF6	0.124136011	-0.356519955	0.130272414	0.718149758	0.854281987
LOC107055018	-0.070402847	-0.356012647	0.07220836	0.788148137	0.895647623
LOC107054412	0.257301165	-0.355523321	0.63472085	0.425628944	0.653942509
ANO4	-0.244378922	-0.355442105	0.346986488	0.555823983	0.752912164
LOC101748323	-0.30106459	-0.353687994	0.990221748	0.319688183	0.557135546
LOC112530154	-0.44847122	-0.353383103	2.1073878	0.146589359	0.355876056
UNC93A	0.402384041	-0.353106356	1.409123569	0.23520194	0.467364802
GUCY2F	0.005491051	-0.352922031	0.000294543	0.986307176	0.993448882
TNN	-0.559458966	-0.352799327	3.590257572	0.058119234	0.1982882
TAGAP	-0.838391307	-0.35265021	6.14379643	0.013187389	0.075477733

LOC101749307	1.278340328	-0.352282847	8.519750792	0.003513126	0.029615004
LOC112533160	0.079594044	-0.35165754	0.074470464	0.784935645	0.89437421
LOC101750745	0.386827115	-0.351537996	1.656615341	0.198060934	0.423698995
LOC101751159	-0.33671198	-0.351269744	0.780542588	0.376975249	0.611748295
HEPHL1	-0.337996564	-0.350501619	1.662614925	0.197250631	0.422790041
LOC107052867	-0.370120247	-0.350114	1.210088501	0.271314595	0.506377385
LOC112532195	-0.025131573	-0.349877197	0.005246294	0.942258674	0.975259608
GRIN1	-0.386725157	-0.349614965	0.914301195	0.338975755	0.575926283
LOC112530004	0.190550775	-0.348300313	0.336580873	0.561809487	0.756326197
PKD1L2	-0.26098024	-0.347952805	0.660309483	0.416450705	0.646602789
LOC107054918	1.946514876	-0.347574685	29.00461195	7.22E-08	5.10E-06
KRT20	-0.498027156	-0.34708718	3.505798948	0.061154342	0.205073304
LOC112532151	-0.027729049	-0.345525091	0.007819893	0.92953479	0.968805103
LOC107055185	-0.585610695	-0.345016829	3.71083022	0.054060495	0.189188738
LOC107053809	-0.252461446	-0.344571059	0.712992095	0.398452295	0.630739058
KCNE2	0.343968067	-0.343513558	0.915912686	0.338550461	0.575738718
LOC101748420	-0.809239869	-0.341886048	6.969344834	0.008291785	0.054576529
LOC112533279	-0.161512639	-0.341802629	0.28293771	0.594782171	0.77845837
LOC107052320	0.845800517	-0.341505545	5.294412459	0.021393947	0.103862114
LOC112531457	-2.029516848	-0.338741146	27.61430394	1.48E-07	9.63E-06
LOC101752322	-0.142591378	-0.338135831	0.256043469	0.612851468	0.790261455
LOC101748767	-0.18598224	-0.337737369	0.382805817	0.536105423	0.737721299
LOC424167	0.728518714	-0.337573104	5.5899157	0.018064166	0.092918363
LOC101751780	0.473903986	-0.337162787	2.367832522	0.123858916	0.319101059
GSG1	-0.307801107	-0.336216624	1.343865241	0.246353474	0.479919939
LOC100859709	-0.11373273	-0.33602306	0.16517684	0.684434367	0.835303382
DHX58	-0.054372914	-0.335997055	0.033596231	0.854568325	0.932364526
AMN	-0.070039323	-0.335462708	0.061315194	0.80442917	0.905491008
SBSPON	-0.320123639	-0.335410388	0.659722249	0.416658017	0.646851271
PRODH2	0.266688279	-0.333919639	0.560105947	0.454217558	0.677067662
LOC112530788	-0.4561423	-0.331953886	2.673900921	0.102005726	0.281959386
PLD6	-0.015116777	-0.331572699	0.003261092	0.954460776	0.980523198

LOC769128	-1.178941128	-0.331059235	16.22121525	5.64E-05	0.001321977
LOC107054371	-0.850379313	-0.33097414	9.542857744	0.00200729	0.020193053
INS-IGF2	-0.415683949	-0.328645555	1.574126658	0.209608734	0.437426118
LOC107049115	-0.400404395	-0.327577605	1.231474745	0.267120427	0.502568789
LOC101750580	0.304372915	-0.32738097	0.73944643	0.389838264	0.623763967
LOC107055338	-0.952010681	-0.327330737	9.057553516	0.002616118	0.023995663
CTNNA3	0.031560216	-0.326887211	0.012282393	0.911754361	0.959572626
MUC13	0.302296003	-0.326757717	0.595345994	0.44035927	0.6651417
LOC112531723	-0.124065604	-0.325530811	0.208412257	0.648014717	0.812187803
LOC112533355	-0.666644446	-0.324427263	4.764352657	0.029054984	0.126716076
LOC107054793	-0.29208064	-0.323235004	0.904866904	0.341480059	0.57844908
POU4F3	-0.169671031	-0.322945811	0.191514418	0.661659159	0.820467329
ANKRD9L	-0.384610428	-0.322612858	1.280328088	0.257838042	0.492541889
LOC112530484	0.9618093	-0.322304438	9.51867332	0.002033916	0.020370105
LOC112530431	-0.341690167	-0.321265398	1.349413261	0.245380718	0.478972682
LOC107054129	1.272069197	-0.320161213	14.9764533	0.000108861	0.002181863
LOC107053502	0.221984741	-0.319981441	0.444792523	0.504818333	0.71526617
LOC112531701	0.340836979	-0.319253234	1.334989426	0.24791953	0.482093632
KLHL34	0.175644757	-0.318895087	0.365061234	0.545708704	0.745755064
LOC107053011	0.196622751	-0.317429767	0.505626483	0.477038272	0.693600685
LOC101752135	1.003670097	-0.312733634	6.046302871	0.013935446	0.078337692
LOC101747497	0.313007768	-0.312134788	1.47325303	0.224833139	0.454444087
LOC100858311	0.156543423	-0.311818064	0.216360948	0.641826793	0.808610358
LOC107055188	-0.852981881	-0.309588311	5.112727335	0.023750955	0.111345291
LOC112533216	0.882356645	-0.309367678	5.794813485	0.01607352	0.086434566
LOC112530951	0.197430648	-0.308892918	0.512801722	0.473928521	0.691907888
CHRNB4	0.394991854	-0.308506955	1.220846776	0.269194536	0.504027293
LOC101751262	-0.079227942	-0.308379561	0.079609477	0.777827335	0.889865642
CX3CR1	-1.015771719	-0.307889133	9.087729957	0.002573301	0.023695391
LOC112533336	0.160756735	-0.30714732	0.353430635	0.552177526	0.750791703
KEL	0.158522298	-0.305996364	0.241032629	0.623461289	0.797563205
LOC107052223	0.665653099	-0.305895666	4.308743909	0.037916949	0.15049741

LOC112532693	0.002592613	-0.304698091	9.00E-05	0.992429722	0.996459368
LOC112531962	-0.071958641	-0.304033002	0.052004853	0.819610692	0.915220816
LOC101747367	-0.073642619	-0.301337821	0.048479239	0.82573096	0.917656617
LOC107049676	-1.312980699	-0.300807025	7.355821325	0.006684597	0.046846268
LOC107053614	0.155658627	-0.298002686	0.218476791	0.640202986	0.807491962
LOC101748308	-0.63021531	-0.29775261	4.179690414	0.040911173	0.158734581
ZP1	-0.229100907	-0.297274966	0.566979276	0.451461774	0.674854969
LOC107051710	-0.630379279	-0.297131553	3.469758129	0.062500449	0.208060871
LOC107049968	0.116874354	-0.297070528	0.103416145	0.747768013	0.872702382
HDC	0.261610458	-0.296907813	0.274242301	0.600500093	0.782591357
BLB2	-0.06525591	-0.296355878	0.039129488	0.843192353	0.926094935
ABCB1LA	0.401630356	-0.29514512	2.440237646	0.118258308	0.31045636
LOC420748	0.022330633	-0.295075507	0.00496952	0.943799821	0.975921243
IL18R1	0.613194711	-0.294612582	2.747090783	0.09743156	0.273636511
KCNK4	0.908473519	-0.294287396	7.637460555	0.005716835	0.041883576
LOC107049725	1.867964277	-0.293698444	16.59712422	4.62E-05	0.001120129
C30H19orf53	-0.246829588	-0.293562556	0.515122581	0.472929699	0.691098524
PAR2L	0.558482168	-0.29224883	2.370458915	0.123650697	0.318775621
HIST1H2B8	0.012710589	-0.291914039	0.001499582	0.969110093	0.987351035
LOC112532951	0.254115369	-0.291827992	0.577775314	0.44718565	0.670752767
GRM8	0.101835403	-0.291519675	0.142697542	0.705613882	0.847571095
LOC107053593	0.093351684	-0.290850016	0.061137638	0.804706808	0.905584088
FRK	-0.071873183	-0.290723075	0.045801598	0.830536858	0.919558875
LOC107052519	0.086088834	-0.289115641	0.054429478	0.815527481	0.912577102
MIR1728	-0.429957451	-0.288577318	2.002941314	0.156994303	0.371332607
FAM178B	-0.380718885	-0.286767627	1.564569595	0.210997396	0.438822314
DYL2	0.325118893	-0.286582715	1.111719608	0.291708449	0.528815967
SGCZ	0.481338172	-0.286059284	2.500212488	0.113830939	0.302755928
SLC26A1	-0.330436457	-0.28598872	1.056675804	0.303974583	0.541885862
LOC107054232	0.440712338	-0.285958332	1.78541509	0.181485788	0.40361517
ARSE	0.12065234	-0.285369803	0.129386513	0.719068973	0.854933522
TREM-B2	0.527049122	-0.285088426	3.115513632	0.077550025	0.237853805

ESX1	0.306274964	-0.284305985	0.596325685	0.439983388	0.664967091
LOC112533214	-0.173935015	-0.283647853	0.227086747	0.633692426	0.80403489
LOC107052331	0.192230522	-0.283264925	0.314748769	0.574780666	0.765335479
LOC107049175	-3.571429113	-0.283150349	51.80862423	6.12E-13	1.55E-10
LOC427826	-0.039439209	-0.282698786	0.014534018	0.904041839	0.956164575
ACSL5	-0.642818962	-0.282049285	2.973897746	0.08461775	0.251242345
FCER2	0.094654803	-0.281702186	0.049673487	0.823632408	0.91690482
B3GNTL	-0.088997897	-0.281428664	0.083446115	0.772680546	0.887261576
LOC112530019	-0.619711321	-0.280720165	3.470046566	0.062489552	0.208060871
RAX	-0.102468714	-0.27996096	0.115621919	0.733832255	0.863996119
FBXO16	0.349632115	-0.279793143	1.67377525	0.195753633	0.420677293
LOC107051928	-0.371430667	-0.279408012	1.442192898	0.229784811	0.460942225
LOC107053148	-0.811220623	-0.278752442	9.23327238	0.002376565	0.022564277
LOC112532748	0.805552214	-0.278385346	4.637389	0.031282562	0.133130044
STX19	-0.626801605	-0.277763256	5.481249168	0.019221518	0.096665673
LOC107052656	-0.630853195	-0.276937706	3.592866508	0.058028068	0.198151184
OPN5	-0.285016262	-0.276677088	0.754484359	0.38506016	0.619718521
GPR22	-0.136662367	-0.275135103	0.22770086	0.633233871	0.803815734
LOC107054323	-0.199034395	-0.275017841	0.288605809	0.591115349	0.776591063
LOC101751181	-0.674777397	-0.274252914	5.689421234	0.017067483	0.089870537
LOC101748427	0.000689918	-0.274055361	7.72E-07	0.999299114	0.999602853
LOC112533635	-0.178344545	-0.272079696	0.326946148	0.567462864	0.760729568
LOC107050476	0.948056346	-0.271918859	8.04157349	0.004571581	0.035821602
LOC112532376	-0.665788178	-0.270934795	5.309993957	0.02120343	0.10328669
LOC112533446	-0.017312736	-0.270721415	0.002598378	0.959346011	0.982513139
LOC107051179	0.446660228	-0.270666845	1.393209915	0.237863769	0.470551613
LOC107050360	2.389461822	-0.27043326	32.71510822	1.07E-08	9.29E-07
MST1R	-0.138594619	-0.269643104	0.137460311	0.710818975	0.849781439
LOC112530078	0.110702572	-0.269029624	0.173659947	0.676879372	0.830079538
LOC112531521	-0.83365225	-0.268743369	11.51339922	0.000690963	0.009191429
SLC34A1	-0.251138316	-0.267686805	0.350353294	0.553913207	0.751847053
TAS1R3	0.324527745	-0.267246334	0.882614631	0.347486552	0.584085637

LOC422150	-0.794629958	-0.264823755	9.051194803	0.002625232	0.024052446
LOC107054499	0.050595988	-0.26268401	0.02272729	0.880168409	0.94624142
LOC112532980	-0.674868783	-0.262158087	4.820215618	0.028127826	0.124420265
METTL7A	-0.15039515	-0.261248993	0.266916889	0.605407488	0.785831049
MIR1661	0.082231617	-0.260882369	0.070570259	0.790508286	0.897090611
GRPR	0.013468039	-0.260738293	0.001066378	0.973949351	0.989715351
LNP1	0.193435296	-0.260378315	0.495223369	0.481606485	0.697173002
LOC107055605	-0.220075652	-0.259780299	0.199093032	0.655453922	0.816217216
ATP6V1G3	-0.418774945	-0.259121815	1.176199625	0.278131036	0.51342228
LOC101751234	2.058561001	-0.257509692	21.52970841	3.48E-06	0.000135847
C9H2orf54	0.016371132	-0.256092265	0.002243033	0.962225778	0.983899168
LOC112533286	-0.354983893	-0.254623071	1.475788598	0.224434599	0.453917322
SLC19A3	0.109325915	-0.254452012	0.140863377	0.707424203	0.848568688
TRIM39.2	-0.847299279	-0.250642567	8.935152577	0.002797341	0.025139403
LOC107051939	0.27541881	-0.250438667	0.887819043	0.346069012	0.582540186
LOC107055113	0.228228685	-0.249921537	0.47511868	0.490641713	0.703969418
PRR15	0.298214318	-0.249281826	1.111749634	0.291701933	0.528815967
LOC107052944	-2.03465637	-0.249173482	52.79478671	3.70E-13	1.03E-10
LOC112530954	0.278994752	-0.247835555	0.758216181	0.383887341	0.618572875
LOC107054854	-0.496115867	-0.246668018	2.350036518	0.125280073	0.321654485
LOC107054457	0.104212327	-0.24642057	0.089966757	0.764219422	0.881657566
LOC112533548	0.460171335	-0.245930903	2.577503629	0.108392485	0.293644771
ESRRB	-0.104556224	-0.245291539	0.125502731	0.72314131	0.857066913
LOC418423	0.042446323	-0.244934907	0.012713134	0.910226752	0.958900701
CELA2A	0.465282381	-0.243313316	1.435337217	0.230895373	0.462346552
LOC107053863	-0.267577497	-0.243133971	0.582317268	0.445405457	0.670062789
HPX	-0.603393531	-0.242549121	3.545955093	0.059690793	0.201438065
LOC101751953	0.123259661	-0.242246312	0.155889167	0.692969813	0.839553694
LOC112531895	-0.606311054	-0.242139099	2.692577575	0.100816608	0.279659017
LOC112530790	-0.157852786	-0.240899928	0.291413031	0.589316446	0.775094087
LOC107052855	-0.158498114	-0.240740387	0.333976995	0.56332662	0.757599665
SH3RF2	-1.111415146	-0.239006945	17.01645318	3.71E-05	0.000935443

LOC107052484	-0.464236451	-0.238174148	3.35038311	0.067189374	0.21763368
NKX2-1	1.156265752	-0.237954028	11.92039373	0.000555227	0.007855776
LOC112533018	-0.11535538	-0.236986848	0.143626636	0.704701937	0.847032167
TNFSF11	-0.643223391	-0.236807819	5.098321229	0.023949011	0.111923027
CCDC180	-0.105667526	-0.234967935	0.150863413	0.697711612	0.842758423
PRLL	0.109512789	-0.233838468	0.137484154	0.710795025	0.849781439
CIDEC	-0.309138646	-0.233488692	0.604621714	0.436820046	0.662986744
CCNO	0.77125352	-0.233393934	8.670875657	0.003233358	0.027870563
LOC112531508	0.279565868	-0.233283344	1.1152687	0.290939511	0.528003711
WNT8B	-0.162695017	-0.232861886	0.235068286	0.627790972	0.800116215
MEIOB	0.07146611	-0.232507004	0.062547649	0.802513667	0.904105326
BPIFB6	0.34058882	-0.232246102	0.801497035	0.370646157	0.605077089
STAP2	0.085661655	-0.230978056	0.093125792	0.760240344	0.879915233
LOC107052134	-0.250558764	-0.230798358	0.597678064	0.439465324	0.66465091
C4H4orf17	0.006987937	-0.230560428	0.000735029	0.978370864	0.990904465
GAS2L2	-0.821474424	-0.230111141	3.537405248	0.059999253	0.202271606
NCF4	-0.207551565	-0.229634434	0.541538669	0.461795751	0.68328829
LOC107053757	-0.017020351	-0.228004501	0.002251814	0.962151964	0.983899168
LOC112530796	-0.181477876	-0.227761852	0.312913297	0.575897948	0.765833434
LOC112533403	-0.13378313	-0.227292472	0.231550456	0.630376564	0.801583732
LOC107055060	0.161453973	-0.227083093	0.180720623	0.670754678	0.825833762
LOC112530993	0.383340636	-0.225660598	1.772362416	0.183089982	0.405538517
NPBWR2	-0.939570367	-0.224820972	9.20744075	0.002410334	0.022702947
LOC107050218	0.133404074	-0.224704575	0.222356406	0.637250291	0.805682178
DEPTOR	-0.051156895	-0.224600933	0.032909611	0.856045722	0.932863069
AHRR	-0.618921505	-0.223744193	3.23834382	0.07193332	0.226571418
FER1L6	-0.42210724	-0.222971507	1.67255905	0.195916121	0.420708925
C1QTNF9	1.1286448	-0.222811862	2.840216274	0.091931839	0.26504255
LOC107055045	0.233882517	-0.221888231	0.457164246	0.498952257	0.710180814
PGR2/3	-0.450466081	-0.221813767	2.434194461	0.118714865	0.311009888
AVPR1A	-0.225650202	-0.221546902	0.191836232	0.661392712	0.820458761
TAPBPL	-0.000151059	-0.220381748	2.17E-06	0.998824528	0.999310367

LOC107053226	-0.15185481	-0.218666663	0.30003187	0.583862442	0.771063923
C3AR1	-0.641445499	-0.218604445	3.983143433	0.045957721	0.169902113
LOC112533484	-0.256086747	-0.21806631	0.662838869	0.415559501	0.645828447
LYG2	1.595660189	-0.217609284	15.95552246	6.48E-05	0.001479999
CFP	-0.282687931	-0.217467414	1.040310204	0.307749338	0.545867775
LOC107053423	0.206397047	-0.215657479	0.599640691	0.438715152	0.664126754
LOC112531062	2.300177128	-0.21369327	24.70611303	6.68E-07	3.47E-05
AHR2	-0.017971134	-0.212486842	0.003356356	0.953801147	0.980523198
NRG2	-0.451333148	-0.21181529	1.284707188	0.257025644	0.491957308
IFNAL1	0.054304522	-0.211222077	0.015201961	0.901872522	0.955177611
LOC101752273	0.557029802	-0.210696108	2.32568904	0.127253861	0.325162171
FEV	-0.224086821	-0.210466601	0.708080675	0.400081718	0.632466584
PCDHAC1	0.244674885	-0.208643365	0.54229319	0.461483905	0.683011122
LOC112529935	-0.939841862	-0.207965234	7.055067476	0.007904133	0.052763697
TXLNB	-0.769198531	-0.207543684	5.54918651	0.018489278	0.09407578
JAK3	0.311046908	-0.207522476	1.405814621	0.235752434	0.467900217
LOC107052152	-0.124570587	-0.205659866	0.169542347	0.680518859	0.832560433
AMH	0.134195792	-0.204492606	0.213653192	0.643919014	0.809954354
SH2D4B	-0.635791263	-0.202997786	3.741876118	0.053064832	0.186816817
HSFL1	-0.118905886	-0.202359341	0.146006372	0.702381405	0.845761261
MMR1L1	-0.756371869	-0.200274693	2.866629424	0.090434084	0.262232041
EYS	0.095250445	-0.200216745	0.080744162	0.776291468	0.889259549
STX11	-0.296972771	-0.200030538	1.082138039	0.298219754	0.534961959
OFCC1	-0.387620309	-0.197855623	1.01261551	0.314277058	0.551930556
LOC112530172	0.167568245	-0.197580421	0.407914918	0.523029712	0.727756969
LOC100859314	0.272833328	-0.196679428	0.898775089	0.343110367	0.580195365
GRAP2	-0.075585859	-0.196322042	0.07092292	0.789997719	0.896648248
LOC107052150	-0.189838516	-0.196313014	0.450296947	0.502193972	0.71305564
TECTA	-0.103929415	-0.194635714	0.100145779	0.751654764	0.87434463
RIPPLY2	-0.181532403	-0.194090588	0.472384929	0.491892031	0.704568538
MIR6621	-0.282405151	-0.19342357	0.937363928	0.332956695	0.570708585
LOC431250	0.60834486	-0.19301849	4.81368771	0.028234562	0.124620508

CYP2J24P	0.348359267	-0.192735218	1.891126265	0.169074945	0.387591003
LOC107055181	-0.762916843	-0.191802382	9.110729847	0.002541145	0.02354422
LOC101748134	-0.610773341	-0.191405509	4.686335346	0.030403424	0.130535311
LOC101748608	0.444657349	-0.19107617	1.631232386	0.201532677	0.428175623
AvBD14	0.92954457	-0.190830596	11.80476075	0.000590795	0.008183101
LOC112530038	-0.841540015	-0.190448274	4.045781182	0.04428188	0.166154137
LOC107052598	-0.798714309	-0.190345353	6.903315251	0.008603606	0.055913246
C19orf35	-0.385718201	-0.189162269	1.776296191	0.182604791	0.40500901
DFNB59	-0.22780142	-0.188828129	0.544101157	0.460738026	0.682585354
LHX8	0.295091935	-0.187739072	1.159076366	0.281657176	0.517377633
CRYBB1	-0.014621539	-0.187723698	0.00179181	0.966235806	0.98594879
LOC107051759	0.246613781	-0.187539429	0.482258853	0.487400927	0.701801037
CD79B	0.890390881	-0.186864164	8.872503005	0.002894979	0.025801375
LOC107051325	-1.169890626	-0.186700803	12.90224676	0.000328188	0.005258357
ACKR4	-1.486378386	-0.185752949	27.99393777	1.22E-07	8.07E-06
LOC107052419	-1.788799232	-0.184710409	24.42384563	7.73E-07	3.84E-05
LOC107054911	0.387731341	-0.184471229	1.892344603	0.168937711	0.38738434
ACOT12	0.955139751	-0.184149587	9.932470033	0.001623885	0.017161865
LOC107053874	-0.093900364	-0.183898345	0.089786657	0.764448552	0.881746876
LOC112531066	1.86739023	-0.183526388	31.20448123	2.32E-08	1.87E-06
LOC112531517	0.826362884	-0.182641885	3.964212252	0.046477264	0.171130762
KLHL30	0.036215269	-0.182627383	0.005172716	0.942664306	0.975445625
RAD21L1	-0.781603296	-0.180284882	4.404726246	0.035839476	0.145470791
KRTC42L	-0.741601975	-0.179897235	3.413649227	0.06465931	0.212623691
VIPR2	0.306008656	-0.179678817	0.658993999	0.416915324	0.647018925
LOC112532784	-0.14577255	-0.179403941	0.155520305	0.693314808	0.839730586
TRIM27.2	0.458295663	-0.178536141	0.997023499	0.318031807	0.555837854
LOC107054057	-0.896740364	-0.177754934	6.625034482	0.01005552	0.062205741
LOC112533088	-0.124379179	-0.176402597	0.220080656	0.638978469	0.806858657
ABCB5	0.22658717	-0.176315045	0.539110372	0.462801651	0.683977112
LOC112533269	-0.343461244	-0.173727445	0.973501928	0.323808358	0.561579526
LOC107050048	2.369572791	-0.173716305	24.6519064	6.87E-07	3.53E-05

IQCA1	0.447179921	-0.173261982	2.659774035	0.102915354	0.28390145
LOC112532710	0.069395704	-0.173136857	0.047791234	0.826952255	0.917915823
SLC34A2	0.24897905	-0.172528977	0.644455555	0.422101966	0.651321067
CYP11A1	0.62062417	-0.171667151	4.303468173	0.038034734	0.150664793
LOC101751949	0.279631591	-0.169255621	0.817983387	0.365770437	0.60103734
HIST1H4D	-0.294741181	-0.168515418	1.138216538	0.286029498	0.522416162
LOC107052089	-0.471274497	-0.167977061	2.634195014	0.104585058	0.286901837
SLC26A3	0.263917722	-0.167342713	0.656853146	0.417673104	0.647645206
ESR2	-0.733679714	-0.167260387	5.493035643	0.019092365	0.096251492
LOC112533223	-0.229797667	-0.166844364	0.555772039	0.455968786	0.678510253
OR6B1L2	1.458912003	-0.166179723	3.04603425	0.080934548	0.244138953
LOC112530989	-1.02696182	-0.165579935	7.494160872	0.006189937	0.044492889
LOC101747457	-0.053703883	-0.165005553	0.023544594	0.878049349	0.945012888
CILP2	-0.3753174	-0.164813586	1.624860216	0.202415429	0.429146995
LOC415913	0.054823595	-0.164541686	0.048570639	0.825569399	0.917634974
LOC107054135	-0.558741297	-0.164112584	3.008362842	0.082835917	0.247739915
NKX1-2	0.66174144	-0.16342529	4.306587734	0.037965041	0.15049741
LOC107055360	-0.043997528	-0.162464558	0.029922287	0.86266668	0.936001285
LOC770078	0.469435572	-0.161803789	3.093515353	0.078604823	0.239615111
LOC107052892	0.492566785	-0.161524378	2.774281583	0.095789628	0.271160185
LOC107057257	1.112815638	-0.160655256	2.284483777	0.130673613	0.330907095
PEX5L	-0.007702655	-0.160607164	0.000734483	0.978378903	0.990904465
LOC107054327	0.749134604	-0.159746308	7.497599848	0.006178128	0.044432294
LOC112531802	0.016869563	-0.159233003	0.003749871	0.951171105	0.979566938
LOC112530000	0.414570412	-0.159008712	2.844822651	0.091668708	0.264447509
LOC107054533	1.287800529	-0.158580395	10.7819213	0.001024962	0.012248189
INHBE	0.177405543	-0.157148155	0.345691882	0.556562042	0.75311398
LOC112532957	0.509029339	-0.156065366	4.074774514	0.043528075	0.164546302
LOC112530383	0.257305148	-0.155884229	0.344810424	0.557065628	0.753115441
LOC768995	0.052493492	-0.154983893	0.040173635	0.841141469	0.925252652
LOC107054020	-0.988585348	-0.154439471	7.334696059	0.006763612	0.047177115
LOC112530133	-0.521911927	-0.153137151	3.70186923	0.054351547	0.189709631

LOC107054261	0.081657465	-0.152674406	0.078541448	0.779283813	0.890741535
INHBA	-0.206369565	-0.15211089	0.575332807	0.448147548	0.671427866
WNT9A	-0.186673195	-0.15141955	0.388904438	0.532875839	0.735103154
CAPN3	-0.121316786	-0.151185353	0.210952013	0.646022247	0.811101486
KLHL1	-0.225725389	-0.150416758	0.511257747	0.474594893	0.692264899
GLDN	-0.105244248	-0.15024871	0.142877252	0.705437225	0.847437973
LOC107052786	-0.165301996	-0.149968759	0.28549603	0.593121344	0.777327412
LOC112533368	-0.290157556	-0.148914103	1.032441868	0.309585832	0.547425629
LOC107051965	0.287002011	-0.148847191	1.379107662	0.240253203	0.472834166
LOC101750112	0.354368065	-0.148642547	0.927070923	0.335625202	0.572942255
LRRC2	0.048132752	-0.14838694	0.028866103	0.865088565	0.937625632
LOC107054220	-0.602479158	-0.148227145	5.291078461	0.021434942	0.103862114
KCNMB2	-0.548600889	-0.147297514	4.363922897	0.036707682	0.14753906
SLC2A6	-0.561707435	-0.146292707	3.165752889	0.075197756	0.233335674
NRGN	-0.350858936	-0.146034412	1.282598663	0.257416416	0.492320522
ARL2	-0.770286904	-0.145861784	11.03031412	0.00089634	0.011056423
LOC112531926	-0.007025402	-0.144580925	0.00054892	0.981308041	0.991828264
LOC107053617	-0.772830258	-0.144515754	7.65837916	0.005650925	0.041604461
LOC107050560	-1.406406169	-0.14427413	8.736441561	0.003119126	0.027141841
LOC112531037	-0.184138905	-0.143650746	0.484978743	0.486175763	0.700617701
LOC112533404	-0.076802613	-0.142777894	0.087180204	0.767793025	0.884179034
LOC112533090	-0.887118045	-0.141833269	7.674488911	0.005600695	0.041327105
IFNAL2	0.512956994	-0.141349625	2.337129938	0.126322103	0.323572571
EFHC2	-0.248927464	-0.14111712	0.714765246	0.39786639	0.630220859
LOC395381	-0.758440641	-0.140827633	6.548262494	0.010498663	0.064126023
LOC112532617	1.148557005	-0.14055887	15.50895087	8.21E-05	0.001783724
LOC100859246	0.430001107	-0.139856209	2.035212472	0.153692706	0.366311338
LOC107055075	-1.787007514	-0.139219372	30.50637814	3.33E-08	2.56E-06
LOC107054749	0.314103886	-0.138852092	1.099101393	0.294463395	0.531351593
MHCBL1	-0.564627176	-0.138279453	2.319825981	0.127734315	0.325921563
LRFN2	-0.071890338	-0.137988462	0.04531143	0.831432405	0.919993291
TMPRSS7	0.450847281	-0.137507128	2.152470014	0.142340734	0.349636778

FEZF2	0.060596918	-0.136613358	0.036541166	0.848402249	0.929334244
LOC427533	0.194433609	-0.135476489	0.514684046	0.473118168	0.691215436
LOC112532846	0.357335957	-0.135243075	0.712854821	0.398497707	0.630750266
POMC	-1.949446582	-0.135033839	18.64782401	1.57E-05	0.000466997
LOC101750039	-0.576873971	-0.135005794	2.767697303	0.096184438	0.271821836
WEE2	-0.435325921	-0.134461203	1.197866258	0.273748562	0.508584463
LOC112533514	-0.10240635	-0.133508772	0.144497733	0.703849976	0.846564677
ANKRD33B	-0.267986716	-0.133311179	1.153588453	0.282799219	0.518491492
LOC107054062	0.471776681	-0.13161201	1.507859059	0.219466066	0.448737316
VTG1	0.543406393	-0.130096737	2.431134186	0.118946809	0.31136967
LOC101748860	-0.459812647	-0.128927807	1.993183964	0.158008366	0.372710387
LOC112533225	-0.205567813	-0.128925003	0.578761771	0.446798077	0.670621395
LOC107052144	0.538047781	-0.128904285	2.765740835	0.096302094	0.271980108
LOC112532897	0.04227188	-0.128661681	0.027580276	0.86809942	0.939156868
CKMT2	0.975828719	-0.128096222	12.23836407	0.000468169	0.006890621
LOC112533302	-1.082725897	-0.128017447	12.1027879	0.000503465	0.007299132
LOXHD1	0.866163011	-0.127730202	7.473848231	0.00626016	0.044865389
LOC112530138	-0.363794166	-0.127085016	1.855277795	0.173170801	0.39249663
TEKT4	-0.051403751	-0.126785984	0.028122343	0.866821518	0.93840635
CFAP65	-0.601917445	-0.126237743	4.682140929	0.030477742	0.130719816
FGF2	-0.468804583	-0.125938383	2.4915005	0.11446255	0.303689336
PAX5	-0.286540326	-0.125825461	0.640209676	0.423634881	0.652951664
SMIM28	-0.543430209	-0.125115825	1.886329723	0.169616474	0.388075512
CCDC60	0.064211493	-0.12422339	0.063482164	0.801074555	0.903085487
ASB14	-0.254740812	-0.123540783	0.929821091	0.334909419	0.572207921
SLC38A3	-0.447865517	-0.121499327	2.834828042	0.092240675	0.265492444
SLC25A47	-0.550507735	-0.120894136	3.602333826	0.057698517	0.197386508
TMEM215	0.300106577	-0.120344968	1.21441239	0.270460017	0.505327532
CCDC110	-0.195283687	-0.120185269	0.420426342	0.516724385	0.722783697
ZP3L1	-1.259272664	-0.119753755	20.19401041	7.00E-06	0.000242908
LOC101749755	0.0683493	-0.117880828	0.059066426	0.807977623	0.907527084
LOC112531860	1.307138764	-0.116915329	14.81841458	0.000118374	0.002341158

LOC107052224	-0.13830986	-0.116331237	0.280775705	0.596193243	0.779404132
LOC112532953	0.180000413	-0.1162029	0.511184454	0.474626564	0.692264899
LOC112530164	-0.235047755	-0.11607678	0.675404273	0.411173753	0.641368033
LOC107053313	0.233242536	-0.115707533	0.670667223	0.412819079	0.643087944
TLX1	-1.280345279	-0.11559363	10.05915627	0.001515926	0.016282355
LOC107053043	0.317941306	-0.115529838	1.006384959	0.315770451	0.553296004
LOC112531841	-2.469602436	-0.114842144	24.9290857	5.95E-07	3.12E-05
PSTPIP2	0.126447452	-0.114494814	0.226637652	0.634028245	0.80403489
HNF4beta	0.152264028	-0.114234864	0.289356197	0.59063339	0.776205769
ANKRD33	-0.631387553	-0.113735301	5.999643783	0.014308767	0.079517313
LOC107053176	-0.98901433	-0.113728796	4.309267276	0.037905285	0.15049741
LOC107055306	-0.661411838	-0.112553984	7.05786096	0.007891817	0.05272162
LOC107054469	-0.866021055	-0.11004035	5.602027277	0.017939707	0.092567539
SGPP2	-0.298118997	-0.109668566	1.146324379	0.28431991	0.520236196
BRICD5	0.592760619	-0.10955868	2.993666608	0.0835907	0.249154317
LOC107051973	0.8467823	-0.108311808	8.960945935	0.00275812	0.024882057
SCTR	-0.186865751	-0.108207838	0.346471052	0.556117612	0.752930284
LOC100858393	-0.183708393	-0.106174989	0.18597999	0.666283713	0.823471421
CCR4	-1.768548139	-0.106026712	26.82264492	2.23E-07	1.33E-05
MIR181B1	0.097892837	-0.105948768	0.11520963	0.734289256	0.864249195
CLEC17A	0.549265256	-0.105010607	1.846433435	0.174198777	0.393633738
LOC112532332	-0.183570538	-0.104429967	0.464010848	0.49575544	0.707454319
LOC107052938	0.072666252	-0.103855466	0.072279257	0.788046639	0.895594133
LCP2	-0.722027679	-0.103170205	4.667877871	0.030731879	0.131417116
LOC112532648	-0.058296157	-0.102653837	0.041506049	0.838564236	0.923661189
LOC101748762	-1.298283612	-0.102128124	20.44505497	6.14E-06	0.000219524
LOC112532105	-0.30481194	-0.102036555	0.81784739	0.365810291	0.601039275
VCAM1	-1.464427066	-0.101808423	15.60317634	7.81E-05	0.001718607
LOC112531588	0.446638596	-0.101422695	2.166144928	0.141079511	0.347993306
LOC112530008	0.61069275	-0.101207603	5.896967381	0.015166977	0.082639938
LOC112530428	0.224419252	-0.101175063	0.735166513	0.391213629	0.624653104
LOC112529998	0.417184841	-0.100797055	2.452642273	0.117327221	0.308947308

LOC100858322	0.45324263	-0.099550032	1.338316758	0.24733103	0.481295186
JAKMIP1	-0.750517193	-0.098779432	7.238874819	0.007134167	0.048872908
LOC107057342	0.441535043	-0.097595678	2.294502333	0.129832819	0.329437015
NEURL2	0.418400124	-0.096218487	1.566326687	0.210741271	0.438511331
KCNA4	-0.174567212	-0.095044017	0.464364049	0.495591461	0.707281655
MIR1722	0.163328207	-0.094646775	0.305893593	0.580211041	0.769333578
CEP126	-0.403711895	-0.092796912	1.59637667	0.206417446	0.433682211
LOC101748824	0.339082406	-0.092150528	1.500896939	0.220533405	0.450049422
CCR2	-0.91959158	-0.091373849	6.450730075	0.011090676	0.066848744
LOC112533577	-0.07155067	-0.090931797	0.068780466	0.793120766	0.898320636
AKAP14	-0.661836274	-0.090286575	6.722056136	0.009522778	0.059900802
ADRA1B	-0.405435783	-0.088850802	1.750066147	0.185868417	0.408503379
CYP3A4	-0.173916414	-0.088175603	0.398300434	0.527968291	0.731720562
LOC107054158	-0.096388876	-0.086766505	0.066749151	0.796130187	0.900610289
LOC112532214	-0.253619397	-0.086280521	0.610492193	0.434602551	0.661129662
LOC107052896	0.161563737	-0.084612298	0.513751219	0.473519475	0.691617519
NTF3	-0.050596349	-0.084488761	0.030616816	0.861098008	0.935139468
LOC112532540	-0.278274	-0.083247045	1.230773154	0.267256731	0.502710277
IL12B	0.074161678	-0.08176356	0.045681002	0.830756722	0.919603556
LOC101751866	-1.331605206	-0.081582392	23.59661176	1.19E-06	5.48E-05
C24H11orf34	-0.444278086	-0.080992802	1.902863953	0.167758102	0.385646767
LOC101749635	0.708360371	-0.080302908	4.03295322	0.044619769	0.166715272
TMEM8C	-1.03385864	-0.079308023	9.886399025	0.001665049	0.017484607
LOC420486	-0.165280177	-0.078435994	0.195085822	0.658717016	0.818546179
LOC101748090	0.105539837	-0.078097769	0.204230371	0.651327664	0.813961802
LOC112532731	-0.507448934	-0.077803886	3.550883677	0.059513748	0.201127971
LOC112532452	0.560255226	-0.07679534	5.619794651	0.017758729	0.091921032
LOC112531727	-0.334165824	-0.075572186	1.291103765	0.25584464	0.490715523
LOC771972	-0.65570512	-0.075383747	4.068285437	0.043695605	0.164914059
LOC101748925	0.562492721	-0.075157584	3.733238674	0.053339875	0.187344215
LOC112533335	0.304651823	-0.074812239	1.343651839	0.246390984	0.479919939
HAPLN4	0.203392008	-0.074129465	0.447250899	0.503643358	0.7140723

LOC101749704	-0.126482355	-0.074013391	0.109826566	0.740341674	0.867900794
STK32A	0.581232546	-0.073940623	3.726066103	0.05356942	0.187949852
IL10RA	-0.473395464	-0.073816734	2.337580525	0.126285562	0.323529336
CYTH4	-0.166104044	-0.073471742	0.341423214	0.559008866	0.754469397
RALYL	-0.045524799	-0.073194024	0.035442653	0.850671121	0.930518733
OCSTAMP	-0.063882759	-0.073024168	0.050220268	0.822680433	0.916471907
LOC107054813	0.243847542	-0.072155414	0.741981922	0.389026736	0.622950073
LOC101749502	0.640486514	-0.07058261	2.914395605	0.087792299	0.256867404
ANGPTRL1	0.636982819	-0.070402031	4.751965124	0.029264856	0.127226739
LOC112531736	-0.332106456	-0.069356673	1.498820208	0.220852981	0.450215041
LOC101747448	0.440427165	-0.067836914	3.273370775	0.070412808	0.224022187
DRD2	-1.250708808	-0.067504686	17.00459247	3.73E-05	0.000939662
DLX2	0.528756613	-0.066638088	4.351150584	0.036983952	0.14825114
S100A9	-0.815019846	-0.066285836	2.607563271	0.106355071	0.290287555
GABRA4	-0.025051584	-0.066238988	0.009741968	0.92137535	0.964209577
LRIT2	-1.187452863	-0.066234059	7.877434108	0.005005545	0.038274401
CLCA2	-0.628847606	-0.065321911	4.308594278	0.037920284	0.15049741
LOC112532631	-1.294544954	-0.065225224	9.347546477	0.002232842	0.021699324
LOC101751655	-1.074297773	-0.064360542	13.46738219	0.000242747	0.004195796
LOC101748847	0.900450591	-0.063855956	7.764035504	0.005329675	0.039899818
LOC112530116	-0.597685336	-0.0631574	4.461312363	0.034670851	0.14237805
CHAD	-0.567292816	-0.061333703	3.45124563	0.06320412	0.209555471
TLR15	0.325755951	-0.06045896	1.979971704	0.159393388	0.374391592
LOC107053212	0.163282938	-0.059479914	0.393407798	0.530513536	0.733450191
LOC107053934	0.781844779	-0.059073859	6.376005292	0.01156735	0.068864234
LOC112532960	0.327656712	-0.057375076	1.802731705	0.179382596	0.400725035
LOC107049090	-0.236302592	-0.056797911	0.345997924	0.556387398	0.753031308
WDR17	-0.461332127	-0.056377414	2.748434435	0.097349706	0.273597379
LOC112530522	-0.106314054	-0.056342058	0.153307128	0.695394772	0.841252829
LOC112532922	0.148300132	-0.056036135	0.217905216	0.640640695	0.807749496
LOC112532993	-0.139751303	-0.055474837	0.247954578	0.618519019	0.794018603
KCNJ6	-0.15609589	-0.054844949	0.201529834	0.653488831	0.815062002

LOC112530126	-0.272728925	-0.054768849	0.774476114	0.378835883	0.613497141
BANK1	0.068080528	-0.054550875	0.050453928	0.822275279	0.916392801
HPRTL	0.258650794	-0.054319704	0.664712439	0.414901188	0.645405182
LOC107051597	-0.439428501	-0.051551179	2.745496474	0.097528781	0.273831189
LOC112530067	-0.216472813	-0.051409313	0.663141762	0.41545297	0.64572387
LOC107053380	0.502933114	-0.051378908	2.98608002	0.083983243	0.249899506
LOC421515	0.215643203	-0.050352867	0.772128188	0.379559485	0.61405408
LOC112532752	-0.09486061	-0.049567751	0.1082656	0.742127432	0.869036926
GNA14	0.771190958	-0.049308836	7.019382135	0.008063205	0.053413864
LOC112532986	-0.463444564	-0.047532558	1.390264443	0.238360447	0.470995489
LOC112532937	0.018088718	-0.046811283	0.004446587	0.94683428	0.977487125
LOC112532380	-0.814721828	-0.045696619	3.428889652	0.06406516	0.211472861
CCDC178	-0.396115095	-0.045239182	1.918713248	0.16599858	0.383692463
LOC107053368	0.426142952	-0.045063096	1.381485557	0.239848265	0.472323431
PKDREJ	-0.738554522	-0.044343425	5.518533035	0.018816031	0.095237709
LOC101751874	-0.266740096	-0.044100193	0.723009883	0.395158384	0.627941208
LOC107051682	-0.283993359	-0.043707605	0.821191164	0.364832128	0.600631582
LOC101749472	0.77596016	-0.043645858	7.453403741	0.00633166	0.045279214
KCNV1	-0.327144814	-0.043610379	1.519907666	0.217633467	0.446528515
LOC107053504	-0.465657303	-0.043429581	2.832497541	0.0923746	0.265645587
LOC112533158	-0.005488243	-0.043145481	0.000391736	0.984209046	0.992958912
LOC112530041	-0.433733455	-0.042622495	2.556971277	0.109808772	0.295971065
SP6	-0.487220033	-0.039941527	2.529580502	0.111729917	0.299578911
SMC1B	0.191022714	-0.039250009	0.365069354	0.545704237	0.745755064
LOC101747452	0.933654674	-0.03893254	11.08423522	0.000870647	0.01083699
LOC107054017	-0.008154393	-0.038178954	0.000459663	0.982894868	0.992424069
LOC101751464	-0.681379572	-0.038020444	5.714519872	0.016825165	0.089050529
LOC101751252	0.060096388	-0.037348612	0.027698611	0.867819356	0.938915609
LOC112532801	0.194728424	-0.037268812	0.466281731	0.494702738	0.706810241
DHX32	0.511066267	-0.035661152	4.124158111	0.04227505	0.161851083
TRAF3IP3	0.376827666	-0.035506523	2.090314942	0.148235508	0.358391903
MYLKSML	0.222820894	-0.035224734	0.82364328	0.364117105	0.599909391

CLDN9	-0.86940956	-0.034937419	12.47645937	0.000412113	0.0062616
LOC112533191	1.515085906	-0.034687393	3.3747347	0.066203238	0.215931472
LOC101750272	0.097532817	-0.033955466	0.123367105	0.725410902	0.858483911
GLRX	0.07463405	-0.033900478	0.081403899	0.775403829	0.888893846
FXYD2	0.460492416	-0.033834748	1.913749409	0.166547362	0.384420935
CPB1	-0.595185236	-0.032247104	2.636055392	0.104462624	0.286823373
MIR15C	-0.200383353	-0.031880273	0.34237448	0.558461823	0.754040314
LOC107050518	0.306760602	-0.031725882	1.316400745	0.251239027	0.485797673
GUCY2C	-0.87018807	-0.031681206	4.838469578	0.027831584	0.123675052
HIST2H3	-0.300180828	-0.031270122	0.968017507	0.325175105	0.563237511
LOC101748814	-1.252223588	-0.030820592	4.720945839	0.029797349	0.12862418
LOC107053558	-0.64093048	-0.030798447	5.565295205	0.018319921	0.093590284
LOC107054964	0.005182684	-0.030353135	0.000364035	0.984777518	0.9929845
LOC769139	0.435968664	-0.030243398	3.246254873	0.071586853	0.226197475
LOC107051776	0.133310836	-0.030131289	0.192986979	0.660442122	0.819884957
BLK	-0.641314412	-0.03003537	5.389429627	0.020259092	0.099989009
LOC107053635	0.069832702	-0.02979321	0.060100863	0.806336594	0.90650898
SYPL1	1.244216024	-0.02826351	19.70023364	9.06E-06	0.00029933
RNF223	0.038033158	-0.028146391	0.013355983	0.907994855	0.957821356
LOC107052635	0.107790146	-0.027681054	0.172117201	0.678236998	0.830633359
CD83	-0.258138913	-0.027045617	0.844388739	0.358144598	0.594439113
CD200R1L	0.175333282	-0.026233914	0.32269627	0.569991773	0.762434642
EPHX1L	-0.112377116	-0.023785785	0.175663528	0.675126731	0.828859984
LOC112531003	-0.305417942	-0.022394482	0.955896573	0.328222903	0.565717803
03/mar	0.276654631	-0.021007024	0.763427278	0.382258088	0.616769322
SLC8A2	-0.202523683	-0.01998927	0.299118364	0.584435656	0.77138756
LOC112533020	-0.045752497	-0.019841972	0.027069749	0.869314822	0.939840514
LOC112529978	-0.180559867	-0.019750203	0.557059691	0.45544737	0.678102286
RGS20	0.305899019	-0.018645819	1.542089654	0.214307032	0.443352051
LOC101752331	0.560519606	-0.01862671	2.474600692	0.115698823	0.305948797
LOC107053694	0.621326529	-0.018360646	3.066736606	0.079909737	0.24221431
LOC107049691	0.716765636	-0.017706458	3.42053661	0.06439008	0.212042203

LECT2	0.042995239	-0.015991658	0.003534773	0.952590538	0.979802073
GABRR3	-0.8889327	-0.015906917	6.99053193	0.0081942	0.054085663
CUTA	-0.199170818	-0.015403328	0.609817972	0.434856355	0.661222386
LOC112529963	0.239920653	-0.015269396	0.629257651	0.427627743	0.655213795
VNN1	-0.796917827	-0.014404108	10.76741618	0.001033026	0.012335592
OLFR1019L1	-0.661862894	-0.014123993	4.457076143	0.034756942	0.142491683
NAT	0.349823199	-0.013766107	1.432344199	0.231382245	0.462905147
LOC107054671	-0.417729059	-0.01375515	0.718884721	0.396509985	0.62935968
LOC112532179	-0.69152882	-0.012765524	5.402307515	0.020110146	0.099346821
MIR6572	0.122692691	-0.012051341	0.126220515	0.722383383	0.856852067
C5orf49	0.145960633	-0.011899386	0.359161525	0.548972272	0.747916769
LOC107053729	1.022546944	-0.009027272	7.406155512	0.006500108	0.046018535
LOC112530390	-0.052637908	-0.00813742	0.032721646	0.856452933	0.933075655
HMX3	0.128522694	-0.008110454	0.221773107	0.637692208	0.805931282
LOC107055316	-0.298123362	-0.007292082	1.634901096	0.201026496	0.427601802
CYP17A1	0.183799748	-0.007006842	0.363698685	0.546459222	0.746408774
LOC107051407	0.240277767	-0.006864563	0.98571181	0.320792709	0.558298979
LOC107052602	0.381728284	-0.006296552	1.941122624	0.163546693	0.380007306
LOC107052088	0.026852501	-0.005776674	0.010669899	0.917728684	0.96231374
RORC	0.665916577	-0.003365713	7.110818546	0.007662012	0.051641932
HRH3	0.356804226	-0.00309641	1.229734645	0.26745865	0.502975095
LOC107052937	-0.651860006	-0.002488454	8.597281637	0.003366652	0.028703757
TMEM179	-0.862239252	-0.001052944	5.846048987	0.015612064	0.084449875
LOC112531933	-0.360721482	0.000398232	2.125244152	0.144889691	0.353732918
LOC101749666	-0.525160387	0.002472537	3.935819958	0.047268092	0.172997432
LOC101750030	-0.134797885	0.002846419	0.230214965	0.631364481	0.802557168
CIB3	0.12925298	0.003774066	0.277563652	0.59830255	0.781222197
LOC107053008	-0.153746352	0.004229395	0.36142325	0.54771686	0.747130003
LOC101749199	2.068844159	0.004554406	40.87005982	1.63E-10	2.36E-08
CEND1	-0.454066403	0.004754516	2.209904466	0.137127163	0.341469047
LOC107050724	-0.387594488	0.005733636	1.376790229	0.240648646	0.473216236
C9orf84	-0.873932262	0.006553449	3.118443417	0.077410698	0.237687384

SLC30A3	0.148051276	0.00828187	0.315419621	0.574373375	0.765040788
IL17REL	0.085966725	0.008505118	0.085699711	0.769716972	0.88505745
LOC112531739	-0.533449739	0.008749586	3.6057364	0.057580562	0.19718796
RASGRF1	0.98044442	0.009221214	12.29136211	0.00045506	0.006739878
LOC112531219	0.038530932	0.009638251	0.018394867	0.892115625	0.951008202
LOC100857975	-0.272455294	0.009760978	0.609143205	0.435110591	0.661347199
LOC107051979	-0.028193678	0.011239879	0.010803075	0.917218678	0.962184964
SDSL	0.002935007	0.011545803	8.19E-05	0.992779963	0.996595552
CHST8	-0.840185463	0.011803104	5.870756003	0.015394439	0.08340978
C2CD4C	0.241300877	0.011985165	0.737899134	0.390334693	0.623951561
LOC112533590	0.525844035	0.012597215	1.624038767	0.202529558	0.429185302
SLC22A7	-0.741840448	0.013782224	7.36133847	0.006664117	0.046730829
LOC107053292	-0.300844138	0.01408978	1.343740187	0.246375454	0.479919939
MIR1725	-0.174747185	0.015283387	0.467286636	0.494238099	0.706269033
CCR5	-0.657288905	0.015750035	4.506612661	0.033764044	0.140023865
ADTRP	0.137909695	0.015773891	0.273904228	0.600724726	0.782591357
LOC112532621	-0.175863674	0.016116821	0.219864271	0.639143357	0.806858657
MOCS2	-0.225686258	0.016720051	0.458083926	0.498520817	0.709917514
LOC112531002	0.290808911	0.018909039	0.866998915	0.351787478	0.588218976
LOC107054637	-0.210256361	0.019572117	0.58358099	0.4449121	0.669687945
SLC6A19	-1.197768231	0.02040011	19.18665096	1.19E-05	0.000373695
TMC5	-0.310533018	0.020599231	2.056444832	0.151563424	0.363261254
JPH2	0.65068112	0.022401546	3.030700048	0.081702758	0.245421484
APOF	0.731223783	0.023098119	6.899315237	0.008622877	0.056016361
LOC101747773	0.507156408	0.023721545	4.999106248	0.025360411	0.116144048
IL5RA	0.600277446	0.024218034	4.435224201	0.035204596	0.143637893
SNORD20	0.240611174	0.024674153	0.889583949	0.345590079	0.582234539
SELE	0.375198645	0.026258616	1.563387377	0.211169932	0.438848349
LOC112533498	-0.170838732	0.026864968	0.357648879	0.549814896	0.748445079
COL20A1	0.463761861	0.02698096	1.64829735	0.199190829	0.425066152
CLC2BL2	0.331271293	0.027256368	1.137929326	0.286090297	0.522416162
LOC112532907	0.869383322	0.027548429	6.889181642	0.008671896	0.056223818

LOC101747932	-1.150539286	0.029363738	3.617015745	0.057191375	0.196197771
CEP350L	-0.165870781	0.029632289	0.372623542	0.541577675	0.742118605
GFI1B	-0.27235506	0.030310467	0.470933709	0.492557932	0.705092716
LOC112531094	-1.672437232	0.030550323	39.23693703	3.75E-10	4.68E-08
PCDHA12	0.362514959	0.030758155	1.845820876	0.174270235	0.393687083
PROK1	-0.430355175	0.03088727	2.584395116	0.107921629	0.292706512
LOC101751443	-0.016241333	0.030943205	0.004052149	0.949243749	0.978504123
LOC112530940	-0.408217759	0.031675812	0.744752815	0.388142623	0.622079172
SLC28A2	-0.324718113	0.031885682	1.932094204	0.16452951	0.381634511
LOC112531035	1.307643163	0.033037332	6.11452042	0.013407579	0.076392558
NOX1	-0.155530067	0.033445865	0.362890906	0.546905067	0.746615874
LOC107054300	0.288275546	0.033691637	0.831519709	0.361833457	0.598210789
DUOX2	-0.425182702	0.034480552	1.351031631	0.245097847	0.478590847
LOC107054778	0.70413238	0.034712038	2.850335739	0.091354857	0.263889312
LOC107053753	0.22529396	0.034737301	0.418753842	0.517559509	0.723314877
CATIP	0.350305231	0.035550436	1.507768486	0.219479912	0.448737316
LOC112533161	0.403976855	0.035714287	1.675476434	0.195526614	0.420463988
LOC107054177	0.207622378	0.035919701	0.49069751	0.483616326	0.698336
KYNU	-0.04252279	0.036644211	0.020473994	0.886221184	0.948791653
C1QL4	0.35094971	0.037498216	1.460061209	0.226920382	0.456755952
BLEC2	0.377323431	0.03844328	0.817439179	0.365929955	0.60110042
MYLK2	-0.763884557	0.038459222	11.56770124	0.000671073	0.009025635
LOC107054016	0.291496088	0.038536197	0.920373631	0.337376888	0.57457428
CCL26	-1.297972747	0.039087888	17.76633336	2.50E-05	0.000674855
CYTIP	-0.282270536	0.039520406	1.400006975	0.236722403	0.469065191
LOC112532296	-0.330918922	0.039543615	1.600734245	0.205799189	0.432697913
LOC112533459	0.277481973	0.04069991	0.826218759	0.363368198	0.599180649
LOC112532755	-0.837341807	0.041101042	6.455391918	0.011061614	0.066698007
PRSS57	-0.055246748	0.043511193	0.044153769	0.833567786	0.921258649
LOC107055337	-0.216953444	0.043919829	0.593982843	0.440883098	0.665449585
LOC112531494	-0.207883291	0.044148692	0.616016609	0.432531402	0.659437063
MIRLET7I	0.325652376	0.04465987	1.13880599	0.285904769	0.522264984

LOC107054103	-0.267467281	0.04568807	1.091445389	0.296151179	0.533052686
LOC112531242	2.016042414	0.045713176	31.46936515	2.03E-08	1.68E-06
SEBOX	0.344523761	0.046418208	2.042896404	0.152918236	0.365312074
LOC101749270	-0.002924739	0.046462395	8.83E-05	0.99250453	0.996459368
LOC101749149	-0.713554353	0.046636569	4.374830622	0.036473455	0.147100663
HIST1H2A3	-1.059497931	0.047289873	11.3443588	0.000756775	0.009797586
LOC107052279	0.401930032	0.047380241	1.568819938	0.21037847	0.438234642
LOC107054091	0.30738637	0.048038711	1.224935996	0.268394132	0.503525873
LOC107051868	-0.528905521	0.048153697	4.256909071	0.039090987	0.153922517
TGM3	-1.009680744	0.048230434	11.16021094	0.000835704	0.010553732
LOC112532099	0.432672674	0.048525416	1.911668897	0.166777993	0.384737399
LOC107052451	0.85866145	0.049247572	4.826460327	0.028026114	0.124170626
LOC112532416	-0.166650873	0.049308403	0.367791395	0.544210615	0.744387837
LOC112530077	-0.095653901	0.050106454	0.083023429	0.773241209	0.887657604
CLRN2	-0.47142146	0.050923606	2.582545084	0.10804781	0.29295217
PGC	0.181424475	0.050936363	0.616366221	0.432400835	0.659360183
ARHGAP15	0.005470071	0.05094195	0.000483364	0.982459497	0.992167118
LOC107054706	0.069695917	0.051085082	0.046248446	0.829724812	0.918963441
CNIH3	-0.624432306	0.051093427	3.348682631	0.06725882	0.21763368
ANKEF1	-0.355740002	0.05159061	1.842539715	0.174653569	0.394160332
LOC101751230	-0.531703591	0.051773247	4.791542655	0.028599801	0.125601973
LOC107053901	-0.189167829	0.052272241	0.372574061	0.541604517	0.742118605
KIF19	0.171719757	0.052933637	0.289812388	0.590340782	0.776026179
LOC107052835	0.461573639	0.053332996	3.569038614	0.058866383	0.199885747
LOC101748473	-0.203304234	0.053792112	0.613542862	0.43345697	0.659992083
LOC101747379	0.409210455	0.054687665	3.260185101	0.070981114	0.224960367
LOC112533298	0.212004697	0.056170095	0.569170708	0.450588643	0.673978377
MAL	-0.170163573	0.056750029	0.351709201	0.553147184	0.751427137
GPR157	-0.047885909	0.057593735	0.035364949	0.850832981	0.930592432
LOC101750426	0.6764986	0.059499092	6.447600524	0.011110229	0.066860676
RPP14	0.028614809	0.060923054	0.013984242	0.905865622	0.956986313
LOC107056992	0.242381071	0.06110312	0.572521756	0.449258574	0.672600295

LOC112531519	-0.261010232	0.062160088	0.318840164	0.572305499	0.764142079
LOC107053268	-0.006016618	0.06232202	0.000470857	0.982687866	0.992336842
LOC107049132	1.675576033	0.06289382	11.3802054	0.000742309	0.009671177
OR52R1L2	1.665849143	0.064384655	30.25804088	3.78E-08	2.87E-06
LOC112532924	0.335740854	0.064511613	0.941590868	0.331869047	0.56937808
NR4A2	0.060724143	0.064540847	0.043669003	0.834470646	0.921720365
HBEGF	0.295035036	0.064602141	1.352018874	0.244925484	0.478367816
CYP4V2	-0.337965803	0.064941063	1.409558721	0.235129662	0.467277607
CNGA2	1.15507276	0.065042155	14.29413075	0.000156352	0.002926924
A1CF	-0.219840752	0.06508406	0.304275357	0.581214488	0.770125921
LOC100859202	-1.187001489	0.065618689	15.41281676	8.64E-05	0.001848786
LOC112533373	0.346657608	0.066233571	1.616746327	0.203546063	0.430292477
LOC107053854	-0.246438567	0.066514348	0.899334241	0.342960288	0.58012043
ADORA1	0.473735019	0.066780586	2.696473622	0.100570467	0.279117395
DAZL	-0.661725817	0.067188003	5.531573244	0.018676305	0.094792905
CXCR1	-0.824338697	0.068072575	10.54138826	0.001167307	0.013404074
SRRL	-0.250482834	0.068999535	0.810635793	0.367932345	0.60268481
IPCEF1	-0.277683056	0.069372482	0.922970431	0.336696241	0.573918636
VTG2	-0.589428581	0.069426127	4.034214233	0.044586434	0.166705242
LOC101747276	-0.060554545	0.069590608	0.045660719	0.830793732	0.919603556
LOC107051660	0.350368124	0.070072827	1.024559911	0.311439822	0.549202656
NODAL	0.12643752	0.070656303	0.200289195	0.654487517	0.815640639
LOC107055472	0.389799437	0.071266248	1.252068507	0.263157771	0.498361276
LOC107054098	0.163069376	0.072172644	0.358164877	0.549527186	0.748347183
LOC769098	0.137485835	0.073318534	0.1998401	0.654849943	0.815897314
LOC101750051	0.23743729	0.074481	0.922185297	0.336901838	0.574121763
MAP3K7CL	0.455109585	0.075268748	2.429887902	0.119041411	0.311567746
LOC426456	-0.185487429	0.076387893	0.455587173	0.499693568	0.710528058
RASSF10	0.377256609	0.078113723	1.655065519	0.198270887	0.423935622
PTGES	-0.459963042	0.07861201	2.378612847	0.123006729	0.31775129
C7H21orf58	-0.187788678	0.078848729	0.697959901	0.403470047	0.635202763
LOC112532307	0.164929296	0.079174344	0.462862382	0.496289265	0.7079291

SLC9A2	-1.820126396	0.079337284	42.39349102	7.46E-11	1.17E-08
LOC107054197	-1.20235252	0.079993584	18.65780491	1.56E-05	0.000465399
LOC107053430	0.02106039	0.081088328	0.004892438	0.944236666	0.976060582
CCL28	-0.254596863	0.08145451	0.718493542	0.3966385	0.629433501
EVX2	-0.492332805	0.081685739	1.873175931	0.171111779	0.389828743
LOC107055309	0.096930832	0.082273057	0.186460906	0.665878644	0.823156351
LOC107052242	0.165791067	0.082284141	0.316616911	0.573647886	0.764869254
HAO1	0.941673838	0.083146839	13.23276183	0.000275098	0.004605938
CEP57L1	-0.354485397	0.085030743	1.982518437	0.159125346	0.374165128
LOC107049568	1.641577295	0.086204899	13.47453882	0.000241823	0.004184218
PTH	-0.758873	0.086274223	2.540632541	0.11095033	0.298363733
HORMAD2	0.283311914	0.086473466	1.280852211	0.257740641	0.492541889
LOC107054810	0.052439282	0.087942973	0.030158234	0.862131683	0.935776837
LOC107052933	0.074537407	0.088293458	0.086811998	0.768269851	0.884464473
CCDC169	0.200064054	0.088319586	0.493937447	0.482176137	0.697256981
BLEC1	-0.765218084	0.09006858	4.379402548	0.036375746	0.146850563
LOC100858315	1.4703784	0.090495892	16.14343063	5.87E-05	0.001360942
LOC101751390	-0.010062512	0.090654956	0.002033806	0.964029406	0.984735636
LOC424473	-0.226366352	0.091394248	0.568687845	0.450780804	0.674143245
CD200L	-0.098718354	0.0915011	0.073499366	0.786308227	0.894849393
KCNG4	-0.81014553	0.092198072	7.325743361	0.006797384	0.047314588
TNNI3K	-0.828200867	0.092397044	7.418102294	0.006457088	0.045954099
LOC107052204	0.133173694	0.093707638	0.221193657	0.638131913	0.806386086
LOC101751546	-0.230705596	0.094382441	0.554202335	0.456605688	0.679028161
BLB3	-0.631288912	0.094661227	5.21872012	0.022344991	0.106823599
LOC101748327	-0.127037057	0.094783743	0.313777708	0.575371232	0.765607847
MIR6650	-0.435411544	0.095002248	2.75929648	0.096690749	0.272657543
ASB16	0.382841021	0.095589565	2.188023153	0.139087726	0.344474494
LOC112529977	-0.065600101	0.095611737	0.054130593	0.816025571	0.912856407
LOC112531200	-1.264349145	0.095700946	18.86674485	1.40E-05	0.000425569
LOC107052267	0.286794608	0.096819042	1.535666135	0.215264047	0.444471699
LOC107052060	-0.350794559	0.098232855	1.095074358	0.295349624	0.532228922

LOC112531850	-0.393606575	0.0992237	1.916413685	0.166252553	0.384009792
LOC112530090	-1.415331869	0.099472009	17.29636304	3.20E-05	0.000822232
IRF4	0.828154716	0.099482844	4.122130123	0.042325753	0.162007506
LOC112531005	0.180922612	0.0997765	0.498384271	0.480210921	0.696466157
LOC112533478	-0.656996914	0.100032689	6.356770904	0.011693417	0.069288863
LOC112531403	-0.699597603	0.100637537	2.269515077	0.131941181	0.333300054
LOC101751629	0.464075473	0.10101402	1.780912877	0.18203727	0.404240659
LOC112532756	-0.137531921	0.102021719	0.326078785	0.567977215	0.760970118
LOC107055085	0.756284434	0.1020592	4.869847858	0.02732991	0.122005878
LOC107055079	-0.611337812	0.103212256	3.69534369	0.05456454	0.190103642
LOC107052922	-0.264564875	0.103396164	1.299312886	0.254338771	0.489090158
NEK5	0.587171833	0.103479671	3.933079876	0.04734516	0.17312547
SLC45A2	0.138012865	0.10393954	0.21460314	0.643183187	0.809389671
SOD3	-0.868504895	0.10432663	6.871460935	0.008758302	0.05658087
LOC101750459	0.135861523	0.105481117	0.237620828	0.625929784	0.799043801
CBLN4	0.053261233	0.10552139	0.025590615	0.87290418	0.94177516
SLA	0.037173128	0.105941675	0.019668385	0.888467192	0.949552787
OR5AS1	1.374292098	0.106852858	15.3448186	8.96E-05	0.001901693
HIST1H46L2	-0.783755232	0.106947082	9.607511354	0.001937831	0.019659066
NOTO	-0.533017165	0.107814882	2.172685415	0.140480731	0.347036546
LOC101748407	0.418763174	0.108499988	3.208586437	0.073252744	0.229114978
LOC107054516	-1.470822435	0.109378257	19.70207474	9.05E-06	0.00029933
LOC107051980	0.54473484	0.11024616	1.013940557	0.313960651	0.551828937
LOC112531994	-0.240310394	0.110722269	0.900605032	0.342619532	0.579782434
CLDN19	0.143290243	0.112424677	0.337383642	0.561343341	0.75591721
LOC107052550	0.476319495	0.113148771	3.921633156	0.047668551	0.173882954
LOC427161	-0.412724423	0.113335354	1.532914983	0.215675484	0.444540058
CD14	-0.31146322	0.113990371	1.494538209	0.221513664	0.451003135
LOC112530140	0.060364369	0.115297799	0.056324921	0.812401783	0.910073616
LOC107053233	-2.23089548	0.115621865	47.29457651	6.11E-12	1.29E-09
LOC112532058	0.046392248	0.115864938	0.030699091	0.8609134	0.935018438
LOC107054737	-0.169353067	0.116346232	0.496443425	0.481067033	0.696704901

LOC107053624	0.747687647	0.116620218	2.526189021	0.111970352	0.299735177
CAPSL	0.459734174	0.11673017	3.651903764	0.05600516	0.193200192
TEX33	-0.253095382	0.117059511	0.976655957	0.323025793	0.560580359
LOC425049	-0.066583724	0.11794592	0.080948481	0.77601615	0.889168286
LOC112533488	-1.663830968	0.118088147	36.145675	1.83E-09	1.91E-07
LOC112530233	-0.223491411	0.118153559	0.829623002	0.362381564	0.598813883
PLAC9	-0.243545607	0.118417593	0.648201287	0.420756501	0.650403779
LOC112532134	-2.047310333	0.119393937	29.17337017	6.62E-08	4.76E-06
LOC107053413	0.270650475	0.119876309	1.128432415	0.288109955	0.524894742
IL12RB1	0.157180297	0.121195053	0.373983456	0.540840909	0.741504513
MALRD1	-0.13496527	0.121560342	0.172840191	0.677599877	0.830409323
DMRTA2	0.232422038	0.121944185	0.721602082	0.395618899	0.628430253
LOC112532757	-0.834569879	0.123139708	7.941905894	0.004830293	0.037368349
PCSK9	-0.865284357	0.123937023	8.062554467	0.004518943	0.035514684
PROK2	-0.089752974	0.124322767	0.123333963	0.725446295	0.858483911
RGS16	-0.271363674	0.125850578	0.331803421	0.564599093	0.758587325
RBP	0.097366733	0.126839619	0.102265715	0.749127445	0.873205161
LOC112532772	0.757094739	0.126900412	9.285078369	0.00231028	0.022102937
LOC112532814	-0.189055198	0.127130939	0.556569212	0.455645872	0.678213733
LOC112530571	-0.667371742	0.127207349	5.064094412	0.024426464	0.113253726
LOC112530028	-0.807723902	0.127813368	7.468433924	0.006279015	0.044959759
LOC107054838	0.594321297	0.128277187	4.202558319	0.040363043	0.15741623
LOC107054255	-0.878370909	0.128445484	10.29346748	0.00133502	0.014783144
HMG20B	-0.619109991	0.128565949	4.213441125	0.040104901	0.156938441
TTC34	-0.09503455	0.128622236	0.181680941	0.669932628	0.82519211
OTOGL	-0.739342975	0.128993786	6.592308827	0.010242026	0.063073553
LOC112530069	-0.747229577	0.129096714	6.335566004	0.011834035	0.069781745
EPYC	-0.704974495	0.130603673	5.43246592	0.01976575	0.098469698
LOC101750820	-0.41681079	0.132223528	2.05779478	0.151429179	0.36317842
SELP	-0.344298719	0.13288424	1.062317898	0.302687121	0.540542759
LOC107053627	-0.096406003	0.133084496	0.078628154	0.779165174	0.890667797
LOC107053538	-0.169928099	0.133503203	0.427529143	0.513203857	0.720604956

LOC427656	-0.04188878	0.13444602	0.019517204	0.888893875	0.949773712
LOC107053088	0.33309187	0.134729157	1.611453872	0.204287553	0.431078698
ELOVL3	0.482379413	0.134834999	3.767749424	0.052249891	0.184698595
NOXRED1	-0.474370292	0.135141426	3.880517556	0.048849497	0.176663399
LOC107054927	-0.06953981	0.136236258	0.04990844	0.823222671	0.916737351
LOC107049554	-0.294453404	0.136297053	1.292077013	0.255665536	0.490546484
LOC101750987	0.556102408	0.137521383	2.157146165	0.141908037	0.349095043
CSTA	-0.797402925	0.137977906	6.682634724	0.009735662	0.060935836
LOC112531992	-0.047793612	0.138682903	0.024234289	0.876290259	0.943959209
LOC107054206	0.143427604	0.140132782	0.302797894	0.582133686	0.770499657
PKDCCB	-0.641815855	0.140345029	4.87170781	0.02730047	0.121907526
LOC112530104	0.053796116	0.140498762	0.020814759	0.885284731	0.9486918
LOC107052875	-0.128309645	0.14087486	0.224288082	0.635791861	0.804827684
SPIK7	0.132571411	0.14157601	0.238289382	0.625444352	0.798796471
LOC107053873	-0.68110591	0.141634057	4.867995646	0.02735926	0.122070668
LOC112532270	0.470978474	0.144498938	3.737311789	0.053209987	0.18719099
SLC35F4	0.463600241	0.146043342	3.126831757	0.077013274	0.237108726
P2RY14	0.425782802	0.146114173	2.856639658	0.090997415	0.263156847
GPER1	0.144640631	0.146341419	0.430924422	0.511535697	0.719032808
NTSR1	0.023705518	0.147045862	0.011138488	0.915948096	0.96163855
LOC112531575	-0.531397571	0.147866174	3.724008637	0.053635458	0.188141434
LOC112532068	0.515424319	0.147878152	1.740795571	0.187038054	0.410088099
LOC107052084	0.775665443	0.147929649	11.72186248	0.000617701	0.00847729
RIIAD1	0.259332455	0.148107363	0.61945539	0.431249739	0.65855744
MHCIA2	0.44549059	0.148928892	0.926364103	0.335809496	0.573091191
LOC107054575	0.366624959	0.150159353	2.091594022	0.14811146	0.358220275
LOC101751425	0.151521012	0.1506172	0.246752098	0.619371367	0.794431043
S100A14	-0.054688514	0.15102932	0.050546117	0.8221157	0.916392801
LOC107049314	-0.546428953	0.152303765	5.375961453	0.020416086	0.100522648
LOC107053801	0.242609566	0.152348878	1.079954438	0.298707749	0.535720546
PTPRR	0.097174974	0.152516887	0.092088601	0.761538536	0.88065738
LOC107054466	0.80460582	0.152847224	7.378221959	0.006601841	0.046503977

IKZF1	0.464827439	0.153097243	3.332367789	0.067929016	0.219170974
LOC112531744	0.110452758	0.153795302	0.151775361	0.6968445	0.842201708
LOC112532265	1.09800541	0.154762159	14.04800897	0.000178202	0.003254518
LOC101748035	-1.157334798	0.155738536	20.56779203	5.76E-06	0.000208152
LOC107054786	-0.341945181	0.157167574	1.563019491	0.211223656	0.438848883
LOC101752000	-1.524547785	0.158332104	34.88307256	3.50E-09	3.47E-07
CLRN1	-0.691895145	0.158846871	7.691635353	0.005547734	0.040991449
LOC107053409	-0.441916341	0.159006971	4.138566667	0.041916648	0.16092824
LOC107052540	-0.05181764	0.159802017	0.018669843	0.891317228	0.951008202
OR52B2L	-0.450321335	0.16019901	2.544104015	0.110706697	0.297805901
LOC101749716	-0.181093581	0.16084283	0.370516996	0.542722596	0.743026497
TRPM8	-0.850521236	0.161363911	10.98197203	0.000920025	0.011306204
NOS1	-0.143623644	0.161698785	0.265061877	0.60666371	0.786666377
LOC112532697	0.313669227	0.161931357	1.181947285	0.276959923	0.512122209
ACPP	-0.447917229	0.1627405	2.494790934	0.114223543	0.303152969
LOC112533447	-0.320632431	0.162768011	1.207849744	0.271758393	0.506602963
LOC112533307	0.05585123	0.163579425	0.044435301	0.833045818	0.92106133
LOC112530857	0.564136763	0.163772097	4.352373551	0.036957405	0.148216938
NGF	0.221511231	0.164364827	0.752501729	0.385685323	0.620194663
LOC112531572	0.459560228	0.165038743	4.433334312	0.035243594	0.143725734
PHYHIP	0.02204487	0.165843462	0.006495325	0.935765175	0.972003524
HCRT	-0.158531465	0.167257038	0.343166621	0.558007067	0.753797412
ADORA3	0.437391204	0.167317355	3.022440972	0.082119777	0.24613496
LOC112533096	-0.093827303	0.168401327	0.073594151	0.786173827	0.894763475
LOC101749673	0.03663231	0.169945392	0.025663697	0.872724372	0.941683904
LOC101751084	0.090129228	0.171351107	0.140627919	0.707657572	0.848663024
LOC107053705	0.62596877	0.174095887	5.079375385	0.024212091	0.112672499
IQCG	-0.218838378	0.174173716	0.982503965	0.321581399	0.559131648
RPP25	-0.232734011	0.174416092	0.594618547	0.440638692	0.6651417
LOC112533251	-0.141556216	0.175754321	0.233776974	0.628737292	0.800640159
HIST1H103	-0.44118382	0.17772852	2.976532603	0.084480077	0.250969429
LOC776874	-3.091576526	0.179454157	63.56873732	1.55E-15	7.28E-13

DOK2	-0.468649264	0.180358263	2.45836498	0.11690041	0.308318039
LOC107054139	-0.466377591	0.181003295	2.873050276	0.090074006	0.26193057
LOC107053834	-0.167091652	0.181443161	0.28212325	0.595312929	0.778810164
LOC107054230	-1.944529803	0.181544893	47.10494781	6.73E-12	1.37E-09
MFRP	-0.499901655	0.182109551	4.836148868	0.027869066	0.123754456
LOC112532783	-0.168306808	0.182227765	0.353124045	0.552349989	0.750840072
KRT9L	-1.293413194	0.182592334	29.65883911	5.15E-08	3.80E-06
LOC107055292	0.608357224	0.182738084	5.091025848	0.024049961	0.112203604
LOC112532170	-0.355213402	0.183251189	1.906961625	0.167301157	0.38535002
OTP	0.148707448	0.1841664	0.132025663	0.716340939	0.853085451
LOC112531738	-0.263626361	0.185167663	0.99719865	0.317989303	0.555837854
XK	-0.457084491	0.187427401	4.083384633	0.043306828	0.164158917
LAMC2	-0.011294593	0.187543563	0.000901781	0.976043386	0.990018742
LOC107054621	-0.866738646	0.187896586	4.428961433	0.035334002	0.143987371
LOC776146	0.362853648	0.190181033	1.699297954	0.192379815	0.416692096
ABCA8	-0.235450984	0.190234227	0.984748886	0.321029188	0.55845712
FADS6	-0.432474987	0.191109038	2.419856526	0.119805903	0.312673455
LOC107053787	-0.791598756	0.191318555	8.212800828	0.00415959	0.033404611
TLR7	-1.179730548	0.191711492	9.615429651	0.001929493	0.019598648
DCDC1	-0.229691052	0.191782418	1.127111195	0.288392366	0.525060454
LOC112532462	-0.008197767	0.192091212	0.000936575	0.975585743	0.990018742
EVX1	-0.231750034	0.19417002	0.437277411	0.508439469	0.717589112
LOC112530339	-0.336952815	0.19417173	1.946655356	0.162947724	0.379400564
CACNA2D4	-0.486874727	0.194200049	3.824282879	0.05051496	0.181094481
LOC101751275	0.899982015	0.194751839	8.760047397	0.00307901	0.026920891
LOC112530277	-0.311068447	0.195272793	0.812324564	0.367433884	0.602396245
LOC107054242	0.143703467	0.196522509	0.329125251	0.566174627	0.759716503
LOC107053073	-0.519525278	0.197723223	2.243688978	0.134160572	0.336834333
C5AR1	-0.171246811	0.198691634	0.29965736	0.584097305	0.771126537
LOC107054314	0.194440462	0.199240903	0.536088743	0.464058214	0.685157282
LOC112532085	0.180342296	0.199270868	0.577146504	0.447432979	0.670925335
LOC112530254	-0.103938503	0.199366724	0.17236615	0.67801744	0.830532564

SLC1A6	-0.177109957	0.200097082	0.461482717	0.496931837	0.708580016
FAM3B	0.059912106	0.200135143	0.074401383	0.785032966	0.89437421
LOC107053660	-0.127985544	0.200680595	0.118301729	0.73088375	0.862250839
LOC112529973	-0.027629376	0.201084607	0.013007253	0.909198677	0.95829261
LOC112532769	-0.231638825	0.201429812	0.570631424	0.450008114	0.673300375
LOC107054592	0.138589965	0.202078811	0.287188887	0.592027615	0.776980894
PRSS56	0.122489449	0.202306722	0.147643436	0.700797624	0.844622054
LOC112532241	0.875467645	0.205261135	15.65746764	7.59E-05	0.001678946
LOC107054505	0.310635901	0.205585255	1.945510144	0.163071498	0.379467221
LOC112530015	-0.160769674	0.205806383	0.385647929	0.534595953	0.736378403
ATP1B4	0.156656312	0.208476103	0.393246043	0.53059806	0.733450191
KIF28P	-0.331318758	0.208916598	1.83073276	0.17604102	0.396056192
LOC112531752	0.136208459	0.209344937	0.186460826	0.665878711	0.823156351
LOC101750257	-0.27674598	0.21021555	1.094541686	0.295467104	0.532228922
LOC107054460	0.397754699	0.210433488	0.836479072	0.360405703	0.597047805
LOC107054281	0.086159318	0.210546491	0.106199628	0.744512996	0.870384134
LOC107054467	1.311624404	0.211271217	9.741611007	0.001801429	0.018607986
CGNRHRL	-0.189879642	0.211596735	0.619888378	0.431088769	0.658466827
VWA5A	1.192283277	0.212245302	23.45322564	1.28E-06	5.84E-05
TNFSF15	-0.247039526	0.212447106	0.829492602	0.362419289	0.598816087
MIR7-1	0.142458014	0.212491104	0.400572486	0.526793749	0.730729536
FAM167B	-0.040263614	0.212919362	0.032614741	0.856685075	0.933188991
LOC112532827	0.353843847	0.215632534	2.268282185	0.132046193	0.333361256
LOC112531945	-1.606765962	0.215976647	13.51765592	0.000236329	0.00410211
SMPX	0.520213402	0.216081335	3.826640222	0.050443953	0.180988995
GFI1	0.104279573	0.21688121	0.095617433	0.757153659	0.877759873
CLDN16	0.133951741	0.21938725	0.155748287	0.693101521	0.83958963
SLCO4C1	-0.000451454	0.21942797	4.78E-06	0.998255335	0.99928772
LOC112531022	0.669589358	0.219592629	8.802026191	0.003008962	0.026505604
LOC107052667	-0.006555753	0.219690317	0.000497858	0.982198492	0.992086194
LOC101751179	-0.000570673	0.220211386	3.34E-06	0.998542077	0.999310367
LOC107054925	-0.028271508	0.221246455	0.014207218	0.905121631	0.956683975

LOC107053667	0.014130481	0.221933164	0.003057383	0.955904556	0.981041173
LOC112532378	0.42923838	0.223437605	0.702314147	0.402007198	0.634366465
LOC107052314	0.320353605	0.223471912	0.869900583	0.350982833	0.587351013
LOC420160	-0.120523601	0.223572015	0.266424477	0.605740411	0.786139165
LOC112532232	0.29195611	0.224583785	1.412248127	0.23468355	0.466672847
PTGER2	-0.464211108	0.225018653	4.249016908	0.039273059	0.154454632
GPR34	-0.830060907	0.225199416	7.084669803	0.007774614	0.052089283
KCNA1	-0.266514563	0.225321341	0.730551638	0.39270444	0.626036771
LOC107053298	-0.305933768	0.225561988	1.203517708	0.272619734	0.507375894
C3H8ORF80	-0.503837074	0.225642354	2.359853221	0.124493902	0.32014752
LOC101751045	0.737824641	0.227106707	7.186018217	0.007347385	0.049965525
MIR3539	-0.121572618	0.228007056	0.253755791	0.614442838	0.791137476
LOC112529989	-0.190964364	0.229999485	0.260472081	0.609796014	0.787717486
SKOR1	0.541634136	0.230525911	2.84399343	0.091716015	0.264537605
LOC112532459	-1.335450483	0.230864427	14.63601579	0.000130399	0.002545324
LOC112532101	0.377764581	0.231673309	1.686486998	0.194064708	0.418721657
INSC	0.756413319	0.231724822	9.293223974	0.00230003	0.022081095
CYP2AC2	-0.154492135	0.232263477	0.348860216	0.554759044	0.752498563
LOC107049412	-0.181203787	0.233173006	0.493858495	0.482211148	0.697256981
MYH13	-1.589640229	0.233532752	11.64203826	0.000644779	0.008739572
LOC107053116	-0.033753098	0.234639207	0.01428607	0.90485995	0.95651142
LOC107049448	0.2557832	0.234686366	0.532651794	0.465494119	0.686036347
LOC107050538	0.747474358	0.235621473	7.275115295	0.006991641	0.048237922
HS3ST2	-0.29458698	0.23592296	1.279699899	0.257954842	0.492541889
LOC112532233	-0.309524369	0.236093158	1.062215101	0.302710515	0.540542759
TMEM81	-0.019579034	0.236284282	0.004033315	0.949361685	0.978504123
PCSK4	0.089237235	0.23637825	0.121529277	0.727381748	0.859661471
PMAIP1	-0.184525935	0.236458294	0.726151824	0.394133383	0.627342312
LOC112531422	0.606977828	0.238201042	3.306751604	0.068995752	0.221280185
LOC107054486	0.318020038	0.238522358	1.437429103	0.230555821	0.461942159
LOC112532113	0.001324651	0.238998656	1.55E-05	0.996862869	0.998628203
LOC101750229	0.485157344	0.239891334	2.567117394	0.109106385	0.294995985

LOC101748751	0.003314457	0.241915174	0.000157256	0.989994655	0.995439172
LOC101748649	-0.225080558	0.243725074	0.80546087	0.369465658	0.604029548
LOC107051622	0.002231538	0.243753839	8.64E-05	0.992583744	0.996459368
REC8	0.986428626	0.244076199	5.064281676	0.024423825	0.113253726
LOC112530323	-0.290080543	0.244985353	1.912949181	0.166636027	0.384517714
HTR1E	0.080958877	0.246789742	0.053043354	0.817849782	0.913936717
CD48	0.055809356	0.250920675	0.043494808	0.834796354	0.921889882
NPAS4	0.221792531	0.25123582	0.288135861	0.591417598	0.776802089
LOC107053403	-0.00709951	0.25349315	0.000813027	0.977252502	0.990525896
NLRC5	-0.515331105	0.253562333	5.01957658	0.025062297	0.115289749
LOC112532947	-0.419786056	0.255315535	3.154966881	0.075696227	0.234396203
IP6K3	-1.262595108	0.255783104	21.88815481	2.89E-06	0.00011543
LOC101748697	0.217208771	0.259358335	0.82352618	0.364151207	0.599909391
CCDC96	-0.324874071	0.260848316	2.075545913	0.149676378	0.360973882
LOC107049890	-3.362687627	0.261839305	109.25655	1.43E-25	1.68E-22
LOC107051761	-0.859313875	0.261879728	6.823780578	0.008995195	0.057549473
SRGN	-0.446998205	0.262203481	3.172306427	0.07489661	0.232872277
LOC107051693	0.28967782	0.263294378	0.2487837	0.617932816	0.793494751
LOC101749448	-0.216413947	0.263686979	0.65364157	0.418813724	0.648558238
IHH	-0.127803582	0.264574555	0.163032253	0.686380001	0.836341361
LOC100858068	-0.03837813	0.264684233	0.01965681	0.888499803	0.949552787
LOC112531741	0.458607082	0.26480046	1.488277467	0.222483913	0.452027755
LOC107054628	-0.225783114	0.264898375	0.716668163	0.397238992	0.629797549
LOC101748470	-1.008276754	0.2654487	15.55705129	8.01E-05	0.001751681
LOC112531014	-2.03739537	0.265537528	22.44151376	2.17E-06	9.02E-05
BCAS1	-0.290704248	0.265758563	1.252415916	0.263091552	0.498293218
LOC107054904	0.80411364	0.265991955	10.4216317	0.001245478	0.014031704
RBM44	0.452768272	0.267248181	3.388231499	0.065663343	0.214723829
LOC771689	-0.317200146	0.267777055	1.421327085	0.233185095	0.464816564
CCDC78	-0.413344622	0.267959143	3.817507316	0.05071964	0.181515455
CCK	0.371848155	0.268199181	2.478985379	0.11537666	0.305474328
R3HDML	-0.289457784	0.268294374	0.743130048	0.388660053	0.622605255

KLHL33	-0.234127831	0.269297243	0.687912838	0.40687521	0.637801433
SHC3	-0.199142553	0.269717957	0.683771174	0.408291151	0.638773924
LOC107054396	0.593011597	0.269916343	5.259769288	0.021823912	0.105137513
LOC107052289	0.248651127	0.269988435	0.546299273	0.459833764	0.681918222
LOC107052250	-0.128364133	0.270270806	0.234180581	0.628441168	0.800445901
FAM19A3	-0.206587689	0.270674057	0.778077958	0.377729614	0.612609974
RPRML	-0.000439378	0.271541879	4.55E-05	0.994619573	0.997544036
LOC107053147	-1.072654768	0.272261058	8.390789649	0.00377127	0.031184047
GADL1	-0.014490183	0.272298548	0.003621882	0.952010626	0.979652938
LOC112532269	-0.936013591	0.272524305	16.00595822	6.31E-05	0.001449129
ANKRD66	-0.135583443	0.27299897	0.410630121	0.521649854	0.72659097
RBP4A	0.017573189	0.273009525	0.004922857	0.944063862	0.975973541
MIR1774	0.19226258	0.273978043	0.610376608	0.434646046	0.661129662
HIST1H4B	0.8187036	0.274283348	9.784270552	0.00176011	0.018272944
LOC107052568	-1.280180064	0.274852794	5.451374286	0.01955294	0.097853899
LOC101751355	-0.243731796	0.275272861	0.793713657	0.372979554	0.607528899
GPR61	0.032374454	0.275351704	0.013851341	0.906311928	0.957150554
WDR27	0.023618157	0.275522201	0.006659026	0.934962529	0.971447144
LOC107051956	-0.620661228	0.275581049	7.615336646	0.005787395	0.04225004
HOGA1	-0.283747501	0.276442929	0.875383609	0.349469197	0.586129409
LOC107051837	-0.545678841	0.278085278	5.269682939	0.021699965	0.104883409
LOC107055199	0.616246718	0.27813753	5.268263106	0.021717672	0.104921986
LOC112532107	0.05600433	0.278302622	0.04717806	0.828048501	0.918504892
LOC112530274	0.280123937	0.279464115	1.435269661	0.230906349	0.462346552
LOC772017	-0.110609508	0.279679932	0.273592199	0.60093221	0.782591357
LOC427400	-0.096972575	0.280236714	0.122517171	0.726320289	0.859117428
LOC107053293	-0.102476447	0.280446217	0.164257548	0.685266565	0.835563601
ATP6V0A4	-0.715053476	0.281533586	6.882165722	0.008706001	0.05631181
LOC112532331	0.463261797	0.281834084	3.30676303	0.068995272	0.221280185
GFAP	0.056836611	0.283403382	0.046626966	0.829040144	0.918867551
LOC107055074	-1.538836943	0.284064618	20.63381279	5.56E-06	0.000202879
HEATR7B2L2	2.333836781	0.284247697	42.36640381	7.57E-11	1.17E-08

HIST1H3H	-0.301342052	0.285383214	1.330870918	0.24865034	0.482857453
TRH	-0.202036914	0.285485809	0.92511988	0.33613424	0.573308899
C8H1ORF111	-0.536039895	0.285727169	3.730726365	0.053420158	0.187466134
SPTBN5	-0.395142978	0.286622319	2.437560899	0.118460294	0.310689216
GIPR	-0.011168119	0.28679419	0.002216766	0.962447442	0.983899168
PPP1R36	-0.404144572	0.287238218	2.517242294	0.112607357	0.30056027
ADAMTS4	0.666282598	0.287348132	5.559969941	0.01837573	0.093729893
LOC101749273	0.222779031	0.287749234	0.888506201	0.345882434	0.582540186
ADRA1D	-0.680764494	0.288477928	7.709074353	0.005494392	0.040725323
ABCA9	-0.197266021	0.288738524	0.287909996	0.591562977	0.776931023
TSHB	-0.050328548	0.289227133	0.051145151	0.821082472	0.915994039
MARCO	0.108635019	0.290152887	0.149223179	0.699278824	0.843658952
CNTF	0.177391989	0.290303764	0.435372314	0.509364541	0.717892027
ANKRD2	0.090294462	0.290424848	0.127158437	0.721396656	0.856216242
LOC101751589	-0.350044146	0.291692587	2.053818897	0.151824946	0.363597655
LOC112532313	-0.278775549	0.291737175	0.853388264	0.355595578	0.592237747
TNFSF13B	-0.876678651	0.291791779	8.265384226	0.004040811	0.032673977
LOC112532929	-0.587084222	0.292311353	5.646240589	0.017492835	0.091176625
TRIM50	-0.152050547	0.292591302	0.394161808	0.530119851	0.733281956
WFDC2L	0.071524966	0.292829005	0.07099046	0.789900093	0.896648248
LOC107054951	-0.334616874	0.293209962	2.572299992	0.108749514	0.294272859
TM4SF19	0.02886215	0.294581445	0.01191078	0.913094211	0.960114864
SFTPC	0.330122702	0.295219339	2.081308663	0.149112288	0.360088451
LOC101749832	-0.257762889	0.295667945	1.323169772	0.250023967	0.484356521
LOC107053559	0.834991593	0.296740135	11.71814757	0.000618935	0.008487151
TRAT1	-0.072227971	0.297508947	0.119013084	0.730107354	0.861583227
GPR160	0.275010376	0.300262194	1.180383867	0.277277862	0.512364651
KL	-0.236935891	0.301312886	0.750103745	0.386443386	0.620928222
LOC112533455	-0.274525652	0.301488803	0.639172238	0.424010701	0.65309226
BATF	-0.68664928	0.301514749	9.018751266	0.002672239	0.024320626
LOC112530975	0.143885329	0.301576209	0.190834801	0.662222726	0.820794617
LOC107051601	-0.697566778	0.302634841	8.969256862	0.002745602	0.024796317

ACTL7A	0.359376118	0.302810547	2.417912888	0.119954655	0.312912785
MCTP1	0.004974651	0.303190929	0.000455849	0.982965971	0.992434965
SLC26A4	0.479665695	0.303731583	4.754899403	0.029215	0.127110744
CFAP126	-0.24991079	0.304778398	0.965496665	0.325805871	0.563899329
LOC107053392	-0.263609197	0.305844794	0.901936674	0.342262948	0.579357762
LOC107051579	-0.956314751	0.305930377	6.557218437	0.010445952	0.063946484
LOC107053358	-0.345871503	0.30699794	1.766014409	0.183876097	0.406459052
MORN3	0.127112211	0.307738423	0.343646265	0.557732052	0.753611506
LOC107054715	0.309488327	0.308448187	0.868943518	0.351247954	0.587734907
LOC107053016	-0.180423369	0.308482147	0.540843453	0.462083382	0.683437728
LOC112532896	-0.000303478	0.308514851	3.01E-06	0.998615828	0.999310367
LOC112532682	-0.615640814	0.308856853	6.471709208	0.010960507	0.066258319
LOC107049726	2.275503045	0.309016144	39.17611568	3.87E-10	4.79E-08
LOC101748436	0.013970761	0.309060982	0.002855611	0.957382993	0.981573571
LOC107051612	-0.276050665	0.310690506	1.276892278	0.258477664	0.492901839
RXFP3	0.226210154	0.311072142	0.81176493	0.367598963	0.602414195
FMO3	-1.022007633	0.312383773	14.07137402	0.000176002	0.003238128
HIST1H111R	-0.248788862	0.312703043	0.534168773	0.464859474	0.685549012
SYK	-0.426330865	0.312718971	2.83494975	0.092233686	0.265492444
LOC107053702	-0.087259948	0.312999134	0.107770695	0.742696584	0.869455911
CHRNA3	-0.020805448	0.31300842	0.005545155	0.940639757	0.974411302
LOC107052630	0.29452158	0.313020099	1.622026789	0.202809412	0.429501786
TMEM158	-0.389857726	0.314131759	2.436986961	0.118503653	0.310692768
SRD5A2	-0.058976667	0.315016614	0.046001852	0.830172432	0.919279096
LOC107053051	-0.395546863	0.316139806	2.539155633	0.111054161	0.298496605
LOC769512	-0.026303001	0.316474861	0.013477111	0.907580452	0.957538022
LOC112533596	-0.442208444	0.31832193	3.328439793	0.068091438	0.219439538
LOC769729	-0.490424423	0.31879115	2.349635288	0.125312322	0.321687092
LOC107050589	-0.221011129	0.31955662	0.660609607	0.416344811	0.646499375
LOC107054551	0.43063073	0.321339127	3.108613552	0.077879229	0.238215389
LOC107053522	0.28998509	0.32203174	1.548553146	0.213349163	0.441814809
LOC112530330	-0.228541671	0.323449542	0.789623001	0.374214146	0.608868315

LOC112529941	0.431718432	0.323564623	1.452958058	0.2280539	0.458364104
LOC112531323	-0.846497648	0.32363178	8.750598429	0.003095005	0.027019761
PSTPIP1	-0.163670751	0.325324158	0.492391635	0.482862386	0.697708164
LOC101751623	-0.277946849	0.325441413	1.064146693	0.302271322	0.54010714
AKR1B10L2	-0.079508375	0.325999475	0.066120351	0.797071643	0.900997038
TDRD5	-1.21149879	0.326880773	16.39513621	5.14E-05	0.001219116
TRIM7	-0.58448958	0.327137655	7.121612929	0.007616016	0.051382345
LOC107055274	0.128707367	0.327261978	0.23685647	0.626485814	0.799319537
LOC112532499	1.304469958	0.327580988	20.44867345	6.13E-06	0.000219524
ACMSD	0.564899372	0.327678316	6.571662532	0.010361512	0.063571467
PDE6H	0.537930735	0.327800349	2.675740283	0.101887938	0.281728453
LOC101749515	-1.557830293	0.33080682	41.69562095	1.07E-10	1.60E-08
LOC107051677	-0.014579341	0.331924152	0.001719557	0.966923164	0.986405497
LOC112532494	-0.711338604	0.332139493	7.924279604	0.004877576	0.037575148
LOC107054867	0.210546745	0.333166495	0.465441114	0.495091979	0.707120781
MIR1804	0.131196942	0.333352288	0.226742517	0.633949794	0.80403489
LOC422295	0.229006182	0.334497344	0.860008815	0.353736225	0.590430739
GSTA4L	-0.453073322	0.334663996	2.917972841	0.087597849	0.256476892
BSPRY	0.071002227	0.335667473	0.104570713	0.746412053	0.871758843
LGALS1	0.243925926	0.336489278	1.039390399	0.307963291	0.545966429
NTRK1	0.902860648	0.336924744	11.6962762	0.000626253	0.008542773
EDKM	0.416247248	0.337164929	2.253498468	0.133312708	0.335198058
LOC421892	-0.917703268	0.33729887	10.28418807	0.00134175	0.014837704
LAT2	-0.028839517	0.338217803	0.015194409	0.901896779	0.955177611
URAH	-0.191147432	0.339684533	0.78283969	0.376274066	0.610791137
HS3ST5	-0.077161827	0.3404335	0.072524815	0.78769551	0.895442461
REEP6	0.179494733	0.340626692	0.597893255	0.439382976	0.664587449
LOC107051395	1.144740374	0.341034158	14.16702419	0.000167277	0.003096217
LOC107052495	0.416336313	0.341318965	2.029686089	0.154252465	0.36713461
PPEF1	-0.246100845	0.341779939	0.702031788	0.402101825	0.634366465
LOC107053572	-0.335119903	0.341972505	2.127841731	0.144644294	0.353434606
MUC2	-0.555637472	0.342356914	4.808358032	0.028322019	0.124748861

NEK10	-0.817960136	0.34297622	10.94201436	0.000940079	0.011486028
SLITRK1	-0.302075126	0.343654488	0.849604931	0.356664125	0.593238469
LOC107054150	0.233382881	0.344006942	0.679956796	0.409601602	0.640051404
LOC112530920	0.692350286	0.344201361	6.099560203	0.013521553	0.076802607
LOC107053206	-0.330713685	0.344229982	1.906125358	0.167394296	0.385402706
TRPM5	-0.031234312	0.345835614	0.019586686	0.888697567	0.949702459
LOC107055277	-0.272965538	0.346251129	1.253461936	0.262892295	0.498030476
CNGA4	-0.109106105	0.346907683	0.196780277	0.657332362	0.817261442
LOC112533567	0.773421395	0.34966502	8.056507446	0.00453405	0.035595323
LOC107055395	-0.228178877	0.349883403	0.684287024	0.4081144	0.638661194
MIR6615	0.023401584	0.350254683	0.009572683	0.922059275	0.964486558
LOC107053243	-0.072550518	0.35075869	0.094881776	0.758060375	0.878437202
LOC107054033	-0.141261412	0.351182259	0.332763287	0.564036477	0.758120015
LOC107051609	0.545336357	0.351733255	3.437985495	0.063713334	0.210691905
LOC101747901	0.586103723	0.35260117	2.586012834	0.107811426	0.292504041
VIL1	-0.280684911	0.353015382	1.02306505	0.311793072	0.54966306
LOC112533178	-0.068358119	0.353338102	0.079579129	0.777868574	0.889865642
LOC107054688	0.073866399	0.356332024	0.061296096	0.804459012	0.905491008
LOC107051636	0.378795842	0.356775938	1.376056855	0.240773952	0.473406068
LOC107053852	0.277547298	0.35756799	1.493077372	0.221739601	0.451179047
NPR1	-0.905533188	0.359572602	11.34949298	0.000754686	0.009785936
MCMDC2	0.048210142	0.360283454	0.041208795	0.839135427	0.92378471
LOC107053968	-0.098284018	0.360935239	0.144532408	0.703816123	0.846564677
LOC100858777	-0.429616877	0.361617501	3.279221636	0.070162197	0.223657293
LOC112532637	0.579024854	0.362357338	5.188063713	0.022742541	0.107909031
CHRNA2	0.508236517	0.362742306	2.522568619	0.112227645	0.299960737
ES1ML2	-0.115038336	0.363744368	0.189054113	0.66370504	0.821642072
ADAM28	-0.531607228	0.366072178	2.05638655	0.151569223	0.363261254
NOG	-0.145946778	0.367134577	0.322002393	0.570406758	0.762633868
HSBP1L1	-0.124299927	0.367286427	0.250403323	0.616791228	0.792666328
LOC112532055	-1.294623015	0.368785819	22.30640955	2.32E-06	9.66E-05
ACOD1	-0.380827338	0.368822907	1.574501479	0.209554492	0.437424733

PADIL	0.268710947	0.369778562	1.084542691	0.297683543	0.534699564
LOC112529975	0.274813183	0.370646514	0.926832293	0.335687407	0.572942255
LOC107053674	0.113032938	0.370658388	0.251856212	0.615771083	0.792054659
LOC107051698	-0.213378923	0.371653266	0.6357211	0.425264509	0.653696874
LOC107049418	0.06802681	0.371743339	0.082607513	0.773794402	0.888044838
LMOD3	0.159616146	0.372584363	0.376033022	0.53973398	0.740769442
CSMD3	-0.231849506	0.372903533	0.804099259	0.369870577	0.604380226
COL4A4	0.099722759	0.373086089	0.096031789	0.756644636	0.877296187
LOC101749151	-0.215102977	0.373269943	0.713922372	0.398144747	0.630434162
CALB2	-0.186793082	0.3742408	0.423936337	0.514979395	0.721804595
LOC107053675	-0.95286549	0.374295744	4.203695036	0.040335998	0.15741623
LOC112530970	-0.168937206	0.375001664	0.518083381	0.471660404	0.690311478
MORN2	-0.512122812	0.376745242	5.890077206	0.015226433	0.082854151
LOC112533489	-0.347576155	0.377676426	2.189680534	0.13893813	0.344313238
LOC107049250	0.757884929	0.377793442	9.059211175	0.002613747	0.023987289
MAP3K15	-0.015272134	0.377826819	0.00150028	0.969102906	0.987351035
VSX2	0.287064003	0.377923863	1.348675192	0.245509855	0.479111085
TMEM45BL	0.199864908	0.378780032	0.766606451	0.381268926	0.615861409
FAM83G	-0.150036944	0.379114381	0.483758472	0.486724797	0.70113425
LOC112529984	0.392266746	0.37916691	1.823621544	0.176882799	0.397135551
S100B	-0.407073031	0.37944845	3.790773241	0.051535836	0.183118587
CMBL	-0.213244908	0.37962556	0.880933669	0.347946082	0.584408777
LOC112531451	0.155783891	0.379943135	0.216207011	0.641945309	0.808697753
WDFY4	-0.113804201	0.381299149	0.265943094	0.606066254	0.786253032
WFIKKN2	-0.296564568	0.381349412	1.65281811	0.198575805	0.424224105
LOC107050595	0.035915619	0.381393046	0.017072685	0.896042311	0.952540618
LOC101749311	-0.378790077	0.38238348	2.1588155	0.141753927	0.348924587
GIPC3	0.083024401	0.382750188	0.138105406	0.710171807	0.84968127
ODF1	0.088364393	0.383075894	0.060441615	0.805799307	0.906069946
PCTPL	-0.447250121	0.383764289	2.166535844	0.141043642	0.347956991
CD3D	0.113735045	0.38445026	0.300076464	0.583834489	0.771063923
TCTA	0.431475344	0.384865907	2.756574759	0.096855407	0.272657543

LOC101750546	-1.612714013	0.385402432	33.13170969	8.61E-09	7.58E-07
LOC107054703	0.030897594	0.386707039	0.016934031	0.896462926	0.95275104
LOC107054465	-0.839076503	0.387549969	4.943286174	0.026192194	0.118568272
KCNIP1	-0.006620008	0.38876296	0.000686276	0.979100316	0.99102514
LOC112532591	-0.471636548	0.390117078	4.185227668	0.040777735	0.158441
KRT222	-0.252945243	0.392515213	1.244819405	0.264544254	0.499779071
LOC112531205	0.239101373	0.393194121	0.976311069	0.323111244	0.560665983
LOC107053820	0.119698918	0.393394644	0.164809577	0.684766511	0.835520758
CA6	0.354168581	0.394881365	1.852873831	0.17344952	0.392857792
NEU2	-0.082796259	0.395543008	0.111261556	0.738712419	0.867012329
LOC101750451	0.086061587	0.395910816	0.072483049	0.787755188	0.89544844
CCKBR	0.051434724	0.397007911	0.041530958	0.838516469	0.923661189
RFESD	0.580662428	0.397576402	2.917074259	0.08764665	0.256532483
NR5A1	0.180242438	0.397968102	0.209327957	0.647294655	0.811708089
LOC112530974	-0.179126972	0.398297879	0.421651115	0.516114318	0.722414672
LOC101748003	0.907535889	0.398609403	12.78459413	0.000349486	0.005513696
MYO15L	-0.646109163	0.399138616	5.438846031	0.019693676	0.098259383
FRMPD2	0.330074783	0.399154148	1.547118273	0.213561367	0.442142965
LOC101750415	-0.213489758	0.399225966	0.898919308	0.34307165	0.580189517
LOC107053372	-0.071919048	0.399743557	0.060987044	0.804942624	0.90566365
SERPINB2	-0.348019394	0.399833291	1.533035593	0.215657427	0.444540058
PRKACA	0.525298181	0.400931298	4.743910012	0.029402174	0.127722486
LOC107054724	-0.461071393	0.401273402	2.597571231	0.10702762	0.291188417
LOC112531978	-0.055366909	0.401773586	0.063048186	0.801741451	0.903384062
LOC101747822	0.065206121	0.403619298	0.069438804	0.792155639	0.897745396
LOC112533583	1.399178387	0.404027999	13.44024269	0.000246284	0.004243559
LOC107055176	-0.200559776	0.40484453	0.878650456	0.348571575	0.585160709
LAMB3	-0.341242189	0.405039239	1.930354089	0.164719711	0.381697263
LOC112530399	-0.548636493	0.405129828	4.259919159	0.039021778	0.153686779
LOC107054686	-0.868149853	0.405165125	5.820541483	0.015840068	0.085430454
LOC101748787	0.03618737	0.406235287	0.023407268	0.878402739	0.945331398
ENPP7	-0.602754738	0.407268153	2.767480472	0.09619747	0.271821836

XPNPEP2	-0.228309657	0.40848041	0.858035202	0.354289111	0.591080536
C20H20ORF85	0.074281654	0.408744317	0.049238942	0.824392902	0.917226694
LOC112532661	-1.453946471	0.410595125	37.6132855	8.63E-10	9.79E-08
NPY5R	-0.152723847	0.410897826	0.468478169	0.493688116	0.705973577
LOC107051618	-0.280422456	0.411338412	1.226642733	0.268060941	0.503183194
JAML	-0.202890191	0.411548013	0.41222647	0.520841595	0.725945349
CNKSR2L	-0.534574173	0.411588479	2.702795215	0.100172482	0.278388481
LOC101749477	-0.536287482	0.412643083	5.00663239	0.02525038	0.115801285
LOC107052248	-0.556119725	0.412754044	6.016914525	0.014169398	0.07911688
LOC107054276	-0.278125378	0.413028077	1.156585486	0.282174808	0.517783055
C7	0.076881744	0.414094433	0.046173951	0.829859905	0.918994867
ENTPD2L	0.111927878	0.414717105	0.128036232	0.720476894	0.855792151
WDR63	0.281365707	0.415415944	1.489915141	0.222229627	0.451845855
LOC112533476	-0.516044522	0.416041396	3.327794962	0.068118142	0.219439538
SPIK4	0.219006722	0.417527557	0.667985873	0.413754706	0.643996754
COR8	-1.118477226	0.418769772	11.5562288	0.000675227	0.009047928
LOC107053373	-0.001770555	0.419232843	4.26E-05	0.9947947	0.997594138
LOC107053791	-0.34599832	0.421472062	2.74736879	0.097414618	0.273636511
LOC107052960	0.277105813	0.421833584	1.293267087	0.25544674	0.490361188
LOC101748951	0.320536468	0.422978217	2.283699812	0.130739662	0.330972483
SERPINF2	0.432342381	0.423078119	1.128776532	0.288036457	0.524818946
ITIH5	-0.346908022	0.423475525	1.926603449	0.165130524	0.382330488
LOC107054090	0.375886297	0.423562438	2.211661534	0.136971078	0.341183634
LOC107055067	0.058381438	0.424415766	0.058857206	0.808311376	0.907716078
LOC112532500	-0.750121813	0.427053563	7.927661204	0.004868469	0.037540136
LOC112530337	0.1849882	0.427293436	0.689886899	0.406202853	0.63730625
RHO	-0.237013734	0.428333854	0.985417381	0.320864992	0.558298979
LOC112531015	1.083860662	0.428463735	6.970789656	0.008285093	0.054576529
DLK1	0.192498232	0.42850667	0.301863576	0.582716478	0.770808073
LOC107054464	-1.120835269	0.429120716	9.195049663	0.002426705	0.022805148
PMCH	0.197852632	0.429651636	0.785295483	0.37552647	0.610073664
DOK3	-0.530457999	0.429891123	6.041390968	0.01397427	0.078480071

PTAFR	-0.057874529	0.430534276	0.063154309	0.801578147	0.903298754
COL4A3	-0.226305754	0.43201674	0.556793665	0.455555016	0.67813984
LOC112532764	0.623147939	0.432161296	5.524348332	0.018753587	0.095038582
TAC3	0.057726531	0.433076268	0.063172401	0.80155032	0.903298754
LOC107054152	-0.274972512	0.433329406	1.358875237	0.243732508	0.476716798
UBXN11	-1.025979272	0.434383034	18.56502715	1.64E-05	0.000484278
LOC772080	-0.316550992	0.4373994	1.222820691	0.2688078	0.503785005
LOC107054471	-0.051266513	0.439162059	0.019506055	0.88892541	0.949773712
LOC107051605	-0.590535629	0.440066385	8.581342892	0.003396248	0.028839264
HRH2	0.30134097	0.440451456	1.740298548	0.187101003	0.410102577
KIAA1211L	-0.474528804	0.440755049	4.096016288	0.042984381	0.163356737
ROM1	-0.192540404	0.440979744	0.731292659	0.392464508	0.625849305
PLPP7	-0.033050851	0.44316889	0.015632812	0.900498817	0.95453991
SLC35D2	-0.032569533	0.443371785	0.020458796	0.886263133	0.948791653
LOC100858531	0.832078446	0.44556671	4.880128565	0.027167596	0.121512037
RAB25	-0.248858477	0.446029043	0.749675387	0.386579024	0.620979506
PABPN1L	0.033639005	0.44662724	0.017084598	0.896006252	0.952540618
LOC107054756	0.9626281	0.448520376	16.49028052	4.89E-05	0.001172325
LOC107053924	-0.110402251	0.449372329	0.174182445	0.676421175	0.829953802
LOC107052622	-0.347464377	0.450637414	2.392336074	0.1219313	0.316313973
USP35	0.116185607	0.45076732	0.269261958	0.603827287	0.784894384
LOC112531075	1.3562788	0.450891387	17.1771999	3.40E-05	0.000867323
LOC101749811	-0.692875149	0.451027523	6.7917859	0.009157825	0.058272238
LOC107054301	0.156870118	0.451611696	0.34397419	0.557544178	0.753481393
LOC107052924	-0.33305619	0.453799799	1.334672225	0.247975722	0.482093632
LOC107054318	-0.457283148	0.454368178	4.452416726	0.034851892	0.142552294
TMEM269	0.423587836	0.455193847	3.419648045	0.064424747	0.212106685
RGS18	-0.081357655	0.456773308	0.097037526	0.755414106	0.876495029
LOC107052908	-0.048993559	0.456995615	0.03622339	0.849054909	0.929677837
LOC107056301	0.530758461	0.457335302	1.609123982	0.204614987	0.431327134
AMY1AP	-0.117466407	0.457819147	0.147869671	0.700579547	0.844482927
LOC112532323	-0.095882214	0.459781107	0.107629417	0.742859322	0.869560491

IFITM5	-0.409964737	0.460120091	2.407084545	0.120787132	0.314336907
LOC769646	-0.446322987	0.460553313	3.213808007	0.073019357	0.228860693
CHRNB3	-0.280800451	0.461088158	0.403223585	0.525429141	0.729995352
LOC112531076	-0.546135516	0.461202605	1.966629202	0.160806108	0.376182046
LOC768391	-0.442007601	0.462088742	2.182190486	0.139615626	0.34532758
LOC112530195	-0.757703053	0.462869617	9.094821002	0.002563343	0.023669926
NR0B1	-0.17177879	0.464708781	0.519698485	0.470970328	0.689669552
LOC112533509	-0.204961922	0.466440751	0.811834116	0.367578549	0.602414195
EPHA8	0.104442902	0.466488913	0.183879096	0.668060584	0.824166458
LOC112530248	0.797899017	0.467424534	5.286643885	0.021489596	0.104095171
LOC107053493	-0.271548157	0.467804209	1.192078851	0.274910628	0.509811425
SRCRB4D	0.37106888	0.468212847	1.932978854	0.164432911	0.381520524
LOC107052732	0.714469019	0.468549445	9.183618277	0.002441908	0.022880678
LOC107052688	0.048695053	0.469461358	0.048541704	0.825620528	0.917634974
CSF3R	-0.02019907	0.470035302	0.007320757	0.931815068	0.970075727
C1QL3	-0.016318341	0.470505338	0.004574063	0.946078726	0.977126103
LOC112530646	-0.33192734	0.470730943	2.071342172	0.150089385	0.361281572
LOC112532189	-0.063523761	0.471464291	0.053545979	0.817004034	0.913581031
IL2RG	-0.04407988	0.471569508	0.042587178	0.83650455	0.922733824
LRRC55	-0.787552679	0.471622401	9.21680891	0.002398032	0.022664911
LOC112532178	-0.117066913	0.472029709	0.198292255	0.656102834	0.816485578
CFAP206	-0.416967526	0.472326213	4.76901925	0.028976328	0.126540732
OASL	-0.126254608	0.473015398	0.364032847	0.546274982	0.746341738
KCTD8	-0.441433487	0.473227833	3.305097028	0.069065267	0.221404434
LRRC23	0.149534538	0.473425504	0.459414071	0.497897935	0.709527196
PADI2	0.246649761	0.474025844	0.614978977	0.432919273	0.659478488
ARHGAP30	-0.073917279	0.474659308	0.112765536	0.737017316	0.865763844
LOC112533328	-0.411095037	0.474968621	1.103763698	0.293441611	0.53067169
MIOX	0.192278318	0.475220012	0.322453727	0.570136763	0.76248378
BAIAP2L2	0.138930452	0.475645189	0.330415991	0.56541424	0.759194353
MIR6599	-0.15601946	0.475771595	0.448229625	0.503176878	0.71381668
APOC3	0.61949663	0.475845955	6.135546105	0.013249062	0.075778003

LOC112530132	-0.664313546	0.476157826	9.143396588	0.002496173	0.023219064
KIAA0040	-0.176978739	0.479805919	0.374428158	0.540600381	0.741360053
MYO1H	-0.342988161	0.481092782	2.634264953	0.104580452	0.286901837
WDR78	-0.559437139	0.481208499	6.337432229	0.01182159	0.069779198
LOC107052935	0.013307003	0.481741588	0.003043506	0.956004637	0.981041173
SLA2	0.284085577	0.48400637	1.346877312	0.245824772	0.479463693
CDH19	0.438957265	0.484095283	2.84541576	0.091634887	0.264396295
LOC101750583	-0.129810101	0.484342295	0.26088374	0.609513663	0.78760321
LOC101749104	0.584592346	0.48447845	4.728377515	0.02966886	0.128271438
LOC101748943	-0.237832859	0.484861567	1.262985654	0.261086703	0.496033549
LOC107056920	0.081505542	0.4850649	0.126961538	0.721603459	0.856304305
CPNE9	0.030245646	0.485599426	0.017695705	0.894173448	0.951827123
CLCF1	-0.497347434	0.48599114	2.849776971	0.091386614	0.263911325
LOC107055713	-0.129913108	0.487801607	0.243910357	0.621396005	0.795958144
BIN2	-0.249234541	0.489914594	0.739083067	0.389954763	0.623829051
LOC100859492	-0.661945144	0.490056384	7.375141536	0.006613159	0.04653862
EXOC3L4	0.402789678	0.490230824	2.827533107	0.092660593	0.26606145
BMP8A	-0.634565323	0.490351414	6.690775927	0.009691302	0.060750616
LOC107054611	0.004696465	0.490620721	0.000542027	0.981425749	0.991828264
LOC101749094	0.225763845	0.490665676	0.647194627	0.421117463	0.650656136
LOC107055388	-0.072417635	0.490698613	0.095034181	0.757872217	0.878348171
LOC112533431	-0.134565771	0.491297032	0.398408862	0.527912133	0.731704359
PTPN7	-0.245833463	0.491685458	1.187513387	0.27583171	0.510915379
LOC112531132	0.145552101	0.492206683	0.281536988	0.595695586	0.779071694
LOC107053383	0.492045397	0.492600436	1.918912603	0.165976584	0.383692463
LOC101747522	0.072569477	0.49381663	0.091650121	0.762089757	0.880756463
PDYN	0.492769392	0.494822972	4.62310748	0.031544042	0.133747599
LOC101748601	0.43348854	0.497484647	3.926497936	0.047530828	0.17353488
LOC112532592	0.473948493	0.498819367	3.583813764	0.058345058	0.19889328
LOC107052497	-0.575722378	0.50038802	5.018645077	0.025075783	0.115289749
LOC112532542	-0.526466372	0.500408293	5.470018016	0.019345426	0.09717002
SLC6A14	-0.514753651	0.50055408	4.268004043	0.038836523	0.15303041

MHCIY		-0.233645563	0.500771476	0.638649253	0.424200346	0.65309226
ARR3		-0.258710316	0.501131469	1.0384073	0.308192181	0.546064643
CELA3B		0.301208654	0.501616675	0.763069275	0.382369704	0.616769322
LOC107052288		0.834208797	0.501681253	5.619261019	0.017764137	0.091921032
KCNE3		-0.438774114	0.501856605	2.605125685	0.106518712	0.290481507
LOC112530240		-0.159197754	0.502814357	0.410757937	0.521585057	0.72659097
OR11A1		2.186214838	0.504489358	12.06926843	0.000512598	0.007388296
EVA1CL		-0.455982362	0.504607528	4.210837197	0.040166508	0.156992849
	04/set	0.222294358	0.504789854	1.010525389	0.314777005	0.552319857
SPTLC3		0.241274717	0.506769029	0.833464388	0.361272674	0.597701775
TBX20		-0.845091684	0.507027274	2.18898704	0.139000703	0.344362628
HIST1H2B7		-0.276480993	0.507097471	1.244412552	0.264622339	0.499811822
PLD5		0.006537353	0.508262771	0.000547502	0.981332197	0.991828264
LOC107051689		-0.267662948	0.508680631	1.567728973	0.210537129	0.438234642
LOC112530292		-0.371335145	0.508688341	3.29873979	0.069333056	0.221830728
LOC107051074		0.00937354	0.509454999	0.001580749	0.968285558	0.98693873
LOC101749344		0.169811374	0.509544533	0.620462126	0.430875609	0.65831552
FAM19A1		-0.714223141	0.509986947	6.931230008	0.008470342	0.055309314
LOC107054991		-0.116631959	0.510576957	0.204023993	0.651492214	0.813994258
ALPK1		-0.082298558	0.511325096	0.151700388	0.696915674	0.842225868
C1QC		-1.595836517	0.511531095	16.61950675	4.57E-05	0.0011119
WDR88		0.42955964	0.51184439	1.890719705	0.169120768	0.387628701
LOC107051582		-0.115846773	0.512349906	0.293800969	0.587795013	0.774114284
ZAR1L		-0.126098924	0.513582146	0.359692728	0.548676936	0.747762049
SLC4A5		0.827663486	0.515506863	6.738380958	0.009436022	0.059558783
LOC112532065		-0.346748091	0.515777933	2.413557893	0.120288699	0.313386722
LOC107052948		0.136232007	0.516675058	0.315678989	0.57421606	0.765040788
GRAP		-0.222429041	0.517196668	0.826437187	0.363304781	0.599136116
LOC107049408		-2.963846945	0.518401542	34.44422976	4.39E-09	4.17E-07
GBX1		0.342134978	0.518749885	2.5691132	0.108968803	0.294672416
LOC101749017		0.216615731	0.51960579	0.947581459	0.330335681	0.568050332
LOC112529918		-0.105074781	0.519890875	0.137110835	0.711170296	0.849781439

LOC107052005	0.392337242	0.520220372	1.325327397	0.249638183	0.483896372
LOC112532556	-0.208085685	0.520857798	0.654667013	0.418449026	0.6482375
TDRD6	1.023513317	0.521217155	11.85546526	0.000574924	0.008051378
LOC107054417	-0.098171504	0.521265683	0.133659254	0.714667766	0.852264423
LOC112532704	0.000553045	0.522167828	3.77E-06	0.99845149	0.999301702
VAMP2	-0.180745846	0.523284832	0.632727765	0.426356514	0.654467617
METTL10	0.163703393	0.524109024	0.63925321	0.42398135	0.65309226
RIPK3	0.234466913	0.524263226	0.96252849	0.326550648	0.564254007
LOC101750982	-0.024988107	0.524480479	0.005449024	0.9411556	0.974643014
FSHR	-0.981997463	0.524494548	8.879835092	0.002883376	0.025771652
SYT8	0.204548363	0.525140891	0.901634777	0.342343745	0.579434924
LOC112531892	-0.356583825	0.526207866	0.886318223	0.346476989	0.582893247
LOC107049937	-1.238658018	0.527200928	23.23002094	1.44E-06	6.42E-05
LOC107056538	-0.358549476	0.527551184	2.371207746	0.123591401	0.318761208
CLDN10	-0.402232836	0.527577022	2.215593162	0.136622542	0.340470079
LOC107053879	-0.313535558	0.52798696	1.347822604	0.245659133	0.479249328
LOC107053484	-0.10400099	0.529175761	0.150242867	0.698303374	0.843036098
LOC107051611	-0.104318487	0.52940957	0.202954521	0.652346537	0.814586931
LOC101749540	0.454598134	0.5299128	3.228774242	0.072354827	0.227375706
LOC417013	-0.173342232	0.531185709	0.438944347	0.507632415	0.717065103
MANSC4	0.0488858	0.532795269	0.024391556	0.875892748	0.943751684
LOC107052588	-1.032948254	0.532855356	11.78046984	0.000598554	0.008267676
LOC107049782	-0.976168151	0.533723638	10.42431342	0.001243671	0.014026459
LOC101747602	0.677246579	0.534682234	3.138310259	0.076472997	0.236046363
VSIG4	-0.224026257	0.535965049	0.820059595	0.365162742	0.600815211
LOC112531990	0.163259807	0.536595737	0.385658472	0.534590368	0.736378403
LOC107053374	-0.049437647	0.539512887	0.04936182	0.824177498	0.917146191
LOC107051217	-0.567676857	0.540031605	4.970129511	0.025788716	0.11735435
KCNK13	-0.321618632	0.54013736	1.97991471	0.159399392	0.374391592
PROX2	-1.056975612	0.540655108	18.94678046	1.34E-05	0.000411118
DAW1	0.787514538	0.54091575	14.5547568	0.000136145	0.002623253
CKMT1A	0.20131474	0.54096567	0.539881333	0.46248191	0.683627365

OTOP1	0.769995959	0.544052852	7.724802682	0.005446731	0.040481464
LOC101748357	0.440543636	0.544760677	3.359113049	0.066834056	0.216895178
LOC101748511	-1.61993948	0.546171271	28.33162488	1.02E-07	6.89E-06
F13A1	-0.822205151	0.546565613	8.922775938	0.002816361	0.025255162
LOC107052053	-0.12025671	0.546610362	0.350759802	0.55368334	0.75174518
LOC107054801	0.156829624	0.546629668	0.419345799	0.517263658	0.723219772
DDO	-0.242176176	0.547713692	0.941610445	0.33186402	0.56937808
LOC107054448	-0.340299387	0.548440083	1.468394512	0.225599174	0.455488885
ABI3BP	-0.00709444	0.549023387	0.000503511	0.982097736	0.992045319
LOC107054107	-0.569347893	0.550290277	3.546712013	0.059663567	0.201428804
LOC107053185	-0.254072116	0.550608136	0.775970402	0.378376374	0.613175422
ENDOUL	-0.152436026	0.552680603	0.335684862	0.562330655	0.756921375
LOC112530210	-0.884579924	0.552841453	9.045316089	0.002633687	0.024089671
LOC107052069	1.073769116	0.556050101	11.46501659	0.000709186	0.009365694
LOC107049033	0.516377259	0.55631186	4.68193305	0.03048143	0.130719816
LOC112530778	0.319107678	0.557061977	0.745930634	0.387767683	0.621841655
NXPH1	-0.561578897	0.558221831	3.172795985	0.074874166	0.232872277
ARMC3	-0.449012474	0.558240382	2.03011683	0.154208753	0.36713461
LY9	0.880577765	0.559410542	9.032107132	0.002652785	0.024183701
LOC101748060	-0.382502448	0.56034831	3.111727024	0.077730499	0.238185355
LIPML2	-0.111146448	0.561819963	0.182645094	0.669109874	0.824672532
GCGR	0.422093898	0.562531946	2.50218808	0.113688245	0.302561875
FAM26E	-0.05325226	0.562839187	0.049779061	0.823448172	0.916892656
LOC100858919	-0.265055478	0.563325306	1.650711795	0.19886208	0.424584861
LOC107052245	0.0883237	0.564578384	0.100854653	0.750806418	0.873913447
LOC101751351	0.888490196	0.565902633	6.219842518	0.012632635	0.073296899
HIST1H2BF	0.157879981	0.566603447	0.370192651	0.542899275	0.743026497
CSRP3	-0.308469156	0.566701562	1.653542612	0.198477448	0.424097198
LOC101748033	0.452919604	0.568047126	2.239839014	0.134494982	0.337365081
LOC419335	-0.747151385	0.568342536	6.59140651	0.010247218	0.063081921
SLC2A5	-0.194747407	0.569199115	0.649276732	0.420371385	0.649991651
MYOZ1	-0.179953163	0.56927998	0.469789434	0.493084054	0.705538967

ERNI	-3.376783937	0.570660426	40.12750898	2.38E-10	3.21E-08
RSPH1	-0.326622969	0.570736541	1.42679241	0.232288633	0.463704852
LOC420107	-0.088315802	0.571443239	0.172344426	0.678036592	0.830532564
HSPB2	-0.147683108	0.571633116	0.232432978	0.629725651	0.801165857
LOC107049643	0.89470009	0.572533667	11.70872694	0.000622077	0.008508953
MIR454	0.125375261	0.57314989	0.247264908	0.619007559	0.794312827
CRYBA2	-0.851386718	0.573796191	9.460513914	0.002099422	0.020835945
LOC107054789	-0.160108058	0.573865122	0.543895211	0.460822893	0.682585354
LOC107054048	-0.075157722	0.574622699	0.065125173	0.798571457	0.901588892
AADAC	0.128142907	0.575255303	0.261299636	0.60922869	0.787498672
LOC107053813	-0.210018822	0.575331769	0.871417768	0.350563109	0.587126307
LOC101750889	-0.110856015	0.576982567	0.281454347	0.595749567	0.779071694
PPP1R14D	0.230671875	0.57724729	0.729388257	0.39308155	0.62615185
SASH3	-0.084181237	0.579261118	0.08072896	0.776311967	0.889259549
LOC107052522	-0.192808146	0.579352012	0.735160199	0.391215664	0.624653104
TNFRSF6B	0.282419054	0.580174275	0.931878861	0.33437518	0.57206733
LOC112530512	-0.295871934	0.581020632	1.320874597	0.250435148	0.484707763
LOC112532430	-0.740608859	0.581023717	7.177325646	0.007383068	0.050139654
MALL	-0.236505955	0.58199725	1.07459153	0.299910626	0.537233764
WISP2	0.276586069	0.582092609	1.36276021	0.243059685	0.476013219
LOC107053579	0.646276522	0.583610505	5.803260416	0.015996485	0.086104732
MARVELD3	-0.173354743	0.583660571	0.422840034	0.515523314	0.722197865
LOC112533379	0.264606994	0.583883465	1.289411697	0.256156396	0.490864503
SCRG1	-0.292513126	0.583927415	1.632198473	0.201399238	0.428057926
TSPAN1	0.36585946	0.584950567	1.361446974	0.243286866	0.476215528
MMR1L4	-0.036012918	0.585059276	0.018590218	0.8915478	0.951008202
LOC112532368	0.72341391	0.585883785	4.780096756	0.028790503	0.126061116
LOC101750102	-0.478783372	0.587617798	4.157298382	0.041455476	0.159753578
GRIK2	-0.119484986	0.588473179	0.232725395	0.629510312	0.801004653
LOC112531743	0.121345978	0.588666897	0.311369709	0.576840891	0.766659514
SPDYA	-0.099565963	0.589123133	0.220974259	0.638298583	0.806449876
SAMSN1	-0.372240254	0.589337414	2.154657834	0.142138104	0.349381997

MIR7468	9.13E-05	0.590493032	8.37E-08	0.999769211	0.999872573
LOC100858759	0.600245566	0.592451413	7.068613513	0.007844594	0.052472679
ADAD1	-0.595870388	0.5933153	4.779675124	0.028797553	0.126061116
CD80	-0.177591303	0.593754369	0.580273061	0.44620531	0.670591686
FGF18	-0.11527184	0.593802825	0.220462265	0.638687921	0.806694024
MIR6573	-0.312135899	0.594110013	1.469127803	0.225483357	0.455366795
P2RY12	-0.281102734	0.594174946	0.93911557	0.332505398	0.570137415
LOC107054906	0.323835772	0.594452074	2.109529403	0.146384328	0.355588148
LOC101750197	0.610051033	0.595030643	8.113753712	0.004393066	0.034753802
HNF1A	-0.13866774	0.595786657	0.309280536	0.578122013	0.767734463
PDZK1IP1	-0.214309992	0.596199987	0.413346614	0.520275765	0.725779864
CH25H	-0.067667148	0.596269545	0.07621349	0.782495908	0.892887345
LOC107051247	-0.993085141	0.596675258	14.25282387	0.000159821	0.002981691
CTXN2	0.284313576	0.596969039	2.112274871	0.146121957	0.355317985
GPR39	-0.489139816	0.597023851	3.684321184	0.054926325	0.190718017
LOC107053790	-0.220235619	0.598076527	0.996397612	0.318183753	0.555985309
SYT10	0.273759949	0.599627703	1.481621468	0.223521001	0.452904577
CD200R1	-0.418269945	0.599933802	2.161443573	0.141511689	0.348484712
LOC107051621	-0.064302837	0.600854141	0.078074893	0.779923407	0.891348776
ARHGAP36	-0.631273444	0.600942869	5.505902708	0.018952395	0.095721811
IL13RA2	-0.173415107	0.601473187	0.382799362	0.53610886	0.737721299
LOC112533186	0.28205535	0.6015735	1.326389332	0.249448576	0.483642786
LOC107053945	-0.214870598	0.602767026	0.75068585	0.386259173	0.620692842
LOC112530194	-0.380322451	0.603775826	3.10843142	0.077887938	0.238215389
C4ORF19	0.204523074	0.60419543	0.624369947	0.429428007	0.656835643
LOC107054419	-0.318997589	0.604456643	2.037236253	0.153488297	0.366150603
MIR6649	0.155103686	0.606287863	0.581566714	0.445698873	0.670320351
SLC23A3	-0.105831281	0.607631947	0.092158978	0.761450198	0.88065738
LOC419250	0.108122013	0.607732463	0.159671224	0.689459393	0.83825922
SOUL	-0.647655993	0.60918637	7.880960432	0.004995794	0.038252947
GNG13	0.055867357	0.609213223	0.040828475	0.839869371	0.924170824
ZP3	-0.284708974	0.609288747	1.853607748	0.173364374	0.392718995

LOC107054619	-0.592642047	0.609300654	9.667473031	0.001875591	0.019199521
WISP1	-1.172340225	0.609577745	15.16146397	9.87E-05	0.00204028
LOC112530347	-0.140711655	0.61101714	0.549651867	0.458459976	0.68090502
LOC101751877	1.548716981	0.611329686	44.67588019	2.33E-11	4.14E-09
LOC112533087	0.105392357	0.612723336	0.285584958	0.593063785	0.777327412
ANKRD22	-0.49725672	0.612969964	4.209524397	0.040197606	0.157042407
LOC112532956	-0.077800257	0.614043677	0.081123617	0.775780455	0.889083953
LOC100859615	-0.097758708	0.614380433	0.144538579	0.703810099	0.846564677
CPXM2	-0.926652592	0.614402467	8.639964616	0.003288678	0.028243841
LOC112532626	-1.157516749	0.615303936	6.6547074	0.009889421	0.061640311
LOC101750283	-0.354647544	0.616283747	2.797909397	0.094387305	0.269024818
SCN4A	0.478800906	0.617107173	4.035495516	0.04455259	0.16669233
LOC101751380	0.156112128	0.618404361	0.220710074	0.638499409	0.806602965
LOC425756	0.396492856	0.619727358	1.232489882	0.266923361	0.502370343
LOC112530161	0.404108386	0.619756245	4.332974114	0.037380876	0.14915187
ABCA4	-0.256411786	0.621297919	0.990555576	0.319606625	0.557135546
STAC	-0.360080139	0.621515192	3.24084054	0.071823781	0.226496804
GOLGA7B	-0.609322938	0.621604415	5.147074072	0.023285578	0.109757716
LOC107049204	-0.427654089	0.622199346	4.950991677	0.026075707	0.118300458
RAB37	-0.448884011	0.622641532	4.365149547	0.036681263	0.147493168
CLCNKB	-0.355100711	0.622861037	1.498602285	0.220886549	0.450227692
LOC112530582	0.749496644	0.623155719	5.695161791	0.017011744	0.089691846
LOC107053991	-0.321664303	0.623775576	1.978323847	0.159567097	0.374636459
LOC107054461	-0.087046388	0.624398624	0.104855183	0.746079234	0.871493846
GIP	-0.252657105	0.625105386	1.352887454	0.244773962	0.478242168
SERPINC1	-0.06379386	0.625727083	0.084215046	0.771664546	0.886527969
PHLDA3	-0.273886974	0.625738613	1.676379909	0.195406173	0.420319684
GPR52	0.050514844	0.626829835	0.034768735	0.852081093	0.931269647
LOC112530035	-0.193599449	0.627011576	0.657805611	0.417335716	0.647366063
LOC112530935	0.24799241	0.628485366	0.438635456	0.507781799	0.717091444
UPB1	0.157946642	0.629047225	0.46097246	0.497169843	0.708810381
LOC107053480	0.193776608	0.629382453	0.506607722	0.476611046	0.693600685

LOC107052612	-0.031699616	0.62952533	0.01899935	0.890368348	0.950676503
C3H1ORF100	-0.318557359	0.629537318	2.562182278	0.109447411	0.295481073
LOC112530249	-0.029757505	0.629724205	0.018431185	0.892009826	0.951008202
SUSD4	-0.065782274	0.63002726	0.093349889	0.759960895	0.879827008
LOC107054444	-0.218829621	0.630336375	0.95729582	0.327869136	0.565581993
BRINP3	-0.598078288	0.632069323	5.723460989	0.016739702	0.088773577
CYBB	0.038054382	0.634053751	0.035448176	0.850659625	0.930518733
VGLL3	0.20162532	0.634906771	1.011227792	0.314608878	0.552201502
LOC107052946	0.682340335	0.635726572	10.65933093	0.00109517	0.012821423
FAM179A	0.024231644	0.637376476	0.010665379	0.917746049	0.96231374
DIRAS2	-1.126576602	0.641512462	10.13803963	0.001452405	0.015785556
SLC16A5	0.06934196	0.642377451	0.080489351	0.776635349	0.889376916
TEKT2	0.018319682	0.642558952	0.008021639	0.928634009	0.968392732
ARSD	0.311576637	0.642903463	0.601675828	0.437939335	0.663807274
EDN2	0.08914554	0.643341194	0.094591659	0.75841901	0.878579652
LOC107054798	-0.128610496	0.64395689	0.408004977	0.522983841	0.727754681
LOC101750071	0.434185473	0.644074885	2.715525619	0.099376232	0.276970849
LOC100859819	-0.931006863	0.645459194	15.50775881	8.22E-05	0.001783724
LOC107052889	0.570811996	0.645722984	7.020406201	0.008058595	0.053404823
FAM69C	0.381215474	0.645821328	3.661180842	0.055694147	0.192490484
KRT15	-0.994026574	0.646293795	6.948805099	0.008387524	0.055043695
ABLIM2	-0.037663844	0.647173165	0.027744806	0.867710194	0.938915609
ITIH2	-0.271425115	0.647330871	1.135940752	0.286511704	0.522903685
LOC771917	0.712345807	0.648275314	10.53515853	0.001171248	0.013430587
LOC112533280	-1.009852757	0.649499351	6.560639874	0.010425887	0.063847403
LOC101751669	0.221555452	0.649758016	0.580510648	0.446112233	0.670528882
LOC107056757	2.158445978	0.650339846	53.72986177	2.30E-13	7.42E-11
MLC1	0.093421143	0.651010339	0.142334341	0.705971302	0.847788218
SLC6A12	-0.202570263	0.651379958	0.918992258	0.337739712	0.574808442
LOC107054125	-0.190164874	0.652310248	0.484872412	0.486223564	0.700617701
LOC112531626	0.071675237	0.652438169	0.10059861	0.751112458	0.874084547
MIR1797	-0.582240041	0.653384409	6.462099232	0.011019938	0.06651984

CACNG7	0.310953142	0.653647289	1.980787839	0.159307434	0.374325836
LOC107051678	-0.267385732	0.653970903	0.967183775	0.325383543	0.56347992
TMEM68L	-0.905457313	0.654396913	12.91852345	0.000325346	0.005223
LOC101751974	-0.058930762	0.656177225	0.047508646	0.827456551	0.918311024
LOC112532944	0.214854571	0.656860402	0.695082178	0.404441104	0.635876015
NOG2	0.073509331	0.657382191	0.052094392	0.819458143	0.915112572
LOC112533468	-0.552273308	0.657409752	4.740333383	0.029463361	0.127819565
LOC107054700	0.49799557	0.658185998	4.786739043	0.028679674	0.12584641
SOCS3	0.206681	0.658299856	0.72552211	0.394338509	0.627445382
LOC112532781	-0.802942758	0.658535467	12.57689326	0.000390545	0.006006004
UMODL1	0.242972834	0.65900129	1.14446679	0.284710447	0.520658654
GJA8	0.041969282	0.660405139	0.032843662	0.856188456	0.93289505
LOC107054454	-0.066219323	0.660465796	0.065300499	0.798306345	0.901588892
PDZD3	0.611677125	0.661866451	5.487014499	0.019158231	0.096494854
ARHGEF38	-0.133209109	0.662242776	0.398802759	0.52770821	0.731483329
LOC112530003	0.122704362	0.663330857	0.33203762	0.56446172	0.75853145
LOC107056111	-0.733076539	0.663540244	9.935011741	0.001621644	0.017149198
LOC112533408	-0.1681243	0.663939083	0.493649558	0.482303821	0.697268439
NEFH	-0.074841099	0.664230838	0.091329549	0.762493665	0.880814575
PAQR5	0.59141427	0.664500461	6.06437698	0.01379354	0.077865936
POU4F2	0.09066791	0.664898502	0.042998069	0.835728908	0.922298307
ACKR1	1.838413526	0.666741698	63.104635	1.96E-15	8.72E-13
LOC429098	-0.031686523	0.667108487	0.013480342	0.907569424	0.957538022
OTOS	-0.356351015	0.668837357	3.199156483	0.073676261	0.229958815
KLHL32	0.40786509	0.669326474	3.745297163	0.05295631	0.186535888
MCOLN2	0.509201413	0.67027604	3.852530508	0.049670997	0.17888734
LOC112532788	0.522484915	0.67114608	2.760916117	0.096592909	0.272583832
SGK2	-0.317776189	0.671773345	0.984584587	0.32106956	0.55845712
LOC107054727	-0.323612835	0.67233003	1.721619179	0.189484755	0.413085802
RPESL	-0.234550766	0.674460239	1.015673869	0.313547382	0.55158832
CD7	0.127339609	0.674567434	0.262132258	0.608659035	0.787473436
LOC107052703	-0.133145356	0.67505719	0.218512877	0.640175375	0.807491962

LOC101748923	0.271538717	0.676281067	2.112389783	0.146110987	0.355317985
HAVCR1	-0.143845162	0.67775278	0.419458967	0.517207133	0.72320219
TIMD4	-1.318203261	0.678235116	21.05259968	4.47E-06	0.000168258
LOC107052672	0.296448723	0.678293165	1.99256378	0.158073072	0.372756147
OPRL1	0.102825832	0.679547071	0.163271471	0.686162241	0.836197732
EGR3	0.31716013	0.679637053	1.950522428	0.162530565	0.378860143
GABRP	0.014069586	0.679660022	0.004111988	0.948870866	0.978488589
LOC101751754	-0.472692451	0.680497383	4.204272155	0.040322275	0.15741623
C11orf97	0.109862644	0.682040609	0.217025943	0.641315404	0.808275503
LOC107054896	-0.532847666	0.682212918	5.662257759	0.017333796	0.090750114
LOC112532904	0.129934272	0.682867478	0.345293635	0.556789457	0.753115441
DPF1	1.133008973	0.682930185	15.74443361	7.25E-05	0.001618729
NFAM1	-0.373800479	0.683775265	2.345003281	0.125685297	0.322292592
GSX1	0.063766784	0.684703151	0.048181333	0.826258659	0.917837275
LOC112533493	-0.307699013	0.688074501	1.578090758	0.209035919	0.43678553
HLA-F10AL4	0.636661646	0.688269706	4.604279449	0.031892253	0.134941379
LOC112532601	-0.081285271	0.689097249	0.070978318	0.78991764	0.896648248
LOC101751743	-0.735696371	0.689106339	5.066354054	0.024394641	0.113233798
LHCGR	-0.291647244	0.69015618	1.249845059	0.263582072	0.498714809
FRMD7	-0.35959694	0.690207675	2.795514263	0.094528434	0.269205523
HAP1	-0.072976762	0.690309871	0.155514813	0.693319948	0.839730586
LOC112531845	-0.842731627	0.690880072	4.882749094	0.027126383	0.121426051
LOC107054336	0.093531504	0.690954528	0.145324143	0.703044434	0.846338149
PCSK1	0.272825652	0.691456862	1.240991102	0.26528013	0.500413628
TRIM29	-0.123037769	0.692052108	0.229276501	0.632060812	0.803037455
POLR3G	-0.372962298	0.69296075	3.031082862	0.081683485	0.245408388
DRC3	-0.248198642	0.693622444	1.377035911	0.240606686	0.473216236
LOC101750765	-0.414637905	0.694199266	3.684747706	0.054912279	0.190718017
LOC107054037	-0.725379314	0.695207578	7.118233282	0.007630386	0.051458199
LOC112531550	-0.074997649	0.696391187	0.083507579	0.772599147	0.88723002
RENBP	-0.22783413	0.69722765	0.950509754	0.329589578	0.567297974
LOC107054482	-0.572799337	0.697956336	7.932234906	0.004856178	0.037480491

PCDHGA2L	0.511597973	0.698859494	4.125263792	0.042247434	0.161820652
ETNPPL	-0.232135763	0.69933175	1.097252795	0.294869792	0.531851631
LOC112532726	0.458171005	0.69963375	4.395072866	0.036042919	0.146008429
LOC107049449	-0.162635945	0.699655171	0.67939838	0.409793967	0.640132877
C1QB	-1.43654747	0.69994396	14.58750808	0.000133799	0.002584103
CABP1	0.006651409	0.700093984	0.000948462	0.975431342	0.990018742
LOC112531372	-0.318680192	0.701105407	0.930370419	0.33476669	0.572087272
KRT10	0.389620513	0.702824588	3.24179439	0.07178198	0.226408374
EXFABP	-0.002390367	0.702938585	0.000116558	0.991386038	0.995986157
SAMD15	0.71754241	0.703124426	9.223640169	0.002389101	0.02260647
MCT2L	-0.300131164	0.703847273	1.874092336	0.171007114	0.389793886
LOC101751990	-0.196268815	0.704081675	0.763884504	0.382115604	0.616744705
LOC107055051	-0.217995986	0.705403071	0.823445472	0.364174713	0.599909391
LOC101750361	-0.045511081	0.706406407	0.020177176	0.887043365	0.949086044
PCDHB15	0.271879347	0.70872177	0.617555233	0.43195723	0.65898908
LOC112532090	-0.71140357	0.708807672	8.003394047	0.004668975	0.036385377
LOC112532647	-0.932797739	0.709622536	14.36330743	0.000150711	0.002843973
LOC101751850	1.504714525	0.710843979	27.82374017	1.33E-07	8.71E-06
LOC107054589	-0.27466522	0.712806692	2.291821804	0.130057187	0.329853731
FAM195A	-0.476840721	0.712889845	4.056983368	0.043989016	0.165511342
GFRAL	-0.244320492	0.712973538	1.592318308	0.206995225	0.434352468
HS3ST4	1.102625394	0.713005076	9.285011445	0.002310365	0.022102937
LOC112530355	0.228779369	0.713128282	1.10418053	0.29335048	0.530584126
BTN1A1	0.268955474	0.713936855	1.293047357	0.25548712	0.490373128
LOC107051716	-0.396746229	0.715190024	1.016826031	0.313273068	0.551313517
LOC112532070	-0.187381413	0.716361865	0.687475721	0.407024311	0.637801433
MSMP	0.06978937	0.717072555	0.071281412	0.789480102	0.896499422
TP53TG5	0.057291062	0.717425343	0.07191037	0.788575325	0.89600932
STEAP1	-0.028429094	0.717647795	0.018157142	0.892810772	0.951217859
HPSE	-0.018138141	0.717846177	0.007433111	0.931295115	0.969881443
LOC107055324	-0.510155382	0.718803011	5.134344771	0.02345694	0.110312644
LOC107052028	-0.122489203	0.720227111	0.314172288	0.575131117	0.765492399

MT3	-0.329109215	0.720339277	2.712046879	0.099593129	0.277387429
LOC101750476	-0.215724838	0.721278875	0.497836716	0.480452196	0.696466157
LOC107053517	-0.142294056	0.723691827	0.342732742	0.558256063	0.753969093
LOC107055059	-0.22206196	0.724325733	0.997592193	0.317893829	0.555837854
LOC112532796	-0.245239694	0.724808666	0.601323843	0.438073365	0.663886934
C3H1ORF115	-0.521285395	0.726314887	5.425773903	0.01984164	0.098662471
SAG	0.546382047	0.727463021	1.825103058	0.176707045	0.397067317
LOC112531570	0.352575857	0.727756002	0.521957427	0.470007892	0.689065646
HAO2	0.246593459	0.728102875	1.212550109	0.270827667	0.505899564
TDRD15	-1.094236719	0.728599062	11.17190863	0.000830452	0.01050352
LOC107051154	1.59625051	0.729213509	18.54055231	1.66E-05	0.000487865
FERD3L	0.058988822	0.729913263	0.045757103	0.830617944	0.919586777
SLC6A5	-0.170575089	0.730592831	0.323282339	0.569641724	0.762379388
C26H1orf116	0.022071244	0.73092558	0.010983427	0.916533043	0.961833624
C10H15ORF59	-0.14859147	0.731625081	0.346992389	0.555820623	0.752912164
LOC112533519	0.801881648	0.731625339	12.33209545	0.000445236	0.006642659
MLNR	-0.343411575	0.732753504	2.334963647	0.126497944	0.323854534
ADGB	-0.017001841	0.732754241	0.006691942	0.934802345	0.971447144
SULT	0.621046375	0.732909102	4.829056387	0.027983943	0.124050586
PRRG4	-0.159948706	0.733025099	0.72430486	0.394735457	0.627589842
LOC107050261	-0.730864627	0.734635125	2.057092934	0.151498957	0.363239886
LOC112533002	0.224130355	0.734875291	0.635144991	0.425474354	0.653827091
LOC107049364	0.854453472	0.734878219	5.925990482	0.01491915	0.081804268
LOC101752112	0.158324386	0.735536115	0.20231417	0.652859363	0.814836227
SCN2B	-0.077313165	0.735613188	0.144963734	0.703395418	0.846389149
ITGB6	-0.338683705	0.736207772	1.969055108	0.160548193	0.376057489
LOC112531068	-0.664083625	0.736558578	2.906250836	0.088236777	0.257938562
SLC22A31	0.591902094	0.738199845	5.364784438	0.020547328	0.100957383
LOC112531061	1.114235221	0.738422664	5.96114188	0.014624566	0.080754108
RAB42	0.041939707	0.738603624	0.04228971	0.83706852	0.923141657
ZBBX	0.12224367	0.738675289	0.368242513	0.543963811	0.74417397
LOC112533190	-0.076509429	0.738713458	0.114605192	0.734960895	0.864717585

LOC112533572	1.820380544	0.739249155	44.88706024	2.09E-11	3.77E-09
LOC107052779	-1.800614419	0.740302051	76.91932447	1.78E-18	1.13E-15
LOC112532458	-0.115137098	0.741200756	0.362292959	0.547235534	0.746725327
PTGFR	0.052138385	0.743401567	0.056136456	0.812710063	0.910356983
LOC107053553	-0.265228854	0.743752125	1.507826527	0.219471039	0.448737316
FGF16	0.366884875	0.744165236	1.878285166	0.170529178	0.389244365
LOC112531773	-0.570728195	0.744220455	6.056250195	0.013857161	0.078088901
LOC107049267	-0.100848077	0.744808604	0.235973546	0.627129478	0.799892687
LOC101751092	0.231747723	0.745866674	1.495734721	0.221328813	0.450849915
LOC101751447	-0.147859864	0.746822935	0.407236798	0.52337534	0.727919042
ANKDD1B	0.042843081	0.74742456	0.032493529	0.856948758	0.933242474
LOC112532797	0.148544491	0.747437268	0.542455998	0.46141666	0.682973027
SH2D1B	1.239164517	0.74777469	10.95612196	0.000932949	0.011430879
TRIM58	-0.00755876	0.751468861	0.000761105	0.97799065	0.990816164
MIR6626	-0.256074277	0.7527498	0.699673123	0.402893551	0.634882978
LOC101751389	0.097848489	0.754562621	0.142862668	0.705451556	0.847437973
LOC107056991	-1.776241241	0.75475272	20.88348029	4.88E-06	0.000181297
ESR1	-0.100227022	0.755043995	0.216426627	0.641776243	0.808610358
TRNAA-AGC	-0.100878542	0.755075243	0.203898231	0.651592536	0.814057792
TNR	-0.856281865	0.755580275	8.219400053	0.00414449	0.033299601
KLHL23	-0.31328808	0.756040912	2.604150147	0.10658428	0.290534586
UGT8L	0.309835627	0.756295441	2.314819344	0.128146188	0.326820447
SLC12A1	-0.018683827	0.757665688	0.00598984	0.938310068	0.973266021
DHH	-0.404289292	0.757838292	1.625721458	0.202295854	0.429146995
PLIN1	0.117883513	0.758156577	0.253737378	0.614455683	0.791137476
LOC112533492	0.2201276	0.758226319	0.742742668	0.388783718	0.622742731
LOC112529965	0.312819611	0.758567418	1.898756746	0.168217545	0.386433017
PHOX2B	0.097051329	0.759083216	0.141819923	0.706478425	0.848162533
LOC107051667	-0.008564043	0.761567738	0.00132424	0.970971298	0.988443637
FADS1L1	-1.517385541	0.762049843	19.16203749	1.20E-05	0.00037493
LOC107052018	-0.289135772	0.762696584	2.404774594	0.120965547	0.314751435
SCARNA15	0.101674446	0.764582315	0.293455364	0.588014713	0.774214049

SNCA	-0.714992067	0.765266395	12.98285594	0.000314356	0.005111393
DSCAML1	0.244985927	0.767738683	1.105106264	0.293148218	0.530432584
DRGX	0.043958838	0.768034497	0.030541947	0.861266222	0.935139468
SAXO2	0.056488124	0.768370113	0.06395906	0.800344496	0.902650356
GSTT1	-0.478393934	0.769048399	6.149750754	0.013143062	0.075276397
ZNF804B	-0.12132591	0.769115861	0.242238659	0.622593892	0.796762373
BLNK	-0.47934017	0.769709485	5.449288199	0.019576301	0.097881507
YF5	-1.744910576	0.770014853	55.95566847	7.41E-14	2.54E-11
SNPH	-0.180844056	0.771350027	0.64244615	0.422826397	0.652377717
LOC101750862	0.145501678	0.773353904	0.412615428	0.520644994	0.72580671
LOC101750386	0.277812354	0.773728386	1.762779482	0.184278205	0.406903552
LOC112531637	-1.034522265	0.774381089	13.3099294	0.000264004	0.0044878
NOXO1	-0.48116119	0.774487134	5.554701421	0.018431116	0.093954158
GABRA1	-0.624896588	0.774939464	7.769207352	0.005314436	0.039858242
LOC107052398	1.391184387	0.777011291	37.73894331	8.09E-10	9.31E-08
KCNK18	-0.877932475	0.777019301	10.36504068	0.001284241	0.014367019
LOC101752242	0.384767469	0.777248762	4.683005857	0.030462401	0.13070634
SLC22A13L	0.322789412	0.778108378	2.519088435	0.112475586	0.300257264
LOC107054234	-0.278970008	0.779913057	2.387865356	0.122280501	0.316820286
SLC2A11	0.286365598	0.780084433	1.813866447	0.178045108	0.398874371
DIO1	0.052992614	0.780227214	0.052970375	0.81797293	0.914012262
KCTD16	0.853268573	0.780427425	15.92212314	6.60E-05	0.001502178
LOC107054889	-0.554159002	0.781010803	5.88074171	0.015307372	0.083074805
LOC101749568	0.458893176	0.781109404	3.328645472	0.068082923	0.219439538
CFAP52	0.41725072	0.781704338	3.409651197	0.064816146	0.212969184
KCNH4	-0.37635839	0.782316059	2.663715898	0.102660649	0.28348397
LOC101749416	-0.654901331	0.78261997	3.989192954	0.045792993	0.169559788
LOC112530111	-1.332656557	0.782726904	23.87607489	1.03E-06	4.87E-05
LOC112530985	0.511804489	0.782875226	5.170804031	0.022969582	0.108735465
LOC101751253	-0.008417586	0.78401298	0.001244438	0.971859193	0.988743849
TTC6	-0.075026874	0.784557697	0.11783606	0.731393411	0.862328105
LOC112532284	-0.002099537	0.784772986	9.03E-05	0.992416607	0.996459368

WDR66	0.418771961	0.784778558	2.986100194	0.083982197	0.249899506
IL1RAPL1	-0.004061098	0.785596821	0.000356087	0.984944606	0.992992257
LOC107052235	-0.373279135	0.786062437	2.739047702	0.097923107	0.274642018
LOC112532753	-0.381352322	0.78679911	2.761768863	0.09654144	0.272485316
CHAC1	0.502949187	0.788101944	3.7316753	0.053389819	0.187466134
LOC112532340	-0.67687475	0.788546027	9.408682287	0.002159604	0.021203031
SNTG2	-0.262923619	0.788977858	1.36894022	0.241994052	0.474840463
AGTR2	-0.779934201	0.78985176	8.083425316	0.004467193	0.035221456
LOC107055358	0.279106947	0.790148224	1.34377875	0.246368676	0.479919939
TDRD12	0.938761742	0.790257787	6.122053815	0.01335056	0.076146781
LOC112532948	0.067993813	0.790609242	0.10853611	0.741816948	0.868980128
SLC46A3	-0.445459082	0.790768929	4.536104159	0.033187041	0.138567052
PARK2	-0.509685156	0.791057109	6.515983711	0.010690917	0.06494847
ADCYAP1	-0.086109634	0.791219755	0.096144364	0.756506549	0.87719789
LPXN	-0.202956962	0.793671994	1.204928868	0.272338777	0.507110397
C8H1orf168	0.193084057	0.794394311	0.63371754	0.425994966	0.654260515
P2RY8	0.052696925	0.794530439	0.07785412	0.780226781	0.891500492
TRPT1	0.053377428	0.79497289	0.068098062	0.794126398	0.899088336
KLHL10	0.45360931	0.795765689	5.317022018	0.021118072	0.102992852
TRPM3	0.186722717	0.795795431	0.953113327	0.328928091	0.56651787
LOC112532084	-0.17766966	0.795931617	0.419892031	0.516990924	0.722961303
FGF5	-0.076165018	0.796188864	0.1273309	0.721215665	0.856063173
AIPL1	-0.780136294	0.796746304	11.4503187	0.000714817	0.00941739
LOC112530784	0.622141529	0.796902824	7.499548756	0.006171445	0.044403644
ADRB3	-0.228463671	0.797192064	0.834128441	0.361081457	0.59750049
LOC112530131	-0.109317119	0.79800969	0.342480327	0.558401017	0.754020084
LOC107051845	-0.378685398	0.798677185	2.606973077	0.106394667	0.290287555
LOC107052361	0.117440506	0.798710466	0.360036589	0.548485916	0.747648785
LOC112530776	-0.281198461	0.799061892	1.616726259	0.203548869	0.430292477
TCTE1	-0.183031462	0.79915759	0.784816695	0.375672059	0.610170585
TCTE3	-0.320273422	0.79918649	2.623254836	0.105308231	0.288309245
LOC107051196	-2.339358501	0.800699749	19.14530057	1.21E-05	0.000376802

BEGAIN	0.224069915	0.804532769	0.647492293	0.42101068	0.65061333
LUZP2	-0.457336257	0.805531941	2.626146789	0.105116535	0.288090036
LOC112532950	-0.05939613	0.805678074	0.10227499	0.749116451	0.873205161
CCDC17	-0.103994179	0.807811614	0.228872192	0.632361345	0.803057713
TRMT10C	0.006485463	0.80879406	0.00074466	0.978229667	0.990904465
MB	-0.946304092	0.809250957	7.482224878	0.006231103	0.044717367
ACSS1L	-0.384012952	0.809599397	2.019663662	0.155273531	0.36842567
MIR6590	0.16899159	0.809879722	0.737972862	0.390311017	0.623951561
CAMK2N2	0.310358873	0.811436121	1.96712626	0.160753224	0.376111802
HIST1H46	-0.428790334	0.811440479	3.082745558	0.079126849	0.240671405
LXN	-0.596782229	0.81338631	9.527085557	0.002024614	0.020320301
Pou5f3	0.038484979	0.813926548	0.033779873	0.854175835	0.932178863
HYDIN	-0.044580202	0.814649901	0.041324513	0.838912813	0.92373597
MAP1LC3C	-0.310357374	0.814689285	1.755148193	0.18523084	0.407836809
NEK11	0.174933505	0.815741943	0.841759119	0.358894146	0.595322901
CNTD1	0.243758337	0.816520912	1.096525293	0.295029924	0.531965527
LOC101747725	-0.652897044	0.816756733	12.12608464	0.000497214	0.007234003
ZNF91L	0.515791386	0.816874393	5.088003724	0.024091909	0.11236745
LOC112530229	0.07721967	0.817573978	0.143233393	0.705087508	0.847186196
TRPM1	-0.67478851	0.81802272	7.110375011	0.007663908	0.051641932
LOC107051584	0.243589886	0.818610058	1.223524572	0.268670061	0.503682048
SLC39A5	0.536403285	0.819029584	5.01454947	0.025135169	0.115498243
LOC101750948	-0.50718373	0.819187434	3.048609303	0.080806309	0.243841522
LOC107056377	-0.614282481	0.819222626	3.750767923	0.052783256	0.186176996
SOHYE2L	-0.559343853	0.819795403	4.051743091	0.04412576	0.165887453
TPSNRL	0.162710961	0.819959827	0.691651106	0.405603345	0.636675604
LOC112531973	-0.247202589	0.819982846	1.423825338	0.232774796	0.464279911
C5H14ORF105	0.16452667	0.82060886	0.550464101	0.458128125	0.680801379
DTX1	-0.504877777	0.82099999	5.790920132	0.016109155	0.086569612
VWA3B	-0.542761874	0.821894401	7.014071574	0.008087156	0.053513207
LOC112532109	-0.792510528	0.822128798	9.337117417	0.002245585	0.021735939
LOC101748220	-0.677784403	0.823255389	7.717215757	0.005469669	0.040578628

FCER1G	0.072740616	0.82387378	0.11684233	0.732484796	0.86296537
LOC101749158	0.154250048	0.825162874	0.176500277	0.67439826	0.828437915
LOC107054010	-0.204681187	0.826555466	0.675175653	0.411252937	0.641368099
LOC107055168	-0.327504199	0.827118371	2.539630318	0.111020777	0.298455627
CPNE7	-0.48782582	0.82713096	3.420758239	0.064381436	0.212042203
LOC112531889	-0.802399758	0.827290664	5.097057018	0.023966473	0.111930673
LOC112531640	-0.598272027	0.828419541	8.516035015	0.003520306	0.02966003
WNT2	0.122226266	0.828588377	0.282856629	0.594834965	0.77845837
LOC107054080	-0.084188658	0.828662991	0.137330246	0.710949668	0.849781439
LOC112532885	0.037583914	0.829166358	0.033174667	0.855473532	0.932733698
LOC112533584	-0.638180176	0.829204935	5.443804331	0.019637852	0.098099835
RAB40B	0.008489037	0.829378183	0.00161903	0.967904038	0.986817198
LOC107053784	0.385020604	0.829775308	2.00292976	0.156995499	0.371332607
BTC	-0.180273993	0.830062572	0.618603724	0.431566624	0.658653601
LOC112530107	-0.040801105	0.831785515	0.046515254	0.829241905	0.918867551
PHF24	-1.253396767	0.833942723	17.41786731	3.00E-05	0.000784798
ANKRD29	-0.064152271	0.834939182	0.074945142	0.784268216	0.894016869
LOC428714	-0.384663063	0.835090311	1.486571992	0.222749097	0.452231511
LOC112532083	0.334596808	0.835337081	2.919075262	0.087538018	0.256476892
LOC112532789	0.440586368	0.835440174	4.218453794	0.039986583	0.15669829
ANKRD61	0.122648966	0.83555963	0.320704917	0.571184323	0.763140471
C1S	-0.166394464	0.835623217	0.641931911	0.423012089	0.65260216
LOC101750628	-0.318581853	0.836154409	2.115711406	0.145794291	0.355022129
C4H2orf81	0.166576543	0.837132713	0.386526351	0.534130974	0.73595064
LOC112532465	-0.189450465	0.837548022	0.907485026	0.340782597	0.577922048
ASL1	-0.234907617	0.837917557	1.040947276	0.307601263	0.54572278
LOC101751329	-0.119477016	0.838681051	0.1375336	0.710745364	0.849781439
LOC107054528	-2.004321664	0.839000804	51.83697835	6.03E-13	1.55E-10
PTH1R	0.025092974	0.83932585	0.005473026	0.941026377	0.974609078
LOC112533499	0.726970033	0.839552236	9.692414166	0.001850303	0.018993419
GPR50	0.494270473	0.840559504	6.183816436	0.012892391	0.074256743
TGM6	0.970328991	0.840851884	16.18186611	5.75E-05	0.001339263

CYP4F11	0.127231056	0.841757804	0.347532278	0.555513383	0.752912164
PLIN3	-0.101371088	0.843726526	0.261921402	0.608803188	0.787473436
LOC112531628	0.499955571	0.843786402	1.953875436	0.162169847	0.378403975
LOC107057089	-0.160929601	0.844419757	0.161651725	0.687640344	0.837288876
LOC101751220	0.173582634	0.844470075	0.692613859	0.405276731	0.636520766
FHAD1	0.230022116	0.845469049	1.162759491	0.280893981	0.516583487
ASCL3	0.229513687	0.846077859	0.67171069	0.412455818	0.642826339
FMNL1	-0.155855195	0.846436051	0.527207772	0.467783141	0.687329661
TNFRSF8	-0.52362359	0.846879229	4.986433949	0.025546804	0.116732013
BHLHE23	0.328425292	0.847299877	2.427520156	0.119221369	0.311642197
NRN1L	0.298804538	0.848639461	1.201993104	0.272923685	0.507692808
LOC112532880	-0.20842866	0.848933054	0.657254386	0.417530925	0.647546783
LOC112532281	-0.012092777	0.849026942	0.003473439	0.953003172	0.979802073
CFAP57	-0.605926835	0.849771699	10.45058662	0.001226105	0.01388545
IL34	-0.086449082	0.850262247	0.13881289	0.709464023	0.849591042
LOC112533454	0.114895998	0.850401112	0.205322078	0.650458883	0.813573027
XDH	-0.393550553	0.850892759	3.512249767	0.060916682	0.204641849
LOC107053348	-0.251346383	0.852630923	1.189774487	0.27537505	0.510396085
FAM107A	0.040881778	0.854779611	0.034709108	0.852206523	0.931269647
LOC107057287	-0.226704481	0.856031427	0.488161243	0.48474868	0.699389701
LOC112531442	-0.145304331	0.856776065	0.397935305	0.528157482	0.731816057
NIM1KZ	-0.256326176	0.857337645	1.487029715	0.222677889	0.452195797
LOC107057261	-0.502735665	0.857958283	2.78240828	0.095304762	0.270386182
LOC101749790	0.289104888	0.858374912	2.348360997	0.125414807	0.321835968
LOC101748261	-0.260277518	0.859141718	1.848111873	0.174003154	0.393551431
BCL2L15	-0.566373448	0.859457771	6.671309929	0.009797715	0.061231068
IRF1	0.04051019	0.860008952	0.033844398	0.85403819	0.932178863
LOC107054678	-0.092532967	0.860550412	0.197523292	0.656727444	0.816752331
LOC107053412	-0.164338285	0.86059013	0.362388958	0.547182454	0.746725327
LOC107054646	-0.613937842	0.860702972	3.438639628	0.063688112	0.210650831
RASL11A	0.15188159	0.861959935	0.373327958	0.541195811	0.74180567
LOC107049468	-0.483917578	0.862712053	4.402822176	0.035879509	0.14556147

CYP8B1	-0.05046539	0.863499695	0.069424587	0.79217643	0.897745396
LOC112530652	-0.229846592	0.864265052	0.517770571	0.471794245	0.690384554
LOC107055024	-1.488146927	0.865195319	46.29739816	1.02E-11	1.94E-09
PDCD1LG2	0.005425622	0.865236017	0.00053838	0.981488325	0.991828264
DEGS2	-0.016744117	0.868036161	0.0058418	0.939075675	0.973691886
CYP24A1	0.07488523	0.868331118	0.052875627	0.818132946	0.914066922
LOC112531876	-0.067104294	0.868391026	0.115191102	0.734309814	0.864249195
LOC107052278	0.28383	0.868653558	1.821429883	0.177143168	0.397557396
SPTSSB	-0.621673936	0.869038095	6.208118564	0.012716571	0.073576366
SYTL2L	-1.014127409	0.869960938	18.82650472	1.43E-05	0.000433842
PRAM1	0.260199439	0.87057761	1.627907485	0.201992717	0.428820818
CDHR4	0.147799097	0.872086166	0.413031739	0.520434712	0.725779864
SPIK2	0.522864141	0.872565626	3.795118931	0.051402221	0.182841235
LOC112532848	-0.30113021	0.87308284	2.03060425	0.154159306	0.36713461
LOC112532073	-0.386466987	0.873680642	2.795510012	0.094528685	0.269205523
CMKLR1	0.485175224	0.87465218	4.318382496	0.037702745	0.150072245
LOC112533019	-0.179456783	0.876598747	0.754011099	0.385209257	0.619731944
LOC112533526	0.062783131	0.878183771	0.103391183	0.747797421	0.872702382
LOC112531851	-0.644253567	0.878210875	3.643361218	0.056293178	0.193787499
LOC107053405	0.095051716	0.878335882	0.21901821	0.639789013	0.807217314
LOC112533200	-1.319792921	0.878757657	22.08064264	2.61E-06	0.000106482
LOC101747919	-0.439165862	0.879898876	4.89753554	0.026895052	0.120785501
RASSF6	-0.084861537	0.881396551	0.140516468	0.707768112	0.848671885
KIF17	-0.159190947	0.882203084	0.646618264	0.42132434	0.650673858
LOC112532316	-0.128959344	0.882278409	0.457145993	0.498960826	0.710180814
MYH1G	-0.8686914	0.882422814	7.556887584	0.005978115	0.043260805
LOC107049572	0.506991774	0.882614502	4.63907766	0.031251794	0.13312148
LOC107049971	0.400252919	0.882957442	4.459246133	0.034712814	0.142421116
CCDC157	0.484878569	0.883000045	5.692454609	0.017038006	0.089772781
LOC107050418	0.744466512	0.884041312	8.656875885	0.003258295	0.028056116
LOC107053883	-0.056730949	0.884924772	0.085333612	0.770195507	0.885380276
CDHR2	-0.206453922	0.886534548	0.606818505	0.43598822	0.662319124

LOC107052216	-1.717827276	0.887464624	33.02415561	9.10E-09	7.97E-07
LOC107052526	-0.067927015	0.888242364	0.114570327	0.734999697	0.864717585
HOXA11	-2.604672074	0.888594803	13.12078973	0.000292037	0.004825204
LOC112532079	0.054241071	0.890435397	0.072643018	0.787526717	0.895442461
MGAT4D	-0.279268826	0.891051753	2.118854543	0.145495325	0.354656516
GRB7	0.039651169	0.891159674	0.022863293	0.879813116	0.946151989
DNASE1L3	0.563649976	0.891412973	5.894787197	0.015185764	0.082714913
LOC112532289	-0.320516935	0.892473878	1.460300134	0.226882372	0.456755952
LOC107054840	-0.281884132	0.893839607	2.54666579	0.110527285	0.297469165
AVD	-0.61741051	0.893883653	7.344087542	0.006728367	0.04705282
LOC422304	0.356559096	0.894599662	3.451874233	0.063180089	0.209555471
C1QA	-1.499199083	0.894927153	20.40981706	6.25E-06	0.000222433
KBP	-0.228202079	0.894993689	1.05753177	0.303778807	0.54181347
C5H11orf16	-0.734508681	0.895041031	8.598831788	0.003363787	0.028694202
H2AFJ	-0.5578639	0.895301225	4.074788556	0.043527714	0.164546302
FBXO15	-0.198756886	0.896456163	0.991033101	0.319490007	0.557135546
LOC107053444	1.328883632	0.897360049	30.94815597	2.65E-08	2.09E-06
LOC112530475	-0.051308717	0.897906756	0.041617198	0.838351204	0.923661189
LOC101750063	-1.093889983	0.898634198	14.26866085	0.000158482	0.002960063
LOC112533535	0.182487515	0.899238484	0.56501374	0.452247155	0.67549848
HSD3B1	0.235545737	0.90034595	1.004597185	0.316200675	0.553730437
LOC107056551	-0.105559423	0.902068553	0.125718542	0.722913173	0.857066913
NIPAL2	-0.355969825	0.90299194	2.957234547	0.08549405	0.252704255
LOC423893	-0.110431128	0.903392597	0.257811126	0.611627944	0.789112265
PLP1	-0.050256006	0.904416675	0.032487892	0.856961032	0.933242474
LOC112531622	0.652820668	0.904491242	6.482570493	0.010893733	0.065951574
LOC107054282	0.051419131	0.905214772	0.041397177	0.838773192	0.923690792
GIMD1	-0.173138551	0.905371174	0.506197822	0.476789439	0.693600685
LOC107049055	2.428303279	0.906777646	53.00422971	3.33E-13	9.61E-11
LOC112533012	-0.791153133	0.907275589	5.700989233	0.016955354	0.089511932
SIX2	-0.551776523	0.907347619	2.970328352	0.08480464	0.251493697
LOC112532133	-0.277134299	0.910065553	0.814270633	0.366860641	0.601983631

GJB2	0.009168364	0.910874811	0.000815968	0.977211408	0.990525896
LOC112530939	0.795120273	0.911433319	9.624805238	0.001919668	0.019564254
LOC776376	1.200907527	0.911633722	31.38050225	2.12E-08	1.73E-06
ADRB1	-0.076977177	0.913092894	0.177398746	0.673618313	0.827811004
LOC101748203	0.030454006	0.913222781	0.026661431	0.870295394	0.940243379
VIP	-0.078956846	0.914252731	0.090911437	0.763021634	0.881102924
SOWAHA	-0.154098527	0.91554294	0.490507045	0.483701211	0.698351687
LOC107054507	0.18609129	0.915865405	0.510259809	0.475026409	0.692343627
LOC112531697	-0.21655085	0.9171603	1.145215735	0.284552908	0.520488895
LOC112531967	-0.176118109	0.917790786	0.124067988	0.724663635	0.857989647
GAL	-0.815627187	0.919804259	9.046781446	0.002631577	0.024083758
LOC112530034	1.188177715	0.920127037	22.80815279	1.79E-06	7.73E-05
LOC101752186	0.745929624	0.92035933	9.245682837	0.002360513	0.022491161
LOC107052325	0.261083523	0.92077301	1.496528518	0.22120628	0.450656101
XKRX	-0.268891223	0.921468581	1.194625497	0.27439852	0.509389401
LOC429348	0.424972872	0.92150655	2.804255371	0.094014486	0.268624477
MIR6660	0.005583564	0.921895493	0.000890971	0.976187369	0.990025465
LOC101747667	-0.502967305	0.92317073	2.871298756	0.090172076	0.262036199
ADM	0.272292123	0.923180316	1.390012412	0.238403004	0.470995489
BLB1	-0.166013279	0.924349508	0.325920604	0.568071115	0.760970118
SLC9A9	-0.034782851	0.925386764	0.018825046	0.890869236	0.950849924
LOC107054395	-0.109955445	0.925764956	0.299253488	0.584350795	0.771337425
LOC107053752	0.343702451	0.925818359	2.557367086	0.109781279	0.295945436
MYOC	0.028852379	0.926696591	0.014455603	0.904299802	0.956314475
EFCAB1	-0.104468826	0.929531122	0.262948896	0.608101424	0.787414637
MOG	-1.090764321	0.929772627	15.05050829	0.000104672	0.002142262
LOC101748755	-0.583588697	0.929784215	8.40618088	0.003739474	0.031033114
MAP3K8	-0.270647546	0.929960117	0.651158969	0.419698622	0.649223958
CYB561	-0.308255608	0.93010513	1.936475255	0.164051757	0.380904707
LOC112532110	-0.046294993	0.931192473	0.032927175	0.85600773	0.932863069
SFRP4	-0.670263526	0.931317117	9.174287553	0.002454389	0.022960188
CORIN	-0.185107436	0.93156578	0.485735374	0.485835849	0.700418329

SLC7A10	0.113719387	0.932026141	0.423728991	0.515082191	0.721887186
OXTR	0.062674156	0.932900428	0.072529095	0.787689396	0.895442461
RFX6	0.608578748	0.93320786	2.085594108	0.148694363	0.359237372
LOC112532407	0.057522842	0.933788237	0.063822771	0.800552839	0.902761579
LOC101751659	0.14198395	0.935197218	0.488687452	0.484513387	0.69923415
HOXA10	-1.925991008	0.935420886	9.579814093	0.001967283	0.019921013
MIR1715	-0.360048709	0.935683245	3.994001175	0.045662509	0.169305225
ST8SIA6	-0.288866814	0.936283907	2.116198291	0.145747935	0.354989728
GC	0.318252496	0.936297134	1.347983384	0.245630974	0.479249328
CEBPB	0.660797973	0.936333084	6.887271928	0.008681166	0.056239599
STX3	0.284131961	0.936489851	2.079332049	0.149305499	0.36039636
HOXC9	-0.358365503	0.936521722	0.541776392	0.461697463	0.683216979
LOC107051722	0.142895005	0.93695089	0.212662687	0.644688374	0.810541251
LOC112532081	0.345247068	0.937507078	2.837612521	0.092080937	0.265283762
SLC26A6	-0.805990811	0.937642223	7.841273681	0.005106661	0.038741407
CFAP58	0.665043105	0.938280088	11.75720428	0.000606082	0.008338694
CLEC2D2L	0.110770796	0.939096646	0.240016929	0.624193888	0.798128093
RBM46	0.409218967	0.940296827	4.03308094	0.044616392	0.166715272
KCTD17	-0.218052644	0.941216344	1.125851453	0.288661965	0.525172427
LOC107049548	0.406644919	0.942651983	1.810041097	0.1785033	0.399410287
LOC112530143	-0.175433832	0.942934376	0.632377274	0.426484653	0.654584923
ANKK1	-0.998075993	0.943370794	10.76453117	0.001034638	0.012345876
LOC107056929	-0.217952759	0.94407452	0.638753894	0.424162391	0.65309226
ERC2	-0.290862497	0.944445773	1.694589934	0.192997018	0.417328557
RASSF5	-0.223865997	0.944583197	0.813304022	0.367145215	0.602224508
LOC112530367	-0.213939741	0.944685821	0.989962831	0.319751459	0.557135546
LOC101750993	-0.439730099	0.944916235	3.226783777	0.072442832	0.227446442
SKAP1	-0.070943363	0.945321272	0.125043234	0.723627785	0.857318396
LOC107053520	-0.355644498	0.945394958	3.731264341	0.053402956	0.187466134
SMIM5	0.042912864	0.945710474	0.041148582	0.83925139	0.923799694
TCTEX1D1	-0.153430658	0.948089715	0.455162787	0.499893371	0.710640814
LOC112532926	-0.436896234	0.948717091	5.185319826	0.02277848	0.108048395

PNAT10	-0.007887482	0.948865086	0.001405702	0.970092167	0.987864058
HIST1H2A4L4	-0.138907197	0.948897767	0.542824414	0.461264549	0.682809298
LOC112530236	-0.326512288	0.949157202	3.560342625	0.059175523	0.200521672
ASAH2	-0.172980237	0.949265007	0.582969639	0.445150666	0.669924475
LOC112530190	-0.048785861	0.950345763	0.050082491	0.822919795	0.916676498
LOC101748818	0.688539914	0.950485844	10.41982946	0.001246694	0.014031704
COLQ	-0.113087951	0.951214947	0.202934446	0.6523626	0.814586931
MMP9	-0.235661627	0.951871198	0.801782753	0.37056089	0.605038643
SNORD12C	0.016364536	0.952025581	0.008033456	0.928581604	0.968392732
LOC107050307	0.601908705	0.953238965	3.851211758	0.049710064	0.178988864
LOC101752071	-0.425412152	0.95433297	4.582110838	0.032307393	0.136033958
CAMK1G	-0.151346967	0.954653446	0.549582192	0.458488461	0.68090502
OTOF	0.276800813	0.955067386	1.157471347	0.281990579	0.517706338
LOC101747516	0.090678248	0.956443644	0.196555247	0.657515838	0.817412048
PROM2	-0.591836661	0.957720786	5.384502549	0.02031638	0.100207722
ST8SIA3Z	-0.860143259	0.957742691	9.314319086	0.002273699	0.021892165
FAM46C	-0.646557532	0.95833081	8.853307269	0.002925583	0.025951735
LOC101751792	-0.63094605	0.958691141	5.94657293	0.014745927	0.081124783
SLC32A1	-0.126259928	0.959170425	0.276620009	0.598925189	0.781613119
LOC107051978	0.364221104	0.959651558	3.267056266	0.070684354	0.224487636
CREB3L3	0.044936467	0.960815962	0.025069247	0.874194636	0.942721236
LOC107053000	-1.838084001	0.960852137	71.79031308	2.39E-17	1.27E-14
LOC107054476	-0.382688212	0.960949371	3.104579445	0.078072393	0.238433783
IL15RA	0.290726731	0.961453551	2.205816528	0.137491076	0.341961254
LOC107054654	-0.286265795	0.961695919	0.223994207	0.636013241	0.804922157
LEPR	-0.041559037	0.963575207	0.023283512	0.878722119	0.945613267
FOXE1	0.105119524	0.96399233	0.167911099	0.681974992	0.833288668
LOC107051706	0.632964063	0.964730191	6.433507153	0.011198724	0.067229113
LRMP	0.136420137	0.965401599	0.354717846	0.551454545	0.749994589
LOC107052981	-0.105615777	0.966498936	0.157173495	0.691772256	0.839091293
LYPD1	0.636591899	0.967119513	7.25766502	0.007059902	0.048485265
ADH1C	-0.057385126	0.967307978	0.069510584	0.792050706	0.897745396

SYTL5	0.054540421	0.967669162	0.048690856	0.825357144	0.917552618
HIST1H2A4	-0.4596197	0.969654333	3.698611849	0.054457757	0.189892433
CDKN1A	-1.604531217	0.969855328	12.45877526	0.000416033	0.0062979
LOC112533443	-0.186421553	0.972985472	0.690907732	0.405855799	0.636943936
METTL11B	-0.404314063	0.973397651	3.402460208	0.065099257	0.213600853
LOC101747537	-0.228994407	0.973462649	0.862739849	0.352973099	0.589542462
LOC107050031	0.205353179	0.974096593	1.028531492	0.310503828	0.548336682
LOC107053369	0.447523202	0.974965521	5.046220447	0.024679716	0.113946332
LOC107053829	0.072498568	0.975492464	0.051737012	0.820067846	0.91542069
LOC112532322	-0.305879445	0.976508979	2.554880455	0.109954131	0.296265798
BOLL	0.050349197	0.978028735	0.071269694	0.789496999	0.896499422
MLPH	-0.742378101	0.978380605	14.63134922	0.000130722	0.002545595
LOC107050760	-0.370178311	0.978753639	3.760438335	0.05247882	0.185269036
LOC112533232	0.550364759	0.979345082	5.433705214	0.019751729	0.098454542
CFH	0.159052862	0.98026377	0.251140361	0.616273257	0.792126125
ALOX5	-0.210187594	0.98075422	1.054081441	0.304568963	0.542507284
LOC112531535	-0.519389869	0.982151691	4.673631527	0.030629096	0.131113886
CHRNA10	-0.992892087	0.98241321	10.95019016	0.00093594	0.011458999
HHIPL2	0.073863378	0.982716434	0.129656005	0.718788971	0.854724131
LOC107052954	0.07606379	0.982797106	0.065485437	0.798027109	0.901457821
MACC1	0.156911186	0.982978272	0.412890336	0.520506118	0.725779864
GCNT4	-0.678075454	0.983390561	7.380610343	0.006593079	0.046462149
ANKS4B	-0.16471118	0.984901252	0.641636033	0.423118986	0.65260216
LOC101749719	-0.227144497	0.987115129	0.895637177	0.343954244	0.580854259
NDST4	-1.005038721	0.987133741	23.35531025	1.35E-06	6.09E-05
GHRL	-0.26947851	0.987336571	1.560575821	0.211580929	0.43919354
ESPN	0.096953971	0.988186887	0.187754919	0.664791785	0.82230691
ARTN	-0.5752951	0.989469899	3.608581546	0.057482128	0.196932836
HSD17B1	-0.333520743	0.990467619	2.774235344	0.095792394	0.271160185
LOC107052934	0.022681986	0.990791155	0.014871088	0.902940936	0.955614537
SDR42E2	0.704098548	0.990956111	6.506948627	0.010745374	0.065173288
APBB3	-0.038248732	0.991289284	0.022956017	0.879571505	0.946151989

LOC112530272	-0.027893841	0.992231565	0.020465535	0.88624453	0.948791653
LOC112531796	-0.21372102	0.993548267	1.119650976	0.28999362	0.526868171
GJB3	0.322194444	0.994244844	2.427151635	0.119249405	0.311660395
LOC112530973	0.236833275	0.994279505	1.623430887	0.202614062	0.429233617
LOC101748347	0.301720374	0.994458962	2.679214654	0.101665855	0.281350763
LOC107054576	0.009545521	0.995489898	0.002347407	0.961357578	0.983777298
TMPRSS13	-0.106427432	0.999276189	0.211630941	0.645492081	0.810805381
ANKRD42	-0.213720563	0.999282274	1.281975316	0.257532082	0.492411155
LOC112532778	0.591914374	1.000395412	9.19168626	0.002431168	0.022833831
LOC107052574	-0.267796579	1.00084811	1.782255161	0.181872649	0.404038672
FGF7	0.001479507	1.00096162	1.30E-05	0.997121727	0.998764949
LOC107056921	0.262775993	1.001593499	1.42600297	0.232417865	0.463792866
GSL	0.037428415	1.001605562	0.026610621	0.870417952	0.940243379
EFCAB2	0.289157692	1.00161484	1.938987638	0.163778497	0.380418954
EAAT5L	-0.419001391	1.002403785	2.329585822	0.126935647	0.324621184
LOC100859173	-0.853604955	1.002647777	6.355513856	0.011701705	0.069313013
LOC107049245	-0.125688489	1.002655571	0.316748535	0.57356824	0.764869254
CALR3	-0.129227077	1.003872521	0.314434775	0.574971495	0.765465692
LOC101751474	0.17337491	1.004227049	0.925074429	0.336146111	0.573308899
SLC15A2	-0.485922808	1.004245858	4.348153296	0.037049099	0.148403829
NANOS3	0.136976115	1.004798421	0.390537428	0.532017042	0.734393751
LOC428541	0.393254086	1.006242561	3.26648046	0.070709171	0.224487636
LOC107051626	-0.181451468	1.006400017	0.77004367	0.380203543	0.614751317
LGALS3	-0.296142829	1.006476504	1.533257425	0.215624221	0.444540058
LOC107052265	0.060698756	1.006808602	0.074105378	0.785450535	0.894435194
LOC112533527	-0.361419686	1.00760801	2.717707009	0.099240487	0.276639372
LOC107052596	-0.306067019	1.009390592	2.189671796	0.138938918	0.344313238
LOC107054952	-0.124149141	1.009814477	0.428659329	0.512647529	0.720192529
LOC107055010	-0.511195144	1.010660106	5.918746919	0.014980609	0.082031922
GIMAP7L5	0.029033721	1.012105248	0.016088117	0.899067874	0.953969685
LOC101750929	-0.044926746	1.012741805	0.056473711	0.812158787	0.909908068
LOC112532326	-0.06510963	1.013373786	0.05956855	0.807179169	0.90693979

TDO2	0.235504384	1.013418069	0.633329295	0.42613673	0.654356093
LOC112530029	-0.285161049	1.014465797	1.335730638	0.247788284	0.481900038
CARD8	0.567291047	1.014718389	10.13265849	0.001456652	0.015800397
MIR1577	-0.218061687	1.015741373	0.887970316	0.346027926	0.582540186
MMEL1	0.137984065	1.016169322	0.300446351	0.583602734	0.770936649
LOC112530112	0.895322469	1.016344685	10.71462617	0.001062919	0.012555877
LOC112531929	1.057070948	1.016690379	15.56254389	7.98E-05	0.001748925
LOC112531891	-0.74275002	1.01669402	4.600062168	0.031970797	0.13516944
SMIM18	-0.348415946	1.017082971	2.483523657	0.115044255	0.304741342
PVALB	-0.360358408	1.01718265	1.374361362	0.241063952	0.473806419
LOC107052080	0.131187764	1.01827683	0.32109369	0.570951118	0.763014752
CHRNA7	-0.363017333	1.018410649	1.910193372	0.16694178	0.384953334
LOC112531515	0.430580232	1.018522395	4.315341721	0.037770184	0.150195356
LOC107055044	-0.045435113	1.0195534	0.0414696	0.838634161	0.92367638
CLDN4	-0.147528084	1.020258332	0.51031524	0.475002423	0.692343627
LY86	-0.001226269	1.020844658	4.68E-05	0.994539155	0.997544036
C1H22orf23	0.567741917	1.021485619	3.897820567	0.04834879	0.175469638
GRID2IP	0.476512371	1.02384439	4.807742498	0.028332138	0.124748861
LOC107054243	-0.308697687	1.024101714	2.752829315	0.097082499	0.27310954
LOC112533409	-1.205495684	1.025843607	19.43862397	1.04E-05	0.000335181
C10orf10	-0.620350121	1.026323856	10.41824381	0.001247765	0.014034157
LOC107051690	-0.254739615	1.028428781	0.693459113	0.404990294	0.636313883
AIFM3	0.422129856	1.028753943	4.298227441	0.038152118	0.151020712
LOC430443	0.071090184	1.029125876	0.018118346	0.892924657	0.951217859
LOC107050789	0.062243106	1.029157135	0.106995049	0.743591513	0.869767312
LOC107053610	-0.424310148	1.029704303	1.844318417	0.174445645	0.393921104
ELFN2	-0.623377675	1.030468964	5.100998363	0.023912077	0.111825554
GJB5	-0.636347893	1.031053482	7.795806747	0.005236762	0.039472239
LOC101750376	-0.737773575	1.031067611	14.94264841	0.000110829	0.002215908
ICAM5	0.153062366	1.031272592	0.403671934	0.525198984	0.729911257
GALNTL6	-0.74580459	1.032479839	13.3254446	0.000261829	0.004463726
GEM	0.936029205	1.032919981	14.42112843	0.000146153	0.002784082

WNT7B	-0.273903139	1.033021065	1.554866939	0.212418385	0.440386106
TMEM117	-0.005102957	1.033413354	0.000773613	0.977810577	0.990744261
LOC107054324	-0.826281696	1.035167949	6.722019845	0.009522972	0.059900802
LOC112531518	-0.317341621	1.036565533	1.24654111	0.264214136	0.499384748
CD274	0.202363499	1.036882001	1.059985875	0.303218406	0.541196355
MIR2129	0.299148516	1.036911826	2.624480494	0.10522694	0.288248594
LOC107054578	-0.10813838	1.037061313	0.262145651	0.608649881	0.787473436
AGBL1	0.404620634	1.038547795	4.202333673	0.04036839	0.15741623
HSD11B1a	-0.661053701	1.038684364	4.90459622	0.026785313	0.120555889
LOC107054735	0.830706298	1.038771979	6.280594179	0.012206717	0.071506416
LOC107053999	1.362000892	1.03995578	32.2565656	1.35E-08	1.15E-06
LOC107053144	0.5018598	1.040441086	5.013868123	0.025145063	0.115511448
LOC107052611	0.134143965	1.040605508	0.467949771	0.493931885	0.706154037
COPRS	-0.072477599	1.042329693	0.09457009	0.758445698	0.878579652
TMEM255B	0.195904438	1.042399408	1.051565932	0.305146714	0.543183598
LOC107052898	-0.174474422	1.042760759	0.746053405	0.38772863	0.62183963
CCDC146	-0.132525055	1.043629051	0.299850442	0.583976196	0.771126537
LOC107052542	0.266632455	1.044164071	2.076067169	0.149625255	0.360903486
LOC112533579	-2.327085944	1.045338428	38.56554933	5.29E-10	6.31E-08
LOC107052210	0.246513702	1.045774137	1.420434869	0.233331839	0.464849933
TRIM14	-0.117484085	1.046637587	0.277401401	0.598409511	0.78124631
LOC107054285	0.370784665	1.046826032	3.434301783	0.063855571	0.211035031
SHOX	-1.244985669	1.047554168	7.185307493	0.007350295	0.049965525
LOC112531986	0.268131521	1.0480588	2.019660059	0.1552739	0.36842567
PIGZ	0.154459246	1.048100068	0.578938238	0.446728799	0.670621395
HCLS1	0.146533246	1.04813557	0.395648956	0.529344916	0.733053537
MIR1683	-0.627008644	1.048604571	9.920085157	0.001634848	0.017244504
SHISA8	0.369428519	1.049649565	3.109626323	0.077830815	0.238215389
LOC101749720	0.378326213	1.051118517	2.437850974	0.118438387	0.310681277
CFAP221	-0.235241637	1.051209162	1.025596202	0.311195242	0.548903174
LOC112532382	0.609534259	1.051635134	9.00756221	0.002688648	0.024429433
LOC107052181	-0.311525525	1.052064565	2.269497642	0.131942665	0.333300054

FYB	-0.17567005	1.055010896	0.328158238	0.566745602	0.760302833
MYO15A	0.101821261	1.056161038	0.202605592	0.652625856	0.814728097
MASP2	0.39414049	1.056597922	4.352406026	0.0369567	0.148216938
KCND2	-0.407357379	1.05742916	3.042593248	0.081106254	0.244477636
PGR	-0.297097772	1.057709581	2.232308849	0.135151754	0.338271869
LOC112530576	0.386090255	1.057824626	4.736520159	0.029528742	0.127899143
LOC107050271	1.358631378	1.0580724	19.02786702	1.29E-05	0.000394746
LOC112533575	0.093337063	1.05857321	0.212997425	0.644428129	0.810398537
LOC101750048	-0.611875495	1.059527325	9.295123061	0.002297647	0.022071093
LOC107054710	0.218798611	1.060540665	1.234638939	0.266506768	0.501815868
LOC112533097	0.160366328	1.062927974	0.686625047	0.407314705	0.637865574
MIR1555	0.003567452	1.064342297	0.00029241	0.986356843	0.993448882
ADGRF4	0.192484095	1.066428046	0.836614816	0.360366733	0.597043354
ACACB	-0.708959785	1.066580864	9.478683593	0.002078731	0.020718058
C1orf210	-0.125074437	1.067448669	0.410413837	0.521759533	0.72666552
LOC112531696	-0.125764525	1.068045968	0.401952981	0.526082376	0.730515158
LOC101747910	-1.264999282	1.068635374	51.80165228	6.14E-13	1.55E-10
LOC107052879	-0.166698273	1.068874206	0.635676682	0.425280683	0.653696874
PRLHR2	0.23563898	1.069351345	1.290541438	0.255948195	0.490715523
S100A13	0.142060632	1.070540873	0.45001226	0.502329132	0.713186011
LOC107050029	0.04885626	1.070575338	0.043869587	0.834096434	0.921639593
PTHLH	-0.864522977	1.070630995	7.062195609	0.007872745	0.052618206
LOC101750761	-0.266080161	1.071691879	1.541232128	0.214434499	0.443504233
LOC112530110	-0.245420576	1.071747384	1.994602612	0.157860467	0.372575155
LOC112533460	-0.567653368	1.071957398	4.398516487	0.035970208	0.145852751
FOXS1	-0.30170852	1.072235913	2.004580737	0.156824648	0.371214577
LOC107054297	0.122214776	1.072307519	0.357131708	0.550103543	0.748776061
HTR2B	0.602610974	1.072538921	8.488644223	0.0035737	0.030033318
NLRC3	-0.731921252	1.072540683	12.94468772	0.000320831	0.005185267
LOC100857820	0.244430059	1.072888849	1.566040813	0.210782916	0.438542533
NGB	-0.347423373	1.072997688	2.803243978	0.094073796	0.268739593
LOC417142	-0.253563078	1.073764346	1.326681767	0.249396394	0.483598593

TLR1B	0.3227844	1.074151656	2.255392993	0.13314965	0.335082178
SLC31A2	-0.040981721	1.074257318	0.04782336	0.826895023	0.917915823
SLC16A13	-0.190326173	1.075963943	0.530002434	0.466605836	0.686577755
LOC112531624	-0.196181944	1.076786993	0.994733637	0.318588178	0.556337522
LOC101748011	-0.008267874	1.077170232	0.00123024	0.972020114	0.98878868
IL31RA	-0.169348926	1.07723408	0.704825468	0.401167002	0.633695211
VWA2	0.175883883	1.078542354	0.511853186	0.474337725	0.69207548
CALHM1	0.319481159	1.078616348	2.396313491	0.121621558	0.315675937
NYAP2	-0.408207136	1.07993154	3.051028879	0.080686013	0.243612539
ADAP2	0.046201134	1.080712294	0.072610271	0.787573465	0.895442461
CMPK2	-0.02304812	1.082014292	0.009343523	0.922994898	0.965045181
LOC107052035	0.065295191	1.082573369	0.086181419	0.76908901	0.884804563
PLCXD1	-0.693633155	1.082905701	6.904343597	0.008598659	0.055903174
FHIT	0.347524624	1.082993195	3.450478054	0.063233478	0.209610573
LOC101750501	-0.184674944	1.08696984	0.546840322	0.459611619	0.681650219
LOC101748973	0.072178677	1.088670669	0.11142087	0.738532258	0.866862708
ATP6V1C2	-0.150457732	1.088962418	0.703515069	0.401605095	0.634066049
KIF12	-0.142587346	1.089000563	0.503912791	0.477785903	0.694090848
LOC107054313	0.027222931	1.089289072	0.015702979	0.900276926	0.954481623
LOC107050017	0.119256115	1.091027962	0.308592808	0.578544981	0.767986259
CRHR1	-0.530485184	1.092070869	4.333601109	0.03736711	0.149133105
LOC107049673	0.246950178	1.092271315	1.85370291	0.173353338	0.392718995
VWA3A	0.652993589	1.092758282	12.83955512	0.000339368	0.00540591
SLC27A6	0.824598293	1.093559079	18.24622982	1.94E-05	0.000551654
NOBOX	-0.312187158	1.094463048	1.231903503	0.267037171	0.5024696
LOC112531018	-1.196028192	1.094868136	20.31179661	6.58E-06	0.000231332
LOC112532477	-1.072834686	1.095088271	27.03472071	2.00E-07	1.22E-05
LOC107052785	0.364113163	1.095436728	2.976727064	0.084469926	0.250969429
NPTX1	0.276218181	1.095991228	1.39955067	0.236798818	0.469065191
LOC107054147	-0.391040443	1.096847115	4.041703322	0.044388998	0.166444744
PIRT	0.355063691	1.097198621	2.110297984	0.146310825	0.355567069
LOC107055169	0.301534625	1.09748565	1.797600429	0.180002862	0.401619945

LOC112531634	0.425751599	1.098042168	3.604771973	0.057613969	0.197247834
STAP1	0.173683244	1.098721392	0.859937787	0.353756102	0.590430739
LOC101750188	-0.109819895	1.098764012	0.121167428	0.727771752	0.859813626
LGALS2	0.422108413	1.098781363	2.16660174	0.141037597	0.347956991
LOC112530151	-0.264816602	1.099164619	1.88191621	0.170116518	0.388949187
ANKRD9	-0.367132521	1.099226333	4.467703361	0.03454139	0.14205913
KCNF1	-0.348220359	1.099792534	1.773733794	0.182920667	0.405436365
TDRD1	0.201368239	1.100784273	0.806296731	0.369217393	0.603962551
LOC107051771	-0.352390825	1.100923456	2.574555482	0.108594603	0.293970224
GPLD1	-0.00093837	1.101898657	1.89E-05	0.99653097	0.998533498
LOC107053396	0.085141502	1.102469718	0.210422683	0.646436311	0.811495384
CMYA5	-1.048912493	1.102818399	11.31361063	0.00076941	0.009898861
LOC107052353	-0.527841438	1.103035582	3.429605907	0.064037381	0.211466004
SSTR1	-0.436411021	1.103947619	4.28622607	0.038422361	0.151725452
ROPN1L	-0.441460707	1.104067047	5.551684391	0.018462911	0.094028848
LOC112532552	-0.133314176	1.104975434	0.327287775	0.567260526	0.760711734
ARMC4	0.411635527	1.105691032	4.684507498	0.030435787	0.130626207
SYT2	0.00548818	1.105857506	0.000444519	0.983178943	0.992467303
LOC112529979	0.95620318	1.106230863	12.31105541	0.000450283	0.006699287
PFN4	0.317307087	1.106375082	2.335387215	0.126463541	0.323839914
USH2A	-0.510911936	1.106618368	8.237616394	0.004103098	0.033080098
ITGB2	-0.22106551	1.109299155	0.800221792	0.371027066	0.605499391
LOC112533561	1.331999776	1.109520579	18.17182897	2.02E-05	0.000567747
TMEM139	0.280885687	1.110395157	1.032794194	0.309503294	0.547425629
LOC112532470	0.578099041	1.111784489	4.113852796	0.042533363	0.162368316
AQP4	-0.405526722	1.112384154	2.389091858	0.12218459	0.316721398
ITGBL1	-0.874370437	1.113931501	15.82281439	6.96E-05	0.001565785
LOC107053889	-0.813549252	1.114456769	10.98051784	0.000920747	0.011306636
LOC107053429	0.87088054	1.114739337	22.44909503	2.16E-06	9.01E-05
LOC112531806	-0.084890816	1.115078675	0.19258303	0.660775424	0.820108576
LOC107052388	0.076756856	1.115255653	0.127878717	0.720641677	0.855792151
FGF19	0.134473434	1.115607223	0.335634642	0.562359893	0.756921375

CHST7	0.130352077	1.116647245	0.532486988	0.465563151	0.686036347
RIMS3	-0.091304651	1.116845431	0.302474186	0.58233547	0.77051357
LOC107054262	-0.015828626	1.116938702	0.006347858	0.93649698	0.972182058
EXTL1	0.011026242	1.118954	0.003717032	0.95138512	0.979652938
LOC112532911	-0.081790072	1.119855773	0.077674978	0.780473289	0.891667567
LOC107051468	-0.298233969	1.119932395	2.048710186	0.152335198	0.36444834
PITX1	0.082581251	1.121039787	0.031953691	0.858129332	0.933773189
PTGER3	-0.098296497	1.121499304	0.286019716	0.592782553	0.777327412
MIR6602	-0.215276344	1.121579842	1.458433604	0.227179515	0.456941563
LOC107050583	-0.274805686	1.123964322	1.275280735	0.25877835	0.493272928
LOC107049660	-0.879269677	1.124464073	14.45694434	0.0001434	0.002746456
LOC770352	0.777554011	1.125009496	5.368442791	0.020504275	0.100810319
LOC101750930	-0.07938131	1.125545157	0.161964119	0.687354601	0.837167124
HLA-F10AL3	1.508794348	1.126160894	44.66535133	2.34E-11	4.14E-09
SNCG	-0.517630259	1.126804842	4.782635196	0.028748096	0.126044495
LOC107049565	-1.211532921	1.12718026	22.81714935	1.78E-06	7.72E-05
VDHAP	-0.220685782	1.127700602	0.659627662	0.416691423	0.646851271
LOC107052921	-0.2025176	1.127702661	0.637971075	0.424446457	0.653162484
CRYGN	0.648840024	1.129236443	10.27382938	0.001349305	0.014877209
LOC112532686	0.278291499	1.129303212	0.93867292	0.332619366	0.570249185
LRRC3B	-0.738037177	1.129698688	6.885734878	0.008688634	0.056243694
BATF3	-0.02713501	1.129990236	0.021640146	0.883048433	0.947789574
LOC107053888	-0.013170422	1.1301502	0.003221314	0.954739072	0.980541221
LOC107053044	0.039504211	1.130593021	0.026279748	0.871218999	0.940731028
PLLP	-0.691165419	1.130792969	4.62556734	0.031498843	0.133637498
CDX1	0.932779385	1.132273149	4.330198686	0.037441875	0.149322844
LOC112532132	-0.189546333	1.13331111	0.647373328	0.421053352	0.650618171
LOC107053028	-0.817523236	1.137374047	9.478947712	0.002078431	0.020718058
LOC112532443	0.147361823	1.137606476	0.523912658	0.469177409	0.688269654
LOC107053576	0.017644631	1.138609816	0.00479891	0.944771388	0.976398266
LOC112530237	-0.509162255	1.140031082	3.232761364	0.072178887	0.227137805
TMEM268	0.034183386	1.140192614	0.026713982	0.870168764	0.940243379

LOC101750621	-0.31562161	1.143363297	2.466490719	0.116297319	0.307120817
TMEM234	0.221356593	1.143437	1.544277979	0.213982158	0.442791299
LOC107051906	-0.497719894	1.146558111	4.808720307	0.028316065	0.124748861
LOC107050775	-0.242245136	1.14680392	1.148690583	0.283823424	0.519963755
SP7	1.003755696	1.146871243	11.27481757	0.000785656	0.01005285
LOC100857659	0.019663877	1.147449453	0.010912513	0.916801949	0.961993117
LOC107054857	0.322407464	1.148661193	1.795030897	0.180314398	0.402151453
RGS17	-0.249222502	1.149431762	1.614952216	0.203797068	0.430597946
TRPA1	-0.862799251	1.149754225	9.095335605	0.002562622	0.023669926
RBM11	1.043545712	1.150186482	21.15159226	4.24E-06	0.000160891
LOC112532503	0.141233856	1.15121076	0.354853366	0.551378532	0.749953194
LOC771456	-0.234742695	1.151437017	1.643063404	0.199905675	0.426039098
LOC112532917	-0.123740503	1.151620127	0.412791773	0.520555902	0.725787779
GAD1	-0.271892418	1.151860288	1.474604532	0.224620605	0.45418187
LOC101749837	-0.109233102	1.152521519	0.279254037	0.597190565	0.780221347
TTLL2	-0.408837234	1.153704112	4.853081698	0.027596785	0.122897185
PPARG	-0.217826462	1.15407674	1.491979329	0.22190961	0.4514183
DRD1	-0.125310441	1.154278936	0.498093008	0.480339239	0.696466157
PERP1	0.208251435	1.154634196	0.950688003	0.329544234	0.567282181
LOC107052870	-0.073700008	1.155014359	0.098791482	0.753284751	0.87500357
CD163	0.314364757	1.156231676	1.916826394	0.166206939	0.38395833
POLD4	0.006841412	1.156991802	0.000909507	0.975941023	0.990018742
COMP	-0.298918268	1.159323337	3.278297519	0.070201716	0.223696599
LOC772381	-0.038325272	1.160341589	0.013210849	0.908493901	0.958196054
FBXL13	0.904757024	1.161754417	14.71797824	0.00012485	0.002451562
LOC107054755	0.112520393	1.162754216	0.245781104	0.620061525	0.795058816
CHRDL2	0.096083064	1.163110573	0.267348834	0.605115767	0.785576327
GDPGP1	-0.091455663	1.163387123	0.206030727	0.649896429	0.813173579
LOC107049482	-0.185563538	1.165028114	0.740170766	0.389606181	0.623574527
KRTCAP3	-0.395272946	1.165184178	2.817461354	0.093243772	0.267164596
LOC112530944	-1.020114437	1.166692228	15.30563788	9.14E-05	0.001931576
CALML3	-0.060347472	1.169215921	0.074677079	0.78464485	0.894260355

KRT80	-0.143332986	1.17035579	0.447287679	0.503625815	0.7140723
LOC107054238	-2.001454431	1.170641343	14.82667765	0.000117856	0.002335044
LOC107054268	0.435323904	1.171773546	5.591983789	0.018042851	0.092862713
DIO2	-0.345912851	1.172200315	1.838217014	0.175160068	0.394884083
KCNS3	0.232900334	1.174065247	1.056150337	0.304094849	0.541951709
LOC101749146	-0.066504811	1.17553872	0.115331043	0.734154581	0.864190116
LOC112532705	-0.070557792	1.175948559	0.09000357	0.764172617	0.881657566
LOC112531987	-0.098666958	1.176557399	0.237248133	0.626200762	0.799103476
LOC107053759	1.473785156	1.176864334	28.35526101	1.01E-07	6.84E-06
THEMIS2	-0.876890266	1.17809763	10.2958463	0.0013333	0.014776945
LOC107053208	-0.204649376	1.178593346	0.791863497	0.373537239	0.608208517
LOC112531706	0.028037058	1.178722645	0.023717003	0.877607168	0.944829372
SLC6A20	0.008203352	1.178724525	0.002162925	0.96290595	0.98401549
PCDHAC2	-0.285020283	1.178838146	1.084187253	0.297762724	0.534783412
LOC107052021	-0.780277219	1.178996965	7.37181861	0.00662539	0.046550293
TMEM52B	-0.87340751	1.1802878	4.387082073	0.036212238	0.146477969
HNF4A	-0.487651661	1.180808476	2.732713699	0.098312108	0.275170223
C14H7ORF62	-0.234036225	1.181779777	1.460904618	0.226786241	0.456755952
LOC112531623	0.198233703	1.182559811	0.721135553	0.39577168	0.628551587
LOC112530489	0.15763136	1.183016198	0.243017044	0.622035488	0.796358357
LOC112533095	-0.105225668	1.183112889	0.355952993	0.550762476	0.749363034
SDR42E2L	0.564071425	1.185268012	5.201645547	0.022565516	0.107347665
DOCK2	-0.666619882	1.187831866	5.595718286	0.018004427	0.092756059
PCASP2	0.319221877	1.188118983	1.864336679	0.172125119	0.391150231
LOC107054161	-0.292202402	1.188798023	1.930159012	0.164741049	0.381697263
EVA1C	0.23000063	1.190051133	1.405556294	0.235795477	0.467900217
LOC101750908	0.033798115	1.190150043	0.040985822	0.839565292	0.923898005
LURAP1L	0.421144582	1.190340378	4.541068219	0.033090936	0.138376454
LOC112532341	0.592780457	1.191752482	6.394430131	0.011447897	0.068326134
SLC9A5	-0.608940602	1.19524913	10.99818597	0.000912011	0.011241305
C1QL2	-0.363785444	1.195289117	2.821607739	0.093003205	0.266661045
LOC112531989	0.257931045	1.195826384	1.985181333	0.158845627	0.373901051

LOC112532945	0.029106516	1.196258147	0.021504898	0.88341185	0.947932311
MFAP3L	0.174043886	1.197162413	0.629532757	0.427526753	0.65520655
LOC112529916	-0.936062234	1.19786175	19.47649107	1.02E-05	0.000329247
LOC112531027	-2.299465923	1.198035149	38.89198604	4.48E-10	5.42E-08
SIGIRR	0.120672862	1.19894185	0.310166218	0.577578205	0.767136118
TUBA4B	-0.913047911	1.199109683	18.36169181	1.83E-05	0.000522824
MPO	-0.405362072	1.200133175	1.160177196	0.281428795	0.517246824
MFNG	0.250238931	1.200715223	1.285064797	0.256959442	0.49188781
MTERF2	0.069088347	1.200843757	0.137253766	0.71102655	0.849781439
MAT1A	0.401153943	1.201595384	2.590007034	0.107539858	0.292007981
GPR20	0.593180634	1.202216498	1.770554869	0.183313425	0.40570577
GDF15	-0.733670448	1.202315195	5.93826372	0.014815607	0.081317816
EPHA6	-0.569328658	1.202422282	6.799666953	0.009117488	0.058063544
HACD4	0.159545181	1.20247733	0.816543304	0.366192765	0.60136746
NXPH3	0.247649664	1.202531104	1.808998199	0.178628452	0.399582814
OPN3	0.006929795	1.205415598	0.0009005	0.97606041	0.990018742
ODF2L	-0.436542697	1.207298042	5.315366573	0.021138146	0.103060206
IFI27L2	-0.393527835	1.208218136	3.846106527	0.049861611	0.179259953
LOC112532908	-0.089168766	1.208307375	0.181805202	0.669826446	0.82518486
LOC107054199	0.271212968	1.208405596	2.360361921	0.124453313	0.320131196
LOC112533486	-1.245407717	1.208600879	30.71560213	2.99E-08	2.32E-06
C6H10ORF90	-0.473706389	1.208952771	4.833181563	0.027917068	0.123921054
LOC112532555	-0.673792468	1.209393854	12.64974916	0.000375616	0.005836414
LOC112531897	0.228831256	1.209719728	0.979735461	0.322264125	0.55990457
LOC112531785	-0.092301281	1.210310841	0.233464643	0.628966664	0.800641717
LRRC66	-0.064576351	1.21124203	0.103084128	0.748159488	0.872932311
DKK1	-0.406394482	1.211302907	0.998012531	0.317791896	0.555772733
GABRA2	0.401523518	1.211773143	2.52102701	0.1123374	0.300105908
LOC107054433	-1.166162769	1.211948549	18.56142314	1.65E-05	0.000484278
LOC107052642	-0.424693245	1.21301762	1.693976997	0.193077542	0.417328557
TMEM154	0.254262023	1.214193611	1.963276055	0.161163383	0.376857108
LOC101748700	0.051994322	1.214246038	0.065286455	0.798327567	0.901588892

SLC2A9L	-0.405392582	1.214986079	3.603020648	0.057674687	0.197346012
GLIPR1L	-0.204791069	1.215238498	1.060622953	0.303073147	0.541014171
LOC101751388	0.287282819	1.215323655	0.979433764	0.322338641	0.559907128
MIR130B	0.507155524	1.215481655	6.308953177	0.012012975	0.070547288
LOC107049106	-3.355056209	1.21589275	83.20416553	7.40E-20	6.09E-17
HTRA3	-0.090927681	1.217037014	0.191194542	0.661924265	0.820734216
LOC112532619	-0.764910675	1.219808998	5.278603645	0.021589055	0.104453956
LOC101749901	-0.95285278	1.220270105	7.076495836	0.007810159	0.052306135
LOC107053731	-0.163427746	1.22070611	0.604704435	0.43678868	0.662986744
IKZF3	-0.331584665	1.225505927	2.699150408	0.100401736	0.278837226
VWA1	-0.30252172	1.225747951	2.440717527	0.118222137	0.310434308
LOC101748122	0.311828786	1.226706162	1.602814767	0.205504773	0.432404944
PAK6	-0.857381409	1.226915987	14.07007002	0.000176124	0.003238128
SUSD3	-0.197782881	1.227013209	0.806216684	0.369241159	0.603962551
LOC107053780	-0.156022534	1.227238084	0.471799178	0.492160625	0.704769218
CAMK4	-0.027187459	1.22782025	0.021515425	0.883383522	0.947932311
YPEL4	-0.165710078	1.231912432	1.135610094	0.286581852	0.522903685
LOC101749506	-0.797478132	1.232235585	17.69772638	2.59E-05	0.000697347
HRC8L	0.220641676	1.232295438	1.082451841	0.298149709	0.534894621
LOC107053962	-0.264894218	1.232340671	1.066720335	0.301687421	0.539435949
LOC101748935	-0.608179686	1.233458122	9.542530832	0.002007647	0.020193053
LOC107052593	-0.317536335	1.233714271	2.530008067	0.111699645	0.299578911
GP1BB	-0.099742548	1.233895178	0.222997301	0.636765555	0.805440633
LOC107053656	-0.440210023	1.234470048	2.848917341	0.091435492	0.264002528
RBPJL	0.240868754	1.235781282	0.530618984	0.466346742	0.686431785
LOC112529953	-0.541867318	1.236304477	5.987963843	0.014403819	0.079902002
LOC425238	0.113404076	1.236815592	0.243712175	0.621537749	0.795959747
LOC107053719	0.280112803	1.237472136	1.330086339	0.248789859	0.482992149
LOC107054848	-0.28861124	1.238838814	1.660877906	0.197484832	0.423181784
FAM183A	0.112723288	1.240159408	0.326187427	0.56791274	0.760970118
CA3A	-0.66495828	1.240248386	9.849480001	0.001698798	0.017759667
HIST1H3A	-0.169966495	1.24103559	0.699339885	0.403005591	0.63489551

UCP3	0.9540753	1.244088628	15.72881051	7.31E-05	0.001629942
CD247	-0.268523947	1.244308281	1.558476284	0.211888461	0.439675237
TLX3	-0.213517112	1.246035629	0.502251298	0.478512588	0.694655901
GJB6	-0.228304792	1.246323691	0.789794756	0.374162194	0.608864607
MIR6594	-0.216969556	1.246393865	1.381440107	0.239855997	0.472323431
LOC107054593	-0.634359641	1.24654208	14.52168564	0.000138555	0.00266036
LOC107051751	-0.066785165	1.248196476	0.15348944	0.695222781	0.841135763
ALDH3B1L1	0.286165088	1.249022095	2.24850211	0.133743808	0.336044337
LOC112533501	0.524651687	1.249036944	7.748321803	0.005376248	0.040175366
SNORD83A	0.373824952	1.249105692	2.293163944	0.129944792	0.329670298
ADCY8	1.819165174	1.249477981	43.00156143	5.47E-11	8.65E-09
TNFRSF4	0.338432749	1.249755904	3.103077917	0.078144421	0.238520953
LOC112532749	0.19814093	1.250239534	1.330841703	0.248655533	0.482857453
LOC107051864	-0.628196606	1.250305366	8.895331307	0.002859007	0.025581819
LOC101748329	0.532138747	1.250495916	4.74404335	0.029399896	0.127722486
IMPG2	0.825572049	1.250762382	10.81652516	0.001005981	0.012065175
LOC107053867	-0.233736206	1.251594584	1.634447853	0.20108895	0.427674638
TLDC2	-0.116999603	1.252673997	0.349640949	0.55431645	0.752208246
COL21A1	0.349356626	1.252683472	3.569209573	0.058860322	0.199885747
ARHGEF37	0.084471535	1.253929902	0.17534828	0.675401714	0.828968937
LOC112532422	0.318441217	1.254356645	3.505026015	0.061182885	0.205127214
LOC112531638	-0.009853054	1.256095248	0.00233929	0.961424393	0.983784491
LOC107053789	0.107835436	1.256488389	0.1099725	0.740175446	0.867862831
FGF22	0.192117442	1.256554706	0.83592125	0.360565907	0.597143069
HNF4G	-0.017472762	1.256611468	0.004710111	0.945283938	0.976621285
LOC107052228	-0.217921585	1.257049235	1.255240542	0.262553917	0.497675926
PTCHD3	-0.433270589	1.257417391	3.570437667	0.058816807	0.199860705
LOC107053180	-0.089138848	1.25783153	0.188477681	0.664186662	0.821867312
SOCS1	0.349463231	1.259054983	3.452411884	0.063159542	0.209555471
LOC112533516	-0.447929897	1.259208211	3.647363412	0.056158046	0.193524744
LOC101750035	-1.592212746	1.259312327	63.28693743	1.79E-15	8.17E-13
CCDC181	-0.146680906	1.259991851	0.749407155	0.386663994	0.620979506

SPO11	-0.233347357	1.26065674	1.279483317	0.257995126	0.492541889
LOC112533598	0.1608297	1.261004089	0.091557508	0.762206366	0.880756463
TMEM221	0.215616385	1.26338776	0.856733842	0.35465432	0.591325809
LOC107052277	-0.014764706	1.263599234	0.00422361	0.948182516	0.978142017
LOC101751319	-0.811719295	1.265494381	8.76578744	0.003069335	0.026850565
LOC107052401	-0.455061313	1.266350419	6.292224348	0.012126877	0.071089333
IGSF6	-0.119700027	1.26792783	0.381885078	0.536596097	0.737836448
FGF9	0.120684936	1.269078608	0.17310523	0.677366709	0.830309087
FIGLA	0.412287347	1.269543143	3.058704585	0.080305668	0.242909885
LMOD2	0.731079404	1.271387854	7.645370719	0.00569182	0.041755635
LOC101749696	-0.093249283	1.271617361	0.157617628	0.691359448	0.839044886
TVP23A	-0.152248452	1.272438514	0.786208775	0.375248976	0.609909315
LOC107054055	-1.321984372	1.273984818	28.68819641	8.50E-08	5.83E-06
CEBPD	-0.32626009	1.2741021	1.319988622	0.250594093	0.484892497
TMOD4	0.092748211	1.274684437	0.237155837	0.626267908	0.799103476
THPO	-0.154608938	1.274933145	0.705021815	0.401101419	0.633652443
LOC107053897	0.223236301	1.274985547	1.279765513	0.257942639	0.492541889
LOC107052167	0.032527374	1.275681561	0.035006493	0.851582054	0.931020045
MYPN	-0.513082907	1.276329002	5.549935004	0.018481373	0.09407578
RAB19	0.649630173	1.276400035	11.17244123	0.000830213	0.01050352
HSF5	-0.079262018	1.277243562	0.199556677	0.655078921	0.815935482
LOC112533170	-0.478497722	1.278557072	3.909777137	0.048005963	0.174743055
LOC112533030	-0.01118499	1.278996838	0.003762629	0.95108822	0.979542885
LOC107051666	0.554035225	1.279278902	3.769143927	0.052206346	0.184584319
LOC107054076	0.091647347	1.279395745	0.138984484	0.709292667	0.849591042
LOC107053414	-0.773476227	1.280096935	8.167238649	0.004265379	0.034005242
RND2	0.025637377	1.280266707	0.019839446	0.887986415	0.949435767
CALHM2	0.237936543	1.280874567	1.516500748	0.218149803	0.447286229
VGLL2	0.103323821	1.281918402	0.234794059	0.627991667	0.800186068
TLR4	0.630752479	1.28218721	7.288278515	0.006940595	0.048015912
CLDN11	-0.331063695	1.284157242	2.619439148	0.105561746	0.288733133
LOC769478	-0.388385524	1.286927469	4.887475401	0.027052216	0.121226639

LOC107052236	0.621348352	1.287243759	10.0602683	0.001515012	0.016282355
MOV10L1	0.02903119	1.28771413	0.018054192	0.893113253	0.951332119
LOC101747620	0.24999541	1.288900898	1.859123421	0.172726004	0.392028469
NR1D1	0.270170145	1.289637541	1.798206923	0.17992942	0.401619454
LOC101750312	-0.947707152	1.290513921	12.77179401	0.000351885	0.005535632
OPN4-1	-1.984283704	1.29051804	45.68123474	1.39E-11	2.63E-09
LOC107054849	-0.490876577	1.290811573	3.713041569	0.053988925	0.189018673
LOC107055012	0.108547657	1.291458589	0.323913016	0.569265501	0.762061813
LOC101750811	0.165118264	1.292133923	0.871074565	0.350657995	0.587225454
LOC107050011	-0.209176827	1.292405072	1.126194658	0.288588483	0.525165885
LOC107054969	-0.222709838	1.293212014	0.756646469	0.384380043	0.619003094
MADCAM1	-0.411898779	1.293733031	5.060261897	0.024480538	0.113440512
LOC107053963	-0.140789147	1.293836747	0.292276392	0.588765444	0.774802893
KCNE4	0.843049347	1.295696399	12.82155676	0.000342649	0.005442356
LOC112532711	0.300289077	1.297182561	1.895309162	0.168604314	0.386889414
FAM155A	-0.360623534	1.299622605	3.226378629	0.072460759	0.227459327
LOC422224	0.082266673	1.299820478	0.192170089	0.661116576	0.820350897
LOC107049461	-0.265509146	1.299885938	2.040958284	0.153113163	0.365618501
LOC112532485	0.155429567	1.300668566	0.874596594	0.349685912	0.586253865
LOC107052027	-0.333490632	1.300676794	2.927020715	0.087108106	0.255820788
BHLHA9	0.247471559	1.301974788	0.190289723	0.662675593	0.821016009
BEST4	-0.867442552	1.302005889	9.552243892	0.001997051	0.020148057
FLRT1	0.090383612	1.302394302	0.221774358	0.63769126	0.805931282
LOC112533301	-0.398128376	1.304461124	3.772653561	0.052096923	0.184395539
LOC101752086	0.065762951	1.305459658	0.159603028	0.689522262	0.83825922
MIR1564	0.02796858	1.306016885	0.021088838	0.884537203	0.948459513
ARHGAP20	-0.391585817	1.306885886	4.189794965	0.040668016	0.158073742
LOC112532590	-0.686570175	1.307706271	6.27661818	0.012234135	0.071641527
LTC4SL	-0.072104716	1.308253133	0.173490539	0.677028105	0.830079538
LOC107056831	0.5271591	1.309123318	7.318896213	0.006823331	0.047434688
LOC112530592	-0.63101529	1.309280518	11.24445432	0.000798613	0.010171186
LOC101750579	-1.143716625	1.309463537	18.56373607	1.64E-05	0.000484278

LOC112531906	-0.875862057	1.31038855	4.288580158	0.038369195	0.151618459
LOC101747310	0.471224607	1.31047405	7.391352253	0.00655382	0.046244899
PIK3C2G	-0.071991125	1.315745453	0.135920092	0.712371162	0.850434818
PAPPA2	-0.146421973	1.316220362	0.183381542	0.668483159	0.824270542
KCNK10	0.060537432	1.318935411	0.071785552	0.78875454	0.896151071
VSTM5	0.088719953	1.3192123	0.265941906	0.606067059	0.786253032
LOC101749220	-0.842958942	1.320291235	12.92338615	0.000324502	0.005214537
MPND	0.239012542	1.320858705	1.962049867	0.161294258	0.377002418
LYPD6	0.669101735	1.321561428	8.876875569	0.002888054	0.025771652
DRC7	0.252528266	1.321858572	1.329707003	0.248857349	0.483065669
LRRTM4	-0.223947484	1.322158175	0.713936487	0.398140083	0.630434162
LOC101751890	-0.160043555	1.322242259	0.243173334	0.621923502	0.796276939
LOC107050009	1.405344846	1.322302389	18.30413419	1.88E-05	0.000536992
LOC101749636	-0.285982622	1.32306299	1.672073619	0.19598102	0.420708925
FAM228	0.109374096	1.323391025	0.313622923	0.575465478	0.765607847
CARNS1	0.235553783	1.323887302	1.732892986	0.188041858	0.411302508
GABRB2	-0.373134078	1.324635754	3.848564425	0.049788588	0.179153995
LOC112530977	0.887627381	1.32634243	15.07318434	0.000103422	0.002124607
LOC107050543	0.073874756	1.32663077	0.124801666	0.72388394	0.857408332
LOC100859625	-1.427274285	1.32690718	26.13921298	3.18E-07	1.83E-05
LOC107054645	-0.04109998	1.327126656	0.028198023	0.866644116	0.93840635
OCM2	-0.583935404	1.328024902	4.718888312	0.029833024	0.128744405
AGR2	-0.066608336	1.32901038	0.115792826	0.73364308	0.863897015
LOC112531874	-2.963666595	1.329191396	32.21810928	1.38E-08	1.17E-06
SYN2	-0.10746565	1.330577678	0.258142147	0.611399407	0.788982376
PHEX	0.020866072	1.330778993	0.010201891	0.919546959	0.962786027
LOC112533255	0.102585702	1.332783715	0.332717303	0.564063406	0.758120015
LOC112533508	-0.066183444	1.333224932	0.062266619	0.802948671	0.904409637
CDH10	-1.008959746	1.333664289	9.826292368	0.001720348	0.017899277
LOC112530672	0.224412861	1.336731793	1.271630663	0.259460991	0.493975542
LOC101749678	-0.122892428	1.337660702	0.138900645	0.709376375	0.849591042
LCK	0.026707021	1.338155395	0.028447287	0.866061539	0.938134876

AADACL4L3	-0.025261563	1.338252348	0.011055529	0.916260532	0.961731649
LOC101749148	-0.616725125	1.33839424	10.01858981	0.001549681	0.016569199
PLA2G4EL5	-0.566954404	1.33880588	6.970159215	0.008288012	0.054576529
CALCR	-0.734895997	1.33885923	14.53006323	0.000137941	0.002651651
LOC112531252	-1.210773372	1.33954903	11.82052574	0.000585813	0.00812094
LOC112530085	-0.045133213	1.342915148	0.058257723	0.809271178	0.908188905
SLC2A2	1.301679598	1.343072876	8.41144525	0.003728661	0.030987435
LOC107055311	-0.321956524	1.343570246	3.103689927	0.078115054	0.238475551
STK32B	-0.717498962	1.343709463	8.314602685	0.003932761	0.032115918
LOC101748199	0.082727443	1.345014585	0.238173294	0.625528582	0.798796471
DM5L	-0.136056893	1.346417924	0.3809725	0.53708323	0.738321182
GDF6	-0.154297824	1.346501134	0.156983224	0.691949314	0.839182338
LOC101749277	-0.277889497	1.347229759	1.057507631	0.303784326	0.54181347
LYVE1	0.584481407	1.348261056	6.63789345	0.009983191	0.061919869
LOC112532586	-0.284686112	1.351962136	2.29546449	0.12975239	0.32942409
NCF1	-0.088321592	1.352329634	0.233597308	0.628869213	0.800641717
LOC112532320	0.125007493	1.352795703	0.33946748	0.560136764	0.755434018
LOC101749542	-0.100521963	1.355083627	0.447940671	0.503314523	0.713873264
LOC107052217	0.094841938	1.355625106	0.187999812	0.664586596	0.822176712
LOC107049730	-1.34147813	1.355706491	20.38098463	6.35E-06	0.000225041
NECAB1	0.110278532	1.356051259	0.3837996	0.535576737	0.737235671
LOC101749752	-0.323369372	1.356614032	1.805480571	0.17905133	0.400196905
LOC107053122	-1.464682919	1.358962033	15.44990351	8.47E-05	0.001822338
UPP2	0.277251677	1.359296248	1.902374198	0.167812811	0.385718649
LRRTM3	-0.267239247	1.360081666	1.014904545	0.313730722	0.551695247
ARHGAP8	0.085527163	1.360394643	0.199425526	0.655184945	0.816005773
LOC101747954	-0.75815626	1.360777515	10.25344275	0.001364298	0.015006361
LOC101749936	0.568002544	1.361359311	8.999863384	0.002699998	0.024478493
EFCAB11	-0.027588472	1.361843656	0.030565208	0.861213936	0.935139468
LOC421690	0.093043141	1.362268466	0.15909274	0.689993186	0.83840108
KCNQ1	0.092776742	1.362440375	0.197300089	0.65690902	0.816916409
P2RY6	-0.280510203	1.363145169	1.969352344	0.160516624	0.376039442

LOC101750125	-0.447246728	1.364222462	2.695298843	0.100644617	0.279276083
IFITM10	-0.250862752	1.364861299	1.529867918	0.216132269	0.444994092
LOC107054667	-0.208207599	1.364863731	1.152612662	0.283002895	0.518749319
LOC101750545	-0.097529435	1.36531141	0.244961654	0.620645291	0.795321101
GSG1L	0.166004778	1.36548723	0.596996109	0.439726447	0.66486251
MILR1	0.545524851	1.366249212	7.940208923	0.004834825	0.037385831
LOC107055034	-0.232502293	1.367786961	1.124484834	0.288954799	0.525444934
LOC107053700	-0.012782655	1.367805339	0.002741276	0.958244082	0.982056949
HSD11B2	0.006485658	1.367921332	0.001111259	0.973407008	0.98946209
SLC38A11	0.279998858	1.368611426	1.08311931	0.298000792	0.534872864
C11orf63	0.194002803	1.369082444	0.87514837	0.349533954	0.586139207
LOC101748294	-0.453392031	1.369873048	4.70940652	0.029998006	0.12932072
EXOC3L	-0.422820527	1.370368967	4.742069519	0.029433644	0.127758008
STK31	0.008558386	1.371635661	0.001229947	0.972023439	0.98878868
LOC107053443	-0.082347629	1.372878559	0.185542755	0.666652529	0.823679784
LOC107055878	0.890551704	1.373858908	12.61669847	0.000382316	0.005912603
LOC107050919	0.050394028	1.375041604	0.072158732	0.788219215	0.895666541
RHEB	-0.255428535	1.375568785	2.189151768	0.138985837	0.344362628
LOC112531956	-0.383916797	1.377116157	3.808373922	0.050996936	0.181989717
MPZ	0.266744091	1.378065212	0.683998957	0.40821309	0.638754887
PDZK1	-0.025008606	1.379897569	0.022418107	0.880980183	0.94668118
OVOL2	-0.251068954	1.380503885	2.130106656	0.144430706	0.353188775
LOC112532660	2.152897213	1.380819394	89.29725706	3.40E-21	3.29E-18
C18H17orf58	-0.2071958	1.381557809	0.815977513	0.366358878	0.601466431
ASIP	-0.853542508	1.382368759	7.368537162	0.006637491	0.046615412
SLC3A1	0.530767331	1.382652974	7.825839657	0.005150451	0.038947922
TMEM140	0.631651216	1.382721983	4.595674318	0.032052732	0.135307261
ITGB1BP2	0.354764109	1.382922951	3.117396722	0.077460443	0.237687384
SLC35E2B	-0.155033143	1.383730456	0.66222137	0.41577681	0.646105148
DBH	-0.331997772	1.3846923	0.603081737	0.437404614	0.66346111
KCNC1	-0.047873697	1.385413655	0.058671261	0.808608527	0.907749244
TTC29	-0.342401775	1.386739989	2.057253627	0.151482978	0.363239886

LOC107050915	1.215501058	1.38717443	10.98934431	0.000916372	0.011278164
NPY	0.588788179	1.388925535	5.324191835	0.02103136	0.102661235
LOC101747923	-0.4286973	1.389394354	3.882512052	0.048791503	0.176579455
LOC107053851	-0.411428034	1.390016342	4.846658254	0.027699747	0.123267332
LOC107052699	-0.376607428	1.391007621	2.593753726	0.107285801	0.291703215
LOC770492	-0.309114463	1.391161606	3.070793029	0.079710579	0.241821086
SUGCT	-0.158672596	1.391291215	0.873805852	0.349903838	0.58649971
LOC107056277	0.238939473	1.391446641	1.424795876	0.232615636	0.464074962
FKBP6	-0.199404298	1.391729476	1.276532088	0.258544832	0.492972794
LOC101749934	0.033668499	1.392400031	0.036423553	0.848643463	0.929382324
PLCD4	-0.419199595	1.393932285	4.095562139	0.04299593	0.163356737
LOC101748607	0.389119426	1.394123496	2.591650505	0.107428336	0.291842445
SLC6A11	0.09464558	1.394608099	0.266755193	0.605516769	0.785910903
PARD6A	0.136179092	1.395279565	0.470948349	0.492551207	0.705092716
LOC107053682	0.21550154	1.395816031	0.631609116	0.426765693	0.654800765
LOC112532562	-0.764595058	1.395818261	12.96617113	0.00031717	0.005141904
MIR1778	0.056489051	1.396341933	0.119251075	0.729848183	0.861397607
LOC112533013	-0.357948575	1.39645155	2.038649784	0.153345708	0.365908299
LOC112530202	-0.354818027	1.39670225	3.785649231	0.051693853	0.183402834
C26H1orf106	-0.158749626	1.398036928	0.627021545	0.42844994	0.656010399
MIR1653	0.188735154	1.39814211	1.009432058	0.315038939	0.552546231
TC2N	-0.10815317	1.398690663	0.225285166	0.635042061	0.804373576
PRIMA1	0.068102884	1.399447494	0.084051117	0.771880725	0.886714419
MYH1F	-2.019282437	1.39987322	20.20468447	6.96E-06	0.000242067
SATB2	0.073983896	1.401389056	0.200431186	0.65437303	0.815640639
LOC107055146	0.006044906	1.402142188	0.001250619	0.971789422	0.988743849
LOC423462	0.614441795	1.402306614	6.357173426	0.011690764	0.069288863
FBXO48	-0.191308374	1.403806106	1.210450112	0.271242997	0.506356841
LOC107052840	0.027777418	1.403979366	0.011391516	0.915002352	0.961017703
CBLN2	0.139536159	1.40445481	0.205048703	0.650676174	0.813613209
LOC107053516	-0.213410264	1.404506499	1.083190136	0.297984996	0.534872864
ATOH1	-0.725549099	1.405714581	3.131509156	0.076792622	0.236677765

ALPI	1.091190042	1.406490645	22.69774516	1.90E-06	8.04E-05
LOC112530987	-1.118108383	1.406547149	33.46201438	7.27E-09	6.53E-07
LOC107053200	-0.346286647	1.406688031	2.648078001	0.103675178	0.28504178
DLGAP2	-0.735983048	1.408374188	12.57724065	0.000390473	0.006006004
CRY4	0.113931884	1.41003596	0.368289501	0.543938116	0.74417397
FAM69A	0.026338839	1.410693113	0.020579987	0.885929058	0.948791653
TSNAXIP1	0.371597536	1.411459018	3.86108881	0.049418249	0.178211107
LOC768418	-0.749531181	1.417280646	10.17181619	0.001426037	0.015602024
LOC101747295	-0.203961993	1.41765116	1.331232828	0.248586015	0.482857453
LOC112530505	0.278899098	1.418294297	2.87139552	0.090166655	0.262036199
LOC112532476	-0.523792827	1.419141912	9.490995476	0.002064828	0.020642004
CAMKV	-0.055248754	1.420220721	0.092668568	0.760811647	0.880207468
LOC112530433	-2.451729506	1.421717015	38.46352039	5.58E-10	6.60E-08
LOC107057413	-3.434611812	1.422895315	58.46310716	2.07E-14	7.41E-12
LOC107055278	1.04724097	1.423808338	24.98954994	5.76E-07	3.03E-05
CAPS2	-0.194608045	1.424124376	1.06291947	0.302550265	0.540373885
FOXI2	0.040295448	1.424628352	0.019680037	0.888434378	0.949552787
CHMP4C	-0.099604155	1.425084871	0.313571131	0.57549702	0.765607847
LOC421415	-0.110377804	1.425779419	0.303203061	0.581881322	0.770465751
RASD1	-0.103238584	1.426089207	0.255961639	0.612908237	0.790261455
LOC112533226	-0.069698119	1.426854796	0.183855108	0.668080942	0.824166458
LOC112532293	-0.744630522	1.427593078	15.89888427	6.68E-05	0.001514451
APOH	0.37357089	1.428683954	1.563185532	0.211199406	0.438848883
CNP1	-0.037256658	1.428834711	0.041664552	0.838260535	0.923661189
LOC107052408	-1.15639252	1.429137348	15.82529211	6.95E-05	0.001565785
EFCAB10	-0.358312657	1.429928002	4.547538663	0.032966103	0.137994716
LOC107054141	-0.185704755	1.430265334	1.159085748	0.281655229	0.517377633
PLA1A	0.241822807	1.430321502	1.654420004	0.198358411	0.4239639
LOC112531131	0.525528924	1.432532323	6.806468242	0.009082824	0.057951866
LOC107053686	-0.431920713	1.432732717	5.687844861	0.017082822	0.08992253
AK7	0.119180481	1.43392616	0.497780579	0.480476943	0.696466157
GABRB3	0.530260762	1.436478605	6.540019988	0.010547416	0.06430446

LOC107050162	-0.588804971	1.437643874	3.356933037	0.066922596	0.217030217
LOC107054178	0.126926419	1.437773171	0.63903717	0.424059667	0.65309226
LOC112533334	-0.143921334	1.438128657	0.488225287	0.484720033	0.699389701
TRPC7	-0.160611463	1.439098197	0.514170866	0.473338873	0.691476487
HS3ST1	0.105901159	1.439173267	0.333671892	0.563504903	0.757622344
LOC107052067	-0.014240083	1.440168209	0.005539644	0.940669206	0.974411302
ABCC2	0.341568744	1.440448183	3.793947146	0.051438213	0.182881114
LOC417937	0.046101358	1.443529374	0.066979378	0.795786669	0.900472399
LOC112530435	-0.108573834	1.444004181	0.400494	0.526834245	0.730729536
LOC112532162	0.043242749	1.44478155	0.034763532	0.852092034	0.931269647
MAP3K19	0.055666065	1.446541111	0.115545819	0.733916539	0.86403353
IL11	0.021049084	1.447120176	0.01281286	0.909876827	0.95876173
LOC112530943	0.226587135	1.44805108	1.318802762	0.250807031	0.485133383
JAKMIP3	-0.08197853	1.44822161	0.185770977	0.666459955	0.823590831
NME1	0.448783567	1.448334275	5.585324201	0.01811158	0.092928514
BG1	-0.154445742	1.448644891	0.433661109	0.510197944	0.718341124
KCNH7	-0.089851422	1.449327299	0.154586922	0.694189912	0.840413111
RAB44	-0.170275008	1.450201597	0.909762528	0.340177435	0.577342947
RYR2	-0.258422227	1.45043515	1.310177359	0.252362546	0.486934484
GJD2	-0.186944416	1.451701496	0.494372047	0.481983489	0.697181562
ZNF708L	0.197509447	1.451701687	0.273557014	0.600955616	0.782591357
LOC100857180	0.194259471	1.451849045	0.9961882	0.318234613	0.556015137
ISX	-0.415643792	1.452699688	2.500159661	0.113834757	0.302755928
LOC107051278	-2.788476999	1.453436341	113.1360352	2.01E-26	2.55E-23
IL17RE	0.216338989	1.453436698	1.433969596	0.231117689	0.462544585
LOC112530121	0.65191438	1.453774813	6.823764796	0.008995275	0.057549473
LOC112533463	-0.742041402	1.454036786	4.170612733	0.041130922	0.159204716
LOC429054	0.113385615	1.454131737	0.201225195	0.653733716	0.815062002
PLS1	0.21397137	1.455656142	1.0183219	0.31291739	0.551054758
FAM124B	0.007983888	1.455831351	0.002034787	0.964020735	0.984735636
LOC107049800	0.30642086	1.456233027	2.231109117	0.135256725	0.33839038
PNOC	-0.608557823	1.456721707	9.325528343	0.002259832	0.021835307

LOC101748231	-0.287552123	1.457751053	1.282553246	0.257424842	0.492320522
SVOPL	-0.195122418	1.458902538	1.144968947	0.284604807	0.520525964
LOC107054529	-0.361294095	1.461861669	2.48653264	0.114824445	0.304404099
LOC107052158	-0.228867421	1.462680102	1.756991131	0.185000258	0.407629787
AWAT1	-0.910751345	1.462886472	21.64266191	3.28E-06	0.000129303
LOC112530030	-0.288775915	1.463269479	2.078155654	0.149420624	0.360578284
FAS	-0.576593344	1.465080784	4.906718859	0.026752414	0.120459391
DLX3	-0.959859915	1.465461845	7.915166715	0.004902206	0.037747218
GLIS1	0.531465349	1.466330762	6.274946869	0.012245679	0.071658127
FIBCD1	0.603273964	1.467005042	8.225061522	0.00413158	0.03326084
LOC112531854	-0.972299941	1.467291047	19.63329546	9.38E-06	0.000307531
LOC107051744	-0.377700108	1.467798206	1.587104598	0.207740294	0.435301993
LOC112532955	-0.099938064	1.470355541	0.257960289	0.611524939	0.789041236
LOC107050939	1.938150273	1.470574434	11.92164327	0.000554855	0.007855776
EFCAB6	-0.606612709	1.470713613	4.275180688	0.038672852	0.152495035
LOC107049700	-0.485880904	1.471037112	5.958421804	0.014647147	0.08076448
DCLK3	-0.252537836	1.471846295	1.514015549	0.21852737	0.447748459
FER1L4	-0.08435426	1.472145735	0.219250164	0.63961185	0.807117561
LOC107049828	0.072337881	1.472219074	0.165096464	0.684507021	0.835303382
LOC112529917	-0.365428777	1.472297436	3.138863137	0.076447077	0.236010628
SCN5A	-0.036800764	1.47391198	0.033255264	0.855300011	0.932606301
CLIC3	-0.340831844	1.474637211	2.748138211	0.097367745	0.273597379
CASC1	0.053357305	1.478561678	0.080556187	0.776545094	0.889376916
EGFL6	0.074000009	1.478780021	0.172277676	0.678095448	0.830532564
LOC101750098	-0.562781733	1.479978955	6.811669414	0.009056406	0.057805724
WNT3A	0.028989012	1.480014094	0.034328245	0.853010342	0.931405785
LOC425462	-0.30604569	1.480192894	3.567921066	0.058906015	0.199896572
CTXN1	-0.200176936	1.480562462	0.993171635	0.318968433	0.556706148
C5H11ORF96	0.276156795	1.480769124	2.708957154	0.099786204	0.277553164
LOC107055362	-0.49596199	1.481331658	4.211553694	0.040149547	0.156992849
LOC100857883	0.041253134	1.483030639	0.04806123	0.826471887	0.917837275
UBAP1L	-0.251835285	1.483333919	1.848222201	0.173990305	0.393551431

LOC107054402	-0.213494542	1.483795811	0.930837731	0.334645336	0.572087272
MOGL4	2.330178329	1.484221138	59.72453356	1.09E-14	4.08E-12
MYO1F	-0.206799337	1.484825205	0.809616408	0.368233684	0.602973955
LOC112531198	-0.059678151	1.484899724	0.034405806	0.852846274	0.931394748
LOC112531259	-0.649419098	1.486417877	6.432656614	0.011204087	0.067236783
CCDC125	0.352601315	1.487094395	3.906685137	0.048094372	0.174854816
APOA5	0.981170014	1.487801046	28.78211825	8.10E-08	5.59E-06
LOC107054907	0.074552622	1.490163338	0.228077505	0.632953008	0.803583192
LOC100857335	-0.2303435	1.491091169	1.632719776	0.201327278	0.427960257
LOC107054707	-1.317311548	1.491845555	31.7418924	1.76E-08	1.47E-06
CECR6	-0.277630832	1.492007218	1.476482407	0.224325694	0.453917322
LOC107049256	0.05285329	1.492147047	0.050587726	0.822043724	0.916392801
MIR6582	0.134379288	1.493335033	0.630518571	0.427165163	0.654896371
CCR7	-0.789254061	1.494009884	9.295816602	0.002296777	0.022071093
LOC101751439	-0.499563929	1.494359471	4.288189707	0.038378008	0.151618459
LOC112531211	0.973821506	1.495395002	15.03944022	0.000105288	0.002147351
ADARB2	-0.157408051	1.495789391	0.589261427	0.442704862	0.667447588
DRD3	0.158030749	1.496119225	0.889250653	0.345680455	0.582327179
LOC107049500	0.956138824	1.496407176	5.10207359	0.023897259	0.111825554
LRAT	0.113933508	1.496630613	0.157347757	0.691610204	0.839091293
IL1RAPL2	-0.032664399	1.497433717	0.019327573	0.889431467	0.950116515
LOC112532935	0.886078798	1.497436659	22.53652347	2.06E-06	8.70E-05
MTHFD2L	-0.167015021	1.497562652	0.830048134	0.362258611	0.598670829
DNAJB13	-0.107548198	1.497900832	0.418430062	0.517721454	0.723314877
SLC5A9	-0.043430566	1.49876937	0.043376776	0.835017436	0.921947852
GLP1R	-0.117583076	1.499144045	0.469252507	0.493331251	0.705551458
LOC423605	-0.153383555	1.499367085	0.525789949	0.468382251	0.687777078
SLITRK5	0.21296986	1.499850341	1.123119076	0.289247826	0.525803467
SULT1E1	0.687566959	1.503629006	12.06868688	0.000512758	0.007388296
LOC112533362	-0.631270338	1.504176805	8.828406333	0.002965773	0.026245656
HRH2L	-0.037254623	1.504361665	0.049691608	0.82360077	0.91690482
PTGDR	-0.363582977	1.504717458	3.22788722	0.072394031	0.227382361

RDH8	-0.226223783	1.505214137	1.309860416	0.252419929	0.486936687
LOC112532596	-0.196519713	1.505332598	1.248639059	0.263812569	0.498861977
LOC112533187	-1.069228627	1.50605054	16.190176	5.73E-05	0.00133529
BHLHE41	-0.593322393	1.507145315	7.15782189	0.007463778	0.050583386
LOC107051614	0.116597859	1.50792225	0.23808811	0.625590405	0.798796471
RBM47	-0.174533213	1.508023546	0.725206929	0.394441235	0.627445382
LOC101750804	-0.416886135	1.508956915	5.401721364	0.020116901	0.099346821
LOC107050215	0.200762481	1.509436228	1.173379605	0.278707909	0.514181965
LCAT	1.007329115	1.510014516	21.26947277	3.99E-06	0.000152701
CPNE4	0.459819629	1.510605607	5.307954833	0.021228262	0.103346466
NMUR1	0.079608277	1.51060591	0.109704879	0.740480378	0.867911148
SLC18A2	0.665268347	1.511137667	10.47368208	0.001210871	0.013760282
GPR158	-0.049987863	1.51167173	0.064194013	0.799985882	0.902431458
DSCAM	-0.753212404	1.511722328	8.465967746	0.003618527	0.030345014
LEXM	-0.092156758	1.51236563	0.132125122	0.716238736	0.853085451
LOC418421	-0.490180397	1.513344194	4.967127597	0.025833515	0.117484916
PLPPR4	-0.293824306	1.514528791	1.872123093	0.171232117	0.389828743
ZNF750	0.435311505	1.515445983	3.217642267	0.072848487	0.228545635
LOC107054591	0.123734386	1.515448085	0.421645524	0.5161171	0.722414672
LOC112532451	-0.018954543	1.51553735	0.010435633	0.918633688	0.962372833
GPD1L2	-0.446745913	1.516489562	4.462749885	0.034641687	0.142329329
BFSP2	1.529617375	1.516716642	4.984929773	0.025569023	0.116765178
IYD	-0.17666438	1.516828159	0.262006532	0.60874498	0.787473436
LOC100857928	0.646862025	1.516830482	11.92288273	0.000554486	0.007855776
SLC7A11	-0.39289026	1.518152746	4.680625514	0.030504639	0.130785262
GRIA4	-0.463831305	1.518172306	2.896973716	0.088746022	0.259147422
SH2D4A	-0.032823672	1.518262266	0.038925804	0.843595727	0.926227243
PPP1R1C	0.141332954	1.518351403	0.341907712	0.558730119	0.754264499
LOC112531963	0.110295547	1.518423482	0.326554127	0.567695223	0.760826266
TMEM116	0.438896694	1.519567164	7.271061114	0.007007439	0.048286186
LOC107049645	1.005645418	1.520531704	18.61440756	1.60E-05	0.000474398
WDR31	0.385885042	1.52172616	2.921815697	0.087389479	0.256233851

LOC112533558	0.303765161	1.522824563	1.212558657	0.270825978	0.505899564
RIPK4	-0.1254716	1.523127716	0.473572816	0.491348082	0.704487482
LOC107052318	0.709946606	1.52420242	8.002971525	0.004670065	0.036385377
LOC112530017	0.774351904	1.524501468	7.609830881	0.005805093	0.042341668
LOC101748166	-0.225959357	1.52621495	1.228654172	0.267668929	0.503053957
REM1	-0.215334588	1.526690939	1.284021657	0.257152613	0.492030236
NAAA	0.09409219	1.526890406	0.28331541	0.594536368	0.778227343
LOC112533410	0.002771368	1.526891886	0.000117494	0.991351533	0.995986157
ENPEP	-0.055015034	1.527831242	0.04777068	0.826988883	0.917915823
FUT4	-0.257727304	1.527871097	1.929285522	0.164836634	0.381757467
TMX1	0.301089995	1.528118682	3.001539564	0.083185434	0.2482438
LOC107053677	-0.375501221	1.528895448	4.41777038	0.035566481	0.144576691
LOC112533023	0.409731047	1.528998877	3.393790695	0.065442335	0.214341882
LOC112532782	-2.251468291	1.529337472	84.13452596	4.62E-20	4.00E-17
ALB	0.180305754	1.52976496	0.683736912	0.408302895	0.638773924
LOC101750503	1.267594147	1.530477236	40.86040137	1.64E-10	2.36E-08
LOC112532842	0.215790618	1.533054663	1.624462909	0.20247062	0.429146995
MC5R	0.006184608	1.534254464	0.001249196	0.97180546	0.988743849
RXFP1	0.205972432	1.534766635	1.386385969	0.239016372	0.471810749
LOC107052876	0.272891736	1.534849374	1.935287418	0.164181134	0.381097553
CLYBL	0.273344754	1.535436202	1.844996645	0.174366437	0.393796284
B3GNT4	-0.088813339	1.535866421	0.251806879	0.615805662	0.792054659
GEMIN7	-0.056880288	1.536119008	0.103439688	0.747740281	0.872702382
MYLK4	0.268384203	1.536639535	1.556804331	0.212133742	0.440017739
CISH	0.280188814	1.538587433	1.270123593	0.259743495	0.494318997
LOC107053039	-0.138255708	1.538613534	0.209992691	0.646773133	0.811526992
LOC768829	-0.144948146	1.540509041	0.684660458	0.407986517	0.638521793
LOC112533522	-0.277254441	1.54072033	1.863223028	0.172253277	0.391322595
DUSP18	-0.722633176	1.540976569	11.15328095	0.000838832	0.010580014
OSGIN1	0.539115969	1.543338308	2.99109468	0.083723553	0.24935223
PPP1R3C	-0.016284863	1.543516007	0.008653849	0.925882854	0.967012404
MYOM2	0.000182642	1.543677779	3.60E-05	0.995209886	0.99763501

LOC107055376	0.089580165	1.544948493	0.190073757	0.662855238	0.821016009
GLIS3	-0.135777374	1.545066265	0.443648204	0.505366858	0.715440088
ITIH6	-0.833300025	1.546801511	12.64175724	0.000377225	0.005855888
LOC101750470	-0.183819496	1.548650807	0.409351601	0.522298791	0.72710885
BGLAP	-0.388456162	1.550145416	4.367244494	0.03663619	0.147395721
CNRIP1	-0.12866575	1.552659446	0.346449795	0.556129728	0.752930284
EDAR	-0.586685436	1.553103581	2.782912295	0.095274779	0.270386182
RGS6	0.244608823	1.55330424	2.574983757	0.108565216	0.293970224
CCDC65	0.346505062	1.553519576	2.727459533	0.098636071	0.27564743
LRTOMT	0.046885133	1.556370877	0.03665239	0.848174508	0.92927036
LOC107052929	-0.02595466	1.557240035	0.022349439	0.88116125	0.94681392
KLHDC8B	-0.403275173	1.560819018	4.423126818	0.035455009	0.144230452
RASD2	-0.46483172	1.561027147	3.343444656	0.067473217	0.218128052
CARD10	-0.30177708	1.561257712	3.155993257	0.075648641	0.23429294
PLA2G4B	0.381010313	1.561437088	3.119443905	0.077363181	0.237684649
LOC107050392	-0.671722117	1.561693542	4.987764127	0.025527172	0.116705787
MIR1452	-0.154973801	1.564163162	0.515578054	0.472734078	0.691022409
MATK	0.618675167	1.564240616	10.95736732	0.000932322	0.011430879
BIK	0.006215225	1.565143987	0.001097799	0.973568491	0.989565107
DOCK2L	0.161198628	1.565869843	0.784703888	0.375706373	0.610170585
MDGA1	0.154458889	1.566172011	0.580483744	0.446122771	0.670528882
PCDHB3	0.056424136	1.566381169	0.048366356	0.825930717	0.917793756
PRDM6	0.150770296	1.566583034	0.567764145	0.451148756	0.674632204
LOC112532898	-0.200807765	1.568033914	0.768191691	0.380777049	0.615429363
CLDN7	0.189958465	1.568477153	1.306333164	0.253059631	0.487650887
C5NT1AL	0.385464813	1.569423793	4.304588124	0.038009698	0.150601874
LOC112530290	-0.109276656	1.569456585	0.282364684	0.595155492	0.778666106
M1AP	-0.183059093	1.570356186	0.658091231	0.41723462	0.647270263
LOC107054535	-0.436045022	1.570430528	2.886251454	0.089338568	0.260373032
ADGRD1	-0.184091118	1.570479936	1.042712052	0.307191558	0.545216422
SYT6	-0.138955961	1.57059772	0.415213211	0.519335275	0.724884379
PPP1R3G	0.04272191	1.570721465	0.035749814	0.850033085	0.930291912

LOC107050638	-0.560770343	1.571177606	7.882714561	0.004990951	0.038241869
LOC107051588	-0.136110451	1.572848465	0.638323955	0.42431837	0.65312259
NYX	-0.013528009	1.573053999	0.003884194	0.95030537	0.97898171
LOC771161	0.504593224	1.57310953	6.570962577	0.010365587	0.063572769
TCF24	0.252081627	1.573669378	1.887770893	0.169453557	0.38797249
LOC107053868	-0.193141353	1.57380538	1.540468794	0.21454804	0.443627544
LOC770936	-0.000591822	1.574356307	9.82E-06	0.997499416	0.998889311
AK8	0.025927015	1.574849842	0.016609586	0.89745404	0.953133605
GNG4	0.220819552	1.576095418	2.283815329	0.130729928	0.330972483
GBP1	0.239587108	1.576641242	2.033871933	0.153828275	0.366528275
ALDOB	-0.58623241	1.577023818	3.349316063	0.067232943	0.21763368
PRPS2	-0.506143217	1.577141288	2.731941905	0.098359623	0.275207638
LOC107054649	0.72843448	1.578974659	9.77789522	0.001766224	0.018315727
LOC112530152	-0.220444866	1.580793542	1.625418124	0.202337959	0.429146995
LOC107052101	-0.587472707	1.581206933	6.948378935	0.008389522	0.055043695
GP1BA	0.800146607	1.581806853	14.60382447	0.000132645	0.002569216
CPSF1	0.286068359	1.582546467	2.387398093	0.122317062	0.316865122
LOC107052872	0.131652746	1.582694166	0.554349081	0.456546087	0.6790102
LOC112532210	0.962691521	1.583218911	4.625347585	0.031502878	0.133637498
LOC112530549	-0.027642766	1.583486433	0.017347643	0.895213319	0.952028382
RAC2	0.365489057	1.584473477	4.835972834	0.027871911	0.123754456
PDK4	-0.605314546	1.585096898	10.24136627	0.001373258	0.015094834
LOC107049411	0.035712129	1.591640079	0.034372103	0.852917544	0.931394748
LOC112531951	0.012638264	1.592456409	0.005822982	0.939173689	0.973732156
LOC101748577	0.091556133	1.592462811	0.167618278	0.682237252	0.83346248
ERICH5	-0.049395464	1.594986627	0.060752671	0.805310245	0.905731091
LRRC56	0.374044966	1.595433939	4.03950205	0.044446935	0.166522158
GJA3	-0.275681668	1.596443847	2.049628759	0.152243309	0.364334445
BPIFB3	0.109578781	1.596616168	0.278260334	0.597843729	0.780841953
SOX17	-0.44307649	1.598885558	4.336827554	0.037296356	0.148899141
FOS	0.137895671	1.599675321	0.586581018	0.443744271	0.668664101
LOC107052453	0.326431637	1.600618294	3.070870378	0.079706786	0.241821086

LOC101749691	-0.136048447	1.600972151	0.441835695	0.506237772	0.715893996
RSPH3	0.073040908	1.601409628	0.160031056	0.689127926	0.83810791
SLC24A2	0.203416614	1.603117876	1.013441798	0.3140797	0.551873406
C12orf40	-0.436273903	1.603226586	5.410470347	0.020016322	0.099088021
LOC107055385	0.39617243	1.603302168	3.774593857	0.052036532	0.184261059
LOC107052121	-0.027159115	1.603327169	0.008153763	0.928050259	0.968067662
PKP1	-0.1792508	1.603753874	0.669768496	0.413132331	0.643271434
LOC107054154	-0.392710706	1.604097322	5.68703149	0.017090742	0.089935451
LOC107052057	-0.024520309	1.604311546	0.01267849	0.910348637	0.95894146
LOC107051581	0.055679548	1.604474254	0.107118783	0.74344851	0.869767312
TMEM71	-0.210696537	1.604770541	0.955914085	0.328218473	0.565717803
C1QL1	0.307356293	1.605411327	2.463066042	0.116551078	0.307692603
CORO6	-0.159312901	1.60588679	0.523849639	0.469204139	0.688269654
LOC107051809	0.272607453	1.606951381	1.875984048	0.17079129	0.389517765
LOC112533177	0.156402889	1.607696505	0.752803984	0.385589922	0.620101854
C16orf96	-0.765418304	1.608249271	12.47142078	0.000413226	0.006266939
GP5	0.352999254	1.609331973	2.788185145	0.09496172	0.269878255
MLIP	-0.091625961	1.611065565	0.164336647	0.685194854	0.835563601
CACNA2D3	-0.402889456	1.611468307	3.096244398	0.078473133	0.239257996
DUSP15	0.043186618	1.611838646	0.060920289	0.805047255	0.905719444
LOC112530014	-0.462425513	1.614340216	3.938466613	0.047193777	0.172938715
LOC107053673	0.043480142	1.615700902	0.055500125	0.813754989	0.911019232
ZONADHL	-0.670835115	1.617972096	4.555190719	0.032819108	0.137623659
ALKBH4	0.299551517	1.618582068	3.943397737	0.047055645	0.17275784
MOCOS	0.05275247	1.619019077	0.096227375	0.756404783	0.877141698
AGBL2	0.953337686	1.619164605	13.31953613	0.000262655	0.004469483
UPK3BL	0.020917184	1.619426963	0.013043586	0.909072498	0.95829261
HCN4	-0.254576394	1.620249775	1.164196098	0.280597003	0.516294721
TMPRSS11F	0.257715951	1.621335553	1.095332738	0.29529266	0.532205993
ADORA2B	0.694105644	1.621559906	10.14596564	0.001446174	0.015738619
DZIP1L	0.039207839	1.622220857	0.041184795	0.839181638	0.92378471
LOC107050412	0.63904006	1.622948603	13.72154155	0.000212009	0.003751188

CLC2IL1	0.199514365	1.624105483	0.78544383	0.375481377	0.610073664
PRLR	-0.145605017	1.624373453	0.534771983	0.464607499	0.685414775
DGKK	-0.705881757	1.624819221	15.90420961	6.66E-05	0.001512279
RAG2	0.057160533	1.62520329	0.086388413	0.768819754	0.884764739
HTR4	-0.581856313	1.626407881	6.840213918	0.008912817	0.05715526
LOC107052454	-0.171821577	1.62694748	1.078427406	0.299049623	0.536099961
NPY7R	0.229336555	1.627230303	1.034891807	0.309012486	0.546988236
LOC112533263	0.933750237	1.627252902	7.250735329	0.007087198	0.048632131
SLC7A14	-0.575006348	1.627287762	4.840146415	0.027804534	0.123588225
TBX6	-0.212200076	1.627809831	1.269088608	0.259937726	0.494539864
ACHRB	0.482970356	1.627981749	4.892040547	0.026980779	0.121071372
HIST1H110	-0.524614109	1.629351893	7.755102404	0.0053561	0.040042995
LOC107054832	-0.203384076	1.630535092	0.58135404	0.445782069	0.670322942
LOC112530145	0.257993009	1.631076923	0.933726186	0.333896546	0.571760197
LOC112531159	0.114784979	1.631748017	0.183045147	0.668769245	0.824382304
SLC5A1	-0.817866519	1.632511213	20.57422956	5.74E-06	0.00020791
LOC107052145	-0.489304911	1.632752392	2.82746138	0.092664732	0.26606145
PCDH20	0.097654695	1.632789532	0.185465284	0.666717931	0.823698742
SLC11A1	-0.159341988	1.634084177	0.579233332	0.446612987	0.670621395
LOC112530147	-0.06733032	1.635514897	0.08568275	0.769739117	0.88505745
LOC107051717	0.168592529	1.636976106	1.045004493	0.306660409	0.544776458
LOC107054034	0.118979788	1.637089034	0.467428959	0.494172351	0.706269033
SLC35D3	0.340396949	1.637582301	2.139536593	0.143545247	0.351926743
SLC35F3	0.154711507	1.638555468	0.547378856	0.459390674	0.681523442
C5H15orf52	-0.326202439	1.638759866	2.972037301	0.084715105	0.251395322
LOC107055180	-0.141775444	1.639451459	0.515704692	0.472679712	0.691022409
FOXN4	0.025695153	1.639573497	0.024481143	0.87566689	0.943683194
LOC112530573	-0.091268609	1.639722049	0.261884262	0.608828587	0.787473436
TRDN	0.022824107	1.640362508	0.015376573	0.901313447	0.954933316
LOC107057021	0.790872994	1.640628843	5.38768325	0.020279378	0.10005912
LOC107049770	0.134518474	1.641136354	0.440253294	0.507000227	0.716418096
GPR135	0.140780926	1.64121202	0.689702778	0.406265495	0.637343762

LOC107054704	-0.929570455	1.641641664	16.1937664	5.72E-05	0.001334652
ASS1	-0.23302371	1.643716119	1.135524541	0.286600005	0.522903685
SLC17A6	-0.68557651	1.644182291	4.182448891	0.040844642	0.158588622
GPR149	-0.381619818	1.6443314	3.439466186	0.063656257	0.210597532
PPM1J	-0.006512596	1.645210589	0.001681417	0.967291841	0.986598106
C2CD4A	0.501173508	1.645669602	5.332824148	0.020927448	0.102244999
LOC112532933	-0.508439566	1.645697796	6.048764186	0.013916033	0.078259853
CASR	-0.126890099	1.647257956	0.433463926	0.510294129	0.718341124
LOC107052502	-0.463706479	1.648330463	7.06558318	0.007857873	0.052540147
HK3	0.202849014	1.648535259	0.988194509	0.320184053	0.557585839
CTGFL	0.304791534	1.649505921	3.529395942	0.060289749	0.203001397
F8	0.94754142	1.649978994	15.47932052	8.34E-05	0.001803625
LOC107052222	0.008794232	1.650208247	0.002328477	0.961513582	0.983793118
HCN3	-0.182863893	1.652704448	1.103065193	0.293594405	0.530818639
NOV	-0.316797049	1.653108505	2.870644138	0.090208759	0.262036199
BARX1	0.467346786	1.654071172	1.052357938	0.304964657	0.542946366
LOC107050034	1.155587883	1.656062032	38.42395184	5.69E-10	6.69E-08
TNX	0.282815788	1.65696532	1.93069202	0.164682754	0.381697263
PRG4	-0.388442281	1.658164603	6.12404044	0.013335565	0.076114022
LOC107054256	0.490699901	1.658181821	4.620037509	0.031600547	0.133948223
ADAM5P	-0.950485309	1.658459045	33.56022743	6.91E-09	6.25E-07
TCTEX1D2	-0.076238857	1.660310595	0.200279633	0.654495229	0.815640639
LEFTY2	-0.841598345	1.660953072	13.09964362	0.000295352	0.004870847
KCNJ15	-0.715969488	1.662118234	18.65846399	1.56E-05	0.000465399
LOC423277	0.210997038	1.662493474	0.828027415	0.362843543	0.599097263
C1orf228	0.262376968	1.662674724	2.683815384	0.101372588	0.280869831
LOC421935	0.053720972	1.662985587	0.071699433	0.78887829	0.896167916
LOC107054545	-0.5445523	1.663055575	8.300647976	0.003963094	0.032220766
LOC101748309	-0.216306618	1.66325428	1.577654459	0.209098873	0.43679089
KCNMB4	0.185073584	1.6658192	0.584230321	0.444658931	0.669522908
PLEKHS1	-0.537136399	1.666522394	9.68102478	0.001861808	0.019087878
SS2	-0.007055752	1.667133473	0.00079802	0.977463354	0.990525896

SLC16A12	0.041411655	1.667475323	0.044600255	0.832740788	0.920945606
LOC112532347	-0.543957766	1.669256894	5.916532823	0.014999447	0.082107752
SLC7A9	0.8674204	1.669374805	5.888782229	0.015237634	0.0828603
LOC421356	0.429376026	1.669401066	6.402843455	0.011393774	0.068077177
SPACA9	0.22050591	1.670248985	2.185300982	0.139333824	0.344876364
LRRN3	-0.883158315	1.671803241	4.242513246	0.039423766	0.154936248
CCDC170	-0.181065754	1.672466982	1.657900957	0.197886971	0.4235471
LOC768760	-0.043332359	1.672881778	0.057322459	0.810779077	0.909184251
LOC101748017	-0.431309816	1.676486834	3.383416268	0.065855419	0.215223618
C5H14orf79	0.338770363	1.677318997	1.719761897	0.189723697	0.413168797
PTCHD3L	0.000988735	1.677600128	1.54E-05	0.996868239	0.998628203
LOC101749984	-0.315488183	1.677787485	3.874447237	0.049026451	0.177147619
C8G	-0.399586783	1.67790314	5.109764186	0.023791554	0.1114721
MIR4732	-0.316626042	1.678491388	3.747539898	0.052885294	0.186384133
LOC107054049	-0.099449574	1.67971367	0.245323585	0.620387305	0.795058816
RNLS	0.096960594	1.68025221	0.301538277	0.582919663	0.770870614
RHOV	0.185569411	1.680691811	0.777670172	0.377854632	0.612700264
LOC101750972	0.292321884	1.681668024	1.333877727	0.248116536	0.482309002
RASSF9	-0.426977373	1.682455074	3.995185513	0.045630429	0.169262559
TWIST3	0.166319943	1.683420282	0.344924567	0.557000368	0.753115441
TMEM27	0.180744169	1.683944435	0.919245818	0.337673074	0.574790159
DDC	0.195872543	1.684105575	0.553613335	0.456845035	0.679322704
PAFAH2	0.317619593	1.684998786	4.502402364	0.033847269	0.140118054
LOC107054292	-0.312056888	1.685123235	2.934096466	0.086727176	0.255111848
VIPR1	-0.281644455	1.686053661	2.305929434	0.128881168	0.328057279
CEBPA	-0.384960181	1.686565149	2.847824285	0.091497685	0.264093038
LOC107049656	-0.704479283	1.686745133	6.092823454	0.013573202	0.076857204
LOC112530308	-0.164269308	1.687239295	1.18470116	0.276401007	0.511559422
CLNK	-0.215982103	1.687429662	1.862406481	0.172347315	0.391438932
WNT10A	-0.038696541	1.687482759	0.031824174	0.8584141	0.933836045
CCDC57	0.168079201	1.687719654	0.687165585	0.407130147	0.637801433
B3GAT2	0.433835172	1.687787532	5.700373744	0.016961301	0.089511932

LOC107055315	0.177876921	1.690382267	1.012574738	0.314286801	0.551930556
LOC771422	-0.682932828	1.69085685	8.716336454	0.003153711	0.027364135
SCN4B	0.135197448	1.691106116	0.600443373	0.438408909	0.664029694
SEMA6C	0.38939452	1.692783701	4.97139108	0.025769914	0.117301225
SPTSSBL	-0.885486116	1.692822059	20.22312654	6.89E-06	0.000240763
ESRP1	0.227977132	1.693240242	1.605186236	0.205169791	0.432066836
SLC12A8	-0.315546911	1.693611451	3.366721927	0.066526003	0.216452808
FAM131A	-0.305536211	1.694292793	2.196129484	0.138357758	0.343493801
EPHX4	-0.047348265	1.694397489	0.063977377	0.800316514	0.902650356
LOC101748510	-0.205715049	1.695062361	1.434607591	0.231013947	0.462505718
LOC107052151	0.03693449	1.695162763	0.050321391	0.82250497	0.916456624
FAM46D	0.105699766	1.695719151	0.400116971	0.527028855	0.730911066
ASPHD2	0.299389054	1.696625003	2.512851816	0.112921418	0.301251935
KLHL6	0.551398022	1.697010324	8.78372634	0.003039297	0.026646546
TM4SF1	0.191536342	1.697293182	1.555610823	0.212309039	0.440270351
LOC112532432	1.037206199	1.6977638	4.279191953	0.038581686	0.152208497
LOC112532558	0.471437585	1.698743008	5.594532282	0.018016621	0.092787167
KCNMB1	-0.165026234	1.699294495	0.545649482	0.460100782	0.682252715
C14ORF166B	-0.190594369	1.70163962	0.992361789	0.319165817	0.556932504
LOC101751681	-0.025610339	1.702269339	0.019092159	0.890102604	0.950586601
PDE11A	-0.020726392	1.703187254	0.012629895	0.91051989	0.95894146
FRMPD1	0.121136459	1.703285545	0.456205277	0.499402803	0.710379765
LOC107054021	-0.136170372	1.70377515	0.616802471	0.432237996	0.659295163
HKDC1	0.138552705	1.703988638	0.231197676	0.630637188	0.801756543
LOC107051679	-0.140113883	1.704446978	0.475191709	0.490608386	0.703969418
ZDHHC4	0.046208731	1.704888456	0.077820609	0.78027287	0.891500492
SULT1C3	-0.021578265	1.705043834	0.009882537	0.920811987	0.963742606
LOC112532409	-0.837258215	1.705619561	13.94989305	0.000187748	0.003398679
VWC2	0.108714058	1.706240835	0.240627963	0.623752934	0.797751439
CD300LG	-1.042771912	1.706390141	41.23729436	1.35E-10	1.98E-08
KCNJ11	0.064326153	1.706431911	0.15731471	0.691640928	0.839091293
FOXE3	-0.066908872	1.70649235	0.080069868	0.77720275	0.889536847

LOC100857840	-0.247446546	1.707167898	1.535069959	0.215353126	0.444471699
LOC101751795	-0.444520177	1.707403526	3.88069442	0.048844351	0.176663399
SPI1	-0.171267463	1.709100841	0.455081159	0.499931818	0.710640814
PFKFB4	-1.313489932	1.709681637	36.59183644	1.46E-09	1.59E-07
USP50	0.128658916	1.710086151	0.715887629	0.397496163	0.629978244
SYTL3	-0.316126853	1.710118598	3.058710829	0.08030536	0.242909885
LOC101748400	-0.132532666	1.710312698	0.325652299	0.568230457	0.76104771
LOC112532987	-0.300510137	1.710728636	2.635748956	0.10448278	0.286830855
CELF4	-0.214168837	1.710811354	0.913562578	0.339170926	0.576114424
LIPE	-0.066073298	1.713208506	0.109127221	0.741139978	0.868436825
MHCDMA	0.19164976	1.713274933	1.183286551	0.276687933	0.511849347
HEXDCL	-0.365419866	1.714238694	5.392081843	0.020228323	0.099867105
LOC112531425	-0.261685926	1.714770911	1.342783574	0.246543675	0.480160513
LOC112530393	0.189608775	1.716492656	1.45783591	0.227274764	0.457058792
DCAKD	0.060497182	1.717407758	0.112987635	0.736768061	0.86564214
LOC112531334	0.179777516	1.718689642	0.626873047	0.428504626	0.656033087
FMOD	0.139987568	1.718924938	0.407198651	0.523394795	0.727919042
IL1RL1	-0.316828621	1.719353691	1.118960726	0.290142347	0.527005473
LOC112530211	-0.495528475	1.719399607	8.305380437	0.00395278	0.032205321
GPR19	-0.064702443	1.719740985	0.076249781	0.782445432	0.892887345
PNLDC1	0.489617207	1.720094624	7.013174264	0.00809121	0.053513207
GBP	2.354246735	1.720854443	22.23494862	2.41E-06	9.92E-05
ADRB2	-0.166611447	1.721993144	0.505780685	0.476971092	0.693600685
NQO1	0.077395429	1.722027876	0.191733613	0.661477647	0.820458761
AKNAD1	-0.229169847	1.722408805	2.182318595	0.139604007	0.34532758
LOC112533471	-0.361525452	1.72267915	4.882289092	0.027133612	0.121426051
LOC107053916	0.15797659	1.722732986	0.88362388	0.347211045	0.583829731
SFN	-0.087986907	1.722951272	0.199581283	0.655059035	0.815935482
GATA6	-0.547092016	1.723198077	2.226642382	0.135648343	0.338760584
LOC112530471	-0.297795384	1.723746876	3.561346923	0.059139733	0.200441669
LOC107049166	-0.095236306	1.72400177	0.254455269	0.61395531	0.790936713
LOC107050906	-0.151414971	1.724388961	0.249343108	0.617537995	0.793398483

LOC107053726	-0.05518064	1.726893274	0.066778042	0.796087045	0.900610289
SSSCA1	0.095465224	1.726928346	0.428557234	0.512697742	0.720201583
SLC7AL	-0.547949403	1.728854444	6.675827885	0.009772911	0.061099258
PRDM13	0.10653686	1.729946965	0.376562035	0.539448945	0.740459825
LOC101750260	-0.240596123	1.731550303	1.377524661	0.240523238	0.473216236
LOC419830	0.990124063	1.731682386	24.87850207	6.11E-07	3.19E-05
PLSCR5	-0.972127051	1.732228555	24.21887711	8.60E-07	4.25E-05
MIR1627	0.167633775	1.732465714	1.079028375	0.298915017	0.535975439
SYCE2	0.11178923	1.732992189	0.382016348	0.536526091	0.737836448
CD44	-1.487288798	1.733693016	14.02315757	0.000180573	0.003286574
RAPGEFL1	-0.276001233	1.735132102	2.449739549	0.11754437	0.309371819
SPRN	-0.188006448	1.736701885	1.274453202	0.258932922	0.493426894
LOC769517	-0.857633497	1.737521047	5.050711652	0.024615826	0.11381102
DYNLRB2	-0.892253371	1.737726104	10.07420234	0.001503597	0.016224058
LOC107055397	-0.009160352	1.738839004	0.001960166	0.964686189	0.985185872
MSTN	-0.430863077	1.741008616	1.860243226	0.172596731	0.391843158
ALPK2	0.005233545	1.744454981	0.000801389	0.97741585	0.990525896
LOC420552	-0.178705962	1.746834682	0.933794913	0.333878757	0.571760197
RASL12	-0.51483796	1.749087168	5.712820613	0.016841458	0.089079459
SPRY4	-0.349251603	1.749362255	2.390361847	0.122085367	0.31659123
MTMR11	0.010076821	1.750884381	0.002468703	0.960372581	0.983096197
LOC101752258	-0.010251273	1.751560034	0.003302268	0.954174496	0.980523198
LOC107050975	-0.061447857	1.751778636	0.144624644	0.703726098	0.846564677
LRP1B	-0.330805963	1.75331654	2.386843259	0.122360491	0.316903551
SLC6A7	-0.167530515	1.754059088	0.815718433	0.366434977	0.601466431
LOC107052156	-0.42517389	1.754343263	5.171031292	0.022966578	0.108735465
LOC112533021	0.07666914	1.754430836	0.125292809	0.723363433	0.857066913
STAR	-0.021151931	1.754801931	0.010424712	0.918676128	0.962372833
LOC112531631	0.545166335	1.755857504	3.391923827	0.065516464	0.214541973
PAQR7	0.380555942	1.756866818	5.784682958	0.016166413	0.08670741
DGKG	-0.509523618	1.758108404	7.787595571	0.005260616	0.039617133
LOC107052050	-0.569304201	1.760225436	9.506966416	0.002046933	0.020488005

APOLD1	0.402706385	1.760703905	4.357510845	0.036846106	0.147914778
CX3CL1	-0.06721735	1.761443014	0.15580941	0.693044369	0.839582206
LOC768462	0.293977446	1.761492575	2.651037846	0.103482315	0.284879329
LOC415756	-0.289476456	1.761710311	2.581385079	0.108127011	0.293044633
LOC112530964	0.022237599	1.763672885	0.020202634	0.886972606	0.949086044
FBP1	-0.762638099	1.763903933	15.01085402	0.000106895	0.002155577
LOC100858360	0.196112722	1.763973564	1.243932548	0.2647145	0.499885477
LOC107052234	-0.858120772	1.766254863	18.90724396	1.37E-05	0.000418172
HNMT	0.469514159	1.766490214	5.696599588	0.016997813	0.08964712
CFC1	0.006392934	1.767945764	0.000693791	0.978986213	0.991002256
LOC101749016	-0.229039596	1.769219899	1.250886421	0.263383243	0.498501411
TAP1	0.962892734	1.770468437	16.58600388	4.65E-05	0.001123407
LOC107054851	0.043280399	1.771719747	0.047244098	0.827930081	0.918504892
TRPV6	-0.517113362	1.772246274	4.277322751	0.03862414	0.15233946
LOC107050978	-0.229273599	1.772257506	1.696668462	0.192724247	0.41714129
LOC112531742	0.301248835	1.773962506	2.565492159	0.109218563	0.295071008
LOC107054418	-0.133341359	1.774015656	0.327173871	0.567327973	0.760711734
LOC101750514	0.037260891	1.774264255	0.02021879	0.886927726	0.949086044
STARD10	0.246643043	1.775087069	2.203235973	0.137721357	0.34232703
LOC107053375	-0.046336322	1.775334296	0.054398289	0.81557939	0.912577102
GJB1	-0.07041995	1.776937352	0.15439978	0.694365736	0.840502295
LOC107049884	-0.365081198	1.777133677	4.000854032	0.045477215	0.16888458
LRRC15	-0.558114828	1.777919094	5.640314882	0.017552055	0.091346954
BTN3A3L1	-0.726434918	1.778050847	8.53281152	0.003488005	0.029433394
LOC771545	0.155452462	1.778904072	0.978526539	0.322562851	0.560071595
NAALAD2	0.093725486	1.779809359	0.295893815	0.586468155	0.773078066
LOC112531846	-0.880308058	1.77998967	11.36954321	0.000746582	0.009703802
LOC107049469	-0.462490187	1.780309644	6.750503241	0.009372125	0.059246374
PTK2B	0.054375964	1.780835315	0.067349123	0.795236294	0.900159126
LOC112532649	-0.336998975	1.782304822	3.073972177	0.079554866	0.241482258
HTR1D	-0.270051329	1.783051895	2.298292957	0.12951627	0.329180906
LOC101751268	0.175225489	1.786997936	1.038960983	0.308063243	0.546012566

TLR2A	-0.916313941	1.787776509	12.01892723	0.00052663	0.007535389
LOC107051303	1.838642391	1.78808641	55.81256568	7.97E-14	2.68E-11
LOC112531034	-1.504334967	1.788427695	21.45204832	3.63E-06	0.000140133
LOC112531837	-0.199782203	1.788654456	1.534652791	0.215415485	0.444471699
LOC430433	-0.203463401	1.789108862	1.040444464	0.307718124	0.545867775
LINGO2	0.154138761	1.789266764	0.458000088	0.498560121	0.709917514
LOC112530181	1.328159327	1.790821439	10.78327998	0.00102421	0.012248096
LOC107052129	0.148207888	1.791239674	0.496816413	0.480902313	0.69668007
SSTR4	-0.127955273	1.792015843	0.498134543	0.480320936	0.696466157
LOC100859726	0.053711344	1.792478619	0.086900213	0.768155512	0.884410786
LRRC46	0.415064385	1.792930708	8.547702253	0.003459586	0.029241529
LOC107049922	0.530455599	1.793676303	2.623090055	0.105319166	0.288309245
CHST1	-0.047674456	1.793904138	0.064457542	0.799584482	0.902345328
TDRD9	0.485689423	1.795102096	4.506371506	0.033768805	0.140023865
C3H1orf95	-0.698414946	1.79664423	7.508827154	0.006139734	0.044214145
LOC112531011	1.013600437	1.796655626	7.4048132	0.00650496	0.046018535
LOC112530406	-0.110854706	1.798536848	0.48483924	0.486238478	0.700617701
PROKR2	-0.571960646	1.799352185	7.601212081	0.005832908	0.042525108
MKX	0.069303014	1.799397327	0.121278408	0.727652067	0.859813626
LOC107054541	-0.348551331	1.799453915	4.351909293	0.03696748	0.14822122
G6PC2	-0.194299681	1.801845045	1.466873484	0.225839636	0.4557507
LOC107053492	-0.136341642	1.803179319	0.675079329	0.411286307	0.641368099
MSLN	-0.727236583	1.804038421	11.71300122	0.000620649	0.008496494
HUC	0.284999481	1.804231766	2.023171552	0.154915284	0.367914655
LOC112530356	0.019468527	1.804296943	0.015403672	0.901226968	0.954933316
SMAD7B	0.133413748	1.804616728	0.298117946	0.585064712	0.771846375
HS6ST3	-0.360168228	1.806109988	3.350782366	0.06717308	0.217628083
LOC107053670	0.031852825	1.806207899	0.049581313	0.823793428	0.91690482
SLCO2B1	0.261154054	1.807058495	1.508577957	0.219356206	0.448737316
FGL1	0.080876972	1.807573068	0.286877685	0.592228366	0.776980894
MATN1	0.277660437	1.808187251	1.399593785	0.236791596	0.469065191
RSPO4	-0.183988288	1.81003453	1.137814911	0.286114522	0.522416162

LOC112533092	-0.028480545	1.810449458	0.033315547	0.855170368	0.932526733
LOC112531203	0.3060526	1.8105804	0.818949431	0.365487507	0.600890352
TCEA3	-0.796727544	1.811343149	18.11578359	2.08E-05	0.000578768
GABRG1	0.14094139	1.81346809	0.46592123	0.494869602	0.706915718
BG2	2.170678336	1.814176101	32.06362111	1.49E-08	1.25E-06
BIRC7	-0.016537097	1.814223497	0.011379774	0.915046005	0.961017703
LOC101750917	0.020345656	1.814230115	0.014276118	0.904892937	0.95651142
MIR6639	0.104112532	1.814249265	0.492951854	0.482613497	0.697654844
LOC107051937	0.148110443	1.815351354	0.242210052	0.622614436	0.796762373
NAT8	-1.334511631	1.815571882	40.22562312	2.26E-10	3.10E-08
FA2H	-0.022576388	1.815944264	0.020491532	0.886172795	0.948791653
GGACT	0.077543592	1.817169604	0.239296176	0.624714917	0.798386013
FSTL5	-0.377163547	1.817195741	2.927990565	0.087055786	0.255758428
PRDM2	0.005062923	1.817730545	0.000989781	0.97490206	0.989942203
STYXL1	0.299923726	1.819364744	3.493007213	0.061628539	0.20628511
INHBB	-0.446098159	1.820060794	5.915166356	0.015011085	0.082144134
ADAM19	-0.380183596	1.820538594	3.895557508	0.048413968	0.175608933
LOC107051833	0.492951179	1.820664004	6.036971833	0.014009294	0.078515985
LOC101751884	-0.249781741	1.821910677	2.641484033	0.104106256	0.286065178
LOC107054149	-0.433405404	1.823548584	6.110150071	0.013440772	0.076484271
FSIP1	0.592317967	1.824418204	12.33437284	0.000444693	0.006642659
LOC422106	0.413747391	1.825272781	6.854880821	0.008839945	0.05688634
SYTL1	-0.055399522	1.825276265	0.066477045	0.796537009	0.900763967
A2M	0.695323279	1.825788965	2.756466918	0.096861938	0.272657543
LOC107052552	-0.169674735	1.82674897	1.143527267	0.28490823	0.520848201
LOC112530072	-0.152085324	1.826784713	0.746755205	0.3875055	0.62171007
PPARGC1A	-0.27286988	1.826924973	2.494805379	0.114222495	0.303152969
LOC107055329	-0.311222767	1.82823212	0.691525081	0.405646128	0.636675604
CNGB1	-0.224613759	1.828830007	0.98548997	0.320847169	0.558298979
LOC107054453	-0.246244678	1.829157308	2.19184868	0.138742704	0.344086088
LOC112532515	-0.438256616	1.829958678	4.771529099	0.028934116	0.126457074
LOC107053523	-0.648177256	1.83250346	13.97314276	0.000185441	0.003360603

LOC101751157	-0.107242862	1.832894266	0.152103224	0.696533491	0.842034358
GRID1	-0.599201011	1.8335421	10.86579441	0.000979567	0.011860764
FBXO6	0.068044151	1.835053575	0.176905666	0.67404606	0.828151117
LOC101748328	-0.50726389	1.835787584	6.621757259	0.010074039	0.062295494
ERMN	-0.119538725	1.835875459	0.511065667	0.474677901	0.692264899
LOC112532921	0.300764585	1.836153089	2.625309938	0.105171967	0.288176051
GRID2	-0.340929523	1.836635597	3.356554558	0.066937981	0.217037335
LOC107049616	-0.100650121	1.83670122	0.33925262	0.560260942	0.755498094
BARX2	0.113246075	1.836996804	0.214222789	0.643477568	0.809574385
LOC772158	0.349763672	1.837766664	4.480380585	0.034286085	0.141397878
C6H10ORF71	0.593055646	1.837940767	6.716293147	0.009553599	0.060070491
LOC112531887	-0.856247748	1.838249124	9.365341755	0.002211268	0.02155593
TSPAN33	0.042350722	1.838557626	0.038235408	0.844971194	0.92724248
TMEM178B	0.770846317	1.838842814	21.99521075	2.73E-06	0.000110108
IFIH1	-0.157847918	1.8393085	0.749595627	0.386604288	0.620979506
CCM2L	-0.06531585	1.841175524	0.13919306	0.709084542	0.849542309
MTTP	0.313022111	1.841558994	1.968568695	0.160599869	0.376057489
FGFBP3	-0.007729844	1.841598288	0.001458525	0.969535681	0.987658702
LOC101747364	0.69924078	1.842395563	14.02124674	0.000180757	0.003286574
LOC100858742	0.222766748	1.842741186	0.812298483	0.367441575	0.602396245
LOC107054748	-0.321080472	1.843012643	3.816436999	0.050752053	0.181527053
LOC112529976	-0.020826164	1.843745043	0.019665088	0.888476482	0.949552787
GLRA4	-1.261835981	1.844975607	30.55178755	3.25E-08	2.51E-06
IL16	-0.835276513	1.845034412	13.12061971	0.000292063	0.004825204
LOC107053349	-0.281065968	1.85019032	2.627211648	0.105046045	0.287944807
LOC100859063	1.704566668	1.850224183	43.78671855	3.66E-11	6.15E-09
LOC112531347	-0.213433007	1.851239897	0.835178527	0.360779366	0.597237914
MDH1B	0.595971157	1.851844396	13.02038042	0.000308119	0.005039863
LOC112532475	0.157047602	1.852046032	0.630849458	0.427043898	0.654871047
MKRN3	-0.571977491	1.85227486	8.73337954	0.003124369	0.027173089
CABYR	-0.603609806	1.852579523	5.208289232	0.022479444	0.107109491
DENND2D	-0.052877281	1.853159237	0.097838148	0.754439528	0.875726752

GABRD	-0.463697244	1.853506777	7.193377723	0.007317311	0.049816449
TNFRSF13B	0.08374058	1.853535572	0.292478829	0.5886364	0.774756996
SPATA1	-0.185434074	1.853767597	0.775639941	0.378477927	0.613269279
PTPRCAPL	-0.017827099	1.855780282	0.015704801	0.900271172	0.954481623
PRKCH	-0.279675638	1.857262044	2.560307126	0.109577294	0.295660128
PLEK	-0.277471222	1.857777714	2.589591742	0.107568059	0.292036365
FAM173A	-0.33162544	1.859013451	4.453567006	0.034828426	0.142552294
MIR6640	-0.423043227	1.859029782	4.847843545	0.027680718	0.123237612
PHOSPHO1	-0.037984656	1.859258317	0.045427656	0.831219603	0.919835926
CASP7	0.628254282	1.860488391	12.53488205	0.000399425	0.006125383
DIRAS1	-0.053906008	1.861056359	0.07432102	0.785146245	0.89437421
LOC112531017	0.064090477	1.862982301	0.058597169	0.808727069	0.907749244
LOC112530064	0.18372749	1.864779162	0.502300035	0.478491246	0.694655901
LOC101747844	-1.110197152	1.865832669	16.95895387	3.82E-05	0.000959584
LOC101751708	0.021418191	1.866891875	0.014644954	0.903678096	0.955902743
LOC107052877	0.046192964	1.867064969	0.082030432	0.774564458	0.888742637
TMEM79	-0.52663186	1.867476175	9.337860446	0.002244674	0.021735939
NOX3	-0.591136666	1.867889377	7.944117215	0.004824395	0.037368349
HTR6	0.40092665	1.869859108	4.093902383	0.043038166	0.16347946
APOA2	0.458534422	1.871785585	1.763721145	0.18416105	0.406794321
TMEM136-1	-0.056181338	1.872194074	0.07562752	0.783312718	0.893298966
LOC107050349	1.242147234	1.873291358	25.67338526	4.04E-07	2.25E-05
MIR1647	0.004940556	1.874422637	0.001033212	0.974357515	0.989871494
LOC107057249	-0.203091188	1.874607881	1.08470178	0.297648112	0.534694288
ADAMTS2	0.336592275	1.874621873	2.207886788	0.137306644	0.341718161
PNAT3	-0.039817126	1.876000544	0.052775614	0.818302019	0.914131685
LRRIQ1	0.214443364	1.877382301	1.008651259	0.315226173	0.552589063
RNF144B	-0.423549745	1.877442778	7.580481571	0.005900368	0.042878815
LOC107053792	0.126404465	1.877567313	0.492451006	0.482835999	0.697708164
LOC107054027	0.043204755	1.879437585	0.041753444	0.838090475	0.923661189
LOC107054120	-0.128069999	1.879656058	0.351117173	0.553481406	0.751671616
ST8SIA3W	-0.735916345	1.879764146	6.040012722	0.013985183	0.078480313

LOC107051755	0.617115528	1.880275768	3.840050658	0.050042009	0.179869214
DNAH7	-0.399795889	1.880364212	5.282879107	0.021536108	0.104228427
ZNF7L	-0.306940612	1.883635734	2.481249787	0.115210672	0.305083941
MGAT4A	-0.14969622	1.88413665	0.730610435	0.392685394	0.626036771
ADPRHL1	0.126992705	1.884648698	0.243817101	0.621462694	0.795958144
SYT15	-0.727702195	1.885121442	11.91192349	0.000557758	0.007878029
TEKT1	0.234531202	1.886808083	2.374803547	0.123307109	0.318327342
MUSTN1	-0.094667613	1.887016785	0.172694139	0.677728457	0.830489082
LOC100858737	0.518426356	1.887228737	10.52929701	0.001174969	0.013454499
DMTN	0.204424233	1.887586143	1.287598707	0.25649095	0.491276753
C20H20orf96	0.493669185	1.887884671	7.896048192	0.004954292	0.038023728
LOC112533003	-0.567706506	1.889511244	5.106826453	0.023831875	0.111597467
THRB	-1.103601994	1.890373568	8.457602377	0.003635207	0.030456893
INA	-0.334504376	1.890591526	2.133885742	0.144075118	0.352738591
NPM2	0.259710742	1.890666229	2.383030499	0.122659393	0.317392968
LOC112531576	-0.567302061	1.891257425	10.36489179	0.001284344	0.014367019
LRRC61	0.338910842	1.891725138	4.132706323	0.042062033	0.161260659
LURAP1	0.08261826	1.891848328	0.23201018	0.6300373	0.801303429
MLXIPL	-0.040274699	1.892646899	0.059801611	0.806809777	0.906648561
APOD	0.670489119	1.892878298	4.324790359	0.037561041	0.149614536
SHISA6	-0.24281823	1.893031181	1.717189899	0.190055169	0.413417842
TEC	-0.495216669	1.893283889	9.110928416	0.002540869	0.02354422
LOC112532882	0.125282397	1.89502434	0.545079151	0.460335351	0.68238923
LOC107053086	-0.788801216	1.895587419	16.53778011	4.77E-05	0.001148967
KCNJ9	0.097652244	1.895747373	0.484601845	0.486345231	0.700710163
SAMD7	1.404474998	1.896067229	13.13568297	0.000289725	0.004801026
WHRN	0.399626014	1.896667097	5.333163993	0.020923368	0.102244999
SCNN1B	-0.244728442	1.897301971	1.842874308	0.174614434	0.394145879
DNAH5	-0.243443155	1.89761707	1.778784836	0.182298612	0.404493483
SFRP5	0.614357847	1.897706778	4.385514396	0.036245553	0.146512375
BHLHE22	0.162085816	1.899183368	0.562194336	0.45337746	0.676182915
DNAI2	-0.213817852	1.900215848	1.294495739	0.255221093	0.49032844

MORN5	0.172825132	1.901475065	1.505723477	0.219792807	0.448999459
LOC112531072	-0.677111314	1.901945644	7.284908789	0.006953626	0.048015912
SYCP2	-0.095644387	1.902246258	0.28538002	0.593196448	0.777327412
LOC112530404	-0.472955437	1.902754609	6.251425531	0.01240934	0.072358501
C10orf67	0.261256599	1.905289852	2.169504428	0.140771592	0.347546369
LOC112532116	-0.089953061	1.90535934	0.310435846	0.577412856	0.76705002
LOC101748621	0.306665059	1.905674268	3.017071193	0.082392141	0.246681709
LRRC10B	-0.071348813	1.907841896	0.124130246	0.724597371	0.857972926
LOC112532329	-0.244786474	1.908568583	1.264001839	0.260894954	0.495752133
LOC112530403	-0.141559516	1.908636521	0.978724107	0.322514007	0.560071595
BRINP2	0.260845779	1.908834399	1.280124918	0.25787581	0.492541889
LOC418108	0.674886032	1.909632824	10.67550936	0.001085633	0.012741861
LOC112531766	-2.334606766	1.91041228	61.49547528	4.44E-15	1.74E-12
LOC107053476	1.005599678	1.910988195	7.820604973	0.00516539	0.039042947
C2orf72	-0.013562374	1.911777299	0.00572231	0.939700774	0.974033149
SPRY3	-0.083834403	1.912620682	0.102612137	0.748717204	0.873168892
MSC	-0.213168659	1.914565841	0.322778646	0.569942546	0.762434642
LOC112530287	-0.423370684	1.915181013	5.416051094	0.019952437	0.098983362
AOX2	0.819672966	1.917062681	24.18120913	8.77E-07	4.32E-05
VPS37D	-0.257103929	1.917284857	2.05323565	0.151883102	0.363684
AS3MT	-0.079285375	1.917880921	0.138519343	0.709757445	0.849639454
LOC101749281	0.011371664	1.918064186	0.00304864	0.955967584	0.981041173
LOC101748543	-0.152403082	1.918064795	1.086053807	0.297347215	0.534387114
SPNS3	0.378980476	1.91831952	3.541821569	0.059839711	0.201857819
LOC112533449	0.300014364	1.918388533	1.571873287	0.209935175	0.437997123
TTC24	0.076200503	1.919075875	0.15918696	0.689906169	0.83840108
NPAS2	-0.748466359	1.919598366	16.69526428	4.39E-05	0.001073131
C6H10orf54	0.004330487	1.919628471	0.000822195	0.977124639	0.990525896
CAPNS2	-0.527861045	1.92145218	10.88374533	0.000970119	0.011781036
LOC107052837	0.4344705	1.923001888	4.810451754	0.028287629	0.124748861
AQP3	-0.914442793	1.923450553	9.594372011	0.001951747	0.019775857
NPS	0.477839656	1.92345582	6.19822686	0.012787836	0.073845165

PARVG	-0.005324244	1.925552685	0.00104657	0.974192352	0.989832365
LOC107054512	-0.38582144	1.925943795	3.866782459	0.049250853	0.17768533
LOC107053371	0.362928934	1.927619014	1.945386571	0.16308486	0.379467221
EPB42	0.866093722	1.927958876	7.69826308	0.005527399	0.040878491
TRIM65	-0.843226727	1.928463543	15.06417186	0.000103917	0.002132114
HRASLS	0.015625116	1.929699479	0.010498128	0.918391264	0.962372833
LOC101749424	0.201756579	1.931700749	1.500689215	0.220565345	0.450049422
PTGDS	0.217036009	1.931875267	1.48547711	0.22291954	0.452323547
ADAM11	-0.417494949	1.933031193	4.028720936	0.044731842	0.166867109
ENKUR	0.668573768	1.933636052	10.15901689	0.001435972	0.01566242
MGAM	0.118437198	1.933724195	0.173532258	0.67699147	0.830079538
LOC112531193	-0.126592346	1.934246606	0.582115672	0.445484238	0.670120042
LOC112531927	-0.186514044	1.937895611	0.912315847	0.339500702	0.576520542
LRRC34	-0.728656759	1.938295605	14.09281845	0.000174007	0.003206364
PCOLCE2	0.030057713	1.938432352	0.03144183	0.859258266	0.934098041
DNAH12	0.528208542	1.939927324	9.08794027	0.002573005	0.023695391
UPF3A	-0.23795449	1.9401242	1.929796706	0.164780688	0.381735354
STPG3	-0.163133183	1.94079417	0.759958918	0.383341379	0.618056281
LOC107054527	0.313425605	1.940810394	1.656841883	0.198030266	0.423698995
LOC101748820	-0.930538397	1.942116449	8.867414493	0.00290306	0.025835508
LOC422090	-0.004024612	1.942624497	0.000316801	0.985799289	0.993345211
PALLDL	-0.145229629	1.944491185	0.356976134	0.550190428	0.748832382
LOC101751026	-0.695047508	1.948005101	17.19008245	3.38E-05	0.000862796
LOC107054290	0.142272909	1.949556383	0.581115704	0.445875333	0.670401919
C1orf53	-0.227335705	1.953138817	1.010602923	0.314758441	0.552319857
LOC112530490	-0.170225665	1.953616132	0.206936008	0.649179603	0.81275402
CYP2AC7	-0.239196248	1.95491205	2.059059052	0.151303575	0.362972935
KCNA2	0.205259825	1.955878764	1.162677648	0.280910911	0.516583487
DDEFL1	-0.491318338	1.956044433	3.274691135	0.070356169	0.223971901
LOC112533507	-0.335830744	1.956269675	2.728220284	0.098589092	0.275617314
LOC112530294	-0.301789665	1.957466634	3.121235841	0.077278154	0.237584212
POU3F1	0.135499152	1.957800943	0.479222407	0.488774745	0.70225654

SVOP	-0.236724304	1.957929691	1.181624405	0.277025547	0.512185997
CHRNG	-1.00110757	1.957933855	19.28239336	1.13E-05	0.000360225
SYCP3	0.120138537	1.95815497	0.766234307	0.381384526	0.61598767
NPTX2	0.426425319	1.958192263	8.143130833	0.004322459	0.034316908
LOC107054441	0.131150066	1.958483805	0.608164535	0.435479733	0.661847142
MFSD2B	0.71994921	1.958863765	8.319224738	0.003922766	0.032066126
SYT12	0.182124421	1.959788521	1.369415525	0.24191233	0.474797763
LOC101752151	0.195596041	1.960606644	0.424211003	0.514843279	0.721736766
CCDC83	-0.026408174	1.960795497	0.025133887	0.8740339	0.942721236
LOC112530995	0.282685327	1.961887144	2.268261821	0.132047929	0.333361256
FUT9	0.598904379	1.962665309	6.213765291	0.012676073	0.073419493
C1QTNF4	0.060691894	1.962754595	0.08240368	0.774066064	0.888294656
PLA2G4EL2	0.132734096	1.962944944	0.188544138	0.664131091	0.821860352
RRAD	-0.628321015	1.962949072	4.116764478	0.042460211	0.16229565
GBP4L	0.751445914	1.963650717	10.87514679	0.000974633	0.011818413
LOC101750994	0.071023005	1.964618662	0.152411962	0.696240975	0.841845405
GFRA3	0.066981446	1.965372011	0.069506762	0.792056293	0.897745396
LOC107054697	0.560580094	1.966015985	6.36030357	0.011670158	0.06925079
AOAH	-0.950863471	1.966462087	13.34873375	0.000258597	0.004418709
LOC101752305	-0.345661541	1.967954849	4.398958374	0.035960888	0.145852751
FAXDC2	0.220838059	1.968309753	2.003244576	0.156962904	0.371332607
GALNT14	-0.383837977	1.968918517	2.756373785	0.096867578	0.272657543
LOC112530299	0.010534881	1.96915042	0.001505587	0.969048337	0.987351035
EFHC1	-0.551728572	1.969282965	8.221522549	0.004139645	0.033293189
ESYT3	-0.337510305	1.969395981	4.556076852	0.03280213	0.137588339
RPE65	-0.609463587	1.970017035	7.961853455	0.004777347	0.037063292
LOC112530018	-0.232500013	1.970338855	0.887736885	0.346091328	0.582540186
LOC107049387	5.459299889	1.970624347	40.28883605	2.19E-10	3.03E-08
LOC101748980	-0.220458199	1.97176345	1.86795295	0.171709715	0.390367969
LOC414835	-0.576095443	1.972869969	8.773086995	0.003057076	0.026758985
LOC107051077	0.108707258	1.974371807	0.51661799	0.472287928	0.690861219
MIR6593	0.130244901	1.974581632	0.681156054	0.409188925	0.639673548

FAM83B	-0.289049352	1.975252328	2.221106914	0.136135424	0.339513248
LOC112531709	0.665362402	1.975952062	5.780163816	0.016208031	0.086902296
LOC112530103	0.48708745	1.976150193	6.23210732	0.012545435	0.072996866
KCNJ12	-0.026243785	1.97628199	0.030029539	0.862423223	0.93597525
LOC107051508	0.066042577	1.976948033	0.191819266	0.661406752	0.820458761
DHRS13	1.480428889	1.977041229	68.54611492	1.24E-16	6.37E-14
LOC107050420	-0.25390533	1.977553912	2.420545602	0.119753215	0.312585525
PIK3R6	-0.388194806	1.977799988	3.846949013	0.049836568	0.179259953
RHOC	0.184747018	1.977801496	1.032413847	0.309592398	0.547425629
NMB	-0.067686817	1.97819934	0.149226453	0.699275686	0.843658952
LOC112531849	-0.729820941	1.978925011	7.901099159	0.004940477	0.037954122
BBOF1	0.089548709	1.979542789	0.283913477	0.594147583	0.777946576
NOXA1	0.703118838	1.979917024	12.20245731	0.000477266	0.00699454
TMEM59L	0.666143093	1.981472003	12.78525356	0.000349362	0.005513696
LOC112531944	0.451431167	1.982612076	4.824769793	0.028053611	0.124192137
PCDH9	0.526449131	1.983619848	5.715008122	0.016820487	0.089050529
LOC107049564	-1.864997522	1.983877756	58.85503655	1.70E-14	6.21E-12
LOC112531499	-0.732070722	1.984571107	0.802778418	0.370263964	0.604793879
LOC107051884	0.419070392	1.984707379	7.871394924	0.00502229	0.038292634
PIK3R5	0.154947145	1.984864857	0.620322049	0.430927636	0.658333882
CNKSR1	-0.269722576	1.985086622	2.065644112	0.150651264	0.36205149
ABI3	-0.748004347	1.985430629	21.45433366	3.62E-06	0.000140133
C3	-0.782910847	1.987648593	11.09080571	0.000867568	0.010815022
QRICH2	-0.395739859	1.988401642	6.364129449	0.011645021	0.069216652
HPGD	0.22420132	1.989514301	1.810405312	0.178459616	0.399368012
LOC107050958	-0.192167052	1.991476285	1.341093161	0.246841279	0.480491801
IRF7	-0.214519655	1.991663886	1.259280381	0.261787349	0.496795159
ZAR1	-0.170209403	1.992000155	0.820568326	0.365014053	0.600737409
GUCA1C	-1.374883162	1.992882875	11.91294252	0.000557453	0.007878029
DUSP26	-0.107684572	1.994123557	0.354299109	0.55168954	0.75025218
LOC107057545	-1.158222039	1.994882001	4.589078912	0.032176301	0.135724439
CFAP99	0.496854246	1.995324637	6.852480654	0.008851828	0.056919436

LOC101750445	-0.041806235	1.996330479	0.064522083	0.799486307	0.902345328
LOC107055328	0.14403691	1.996554788	0.85829291	0.35421685	0.591019901
LOC101751855	-0.394475856	2.000734485	2.370391575	0.123656031	0.318775621
LOC107049680	-0.522687066	2.000762389	6.08830726	0.01360794	0.077027399
SIRPA	-0.965142199	2.001053732	20.17738981	7.06E-06	0.000244512
UBE2L6	0.006093338	2.001648482	0.001256958	0.971718045	0.988743849
LOC415478	-0.329595587	2.004935676	3.539879914	0.059909798	0.202033125
TPTE2	-0.244950528	2.00650656	2.578872613	0.108298771	0.293439202
TMEM121L	0.174484138	2.006865458	1.428613304	0.231990881	0.463558154
ANKDD1A	-0.383680965	2.007481753	4.159741143	0.04139573	0.159710841
LOC112530473	-0.344336578	2.008220888	3.177595064	0.074654533	0.232444675
ABHD3	-0.240184468	2.009761042	1.930971229	0.164652227	0.381697263
LOC100858460	-0.319125808	2.010619201	3.422678672	0.06430659	0.211972143
ZNF737L	-0.535009617	2.011219388	1.427365245	0.232194914	0.463704852
RAP1GDS1L	0.017536181	2.011629965	0.010407534	0.918742925	0.962372833
RAB3B	0.241455404	2.012005582	1.98742458	0.158610423	0.37362099
ANKS1B	0.224672779	2.013141211	2.118525014	0.145526636	0.354656516
LOC112532618	0.226806486	2.014366473	1.692932409	0.193214864	0.417401941
LOC107051634	0.027541549	2.014729706	0.041009533	0.839519522	0.923898005
LOC112533587	-0.908000484	2.014772865	4.571931236	0.032499907	0.136508109
NRROS	0.249538559	2.016577615	1.367886983	0.242175261	0.475028094
DGKI	-0.472767051	2.017086861	7.43397585	0.006400378	0.045612047
LOC107053511	-0.411168762	2.017926181	5.184599202	0.022787928	0.108058452
CYP2R1	-0.177099234	2.018112798	1.20959714	0.271411922	0.50640736
GRM5	-0.096865664	2.018552715	0.224679091	0.635497582	0.804702787
PDZD7	-0.258003639	2.018934086	1.580360107	0.208708831	0.436268268
FAM196A	0.258806869	2.019267818	1.889547429	0.169252976	0.387675072
MIR2954	0.474615927	2.019338435	5.142671801	0.023344694	0.109941883
LOC112532894	-1.229153998	2.019340983	36.67729537	1.39E-09	1.54E-07
ANKRD24	-0.116925405	2.019557096	0.399258067	0.527472671	0.731324607
LOC107055155	-0.225618987	2.021594057	1.900627442	0.168008105	0.386069383
LOC107050006	0.581927867	2.022324242	12.14186142	0.000493025	0.007198521

ANKMY1	0.076070626	2.022458608	0.219163507	0.639678024	0.807139168
PKIB	0.011326637	2.02273924	0.004161764	0.948562766	0.978350277
CDH17	0.036335382	2.023076998	0.048279572	0.826084454	0.917837275
CYP2W2	-0.311693557	2.023973009	1.789479818	0.180989555	0.403134632
SYNDIG1L	0.21059483	2.025209272	1.215771827	0.270192031	0.504941497
CDHR3	-0.229517676	2.025802316	2.043008767	0.152906943	0.365312074
GSC	-2.214525829	2.026286806	15.01762261	0.000106512	0.002155577
POPDC2	-0.518949424	2.026588849	5.629643631	0.017659221	0.091695325
RGS7	0.277664891	2.027293947	0.779207576	0.377383601	0.612229829
C1H11ORF70	0.378763369	2.028836603	8.371664072	0.003811165	0.031356359
ARHGDIG	-0.035050428	2.029407257	0.02771025	0.867791843	0.938915609
LOC112530938	-0.514002189	2.030280471	6.097688876	0.01353588	0.076804448
CISD3	0.172700178	2.031120628	1.021718597	0.3121117	0.550023148
LOC112530927	-0.189012735	2.03174722	1.563529057	0.211149246	0.438848349
GSTT1L	0.027820069	2.032072033	0.026714486	0.870167549	0.940243379
MAK	0.695606793	2.032410654	16.9141797	3.91E-05	0.000976522
APLN	-0.497691897	2.032768853	7.707479524	0.005499248	0.040742967
LOC107052414	0.092684891	2.034302518	0.164556304	0.684995817	0.835563601
LOC107054458	0.596849772	2.034438249	13.91955487	0.000190803	0.003442614
HOXD11	-2.784111567	2.034731405	7.324851258	0.006800759	0.047317756
LOC107053391	-0.632606557	2.036567238	15.75357524	7.22E-05	0.001615309
STEAP3	0.256220855	2.036764482	1.22065157	0.269232819	0.504027293
MINDY4B	-0.298334183	2.0375085	2.790199772	0.094842403	0.269696998
LOC770295	0.375718736	2.037666046	5.245860408	0.021999047	0.105537701
ASL2	0.451354601	2.039549087	10.62731551	0.001114294	0.012985624
CPS1	-0.1648494	2.040269027	0.776548057	0.37819895	0.61306903
CPEB1	0.204030557	2.040306421	2.159725695	0.141669979	0.348770123
HFM1	-0.497356322	2.0405845	6.32866969	0.01188014	0.069966965
LOC107054532	0.37389792	2.04075063	4.196557391	0.040506131	0.157683556
LOC769704	-0.531327325	2.042353702	10.45544942	0.001222882	0.013858482
CFAP61	0.077739276	2.043984221	0.191524632	0.661650698	0.820467329
PARM1	0.043384928	2.044075262	0.085221161	0.770342718	0.885380276

LOC107050574	0.161328503	2.044407148	0.621711518	0.430411985	0.657851497
SLC4A10	-0.415581867	2.044586948	3.459878326	0.062874941	0.208973648
ZMYND10	0.348081482	2.044604762	2.397347462	0.121541181	0.315600463
NCAM2	-0.982104244	2.046225508	11.52567285	0.000686416	0.009153141
MR1	-1.045755813	2.04645864	24.60196392	7.05E-07	3.61E-05
VGLL1	-0.298153893	2.047123403	3.463740431	0.062728265	0.208643883
NRIP3	-0.029927014	2.047344491	0.02754174	0.868190757	0.939193932
PTPRC	-0.088595244	2.047626479	0.315134443	0.574546444	0.765147438
BTBD19	0.376567941	2.048223586	6.829594512	0.008965962	0.057451285
LOC107055431	-0.0408468	2.048317503	0.036971346	0.847523397	0.928742508
AASDHPPT	0.891333096	2.048720863	31.66932361	1.83E-08	1.52E-06
LOC107052012	-0.360999998	2.048791854	4.203207353	0.040347599	0.15741623
LOC107054937	-0.315680252	2.050220135	2.651501443	0.103452143	0.28485693
LOC107052582	0.395493432	2.050821044	3.359629959	0.06681308	0.216895178
AMIGO3	-0.299281084	2.05108511	2.207379588	0.137351803	0.341718161
LOC100858131	-0.800262209	2.051613445	23.22804507	1.44E-06	6.42E-05
TAP2	1.256901897	2.052039141	53.40004095	2.72E-13	8.45E-11
GART-B	0.351091794	2.052104956	4.009176312	0.045253254	0.168242723
BST1	-0.079839068	2.052770341	0.192506126	0.660838925	0.820108576
LOC112533329	0.14072334	2.053288076	0.550360496	0.458170433	0.680801379
LOC112531036	-0.170207912	2.053452317	1.217068781	0.269936673	0.504595753
ADGRA1L	-0.054858112	2.053560116	0.115704549	0.733740773	0.863950227
GPR156	-0.179974028	2.055794023	0.773579103	0.379112101	0.613681567
MEF2B	0.023412717	2.057248676	0.019501816	0.888937401	0.949773712
LOC107055390	-0.14422231	2.058117504	0.917715983	0.338075396	0.575168594
CES1L1	-0.609763926	2.058519382	9.238355761	0.002369977	0.022537402
LOC417328	-0.277615126	2.060697537	2.28900258	0.13029363	0.330300674
UGT1A1	0.164414003	2.061209794	0.529500708	0.466816848	0.686608642
IQUB	-0.611285869	2.061361952	13.55772252	0.000231338	0.004023954
ATP6AP1L	0.207921966	2.061563262	1.426835195	0.232281632	0.463704852
ABCG5	0.304305226	2.061724433	2.879934338	0.089689679	0.261211269
IRF9	-0.114920169	2.062436228	0.697473288	0.403634011	0.635396312

LOC112530949	-0.482976225	2.064194739	3.839274134	0.050065191	0.179873956
LOC107054924	0.176668538	2.064274884	1.308678581	0.252634045	0.487280718
LOC107054253	0.191763483	2.064567237	1.360761151	0.243405611	0.476360529
LOC107053437	-0.06376202	2.065103611	0.183967118	0.667985898	0.824166458
LOC769155	0.023115956	2.067598326	0.018850594	0.890795673	0.950833083
LOC112531386	0.049676618	2.068165357	0.035989778	0.849536602	0.929957743
LOC100859149	-0.140989176	2.070245872	0.168370434	0.681564134	0.83319824
RAB39A	-0.591239385	2.070683022	16.4636624	4.96E-05	0.001186087
LOC100857473	-1.182429323	2.071018909	49.14591608	2.38E-12	5.51E-10
SRRM3	-0.50227062	2.071814176	2.869454001	0.090275491	0.262036199
PGF	-0.152017379	2.072019113	0.522388382	0.469824641	0.68897286
NXNL2	0.086489344	2.0727472	0.300519333	0.583557029	0.770936649
EGR1	0.61816274	2.074286083	7.023427621	0.008045008	0.053336266
LOC107051134	-0.635138636	2.07496251	3.130012284	0.076863161	0.236806463
LOC107052132	0.539665334	2.077254398	10.56459081	0.001152745	0.013292521
LOC107053751	-0.191385701	2.078592311	1.262837288	0.261114713	0.496033549
LOC101748504	-0.260905709	2.078721706	1.959765343	0.161538417	0.377305131
TSPAN8	0.238840313	2.078799091	0.96525823	0.325865617	0.563899329
ADAM8	-0.337701688	2.079701703	2.9800236	0.084298043	0.25060963
BAAT	-0.089189009	2.079895649	0.191120146	0.661985961	0.820748868
ASB12	0.051449129	2.081319251	0.043300854	0.83515981	0.921947852
ACSBG1	1.078654684	2.08165193	36.96109991	1.21E-09	1.35E-07
RAB7B	-0.056774356	2.082619421	0.15530667	0.693514835	0.839813077
KCNQ4	-0.073935782	2.082727933	0.134558531	0.713751642	0.851503174
CNN1	-0.278598176	2.083511947	1.641450622	0.200126553	0.426288988
FSTL3	0.101988327	2.083604867	0.188564914	0.664113721	0.821860352
LOC425940	0.416281728	2.085936276	3.038857761	0.0812931	0.24464664
HAL	-1.251291289	2.086017056	27.27040154	1.77E-07	1.11E-05
HPD	-0.333987166	2.086255556	3.049685368	0.080752786	0.243766729
ACCSL	-0.318556377	2.08735878	3.915727654	0.047836303	0.174301674
LOC395991	-0.094586883	2.088874601	0.345716273	0.556548119	0.75311398
SPEF2	0.02397084	2.089795711	0.017467439	0.894854227	0.951827123

THY1	0.407432526	2.091427793	7.556792475	0.00597843	0.043260805
TCFL5	0.261652695	2.091744125	3.040379461	0.08121693	0.244534238
LOC107053811	-0.664512207	2.093155438	8.904972324	0.002843952	0.025488686
FAH	0.062404045	2.093559115	0.076465094	0.782146223	0.892834971
TTC25	0.043143799	2.093644109	0.083668431	0.772386277	0.887047473
LOC112533159	-0.00821533	2.094420881	0.002530946	0.959876543	0.982787361
GDF2	0.240792017	2.09542162	1.410045345	0.235048866	0.467274603
LOC112533440	-0.267625172	2.096145868	2.162521272	0.141412489	0.348396842
A4GALT	-0.20443364	2.09767791	1.471291626	0.225142015	0.454911306
LSM10	0.510813338	2.098507438	10.27309274	0.001349843	0.014877209
CA8	0.324325814	2.100253586	3.23841381	0.071930247	0.226571418
NANOS1	0.057515572	2.1003761	0.099243704	0.752739113	0.874740262
VEPH1	-0.022907335	2.100736845	0.012125603	0.912317129	0.959684907
LBX2	-0.505710262	2.101175002	5.188453959	0.022737435	0.107909031
MGP	-0.509750354	2.102252673	6.109137854	0.013448471	0.076493121
ETV7	-0.099197214	2.102722024	0.439385323	0.507419283	0.716891343
CFAP70	0.721440378	2.103340714	20.27949179	6.69E-06	0.00023427
TMEM184A	-0.18404189	2.103348434	1.604439119	0.205275256	0.432110059
ADHFE1	-0.273536977	2.10536837	2.604462521	0.10656328	0.290534586
LOC107050235	-0.04851001	2.105614866	0.099471991	0.752464188	0.874606076
LOC112532478	0.955222887	2.106448503	21.99276581	2.74E-06	0.000110108
GLYATL3	-0.08248111	2.106885391	0.179863257	0.671490782	0.826344165
CACNG2	-0.443924646	2.107109401	2.475388483	0.115640868	0.305948797
CFAP44	0.121444026	2.107328878	0.591847104	0.441705743	0.666446726
FAM19A4	-0.411775533	2.108824468	7.088217551	0.007759238	0.052035633
STK17A	0.271272017	2.10916377	3.01093607	0.082704518	0.247481876
HPGDS	-0.665540633	2.110453583	6.213958833	0.012674687	0.073419493
SLAMF8	0.006302597	2.111396835	0.001606134	0.96803206	0.986863736
ABHD8	0.071675324	2.111818838	0.197070615	0.657095824	0.817086963
DYTN	-0.149955215	2.112855639	0.796316998	0.372196813	0.606626244
MYOD1	-0.101286232	2.114879632	0.107049809	0.743528214	0.869767312
BARL	0.10343893	2.114938158	0.228856664	0.632372894	0.803057713

CFAP45	0.130470432	2.115882849	0.758694733	0.383737312	0.618452249
PKD2L2	-0.297508878	2.11619794	1.830245486	0.176098553	0.396131467
LOC112532547	-0.158595979	2.116970448	1.155174307	0.2824686	0.518116242
NFATC1	0.198144232	2.11772348	1.560425427	0.21160294	0.43919354
SYCN	0.396390439	2.119060477	5.383995549	0.020322284	0.100207722
GALNT15	1.004045785	2.119823043	23.66560711	1.15E-06	5.31E-05
CA3B	-0.379392776	2.12024549	6.410226658	0.011346493	0.067889369
ARHGAP25	-0.286851835	2.120554271	4.510565807	0.033686096	0.139940597
S100A16	-0.279561098	2.120676328	1.708085467	0.19123396	0.415358344
ADGRB3	-0.457928457	2.12112436	5.082053738	0.024174718	0.112607009
LOC107051264	-0.284289574	2.121134869	4.460257757	0.034692262	0.142421116
C4H4ORF50	-0.238746554	2.123038097	0.930496668	0.334733899	0.572087272
C12H3ORF67	-0.17509507	2.123613728	1.446679187	0.229061553	0.459827725
SLC46A2	0.176706159	2.124374744	0.961482292	0.326813698	0.564378194
LOC107055361	-0.399151838	2.126600689	5.787570958	0.016139875	0.086664858
SPAG1	0.262719652	2.126709466	2.495608078	0.114164274	0.303152969
LOC107054283	0.326244174	2.127507776	3.990795927	0.045749448	0.169512987
RDH16	0.097154119	2.128734456	0.375741899	0.539890957	0.740788063
FCN2	-0.59279804	2.129387748	14.91039254	0.00011274	0.002249467
SLC51AL	0.427186893	2.130418996	5.513922421	0.018865692	0.09543036
LOC112532141	-1.425692558	2.130485995	10.71231047	0.00106425	0.012562584
RLBP1	-0.326127196	2.130946332	3.451367127	0.063199475	0.209555471
NKIRAS1	0.192282826	2.131148595	1.391754647	0.238109005	0.470793722
MYOZ2	-0.330577537	2.134162236	1.702046999	0.192020492	0.416187723
KIF9	-0.01228024	2.134681365	0.005964738	0.938439213	0.973338622
KCNK3	-0.394715746	2.135267029	2.29485543	0.129803296	0.32942409
TTC16	-0.333975208	2.138858905	3.403240912	0.065068457	0.213542373
OTX5	1.049356891	2.139159076	7.55699027	0.005977774	0.043260805
TPPP	0.426425888	2.139584073	6.019941757	0.014145114	0.079034921
NIPAL3	-0.253892672	2.139872278	3.513870158	0.060857138	0.204535173
GAREM2	0.071487632	2.141746056	0.098136103	0.754077953	0.875492324
SCEL	-0.627633758	2.143409705	14.03120792	0.000179802	0.003280084

YF6	-1.158046567	2.143800893	36.44223654	1.57E-09	1.70E-07
LOC107053451	-0.449387846	2.145346594	5.526412308	0.018731476	0.094985034
FAM161A	0.746085422	2.14562841	17.10500706	3.54E-05	0.000898142
LOC101749943	-0.198153553	2.146264298	1.143540266	0.284905493	0.520848201
SLC12A3	-0.397186006	2.146821492	2.736131469	0.098101998	0.274842017
LOC107052326	0.336408641	2.147342387	5.100325198	0.023921358	0.111825554
SH3GL2	-0.15096522	2.147507452	1.332559598	0.248350373	0.482536945
GRIN2C	0.320313963	2.148894022	1.516215864	0.218193045	0.447286229
MIR6559	-0.314975624	2.149223186	2.701342945	0.10026376	0.278548061
LOC107049603	-0.07867975	2.150959898	0.255808009	0.613014848	0.790261455
CARD11	-0.095459646	2.151225036	0.188376237	0.664271511	0.821910498
TM6SF1	0.163573535	2.151923847	1.426184092	0.232388208	0.463789928
TMPRSS2	-0.22210073	2.153466176	1.369672654	0.241868134	0.474797763
SLCO2A1	0.024134852	2.154386276	0.019909107	0.887791231	0.949358246
PON2	-0.328895736	2.155307915	5.30185335	0.021302746	0.103586491
IFI30	-0.385234534	2.157132361	3.052097987	0.08063292	0.243496918
LOC107053877	0.243585138	2.159173853	1.726205373	0.188896233	0.412513273
TCTN1	-0.463598345	2.16076467	11.61502683	0.000654211	0.008831043
AMHR2	-0.320920756	2.161349971	2.803011451	0.094087438	0.268739593
LOC771657	-0.048835521	2.162924195	0.086249306	0.769000664	0.884767126
CIDEA	0.226090216	2.163182478	0.820346257	0.365078948	0.600737409
LOC769756	-0.424836667	2.164478635	1.399658742	0.236780717	0.469065191
UGT8	0.545842262	2.16468075	7.634320553	0.005726795	0.041919225
FABP6	0.219329258	2.165173188	2.185996227	0.139270924	0.344807055
PLAU	0.38574212	2.165407492	5.097782389	0.023956452	0.111926015
DLL3	0.053472685	2.166222353	0.099839319	0.752022542	0.874401564
LOC425214	-1.192572965	2.166494268	29.43725341	5.78E-08	4.21E-06
LOC112531039	2.880724513	2.16708686	58.35080294	2.19E-14	7.68E-12
LOC107051752	0.065508518	2.167547363	0.068436543	0.793626921	0.898770199
LOC101749541	0.503433848	2.167957743	7.221479843	0.00720363	0.049205365
KCNA5	0.138045559	2.16848229	0.742002421	0.389020185	0.622950073
PTPRE	0.033577525	2.169333586	0.055143715	0.814343019	0.911441598

ONECUT3	-0.12152332	2.170164511	0.547090388	0.459509003	0.68155946
LOC107050024	-1.371482396	2.171017345	31.14836868	2.39E-08	1.90E-06
C2H8ORF46	-0.407906573	2.17251505	2.965578547	0.085054027	0.252037461
TMEM17	0.237351401	2.172699275	2.38353236	0.122620003	0.317392968
ENPP6	0.198796053	2.173014308	1.279034794	0.258078576	0.492597491
NKX2-3	0.702480319	2.173401083	4.041297086	0.044399684	0.166444744
ELF3	-0.238137237	2.173615571	1.685994587	0.194129813	0.418721657
IL1R1	-0.245718579	2.173931653	2.34592909	0.125610651	0.322171313
LOC112533555	-0.803613868	2.174977583	11.7142497	0.000620233	0.008496494
CACNA1I	-0.550432714	2.175122718	5.297489642	0.021356182	0.103815649
LOC101747727	-1.083306324	2.176218308	27.40370421	1.65E-07	1.05E-05
CEMIP	-0.197456466	2.17654671	1.282032419	0.257521483	0.492411155
LOC769510	-0.300686146	2.176748416	4.418075664	0.035560118	0.144576691
AMDHD1	-0.376141782	2.176870005	4.028365229	0.044741275	0.166867109
LOC107054274	-0.041553722	2.177299502	0.070340637	0.790841458	0.897267524
LOC107053519	0.072745277	2.179274426	0.268290397	0.6044809	0.785137502
LOC112532527	0.275974742	2.179503241	3.301433683	0.069219444	0.221567349
KLHL40	-0.057495292	2.180266781	0.100061587	0.75175574	0.874361717
LOC100859067	0.960724615	2.180506734	16.04494308	6.19E-05	0.00142756
PRR16	0.29821458	2.181696545	3.522521803	0.060540267	0.203636568
STKLD1	0.026104932	2.182058318	0.027387604	0.868556742	0.939356373
SGCA	-0.30071753	2.18215659	3.262699905	0.070872347	0.224788833
SPHKAP	0.81381156	2.182678719	14.90971107	0.000112781	0.002249467
CFAP73	0.154191203	2.182775836	1.013127183	0.314154827	0.551930556
C2H8ORF34	0.342281075	2.183930223	4.582102607	0.032307548	0.136033958
MSLNL	-0.048144602	2.184513251	0.054311681	0.815723615	0.912676418
POPDC3	0.267820848	2.185497349	1.983690827	0.159002125	0.373982271
LOC107053980	0.235838722	2.18707912	1.159729339	0.28152168	0.517301982
CRB1	-0.934363753	2.187718395	14.34480525	0.000152199	0.002862918
LOC112530305	-0.214768181	2.188923888	1.479943027	0.223783436	0.453101568
KLF-4	0.291973368	2.191132112	1.169249015	0.279555601	0.514855494
LOC100859910	1.507393393	2.191695235	49.91624549	1.60E-12	3.83E-10

SFT2D3	-0.318582333	2.19203037	3.160571837	0.075436755	0.233768701
LOC112530218	-0.19371799	2.19237939	1.551035792	0.212982595	0.441341589
WNT2B	0.762899616	2.193303061	9.949649219	0.0016088	0.01709026
CHSY3	-0.021740529	2.193499923	0.011537444	0.914461742	0.960946929
LDHD	-1.088400071	2.194195655	16.16960812	5.79E-05	0.001346054
FAM150B	-0.377977926	2.194202616	5.137437776	0.023415182	0.110179244
NHEJ1	0.724398159	2.194531255	20.72698855	5.30E-06	0.000194537
EPHA1	-0.41715355	2.19701268	5.22090619	0.022316919	0.106751424
LOC101749434	-0.119705211	2.197140097	0.394387337	0.5300022	0.733281956
LOC101751514	-0.102503811	2.19769243	0.508354184	0.475852186	0.692872111
MREG	-0.663126871	2.198817551	11.62714196	0.000649963	0.008795353
ARL10	0.297139084	2.20028002	2.305414696	0.128923868	0.328091904
POU6F1	-0.357983551	2.204199097	5.369768339	0.020488699	0.100789696
SGIP1	0.047861064	2.20590416	0.075921381	0.782902668	0.893053877
CACNG4	-0.522675292	2.206216637	6.458933474	0.011039589	0.066589601
GRASP	-0.219879028	2.2066994	1.507925446	0.219455918	0.448737316
LOC112531000	0.26377899	2.207216418	1.112694048	0.291497071	0.52872402
LOC101749699	-0.516249373	2.207654036	7.347110776	0.006717062	0.047000783
LOC107057197	-0.205603343	2.207735291	1.248619641	0.263816282	0.498861977
AGT	-0.250808087	2.210226167	1.255899641	0.262428662	0.497588898
LOC107054117	-0.507545765	2.210371526	6.216781318	0.012654496	0.073372
MYBPH	-0.063930128	2.211252881	0.141655542	0.706640695	0.848162533
LOC107051941	0.0878355	2.211446857	0.201897885	0.653193269	0.815005705
LOC107054254	-0.255197784	2.212240807	2.35305789	0.12503752	0.321282385
TRIM67	-0.354711744	2.213143195	2.654480047	0.103258516	0.284514214
PDE4C	0.295538781	2.214156212	3.355635395	0.066975359	0.217115747
RASEF	0.148883408	2.215190661	0.835181648	0.360778468	0.597237914
LRRC70	0.24796864	2.215260165	1.816050664	0.177784097	0.39861525
FAM149A	-0.331943492	2.215965528	5.5717475	0.018252536	0.093383174
LOC101751837	-0.077626744	2.216293784	0.35317046	0.552323873	0.750840072
CRYAB	0.000344238	2.216804374	4.08E-06	0.998387677	0.999301702
LOC112532928	-0.463225408	2.21709838	6.660821037	0.00985555	0.061521452

TMC3	-1.064643973	2.218967398	17.31755074	3.16E-05	0.000816944
LOC107055026	0.252567367	2.219241048	1.31036319	0.252328908	0.486934484
MYOCD	-0.780845386	2.219260214	7.769395631	0.005313883	0.039858242
LOC107055111	-0.815665201	2.219442503	2.75205654	0.097129425	0.273160945
LOC112530086	0.80388053	2.219987535	11.75935521	0.000605382	0.008338694
ST6GALNAC1	-0.443937649	2.220613654	5.977314765	0.014491049	0.080286265
KLF2	-0.68198238	2.222025541	9.224682286	0.002387741	0.02260647
DNAJC28	0.900263155	2.222702053	34.77439024	3.70E-09	3.65E-07
RHOG	0.137258014	2.222864818	0.538059317	0.463238123	0.684376308
LOC107052850	-0.238949734	2.223311552	2.072316256	0.149993569	0.361281572
LOC107054431	-0.112640665	2.224960628	0.699655312	0.402899539	0.634882978
LOC107049672	0.229915197	2.224969345	1.191291647	0.275069171	0.509944029
LOC107052170	0.301848585	2.225293036	3.018684703	0.082310198	0.246526086
LOC112531488	0.072613343	2.22557539	0.21471786	0.643094459	0.80933991
SCN2A	-0.501329906	2.225806678	5.403236153	0.02009945	0.099346821
LOC107053399	-0.363188605	2.226033151	4.062494751	0.043845678	0.165249801
LOC112532059	0.745531002	2.226826188	11.19058661	0.000822133	0.010438431
SYNC	0.012268406	2.228050691	0.005046798	0.943365269	0.975792553
C7orf49	-0.16850199	2.228929809	1.089328479	0.296620044	0.533680327
CHRND	-0.097718091	2.229225641	0.195903319	0.65804809	0.817900243
MMACHC	-0.314225926	2.229306271	4.112289767	0.042572686	0.162461399
FKBP1B	-0.166455975	2.229442517	0.964621872	0.326025141	0.564056745
LOC107053900	0.135443032	2.230907611	0.346947126	0.555846397	0.752912164
LOC107054128	0.06239646	2.234453797	0.123822593	0.724924999	0.858175601
LOC422147	0.206573266	2.234692761	1.776790928	0.182543876	0.404983077
MYOG	0.270425846	2.234822174	0.961424217	0.326828308	0.564378194
TMEM182	-0.712975826	2.235995178	6.734166626	0.009458341	0.059676764
PSD2	-0.128837315	2.236151141	0.391593032	0.531463223	0.733850867
TPD52L1	-0.569968785	2.236175492	11.57604591	0.000668068	0.008995963
NRG3	-0.021610118	2.237607109	0.019304073	0.889498274	0.950126199
NKX2-8	1.018560428	2.238158362	31.14888925	2.39E-08	1.90E-06
RNF180	-0.107890893	2.238225401	0.479708695	0.488554295	0.702150803

LOC101748259	-1.200681677	2.24072302	14.45203458	0.000143774	0.002747745
RADIL	0.103120626	2.241149337	0.433378717	0.510335704	0.718341124
LOC107054051	0.564478515	2.241727131	12.86855578	0.000334149	0.005333102
LOC112533573	-0.212055886	2.242197548	1.637035971	0.20073263	0.427192891
LRRC73	-0.087155503	2.244038487	0.367483723	0.54437906	0.744408787
FAM81A	-0.307640675	2.244631147	2.565281377	0.10923312	0.295071008
BAALC	-0.566164323	2.248126654	8.082569088	0.004469304	0.035221456
LOC112531021	-0.10377515	2.248216338	0.198952539	0.655567658	0.816258385
YBEY	-0.145796725	2.248412281	1.199573166	0.273407005	0.50812201
LOC101750863	-0.186041148	2.24843242	1.066130616	0.301821087	0.539481423
LRRC3C	-0.240872581	2.248585197	2.343220945	0.125829141	0.322611175
KLF13	-0.402681032	2.248682004	3.445900117	0.063408874	0.210008866
TMEM61	-1.229126503	2.24881202	14.61351894	0.000131964	0.002562122
SLC25A34	-0.1286641	2.249366937	0.813274777	0.36715383	0.602224508
ECE2	0.125307263	2.249484722	0.598526683	0.439140716	0.664502993
CHAT	-0.31206113	2.251258018	1.57025891	0.210169418	0.438045609
CCDC148	-0.351298075	2.252118248	4.426656424	0.035381755	0.144106186
WIPF3	-0.17774574	2.252465948	1.672006952	0.195989935	0.420708925
MTUS2	0.295060952	2.252560529	2.600401276	0.106836662	0.290914656
ARHGEF5	-0.262484537	2.25330349	2.304342224	0.129012885	0.328267671
NTN4L	0.560687861	2.253960122	4.140999145	0.041856457	0.160809713
CDK15	0.025232113	2.25485563	0.025080347	0.874167019	0.942721236
LOC107055350	-0.551498622	2.255127315	4.900549004	0.026848159	0.120706681
HIST1H2B7L2	-0.225313123	2.255782887	1.003535111	0.316456624	0.55408531
RASL10B	0.180495148	2.255801577	1.30096644	0.254036768	0.488623613
FES	0.037887447	2.257373464	0.050769069	0.821730399	0.916282016
GRIN2B	-0.167378985	2.259093659	0.547283384	0.459429831	0.681523442
FAM212B	-0.245006843	2.260710342	1.956478729	0.161890413	0.377912718
SORCS3	-0.111623879	2.260900525	0.276136795	0.59924455	0.781804074
LOC112531269	0.42648429	2.261067322	2.846641123	0.091565056	0.264241143
MAATS1	-0.310045573	2.262005677	4.081065973	0.043366292	0.164270796
GCNT7	0.504184189	2.263024779	9.44582664	0.002116301	0.020892566

LOC107052777	0.098966676	2.264292911	0.091720197	0.762001566	0.880756463
TRPV3	0.368831804	2.264373849	3.11045513	0.07779122	0.238215389
BTK	0.070613405	2.264526512	0.232030079	0.630022624	0.801303429
LOC107054986	0.267386566	2.264713699	1.699534202	0.192348905	0.416679993
ACOT1L	0.114745914	2.265407339	0.418573357	0.517649771	0.723314877
FAM160A1	0.179536785	2.26545305	1.098178021	0.2946663	0.531647862
POU4F1	-0.302813839	2.267279609	0.970553881	0.324542081	0.562614828
LOC107052000	0.042449364	2.268506893	0.076184592	0.782536111	0.892887345
AMPD1	-0.318472508	2.268861797	2.220257954	0.1362103	0.339648505
LOC107054884	-0.217060627	2.271679744	1.733855708	0.187919236	0.411143602
LOC107055354	-0.251981694	2.272960204	1.925942847	0.165203002	0.382444485
TSPAN15	0.218189317	2.273873157	1.861141635	0.172493097	0.391715969
FOXJ1	-0.045294094	2.274444611	0.077411884	0.780835874	0.891855827
LOC112530550	0.285760805	2.27450599	2.230047089	0.135349723	0.33839038
LOC107053814	-0.031757284	2.276344942	0.049225847	0.824415874	0.917226694
PNPLA4	-0.029115044	2.276815397	0.044289607	0.833315722	0.921086197
BAIAP2L1	0.186865451	2.276965958	1.096291657	0.295081373	0.532
CACNB4	0.168881472	2.278562122	1.320469951	0.250507727	0.484782388
FNDC4	0.286566838	2.279068975	3.370641046	0.066367926	0.216110872
PDGFB	-0.22318206	2.279443783	2.437860267	0.118437685	0.310681277
ELOVL2	-0.463680461	2.279586198	6.908992631	0.00857633	0.055802097
IL20RA	-0.409491065	2.279640048	6.345673669	0.011766791	0.069573321
WDR73	-0.203081271	2.281642827	1.491332729	0.222009794	0.451566275
LOC107053537	0.160478925	2.283066424	1.107646257	0.292594173	0.529837913
PROCR	-0.419558367	2.283441247	9.312511213	0.002275943	0.021900962
TEX2L	0.355861283	2.284099685	7.191541976	0.007324801	0.049846815
TRPC5	-0.269199202	2.284460341	1.913257275	0.166601884	0.384492847
ABCA7	0.159768175	2.286088087	0.8260447	0.363418744	0.599203952
NECAB2	-0.031783408	2.286216518	0.029873066	0.862778559	0.936001285
HRH1	-0.115732862	2.287375365	0.306757074	0.579677026	0.76882734
VSNL1	0.627705921	2.287561601	10.46172963	0.001218731	0.013840042
LOC100858381	0.099972815	2.289181934	0.394717831	0.529829876	0.733281956

LOC107052653	-0.613769915	2.289893605	12.06889224	0.000512702	0.007388296
NTN4	-0.83472758	2.289911602	18.52142727	1.68E-05	0.000489296
MAEL	0.164604192	2.290267716	0.826643783	0.363244813	0.599097263
TMEM51	0.188964017	2.29114649	1.534737968	0.215402751	0.444471699
CSMD1	-0.736753301	2.29119948	8.79697771	0.0030173	0.026536434
BMP3	0.610415309	2.291644778	7.21704371	0.007221454	0.049286202
ADAM9L	-0.257911993	2.292146632	1.903572011	0.167679042	0.385518881
CLC2DL3	1.103837159	2.29333681	5.212019832	0.022431261	0.107108701
LOC112531964	-0.318410068	2.293572176	2.45400669	0.117225304	0.308917811
LOC107053345	0.064963345	2.293995705	0.165062648	0.684537593	0.835303382
LOC424918	-0.507284363	2.294513416	6.574209553	0.010346694	0.063567662
CYP27A1	-0.018714171	2.294794589	0.013791828	0.906512487	0.95723949
KCTD21	-0.113602768	2.296642548	0.416912403	0.51848173	0.724065331
LOC107053885	-0.044775532	2.298689227	0.050305158	0.822533124	0.916456624
CORO2A	-0.141643559	2.298982761	0.446294931	0.504099704	0.714589993
LOC107055206	-0.610406792	2.301200928	13.03042668	0.000306471	0.005017886
CCDC27	0.632621289	2.301303507	16.30739871	5.39E-05	0.001269564
UROC1	-1.494772821	2.302936206	39.72277862	2.93E-10	3.82E-08
FAM43B	-0.63408401	2.30468725	11.79126623	0.000595092	0.008228778
LOC112532088	0.022617535	2.304782921	0.023158865	0.879044677	0.945898519
LOC107054625	-0.059263967	2.304830142	0.141763012	0.706534592	0.848162533
GABBR2	0.295566615	2.305865223	2.311952644	0.128382684	0.327322135
LOC107050328	0.883514872	2.307087161	8.337678659	0.003883118	0.031789408
KCNJ3	0.089015475	2.307522901	0.280940517	0.59608543	0.779387029
CLC2BL1	-1.966480883	2.307683733	62.83853698	2.24E-15	9.72E-13
DRP2	-0.096592372	2.308090462	0.252026874	0.615651495	0.792054659
MIR1713	0.196156305	2.308124345	1.734258696	0.187867935	0.411143602
OCA2	-0.573968395	2.308275724	9.324013425	0.002261701	0.021840542
IGSF10	0.461954382	2.308486991	4.922573864	0.026507998	0.119700631
DRC1	0.070494376	2.309043576	0.244723808	0.620814957	0.795404761
LOC107051628	-0.018386865	2.309626866	0.014776737	0.903247808	0.955693331
LDB3	-0.232524327	2.3101362	1.130666803	0.287633153	0.524408532

LOC112532613	-0.05947232	2.310519472	0.090128937	0.764013303	0.881657566
LOC112533470	0.083982804	2.311287922	0.263796949	0.607523518	0.78708759
TPH1	-0.080584592	2.312520809	0.240626658	0.623753875	0.797751439
UNC13D	-0.212531433	2.313320047	1.539366748	0.214712088	0.44383734
LOC112533333	-0.303588974	2.31334611	2.771262169	0.09597046	0.271525777
TPH2	-0.141374385	2.313828682	1.172966437	0.278792555	0.514181965
SLC25A48	0.344141533	2.316100297	5.725052996	0.016724532	0.088746266
RASGRF2	-0.890466663	2.31708964	17.58535118	2.75E-05	0.00073023
TERT	-0.21760661	2.317308785	1.757783796	0.184901185	0.407466051
LOC107051926	-1.388433355	2.319430481	43.55995046	4.11E-11	6.82E-09
STOM	-0.005627826	2.319487787	0.001405538	0.970093912	0.987864058
MANEAL	0.202919722	2.320291727	1.798276563	0.17992099	0.401619454
GCG	0.468415181	2.320945377	1.064763381	0.302131277	0.539918567
ABCA12	-0.630281232	2.321115532	11.49410771	0.000698172	0.00924249
FHL2	0.160160752	2.322948561	0.504097373	0.477705283	0.694090848
BICDL1	-0.006604264	2.323781122	0.001342815	0.970768509	0.988350633
INSRR	-0.054683097	2.324497071	0.090024483	0.764146033	0.881657566
RHOBTB3	0.237851153	2.325106391	1.270989539	0.259581125	0.494147086
KCNIP2	-0.204632861	2.325236748	0.994125909	0.318736053	0.556418603
MEI1	-0.673094223	2.325561762	15.01109942	0.000106881	0.002155577
AGXT2	0.051296083	2.325710852	0.131954005	0.716414601	0.853085451
LOC100859467	0.248305626	2.327843004	1.235370074	0.266365223	0.501721582
UTS2RL	-0.093435403	2.328468095	0.325038036	0.568595585	0.761412788
CCDC40	0.174132033	2.329125153	1.726501617	0.188858291	0.412485157
PDPN	0.119147173	2.329777353	0.68062772	0.409370655	0.639836068
CD101	0.04729771	2.330682939	0.042452573	0.836759493	0.922908871
MYOZ3	0.180277713	2.331479146	0.983905164	0.321236582	0.55859114
MYO3B	-0.269856084	2.331683273	2.915460631	0.087734358	0.256743528
SLC18A1	-0.186119883	2.332385523	1.87381813	0.171038424	0.389811256
ZNF804A	0.032504569	2.332543796	0.03920342	0.84304621	0.926094935
B3GALT2	-0.487067703	2.332710591	6.040548869	0.013980937	0.078480313
IL1RL2	-0.128866869	2.332942252	0.69587425	0.404173489	0.635646128

FTO	-0.092971005	2.333795813	0.315946625	0.574053818	0.765001241
SLC28A3	-0.235909079	2.333857109	0.90515102	0.341404278	0.578399406
EPGN	0.178507968	2.334641983	0.677823662	0.410337147	0.640661251
LOC107052253	-0.0145833	2.334712949	0.012153549	0.912216552	0.959684907
LOC112532872	0.403730005	2.334959702	1.540788039	0.214500545	0.443585079
SSPN	-0.38251346	2.335006817	3.021759909	0.082154268	0.246148666
KIAA1614	-0.200705639	2.337880199	0.880426537	0.348084881	0.584582233
B3GALT5	-0.209210831	2.337907862	1.64504035	0.199635312	0.425538311
GNLY	0.289664626	2.342455439	2.525734814	0.112002595	0.29977185
SPATA17	0.986801922	2.342824668	39.69025155	2.98E-10	3.83E-08
PDE8B	-0.011261582	2.342917456	0.003610182	0.952088106	0.979652938
FAM184B	-0.563846384	2.343471757	10.14341497	0.001448176	0.015749994
FOXL1	-0.085324727	2.34384809	0.228811257	0.632406668	0.803057713
LOC107054226	-0.232035723	2.34389603	2.03046482	0.154173449	0.36713461
KLF4	0.471027752	2.344322435	9.942981231	0.001614638	0.017119694
DLX5	-0.564416366	2.345154307	3.691163472	0.054701447	0.190379083
SNTG1	0.101458033	2.345530736	0.433070543	0.510486113	0.718444149
DEXI	-0.010599608	2.34908164	0.005402416	0.941407343	0.974802494
FNDC7	0.176217208	2.353305757	1.645590349	0.199560172	0.425468079
CALN1	-0.353161792	2.353388778	3.715472753	0.053910356	0.188894078
LOC100859602	0.096270346	2.35345707	0.194813873	0.658939913	0.818699506
LOC101751352	0.812209437	2.354211429	23.76012223	1.09E-06	5.09E-05
SOX12	0.180276482	2.354312183	0.629606063	0.427499849	0.65520655
COA4	0.106811226	2.354314255	0.564532259	0.452439869	0.67549848
GABRG2	-0.520579047	2.355994042	8.689463387	0.003200548	0.027631177
LOC107053886	-1.570756034	2.357630156	49.75801057	1.74E-12	4.09E-10
F10	-0.077362028	2.358614203	0.194545914	0.65915972	0.818787137
LOC107049117	-0.575199481	2.358640867	1.714198803	0.190441502	0.413964983
LOC101748752	0.118241546	2.362233403	0.692499873	0.405315381	0.636520766
OPN1LW	-0.30158196	2.362260469	2.078114094	0.149424693	0.360578284
TMCO4	0.220188161	2.36275668	1.635603878	0.200929702	0.427501713
MYL2	-0.990807958	2.36449028	10.58892527	0.001137671	0.013211274

ENO4	-0.335798037	2.365765196	3.04496915	0.080987655	0.244254373
CLEC3B	-0.405546268	2.365912599	5.311539281	0.021184631	0.103225674
RBBP8NL	0.103302812	2.366082964	0.441410539	0.506442433	0.715978975
MYO3A	-0.623649656	2.366515336	11.55164975	0.000676892	0.009062858
FICD	0.227631068	2.368729431	2.657263257	0.10307795	0.284111836
RHBG	-0.0002946	2.369528713	3.82E-06	0.998440073	0.999301702
PIK3AP1	-0.01321099	2.371471175	0.008639182	0.925945508	0.967012404
GRIK3	0.531721281	2.37281218	6.617400668	0.010098712	0.062377741
LOC107051951	-0.472805017	2.373187113	7.767518045	0.005319409	0.039858242
LOC101747820	0.135792768	2.374163708	0.561970956	0.453467203	0.676255467
SLC16A7	-0.251475674	2.374649326	2.508536853	0.113231017	0.301931031
TRPV4	0.416356024	2.376048164	3.121009971	0.077288866	0.237584212
LOC107050186	2.121182664	2.376074173	47.50645026	5.48E-12	1.19E-09
LRGUK	0.274767055	2.377379146	3.696258497	0.054534627	0.190053395
GDNF	0.347990549	2.377461555	3.593744213	0.057997432	0.198120768
LOC112530273	-0.141365206	2.378907472	0.61973568	0.431145526	0.658483352
RIPPLY3	0.801507813	2.379559907	5.795170346	0.016070258	0.086434566
LOC420368	0.43443292	2.380490617	6.391695977	0.011465543	0.06840664
LOC107052824	-0.127138989	2.382359592	0.281744483	0.595560094	0.779009646
IRX4	-0.349277692	2.382725921	1.769492306	0.183444923	0.405878522
LOC112532597	-0.013187513	2.38358971	0.009578917	0.922033982	0.964486558
PHACTR1	-0.550045704	2.384009958	9.03083422	0.002654633	0.024187147
DDTNFR23	-0.218323765	2.384032777	2.004283805	0.15685536	0.371214577
AMPH	0.400543397	2.384381549	4.19120161	0.040634286	0.158032896
HS3ST6	-0.29787675	2.384856066	2.810069388	0.093674324	0.268071478
SLC35E4	0.052692521	2.386654586	0.141128694	0.707161506	0.84841433
CHRM3	-0.045161055	2.387300535	0.057072364	0.811184498	0.909337575
SLC16A4	0.066146393	2.38828062	0.138372877	0.709903981	0.849641566
ST6GALNAC2L	0.660856927	2.38842277	14.82561495	0.000117923	0.002335044
PKP3	-0.159583839	2.389822754	0.999446548	0.317444464	0.555342192
LOC107052834	-1.040482736	2.390952979	14.55599454	0.000136055	0.002623253
LRIT1	-1.065309225	2.394401956	7.410844396	0.006483189	0.045954099

ACP5	-0.01531567	2.39483263	0.010560146	0.918151413	0.962365851
LOC112533169	-0.317713196	2.395721134	4.701405063	0.03013797	0.129676545
KIAA1257	-0.616497658	2.39587091	8.270591058	0.004029239	0.032644571
C1QTNF5	-0.458494392	2.396326631	7.22641407	0.007183856	0.049131485
PRPH	0.407360148	2.396362792	5.583264393	0.018132893	0.092977494
LOC416169	-0.042544217	2.397586251	0.087198988	0.767768729	0.884179034
PKHD1	0.169541532	2.397736427	0.626037166	0.428812644	0.656275148
PPP1R1B	0.103745327	2.399329552	0.425363119	0.514273011	0.721306034
SHISA4	0.101377467	2.399824977	0.265010878	0.606698325	0.786666377
LOC112531373	-0.068230429	2.403739795	0.144099237	0.704239352	0.846723552
LPAR6	0.20484265	2.40641566	2.441439929	0.118167709	0.310416611
SPON2	-0.129669696	2.40655336	0.362680371	0.547021381	0.746680782
LOC107054475	0.053222463	2.406682743	0.09045826	0.763595379	0.881504277
COCH	-0.05207071	2.406737227	0.035798723	0.849931754	0.930275273
LOC112533566	-0.251064181	2.407050491	2.474485761	0.115707281	0.305948797
LRTM2	-0.171105052	2.407149307	0.947888678	0.330257299	0.56803427
LOC107051962	-0.180024909	2.407389646	1.067237548	0.301570253	0.539384621
RTN4R	0.085185582	2.407395255	0.363275061	0.546692953	0.746542119
KCNJ16	-0.428404314	2.408749373	3.360891073	0.066761935	0.216817351
DMB2	0.208441346	2.409790865	1.729326999	0.188496869	0.412023908
CLIC5	0.301233	2.410232694	2.204098608	0.13764433	0.342238962
CTSS	-0.236714329	2.410449102	1.305087507	0.253286019	0.48783004
LRRTM2	-0.219786409	2.41080997	1.038738819	0.308114971	0.546031773
C5	-0.256726385	2.411398113	1.613658437	0.2039783	0.430812852
LOC101752211	0.29093246	2.411738781	0.89073457	0.345278324	0.582007255
LOC107054877	0.0185213	2.412158948	0.007285353	0.931979742	0.970083458
CDKN1AL	-0.39269499	2.412545087	4.65016236	0.03105061	0.132470261
CPA6	-0.063640265	2.415215734	0.04721834	0.82797626	0.918504892
ITGA2B	-0.165682819	2.416125285	0.687140714	0.407138636	0.637801433
LOC112533175	-0.006525802	2.416126745	0.001847744	0.96571318	0.985815656
LOC101747455	-0.641242849	2.41629296	8.581908552	0.003395193	0.028839264
C6orf132	-0.21281319	2.418016168	1.204683237	0.272387655	0.507144022

LOC107052595	0.204448902	2.418108029	2.337856316	0.126263202	0.323522422
LOC112530963	0.243633312	2.418914941	2.487529505	0.114751725	0.30435842
LINGO3	0.214752539	2.419599478	1.562542078	0.211293399	0.438848883
LOC112533547	-0.030018369	2.420039251	0.046332111	0.829573228	0.91892477
CADNL	0.39194487	2.42081782	5.777408549	0.016233458	0.086959286
FAM19A5	-0.207992336	2.422449667	1.79403193	0.180435683	0.402367417
PLEK2	-0.152317203	2.423464348	0.684984762	0.407875506	0.638408775
LOC107053406	0.29876002	2.423540281	2.839951541	0.091946987	0.26504255
LOC101751325	0.334829262	2.423900448	5.414417215	0.019971119	0.098983362
LOC101748065	-0.050776484	2.424900242	0.114022739	0.735609976	0.86485154
FAM102B	0.236771207	2.425092763	2.455447927	0.117117755	0.308693362
PLPPR5	-0.019252661	2.425343748	0.015694275	0.900304422	0.954481623
EML5	-0.547401595	2.425887481	8.202476012	0.004183326	0.033546117
LOC107053630	-0.050173836	2.426320265	0.057347456	0.810738607	0.909184251
LOC112530965	-0.317045269	2.429256938	2.776482016	0.095658078	0.27101475
CFI	-0.176286963	2.430187827	1.611116779	0.204334888	0.431123296
RPS6KA2	-0.520309226	2.430405471	10.87926621	0.000972468	0.011800852
SH3TC1	-0.272956558	2.43088147	3.090820428	0.078735101	0.23984377
FAM189A2	-0.153209797	2.431581357	1.291931395	0.255692324	0.490546484
LOC112533252	-0.254148768	2.432374161	1.904782905	0.167543935	0.385423663
LOC416695	-0.97067085	2.433507901	35.27684823	2.86E-09	2.87E-07
LOC112531065	-0.163362772	2.433689461	1.029739091	0.310219956	0.54812855
ARMC10	-0.021364541	2.43472052	0.018192082	0.892708312	0.951217859
BTBD18	-0.148232861	2.436204338	0.978519952	0.322564479	0.560071595
IGFN1	-0.589794422	2.43793909	10.45905682	0.001220496	0.013840977
TMEM243	0.153311367	2.439614137	1.237300592	0.26599193	0.501190566
FAM124A	-0.269561219	2.439829096	3.553560876	0.059417811	0.200970211
NEUROG1	-0.067312382	2.440877293	0.037353348	0.846747409	0.928324913
NT5C1A	-0.492636052	2.441007288	11.68177048	0.000631154	0.008583179
NFATC2	-0.064233634	2.441154223	0.2472173	0.619041313	0.794312827
NUP210L	0.647248668	2.441874401	15.2720295	9.31E-05	0.001956206
GRIA3	0.135259634	2.442140318	1.076233866	0.299541596	0.536806465

METAP1D	0.055783008	2.442297246	0.163906715	0.685584875	0.835837528
RTN4RL1	-0.075674719	2.442305912	0.156168573	0.6927088	0.839401545
MOB3C	-0.44292495	2.443018982	7.718060206	0.005467111	0.040577946
GLRB	-0.573214683	2.444144384	7.851634366	0.005077479	0.038573365
RGS4	-0.728234792	2.444329699	8.285834256	0.003995554	0.03241955
LOC101748439	-1.202267641	2.446954385	13.40688855	0.000250702	0.004310658
SHANK2	-0.188421001	2.447045209	0.694189947	0.40474287	0.636107348
LOC101752064	0.411152254	2.448959944	7.874452463	0.005013805	0.038284066
OSR2	-0.294572714	2.450450151	1.284301127	0.257100843	0.492030236
LOC112532183	-0.313755478	2.451174581	1.772973949	0.183014457	0.405516403
DYNLT3	-0.087179874	2.451597349	0.374318503	0.540659673	0.741379577
IKBKE	0.516173456	2.453549627	9.944827318	0.00161302	0.017119694
TRIM35	-0.220771081	2.454425158	2.287313183	0.130435546	0.330525502
KIAA1456	-0.518413703	2.454475961	6.111739778	0.013428688	0.076469976
LOC107052249	-0.4370694	2.454824538	3.553839325	0.059407842	0.200970211
CFAP74	-0.130656728	2.45747807	0.534055533	0.464906802	0.685549012
LOC112530302	0.139815633	2.458587507	0.200195466	0.654563117	0.815663468
GPR62	-0.068870153	2.460051868	0.246510639	0.619542832	0.794589033
LOC107057099	-0.71561092	2.461290152	16.63854461	4.52E-05	0.001104062
CAV2	0.115934002	2.461460165	0.829193773	0.362505761	0.59885988
FAM163B	0.121331774	2.461744358	0.463789147	0.495858414	0.707539907
RHBDD2	0.293614122	2.462181224	3.930261865	0.047424559	0.173377276
C8B	0.389097419	2.462378412	3.690113925	0.054735877	0.190453031
PEAR1	0.040381886	2.462733834	0.058655102	0.808634374	0.907749244
LOC107054784	0.163531486	2.463011916	1.413430557	0.234487735	0.466452573
PLXNA4	-0.40362033	2.463156963	3.022523881	0.082115579	0.24613496
LOC771200	-0.98939525	2.464081023	23.95723444	9.85E-07	4.76E-05
SNX20	-0.789110185	2.464184323	23.49227675	1.25E-06	5.75E-05
GPRIN2	-0.214403126	2.466570974	3.036213194	0.08142566	0.244857316
LOC430422	0.117565412	2.467691733	0.689914729	0.406193386	0.63730625
LOC416263	-0.0796249	2.468114701	0.251870682	0.615760942	0.792054659
LOC112532889	0.057055033	2.468837019	0.090363365	0.763715719	0.881519512

MAPK13		-0.188247664	2.469792332	1.72493041	0.189059628	0.41259631
EDN1		0.088287818	2.470180982	0.224124416	0.635915131	0.804921806
LIPML3		-0.442540987	2.470455252	5.882419975	0.015292789	0.083045919
	02/mar	-0.241284471	2.47055342	2.355969431	0.124804278	0.32083337
IL6		-0.182309865	2.471657147	1.147887876	0.283991728	0.520055692
DCDC2B		-0.15580378	2.472735556	1.146058555	0.284375755	0.520280525
BCO2		0.513681925	2.47314031	11.03559121	0.000893792	0.011049335
LOC421238		0.088379171	2.473344313	0.324186783	0.569102339	0.762029375
GRIN2A		-0.110916309	2.473365891	0.399768774	0.527208696	0.731098862
LOC112531303		-0.340634639	2.47511797	4.153233387	0.041555101	0.160025552
OPRD1		-0.203795011	2.476286714	1.606637214	0.204965151	0.431788703
LOC101749222		-0.173174254	2.476817514	1.242691345	0.264952996	0.500091941
CACNA1S		-0.329095944	2.478407582	4.217150684	0.040017306	0.156744768
LOC107054477		0.089291166	2.478578762	0.20240494	0.65278661	0.814807227
MISP		0.532234302	2.47866755	13.13856576	0.000289279	0.004798477
LOC112530621		-0.340481883	2.48019082	0.850856163	0.356310246	0.592829636
ERAP1		0.179750721	2.480515224	1.207175362	0.271892257	0.506705893
LOC112533222		0.177450836	2.481198365	1.522643612	0.217219874	0.446236333
P2RY2		0.086730142	2.482076278	0.242458745	0.622435893	0.79668502
LOC101749321		-0.393367937	2.483368947	5.360856298	0.020593659	0.10100503
ARMC2		0.340698755	2.484383688	5.256404303	0.021866149	0.105197013
SYT13		0.100623688	2.484891572	0.271851904	0.602092196	0.783443547
SLC13A4		-0.086737708	2.48505802	0.350486944	0.553837612	0.75180646
KCNH1		0.346423299	2.486206625	4.325192171	0.037552174	0.149614536
KSR2		-0.017677817	2.486284648	0.011934092	0.913009541	0.960095348
C1QTNF1		0.216675386	2.487024772	2.193969488	0.138551843	0.343867819
LOC107053494		-0.302712474	2.487215961	4.686151617	0.030406676	0.130535311
RAMP1		-0.691346031	2.487324305	13.36698042	0.000256093	0.004385032
HHLA2		-0.738950531	2.4883097	17.64651334	2.66E-05	0.000715208
LOC112532577		0.120617695	2.489300751	0.731534187	0.39238635	0.625832839
LOC107053870		-1.62978611	2.489466673	10.57059212	0.001149009	0.013292521
DNAI1		-0.166939748	2.49005034	1.087481492	0.297029903	0.533991812

APBB1IP	0.08611938	2.490786744	0.288864584	0.590949052	0.776434578
LOC100858220	0.038528662	2.491381117	0.094362442	0.758702787	0.878744052
LOC100858196	-0.017533763	2.493504382	0.011725495	0.913770157	0.96052689
PTGER4	-0.202642691	2.494900489	1.184193422	0.276503949	0.51165495
TRPC6	-0.456198375	2.495248725	5.261165252	0.021806414	0.10513491
ANXA8	-0.275604224	2.495751423	1.656766448	0.198040478	0.423698995
MAFA	-0.13697682	2.496566438	0.606809732	0.435991537	0.662319124
ANKRD34B	-0.617872614	2.497358773	15.46096574	8.42E-05	0.001814074
GAD2	-0.321568113	2.497542089	2.252332418	0.133413179	0.335316002
PLCXD3	-0.246196854	2.499149078	1.205268941	0.272271124	0.5070418
GBGT1	0.009557896	2.49948314	0.003313637	0.954095765	0.980523198
LOC101751112	0.195019882	2.501571835	0.952531654	0.329075723	0.566653518
LOC101749795	0.076366154	2.504650376	0.28705105	0.592116514	0.776980894
LMO3	-0.116565932	2.505121562	0.346874833	0.555887565	0.752912164
SIAH3	-0.099777543	2.505551926	0.18490969	0.667187445	0.823907648
C26H1orf74	0.525625279	2.50790578	10.71669778	0.00106173	0.012550988
COX16	-0.243174001	2.507986375	3.321170483	0.06839312	0.219956537
P2RX3	-0.07380569	2.510197845	0.233459294	0.628970594	0.800641717
FAP	-0.187907573	2.510827852	0.999277648	0.317485359	0.555354692
PPEF2	0.391691562	2.51246967	3.474203183	0.062332736	0.207858446
TMEM150A	0.167144194	2.513351926	1.752201548	0.185600206	0.4081865
LOC107053647	-0.043968193	2.513583508	0.107543651	0.742958173	0.869560491
ATP8B1	-0.071731897	2.514248798	0.160623735	0.688582907	0.837878558
LOC107054744	-0.168897508	2.514747811	1.363598102	0.242914871	0.475966207
LOC101749214	0.004867829	2.514856271	0.001208055	0.972273444	0.988971101
KIF25	0.005130608	2.518995675	0.001203741	0.97232297	0.988971101
TGFA	-0.068336758	2.520639357	0.300224057	0.583741991	0.771028614
DGAT2	-0.39962673	2.520687678	7.78609319	0.005264992	0.039631953
LOC101750739	-0.045001744	2.52130527	0.115873594	0.733553733	0.863886574
NKX2-2	0.289057136	2.521377996	2.769314212	0.09608732	0.271716249
LOC107052229	-0.81252174	2.522686642	19.53184659	9.89E-06	0.000321739
ABCB9	-0.043442844	2.524299099	0.078782468	0.778954203	0.890625824

LOC112529956	0.181095773	2.525765259	1.054815994	0.304400522	0.542324662
LOC107052592	0.08994522	2.525776188	0.392762356	0.530850953	0.733558252
LOXL4	0.246105204	2.527840777	1.720705558	0.189602249	0.413123016
NCMAP	0.052180107	2.528799685	0.144171154	0.704169035	0.846723552
SNX10	-0.236856017	2.528915373	2.297718376	0.129564197	0.329212302
BF2	1.483451841	2.531355833	62.62506223	2.50E-15	1.03E-12
DISP2	0.137800289	2.531693937	0.534080892	0.464896203	0.685549012
ALDH1A1	-0.832173927	2.53179058	7.124734267	0.007602768	0.051314008
FLNC	-0.443616463	2.53220404	4.175578086	0.04101057	0.158919708
CLIC6	-0.529430201	2.533537836	11.5601095	0.000673819	0.009047748
GMIP	-0.29273163	2.535405783	4.517411566	0.033551556	0.139557848
SOST	-0.189067355	2.535736746	0.922773621	0.336747762	0.573918636
DHRS12	0.136046897	2.536128106	0.556121133	0.455827333	0.678361107
CYP26C1	-0.662257071	2.536654573	11.37622498	0.000743901	0.0096766
ZMYND12	-0.226928743	2.537014789	1.779775149	0.182176939	0.404441654
NEGR1	-0.08652269	2.538081864	0.3071228	0.57945114	0.768631077
DPEP1	-0.124254202	2.538459626	0.721808913	0.395551193	0.628430253
LOC107053389	0.007545029	2.539278602	0.001439992	0.969729764	0.987658702
LOC107049165	0.116376446	2.539702433	0.462816089	0.496310803	0.7079291
LOC107054771	-0.588753373	2.540881562	13.35281359	0.000258035	0.004413689
HCN2	0.284654569	2.542592608	3.4785445	0.062169399	0.207585929
PEX11A	0.096194736	2.542873158	0.492708327	0.482721663	0.697663204
HSD17B11L	-0.179621674	2.546462172	1.870139321	0.171459124	0.390103934
ZNFX1	0.204478543	2.547462105	2.130646256	0.144379873	0.353116945
CD9	-0.143078918	2.548023627	0.471424576	0.492332526	0.704892692
PLXDC1	0.279578934	2.548693539	3.040432304	0.081214286	0.244534238
LHFPL5	0.316597714	2.548880982	5.165770645	0.023036235	0.108894354
GRK7	-0.285841792	2.549155076	2.08996239	0.14826972	0.358421955
LOC107050827	-0.003299931	2.550330914	0.00058647	0.980679399	0.991769376
LOC107054540	-0.374344228	2.550584573	5.941587658	0.014787692	0.081245901
MIR1600	-0.213438064	2.550859941	2.535444274	0.111315551	0.298955018
LAMA3	0.031912056	2.550890551	0.057287676	0.810835406	0.909185459

LOC107054901		0.310715029	2.552310385	6.327265968	0.011889547	0.069997315
MYT1L		-0.459680998	2.552507529	5.594034274	0.018021743	0.092787167
LAPTM5		-0.370092767	2.553583921	3.315080454	0.068646961	0.220622214
LOC100859648		0.235757996	2.553737678	2.36093361	0.124407715	0.320113987
NT5DC4		0.928025272	2.55384096	17.8943484	2.34E-05	0.00064148
NFKBIE		0.105614055	2.55522486	0.413175486	0.520362138	0.725779864
NRTN		0.542967191	2.55618404	5.944366494	0.014764397	0.081172118
CCSER1		-0.110345143	2.557431062	0.562747084	0.453155514	0.675974433
TRPM2		-1.073086267	2.558135293	25.82646453	3.74E-07	2.12E-05
TRPC4		-0.12168496	2.558174295	0.370223528	0.542882451	0.743026497
CRLF1		0.027391808	2.558765576	0.03233023	0.857304803	0.933498024
MYRIP		-0.16584184	2.560079593	0.869911821	0.350979721	0.587351013
ENO2		-0.072979165	2.560689666	0.218770361	0.639978443	0.80739441
ERICH3		0.208771032	2.561115664	1.933509766	0.16437497	0.381439871
LOC107049826		-0.090223961	2.562319637	0.536850821	0.463740786	0.684929547
CAV1		0.102333291	2.56355562	0.423308682	0.515290677	0.722011561
TMEM9		-0.014370592	2.56368915	0.010640449	0.917841898	0.96231374
	04/mar	-0.590538229	2.564238855	6.336737801	0.011826219	0.069779198
TUBA4A		-0.679240097	2.565245935	17.54443027	2.81E-05	0.000740137
SNCB		0.110276602	2.566019009	0.519091834	0.471229337	0.68998743
LOC107049644		0.410878583	2.566134224	3.122186985	0.077233063	0.237584212
FOXA1		0.191715878	2.56722064	0.91456462	0.338906184	0.575913753
APOBEC2		-0.04078918	2.568304933	0.045656869	0.830800758	0.919603556
LOC107051627		-0.20776581	2.568502957	1.588504844	0.20753988	0.435096028
GDPD2		0.09362137	2.568919374	0.34266079	0.558297375	0.753969093
CNTN1		-0.472990064	2.568925479	6.761306183	0.009315555	0.058956713
LOC101748673		-0.669634506	2.569208588	18.32573102	1.86E-05	0.000531861
LOC112532912		0.002662968	2.569426711	0.000412272	0.983800474	0.992668433
MAP3K6		0.391025746	2.570374513	5.844976373	0.015621584	0.084473598
GIMAP5L		0.647306529	2.571175224	9.825750992	0.001720854	0.017899277
RHOQ		-0.341180394	2.573096049	5.421964995	0.019884969	0.098794432
KLF5		-0.115049399	2.573886389	0.378811767	0.538239845	0.739416986

CYHR1	0.273139	2.574641666	3.928197094	0.047482824	0.173440526
TTC9	-0.59439802	2.574652953	13.91380016	0.000191388	0.003449388
LOC107051159	0.283302598	2.57666547	2.892962288	0.088967206	0.259520542
SLC17A9	0.707660104	2.576716275	23.9238939	1.00E-06	4.79E-05
LOC107054742	0.206214507	2.577610479	2.622318506	0.105370379	0.288401462
ATP7B	0.206561285	2.579126372	1.483037064	0.223299949	0.452729068
CIART	0.394249039	2.579722976	7.939211353	0.004837491	0.037388877
LOC768392	-0.276703682	2.580294128	2.28107707	0.130960899	0.331481554
ALC	-0.7719721	2.581995684	3.301293928	0.069225333	0.221567349
LOC107053422	-0.198404484	2.58215666	2.440599365	0.118231043	0.310434308
MTMR9LP	0.183325721	2.582655143	0.867339663	0.351692857	0.588180299
LCA5L	-0.162022146	2.582737479	1.22414832	0.268548076	0.503585025
LOC419602	-0.046114835	2.582774113	0.094396303	0.758660844	0.878744052
GIMAP1	-1.932966157	2.582912013	149.4345856	2.30E-34	7.58E-31
CDH8	0.104252115	2.583391408	0.490247255	0.48381703	0.698351687
LAD1	0.317220264	2.583633213	2.386716515	0.122370414	0.316903551
LOC112530469	-0.732665388	2.58442836	8.900112451	0.002851531	0.025528804
SLC44A3	-0.145987704	2.58470469	1.136879345	0.286312705	0.522676831
LOC101749407	-0.434107672	2.586677323	2.758466526	0.096740927	0.272657543
RXFP2	0.820150683	2.586744851	12.9866045	0.000313727	0.005106216
LOC112530036	-0.103856522	2.587928053	0.408327809	0.522819466	0.727587475
ATP2B2	-0.304658357	2.588789131	3.411086681	0.064759788	0.212869018
PLEKHA2	-0.130427742	2.588837279	1.011940316	0.314438448	0.552020128
NFIA	0.424869642	2.588849362	6.174849466	0.012957894	0.074449071
CD3E	-0.663148272	2.58887894	20.41301857	6.24E-06	0.000222433
SLC6A13	-0.115126332	2.589008196	0.772605452	0.37941224	0.614007514
EPHA10	-0.519189983	2.589724675	9.209849196	0.002407165	0.022701934
ITGB4	-0.841338367	2.589993443	29.74809712	4.92E-08	3.66E-06
LOC107050990	0.079209558	2.590238898	0.239249316	0.624748825	0.798386013
TMEM173	-0.458162667	2.591062148	8.986035522	0.002720506	0.024611462
FRMPD3	-1.121691734	2.591590418	12.09150374	0.000506521	0.007330523
RNF128	-0.100451732	2.592688967	0.473116225	0.491557042	0.704487482

LOC101749170	-0.627856712	2.592745846	9.549892123	0.001999612	0.020161526
LOC101747532	-0.067744568	2.593007421	0.20833943	0.648072067	0.812187803
TMEM163	-1.013561641	2.59352829	24.38137224	7.90E-07	3.92E-05
LOC419851	0.30649012	2.594029029	1.932299038	0.164507138	0.381634511
GPR142	0.063401693	2.594377433	0.21782563	0.640701697	0.807749496
NFIX	0.200398987	2.59569478	1.718666557	0.189864779	0.413257267
LOC107053557	0.51266207	2.596173943	10.56924053	0.00114985	0.013292521
PARD6B	-0.279161709	2.596234442	3.458947499	0.062910347	0.208973648
KCNK9	0.417446012	2.597329238	4.873106375	0.027278355	0.121841837
OVCH2	-0.145849252	2.597575218	0.964025634	0.326174702	0.564154365
LOC107055587	-0.362798523	2.601012317	2.027988789	0.154424845	0.367348446
ECHDC1	0.121829993	2.601679922	0.919936893	0.337491543	0.574603067
LRRTM1	-0.301451079	2.601709373	1.811082021	0.178378486	0.39929506
SCN3A	-0.0919499	2.6026533	0.126203789	0.722401017	0.856852067
STS	0.17515467	2.603786202	0.483372133	0.486898836	0.70132356
ENTPD8L	-0.594840302	2.603817115	14.24523638	0.000160466	0.002986963
LOC418543	-0.792824071	2.604313468	2.604048046	0.106591145	0.290534586
NRAP	-0.354191998	2.60435541	3.029908824	0.08174261	0.245496376
PACRG	-0.088566977	2.605670009	0.485172914	0.486088495	0.700617701
LOC427799	0.017831967	2.606118095	0.009226244	0.923478211	0.965427825
MT4L	-0.170241547	2.606371131	1.278596814	0.258160097	0.492671516
SULT6B1	-0.471195349	2.607140897	10.6230475	0.001116868	0.013006418
KLHL42	-0.364844303	2.608092298	7.745981135	0.005383221	0.040190968
KCNG3	-0.310210543	2.60907313	2.74513561	0.097550801	0.273831189
LOC107051620	-0.203108872	2.610645184	1.678270645	0.195154402	0.42015775
KCNC4	0.396010583	2.611930733	5.354346992	0.020670674	0.101291226
NOL4	-0.549503284	2.612296966	4.013204944	0.045145257	0.168055361
LOC112532304	0.325272608	2.612609763	3.871267708	0.049119407	0.177366655
NT5C1B	-0.010026404	2.612679242	0.006245891	0.937008006	0.972589841
AKR1B10L4	0.940559371	2.613493547	12.50506987	0.000405849	0.006189294
FBLN5	-0.667000069	2.61379334	13.24646129	0.000273095	0.004586857
TRIM25	-0.3654144	2.613891793	4.792305517	0.028587138	0.125601973

MCOLN3	-0.113096412	2.6145491	0.67694326	0.410641293	0.640908895
SHD	0.060523293	2.614794463	0.135800729	0.712491868	0.850454908
PARP14	0.449984265	2.614846492	9.16801262	0.002462819	0.022986774
LOC100857706	0.665664294	2.615687977	13.39397743	0.000252434	0.00433138
LOC100857512	-0.022120494	2.615952655	0.024915733	0.874577219	0.942879391
DNAH5L	0.388795909	2.616917456	7.183026588	0.007359645	0.050001224
LOC430303	0.229417281	2.617109537	2.774912575	0.095751885	0.271140468
LOC107049174	-0.050919698	2.617455509	0.022801878	0.879973423	0.946151989
LMO1	0.016401359	2.61748894	0.010634357	0.917865337	0.96231374
BDNF	0.075222283	2.618026937	0.294566793	0.587308775	0.773658452
KCNK15	0.06465316	2.619760593	0.184743984	0.66732764	0.824018934
GPR146	-0.210654267	2.620164199	1.752271072	0.185591482	0.4081865
AMER3	0.091938628	2.620603527	0.191643168	0.661552527	0.820458761
IMMP2L	-0.060286952	2.62137901	0.186015835	0.6662535	0.823471421
LOC101748371	1.329282306	2.621716181	21.03708402	4.50E-06	0.000169238
TMEM37	-0.032495493	2.621921702	0.02282832	0.879904375	0.946151989
ANK1	0.008239147	2.623661768	0.001182651	0.972566405	0.989117842
RSPO2	-0.29408269	2.623964628	0.83126315	0.36190753	0.598210789
SCARB1	-0.139713517	2.624374279	0.602534891	0.437612482	0.66361749
PIGL	-0.039651993	2.625369698	0.112087983	0.737779405	0.866307619
CHAC2	0.012467063	2.625557283	0.00928312	0.923243438	0.965243711
RUNX2	-0.347125259	2.625870363	1.879582718	0.170381582	0.389194822
MTFR1L	-0.027258377	2.625921565	0.046482002	0.82930201	0.918867551
ENPP3	-0.537072674	2.62750907	10.7028647	0.001069698	0.012608794
C9orf116	0.180810346	2.628518358	1.878409019	0.170515083	0.389244365
ZSWIM1	0.198347689	2.629116857	2.099331706	0.147363536	0.35717587
BF1	1.369173417	2.630238515	36.19168617	1.79E-09	1.89E-07
DOCK8	0.01877873	2.632731929	0.015570887	0.90069506	0.95464974
LOC770248	0.385142917	2.633651092	4.028867385	0.044727959	0.166867109
SYT17	0.064679418	2.633713051	0.238978656	0.624944758	0.798467738
LOC101752077	0.250911748	2.6354991	2.157705537	0.141856376	0.349020135
HIST2H3D	-0.515101029	2.635645527	8.525632311	0.003501791	0.02953458

RAB2B	-0.072376727	2.635923309	0.212015427	0.645192299	0.81068921
GIN1	0.089194235	2.636324522	0.270410236	0.603056715	0.784202485
LOC107049581	-0.255802476	2.636473966	0.485715625	0.485844716	0.700418329
ABLIM3	-0.815955933	2.637328772	33.32125646	7.81E-09	6.95E-07
HHATL	-0.270555049	2.637412498	1.564275954	0.211040235	0.438848349
LOC107053920	-0.069526058	2.637835204	0.172895776	0.677550959	0.830409323
FAM221A	-0.155320577	2.638378262	0.987448883	0.32036669	0.557728522
UBXN10	0.252955857	2.638657155	4.344020747	0.037139119	0.148542664
KCNIP3	-0.062819756	2.639485039	0.121178161	0.727760175	0.859813626
HMGCLL1	0.052261622	2.639942283	0.115005348	0.734516029	0.864368252
ETFBKMT	0.041694963	2.640466648	0.100492847	0.751238998	0.874169982
ENTPD3	0.026253162	2.640778892	0.027841795	0.867481304	0.93881523
ESM1	0.103775781	2.641085007	0.444324853	0.505042385	0.71537165
DRCC1	0.051810446	2.642365883	0.147260461	0.701167229	0.844881865
MPP4	-0.033636093	2.643409218	0.07400264	0.785595675	0.894476669
KLF9	0.407632287	2.643607263	7.45680637	0.006319703	0.045213352
LOC107055055	-0.572025822	2.644747105	7.858741584	0.005057558	0.038475321
TSEN15	0.355608897	2.644954841	7.942366087	0.004829065	0.037368349
MICAL1	-0.241208136	2.645616104	2.634358507	0.104574292	0.286901837
CYP2J23	0.076104059	2.646304389	0.30673196	0.579692544	0.76882734
LOC101749362	-0.324440926	2.646519026	5.292322653	0.021419634	0.103862114
LOC112530066	-0.068694054	2.646786962	0.264688431	0.606917282	0.786666377
LOC107051098	0.064820972	2.647242211	0.187533866	0.664977134	0.822412532
RHNO1	0.211588121	2.648283279	2.797749249	0.094396734	0.269024818
C4H4orf54	-0.056886653	2.648542777	0.182934141	0.668863719	0.824430898
SCG5	-0.457440317	2.649831443	9.377135069	0.002197087	0.021506877
SLC4A11	-0.106841875	2.650949206	0.527461921	0.46767588	0.687291828
AAREL	0.522354764	2.653707764	8.016655513	0.004634908	0.036199874
LOC424430	0.481901417	2.65388055	12.63341971	0.000378911	0.005876519
FGF14	-0.365378486	2.654347961	4.370463683	0.03656704	0.147261539
LGR6	-0.103747425	2.655677886	0.504818714	0.477390439	0.693818648
GPR182	0.074794486	2.656203077	0.232922314	0.629365392	0.800882185

TP53INP2	-0.050582871	2.657323225	0.09096594	0.762952736	0.881102924
LOC101750669	0.077660691	2.657791871	0.140194081	0.708088145	0.848914958
LOC770141	-0.352512414	2.658080892	4.459091412	0.034715958	0.142421116
RBM20	-0.103964785	2.658654991	0.512117816	0.474223504	0.69207548
LOC419112	-0.074552466	2.658909116	0.224031978	0.635984778	0.804922157
PLAT	-0.064670905	2.659693546	0.295496391	0.586719652	0.773223761
ICOSLG	-0.281760464	2.659724621	4.505746763	0.033781143	0.140023865
AQP11	0.395637321	2.660364785	4.452440382	0.034851409	0.142552294
KCNRG	0.194398821	2.66124321	2.296948235	0.129628467	0.329223095
SMTNL1	-0.024148711	2.661339401	0.020827556	0.885249718	0.9486918
KCNN2	-0.214291825	2.661840618	1.628652916	0.20188947	0.428712251
NFIC	0.170856775	2.663206972	0.89544327	0.344006484	0.580874981
LOC107053031	0.348313518	2.663217687	1.154168866	0.282678157	0.518385053
FBXL16	0.028091476	2.664358627	0.022856076	0.879831944	0.946151989
TMEM82	-0.00824501	2.664420586	0.004021193	0.949437734	0.978504123
LOC101750341	1.096568147	2.664436486	47.13346363	6.63E-12	1.36E-09
ADRA2B	-0.43739447	2.664772351	7.304393625	0.006878621	0.047738381
ADAMTS13	-0.161389899	2.665088294	1.194346478	0.27445457	0.509412101
PATL2	0.140699784	2.665157477	1.361356194	0.24330258	0.476215528
MOK	-0.133079512	2.66741209	0.552330783	0.457366898	0.679975814
TSC22D3	0.050079769	2.668062244	0.141171017	0.707119627	0.84841433
GLA	-0.11771975	2.668148743	0.897482217	0.34345772	0.58056982
CCDC13	-0.134772518	2.668322893	0.723920269	0.394860993	0.627589842
GATA4	-0.025939481	2.669422513	0.006277124	0.936851035	0.972488253
DHRSX	0.00216201	2.671095741	0.000246472	0.987474176	0.99418059
GIPC2	-0.180074692	2.671569209	1.262286241	0.26121878	0.496116692
ABHD11	-0.325848131	2.673676524	5.719064315	0.016781671	0.088877503
TMEM109	0.671236992	2.675332124	16.42898471	5.05E-05	0.001204482
MAB21L1	-0.270232396	2.676174914	0.995272267	0.318457192	0.556167808
MBP	-0.070862553	2.67617598	0.253637914	0.614525079	0.791137476
KDM4C	0.176560513	2.67667565	1.614758356	0.203824212	0.430597946
LOC107054808	0.074148125	2.6767605	0.290337612	0.590004262	0.77575071

LOC769359	-0.033458826	2.676942154	0.04735626	0.827729147	0.918504892
LOC101747846	-0.430578299	2.677009803	8.79028967	0.003028382	0.026604583
DPF2	0.427263463	2.677429891	10.15177707	0.001441622	0.015699465
TSPAN2	-0.138751503	2.678659991	0.855620993	0.354967034	0.591551807
LOC112530196	-0.516102802	2.678923137	9.372495954	0.002202654	0.021535752
GATA3	-0.166601075	2.681530051	0.791306889	0.373705243	0.608421864
LMOD1	-0.848700846	2.683871299	4.307596223	0.037942539	0.15049741
PPFIA4	0.275458139	2.685193934	2.307610846	0.128741797	0.327932859
GPR137B	-0.207366768	2.686180276	1.704044313	0.191759917	0.416061371
LOC112532097	-0.491676336	2.687311391	10.99202351	0.000915049	0.011270302
BLEC3	-0.381563756	2.687444036	2.428599637	0.119139287	0.311623109
FOSL2	0.157566748	2.690381443	0.679897891	0.409621887	0.640051404
RGCCL	-0.031007999	2.691743348	0.042796282	0.836109338	0.922500949
LOC112530856	0.139522102	2.691922069	0.881250901	0.347859296	0.58438231
LOC107053022	-0.055153452	2.692812106	0.026632817	0.870364399	0.940243379
KCNG2	-0.046521947	2.692974898	0.101334955	0.75023348	0.873555895
TNS4	0.142018722	2.693054566	1.108159519	0.292482378	0.529693764
GPC6	-0.417034174	2.693629949	7.193834614	0.007315448	0.049816449
TP53I3	0.445685995	2.695080946	4.725795234	0.02971344	0.128430434
TLR21	-0.158252988	2.695407532	1.1022115	0.293781284	0.530848821
VSX1	0.066739153	2.695593591	0.132430087	0.715925628	0.852989434
SMIM8	-0.139345021	2.695832812	0.88829546	0.345939641	0.582540186
GCHFR	-0.450303041	2.696917427	3.836784601	0.050139588	0.180023331
NXPH2	0.435433694	2.698155465	6.679291027	0.009753941	0.061027035
PTRF	-0.480755851	2.698700593	3.289118923	0.069740436	0.222744348
MIR6565	-0.033965505	2.698897792	0.051568236	0.820356553	0.91568085
IRX2	0.089490509	2.698987534	0.232041859	0.630013937	0.801303429
HIST1H4I	-0.915471924	2.699888408	21.48584646	3.56E-06	0.000138334
GCNT1	-0.139533519	2.700190565	0.626011069	0.428822266	0.656275148
ATP2C2	-0.73117966	2.703750806	11.8243369	0.000584615	0.008118013
C5H11orf74	0.128766308	2.704661967	0.549367583	0.458576215	0.680973884
PPP2R2C	-0.157107964	2.704840679	1.044839386	0.306698624	0.544776458

GATSL3	0.233016893	2.706069273	2.533138728	0.111478271	0.299285106
RHOH	-0.155047978	2.706875769	0.997028755	0.318030532	0.555837854
KCNIP4	-0.063106872	2.708580829	0.169906848	0.680194608	0.832287498
LOC107057189	-1.817775155	2.708633233	42.32216922	7.74E-11	1.19E-08
MXRA8	-0.335972063	2.709230524	4.029860078	0.044701648	0.166867109
BMP2	-0.387244523	2.709391846	4.674877768	0.030606881	0.131086992
LOC768735	0.489240975	2.709498722	9.123136641	0.002523968	0.023411442
LOC771612	0.115795464	2.710047263	0.659306948	0.416804723	0.646908293
CHID1	0.33280366	2.710637537	6.544551673	0.010520584	0.064235586
SRD5A1	-0.107503367	2.711247614	0.764060001	0.382060935	0.616740878
CABP7	0.204386968	2.711403993	1.941231225	0.163534912	0.380007306
P2RX6	-0.308958766	2.713079698	5.160337612	0.023108405	0.10912074
DEPDC7	-0.118128446	2.713608085	0.719590396	0.3962783	0.629052616
RAB15	-0.240302237	2.714268608	2.801477619	0.094177478	0.268763511
LOC112529982	0.65172507	2.714426578	17.91028337	2.32E-05	0.000637195
SLC19A2	0.016046425	2.715762798	0.014654191	0.903647871	0.955902743
MAB21L3	0.094517564	2.716187122	0.267207024	0.605211507	0.785638636
PCP4	-0.155023637	2.716464818	0.870002157	0.350954712	0.587351013
B3GNT7	-0.198937456	2.716474546	1.720121633	0.189677389	0.41314838
NTRK2	-0.0478922	2.717932071	0.057387244	0.810674209	0.909184251
LOC112530905	-0.240869516	2.71854009	3.510934033	0.060965076	0.20464218
NME5	-0.174789667	2.718841533	1.847366528	0.174089994	0.393575338
PXDNL	-0.939796731	2.719603082	10.59812989	0.001132021	0.013166942
LRRC25	-0.29371384	2.721137803	4.47048697	0.034485162	0.141863334
FUT10	0.494085791	2.722755336	9.275112734	0.002322884	0.022196896
LOC107053812	-0.283227255	2.725640725	2.288651701	0.130323091	0.330324469
GCH1	-0.204603577	2.72583317	2.894629711	0.088875195	0.259344091
LOC112533010	0.200817916	2.727017636	1.30403689	0.253477154	0.48788941
MYH7	0.546588803	2.728664853	7.493013431	0.006193882	0.044492889
LOC107052340	0.130359329	2.732231035	0.987444889	0.320367669	0.557728522
SDR16C5	0.034325242	2.732730839	0.066409162	0.796638637	0.900812774
NRXN3	-1.251697312	2.733643183	35.55299178	2.48E-09	2.52E-07

GPR21	-0.112545526	2.734707187	0.657097766	0.417586415	0.647571808
TLR5	-0.10762846	2.734757149	0.41591588	0.518982008	0.724514206
C4	2.021927842	2.7348473	62.11647953	3.24E-15	1.30E-12
THEM4	-0.035837374	2.735771342	0.061707631	0.803817037	0.905139899
UNC5A	-0.036941794	2.736508301	0.039123156	0.843204877	0.926094935
LAPTM4B	0.123465206	2.737270672	0.806398362	0.369187223	0.603962551
DMC1	-0.342246413	2.73745295	4.920544822	0.026539147	0.119775551
PPP4R4	-0.018401001	2.737641269	0.01645617	0.897926122	0.95334891
SPATA7	-0.203156405	2.738388049	1.407295401	0.235505891	0.467742842
STYK1	-0.148907818	2.739017285	0.936705303	0.333126596	0.570880873
LOC101749129	-0.544141592	2.739256623	15.52265327	8.15E-05	0.001779113
NLGN4L	-0.056197689	2.740235681	0.080832476	0.776172419	0.889259549
AGPAT9L	0.040419825	2.742180261	0.033854922	0.854015753	0.932178863
ROBO4	0.091620414	2.74259683	0.390494904	0.532039374	0.734393751
SEMA3E	0.009574875	2.743248111	0.003792025	0.950897764	0.979408031
GSTA3	-0.18496221	2.743530285	1.05988689	0.303240984	0.541196355
LOC112531627	-0.418113752	2.744372464	8.36336253	0.003828615	0.031452747
FAM169B	0.221297305	2.744613136	2.447977498	0.117676404	0.309620278
TRPV1	0.520804641	2.745275559	7.513316869	0.006124449	0.044142708
LOC112530455	0.089070326	2.745510354	0.400456583	0.526853553	0.730729536
C20orf196	0.427184371	2.747694957	9.577202472	0.001970084	0.019937102
LOC100857609	0.700838704	2.751828715	18.13278163	2.06E-05	0.000577534
PRUNE2	-0.153656025	2.752881045	0.5446465	0.46051342	0.682557046
FAT2	-0.239088269	2.753550531	1.73156617	0.188211007	0.411617772
FLVCR2	-0.132697266	2.754106253	0.989403059	0.319888316	0.557247749
LOC112532692	0.399474336	2.755636582	9.221093853	0.002392426	0.022624922
MOV10	0.076332103	2.755766138	0.37891984	0.538181888	0.739399095
GNAZ	0.076698433	2.757916625	0.323822024	0.569319751	0.76207244
LOC417507	0.177172676	2.758222426	1.832484142	0.175834413	0.395645461
BCAN	0.073359943	2.7584194	0.256911871	0.612249723	0.789728713
SLC8B1	0.159786033	2.759286344	1.226156233	0.268155864	0.503250996
CCDC3	0.200405737	2.759327922	0.743821948	0.388439316	0.622372828

MPL	0.638205156	2.762894349	13.81856776	0.000201337	0.003597171
LOC107054133	-0.391092905	2.763356808	5.431017264	0.019782153	0.098521586
USP18	-0.318106386	2.763797757	5.987559099	0.014407125	0.079902002
FAM198B	-0.292363985	2.764143339	1.429802544	0.231796666	0.4635017
SS18L1	0.001489668	2.764391189	9.18E-05	0.992356287	0.996459368
LOC101750578	-0.111769449	2.766165623	0.309730356	0.577845697	0.767429454
SEC16B	0.019423941	2.766171708	0.005680179	0.939922743	0.974140501
LOC107052116	0.05249987	2.766583821	0.201354116	0.653630055	0.815062002
CDH18	-0.343619656	2.768247327	5.75179503	0.016471817	0.087830122
SELENOP2	-0.412628408	2.76907763	5.552723361	0.018451956	0.094002146
MIR1723	-0.011826392	2.769683844	0.007980134	0.928818386	0.968392732
LOC107055405	0.151709849	2.769947372	1.210646114	0.271204198	0.506356841
LOC112530773	-1.75620151	2.770595605	117.8886359	1.83E-27	2.74E-24
RUNX1	0.081259878	2.77165593	0.18962947	0.663225185	0.821295185
SRPX2	-0.136693054	2.772616955	0.976096333	0.323164463	0.560699202
LOC112530352	-0.538242381	2.772732637	6.197933517	0.012789956	0.073845165
LNX1	-0.004522036	2.772861287	0.00070889	0.978758837	0.990993103
C12orf75	0.234472678	2.773042519	2.617762929	0.105673325	0.288990288
AGTR1	0.319252534	2.773314018	2.547915734	0.110439862	0.297282502
SLC6A17	0.053355647	2.773938296	0.054139126	0.81601133	0.912856407
LOC107053808	0.297965816	2.775705816	3.43990889	0.063639202	0.210597532
ABHD14A	0.01996923	2.776775819	0.023966377	0.876970493	0.944345884
FSD1L	-0.210189445	2.777724222	1.779472305	0.182214138	0.404469666
FHL5	-0.410122711	2.778403321	3.052115398	0.080632056	0.243496918
LOC107050830	-0.148689915	2.779113049	1.031962796	0.309698109	0.547547486
CERKL	-0.398663515	2.779711486	6.781382817	0.00921135	0.058522304
SCNN1A	-0.108732945	2.780506601	0.827203765	0.363082338	0.599097263
LOC107049737	-0.263232305	2.782128974	3.227213598	0.072423819	0.22743014
LOC107054636	-0.190646837	2.782355405	1.622798186	0.202702061	0.429329698
LOC101751186	0.068417661	2.784184701	0.228764431	0.632441501	0.803057713
LOC107054026	-0.066200682	2.784995579	0.121206789	0.727729298	0.859813626
ZNF185L	-0.074574893	2.785097488	0.285793094	0.592929114	0.777327412

MAF	-0.007373137	2.785668732	0.002211368	0.962493157	0.983899168
LOC431499	1.236355742	2.787303447	37.71847371	8.17E-10	9.34E-08
LOC112530215	-0.271812787	2.788154873	2.231944286	0.135183642	0.338271869
LYRM9	-0.059189611	2.789653658	0.21335351	0.644151559	0.81017457
PPP1R3B	-0.411604006	2.789698552	5.45224309	0.019543218	0.097853209
PMLL	-0.410711572	2.792114868	9.180602746	0.002445935	0.022894115
LOC112531862	0.017909225	2.792353275	0.010413458	0.918719882	0.962372833
DHDH	0.052220594	2.792376264	0.059831846	0.806761913	0.906648561
FYTTD1L	-0.081079693	2.793732755	0.401738913	0.526192574	0.730547482
HINT2Z	0.205393553	2.794942204	1.591726646	0.207079618	0.434352468
LOC112532835	0.19714047	2.795355422	1.874456313	0.170965564	0.389793886
RSPH14	0.552155433	2.795611005	12.22336191	0.000471948	0.006933845
FOXA2	0.128793444	2.796610659	0.547250542	0.459443302	0.681523442
SLC1A1	-0.305046735	2.796791286	4.197765849	0.040477274	0.157645807
LOC107049688	0.915482679	2.797425188	27.47352169	1.59E-07	1.02E-05
SLC2A4RG	-0.004807205	2.798001951	0.001136895	0.973102125	0.989274401
LOX	-0.550418831	2.798834602	6.292812198	0.012122856	0.071089333
KBTBD3	0.131674888	2.799865184	0.918082371	0.337978983	0.575072378
WNT1	0.253747478	2.800782387	2.506102314	0.113406109	0.302348917
SYPL2	-0.033634376	2.800881849	0.060855618	0.805148677	0.905731091
CES1L2	-0.077230275	2.802309675	0.236137869	0.627009572	0.799801744
FHL1	0.008292462	2.802855842	0.003672747	0.951675229	0.979652938
STAMBPL1	-0.442910432	2.803522019	10.63925241	0.001107124	0.012919401
B3GALT1	-0.038946223	2.803526533	0.074066434	0.785505538	0.894435931
CHADL	-0.525226291	2.805182094	6.430728093	0.011216259	0.067285287
RHOBTB1	0.064581668	2.807331387	0.207666888	0.648602251	0.812444812
SHISA3	0.223774031	2.808723257	1.402858295	0.236245585	0.468533337
RGS19	-0.03020483	2.808933212	0.067502981	0.795007746	0.899962332
CFD	0.150082175	2.809154743	0.73835757	0.390187515	0.623951561
CDS1	-0.225167779	2.812462048	2.123269939	0.145076512	0.35403144
RLTPR	-0.009033719	2.813393433	0.00428553	0.947804602	0.977997412
TBKBP1	-0.185945308	2.813469064	1.607252966	0.204878381	0.43171645

ASIC4	0.297895789	2.813885102	2.329330505	0.126956469	0.324621184
CATH3	-0.244382012	2.814761024	2.485918664	0.114869259	0.304424812
TUSC3	-0.121559607	2.815031014	0.805585764	0.369428548	0.60402889
LOC112532504	-0.803772601	2.815170577	38.24326712	6.25E-10	7.29E-08
GPAA1	-0.091020759	2.815752759	0.533095016	0.465308549	0.685957013
SCNN1G	-0.219634953	2.817323009	2.259564905	0.132791365	0.334570803
KLF15	0.118709535	2.81749728	0.70090854	0.40247858	0.634660102
RNF19B	-0.086293393	2.821721982	0.394884854	0.529742826	0.73325355
LOC107055339	0.019035041	2.822240448	0.014981451	0.902583239	0.955420212
LOC112532155	-0.054406195	2.822999919	0.209960287	0.646798532	0.811526992
STBD1	0.29631349	2.826405859	3.928511737	0.04747394	0.173440526
MOGAT1	-0.357955473	2.827104686	8.445652088	0.003659172	0.030579826
LOC107053581	0.440781104	2.827822625	11.40726435	0.000731575	0.009554018
MFSD4A	-0.069287546	2.830459353	0.301372956	0.583022978	0.770877221
OLIG3	0.437609576	2.831266968	7.781088772	0.005279597	0.039723716
LOC107055095	-0.366467136	2.832503385	6.566489018	0.010391675	0.063709024
DDIT3	-0.054944107	2.83438206	0.156615655	0.69229171	0.839288299
FBXO2	-0.138469235	2.834890797	1.017556944	0.313099211	0.551100049
LRRC6	-0.200775672	2.835999729	2.49868781	0.113941198	0.302843227
C2	0.179036291	2.836657799	2.457925265	0.116933144	0.30833977
LOC112530118	0.536690575	2.838509345	4.525797197	0.033387517	0.1391217
HPDL	-0.089597005	2.838532834	0.395829874	0.529250781	0.73304986
PCBP3	-0.009770608	2.84245656	0.005783565	0.939379513	0.973822837
LOH11CR2A	-0.051849032	2.842630085	0.086041759	0.769270876	0.884870729
DCAF11	-0.104666335	2.843118345	0.705051316	0.401091567	0.633652443
SERF2	0.016675264	2.843529555	0.014660599	0.903626909	0.955902743
LOC100858152	0.065867536	2.845705248	0.247154123	0.619086114	0.794312827
ST8SIA4	-0.026016821	2.847108688	0.04639149	0.829465731	0.918867551
SEC14L2	-0.349981342	2.847214853	5.725724192	0.01671814	0.088740967
LRP2BP	-0.297526997	2.84859268	2.720685989	0.099055436	0.276263932
CDADC1	-0.127460104	2.851876579	0.845825367	0.35773601	0.59400061
KCND3	-0.270926825	2.852546092	2.602515627	0.106694239	0.290719275

LOC112532394	0.254587527	2.853010563	1.962427024	0.16125399	0.376961843
JADE2	-0.512416178	2.853360221	12.47437778	0.000412572	0.006262798
FAM132A	0.744245223	2.853850451	9.169525447	0.002460784	0.022983247
GJA4	-0.125300166	2.856250487	0.80751654	0.368855506	0.6036918
DTX3L	-0.581797093	2.856643949	16.76821476	4.22E-05	0.001043506
NKAIN2	-0.262280304	2.857997564	3.941924861	0.047096859	0.172801769
LOC107050073	1.020336402	2.858588949	34.60587574	4.04E-09	3.93E-07
ASCL1	-0.048989772	2.859850811	0.031962402	0.858110201	0.933773189
CDCP2	0.427693624	2.860002664	2.531513893	0.111593106	0.299407232
GGT7	0.15026206	2.860151718	0.956400434	0.328095455	0.565717803
LBX1	-0.225766291	2.860326228	0.38631804	0.534241174	0.736012937
S100A1	0.055573538	2.86322709	0.171935559	0.678397313	0.830767864
LOC107053933	0.191302892	2.86335782	1.535005225	0.215362801	0.444471699
AVPI1	-0.019234282	2.863383352	0.019906503	0.887798523	0.949358246
PARP11	0.086190443	2.863589106	0.417934397	0.517969544	0.723469047
LOC112530508	0.080276926	2.863799118	0.363360173	0.546645978	0.746539926
AMN1	-0.043249661	2.863905885	0.094326091	0.758747826	0.878744052
FBXO4	0.037101677	2.864155703	0.065040078	0.798700267	0.901588892
AOC3	-0.083195392	2.864596222	0.256188728	0.612750725	0.790251052
LOC107053805	-0.670639599	2.866090635	23.98266173	9.72E-07	4.72E-05
LOC100859691	0.060202908	2.86772729	0.042824446	0.836056184	0.922500949
LOC112532082	-0.186540717	2.867806976	1.595181748	0.206587366	0.4339838
DPP4	0.049755938	2.868062001	0.049399734	0.824111092	0.917134318
TRIM66	-0.719591282	2.868091696	4.473613533	0.03442212	0.141639405
TMEM240	-0.614602002	2.868900931	15.30994664	9.12E-05	0.001931576
LOC112533366	0.458380668	2.869097348	15.16406189	9.86E-05	0.00204028
RILP	-0.36347548	2.871814118	5.961621602	0.014620588	0.080754108
KIF20AL	0.337690117	2.874175335	6.105731848	0.013474414	0.076587732
NR5A2	0.233716519	2.874369796	2.060889753	0.151121907	0.362758714
LOC420807	-0.039819712	2.875307921	0.083862143	0.772130216	0.886877196
TSPEAR	-0.068153987	2.875558582	0.161229583	0.688026984	0.837680081
JPH3	-0.161698474	2.875733398	1.473266267	0.224831057	0.454444087

LOC107051009	-0.035627169	2.876313459	0.066375098	0.796689657	0.900812774
SLC51B	0.256050107	2.877796044	0.803998615	0.369900532	0.604380226
ZDHHC23	0.163306954	2.879225617	1.517256644	0.218035117	0.447185324
LOC107052690	-0.03644995	2.881913519	0.099472004	0.752464172	0.874606076
NTRK3	0.079879887	2.882029471	0.234299302	0.628354124	0.800445901
SIRT4	0.153843576	2.882694972	1.76825863	0.183597734	0.406061924
CRYBA1	0.145400913	2.883277484	0.954390563	0.328604229	0.566137849
LOC107052369	-0.61897716	2.883427177	13.99572232	0.000183227	0.003327815
LOC101749597	0.292700132	2.883521205	3.79441494	0.051423842	0.182878607
SULT2B1L1	0.156138357	2.883847122	0.835425619	0.360708332	0.597237914
PDLIM3	-0.337714893	2.884612965	2.50020145	0.113831736	0.302755928
DGKB	-0.109296331	2.886877396	0.634052921	0.425872561	0.654194641
TNFRSF11B	-0.195883994	2.886986034	2.071008443	0.150122228	0.361300171
FBXO41	0.233975393	2.88818108	2.23744779	0.134703154	0.337732806
VDR	0.120721657	2.888457061	0.735377535	0.391145654	0.624653104
RGS8	-0.129836151	2.888856839	0.596183049	0.440038083	0.664967091
LOC100857549	-0.322304508	2.889679654	2.602112909	0.10672135	0.290745002
CAMK2N1	-0.160698494	2.890311826	0.935182318	0.333519914	0.571376385
RMDN2	-0.366930683	2.890739606	8.179175476	0.004237402	0.033847788
ARAP2	-0.510606349	2.891442027	13.65910847	0.000219176	0.003849022
LOC112530893	-0.286550458	2.892956824	3.921870096	0.047661833	0.173882954
ALG14	0.63998537	2.893103406	25.75054019	3.89E-07	2.18E-05
LOC107054574	-0.198321006	2.89374391	2.308607081	0.128659299	0.32784178
PLCB2	0.202224794	2.894524021	2.315281341	0.12810812	0.326774006
TCP11	-0.108971602	2.896991454	0.377098375	0.539160243	0.740257537
PTPN5	0.07706528	2.898008249	0.307761784	0.579056903	0.768307327
ARID5A	-0.028984805	2.899106461	0.043542547	0.834707024	0.921880937
MIR6681	-0.18230814	2.899546823	1.789350497	0.181005318	0.403134632
SUB1L1	0.115320202	2.900887243	0.867081469	0.35176455	0.588218976
SPAG16	0.938230912	2.903663581	38.94855303	4.35E-10	5.30E-08
CDH20	0.041307074	2.904074814	0.066177594	0.796985741	0.900997038
LOC101747742	0.48402105	2.90483313	17.56203716	2.78E-05	0.000734492

HECTD3L	0.059046908	2.905121118	0.301230476	0.583112049	0.770877221
C1orf145	-0.143170967	2.906493674	1.304729682	0.253351097	0.48783004
CUBN	-0.387822837	2.90698336	5.122240315	0.023621103	0.110894506
ALK	0.125060453	2.907213778	0.789542573	0.374238477	0.608868315
FAM20A	-0.2984823	2.908075837	3.101318647	0.078228905	0.238646021
SLC15A5	-0.05987918	2.908810606	0.048724519	0.825297757	0.917552618
LOC107052450	-0.022747852	2.9097008	0.031647188	0.858804209	0.933947352
LOC415787	-0.025361922	2.910893058	0.036016841	0.849480718	0.929957743
METTL8	-0.077820816	2.911541736	0.251423392	0.616074602	0.792054659
LOC112530022	0.338312856	2.911543255	5.348509629	0.020739991	0.101600643
SCX	-0.376656497	2.911841543	2.765755421	0.096301217	0.271980108
LOC112532150	0.39175878	2.914035401	7.013609842	0.008089242	0.053513207
TLCD2	-0.005760821	2.91411225	0.001941175	0.964857559	0.985275608
AIFM2	-0.492049006	2.914522932	8.328396263	0.00390301	0.031936364
NLRX1	0.162108075	2.916015096	1.511317509	0.218938156	0.448199473
ATP5S	-0.063323332	2.917962221	0.260176112	0.609999189	0.787752053
MYH7B	-0.301314719	2.918170512	0.918063708	0.337983893	0.575072378
LOC107050650	-0.184530535	2.919582742	1.972034346	0.160232093	0.375748545
SLC22A4	-0.174365445	2.919789603	1.306371683	0.253052634	0.487650887
LOC107049647	-0.256164302	2.920194657	2.397738752	0.121510779	0.315600463
LOC112530959	-0.188617023	2.920227036	3.209011645	0.073233708	0.229108265
AIM1L	0.057750361	2.921837118	0.130264876	0.718157565	0.854281987
MYF6	-0.026487521	2.922653476	0.029017804	0.864737934	0.937551759
KATNAL2	0.10847851	2.923016948	0.610484457	0.434605462	0.661129662
MKNK1	-0.13522706	2.923258087	1.301936035	0.253859887	0.488454682
CAPG	-0.704502808	2.924626894	11.64387419	0.000644143	0.008738149
CRHR2	-0.355576689	2.925086959	3.564026968	0.059044336	0.200200812
ABHD5	-0.171499293	2.925270235	1.749619753	0.185924542	0.40857216
RSPO3	-0.122930057	2.92562179	0.686607343	0.407320752	0.637865574
TMEM130	-0.39745063	2.925834782	12.03308249	0.000522646	0.007497938
ANKRD39	-0.066746323	2.926422016	0.136501209	0.711784361	0.850204099
TRABD2A	0.310814147	2.926495898	2.303796833	0.12905818	0.328281397

RYR3	-0.314218412	2.926564791	3.568023125	0.058902395	0.199896572
LOC107054741	-0.042376747	2.926676091	0.10033459	0.751428482	0.874274211
PPFIA2	-0.636556749	2.927029538	12.95018373	0.00031989	0.005175803
FNDC5	-0.012427136	2.928201046	0.005230457	0.942345738	0.975259608
WDR25	0.256834087	2.928845137	3.600357701	0.05776714	0.19753913
TRHDE	-0.470184741	2.929933471	11.63134681	0.000648496	0.008782715
UNC93B1	-0.200250315	2.929941742	1.458874239	0.227109326	0.456912087
MAP6D1	0.037983125	2.930041485	0.095576034	0.757204582	0.877759873
ADCY2	0.404584155	2.930276585	7.87730303	0.005005908	0.038274401
PTPN6	-0.174945279	2.931138975	1.27963268	0.257967343	0.492541889
UBAP1	-0.161470801	2.931146124	0.962053922	0.326669936	0.564326983
DNALI1	0.203429641	2.931380075	3.22787016	0.072394785	0.227382361
OPRM1	-0.237867144	2.931410015	0.866025186	0.352058061	0.588551802
LOC112530295	0.235518789	2.931616134	1.464416447	0.226228723	0.456254766
IGFBP3	-0.140086349	2.931940648	1.38464398	0.23931169	0.471874002
PERP2	0.130523562	2.932081246	0.263051177	0.608031662	0.787414637
LOC112529987	-0.430415289	2.933324483	8.651663453	0.003267629	0.028107075
ETNK2	-0.269435505	2.934800794	5.008596586	0.025221746	0.115702211
MEGF6	0.041230067	2.935415147	0.04037929	0.840740808	0.924944173
SPIRE2	-0.390317613	2.937092734	3.765752456	0.052312316	0.184839845
SPG20	0.107705458	2.937243438	0.53267041	0.465486322	0.686036347
LOC101747724	0.22764414	2.937513242	1.226649352	0.26805965	0.503183194
SNRKL	0.125000128	2.938455602	0.81958937	0.365300252	0.60086122
CDH22	0.310012586	2.938637703	3.822958001	0.050554914	0.181174895
CFAP54	-0.29043667	2.939030979	3.949771389	0.046877736	0.172329611
RAMP3	0.268620968	2.944814917	4.742392356	0.029428121	0.127758008
NEUROG2	-0.089266216	2.946202908	0.087327658	0.767602378	0.884179034
SLC6A2	-0.120216644	2.948264546	0.495711347	0.481390605	0.69692789
TPBGL	-0.143670628	2.949072049	0.633805384	0.4259629	0.654260515
ANKRD1	0.556552118	2.949362507	6.163875418	0.013038524	0.074860052
LOC428824	0.402671555	2.949774119	9.743677328	0.001799406	0.018598756
INSM1	-0.194356922	2.950948923	0.764516366	0.381918823	0.616619532

PEX11B	0.338221254	2.953459038	9.000768004	0.002698662	0.024478493
LOC107050732	-0.115353931	2.954147949	0.669915109	0.413081205	0.643252695
COQ2	0.355110648	2.954181409	8.188512922	0.004215647	0.033755944
LOC417386	0.292599607	2.954853009	3.755514805	0.052633586	0.185735717
WNT16	0.187676928	2.95580894	1.031111095	0.309897846	0.547741793
CCDC107	-0.026103661	2.956477157	0.045914818	0.830330716	0.919392499
EMX1	0.259059641	2.957009961	2.066479698	0.150568719	0.361958842
LOC101751854	-0.430272705	2.960380592	2.771702975	0.095944037	0.271497701
AQP1	0.06934669	2.961119182	0.064350712	0.799747097	0.902345328
LOC112533257	-0.442755044	2.961147471	8.635909732	0.003296006	0.028292008
ENPP1	-0.360192925	2.961296771	4.050086076	0.044169093	0.165974519
MPG	0.087639124	2.961594539	0.55889905	0.454704172	0.677486161
CYBRD1	-0.102027231	2.961707543	0.469461249	0.493235124	0.705551458
VRTN	0.531423611	2.962438642	18.12468559	2.07E-05	0.00057828
CZH5ORF34	0.304638732	2.96288719	5.414887138	0.019965744	0.098983362
RAB3C	-0.196909694	2.963477949	0.877384912	0.348918933	0.585564614
AGTPBP1	-0.297020202	2.963774525	2.658951457	0.102968592	0.283905528
TRIM54	-0.216409267	2.963990032	2.350764195	0.125221608	0.321571728
HTR7	-0.236630235	2.965903564	3.421018574	0.064371285	0.212042203
OBSL1	-0.303000105	2.966678156	2.127446668	0.144681586	0.353434606
CDCP1	-0.358985179	2.966790368	6.642562718	0.00995706	0.06189778
GATM	-0.105796661	2.967193163	0.52504058	0.468699398	0.688181368
MAOA	0.019517111	2.96742952	0.021241042	0.88412422	0.948083049
LOC101747789	0.248609633	2.967530115	3.559981245	0.059188408	0.200524037
LOC101747443	0.232793996	2.967567983	4.202095545	0.040374058	0.15741623
GPR37	0.231184523	2.967809227	2.082624881	0.148983787	0.359883766
RIPK2	-0.003369548	2.969006926	0.000691728	0.979017482	0.991002256
LOC112530929	0.0410404	2.969602876	0.138066642	0.710210647	0.84968127
FAM214B	0.008820434	2.969687157	0.004166574	0.948533091	0.978350277
PPP1R3D	-0.117179829	2.969838857	0.479944	0.488447683	0.702128818
LMX1A	0.282036098	2.969890104	4.44200869	0.035064968	0.143174449
EGLN3	0.602070949	2.970522731	12.12139996	0.000498465	0.007235982

TPPP3	-0.094548475	2.970946613	0.395462382	0.529442026	0.733083855
LOC421506	0.580479275	2.971247108	11.0437634	0.00088986	0.011009513
LOC112533358	0.088155856	2.971773297	0.310347919	0.577466767	0.76705002
CCDC173L	-0.055869148	2.97279923	0.214750232	0.643069426	0.80933991
LOC112532077	0.24847353	2.973847561	3.666045955	0.055531778	0.192171483
KCNS1	-0.149925063	2.973972593	0.827048825	0.363127283	0.599097263
OSMR	-0.036699496	2.974040398	0.058912962	0.808222371	0.907716078
LOC112533000	0.035471793	2.974974921	0.090194407	0.763930153	0.881643334
TMEM151B	0.157265084	2.97500457	0.840892743	0.359141571	0.595433203
HENMT1	-0.111806392	2.976139388	0.498210564	0.480287442	0.696466157
CLPP	-0.009001549	2.978062251	0.005049942	0.943347659	0.975792553
IRAK1BP1	-0.039191562	2.978768061	0.113668929	0.736005161	0.865130718
RHOF	-0.28147193	2.978775422	5.197489554	0.022619533	0.10750919
F2RL1	-0.371267938	2.981946214	7.08550538	0.00777099	0.052086209
ELFN1	-0.157682276	2.98238012	0.615503117	0.432723281	0.659478488
LOC112530311	0.205123838	2.983415364	2.142315278	0.143285499	0.351432834
PEX12	0.035109131	2.9855597	0.047238689	0.827939776	0.918504892
IGBP1L	-0.300577755	2.988198047	5.012116451	0.025170517	0.115563854
LOC107051262	0.217099714	2.988496299	2.026606377	0.154565407	0.367433368
EDA2R	-0.822132509	2.989247955	9.485969253	0.002070492	0.020673511
C2H7orf25	0.165636253	2.989653195	2.035361419	0.153677651	0.366311338
CTU1	-0.218203435	2.989679539	2.022016566	0.155033134	0.368084048
DPYS	-0.034492743	2.989977515	0.048800178	0.825164362	0.917552618
NWD2	0.1068652	2.990209459	0.663646947	0.415275379	0.645642722
TFAP2C	-0.165352982	2.990638381	0.560269663	0.454151612	0.677030691
LOC107050670	0.160934863	2.992529806	0.963679559	0.326261553	0.564154365
TTPA	0.254671451	2.993120173	2.80206423	0.094143031	0.268763511
FGD4	0.179695702	2.994327024	1.707780325	0.191273615	0.415364966
SLC22A13	-0.04421596	2.994399371	0.122856588	0.725956706	0.858902618
EPM2A	-0.178567106	2.994891642	2.256541108	0.133050942	0.335019625
LOC112532893	0.124745889	2.995639263	0.979316226	0.322367677	0.559907128
TMEM63A	-0.088401755	2.995789681	0.559179357	0.454591081	0.677378995

LOC112530898	0.09672989	2.995858467	0.560819808	0.453930118	0.67676181
RTN4RL2	0.336072783	2.99636163	5.839917214	0.015666564	0.084681339
PODN	-0.459665227	2.997636286	3.164671337	0.07524758	0.233446254
LOC112531349	-0.454790129	2.997799184	2.591166602	0.107461159	0.291842445
IL13RA1	-0.168153154	2.998327224	0.944134777	0.331216746	0.569089648
ONECUT1	-0.152021696	2.998423988	0.772295293	0.37950792	0.61405408
HOXC8	-0.332313988	2.99858275	0.870441034	0.350833241	0.587339605
ECM2	-1.251861224	3.001401678	44.00480724	3.28E-11	5.61E-09
GCK	-0.216114507	3.001724345	1.875447112	0.170852517	0.389603404
MYLPF	-0.083125311	3.002585867	0.113536058	0.736153747	0.865243565
HHAT	-0.275459834	3.005892692	4.97251394	0.02575319	0.117283555
ASB13	-0.012934108	3.006015194	0.010046535	0.920159819	0.963366407
FAM109A	-0.188674783	3.006033027	2.165902899	0.141101723	0.347995932
LRFN5	-0.121116713	3.006114433	0.696347779	0.404013621	0.635646128
MBOAT1	-0.213577558	3.007109898	2.698284276	0.100456299	0.27894168
LIX1	-0.181393609	3.007135788	1.037392042	0.308428791	0.5463074
OXSM	0.259681579	3.007308334	4.240311012	0.039474935	0.1551003
TSTD3	-0.131088181	3.008246319	1.17011171	0.279378288	0.514715552
KIAA0319	0.0572182	3.009302539	0.198182562	0.656191846	0.816485578
TMEM30CP	0.038054718	3.009687297	0.056997664	0.811305775	0.909337575
LOC112533553	-0.179146506	3.009880956	0.797350698	0.371886649	0.606360961
STEAP2	0.237661993	3.010861327	2.762899397	0.096473249	0.272339564
LOC107050437	-0.395789016	3.011918257	7.247647562	0.007099396	0.048695523
LOC107053500	-0.145975913	3.01346938	1.320849169	0.250439708	0.484707763
TTC32	0.458099336	3.013625864	8.547518005	0.003459937	0.029241529
DEPDC6	0.06851797	3.01403446	0.30273817	0.582170904	0.770499657
LOC112532264	-0.247879596	3.014662797	2.031623723	0.154055942	0.366964465
LOC107050335	0.008958814	3.015145697	0.00391434	0.950113148	0.97884497
LOC112530338	0.08390928	3.015653993	0.317012908	0.573408332	0.764869254
LOC112531348	-0.139455003	3.015654299	0.865692944	0.35215045	0.58864645
MARVELD1	0.239292513	3.016310675	3.394303198	0.065422	0.214317939
PROM1L	-0.017712994	3.016544353	0.022803791	0.879968426	0.946151989

SERINC4	0.222890779	3.017074126	2.156798968	0.141940113	0.349121758
LOC112532982	-0.814246251	3.017802148	11.56688194	0.000671369	0.009025635
HOXD10	-1.77350457	3.018223626	5.83904129	0.015674365	0.084681339
ZBTB7C	0.262899848	3.018428431	2.254803953	0.133200323	0.335088109
LOC112532310	-0.317860553	3.020141795	4.902498024	0.026817875	0.120669438
GUCY1B4	-0.439175855	3.020571302	11.3607583	0.000750122	0.009742112
LDLRAD1	-0.931238142	3.022496868	32.65312299	1.10E-08	9.54E-07
LOC112530336	0.346558157	3.023180663	7.018325987	0.008067962	0.053423872
OTOA	-0.210876049	3.02373754	1.083487076	0.297918781	0.534872864
PABPC1L	-0.055109508	3.023847232	0.208640067	0.6478354	0.812138618
NUDT13	-0.109925947	3.025480075	0.847385909	0.357292905	0.593806442
BTD	0.450164089	3.025486379	8.797926597	0.003015731	0.026536434
MFAP5	0.319345144	3.026393578	4.306003623	0.03797808	0.150512839
CCDC112	0.004107047	3.026727507	0.00066829	0.979375936	0.99115393
HOXD9	-0.824536883	3.027199407	3.034399226	0.08151672	0.245086357
ADRA2A	0.073232278	3.027331869	0.238108476	0.625575623	0.798796471
SEC14L3	-0.070996132	3.027656558	0.260832367	0.609548883	0.78760321
BPIFB2	-0.259576792	3.029031832	1.506177225	0.219723336	0.448987957
LYGL	0.791004862	3.029163778	13.24719814	0.000272988	0.004586857
LOC112531237	-0.147967719	3.029231071	0.382360759	0.536342499	0.737796005
NDUFAF8	0.214264737	3.029453973	2.734593059	0.098196512	0.274986999
HDDC3	0.03561884	3.029652304	0.081797519	0.774876087	0.888852284
TEX30	0.070654824	3.029895048	0.337341366	0.56136787	0.75591721
CDH12	-0.603698445	3.030666332	6.649279204	0.009919595	0.061734844
CENPA	0.368795658	3.030989277	6.966731226	0.008303905	0.054601899
CBX4	-0.118614359	3.031155012	0.638648045	0.424200784	0.65309226
LOC101749423	0.017964975	3.031302714	0.013031559	0.909114247	0.95829261
ALKBH2	0.033756556	3.031323295	0.071023409	0.789852485	0.896648248
PROX1	-0.024390716	3.031369599	0.021837002	0.882521539	0.947489154
LOC107050798	1.230738706	3.032096712	35.64794831	2.36E-09	2.43E-07
SLC6A1	-0.141613519	3.03224472	0.85629664	0.35477713	0.591415021
PCGF2	-0.101542769	3.032332134	0.473024584	0.491598999	0.704487482

LOC107052931	-0.36645392	3.032900894	7.946244362	0.004818727	0.037349108
JDP2	0.072563657	3.034579632	0.317621344	0.573040649	0.764693721
PLCXD2	0.106962632	3.034974124	0.582379635	0.445381089	0.670062789
FOXF2	0.336455681	3.035820065	3.412978735	0.064685584	0.21266759
CFAP69	0.151103685	3.03598366	1.525654502	0.2167658	0.445915894
SMPDL3A	0.121855466	3.036984232	1.187311302	0.27587257	0.510915379
NTM	0.194970568	3.037043177	1.583381345	0.20827431	0.436024652
NT5E	0.052764731	3.037590912	0.020532887	0.886058776	0.948791653
CCDC28B	0.042693662	3.038152757	0.09861495	0.753498122	0.87513434
ZC3HAV1	-0.13134892	3.038818426	1.137891359	0.286098335	0.522416162
SMYD1	-0.120545905	3.038841719	0.362362059	0.547197326	0.746725327
KDM1B	-0.039973261	3.039706738	0.10983272	0.740334663	0.867900794
GK5	0.185610594	3.040169426	2.077351363	0.149499392	0.360652762
ERICH1	-0.138537793	3.040382753	1.106088226	0.292933865	0.53027797
PTRHD1	-0.058935993	3.040790567	0.25102128	0.616356879	0.792169995
LOC107057116	0.676058049	3.041905626	11.4290303	0.000723054	0.00950308
LOC422442	0.258356298	3.042626101	3.941102319	0.047119892	0.172801769
MYB	0.095888686	3.045054779	0.590378369	0.442272843	0.667057711
NAT8B	-0.142056109	3.045504722	1.304617088	0.253371579	0.48783004
MIEF2	-0.183012609	3.045529697	1.477877953	0.224106832	0.453588919
LOC107057394	-0.072297506	3.045588892	0.249195737	0.617641954	0.793398483
KRT14	-0.612372155	3.046488629	6.804048188	0.009095142	0.058007972
PDF	0.222582795	3.04805759	2.453698092	0.117248347	0.308917811
LOC107052763	0.36900266	3.048103131	7.071267875	0.007832981	0.052416307
FGB	0.584877993	3.048408025	1.355872939	0.24425402	0.477487933
GRM7	0.26017651	3.048556058	2.472467181	0.115855941	0.306250523
ANXA4L	0.253918015	3.049038838	3.301139011	0.069231862	0.221567349
LOC101751615	-0.310832485	3.049628751	7.275877797	0.006988673	0.048237675
INCENPL	0.113561578	3.050369106	0.659522206	0.416728673	0.646851271
LOC107051775	0.253892663	3.050773951	1.372103639	0.241450778	0.474290745
BCL11B	0.043452939	3.050974921	0.018110339	0.892948178	0.951217859
WNT7A	0.206575253	3.050983797	1.833826721	0.17567622	0.395349213

PCLO	-0.150488651	3.051213623	0.860747386	0.353529626	0.590232345
LOC112532292	0.119285059	3.051598724	1.22505339	0.268371197	0.503525873
HARBI1	-0.081763597	3.052077053	0.38753579	0.533597547	0.735495698
TAC1	-0.21017157	3.052143421	0.670402252	0.412911399	0.643109992
TNIP2	0.013979358	3.05226825	0.017302795	0.895348075	0.952048632
LOC112532091	-0.236464806	3.052619034	1.755130858	0.18523301	0.407836809
BEST2	-0.119962286	3.053113267	0.592360349	0.441507835	0.666270307
PUDP	0.384878243	3.053197955	8.369256249	0.003816218	0.03138224
SSX2IP	-0.003237738	3.053326816	0.000875297	0.976397691	0.99017774
PCDH12	-0.095797288	3.053371068	0.351622563	0.553196071	0.751431513
CYP2D6	0.025378352	3.054329418	0.044775546	0.832417292	0.920773496
LOC107054809	0.089429934	3.057078817	0.194705753	0.659028582	0.818747854
C14orf132	-0.412195644	3.057644713	5.160168733	0.023110652	0.10912074
CD109	-0.802848504	3.058274897	15.99457825	6.35E-05	0.001455835
WDPCP	0.055548441	3.058678178	0.156193099	0.692685902	0.839401545
SCGN	-0.239895103	3.05874072	1.878374392	0.170519024	0.389244365
MGAT4C	-0.107167332	3.058811591	0.64582964	0.42160765	0.650802428
ARL16	0.106516701	3.061167944	0.928423109	0.335273015	0.572606457
MMP28	-0.038906441	3.062664914	0.056987565	0.811322176	0.909337575
AOX1	-0.156245615	3.063636789	1.155431346	0.282415058	0.518075784
LOC107055285	-0.046481675	3.064038374	0.110871388	0.739154246	0.867283451
SH3GL3	0.061235845	3.065857248	0.192656057	0.660715137	0.820100142
ZBTB6	-0.027768332	3.066331853	0.044145192	0.833583715	0.921258649
TTC39A	0.157338434	3.067635309	1.417172832	0.233869302	0.465616378
CILP	-0.386109048	3.068719471	9.389597612	0.002182201	0.021373879
DTD2	0.452415592	3.069301664	12.50637948	0.000405565	0.006189294
FGF8	-0.173581734	3.069597233	0.609787743	0.43486774	0.661222386
PDE9A	0.128964895	3.070047455	0.928217308	0.335326585	0.572606457
SPTBN4L	0.553616939	3.070862521	8.557227158	0.003441532	0.029115892
TNFRSF10B	-1.032224911	3.071537923	21.74134118	3.12E-06	0.000123708
METTL25	0.439834826	3.071847912	9.531497779	0.002019752	0.020290002
INPP5D	-0.147287101	3.073199325	1.130579036	0.287651863	0.524408532

FAM13AL	0.438941601	3.074429029	8.71579401	0.00315465	0.027364135
HEBP2	-0.026227992	3.075259409	0.035705697	0.850124551	0.930291912
LOC107053084	-0.192338788	3.076616882	3.245678457	0.071612036	0.226219247
CRACR2B	-0.041632207	3.076898664	0.109453995	0.740766613	0.868184802
ZFPM2	0.5895098	3.077360414	8.856050191	0.00292119	0.02594744
KCNQ5	-0.558289083	3.077828253	13.66037304	0.000219028	0.003849022
SNAP25	-0.259876479	3.077861863	1.9146695	0.166445484	0.384239681
NACC2	-0.103987178	3.078109883	0.847376525	0.357295567	0.593806442
LOC101748095	-0.280507986	3.078997495	2.561054574	0.109525501	0.295643393
ITGA1	-0.082328306	3.079548741	0.261459601	0.609119158	0.787473436
PMFBP1	0.572318407	3.080594923	19.25710496	1.14E-05	0.000362914
DKK2	-0.245142258	3.080849583	1.770113264	0.183368062	0.405772118
NAB2	0.247130227	3.081332314	3.143536891	0.076228338	0.235512074
GRHL1	-0.023856829	3.081544321	0.035539871	0.850468871	0.930485775
BVES	0.059060686	3.082138589	0.20722143	0.64895399	0.812579461
CERS3	0.156926768	3.083335092	1.520696575	0.217514111	0.446474354
CCDC113	-0.308742243	3.083518437	5.646720581	0.017488047	0.091176625
UNC13C	-0.020190025	3.084769724	0.014191928	0.905172459	0.956683975
LOC107049262	-0.471183895	3.085190703	10.70015137	0.001071268	0.01261826
LOC772096	0.313877763	3.085239212	2.362341802	0.124295476	0.31992524
LOC112530451	0.07694054	3.085718999	0.416018246	0.518930579	0.724503875
C1HXORF59	-0.524012063	3.085754549	7.828745103	0.005142179	0.038921137
CD226	0.031561016	3.087714779	0.057594702	0.810338814	0.909099927
LOC107054601	0.348554831	3.087769637	3.232042914	0.072210556	0.227194015
LOC112530487	-0.424409801	3.088894183	1.667980441	0.196529256	0.421573317
USHBP1	0.21904911	3.089323939	2.22571098	0.135730163	0.33886206
LOC101747660	0.36354361	3.089462549	6.376369718	0.011564975	0.068864234
LOC107049018	-0.144990383	3.090108336	1.275277631	0.25877893	0.493272928
SOSTDC1	-0.05678081	3.090853576	0.110889377	0.739133856	0.867283451
NRN1	0.440076891	3.091002917	2.65906317	0.10296136	0.283905528
DUSP27	-0.455772517	3.091691709	7.320686712	0.006816536	0.047407482
PIH1D2	-0.09897946	3.092851553	0.563585516	0.45281918	0.675632701

TCEANC	0.335857543	3.093574412	5.629086606	0.017664834	0.091695532
LOC107054608	-0.283355006	3.095731239	5.598595424	0.017974883	0.092667468
ADPRHL	0.129809115	3.096114165	0.805148456	0.36955851	0.604121327
SLC35F6	-0.090579257	3.096298411	0.420833379	0.516521497	0.722717913
KCNA6	-0.127426325	3.096698529	1.044210541	0.306844229	0.544790871
MEGF11	-0.432988822	3.097421304	8.602863454	0.003356349	0.028660467
FN3K	0.341612237	3.098509034	6.700443948	0.009638891	0.060491209
TMPRSS9	-0.007153292	3.099360721	0.003042687	0.956010556	0.981041173
CAMKK1	-0.057825173	3.100069861	0.180838947	0.670653252	0.825783734
PHYHIPL	-0.265668697	3.100108332	2.266142317	0.13222868	0.333699147
B3GALNT1	-0.139963848	3.101177436	1.488513272	0.222447277	0.452027755
LOC100859636	-0.146681332	3.101295193	1.176594604	0.278050359	0.513330938
KLHL22	-0.150076712	3.101365733	1.462888435	0.226471099	0.4564093
PPP1R3E	0.457982055	3.102128356	4.9815262	0.025619372	0.116865103
DNAH9	0.091343555	3.102257941	0.452658791	0.501075034	0.711836008
DNAJC24	0.687055352	3.102723594	29.108871	6.84E-08	4.90E-06
LOC107050086	-0.069775933	3.10353813	0.220621455	0.638566808	0.806602965
LYPD6B	0.144178458	3.104201287	1.221859818	0.268995971	0.504022853
PRIMPOL	-0.112747415	3.105616326	0.807157511	0.36896197	0.603767292
ACOT4	0.25894942	3.105629991	4.048647484	0.04420675	0.166040189
ZIC5	0.201166615	3.105684186	1.627136286	0.202099598	0.428992375
SYN3	0.033877671	3.106483577	0.035078927	0.85143037	0.930961329
TENM1	-0.106977664	3.106865829	0.606463663	0.436122419	0.662345819
SLC35G1	-0.13690162	3.108486247	0.893688059	0.344479832	0.581324676
TCERG1L	0.241837695	3.108702837	3.475503574	0.062283762	0.207801968
C9H2ORF82	-0.011153435	3.108922405	0.005579224	0.940458019	0.974411302
LOC107056275	-0.028516483	3.109697443	0.038755328	0.843934178	0.926328616
LOC100857892	-0.131743087	3.110299867	0.681712052	0.408997808	0.639496288
TBXA2R	0.063405645	3.111270116	0.229877213	0.631614888	0.802751447
DNAAF2	-0.039014218	3.112575173	0.068794507	0.79310013	0.898320636
IMPACT	-0.062229429	3.113144069	0.311950708	0.576485612	0.766303478
TMEM52	-0.104867791	3.113326742	0.718287452	0.396706231	0.629433501

DDX60	-0.305910616	3.114112636	5.582789004	0.018137816	0.092977494
RAP1GAP2	-0.015875942	3.114243968	0.014012276	0.905771755	0.956948586
BZFP1	-0.29850021	3.115287891	6.603533884	0.010177657	0.062771119
TAL1	0.219022132	3.115956846	1.743862863	0.18665012	0.409510363
ZNF488	0.311240807	3.116073814	2.938235197	0.086505199	0.254777708
CDO1	0.304039234	3.116716416	5.072378024	0.024310012	0.112936547
LOC770126	0.159566715	3.11672384	1.617682183	0.203415277	0.430286462
FOLH1	-0.253848433	3.116787898	2.76978764	0.096058904	0.271682584
MPZL2	-0.015193189	3.117607157	0.013650112	0.906991843	0.957377047
LOC107056270	0.599691058	3.117928977	10.35408416	0.001291885	0.014431755
SH3YL1	0.199460445	3.118005949	2.859264097	0.090849053	0.262937133
ITGA7	-0.281965003	3.118759892	1.836819774	0.175324147	0.395037497
SPARCL1	0.38697094	3.119029927	6.030375466	0.014061742	0.078729487
CTSK	0.298464234	3.119355748	4.673902842	0.030624258	0.131113886
MCEE	-0.011081956	3.119499898	0.008003557	0.928714276	0.968392732
IRF5	-0.383996423	3.120189661	9.833420935	0.001713693	0.017847357
ANKRD16	0.296826737	3.120790656	3.806096891	0.051066318	0.182071677
LOC112531859	0.129045683	3.120963039	1.217617314	0.269828762	0.504595753
PTGS2	-0.187411143	3.122473346	1.008997135	0.315143214	0.552589063
TAPBP	-0.553796612	3.12305532	9.837781897	0.001709635	0.017827661
TTR	1.293147192	3.123465205	7.227482392	0.007179582	0.049122672
TMC1	0.375868134	3.123664166	5.611395013	0.017844052	0.092146097
MEST	0.067956651	3.123851772	0.390164162	0.532213124	0.734571964
GPR183	-0.144306505	3.125266194	0.842852383	0.35858226	0.594925498
DLEC1	-0.114194681	3.126410723	0.879986004	0.348205513	0.584725147
B3GNTL1	-0.080907703	3.127414297	0.234888157	0.627922785	0.800160259
LOC107050350	0.208855268	3.128761864	3.296510047	0.069427244	0.222057458
METTL21A	0.056711566	3.128981104	0.261428783	0.609140256	0.787473436
CYP2W1	0.153289934	3.129186982	0.897656733	0.343410805	0.58056982
MIGA1	0.006100912	3.130007604	0.002353201	0.961309951	0.983777298
LHFPL4	0.353932882	3.130479068	9.463032775	0.002096542	0.020835945
KCTD7	0.00980969	3.13050946	0.007146679	0.932628664	0.970554305

USH1C	0.114108133	3.131584676	0.822431882	0.364470099	0.600155657
NUDT5	0.318026884	3.131628629	5.207518376	0.022489413	0.107109491
CDK5R2	-0.599435513	3.132307578	4.926988553	0.026440356	0.119526389
FZD5	0.04197288	3.132332924	0.081061858	0.775863538	0.889117245
CACNA1B	0.248275061	3.133135244	2.306650666	0.128821365	0.327983222
KCNJ2	-0.165916267	3.133537698	0.731865342	0.392279226	0.625832839
LOC112531016	0.33013872	3.134246333	2.571831681	0.108781709	0.294311579
MSANTD1	-0.170926845	3.134641763	1.73954122	0.187196968	0.410108655
DOCK3	-0.177132588	3.134983166	1.241850811	0.265114655	0.500215481
RAB10L	-0.102560644	3.13619852	0.676615966	0.410754445	0.640927017
FTL	-0.006254053	3.136657953	0.002492665	0.960180882	0.983001084
HOMER1	0.107990271	3.137758468	0.441046192	0.506617935	0.716062371
C1orf167	-0.001037453	3.137969275	7.83E-05	0.992937775	0.996693168
KIF20BL	0.807731552	3.138027748	6.790672503	0.009163538	0.058286054
LOC107053273	0.268605576	3.138383918	1.857693065	0.17289129	0.392295391
LOC427783	-0.264116638	3.138463044	3.296104134	0.069444405	0.222057458
H1F0	0.491045737	3.138637758	3.988687041	0.045806745	0.169572551
LOC101752020	-0.084218983	3.138747435	0.424389646	0.514754783	0.721735682
PTPN3	-0.253070799	3.140631416	4.175177091	0.041020276	0.158919708
WNT9B	-0.353613025	3.14164728	2.756389445	0.09686663	0.272657543
LOC771494	-0.316363086	3.142052855	5.169303427	0.022989433	0.108798134
LOC112530427	-1.230287264	3.143383762	10.54782704	0.001163248	0.013379263
XKR8	-0.577780231	3.143650034	17.61197591	2.71E-05	0.000724426
LRRC27	0.102246087	3.144670267	0.835939232	0.360560741	0.597143069
SULT1B1	-0.58050612	3.144781356	16.02767143	6.24E-05	0.001436612
FMC1	-0.102801155	3.146536202	0.458445131	0.498351541	0.709783189
FAM212A	-0.206746804	3.149863961	1.430085454	0.231750493	0.46347282
LOC112530945	-0.19420381	3.150074098	0.35214076	0.552903792	0.751220535
NHLH2	-0.062289863	3.150255694	0.091924416	0.761744768	0.880727245
ARX	-0.060554872	3.152786723	0.051883802	0.81981715	0.915327127
GRTP1	-0.416246139	3.153490741	10.72929889	0.001054524	0.012501579
LHX5	-0.474926225	3.154248083	3.649402566	0.056089328	0.193368927

ACOX2	-0.191879963	3.155408389	2.496624336	0.11409061	0.303093475
CENPV	0.227839275	3.156228411	3.066006494	0.07994564	0.242221599
SST	-1.805450581	3.157148807	25.72758838	3.93E-07	2.20E-05
TRIM55	-0.104906318	3.157435553	0.26149596	0.609094268	0.787473436
CNNM1	-0.545905022	3.157809785	9.092949171	0.002565968	0.023680874
LOC101747877	-0.128482137	3.157865428	0.998292187	0.317724102	0.555713233
SLC22A16	-0.222231873	3.15851437	3.123917926	0.077151077	0.23738238
ARL11	0.101429904	3.15853051	0.727667422	0.393640314	0.626800017
BTBD17	-0.377477198	3.158684669	4.465467085	0.034586632	0.142174128
NTNG2	0.196036816	3.158726973	1.627948448	0.201987042	0.428820818
SETD4	0.186895511	3.159285089	1.550064644	0.213125898	0.441463576
NIT1	0.051452917	3.159527341	0.174597282	0.676057964	0.829570007
DUSP1	0.107757448	3.159789075	0.739916878	0.389687506	0.62364403
LRRC71	0.2449239	3.160042188	2.399292565	0.121390134	0.315584858
MDGA2	-0.197005615	3.160993428	1.544701318	0.213919378	0.44277275
SUPT16H	-0.186611995	3.161033995	1.736610344	0.187568892	0.410759399
SDCCAG3	-0.174740507	3.161922262	1.827715048	0.176397671	0.396589338
LOC112532636	0.142731736	3.161954703	0.156340195	0.692548609	0.839401545
LOC101749517	-0.066119635	3.163246032	0.326016287	0.568014312	0.760970118
ACBD7	-0.555172128	3.163285065	19.03195911	1.29E-05	0.000394746
LOC107054306	0.064315163	3.164187343	0.137572707	0.710706094	0.849781439
SH3TC2	-0.280624655	3.165041102	3.968177433	0.046367934	0.170842892
WBP2	-0.270986412	3.166839096	6.259577393	0.012352367	0.072154133
LOC112530551	0.330169633	3.167407631	2.67199808	0.102127736	0.282201828
IRF6	-0.246443453	3.16813301	2.781073998	0.095384186	0.270518231
SLC36A4	-0.491073491	3.169935661	17.2797404	3.23E-05	0.000827303
PLIN5	0.099806317	3.171363364	0.475864202	0.49030166	0.703822194
SLC25A26	-0.056848531	3.172742656	0.211569476	0.645540036	0.810805381
CSGALNACT1	-0.347822461	3.172844679	8.619194546	0.003326389	0.028478528
LIM2	0.356848541	3.173866578	3.278893236	0.070176238	0.223658724
PARP3	-0.195699122	3.173878528	2.822535001	0.092949499	0.266553503
ARHGAP44	0.294745038	3.17392488	3.448927359	0.063292832	0.209722827

PLCD3	0.475880014	3.175716143	13.62375411	0.000223342	0.003909667
ATP6V1AL	0.219164798	3.175935663	2.978017835	0.08440258	0.250875081
LOC112529957	1.29716984	3.176078927	77.79166125	1.15E-18	7.85E-16
ADGRB2	-0.445026358	3.176231351	7.712920673	0.005482697	0.040656956
TMEM216	0.169687572	3.176237308	2.109836951	0.146354911	0.35556918
SCIN	-0.572460185	3.176366937	11.41995928	0.000726593	0.009519177
GAL3ST1	-0.003385604	3.179358598	0.000533043	0.981580306	0.991828264
CYSLTR1	-0.911800893	3.179492471	47.62745199	5.15E-12	1.13E-09
PAPSS2	-0.074149233	3.180445453	0.1940716	0.659549246	0.81914732
LOC107049467	-1.867271008	3.181013831	28.81762643	7.95E-08	5.54E-06
GRIA1	-0.009537201	3.182497145	0.002458682	0.960453022	0.983096197
CDH23	-0.58830285	3.183451715	11.42164046	0.000725936	0.009518145
PPP1R1A	0.106005974	3.185014166	0.931493818	0.334475058	0.572087272
TEX14	-0.557245067	3.185119772	13.24638051	0.000273107	0.004586857
SDR42E1	-0.144717726	3.186281805	1.150173608	0.28351281	0.519510389
LOC112530289	-0.297326661	3.18634552	5.775203923	0.016253834	0.087034442
RNF24	-0.021733337	3.187298104	0.035076723	0.851434982	0.930961329
SCARA3	-0.079040925	3.187303334	0.300648215	0.583476333	0.770936649
PAPLN	0.235284192	3.187652818	2.207595516	0.137332575	0.341718161
LOC107054245	-0.082763653	3.188286287	0.362563747	0.547085832	0.746706815
LOC107057437	-0.097606076	3.18942565	0.511223121	0.474609855	0.692264899
HLX	0.722272035	3.18948739	12.81209593	0.000344386	0.00545803
LOC112532559	0.508266005	3.189997393	21.61043011	3.34E-06	0.000130868
OTOG	0.547938045	3.190156998	7.901050542	0.004940609	0.037954122
SRR	0.071230455	3.19029205	0.414011674	0.519940329	0.725544277
EMID1	0.037493594	3.1903145	0.074297387	0.785179571	0.89437421
HSPB11	-0.302201202	3.192392366	6.003578801	0.014276889	0.079393786
MAP10	-0.502252974	3.192661556	15.91419094	6.63E-05	0.001506403
CEP19	-0.230453266	3.193119614	3.886595384	0.048672998	0.176296321
ARSH	-0.447392061	3.193222624	5.29503409	0.021386312	0.103862114
LOC107050692	0.04627293	3.193361087	0.088522535	0.766063916	0.883053012
SLC26A8	0.371113636	3.193506776	10.43475849	0.001236657	0.013976095

PYROXD1	-0.116329936	3.193658044	1.172255159	0.27893835	0.514277933
ARHGAP27	-0.244887041	3.19423427	2.880747151	0.089644418	0.261125669
ARMC5	-0.255858236	3.194319158	3.359404406	0.066822232	0.216895178
LHX6	0.113913131	3.194323318	0.670424217	0.412903745	0.643109992
LOC420716	0.017666878	3.194371305	0.01820285	0.892676756	0.951217859
LOC112530169	-0.268705031	3.194402537	4.347328658	0.037067044	0.14840849
MGLL	0.29791176	3.194613601	3.378390028	0.066056552	0.215538482
UNC45B	0.09192704	3.195219926	0.131943035	0.71642588	0.853085451
C5H14orf159	-0.988163348	3.195465739	62.72684949	2.37E-15	1.00E-12
LOC107054934	0.087552904	3.19598218	0.3913356	0.531598188	0.733969474
METTL23	0.293790902	3.197464718	5.080979761	0.024189697	0.112607009
SCN9A	0.207477344	3.198917816	1.251428308	0.263279853	0.498477734
ABCG1	-0.540502201	3.199095274	12.77385725	0.000351497	0.005534821
CRTC2	-0.064420022	3.199429347	0.301445753	0.58297748	0.770877221
CPM	-0.48492602	3.202720337	6.021682245	0.014131171	0.079032682
LOC422895	-0.077492392	3.202798051	0.309085089	0.578242154	0.767832041
SLC2A10	0.083091529	3.203305353	0.502153861	0.47855526	0.694656563
CD38	-0.138772838	3.203728617	0.729820553	0.392941361	0.626150638
KIAA1161	0.207123093	3.205035472	2.38014303	0.122886295	0.317627753
IRX1	0.288388562	3.205124368	4.424906734	0.035418049	0.144151371
MYCT1	0.014334963	3.205209278	0.015359227	0.90136884	0.954933316
TBXAS1	-0.002641797	3.207807332	0.000352218	0.985026595	0.992992257
SPAG17	-0.277351961	3.207828029	3.80025709	0.051244714	0.182478202
IMMP1L	0.012923855	3.208638605	0.015218261	0.901820197	0.955177611
GPR12	0.177003933	3.209628553	1.31530408	0.251436564	0.4860083
NALCN	0.107965201	3.211604801	0.694317189	0.404699815	0.636100435
STXBP5L	-0.216084059	3.211694184	3.572663298	0.058738033	0.199655926
SLC13A3	0.632059785	3.212379517	26.06316755	3.30E-07	1.90E-05
KIAA1644	-0.297868255	3.212628244	3.873881883	0.049042966	0.177168389
EXOSC6	0.046846392	3.213913453	0.07489716	0.784335579	0.894031723
LOC107052590	-0.167358505	3.21433265	0.81535927	0.36654051	0.601578305
ILDR1	-0.151010152	3.214344687	1.330843462	0.248655221	0.482857453

CHODL	0.230708421	3.215240181	2.115134277	0.145849261	0.355022129
GCFC2	-0.05265024	3.215270165	0.235180096	0.627709186	0.800097516
FAM19A2	0.419947942	3.216037521	7.139809477	0.007539117	0.051009937
UPP1	0.07098862	3.216229391	0.261812269	0.608877827	0.787473436
FABP3	-0.333923008	3.216678282	2.052049407	0.152001461	0.363861514
LOC768772	0.320683639	3.2171529	6.155927013	0.013097245	0.075127552
RNF213	0.355762623	3.217412427	8.144272777	0.004319738	0.034316908
STC1	0.043469562	3.217674301	0.140685248	0.707600731	0.848656708
UCKL1	0.559609197	3.217784551	12.12063452	0.000498669	0.007235982
FANCB	0.104633353	3.220159598	0.548214623	0.459048117	0.681397294
MIR7466	-0.197111546	3.221438096	2.379956149	0.122900997	0.317627753
PLA2G2E	-0.189936628	3.221915425	2.777499379	0.095597323	0.270889262
P2RY1	-0.398124219	3.22235524	5.361802178	0.020582493	0.10100503
DNAH1	-0.432012777	3.226189899	8.465770388	0.003618919	0.030345014
IFT43	0.020925168	3.226771896	0.0361266	0.849254287	0.929809585
PLEKHD1	-0.602126592	3.227166846	20.85646433	4.95E-06	0.000183458
ZNF385B	-0.139673898	3.227382684	0.741513556	0.389176463	0.623068564
ASB2	0.239571936	3.228374485	3.026464761	0.081916321	0.245826006
GREM1	-0.671168085	3.228941291	3.605933736	0.057573729	0.19718796
DUSP5	0.160365268	3.229749821	0.823872734	0.364050297	0.599884603
LOC419429	0.126207866	3.231283246	0.527702834	0.46757424	0.687221568
INPP1	0.338969341	3.231806448	6.350474032	0.011734994	0.069426128
FAHD1	-0.098134332	3.232394139	0.920386462	0.33737352	0.57457428
TAL2	-0.462565549	3.232428413	7.572919279	0.005925174	0.042984717
P4HTM	-0.009398815	3.233543785	0.005572816	0.940492158	0.974411302
TRIM47	0.392339969	3.234425085	7.409971097	0.006486337	0.045954099
ALS2CL	-0.000768491	3.234565638	3.45E-05	0.995310876	0.997675467
TMCC3	-0.5729576	3.234832896	7.796712666	0.005234137	0.039471921
MKRN2OS	0.168080606	3.236036625	0.904024626	0.341704848	0.578651155
ADGRF5	0.066852231	3.236492186	0.2003576	0.654432356	0.815640639
AKR1B1L	1.591811814	3.23801571	12.89178005	0.000330028	0.005277568
MMP11	-0.205985391	3.238141741	2.289917089	0.130216879	0.330156971

CIB2	0.243066087	3.238350516	4.593377534	0.032095707	0.135453928
RND1	0.211033898	3.2388254	3.787457415	0.051638032	0.183378723
CACNG3	-0.788155731	3.238844369	25.1015795	5.44E-07	2.89E-05
IL10RB	0.053138738	3.239194923	0.258620796	0.611069274	0.788886309
ACTL6B	0.010151165	3.242283684	0.003259414	0.954472483	0.980523198
RWDD2A	-0.455677324	3.243304617	10.92027293	0.000951176	0.011576627
TXNL4B	-0.277908142	3.24430606	4.807852179	0.028330334	0.124748861
CCDC73	-0.181725981	3.244345716	1.950132628	0.162572559	0.378860143
CADPS2	0.180658276	3.245413016	2.268959376	0.131988501	0.333361256
LOC112533194	-0.494128076	3.245870695	4.502044126	0.033854361	0.140118054
HMBOX1	0.141259782	3.246255799	1.058340612	0.303593958	0.541708803
LOC418114	0.762307604	3.246800088	38.09977337	6.72E-10	7.79E-08
MUSK	0.17970021	3.246982922	0.991986446	0.319257354	0.557033163
NUDT19	0.333107449	3.248205532	5.199552925	0.022592698	0.107433013
SAMD14	-0.169524026	3.249047559	0.930360487	0.33476927	0.572087272
SDHAF3	0.123086296	3.24940302	1.099729082	0.294325567	0.531277666
ING2	0.047726187	3.251212203	0.161819851	0.687486521	0.837192573
CKLF	-0.369083338	3.251238336	9.406501325	0.002162174	0.021215608
EPT1L	-0.003600221	3.251451289	0.000667309	0.979391074	0.99115393
CECR1	-0.316596932	3.251478577	7.597727306	0.005844192	0.042570245
NEUROD1	-0.554714938	3.252490966	5.217612491	0.022359229	0.106860618
LOC107052205	0.062179461	3.253088406	0.219671636	0.639290229	0.806959245
PARVA	-0.202848188	3.254056727	1.775267677	0.182731504	0.405180824
TASP1	-0.039818492	3.254552906	0.132922019	0.715421422	0.852753314
C6H10orf11	-0.020258363	3.254949444	0.019911161	0.887785482	0.949358246
CDK20	-0.060054632	3.255241004	0.235386683	0.627558134	0.800067335
PTPRN	-0.005527595	3.255856537	0.002030799	0.964055986	0.984735636
GALNT9	-0.33502192	3.256369167	3.735675644	0.053262122	0.18719099
COL17A1	-0.767022618	3.257025854	12.96370956	0.000317587	0.005143601
CHST9	-0.264077643	3.257355542	2.921538843	0.087404473	0.256233851
LOC107054075	-0.231617065	3.258034398	1.761778665	0.184402815	0.407006953
FBLN7	-0.064097255	3.258403343	0.276106863	0.599264345	0.781804074

GGT1	-0.195460115	3.258837398	1.846101092	0.174237542	0.393667274
TRPV2	-0.298578456	3.260625447	5.989475897	0.014391478	0.079896007
KLF8	-0.195689826	3.261805634	2.029599642	0.154261239	0.36713461
LOC107052945	-0.113854103	3.262751621	0.846035164	0.357676396	0.59400061
MBLAC2	-0.04995784	3.26313588	0.1561422	0.692733425	0.839401545
ZNF385A	0.206057618	3.263562596	3.007922772	0.082858411	0.247762159
COBL	-0.468539025	3.263818899	7.374020616	0.006617282	0.04653862
CZH5ORF63	-0.25841008	3.263853885	2.918517739	0.08756827	0.256476892
ACOT11	-0.009834782	3.264025485	0.003285976	0.954287552	0.980523198
ST6GALNAC4	0.171498836	3.264334015	1.908058653	0.167179066	0.385176636
HBBA	-0.60790893	3.264479424	1.220451951	0.269271975	0.504027293
LOC107053678	-0.212036395	3.264553194	3.073024395	0.079601253	0.241578499
FAM206A	-0.092788704	3.266340499	0.716969152	0.397139885	0.629797549
B9D2	0.007276187	3.267206789	0.00456555	0.946128848	0.977126103
MMP23B	-0.257236083	3.268394331	3.968548786	0.046357709	0.170842892
RAPGEF4	-0.206033646	3.269885091	2.935232345	0.086666193	0.254978044
NSUN6	-0.179233364	3.270069081	2.603526104	0.106626247	0.290582128
LOC100858426	-0.355126799	3.270340294	9.436242038	0.00212739	0.02094925
LOC101748084	-0.211615494	3.270348459	2.917948303	0.087599181	0.256476892
UNC5D	-0.096152468	3.270399846	0.270126875	0.603246676	0.784359775
MTF1	-0.354246186	3.27190204	5.798498968	0.016039862	0.086309983
SLC24A5	0.040000917	3.271944741	0.123373731	0.725403826	0.858483911
BBS10	0.083560776	3.273266628	0.384859343	0.535014004	0.736769222
PBLD	0.5896538	3.275210529	27.57730408	1.51E-07	9.74E-06
MIR7476	0.044720567	3.27654568	0.179247276	0.672020919	0.826403425
CPEB3	0.00332073	3.277452562	0.000611643	0.980269194	0.991676559
DNAL1	0.170244441	3.277469421	2.13289115	0.144168607	0.352809999
LOC107053089	-0.510526486	3.277487322	4.050923794	0.04414718	0.16593007
CSNK2B	-0.228697454	3.278307527	3.786100005	0.051679931	0.183392983
RXRG	0.148561088	3.279152425	1.36710186	0.242310449	0.475074917
PAK5	-0.201793651	3.279581641	2.072075448	0.15001725	0.361281572
GRIN3A	0.004036417	3.279891686	0.000708397	0.978766224	0.990993103

LHFPL3	-0.392306489	3.279924981	10.06516556	0.00151099	0.016278211
LOC107051953	0.314933575	3.281068994	5.575013707	0.018218522	0.093279422
HAGH	0.242192254	3.281798264	3.006257203	0.082943607	0.247845934
LOC101747934	0.478798221	3.281910502	14.60282414	0.000132716	0.002569216
MHCIA9	1.878619716	3.283594078	28.77668929	8.12E-08	5.59E-06
COQ10A	-0.054256506	3.283668049	0.281876507	0.595473917	0.778958845
PDE7B	-0.010907855	3.285785458	0.008595681	0.926131654	0.967021008
STARD5	-0.301916018	3.28656944	4.489001688	0.034113593	0.140898388
LGI3	0.346911063	3.286798501	4.095589816	0.042995226	0.163356737
LOC107052292	-0.417881506	3.287723394	6.342540141	0.011787596	0.06964812
FCGBPL	-0.420707606	3.290171904	3.924093763	0.047598838	0.173744207
CLC2DL4	-0.171571701	3.2901869	1.686906591	0.194009251	0.418684257
LRRN2	-0.398781831	3.291286733	3.177555476	0.074656342	0.232444675
C33H11orf95	0.224387757	3.292117376	4.39013543	0.036147442	0.146251821
SLC12A5	0.003596069	3.292247999	0.000892283	0.976169856	0.990025465
MIR1306	-0.134562562	3.292685803	1.077741218	0.299203411	0.536317225
LOC101750925	0.426750229	3.292814186	7.41224557	0.006478142	0.045954099
GPR37L1	0.184747909	3.293944039	1.313418891	0.251776578	0.486494081
PANX3	-0.248357685	3.295153627	4.037607065	0.044496874	0.166612097
SH3BP2	-0.203606534	3.295458434	3.380555835	0.065969803	0.215420225
KLHDC7A	0.059552637	3.295752728	0.233932234	0.628623341	0.800557006
SMIM3	0.08457509	3.296439585	0.462887288	0.496277678	0.7079291
LOC107054750	-0.28327867	3.296564053	5.986626316	0.014414746	0.079917332
FAM105B	-0.07733301	3.296630456	0.53783687	0.463330583	0.684451453
VOPP1	0.061990335	3.297445185	0.209637592	0.647051604	0.811526992
LOC107050512	-0.512394797	3.297529592	3.813963847	0.050827033	0.181580292
TNFAIP3	0.151930748	3.297576307	1.857394371	0.172925829	0.392303049
FAM185A	-0.158854014	3.297604424	1.900380012	0.16803579	0.386069383
MNX1	-0.299019616	3.298249284	3.209221111	0.073224333	0.229108265
LOC107050300	-0.331559662	3.299367743	7.337180751	0.006754269	0.047133796
H2AFX	0.193116632	3.300446978	1.633571745	0.201209737	0.427820936
LGI1	0.084342348	3.300602673	0.479646236	0.488582601	0.702150803

KNDC1	0.158165787	3.301963198	0.979522206	0.322316794	0.559907128
LOC107051359	-0.07769007	3.303313628	0.337703116	0.561158037	0.75591721
ST6GAL1	-0.249837263	3.303882755	3.850010552	0.049745678	0.179038742
OGN	-1.262809425	3.303889583	18.09572445	2.10E-05	0.00058391
GLYATL3W	0.073289987	3.304317799	0.26292544	0.608117424	0.787414637
TMEM174	-0.076640486	3.306315729	0.337435366	0.561313331	0.75591721
SV2C	0.081439446	3.306329276	0.217552571	0.640911098	0.807951591
DOLK	-0.141195981	3.30838987	1.403688141	0.23610703	0.468336464
RIN3	-0.377759192	3.30860716	10.18361057	0.001416945	0.015523184
AEN	-0.195865924	3.309384399	1.717688016	0.18999092	0.413388669
RSPH9	-0.195277832	3.310752966	3.309945743	0.068861766	0.221096654
KIAA1755	0.085846164	3.311369413	0.466611085	0.494550373	0.706653907
AK3	0.056237573	3.311950018	0.202165647	0.652978448	0.814861243
CLEC19A	-0.150850342	3.311969775	0.944823887	0.331040341	0.568878379
DOK1	-0.09823963	3.312325509	0.86997001	0.350963611	0.587351013
PACRGL	-0.035357127	3.313265545	0.073303272	0.786586571	0.894985619
AP4S1	-0.03574372	3.314143754	0.10164941	0.749859185	0.873444126
CHRD	-0.370045689	3.314854705	9.553713814	0.001995453	0.020148057
PCSK2	-0.107083562	3.31518085	0.776369474	0.378253789	0.613097527
LOC112530071	-0.281242883	3.315435549	6.508259521	0.010737455	0.065149273
LOC107052719	3.307530347	3.316053529	205.0658036	1.64E-46	1.35E-42
LONRF1	0.020053052	3.317031063	0.033080092	0.855677423	0.932779908
MTERF4	0.123678808	3.317375949	0.92567963	0.335988093	0.573217632
WASF3L	-0.115646257	3.320381105	0.828273648	0.362772197	0.599097263
PACSIN1	-0.196552629	3.320751111	3.17085106	0.074963375	0.232906818
SCARA5	-0.137710901	3.321034048	0.692087423	0.405455277	0.636628942
SIAH2	-0.016560326	3.321362596	0.022600409	0.880500855	0.946351332
INPP5J	-0.207782917	3.321641132	2.83298913	0.092346333	0.265645587
LRRC38	-0.101953849	3.321656981	0.891129883	0.345171304	0.581886467
SEZ6	-0.743187479	3.322053309	8.588284417	0.003383326	0.028801153
ESRRG	-0.082697857	3.322219855	0.475888983	0.490290363	0.703822194
SLCO4A1	-0.532334707	3.322855289	9.159801534	0.002473894	0.023077055

LOC112532802	0.087551565	3.32293753	0.386540563	0.534123457	0.73595064
RBM41	0.283050002	3.323060968	6.586636067	0.010274714	0.063191969
LOC100859284	0.140930257	3.323369571	1.670567846	0.196182493	0.421049032
LYPLAL1	0.068325544	3.323650923	0.303609696	0.581628265	0.770401119
ABHD1	0.162077498	3.324479696	1.560202692	0.211635545	0.439205813
IQCC	-0.040672426	3.324530289	0.137604384	0.71067429	0.849781439
KIAA0513	-0.174349616	3.324689982	1.6049709	0.205200182	0.432066836
BHLHE40	-0.074607496	3.324821662	0.434248585	0.509911562	0.718249851
ATP8B3	0.37044794	3.325873425	4.554625594	0.032829941	0.137623659
RAB32	0.023717894	3.326987128	0.052716283	0.818402399	0.914146449
LOC100859020	0.271197772	3.32740314	3.005263206	0.082994496	0.247943798
RFK	0.169238834	3.327442436	1.12771026	0.288264272	0.525003295
UPK1B	-0.780034392	3.327856678	11.89972969	0.000561421	0.00788956
LOC107051983	-0.282313165	3.330416078	3.001029279	0.083211637	0.248276968
CD40	0.093531793	3.33073256	0.467925569	0.493943055	0.706154037
CHRM2	-0.066431035	3.332004284	0.14498176	0.703377852	0.846389149
AIM1	-0.266819074	3.33208994	4.445761682	0.034987979	0.143002285
SYT14	0.194051827	3.33248373	1.753702571	0.185411944	0.407990577
PASD1	-0.437297793	3.335018899	10.09009725	0.001490682	0.016116412
ABCG4	-0.415125363	3.335831487	9.13940355	0.002501626	0.023243513
SBK1	-0.010673036	3.340242381	0.004039075	0.949325588	0.978504123
DYX1C1	0.041803808	3.341944196	0.10738112	0.743145622	0.869610383
NUDT12	-0.268754403	3.343826357	4.104032623	0.042781059	0.163008754
HTATIP2	-0.270840264	3.344025107	1.954371299	0.162116579	0.378333331
ADAMTS20	0.238019915	3.344269816	3.088434389	0.078850641	0.239970995
SEZ6L	-0.115122207	3.345567471	0.264744154	0.606879432	0.786666377
DNM3	-0.192800885	3.345840229	2.028528847	0.154369972	0.367287145
MAP7	0.078298274	3.345856137	0.275449794	0.599699212	0.782152423
ACAP1L	0.2635938	3.34591851	3.401286724	0.065145583	0.213625062
RASL11AL	0.349108017	3.346090485	8.402351623	0.003747359	0.031033114
TPST2	-0.14237982	3.346204203	1.278343931	0.258207181	0.492671516
ANTXR1	-0.341099206	3.346816871	1.84116893	0.174814003	0.394310525

KCNA3	-0.016059907	3.347639437	0.013462612	0.907629957	0.957538022
DOK7	-0.245997194	3.348087911	4.512167919	0.033654559	0.139880214
MRC1	-0.082630687	3.348658511	0.227732207	0.633210485	0.803815734
RET	0.085635675	3.349470262	0.224728226	0.635460624	0.804702787
LOC101750931	0.278686483	3.350615097	4.255103965	0.039132553	0.154012474
DZANK1	-0.07386623	3.351616231	0.483018707	0.487058139	0.701368835
CASQ2	-0.014063836	3.352359849	0.003513565	0.952732808	0.979802073
LHFPL2	0.022033038	3.354183789	0.031623293	0.858856963	0.933947352
KCNB2	-0.282232522	3.354610931	3.244586961	0.07165975	0.226288278
LOC107051165	-0.175659387	3.35557682	1.406535006	0.235632455	0.467852909
SLC35G2	-0.081864907	3.3557395	0.615019551	0.432904096	0.659478488
ACSF3	0.020374802	3.357487419	0.03355019	0.8546669	0.932410252
UBTD1	0.061617804	3.357631458	0.347370021	0.555605686	0.752912164
LOC107051772	0.011767853	3.35875992	0.008952775	0.924617373	0.966229608
PRDM5	-0.183272547	3.359356794	2.76378351	0.096419959	0.272235831
ACVRL1	0.116793516	3.359358097	0.941829871	0.331807689	0.56937808
DAO	0.536600191	3.359766547	23.41804716	1.30E-06	5.93E-05
OGG1	0.073441739	3.360976877	0.287802398	0.591632259	0.776938793
DNASE2B	-0.401727561	3.362409829	13.09465473	0.00029614	0.004877855
KCNK12	-0.151454479	3.363554194	1.403623288	0.236117855	0.468336464
RAPSN	-0.026947897	3.36377229	0.039777862	0.841915537	0.925494032
SLC9A7	-0.40239856	3.36380195	10.85922181	0.00098305	0.011888369
AIG1	-0.133689538	3.365598273	1.567620392	0.210552927	0.438234642
LOC107051875	-0.788353621	3.36599259	17.40246867	3.02E-05	0.000788674
LOC107054179	-0.210790734	3.367347741	2.942140493	0.086296307	0.254526927
LOC107050733	0.151432867	3.367612426	0.915331881	0.338703661	0.57576162
ST3GAL2L	-0.353470523	3.37006528	10.02525244	0.001544085	0.016520104
FAM131B	0.154007719	3.370395225	1.201914515	0.272939364	0.507692808
ARHGEF26	-0.20665391	3.371805644	2.620259792	0.105507166	0.288631822
SMPDL3B	-0.116678302	3.372459525	1.383811237	0.23945302	0.472049772
EML6	-0.314450999	3.37246709	5.752875229	0.016461693	0.087804588
LOC107051594	-0.358229088	3.372581104	12.55711116	0.000394701	0.00606425

LOC101748692	0.00461011	3.374259468	0.000519114	0.981822508	0.991828264
COX14	-0.131265455	3.37465568	1.061994387	0.302760752	0.540559362
ATOH8	0.145405717	3.374934955	1.84133538	0.174794513	0.394310525
CLEC18B	0.078980248	3.375295946	0.251554273	0.615982786	0.792054659
QPRT	-0.323316339	3.376090845	0.514934879	0.473010353	0.691119282
STK32C	0.25101549	3.376538287	4.733656808	0.029577935	0.127945565
AGPAT2	-0.149368225	3.377131195	1.288840143	0.256261808	0.490952153
VLDLR	-0.166525926	3.377445831	1.321273722	0.250363585	0.484683162
LOC112530930	0.081886106	3.377654415	0.504079154	0.47771324	0.694090848
LOC100858032	0.142344657	3.377955231	1.367443452	0.24225162	0.475074917
MAPKAPK3	0.176522253	3.378489252	1.941056595	0.163553857	0.380007306
NUDT6	-0.171796482	3.37929608	2.398245888	0.121471388	0.315600463
MINPP1	-0.128338332	3.380679591	1.468860318	0.225525596	0.455396206
MRVI1	-0.254519889	3.381043472	2.103193318	0.146991867	0.356570325
LOC107053353	-0.38620527	3.382447022	9.077720811	0.002587423	0.023774878
GRIA2	-0.410568317	3.383116696	7.048669308	0.007932416	0.052881874
GRHPR	0.473054304	3.383546804	14.02218114	0.000180667	0.003286574
DSC1	0.02964101	3.384889517	0.020749057	0.885464675	0.9486918
TAS1R1	-0.062847462	3.385056833	0.322333081	0.570208911	0.762518296
GAMT	0.09193106	3.385269243	0.437486523	0.508338105	0.717589112
LOC107050964	-0.023799388	3.385874633	0.039713506	0.842041785	0.925549512
LOC107053352	-0.306385466	3.387656807	3.784899291	0.051717023	0.183445485
AMIGO2	0.260387802	3.38798416	5.051372012	0.024606447	0.11381102
STARD9	-0.415524492	3.389561799	6.933317566	0.008460461	0.055285999
XKR7	-0.108621618	3.391980465	0.426371107	0.513774984	0.720949939
SEC11C	-0.075991548	3.392440731	0.420769456	0.51655335	0.722717913
HISTH1	-0.234079587	3.393093197	2.824885918	0.092813487	0.266209853
STMN4	0.303574198	3.393659677	1.876064275	0.170782144	0.389517765
CDKL1	0.189624978	3.393748988	2.816690961	0.093288544	0.267199824
TDRKH	0.826554291	3.395391374	26.74730114	2.32E-07	1.37E-05
PDGFRL	-0.050796293	3.39560311	0.186387465	0.665940462	0.823170847
LOC107051702	0.00225694	3.395916432	0.000266727	0.986969715	0.993855129

2KTGRL	0.501532348	3.396091566	16.99251931	3.75E-05	0.00094421
SLAIN1	0.139558516	3.396112658	1.26613115	0.26049373	0.495196896
ACOX3	0.530360836	3.396887657	14.90141221	0.000113278	0.002255722
GNG11	-0.350414643	3.398027042	7.331563284	0.00677541	0.047201258
PAXX	0.117459302	3.39818029	1.249635144	0.263622174	0.498724175
FSBP	0.258742791	3.398349512	4.953041421	0.026044811	0.118192876
GPR17	-0.560731912	3.399137071	9.698678139	0.001844006	0.018952605
DRD5	0.012785112	3.399352435	0.013019783	0.909155143	0.95829261
GLT8D2	-0.397234301	3.399958513	6.360389732	0.011669591	0.06925079
SYNDIG1	-0.151395355	3.400741645	1.093193106	0.295764801	0.532600858
OPA3	-0.04449841	3.400755067	0.132130178	0.716233541	0.853085451
LRRC7	0.150539486	3.402110526	1.569878747	0.210224623	0.438045609
RCCD1	0.352091589	3.40229315	9.451189824	0.002110122	0.020869886
LOC101751742	-0.393658278	3.403204321	6.159627098	0.013069876	0.075013885
LOC112531805	-0.119127768	3.403931342	1.161481886	0.281158422	0.51686536
C19orf60	0.183094563	3.405822549	1.689673351	0.193644034	0.418164381
LOC107049457	0.08282865	3.406013181	0.435396511	0.509352774	0.717892027
SNX29	-0.239020032	3.406648938	3.574967615	0.058656593	0.199525308
ARSB	-0.148439514	3.407276831	1.524725498	0.216905783	0.44609232
KLHL8	-0.282662992	3.408101043	5.894052608	0.0151921	0.082722039
MANSC1	-0.1283645	3.408203665	1.259415818	0.261761697	0.496795159
FBXO8	-0.243009602	3.408676085	4.93844776	0.026265614	0.11876908
TESK1	-0.1105643	3.410822613	0.636395728	0.425018978	0.653554554
LOC776594	-0.190569319	3.411010725	2.468268052	0.116165865	0.306921854
NPY2R	-0.403595661	3.411655062	3.290667774	0.06967468	0.222577531
SYT4	-0.224772253	3.412718625	0.696452198	0.403978381	0.635646128
NPL	-0.058026564	3.414503657	0.179393814	0.671894706	0.826371731
MIIP	0.082134536	3.415243289	0.389468115	0.532579116	0.734892189
LIN7A	0.021282072	3.416240681	0.032522752	0.856885141	0.933242474
LOC112531249	-0.334202473	3.417012062	6.766625375	0.009287829	0.058871815
TMEM8B	0.314406111	3.417946667	2.958244947	0.085440635	0.252637134
LOC770653	0.559763212	3.41824897	10.78699703	0.001022156	0.012232416

F2RL2	0.189617074	3.419187502	1.405282866	0.235841046	0.467900217
ALX4	0.016409297	3.419940936	0.00411128	0.94887526	0.978488589
SMYD3	-0.35124022	3.419997299	8.377371318	0.003799215	0.031320685
LOC112533441	-0.422904657	3.420047556	13.4126048	0.000249939	0.004302038
DPF3	0.067777079	3.421317227	0.298655253	0.584726687	0.771586017
DGKH	-0.248461189	3.422210118	1.157002412	0.282088082	0.517764573
FAM151BL	0.040804918	3.423999529	0.078697045	0.779070962	0.890625824
RIMS2	-0.184254718	3.424434748	2.054170225	0.151789927	0.363587427
TOR1AIP2	0.048319798	3.424472004	0.19865099	0.655811939	0.816415906
CPN1	-0.130493799	3.425280835	1.020588838	0.312379376	0.550288717
LOC415664	-0.227441804	3.425575884	1.573945845	0.209634905	0.437426118
CYP4A22	-0.091256867	3.426811972	0.731537848	0.392385166	0.625832839
LRRC39	0.008142088	3.426929597	0.004521821	0.946387068	0.977331442
MATN4	-0.032527041	3.427000396	0.018751612	0.891080966	0.950952546
EDARADD	-0.145436664	3.428459248	1.308346206	0.252694302	0.487280718
ZBTB45	-0.100446074	3.429087424	0.987132151	0.320444313	0.557802938
LYRM4	-0.056684539	3.43074059	0.315444458	0.574358307	0.765040788
LPL	0.067530648	3.430941292	0.327166421	0.567332385	0.760711734
ARSG	0.277938195	3.433147449	3.689810302	0.054745842	0.190453031
MME	-0.012186652	3.433766169	0.004952735	0.943894657	0.975921243
LOC768709	0.122408044	3.433769689	0.916023929	0.338521129	0.575738718
DSEL	0.163610111	3.433873346	1.557020823	0.212101963	0.440017739
MIER3L	0.462175157	3.433986041	1.492915991	0.221764578	0.451179047
RMDN1	-0.128244985	3.434018548	1.256477947	0.262318822	0.497574203
CSDC2	0.074101922	3.43404434	0.340055894	0.559796961	0.755223333
SNX16	0.083986631	3.434737816	0.753043425	0.385514372	0.620040953
MAOB	0.477433783	3.43523259	8.940083506	0.0027898	0.025099045
GAS6	-0.511218916	3.436081365	10.9214929	0.00095055	0.011576627
OBSCN	-0.099420215	3.437239436	0.15372483	0.695000892	0.840961811
MXD3	0.161479883	3.437506678	1.476069218	0.224390544	0.453917322
LOC107054623	0.222707893	3.437767357	3.600824931	0.057750907	0.197524669
CEP128	-0.16941385	3.438880351	2.408886208	0.120648179	0.314024958

DLG2	0.108400933	3.439040674	0.902321472	0.342160001	0.579261353
VAMP1	0.053673754	3.440257447	0.170836841	0.679369149	0.831648516
ADCK1	-0.242239852	3.440900439	3.992173697	0.045712056	0.169412587
C4H4orf33	0.431867148	3.441089574	9.940420462	0.001616886	0.017131913
LOC107052009	-0.285207192	3.441752598	1.705668458	0.191548332	0.415657101
NINJ2	-0.608256734	3.442043672	16.78919882	4.18E-05	0.001034611
TNFRSF11A	-0.372790698	3.442291659	9.207789792	0.002409875	0.022702947
SLC37A2	0.08404997	3.44319983	0.435875548	0.5091199	0.717892027
HSPB9	0.236158175	3.444954629	1.500262925	0.220630911	0.450049422
TOM1L1	-0.414603569	3.445018122	2.162227625	0.141439511	0.348411251
GJD4	0.219828475	3.445638647	0.985625395	0.320813922	0.558298979
TIGD5	0.051801623	3.446569651	0.246799622	0.619337632	0.794431043
AMY2A	-0.324375731	3.446570021	5.51460408	0.018858341	0.095422511
TMEM35B	0.437882538	3.446761717	8.032197219	0.004595306	0.035964506
ADGRG6	-0.073644574	3.446796756	0.315438027	0.574362209	0.765040788
LOC112532514	-0.416928896	3.447788592	12.10052893	0.000504075	0.007301547
TP73	0.023445731	3.447889111	0.039951016	0.841576386	0.925494032
CYB561A3	0.091865691	3.448777136	0.767715607	0.380924677	0.615531007
PCGF1	-0.262028368	3.448911519	4.791570231	0.028599343	0.125601973
C1H12orf50	-0.091566908	3.450531391	0.577094608	0.447453401	0.670925335
POMK	0.040933093	3.451350586	0.170095232	0.680027186	0.832144519
COL19A1	-0.087007447	3.452662277	0.363988249	0.546299564	0.746341738
PLPP1	-0.11382509	3.453577188	0.731972215	0.392244663	0.625832839
MFSD7	-0.162155084	3.45361382	1.372082017	0.241454486	0.474290745
HIVEP3	-0.08353182	3.453908996	0.464890812	0.495347072	0.707178212
LOC107054087	0.172297157	3.454154831	1.38604215	0.239074624	0.471812538
LOC107050901	0.84012243	3.454490678	23.59476501	1.19E-06	5.48E-05
PCMT1L	-0.096173185	3.455674199	0.678675317	0.410043247	0.64033991
SGO2	-0.213738158	3.456958985	3.76236384	0.052418424	0.185095531
LOC107055288	0.708668984	3.457044081	23.30044699	1.39E-06	6.21E-05
LOC112530189	-0.097691351	3.457355338	0.971583511	0.324285573	0.562229386
SHISA9	-0.246645028	3.45766032	4.365931857	0.036664425	0.147473261

KLHL26	0.005379131	3.457743947	0.002843394	0.957474165	0.981573571
ANKS6	-0.004953187	3.460097257	0.001810125	0.966063791	0.98589554
ACYP2	0.442931667	3.460130465	14.23316898	0.000161499	0.002999964
SOX8	-0.50757647	3.461375677	5.644524498	0.017509964	0.091211829
PLEKHN1	0.208162792	3.461507158	3.188832042	0.074142967	0.231064871
DNAJC5G	-0.297370347	3.461649261	7.346841005	0.00671807	0.047000783
SMOX	-0.123483941	3.463452594	1.293740675	0.255359735	0.490348661
LOC107049615	-0.835182807	3.46357735	19.75462389	8.80E-06	0.000296286
BBS1	0.003879904	3.463616989	0.000969277	0.975163307	0.990018742
HPCAL4	0.003302984	3.464360509	0.000843814	0.976825925	0.990428899
ZFRL1	0.552618549	3.464852327	2.185842074	0.139284868	0.344807055
CNTN3	-0.360640109	3.465183414	11.45289746	0.000713826	0.009411866
JAZF1	-0.063101003	3.465742473	0.283863517	0.59418004	0.777946576
LOC107055029	0.002047686	3.465962828	0.000332872	0.985443569	0.993236215
LOC101749404	0.536419314	3.465997429	6.038375432	0.01399816	0.078480313
LOC107054204	-0.372521423	3.466244129	1.920029531	0.165853405	0.383518518
CYP2J22	-0.274961403	3.468517991	4.344149243	0.037136317	0.148542664
LOC107053100	0.407353146	3.469593739	10.94497391	0.000938579	0.01148276
ITM2C	-0.468390199	3.469947034	18.68497935	1.54E-05	0.000461316
NCKAP1L	-0.153927888	3.469980042	1.358074581	0.243871452	0.476931869
NLGN1	-0.120253056	3.470047086	1.132203014	0.287305919	0.523893939
TFAP2E	0.193054472	3.470869343	1.744874196	0.186522417	0.40933934
IKZF4	0.020828737	3.470890855	0.046220389	0.829775679	0.918963441
GSTAL1	-0.322605066	3.471409042	6.342489131	0.011787935	0.06964812
CBLN1	0.539377921	3.472986001	7.285044303	0.006953102	0.048015912
BPHL	0.289579434	3.473950591	5.897687764	0.015160775	0.082633506
SNRNP35	-0.09788949	3.474833168	0.847962976	0.357129241	0.593652052
LOC107049666	0.179331362	3.476792328	3.390069202	0.065590196	0.214697967
ADGRG2	-0.382452731	3.477492733	5.872962021	0.015375161	0.083332764
MPPE1	0.003340401	3.477567615	0.000995278	0.974832489	0.989932647
SIDT1	0.09285007	3.477783014	0.854290047	0.35534153	0.59199604
SEC14L5	-0.030898074	3.479761656	0.072907482	0.787149595	0.895378583

POLG2	0.63423494	3.480364711	24.57093978	7.16E-07	3.65E-05
ADRA2C	0.004676751	3.480378979	0.001314014	0.971083551	0.988443637
KIAA0825	0.188834244	3.480379327	2.208762562	0.137228708	0.341618515
LOC101752215	-0.222010352	3.480904222	2.97726498	0.084441853	0.250946486
CD74	0.307047042	3.48122502	4.972130193	0.025758904	0.117283555
ERN2	0.376194899	3.483098847	11.77936125	0.00059891	0.008267676
IDNK	0.076406828	3.483347584	0.43078598	0.511603532	0.719032808
PRRT1B	0.074854491	3.483532908	0.394976009	0.529695328	0.733249485
RSPO1	0.177355432	3.483677293	1.604861537	0.205215618	0.432066836
SDC4	-0.010769212	3.484354663	0.008684292	0.925752976	0.96700725
C14H16ORF52	-0.086892777	3.486529221	0.811881254	0.367564641	0.602414195
GRIK1	0.174842927	3.486732426	1.429516762	0.231843319	0.4635017
MLYCD	-0.055036736	3.486941182	0.237643427	0.625913361	0.799043801
ERBB4	-0.233501439	3.489092955	1.520828009	0.217494234	0.446474354
MIR1652	0.008954522	3.489850061	0.005517732	0.940786447	0.974421916
MAFG	-0.146128617	3.489916541	1.907727419	0.167215919	0.385207609
MCTP2	0.215004075	3.490604687	3.137244064	0.076523009	0.236112153
RNASEL	-0.102058167	3.49080173	0.757937786	0.383974659	0.618652992
OPN2SW	0.495825	3.490862787	3.738584008	0.053169485	0.187121781
ELMO3	-0.340920615	3.49181243	10.13322448	0.001456204	0.015800397
ANGPT2	0.176671303	3.492027526	1.624705638	0.2024369	0.429146995
EFNB2	0.013570027	3.492066469	0.017760599	0.893980724	0.951763251
CGRRF1	-0.345034597	3.492256635	6.481238535	0.010901899	0.065976739
OSCP1	0.191555719	3.492713749	2.938526751	0.086489585	0.254777708
METTL4	0.142062135	3.492847889	1.157457627	0.281993431	0.517706338
LDLR	-0.277113287	3.492905306	5.76087927	0.016386869	0.087518967
LOC107050771	-0.216377768	3.493203428	3.736311014	0.05324187	0.18719099
TRIM36	-0.224581693	3.493364193	3.468683777	0.062541057	0.208111848
ANO6	-0.289210092	3.494301376	4.984586712	0.025574093	0.116765178
INF2	-0.407299698	3.494853633	10.22317653	0.001386868	0.01522409
TNFAIP8L3	-0.080276639	3.495022642	0.271301243	0.60246022	0.78367454
MIR3064	-0.112796456	3.495974993	0.578841516	0.446766768	0.670621395

LOC101749419	-0.228211197	3.496152972	1.951141668	0.162463878	0.378860143
SHOX2	-0.419873757	3.496207397	2.110484173	0.146293025	0.355567069
CDA	-0.187017248	3.496572229	2.466928769	0.116264905	0.307120817
LOC107050351	-0.166520971	3.49723541	0.962643856	0.326521658	0.564254007
UROS	0.102221586	3.497712502	0.733721334	0.391679612	0.625251069
EBF1	0.238906997	3.497894015	3.423083524	0.064290823	0.211962632
TMEM42	-0.183515912	3.498099599	2.805325602	0.093951769	0.268624477
OTUD7A	-0.167559944	3.498566796	1.964586303	0.16102367	0.376637454
CRYL1	0.353543091	3.498660147	11.11021869	0.000858534	0.010732799
LOC112531208	-1.405185853	3.499231627	22.17919622	2.48E-06	0.000101657
GTPBP6	0.228595084	3.499793823	5.091804125	0.024039171	0.112185069
F2	-0.173394975	3.500622139	1.657529093	0.197937271	0.423599662
DIO3	0.383613713	3.500842308	3.513480972	0.060871434	0.204541442
LOC107049569	0.123151757	3.501530047	1.142099843	0.285209057	0.52122557
C7H2ORF76	0.265998458	3.502340742	5.383525054	0.020327765	0.100207722
PIGM	-0.045242937	3.503392002	0.151431724	0.69717089	0.842410559
SPATA18	-0.081221461	3.506886661	0.489766831	0.484031334	0.698599737
C15orf39	0.217725741	3.507517745	2.712340801	0.099574783	0.277383283
LOC112533173	0.137755903	3.511016259	1.581052376	0.208609172	0.436226195
PDE1B	0.163642093	3.51178066	1.383530228	0.239500735	0.472087278
SREK1IP1	0.260115443	3.512138812	4.68709908	0.030389912	0.130531456
FAM110C	0.269185991	3.512372903	2.217762099	0.136430693	0.340094993
PPP1R9A	-0.505632885	3.512918437	12.46791967	0.000414001	0.006272914
ETFRF1	-0.028944215	3.515321086	0.089931731	0.764263963	0.881657566
SRL	-0.205451959	3.515470502	0.775035294	0.378663837	0.613278882
SHROOM1	-0.024299873	3.516418883	0.05574279	0.813355764	0.910770269
PRKAB2	-0.06559593	3.516472805	0.427092616	0.513419017	0.720784056
LOC107055373	-0.208360589	3.517220096	4.529093176	0.033323271	0.13895956
CYP2J21	0.403727761	3.517999678	5.785724536	0.016156837	0.086690461
GSTAL2	0.053491803	3.518375352	0.205303839	0.650473375	0.813573027
RHBDD1	0.360936782	3.518526547	9.107439968	0.002545719	0.023560072
LRRC57	0.064886006	3.518742344	0.355713232	0.55089669	0.749421712

SLC2A12	0.078217879	3.518795299	0.474315061	0.491008708	0.704284195
RPGR	0.106919103	3.518992458	0.977607773	0.322790123	0.56028602
KITLG	-0.002695004	3.51905647	0.000353286	0.985003922	0.992992257
LOC101750351	0.265957381	3.520342704	2.920312448	0.087470925	0.256382984
LOC101747677	-0.075430585	3.521274448	0.219565026	0.639371547	0.806971567
GGA2	0.132207463	3.522365983	1.570610613	0.21011836	0.438045609
LOC431003	0.024372487	3.524185535	0.018522244	0.891745034	0.951008202
S100A6	0.299264299	3.525576741	3.309132746	0.068895842	0.221162912
ASRGL1	0.087185691	3.525624192	0.852076643	0.355965529	0.592375887
FAM64A	0.105652726	3.525795231	1.290738868	0.255911831	0.490715523
KIAA1024	-0.528332002	3.525870011	10.0063562	0.001560009	0.016647174
TRIM45	0.513701555	3.526530478	19.71437051	8.99E-06	0.000299042
MCF2L2	-0.522902886	3.526568195	10.71667562	0.001061743	0.012550988
BEND2	-0.267650942	3.52661057	3.970837515	0.046294741	0.17068787
CLN5	0.364608356	3.527191281	10.16592549	0.001430601	0.015641553
LOC107052651	-0.497112787	3.527225489	11.32681926	0.000763956	0.009851805
NAV3	-0.467139798	3.527458685	13.1816739	0.0002827	0.004717881
TRIM63	0.5609763	3.527463753	10.83190958	0.000997657	0.012017895
COL15A1	-0.045505284	3.528557688	0.128467856	0.720025938	0.855582231
HYAL3	0.036952623	3.529229235	0.073156128	0.786795697	0.895117541
COLEC10	1.768137553	3.529395791	10.6955049	0.001073962	0.012631907
SLC27A2	0.016612166	3.529778192	0.022028601	0.882011039	0.947277107
LOC107053815	0.355174818	3.530285442	8.004431131	0.004666302	0.036385377
ZBTB8B	-0.197789719	3.530704169	3.698651249	0.054456471	0.189892433
LYSMD1	-0.120517707	3.532255379	0.894150159	0.344355127	0.581257825
SHC4	0.577834928	3.53230742	27.34972639	1.70E-07	1.07E-05
JADE1	-0.012941922	3.533533596	0.014376214	0.90456169	0.956421967
ALDH1A3	0.325533961	3.533646427	2.206232606	0.137453987	0.341920688
ARHGAP6	-0.048953396	3.533925211	0.148849808	0.699636954	0.843779396
SLC25A19	-0.069214322	3.535599834	0.383960819	0.535491059	0.7371794
LBP	0.264175329	3.536756152	4.701084517	0.030143591	0.129676545
SMPD2	-0.110456111	3.537983651	1.016392837	0.313376169	0.551390597

RNF122	-0.163274573	3.540230234	1.344821983	0.246185389	0.47986029
C11H16ORF87	0.040651094	3.541057929	0.153728239	0.694997679	0.840961811
SPA17	-0.232197036	3.542378376	2.917710924	0.08761207	0.256476892
JMJD7	-0.322902021	3.542412145	10.54739396	0.00116352	0.013379263
PDE4D	0.175547204	3.542670266	1.858045159	0.172850587	0.392257123
NF2L	0.089045636	3.54342149	0.356637656	0.550379551	0.749027805
TRAF1	-0.346284441	3.544548596	7.074489197	0.007818911	0.052343441
JAM2	0.129475513	3.54522768	1.435945462	0.230796581	0.462262064
RPH3A	0.125918654	3.54601565	0.716508872	0.397291456	0.629810299
CEP76	-0.051092075	3.546539741	0.286825554	0.592262009	0.776980894
TOM1	0.095925256	3.547126287	0.875719375	0.349376795	0.586080157
TPGS1	-0.12271103	3.547867061	1.017922718	0.313012253	0.551100049
ABCG2	-0.266791419	3.548213819	7.287414121	0.006943935	0.048015912
GHDC	-0.63383811	3.549892959	19.0313352	1.29E-05	0.000394746
KRT17	-0.693957478	3.550547461	6.214223108	0.012672795	0.073419493
LOC427369	0.046852054	3.55107845	0.143325631	0.704997015	0.847186196
LOC112533354	-0.786071506	3.552141572	36.174993	1.80E-09	1.89E-07
IKBIP	-0.227565173	3.554696285	4.035799262	0.044544571	0.16669233
LOC107050626	-0.215978626	3.555818231	4.272110396	0.038742785	0.152734193
DNAH3	-0.368744754	3.556234434	4.988622513	0.025514512	0.116705787
KCNMA1	0.25219501	3.558387091	2.689786371	0.100993353	0.279967694
ACYP1	-0.052830919	3.55867549	0.271943287	0.602031168	0.783443547
TRPC3	-0.309535429	3.559505676	5.704954773	0.016917091	0.0893933
SLC45A3	0.034319862	3.559528688	0.050800967	0.821675348	0.916282016
GALNT12	0.059320121	3.560803706	0.243586892	0.621627391	0.795959747
SLC25A43	0.176250054	3.561014888	2.994364083	0.083554711	0.249154317
LOC112532875	0.020095945	3.561262615	0.03880734	0.843830835	0.926328616
VWC2L	0.669203419	3.562226686	25.86107341	3.67E-07	2.09E-05
LOC101749175	0.244945964	3.562329083	3.325563036	0.068210655	0.219520112
PHYH	0.058597058	3.562808826	0.22937552	0.631987258	0.803037455
OSR1	0.49922605	3.563978484	6.254952909	0.012384654	0.072240157
BRF2	-0.031163947	3.564568317	0.081010483	0.775932677	0.889134555

SIL1	-0.341716251	3.564836326	7.623284867	0.005761944	0.042101594
PTPRT	-0.115353893	3.567071717	0.651385071	0.419617916	0.649223958
RUBCNL	-0.33448864	3.567245327	6.929923904	0.00847653	0.055316575
LOC107050886	0.310759941	3.569042125	5.199259286	0.022596515	0.107433013
LOC107051764	-0.524092918	3.569338934	25.03065916	5.64E-07	2.98E-05
RORA	-0.008958194	3.570256834	0.004047837	0.949270726	0.978504123
TOX2	0.054076204	3.570439927	0.085753395	0.769646894	0.88505745
LOC426355	-0.29447585	3.571320048	2.499199403	0.113904189	0.302842189
GLB1L	-0.226557278	3.571462303	2.505337278	0.113461191	0.302406036
LOC426743	0.384332074	3.571739538	12.59570347	0.000386635	0.005968171
CCDC91	-0.0046361	3.572835905	0.002277557	0.961936397	0.983899168
DPP6	-0.064502956	3.574808006	0.198266291	0.656123901	0.816485578
TTLL9	-0.292362803	3.575045616	5.415424369	0.019959601	0.098983362
CFTR	-0.080201925	3.57588452	0.374540041	0.540539897	0.741338891
PHF19	0.03430665	3.576227161	0.080250239	0.77695858	0.889536847
PSPH	0.264052182	3.57688103	5.768371673	0.016317147	0.087272584
CCDC142	0.109488162	3.577192653	0.680694754	0.409347591	0.639836068
TIMP4	-0.049828164	3.577216766	0.160055075	0.689105815	0.83810791
KLHL14	-0.362108583	3.577805244	9.444893592	0.002117378	0.020892566
GLMP	-0.169500627	3.579897843	2.792178368	0.09472538	0.269554615
COLEC11	0.51871066	3.580071147	12.68367632	0.000368862	0.005753192
HELQ	0.228668753	3.580166414	3.161787303	0.075380613	0.233726773
TWIST2	0.065325834	3.580392339	0.111843476	0.738055047	0.866549825
BCDIN3D	0.02766262	3.580878898	0.072807791	0.787291666	0.895403091
C5H11orf49	0.071917931	3.581013132	0.498488455	0.480165036	0.696466157
RUNDC3A	0.210887365	3.581032334	2.432403798	0.118850522	0.311266169
MFSD12	0.030546577	3.581497068	0.088980699	0.765477006	0.882561949
LOC770705	-3.834441674	3.582968175	39.92271172	2.64E-10	3.51E-08
LOC112532890	-0.059025352	3.583047537	0.223740834	0.636204255	0.80506714
SOCS4	0.084937159	3.583086436	0.790487656	0.37395271	0.608644099
CHRNB2	-0.066124579	3.583697821	0.334097265	0.563256372	0.757599665
DMGDH	0.432491853	3.58405558	8.7835828	0.003039536	0.026646546

PEX11G	-0.195341527	3.584504801	3.782547384	0.051789758	0.183584762
ELAVL2	0.241180938	3.584968474	2.132282602	0.144225843	0.352897583
SLC25A21	0.064245905	3.585085039	0.302374522	0.582397624	0.77051357
RAD51D	0.009852273	3.585903758	0.007901271	0.929170052	0.96854765
PPARGC1B	0.183792634	3.58722749	1.09457675	0.295459369	0.532228922
ZFAT	0.060971751	3.588580813	0.234236443	0.628400208	0.800445901
IGSF11	0.268770253	3.589287985	3.277776361	0.070224013	0.223724325
LOC107049895	-0.069105415	3.589576736	0.375976322	0.539764546	0.740769442
TM2D1	0.001000117	3.589661425	9.56E-05	0.992197934	0.996459368
FOXRED2	0.213972601	3.589736538	4.783552782	0.028732782	0.126044495
LOC107052478	0.23828724	3.589935594	3.974386516	0.046197279	0.170404892
NAPEPLD	-0.074026338	3.590055271	0.485093676	0.486124105	0.700617701
FAM65B	-0.340794194	3.59016122	3.52531575	0.060438312	0.203418374
ATXN7L2	0.3235021	3.590180455	7.026129127	0.00803288	0.053336266
ZDHHC12	-0.225024056	3.591388952	3.833574241	0.050235699	0.180329064
CLDN3	0.057501619	3.591394387	0.112096422	0.737769897	0.866307619
P2RX4	0.398013634	3.591489798	17.57098986	2.77E-05	0.000733397
MIR6625	-0.068053475	3.592673465	0.426231583	0.51384387	0.720949939
RAMP2	0.006245426	3.593380419	0.001623094	0.967863805	0.986817198
SLC22A15L	0.104977352	3.593544952	0.635172347	0.425464386	0.653827091
MTCP1	-0.174993591	3.593638571	2.57444852	0.108601944	0.293970224
ZNF226L	-0.527893444	3.594875413	16.20469929	5.69E-05	0.001330746
PUS3	0.116858614	3.595974883	1.535650668	0.215266357	0.444471699
KIAA0895L	0.003022217	3.596742811	0.00080139	0.977415829	0.990525896
ASTE1	0.028516264	3.597611503	0.074135622	0.78540783	0.894435194
LOC107051305	0.463824525	3.598594556	11.83074037	0.000582608	0.008108258
PARP8	-0.212549673	3.598970297	2.148622467	0.142697871	0.350357126
BDH2	-0.203822123	3.599219978	2.065046349	0.150710347	0.362140589
SCO2	0.688119367	3.599857718	31.09834624	2.45E-08	1.94E-06
FAM134C	-0.03127698	3.600346152	0.101675904	0.749827678	0.873444126
MMAA	0.232474084	3.600507066	3.916644102	0.047810229	0.174245254
LOC107054288	-0.308400046	3.600921788	3.73843588	0.053174199	0.187121781

AMPD3	0.110099909	3.601817826	0.564567641	0.452425703	0.67549848
TNFRSF1B	-0.045902206	3.601986217	0.193562274	0.659968159	0.819543887
LRRC31	0.087970527	3.602022595	0.503948106	0.477770476	0.694090848
SUSD1	0.404058457	3.602225283	6.422771562	0.011266619	0.067538285
MSRA	-0.109432627	3.605303211	1.428444084	0.232018533	0.463558154
KLB	-0.421569664	3.605421328	13.52622598	0.000235253	0.004087732
RGS9BP	0.181194262	3.606145955	2.876972278	0.089854828	0.261599646
LOC112532276	0.22378494	3.607761231	3.550554145	0.059525568	0.201127971
HSF2BP	0.088406373	3.60857967	0.32271601	0.569979976	0.762434642
LOC112531154	-1.011522367	3.609762128	31.39330522	2.11E-08	1.72E-06
AKIP1	0.295706804	3.610023515	5.332253468	0.020934301	0.102248123
LOC107054036	0.399524548	3.610076122	11.32775904	0.00076357	0.009851805
CLN3	0.242857125	3.610985141	3.365343756	0.066581687	0.216472633
PEA15L1	0.049762311	3.611300103	0.116485981	0.732877427	0.863242524
LOC416959	0.224831532	3.611604957	2.536666728	0.111229379	0.298821132
PENK	0.185347046	3.613154204	0.868084784	0.351486067	0.587969672
LOC112532969	0.040895555	3.613558137	0.006829639	0.934136499	0.971324872
CCDC191	0.039152999	3.61393594	0.129891637	0.71854442	0.854609585
KCNB1	-0.117674855	3.614769848	0.925914652	0.335926755	0.57321078
GDAP1	-0.102172817	3.614960113	0.67186708	0.412401414	0.642802432
PGGHG	-0.430043308	3.615324246	16.59738129	4.62E-05	0.001120129
LRFN3	0.101073721	3.616279099	0.90932097	0.34029465	0.577392087
SNAP91	-0.723150494	3.616290328	19.03412559	1.28E-05	0.000394746
RBM48	-0.001096618	3.618337389	8.70E-05	0.992556117	0.996459368
MKS1	0.185612592	3.618550263	3.031488199	0.081663083	0.245391896
EVA1A	0.288970475	3.621134639	6.310564727	0.01200206	0.070508356
LOC107052490	0.010101722	3.621370455	0.008905031	0.924818048	0.966337375
BOK	-0.130868327	3.621711695	1.385044275	0.239243789	0.47181432
PRKAA2	-0.312452494	3.621970295	7.504297448	0.006155195	0.044306092
LOC107049082	0.27944884	3.623466913	3.713182851	0.053984356	0.189018673
PCBD2	0.084640187	3.624826921	0.605382031	0.436531874	0.662740596
SLC10A4	-0.28111428	3.625117924	1.568367337	0.210444274	0.438234642

NDP	0.328189858	3.625419847	4.377158592	0.036423668	0.1469787
LOC112532616	0.01862614	3.625597428	0.037827876	0.845789175	0.927768873
NAPB	-0.300586518	3.625877631	4.577027157	0.032403385	0.136297982
VILL	-0.049343277	3.627356096	0.080112648	0.777144812	0.889536847
CHIC1	-0.029557618	3.627886246	0.091311704	0.762516172	0.880814575
CA10	-0.441880501	3.62820558	7.817954512	0.005172971	0.039082295
XRCC4	0.130796278	3.628501161	1.039157709	0.308017448	0.545990208
GRHL2	-0.025476136	3.629131916	0.044507887	0.83291152	0.921010621
IL18	0.695904321	3.630244621	44.26564909	2.87E-11	4.97E-09
TMPRSS5	-0.020346588	3.630832905	0.039793711	0.841884463	0.925494032
C19orf24	-0.00943469	3.631360918	0.007424735	0.931333739	0.969881443
RWDD2B	-0.281684937	3.632770123	6.543904995	0.010524408	0.064235586
LOC770996	0.154300283	3.63522623	0.792646295	0.373301142	0.607944407
GHR	0.034971623	3.63562315	0.110743404	0.739299363	0.867380435
LOC101749299	0.097039434	3.636890945	0.513247807	0.473736275	0.691749969
PIGBOS1	0.158079855	3.637003348	1.38900646	0.238572956	0.471215677
RPAIN	-0.250156181	3.637177056	5.965685132	0.014586932	0.080708796
LOC101751399	-0.189728724	3.637714834	1.962759539	0.161218498	0.376932422
UNC13A	-0.45601842	3.63806066	3.746888654	0.052905905	0.186416847
NFKBIA	-0.200610206	3.640373788	2.939446826	0.086440331	0.254776398
LOC107051747	-0.220800901	3.640593216	0.48735053	0.485111559	0.699830301
KCNK2	0.06832779	3.640858571	0.371952011	0.541942173	0.742391406
C22H8ORF4	0.050599759	3.641368492	0.101638785	0.749871821	0.873444126
AHI1	0.23719761	3.642116787	4.54458252	0.033023074	0.138198038
LGI2	-0.170893895	3.642474539	2.046386523	0.152567925	0.364686985
ACTR10L	-0.868909783	3.645260992	24.51346171	7.38E-07	3.73E-05
SAMD13	-0.155891911	3.646687258	2.366452584	0.123968473	0.319333316
TRAF5	-0.04001645	3.647207875	0.21684356	0.641455564	0.808362337
LOC101749287	-0.071527736	3.647557158	0.300870499	0.58333721	0.770936649
ENTPD2	0.359748922	3.648733048	8.16501027	0.004270623	0.034026847
ANXA7	-0.271997143	3.648814746	5.838920674	0.01567544	0.084681339
FBXO32	-0.197147315	3.649314682	1.977805638	0.159621769	0.374636459

HSPBAP1	0.033126926	3.649821576	0.116553279	0.732803225	0.863216914
B3GAT1	-0.12520007	3.650402268	0.773526414	0.379128334	0.613681567
LOC107049379	4.842649835	3.652007919	52.31399626	4.73E-13	1.28E-10
LOC112532687	-0.139750129	3.65330472	1.286747538	0.256648204	0.491406353
AK5	0.221659434	3.653471178	2.710819027	0.099669808	0.277460106
MATN2	0.369707994	3.654439913	10.27089334	0.001351453	0.01487938
NINJ1	-0.157174574	3.654455238	2.15577843	0.142034444	0.349301565
GPR153	-0.045719048	3.654710808	0.117660733	0.731585594	0.862408819
CCBE1	0.017133643	3.655742992	0.010217637	0.919485108	0.962786027
IL11RA	0.163114077	3.657701595	1.367365922	0.242264971	0.475074917
METTL15	0.065879013	3.657760302	0.465060448	0.495268414	0.707178212
LOC100858809	0.241423248	3.657939683	4.073396924	0.043563584	0.164639132
ULK4	0.841114933	3.658948471	43.54491258	4.14E-11	6.82E-09
ANO3	0.069338961	3.659488293	0.444572266	0.504923833	0.715326876
RITA1	-0.161646593	3.659698177	1.659093386	0.197725778	0.423467175
L3HYPDH	0.322124775	3.660399804	7.10816177	0.007673376	0.051684567
NMNAT2	0.223242697	3.660420459	1.67291738	0.195868231	0.420708925
MAMDC2	0.000697638	3.660592	2.04E-05	0.996400489	0.998524363
FRMPD4	-0.264642058	3.661069056	7.723249133	0.00545142	0.040498021
CSPG4	-0.281124246	3.661671915	3.648124367	0.056132392	0.193476856
RSPH10B	0.503356373	3.663844481	17.03584146	3.67E-05	0.000930021
UNC80	-0.480209008	3.664288043	9.024429738	0.00266395	0.024258606
RPS6KL1	0.111650729	3.664908452	1.186527713	0.276031078	0.511145116
PRICKLE2	-0.055100248	3.665500235	0.23589641	0.627185782	0.799902499
AZIN2	-0.373119686	3.667874678	9.342653467	0.002238811	0.021721486
SYTL2	0.148615266	3.668161592	1.188774579	0.275576878	0.510712639
IGSF21	0.217923691	3.66944827	3.211582583	0.073118729	0.228956932
ITGA11	0.10239686	3.671109587	0.491376371	0.483313979	0.698061617
F3	-0.126716295	3.67116968	0.947747986	0.330293191	0.568036629
IFITM3	0.375960801	3.672761842	6.341856455	0.01179214	0.06964812
LOC431092	-0.220647598	3.672944056	3.791488645	0.051513814	0.183079874
LOC107050985	0.041588174	3.673039934	0.169345098	0.680694497	0.8327134

ZBTB20	-0.016816274	3.67423134	0.01261516	0.910571882	0.95894146
HERC4L	-0.142243627	3.674276753	2.256783792	0.133030088	0.33501838
C22H2ORF42	0.174376693	3.674395773	2.070148494	0.150206896	0.361300171
ADGRL4	0.005792816	3.674692004	0.00256043	0.959643714	0.982756866
ADGRB1	-0.294401998	3.675521784	4.028743562	0.044731243	0.166867109
LOC112532971	-0.138595324	3.675883674	0.597042843	0.439708545	0.66486251
SYT16	-0.242395705	3.676168214	5.426030281	0.019838727	0.098662471
ATF3	0.360391898	3.678498469	4.339739547	0.037232619	0.148812907
RAB36	0.094535936	3.679395344	0.926905015	0.335668449	0.572942255
AADAT	-0.261519891	3.680360294	5.485057963	0.019179684	0.096543806
ENDOD1	-0.232898254	3.681014907	4.247696964	0.039303596	0.154537797
ARSK	0.005966786	3.681212819	0.002190212	0.96267287	0.983917412
VSTM2B	0.179624595	3.681273683	2.141586551	0.143353568	0.351547386
NOD1	-0.022829659	3.682834276	0.039594755	0.842275019	0.925580035
CASP1	0.16433191	3.684266781	2.443480802	0.118014092	0.310260726
LOC107055446	0.511757678	3.688264016	2.673550433	0.102028187	0.281974105
IRAK4	-0.290841574	3.688915619	8.877813645	0.00288657	0.025771652
CPNE8	-0.067547315	3.689032134	0.286018204	0.592783531	0.777327412
MEGF10	-0.052109926	3.689887928	0.099567423	0.752349362	0.874596167
LOC112530321	-0.12987382	3.692735587	0.30126767	0.583088795	0.770877221
GDPD1	0.04876052	3.69328461	0.239834405	0.624325742	0.798234661
DUSP10	0.216873974	3.694267903	4.515668758	0.033585754	0.139664791
FBXL8	-0.291699243	3.694805028	7.650970615	0.005674178	0.041738313
DCLRE1C	-0.119119517	3.695963074	1.467527087	0.225736269	0.455653866
LOC429800	-0.058236602	3.696407066	0.196248419	0.657766209	0.817611646
PDIK1L	-0.141038447	3.698773578	0.875704435	0.349380905	0.586080157
CA13	0.494504969	3.699309887	3.436578073	0.063767638	0.210829111
FAM78A	0.27832916	3.700233541	4.804038704	0.028393103	0.124922062
SPIK5	-0.410988185	3.701046181	3.81747444	0.050720636	0.181515455
ANAPC13	0.019903296	3.701092344	0.031158811	0.85988655	0.934387716
LYRM7	0.349695893	3.702708206	8.356110909	0.003843924	0.031547016
CZH9orf72	0.044354445	3.702796258	0.128577493	0.719911526	0.855564362

ABCB11	0.025189854	3.703663464	0.04365028	0.834505621	0.921720365
PRDM1	-0.339846461	3.703936955	1.6379731	0.200603794	0.426973927
LOC107051255	-0.05862546	3.705287313	0.248979048	0.61779488	0.793398483
CCDC84	0.044687106	3.705779778	0.133525729	0.71480409	0.852264423
ATG9B	-0.115151194	3.707070401	1.076645759	0.299449136	0.53669922
LOC424727	-0.142734242	3.707266572	1.426234724	0.232379918	0.463789928
LOC112530167	-0.054292013	3.707399854	0.321253524	0.570855295	0.763014502
C2orf88	-0.270930705	3.707446234	3.241977448	0.071773961	0.226408374
ENPP4	-0.117687393	3.707868142	1.243352854	0.264825854	0.499966663
TRIM69	0.090295605	3.70789946	0.699001796	0.403119309	0.634950534
CNGA3	-1.171805834	3.70799069	24.69414933	6.72E-07	3.47E-05
LRRC4C	0.417665665	3.708775899	8.740552402	0.003112102	0.027095049
LIG4	0.055386261	3.709045798	0.353176408	0.552320527	0.750840072
LOC107051301	-0.018184312	3.709158951	0.020701035	0.885596381	0.948729717
RAB20	0.084799188	3.709726564	0.492652478	0.482746475	0.697663204
PPP1R16B	-0.0649185	3.709790641	0.374937389	0.540325192	0.741229765
CINP	0.193919902	3.710055066	2.930491612	0.086921021	0.255545005
SLITRK6	-0.203326835	3.71090727	0.76401243	0.382075753	0.616740878
R3HCC1L	0.266613248	3.711829738	5.065236415	0.024410376	0.113242947
SMAGP	0.200611353	3.712898835	3.912556321	0.047926644	0.174592192
C28H19ORF44	-0.082015023	3.713146201	0.358635484	0.549265031	0.748237869
NLGN3	0.11209018	3.714620882	0.565511939	0.452047884	0.675362987
RDH5	0.369847037	3.715022326	13.67096765	0.000217796	0.003837075
PARPBP	0.217314933	3.715485137	5.048834787	0.024642505	0.113870378
GADD45B	-0.169782045	3.715596872	1.511598826	0.218895282	0.448199473
RTN2	-0.044859817	3.716095589	0.149610583	0.698907777	0.843494384
LOC107055568	-0.322516728	3.716424705	0.513443854	0.473651826	0.691702838
MYOT	-0.055369233	3.717043784	0.043358539	0.835051624	0.921947852
MPZL3	-0.00731876	3.717498619	0.003276717	0.954351929	0.980523198
GPATCH3	0.114879917	3.717964559	0.729744162	0.392966128	0.626150638
SLC41A2	0.342251618	3.718092403	8.183631261	0.004227006	0.033787267
EFHD2	0.345287194	3.718769257	7.654645519	0.005662632	0.041672005

USP12	0.161215947	3.719084539	2.538542646	0.111097287	0.298563752
LOC112532892	0.110420052	3.719537392	0.841297022	0.359026085	0.595343031
HS3ST3B1	0.273790963	3.720353942	3.735816754	0.053257623	0.18719099
CCDC34	0.300881658	3.722252047	7.869857033	0.005026563	0.038292634
ZBTB25	-0.017009156	3.722323872	0.017649812	0.894309959	0.951827123
USF3	-0.17279909	3.723642346	0.699157882	0.403066803	0.634928608
NDAHNAKL	-0.462606881	3.724168868	5.367626429	0.020513874	0.100823118
SERTM1	-0.028557648	3.724916741	0.021031521	0.884693121	0.948564886
TEN1	-0.339399075	3.725329843	5.634805148	0.017607302	0.091483473
LOC101747896	-0.41613726	3.726364693	4.749815751	0.029301432	0.127352102
REL	0.013137632	3.726513206	0.017504407	0.89474367	0.951827123
NRG4	0.280955416	3.727096967	8.928993882	0.002806789	0.025183913
PROSER2	0.106691682	3.728602931	0.737919593	0.390328123	0.623951561
ARHGAP45	-0.145155963	3.729098597	1.924062145	0.165409543	0.382814912
CCNJL	-0.044864031	3.729750997	0.191059669	0.662036124	0.820749221
XPO1p	-0.259258002	3.729811793	7.395193575	0.006539839	0.046185856
ZNF764L	-0.587310732	3.730403006	12.49487892	0.000408069	0.006217386
SLC26A9	0.117633865	3.730572803	0.592102322	0.441607314	0.666359316
PPP1R14C	0.108643965	3.731231085	0.737697503	0.39039945	0.623994458
PIK3CB	-0.094731476	3.732069546	0.944750142	0.331059213	0.568878379
TESC	-0.573616924	3.733676025	17.51152772	2.86E-05	0.00075065
EME2	-0.111228613	3.733838087	1.009729214	0.31496772	0.552546231
CRADD	0.022666603	3.733913858	0.05996079	0.806557925	0.906551274
CACNA1E	-0.212403001	3.733963175	1.857021672	0.172968936	0.392309282
PAMR1	-0.052714934	3.734006454	0.111422697	0.738530193	0.866862708
CDK2AP2	0.129807133	3.734098567	1.696815805	0.192704927	0.41714129
EYA4	0.184545751	3.734444167	1.842182943	0.174695308	0.394160332
CNTNAP1	0.207139351	3.734477559	2.332951297	0.126661533	0.32408887
ABCC13	-0.228664864	3.734539651	5.68001179	0.017159252	0.090137367
SLC5A3	0.271394361	3.73563745	6.382989766	0.011521918	0.068707547
LOC100858796	-0.033440431	3.736447068	0.066135531	0.79704886	0.900997038
LOC107055491	-1.336506489	3.739131822	3.234340363	0.072109337	0.226962345

EGF	-0.121167355	3.739305896	0.407009547	0.523491257	0.727968278
RASA4	0.15730013	3.740494748	2.335358984	0.126465833	0.323839914
NR4A3	-0.05322996	3.742998622	0.183579814	0.668314685	0.824242356
SOX18	0.157560765	3.744505138	2.442494704	0.118088288	0.310350803
ISL2	-0.54999688	3.745874318	5.957959743	0.014650986	0.08076448
LOC107054413	-0.474442164	3.749555727	12.06331192	0.000514239	0.007403145
CDPF1	0.091025525	3.750180594	0.72410476	0.394800765	0.627589842
MPEG1	-0.275252271	3.750866244	4.456401079	0.034770682	0.142503503
TIGAR	0.052393202	3.750903511	0.299671112	0.584088678	0.771126537
TYW5	-0.229593116	3.751238142	6.415033934	0.011315817	0.067783677
TJP3	0.033828377	3.752199398	0.083284591	0.772894615	0.88744546
EN1	-1.069937264	3.753682426	6.032782342	0.014042581	0.078675749
LOC107054531	-0.223012006	3.753735671	2.486615372	0.114818408	0.304404099
FAM46B	0.078611408	3.754174604	0.626102116	0.428788698	0.656275148
C10H15ORF58	0.119312002	3.754504327	1.406131998	0.235699566	0.467900217
HR	0.34478801	3.754706621	11.21320716	0.000812172	0.010335882
IQCH	0.023033293	3.754821353	0.044056033	0.833749398	0.921335057
AHR1B	0.1835496	3.756366909	2.551583042	0.110183805	0.296836036
LOC112530996	-0.256670851	3.756439602	4.767071747	0.029009126	0.126650351
CADM2	-0.32063576	3.757141574	7.543436657	0.006022906	0.043467944
C23H1ORF109	-0.073834933	3.757312467	0.520707714	0.470539948	0.689405693
SCRT2	-0.524045275	3.757585558	6.094236454	0.013562352	0.076843124
DAPK2	-0.189890528	3.757958845	3.77662178	0.051973494	0.184117082
KLHDC1	-0.048893485	3.758346739	0.131094418	0.717299997	0.853728044
NXPE3	-0.114628502	3.760582221	1.420366528	0.233343084	0.464849933
LOC101748000	-0.096317449	3.761058795	1.093166465	0.295770686	0.532600858
CTBS	-0.426690995	3.761740613	15.01479917	0.000106671	0.002155577
LYRM2	-0.364438723	3.761931531	12.58335854	0.000389197	0.006002092
LEKR1	-0.054661129	3.762018238	0.26464681	0.606945556	0.786666377
NICN1	0.05749252	3.762099113	0.282700752	0.594936488	0.77845837
ST6GALNAC2	0.296566239	3.762107857	5.116505778	0.023699291	0.111229844
PLEKHA3	0.00933039	3.762172579	0.006419129	0.936142239	0.972120443

SLC6A4	0.696489271	3.762339608	13.26269664	0.00027074	0.004583313
ELOVL7	-0.181730989	3.762709523	1.655038124	0.1982746	0.423935622
NUDT2	-0.04230155	3.762984369	0.108598822	0.74174503	0.868980128
ANO10	0.056267667	3.764018603	0.358376264	0.549409403	0.74832644
LOC107054556	0.231250049	3.764186998	6.555407497	0.010456589	0.063987792
CZH5ORF51	0.01081779	3.764236213	0.009477139	0.922447975	0.964657225
TNFAIP2	-0.097525303	3.764362838	0.702078371	0.402086212	0.634366465
PHYHD1	-0.218639462	3.765044265	3.950517969	0.046856943	0.172297429
CALCRL	-0.059014997	3.765134767	0.333664957	0.563508956	0.757622344
SERPINB6L	-0.617067201	3.765518842	36.58617438	1.46E-09	1.59E-07
TSPAN18	-0.065623151	3.765743737	0.323931281	0.569254612	0.762061813
RNF43	0.067445154	3.766170542	0.294889654	0.587104029	0.77348253
MUS81	0.429814723	3.766909318	18.90876973	1.37E-05	0.000418172
LOC107053055	-0.050630519	3.767634826	0.158672768	0.690381418	0.838516846
RFLNB	-0.211368286	3.768054574	2.73570746	0.098128037	0.274842017
BCL6	0.082008082	3.769678017	0.208960251	0.647583572	0.81200851
EDN3	-0.136174796	3.770209994	1.502614525	0.220269512	0.449749948
UNC13B	0.035088123	3.771101167	0.064295808	0.799830726	0.902345328
KCNQ3	-0.259085936	3.771830345	2.956141508	0.085551873	0.252739878
FXN	0.04767314	3.771895135	0.142252159	0.706052248	0.847788218
ACOT2L	0.046035575	3.773129296	0.16426683	0.685258149	0.835563601
EEF2KMT	-0.119341067	3.774337767	1.843747375	0.174512366	0.394017699
EPHX1	0.207422792	3.77608338	4.96628798	0.02584606	0.117485334
PMEL	0.490385597	3.776970789	5.919149129	0.01497719	0.082031922
ECM1	-0.305028227	3.777021586	5.762659261	0.016370277	0.087487144
RHCE	-0.091169562	3.778102858	0.43552193	0.509291786	0.717892027
LHX3	-0.28611386	3.778485817	1.90812421	0.167171773	0.385176636
FGF12	0.003248167	3.778717623	0.000542272	0.981421558	0.991828264
LOC112531456	-0.762409835	3.778828427	26.61672628	2.48E-07	1.46E-05
RGS2	-0.046919646	3.77915645	0.09462997	0.758371616	0.878579652
MYLK3	-0.167268735	3.779804718	2.52807883	0.111836306	0.29962006
DOC2B	0.102288468	3.779922831	0.950259765	0.329653185	0.567297974

RP9	-0.15829774	3.782202368	2.935489291	0.086652404	0.254978044
UBXN6	0.033410497	3.783428063	0.090817355	0.763140618	0.881102924
ABCC4	0.079034017	3.783698964	0.301539467	0.582918919	0.770870614
CHRDL1	-0.165448824	3.785481746	0.502258771	0.478509315	0.694655901
FAM161B	0.133087906	3.786038916	1.070113953	0.300919708	0.538455175
LOC427545	0.177281901	3.786076213	2.526258708	0.111965405	0.299735177
VDAC1	0.448866674	3.786109216	10.81799117	0.001005185	0.01206472
GDF7	0.226173117	3.786239457	2.453552346	0.117259231	0.308917811
TMEM136-2	-0.175322189	3.787107715	3.190273498	0.074077617	0.230904942
SPHK1	0.001572156	3.787292866	0.000218537	0.988205298	0.994673243
DNAJB5	0.199751962	3.788340445	0.925432178	0.33605269	0.573268404
TUSC1	0.14663065	3.78861982	1.268745986	0.260002064	0.494605082
ITGB8	0.133352414	3.788851699	1.098060216	0.2946922	0.531647862
MECP2	-0.136758133	3.789371799	1.739960902	0.187143781	0.410102577
GPR85	0.041777898	3.789481325	0.130472672	0.717942459	0.854176517
MMRN1	-0.384571021	3.789826436	10.32672818	0.001311174	0.014597681
C8H1orf123	0.152666281	3.791586722	1.657979136	0.197876398	0.4235471
CDK5R1	-0.199813412	3.792959507	1.959256949	0.161592809	0.377378607
ENKD1	0.044409475	3.794137392	0.189773996	0.663104785	0.821207891
WDR41	0.26123646	3.795191754	3.675712447	0.055210653	0.191495608
ZNF385D	-0.069455729	3.795958334	0.56702506	0.451443506	0.674854969
SERAC1	0.32230443	3.797566561	5.885463196	0.015266381	0.082961791
SLC29A3	-0.160214723	3.79782278	1.776399945	0.182592015	0.40500901
RASGEF1A	0.025394234	3.798625912	0.045172774	0.831686652	0.920212739
FAM114A1	0.278951972	3.799037421	7.645571052	0.005691188	0.041755635
CALML4	0.273010633	3.799790836	7.295191124	0.006913941	0.047922876
TMEM106C	0.325729103	3.800794083	11.51181315	0.000691553	0.009191844
GADD45G	-0.05690001	3.800971751	0.12192552	0.726955419	0.859404514
HYLS1	0.373791796	3.801267221	12.74268172	0.000357405	0.005604466
RASGRP3	-0.182061027	3.80130966	3.257055106	0.071116738	0.225216689
TMEM129	0.046969453	3.801766668	0.167034536	0.68276087	0.833753626
POMGNT2	0.1666225	3.80228598	1.950555642	0.162526988	0.378860143

SYNJ2	0.054319295	3.802307149	0.225598569	0.634806805	0.804262718
ANKFN1	-0.085977027	3.803722643	0.251974015	0.61568853	0.792054659
SLC5A7	-0.861917074	3.804465538	8.326096085	0.003907955	0.031960935
FKBP11	0.287353086	3.804761341	9.002583119	0.002695983	0.024478493
GREM2	0.433903729	3.80494641	11.41088736	0.000730149	0.009542978
PPCDC	0.023580435	3.805134112	0.050992604	0.821344984	0.91616267
TMEM251	0.072079425	3.80519983	0.402925581	0.52558222	0.730135536
HECW1	0.032076106	3.80571694	0.066799001	0.796055754	0.900610289
BNIP1	0.039203536	3.806136865	0.149653529	0.698866678	0.843494384
SCG2	0.154481518	3.806860418	1.849794769	0.173807266	0.393451446
EXOSC3	0.152533986	3.807386706	2.682585138	0.101450917	0.281039536
DERL3	0.403237896	3.808141531	11.11914115	0.000854413	0.010699675
KDM8	0.081295626	3.808718302	0.715838824	0.397512252	0.629978244
EPDR1	-0.196294668	3.809607712	2.830310968	0.092500447	0.265821665
CSRP1	0.289316491	3.810462333	6.209303675	0.012708061	0.073556811
CDH13	-0.24462532	3.811754971	1.745161757	0.186486125	0.409314284
SLC2A9	0.184686894	3.813404264	2.656519086	0.103126195	0.284197211
CITED2	0.201803301	3.813669334	3.634427572	0.056596064	0.194626589
LOC107049129	0.100097509	3.814393455	0.453246296	0.500797358	0.71159905
TIMM10B	-0.151578613	3.814804808	2.033902066	0.153825226	0.366528275
LOC100857843	0.284694014	3.815617718	6.422768003	0.011266642	0.067538285
RNF157	0.205884043	3.815704309	3.710100274	0.054084142	0.189191019
LOC107049095	-0.128165333	3.815726966	0.420322699	0.516776068	0.722783697
RPH3AL	-0.012889383	3.816094932	0.012305793	0.911670684	0.959545932
NELL1	0.088178538	3.816392411	0.618575268	0.431577218	0.658653601
BG8	-1.129339451	3.817493716	66.97383338	2.75E-16	1.33E-13
MCCC2L	1.050269198	3.817760228	40.13540327	2.37E-10	3.21E-08
DENND2C	0.009144205	3.81811374	0.008049003	0.928512712	0.968392732
CYB5D2	-0.16618759	3.818301654	2.743800722	0.097632304	0.273991768
PLCH2	-0.405793767	3.818609629	5.339689847	0.020845181	0.101933867
TPRN	0.1196336	3.818961219	1.308059279	0.252746334	0.487280718
ST7	0.234613639	3.819414704	4.327040355	0.037511417	0.149527704

QRSL1	-0.021594469	3.819478087	0.048735031	0.825279217	0.917552618
KIN	0.096036805	3.819535782	1.079422421	0.2988268	0.535875654
GBX2	0.055325643	3.819719502	0.119835972	0.729212459	0.860959389
CTPS2	-0.295592617	3.822617268	10.6587257	0.001095528	0.012821423
ANKRD12	0.091699532	3.823257829	0.65137408	0.419621838	0.649223958
TRABD2B	0.098934264	3.824184003	0.509064029	0.475544312	0.692572543
OGDHL	0.138949949	3.824687263	0.951123229	0.329433554	0.567150986
ATXN1	-0.017562027	3.824740332	0.022235198	0.881463117	0.94696347
GALNT5	-0.299185555	3.825408703	8.421709475	0.00370767	0.030890995
MRPS18C	0.001289195	3.825542068	0.000172781	0.989512424	0.995074915
AAMDC	0.302228301	3.827163507	6.84519807	0.008887984	0.057085006
PIGN	0.012515015	3.827241545	0.0155391	0.900795952	0.954678837
SEMA3DL	-0.057931525	3.827347534	0.217931517	0.640620539	0.807749496
BBIP1	-0.063006884	3.827496806	0.343022332	0.558089848	0.753847353
NTNG1	-0.001080827	3.828246755	7.18E-05	0.993237654	0.996933365
PTS	-0.042379304	3.828438298	0.245518882	0.620248196	0.795058816
RGCC	0.188913719	3.829103343	1.82770802	0.176398503	0.396589338
UBE2J1	-0.069195913	3.830546468	0.44724177	0.503647713	0.7140723
CCDC120	-0.050713078	3.830936589	0.261202097	0.609295499	0.787523166
SQSTM1	0.020579595	3.831986987	0.041696696	0.838199017	0.923661189
LOC107054225	-0.242325519	3.833106006	5.184156525	0.022793734	0.108058452
TMEM80	-0.045050063	3.833934675	0.190810668	0.66224276	0.820794617
LOC101749495	-0.624535988	3.834828241	26.94757518	2.09E-07	1.27E-05
ONECUT2	0.193892703	3.835129863	1.535519671	0.215285927	0.444471699
RAB26	0.09564846	3.835410996	0.963402443	0.326331121	0.564170897
BLVRA	-0.247239461	3.835819068	2.373545658	0.123406478	0.318433997
STAT6	0.002402037	3.835823107	0.000591273	0.980600469	0.991750505
TTI2	-0.126363878	3.836213257	1.762844155	0.184270156	0.406903552
RDH14	0.081524132	3.836424086	0.579253764	0.44660497	0.670621395
PIGQ	-0.182088593	3.838545868	3.990227761	0.045764878	0.169531981
LOC107055082	0.080892627	3.83864877	0.516004903	0.47255087	0.691022409
RASGEF1C	-0.020156526	3.839027242	0.020760474	0.885433387	0.9486918

LOC107050883	0.077666904	3.841070011	0.476449336	0.490035039	0.70373877
CDYL2	-0.126157377	3.841084339	1.45987953	0.22694929	0.456757652
PITPNC1L	-0.000888762	3.841121227	6.66E-05	0.993488351	0.997124173
NDUFAF3	0.310816921	3.841438687	5.479645351	0.019239162	0.09670764
PIGC	0.431900976	3.842108614	14.98598298	0.000108313	0.002177888
ZFP2	-0.022527801	3.842308486	0.061469574	0.804188117	0.905309945
ACE	-0.323543739	3.843651577	5.155749398	0.023169535	0.109367384
LOC101747670	-0.077749485	3.843678642	0.551712626	0.457618759	0.680288796
KCNN3	-0.159901763	3.843977704	2.804546313	0.093997431	0.268624477
NME6	0.146485908	3.844481244	2.150795429	0.142496048	0.349966041
GSKIP	0.15669607	3.845436159	2.737131834	0.098040592	0.274842017
NHLRC1	0.093679341	3.846028608	0.802279415	0.370412735	0.604916787
RPTC15L	0.523959164	3.846312167	0.943361457	0.331414858	0.569251721
ZNF408	-0.617081827	3.846452372	34.54525819	4.16E-09	4.01E-07
GTPBP3	-0.012165818	3.84657388	0.016164584	0.89882958	0.953839851
CD55	0.354292354	3.846828271	6.189233391	0.012852985	0.074143318
PLCL1	-0.114910991	3.846994514	1.181166637	0.277118619	0.512300515
FAM49AL	-0.236897334	3.847171585	3.276566085	0.070275824	0.223846047
SLC9B2	-0.523005867	3.847694333	20.82570366	5.03E-06	0.000186009
CREBRF	-0.145024502	3.84792787	2.018801871	0.155361686	0.368580817
ADAMTSL3	-0.119689421	3.848057057	0.43342413	0.510313545	0.718341124
MTFP1	-0.65382276	3.848162265	26.58642405	2.52E-07	1.48E-05
CDKL2	-0.298658559	3.849484627	7.662964204	0.005636582	0.041536033
EBF2	-0.220278282	3.849590118	2.874333446	0.090002234	0.261806415
KLHL31	0.095611346	3.849704055	0.255165414	0.613461208	0.790609663
DCUN1D2	0.21138706	3.850750882	4.426286192	0.035389432	0.144106186
LOC100857745	0.099749502	3.851636979	0.208836513	0.647680866	0.812068626
LRRC75A	0.454733833	3.852407998	12.3809464	0.000433737	0.006511995
NFKBIZ	-0.308206953	3.852519733	8.990634896	0.002713667	0.024588872
SLC22A15	0.134204174	3.854792348	1.185023129	0.276335754	0.511542899
OSBPL7	-0.16226337	3.855332602	2.499023799	0.113916891	0.302842189
SYBU	0.018005322	3.856965017	0.0150193	0.902460874	0.955420212

LRRC49	0.130601991	3.857981549	1.480210583	0.223741577	0.453072564
SVIP	-0.141912745	3.859030975	2.416745454	0.120044101	0.313021397
NEIL3	0.054656206	3.85944459	0.222481648	0.637155498	0.805669606
ST8SIA1	0.519066927	3.860057739	16.72554873	4.32E-05	0.001060865
ELOVL4	-0.185798589	3.86021715	2.623426914	0.105296814	0.288309245
ANKRD13D	0.140265404	3.860309445	1.254780024	0.262641478	0.497784558
APCDD1L	-0.44623634	3.860546802	5.503224113	0.018981446	0.095739139
GSTZ1	0.050251142	3.861237784	0.239335692	0.624686325	0.798386013
ZBTB40	-0.103211451	3.861612571	1.405567406	0.235793625	0.467900217
ADPRM	-0.109538671	3.86166386	1.38502201	0.239247565	0.47181432
CAPN6	-0.129219435	3.861785628	1.154815102	0.282543444	0.518195761
KDF1	-0.116634117	3.862418059	0.989963528	0.319751289	0.557135546
SMAD4	0.371942981	3.862821112	4.623050574	0.031545088	0.133747599
TMEM246	-0.269961713	3.86294153	5.561472385	0.018359966	0.093729893
XRCC2	-0.272789971	3.863212247	6.694014615	0.009673712	0.060663465
ZDHHC21	0.139678138	3.864540726	1.534900372	0.215378474	0.444471699
FBXO25	-0.292767089	3.86465218	8.480163314	0.003590399	0.030142862
MYOF	-0.116110124	3.864747091	0.988511746	0.320106389	0.557509593
GPATCH2L	-0.135257923	3.864872312	1.118854359	0.290165274	0.527005473
TRIB1	-0.050077304	3.865113064	0.20975115	0.64696252	0.811526992
C3orf33	-0.280386115	3.865285298	5.261160868	0.021806469	0.10513491
TMEM35A	0.03069174	3.866860235	0.119974809	0.729061813	0.860843293
LOC107050564	0.155718408	3.867324719	1.293626999	0.255380615	0.490348661
TTLL7	-0.363416287	3.867731943	11.69677089	0.000626087	0.008542773
MSRB3	-0.377796362	3.867960384	9.321432825	0.002264888	0.021845683
ACCS	-0.206453067	3.868011871	4.478196515	0.034329929	0.141480755
ST18	-0.334843514	3.868658281	3.101360482	0.078226895	0.238646021
CNTNAP5	-0.056991786	3.869106307	0.222796686	0.636917197	0.805508606
CYP39A1	0.023587695	3.869352353	0.033702296	0.854341503	0.932178863
LRRC20	0.061682972	3.869401756	0.2401909	0.624068271	0.798029483
GRHL3	-0.085687197	3.869756509	0.841490373	0.358970871	0.595330143
PML	-0.252483737	3.870174138	5.081699352	0.02417966	0.112607009

LCA5	0.184316332	3.870964751	3.667963013	0.055467935	0.192071733
LOC419584	0.383753125	3.872162718	9.063189023	0.002608067	0.023948518
DNLZ	-0.170832647	3.872428158	2.468550088	0.11614502	0.30691606
CREM	0.178587629	3.872617096	3.090757685	0.078738137	0.23984377
TCF21	0.259673716	3.873235387	3.104892593	0.07805738	0.238432186
PRODH	-0.26349159	3.873373086	4.167987033	0.041194715	0.15927139
MARVELD2	0.092177434	3.873592185	0.565864177	0.451907079	0.675275243
ARHGEF28	0.188551129	3.874048956	1.751974194	0.185628741	0.408194699
PRKCQ	0.084888249	3.874390748	0.60213508	0.437764554	0.663725766
PIANP	0.046285849	3.874722811	0.16430359	0.685224821	0.835563601
CPQ	-0.432571797	3.874959825	8.308706051	0.003945549	0.032188403
FAM163A	0.271188341	3.875363989	7.150561629	0.007494052	0.050746757
FMN1	-0.073783387	3.875837859	0.359477951	0.548796311	0.747861797
KXD1	-0.009598573	3.876052004	0.010359668	0.918929352	0.962506842
MPP2	0.189808212	3.876056478	3.613837735	0.057300747	0.196475056
LOC107050884	-0.021601342	3.878486846	0.016943901	0.896432926	0.95275104
POLK	-0.081273769	3.879277662	0.480466659	0.488211015	0.702056988
GPC5	-0.516956318	3.880451476	9.959372191	0.001600326	0.017011219
KCNJ5	-0.15470115	3.880892024	1.166982039	0.280022219	0.515352378
MST1	-0.111221774	3.880972405	0.7465451	0.387572282	0.62171007
CACNB2	-0.377120987	3.881530697	7.037579695	0.007981682	0.053109009
TUFT1	-0.281899736	3.882050212	5.87805879	0.015330715	0.083146643
PLSCR1	-0.161217207	3.882574739	1.85538354	0.173158553	0.39249663
FCHSD1	-0.086086692	3.883274487	0.637693327	0.424547313	0.653238372
UTP23	0.063004416	3.883699176	0.349935735	0.554149511	0.752043729
PHLDA2	-0.225834081	3.88388235	1.985490522	0.158813185	0.373901051
LPP	0.033437852	3.883894135	0.018589837	0.891548905	0.951008202
SMCO4	0.358783943	3.884233984	11.82136264	0.00058555	0.00812094
CSF1R	-0.509373707	3.885989576	4.817234804	0.028176513	0.12456865
GTSF1	0.717247255	3.88662594	13.3408699	0.000259684	0.004432674
DCLRE1B	-0.108000471	3.887199612	1.056610303	0.303989571	0.541885862
IRF2	-0.08478209	3.888885441	0.981051533	0.321939337	0.559635727

SOX7	-0.283071185	3.888969533	4.775891149	0.028860905	0.12630484
C1H3ORF38	-0.030642887	3.889826576	0.088675994	0.765867151	0.882949903
MPPED2	-0.02881413	3.890045577	0.087753151	0.76705323	0.883945718
TMEM141	0.113949203	3.890087558	0.646751165	0.421276623	0.650673858
LOC100858962	-0.107870929	3.890560155	1.218198865	0.269714415	0.504564655
PPP2R2B	-0.175956498	3.892084229	3.394347754	0.065420232	0.214317939
RNPEPL1	0.194261324	3.892681155	3.67363283	0.055279572	0.191580742
STARD13	-0.206899385	3.893054334	2.76838531	0.096143101	0.271780574
CLIP4	-0.25722282	3.8931804	2.833217272	0.092333217	0.265645587
SEMA4G	0.112789787	3.893867672	1.304761735	0.253345266	0.48783004
ZBTB24	-0.191895032	3.894626295	3.458508868	0.062927039	0.208973648
CYP2U1	-0.072133984	3.895165466	0.53532883	0.464375084	0.685317669
FAM219B	0.028363123	3.895178711	0.090376833	0.763698636	0.881519512
CD200	0.051398387	3.895188252	0.184147906	0.667832565	0.824166458
STYX	-0.129217059	3.89568711	1.312593919	0.251925549	0.486724775
BORCS8	-0.265650352	3.897148787	6.79554034	0.009138586	0.058172315
HOXD1	-0.322224046	3.897886516	4.505666634	0.033782725	0.140023865
MBLAC1	-0.250345406	3.898366233	5.433263549	0.019756724	0.098454542
IQCD	-0.084659552	3.898711506	0.55772309	0.455179102	0.677886878
BAG2	0.050761986	3.900049114	0.264626769	0.606959173	0.786666377
AP1S3	0.067833121	3.901362735	0.230222673	0.631358769	0.802557168
UBE2W	-0.209334925	3.901844256	4.021079305	0.044934949	0.167440587
FRMD3	-0.054474055	3.902123821	0.181366905	0.670201166	0.825399311
LOC112530306	0.074051359	3.902157564	0.523972393	0.469152074	0.688269654
F5	0.04072001	3.90342473	0.093160357	0.760197217	0.879915233
FAR2	0.081360569	3.903445501	0.535330424	0.464374419	0.685317669
SELENBP1	-0.441428142	3.904360163	11.82814758	0.00058342	0.008108258
GUCY1A3	-0.080344066	3.905539555	0.524000862	0.46914	0.688269654
PTPDC1	0.163818596	3.905707924	3.552602368	0.05945214	0.20100369
IGF2	0.022235349	3.906919321	0.031895004	0.858258292	0.933836045
SLC30A10	0.151181446	3.907947018	0.425958465	0.513978761	0.721016241
NPAS3	-0.186768596	3.908354485	1.758795821	0.184774785	0.407359715

PITPNM3	-0.200576501	3.908778974	3.190667201	0.074059779	0.230893078
IGSF9B	0.086586768	3.909807128	0.255031174	0.613554542	0.790643951
TECPR1	-0.303817264	3.910377577	7.942709631	0.004828149	0.037368349
MGTNIPA2L	-0.016368066	3.91155269	0.031645453	0.858808038	0.933947352
PIGB	-0.158898893	3.913120413	2.099425162	0.147354528	0.35717587
FOPNL	0.046624075	3.913235156	0.263020369	0.608052673	0.787414637
NR2C2AP	0.263355074	3.914346538	3.867992043	0.049215368	0.177598932
STOML1	-0.047016264	3.914704385	0.212030138	0.645180835	0.81068921
MAP3K5	-0.025041995	3.915954173	0.048200534	0.826224596	0.917837275
SH3BGR	-0.099705712	3.916473958	0.544162968	0.46071256	0.682585354
ALG11	-0.197218425	3.916516665	2.804734961	0.093986375	0.268624477
RPP38	-0.086567212	3.91659744	0.664373714	0.415020091	0.645416842
NTS	-0.254370334	3.916768273	0.219955554	0.639073786	0.806858657
ENTHD2	0.044732463	3.916788434	0.229179702	0.632132735	0.803037455
CHRNA5	-0.177082186	3.916802317	2.752630828	0.097094549	0.27310954
KHDRBS2	-0.106610232	3.918099885	0.989804964	0.319790048	0.557135546
SCML4	-0.868111315	3.918286783	17.57251766	2.77E-05	0.000733397
STXBP6	-0.118141132	3.918837834	1.616041618	0.203644612	0.430439575
MRVLDC3	-0.248425938	3.918855626	6.515717165	0.010692519	0.06494847
GSTAL3	-0.115645701	3.920154776	1.377419488	0.240541192	0.473216236
C7H2ORF69	-0.137087841	3.920628216	1.565475457	0.210865306	0.438608578
LOC107049738	0.132424463	3.920761206	0.940312676	0.332197442	0.569703899
FAM160B2	-0.008802133	3.920937666	0.003029603	0.956105137	0.981041173
LOC112530162	-0.024327202	3.921047621	0.070258466	0.790960826	0.897294753
HESX1	-0.023361571	3.921990983	0.041688531	0.838214642	0.923661189
IRX5	-0.105776267	3.92238793	0.345193735	0.556846532	0.753115441
APLF	0.110580407	3.922513992	1.433074415	0.231263347	0.46277981
SPP1	0.016443595	3.922561316	0.009594784	0.92196964	0.964486558
YAE1D1	-0.272647936	3.922619546	9.346686682	0.00223389	0.021699324
ARRDC1	-0.459202634	3.923022348	15.70213884	7.41E-05	0.001646395
RCSD1	0.054561776	3.924061749	0.177228057	0.673766307	0.827869219
XKR5	-0.145603929	3.924451178	1.792402462	0.180633721	0.4025908

MNS1	0.021934389	3.925229271	0.061224025	0.804571675	0.905502962
GLTP	-0.085531322	3.925736824	1.01495932	0.313717664	0.551695247
CAPN2	-0.375672283	3.926642317	11.09455477	0.000865816	0.010801362
SRA1	0.055933	3.926811066	0.299314977	0.584312188	0.771337425
NT5DC3	0.053870146	3.928696979	0.334745089	0.562878276	0.757393249
TFPI	-0.141311225	3.928815605	2.02135578	0.155100604	0.368173752
LRRC32	0.248145333	3.929716954	1.516854112	0.218096181	0.447254818
SNTB2	0.08131158	3.930868482	0.446921655	0.503800448	0.714227309
HIGD1A	0.034474778	3.931316656	0.095271554	0.757579486	0.878070751
GPAT2	0.098765869	3.931893231	0.596041754	0.440092274	0.664987913
TOX	-0.041087114	3.932077577	0.159212933	0.689882186	0.83840108
LOC107052489	0.022926451	3.932236362	0.053434619	0.817191052	0.913634921
PPARA	-0.274380834	3.93236713	3.928671685	0.047469425	0.173440526
CCDC130	0.109920151	3.933831875	0.957120407	0.327913457	0.565599155
LOC107053905	-0.014946346	3.934951227	0.016592089	0.89750777	0.953133605
TBX22	0.490136274	3.935337969	3.089321646	0.078807655	0.239970995
GINM1	-0.20873582	3.936428174	5.95668646	0.014661571	0.080768714
PITX3	0.02806514	3.937096448	0.034498531	0.852650383	0.931394748
CASP9	-0.151845456	3.9375697	1.882299824	0.170072988	0.388944472
SERINC2	0.181523825	3.937943401	4.15690696	0.041465058	0.159753578
SLC26A11	-0.223005818	3.939400807	5.296609284	0.021366979	0.103837461
BRK1	0.339249966	3.939470309	11.3077986	0.000771822	0.009922137
ZFAND1	-0.114006182	3.939595461	1.428071299	0.232079462	0.463623593
SMAD9	-0.322836861	3.939968458	4.166368574	0.041234088	0.159386169
CARD9	-0.127875616	3.940048106	1.072236691	0.300440776	0.537832134
SH3BGRL2	0.153591414	3.940105326	1.75836825	0.184828176	0.407359715
TMEFF2	0.277549047	3.940166195	8.384341985	0.003784672	0.031263443
PCSK6	0.207385778	3.94116316	4.549427992	0.032929746	0.137877599
PARP9	0.269769227	3.941736026	8.844223042	0.00294018	0.026053133
RGS7BP	0.245456557	3.942571841	3.457411766	0.062968809	0.20902799
EIF4E3	0.042397954	3.943302229	0.252247732	0.615496809	0.792054429
AJAP1	0.243313238	3.943928078	3.489797014	0.061748156	0.206601444

LOC112533143	0.178706774	3.944342161	4.076285551	0.043489162	0.164546302
ARHGEF4	-0.172775071	3.945632951	1.947298325	0.16287828	0.379345755
TESK2	-0.358311716	3.947323626	9.091130653	0.00256852	0.023691145
HTRA1	0.241914725	3.947774743	1.87275071	0.17116037	0.389828743
HABP4	0.126545396	3.948330926	1.224225234	0.268533039	0.503585025
ERMARD	-0.0624769	3.949377339	0.382037846	0.536514629	0.737836448
RFX2	0.219920856	3.94966308	4.913959046	0.026640512	0.120167111
TCN2	0.20746767	3.950228525	4.034450797	0.044580183	0.166705242
KAZN	-0.100547603	3.950700658	1.364067351	0.242833816	0.475864052
PSTK	0.386298436	3.953513864	10.28025635	0.001344613	0.014859369
SFXN3	0.276773119	3.956085346	5.224201247	0.022274674	0.106611334
ADGRV1	-0.261640494	3.956259284	1.671945925	0.195998096	0.420708925
TRMT12	-0.195852233	3.956266881	2.428363286	0.119157253	0.311623109
LOC112530984	0.085666159	3.956814568	0.695826196	0.404189717	0.635646128
C9H3orf58	-0.109617145	3.957294395	0.973633497	0.323775664	0.561579526
ICA1	-0.237804121	3.957865408	5.16447558	0.023053417	0.108944277
PRKRIP1	0.016671106	3.958531122	0.035059148	0.851471771	0.930961329
CYBA	-0.027674697	3.959620335	0.056493915	0.812125817	0.909908068
OGFOD2	0.039847427	3.959987248	0.206157791	0.649795702	0.813173579
UBE3D	0.111471157	3.960146649	1.085152764	0.297547701	0.534630641
EHHADH	-0.54563633	3.962085429	20.7642681	5.19E-06	0.000191213
MGAT5B	-0.047307662	3.962336483	0.111081252	0.738916488	0.867189987
HNF1B	0.235399767	3.962492241	1.638683742	0.20050616	0.42687655
PURG	0.001574482	3.963751466	0.000200903	0.988691149	0.994817358
LOC101748987	0.335872724	3.9638946	9.272010329	0.002326822	0.02222162
CENPM	0.269643586	3.964089141	5.65798565	0.017376068	0.090855801
LOC112531450	0.215473993	3.964822539	2.139474728	0.143551036	0.351926743
MYBPC1	-0.724104834	3.966299276	5.364177953	0.020554474	0.100962349
EHBP1L1	0.261484299	3.966931598	7.363093924	0.006657614	0.046716858
FBXL15	-0.115771972	3.966947708	1.684423896	0.194337656	0.418892604
LOC112530990	-0.270583475	3.96706369	3.252303704	0.071323149	0.225696618
LOC415780	0.091895438	3.967286484	0.730332496	0.392775435	0.626089294

LOC112530283	-0.009648527	3.967917145	0.008207133	0.927815814	0.967994498
POU3F4	0.000775918	3.968233573	2.64E-05	0.995901454	0.998206641
LOC416147	-0.165703143	3.969034694	3.246531351	0.071574776	0.226197475
NLN	0.035819034	3.96922454	0.103345877	0.747850806	0.872702382
GSDMA	0.090697502	3.970924886	0.831087211	0.361958339	0.598234679
SERGEF	-0.010102086	3.97140366	0.007523845	0.930878101	0.969780408
LOC107053589	-0.178886908	3.97153145	1.814028104	0.178025775	0.398874371
CHDH	-0.179160301	3.971894477	3.623220463	0.056978477	0.195696271
KLHL17	0.019385984	3.972096468	0.02403042	0.876807535	0.944232199
FAM84A	-0.131086432	3.972435047	0.893591829	0.344505809	0.581324676
PTPRVP	0.060684206	3.973572794	0.285328982	0.593229496	0.777327412
ZFYVE27	0.017674955	3.974311683	0.03210277	0.857802287	0.933726046
TMED3	0.022177748	3.974438216	0.05192799	0.819741756	0.915305055
TBC1D32	0.118745748	3.974509541	1.425247417	0.232541631	0.463983575
ADAMTS5	-0.044071765	3.974630009	0.097162198	0.755262057	0.876495029
SLC39A8	0.678603092	3.974780612	13.63475615	0.000222037	0.003895111
SYNPO2L	0.297076567	3.97699569	1.32124861	0.250368087	0.484683162
LOC112533543	1.203438933	3.977047001	46.62441186	8.60E-12	1.68E-09
LOC107057170	0.103556995	3.977539129	0.861313917	0.353371262	0.590030781
SGSH	-0.123357929	3.97872201	1.172558635	0.278876133	0.514237354
TSNARE1	0.025696206	3.978791845	0.056449254	0.812198706	0.909908068
WNT5B	0.164101418	3.979651011	2.334804096	0.126510905	0.323854534
TOR4A	-0.161858958	3.979680964	2.722511104	0.098942248	0.276135463
PDE6G	0.385606109	3.980574735	13.8863303	0.000194206	0.003487035
PI15	-0.102277929	3.981902266	0.255885598	0.612961	0.790261455
DNAH10	-0.508116781	3.982012004	6.517834104	0.010679799	0.0649332
TTC39B	0.065492157	3.982192344	0.322815376	0.569920599	0.762434642
LOC770165	0.334701457	3.982296263	12.2135786	0.000474429	0.006964082
LOC112530040	-0.254701625	3.982511703	4.706678465	0.030045649	0.129461695
PNKP	-0.040232034	3.9834138	0.216808453	0.641482552	0.808362337
CBX8	-0.199643278	3.983552937	3.212814386	0.073063707	0.22887175
PIGW	-0.238257234	3.983619972	4.92498189	0.02647108	0.119566735

MID2	0.099414385	3.984620651	0.432079382	0.510970387	0.718764262
KLHDC3	-0.124352051	3.984892118	1.411463911	0.234813528	0.466874891
PLD3	0.138761596	3.98573247	1.429458859	0.231852773	0.4635017
WNT6	0.058822516	3.986068574	0.125507905	0.723135838	0.857066913
KCNQ2	0.086922826	3.986255537	0.272424827	0.601709796	0.783255652
VSTM2L	-0.069550524	3.986758439	0.247689458	0.618706721	0.794077342
ZRANB3	0.121767434	3.986806501	1.382716325	0.239639	0.472246676
CMTM7	-0.178657964	3.988129945	3.81591057	0.050768004	0.181527053
SNRNP48	-0.052005383	3.988691005	0.289019318	0.590849661	0.776386577
TINAGL1	0.233062076	3.988785094	2.82502706	0.092805328	0.266209853
CNTLN	0.143342653	3.989116209	1.208603815	0.271608809	0.506496255
N4BP2L1	0.483157323	3.989427632	12.4273328	0.000423096	0.006381338
ZNF541	-0.033546798	3.989486516	0.089468434	0.764854022	0.882090898
SLC52A3	0.134667756	3.990369569	1.997435948	0.157565552	0.372092155
LAMP5	-0.164163017	3.990459399	1.191954432	0.274935679	0.509811425
IFI35	0.311311122	3.990787753	8.745967348	0.003102875	0.027043327
KIAA1841	0.261335754	3.991431926	5.962747826	0.014611252	0.080754108
COL6A2	-0.084719033	3.991581078	0.136923302	0.711359029	0.849891003
UNCX	-0.18992762	3.992684609	1.998320485	0.157473612	0.372055685
ZMAT4	-0.235819752	3.99300518	3.692629192	0.054653401	0.190292365
SETD9	-0.090786531	3.993268278	0.315661482	0.574226676	0.765040788
SOCS5	-0.048776423	3.993402997	0.330096796	0.565602097	0.759381732
KCNH2	0.144279637	3.993792202	0.872285465	0.350323371	0.586844251
PRSS12	-0.224375518	3.993926175	2.641970017	0.104074418	0.286043852
ORMDL1	0.291682814	3.994001977	5.734586185	0.016633987	0.088436916
PIK3CG	-0.131637615	3.994183411	1.870468634	0.171421416	0.390089808
ZNF414	0.335257601	3.995040119	8.900648306	0.002850694	0.025528804
TOR3A	-0.317198506	3.995361325	11.38300127	0.000741193	0.009664283
LOC107055114	0.018632312	3.997249139	0.029495072	0.86364092	0.936673566
SMIM4	-0.286405293	3.997656963	6.974489683	0.00826798	0.054507054
MET	-0.147701183	3.998401898	1.931493904	0.164595097	0.381681556
SLITRK4	0.082960775	3.998624689	0.260919114	0.609489413	0.78760321

LOC107054394	-0.165847932	3.998993292	2.274890157	0.13148444	0.33244875
SELENOO	-0.053518296	3.99902528	0.170264956	0.67987644	0.832132271
ACER2	-0.29919755	3.999276971	6.867970867	0.008775424	0.056627293
VAV3	0.068069051	3.999520244	0.374708978	0.540448594	0.741275453
ZNF71L	-0.105678182	4.00024333	0.837258649	0.360181977	0.596797345
RAD51C	-0.203985828	4.000373313	3.984311236	0.045925873	0.169860698
TEF	-0.066736974	4.000625277	0.373639344	0.541027166	0.741636289
PPIP5K1	0.075424375	4.000637186	0.696159599	0.404077141	0.635646128
PHOSPHO2	0.029088323	4.00081842	0.093126004	0.76024008	0.879915233
LOC107050955	0.021864645	4.000961657	0.037630443	0.846187097	0.927957926
EFEMP1	0.228365237	4.001311178	2.661039833	0.102833489	0.283723182
ASTL	0.739439817	4.001948823	11.99072247	0.000534661	0.007623778
COX17	0.42146191	4.002325217	16.70557349	4.37E-05	0.001069521
DGKA	0.095516027	4.004143641	0.962710036	0.326505029	0.564254007
PLCG2	-0.358333857	4.005044073	6.086238989	0.013623879	0.077091104
MAPK10	-0.098389427	4.005695958	0.333930186	0.563353965	0.757599665
LOC424401	-0.127448715	4.006216484	1.200115143	0.273298666	0.507978036
ZNHIT6	0.189454854	4.006234396	4.64316378	0.031177475	0.132873572
DDIAS	-0.255212523	4.006445284	3.243854262	0.071691799	0.226300218
SRRD	-0.123639335	4.006524985	2.201282175	0.137895996	0.342554139
LOC771069	0.156723258	4.007361489	0.863853553	0.352662544	0.589214862
C1QTNF6	-0.250282521	4.007491319	2.476668164	0.115546793	0.30587556
NCALD	0.003646489	4.007589468	0.000923902	0.975751436	0.990018742
PRICKLE4	-0.326052541	4.010774462	12.74153969	0.000357623	0.005604466
IQCB1	0.020698744	4.011102572	0.047231091	0.827953398	0.918504892
ANGPT1	0.008935549	4.0111594	0.003515305	0.952721122	0.979802073
LOC101749253	0.028833874	4.011532461	0.041385537	0.838795549	0.923690792
PREPL	0.187928808	4.011656833	3.201839522	0.073555494	0.229721483
LOC101751097	-0.608316585	4.012733744	9.332218536	0.002251596	0.021781311
ZBTB42	-0.024585602	4.013533918	0.050481178	0.822228094	0.916392801
HTR1B	-0.230491703	4.013667038	3.685152515	0.05489895	0.190718017
XYLB	0.153271527	4.014196542	1.606862578	0.204933388	0.431777069

CD164L2	0.200593926	4.014717565	2.593294107	0.107316932	0.291739652
KCNJ8	-0.27378084	4.015042164	2.922335757	0.087361322	0.256233851
ZFYVE28	-0.003112829	4.015793568	0.001115563	0.973355572	0.98946209
C11H19orf12	-0.06351621	4.017215582	0.375700712	0.539913172	0.740788063
LOC107055489	-0.153196217	4.01784973	0.569885645	0.450304362	0.673614388
MRPS36	0.191300758	4.018865797	2.648906719	0.103621139	0.284988442
VWCE	0.056203722	4.02321511	0.184519853	0.667517383	0.824129542
TMEM260	0.234806305	4.024289637	4.161364476	0.041356076	0.159710841
SGMS1	-0.136456834	4.024724705	2.230024599	0.135351693	0.33839038
IFT22	0.382188333	4.024894716	16.8764752	3.99E-05	0.000993099
SOX1	0.37607771	4.025372499	7.588300894	0.005874829	0.042736656
IQCK	-0.153865825	4.025401446	3.51071886	0.060972994	0.20464218
PLEKHH3	-0.088430985	4.025478117	0.754149894	0.385165522	0.619731944
NDUFAF2	0.141410859	4.02628994	1.217633977	0.269825485	0.504595753
USP13	0.147997846	4.026766945	2.154506828	0.14215208	0.349381997
PDGFA	0.109667018	4.02708537	1.046941095	0.306212634	0.544433159
LYN	0.237766858	4.028279183	5.899841935	0.015142244	0.082614595
PRDM8	-0.010740838	4.028820181	0.004984208	0.943716967	0.975921243
LOC107051391	-0.250823768	4.030126591	6.943124589	0.008414201	0.055161622
KCNT2	-0.088721332	4.031404276	0.564406496	0.452490227	0.67549848
PLBD1	-0.092877293	4.032446977	0.607281221	0.435813319	0.6622318
DTWD2	0.063829243	4.03337555	0.387734918	0.533492435	0.735495698
HS1BP3	0.089727663	4.033855427	1.056439264	0.304028714	0.541896933
APLNR	0.171877314	4.035413401	1.537008133	0.215063689	0.44430295
PDLIM1	-0.359153989	4.036105791	10.76303695	0.001035473	0.012346894
LOC100858295	-0.373428328	4.036908775	8.261494206	0.004049478	0.032707643
HYI	-0.178503412	4.036926551	3.595063934	0.057951399	0.198004626
HPS1	0.262584862	4.037061669	4.361252886	0.036765256	0.147662264
ZNF507	-0.146217806	4.037101976	1.747235385	0.186224661	0.409013186
DISC1	-0.112637259	4.037243824	0.953320538	0.328875521	0.566486621
LAMB2	0.306075155	4.039929792	4.501033272	0.033874379	0.140156626
CCDC82	0.131136467	4.040587264	1.419629512	0.233464393	0.464979011

PRDM12	-0.150166742	4.043647649	0.371874865	0.541984075	0.742391406
PIGP	-0.554078343	4.044042027	14.98076838	0.000108612	0.002179534
TPM4	-0.129673077	4.044737934	0.965867336	0.32571302	0.563872462
ANOS1	-0.331635137	4.045738203	7.156309979	0.007470072	0.050605199
MRM2	0.001176372	4.045739001	0.000170826	0.989571918	0.995074915
CDC26	0.215265696	4.045846604	3.317156814	0.068560302	0.22042981
LAMC3	0.647137363	4.047050928	19.8771523	8.26E-06	0.000281593
PAX9	0.230508452	4.047438262	1.892763069	0.168890604	0.387384288
LOC107052740	-0.43377362	4.048090038	25.33198488	4.83E-07	2.60E-05
SLC23A2	-0.222565652	4.048255821	3.215278076	0.072953794	0.228832384
INTS5	0.102582656	4.04828745	0.708180548	0.400048488	0.632466584
CORO2B	-0.199130494	4.050411936	2.652772564	0.103369464	0.284724561
LZTS1	-0.027519246	4.051791991	0.063549431	0.800971403	0.903047923
RNF169	-0.33370703	4.052050125	8.653855902	0.003263699	0.028087957
MED8	0.085387535	4.052261385	0.791978643	0.373502497	0.608208517
HECTD2	0.348856034	4.052657956	5.141744445	0.023357167	0.10996915
GMPR	-0.109174762	4.05343978	0.824705597	0.363807942	0.599605338
LOC426155	0.313142396	4.053612879	4.884377379	0.027100807	0.121411322
HHEX	0.052809861	4.053893125	0.304941142	0.580801223	0.769750998
ZBTB14	0.12536545	4.053994951	1.738559669	0.18732143	0.410326694
CASP10	-0.097961729	4.055048224	1.02706376	0.310849305	0.548528184
BCL2L11	-0.061113951	4.055070627	0.311360316	0.576846639	0.766659514
KCNC2	-0.236378486	4.055271091	5.504422311	0.018968445	0.095739139
LMINA	-0.304337415	4.056681085	8.255881334	0.004062018	0.032781025
PCMTD1	0.047623362	4.05894744	0.217944545	0.640610555	0.807749496
CYGB	-0.10447608	4.059343226	0.635641464	0.425293507	0.653696874
TNFAIP8	0.098681382	4.059358358	1.177220245	0.277922626	0.51321028
ZDHHC2	-0.113898941	4.059493965	1.877688023	0.170597152	0.389309994
NEIL1	0.059809156	4.059534359	0.300551845	0.58353667	0.770936649
LOC101750502	0.806038908	4.062179064	21.55026497	3.45E-06	0.000134718
CFAP43	0.387673121	4.062218809	9.457646749	0.002102707	0.02085596
LHX4	-0.570574645	4.06251261	6.937205743	0.008442089	0.055256395

LHX2	-0.290246675	4.062654808	0.3444678	0.557261606	0.753161374
IDS	-0.007097189	4.063071528	0.005157096	0.942750787	0.975445625
LOC112531007	0.044026859	4.063501748	0.23920105	0.624783755	0.798386013
KIAA1958	-0.317317097	4.063619944	4.815516023	0.028204626	0.124609464
BRAF	-0.154234856	4.063732492	2.33874938	0.126190828	0.323387334
FAM181A	0.287227207	4.06426831	3.661886262	0.055670573	0.192449429
LOC107052751	0.059050285	4.064587842	0.372223237	0.541794901	0.742288112
BEND6	-0.224213491	4.065073176	3.652799909	0.055975037	0.193149094
PEX7	0.148714713	4.065478103	2.094098429	0.147868916	0.357979712
PDE1C	0.479025709	4.065998576	19.89311818	8.19E-06	0.000280161
C21H1orf50	0.158246008	4.066551764	2.972795671	0.084675406	0.251322836
LARP6	0.010814434	4.067684264	0.012745975	0.910111365	0.958886062
LOC112531158	-0.167781201	4.068024561	2.041921397	0.153016262	0.365440144
NEK8	0.005827481	4.06807597	0.002688786	0.958645424	0.982278445
CYP21A1	0.354351287	4.0691166	5.824860356	0.015801223	0.085248892
NSMCE2	-0.166190425	4.069150644	2.790254257	0.094839179	0.269696998
MFSD8	-0.17965171	4.069260269	3.816758961	0.050742301	0.181527053
LOC112530174	0.235434961	4.069880088	2.696961492	0.100539691	0.279117395
FAM46A	-0.067465957	4.070126359	0.435842501	0.509135959	0.717892027
ZFAND2A	-0.408289136	4.0704695	9.488271824	0.002067895	0.020660118
TRMT13	0.012165978	4.072196934	0.018489372	0.891840545	0.951008202
VASP	0.32404702	4.072524679	9.086172339	0.002575493	0.023702317
RANBP17	-0.627522213	4.073104083	10.03882393	0.00153275	0.016409503
FAM129A	-0.592492952	4.073340473	25.7121779	3.96E-07	2.21E-05
NTHL1	0.750967145	4.073365112	41.82734152	9.97E-11	1.51E-08
C16orf71	-0.145759477	4.0744471	2.063687406	0.150844761	0.362345843
ENTPD1	0.022250642	4.075034449	0.020782198	0.885373872	0.9486918
LOC416055	0.120255915	4.075349539	1.929376046	0.164826725	0.381757467
CAMKMT	0.067549393	4.076177278	0.534105164	0.464886058	0.685549012
AKAP6	-0.229840215	4.076966977	2.186127182	0.13925908	0.344807055
NR1D2	-0.138483802	4.077210743	1.840724553	0.174866049	0.394329291
KIT	-0.076826097	4.077861252	0.386823529	0.533973834	0.735891075

ZNF746	-0.032070645	4.078361103	0.066685567	0.796225172	0.900610289
ATP6V0E2	-0.007027024	4.079030419	0.004100257	0.948943753	0.978497898
LOC112530509	0.009840303	4.079964678	0.005908171	0.938731239	0.973518783
PDCD2	0.096085559	4.080023333	1.102391124	0.293741951	0.530848821
SLC27A1	0.177509439	4.081580836	3.260308163	0.070975787	0.224960367
LOC107049847	0.020406895	4.081619315	0.016819572	0.896811464	0.952793351
OSBPL3	-0.164749135	4.082659125	2.52295707	0.112200007	0.299960737
ELP6	-0.340599462	4.083654116	7.205191861	0.007269298	0.049551075
TUBD1	0.15205113	4.084058553	3.490417865	0.061725003	0.206565979
COA6	-0.001060326	4.084136844	0.000121037	0.991222105	0.995986157
PDKI2	0.06247434	4.084266774	0.391715084	0.531399256	0.733850867
RPUSD1	0.109274408	4.084537873	1.448684643	0.22873913	0.459465614
TRAPPC13	0.133130416	4.084584051	1.310537907	0.252297287	0.486934484
MAP2K6	0.107040275	4.084641767	0.849153974	0.356791785	0.593318715
ADAM12	0.103262824	4.0851189	0.624095398	0.429529469	0.656929772
CHST12	-0.329511114	4.086484224	10.69602788	0.001073658	0.012631907
RNF103	-0.113486172	4.087255889	1.797049905	0.180069557	0.401714283
PLCL2	0.077979362	4.087344938	0.808828386	0.368466866	0.603219096
DEAF1	0.191725233	4.087482075	3.908396584	0.048045416	0.174792686
C15orf61	-0.227582112	4.087579645	2.750290022	0.097236789	0.273369446
DMRT2	0.340960533	4.088036658	2.797696575	0.094399836	0.269024818
MAPK15	0.05999141	4.088493358	0.462577065	0.496422035	0.707975783
MRPL55	0.318937865	4.089814131	7.4792564	0.006241384	0.044750317
TLL2	-0.018702988	4.090162113	0.029093553	0.864563208	0.937426699
LOC100857467	0.166206328	4.090275373	2.583625113	0.107974127	0.292800637
EDNRB2	-0.353227108	4.090896472	1.87342618	0.17108319	0.389828743
INVS	0.101848936	4.091432341	1.100381678	0.294182357	0.531116725
CAMK2B	0.126744481	4.092622536	1.05250979	0.304929767	0.542946366
NUDT15	0.011727637	4.092711121	0.012516603	0.910920439	0.959124381
SLC46A1	0.017295216	4.092729504	0.028822861	0.865188686	0.937672385
TNNI2	-0.313837104	4.093665249	6.049314242	0.013911699	0.078259853
CRTAC1	0.300235834	4.093758681	6.180279658	0.012918186	0.074298758

HOXC4	0.016966061	4.094294824	0.016232095	0.898619671	0.953678599
STK17B	-0.177228499	4.094338437	2.324622855	0.127341081	0.325220779
NGEF	0.218499912	4.094665058	4.762447169	0.029087164	0.126765425
LOC107053315	0.158745041	4.094971293	2.723940271	0.098853714	0.275981992
LOC100859161	0.168813097	4.095669898	3.055004045	0.080488795	0.243240242
PIK3R1	0.139706527	4.095685442	1.556049864	0.212244535	0.440192062
MYO16	-0.264274352	4.095885537	4.003092656	0.045416857	0.168724131
CPLX1	0.901834637	4.096252374	23.05198668	1.58E-06	6.92E-05
RPS27L	0.124328246	4.096377289	1.609434323	0.204571337	0.431327134
LOC112530953	0.240986174	4.096559265	6.624994693	0.010055744	0.062205741
xcc-b100_1894L	-0.120468385	4.096678697	1.22819154	0.267759029	0.503053957
PCDH17	-0.212802906	4.097566516	2.094746232	0.147806251	0.357971694
GALNT6	0.301613952	4.098248447	9.199003213	0.002421469	0.022786542
ATCAY	0.19741398	4.09890698	2.50427396	0.1135378	0.302406036
PDCD6	0.008667612	4.099589754	0.009560449	0.922108936	0.964486558
GYG2	0.09316135	4.100068278	0.293422388	0.588035684	0.774214049
FBXL18	-0.204123157	4.100586446	4.17842007	0.040941851	0.158779656
SLC25A29	-0.220355727	4.100653161	6.600712242	0.010193798	0.062823577
GAA	0.525566592	4.101081783	12.99323605	0.000312618	0.005098248
GAB3	-0.273395686	4.101382261	5.859172191	0.01549608	0.083905233
SLC35F2	-0.042414004	4.101982147	0.182414965	0.66930602	0.824790725
KAT2B	-0.190354538	4.102175967	4.587673396	0.032202699	0.135766182
CDC14A	-0.216186731	4.102203892	4.070910274	0.043627758	0.164805959
TMEM218	0.116872598	4.102282328	1.328777069	0.249022895	0.483272997
TMEM53	0.03676391	4.102610637	0.141570392	0.706724795	0.848162533
FAM69B	-0.003711998	4.102660691	0.001753297	0.966600425	0.986147777
AKT3	0.197394519	4.103220957	2.563599879	0.109349332	0.295264729
DCAF17	-0.174733383	4.103334079	2.127864688	0.144642128	0.353434606
TDRD7	0.361799912	4.103489722	10.86841373	0.000978183	0.011852723
EFCAB7	0.280184823	4.103515017	5.618577274	0.017771069	0.091922981
PRADC1	-0.06061661	4.103695491	0.496256981	0.481149405	0.696704901
NQO2	-0.436790293	4.103762517	20.31347833	6.57E-06	0.000231332

ZNF536	-0.202225923	4.103943013	1.6253174	0.202351943	0.429146995
CNTNAP2	-0.131627865	4.10407124	0.995420199	0.318421229	0.55616403
IL6R	0.031163935	4.104844732	0.076011269	0.78277741	0.892997939
PAGR1	0.268580739	4.106972713	6.023157232	0.014119366	0.078998356
AKAP7	-0.03284371	4.107316986	0.146153065	0.702239073	0.845693564
CSRNP3	0.239429732	4.107336683	7.929625787	0.004863185	0.037516979
OTUD1	0.048697268	4.1076898	0.212134338	0.645099651	0.81068921
RBPMS2	0.049121888	4.107907279	0.157758022	0.691229095	0.838989066
MCUB	0.077313595	4.10814542	0.638180349	0.424370488	0.65312259
OCLN	-0.445583623	4.108319839	23.77229716	1.08E-06	5.07E-05
GZMM	0.032510065	4.108753543	0.135679483	0.712614541	0.850454908
RAB28	0.170221502	4.108888101	4.178410196	0.040942089	0.158779656
B2M	0.152993079	4.108988443	2.375511071	0.123251256	0.318327342
WIF1	-0.303089177	4.109263089	2.222230612	0.136036389	0.33940912
ANXA11	0.196593121	4.109299894	2.471130715	0.115954482	0.306461773
THBD	0.000179706	4.111101274	2.55E-06	0.998725776	0.999310367
S100A10	0.013964993	4.111660645	0.015362458	0.901358519	0.954933316
HOOK2	0.07434282	4.112227564	0.717301593	0.397030464	0.629760583
SCN8A	-0.220567486	4.112336119	3.087022863	0.078919077	0.24012822
LCT	0.335522259	4.113817024	4.449214381	0.034917307	0.142748889
DEF8	0.050517819	4.114049772	0.257455471	0.611873694	0.789305554
AFG1L	-0.290961324	4.114194717	10.29783532	0.001331864	0.014776945
LOC107051795	0.216026088	4.114223042	3.769385634	0.052198802	0.184584319
MTIF3	-0.013303993	4.116357229	0.016841813	0.896743644	0.952793351
TLE2L	0.102614922	4.116927047	0.81919051	0.365416948	0.600872977
MASP1	-0.066585337	4.117287709	0.263543102	0.607696379	0.78724956
CP	0.163093602	4.117407974	2.727136241	0.098656043	0.27564743
NUDT8	0.173058266	4.117872588	2.714909947	0.099414581	0.277030809
TSR3	-0.030103332	4.119505313	0.098512734	0.753621764	0.875209692
RAB40C	-0.045212028	4.119961051	0.312361805	0.576234491	0.766093443
PTCD2	0.132556306	4.120381929	1.22157443	0.269051891	0.504027293
VCPIP1	-0.12274115	4.120841539	1.466318282	0.225927485	0.455872075

CLSTN2	0.145810222	4.122564467	1.051021757	0.305271884	0.543347632
LOC112530350	0.021413696	4.123462829	0.047161734	0.828077792	0.918504892
ME1	0.004192441	4.124178449	0.00161677	0.967926441	0.986817198
CGN	-0.360915495	4.124906672	9.347661799	0.002232701	0.021699324
GPX8	-0.088735888	4.125442501	0.49367852	0.482290973	0.697268439
MAN1C1	-0.105487257	4.125581384	1.617938743	0.20337944	0.430265966
DNAJC27	-0.319799882	4.125827593	11.12994445	0.000849451	0.010661878
NMNAT1	-0.209741261	4.12618285	6.138815691	0.013224586	0.075664311
AMACR	0.200769772	4.126595828	3.361846944	0.066723197	0.216810861
MEIKIN	-0.049583636	4.126779299	0.297359451	0.585542563	0.772343452
CELF2	-0.190851477	4.126795886	3.049405358	0.08076671	0.243766729
BLOC1S1	-0.136167609	4.126900216	2.147217127	0.142828568	0.350573402
RAB38	-0.179530788	4.127323488	1.008711796	0.315211651	0.552589063
LOC101751955	0.08109721	4.127588967	0.656069845	0.417950874	0.647709704
TFPI2	-0.248755462	4.128023066	4.815281575	0.028208463	0.124609464
TBC1D2	0.329769225	4.128456201	7.577279162	0.005910859	0.042922855
TMEM14A	-0.004201008	4.129201276	0.002323779	0.961552398	0.983793118
TMEM45A	0.180328082	4.130060675	1.524928336	0.21687521	0.446085197
SLC7A2	-0.420246612	4.130425802	14.73715262	0.000123587	0.002432561
SPRY2	-0.018369797	4.131685972	0.019910785	0.887786535	0.949358246
MTRF1L	-0.108479015	4.13173244	1.772842327	0.18303071	0.405516403
C4HCXorf57	-0.358644827	4.13176672	11.49560754	0.000697609	0.00924247
LOC112532614	0.608545915	4.131999626	15.04094061	0.000105204	0.002147351
CAMK1D	-0.067170085	4.133272339	0.382441109	0.536299683	0.737796005
RUBCN	-0.1458216	4.133566179	1.839666327	0.174990061	0.394554871
SLC41A3	0.141227206	4.13394044	2.018012357	0.155442498	0.368719375
SRD5A3	-0.111560141	4.134373143	1.242366195	0.265015517	0.500152579
ISM1	0.023750352	4.134709851	0.030430664	0.861516638	0.935232635
TUBB1	-0.16849176	4.135455441	1.28090994	0.257729916	0.492541889
D2HGDH	-0.132234262	4.136846874	2.268571754	0.132021521	0.333361256
SCARB2	0.015645971	4.136920898	0.035710054	0.850115514	0.930291912
LAMB4	-0.088661928	4.136930332	0.360005906	0.548502955	0.747648785

TATDN3	-0.421402771	4.137455017	19.04566893	1.28E-05	0.000394016
ZNF365	-0.118664886	4.138372882	1.146573794	0.284267525	0.520218204
MED30	0.247334312	4.138656869	6.648349682	0.009924771	0.061743707
PTPMT1	-0.025266375	4.138800176	0.092247226	0.761339479	0.880629912
HEY2	0.013065483	4.139118493	0.016803964	0.896859084	0.952793351
NME7	0.419597926	4.139358776	15.13341608	0.000100174	0.002065626
MANBA	-0.525547592	4.139418429	19.83602147	8.44E-06	0.000285686
FAM105A	-0.080284162	4.140027785	0.652595589	0.419186215	0.648951845
SYT1	-0.034853648	4.140593448	0.097040683	0.755410254	0.876495029
RAD1	0.310947803	4.142651059	4.946154376	0.026148771	0.118501248
SORCS2	-0.351385753	4.143200282	3.080485032	0.079236893	0.240833472
SOX6	0.460394435	4.143289504	5.100379767	0.023920606	0.111825554
FAM20C	-0.478154122	4.143334465	8.291402932	0.00398332	0.032368166
BMP6	0.3068688	4.144010226	4.15862004	0.041423139	0.159741682
FIG4	0.073108685	4.144375933	0.615266015	0.432811924	0.659478488
VPS37A	-0.274334427	4.144392797	9.375261064	0.002199334	0.021516075
ZKSCAN7L	-0.10999398	4.144433514	0.772249086	0.379522178	0.61405408
SCRN3	-0.137598264	4.145202014	1.02565045	0.311182446	0.548903174
FERMT1	-0.120737707	4.145725828	1.382974924	0.239595059	0.472216636
SOCS2	0.472234337	4.14593812	10.65962173	0.001094998	0.012821423
BCL2L10	0.044088215	4.14646067	0.212341179	0.644938568	0.81068921
VKORC1L1	0.083116082	4.146704536	0.793793251	0.372955589	0.607528899
FAM222A	0.070387884	4.146836594	0.61615066	0.432481332	0.659421823
GYPC	-0.163974805	4.147807327	2.524133907	0.112116324	0.299888832
DLK2	-0.03311741	4.148985181	0.125747922	0.722882132	0.857066913
LOC112532876	0.21132035	4.149307126	5.00084081	0.025335008	0.116092331
RABL2L	0.020257441	4.14969182	0.059491208	0.807301922	0.907015781
RAD51B	0.218851216	4.151317607	2.993560655	0.083596168	0.249154317
HOXC6	-0.140304837	4.151747071	1.337467945	0.247480995	0.481416217
LOC101748569	0.250332922	4.152301978	9.152190212	0.002484205	0.02313662
C1H11ORF54	-0.132911671	4.15243074	1.967685462	0.160693752	0.376111802
TECR	0.034698133	4.153208142	0.078440491	0.779422038	0.89083765

TYW3	-0.640820321	4.153887078	23.68614674	1.13E-06	5.27E-05
HIVEP2	0.003548841	4.155755572	0.001380663	0.970359604	0.988012332
TRERF1	-0.254577864	4.156967183	5.059047789	0.024497694	0.113488052
TIMM21	0.066856689	4.158137938	0.393770517	0.530324087	0.733379515
LRP3	-0.288050147	4.158397733	4.602751497	0.031920687	0.135021446
PHC3	-0.303875361	4.158895578	5.152847743	0.023208281	0.109487463
LCP1	-0.650394134	4.158913335	18.89884758	1.38E-05	0.00041924
ELL2	0.063970204	4.161530826	0.293081669	0.588252458	0.774437491
ZC3H12A	-0.152020509	4.161849233	2.757305014	0.096811199	0.272657543
ASIC2	-0.002214599	4.161976403	0.000420542	0.983638835	0.992657841
ELP4	0.099667194	4.163105306	1.196627788	0.273996718	0.508957412
WWOX	0.232322087	4.163129593	5.175499208	0.022907588	0.108535666
ACTG2	-0.234400718	4.164498695	0.932240294	0.334281462	0.572053485
MAGI2	-0.444855861	4.165273369	12.26223671	0.000462217	0.006827454
CDKN3	-0.100705663	4.165378302	0.459726381	0.497751877	0.709441935
CD320	-0.106404868	4.165848464	0.882819883	0.347430498	0.584085637
PIP5K1B	0.078559444	4.166332623	0.646501723	0.421366189	0.650673858
ZUFSP	0.140956143	4.167018506	2.55935542	0.109643279	0.295670298
FRY	-0.309538633	4.167989965	5.065839841	0.024401879	0.113235453
CCDC71L	-0.02286664	4.168272534	0.087203821	0.767762477	0.884179034
TMEM206	-0.138895752	4.168361609	2.039161181	0.153294158	0.365854976
ST8SIA5	-0.313166195	4.168548523	6.776468862	0.009236744	0.058593149
OSTM1	-0.186284903	4.16859875	3.983537655	0.045946968	0.169900529
ZIC4	-0.100022331	4.169224166	0.334423599	0.56306585	0.757521753
FRMD5	0.500516715	4.169716538	15.79369342	7.06E-05	0.001587898
C22orf39	-0.024886237	4.170079497	0.042601623	0.836477216	0.922733824
VRK2	-0.162512032	4.170258649	1.836491506	0.175362721	0.395038977
SYNPO2	-0.13858674	4.170590452	0.646143458	0.421494878	0.650750442
RALY	0.294556838	4.170966746	5.814821333	0.015891668	0.085680666
SLC25A46	0.033375277	4.171261409	0.105153824	0.745730372	0.871333755
NRBP2	0.069703483	4.172084085	0.394245675	0.530076095	0.733281956
CISD2	-0.020789093	4.172137235	0.060547985	0.805631915	0.905943632

SGCB	-0.114260699	4.172217724	1.909476725	0.167021396	0.384975078
NSUN3	0.079644434	4.172272399	0.314178679	0.57512723	0.765492399
IER5	-0.083743851	4.1725715	0.694559028	0.404618002	0.636032596
RRNAD1	0.011443708	4.173199044	0.016293824	0.898428125	0.953567235
PKIA	-0.084271519	4.173382675	0.747855001	0.387156201	0.621284893
MTHFS	-0.153169618	4.174031811	2.130821218	0.144363395	0.353116945
NAGA	0.259004332	4.175175873	5.276175529	0.021619185	0.104538259
RAB27A	0.089703156	4.175359209	1.034105557	0.309196337	0.54712396
PTGS1	-0.01069579	4.176179065	0.009575632	0.922047307	0.964486558
ACSS3	-0.358650555	4.176989284	9.151974989	0.002484498	0.02313662
FANCF	0.1853466	4.177826151	4.174920329	0.041026492	0.158919708
MAPK12	-0.124250736	4.1779529	1.969989666	0.160448959	0.375934447
TRMT10A	0.266930208	4.17802826	8.412537116	0.003726423	0.03098483
PROCA1	0.527671954	4.178089212	31.39498362	2.11E-08	1.72E-06
CACUL1	0.207393817	4.178318757	6.541737886	0.010537236	0.064266205
ROBO3	-0.15276544	4.178508647	0.460358741	0.497456363	0.709143589
SLC37A1	-0.106714593	4.178936766	0.818566478	0.365599628	0.600933161
PMP22	0.077274816	4.179309928	0.453193803	0.500822157	0.71159905
DLGAP3	-0.109904422	4.179453009	1.169205741	0.279564499	0.514855494
ELMOD3	0.217976475	4.179522027	4.510961486	0.033678304	0.139940597
MTUS1	-0.100083694	4.17971668	1.013968658	0.313953945	0.551828937
MTX3	0.016017323	4.179782185	0.021604392	0.883144393	0.947830745
PTGES3L	-0.283551211	4.179844911	9.840319322	0.001707279	0.017814375
C8orf59	-0.100239868	4.180554584	1.039372064	0.307967558	0.545966429
ACAD6L	-0.447689073	4.180590369	23.93107521	9.98E-07	4.79E-05
SH3RF3	0.106368508	4.180677044	0.748952985	0.386807925	0.621136991
DOK6	0.010400122	4.18083734	0.005871452	0.938921553	0.973646548
CCDC127	0.046526269	4.181173264	0.265075892	0.606654199	0.786666377
CEP55	-0.025545644	4.18154846	0.074329299	0.785134572	0.89437421
F2RL3	0.203789103	4.182015767	3.327032112	0.068149747	0.219452854
ADCY7	0.139144375	4.182339662	1.463546411	0.226366692	0.456254766
ATP5SL	0.10599043	4.182696935	1.210378881	0.271257098	0.506356841

FUCA2	-0.229796777	4.183151109	4.817659215	0.028169575	0.12456865
TMEM100	-0.065015969	4.183182543	0.27211	0.601919866	0.783442907
TTC13	-0.051939592	4.183485511	0.42403302	0.514931475	0.721798912
E2F2	0.023394732	4.183638855	0.055387895	0.813939939	0.911114401
PIBF1	0.016892049	4.184503686	0.037391517	0.846670099	0.928302005
HDAC11	-0.05790012	4.184824559	0.460106856	0.497574038	0.709249896
FAM83H	-0.256323764	4.184956458	7.759852533	0.005342032	0.039955973
NMRAL1	-0.337671996	4.185330255	5.911012763	0.015046519	0.082217488
HGH1	0.007887076	4.185471059	0.00441669	0.94701305	0.977487125
MARS2	0.018145659	4.185698449	0.039813302	0.841846059	0.925494032
SLC2A11L5	0.15394565	4.186012631	2.027134763	0.154511664	0.367433368
FGF10	-0.336668462	4.186593173	0.969959747	0.324690218	0.562753058
SHTN1	0.006803586	4.186905162	0.00440523	0.947081738	0.977496707
WRNIP1	-0.256934806	4.187064812	4.789980007	0.028625759	0.125652824
ANKRD40	-0.223270664	4.187270495	6.417647261	0.011299176	0.067708645
THRA	0.031047555	4.187844023	0.044422601	0.833069327	0.92106133
C12H3orf14	-0.188839186	4.18849551	3.777619901	0.051942497	0.184061327
LOC107053477	-1.644965883	4.18920815	22.47849225	2.13E-06	8.90E-05
C14H16orf59	-0.217708413	4.190074876	6.78710104	0.00918189	0.058380214
LOC107049815	-0.149534266	4.190801738	1.976121607	0.159799584	0.374946836
JPH1	0.296721212	4.190890187	9.891733781	0.001660229	0.017446238
UTS2B	-0.505706871	4.191083426	4.773537571	0.028900383	0.126376774
ACTN2	-0.5663484	4.191564914	8.027681723	0.004606777	0.036028759
OLFM2	0.14885758	4.192943957	1.807262172	0.178837007	0.399886256
EMP1	0.202600855	4.193191307	1.092719915	0.295869348	0.5327113
LRRC17	0.158170256	4.193835766	1.148151659	0.283936407	0.520055692
PGAP1	-0.071252706	4.19396087	0.181563498	0.670033024	0.825253998
NARFL	0.000634153	4.19475429	5.04E-05	0.994338385	0.997544036
EFHD1	-0.080823534	4.19499716	0.764927486	0.381790864	0.616522931
RNF182	0.127654267	4.195469113	1.717120101	0.190064174	0.413417842
PTPRJ	-0.02865632	4.197275712	0.069595859	0.79192612	0.897745396
RARRES1	0.396515443	4.197368822	7.269962809	0.007011726	0.048295072

COA3	-0.123743755	4.19765912	1.381714775	0.239809275	0.472323431
TRAPPC5	0.183461048	4.197728041	4.906451668	0.026756553	0.120459391
ADARB1	-0.23464672	4.197736153	6.120287886	0.013363903	0.076196477
INPP4B	0.086416261	4.198079717	0.756932198	0.384290292	0.61891913
LOC107051115	0.071944159	4.198768223	0.729272509	0.393119098	0.62615185
ADAT2	0.2160303	4.200528007	5.358398464	0.020622704	0.101086267
DPH3	0.00730874	4.200567761	0.004996532	0.943647544	0.975911026
ATXN10	0.287241815	4.200603881	12.62485158	0.000380652	0.005897957
LOC107055283	0.166415326	4.201074433	1.819739058	0.17734434	0.397900344
ZNF653	0.202475995	4.202322326	4.507716029	0.033742269	0.140023865
ALKBH8	-0.71577306	4.20258938	19.90518672	8.14E-06	0.000278978
ZNF624	0.376597043	4.202963217	10.52694736	0.001176464	0.013462247
WNK4	0.09815907	4.203409143	0.633549548	0.426056298	0.654293643
ZNF555	-0.056938215	4.203436733	0.436679764	0.508729361	0.717813551
SCRN2	0.203705914	4.205580919	4.44265761	0.035051644	0.143174449
AKAP5	0.023442396	4.205908279	0.04211664	0.837397594	0.923303231
HSDL1	0.022452959	4.206105266	0.057539719	0.810427641	0.909099927
PCDH7	-0.253474835	4.206110312	5.762029443	0.016376146	0.087490093
ATP10A	0.227505459	4.206192477	4.150427933	0.041624003	0.160186254
PLEKHF2	0.029875006	4.206252386	0.093025879	0.760365054	0.879947485
ZNF862L	-0.187836181	4.20671991	4.380820152	0.036345505	0.146800511
NEK3	-0.261291623	4.207204234	5.881934867	0.015297003	0.083045919
OLFM3	0.086848593	4.207264241	0.942447107	0.331649302	0.56937808
BCORL1	0.120986679	4.207387708	1.221979205	0.268972582	0.504022853
BET1	0.06858134	4.207558694	0.491731826	0.483155792	0.697948254
RAD9B	0.00451608	4.207731295	0.002918156	0.95691926	0.981347249
JMJD8	-0.032146642	4.208162006	0.14538539	0.702984837	0.846328321
INO80E	0.175697794	4.208502291	3.894600943	0.048441546	0.175612611
COMTD1	1.040376197	4.208734812	36.10496689	1.87E-09	1.93E-07
DLL4	-0.01506342	4.20937272	0.027032072	0.869404983	0.939840514
CITED4	0.157409762	4.210057793	0.919225921	0.337678302	0.574790159
B3GNT2	-0.069730452	4.210370802	0.339022213	0.560394164	0.755533467

SPTSSA	0.021929997	4.210577033	0.05920422	0.807758153	0.907466403
SATB1	0.313449955	4.210838036	8.455657787	0.003639096	0.030463772
AHDC1	-0.037690592	4.211095957	0.198868396	0.655635799	0.816258385
MYBL1	-0.429670528	4.211162605	13.20309262	0.000279488	0.004673747
STK11IP	-0.487581067	4.21296882	22.79017374	1.81E-06	7.75E-05
XKR4	-0.184617736	4.213116997	1.737749415	0.187424244	0.410497262
LOC101750883	0.257875946	4.213272715	2.78508662	0.095145549	0.270217085
CEP44	-0.233869676	4.213956663	5.96998759	0.014551383	0.080566289
CRIP1	-0.140456465	4.214660536	1.19144854	0.275037563	0.509942885
PHACTR3	0.09012398	4.214822724	0.523813126	0.469219628	0.688269654
ABHD6	-0.088515454	4.214848472	0.729769555	0.392957895	0.626150638
PFKM	-0.216938553	4.215091442	2.217471422	0.136456387	0.340107519
CCDC189	0.305395508	4.215156376	11.13781967	0.000845853	0.010632929
GPRIN1	0.050177521	4.215170554	0.247047203	0.61916195	0.79434819
ANKFN1L	0.261722287	4.215515337	6.658005893	0.009871132	0.061549629
CLIC2	0.066145538	4.216053579	0.391649099	0.531433837	0.733850867
ZBTB49	-0.101427601	4.216508584	1.429134301	0.231905773	0.4635017
MSX2	-1.046832952	4.217202159	9.617128824	0.001927709	0.019598353
TMEM5	0.0364403	4.217504837	0.178368946	0.672778706	0.827149851
ABCC6	0.26957359	4.218129603	7.740996179	0.005398101	0.040251089
HPRT1	-0.055385974	4.218325234	0.366834244	0.544734954	0.744733604
PAAF1	-0.063904857	4.218481757	0.505861333	0.476935963	0.693600685
NUPL2	0.197955181	4.218568693	3.907246033	0.048078322	0.174835091
ARRDC2	-0.084252796	4.21898565	0.570753595	0.449959613	0.673300375
EEPD1	0.050956713	4.21899153	0.238741547	0.625116517	0.798625177
MTG2	-0.032648782	4.21920206	0.137695003	0.710583329	0.849781439
ABCD2	-0.241909287	4.219381303	5.360839784	0.020593854	0.10100503
PRAG1	-0.016529889	4.219598418	0.045439174	0.831198528	0.919835926
UVRAG	-0.419069332	4.220234941	15.69009685	7.46E-05	0.001654677
C2H8ORF37	0.008371629	4.221452953	0.010540389	0.918227743	0.962372833
LOC112529955	0.001172494	4.221456313	5.15E-05	0.994276284	0.997544036
HEXDC	-0.110321211	4.221529848	2.068107358	0.150408075	0.361678338

TBC1D30		-0.324969283	4.221699579	6.551634518	0.010478785	0.06407596
AVEN		0.099092262	4.221882537	1.61297755	0.204073755	0.430927383
SSH3		-0.06301284	4.222339228	0.418418389	0.517727294	0.723314877
LOC418298		-0.017583977	4.222503366	0.037048229	0.847366887	0.928742508
KIF24		0.002703225	4.2237378	0.00056764	0.98099204	0.991828264
LOC107054271		-0.015384063	4.224187615	0.018760188	0.89105622	0.950952546
VEGFA		0.082086324	4.224417028	0.494747003	0.481817382	0.697173002
CELF3		-0.034054634	4.225776338	0.036899827	0.847669142	0.928840363
TMC7		-0.204219776	4.225840468	6.866203061	0.008784109	0.056638916
	01/mar	0.16042253	4.226725365	3.158259517	0.075543684	0.234055982
FBXL4		0.061711322	4.227293548	0.336903935	0.561621804	0.756197266
ENHO		-0.049536578	4.227443548	0.135909211	0.712382163	0.850434818
CCDC171		0.169301773	4.227912081	2.010833227	0.156179521	0.370094184
PIK3CD		-0.34219433	4.228211634	11.96854517	0.000541062	0.007701704
ALG1		-0.052247632	4.228616075	0.308074659	0.578864062	0.768285864
ABCB6		0.375488877	4.229384656	12.20211158	0.000477355	0.00699454
VEGFC		0.332144103	4.229641932	11.67180164	0.000634545	0.008615052
RAB3IP		0.131833156	4.230645703	1.651413522	0.198766652	0.424436188
TMEM147		-0.044911397	4.231544038	0.177859483	0.673219257	0.827473733
CABP2		0.140119086	4.231862336	2.49358266	0.114311245	0.303336805
GKAP1		0.044362401	4.231924881	0.138514167	0.709762622	0.849639454
GFER		-0.120904832	4.232373776	1.824034546	0.176833783	0.397085792
OSBPL6		-0.181084516	4.232676405	2.035906074	0.153622615	0.366250381
SMAD7		0.080150061	4.232677079	0.341846407	0.558765375	0.754264499
TPMT		0.196748484	4.232865012	4.618287737	0.0316328	0.134015893
LOC107052003		0.081271551	4.232985496	0.603861783	0.437108363	0.663219743
MUL1		0.122459634	4.233395798	1.926724835	0.16511721	0.382330488
FOXD2		0.190511267	4.233803098	2.05943487	0.151266261	0.362972935
RABGGTB		-0.156947518	4.233830594	3.335914932	0.067782699	0.218896507
GRIK4		0.113842546	4.233917758	1.601784237	0.205650542	0.432512738
SLC38A9		0.12067576	4.234207054	1.484708435	0.223039295	0.452356596
LOC112533407		-0.156226548	4.234283113	2.592436507	0.107375044	0.291801215

C3orf70	-0.045392593	4.23474563	0.240450108	0.623881212	0.797852284
EXD3	-0.01308013	4.235271124	0.02280166	0.879973991	0.946151989
POLR2K	0.229812885	4.23638493	5.052577401	0.024589335	0.11381102
LOC101750513	0.083429042	4.236964376	0.374762826	0.540419497	0.741275453
RC1IPL	0.060007378	4.237703263	0.430294183	0.511844632	0.719310225
SLC39A3	0.124599595	4.238055623	1.397919162	0.237072282	0.469437352
CCDC18	-0.324746102	4.238069951	7.093173152	0.007737812	0.051960495
ANKRD49	0.071063519	4.238100336	0.600841739	0.438257045	0.663886934
DNAJC21	0.143554304	4.238114955	2.02027308	0.155211226	0.3683832
TYSND1	0.013787279	4.239812655	0.019093759	0.890098028	0.950586601
WDFY2	-0.090549885	4.239986125	1.232878237	0.266848019	0.502343456
IL4R	0.002199534	4.240241628	0.000408884	0.983867178	0.992674867
AMY1A	-0.099459723	4.240622473	1.22583877	0.268217827	0.503309881
COBLL1	-0.322449771	4.241068452	9.846921134	0.001701162	0.017773097
TRMT	-0.200595565	4.241505701	4.943692223	0.026186042	0.118568272
ALG13	0.132551504	4.241817464	2.155195896	0.14208832	0.349381846
ADAMTS15	-0.553549899	4.242045372	3.425938617	0.06417975	0.211681257
ENTPD7	0.190854997	4.242237131	4.316711481	0.037739789	0.150147059
CETP	0.212422713	4.242890956	2.042906115	0.15291726	0.365312074
C2H1orf35	-0.021990227	4.2430676	0.048086527	0.826426953	0.917837275
MRS2	0.261559909	4.243199577	8.689707815	0.003200119	0.027631177
DPH2	-0.356573152	4.243247312	15.33058283	9.02E-05	0.001913608
F8A3	0.069201094	4.243932906	0.300885294	0.583327953	0.770936649
MIS12	0.24684044	4.244112369	7.60043831	0.005835411	0.042525108
CHRNA4	0.107427494	4.24425649	0.838953436	0.35969626	0.596167286
SLC24A4	-0.179023987	4.245217444	1.889708465	0.169234808	0.387675072
ZBTB8AL	-0.009024536	4.245762534	0.011684316	0.913921115	0.960591451
VTI1A	0.000959349	4.246126028	0.000120333	0.991247685	0.995986157
FBXL12	-0.103516707	4.246220967	1.460059292	0.226920687	0.456755952
SH3GLB2	-0.090493901	4.246996573	0.754948097	0.384914142	0.619681264
SLC10A7	0.153288703	4.247915293	3.103727344	0.078113259	0.238475551
DNER	-0.0195643	4.248773736	0.022120734	0.881766367	0.947093053

ARHGEF3	-0.269743014	4.249378865	6.312903723	0.011986237	0.070465715
KIAA1211	-0.100082718	4.249483852	1.06635732	0.301769693	0.539448158
CYP46A1	0.500518601	4.249698068	23.11713368	1.52E-06	6.74E-05
FLT1	-0.172711077	4.249883359	2.414360477	0.12022706	0.313275736
LOC424109	0.064108875	4.250084695	0.386914263	0.533925872	0.735886609
SLC35A3	-0.159367707	4.250124788	1.415508558	0.234144089	0.465994314
TBX18	-0.097279077	4.250170588	0.50649255	0.476661159	0.693600685
TRAPPC2L	0.000673215	4.251017566	5.88E-05	0.993879342	0.9973341
CBARP	-0.101616921	4.251078899	0.894874481	0.344159779	0.581074202
FGGY	0.003383461	4.251307343	0.000725806	0.978506965	0.990912186
BLOC1S6	-0.229083802	4.252700549	6.886460434	0.008685108	0.056242994
LYSMD3	-0.124058742	4.253161172	0.932598881	0.334188517	0.571985858
DAPP1	-0.052638897	4.253300901	0.382369135	0.536338035	0.737796005
FASTKD5	0.141142994	4.253520964	2.054260293	0.151780951	0.363587427
MYL3	-0.56616278	4.254026378	4.069321485	0.043668812	0.164885338
LOC112530570	0.086374745	4.254103485	0.521509563	0.470198455	0.689152541
MANBAL15	-0.399657365	4.25463164	10.94169354	0.000940242	0.011486028
FSD2	-0.088079142	4.255036243	0.904527604	0.341570588	0.578542874
COL23A1	0.214063164	4.256061912	2.682180503	0.101476695	0.281062152
HSPB8	0.221710172	4.25650267	1.847065362	0.174125096	0.393575338
ELP5	0.041137491	4.256969188	0.168233804	0.681686276	0.833245258
NRG1	0.199772833	4.258516018	2.285737746	0.130568044	0.330792481
EMC8	-0.034890405	4.258967464	0.152925996	0.695754709	0.841440815
FHOD1	-0.161276491	4.259244527	2.649924246	0.103554831	0.284879329
ZNF469	-0.317219315	4.260383271	5.103182472	0.023881988	0.111800316
METTL18	-0.100426673	4.260475183	0.91891923	0.337758907	0.574808442
PCASP3	-0.148776868	4.261029805	1.865378308	0.172005348	0.390986047
LOC107051736	0.053887195	4.261111142	0.18046835	0.670971057	0.826032043
ZSWIM7	0.124134046	4.261364074	2.107098491	0.146617082	0.355890852
LOC424919	0.228736928	4.262692561	5.68571115	0.017103606	0.089974374
ARV1	-0.028464933	4.263291514	0.09159723	0.762156344	0.880756463
LOC112532338	0.078024392	4.26518557	0.771907493	0.3796276	0.61405408

MBTPS2	-0.349516967	4.265482857	14.66424018	0.00012846	0.002513456
CLCN4	-0.649397587	4.26643612	21.78757501	3.05E-06	0.000121054
APH1AL	0.17129793	4.26650328	2.718166945	0.099211892	0.276606519
FAM104A	-0.047381621	4.266814809	0.252693572	0.615184804	0.791776766
SCLT1	0.110319351	4.267270596	1.443494064	0.229574761	0.460633178
THAP1	0.127149455	4.267806045	1.726728422	0.188829248	0.412476474
GABARAPL2	-0.01644764	4.268158066	0.037569403	0.846310341	0.928031232
ENGASE	-0.086652858	4.2686039	0.861307562	0.353373038	0.590030781
GPSM1	0.006313231	4.26860477	0.003485657	0.952920681	0.979802073
TCIRG1	0.131449103	4.268775499	1.946274135	0.162988915	0.379400564
FAM167A	-0.191825424	4.26879071	2.345832676	0.125618423	0.322171313
TMEM230	0.108763591	4.268800553	1.744606552	0.186556203	0.409358891
RAP1GAP1	-0.031405303	4.268940615	0.146178075	0.702214814	0.845693564
C17ORF80	-0.183915355	4.268955419	2.439341988	0.118325852	0.31056364
PGGT1B	0.159123822	4.269031185	2.65405725	0.103285976	0.28454223
GATAD1	-0.062412271	4.269198325	0.413172048	0.520363874	0.725779864
COX18	0.190463448	4.269640553	4.596138818	0.032044048	0.135305313
NOS2	0.036356546	4.269765336	0.166906446	0.68287591	0.833770356
EVPL	0.051917304	4.271449141	0.144675946	0.703676041	0.846564677
LOC107052086	0.050850232	4.271472318	0.31667046	0.573615481	0.764869254
S100A11	-0.191991253	4.272068688	1.208735766	0.271582645	0.506496255
CSF1	-0.027107176	4.27263138	0.075823776	0.78303877	0.893110346
CREBL2	-0.146272372	4.273069654	2.736182924	0.098098838	0.274842017
PDE4B	0.171494343	4.273182558	3.136390126	0.07656309	0.236147263
HARS2	-0.267252639	4.276824162	7.637745408	0.005715932	0.041883576
CREG2	-0.040369495	4.276907627	0.18897127	0.663774203	0.821665877
ISOC1	0.044893037	4.277357065	0.167554145	0.682294728	0.83346248
BRSK2	0.22618992	4.277405207	3.906162135	0.048109343	0.174870609
LOC112533474	-0.204763832	4.277608005	4.300287713	0.038105926	0.150874161
TGDS	0.04816276	4.280351275	0.280848445	0.596145654	0.779403841
BORCS6	0.058912052	4.280696542	0.232138715	0.629942518	0.801303429
TMOD2	-0.352017311	4.281284219	9.43950356	0.00212361	0.020924549

HSPA13		-0.138236468	4.28177398	2.263903202	0.132419933	0.333992948
STK36		-0.046305491	4.281813367	0.308657297	0.578505293	0.767986259
CRISPLD2		0.284151974	4.282398164	3.974728876	0.046187888	0.170404892
	10/set	0.104734257	4.282404084	1.246701473	0.264183414	0.499384748
SLC39A1		0.105309168	4.282625643	0.631941095	0.4266442	0.654768729
BTG1L		0.032110461	4.282803814	0.12529817	0.723357757	0.857066913
SNX21		0.130783632	4.283284075	1.366904149	0.242344507	0.475074917
PRKCE		-0.026771154	4.283404257	0.057863542	0.809905132	0.908699642
C9orf69		0.114928932	4.28341434	1.487168791	0.222656258	0.452195797
WDR7		-0.117591641	4.283436297	1.406946624	0.235563933	0.467801656
PMEPA1		-0.250613141	4.283714996	1.799622912	0.17975809	0.401454854
PPM1F		-0.109693389	4.283829484	0.73599347	0.390947343	0.624445596
	08/mar	-0.177452705	4.284113192	4.02500852	0.044830393	0.167123725
LOC101749275		0.207802719	4.284644482	4.041076201	0.044405495	0.166444744
OXNAD1		0.725607676	4.287020078	50.1071513	1.46E-12	3.52E-10
STXBP3		-0.048063839	4.287985557	0.317377381	0.573188022	0.764747751
CENPP		0.258519809	4.288432786	4.559596084	0.032734793	0.137340901
TNNT1		-0.233004301	4.289064453	2.103533941	0.146959134	0.356570325
CDCA4L		-0.216841794	4.289978532	4.421654851	0.035485606	0.144319239
LINGO1		0.292564968	4.292285229	5.611122067	0.017846831	0.092146097
TTC7B		-0.155964336	4.292981271	2.899472842	0.088608526	0.25880812
IGFBP5		-0.022723931	4.293048354	0.027960198	0.867202432	0.938655886
CREB5		0.307946374	4.293760072	9.857337271	0.001691558	0.017695219
PQLC2		-0.18346908	4.293875355	4.481876931	0.034256081	0.141309554
CXCR4		-0.129119213	4.294914996	0.987634017	0.32032133	0.557728522
STAM2		-0.087078532	4.295485825	0.908942073	0.340395274	0.577396397
HEXB		-0.143299007	4.295721242	1.115550288	0.290878613	0.527951421
RBM43		-0.195329782	4.296106693	4.292788594	0.038274341	0.151395258
AMER2		-0.153558642	4.296341066	2.071396121	0.150084076	0.361281572
WDR60		0.205919642	4.296838593	6.394838434	0.011445265	0.068326134
GALNT13		-0.377283121	4.297223753	9.775561561	0.001768467	0.018325015
HBP1		-0.031079633	4.297877614	0.102373184	0.749000095	0.873205161

GPRC5B	0.195867128	4.297985733	3.292315444	0.069604801	0.222440669
TRIM41	-0.078619748	4.298563163	0.892540654	0.344789742	0.581541124
BCL2	0.05674546	4.299438686	0.367489778	0.544375744	0.744408787
FRMD8	0.237657771	4.300019048	6.678207712	0.009759871	0.061040927
XIAP	-0.013317053	4.300386149	0.018425288	0.892026997	0.951008202
LIPT1	-0.140437853	4.301631684	2.935441939	0.086654945	0.254978044
NMI	0.253897307	4.301765411	5.56729398	0.018299019	0.093541585
LOC107049631	0.045709571	4.301960517	0.208516928	0.647932312	0.812187803
SUPT3H	-0.022591744	4.303014373	0.049202221	0.824457329	0.917226694
NMRK1	0.186788049	4.303765646	2.638431116	0.104306503	0.286538148
DUSP23	-0.04288921	4.303921327	0.147330588	0.701099508	0.844862132
LPCAT2	-0.179010953	4.305001383	2.92237556	0.087359167	0.256233851
UBIAD1	0.041906409	4.306720381	0.287654565	0.591727475	0.776938793
GOLGA5	0.00035223	4.306774528	1.30E-05	0.997126137	0.998764949
CMSS1	0.164471617	4.306946387	3.108188754	0.077899545	0.238215389
GALC	0.087240027	4.307390869	0.856654385	0.354676635	0.591325809
C9orf85	0.192945115	4.307404221	2.867443368	0.090388352	0.262188195
C1GALT1C1	-0.232521741	4.307788442	7.21741068	0.007219978	0.049286202
PCDH15	-0.472254333	4.307992113	8.288847901	0.003988929	0.032397741
LOC112530139	0.000971529	4.309237077	0.000126032	0.991042807	0.995986157
CENPU	0.203481512	4.309359489	5.53401206	0.018650292	0.094719308
ZDHHC1	-0.02090165	4.309902917	0.038842222	0.843761567	0.926328616
WDR34	-0.010732919	4.310240388	0.01903747	0.890259119	0.950652593
OPNVA	0.122403255	4.310515882	0.70925534	0.399691132	0.63209203
ADAMTS8	-0.505430712	4.31061096	11.94745372	0.000547221	0.007773013
RABGGTA	-0.044984978	4.311009688	0.256508006	0.612529416	0.790027554
SERTAD2	-0.099201394	4.311069903	0.802280095	0.370412532	0.604916787
PLK2	0.336588879	4.312869083	4.113792829	0.042534871	0.162368316
PRKCD	-0.009201801	4.312912984	0.010883753	0.91691126	0.96204647
USB1	0.062503074	4.313483274	0.348323697	0.555063582	0.752697304
UNC79	-0.414409667	4.313856324	7.79801752	0.005230359	0.039461511
DUSP12	-0.167838822	4.314983252	3.458778895	0.062916763	0.208973648

SPATA2	0.095129881	4.31511064	1.288581579	0.256309512	0.490986382
LOC107050393	-0.232315558	4.315846196	4.73392206	0.029573375	0.127945565
LRCH1	0.180767266	4.316514557	4.822671616	0.028087778	0.124309948
MVB12A	0.137150699	4.316780954	2.258127325	0.132914703	0.334779035
MAPK9	-0.062048845	4.316794715	0.418481754	0.517695593	0.723314877
C1H21ORF59	-0.07310931	4.317022929	0.454558805	0.500177963	0.710806493
LOC107054677	-0.042761428	4.317651237	0.225786167	0.634666079	0.804224916
CMTM8	0.058095121	4.318152135	0.275865019	0.599424327	0.781939467
SGSM1	0.107248307	4.318208917	0.955584565	0.328301856	0.565794621
SULT4A1	-0.19834039	4.318603434	5.381831422	0.020347507	0.10024498
RMI2	0.821789899	4.318981775	53.04168912	3.27E-13	9.60E-11
DNAJB9	-0.164504048	4.318996241	2.021944944	0.155040446	0.368084048
ASPA	0.266393258	4.31941887	4.529457203	0.033316183	0.13895956
FOXC1	0.360915437	4.320649839	8.344286872	0.00386902	0.031721337
PLEKHO2	-0.123139685	4.321199801	2.147980272	0.142757578	0.350451433
CZH5ORF30	0.224999578	4.321488695	3.582194493	0.058401952	0.198924473
CXorf40B	0.171033454	4.321489067	2.420632665	0.11974656	0.312585525
LOC107051491	-0.94112109	4.321518447	25.89164146	3.61E-07	2.06E-05
KCTD18	0.143777745	4.322326186	2.765561972	0.096312858	0.271980108
HMOX1	-0.267628464	4.322443212	5.76489957	0.016349419	0.08740406
GALT	0.163065445	4.323025801	2.010221408	0.156242515	0.370190148
ZBTB7A	-0.080642794	4.323074979	0.679750786	0.409672553	0.640064742
SLC26A5	-0.11515738	4.323398321	1.123900747	0.28908007	0.525556574
SYDE2	-0.019644237	4.32366222	0.053659554	0.816813505	0.913461073
TTLL11	-0.141771142	4.323743497	2.501468969	0.113740162	0.302651095
CLCN1	0.242246595	4.323837587	5.693279804	0.017029997	0.08975932
SYNJ2BP	-0.229348112	4.325476291	5.654099149	0.017414618	0.090912924
ACAD8	-0.064641351	4.325689622	0.686453212	0.407373401	0.637865574
ARSJ	-0.244102342	4.32591244	3.162069457	0.075367587	0.233726773
AKR1E2	0.017518195	4.327052177	0.020985266	0.884819102	0.94863815
RNF208	0.003826414	4.329369523	0.001818224	0.965988002	0.985879339
CKS2	0.239835132	4.329486794	3.650834714	0.056041118	0.193283715

FAM35A	0.12749467	4.330867555	2.247232214	0.133853626	0.336206832
SP2	-0.02706415	4.330969433	0.106692141	0.743941979	0.869965632
P2RX5	-0.067469245	4.331364583	0.284093951	0.594030365	0.777946576
LOC101751794	-0.218675456	4.331809909	6.220073468	0.012630987	0.073296899
MRPS24	0.334860624	4.333267797	11.55701869	0.00067494	0.009047928
BANP	0.192762775	4.333906735	3.349828644	0.06721201	0.21763368
UVSSA	-0.190661741	4.334236599	5.728326155	0.016693387	0.088638166
KCNH6	-0.012793064	4.335038925	0.018016697	0.893223637	0.951354873
WSCD1	0.153386697	4.33514032	1.872486691	0.171190547	0.389828743
SYT7	-0.048010599	4.335258143	0.138838403	0.709438538	0.849591042
CENPN	-0.042299019	4.335685581	0.226796113	0.633909706	0.80403489
C1H2ORF40	-0.32716001	4.335783527	6.205796408	0.012733265	0.073626795
TMEM107	0.108762171	4.337341967	1.152208118	0.28308739	0.518846402
CEP41	-0.030062432	4.338217153	0.112122572	0.737740439	0.866307619
NHSL2	0.080787907	4.338782693	0.823893932	0.364044126	0.599884603
GFRA1	0.030058212	4.339020736	0.024565472	0.875454679	0.943516292
MBNL2	0.012397219	4.339217703	0.01501728	0.9024674	0.955420212
LOC112533549	-0.056435562	4.339937256	0.338188117	0.560876947	0.755874706
DBNDD2	0.049542717	4.34020137	0.246864038	0.619291912	0.794431043
DUS4L	0.021466767	4.340253471	0.05372867	0.816697662	0.9133936
MRPS16	-0.045908914	4.34065517	0.274018623	0.600648697	0.782591357
SLC30A6	0.127287426	4.340836055	2.275982878	0.131391804	0.33226558
CDCA9	0.097860016	4.341185586	0.658534292	0.417077871	0.647149115
STK10	0.403614468	4.341855613	11.38746567	0.000739413	0.009648729
CSTB	-0.040218736	4.34200155	0.209766901	0.646950166	0.811526992
SURF1	0.139643805	4.343213173	2.867385712	0.090391591	0.262188195
WDR54	0.09879574	4.345215753	1.553764334	0.212580582	0.440666854
NSMCE4A	0.011253207	4.345806475	0.017513693	0.894715917	0.951827123
SV2B	0.027038694	4.345849968	0.05607861	0.812804793	0.910401121
DNAJC6	-0.363324142	4.346378636	5.912692381	0.01503218	0.082201308
DRAM2	0.241223359	4.347341283	9.149142427	0.002488347	0.023159357
FBLIM1	0.126497364	4.347729729	1.480904363	0.22363308	0.452981985

ZNF518A	-0.080287517	4.34810667	0.731267216	0.392472743	0.625849305
ATP8B4	-0.15290441	4.348170743	1.232059863	0.267006817	0.5024696
LOC100859072	0.163961998	4.349795766	0.0682506	0.793901142	0.898895155
KIAA1328	-0.025907712	4.350861866	0.075898197	0.782934988	0.893053877
SMIM11	-0.045966781	4.351034142	0.184987061	0.667122011	0.823888674
SLC4A4	-0.234616137	4.35115884	4.361698276	0.036755646	0.147662131
LMBRD1	0.253900697	4.351309928	6.763293102	0.009305188	0.058936442
CNR1	-0.217001576	4.35181016	3.756213719	0.052611586	0.18569791
HELZ2	-0.319354864	4.352572791	5.661553071	0.017340761	0.090756386
LZTS3	-0.301691433	4.35294886	5.611277068	0.017845253	0.092146097
CBX7	-0.122454265	4.354493599	1.805120498	0.179094682	0.400196905
IGFBP7	0.031476961	4.354787696	0.089724394	0.764527824	0.881776501
PAOX	0.230586831	4.354836907	4.703591132	0.030099663	0.129589207
TMEM60	-0.301192058	4.355646922	9.077516455	0.002587712	0.023774878
MYL12B	-0.342118522	4.356371933	11.19302857	0.000821052	0.010432753
LRR1	-0.040842138	4.356790916	0.204378868	0.651209327	0.813888005
TTC33	-0.020728447	4.357958596	0.037413742	0.846625103	0.928302005
SP5	0.063823022	4.358377369	0.251202956	0.61622931	0.792126125
A2ML4	-0.73263929	4.358890758	13.69356845	0.00021519	0.003795235
ERO1A	0.280355245	4.358949922	5.055696378	0.024545116	0.113675736
ARL6	0.154995668	4.359316701	3.172435472	0.074890693	0.232872277
ZDHHC18	-0.131696735	4.359772871	2.441507966	0.118162584	0.310416611
CCSAP	0.06068694	4.35995398	0.487038122	0.485251514	0.699931071
CASZ1	-0.085319003	4.360336683	0.494677446	0.481848189	0.697173002
LOC421255	0.074682275	4.360529398	0.69189324	0.405521165	0.636661652
MELTF	0.101265952	4.360809204	0.598461579	0.439165606	0.664502993
SP8	-0.035180258	4.361107319	0.14030993	0.707973094	0.848855819
GALM	0.202690328	4.361464668	4.643376973	0.031173602	0.132873572
TMEM132B	0.330651884	4.36156332	8.232073595	0.004115648	0.033148795
NSMCE1	-0.05376172	4.361755678	0.38256598	0.536233157	0.737796005
PLEKHF1	-0.004794599	4.362074933	0.002658566	0.95887827	0.982278445
HINFP	-0.077331179	4.36234988	0.896380538	0.343754079	0.580746753

LOC100858941	0.132027687	4.362505841	2.070214479	0.150200397	0.361300171
GNPDA2	-0.036723501	4.365249011	0.192827274	0.660573846	0.81998662
RCAN1	-0.278546165	4.366423023	7.684446099	0.005569877	0.041136594
KIRREL3	-0.137344101	4.366650659	0.846129412	0.35764962	0.59400061
ASB5	0.118141629	4.367108336	1.968310228	0.160627336	0.376057489
TOB1	-0.143910518	4.367290633	1.549149515	0.213261039	0.441687905
LOC112532087	0.232503889	4.367793453	6.488888006	0.010855086	0.065765993
SLC18A3	-0.447913843	4.368116617	4.81620527	0.028193349	0.124609464
POF1B	0.048472014	4.368372612	0.275416159	0.59972149	0.782152423
TRMT10B	0.082838353	4.368381383	0.544155294	0.460715721	0.682585354
ANGPT4	-0.234895026	4.368519501	3.686376418	0.054858675	0.190718017
C2H8ORF76	0.004034177	4.369317793	0.002027023	0.964089401	0.984735636
PCGF5	0.252563694	4.370077464	5.235230518	0.022133875	0.106122644
LOC101749363	-0.948035287	4.370189424	34.1263229	5.16E-09	4.86E-07
NDUFAF5	0.038472214	4.370850223	0.162397869	0.686958384	0.836953962
DHPS	-0.045264558	4.371297989	0.245602266	0.620188823	0.795058816
NSDHL	0.312271439	4.371548801	7.883550596	0.004988644	0.038241869
LOC422426	-0.009804885	4.37366651	0.010564905	0.918133035	0.962365851
TSPAN12	0.029532659	4.374154705	0.082861124	0.773456906	0.887781347
PPIL3	0.210218324	4.374766724	6.038716673	0.013995454	0.078480313
BRINP1	0.060942251	4.374855231	0.207336821	0.648862833	0.812579461
AASS	-0.160439432	4.374871977	2.247045563	0.133869776	0.336206832
KLHL41	-0.261327761	4.37513479	2.260495852	0.132711562	0.334472163
VCPKMT	-0.248961335	4.376145728	7.880123704	0.004998106	0.038252947
IKZF2	-0.237582347	4.376411325	6.256337795	0.012374976	0.072225967
LOC101748312	-0.033090429	4.376958068	0.127913141	0.720605655	0.855792151
ECEL1	0.049937015	4.377197387	0.095364662	0.757464773	0.877999637
NOCT	-0.253710831	4.377654332	9.450902629	0.002110452	0.020869886
OIP5	0.159029912	4.379550631	1.692935084	0.193214512	0.417401941
TMEM120A	0.037979578	4.379758774	0.190043725	0.662880228	0.821016009
LMBRD2	-0.151252204	4.380273964	1.583567425	0.208247582	0.436024652
C10H15orf40	-0.278296642	4.380548787	8.176557788	0.004243521	0.033880222

ABHD10	-0.00014997	4.380695808	2.39E-06	0.998766592	0.999310367
EPB41L4B	-0.232226014	4.38166895	4.842690775	0.027763542	0.123439363
AHR	-0.156963651	4.381818932	3.150736684	0.075892693	0.234916151
C3H2ORF3	-0.106020872	4.382454302	1.498834682	0.220850752	0.450215041
MOB2	-0.068109544	4.383761205	0.657479101	0.417451329	0.647484364
DUSP16	-0.239992126	4.384075827	5.521436285	0.01878483	0.095109037
SKIL	-0.143436655	4.384558276	2.460452097	0.116745176	0.308106153
GPT2	0.135743917	4.385258753	0.74863608	0.3869084	0.621136991
ELMO1	-0.285954991	4.386197242	9.867950392	0.001681828	0.017627053
ESAM	-0.016963329	4.386210743	0.028441969	0.86607394	0.938134876
RAD9A	0.237686854	4.387454969	6.310619303	0.012001691	0.070508356
SLC2A14	-0.135588828	4.387735219	2.520907175	0.112345937	0.300105908
ST6GALNAC6	0.173303435	4.387813215	2.831324102	0.092442113	0.265793286
PPM1H	0.13136348	4.388537268	1.762407848	0.184324465	0.406903552
SGK1	-0.111359749	4.388580205	1.08335219	0.297948857	0.534872864
LOC769660	-0.26245507	4.389458468	4.580311614	0.032341333	0.136141375
LOC107051855	0.357222149	4.390494461	8.728160294	0.003133325	0.027222208
HINT3	0.0034054	4.390684462	0.001632572	0.967770167	0.986817198
FASTKD3	0.009761881	4.392232833	0.01573082	0.900189024	0.954481623
HOOK1	0.032630073	4.392236955	0.096886329	0.755598648	0.876577535
TFB1M	-0.345342959	4.392496466	10.73590598	0.001050765	0.012474997
ORAI1	-0.102142129	4.392986628	1.11458012	0.291088495	0.528215834
HVCN1	-0.157547509	4.393244574	2.671525271	0.102158077	0.282238273
FOXO1	0.131141435	4.395733089	1.260040539	0.261643417	0.496655726
TLE1L	0.130665618	4.395881251	0.82775894	0.362921356	0.599097263
TEX11	0.052920383	4.395951506	0.447456649	0.503545232	0.7140723
HSD17B7	-0.117795689	4.396881447	1.584679414	0.208087947	0.43586316
CACNG5	-0.105088059	4.397470129	1.133147729	0.287104917	0.523619173
CDH7	0.414633643	4.397872772	14.76363839	0.000121863	0.002404381
FEM1C	-0.042070755	4.398333593	0.165493827	0.684148037	0.835137682
BDH1B	-0.193611875	4.39878558	6.098015969	0.013533374	0.076804448
TMEM19	-0.069536257	4.399537847	0.777525	0.377899153	0.612700264

COX5A	0.047789705	4.40002116	0.255292821	0.613372652	0.790557452
SFMBT2	0.220893211	4.400241369	4.780000251	0.028792117	0.126061116
TRAFD1	-0.19071537	4.400346463	4.289329028	0.038352298	0.151618459
EARS2	0.086622523	4.400394263	1.267738236	0.260191414	0.494793681
ARL6IP5	0.025810437	4.400452169	0.068559678	0.793445547	0.898626641
LHX9	-0.483198449	4.400666566	0.729465799	0.393056399	0.62615185
FOXF1	0.629548252	4.400959828	18.56196187	1.64E-05	0.000484278
TBC1D8	-0.423282167	4.402545334	17.25466665	3.27E-05	0.000836553
GSTM2	0.268030274	4.403572574	5.826965388	0.015782325	0.085202809
WDR19	0.063443881	4.404025041	0.476416286	0.490050092	0.70373877
SUMF2	0.221755926	4.404116167	5.738017342	0.016601522	0.088321386
HHIPL1	0.09596548	4.404576312	0.783545888	0.37605887	0.610590634
GALK1	0.124595124	4.404703191	1.642373203	0.200000166	0.426130096
TAF1CZ	-0.077043785	4.404826726	0.535850592	0.464157481	0.685242361
LOC112531698	0.077875233	4.405429427	0.483094893	0.487023792	0.701368835
GGT5	0.178501581	4.40569155	1.968527026	0.160604297	0.376057489
FARS2	-0.091933906	4.40609613	1.100946947	0.294058383	0.531081633
TXNDC15	0.014356865	4.406104547	0.031540329	0.85904029	0.933987549
C14H16orf91	0.228589363	4.406105503	8.110827666	0.004400163	0.034793217
TP53RK	-0.048342458	4.406529878	0.338229062	0.560853229	0.755874706
LOC422372	-0.001743111	4.407031733	0.000372864	0.984594065	0.992968171
PXDC1	0.068147469	4.40720136	0.473532877	0.491366354	0.704487482
TBX1	-0.027293908	4.407339258	0.017897683	0.893574786	0.951501845
UQCR11	-0.012493345	4.407804044	0.01840186	0.892095245	0.951008202
ST6GALNAC5	0.038744443	4.40791961	0.191882903	0.661354092	0.820458761
MYD88	-0.078621812	4.408410633	1.027694474	0.310700785	0.54838372
PYM1	-0.011156453	4.409853386	0.01585816	0.899787981	0.954303011
C21H1orf159	-0.048516567	4.411174159	0.344879196	0.557026307	0.753115441
SNAPC3	0.128136673	4.411592161	1.74038877	0.187089574	0.410102577
SIPA1L3L	0.032496546	4.412536769	0.103320361	0.747880878	0.872702382
ZNF438	0.068089052	4.412631333	0.500953614	0.479081412	0.695175012
MTCH1	-0.032188661	4.412695286	0.156372579	0.692518394	0.839401545

N4BP2L2	0.166812671	4.412898642	2.680261314	0.101599055	0.281213197
B3GNT9	0.009316724	4.413189144	0.010031049	0.920221172	0.963369347
RASSF1	-0.003191173	4.413386456	0.000737163	0.978339499	0.990904465
TMEM86A	-0.058384657	4.413530261	0.319633049	0.571828252	0.763690763
COLGALT2	-0.482080392	4.414037135	10.50992711	0.001187349	0.01356794
FHDC1	0.003098161	4.414372562	0.000912482	0.975901719	0.990018742
DNAJC15	0.219995933	4.414451704	4.596727919	0.032033038	0.135293541
LOC107054200	-0.062024355	4.414800271	0.387761941	0.533478173	0.735495698
C1HXORF36	0.351065365	4.415230144	13.08024601	0.000298426	0.004910604
MB21D2	0.062353653	4.416072723	0.428750628	0.512602633	0.720190944
UBXN2B	0.087376565	4.416402912	0.403208387	0.525436947	0.729995352
TFCP2L1	-0.476829425	4.417593347	9.717736515	0.001824981	0.018804051
PUS7L	0.225782128	4.417730014	4.011374744	0.045194286	0.16817548
LOC112529922	-0.112104704	4.41806071	1.072873273	0.300297342	0.53775933
RAP1A	-0.008024287	4.418185551	0.01071053	0.917572744	0.96231374
NUP210	0.136932076	4.419652746	1.163724907	0.280694365	0.516358387
NUMBL	-0.323094624	4.419703564	11.26967456	0.000787835	0.010072907
TMEM242	0.058383004	4.420513748	0.420401412	0.516736816	0.722783697
PDGFC	-0.043240144	4.420579895	0.150851519	0.697722941	0.842758423
MAP1LC3A	-0.098723265	4.420883768	1.463789452	0.22632814	0.456254766
UXT	0.119982239	4.421185985	0.999511075	0.317428842	0.555342192
TTC38	0.145303855	4.422137965	0.928121728	0.335351469	0.572606457
CDK7	-0.097288955	4.422561156	0.855987137	0.354864105	0.591500085
TADA2B	-0.122155711	4.423903632	1.761138635	0.184482556	0.407034119
NUB1	0.087758799	4.424527795	1.002083624	0.316806857	0.554580513
ALKBH3	0.004371057	4.424661622	0.002097588	0.963470108	0.984418122
ARHGEF9	0.123849313	4.425894507	1.714742723	0.190371181	0.41386966
STARD4	-0.145477492	4.426042598	1.897139654	0.168398832	0.386679623
RASGEF1B	0.299944751	4.426828409	8.58402159	0.003391256	0.028839264
MGMT	-0.005516281	4.427646489	0.003833275	0.950631758	0.97925664
SUMF1	-0.036573069	4.42783929	0.160906271	0.688323503	0.837754371
COQ3	0.179759826	4.428033486	3.614256344	0.057286328	0.196466554

ENOX1	0.386318211	4.428726915	8.382930333	0.003787613	0.031272035
FAM134B	-0.019938636	4.428921202	0.030027326	0.862428243	0.93597525
FUT8	0.127287718	4.429405957	2.431924383	0.118886871	0.311311817
MPP3	-0.403916552	4.429975591	14.18253126	0.000165904	0.003077727
CNTN4	0.179193992	4.430025125	2.475358212	0.115643094	0.305948797
TNK2	-0.112877741	4.430203744	1.069777733	0.300995656	0.538532513
NUDT9	-0.325041356	4.430450828	10.74846414	0.001043659	0.012426489
VWA5B1	0.701469609	4.430464661	38.65579687	5.06E-10	6.07E-08
ARL9	0.216498035	4.430657282	5.285735579	0.021500808	0.104118832
SOGA3	-0.258753107	4.430915822	2.138269627	0.143663857	0.352098417
CDH1	-0.04900617	4.431156397	0.157196532	0.691750828	0.839091293
POP4	0.158246036	4.43190054	2.963601274	0.085158077	0.252254934
LOC107057564	-0.189018095	4.432270197	2.593869095	0.107277989	0.291703215
GGPS1	-0.037814615	4.432974563	0.214332245	0.643392819	0.809529656
SLC25A30	-0.309300952	4.433123481	8.376372174	0.003801304	0.031322216
MCAT	0.093326682	4.433249303	1.461624276	0.226671858	0.45664611
PATJ	-0.354904925	4.43414405	11.2925203	0.0007782	0.009988515
DPH6	0.182868111	4.434687329	5.000237928	0.025343834	0.116100444
ANKRD13B	0.060884597	4.434986827	0.326466172	0.567747381	0.760834201
POLN	0.110875021	4.435058151	0.718161137	0.396747753	0.629433501
GDE1	-0.075049979	4.435059315	0.884598295	0.346945329	0.58353872
KIAA1147	-0.294017562	4.435149434	10.66306605	0.001092961	0.012818723
ADAMTSL1	-0.178996172	4.435282224	1.476913598	0.224258044	0.453839147
DFFB	-0.089654451	4.436718816	1.210012477	0.271329651	0.506377385
TSPOAP1	-0.284644575	4.436881258	6.002604444	0.014284776	0.079410806
LOC423240	0.093552064	4.437662155	1.362948627	0.243027112	0.476013219
DSTYK	-0.14889168	4.437983553	2.607399766	0.106366039	0.290287555
SNX5	-0.040486304	4.438374593	0.226325723	0.634261735	0.80403489
LOC107050459	0.193099994	4.438932739	4.577053908	0.032402879	0.136297982
EIF2AK2	0.006648594	4.438949507	0.006774484	0.934402386	0.971393237
PPL	-0.265889389	4.43904001	5.574930322	0.01821939	0.093279422
PRKCB	-0.261667462	4.439094577	5.248873112	0.021960989	0.105462945

DTX2	0.061654809	4.440076835	0.512608546	0.474011812	0.691936275
LRRK2	0.132876494	4.440160345	1.580556632	0.208680533	0.436268268
MICAL2	-0.209970328	4.44045526	4.534048969	0.033226915	0.138698346
ID4	0.118332282	4.440632675	0.795709642	0.37237922	0.606803333
GPR176	-0.237589879	4.440974944	5.966097017	0.014583525	0.080708796
SIRT7	-0.066325584	4.440987364	0.660632063	0.41633689	0.646499375
MAB21L2	0.233575102	4.441071072	0.632718579	0.426359872	0.654467617
IFT46	0.013991838	4.441898297	0.028933222	0.864933316	0.937580877
PIEZO2	-0.325515426	4.442187831	9.429805908	0.002134869	0.021007195
ADGRG1	0.032527184	4.443092791	0.067065832	0.795657835	0.900396293
MYO6	-0.093268239	4.443111268	0.732114939	0.392198512	0.625832839
SHB	0.199120596	4.443251289	4.49229855	0.034047869	0.140662237
FAM184A	-0.16166208	4.443816013	3.804264841	0.051122213	0.1821997
PKP2	-0.050621979	4.443827305	0.100166261	0.751630205	0.87434463
VSTM4	0.187993395	4.443925251	2.895387516	0.088833412	0.259268143
FASTKD1	0.281676305	4.444203713	11.35755871	0.000751415	0.009751213
KIAA1324L	0.092316696	4.444253097	0.579494161	0.446510662	0.670621395
FBXO28	-0.085824149	4.445068557	0.826958072	0.363153612	0.599097263
PRKG2	0.150094989	4.445082861	2.743380773	0.097657959	0.273991768
PDE1A	-0.427674706	4.445652935	7.137643375	0.007548229	0.0510506
LOC107055182	-0.172333884	4.446886647	2.961962544	0.085244416	0.25228531
FRRS1	-0.362880619	4.446906655	9.877561629	0.001673065	0.01755758
OGFRL1	-0.324982259	4.447116787	11.12389743	0.000852225	0.010688541
PGBD5	-0.157667256	4.44724437	1.18044143	0.277266148	0.512364651
KAZALD1	-0.189433644	4.447581802	2.566607231	0.109141584	0.294996353
PPP2R3B	-0.018938756	4.447723854	0.039683417	0.842100847	0.925549512
CERS1	-0.103322592	4.447749406	0.980502928	0.322074673	0.559752719
GM2A	1.645493093	4.447943451	135.2748635	2.87E-31	6.76E-28
ADAM22	0.418605146	4.447943985	16.40469557	5.12E-05	0.001214922
EPN3	0.14855165	4.448358032	1.972335851	0.160200143	0.375748545
SGSM3	-0.224033303	4.448554166	6.942087217	0.008419082	0.05517164
SLC25A22	0.286730866	4.449176116	11.54280938	0.000680118	0.009094651

MEA1	-0.058977335	4.449187404	0.242172244	0.622641589	0.796762373
PTPRM	0.011295535	4.449247452	0.01307719	0.908955955	0.95829261
LIN9	-0.001959318	4.449422902	0.00052498	0.981720108	0.991828264
PAQR8	0.167699686	4.449429162	1.872416348	0.171198589	0.389828743
LOC112530288	-0.26520518	4.449511136	7.285425411	0.006951627	0.048015912
C1H11ORF87	-0.623584444	4.450091098	22.78926278	1.81E-06	7.75E-05
LOC107049790	-0.019327598	4.450451634	0.027397971	0.868532093	0.939356373
MYO1D	-0.01779602	4.450455929	0.03770445	0.846037811	0.927917895
LOC101751456	-0.506499006	4.450469602	1.042604751	0.307216449	0.545216422
GANC	-0.204914052	4.451236679	6.222838674	0.012611276	0.073250456
PQLC3	-0.439753685	4.451670212	19.17559172	1.19E-05	0.000373695
EHD2	0.008585361	4.452760389	0.006095538	0.937769249	0.973073086
GRAMD3	-0.185372035	4.452785409	2.720291989	0.099079889	0.276285304
KMT5A	-0.109276285	4.452846161	1.618989765	0.203232708	0.430265966
FAM210B	0.153131677	4.452933061	3.441228285	0.063588403	0.210490276
MED11	-0.009025409	4.453276943	0.01437133	0.904577825	0.956421967
PTX3	-0.13426815	4.453284992	1.583524055	0.208253811	0.436024652
GALK2	0.284789389	4.453385218	7.54836979	0.006006439	0.043387161
SYTL4	-0.428093438	4.453451089	20.2811721	6.69E-06	0.00023427
HOXA9	-0.830609485	4.453738076	10.35783117	0.001289266	0.014412275
ERCC8	0.062107381	4.454161782	0.268444344	0.604377233	0.785137502
TRAPPC6B	-0.008685893	4.454180557	0.011004974	0.916451513	0.961809405
CCBL1	-0.242498096	4.454441046	9.367689736	0.002208437	0.021548861
FOXD3	0.302993261	4.454981632	3.574779699	0.058663229	0.199525308
MITF	0.06602915	4.45510167	0.472591037	0.491797579	0.704555909
TEX264	-0.108413281	4.455540336	1.461271329	0.226727947	0.456703192
SLC35A1	-0.004879584	4.455879766	0.003550865	0.952482875	0.979802073
GDF10	0.441713619	4.456454693	8.306987625	0.003949284	0.032202907
DPH5	0.068778344	4.458493999	0.775983594	0.378372321	0.613175422
PRNP	-0.139379549	4.458648529	2.278097711	0.131212724	0.33196577
TRIQK	0.180466455	4.45865164	3.812983416	0.050856789	0.18164716
ADCK2	0.18269615	4.45924982	4.722382193	0.02977247	0.12858425

VWF	0.198159202	4.460188164	1.92021506	0.165832954	0.383518518
TCHP	0.119618362	4.460561406	1.589777398	0.20735794	0.434769993
UBALD1	-0.197469395	4.46062327	3.577974684	0.058550495	0.199265438
MTMR7	-0.220548582	4.461391921	6.552231946	0.010475267	0.06407596
SERHL2	0.130169082	4.461778837	1.84285199	0.174617044	0.394145879
SCARF1	-0.03582795	4.464308417	0.150335852	0.698214612	0.842990788
CACNA1D	-0.424805126	4.464915764	10.9866756	0.000917693	0.011285975
IKZF5	-0.135017482	4.465515572	1.459452211	0.2270173	0.456782792
RIC3	-0.013173742	4.466832027	0.014924471	0.90276775	0.955554055
ELF1	-0.089735267	4.467055701	0.959651702	0.327274647	0.565030355
CCDC93	0.06991873	4.467145497	0.714039344	0.3981061	0.630434162
LOC107051705	0.853944447	4.467378861	37.43035238	9.47E-10	1.07E-07
MRM3	0.053725812	4.467547847	0.338080725	0.560939164	0.755896646
SHQ1	-0.165752557	4.467582674	3.273512665	0.070406719	0.224022187
SSNA1	0.125625384	4.467621286	2.126324805	0.144787544	0.353614387
THSD7A	0.001975582	4.469551721	0.000232628	0.987831017	0.994418168
DBNDD1	0.031749399	4.470069856	0.063289058	0.801371002	0.903215783
MMAB	0.095108775	4.470382877	1.258394371	0.261955233	0.49705643
INSIG1	-0.177140953	4.471047363	3.26549156	0.070751815	0.224579691
MAP3K2	0.018666796	4.47126593	0.041939926	0.837734328	0.923550741
SLC47A1	0.095255891	4.471983621	1.239592602	0.265549584	0.50073011
PROZ	0.089651793	4.472739995	1.109694747	0.292148313	0.529380079
LOC107051310	0.291757569	4.472784774	6.265624318	0.012310278	0.071946079
ATG10	-0.272431735	4.473136337	5.525627858	0.018739877	0.094998357
SIMC1	0.13764969	4.473341437	2.189985773	0.138910599	0.344313238
ZNF385C	0.062813827	4.473549065	0.18303933	0.668774195	0.824382304
OSGIN2	-0.298579341	4.473998852	8.542170944	0.003470115	0.029297457
TAF4	-0.234573983	4.474014774	4.423660709	0.035443919	0.144220989
GTDC1	-0.126407336	4.474637165	1.982614452	0.15911525	0.374165128
OSER1	-0.044520887	4.474993945	0.274883157	0.60007476	0.782392568
ITFG2	0.103006227	4.475418376	1.217240779	0.269902831	0.504595753
EVC2	-0.302368819	4.475595024	13.25571066	0.000271751	0.004586857

ARL8BL	0.12878338	4.477926378	1.281013003	0.257710769	0.492541889
SMYD4	0.025677326	4.477976078	0.097617021	0.754708262	0.875976896
MED26	-0.222603062	4.478414204	4.792965333	0.02857619	0.125601973
RASL11B	0.038411249	4.479135575	0.077196057	0.781133814	0.891988682
GLT1D1	-0.083840136	4.479694413	0.867922165	0.351531184	0.587969672
ECSCR	0.069492612	4.480025289	0.475008549	0.490691981	0.703969418
ARL2BP	-0.10794177	4.48038009	0.435122297	0.50948616	0.717892027
PARS2	0.208295331	4.481031275	5.210554753	0.02245017	0.107108701
GTSF1L	0.094582105	4.481158519	1.170854678	0.279225697	0.514618765
TMEM41A	0.168985175	4.481235799	4.058901459	0.043939075	0.165488093
SARM1	-0.134157022	4.481835953	1.012750575	0.314244787	0.551930556
KBTBD8	-0.163963985	4.481836722	2.213968357	0.136766459	0.340725525
GNMT	-0.008521444	4.482038976	0.006973073	0.933450057	0.970979246
MIR6578	0.082706454	4.482594051	1.174740505	0.278429331	0.513857631
CYB5R4	-0.1103069	4.483148775	2.03216715	0.154000876	0.366886408
SS18L2	0.312772104	4.483491469	15.5099893	8.21E-05	0.001783724
ITPRIPL2	-0.128618616	4.483690446	2.134301438	0.144036064	0.352738591
ARHGAP40	-0.417442607	4.483814791	10.42068614	0.001246116	0.014031704
DOLPP1	0.040334799	4.484270886	0.252538723	0.615293131	0.791854252
SMIM20	0.006554391	4.484296301	0.006733457	0.934600872	0.971393237
ACP6	-0.129942548	4.485677736	2.711017396	0.099657415	0.277460106
CABLES2	0.033426538	4.486021386	0.154197657	0.694555774	0.840670486
SAYSD1	-0.212607501	4.487147316	5.759534161	0.016399419	0.087557574
LOC112530904	-0.215067465	4.487604517	2.971333721	0.084751955	0.251459324
MXD1	-0.114513224	4.489110703	1.587971388	0.207616205	0.435200594
PURA	-0.058640697	4.489491609	0.543910589	0.460816555	0.682585354
CAMK4L	0.319448545	4.490343623	10.01079136	0.001556256	0.01661791
ZBTB34	-0.115525464	4.490402501	1.37449812	0.241040545	0.473806419
BMP5	-0.18641839	4.490425876	2.354468622	0.124924447	0.321092123
LOC425431	0.049408188	4.491371211	0.113884197	0.735764638	0.864920575
BBS12	0.131784299	4.493039666	3.067219719	0.079885989	0.24221431
PPP2R3C	0.235150109	4.493212831	7.048122732	0.007934837	0.052881874

CNTFR	0.172954937	4.493341531	2.170633938	0.140668236	0.34739544
LOC107052718	4.016667525	4.493620662	478.1218094	5.48E-106	9.01E-102
SYNM	-0.048557953	4.493712589	0.294942285	0.587070667	0.77348253
NPHP3	-0.144439397	4.494063618	1.111215318	0.291817918	0.52883963
ZNF692	-0.379825929	4.494376413	7.83170978	0.005133752	0.038875233
MYF5	-0.048601409	4.495043168	0.114197688	0.735414821	0.864745668
LOC101748630	-0.197946009	4.495426775	4.835966395	0.027872015	0.123754456
NFIB	-0.308733096	4.495927908	4.968264159	0.025816544	0.117448503
CMAS	0.057265281	4.496101519	0.532414074	0.465593697	0.686036347
CNST	-0.362913519	4.496619865	17.16097794	3.43E-05	0.000873409
STOX1	-0.745019188	4.496635307	23.7998279	1.07E-06	5.01E-05
FZD9	0.159520281	4.496963297	2.37426529	0.123349619	0.318387133
TBC1D19	-0.409848984	4.497153335	26.57181437	2.54E-07	1.48E-05
RFTN1	-0.039400916	4.497381102	0.152410109	0.69624273	0.841845405
RPS6KA5	-0.114606686	4.497481725	1.826501913	0.176541281	0.39680191
RGS9	-0.60448673	4.499085952	13.77737753	0.0002058	0.003653108
CD82	0.198720889	4.499527675	3.1428957	0.076258307	0.235560436
ZNF341	-0.105073318	4.500525599	1.211020146	0.271130179	0.506349687
DAGLB	-0.138858377	4.50141124	2.433364825	0.118777695	0.311124956
THBS2	0.090657909	4.501480323	0.605335998	0.436549313	0.662740596
GUF1	0.273118062	4.50260508	7.610610589	0.005802583	0.042341668
LOC417551	-0.190074164	4.502623894	1.931891839	0.164551617	0.381634511
EDIL3	0.149869407	4.503117542	0.909975851	0.340120826	0.577335279
VWDE	-0.463294448	4.503218074	2.201989011	0.137832788	0.342448818
ME3	-0.032287737	4.50469365	0.093315533	0.760003713	0.879827008
COMT	0.120475301	4.504722602	1.500236438	0.220634986	0.450049422
CLUAP1	0.201707098	4.504737292	5.362304168	0.020576569	0.10100503
ACOT8	-0.085836523	4.504953617	0.719607622	0.396272646	0.629052616
RIMBP2	-0.339744252	4.50505011	6.106797956	0.013466288	0.076567994
TMEM62	0.248698203	4.505052485	7.265159154	0.007030504	0.048384333
DYNC2LI1	0.341980837	4.505623138	13.38529076	0.000253605	0.004346954
CERS6	-0.022766841	4.506331695	0.046889609	0.828566781	0.918759906

GOLPH3L	-0.085870551	4.50702304	0.696569198	0.4039389	0.635646128
FOXB1	0.212598384	4.50707534	1.985101728	0.15885398	0.373901051
TLDC1	0.019189065	4.507896203	0.034618038	0.852398312	0.931293687
KATNA1	0.069819927	4.508703483	0.699859257	0.40283099	0.634882978
TNFAIP6	-0.021625282	4.509639752	0.011037847	0.916327281	0.961740363
EFCAB5	0.011176906	4.510030751	0.011077304	0.916178412	0.961731649
GEMIN6	0.180564857	4.511148083	3.868619345	0.049196976	0.177598932
LOC112530227	-0.070650089	4.511471947	0.639442653	0.423912693	0.65309226
GATA2	-0.115830828	4.511939943	1.053165971	0.30477906	0.542764009
MT	-0.388163571	4.5123123	12.296886	0.000453715	0.006732078
PYGO1	-0.01276933	4.513459203	0.015945197	0.899514806	0.954259309
LOC107050152	-0.056215203	4.513519632	0.12039981	0.728601268	0.860484738
GRM3	-0.806763344	4.513530228	25.41799544	4.62E-07	2.51E-05
DYNLL1	-0.023884879	4.513601404	0.070691641	0.790332402	0.896952871
TRADD	-0.297388504	4.514109516	12.37360981	0.000435445	0.006531674
PIWIL1	-0.277078026	4.515732234	2.734042973	0.098230331	0.2750349
BTN1	-0.376254776	4.515873397	15.1193811	0.000100922	0.002078434
HYAL1	-0.051870587	4.515980659	0.305165286	0.580662224	0.769731709
PRELID3A	-0.174377393	4.516165349	4.021049531	0.044935742	0.167440587
MYO1A	0.041194591	4.516224848	0.086523908	0.768643694	0.884709423
ZBTB43	-0.103873103	4.516622746	1.206756953	0.271975351	0.506750574
SPIN1W	0.252890389	4.517409151	0.136716797	0.711567026	0.850067911
LOC107051108	0.175400576	4.518490395	2.938264687	0.08650362	0.254777708
ITGB1BP1	-0.021326912	4.51870587	0.051450722	0.820557867	0.915781315
CELF6	-0.175039354	4.519385212	4.55432827	0.032835642	0.137623659
LOC107050194	-0.218642648	4.519716348	2.364696622	0.124108038	0.319592766
CPE	0.003872005	4.520109315	0.001036393	0.974318091	0.989871494
ZNF277	0.031892078	4.520641329	0.133269604	0.715065801	0.852391173
ZFP92	-0.216140287	4.520664952	7.033107105	0.00800164	0.053220288
TRIM23	-0.151089078	4.521053384	2.029347766	0.154286808	0.367142361
LOC101751371	-0.271336352	4.521217054	2.790092184	0.094848771	0.269696998
DOCK10	-0.334583959	4.521471068	7.767186922	0.005320384	0.039858242

MED19	0.010252347	4.522571482	0.017224324	0.89558429	0.952238271
C3H1ORF131	0.075917542	4.522693048	0.516702732	0.472251602	0.690861219
RHPN2	-0.263877658	4.52314484	8.86529409	0.002906434	0.02584274
CLGN	0.201519082	4.523300885	3.784409644	0.051732156	0.18345962
LOC107050879	-0.091912356	4.52466495	0.229739623	0.631716961	0.802819169
SLC48A1	0.042985058	4.525036952	0.277087895	0.598616297	0.781392287
LOC425795	-0.081953775	4.525234784	0.703352889	0.401659363	0.634066049
LOC107052842	0.31981423	4.525450875	12.78823236	0.000348807	0.005513556
ZSWIM6	-0.00796944	4.52587912	0.004835569	0.944561182	0.976242337
PLTP	-0.347670561	4.526042119	7.30040323	0.006893914	0.047809051
ZNF511	0.11085791	4.526214335	1.781350657	0.181983561	0.404175934
ZGRF1	0.061212219	4.526451627	0.431640938	0.511184861	0.71887419
UBE2R2L	0.220322037	4.526545298	0.12862245	0.719864627	0.855564362
EYA2	0.16773411	4.526581757	2.955292271	0.085596829	0.252781015
GUCA2A	1.254577487	4.52766325	5.713207765	0.016837745	0.089079459
LTK	0.020624334	4.527896613	0.032550808	0.856824091	0.933242474
DEF6	-0.268772357	4.528246884	6.51263252	0.010711082	0.065013228
LOC420411	-0.011823637	4.52858945	0.013568273	0.907269812	0.957480331
LOC425347	0.102516841	4.528706911	0.024708262	0.875096198	0.9432535
ACBD4	0.10262209	4.529784111	0.896018644	0.343851507	0.580845021
RUFY2	-0.071922474	4.530359094	0.758560118	0.383779506	0.618459678
ZFAND2B	-0.011839339	4.530553937	0.015831893	0.89987057	0.954329094
PLPP2	0.030059372	4.531035838	0.039100294	0.843250101	0.926094935
CNIH4	0.16987568	4.531072661	5.206652712	0.022500614	0.10713183
PLK3	-0.283216223	4.531106539	10.0570496	0.001517661	0.01628494
PDCD7	-0.156151692	4.53193412	4.19527627	0.040536748	0.157765417
TP63	-0.133695683	4.532302372	2.039092028	0.153301128	0.365854976
FBXL17	0.175129988	4.532568388	2.960993776	0.085295502	0.252328347
FIBIN	-0.2706253	4.53276623	1.797776821	0.179981499	0.401619945
PLEKHG5	-0.182275169	4.534246846	2.982381014	0.084175359	0.25029012
SRXN1	0.191435582	4.534308752	2.16901894	0.140816043	0.347551822
LOC112530523	-0.102103701	4.534444293	0.919910207	0.33749855	0.574603067

DNAJC30	0.006790552	4.534553247	0.006873664	0.933925043	0.971309744
TSFM	0.225368645	4.534712833	8.77978678	0.003045868	0.026687837
SNX30	0.022711119	4.535474814	0.060146367	0.806264751	0.90650898
TNFRSF1A	-0.23391366	4.535534357	7.550931645	0.005997906	0.043344549
TBC1D8B	0.059050545	4.535971715	0.362848564	0.546928456	0.746615874
ZBTB38	-0.27502446	4.536278475	7.762041181	0.005335563	0.039925733
LACTB2	0.041768061	4.536664136	0.280701185	0.596242003	0.779405955
COX19	-0.055732551	4.537528794	0.567337903	0.451318706	0.674763702
LOC101748546	-0.103855854	4.53778114	1.40245632	0.236312735	0.468610034
NCOA7	-0.009183275	4.538397458	0.012941327	0.909428086	0.958432772
LOC101747556	0.486372559	4.538769091	19.41025259	1.05E-05	0.000339532
SARAF	-0.332799461	4.539079843	12.74646135	0.000356683	0.005600405
ST3GAL4	-0.040252732	4.539619562	0.195404504	0.658456052	0.818283689
TUBE1	-0.119221026	4.539749515	1.385269068	0.239205668	0.47181432
C23H1orf216	0.134812167	4.540176652	1.388844306	0.238600366	0.471215677
CCNI2	0.069743301	4.540646788	0.612820719	0.433727732	0.660282156
EVA1B	-0.606554845	4.540897731	19.5898107	9.60E-06	0.000313985
VHL	-0.086059981	4.541204999	1.147212371	0.284133459	0.520184253
TMTC1	-0.172582701	4.541375212	2.236055705	0.134824511	0.337831175
CENPJ	-0.122364533	4.541549371	1.512257455	0.218794942	0.448129296
EPB41L4A	0.144801648	4.541799212	1.294566555	0.255208095	0.49032844
TM2D3	0.045062308	4.541844622	0.304927006	0.580809991	0.769750998
LHPP	-0.028130294	4.542113577	0.079446403	0.778049034	0.889948343
NTL	-0.432562192	4.54261402	22.25560537	2.39E-06	9.87E-05
LOC112532285	0.29004491	4.54346437	11.28327626	0.000782084	0.010014937
NT5M	-0.024303348	4.543834893	0.067061229	0.795664692	0.900396293
TMEM11	0.012476134	4.544241619	0.022869066	0.87979806	0.946151989
CBFA2T3	-0.065407502	4.54434526	0.234991294	0.627847305	0.800126038
GPKOW	-0.005392391	4.54530012	0.003620269	0.952021294	0.979652938
P4HA2	-0.069421785	4.546986626	0.32531316	0.56843199	0.761255669
LOC112530201	-0.002042724	4.548744204	0.000554486	0.981213533	0.991828264
LOC112533391	-0.07646267	4.548920156	0.350885039	0.553612558	0.75174518

KIAA1324	0.059103673	4.549119634	0.19796299	0.65637011	0.816610278
ARPC1B	-0.148935611	4.54912517	1.702587774	0.1919499	0.416144349
SFR1	-0.167716972	4.549228957	3.975632452	0.046163114	0.170404892
LOC101751687	-0.144395124	4.549253987	3.025071105	0.081986727	0.245915347
RRP8	0.572476207	4.54959079	27.95925743	1.24E-07	8.19E-06
MED7	-0.08544934	4.550746443	0.99456683	0.318628757	0.556348428
EXTL2	-0.057226252	4.550831018	0.526666183	0.468011848	0.687539948
ILVBL	0.15370337	4.550866661	2.58126315	0.108135339	0.293044633
MANBAL4	-0.180128603	4.552549174	6.265322331	0.012312377	0.071946079
EAF2	-0.084824777	4.55317436	0.741348325	0.389229304	0.623092537
SFMBT1	0.115027288	4.553654865	1.405391539	0.235822933	0.467900217
UBALD2	-0.050465911	4.55369636	0.315503227	0.574322656	0.765040788
IRX6	-0.331902159	4.554335171	2.323179193	0.127459286	0.325421653
DTWD1	0.062507134	4.555012858	0.540927298	0.462048678	0.683437728
EPS8L2	-0.335482697	4.555712309	6.815680482	0.009036086	0.0577331
HSD17B11	-0.089875039	4.555806934	0.881335572	0.347836138	0.58438231
SPR	-0.003083192	4.556919261	0.000900695	0.976057811	0.990018742
PPIL1	-0.003436898	4.557357756	0.000826054	0.977071029	0.990525896
TRMT44	-0.113642341	4.557790981	1.72092266	0.189574322	0.413123016
GPX7	0.00196212	4.558205394	0.000450364	0.98306874	0.992467303
TCF4Z	-0.041093728	4.558503158	0.122350553	0.726498977	0.859179567
LETM2	-0.249214996	4.560079245	11.05028206	0.000886737	0.010979126
TTC14	-0.034326191	4.560129103	0.166362672	0.683364857	0.834243543
PTBP3	0.138516643	4.560311568	1.550808738	0.213016089	0.441347235
SMAD6	-0.517653245	4.560652824	8.690356372	0.003198981	0.027631177
CLN6	0.181040937	4.562012856	4.825292513	0.028045106	0.124192137
SLX4IP	0.004383276	4.562330747	0.002089651	0.963539245	0.984418122
SERPINI1	-0.169415016	4.562997047	0.751352604	0.386048326	0.620596444
PHKG1	0.117727995	4.563034208	1.912330903	0.166704568	0.384621938
CFLAR	-0.036415435	4.563141704	0.125309686	0.723345566	0.857066913
SLC36A1	-0.123369656	4.563557022	2.607678624	0.106347334	0.290287555
MFSD9	-0.463895712	4.564675457	33.74245226	6.29E-09	5.78E-07

EXOC6	0.006045724	4.566285594	0.005652964	0.940066567	0.9742282
BTBD9	0.046012196	4.566361084	0.177454206	0.673570246	0.827811004
MTM1	-0.157309238	4.566449572	4.175355011	0.041015969	0.158919708
RAI2	0.112375376	4.567056585	1.720794572	0.189590798	0.413123016
PPIC	0.205778563	4.567124561	3.557775938	0.059267098	0.200625406
LOC107054696	0.36093157	4.567379238	1.141201112	0.28539867	0.521514171
FAM110D	0.020496713	4.567515253	0.041254694	0.839047089	0.923760193
NCKAP5	-0.127675144	4.568144406	1.70280445	0.191921624	0.416137874
DERA	-0.397588146	4.568532913	11.61569799	0.000653975	0.008831043
ESRP2	-0.032711902	4.569593654	0.048678387	0.825379146	0.917552618
NDOR1	0.084271021	4.569708002	0.906875239	0.340944872	0.57807809
CMC2	0.083098958	4.570249578	0.531554629	0.465953994	0.686100024
TNNT3	0.089753738	4.570885361	0.158996285	0.690082299	0.83840108
DBX1	0.147832495	4.571381216	0.72462236	0.394631864	0.627528733
GPR180	-0.086481151	4.571989918	1.25015443	0.263522984	0.498708534
OIT3	0.164473907	4.572669569	1.487969387	0.222531788	0.452069207
C1R	-0.084289434	4.572724218	1.259987225	0.261653509	0.496655726
SOX2	0.284745299	4.573173927	4.225298783	0.039825607	0.156179781
LRFN1	0.126603848	4.574010482	1.960072548	0.16150556	0.37728194
TSPO	-0.083162646	4.574654388	1.254315637	0.262729812	0.497862243
SLCO3A1	0.104148047	4.574764144	1.354565626	0.244481531	0.477784275
OPCML	0.13053616	4.574840145	0.922779529	0.336746215	0.573918636
BRIP1	0.350137157	4.575090617	16.73027383	4.31E-05	0.001059808
GDF11	-0.059001445	4.575508163	0.142560579	0.705748603	0.847659825
ANKRD27	-0.13603165	4.575762606	2.773969786	0.095808284	0.271160185
NSUN4	0.15245313	4.576562998	4.002836628	0.045423756	0.168724131
SGMS2	-0.101895413	4.577683945	1.49477266	0.221477429	0.451003135
LOC107055013	0.057073124	4.579115205	0.490820065	0.48356172	0.698336
CALCB	0.668808185	4.579193736	24.49674864	7.44E-07	3.75E-05
SDPR	0.156077481	4.580471378	1.041344019	0.307509094	0.545618088
RIDA	-0.152752463	4.58140192	2.862437763	0.090669993	0.262671608
THNSL2	-0.106392244	4.581688044	0.788652543	0.374507878	0.60912594

MYLIP	0.014569856	4.581886565	0.013099864	0.908877404	0.95829261
SDHAF4	0.045008679	4.582664481	0.237936474	0.62570049	0.798875044
CELF5	0.045184104	4.58275429	0.270013569	0.60332267	0.784362372
UBE2QL1	-0.00091277	4.58275563	0.000126666	0.99102031	0.995986157
TTC12	-0.287289725	4.582881336	11.43037624	0.000722531	0.00950308
OTUD3	0.024267864	4.584030598	0.101810306	0.749667918	0.873444126
STAT2	0.094635824	4.584322498	0.931236647	0.334541789	0.572087272
SPSB3	-0.169679365	4.584437518	4.280081488	0.0385615	0.152201841
MZT1	0.201678904	4.584676848	4.013635009	0.045133744	0.168055361
TTC38L	0.133363309	4.585054785	0.846121415	0.357651891	0.59400061
MECR	0.146599328	4.585162824	3.230407978	0.072282679	0.227334
FAM198A	-0.357258925	4.585273387	9.871796129	0.001678316	0.017601458
METTL5	0.196810501	4.587040031	6.225226559	0.012594279	0.073229281
MUL1L	0.04955119	4.587514014	0.366629148	0.544847429	0.744825492
CHN2	0.055858281	4.587802061	0.316418849	0.573767775	0.764869254
SH2B3	-0.20787322	4.588277241	3.39147133	0.065534445	0.214558157
PSMG4	0.01679847	4.588317203	0.022781094	0.880027723	0.946151989
PMM1	0.128823066	4.589690197	3.076972818	0.079408197	0.241170521
PPM1M	0.295508632	4.590233956	12.36937921	0.000436433	0.006540528
PDSS2	0.117109126	4.591114587	2.081353216	0.149107937	0.360088451
CDH15	0.025635302	4.591174828	0.03222817	0.857527797	0.933674313
ZDHHC13	-0.171934573	4.592053582	5.455590376	0.019505812	0.097736948
CUEDC2	0.20157774	4.592131188	4.890390804	0.027006572	0.121154075
CCDC167	0.043531793	4.59214221	0.279209617	0.59721973	0.780221347
CAMK2A	-0.041482974	4.593271409	0.13045081	0.717965081	0.854176517
RAB24	0.144613786	4.593673306	2.983113302	0.084137288	0.250222135
TERB1	-0.198635652	4.593765661	1.59040744	0.207267932	0.434636653
HIST1H101	-0.059873549	4.59380751	0.160455291	0.688737687	0.838002889
TEX9	-0.190656149	4.594335228	6.095083077	0.013555855	0.076843124
SPTB	0.06244295	4.594641378	0.173340156	0.677160207	0.830158199
ISLR	-0.024923297	4.595639303	0.063582989	0.800919964	0.903047923
BCAR3	0.057646345	4.596014817	0.503210957	0.478092643	0.694475145

TMEM208	0.043820037	4.596652723	0.207192098	0.648977167	0.812579461
CZH9ORF3	0.247969649	4.597541885	6.498893025	0.010794168	0.065445111
ALPK3	-0.181150786	4.598174892	3.485534228	0.061907376	0.207007901
NIPSNAP1	0.111090877	4.598513364	1.427507265	0.232171685	0.463704852
OPTC	0.233858366	4.598995132	4.087962797	0.043189669	0.163790274
LOC107050706	0.043675246	4.59956349	0.15742901	0.69153468	0.839091293
ERBB3	0.122691981	4.600304878	0.942083145	0.331742685	0.56937808
SNAI1	-0.449952526	4.600627911	6.005510569	0.014261267	0.079368477
ZNF252L	0.327339774	4.601486253	9.891616183	0.001660335	0.017446238
EPHA7	0.282216232	4.601487631	8.338773871	0.003880778	0.031786064
ATG14	-0.129430085	4.601779862	3.052376619	0.08061909	0.243496918
MMP16	0.222885127	4.602015999	4.228350306	0.039754062	0.156010755
SAMD8	-0.236930753	4.602711584	4.699933449	0.030163785	0.129695607
FANCE	0.2335858	4.60398826	6.625478278	0.010053014	0.062205741
CCDC77	0.106968546	4.605357494	1.638820097	0.200487432	0.42687655
SAMD3	-0.155802341	4.605974959	1.024452618	0.311465159	0.549202656
BZFP2	0.881723327	4.606758832	9.428982792	0.002135827	0.021007195
RBFA	0.569757633	4.606906989	48.83635288	2.78E-12	6.27E-10
TTC19	0.011827021	4.607291396	0.01892212	0.890589992	0.950676503
PLA2G4A	0.064893749	4.607875108	0.67288654	0.412047034	0.642310909
CREG1	-0.242855732	4.609452286	3.797279601	0.051335925	0.182684396
PID1	0.092853005	4.609562012	0.857119841	0.354545941	0.591269226
SLC8A1	0.02093177	4.610366573	0.017500197	0.894756254	0.951827123
CTNS	-0.091707474	4.610380235	1.310436308	0.252315675	0.486934484
CCDC149	-0.167072435	4.61045246	3.134018797	0.076674511	0.236375962
TST	0.336395575	4.61073738	5.292537622	0.02141699	0.103862114
CDKL5	0.231849868	4.610790678	7.964542709	0.004770254	0.037043196
TOPORS	-0.020759628	4.61110994	0.031981418	0.858068446	0.933773189
NDUFA2	0.027859204	4.611225133	0.087265031	0.767683329	0.884179034
NAGLU	-0.001986208	4.611227954	0.00051911	0.981822582	0.991828264
CARD19	-0.263938401	4.611689845	1.85478702	0.173227661	0.392542358
PALMD	-0.005691749	4.61215991	0.001592263	0.96817032	0.98693873

LOC107051642	0.180176145	4.61357446	2.986675423	0.083952364	0.249899506
CHCHD10	0.012413348	4.614102057	0.023086675	0.879231893	0.94602618
CSRP2	0.076299293	4.614264178	0.351203594	0.553432594	0.751671616
FBXW9	0.010933351	4.614393282	0.01628458	0.898456786	0.953567235
GAREM1	-0.120591971	4.615121565	1.73077891	0.188311454	0.411782721
WDR53	0.015512236	4.615356543	0.034499581	0.852648167	0.931394748
MYO7A	-0.209434364	4.61537917	3.009793416	0.082762838	0.247587977
LPIN1	-0.479312519	4.615524106	17.60765321	2.71E-05	0.000724426
PTAR1	-0.016108601	4.616766638	0.015525689	0.900838548	0.954678837
FAM210A	0.137864801	4.616857774	3.009651841	0.082770067	0.247587977
SGK3	-0.227156856	4.617388433	7.125739509	0.007598507	0.051307362
APAF1	0.084505376	4.617409561	0.906625587	0.341011338	0.57809447
VIT	0.109438853	4.61796861	0.737958156	0.39031574	0.623951561
EDNRA	-0.192975454	4.618672879	2.41942246	0.119839105	0.312710511
PRKCZ	-0.051330483	4.619054425	0.48072535	0.488093947	0.702056988
CHRNA1	-0.333837854	4.620819296	4.427956554	0.035354812	0.144036502
GRIN3B	0.307766036	4.620965262	4.706633083	0.030046443	0.129461695
PLCB1	-0.042914297	4.621003067	0.188895926	0.663837121	0.821681949
LMBR1L	0.42022019	4.621119577	26.4782385	2.67E-07	1.54E-05
XIRP1	-0.066771716	4.621512808	0.065187139	0.798477713	0.901588892
KLHL28	-0.178608338	4.621762119	3.372209399	0.066304779	0.216076753
TSPO2	0.359597196	4.622908733	3.106533138	0.07797878	0.238280562
DIP2C	-0.165675033	4.623126882	2.289963883	0.130212953	0.330156971
EXOSC5	-0.04533918	4.623359096	0.320362325	0.571389982	0.763291294
TM7SF3	-0.059155239	4.623380109	0.313625662	0.57546381	0.765607847
CBR4	-0.115267734	4.623891148	1.356211705	0.244195107	0.47745134
EIF2AK3	0.014562496	4.624216113	0.01507631	0.902276858	0.955360193
EDA	0.06343383	4.62470947	0.632695108	0.426368451	0.654467617
C1H2ORF49	-0.151589277	4.624891926	2.723602745	0.098874615	0.275993518
CMC1	-0.027859368	4.625446426	0.083095051	0.773146099	0.887610344
TSPAN9	-0.101560976	4.625515791	1.289099105	0.256214041	0.490917797
PGAP3	-0.088515529	4.626229918	1.468156488	0.225636784	0.455508929

LOC107054950	-0.225446876	4.627058273	3.293411457	0.06955836	0.222335434
TMEM177	0.321246676	4.627369087	15.6255992	7.72E-05	0.001702901
MTHFSD	0.021395827	4.627646233	0.046282436	0.829663213	0.918962586
RINT1	-0.106126602	4.628463686	1.771259582	0.183226273	0.40570577
TMEM120B	-0.083053834	4.629582756	0.950245421	0.329656835	0.567297974
ADD2	-0.09113173	4.629599813	0.395590034	0.529375581	0.733053537
GXYLT2	0.211581637	4.629698647	5.54793317	0.018502523	0.094114068
SPATA2L	-0.017254975	4.6297581	0.03071936	0.86086796	0.935018438
LAMA2	-0.36359316	4.630027311	12.26270832	0.0004621	0.006827454
SENP2	-0.087660604	4.630282646	1.200822372	0.273157375	0.507798755
RAPGEF3	0.255840182	4.630705546	6.966367277	0.008305594	0.054601899
PTPRQ	-0.921168724	4.631836958	25.53269367	4.35E-07	2.40E-05
ZNF830	0.055314822	4.631899258	0.452657918	0.501075447	0.711836008
UNC50	0.044686014	4.631934832	0.286938858	0.592188893	0.776980894
DCAF5	-0.192519195	4.632363003	5.07657342	0.024251252	0.112764397
CDC37L1	-0.048233223	4.632415813	0.31618784	0.573907671	0.764915821
SUOX	0.128542364	4.632476191	2.361367188	0.124373145	0.320075086
BAG5	-0.114030718	4.633011498	1.391634831	0.23812921	0.470793722
RBKS	0.292697016	4.633055324	11.8350318	0.000581267	0.008105403
R3HDM4	-0.077575399	4.633075613	0.685235847	0.407789589	0.638395745
FGF13	-0.213764764	4.633182573	1.970861985	0.160356397	0.375840233
SLC35E1	-0.059976267	4.633580116	0.480356442	0.488260907	0.702056988
NEU3	0.064100523	4.634025805	0.28899491	0.590865336	0.776386577
LAMTOR5	-0.002276544	4.634363771	0.000918565	0.975821552	0.990018742
LOC396224	-0.434771986	4.634431661	19.39343583	1.06E-05	0.000341866
LOC112530493	0.227679047	4.634894287	0.11425015	0.735356333	0.864745668
ZNRF2	0.051953517	4.635921999	0.362126736	0.547327467	0.746757539
ALG2	-0.218082323	4.636117836	4.6023809	0.031927587	0.135021446
DENND3	-0.114089961	4.636415304	1.746445634	0.18632419	0.409122572
CPEB2	-0.134947286	4.636990922	1.58275857	0.208363791	0.436100304
CD34	-0.001737597	4.637563643	0.000460847	0.982872859	0.992424069
CPEB4	-0.078288518	4.638289006	0.568761945	0.450751306	0.674143245

MIF4GD	-0.069690169	4.639081676	0.577293306	0.447375218	0.670925335
CHST11	-0.319592202	4.639338665	4.630934707	0.031400452	0.133374921
CPTP	-0.148612813	4.639370676	2.760046329	0.096645438	0.272638555
FAIM	0.627732771	4.639981372	34.91467251	3.44E-09	3.44E-07
MBIP	-0.074318181	4.640157383	0.757299122	0.384175079	0.61879414
FAIM2	-0.286456375	4.640325242	5.046781969	0.024671719	0.1139418
TMEM192	-0.030180593	4.64166421	0.138515604	0.709761185	0.849639454
SH2B2	0.044677158	4.641826363	0.233444044	0.628981797	0.800641717
NCKAP5L	0.068585844	4.642009383	0.585472778	0.444175127	0.669129516
TPCN2	0.091608645	4.642018796	1.035260939	0.308926219	0.546952974
LOC107055019	0.346203479	4.642286604	9.188847589	0.002434941	0.022856225
RFXANK	0.306048081	4.643315153	16.62770493	4.55E-05	0.001108745
FYCO1	-0.190365669	4.643330367	3.730869932	0.053415567	0.187466134
DIRC2	0.23784483	4.643761747	8.853456157	0.002925344	0.025951735
TIMM10	-0.012657137	4.644064358	0.011872653	0.913232866	0.960146113
SURF2	-0.184457079	4.64525849	4.549795097	0.032922686	0.137877599
LOC107054160	-0.158528001	4.645915097	1.325779792	0.249557387	0.483796749
PLEKHB1	0.269195308	4.647947061	8.40456208	0.003742805	0.031033114
METTL24	-0.043057879	4.648795149	0.249132535	0.61768655	0.793398483
ST3GAL5	0.075571593	4.649344928	0.523841309	0.469207673	0.688269654
RFX5	0.149957176	4.64981646	1.9858549	0.158774962	0.373901051
DOCK5	-0.083105932	4.650076321	0.735118484	0.391229103	0.624653104
MYOM3	-0.014447425	4.650102902	0.017520947	0.894694241	0.951827123
ADAP1	0.027249978	4.651477766	0.069847693	0.791558668	0.897601674
MRAS	0.111364404	4.651611013	1.531840319	0.215836456	0.444615533
WSCD2	0.237491155	4.651636525	3.552707009	0.059448391	0.20100369
MBD4	0.223943708	4.651717319	7.87089492	0.005023678	0.038292634
PCYT1B	0.087115573	4.651917695	0.53518096	0.464436784	0.685347258
SNAP47	0.500230956	4.652145282	30.27262675	3.75E-08	2.86E-06
CARF	-0.044545664	4.652318521	0.263211061	0.607922645	0.787414637
RGS3	0.073047543	4.653512651	0.718165789	0.396746223	0.629433501
LOC112532685	0.061652217	4.654471336	0.641573797	0.423141477	0.65260216

RELL1	0.158617709	4.655539129	3.424376717	0.064240488	0.211839125
ANAPC10	0.022526379	4.656354353	0.081521834	0.775245565	0.888893846
SOCS1L	-0.020097726	4.656370534	0.037013065	0.84743845	0.928742508
P4HA3	-0.296671996	4.656380149	2.149588467	0.14260811	0.350188994
TNFRSF9	-0.11342209	4.656404803	2.023669818	0.154864474	0.36787858
LOC101749147	-0.183114126	4.656596224	3.495537291	0.061534438	0.206078422
CHRM4	-0.35074147	4.656665417	8.43004524	0.003690712	0.030780872
MAD2L2	0.001151219	4.657245277	0.000236227	0.987737255	0.994384615
AXDND1	-0.004380977	4.657407977	0.002669375	0.958794835	0.982278445
PIH1D3	0.851405022	4.657720923	115.293415	6.79E-27	9.31E-24
VPS9D1	-0.049208791	4.657774527	0.269422962	0.603719117	0.784815774
SENP8	0.086897403	4.657968958	1.187489882	0.275836462	0.510915379
RFKL	0.217425706	4.658855025	4.089012318	0.043162858	0.163790274
WDR86	-0.011504823	4.658913145	0.023569094	0.877986414	0.945006962
LONRF3	0.216732493	4.659003473	4.182046404	0.040854342	0.158588865
FIGNL1	0.275111274	4.659173439	10.08524102	0.001494616	0.016148331
LACTB	-0.039507164	4.659902481	0.22289173	0.636845344	0.805479642
UBE2B	0.119857126	4.65990334	1.693962498	0.193079447	0.417328557
ADA	0.136824765	4.661017593	0.891464863	0.345080653	0.581852868
GGCX	0.101638614	4.661026452	1.200752393	0.273171351	0.507798755
LOC420294	-0.297669033	4.661430657	13.60992788	0.000224993	0.003934381
TMEM56	0.101383643	4.661563599	1.629131701	0.201823189	0.428682144
TRAF6	0.049606414	4.661618856	0.412935547	0.520483286	0.725779864
DNAJC5	0.117299095	4.661845589	1.273004423	0.259203808	0.493771551
RAB22A	-0.099852483	4.662182735	0.890360957	0.345379511	0.582114478
CPED1	-0.029182515	4.663674624	0.066677706	0.796236918	0.900610289
LGALSL	-0.212513734	4.663887819	8.414859805	0.003721665	0.030976225
TXNRD2	0.129397475	4.664031173	1.8134416	0.178095928	0.398882296
ATP10D	0.080980192	4.664151239	0.598078668	0.439312042	0.664541243
TAF1A	-0.188609399	4.664217976	6.361119871	0.01166479	0.06925079
RBFOX3	0.134902424	4.664945934	2.350680913	0.125228297	0.321571728
TTC31	0.053089737	4.665261034	0.435930228	0.50909333	0.717892027

ALG10	-0.030325666	4.665312219	0.141097477	0.7071924	0.84841433
RBFOX1	-0.686146307	4.665708121	30.78687279	2.88E-08	2.26E-06
BPNT1	0.067050798	4.665940251	0.93164546	0.334435718	0.572087272
ISCA2	0.073557275	4.666142458	0.89123588	0.345142616	0.581886467
HAX1	0.012236878	4.666333683	0.014130198	0.905377946	0.956839699
SLC25A38	-0.202542223	4.667219783	4.246492802	0.039331477	0.154610477
ARHGAP24	-0.122900561	4.667966698	1.61817017	0.20334712	0.430265966
NKX6-2	0.165989752	4.66833676	1.78313544	0.181764783	0.403908104
CLDN5	0.174174163	4.668542208	3.044097958	0.081031121	0.24434068
C15H12ORF65	0.105354444	4.669493049	1.591129908	0.207164776	0.434475705
MAP3K13	0.062322323	4.669645781	0.465843825	0.494905443	0.706915718
CYB561D2	0.154985877	4.669961219	3.77091032	0.052151243	0.184468767
SIRT5	0.13949182	4.670868249	1.533583634	0.215575401	0.444540058
TBCEL	-0.335920634	4.670979765	15.48002246	8.34E-05	0.001803625
OFD1	-0.146876738	4.67100225	3.839541547	0.050057207	0.179873956
RGN	-0.324287258	4.671709098	9.445016223	0.002117236	0.020892566
LOC100858386	-0.612588261	4.671726874	52.1752576	5.08E-13	1.35E-10
RASGRP1	-0.20407054	4.671996417	2.566595756	0.109142376	0.294996353
FABP5	-0.069468809	4.67306214	0.296724371	0.585943271	0.772448047
METTL22	-0.134765655	4.673127156	2.229943382	0.135358808	0.33839038
GADD45A	-0.144776503	4.674138377	3.563284084	0.059070762	0.200249154
ABCC8	0.279473004	4.67455568	6.826045437	0.008983795	0.057520761
ARFGEF3	-0.215796394	4.67597613	3.201776216	0.073558341	0.229721483
KIAA1210	0.340263464	4.677247986	12.24602975	0.000466249	0.006874668
LRRCC1	-0.067413199	4.677762189	0.687416721	0.407044442	0.637801433
LOC112530217	-0.197290892	4.678072409	2.085661085	0.148687842	0.359237372
RAB18L	-0.109417417	4.678398053	1.789856463	0.180943652	0.403134632
C8H1ORF21	0.145815008	4.678544064	2.25165211	0.133471836	0.335412196
SLC25A44	-0.003322674	4.678616143	0.001826713	0.965908746	0.985879339
TPK1	0.404233354	4.678776468	18.669509	1.55E-05	0.00046423
DYNC1I1	-0.314484374	4.678827719	7.481367374	0.006234071	0.044717367
SLC25A32	-0.158671342	4.679564025	3.433554566	0.063884464	0.211088123

DHX35	-0.228457056	4.67993579	9.860548185	0.001688608	0.01768685
SLC35A5	-0.037781181	4.680281649	0.239614168	0.624484922	0.798314123
OLIG2	0.262598875	4.680488499	1.880308328	0.170299108	0.389184076
CDC42EP3	-0.132758049	4.681322228	1.714732532	0.190372498	0.41386966
CG-16	0.335825212	4.681597757	6.00473773	0.014267515	0.079368477
NFIL3	-0.039179549	4.682048188	0.14912819	0.699369887	0.843704655
CHST15	0.173265739	4.682073088	3.173840219	0.074826317	0.232841726
HIGD2A	0.031998028	4.682147154	0.04541963	0.831234288	0.919835926
TTC39C	-0.214049577	4.683032572	6.049368365	0.013911272	0.078259853
ENTPD4	0.058898865	4.683445537	0.656453605	0.417814753	0.647681748
BTN3A3L2	1.427246674	4.683559164	29.09217205	6.90E-08	4.92E-06
TDRP	-0.058910351	4.684029076	0.316452115	0.573747636	0.764869254
LOC101748080	0.018229641	4.684307159	0.043478344	0.834827173	0.921889882
SLC16A2	0.051647418	4.684380308	0.333948478	0.563343279	0.757599665
USP40	0.053283726	4.684532714	0.455524736	0.499722955	0.710528058
CSPP1	0.042490998	4.684985078	0.251490636	0.616027425	0.792054659
SUSD5	-0.553096346	4.685646877	15.85027076	6.86E-05	0.001547464
DZIP1	0.126569865	4.685812213	1.915538482	0.166349331	0.384172449
FAM78B	-0.098638923	4.686440099	1.524046385	0.21700818	0.446105303
TRAF4	-0.332056887	4.686511681	17.6066824	2.72E-05	0.000724426
UQCC3	0.0202668	4.686627529	0.058199408	0.809364822	0.908217277
FBXL21	-0.079710451	4.687897231	1.008624186	0.315232667	0.552589063
PCYOX1	-0.048803892	4.688388775	0.38456632	0.535169494	0.736860014
UBXN2A	0.094767205	4.688549893	0.820463752	0.36504461	0.600737409
ZC3H12C	-0.293151375	4.68866756	5.700664501	0.016958491	0.089511932
NOM1	0.53872899	4.688940945	40.50797546	1.96E-10	2.77E-08
BAIAP2	-0.379177076	4.68908487	13.06536035	0.000300807	0.004939905
AR	-0.441980308	4.689238353	8.581766316	0.003395458	0.028839264
UBAP2	0.431238502	4.689583107	0.843209508	0.35848046	0.594816575
ERI2	-0.279358432	4.689610915	5.29105984	0.021435171	0.103862114
ZC3H10	0.027300584	4.689621327	0.069484149	0.792089344	0.897745396
GPM6A	-0.310078979	4.690086239	1.998223219	0.157483719	0.372055685

TTC8	0.136397239	4.690218296	0.905108626	0.341415584	0.578399406
SESN2	-0.213467342	4.690632349	2.036393411	0.15357339	0.366186078
DGKQ	-0.14719263	4.690722707	1.809843095	0.178527053	0.399410287
ABTB2	-0.036548097	4.690726093	0.21049282	0.646381411	0.81149051
FAM120B	0.24000868	4.690852432	7.558591104	0.005972467	0.043260805
COL25A1	-0.336957729	4.690853445	10.33979503	0.001301925	0.014504516
TDH	0.144472049	4.691836378	0.701173068	0.402389807	0.634650123
MSRB1	-0.255618466	4.693689065	7.429240274	0.006417243	0.045692658
ADAMTS18	-0.151841409	4.693797481	1.70898359	0.191117298	0.415159753
LOC770617	-0.668814522	4.694043787	40.50012363	1.97E-10	2.77E-08
KPNA5	-0.102235222	4.694367149	1.307605064	0.25282873	0.487382468
RXRA	-0.653331536	4.69465868	23.13040301	1.51E-06	6.73E-05
GULP1	0.08888531	4.694999209	1.097095134	0.294904486	0.531855909
PM20D2	-0.260758673	4.695656397	5.846621323	0.015606987	0.084449875
ARMC7	-0.072975462	4.696839751	0.618223534	0.431708199	0.658792396
DMXL2	-0.308342006	4.697905624	9.173001591	0.002456114	0.022963271
TMEM200B	0.296304078	4.699739226	6.94070723	0.008425579	0.05519224
CCDC51	-0.074067078	4.699993037	0.930013792	0.334859341	0.572181771
FAAH	0.238360228	4.700691009	8.189249905	0.004213934	0.033755944
ESCO1	-0.036243656	4.701336467	0.185198089	0.666943624	0.82382197
BSN	-0.00086852	4.701373773	5.90E-05	0.993870508	0.9973341
MXD4	-0.13654143	4.701439411	1.415533706	0.234139934	0.465994314
PANK4	0.008989115	4.701640284	0.011809354	0.913463563	0.960327302
MOCS3	-0.01462757	4.704140158	0.024198091	0.876381941	0.943959209
YRDC	0.027428085	4.704579784	0.122340375	0.726509897	0.859179567
MITD1	0.064088356	4.704649806	0.637616635	0.424575168	0.653238372
ACTR5	-0.126222673	4.704835793	2.452893552	0.117308444	0.308947308
KTI12	-0.035166806	4.705060172	0.214150955	0.643533201	0.809582479
UPRT	-0.052013633	4.705303751	0.456431889	0.499296273	0.710379765
TMEM204	-0.07296388	4.705369564	0.475060866	0.490668101	0.703969418
DUSP11RC1I	-0.035201586	4.705691706	0.14888463	0.699603532	0.843779396
SNX25	-0.114714685	4.705930961	1.6983618	0.192502357	0.416902644

CNP	0.346996522	4.706164043	15.03147782	0.000105733	0.002150598
TPGS2	-0.057129988	4.706214448	0.234645873	0.628100178	0.800200405
SGTB	-0.188159997	4.706220296	2.598336301	0.10697596	0.291149424
TOR2A	0.114760925	4.706326592	2.138871596	0.143607489	0.352012696
IRF8	-0.087122739	4.706783676	0.693498232	0.404977045	0.636313883
LOC107050563	-0.143663746	4.706965621	2.451314106	0.117426525	0.309111096
CZH9orf64	0.234736969	4.707205781	3.866098554	0.049270929	0.177718793
FRA10AC1	0.012048587	4.707675813	0.024139621	0.87653018	0.944020895
PARP12	-0.232709349	4.708409046	8.675157062	0.003225771	0.027819735
BAG4	-0.051996397	4.708772195	0.442250098	0.506038423	0.715735109
RTN4IP1	0.228826951	4.70929513	7.90831972	0.004920795	0.037854927
CCDC137	-0.087193524	4.709573147	0.663150207	0.41545	0.64572387
TLX2	-0.006599443	4.709905459	0.001585389	0.968239064	0.98693873
MRPL42	0.055096655	4.710629772	0.555439755	0.456103492	0.67858644
LOC107050951	-0.12154141	4.711362057	1.905526566	0.167461023	0.385409109
PPP1R17	-0.432315652	4.711365877	7.874759272	0.005012954	0.038284066
PSD	0.126919422	4.711870228	2.222048668	0.136052419	0.33940912
LOC107050845	-0.259134532	4.712000876	1.508852486	0.21931427	0.448737316
CETN3	0.216599997	4.71242051	4.909222575	0.026713662	0.12033214
MOB3B	-0.04695144	4.712715867	0.197816136	0.656489403	0.816612028
TMEM101	-0.118887039	4.71274426	2.107827528	0.146547235	0.355876056
MYL10	-0.3438159	4.712768102	1.985725129	0.158788573	0.373901051
FAM181B	0.040432843	4.712832078	0.099352227	0.752608376	0.874711882
FAM118B	0.118668956	4.713050229	1.930278198	0.164728012	0.381697263
SMYD2	0.036055799	4.713416828	0.194634127	0.659087339	0.818759034
LOC107057416	-0.312616005	4.713502132	8.581172436	0.003396566	0.028839264
USP2	-0.140186211	4.713673265	2.080805657	0.149161429	0.360101426
TRAK2	-0.217850958	4.713713342	5.903383668	0.015111826	0.082475987
SHPRH	-0.087408045	4.714287443	1.021636953	0.312131034	0.550023148
KIF27	0.105405263	4.71436668	0.655035153	0.418318212	0.648095865
MND1	0.116259046	4.714585694	1.514039576	0.218523716	0.447748459
INTS12	-0.186653122	4.714728759	4.395587022	0.036032053	0.146000354

COL9A1	-0.44638718	4.714920606	11.82940731	0.000583025	0.008108258
HTR3A	0.000353351	4.715016511	8.94E-06	0.997614521	0.998889311
NDUFAF7	-0.013927802	4.715287718	0.032999524	0.855851355	0.932839865
UBE2Z	-0.190991113	4.715560461	5.733397894	0.016645246	0.088439624
PDE8A	-0.043608763	4.715649638	0.292175355	0.588829872	0.774825713
ATP2A3	0.47361384	4.715831927	14.63372481	0.000130557	0.002545401
CYR61	0.760901367	4.716682485	23.34345448	1.36E-06	6.11E-05
GRAMD1C	-0.059617419	4.717144242	0.470342306	0.492829734	0.705297727
TAF10	0.221460869	4.717467979	5.650819925	0.017447212	0.091025327
APTX	0.379141815	4.717549348	9.340856574	0.002241008	0.021728227
HAPLN3	0.088179864	4.718590589	0.931271714	0.334532689	0.572087272
RNF207	0.563961692	4.719112308	27.59625526	1.49E-07	9.68E-06
REPS2	0.161647178	4.71932789	2.951984626	0.085772167	0.253162512
HEPH	0.032150018	4.719387519	0.124726005	0.723964227	0.857408332
LOC101750715	-0.316120755	4.719653215	13.29281098	0.000266426	0.004519624
OMA1	0.155933269	4.720095213	4.364869135	0.036687301	0.147493168
PCDH8	0.069983196	4.720430582	0.268602391	0.604270844	0.785137502
CANT1	0.041155879	4.720611634	0.166566371	0.683181588	0.83408169
C15H12orf43	-0.18017361	4.721007137	4.383018093	0.03629867	0.146647339
FAM117A	0.083198715	4.722389046	0.984494773	0.321091633	0.55845712
SMAD2W	0.386009697	4.723325518	0.347571037	0.555491338	0.752912164
GRIP2	-0.161803576	4.723473595	2.474810536	0.115683382	0.305948797
ABHD12B	0.046733665	4.724447691	0.376733925	0.539356389	0.740394542
ECHDC2	-0.025427423	4.725204463	0.101589385	0.749930583	0.873450435
SLC17A5	0.075792814	4.725655934	0.821548	0.364727955	0.600520163
SGSM2	-0.001659893	4.725746366	0.000369044	0.984673174	0.992968171
STX5	0.101617109	4.725923789	1.447203889	0.228977143	0.459770455
LOC107052122	0.049708205	4.726198677	0.375807349	0.539855658	0.740788063
MYO1E	-0.1171521	4.726646353	1.819363691	0.177389037	0.397946367
UBOX5	-0.007465699	4.7270896	0.007425285	0.931331204	0.969881443
TTLL1	-0.077031064	4.727552952	0.62722723	0.428374212	0.655955486
LOC107056280	0.105577904	4.727820922	1.092595857	0.295896766	0.5327113

PRR5	0.18656319	4.728472455	5.241449677	0.022054888	0.105774756
MPV17	0.044409348	4.728713436	0.295521574	0.58670371	0.773223761
NARS2	0.064223989	4.728967233	0.624576093	0.429351847	0.656780203
COX11	-0.13743698	4.729274571	2.800229924	0.09425079	0.268926088
HS6ST2	0.067446449	4.729539521	0.725224419	0.394435533	0.627445382
YPEL2	-0.050473302	4.729819124	0.224489008	0.635640602	0.80476003
GAS2L3	0.306014095	4.729855827	9.612781352	0.001932278	0.019614823
SYDE1	-0.074782675	4.730488095	0.980043334	0.322188108	0.559831607
LOC422901	-0.110677085	4.730685554	1.566353172	0.210737413	0.438511331
RNF121	0.148266815	4.731274769	3.72053552	0.05374713	0.188452808
ALAD	0.066227527	4.731598573	0.570055409	0.450236899	0.673574705
GTPBP8	0.153070883	4.732350224	1.97665847	0.159742873	0.374867224
SWT1	0.162910286	4.732520502	4.376703739	0.03643339	0.1469787
TCF7	-0.038461132	4.734733038	0.212247142	0.64501179	0.81068921
VWA5B2	0.080910396	4.735224389	1.046513556	0.306311415	0.544491124
BRAT1	0.082957826	4.735650094	1.104856219	0.293202832	0.530454672
TAF1B	0.01591248	4.736142999	0.03640962	0.848672064	0.929382324
NDUFS6	0.031149879	4.738410727	0.136915095	0.711367292	0.849891003
CBWD1	0.124077911	4.73869425	1.19832561	0.273656592	0.508471005
FAM175B	-0.068137143	4.739407902	0.709614609	0.399571782	0.632024769
SC5D	0.215501064	4.74011081	4.465787683	0.034580143	0.142174128
NEURL1B	0.024832871	4.74024912	0.055308762	0.814070465	0.911198524
TNNC2	-0.326775178	4.740859206	1.606389285	0.205000101	0.431807048
CCND3	0.018067677	4.741636759	0.021953715	0.882210295	0.947322527
SLC25A42	-0.141455526	4.743185212	2.225150924	0.135779389	0.338933531
PTER	-1.088031994	4.743326865	77.15228674	1.58E-18	1.04E-15
TMEM170A	-0.281782486	4.743558515	10.72582062	0.001056508	0.012516083
NTPCR	-0.069824658	4.743984571	0.641665947	0.423108177	0.65260216
NEPRO	0.234003526	4.744530473	8.448056698	0.003654337	0.030570471
GSTCD	0.145687904	4.745132326	4.067331304	0.043720296	0.164914059
CSAD	0.133626205	4.745216572	2.442273541	0.118104936	0.310350803
LPAR2	0.365236585	4.745676244	12.69078029	0.000367463	0.005742262

SHC2	0.322297779	4.746045168	8.083698767	0.004466519	0.035221456
TMEM26	-0.103474674	4.746144071	1.568487075	0.210426863	0.438234642
TMEM170B	0.031304226	4.746456878	0.1470567	0.7013641	0.845057207
SOX3	0.394807132	4.749323516	8.505630728	0.003540492	0.029769438
LRRN4	-0.168884472	4.749509965	1.500485624	0.220596656	0.450049422
SPSB1	-0.080027281	4.750319125	0.788248861	0.374630157	0.609264601
FGR	0.019122961	4.75043617	0.043735404	0.834346668	0.921720365
SNTA1	0.0486759	4.750516927	0.244375131	0.621063869	0.795609651
LDLRAD4	-0.168971404	4.751880451	3.119212116	0.077374187	0.237684649
CTC1	0.004908963	4.751998087	0.003118809	0.955464251	0.981041173
SARDH	-0.116032215	4.752732459	1.769060646	0.183498374	0.405896726
PUSL1	-0.098545987	4.75285112	0.733063981	0.391891832	0.625468487
ORMDL2	-0.082070477	4.755048796	0.852898877	0.355733551	0.592239624
ACHE	-1.326534545	4.755177706	82.39395684	1.11E-19	8.74E-17
MRPL49	0.341173604	4.7556867	16.33090204	5.32E-05	0.001258152
SYT9	-0.220819038	4.755999809	3.592799159	0.05803042	0.198151184
H6PD	-0.061110646	4.756071566	0.605331768	0.436550916	0.662740596
ISPD	-0.408408695	4.757158427	19.167767	1.20E-05	0.000374517
RPP30	-0.047886403	4.757625033	0.405574036	0.524224541	0.728803213
DONSON	-0.031760394	4.757728834	0.150704136	0.697863367	0.842758423
FZD8	0.144404497	4.757793636	1.409789701	0.235091307	0.467274603
LOC100859848	0.055151141	4.758064503	0.222088458	0.637453205	0.805814895
ZDHHC15	0.142899816	4.758408476	2.759417544	0.096683432	0.272657543
ZCCHC9	0.153669404	4.758428866	2.786026036	0.095089774	0.270148867
POLQ	-0.201060373	4.758856178	4.057677545	0.043970935	0.165511342
ANGPTL2	-0.031603145	4.75918306	0.129873583	0.718563149	0.854609585
LOC101747860	0.106043595	4.760084944	0.818684764	0.365564991	0.600933161
RUSC2	-0.363920105	4.760571229	4.652593429	0.031006668	0.13235142
ASB7	-0.145428082	4.76111982	3.550963627	0.05951088	0.201127971
NABP2	0.011666725	4.761374117	0.024130253	0.87655395	0.944020895
RERGL	-0.499061149	4.761705468	12.81076616	0.00034463	0.00545803
MED4	0.070912301	4.762525172	0.700515019	0.402610695	0.634696204

ZBED1	-0.194074268	4.762551737	7.223489491	0.007195569	0.049191149
RNF113A	0.116252823	4.76305119	2.39623324	0.121627799	0.315675937
LOC419389	-0.386361292	4.763182358	2.411043675	0.120482017	0.313840682
RPUSD4	0.010840218	4.763882649	0.017906304	0.893549309	0.951501845
MRPL43	-0.323144795	4.764115329	7.938138217	0.004840361	0.037393492
EMP2	-0.150659964	4.764693036	2.175625788	0.140212472	0.346529922
LOC107052306	-0.014501783	4.764867371	0.010309617	0.919124758	0.962650238
LOC101749023	0.060160509	4.764935242	0.576494419	0.447689687	0.671045162
SLC35B3	0.098793531	4.765355809	1.727843824	0.188686495	0.412324268
EHD4	0.238547374	4.765488937	9.241502579	0.002365907	0.022516487
FAM189A1	-0.014149564	4.767363307	0.038193869	0.845054362	0.927271908
GRK5	0.031290541	4.768736186	0.108693102	0.741636954	0.868957283
MED21	0.022673063	4.769285549	0.107959735	0.742479013	0.8693249
F2R	-0.172409538	4.769535815	2.504300675	0.113535874	0.302406036
PRSS35	0.358519292	4.770943991	8.41002283	0.00373158	0.030996035
KYAT3	-0.069785069	4.771287521	0.693843199	0.404860234	0.636231034
BEND7	-0.092346825	4.772690179	1.758479682	0.18481426	0.407359715
LOC107052100	0.232943531	4.772826051	4.504004564	0.033815573	0.14005418
CLC2DL2	0.225606245	4.773157959	4.09693476	0.042961034	0.163356737
IDUA	-0.151940339	4.773328539	2.074686503	0.149760708	0.361071422
G6PD	0.205758808	4.773905006	8.164330023	0.004272225	0.034026847
LOC107050176	-0.07165505	4.775987065	0.674566588	0.411464003	0.641584399
RAD17	0.066055014	4.77610185	0.457399486	0.498841841	0.710180814
ARID3C	0.019122372	4.77711433	0.028418564	0.866128535	0.938134876
ATP8A2	0.038839794	4.777176236	0.087337044	0.767590249	0.884179034
SNN	0.217205412	4.777338537	9.230222044	0.002380528	0.022564277
LRRC41	-0.12990974	4.777612685	2.928428704	0.087032161	0.25573468
HACD1	-0.071582468	4.778088496	0.72625209	0.394100736	0.627342312
TOX3	0.056872445	4.779060242	0.289427956	0.590587343	0.776205769
HSD11B1L	0.439285084	4.779150502	29.69964806	5.04E-08	3.74E-06
PECR	-0.345558659	4.779322902	20.54428263	5.83E-06	0.000210262
CRIP2	0.20634546	4.780480427	5.720075096	0.016772013	0.088877503

CAB39L	0.008848572	4.780866787	0.007974179	0.92884488	0.968392732
DCP1B	0.193406792	4.780928416	5.591512762	0.018047703	0.092862713
ST3GAL1	-0.094969928	4.781034126	0.844607293	0.358082399	0.594395831
TBC1D20	-0.01314695	4.781039137	0.033338366	0.855121327	0.932526733
TAB1	-0.031076389	4.78146046	0.141599044	0.706696492	0.848162533
NAA38	-0.167705031	4.781640185	4.881585334	0.027144677	0.121442539
FAM96A	-0.070040715	4.781748588	0.74952548	0.386626508	0.620979506
SCLY	0.16848996	4.781920963	4.350380087	0.037000688	0.148282103
LOC426385	0.524112678	4.781956848	34.63906744	3.97E-09	3.89E-07
EPHX2	0.406614491	4.782127571	19.1248361	1.22E-05	0.000379729
GAS8	0.138769862	4.782168882	2.962218862	0.085230906	0.25228531
OTOR	-0.085670266	4.78222662	0.688181453	0.406783626	0.637791764
LOC107051524	0.052863004	4.782754159	0.501595193	0.478800045	0.694950582
DUSP4	0.013452283	4.782796758	0.020889452	0.885080519	0.9486918
RIMS1	-0.218423263	4.782877506	4.193862012	0.040570574	0.15785973
COMMD9	0.014995349	4.783199162	0.036975319	0.847515305	0.928742508
KLHL3	-0.089422781	4.783274712	1.481249667	0.223579103	0.452966527
FAM188B	-0.059992021	4.783652544	0.600091183	0.438543238	0.664069883
FSTL4	-0.02567161	4.784129614	0.032913222	0.856037909	0.932863069
CAMLG	0.064985802	4.784478546	0.70843639	0.399963382	0.632401023
LOC112530285	-0.058022955	4.784585515	0.629990682	0.427358732	0.65507107
TGFBR2	0.333416252	4.785317399	14.42067247	0.000146189	0.002784082
WRAP73	-0.067271273	4.786115155	0.826760749	0.363210868	0.599097263
PPP1R12B	-0.134868592	4.786223098	3.511991233	0.060926188	0.204641849
BMPER	0.20306246	4.786318652	1.671943226	0.195998457	0.420708925
EEF1AKMT1	-0.201849644	4.786433622	2.908834497	0.088095517	0.257571382
LYSMD2	-0.057211654	4.787963759	0.469871157	0.493046449	0.705538967
PTHRL	0.120743963	4.788135394	0.555504972	0.456077049	0.67858644
MANBAL17	-0.173422215	4.788143392	3.172865705	0.07487097	0.232872277
NFX1	-0.19301278	4.789076342	3.11767802	0.077447071	0.237687384
TTC7A	0.145666975	4.789119384	3.735787664	0.053258551	0.18719099
MFSD5	0.011598198	4.789257845	0.018509306	0.891782615	0.951008202

RWDD4	0.02855921	4.790119612	0.119205573	0.729897711	0.861397607
TM4SF18	0.211855484	4.790126493	7.027641876	0.008026097	0.053336266
AGMAT	-0.012078647	4.790755177	0.0313402	0.859483541	0.934196556
DNAJC25	-0.010577574	4.790851455	0.009717199	0.921475043	0.964252581
LOC101747373	-0.961898549	4.790904562	72.9961366	1.30E-17	7.13E-15
APBA1	-0.165178772	4.790974469	3.583173204	0.058367558	0.19889328
LOC107053182	0.074368889	4.791265127	0.532964479	0.465363191	0.685976109
NDUFA7	0.124288384	4.79182358	1.868911983	0.171599743	0.390333671
BCHE	0.007381473	4.79280007	0.005861588	0.938972778	0.973646548
SIVA1	0.217186404	4.793005247	8.558400211	0.003439315	0.029112105
NECAP2	0.06556303	4.793040074	0.7905636	0.37392976	0.608644099
EFCAB14	-0.074871262	4.793110058	1.034749033	0.30904586	0.546988236
VMP1	-0.00133916	4.793646749	0.000356942	0.984926542	0.992992257
TMSB4Y	-0.248275335	4.793710231	3.946912689	0.046957443	0.172512778
MIER3	-0.008019235	4.794568313	0.006144231	0.937521687	0.97293891
ASMTL	-0.172794324	4.794696299	5.100429535	0.023919919	0.111825554
SDCCAG8	0.299752738	4.794838519	14.34327938	0.000152322	0.002862918
TRIP4	0.190763927	4.795209579	7.027776519	0.008025494	0.053336266
HMGCL	0.065725874	4.795780472	0.946596322	0.33058719	0.568364039
NACAD	-0.482813943	4.79645624	12.82646386	0.000341751	0.005438601
KIAA0895	-0.108831436	4.796638387	1.206675372	0.271991557	0.506750574
TEX2	-0.091468189	4.798450462	1.208708815	0.271587989	0.506496255
PKN3	0.088733649	4.798649988	1.272742831	0.259252757	0.493807629
FNIP1	-0.044976741	4.799269604	0.236397545	0.626820192	0.799662182
LOC112531361	0.060164942	4.799290784	0.435395337	0.509353344	0.717892027
FAM84B	-0.405960437	4.799419467	9.75641246	0.001786983	0.018493587
GAS2L1	0.02137228	4.800278201	0.07279645	0.787307834	0.895403091
FBXO18	0.109672804	4.800326201	1.156672467	0.282156712	0.517783055
TIMM9	0.0788566	4.801164532	0.943085945	0.331485477	0.569313592
ZNF148	0.097659254	4.802112362	1.616857665	0.203530499	0.430292477
EDEM1	0.025769863	4.802461016	0.102612782	0.748716441	0.873168892
RAD51AP1	0.084325845	4.80250041	0.722884214	0.395199461	0.627945842

CCNY	-0.109233123	4.803406796	1.204180998	0.27248763	0.507272763
C3H6orf120	0.177581152	4.804285646	5.585514493	0.018109613	0.092928514
ALG5	0.007080308	4.805059284	0.009941358	0.920577453	0.963681022
LOC422757	-0.471891803	4.805389419	14.38267352	0.000149169	0.002821343
ENSA	-0.16481245	4.805821691	4.900611009	0.026847195	0.120706681
GLMN	0.200404271	4.805876136	6.723255987	0.009516374	0.059900802
C8H14ORF80	0.226783377	4.806255593	4.070327498	0.043642812	0.164824988
CECR5L	-0.211761637	4.806866855	6.127565526	0.013309001	0.076018726
C8H1orf52	-0.034492568	4.80696871	0.165078522	0.684523242	0.835303382
CCDC58	0.264678593	4.807648658	7.635897856	0.00572179	0.041901223
KLHL4	0.082382575	4.808025614	0.624785817	0.429274386	0.656722762
TSEN2	0.036439591	4.808238595	0.141971945	0.706328449	0.848057981
ELMOD1	-0.008442745	4.808623389	0.004597994	0.945938067	0.977051716
INTU	0.284223799	4.809819109	10.56475605	0.001152642	0.013292521
LOC107057631	0.243884627	4.810238639	6.533517577	0.010586041	0.064516038
MYH1D	-1.763680459	4.811090643	19.51310059	9.99E-06	0.000324271
DENND1B	-0.11083844	4.811479098	1.112876629	0.291457487	0.5287105
CEP63	-0.157179287	4.811516065	4.809058511	0.028310508	0.124748861
CIB1	-0.100294127	4.811752837	1.027826064	0.31066981	0.54838372
PACS2	0.142673173	4.811960266	2.936919306	0.086575709	0.254939744
LGALS8	0.202607165	4.812949164	6.447167593	0.011112937	0.066860676
MYCBPAP	0.284867319	4.813732328	7.853394523	0.005072538	0.038571445
B3GAT1L	0.076745954	4.814239082	0.360489289	0.548234622	0.747425593
PKIG	0.064786008	4.814431455	0.55841051	0.454901382	0.67768554
ABHD17B	0.130020566	4.81458125	1.779152589	0.182253418	0.404493483
CRIM1	0.065112785	4.815092674	0.300601342	0.583505679	0.770936649
ARHGAP39L	0.407704958	4.815287323	18.39244197	1.80E-05	0.000517152
DDX55	0.044181057	4.815382904	0.389990845	0.532304214	0.734636068
GPRC5C	-0.053544204	4.815388836	0.268203882	0.604539176	0.785137502
PRMT8	-0.246223126	4.815752613	7.773688078	0.00530127	0.039832147
KLF7	-0.072043835	4.815907594	0.611539974	0.434208571	0.660769633
PLPPR3	-0.008478219	4.816327915	0.006023904	0.938135257	0.973257994

CTDNEP1	0.059022311	4.817224233	0.426003619	0.513956455	0.721016241
FUCA1	-0.134312275	4.818128682	2.297096421	0.129616098	0.329223095
IFT81	0.84391235	4.818193612	104.6086151	1.49E-24	1.63E-21
MMS22L	0.093802111	4.818285964	1.449706971	0.228574977	0.459299212
AVL9	-0.273707478	4.818454765	8.872280897	0.002895332	0.025801375
DOCK4	0.081650193	4.818984459	0.622388361	0.430161138	0.657590258
DNAAF5	0.134660262	4.819347314	2.522768503	0.112213422	0.299960737
TPCN3	0.021322794	4.81943786	0.06786036	0.794477953	0.899424473
ITGA2	-0.115307383	4.821624711	0.841553022	0.358952983	0.595330143
TWISTNB	0.208083663	4.821643773	3.415035451	0.064605026	0.212530128
SERPING1	-0.043113489	4.821732495	0.204180642	0.651367306	0.813961802
CEP83	0.226543319	4.822232471	8.40399268	0.003743978	0.031033114
NUBPL	-0.142752743	4.822325108	2.921847485	0.087387758	0.256233851
C2H18ORF8	0.226326916	4.82235162	8.185095695	0.004223595	0.033786707
HEMK1	-0.397766262	4.822361068	14.23280982	0.00016153	0.002999964
BOLA3	0.052503663	4.822557373	0.347583333	0.555484345	0.752912164
NRXN1	-0.016272314	4.82267552	0.016352949	0.898245009	0.953465464
EPHA2	-0.201218295	4.824198319	5.531714527	0.018674797	0.094792905
LOC112532089	0.210666398	4.824287943	5.22318582	0.022287684	0.106642581
GUCD1	-0.147532353	4.824320148	4.088080613	0.043186658	0.163790274
ACBD5	-0.397223398	4.824529407	19.49470016	1.01E-05	0.000326765
ZBTB48	-0.050673037	4.824759508	0.530536274	0.466381486	0.686431785
KLHL29	0.174985647	4.825388271	2.332982361	0.126659006	0.32408887
CASKIN1	-0.290011849	4.825390799	4.618422278	0.031630319	0.134015893
CEP95	-0.047015414	4.825557115	0.271666436	0.602216097	0.783542807
NMRK2	0.313767789	4.825571663	15.36899812	8.84E-05	0.001879937
POC1A	0.158402308	4.825650947	5.145624082	0.023305032	0.109817956
RHBDF1	-0.22832785	4.826351473	5.560009971	0.01837531	0.093729893
AMOTL1	0.138965636	4.826438299	3.708012053	0.05415185	0.189347364
LOC101751579	0.221310759	4.826739461	0.969057407	0.324915371	0.562965403
TEK	0.134102356	4.826798808	1.227661575	0.267862288	0.503053957
DBT	0.164025736	4.826810543	3.47823141	0.062181163	0.207585929

KIAA0556	0.026871236	4.826845749	0.104881412	0.746048572	0.871493846
ZNF853	0.020599279	4.827249375	0.051487631	0.820494612	0.91577283
PIN4	0.054324987	4.827377426	0.393461949	0.530485245	0.733450191
CDK2AP1	-0.028894093	4.827560596	0.12789665	0.720622911	0.855792151
CSMD2	-0.092121009	4.827853094	0.625530992	0.42899933	0.656424026
GYG1	0.18332986	4.828520231	4.508997092	0.033717005	0.140010949
BORL1	-0.158277921	4.828651766	1.246643638	0.264194494	0.499384748
CFAP97	0.06588655	4.828984633	0.641123328	0.423304316	0.652625553
BICD1	-0.046941922	4.829446171	0.330412327	0.565416396	0.759194353
DDX28	0.043516892	4.830314437	0.168940333	0.68105529	0.83296899
CHCHD1	0.147119466	4.830762328	3.94071937	0.047130619	0.172801769
PFKFB2	-0.079916971	4.830918507	0.738821742	0.390038578	0.623902478
GFOD1	0.056669457	4.831477747	0.578484767	0.446906858	0.670621395
C3H6ORF203	0.215537412	4.831581073	7.401919907	0.00651543	0.046066172
C21H1orf174	0.014982959	4.831689107	0.031844368	0.858369661	0.933836045
OSGEPL1	0.300683115	4.831701461	14.9703438	0.000109214	0.002186276
CTSO	0.014166857	4.832163529	0.033412155	0.854962857	0.932526733
TRAF3	0.067546876	4.83318974	0.788919556	0.374427028	0.609054641
SSFA2	-0.08880605	4.834081226	1.251089758	0.263344441	0.498485308
PRKAR2B	-0.051458346	4.834176104	0.304558067	0.581038932	0.769992399
CPLX2	0.262052838	4.834556083	5.10833257	0.023811194	0.111532365
CHDB1	-0.638648951	4.834664361	0.943703515	0.331327209	0.569160583
PLA2G10	-0.261022909	4.834707786	9.691285238	0.00185144	0.018993419
PHF2	-0.171093039	4.834902365	5.113220713	0.023744203	0.111345291
SLC22A23	0.35712262	4.83495072	16.40440236	5.12E-05	0.001214922
ITPRIP	-0.05100521	4.835192165	0.312641472	0.576063778	0.765928367
RNF19AL	-0.095920036	4.836176052	1.074890403	0.299843426	0.537171865
LOC112532770	-0.947532555	4.836247873	44.40346938	2.67E-11	4.68E-09
TGFB1	0.30747196	4.836465255	15.26530498	9.34E-05	0.001960678
SNX24	0.023767343	4.837383734	0.046419763	0.829414572	0.918867551
L2HGDH	-0.251362327	4.837805328	9.460571224	0.002099357	0.020835945
NPRL2	-0.062333533	4.838149733	0.582848912	0.4451978	0.669934138

DNAH17	-0.060753215	4.838673765	0.236354515	0.626851564	0.799662182
GPR137C	-0.026447189	4.839445856	0.076119855	0.782626204	0.892887345
STRIP2	0.168806339	4.839787589	4.487317823	0.034147213	0.14096648
ALG6	-0.046712525	4.839886626	0.469509495	0.49321291	0.705551458
BRI3	0.137351698	4.840124529	3.076024094	0.079454538	0.241266734
UNC119	-0.080638174	4.840282374	0.83439228	0.361005523	0.597476225
CHGA	-0.259685113	4.840282638	1.761401439	0.184449809	0.407016441
KATNAL1	0.280542677	4.840506408	15.03452624	0.000105562	0.002149784
MSX1	-0.29415541	4.840663738	1.824196985	0.176814509	0.397085792
BCS1L	-0.219343387	4.841993619	7.432901551	0.0064042	0.045619527
CEP112	0.129016383	4.842457386	1.885322024	0.169730496	0.388282401
DAZAP2	0.158569959	4.842486619	2.893075179	0.088960973	0.259520542
STK39	0.078330765	4.842609646	1.234407707	0.266551553	0.50184277
DCXR	-0.012486094	4.842686707	0.018343512	0.892265404	0.951106253
ATP6V1H	0.067964194	4.843080748	0.856615068	0.354687678	0.591325809
SLC35A4	0.018164252	4.843159596	0.060684811	0.805416824	0.905763658
ELOF1	0.001455809	4.843565224	0.000173658	0.989485821	0.995074915
CEP162	0.221199931	4.844038592	5.958005094	0.014650609	0.08076448
GALNT16	-0.075660132	4.844932274	0.598295377	0.439229157	0.664523999
NPRL3	0.117071223	4.84501084	1.903963987	0.167635293	0.385472156
ALX1	-0.058092091	4.845367255	0.172556781	0.67784944	0.830529601
HSF1	0.022553499	4.846383028	0.062227103	0.803009922	0.904416719
NAA60	-0.192302756	4.84653615	5.996819126	0.014331695	0.07959097
CABLES1	0.008818145	4.846620963	0.010209434	0.919517322	0.962786027
MTRF1	0.157564026	4.846968137	4.633090207	0.03136103	0.133294601
RD3	-0.221749853	4.847122198	9.169331796	0.002461044	0.022983247
PDCD2L	0.0749761	4.847340656	0.720697703	0.395915145	0.628718753
RFNG	-0.079675255	4.84734348	1.115812557	0.290821909	0.527918949
MGAT1	-0.060291842	4.847421972	0.431411886	0.511296969	0.718970401
DDHD2	-0.088885489	4.847725724	0.904324411	0.341624818	0.578575173
AMT	0.139806627	4.847816226	2.421647135	0.119669043	0.312514538
SORBS2	-0.475215769	4.84851823	18.52476562	1.68E-05	0.000489296

PAM16	-0.00192454	4.848768634	0.000522566	0.981762181	0.991828264
GADD45GIP1	0.127022784	4.848894462	1.302905795	0.253683128	0.488228757
STMN3	-0.256706181	4.848991581	1.234944379	0.266447625	0.501813162
LOC112530486	0.31894619	4.849047458	0.179717199	0.671616388	0.826344165
BNC2	0.002936515	4.85056791	0.000708309	0.978767543	0.990993103
SRCIN1	0.079504967	4.85067451	0.438001849	0.508088458	0.717339817
HAGHL	0.215470833	4.851427376	4.081760887	0.043348462	0.164249407
C1H12orf29	0.27881817	4.851592037	13.45244392	0.000244687	0.004224899
CHORDC1	0.223068194	4.852238975	6.076864074	0.013696369	0.077474649
COX15	0.102724398	4.852606569	1.428651486	0.231984643	0.463558154
C1H21ORF91	0.058645291	4.853277672	0.502334567	0.478476126	0.694655901
IPO4	0.086449732	4.853354497	1.013413041	0.314086566	0.551873406
TMEM144	0.150181618	4.853821437	2.144263741	0.143103674	0.351069729
C1H12ORF73	0.018746438	4.853931302	0.050282147	0.822573043	0.916456624
DNAJC12	-0.297388902	4.85394515	15.01860022	0.000106457	0.002155577
ALKBH1	-0.590295558	4.854734364	73.74060562	8.91E-18	5.05E-15
CLK3	-0.12400516	4.856385284	2.446479571	0.117788775	0.309816862
SYNPR	-0.258762378	4.85790411	4.539602884	0.033119274	0.138424603
APRT	0.176476858	4.858505659	4.392077943	0.036106282	0.146157165
ENDOG	-0.073581614	4.858785037	0.835773464	0.360608367	0.597143069
RGP1	0.111898711	4.858894222	1.236402512	0.266165506	0.501460201
CTIF	0.124407702	4.859550317	1.970799101	0.160363067	0.375840233
NRSN1	-0.029617132	4.859639883	0.048860579	0.825057946	0.917552618
ZCCHC10	0.020205587	4.860211327	0.074599454	0.78475405	0.894316745
LOC107052649	-0.09969242	4.860585965	1.229057371	0.267590435	0.503053957
TRMU	-0.770493894	4.861634015	79.30744896	5.32E-19	3.80E-16
MED18	0.228891411	4.861678222	5.420973552	0.019896263	0.098820708
CDK10	0.032348937	4.861753003	0.154002943	0.694738982	0.840768548
PLA2G7	0.253300958	4.863451705	6.447162049	0.011112972	0.066860676
TIRAP	-0.107001587	4.865110825	1.87000036	0.171475038	0.390103934
PLCB4	-0.009723245	4.865270318	0.013585263	0.907212036	0.957480331
FAM217B	-0.039116471	4.865719859	0.201423446	0.653574326	0.815062002

USP21	0.276634794	4.865949991	11.29001008	0.000779253	0.009994233
PARP4	-0.198374626	4.867102346	2.841826702	0.091839753	0.264848079
BCL11A	-0.271655998	4.867396514	1.85604564	0.173081885	0.392403198
HAND2	-0.160511609	4.86743261	0.297445969	0.585488017	0.772342899
TMEM135	0.287019455	4.867872519	10.81794488	0.00100521	0.01206472
TANK	0.038036121	4.867933315	0.278159678	0.597909973	0.780841953
CZH9ORF40	0.062923978	4.869631698	0.508556277	0.475764501	0.692805741
LOC112530083	-0.040534526	4.869757555	0.204731711	0.650928351	0.813770371
RAB7A	0.096195227	4.870531105	1.623311866	0.202630613	0.429233617
ITPKA	0.179293311	4.870723299	5.522681044	0.018771468	0.095070641
TGFBR3	0.155277386	4.871155115	2.786710078	0.095049184	0.27008018
NAT9	-0.274103922	4.871439536	14.05899688	0.000177164	0.003239391
C2CD3	0.027741436	4.871762165	0.117857105	0.731370354	0.862328105
TAMM41	-0.05640009	4.873795304	0.74862143	0.386913045	0.621136991
METTL2A	0.134239387	4.873911043	2.8398026	0.09195551	0.26504255
ATPAF2	-0.211877732	4.874096348	6.586306678	0.010276616	0.063191969
SMPD1	-0.028954929	4.874151835	0.104249435	0.746788539	0.872136651
IFT57	-0.378988147	4.874495338	12.86208528	0.000335307	0.005346388
ACSS1	0.182974319	4.875172573	1.244631034	0.264580404	0.499789983
MUT	0.49210209	4.875456054	20.99622297	4.60E-06	0.0001721
MAPK8IP1	-0.115322134	4.875696229	2.41023274	0.120544443	0.31390391
C8H1orf27	-0.04745967	4.876331022	0.408950666	0.522502587	0.727331026
RASSF2	0.05552488	4.876574555	0.226289313	0.634289002	0.80403489
LOC112531893	-1.132635026	4.876708938	67.93866485	1.69E-16	8.41E-14
EDNRB	-0.360695601	4.87683259	10.5301463	0.001174429	0.013454499
PMVK	-0.296730099	4.877379705	11.98002311	0.000537739	0.007661037
DBX2	0.228803508	4.877732285	4.346856629	0.03707732	0.14840849
TMEM64	-0.146376834	4.877781418	3.483137188	0.061997101	0.207139553
DOCK11	0.026242742	4.877810283	0.073145149	0.78681131	0.895117541
FAM65C	0.118159705	4.877860623	2.075080424	0.149722048	0.36103111
USP49	0.115828537	4.877888394	1.900387457	0.168034957	0.386069383
ATP9B	-0.03466515	4.877962459	0.208723565	0.647769705	0.812118133

TRDMT1	-0.107568056	4.878038456	1.76378398	0.184153235	0.406794321
TMEM223	-0.055335674	4.87874756	0.563633301	0.452800023	0.675632701
HACL1	-0.049419971	4.878848949	0.42134246	0.516267942	0.72250289
SLC25A33	0.01722634	4.879066563	0.035613354	0.850316189	0.930439746
SLC16A14	-0.40732998	4.879621553	9.792871026	0.001751897	0.018199159
TMEM108	0.285485801	4.880167101	5.699556144	0.016969204	0.089524927
PCOLCE	0.029042483	4.882064597	0.126939164	0.721626971	0.856304305
RPP40	0.121295445	4.882783009	2.661397614	0.102810362	0.283723182
ENTPD6	0.055135637	4.88286981	0.445572306	0.50444513	0.715018056
EEF2K	-0.02538343	4.883174914	0.061624645	0.803946308	0.905174561
LOC112531384	0.122909656	4.883340614	1.45771931	0.227293351	0.457058792
HELB	-0.429645425	4.884004499	33.78608187	6.15E-09	5.69E-07
VTI1B	0.111508071	4.884421698	1.921653433	0.165674501	0.383266403
CCDC9	0.185930611	4.884841837	5.785599637	0.016157985	0.086690461
MAX	0.037035219	4.885281318	0.278175412	0.597899618	0.780841953
ASB3	0.009763575	4.885374222	0.017880533	0.893625485	0.951501845
TRAPPC12	0.465441095	4.886111273	18.38055261	1.81E-05	0.000519481
MYL1	-0.222236647	4.886413648	1.625202185	0.20236794	0.429146995
HAUS1	0.399437856	4.886550267	10.64963438	0.001100927	0.012866299
NUAK2	0.045088651	4.887015409	0.125499354	0.723144881	0.857066913
POLR1D	0.053356652	4.887253629	0.502291006	0.4784952	0.694655901
ARPC4-TTLL3	0.085965099	4.887259052	0.596499649	0.439916694	0.664967091
GAP43	-0.232000009	4.887520648	1.727900234	0.188679279	0.412324268
AEBP1	-0.149778202	4.887655293	1.006442155	0.3157567	0.553296004
DENND4C	-0.133892286	4.888226654	1.532806317	0.215691755	0.444540058
ZDHHC16	0.125483086	4.888826035	3.257356731	0.071103656	0.225216689
DCAF4	-0.036848406	4.889837583	0.225895202	0.634584321	0.804224916
CLOCK	-0.066867551	4.890193227	0.578287513	0.446984345	0.670631111
NPNT	-0.37139174	4.891184963	9.552615228	0.001996647	0.020148057
RPGRIP1L	0.010540624	4.891574173	0.014816578	0.903118107	0.955679
PPP1R26	0.094420285	4.891729218	1.049445106	0.305634918	0.543817321
ASIC1	0.15630279	4.891832908	1.94310153	0.16333217	0.379877153

KLF11	-0.017258718	4.892408506	0.023678186	0.87770658	0.944829372
RERG	-0.151001814	4.893117283	3.207509167	0.073300993	0.229178766
PNPLA6	0.291896272	4.893148567	7.575411175	0.005916988	0.042948407
UFC1	-0.006642745	4.893261521	0.004486799	0.946594781	0.977439625
COMMD8	0.15126554	4.893391695	3.822305833	0.050574593	0.181174895
CYP26A1	-0.548256862	4.893461007	9.308491639	0.002280942	0.021936235
PDXP	-0.05968214	4.894078329	0.576597668	0.447649025	0.671045162
PORCN	0.048222144	4.894080897	0.351801393	0.553095172	0.751418515
MPV17L2	0.219296453	4.894246599	7.706184898	0.005503194	0.040753849
ARHGAP10	-0.055906596	4.894257014	0.398823024	0.527697723	0.731483329
ETV6	0.213162172	4.894336049	5.809476144	0.015940042	0.085828988
WBP1L	0.014545377	4.894484769	0.039317234	0.842821509	0.926056886
C19orf70	0.105479438	4.894715976	1.482454948	0.223390818	0.452808069
NFKB2	0.178258988	4.894771026	3.372719171	0.066284268	0.216067281
ISM2	-0.336854043	4.895923239	11.13391455	0.000847635	0.010647204
PNPO	0.079960457	4.897114442	0.863828473	0.352669534	0.589214862
SCAPER	-0.126643702	4.897130689	1.387131102	0.238890186	0.47173143
GID4	-0.198971496	4.897313046	5.644453239	0.017510676	0.091211829
ACAA1	0.121322936	4.897930706	1.891733793	0.169006497	0.387488073
LOC107050662	0.054885521	4.898533241	0.502253752	0.478511514	0.694655901
MFSD10	-0.164807297	4.898655998	3.415780248	0.06457588	0.212476725
GDAP1L1	-0.472093005	4.8988927	33.58340845	6.83E-09	6.21E-07
TMEM18	0.260149572	4.898948858	9.339925024	0.002242147	0.021728227
ESCO2	-0.066216668	4.898949722	0.407492823	0.5232448	0.727919042
BLZF1	-0.007172966	4.899094084	0.006201978	0.937229376	0.972758255
GINS1	0.163664976	4.899454037	4.430795223	0.035296059	0.143904028
SLC25A37	0.377646263	4.899598882	4.982225738	0.025609016	0.116859776
PIF1	0.369474262	4.89972757	9.285430283	0.002309837	0.022102937
PIGF	0.053829072	4.899979526	0.561619213	0.453608572	0.676404989
TAF4B	-0.049755219	4.900027124	0.268326366	0.604456676	0.785137502
PM20D1	0.071550451	4.900582546	1.056875835	0.303928818	0.541885862
MCM9	-0.270198194	4.90105129	4.385274248	0.03625066	0.146512375

CREBZF	-0.210552634	4.901134254	3.166017056	0.075185592	0.233335674
C5H14orf1	0.033888985	4.901193977	0.174770187	0.675906727	0.829446281
TMEM266	0.030279965	4.901453793	0.137188375	0.711092303	0.849781439
ZFYVE21	-0.065745248	4.901487065	0.550224737	0.458225881	0.680822291
ARRDC4	-0.160111276	4.901579945	0.945890682	0.330767499	0.568614626
PAK1	-0.034999436	4.902326682	0.205499876	0.650317655	0.813532153
GDF3	0.015293216	4.902600661	0.027945491	0.867237036	0.938655886
GFRA2	0.397676715	4.902987939	9.212088421	0.002404223	0.022697352
ARFRP1	-0.068389335	4.903108756	0.984082673	0.321192934	0.558574269
LOC107050551	-0.067530738	4.90369772	0.481840041	0.487590034	0.701827677
PEX1	0.056258683	4.904051048	0.630716014	0.427092797	0.654871047
MEOX1	0.323424795	4.904461928	6.685813302	0.009718317	0.060850424
ING5	0.027020843	4.904734985	0.127640372	0.720891238	0.855801553
ASB8	-0.004197086	4.904921377	0.003332822	0.953963222	0.980523198
TKFC	0.790008524	4.905127422	23.80200257	1.07E-06	5.01E-05
MTMR12	0.063725115	4.905162644	0.540843401	0.462083404	0.683437728
CGNL1	-0.081741967	4.905207455	0.880961876	0.347938365	0.584408777
IL17RC	-0.176240418	4.905244292	3.130183695	0.07685508	0.236806463
SEMA3BL	0.518140544	4.905298191	13.88518165	0.000194325	0.003487035
TRMT5	0.251677961	4.905933667	10.74592357	0.001045093	0.012430121
TIPARP	0.043715329	4.906088335	0.238405455	0.625360159	0.798796471
NR2C1	0.628075974	4.906446465	36.29897982	1.69E-09	1.80E-07
GAAGSD	0.089692586	4.906729507	1.179631972	0.277430931	0.512532388
CZH18ORF25	0.221105867	4.906764768	5.291162065	0.021433913	0.103862114
BFAR	0.041331244	4.906947338	0.339825085	0.559930204	0.755279244
TMEM97	0.105816536	4.906993615	2.056337563	0.151574097	0.363261254
TMCO3	-0.176585145	4.907141602	5.632991704	0.017625525	0.091549249
SNAPC1	0.012455243	4.90731527	0.02220969	0.881530625	0.94696347
TRPC2L	0.002313292	4.908193845	0.000906942	0.975974957	0.990018742
APC2	-0.293679582	4.908735502	2.423097191	0.119558339	0.312274995
GALNT3	0.043602732	4.909142393	0.235154813	0.627727678	0.800097516
PLA2G6	-0.19608158	4.90941541	6.933272227	0.008460675	0.055285999

FILIP1	-0.168972668	4.910141338	2.722017196	0.098972865	0.276174069
MRPS12	-0.131649502	4.910821409	1.202677388	0.272787211	0.507543368
LOC101747821	-2.142221072	4.911509956	39.70663031	2.95E-10	3.82E-08
RTN1	-0.230993355	4.911629538	2.099367913	0.147360046	0.35717587
ZCCHC7	0.084211532	4.912094559	0.92832949	0.335297383	0.572606457
PHKA2	-0.39835561	4.912232298	27.1374881	1.89E-07	1.17E-05
SNX8	-0.163510628	4.912332767	5.441192357	0.019667239	0.09821682
TUBAL3	-0.581624298	4.912390327	20.31892683	6.55E-06	0.000231332
HSD17B2	0.272607229	4.912663786	2.241234616	0.134373652	0.337173706
DUS2	0.049960526	4.913133902	0.402787219	0.52565332	0.730140989
LOC100857932	-0.044593632	4.913937087	0.222635557	0.637039052	0.805600799
IQSEC3	-0.139788675	4.914141347	3.528254537	0.06033127	0.20309964
LOC422171	0.423524498	4.915410695	19.86640719	8.30E-06	0.000282342
HGF	-0.799031549	4.915793039	3.244549398	0.071661393	0.226288278
TMEM159	0.004410668	4.915903939	0.003475938	0.952986289	0.979802073
IFNGR2	-0.080130991	4.916518698	1.109377522	0.292217301	0.529417214
LIPG	-0.218466332	4.916594161	1.705691422	0.191545342	0.415657101
YIPF1	-0.121852113	4.916991214	2.287229531	0.130442577	0.330525502
UROD	0.085887821	4.917325406	0.886369426	0.346463059	0.582893247
CHGB	-0.204414573	4.917651853	3.078199299	0.079348332	0.241066582
RFLNA	-0.04035889	4.918753903	0.102258273	0.749136266	0.873205161
FAXC	-0.312366234	4.919037003	5.138858449	0.023396027	0.110120604
C26H6orf89	-0.129822997	4.919478881	2.077487594	0.149486047	0.360652762
GCC1	-0.221452347	4.920280564	9.796028713	0.001748891	0.018179412
SFXN1	0.183692669	4.920692344	6.638742095	0.009978437	0.061913716
DOPEY1	-0.195729722	4.920893162	5.24909619	0.021958174	0.105462945
FAM57A	-0.142618833	4.921251433	2.802452741	0.094120225	0.268763511
POC5	0.032843647	4.921966605	0.085266371	0.770283519	0.885380276
LOC112533588	-0.457391025	4.922239302	4.215281894	0.040061409	0.15680554
C2H18ORF21	0.058090105	4.922399223	0.494803591	0.481792322	0.697173002
FILIP1L	0.20519139	4.922636767	1.994228367	0.157899468	0.372585592
PBXIP1	0.015955716	4.922650493	0.021840987	0.882510897	0.947489154

MAP3K21		-0.090984027	4.922857935	1.294745367	0.255175278	0.49032844
CEP57		-0.063540756	4.922907106	0.432940008	0.510549846	0.718458116
CBY1		0.257328936	4.922942024	7.325731732	0.006797428	0.047314588
PTPRB		0.001132415	4.923458176	0.000108829	0.991676537	0.996095557
TBX15		-1.05362578	4.923496759	9.978566859	0.001583729	0.016856573
STC2		0.72988952	4.9237167	13.77930617	0.000205588	0.003653108
TMEM38B		-0.040774931	4.923737054	0.17533078	0.675416988	0.828968937
STK11		0.023788426	4.924052215	0.113875975	0.73577382	0.864920575
BBOX1		0.826111276	4.924300687	20.58287756	5.71E-06	0.00020743
THSD7B		0.065397425	4.924603494	0.437104888	0.508523123	0.717645625
SEMA3G		0.090913563	4.924766164	0.790510234	0.373945886	0.608644099
	03/set	-0.421884488	4.924976863	4.138807518	0.041910684	0.16092824
YARS2		0.127047636	4.925506646	2.264125302	0.132400949	0.333992948
FNTA		0.074290994	4.92557551	0.888841374	0.345791478	0.582454577
TRIM9		-0.029513493	4.925703785	0.10032665	0.751437993	0.874274211
HS3ST3B1L		-0.431178036	4.926607661	7.557587652	0.005975793	0.043260805
ABCB10		0.093816796	4.927304017	1.441825286	0.229844198	0.461005153
TTC30B		0.318259035	4.927554425	11.67842196	0.000632291	0.00859154
BAG1		0.230354383	4.927692339	5.228452646	0.022220292	0.106412951
GPR63		0.083330422	4.92775807	0.912014608	0.339580449	0.576596459
EXOSC1		0.304454057	4.928195465	16.44001389	5.02E-05	0.001199233
LARP1B		0.238399637	4.928399033	7.361023385	0.006665284	0.046730829
GPR89B		-0.031740459	4.928429156	0.104740102	0.746213814	0.871589176
SIM1		0.536986292	4.928444108	24.05841647	9.35E-07	4.55E-05
DIAPH3		0.220442031	4.928965409	7.736387884	0.005411895	0.040331854
MCCC1		-0.188090648	4.929114522	3.649481502	0.056086669	0.193368927
ATP6V0E1		-0.087862046	4.929903363	1.30068163	0.254088754	0.488666485
CHMP1A		-0.160442092	4.929970515	4.630993299	0.03139938	0.133374921
NAALADL2		0.097015966	4.930219207	0.787421058	0.374881082	0.609552194
LOC100857622		0.028469443	4.930241353	0.113291056	0.736427981	0.865442253
EFR3B		-0.005710773	4.930377007	0.0047176	0.945240528	0.976621285
SHMT1		-0.10858366	4.931258406	1.261033414	0.26145557	0.496451812

PLXDC2	-0.033413668	4.931666	0.173799707	0.676756732	0.830056055
ZNF142	-0.001711749	4.931847775	0.000430627	0.983443842	0.99261292
ATL1	0.418833293	4.932385297	28.12101602	1.14E-07	7.59E-06
LOC112533545	-0.038666412	4.933553738	0.234117469	0.628487453	0.800445901
SDHAF2	0.050964863	4.934259468	0.214842166	0.642998349	0.80933991
PALB2	0.05572564	4.934582061	0.628871766	0.42776946	0.655213795
L3MBTL4	-0.039090665	4.934607656	0.183288415	0.668562328	0.824306391
RGS14	0.069597816	4.934900665	0.811395331	0.367708042	0.602532945
EVC	0.284475924	4.935129544	12.08868449	0.000507288	0.007335164
ARPC5L	-0.03606509	4.935684218	0.18782669	0.664731634	0.822294319
CEP68	0.006140787	4.935687168	0.003999951	0.949571281	0.978531778
CACNA2D1	0.06476049	4.935869971	0.342201259	0.558561361	0.754112832
TRIT1	0.01081185	4.936796113	0.022889201	0.879745557	0.946151989
HAUS3	0.066494472	4.937033784	0.783488557	0.376076334	0.610590634
PHF21B	0.028398147	4.937101164	0.035516052	0.850518398	0.930485775
TPPNR1L	0.057600432	4.937315727	0.566599548	0.451613338	0.674958899
MRPL50	-0.084631418	4.937638962	0.509043687	0.47555313	0.692572543
TTLL4	0.258164626	4.937787926	10.01213327	0.001555123	0.016616589
LOC107050456	0.225983461	4.938074563	5.959547063	0.014637801	0.08076448
GRIP1	-0.168396623	4.93810189	3.371994287	0.066313437	0.216076753
EZR	0.014363457	4.938578899	0.0150709	0.902294303	0.955360193
CYP7B1	-0.041284824	4.938713061	0.167526121	0.682319846	0.83346248
NRCAM	0.157062368	4.939138348	1.639678059	0.200369645	0.426751555
NECAB3	0.125983369	4.941243271	1.485397927	0.222931873	0.452323547
PIGU	-0.112644425	4.941366542	2.564897241	0.109259657	0.295071008
TGFBRAP1	-0.149844669	4.941498161	3.777488724	0.05194657	0.184061327
CACNA1H	-0.15808974	4.942233852	2.32524655	0.127290051	0.325191397
RMI1	0.183936991	4.942303176	3.877631593	0.04893354	0.176889589
LOC107049475	-0.581758271	4.942964967	11.06776766	0.000878414	0.010906527
LOC426514	0.021914431	4.94409583	0.067070441	0.795650969	0.900396293
LOC101749773	-0.004971774	4.94436086	0.004681384	0.94545079	0.97671734
TMEM229B	0.046783329	4.94453772	0.171785925	0.678529453	0.830806023

ZBTB41	-0.221891209	4.944752176	4.65555416	0.03095324	0.132226261
DIS3L	-0.196836969	4.94488976	7.222491484	0.007199571	0.049198065
KLF3	0.006708159	4.945823346	0.005307088	0.941925668	0.975110844
ZNF800	-0.173714941	4.946675569	5.489319974	0.019132983	0.096426721
NSUN5	0.060555266	4.946958023	0.457030578	0.499015016	0.710196514
ERCC5	0.010506058	4.94715853	0.020035196	0.88743883	0.949282062
TIMP3	0.088090735	4.947251871	0.512561581	0.474032065	0.691936275
C11orf24	-0.017958449	4.94743409	0.065624897	0.797816817	0.901282145
DDHD1	0.211993549	4.947535499	5.587715881	0.018086866	0.092927773
BTBD11	-0.36998872	4.947661746	14.12352939	0.000171189	0.003161521
PANX1	0.050992668	4.94825076	0.357791388	0.549735408	0.748398787
KCTD1	0.12237364	4.948762339	2.254375634	0.133237184	0.335129604
GRK6	-0.098695698	4.948857707	1.941883808	0.163464139	0.380007306
MAP3K14	-0.213477689	4.949104276	3.148831132	0.075981372	0.235102197
DPY30	0.100172383	4.949173942	1.747689016	0.186167519	0.408942268
PI4K2A	-0.014027062	4.949318414	0.024852642	0.874734802	0.942987498
KATNB1	0.029583222	4.949342384	0.120731772	0.728242175	0.860184121
RPUSD3	0.101029043	4.94976342	1.513652269	0.218582627	0.447805918
ANKH	-0.004329008	4.94985229	0.001881756	0.965399246	0.985706416
DVL1	-0.159666184	4.950386294	3.345788208	0.067377202	0.217946111
LRRC42	-0.009297086	4.950406719	0.012552656	0.910792772	0.959051325
ATP6V1C1	0.131893306	4.950942714	3.085157095	0.079009635	0.240359317
SMC1A	-0.020677598	4.951323462	0.056871553	0.811510708	0.90944689
UEVLD	0.018590427	4.951928665	0.04791629	0.826729584	0.917915823
POMT1	0.260361602	4.952268636	4.532799124	0.033251188	0.138764469
PUS10	-0.171131982	4.952443003	5.449510285	0.019573813	0.097881507
WASHC1	-0.058339541	4.952656433	0.530164713	0.466537619	0.686538769
DCAF8	0.012480693	4.952772995	0.028181302	0.86668329	0.93840635
RBM38	-0.036613839	4.953181933	0.168434071	0.681507264	0.83319824
HDHD2	0.235686699	4.95329812	6.987768792	0.00820686	0.054147504
FKBP14	0.066279908	4.953485936	0.352448512	0.552730348	0.751170952
LY75	-0.16870958	4.953546725	3.128905033	0.076915385	0.236878656

NDUFAF4	0.324337705	4.954046737	12.30540678	0.000451648	0.006713522
MRPS10	0.244023431	4.954449718	8.412516513	0.003726465	0.03098483
PIK3CA	-0.278697866	4.954642447	5.656268497	0.01739309	0.090911496
TMEM203	0.059743349	4.954827122	0.497619336	0.480548037	0.696466157
DALRD3	0.133054413	4.955121768	3.17060601	0.074974623	0.232906818
TPRKB	0.166814921	4.955132213	5.247145941	0.021982799	0.105490509
BBS2	-0.106086691	4.955177247	1.221249399	0.269115596	0.504027293
RCBTB2	0.008094695	4.955907116	0.011618652	0.914162395	0.960755075
XPNPEP3	0.107605383	4.956297341	1.787898964	0.181182363	0.403338029
BMT2	-0.15092211	4.95684418	3.386252897	0.065742195	0.214896271
SCMH1	-0.009602142	4.957115666	0.014865342	0.902959597	0.955614537
BLOC1S2	0.13642622	4.957274316	3.194957252	0.073865701	0.230418979
DNAJC19	0.066239617	4.957429826	0.698675655	0.403229051	0.635001822
MAFF	-0.156279331	4.958185519	2.242294159	0.134281619	0.337035395
TMA16	0.094791474	4.958856326	1.03784317	0.308323625	0.546238721
UGCG	0.145366792	4.958940721	2.792046543	0.094733171	0.269554615
STARD3NL	0.136882958	4.959566805	3.420480328	0.064392275	0.212042203
TMEM150C	-0.247089424	4.960311931	8.161403612	0.004279124	0.034048834
CYTL1	0.139737379	4.96042108	1.30436929	0.253416662	0.48783004
ULK3	0.162124594	4.960435747	4.441588118	0.035073607	0.143174449
RNF138	-0.209587383	4.961204698	5.570805741	0.018262355	0.093383174
AGBL4	-0.023526069	4.961558102	0.103968843	0.747117871	0.87245934
UXS1	-0.005979963	4.96220179	0.004447397	0.946829444	0.977487125
SLC25A16	-0.12134575	4.962625372	2.390227046	0.122095895	0.31659123
STX17	0.155041478	4.965436928	4.64971347	0.031058731	0.132470561
JKAMP	0.034730381	4.966969341	0.264027047	0.60736692	0.787008636
PTPRD	0.063991113	4.967378383	0.303880789	0.581459681	0.770363853
ZNF276	-0.187987576	4.967561477	5.536528888	0.018623487	0.094641591
TUSC2	-0.148589254	4.968314032	4.099321253	0.042900433	0.16329554
GTF3C3	0.302562507	4.968350776	17.27875015	3.23E-05	0.000827303
NR3C2	0.04688211	4.970039735	0.292555569	0.588587497	0.774756996
TFAP2A	0.253863961	4.970860821	3.944491633	0.04702506	0.172684081

DPP10	-0.049038643	4.971139675	0.287281265	0.59196805	0.776980894
MFSD13A	0.084642193	4.971160758	1.529713551	0.216155441	0.444994092
TMEM67	0.162471122	4.971373007	4.113651438	0.042538427	0.162368316
INAFM2	0.12477648	4.971554347	1.292908938	0.255512562	0.490373128
UIMC1	-0.010292827	4.972259383	0.018531173	0.891719103	0.951008202
KCNH5	0.098895995	4.972481167	0.334048613	0.563284787	0.757599665
KCTD6	-0.014481764	4.973560964	0.018409066	0.892074249	0.951008202
COL7A1	-0.024827008	4.974030117	0.093845235	0.759344496	0.879311307
BCAS3	0.26878459	4.974057115	8.928931376	0.002806885	0.025183913
TMEM199	0.31413619	4.974199219	15.85584503	6.84E-05	0.001545031
SLC16A10	-0.054741225	4.974828853	0.28361339	0.594342595	0.778035592
NADSYN1	-0.019288347	4.975085262	0.056965572	0.811357902	0.909337575
THUMPD2	-0.004128852	4.975141049	0.002623271	0.959151904	0.982436637
RAB3A	-0.162101952	4.976508962	3.214160589	0.073003627	0.228860693
POP5	0.078206692	4.977089923	0.92452902	0.336288603	0.573492482
MTR	0.239799137	4.977395028	6.054464209	0.013871184	0.078141159
TMEM126A	-0.100445532	4.97796913	1.612711322	0.204111092	0.430927383
GTPBP2	-0.062642707	4.978419568	0.476272081	0.490115781	0.703739544
PEX26	-0.305104633	4.979010422	19.73371048	8.90E-06	0.000297719
REXO4	0.165922148	4.979263494	4.220913966	0.039928648	0.156546557
PIGH	0.247291347	4.980036196	9.630570967	0.001913651	0.019522089
NRBF2	-0.053504152	4.98031053	0.395285715	0.529534009	0.733087844
N6AMT1	-0.220155915	4.981165353	7.861512226	0.005049814	0.038439969
ZNF302	0.025735684	4.981255816	0.084299865	0.771552783	0.88652336
ADAMTS12	0.310056132	4.981767766	6.176683787	0.012944466	0.074423897
TRAF3IP2	-0.261806268	4.982375558	9.230957697	0.002379572	0.022564277
IFNAR1	-0.096999993	4.982390185	1.66704885	0.196654283	0.421731556
TBC1D23	-0.013764628	4.98246286	0.036611332	0.848258536	0.929300547
STIL	0.10195445	4.982470362	1.800850457	0.179609713	0.401177932
SAMD12	0.001403678	4.98270807	0.000201684	0.98866919	0.994817358
TFE3	-0.07689825	4.982797549	1.039962819	0.307830119	0.545952211
RAB11FIP5	-0.234295228	4.982847896	8.053880819	0.004540628	0.035612984

DUSP8	0.307149318	4.983133665	11.49977824	0.000696045	0.009234711
PWWP2B	0.100717949	4.983208675	1.609143695	0.204612214	0.431327134
SLC16A9	-0.07188996	4.983808567	0.584504577	0.444552068	0.669452208
TNNC1	0.245382904	4.984549364	4.35835602	0.036827829	0.147877484
ISL1	0.000402899	4.984637444	1.02E-06	0.99919563	0.999602853
SCYL1	-0.050133856	4.984691987	0.387976473	0.533364976	0.735495698
RBM42	-0.022515495	4.985045529	0.04784458	0.826857232	0.917915823
YKT6	0.088685915	4.985164744	1.089590533	0.296561951	0.533680327
TICAM1	-0.111390577	4.985598682	1.979874049	0.159403676	0.374391592
COL27A1	-0.063215758	4.985929612	0.332888711	0.563963041	0.758108965
CRAMP1	-0.11811409	4.985956619	1.278093117	0.258253889	0.492703507
PLEKHH1	-0.197365121	4.988093819	3.23871999	0.071916805	0.226571418
MPHOSPH8	-0.083333539	4.988252465	1.186324283	0.276072248	0.511145116
LOC107049214	-0.07573344	4.989287793	0.831274245	0.361904326	0.598210789
NFKB1	0.334694768	4.989832433	18.44767521	1.75E-05	0.00050414
LOC107057318	-0.375621147	4.990671668	23.94439841	9.92E-07	4.77E-05
MTHFD2	0.336411682	4.990819585	13.17539612	0.000283649	0.00472413
LOC693265	-0.007983183	4.991096346	0.010672253	0.917719638	0.96231374
ADAM23	0.098067201	4.991122001	1.446701935	0.229057893	0.459827725
CYP26B1	0.595341482	4.991412701	26.98387649	2.05E-07	1.25E-05
KLF6	0.037912834	4.991489203	0.158015927	0.690989813	0.838946165
GDPD4	0.193054885	4.99165001	1.951425439	0.162433329	0.378860143
STK16	0.212895198	4.991765303	7.33772531	0.006752223	0.047133796
MRPL30	-0.029149147	4.992483332	0.159131366	0.689957509	0.83840108
COL16A1	-0.104696975	4.992495467	0.345102746	0.556898525	0.753115441
MAVS	0.237046824	4.99252196	5.004646323	0.025279368	0.115869639
CR1L	-0.031552473	4.992988812	0.036738885	0.847997649	0.929138454
BBS9	0.254943839	4.993219776	10.59282431	0.001135274	0.013192752
HLF	-0.24833425	4.993720016	6.050917735	0.013899071	0.078259853
ACOT13	0.172109569	4.993953954	3.958771716	0.046627715	0.171531198
HPCAL1	0.054060844	4.993977236	0.349159431	0.554589342	0.752428986
TXNL4A	0.148984977	4.994169883	2.869557105	0.090269708	0.262036199

MIB2	0.069254915	4.994336206	0.746208276	0.387679375	0.621821241
NUDT7	0.165142083	4.994470192	4.96677196	0.025838828	0.117484916
VPS18	-0.072068916	4.994730317	0.900897545	0.34254116	0.57970943
LOC422320	0.149166465	4.995036753	3.327505831	0.068130119	0.219439538
ADCY5	0.493785766	4.995311908	27.0372889	2.00E-07	1.22E-05
PKD1	-0.056721402	4.995554669	0.322485193	0.570117949	0.76248378
MFHAS1	0.062020398	4.995642238	0.598214055	0.439260257	0.664523999
PNPLA2	0.114732747	4.9958624	2.513586501	0.112868797	0.301160379
MPC2	-0.156311906	4.995903856	3.365431761	0.06657813	0.216472633
PLEKHM3	0.058543962	4.996116582	0.330939119	0.565106624	0.758964209
CASP8	0.106280697	4.996172714	2.065670872	0.15064862	0.36205149
CA5A	-0.129837417	4.996181074	2.255678274	0.133125115	0.335082178
TBK1	-0.087242464	4.996273102	1.462177005	0.226584054	0.456544471
CAP2	-0.161747031	4.996625269	4.307787946	0.037938263	0.15049741
HPSE2	-0.300663164	4.996922935	5.303785198	0.021279134	0.103502261
GPAT3	0.082009244	4.997205045	1.201599401	0.273002244	0.507692808
CEP97	-0.169675542	4.997609948	4.10385853	0.042785464	0.163008754
CZH18ORF32	0.071539526	4.997645178	0.436423965	0.508853525	0.717892027
ZIC3	0.194154955	4.997857829	1.205863613	0.272152873	0.506936328
ITGAD	-0.038289849	4.998037353	0.111599385	0.738330555	0.86674961
TLL1	-0.040935256	4.999055648	0.259483675	0.610475099	0.788304775
LMX1B	-0.125852993	4.999190362	0.401214601	0.526462652	0.730618448
ALG3	-0.101981939	4.99985227	2.109046138	0.146430566	0.355647966
TMEM14CL	-0.061553435	5.000461486	0.664610254	0.414937053	0.645405182
RNF114	-0.052463414	5.000530029	0.518392443	0.471528227	0.690240812
FAM110A	-0.2155241	5.000569024	4.711042483	0.029969473	0.12923157
GSTA2	-0.271284512	5.001065579	6.639001874	0.009976982	0.061913716
TBX5	-0.976242417	5.001394222	3.748824711	0.052844656	0.18628081
ADSSL1	-0.115757431	5.001487979	1.094670382	0.295438715	0.532228922
FBXO39	0.231625321	5.001934934	9.937937117	0.001619069	0.017136587
ABCC9	-0.285865438	5.002105199	1.743216051	0.186731847	0.409635055
RRAGD	-0.159088867	5.002484439	2.838538943	0.092027858	0.265204623

SMYD5	-0.208166459	5.002580197	7.767416266	0.005319709	0.039858242
EEF1A2	-0.506373516	5.002739691	4.651718107	0.031022482	0.132384582
KCNG1	-0.466067981	5.002957051	1.602642661	0.205529109	0.432404944
MPST	-0.085990214	5.003713452	1.408192736	0.235356641	0.467559282
COL6A1	-0.454002141	5.004007328	6.011703554	0.014211301	0.079236332
CADPS	0.318812031	5.004158289	6.433660936	0.011197754	0.067229113
KLHL18	-0.113324633	5.005366212	2.359794727	0.124498571	0.32014752
KLHL35	-0.260326135	5.005899971	3.228909239	0.072348862	0.227375706
NDUFA1	0.183111279	5.006534374	4.791466282	0.028601069	0.125601973
PACSIN3	0.03096688	5.006842792	0.20451692	0.651099358	0.813812377
COX20	0.167339627	5.007036742	4.040671556	0.044416144	0.166446742
EPS8	0.288618784	5.008487703	9.078996105	0.002585619	0.023774878
RABEPK	-0.054594194	5.0091777	0.523879219	0.469191592	0.688269654
NAIF1	-0.04491266	5.010041483	0.383584398	0.535691143	0.737331472
JMJD6	0.065592541	5.011015216	0.232986417	0.629318233	0.800882185
THSD1	-0.191229337	5.011596301	3.387084167	0.065709055	0.214830618
THAP11	0.074486321	5.011978239	0.949802224	0.329769645	0.567404701
TBCE	0.165962461	5.013306831	5.474354469	0.019297487	0.096958822
MAPKKK3L	-0.083749375	5.01385395	1.147518815	0.284069151	0.520124389
PGM1	-0.014094298	5.014083218	0.039259298	0.842935848	0.926094935
BCL10	0.063153634	5.014127194	0.548165127	0.459068393	0.681397294
VPS13A	0.191793016	5.015029643	2.993260218	0.083611677	0.249154317
PEX13	0.171177305	5.015120708	6.22394597	0.012603391	0.07323051
PCTP	-0.319849397	5.015182459	8.183302723	0.004227772	0.033787267
PEX10	0.116397657	5.015317302	2.383208996	0.122645381	0.317392968
TMEM220	-0.262499853	5.015949319	6.894586289	0.008645717	0.056120423
SLC39A11	-0.242016012	5.017221054	11.17258776	0.000830148	0.01050352
PLPPR1	0.021470595	5.018093611	0.029892659	0.862734012	0.936001285
MRPL41	-0.056002431	5.018138286	0.316298354	0.573840737	0.764888564
PPDPF	0.007497766	5.01833625	0.005808836	0.939247475	0.973747303
ZNF367	0.039515224	5.018986567	0.102601474	0.74872982	0.873168892
POLR3H	0.103753606	5.019529127	1.652713658	0.19858999	0.424224105

ARID5B	0.017807444	5.020482482	0.034680223	0.852267325	0.931274244
YDJC	0.13628249	5.022003266	3.558588612	0.059238087	0.200568461
CRYBG3	-0.131974238	5.022856748	1.917938912	0.166084052	0.38379302
ANO1	0.11201396	5.023030361	0.233374595	0.629032829	0.80064474
PDGFD	-0.766162284	5.023337375	53.59118808	2.47E-13	7.81E-11
HAND1	-0.075615929	5.023443022	0.027358339	0.868626348	0.939356373
PDZRN4	-0.002680562	5.023570973	0.000631646	0.97994923	0.991518452
FREM3	-0.025572269	5.023876579	0.050846348	0.821597055	0.916282016
ASAH1	0.211127311	5.024080253	7.087976454	0.007760282	0.052035633
LOC421106	0.302125492	5.024625145	13.44479735	0.000245687	0.004237707
TMEM121	0.178663281	5.024636046	2.958681489	0.085417569	0.252614323
COL22A1	-0.140597638	5.025858843	1.029391727	0.310301576	0.548208336
ATXN7L1	0.063630819	5.026003885	0.884012167	0.347105129	0.583711283
PDP1	-0.190454916	5.026106021	2.498947594	0.113922403	0.302842189
ANKRD26	0.00177656	5.026220004	0.000418252	0.983683428	0.992657841
DOK4	-0.162740559	5.026337035	3.93686396	0.047238763	0.172967032
GSAP	-0.009391324	5.026558748	0.01381086	0.9064483	0.95723314
PHF13	-0.056769948	5.02687126	0.709636342	0.399564564	0.632024769
TMEM74	-0.294730008	5.026956138	3.260702879	0.070958705	0.224960367
VAMP4	-0.138012551	5.027770827	3.400714341	0.065168191	0.213656623
AUH	0.257008638	5.027782388	8.845021635	0.002938894	0.026053133
DHRS7B	0.026909456	5.028578625	0.140552235	0.707732632	0.848671885
SCPEP1	-0.151607424	5.028958679	2.228902573	0.135450024	0.338419396
ERCC4	-0.09423347	5.02958229	1.458557061	0.227159847	0.456941563
PASK	0.259064453	5.029590881	10.5003948	0.00119349	0.013619199
FST	0.195343661	5.029701685	2.32918653	0.126968213	0.324621184
CAPRIN2	-0.044059492	5.030413437	0.207376587	0.648831425	0.812579461
WDR6	-0.21708452	5.030946039	8.171319065	0.004255795	0.033945274
SYS1	-0.01795683	5.031186038	0.052313131	0.819086051	0.914759127
CAPN7	-0.147301209	5.031280548	3.530670489	0.060243422	0.202886923
AFTPH	-0.081414059	5.031692253	1.180998272	0.277152861	0.51230626
TMEM241	-0.010805665	5.031692995	0.023878393	0.877194736	0.944525545

PPCS	-0.133638137	5.031732176	2.137477133	0.143738105	0.352175478
ATXN7	0.015809652	5.031898556	0.044956349	0.832084315	0.920518332
SLC4A1	0.359897853	5.032135024	1.475891799	0.224418396	0.453917322
CACNA1C	-0.083795084	5.032193139	0.49065245	0.483636406	0.698336
LOC107057415	-0.33545473	5.032548061	16.22445306	5.63E-05	0.001321977
DDX59	-0.069442306	5.032581154	1.026226739	0.311046549	0.548758546
RFFL	0.133568991	5.032716543	1.574270388	0.209587932	0.437426118
TRMT61B	-0.080897923	5.033356601	1.40135951	0.236496077	0.468804114
REEP4	0.166419131	5.033798821	3.595681365	0.057929877	0.198004626
LOC112533016	0.050987088	5.033853162	0.496903456	0.480863886	0.69668007
DPCD	0.081310283	5.036427455	1.044296292	0.306824369	0.544790871
FDX1	-0.020161201	5.036695271	0.070462916	0.790663964	0.897205401
UMAD1	0.021812993	5.037284561	0.064412339	0.799653271	0.902345328
PRKD1	0.030473849	5.037352475	0.118254772	0.730935093	0.862250839
ECHDC3	0.23068655	5.03754685	9.858253247	0.001690716	0.017695219
SPG21	0.140712715	5.038071076	3.204002819	0.073458275	0.229539674
FAM171A2	0.104958956	5.038237294	1.367018511	0.242324806	0.475074917
SIDT2	-0.174826661	5.038448019	4.06406801	0.043804851	0.165133753
SPEG	0.07358966	5.038858498	0.410606464	0.521661849	0.72659097
LOC101750700	0.915011505	5.039107825	51.73216228	6.36E-13	1.59E-10
EOGT	0.240638155	5.03937377	8.827840687	0.002966692	0.026245656
LOC107056278	0.312437184	5.040022991	8.610209014	0.003342839	0.028574765
GTF2H2	0.101407282	5.040191223	1.033994606	0.309222293	0.54712396
PLPP4	0.59078607	5.040207512	27.09565012	1.94E-07	1.19E-05
DBF4B	0.062520994	5.040819856	0.598560359	0.439127842	0.664502993
DCP2	0.021947167	5.040821293	0.046531668	0.829212244	0.918867551
GSG2	-0.007710583	5.04086143	0.008874538	0.924946498	0.966348866
VTN	0.15295558	5.040950431	1.243734834	0.264752473	0.499885477
ADAMTS6	0.20751391	5.041794805	3.193534642	0.073929998	0.230566212
CCDC47	-0.021582445	5.041910233	0.077244703	0.781066622	0.891973854
ANAPC11	0.126200406	5.042058931	1.522005998	0.217316178	0.446378444
ACAN	-0.102673349	5.043509104	0.299742904	0.584043642	0.771126537

TAF9	0.074757741	5.043613445	1.156348983	0.282224018	0.517783055
CLC2DL5	0.25068594	5.043622818	3.471465423	0.062435975	0.208014573
CENPO	0.024097126	5.043646469	0.122179858	0.726682179	0.859204948
C1H12ORF66	0.06624371	5.044590817	0.635605747	0.425306514	0.653696874
LOC112533506	0.460221137	5.044947472	25.49524554	4.43E-07	2.43E-05
FAM174A	0.15288796	5.045577977	1.617966386	0.203375579	0.430265966
FOXRED1	0.036024628	5.046047981	0.131997481	0.716369906	0.853085451
MICU3	-0.149465532	5.046346379	4.476226262	0.034369531	0.141562306
CDC34	0.25192634	5.046755989	9.089319672	0.002571065	0.023695391
COL4A3BP	0.008307278	5.046783645	0.006844941	0.934062923	0.971309744
LOC112530206	-0.209318102	5.046893987	5.58708084	0.018093425	0.092927773
MAP3K9	-0.022164531	5.047215379	0.049706256	0.8235752	0.91690482
INO80B	0.188891862	5.04768928	4.048123732	0.044220468	0.166051447
LSM8	0.002833981	5.047782398	0.000925849	0.9757259	0.990018742
SNAPIN	-0.302491566	5.048180773	23.00554716	1.62E-06	7.03E-05
FPGT	0.19358715	5.048434977	5.427686934	0.019819914	0.098620107
KIZ	-0.103304358	5.048931284	2.089526072	0.148312073	0.358471676
IPP	0.095355749	5.049036304	1.629329927	0.201795754	0.428679206
PARP6	-0.023416578	5.049119254	0.092034318	0.761606699	0.88065738
ZNF704	0.253922862	5.051628286	7.55259627	0.005992368	0.043342597
C1H12ORF4	-0.060576222	5.051663337	0.634303342	0.4257812	0.654115362
METTL6	-0.187565031	5.052297655	5.207751305	0.0224864	0.107109491
MANEA	0.085226054	5.052459154	1.720029764	0.189689214	0.41314838
C4H4ORF32	0.169138531	5.05270928	2.995365846	0.08350305	0.249056134
STAMBP	-0.157611996	5.052769591	5.333398906	0.020920548	0.102244999
TUBGCP6	-0.205858357	5.053638685	7.827856219	0.005144708	0.038922379
GABPB2	0.202944015	5.053721673	5.402339789	0.020109775	0.099346821
AATK	-0.297340366	5.054243904	10.57990814	0.001143233	0.013238498
PITPNM2	0.322744133	5.054498992	15.27228535	9.31E-05	0.001956206
FKTN	0.138796076	5.055065312	2.540262839	0.110976311	0.298384837
NIPBLL	0.433207776	5.055587197	0.367588928	0.544321451	0.744408787
SPTLC2	0.130725411	5.055711395	1.981039906	0.159280898	0.374316934

PJA2	0.120385059	5.055763751	1.79370768	0.180475071	0.402400717
SSH2	-0.054590086	5.056763714	0.29827504	0.584965842	0.771777817
TMBIM4	-0.176671909	5.056792155	3.471580312	0.062431639	0.208014573
ATG2B	-0.15311019	5.05689597	2.01377606	0.155876925	0.369468388
TMEM167B	0.002037517	5.057149725	0.000376137	0.98452659	0.992968171
CHTF8	0.056186416	5.057172885	0.322670205	0.570007351	0.762434642
RPAP2	-0.190903306	5.057364296	4.763481955	0.029069684	0.126746594
PGM5	-0.019167881	5.057718344	0.005689315	0.939874541	0.974140501
TMEM200C	-0.123895463	5.05802952	2.23438644	0.134970192	0.338041781
COX10	0.064008404	5.058151012	0.476706838	0.489917783	0.703700865
MAFB	-0.258314353	5.058195034	2.726856741	0.098673312	0.27564743
ABRACL	0.021351491	5.058283632	0.061018442	0.804893433	0.90566365
CDIP1	-0.255933385	5.058316404	10.3051167	0.001326619	0.014739709
SPRYD7	0.055064145	5.058569898	0.579349369	0.44656746	0.670621395
PGAP2	0.194389241	5.059077002	6.447719738	0.011109484	0.066860676
TRAPPC4	0.073475282	5.059893314	1.024556424	0.311440645	0.549202656
STK38L	0.24961366	5.060405997	9.182718698	0.002443109	0.022880678
GUCY1B3	0.221887842	5.06100257	9.032991847	0.002651502	0.024183701
LOC100857197	-0.096788004	5.06161563	1.280616934	0.257784358	0.492541889
FBXO30	-0.018553373	5.062587428	0.057020198	0.811269181	0.909337575
MEX3D	-0.183215442	5.063161567	2.798732824	0.094338839	0.269024818
ANGPTL1	-0.039664797	5.063244818	0.117481872	0.731781814	0.862408819
GNB5	-0.140533586	5.063283569	4.495129024	0.033991548	0.140464824
TADA2A	-0.143587617	5.063353526	1.724299284	0.189140572	0.412641691
CCDC174	-0.063209482	5.063719835	0.953546728	0.328818148	0.566447093
RELA	0.024723722	5.063977697	0.109767424	0.740409076	0.867900794
NPDC1	0.119910665	5.064160803	2.591423106	0.107443759	0.291842445
CMTR2	-0.036510162	5.064253567	0.163246205	0.686185231	0.836197732
KCNAB1	-0.400424386	5.0644391	6.009752682	0.014227021	0.079236332
MTRR	0.111991864	5.065903933	2.397541607	0.121526095	0.315600463
TMEM138	0.045461342	5.066107525	0.346794144	0.555933521	0.752912436
MED27	0.189717938	5.066923879	5.215084177	0.022391763	0.106953979

LOC112532888	0.088453827	5.067250368	1.460358666	0.226873062	0.456755952
FBXL2	0.00412945	5.06753469	0.002245578	0.962204368	0.983899168
CCDC186	0.017615184	5.068352808	0.049589088	0.82377984	0.91690482
MCPH1	0.2941533	5.068908856	10.39962324	0.00126041	0.014147375
LRRC1	0.023759379	5.069280053	0.150728848	0.697839816	0.842758423
SFXN4	-0.097538299	5.069314971	1.69577558	0.192841367	0.417197567
ARHGEF39	0.283483922	5.069484403	4.268569397	0.038823603	0.153016142
WBSCR27	-0.0668509	5.069942519	0.515909171	0.472591949	0.691022409
BBS5	-0.281639181	5.070179412	10.41393467	0.00125068	0.014057334
TMOD1	0.246425564	5.070180882	5.733445588	0.016644794	0.088439624
IGHMBP2	-0.027576431	5.070187728	0.173922756	0.676648804	0.830023971
MYC	0.077624372	5.070510277	0.472224453	0.491965592	0.704612571
ANO8	0.046617608	5.071469984	0.245835245	0.620022998	0.795058816
SRPX	-0.005025478	5.07164748	0.004024273	0.9494184	0.978504123
HAS2	0.136467771	5.071933273	1.35307427	0.244741387	0.478235307
RCC1L	-0.101205875	5.071989484	2.176829261	0.140102841	0.346310988
SDHAF1	0.392259532	5.073310895	22.70151097	1.89E-06	8.04E-05
SLC1A3	0.30632419	5.074037837	5.210553075	0.022450192	0.107108701
VPS39	-0.05459688	5.074721612	0.54994477	0.458340262	0.680869279
GRM4	-0.234247438	5.074987376	1.934118905	0.16430852	0.381339448
WFS1	-0.058977502	5.075447377	0.580916468	0.445953319	0.670457913
GPALPP1	0.207014396	5.075776822	6.866221781	0.008784017	0.056638916
SERINC5	0.153189162	5.075797165	3.213204998	0.073046269	0.228860693
NDEL1	-0.076867968	5.076883339	0.805987727	0.369309145	0.604000819
PDXDC1	-0.072752343	5.077206736	0.978315161	0.32261512	0.560100422
MEF2D	0.036409293	5.07737305	0.195622983	0.658277289	0.818123323
PIBPPDD4L	0.070802038	5.077597358	0.630882649	0.427031737	0.654871047
UCK2	0.120737216	5.077701833	2.152872641	0.142303419	0.349597307
TMEM68	0.129554629	5.077827337	3.720727589	0.053740948	0.188452808
KPTN	-0.155708625	5.078046799	3.370103729	0.066389575	0.216110872
TRUB1	0.001295856	5.078865868	0.000213581	0.988339813	0.99474779
COL13A1	0.020150096	5.078937792	0.043666908	0.834474558	0.921720365

TMEM98	-0.010885203	5.079441844	0.020520471	0.886092996	0.948791653
HMP19	-0.078293453	5.079655962	0.258419232	0.611208249	0.788965932
FAN1	-0.078230909	5.080284048	1.105560829	0.293048966	0.530369636
COMMD1	0.122197834	5.08047494	2.294802688	0.129807706	0.32942409
ERMP1	0.012502871	5.080901826	0.013920728	0.906078643	0.957027028
HPS4	-0.066356832	5.081315625	0.612979807	0.433668061	0.660252401
CDR2L	-0.100764699	5.081523268	0.969398552	0.324830224	0.562889668
OARD1	0.022208246	5.082432148	0.09484226	0.758109189	0.878437202
JAK2	0.072689587	5.082817751	0.744539455	0.388210598	0.622127521
SLC4A2	0.089833134	5.082891731	0.663513482	0.415322285	0.645642722
TFEB	-0.195920998	5.083824801	4.482250041	0.034248604	0.141309554
EXOC6B	0.010751088	5.083826545	0.019171461	0.889876054	0.95046803
CYB5R2	0.12771238	5.084019092	1.395742957	0.237437641	0.469821594
FBXW8	0.051487243	5.084532165	0.678720166	0.410027779	0.64033991
NR3C1	0.130864593	5.085195615	3.109452214	0.077839136	0.238215389
ACAD11	0.287351983	5.085336214	9.44016766	0.002122841	0.020924549
WDSUB1	0.037449828	5.085403745	0.226162793	0.634383773	0.80403489
STAB2	0.217348946	5.086577641	2.859776625	0.09082011	0.262921341
DOCK9	-0.22301621	5.086893271	8.049961788	0.004550461	0.035673103
AIP	-0.106560985	5.087295843	1.612371467	0.204158767	0.430972163
RNF150	-0.174746038	5.087628048	4.009842836	0.045235367	0.168242723
IFT88	0.012176699	5.088442733	0.021431997	0.883608224	0.947979469
CBFA2T2	-0.000298367	5.089309426	1.04E-05	0.997427587	0.998885697
SNX11	-0.021256509	5.089398948	0.061777546	0.803708198	0.905079278
ECI2	-0.057598167	5.089473641	0.66722919	0.414019309	0.644347652
MTFMT	0.063607761	5.090016095	0.291112402	0.589508557	0.775222833
ISCA1	0.097483707	5.090204897	1.036805903	0.3085655	0.546470046
SLC20A2	0.047834864	5.090471483	0.255692587	0.61309497	0.790261455
TIAM2	0.187696193	5.090821331	4.089880385	0.043140695	0.16375551
ZNF76	-0.153315173	5.0915728	4.720977505	0.0297968	0.12862418
GAS2	-0.13297627	5.09157823	3.569613094	0.058846021	0.199885747
FOXO6	0.371577054	5.09216925	17.40342694	3.02E-05	0.000788674

MLLT6	0.006212008	5.092307901	0.00420779	0.948279516	0.978180757
LIFR	-0.315436919	5.093671452	5.530049674	0.018692575	0.094846228
ADPRH	0.161224236	5.094038855	4.478094643	0.034331975	0.141480755
RAD52	-0.131561669	5.094259652	3.268304259	0.070630597	0.224368045
SMAD2Z	0.044949195	5.094998196	0.21178159	0.645374581	0.810783229
CCDC141	0.215590031	5.096028817	3.017491539	0.082370785	0.24666265
HSCB	0.06926043	5.096036197	0.7794701	0.377303251	0.61215983
PRR11	0.203409105	5.096058318	7.699855915	0.005522523	0.040878491
ITPR1	0.135278085	5.097605529	4.047762588	0.04422993	0.166051447
MAN1A1	-0.112094706	5.098367586	1.79310316	0.180548531	0.402481937
SNX33	0.108006027	5.09885982	1.570316672	0.210161031	0.438045609
FBXO46	-0.029096989	5.09961512	0.114549315	0.735023085	0.864717585
CTTNBP2	-0.216507726	5.099649306	7.333360071	0.00676864	0.047177115
CARHSP1	0.009938352	5.100021547	0.019393011	0.889245652	0.950041371
HIBADH	0.324989661	5.100399469	19.67408906	9.18E-06	0.000302845
TTI1	-0.003568634	5.10068047	0.003026629	0.956126666	0.981041173
LOC107052984	0.298831955	5.101984524	6.875364614	0.008739193	0.056482095
SLC24A3	-0.125264895	5.10206499	2.370634046	0.123636826	0.318775621
ZFYVE26	-0.351849095	5.102360373	16.06769416	6.11E-05	0.001412492
LMF1	-0.030041749	5.102535056	0.176275063	0.674594131	0.828521854
TMEM255A	-0.202868558	5.102604629	4.121371131	0.042344746	0.162042509
NDUFA6	-0.051517237	5.103033486	0.404302577	0.524875551	0.729584996
LRSAM1	0.0433216	5.103854585	0.321171342	0.57090456	0.763014502
LYRM1	-0.176589393	5.104369702	6.522487489	0.010651892	0.06483625
MRPS11	-0.023237815	5.104581542	0.102272071	0.749119911	0.873205161
BMP4	0.092897012	5.104621619	0.445275083	0.504587325	0.715096411
S1PR1	0.166041474	5.105602366	5.234351019	0.022145069	0.106145385
FUT11	0.234890214	5.106345465	6.799572522	0.00911797	0.058063544
COQ9	0.183768073	5.106675614	4.795982208	0.028526187	0.125474048
ZIC2	0.074729844	5.107164026	0.318151242	0.572720805	0.764572517
MRPP3	0.116154137	5.107512157	1.848830137	0.173919518	0.393551431
RIPK1	-0.084098887	5.108076922	1.327570545	0.249237881	0.483462139

SYNJ1	-0.148750013	5.10820856	3.388935716	0.065635303	0.214698165
CDKAL1	0.152494101	5.109003411	3.484905785	0.061930887	0.207044441
PHF5A	0.18859217	5.109126024	6.618066591	0.010094936	0.062377741
LOC112530449	0.153549282	5.109545029	0.601286915	0.43808743	0.663886934
LRP8	-0.350737727	5.110046228	14.3018763	0.00015571	0.002921552
SORCS1	0.205538101	5.110921094	2.875277215	0.089949484	0.261736298
FAM173B	0.039856324	5.111344246	0.211128643	0.64588422	0.811051957
C14H7orf26	-0.082083673	5.111405986	1.381425381	0.239858502	0.472323431
APBA3	0.278455772	5.11250495	14.24789489	0.00016024	0.002986125
STMN2	-0.715134309	5.112608589	9.545393407	0.002004519	0.020186263
OSBP2	-0.209964223	5.112679421	8.300588814	0.003963223	0.032220766
MAP3K20	0.040533634	5.112745012	0.241758604	0.622938834	0.796956811
HOXC5	-0.026292061	5.11306151	0.08432764	0.771516197	0.88652336
ATF6	-0.038777968	5.113240661	0.2606973	0.609641505	0.787661037
DNAJC18	-0.0464433	5.113469424	0.507735926	0.476120602	0.693140273
RBL2	-0.157689646	5.113504198	3.408468798	0.064862607	0.213036765
CPNE3	-0.134235311	5.113788716	3.853696999	0.049636467	0.178841268
FBXO21	-0.069625466	5.113797637	1.01754521	0.313102001	0.551100049
ACOX1	-0.076071532	5.113877473	0.779964581	0.377151972	0.611974727
MAP7D2	-0.420770332	5.114597717	19.72535932	8.94E-06	0.000298416
SLC43A3	-0.035271004	5.114760988	0.194303579	0.659358665	0.818972436
MLA3A	-0.146552312	5.115099569	1.604770532	0.205228465	0.432066836
YAF2	0.092337718	5.115269843	1.430853985	0.231625118	0.46333471
RUNDC3B	-0.06835564	5.115441693	1.020577488	0.312382066	0.550288717
GATC	0.105305136	5.115461128	1.649459015	0.199032578	0.42483864
LOC422894	0.084914556	5.116095715	0.737405577	0.390493234	0.624023129
RBMS2	-0.028899538	5.116208667	0.158802731	0.690261213	0.838473731
CORO7	-0.207976693	5.116347169	7.349672977	0.006707496	0.04696674
SLC6A9	-0.109195399	5.116788864	1.36409962	0.242828243	0.475864052
AK1	-0.09287687	5.11738155	0.799472167	0.37125123	0.605641348
CUL4A	-0.076916521	5.117650324	0.968720619	0.32499946	0.563051813
PC	0.263856181	5.117746469	8.208059251	0.004170473	0.033475678

NPAT	-0.185047472	5.118214694	3.973112628	0.046232237	0.170495622
MCF2	-0.561613443	5.118486957	31.25989424	2.26E-08	1.83E-06
PPP6R2	0.136102474	5.118569603	3.629153125	0.0567757	0.195081258
NIT2	-0.019105413	5.119951895	0.056782331	0.811655839	0.909485654
FLAD1	0.197873555	5.120244019	6.271880246	0.01226689	0.07175673
MCM8	-0.003842252	5.121138185	0.002844087	0.957468989	0.981573571
HOXD8	0.001652023	5.12117644	0.000110122	0.991627226	0.996095557
CCNE2	-0.027089736	5.121506565	0.08626179	0.768984422	0.884767126
SELENOU	0.114040672	5.121544636	1.915316155	0.166373925	0.384172449
CCDC102A	0.175284063	5.12237664	2.918319759	0.087579016	0.256476892
ATP8A1	-0.169681085	5.123230242	3.144539231	0.076181515	0.235455828
OSTF1	0.064109453	5.123436282	0.676126665	0.410923693	0.641106415
MAN2B2	-0.332495243	5.124524187	12.51219354	0.000404305	0.006188682
GDAP2	0.067153095	5.125010449	0.916310868	0.338445486	0.57569508
COQ5	0.088870811	5.125314638	1.646129616	0.199486531	0.425421315
PTRH2	0.072867591	5.125897447	0.731058903	0.392540172	0.625896176
CRYZL1	0.110500338	5.126177908	2.461575622	0.116661705	0.307935251
KIF3C	-0.199280617	5.126449184	5.411795906	0.020001129	0.099042604
AFAP1L1	-0.058998307	5.126786769	0.621337903	0.430550547	0.658002159
TCP11L2	-0.392541385	5.126936583	20.01741146	7.67E-06	0.000264176
MAP2K3	-0.089722927	5.127676941	1.170592238	0.279279585	0.514618765
MYO18B	-0.078026154	5.127797103	0.786790209	0.375072464	0.609682643
EGFL7	0.135304528	5.128962671	2.036561666	0.153556398	0.366186078
CLK2	-0.139668016	5.129116609	3.426685994	0.064150709	0.211670325
STARD3	-0.178517494	5.129263239	6.857297326	0.008827998	0.056834133
AK4	0.035696237	5.129423946	0.209689572	0.647010823	0.811526992
PHTF2	-0.05074281	5.129806843	0.522414654	0.469813473	0.68897286
LOC107048987	-0.77922954	5.129810682	32.10654258	1.46E-08	1.23E-06
LHX1	0.324969914	5.13066789	6.936400935	0.008445888	0.055259282
CEPT1	0.099963223	5.131228029	2.070474744	0.150174768	0.361300171
FAM136A	-0.05136014	5.131963877	0.226192589	0.634361451	0.80403489
FGFR1OP2	-0.184437292	5.13216802	4.338157451	0.037267233	0.148842797

TACC1		-0.0317389	5.133675476	0.135534258	0.712761557	0.850568672
LOC112531536		0.306894247	5.133696018	12.50949132	0.00040489	0.006189294
HSPB1		0.181485882	5.133922724	1.993650804	0.157959679	0.372648962
LOC107052491		0.189192673	5.133938064	4.37172736	0.036539933	0.147260496
SHE		-0.033152239	5.134321573	0.16073825	0.688477736	0.837812539
USP32		-0.062147393	5.134648516	0.527202981	0.467785164	0.687329661
ING3		-0.054076502	5.134927491	0.226382011	0.634219587	0.80403489
TTF1		0.033986238	5.134942379	0.262864831	0.608158774	0.787414637
C1orf226		0.030885498	5.135046665	0.138947454	0.709329635	0.849591042
GABRA3		0.002625329	5.1352704	0.000371422	0.984623877	0.992968171
CLP1		0.109722361	5.136012497	1.693991435	0.193075645	0.417328557
UBAC2		0.145580714	5.136123764	4.791661538	0.028597827	0.125601973
KCNK1		-0.36783297	5.136227329	4.457664973	0.034744962	0.142491683
FANCA		0.123808375	5.13630215	1.499278348	0.220782433	0.450182768
DNAJC17		0.058797303	5.136590189	0.527883355	0.467498103	0.687214693
HAUS2		0.140077464	5.136603319	3.228633376	0.072361051	0.227375706
N4BP1		0.036213632	5.13695397	0.27109634	0.602597285	0.783728922
LOC416622		0.049745903	5.137005552	0.349111893	0.554616297	0.752428986
MCF2L		-0.250842429	5.137051915	8.79984314	0.003012564	0.02652314
ZCRB1		-0.0087351	5.137964899	0.017377419	0.895123948	0.951994866
STXBP5		-0.029356932	5.138165562	0.142517718	0.705790779	0.847659825
LOC101751749		0.118830068	5.138742035	1.947390735	0.162868302	0.379345755
C8H1ORF112		0.147136396	5.139169714	3.074115864	0.079547836	0.241482258
MICU1		0.180041071	5.13940797	7.024385309	0.008040707	0.053336266
MIEN1		-0.26726542	5.139859056	14.45510745	0.00014354	0.002746456
CWC25		-0.052264399	5.140558356	0.602847747	0.43749354	0.663498267
ACP2		0.056742083	5.140873665	0.739090646	0.389952332	0.623829051
CLVS1		0.054142425	5.140881343	0.419964427	0.516954795	0.722961303
TCEANC2		-0.156032536	5.141623299	2.696708326	0.10055566	0.279117395
B4GALNT3		-0.147635459	5.141870364	1.594380666	0.206701374	0.434057058
	12/set	0.303759468	5.14314892	12.1679596	0.000486175	0.007117441
MMRN2		0.07278958	5.143534947	0.713419488	0.398310957	0.630636659

IL1RAP	0.058928414	5.143680318	0.619057595	0.431397705	0.658562876
AFAP1	-0.154447663	5.143812142	2.122976055	0.145104345	0.354034665
NBN	0.077731938	5.144607128	0.628869228	0.427770392	0.655213795
RIMS4	-0.285972933	5.144923568	3.715384489	0.053913207	0.188894078
GLUL	-0.122411359	5.144961652	2.118627355	0.145516911	0.354656516
ROR2	-0.010767432	5.146395878	0.007726683	0.92995492	0.969181595
SPECC1	0.249953484	5.146531466	6.164856392	0.013031295	0.074844664
NSMF	0.060037362	5.147572084	0.435039086	0.509526648	0.717892027
RECQL	-0.286074455	5.147578432	15.97093192	6.43E-05	0.001470039
IL12RB2	0.298551154	5.147647481	14.74355449	0.000123168	0.002427218
ARNTL	0.028124207	5.147964524	0.176648346	0.674269563	0.828363869
ATP6V0B	-0.062191095	5.148041722	0.753175532	0.385472697	0.620034529
TMEM175	-0.09969882	5.148641939	1.122468126	0.289387623	0.525999484
LOC107051649	0.088603498	5.149022846	0.677627875	0.410404756	0.640661251
C5H15orf41	-0.113383983	5.14932301	2.04673539	0.152532958	0.364656375
LIMS1	0.149916609	5.150131005	2.86985142	0.090253202	0.262036199
KATNBL1	-0.06670654	5.150614464	0.93217821	0.334297557	0.572053485
MAST3	-0.209117767	5.151707328	4.876729188	0.027221155	0.121718505
ARHGAP22	-0.119200014	5.151882261	2.091594199	0.148111443	0.358220275
NDUFV3	0.086307228	5.15191606	1.184111017	0.276520661	0.51165495
IER3IP1	-0.133495351	5.152430285	2.13156792	0.144293093	0.353009642
FBXO34	-0.099592348	5.152955844	2.623691364	0.105279271	0.288309245
PCBD1	-0.038156404	5.15307715	0.294530824	0.587331594	0.773658452
SHH	0.189291043	5.153345444	1.506570048	0.219663214	0.448957667
TAPT1	-0.007897488	5.153443189	0.01080524	0.917210411	0.962184964
PROSC	-0.013484702	5.153757801	0.044681519	0.832590736	0.920860571
WDR89	0.030497516	5.153819087	0.131066669	0.717328633	0.853728044
NAGPA	0.106429987	5.153849489	2.50513378	0.113475848	0.302406036
LOC107051471	0.404423565	5.154651358	9.369623828	0.002206108	0.021546535
SEC22C	-0.20485713	5.155382205	6.917017435	0.008537926	0.0556182
RNPEP	-0.139610118	5.15545125	2.048164381	0.152389827	0.364526036
MOSPD2	-0.014588366	5.155488948	0.033076129	0.855685974	0.932779908

TDP2	-0.137044326	5.156254747	3.715208231	0.053918899	0.188894078
C24H11orf57	-0.300308065	5.156537043	18.80496301	1.45E-05	0.000437159
LOC100858649	-0.238218983	5.156914689	6.714801728	0.009561592	0.060097784
LOC112530907	0.224314813	5.15742678	4.506602756	0.033764239	0.140023865
FBXW4	0.082192123	5.157824803	1.202782402	0.272766274	0.507543368
COA7	-0.008940702	5.157909914	0.012454164	0.911141979	0.959234886
LOC107051250	-0.169571942	5.158162308	4.454648344	0.034806382	0.142543309
BBS4	0.076051753	5.158370046	0.531615506	0.465928458	0.686100024
B4GALT4	-0.230866849	5.158608516	10.45906525	0.00122049	0.013840977
SNRNP25	0.132957457	5.158615134	2.662421089	0.102744237	0.283626136
GPR155	0.325104199	5.158705952	24.45376085	7.61E-07	3.81E-05
THNSL1	0.159410022	5.15871785	3.058023408	0.080339344	0.242967083
VSIG10L	-0.051332496	5.159097566	0.152173756	0.696466635	0.842034358
TMEM181	-0.241735005	5.159197851	7.660381889	0.005644656	0.041576906
E2F5	0.044356567	5.159530973	0.392670253	0.530899133	0.733558252
PRRX2	-0.346381355	5.159953867	1.414914614	0.23424225	0.466133295
DAB2	0.172155413	5.160411709	1.812841685	0.178167719	0.398986093
LOC112530919	-0.284779487	5.160610832	5.977943395	0.014485884	0.080284685
GLTSCR1L	-0.098230114	5.160812548	0.619156613	0.431360867	0.658562876
PCSK7	-0.15717596	5.161630482	4.329397177	0.03745951	0.149356976
SPIRE1	-0.071008003	5.161784214	0.888014233	0.346016	0.582540186
ADAMTS14	0.088390838	5.161989981	0.8990957	0.343024303	0.580169072
DDB2	-0.131866315	5.162991458	3.370249825	0.066383688	0.216110872
SORL1	-0.109668032	5.163199316	1.877614244	0.170605552	0.389309994
SCAMP3	0.09395759	5.163364992	1.401599454	0.236455953	0.46878105
GMEB2	-0.081351593	5.163653658	1.228020827	0.267792286	0.503053957
E2F6	0.044825655	5.163843155	0.221852408	0.637632086	0.805931282
POT1	0.231773675	5.163954956	9.287833994	0.002306808	0.022102937
MB21D1	-0.049193299	5.166502545	0.423333071	0.515278575	0.722011561
ARL13B	-0.010609886	5.166729783	0.02042875	0.886346113	0.948791653
CCDC90B	0.156359365	5.167012026	5.587943476	0.018084516	0.092927773
MRPS18A	0.033187056	5.167218555	0.207910219	0.648410307	0.812426072

ROGDI	-0.087146744	5.168404718	1.651761881	0.198719299	0.424390145
CRBN	-0.108104523	5.168696092	1.713021832	0.190593772	0.414241252
ZHX1	0.159256279	5.168704408	2.857720048	0.090936307	0.263026356
LRP2	0.214224515	5.168751011	3.161973362	0.075372023	0.233726773
LOC107049543	0.143916233	5.168908604	1.906440676	0.167359171	0.385402706
AEBP2	0.022990538	5.169708109	0.065135302	0.79855613	0.901588892
HIF3A	-0.137341897	5.170046276	2.427536931	0.119220093	0.311642197
OPTN	-0.062454622	5.171201901	0.916275424	0.338454829	0.57569508
ANGEL2	0.176281285	5.171482403	7.185049045	0.007351354	0.049965525
LANCL3	-0.132200705	5.172373296	2.114148291	0.145943228	0.355040776
EEFSEC	-0.169031605	5.172380392	6.030661841	0.014059461	0.078729487
GAS7	-0.176740992	5.172398001	3.846586796	0.049847333	0.179259953
PPOX	0.254900945	5.17290751	9.329695892	0.002254698	0.021798502
LZTFL1	-0.099532722	5.173457442	1.644970664	0.199644835	0.425538311
CEP120	-0.075040788	5.174189466	0.826721689	0.363222203	0.599097263
SH3D19	0.007504487	5.174326275	0.011125612	0.915996514	0.96163855
ST14	-0.060049413	5.174531624	0.391584516	0.531467687	0.733850867
OSBPL10	-0.099903334	5.174553388	1.690479343	0.193537793	0.417989813
C20orf27	0.147866499	5.17500895	3.882567362	0.048789895	0.176579455
DERL2	0.003800713	5.175399606	0.002424634	0.960727578	0.9832549
WDR35	0.023694701	5.176023785	0.043113186	0.835512295	0.922151373
MERTK	-0.269425759	5.176121506	13.24590127	0.000273177	0.004586857
GK	-0.151205514	5.176186822	2.620751379	0.105474486	0.288590399
SNX19	-0.061675988	5.176536731	0.328150458	0.5667502	0.760302833
KANK3	0.110634722	5.17705834	1.271958679	0.259399554	0.493923796
PTCHD1	0.097083569	5.177210883	1.421897919	0.233091267	0.464685826
TMEM189	-0.084062372	5.177695951	1.412753406	0.23459985	0.46656279
LAMA4	-0.002511269	5.178481511	0.000595224	0.980535776	0.991750505
FBXO5	-0.014170253	5.178686766	0.020434994	0.886328864	0.948791653
PDP2	-0.148877969	5.179030444	3.446344785	0.063391815	0.210008519
ZNF703	0.107340738	5.179041586	1.094830826	0.295403327	0.532228922
TM2D2	0.033905455	5.179406468	0.185585964	0.666616058	0.823679784

ATF7	-0.120986876	5.180155332	2.447206553	0.117734223	0.309722885
LOC107049632	0.250509791	5.180201453	5.754227426	0.016449027	0.087765482
FZD10	-0.019817043	5.18046212	0.028329613	0.866336236	0.938298082
LSM6	-0.103565102	5.180659888	1.594518338	0.206681775	0.434057058
IFNGR1	-0.468566874	5.181548032	21.79612009	3.03E-06	0.000120808
ARL14EP	0.329536284	5.181556957	15.37501016	8.81E-05	0.001876389
ORC5	0.304360031	5.181725325	10.84889198	0.000988549	0.011928003
LIMK1	0.092497817	5.182230797	1.62886483	0.20186013	0.428705272
EED	0.292187709	5.182244177	16.24889414	5.55E-05	0.001307502
SLC7A6	-0.28236983	5.182294276	11.3300495	0.000762629	0.009851805
CRAT	-0.092729276	5.182618612	1.805096771	0.179097539	0.400196905
STON1	-0.052792432	5.183561536	0.497868074	0.480438373	0.696466157
NEK7	0.024209329	5.183747477	0.137541897	0.710737032	0.849781439
C26H6ORF125	0.060610421	5.183824414	0.683392167	0.408421085	0.638837354
TMEM186	-0.196168563	5.184127246	5.144351946	0.023322114	0.109866986
DCUN1D4	-0.101451567	5.184420417	2.061400138	0.151071304	0.362690152
TBCCD1	-0.052078878	5.184662409	0.442024373	0.506146992	0.715827138
PGRMC2	-0.068714009	5.184748817	0.725352245	0.394393868	0.627445382
ECH1	0.144236507	5.184931655	1.984175148	0.158951254	0.37396064
MANBAL7	0.67895466	5.18503385	11.29647887	0.000776542	0.009975021
TMCC2	0.16097998	5.18541788	3.798697268	0.051292475	0.182569259
CHEK2	0.075106286	5.185905178	0.777527801	0.377898294	0.612700264
MRPL14	0.167083682	5.185923789	3.713089538	0.053987374	0.189018673
B4GALT6	0.270380739	5.186278814	10.08057482	0.001498406	0.016178653
IFT74	0.29673718	5.186722141	9.289492296	0.00230472	0.022102937
SMIM10L1	0.061906	5.187314272	0.73824367	0.390224075	0.623951561
LOC107049913	0.166600107	5.18825214	3.146498458	0.076090081	0.235242786
WWTR1	-0.006492237	5.188516643	0.008628078	0.925992978	0.967012404
CNOT4	0.089591147	5.188659329	1.745647284	0.186424866	0.409285755
ELP2	0.267844528	5.188732106	15.54207109	8.07E-05	0.001763271
NT5DC1	-0.065842445	5.189171668	0.676571692	0.410769755	0.640927017
SELENOP1	-0.035546483	5.189747465	0.052834769	0.818201997	0.914082005

ASXL2	-0.043761921	5.190093619	0.329868053	0.565736794	0.759500607
GNPTG	-0.15775222	5.190252187	4.781735639	0.028763116	0.126044495
COG8	-0.085581728	5.19045442	0.897135335	0.343550996	0.580582484
MCUR1	0.049060521	5.190603303	0.41161847	0.521149175	0.726233326
SSTR2	-0.153495556	5.190621619	1.547549809	0.21349752	0.442066402
PRKX	-0.098035206	5.190889774	1.406450013	0.235646606	0.467852909
TIMP2	-0.23738263	5.191038818	5.944517212	0.014763134	0.081172118
MAN2C1	0.052747294	5.191669484	0.1371782	0.711102536	0.849781439
VWA8	-0.077101007	5.191902313	0.604585109	0.436833928	0.662986744
FECH	0.02103762	5.192417313	0.029527976	0.863565628	0.936653643
LOC424111	-0.038562905	5.192766188	0.235527271	0.627455386	0.799999777
TECPR2	-0.046056533	5.192853012	0.424550403	0.514675171	0.721735682
PTPRO	-0.581963768	5.193038252	16.70447811	4.37E-05	0.001069521
DQX1	-0.00891891	5.193727203	0.014110676	0.905443025	0.956847022
PITPNM1	0.054047944	5.193807612	0.368388455	0.543884011	0.74417397
SFI1	-0.414859229	5.194052883	34.12785512	5.16E-09	4.86E-07
BAHD1	-0.156500528	5.194128054	4.628092314	0.031452517	0.133492691
NHLRC3	0.031528103	5.194290363	0.160401713	0.688786939	0.838002889
CHD5	-0.235361646	5.194323415	5.683733822	0.017122891	0.090018266
WIPF1	-0.116775278	5.194445653	2.448712079	0.11762134	0.309524893
PAX6	0.206794509	5.19512125	3.260287858	0.070976666	0.224960367
CCNE1	0.048924019	5.195824018	0.39278026	0.530841588	0.733558252
TBPL1	0.128120874	5.196087752	2.659171176	0.102954369	0.283905528
CWF19L2	-0.155169511	5.19644894	5.655796663	0.01739777	0.090911496
DDX25	-0.184739557	5.196526739	6.258406852	0.012360531	0.072176202
DARS2	-0.171445008	5.196663459	5.479422278	0.019241617	0.09670764
TTPAL	-0.299338625	5.197627848	17.94404399	2.27E-05	0.000627041
LHFP	0.06728767	5.197642393	0.571089989	0.449826109	0.673300375
LOC419074	0.005293689	5.197975346	0.005771791	0.939441132	0.973825364
TANGO2	-0.027855886	5.197988666	0.176463165	0.674430526	0.828437915
RECQL5	0.241177088	5.198224796	7.492785869	0.006194665	0.044492889
STK3	-0.039451666	5.198393904	0.253486231	0.614630942	0.791187683

B3GALT6	0.13941099	5.198515141	3.119808451	0.077345875	0.237684649
A2ML1	0.018752597	5.198562473	0.043314693	0.835133848	0.921947852
PCNX4	0.038139807	5.1988712	0.285750968	0.592956366	0.777327412
TSSC1	-0.158102464	5.199052715	4.981187586	0.025624387	0.116865103
IFFO1	-0.096617672	5.199298019	1.957210207	0.161811996	0.377783257
MASTL	-0.187368116	5.199774206	4.843878295	0.027744431	0.123421092
MAPRE3	0.118617947	5.200134685	1.944040183	0.163230528	0.379719374
OGFOD3	-0.501311442	5.200323099	29.49267696	5.61E-08	4.12E-06
BROX	0.315202028	5.201283625	14.80665133	0.000119115	0.002352979
DPM2	0.072461391	5.20164855	0.716649497	0.397245139	0.629797549
HPS6	-0.168599699	5.201888368	5.540984948	0.018576125	0.09445925
TTC36	0.003480193	5.202397398	0.002526421	0.959912395	0.982787361
FKBP7	0.020978943	5.203345475	0.065677658	0.797737321	0.901282145
POLL	-0.09801356	5.203802737	1.803080595	0.179340512	0.40068542
MRPS14	0.265269413	5.203929549	10.43333227	0.001237612	0.013977291
PLEKHM1	0.000974532	5.204511895	0.00020019	0.988711217	0.994817358
MRPL58	0.022006426	5.204717965	0.080016068	0.777275637	0.889554058
AFAP1L2	0.128806714	5.204864817	1.663876637	0.19708072	0.422518563
PAQR9	-0.168857586	5.205632918	1.611582277	0.204269525	0.431078698
ATG12	-0.052812576	5.206605461	0.405122708	0.524455465	0.729062657
SMURF1	-0.005420487	5.206716551	0.006457319	0.935952969	0.972046586
PXK	-0.027647431	5.207532067	0.131066594	0.717328711	0.853728044
PBX4	-0.15735656	5.207680208	5.962053536	0.014617007	0.080754108
LRRC28	0.244436434	5.207781891	13.39970152	0.000251664	0.00432269
SERINC3	-0.08532562	5.208832938	1.056775312	0.303951815	0.541885862
AvBD10	-0.638025975	5.209058115	4.296874477	0.038182484	0.151104562
RARRES2	-0.303014703	5.209288785	6.014414788	0.014189483	0.079202153
SOBP	0.007628547	5.209531886	0.008220233	0.927758384	0.967994498
SKIDA1	0.188396929	5.210417222	5.34450692	0.020787662	0.101743301
RMND1	-0.350376102	5.21068831	33.30907479	7.86E-09	6.95E-07
NIPSNAP3L	0.096626138	5.210728855	0.820525659	0.36502652	0.600737409
KCNK5	0.017998236	5.210755305	0.03894566	0.843556355	0.926227243

PIAS4	0.087918129	5.211821087	1.581346471	0.208566852	0.43619313
TMEM104	-0.205301494	5.211953684	5.434334639	0.019744611	0.098453812
MTHFR	0.093011111	5.212084466	1.552100815	0.212825571	0.441119131
FRMD4B	-0.120106519	5.212088782	1.759494174	0.18468762	0.407268131
NUDT14	-0.124968933	5.212159723	3.643648923	0.056283452	0.193787499
ANGPTL4	-0.160010187	5.212311554	3.546257684	0.059679908	0.201438065
PCDH10	-0.424622458	5.212683303	2.236223118	0.13480991	0.337831175
SPRY1	0.172372695	5.212737977	2.688733931	0.101060084	0.280098313
TFB2M	-0.164386265	5.212887374	5.154068853	0.023191968	0.109441877
MPHOSPH9	-0.236280872	5.212962902	9.917524371	0.001637124	0.017257451
MED28	0.07912803	5.213611462	0.997319786	0.317959911	0.555837854
SYAP1	-0.129305223	5.213895095	3.807707042	0.051017246	0.182022719
CEP290	0.030019594	5.213969724	0.122297142	0.726556286	0.859179567
RNF7	0.007715519	5.214922213	0.01158734	0.914277689	0.960814878
ZFAND4	-0.1555434	5.215813113	4.609776583	0.031790175	0.134578679
NBEAL1	-0.116452096	5.216348955	1.239512772	0.265564975	0.50073011
GRK4	0.095311213	5.216601278	2.046982651	0.152508181	0.364656375
SEMA3D	0.168494013	5.217505654	3.787482863	0.051637247	0.183378723
C17H9orf16	-0.00378397	5.217534645	0.003099618	0.955601344	0.981041173
COG5	0.099225908	5.217693763	2.522784404	0.112212291	0.299960737
CDK18	0.18812427	5.217883982	3.401604921	0.065133018	0.213625062
ACVR2B	0.124643232	5.21795876	1.527116528	0.216545719	0.445574566
MRPL34	0.128488402	5.218306967	3.195634243	0.073835125	0.230367269
QTRT1	0.133473551	5.218761284	2.581683259	0.108106646	0.293044633
RNF215	0.068028096	5.219024371	0.815128313	0.366608394	0.601629712
ATRAID	0.283520883	5.219363123	5.025291704	0.024979718	0.115008744
ATMIN	-0.174077506	5.219737002	3.355186182	0.066993635	0.217132216
DYNC2H1	0.050198017	5.219764909	0.39434969	0.530021836	0.733281956
WDR37	-0.050393533	5.219938043	0.306363334	0.57992041	0.769067565
FAHD2AL	0.079721241	5.219954739	0.490285434	0.483800006	0.698351687
HACE1	0.123049663	5.220074817	3.006323176	0.082940231	0.247845934
CHPF2	-0.098619159	5.220296031	1.501919986	0.220376177	0.44987235

ATP23	0.153202957	5.220349404	4.189662688	0.040671189	0.158073742
LOC112533544	0.532925187	5.220618271	48.46844189	3.36E-12	7.46E-10
LOC107050992	0.023991022	5.220776523	0.124445429	0.724262198	0.857699494
MRPL53	-0.122003287	5.221511978	2.128279722	0.144602963	0.353434606
PSRC1	0.268326947	5.221659308	15.16369493	9.86E-05	0.00204028
AKAP13	0.142273023	5.221927703	1.359295795	0.243659564	0.47663078
L3MBTL1	0.076847104	5.221929485	0.963656354	0.326267378	0.564154365
PCDH11X	-0.224459613	5.221977086	3.676049889	0.055199479	0.191495608
GTF3C5	0.071000406	5.222203795	0.865180072	0.352293134	0.588825141
LOC107052240	0.037599339	5.222318731	0.112951588	0.736808496	0.86564214
TMEM245	-0.10775708	5.222327279	0.963810786	0.326228617	0.564154365
FXYD6	-0.1305815	5.222715533	1.70114974	0.192137685	0.416277235
DACH1	0.268555108	5.222750571	10.34385652	0.001299063	0.014482442
RALGPS1	0.170174393	5.223060664	4.723203319	0.029758257	0.128556609
POLR3F	0.072200389	5.22340966	1.008726313	0.315208168	0.552589063
STX8	-0.05230165	5.223542729	0.448908434	0.502853778	0.71365807
LOC107053735	-0.147494795	5.223571762	1.671989978	0.195992205	0.420708925
CCSER2	0.024619046	5.223607035	0.099579477	0.752334863	0.874596167
GMEB1	-0.060145439	5.223765883	0.864922469	0.35236483	0.58884782
CYTH1	0.117806331	5.223943165	1.729878045	0.188426473	0.411979486
RHBDF2	-0.089587677	5.224459737	1.286082702	0.256771116	0.491584492
CUL9	-0.346170309	5.224717172	18.77379663	1.47E-05	0.000441929
GFM2	0.419978959	5.224749672	16.42403035	5.06E-05	0.001205887
TTBK2	0.136197619	5.225558726	2.531613192	0.111586084	0.299407232
PPP3CC	0.052947961	5.226155444	0.358041929	0.549595714	0.748347183
BCKDHB	0.06121095	5.226196476	0.645037506	0.421892508	0.651181055
SLC4A3	0.030091918	5.22639366	0.168402201	0.681535743	0.83319824
CCDC39	-0.176688417	5.226671671	3.978127383	0.046094782	0.17025581
RPP25L	0.07833632	5.227088054	0.812644939	0.367339427	0.602348807
SCAMP1	0.149033265	5.227094718	3.061576812	0.080163842	0.242614681
SLIRP	0.121466261	5.22715896	2.295920075	0.129714325	0.329390312
CDC14B	-0.145920595	5.227187647	1.772509019	0.183071873	0.405538517

RAB4A	-0.3852936	5.228082102	23.30024165	1.39E-06	6.21E-05
ATPAF1	-0.180566001	5.228284188	7.590566126	0.005867452	0.042701868
HS6ST1	-0.188683265	5.22867264	6.013216895	0.014199119	0.07922906
DIMT1	0.07581367	5.229153276	0.611089986	0.434377706	0.660965892
ZNF410	0.016138737	5.229248415	0.049898619	0.823239778	0.916737351
RNF115	-0.112758704	5.229314511	2.356813479	0.124736753	0.320709885
FAM114A2	0.078246074	5.229603563	1.249819706	0.263586915	0.498714809
RNF139	-0.071142374	5.229906154	1.21533014	0.270279065	0.505046787
THAP12	0.162561845	5.230025418	5.425353592	0.019846416	0.098662471
ZHX3	-0.004146363	5.230725166	0.002252283	0.962148025	0.983899168
CARS2	-0.122333015	5.230938475	3.088389398	0.078852821	0.239970995
CCDC28A	0.021839888	5.231035578	0.091463734	0.762324503	0.880756463
KIAA0355	-0.04576648	5.231713283	0.30099137	0.583261588	0.770936649
ALDH5A1	0.006875095	5.231869278	0.006738332	0.934577255	0.971393237
CHD1L	-0.025126279	5.231901438	0.078734448	0.779019829	0.890625824
LOC112530279	0.270765124	5.232012937	5.382053893	0.020344913	0.10024498
GPATCH11	0.240815777	5.233412022	10.159437	0.001435645	0.01566242
ARHGEF1	0.072549196	5.233593902	0.827481397	0.363001822	0.599097263
FAM188A	-0.094566087	5.233959435	1.685792327	0.194156563	0.418721657
ADORA2A	0.01656297	5.234398008	0.032143945	0.857712099	0.933726046
ADAMTS17	0.053957681	5.234974623	0.190453083	0.662539787	0.820943839
SOX21	0.228581489	5.235949926	3.055170114	0.080480567	0.243240242
EPG5	-0.282841531	5.236178978	9.347144345	0.002233332	0.021699324
LOC100859454	-0.033494734	5.236691334	0.215348496	0.64260722	0.809098003
MIGA2	0.024604223	5.236836472	0.092710648	0.760759004	0.880207468
GEMIN8	0.157538642	5.237858688	4.413118027	0.035663598	0.144864108
SEMA4D	-0.086253101	5.238191808	0.975023496	0.323430521	0.561101658
MDFI	-0.001480891	5.238658365	0.000118533	0.991313366	0.995986157
NEXN	-0.311364763	5.238856771	9.352533986	0.002226774	0.021681398
MMP24	-0.139781213	5.23977366	2.504921735	0.113491123	0.302406036
TMEM128	-0.015175301	5.240148864	0.033867557	0.853988819	0.932178863
PANK2	-0.097845017	5.240905524	1.253754743	0.262836552	0.497982206

RTCA	0.003494244	5.241118381	0.003044878	0.955994733	0.981041173
CCZ1	-0.118000323	5.241181625	2.351273862	0.125180677	0.321549803
BCKDHA	0.194026398	5.241494771	5.266822884	0.021735648	0.10493777
RAB9B	0.081035919	5.241754838	1.170031974	0.279394671	0.514715552
FAM234B	-0.05061375	5.242066346	0.445373586	0.504540192	0.715091203
AARS2	-0.038017689	5.242271085	0.292026861	0.588924588	0.774881119
CTTNBP2NL	-0.278490556	5.24228428	4.736349981	0.029531663	0.127899143
EMX2	0.054628672	5.242610751	0.251583656	0.615962178	0.792054659
FBXL3	-0.196271602	5.24289829	6.817327588	0.009027756	0.057712403
MRPL47	0.094777771	5.243167908	1.972029503	0.160232606	0.375748545
SGCG	0.106981391	5.243371249	0.887388254	0.346186049	0.582582475
PSMD10	0.140239976	5.243411082	3.263873664	0.070821642	0.224671317
PCNX2	-0.17793001	5.243916803	4.007893844	0.04528769	0.168332719
GPR1	-0.459848663	5.244045431	39.83930977	2.76E-10	3.63E-08
LOC107051419	0.118587289	5.244665051	2.1191589	0.145466412	0.354656516
LOC107052392	0.180788704	5.244850202	4.142535873	0.041818478	0.16073886
MEOX2	0.226233938	5.245712626	4.212431451	0.040128777	0.156957222
CRTAP	-0.090991358	5.246244897	1.51573816	0.218265578	0.447323462
WBSCR17	-0.074182024	5.246253434	0.242004385	0.622762176	0.796854712
NKAIN1	0.098502528	5.2465779	1.397229675	0.237187964	0.469553411
SLC16A3	0.446867022	5.247047944	10.6567209	0.001096717	0.012826205
PRMT7	0.257763935	5.248569637	9.494632905	0.002060738	0.020613645
UBE2T	0.098390502	5.24959129	1.686885788	0.194012	0.418684257
NDUFAF6	-0.013277386	5.250367329	0.027429694	0.868456698	0.939356373
SDF2	0.020423328	5.250856934	0.071513334	0.789145978	0.896286379
POLE4	0.377249332	5.251144577	24.69622038	6.71E-07	3.47E-05
FANCG	-0.278550062	5.251725731	7.837835699	0.005116383	0.038797271
NKAP	-0.008288284	5.251730084	0.017870522	0.893655089	0.951501845
ELL	-0.213666492	5.252158862	6.004978703	0.014265566	0.079368477
KBTBD4	-0.116232322	5.253017955	3.270410591	0.070539967	0.224259874
SEMA6B	0.104056759	5.253515261	1.417887126	0.233751485	0.465438127
POU3F3	0.23913267	5.254180178	4.058195715	0.043957443	0.165511342

KREMEN1	-0.03256838	5.254288459	0.153118311	0.695573024	0.841406609
RAB9A	0.060429217	5.255019679	0.520513885	0.470622555	0.689405693
BBS7	-0.115072761	5.255262567	2.71173807	0.099612408	0.277394173
LCMT1	0.112449742	5.255535826	2.600420325	0.106835377	0.290914656
ATOX1	0.002414009	5.255555175	0.000666197	0.979408259	0.99115393
WIPF2	0.079662497	5.255704147	1.194102353	0.274503622	0.509412101
POLG	0.158063532	5.255775854	2.485312784	0.114913501	0.304493021
PPP1R21	-0.096692724	5.255833258	1.532288589	0.215769293	0.444608321
CAMK1	-0.21346273	5.256105955	9.042161154	0.002638236	0.024104481
TATDN1	0.120685258	5.256768946	3.330560928	0.068003678	0.219368855
EMB	0.018901957	5.25695179	0.02823526	0.866556918	0.93840635
PDRG1	-0.179117112	5.257639243	5.259079215	0.021832567	0.105137513
ZNF513	-0.118615565	5.257669745	2.231924154	0.135185403	0.338271869
SNUPN	-0.132128734	5.258008915	3.454619594	0.063075247	0.209339088
NANP	-0.261691594	5.258096651	12.6147958	0.000382705	0.005913067
ZNF131	0.146479263	5.25816583	1.373306895	0.241244525	0.474048084
SDHB	0.160299618	5.259510688	4.30837607	0.037925149	0.15049741
DDX11	0.149977169	5.260170992	4.323992167	0.037578662	0.149614536
RILPL2	0.189493316	5.261075996	5.412889155	0.019988607	0.099040208
PRR5L	-0.267906447	5.26109711	10.58253031	0.001141613	0.013229044
STK24	-0.18579695	5.261359948	5.165799471	0.023035853	0.108894354
ZNF143	0.034857507	5.261701667	0.203065727	0.652257577	0.814579419
MON1A	-0.134347377	5.261797387	3.414160833	0.06463927	0.212600278
ZNHIT3	0.07661855	5.261802808	0.887650661	0.346114752	0.582540186
FLOT1	-0.110186189	5.262099364	1.631625133	0.201478417	0.428133421
SH3PXD2A	-0.076798325	5.264109876	0.459461688	0.497875661	0.709527196
SORD	0.093323209	5.265149671	1.701183366	0.192133291	0.416277235
MTG1	-0.011921604	5.265237013	0.017487141	0.89479529	0.951827123
HIPK3	-0.164950189	5.265268633	3.107332009	0.077940537	0.238280562
ADAMTS7	0.070022554	5.26556906	0.44325275	0.505556656	0.715440088
SEL1L3	0.274855535	5.26639609	15.97726832	6.41E-05	0.001467164
RABIF	0.059934054	5.266515969	0.551422858	0.457736897	0.680402949

OLFML2B	-0.179767204	5.266574147	2.062988834	0.15091391	0.362396975
ECSIT	0.071664569	5.2669912	0.587440706	0.443410492	0.668222332
XYLT2	-0.108390652	5.267222391	1.460528645	0.226846026	0.456755952
ABHD17C	0.032312391	5.267551362	0.201302602	0.653671471	0.815062002
MLH1	-0.087236579	5.268569441	1.464315424	0.226244738	0.456254766
TMX2	0.049986373	5.268915219	0.55823168	0.454973604	0.67768554
LOC107050229	-0.055310643	5.269424091	0.542932223	0.461220052	0.682804855
MANBAL14	-0.36160722	5.269762931	7.10367534	0.007692605	0.05175054
CACNA1G	0.210708328	5.27020646	4.498686141	0.033920907	0.140278594
EXOSC8	0.047225617	5.270434721	0.377971709	0.538690747	0.739789371
PMS1	0.075904673	5.270525945	1.10475521	0.293224898	0.530454672
PNKD	0.062679231	5.270855852	0.579406403	0.446545086	0.670621395
MRPS28	-0.182977154	5.270870041	6.209211627	0.012708722	0.073556811
MAMLD1	0.108953933	5.270896015	1.950843089	0.162496029	0.378860143
FAM151B	-0.09517165	5.272294359	0.819296631	0.365385895	0.600872977
ERRFI1	0.002300554	5.272582601	0.000700192	0.978889532	0.991002256
ZNF236	0.013767137	5.27277255	0.038753808	0.843937199	0.926328616
PELO	0.211459328	5.27321822	3.968626885	0.046355559	0.170842892
CEP135	-0.060217523	5.273577189	0.511103849	0.474661398	0.692264899
MFSD6	-0.033804168	5.274665008	0.217207439	0.641175998	0.808161704
CDC6	0.138410298	5.275099816	3.280139033	0.070122988	0.223628159
SIRT3	-0.018318849	5.275268073	0.068252424	0.793898451	0.898895155
OTUD5	0.048328511	5.275421553	0.567377772	0.451302806	0.674763702
PAPD4	0.208563734	5.275863327	4.596901445	0.032029795	0.135293541
TRIR	-0.03050438	5.276067124	0.148676855	0.699803023	0.843917827
TRIM32	0.076730453	5.276474343	1.005176506	0.316061178	0.55362856
EDEM2	0.461466476	5.276781268	43.29733302	4.70E-11	7.66E-09
SLC9A6	-0.134530129	5.276840276	4.131039656	0.042103477	0.161381949
PRR7	0.316192512	5.278579592	20.09519081	7.37E-06	0.000254179
RABL3	0.052605861	5.280005299	0.422630861	0.515627207	0.722215551
LOC112530910	0.390103887	5.280409495	12.71877366	0.000362003	0.005667708
TFAP2B	0.071624727	5.280439065	0.563420449	0.452885365	0.675632701

LOC107052085	0.266985082	5.280632013	8.219805753	0.004143564	0.033299601
CPNE2	0.226677612	5.280956031	5.942176398	0.014782753	0.081245895
MNT	0.090427143	5.281960519	0.715663644	0.397570007	0.630009097
LOC107057310	0.190686127	5.281970838	8.560781539	0.003434819	0.029103992
BORCS5	-0.018821464	5.282033788	0.09266686	0.760813784	0.880207468
AARSD1	-0.098180149	5.282421513	1.739956425	0.187144348	0.410102577
CDK5RAP3	0.146578455	5.282793122	3.764926964	0.052338144	0.184886683
ADCY3	-0.183934251	5.282898161	6.520390764	0.010664457	0.064873803
LCORL	-0.053007079	5.283021668	0.69630527	0.404027969	0.635646128
LOC101747869	0.086350532	5.283081379	1.119711306	0.289980626	0.526868171
DYRK1A	-0.008010948	5.283451614	0.008461313	0.926709642	0.967217925
RBBP7	0.194936257	5.28377164	6.118495608	0.01337746	0.076247352
SLC12A7	-0.027350042	5.283932776	0.071141992	0.789681241	0.89658489
DRAXIN	-0.017283953	5.284033449	0.017535325	0.894651295	0.951827123
PSMG2	-0.077689084	5.284059516	1.376927837	0.240625142	0.473216236
MSTO1	0.118779621	5.284538813	2.649781147	0.103564153	0.284879329
CD93	0.123093689	5.285293738	1.587727174	0.207651158	0.435218419
IVD	0.380899288	5.28536254	29.37211993	5.97E-08	4.33E-06
SGCE	0.141994748	5.285455442	4.150345238	0.041626036	0.160186254
TRIM13	-0.134334084	5.285753223	3.268915444	0.070604286	0.224327772
SOCS6	-0.066318403	5.285789434	0.799116003	0.371357802	0.60567622
COG2	-0.206714962	5.285911249	11.53973399	0.000681244	0.009098919
INPP5F	-0.050487915	5.285954744	0.603018299	0.43742872	0.66346111
ITGB3BP	0.405691039	5.286172136	25.50564846	4.41E-07	2.43E-05
NDRG1	0.380130409	5.286353702	19.23987381	1.15E-05	0.000365498
AKTIP	-0.149422099	5.286608078	3.258189205	0.071067565	0.225190983
PRKAA1	-0.051113093	5.287579585	0.34611931	0.556318158	0.753031308
RNF144A	0.042456028	5.288430508	0.210109997	0.646681203	0.811526992
TUBGCP5	0.121303696	5.288829408	3.541944228	0.059835286	0.201857819
MOGS	0.162137319	5.290239113	3.933551367	0.04733189	0.173124253
RTTN	0.027631494	5.290322199	0.103368851	0.747823734	0.872702382
CTSZ	0.265977647	5.290711344	13.07761506	0.000298846	0.004912594

CCDC66	-0.186729155	5.290936017	5.626568409	0.017690229	0.091711634
ATG4C	0.045700067	5.291460228	0.486125229	0.485660861	0.700316086
TMEM231	0.019399598	5.291952421	0.062570007	0.802479103	0.904105326
DCBLD1	-0.14838018	5.292273605	3.881423863	0.048823135	0.176645709
GNG10	0.189696801	5.292364789	2.712968737	0.099535601	0.277321083
INPP5B	-0.136430534	5.29360265	3.578734346	0.058523724	0.199215532
ARMC1	-0.29514774	5.294127723	9.777651952	0.001766458	0.018315727
DDT	0.042581162	5.294304057	0.268831793	0.604116494	0.785137502
SNTB1	-0.078093836	5.29450919	0.543984199	0.460786219	0.682585354
OTUD7B	-0.025264473	5.294664011	0.168999536	0.681002487	0.832966321
MXI1	0.198565423	5.295091307	8.434981805	0.003680706	0.03072857
STN1	0.661765495	5.295759082	52.66868933	3.95E-13	1.08E-10
WRN	0.177269831	5.296861834	6.761307765	0.009315547	0.058956713
DEPDC5	0.179273923	5.297233925	6.952900795	0.008368343	0.054948559
UHRF1BP1L	-0.106072811	5.297295933	1.703818886	0.191789306	0.416070275
SPATA6	-0.070802623	5.297413862	0.763003609	0.382390182	0.616769322
WBSCR22	0.031153452	5.298440378	0.15109019	0.697495703	0.84267928
BNIP3	-0.046665424	5.298813472	0.403725706	0.525171393	0.729911257
RDM1	0.240182187	5.299082267	5.769414278	0.016307469	0.087264846
PCMTD2	-0.294805052	5.299147023	15.78214705	7.11E-05	0.001595438
MYNN	-0.149265807	5.300081869	4.169642079	0.041154493	0.159228116
RNFT1	0.168912932	5.300960931	5.285037322	0.021509431	0.104129947
PRR14L	-0.237994075	5.301071985	2.985543681	0.08401107	0.24993711
PREB	0.046813126	5.30107878	0.260284815	0.609924551	0.787717486
GCC2	-0.153992849	5.301079543	3.294449658	0.069514399	0.222238088
ZNF839	-0.060780643	5.301317594	0.622699015	0.43004608	0.65753654
TMCO6	-0.156210953	5.301341428	3.458100447	0.062942585	0.208983099
POLH	-0.201907992	5.30143117	8.870663705	0.002897898	0.02580352
PDZD8	-0.119633221	5.301599283	3.173943043	0.074821607	0.232841726
CAND2	-0.012952289	5.301684415	0.039809672	0.841853174	0.925494032
MAFK	-0.026730408	5.301778541	0.110020222	0.740121115	0.867860967
ASAP2	-0.160280299	5.30211172	4.507470856	0.033747106	0.140023865

LAMTOR3	0.021941832	5.302330565	0.092481732	0.761045542	0.880351803
AMDHD2	0.172827736	5.30254513	5.232976641	0.022162573	0.106170104
ANKMY2	0.021059758	5.302665371	0.090897991	0.763038636	0.881102924
PRDM15	-0.053150858	5.302962858	0.638627717	0.424208158	0.65309226
ITGB3	0.088612707	5.303122577	0.748795896	0.386857725	0.621136991
GLYCTK	-0.310914984	5.303348007	10.84585556	0.000990171	0.011936459
HEXA	0.106534321	5.303902353	3.223993673	0.072566386	0.227747449
KCNAB2	0.124223577	5.304349946	2.262490715	0.132540738	0.334143994
LRP5	-0.021017154	5.304863352	0.074376168	0.785068503	0.89437421
C6H10ORF88	0.02584923	5.304975331	0.151596074	0.697014735	0.842283724
LDAH	0.053023497	5.305341126	0.576904566	0.447528196	0.670925335
ELOVL1	0.073314083	5.305761828	1.381305629	0.239878876	0.472323431
AGBL5	0.059323074	5.306608727	0.759971954	0.3833373	0.618056281
SMIM12	0.141914684	5.306970117	3.240063883	0.071857836	0.226560777
CSGALNACT2	-0.112831392	5.307735093	1.676171366	0.195433966	0.420319684
P3H2	-0.027184511	5.308681328	0.091687159	0.762043141	0.880756463
RPS19BP1	-0.134718559	5.309197203	3.306659654	0.068999613	0.221280185
ZNF407	0.074425095	5.309600464	0.742049838	0.389005032	0.622950073
LOC418927	0.105653931	5.309724032	2.1822486	0.139610355	0.34532758
ALPL	0.300445023	5.309911443	14.06150662	0.000176928	0.003239391
TRIM62	-0.006537671	5.309967797	0.006850469	0.934036365	0.971309744
TMEM38A	-0.249452704	5.310086895	12.36115572	0.000438359	0.006563423
NEMP1	-0.039176307	5.310641583	0.226303172	0.634278623	0.80403489
GSTK1	0.517671286	5.311559871	53.21372047	2.99E-13	9.12E-11
CDYL	0.219140534	5.311840774	7.644586031	0.005694296	0.041755635
ZMAT3	-0.015563269	5.311958866	0.057096397	0.811145499	0.909337575
COPS9	0.358318722	5.312107289	26.86142021	2.19E-07	1.32E-05
LMLN	-0.159904329	5.312232291	4.832261038	0.027931976	0.123953794
ADAT1	0.012423669	5.312373509	0.012708852	0.910241808	0.958900701
NATD1	-0.194281488	5.312946089	4.343248008	0.037155977	0.148542664
EXOC8	-0.17310996	5.312994399	6.152574828	0.013122092	0.075234853
COQ6	-0.056664287	5.313070354	0.590985168	0.442038413	0.666826374

GINS4	-0.061327795	5.313455323	0.991614928	0.319347992	0.557132232
LOC423110	-0.08410114	5.31353621	1.496970305	0.221138119	0.450573025
SLC25A24	-0.202459893	5.313870707	8.749272893	0.003097255	0.027022977
EFNA2	-0.131378129	5.314178076	2.398101144	0.121482629	0.315600463
RNFT2	0.163272784	5.314769563	3.069199787	0.079788738	0.24196898
PDSS1	-0.162791352	5.31480762	7.547425203	0.006009588	0.043390863
SLC25A25	0.057128757	5.315661511	0.647920562	0.420857115	0.650469233
GGNBP2	-0.12267688	5.316429349	3.243834136	0.071692679	0.226300218
MSH3	0.130562349	5.316460508	1.592387604	0.206985343	0.434352468
TMEM50B	0.029005665	5.316965412	0.208118539	0.648246088	0.812282164
ZBTB21	-0.095535527	5.317136318	0.789128605	0.374363746	0.609011908
LYST	-0.07731358	5.317141948	0.825382124	0.363611244	0.599401224
C14H16orf45	0.087957281	5.317392513	1.87096056	0.171365106	0.390069556
E2F3	0.010672455	5.317821871	0.025814787	0.87235347	0.941530654
WHAMM	-0.008205603	5.317854172	0.007498231	0.930995566	0.969780408
APOA1BP	-0.100152822	5.318019906	0.909406079	0.340272052	0.577392087
RDH12	0.083819255	5.318079114	1.125096074	0.288823778	0.525322788
WWP1	-0.351864758	5.318167762	14.59677343	0.000133142	0.00257445
DFFA	0.005326615	5.31821586	0.004999944	0.943628335	0.975911026
TXN2	0.074252462	5.320270016	1.04479327	0.306709299	0.544776458
NMD3	-0.050759874	5.320396547	0.570676344	0.44999028	0.673300375
HPS3	-0.016684902	5.321027692	0.033768102	0.85420096	0.932178863
ANKS3	0.007987557	5.321507745	0.012151468	0.912224037	0.959684907
PCBP2	-0.027877672	5.321614934	0.065060595	0.798669201	0.901588892
ANKZF1	-0.035659425	5.322363131	0.262675858	0.608287736	0.78745769
BCL7B	-0.015599662	5.323152641	0.048596203	0.82552424	0.917634974
NFYB	0.240021736	5.323160536	7.246127142	0.00710541	0.048716466
PTPN9	0.011912639	5.323735611	0.021239464	0.884128495	0.948083049
LARGE1	-0.10601878	5.324008369	1.847196544	0.174109805	0.393575338
TCF15	-0.0504813	5.324128197	0.211989756	0.645212304	0.81068921
KIAA0930	-0.257889041	5.324144615	11.7578724	0.000605864	0.008338694
CNOT6L	-0.236225535	5.324197328	9.702256462	0.001840419	0.018937498

MUTYH	0.354790858	5.324231857	15.208117	9.63E-05	0.002010698
FBXL14	0.109119311	5.32514591	1.991993763	0.15813257	0.37284302
EME1	0.025089519	5.325177181	0.081411664	0.775393405	0.888893846
TELO2	-0.184107675	5.325245609	9.624332444	0.001920162	0.019564254
DYM	0.272584682	5.325463712	11.1844577	0.000824854	0.010464893
EAPP	-0.124876468	5.325576228	3.21120126	0.07313577	0.228966723
LHX4-AS1	-0.063078575	5.325622302	0.854024187	0.355416402	0.592060831
UFSP2	0.091159834	5.325653893	0.945412464	0.330889771	0.5687654
SPRTN	-0.3909908	5.325932697	23.35632575	1.35E-06	6.09E-05
CCDC14	-0.118252407	5.326223718	2.235177772	0.134901108	0.337938035
PGM3	-0.215085751	5.326372355	8.508741896	0.003534443	0.029733777
VPS50	-0.070722007	5.326604267	1.088766266	0.296744726	0.533680327
CARMIL3	-0.142490898	5.326857331	0.683542794	0.408369439	0.638817294
SFXN5	-0.12111933	5.327071839	2.378909233	0.122983391	0.317740886
MRPL21	0.087106108	5.327260117	1.334702531	0.247970353	0.482093632
FAM204A	0.077693132	5.327549617	1.230374125	0.267334292	0.502798694
HOXB2	0.334129715	5.327566436	14.71410319	0.000125107	0.002453678
FGFR1OP	-0.040244835	5.327977205	0.302345791	0.582415544	0.77051357
IL17RA	-0.386306312	5.328004419	18.28583262	1.90E-05	0.000541238
MMD2	0.297885185	5.328093881	5.502904308	0.018984917	0.095739139
FAM3C	-0.027801882	5.328111034	0.150535453	0.698024184	0.842822714
ORC3	0.070871541	5.328173789	0.867972931	0.351517099	0.587969672
SLC25A51	-0.079359958	5.328816684	0.600811407	0.438268605	0.663886934
TMEM63C	0.039046776	5.329156506	0.313428905	0.575583655	0.7656612
RNASET2	-0.204065391	5.329223511	5.549246267	0.018488647	0.09407578
FAM169A	-0.193047899	5.329556944	4.116263282	0.042472793	0.162306042
B3GALNT2	0.110097851	5.330366703	2.527614729	0.111869208	0.299659421
PDE12	-0.065152942	5.332264327	0.644702637	0.422013017	0.651251295
PEMT	0.10854585	5.332536525	2.323653893	0.127420405	0.325372868
NBL1	0.048735731	5.332690862	0.327502422	0.567133466	0.760668794
ZHX2	0.053011219	5.333289395	0.692644128	0.405266468	0.636520766
FER	0.177205926	5.33330753	3.660561938	0.055714839	0.192521561

TADA1	0.059687717	5.334084408	0.622187537	0.430235543	0.657642904
ALDH4A1	0.096132072	5.334149263	1.624356526	0.202485401	0.429146995
TAF6L	0.188516984	5.334328481	5.741532286	0.016568332	0.088258954
CCDC97	0.003757732	5.334422447	0.002273719	0.961968458	0.983899168
MRRF	-0.05790839	5.336493057	0.770522528	0.380055453	0.614685716
KIF26B	-0.404509571	5.336598766	5.40195667	0.020114189	0.099346821
TULP3	0.1387041	5.336636908	4.046074184	0.044274193	0.166154137
SETD1B	-0.157781401	5.336875353	3.942354567	0.047084831	0.172801769
NLRPL	-0.027197183	5.337357709	0.152083154	0.696552518	0.842034358
GNB1L	-0.002615248	5.337588384	0.001142447	0.973036552	0.989268858
ACSBG2	-0.18776673	5.337843265	3.483668354	0.061977207	0.207115138
G6PC3	0.101748586	5.3384794	1.347771585	0.245668069	0.479249328
VSIG10	-0.034031775	5.339122442	0.2276058	0.633304802	0.803843762
ITSN2	-0.039955343	5.3393476	0.291887903	0.58901325	0.774881119
GCLC	-0.227956294	5.339909196	7.314147863	0.006841383	0.047540102
SHCBP1	0.32358412	5.340293669	18.18546342	2.00E-05	0.000564662
ADAMTS19	0.177969187	5.340564634	1.293838621	0.255341745	0.490348661
NETO2	0.232544092	5.340877379	4.702199226	0.030124047	0.129660267
NFU1	0.131995031	5.340936673	4.404240745	0.03584968	0.14547632
SNAP29	0.083770631	5.340978231	1.434116653	0.231093772	0.462544585
ZNF687	-0.17358413	5.341832264	6.636337183	0.009991916	0.061922705
SLC35B2	0.007787384	5.34265019	0.01205311	0.912578572	0.959845606
ALKBH5	-0.003940502	5.342720749	0.00324661	0.954561901	0.980541221
LOC112530326	-0.038291278	5.343666915	0.316692121	0.573602374	0.764869254
UBE2A	0.017088877	5.344346944	0.079378031	0.77814206	0.889992883
CRYZ	0.109758249	5.344456611	2.457750912	0.116946126	0.30833977
PRPF38A	0.004100731	5.344664404	0.003336942	0.953934809	0.980523198
ARSA	-0.162590137	5.344908814	6.182063295	0.01290517	0.074256743
RREB1	-0.258919135	5.345215378	7.721310369	0.005457277	0.040523239
ASNSD1	0.119439957	5.345277725	2.610753622	0.106141311	0.289980951
RASSF7	0.183273023	5.345663725	3.764610674	0.052348043	0.184886683
GNPNAT1	-0.056069689	5.346255816	0.592457998	0.441470197	0.666270307

CSPG5	0.203633264	5.347089667	3.404027939	0.065037423	0.213483102
EXOSC7	0.080828556	5.347594053	1.112879882	0.291456782	0.5287105
KCNN1	-0.238657857	5.34804579	6.748713369	0.009381531	0.059263866
KLHL5	-0.088311999	5.34845352	1.464792707	0.226169088	0.456247682
WDR13L	0.198074782	5.348761164	5.65936714	0.017362387	0.09081312
ANKRD40L	-0.190806253	5.348828028	5.24942135	0.021954071	0.105462945
PLEKHA6	-0.3539639	5.349097648	13.76295683	0.000207385	0.003677292
FURIN	-0.115901883	5.349501852	2.938329132	0.086500168	0.254777708
ARF5	0.077331204	5.349804875	0.564268481	0.452545501	0.67549848
KCNT1	0.11563327	5.350068423	0.812857838	0.367276677	0.602336876
FCHSD2	0.206437962	5.35021714	8.232569043	0.004114525	0.033148795
EGFLAM	0.183776226	5.351973461	2.0882547	0.148435562	0.358717459
MFF	0.103836015	5.352825593	2.684791344	0.101310495	0.280745066
HOXA1	0.204127891	5.353257052	2.167310833	0.140972563	0.347885951
LETM1	-0.034746238	5.354440665	0.17626882	0.674599562	0.828521854
EXOC3	-0.116297493	5.354657107	3.282144252	0.07003737	0.223432516
IKBKB	0.035753639	5.354899684	0.217851554	0.640681825	0.807749496
TBC1D4	-0.132719525	5.355373654	3.228543332	0.07236503	0.227375706
ARNTL2	-0.130882447	5.355635894	2.944477538	0.08617156	0.254204558
N4BP3	-0.009293957	5.355639862	0.018227137	0.892605618	0.951217859
THAP7	-0.055255678	5.355756911	0.724899975	0.394541317	0.627445382
TIMM13	0.085833314	5.355811687	1.016717657	0.313298857	0.551313517
VAT1L	0.286960045	5.356245675	5.600101991	0.017959432	0.092640267
TLE4Z1	0.416435106	5.356511647	17.84291656	2.40E-05	0.000654682
KDELR3	-0.032737892	5.356533555	0.249086161	0.617719276	0.793398483
ARPP21	-0.079436895	5.356653837	1.295028381	0.255123348	0.49032844
PIGA	0.019364037	5.356722556	0.041507901	0.838560685	0.923661189
FAM102A	-0.056156979	5.356840856	0.51506568	0.472954146	0.691098524
KSR1	-0.032806365	5.35743089	0.179527425	0.671779679	0.826371731
ITPA	-0.149663815	5.357576659	2.092037525	0.148068476	0.358220275
KIF14	-0.053093771	5.357893665	0.50494158	0.477336845	0.693818648
CADM3	0.088371295	5.358962706	0.438260093	0.507963432	0.717224839

NELFE	0.064723535	5.359204668	0.851313355	0.356181061	0.592674625
GAB1	-0.056840788	5.359518053	0.579237297	0.446611432	0.670621395
CENPW	0.217434978	5.35953156	6.442449936	0.011142487	0.066989268
TSTA3	-0.061710638	5.359770608	0.908915027	0.340402458	0.577396397
SLC35C2	-0.060606427	5.359818857	0.766809761	0.381205791	0.61581988
RARS2	-0.081382571	5.360020912	1.668846364	0.196413126	0.421489043
PQLC2L	0.298217981	5.360296624	10.06913475	0.001507738	0.016258081
LOXL3	0.245054233	5.360648523	7.409655847	0.006487473	0.045954099
EPC2	-0.036854403	5.361070379	0.303401142	0.581758025	0.770421418
AGGF1	0.086545307	5.361111984	0.85712536	0.354544391	0.591269226
NPPC	-0.250442829	5.361398777	6.448937813	0.01110187	0.066860676
RNF165	0.054931409	5.361535789	0.344957837	0.556981349	0.753115441
USP30	-0.267831913	5.363612556	14.444155	0.000144377	0.002756064
NDRG4	-0.392484212	5.363746769	18.78508908	1.46E-05	0.000440123
LIMCH1	-0.447034884	5.364195595	18.79059828	1.46E-05	0.000439657
DMTF1	0.069077816	5.364344123	1.256922024	0.262234514	0.497528991
SLC4A1AP	0.105913631	5.366037536	2.20382646	0.137668626	0.342247657
THYN1	0.011373425	5.366407772	0.022891144	0.879740493	0.946151989
PER3	-0.216754081	5.36647234	6.188940459	0.012855113	0.074143318
RWDD3	0.01309314	5.366571992	0.024300139	0.876123653	0.943866356
RBBP8	0.078189558	5.366873065	1.306185177	0.253086513	0.487650887
TCP11L1	-0.301238236	5.367232481	12.48118978	0.00041107	0.006251536
PTGR2	0.44663886	5.36726794	24.48681098	7.48E-07	3.75E-05
ARFIP1	0.02713889	5.367461697	0.190020025	0.662899951	0.821016009
WDCP	0.210777065	5.368497184	10.38836288	0.00126812	0.01422421
LOC426821	0.197961443	5.368569888	7.310654399	0.006854696	0.047592415
POLB	-0.043386374	5.368662157	0.350728384	0.553701099	0.75174518
CLCN5	-0.204574987	5.369041723	4.096197836	0.042979765	0.163356737
ULK1	-0.189378088	5.369165615	6.044469257	0.013949926	0.078370103
MYO19	0.352340269	5.369761354	16.58872819	4.64E-05	0.001123407
ARHGEF16	-0.012422937	5.370092497	0.018949475	0.890511433	0.950676503
USP43	0.314672235	5.370320331	7.698237466	0.005527477	0.040878491

S1PR2	0.160765757	5.370360496	4.311241554	0.03786132	0.150485028
LOC101751422	-0.033971002	5.370885243	0.055602644	0.813586215	0.910966329
BBX	0.006122027	5.370893704	0.005038033	0.943414386	0.975792553
MAP9	0.21262091	5.371321474	8.826852398	0.002968299	0.026245763
NUBP1	-0.067493692	5.371497645	0.480261274	0.488303994	0.702056988
AXIN2	-0.24553646	5.373723466	7.271639613	0.007005183	0.048286186
WNT11	-0.164461051	5.373754279	0.910585198	0.339959196	0.577180001
UBE2G2	-0.060876398	5.374465695	0.909001029	0.340379614	0.577396397
FAM53B	0.267858886	5.375463041	7.972735383	0.004748713	0.036910754
TMEM183A	0.092624971	5.375778534	2.322154452	0.127543265	0.325528727
LAMTOR2	0.051966819	5.376588766	0.339714553	0.559994034	0.755303428
LOC107052159	-0.232851288	5.376624323	4.256386424	0.039103017	0.15393305
MRPL39	0.09303973	5.377175515	1.837377183	0.175258669	0.394944042
SLC18B1	-0.032729934	5.377769917	0.184360922	0.667652012	0.824166458
CAPN1	0.000998158	5.378181741	0.00012365	0.99112787	0.995986157
DSE	0.063092101	5.378189111	0.785746526	0.375389389	0.610055909
CACNB1	-0.080777945	5.378673429	0.81046147	0.367983852	0.60268481
ZC3HC1	-0.049250518	5.378720386	0.494358453	0.481989514	0.697181562
RRP7A	-0.084545406	5.378905359	1.579913207	0.208773196	0.436347382
LOC107054405	0.050302091	5.37903578	0.23554093	0.627445405	0.799999777
MRPL16	0.074398101	5.37909739	0.665759646	0.414533905	0.644965527
NADK	0.02281519	5.379182795	0.075143426	0.783990089	0.893823663
KIAA1191	-0.021166258	5.379277742	0.089808402	0.764420874	0.881746876
COG1	-0.052564544	5.380193722	0.262071305	0.608700698	0.787473436
LOC112530494	0.40874255	5.380589371	0.318761653	0.572352798	0.764143229
SNRK	-0.080163032	5.380788363	1.032501776	0.309571796	0.547425629
RPS6KB2	0.38880384	5.381086567	24.84481833	6.21E-07	3.24E-05
SENP7	-0.091872192	5.381658677	1.336670772	0.247621939	0.481633452
ASCC1	0.535770835	5.381959506	27.25840218	1.78E-07	1.11E-05
PDE6D	0.142276236	5.382354342	5.051073438	0.024610687	0.11381102
ANKRD6	-0.112364158	5.382438488	0.831440906	0.361856207	0.598210789
AACS	-0.056068436	5.382749575	0.351066778	0.553509873	0.751671616

LOC107053944	0.313457339	5.382883327	8.138181506	0.004334273	0.034360863
SPATA5	0.072368253	5.38319333	0.832166908	0.361646692	0.59801993
PEX3	0.079498724	5.383991625	1.568161817	0.210474163	0.438234642
UAP1L1	0.050067823	5.384837265	0.481946972	0.487541739	0.701827677
PNPLA8	0.107258487	5.385636679	2.303469025	0.129085413	0.328293098
LOC107050659	0.238326428	5.385918763	8.515122468	0.003522072	0.02966003
PDZD2	0.296711461	5.38627667	8.207119733	0.004172633	0.033476685
CYLD	-0.046761074	5.38716598	0.258296716	0.611292756	0.788982376
HEBP1	-0.066300578	5.387232658	0.476166039	0.490164095	0.703747508
RAPGEF5	0.054531573	5.387332355	0.283871375	0.594174935	0.777946576
LOC107052546	0.155592418	5.387612397	1.542756494	0.214207972	0.44320284
FAM92A	0.053300605	5.388602824	0.691586708	0.405625206	0.636675604
CENPL	-0.052860318	5.388647846	0.515075589	0.472949888	0.691098524
PRRG1	-0.046405043	5.388777502	0.339872814	0.559902645	0.755279244
FAM162A	0.073574427	5.388788424	1.108452442	0.292418601	0.529636552
ZYG11B	-0.354766095	5.388915535	24.51040265	7.39E-07	3.73E-05
YPEL5	-0.118205656	5.389307889	3.112597369	0.077688977	0.238146818
MAPT	0.237453759	5.389451255	4.679469469	0.030525174	0.130805141
TSPAN14	-0.068425683	5.389473	0.623318373	0.429816824	0.657247082
APIP	0.044524379	5.389745892	0.337858385	0.561068019	0.75591721
POLR3A	0.073931494	5.389846915	0.740583178	0.38947413	0.623423813
AGK	-0.055577048	5.38998046	0.58261696	0.44528838	0.670009172
TCF7L2	0.040484303	5.390758327	0.177831715	0.67324329	0.827473733
CEP104	-0.043710297	5.390836433	0.268021528	0.604662046	0.785222088
SARS2	0.101682724	5.391490539	1.808633513	0.17867224	0.399626438
RAB33B	-0.091027144	5.391507131	1.989957066	0.158345369	0.373291266
DTNBP1	0.20047159	5.392487852	12.14618215	0.000491885	0.007188242
UBL7	-0.159008866	5.3924894	4.235208231	0.039593766	0.155492942
ACVR1B	-0.093355809	5.392504255	1.668505263	0.196458862	0.421532217
MEF2C	-0.145838734	5.392636582	1.999831179	0.157316728	0.371825159
DDX52	0.043066455	5.392664321	0.226303145	0.634278643	0.80403489
WASF3	-0.128615163	5.392726586	2.790280116	0.094837648	0.269696998

FAM13A	-0.074325857	5.393117229	1.145407017	0.28451269	0.520473187
TMED7	0.111539582	5.393307066	1.570028249	0.210202911	0.438045609
YIPF4	-0.099740495	5.393512356	2.681920807	0.101493243	0.281062152
VPS54	0.041506624	5.394045195	0.431119093	0.511440337	0.719032808
TAF8	-0.017849856	5.39419021	0.034390634	0.852878354	0.931394748
EGFR	0.111813961	5.394493642	2.193755321	0.138571104	0.343867819
RNF146	-0.125682706	5.394493753	3.335719813	0.067790738	0.218896507
LOC416755	-0.170196914	5.394840497	4.133452201	0.042043499	0.161260659
PIGG	-0.082486259	5.395200226	0.706652646	0.400557297	0.633035951
CASP8AP2	-0.210696885	5.395816212	4.900610499	0.026847203	0.120706681
TSHZ3	-0.058074245	5.396627972	0.320938515	0.571044177	0.763077143
LPAR4	-0.049056837	5.396838568	0.541755657	0.461706035	0.683216979
TRIM8	-0.009760063	5.397199121	0.018007665	0.893250245	0.951354873
FKBP5	0.262474552	5.397400311	12.66841428	0.000371885	0.00578937
SIK3	0.081785269	5.397473924	0.990155213	0.319704442	0.557135546
FBXO33	0.064948551	5.397888106	0.873187988	0.350074248	0.586665827
FBXO38	-0.180812897	5.399340353	6.39903547	0.011418238	0.068198588
ZCCHC17	-0.218584665	5.399395849	4.855068097	0.027565026	0.122788981
RNF152	0.258891561	5.399815549	4.523987657	0.033422844	0.139198934
KLF10	-0.05999673	5.399978141	0.421507035	0.51618602	0.722449685
IFNAR2	-0.293180059	5.400308918	19.1793203	1.19E-05	0.000373695
HIP1R	0.124845108	5.400436718	3.287198721	0.069822051	0.22291848
MRPL32	0.145099155	5.400643292	3.733461194	0.053332771	0.187344215
KLHL24	0.044770383	5.401176859	0.282808484	0.594866318	0.77845837
BRAP	-0.031606762	5.401217918	0.141621136	0.706674673	0.848162533
LOC112531294	-0.573541106	5.401594079	13.29724132	0.000265797	0.00451361
FGD6	0.000420184	5.401812604	3.84E-05	0.995057891	0.99763501
RAD54B	-0.165133585	5.401899385	4.26339535	0.038942012	0.15340934
ITGA9	0.139508123	5.402031634	2.880931404	0.089634162	0.261125669
FAM177A1	0.368483228	5.402439251	35.62675836	2.39E-09	2.44E-07
THAP4	0.019181229	5.403490875	0.086979406	0.768052921	0.884354546
CCDC177	0.209464288	5.404174193	4.940608251	0.026232804	0.118685946

LOC112533383	-0.068456477	5.404833838	0.713089025	0.398420234	0.630739058
EYA1	-0.043519122	5.404957301	0.179424095	0.671868632	0.826371731
GALNT4	-0.049752324	5.405110663	0.381923861	0.536575413	0.737836448
PRKD3	0.148969761	5.405227765	3.019596964	0.082263908	0.246432297
FNDC10	-0.015205648	5.405333466	0.032328237	0.857309154	0.933498024
RACGAP1L	0.428448674	5.406645864	21.24319393	4.05E-06	0.000154092
ATG7	0.135089404	5.406749556	3.118973707	0.077385509	0.237684649
SPOUT1	-0.116507924	5.407059443	1.837938423	0.175192769	0.394903701
LOC112531155	-1.353225436	5.408006388	124.2262491	7.52E-29	1.37E-25
TTC26	0.182438496	5.408591555	6.155715499	0.013098812	0.075127552
EML1	-0.155346841	5.408622715	2.270369668	0.131868445	0.333266051
RBX1	0.014030468	5.408666933	0.032998135	0.855854357	0.932839865
ANXA1	0.379223592	5.40895149	1.68546282	0.194200151	0.418760776
MNAT1	0.091076327	5.409286415	1.440755156	0.230017179	0.461239663
C31H17orf49	-0.960302874	5.40930949	127.5499048	1.41E-29	2.90E-26
TOB2	-0.071379857	5.409942056	0.418199305	0.517836926	0.723406624
PROM1	-0.17625953	5.410008637	6.13234675	0.013273058	0.075888869
ACTR3B	0.065627494	5.410201159	0.529357308	0.466877186	0.686608642
NSRP1	0.023124725	5.411281799	0.117933581	0.731286584	0.862328105
GTF3A	0.074393603	5.411473952	0.913484656	0.339191525	0.576114424
LOC107050286	-0.07599837	5.412217972	1.226689232	0.268051871	0.503183194
FDXR	0.003264977	5.41232726	0.002188381	0.962688463	0.983917412
SLC40A1	-0.009913388	5.412697656	0.018431599	0.892008622	0.951008202
CENPS	0.128645868	5.413292883	3.311421802	0.068799943	0.220941268
IFFO2	-0.176210386	5.414251151	5.676679243	0.017191877	0.090236788
RP2	0.058497086	5.415006864	0.915336293	0.338702496	0.57576162
TXNDC11	0.142967622	5.415394059	3.708622368	0.054132052	0.189318367
NMT2	-0.072465334	5.415416101	1.357282596	0.244008987	0.477144133
ZNF319	-0.00438973	5.41657559	0.001960352	0.964684515	0.985185872
ABCC10	-0.117215873	5.416792792	2.529065501	0.11176639	0.299579077
PRRG3	-0.026327814	5.418173037	0.159009263	0.690070307	0.83840108
UBA7	0.060044628	5.418210346	0.432871376	0.510583361	0.718458116

CBLB	0.192430839	5.418565311	5.873453884	0.015370866	0.083332764
ICE2	0.223169352	5.41888522	6.40968362	0.011349964	0.067889369
SMDT1	0.095720764	5.419734284	1.922080692	0.165627468	0.383258527
UNKL	0.174395419	5.420143267	5.598489612	0.017975968	0.092667468
ABLIM1	-0.099924391	5.420154179	0.435935188	0.50909092	0.717892027
TMEM119	6.43E-05	5.420215212	1.88E-07	0.999653905	0.999836191
BARD1	0.076589609	5.42072022	1.130064044	0.287761679	0.524550617
SELENOK	-0.044820261	5.420782728	0.273770445	0.600813667	0.782591357
LOC101748153	0.350169201	5.421708002	9.738612629	0.00180437	0.018626671
ELOVL6	0.102046771	5.42236299	2.30399651	0.129041595	0.328281397
LOC112530163	-0.544085234	5.422641437	21.62894927	3.31E-06	0.00012992
WDR74	0.055176537	5.422807037	0.754396192	0.38508793	0.619718521
SNIP1	-0.030156771	5.423063671	0.198153739	0.65621524	0.816485578
NFXL1	0.000282634	5.423158973	1.56E-05	0.996845806	0.998628203
MDM1	0.069209469	5.423163332	1.053854269	0.304621081	0.542541389
PINX1	0.037773996	5.424060005	0.305887995	0.580214507	0.769333578
RNASEH1	0.002644297	5.424084276	0.001435954	0.969772206	0.987658702
ATF2	-0.114484306	5.424888299	3.301366624	0.06922227	0.221567349
SYNGR3	-0.080230184	5.42518255	0.955940682	0.328211744	0.565717803
HYKK	-0.188634756	5.426270816	7.16954705	0.00741515	0.050336751
AKAP10	-0.111187624	5.426589221	2.559445423	0.109637037	0.295670298
FAM63B	0.100617265	5.427438041	2.482007096	0.115155218	0.304986175
C12H3orf18	-0.191054283	5.427521699	4.28178321	0.038522913	0.15208602
SYNGR2	-0.181591695	5.427561747	5.259167263	0.021831462	0.105137513
SETMAR	0.102989767	5.427658653	1.298755458	0.254440679	0.489228952
CCDC117	0.050699888	5.427819562	0.653829058	0.418747009	0.648558238
LOC107050103	-0.024350245	5.427833883	0.143772283	0.704559284	0.846922567
NOL12	0.120252639	5.42813337	2.073174814	0.149909174	0.361281572
SEPSECS	0.248107343	5.428361589	11.08037714	0.000872461	0.010851352
PIP4K2A	0.085391457	5.428743343	1.533603191	0.215572475	0.444540058
KIAA0408	-0.147459359	5.4288343	1.439555345	0.230211309	0.461516458
MYO5C	0.043953246	5.429068075	0.413272776	0.52031303	0.725779864

APMAP	-0.080102872	5.429505431	0.81207542	0.367507363	0.602414195
FBXL20	-0.014617272	5.429966155	0.046819412	0.828693162	0.918759906
CTHRC1	-0.049349913	5.431311124	0.620725697	0.43077774	0.658237099
SH2D3C	0.094878734	5.431431049	1.068871283	0.301200535	0.538840488
MRPS2	-0.004033234	5.432123334	0.002675123	0.958750536	0.982278445
HECTD3	-0.180805862	5.432247562	7.142486377	0.007527871	0.050954802
ZNF606L	0.118257092	5.432266088	2.211248748	0.137007729	0.341223275
LOC107049904	0.034329217	5.432813402	0.148113574	0.700344653	0.84438535
C17H9orf78	0.044579969	5.432931039	0.425548162	0.514181522	0.72123919
TBL2	0.095137873	5.433106905	2.521924146	0.112273514	0.300009852
BNIP2	0.02143536	5.433834702	0.114265925	0.735338748	0.864745668
EAF1	-0.055548353	5.434229926	0.531755859	0.465869594	0.686100024
CEP85L	0.024089242	5.434499841	0.051021241	0.821295673	0.91616267
MRPL33	0.133192528	5.434671718	2.798744748	0.094338137	0.269024818
NPHP4	0.120687446	5.434877867	3.264015562	0.070815515	0.224671317
FAM45A	-0.115143127	5.435298834	3.704993198	0.054249893	0.189579921
APBB1	0.183517218	5.435547328	4.536166134	0.033185839	0.138567052
RBM24	0.064034132	5.436036005	0.378362754	0.538480768	0.739658146
NAF1	0.094002778	5.436137609	1.437480736	0.230547448	0.461942159
TRAF2	-0.000590134	5.436143678	4.52E-05	0.994634153	0.997544036
SSH1	-0.186846903	5.436823548	7.409990309	0.006486267	0.045954099
EPHA5	0.086688616	5.437227035	1.029725686	0.310223105	0.54812855
UBE2E2	0.231876664	5.437658977	5.988012753	0.01440342	0.079902002
KIAA1549L	0.088293537	5.438134494	1.582055144	0.208464915	0.436100304
PPARD	0.012685963	5.438235897	0.022582255	0.880548498	0.946351332
KIF13B	-0.236901172	5.438402119	9.107764088	0.002545268	0.023560072
RILPL1	0.076906462	5.439426063	1.409738833	0.235099753	0.467274603
ING1	-0.078306844	5.439901784	1.438818045	0.230330702	0.461643326
ADAL	-0.07616162	5.440074948	0.997090541	0.318015537	0.555837854
POC1B	0.127647143	5.44030825	3.337868504	0.067702259	0.218739577
OXR1	-0.012235593	5.440376858	0.034624915	0.852383819	0.931293687
LMCD1	0.324090055	5.441001886	9.316287788	0.002271257	0.021881459

LOC107050995	-0.428512057	5.441133703	33.98566181	5.55E-09	5.19E-07
PRDM4	-0.026989839	5.441237027	0.159822986	0.689319541	0.83825922
VPS13C	-0.053472508	5.441816428	0.339203969	0.560289067	0.755498094
RBPJ	0.02484743	5.442111951	0.132499481	0.715854439	0.852989434
SPTY2D1	-0.056853979	5.442502042	0.507391441	0.476270267	0.693258672
MGAT3	0.149563063	5.443001552	1.940881258	0.16357288	0.380007306
CHMP1B	-0.027565997	5.443112115	0.156637404	0.692271438	0.839288299
LOC107054251	0.058144772	5.443354938	0.479201541	0.488784208	0.70225654
EFL1	0.077386415	5.443372403	0.885699353	0.34664541	0.583116971
POU3F2	0.031992332	5.443808029	0.050917268	0.821474777	0.916245337
TRMT11	0.047069219	5.443876915	0.197689488	0.656592324	0.816646008
ANKIB1	-0.061596735	5.443889401	0.79995001	0.371108316	0.605520338
SLC25A10	0.029134548	5.444010909	0.155193135	0.693621204	0.839848191
NUBP2	-0.163965065	5.444500526	4.605813235	0.031863737	0.1348554
SLC39A13	0.002123027	5.445562321	0.000792634	0.977539521	0.990542078
RAB31	0.118057647	5.445902958	1.845052984	0.17435986	0.393796284
LOC112531444	-0.215368083	5.446086847	6.308305019	0.012017367	0.070547906
ADCYAP1R1	-0.146228317	5.446561338	1.997220255	0.157587981	0.372092155
ZNF618	-0.243787858	5.446934062	4.071966803	0.04360048	0.164740733
PTPN4	0.067198663	5.447156774	0.765388851	0.381647339	0.616351651
NKAIN4	0.260969668	5.447179658	7.572298497	0.005927215	0.042984717
CCPG1	-0.125693287	5.447210073	3.802383165	0.05117969	0.182325567
TNFRSF21	-0.023967628	5.447803044	0.118627557	0.730527808	0.862017575
FAM207A	-0.021916703	5.44790573	0.089258299	0.765122201	0.882338343
SAP30	-0.093237316	5.447913798	1.523192146	0.217137065	0.446236333
SLC30A9	-0.151631109	5.448219529	2.731681657	0.09837565	0.275207638
CRLS1	0.16571048	5.448702948	5.042005461	0.024739833	0.114127827
FAM222B	-0.143299327	5.449709674	4.219751987	0.039956001	0.156616482
SRI	-0.007206623	5.44978598	0.010633725	0.91786777	0.96231374
ACSL6	0.223564956	5.450606553	2.439090192	0.118344848	0.31056364
ARMC8	0.040356813	5.450666183	0.394254347	0.530071571	0.733281956
GRK3	-0.005203089	5.450898679	0.005116295	0.942977319	0.975585777

GDPD5	-0.185016169	5.451306108	3.358931791	0.066841413	0.216895178
LIAS	0.109051954	5.451408733	3.548486603	0.059599786	0.201337401
ZDHHC5	-0.101955222	5.452128192	2.678122405	0.101735615	0.281449149
SBNO2	-0.125927139	5.452398437	3.874988213	0.049010654	0.177129434
ERG	-0.085672885	5.453480837	0.897425612	0.343472938	0.58056982
ROMO1	0.077541274	5.45357253	0.634771472	0.425610489	0.653942509
DCAF6	0.016746415	5.454130233	0.056835856	0.81156876	0.90945001
LATS2	0.140629979	5.454356166	1.946336902	0.162982132	0.379400564
POLR2I	-0.13294772	5.454448054	2.721538783	0.099002531	0.276210011
EMC3	-0.196753187	5.454729048	10.58538921	0.001139849	0.013218938
SLC30A7	0.069671428	5.455303369	1.096570456	0.29501998	0.531965527
FBXL7	-0.06956372	5.455710194	0.457229495	0.498921627	0.710180814
STAB1	-0.147335098	5.455756162	3.080448428	0.079238676	0.240833472
ABCD4	-0.058464828	5.456024291	0.801457426	0.37065798	0.605077089
AP3S2	0.061842932	5.456266158	0.700759406	0.40252864	0.634660102
GID8	-0.012800969	5.45636965	0.02586132	0.872239463	0.941469358
FAAP24	0.149746049	5.456475966	3.816241989	0.050757961	0.181527053
IBA57	0.294211101	5.457236664	16.91873767	3.90E-05	0.000976522
EMILIN3	0.285824916	5.457302518	2.778640017	0.095529256	0.270836303
AP4B1	-0.120248973	5.457460831	3.141588535	0.076319442	0.235660803
DENND6B	-0.112293374	5.457534675	2.888910899	0.0891912	0.260127826
LPGAT1	-0.086296856	5.4582053	1.424076752	0.232733553	0.464253924
C5H15orf57	-0.328504088	5.458854787	17.24610442	3.28E-05	0.000839026
COMMD4	0.12391076	5.459300235	1.656382205	0.1980925	0.423711438
VPS53	-0.125114855	5.45985933	2.824989034	0.092807526	0.266209853
ITPKB	-0.037308502	5.46008229	0.288203236	0.591374246	0.776802089
EZH1	-0.098849978	5.460099684	1.936102881	0.164092303	0.380945096
CHCHD4	0.050823133	5.460237496	0.406668559	0.523665275	0.728148732
TBC1D7	-0.102265094	5.460868919	2.169782532	0.140746136	0.347535665
CNEP1R1	-0.118696825	5.461129375	3.673871045	0.055271673	0.191580742
DELE	-0.030552256	5.461137195	0.167997336	0.681897806	0.833288668
WASHC3	-0.073656359	5.461249524	1.31010987	0.252374764	0.486934484

MRPL27	0.053429333	5.461341357	0.444827452	0.504801606	0.71526617
PGM2	-0.122168007	5.462748932	2.592885018	0.107344648	0.291766796
RUSC1	-0.154477306	5.463088088	3.975099702	0.04617772	0.170404892
LOC107055115	-0.025403832	5.463194977	0.038737544	0.84396953	0.926328616
KDELC2	-0.169400513	5.463548268	5.269414403	0.021703312	0.104883409
OSBPL2	-0.144831144	5.464284655	4.216229785	0.040039033	0.156792547
WTIP	0.130700496	5.464775605	1.805700408	0.179024868	0.400196905
NFE2L2	-0.172411478	5.465098783	6.651837756	0.009905361	0.061669583
TARBP2	0.085825452	5.465159606	1.382078837	0.239747363	0.472323431
PRPF18	-0.034495854	5.465723042	0.298370856	0.584905555	0.771760156
ZNRF1	-0.084482938	5.466478112	0.96164526	0.326772703	0.564378194
P3H3	-0.206297669	5.466588677	7.414475384	0.006470118	0.045954099
TMBIM1	0.102229666	5.467219177	2.013619739	0.155892982	0.369468388
ALDH1L2	-0.13878442	5.467336735	3.702598724	0.054327791	0.189680416
EXOC3L1	-0.041328468	5.467527684	0.163793082	0.685688058	0.83583947
EXOC1	-0.06776978	5.46758666	0.827162565	0.363094288	0.599097263
MED9	-0.141923072	5.468280996	4.100364593	0.042873967	0.163232561
KIAA1107	0.011131609	5.468423116	0.012132295	0.912293034	0.959684907
NIPA2	0.040785348	5.468467646	0.341540465	0.558941383	0.754440198
DCLK1	0.15670983	5.469340156	1.850143903	0.173766658	0.393413644
DTNB	0.313351932	5.470325373	21.29961768	3.93E-06	0.000150669
SAMHD1	0.179945545	5.470545655	4.912787616	0.026658584	0.120183209
ORC2	0.020849828	5.471490686	0.07563947	0.783296026	0.893298966
MRPS27	0.150019429	5.471536349	2.128181549	0.144612226	0.353434606
PGP	-0.163159709	5.471542382	3.736196789	0.05324551	0.18719099
SELENOI	-0.072334779	5.471621839	1.237499285	0.265953547	0.501175632
TMEM14C	0.237134784	5.472646061	7.352029071	0.006698711	0.046925198
MRNIP	0.245602252	5.473503354	4.857885634	0.027520043	0.122655011
FGD5	-0.163367492	5.473718835	4.518564191	0.033528958	0.139499117
TAGLN3	-0.13862849	5.474027073	0.827098096	0.363112989	0.599097263
ATG5	0.125408863	5.474094162	2.901242554	0.0885113	0.258603239
MGME1	-0.055665473	5.474146108	0.545169673	0.460298108	0.68238923

PLEKHG3	-0.241970697	5.474683487	7.235668482	0.007146919	0.048939892
ZNF398L	-0.088905978	5.474811488	1.996748781	0.15763702	0.372154543
ABAT	-0.16636204	5.474909721	4.782204079	0.028755293	0.126044495
GAB2	-0.131556489	5.475081177	1.565457171	0.210867971	0.438608578
ZDHHC14	0.138278714	5.475373723	4.705641643	0.030063778	0.129502476
SEMA7A	-0.110984367	5.475814147	1.703400639	0.191843848	0.416091641
QPCTL	-0.011326665	5.476380885	0.03032981	0.861743996	0.935417736
-0.332699626	-0.332699626	5.476771486	11.90801011	0.000558931	0.007882433
RETSAT	0.053743159	5.477117295	0.475360581	0.490531332	0.703969418
DYNLT1	0.253670921	5.477654979	15.42414035	8.59E-05	0.001840134
PPIF	-0.158174313	5.477882851	4.562707286	0.032675383	0.137126606
RSRC1	0.125307455	5.478156575	3.249087681	0.071463224	0.225982928
DEPDC1	0.046015812	5.478346149	0.388838254	0.532910698	0.735103154
IMPA2	-0.125809089	5.478470008	2.960803569	0.085305536	0.252328347
ATG3	0.147948237	5.479346459	5.111962893	0.023761422	0.111362633
SNAPC5	0.114370305	5.479406864	3.136802995	0.076543709	0.236131745
ZNF592	-0.115121028	5.47971446	2.874276777	0.090005402	0.261806415
BEND4	0.088665206	5.479811984	1.333372879	0.248206064	0.482370472
STXBP1	-0.126603949	5.479868277	1.963666958	0.161121686	0.376813153
MSMO1	-0.047100349	5.480485701	0.24601301	0.619896537	0.794980713
LLPH	0.21061941	5.480984396	6.904636371	0.008597251	0.055903174
SPATS2	0.064273139	5.482728347	0.808765661	0.368485435	0.603219096
EEF1G	-0.005420001	5.482755889	0.003657336	0.951776597	0.979652938
AKR1B10L3	-0.419847066	5.483130722	13.05614965	0.00030229	0.004954368
PLA2R1	0.327574598	5.483173373	9.278546302	0.002318534	0.022168199
CHPT1	0.140018847	5.484391388	3.786952153	0.051653624	0.183378723
MOB3A	0.251631105	5.484607886	9.666879211	0.001876197	0.019199521
BAMBI	-0.761367571	5.485288018	9.546389624	0.002003431	0.020186263
NIPA1	-0.053427346	5.486318491	0.521025488	0.470404566	0.689331831
CPSF7	-0.074847701	5.486893548	1.290480802	0.255959364	0.490715523
NPHP1	0.087047963	5.487012207	1.673924823	0.19573366	0.420677293
EMC7	0.000890348	5.487077554	0.000181326	0.989256232	0.995057477

TMC6	0.011520023	5.487129589	0.011062211	0.916235324	0.961731649
PCYOX1L	-0.130564456	5.487135513	2.637612452	0.104360273	0.286638005
CGTL	0.320805124	5.487284625	3.037983389	0.081336903	0.24464664
RNF168	-0.108511716	5.487593566	3.001659411	0.083179281	0.2482438
HES6	-0.406399107	5.487826095	6.403958354	0.011386621	0.068059154
POP1	-0.004521818	5.488111765	0.002731238	0.958320531	0.982074132
ZMYND19	-0.026972778	5.488271441	0.113478083	0.736218609	0.865257997
TMEM167A	0.195764628	5.488562863	4.820240509	0.02812742	0.124420265
PPT1	0.026872606	5.488565014	0.138202859	0.710074191	0.849641566
DEPDC1B	0.235422416	5.48861092	5.21059558	0.022449643	0.107108701
SUV39H1	0.130172246	5.488640367	2.389381709	0.122161936	0.316712567
DOK5	-0.492107038	5.488943314	3.938001245	0.047206835	0.172938715
CALCOCO2	-0.094509622	5.489618412	2.305819029	0.128890326	0.328057279
DAPK1	0.05972455	5.489834442	0.540792641	0.462104416	0.683437728
DCTN6	0.137011463	5.489953622	1.763600151	0.184176098	0.406794321
HES4	-0.137062204	5.490152509	1.255785651	0.262450319	0.497588898
VRK3	-0.098020832	5.491224553	2.380929175	0.122824472	0.317579619
PLAGL1	-0.142095708	5.491716953	2.721172448	0.099025253	0.276226572
COA5	0.323157297	5.492478386	23.57878233	1.20E-06	5.51E-05
MRPS21	0.083294344	5.493332752	0.617890167	0.431832395	0.658920814
LOC107051046	0.13736154	5.493729755	0.505567394	0.477064019	0.693600685
PLAG1	0.131561335	5.494077001	2.931090377	0.086888791	0.255495898
STIM1	0.024502295	5.494083575	0.158279301	0.690745688	0.838773544
PDE3B	0.035652443	5.494656337	0.239384274	0.624651178	0.798386013
ZMAT5	0.053585028	5.494726778	0.520560317	0.470602765	0.689405693
MTERF3	0.253150362	5.495014397	8.083141943	0.004467892	0.035221456
USP45	-0.096027618	5.495413268	1.879137275	0.170432234	0.389238365
METTL16	0.100115824	5.496783234	1.582304682	0.208429035	0.436100304
TAF11	-0.058983276	5.497087268	0.696072691	0.404106482	0.635646128
MOSPD1	0.121807173	5.497640936	3.452174587	0.06316861	0.209555471
GMCL1	0.117053565	5.498409976	2.784413299	0.095185547	0.270280962
SLC33A1	0.065849435	5.499140101	1.20343842	0.27263553	0.507375894

RNPC3	-0.009371117	5.499170657	0.016425182	0.898021748	0.953351475
XRCC3	0.140043235	5.499269128	4.88807251	0.027042861	0.121226639
SPSB4	0.068311297	5.499386172	1.014732656	0.313771705	0.551695247
VPS45	0.28864728	5.499443868	7.041334263	0.007964967	0.05301923
DNAL4	0.136771223	5.499519025	4.810758693	0.028282591	0.124748861
TAF13	0.081211452	5.499564559	1.343704874	0.246381662	0.479919939
CPXM1	0.278140771	5.499808304	6.010833672	0.014218308	0.079236332
RIOK3	-0.173800854	5.500491053	3.520279759	0.060622214	0.203870535
TSEN54	-0.006802766	5.500791294	0.007479765	0.931080374	0.969801718
TTC1	0.07217539	5.500912808	1.301758934	0.253892184	0.488459708
CFAP36	0.03868931	5.501334936	0.401385394	0.526374648	0.730603439
TSTD2	-0.012340641	5.501355922	0.015876716	0.899729675	0.954302681
USP25	0.126840613	5.501469532	3.628365521	0.056802576	0.195132857
FGFR4	-0.121503882	5.501543513	2.416015898	0.120100035	0.313093484
PELP1	0.124753847	5.501605697	2.853187564	0.091192965	0.263653048
ASAP3	0.212525906	5.501745357	6.561631609	0.010420078	0.063835587
GATA5	0.186375334	5.501855965	0.619872444	0.43109469	0.658466827
GSTO1	-0.316533145	5.501958309	16.78730971	4.18E-05	0.001034611
MEPCE	0.177935447	5.503031149	5.075564335	0.024265372	0.112764397
ELP3	0.113996469	5.503200417	1.62503118	0.202391686	0.429146995
REEP5	-0.121738894	5.503833519	1.953238318	0.162238318	0.37851007
STX1A	-0.151623113	5.50416593	2.370016388	0.123685753	0.318775621
RFT1	-0.450285447	5.504537938	23.83567078	1.05E-06	4.96E-05
PDCD5	-0.027135754	5.505464193	0.14141122	0.70688208	0.848227567
GNAL	-0.118853755	5.505714777	3.01431636	0.082532248	0.247011303
KLHL20	-0.130940191	5.506331894	3.821912043	0.05058648	0.181174895
VAMP3	-0.068659913	5.506542358	0.891952689	0.344948696	0.581689976
RFWD3	-0.017689907	5.506550707	0.044330904	0.83323917	0.921086197
AP3B2	-0.01362485	5.506797284	0.027197981	0.869008442	0.939646072
ZC3H4	0.211781849	5.506827129	3.895376434	0.048419187	0.175608933
SEC24D	0.17164849	5.506999382	5.196530413	0.022632019	0.10750919
CENPC	-0.001084838	5.507527769	0.000173525	0.989489869	0.995074915

TGIF1	-0.021583698	5.508100469	0.053453751	0.817158907	0.913634921
FZD6	-0.355021906	5.508745398	21.37373016	3.78E-06	0.000145294
MYH11	-0.41816305	5.509074533	8.446146362	0.003658178	0.030579826
COG3	-0.376851258	5.509094209	28.23933586	1.07E-07	7.17E-06
MAPKAP1	0.015745345	5.509280489	0.063448721	0.801125861	0.903085487
LOC107055157	0.026109538	5.50937042	0.057561444	0.810392538	0.909099927
HUS1	0.004111542	5.509723061	0.002935755	0.956789679	0.981299811
GLRX2	-0.19563235	5.510075546	3.704545633	0.054264444	0.189579921
STRADB	0.453146544	5.510914063	28.88957871	7.66E-08	5.39E-06
NDUFB6	0.014524284	5.511241239	0.01970633	0.888360361	0.949552787
MZT2BL	-0.093309583	5.511576806	1.537543453	0.214983828	0.444193733
TRPS1	-0.038937237	5.511607061	0.129003149	0.719467855	0.855189331
TBC1D25	0.016652127	5.511678105	0.032688749	0.856524325	0.933075655
ATG4A	-0.005993132	5.511887381	0.008890986	0.924877184	0.966337803
KIAA2022	0.210026999	5.51207495	5.757663279	0.016416891	0.087622426
MFSD4B	-0.31612144	5.512258233	19.74872204	8.83E-06	0.000296595
CERS4	0.464642207	5.512606471	29.00829466	7.21E-08	5.10E-06
RAB11FIP1	-0.216382407	5.513650869	9.927682682	0.001628114	0.017184485
SRSF4	0.290178079	5.514763674	10.05646028	0.001518146	0.01628494
NRDE2	-0.393630542	5.514812398	27.75989252	1.37E-07	8.97E-06
KANSL1L	0.180562188	5.515017036	8.072938522	0.004493119	0.035358335
USE1	0.079479572	5.515247078	1.282674992	0.257402258	0.492320522
GPATCH2	-0.230533764	5.515411187	11.50965613	0.000692356	0.009195089
PDE10A	0.013143868	5.516308727	0.017569971	0.894547881	0.951827123
DKK3	0.220687697	5.516373961	5.773696614	0.01626778	0.087080782
CCDC134	0.067335927	5.516562366	0.688590608	0.406644181	0.637633886
CNNM4	-0.056538788	5.516759129	0.890238492	0.345412687	0.582114478
ITGAV	0.102593269	5.516950582	0.692068664	0.405461641	0.636628942
FAM20B	-0.236710512	5.517212112	13.90240957	0.000192551	0.003466558
PCGF6	0.076453583	5.51806237	1.209779548	0.271375786	0.506406051
APH1A	-0.009901585	5.518372171	0.022578557	0.880558204	0.946351332
TAGLN	-0.055720409	5.518469317	0.193344698	0.660147313	0.8197045

LOC101750701	0.251040205	5.518684074	4.734681991	0.029560313	0.127936597
ANPEP	0.205311221	5.519165408	6.495633	0.010813979	0.065541075
ADAM33	-0.158461635	5.519390312	2.872944585	0.090079921	0.26193057
NDUFA12	0.123459313	5.519790189	3.653620057	0.055947483	0.193122685
ERO1B	-0.017724108	5.519871654	0.07966453	0.777752545	0.889865642
ASCC3	-0.021692962	5.520192216	0.061571424	0.804029261	0.905193041
RPS6KC1	-0.366252286	5.521512205	22.53179111	2.07E-06	8.70E-05
CHST13	0.268930706	5.521571146	10.74482748	0.001045712	0.012430121
NPC2	0.372908818	5.521583744	9.478692952	0.00207872	0.020718058
E4F1	0.020392867	5.521719476	0.125414159	0.723235004	0.857066913
ANAPC15	0.159987162	5.522163282	1.201777605	0.272966682	0.507692808
CTSH	0.310110616	5.522227557	16.9010296	3.94E-05	0.00098182
ERGIC3	-0.055865268	5.522764249	0.777410654	0.377934225	0.612700264
CMTM6	-0.308703585	5.522941774	17.31192558	3.17E-05	0.000818082
ZBTB17	-0.201336547	5.523795621	9.942919594	0.001614692	0.017119694
ALDH6A1	0.14148149	5.52393743	3.108282835	0.077895045	0.238215389
SNRPA1	0.090339731	5.524630316	0.863413021	0.352785341	0.589348507
PDLIM4	0.142784683	5.524703981	2.709509231	0.099751675	0.277547145
LOC101752260	0.326296336	5.524989794	20.67906377	5.43E-06	0.000198581
HPS5	-0.034122369	5.525265115	0.224773029	0.63542693	0.804702787
SPC24	0.157088337	5.525849391	3.206682872	0.073338025	0.22925099
LOC107050083	0.152592756	5.526065732	4.742172357	0.029431884	0.127758008
DCUN1D3	-0.0937495	5.52624769	2.425750534	0.119356064	0.311808626
SPRYD3	0.02530411	5.52630125	0.117979723	0.731236057	0.862328105
CCP110	0.028228358	5.526990354	0.157771988	0.691216132	0.838989066
PEX2	0.036732736	5.527204158	0.258095829	0.611431374	0.788982376
TNFRSF14	0.009932924	5.527455596	0.011496567	0.914612828	0.960982957
COMMD7	6.34E-05	5.527504619	8.56E-07	0.999261798	0.999602853
MTMR2	-0.08030462	5.528854104	1.220441545	0.269274016	0.504027293
PRUNE1	0.110128056	5.528941385	2.988955786	0.083834212	0.24963662
ZBTB32	0.013070802	5.529297118	0.019969846	0.887621332	0.949358246
SRP14	-0.06021865	5.529320863	0.597456516	0.439550129	0.664718075

ERLEC1	-0.07198193	5.529336152	1.369402426	0.241914582	0.474797763
RAB34	0.03147434	5.52955493	0.181912095	0.66973514	0.825134152
DCLK2	-0.076810814	5.529605551	0.669932152	0.413075263	0.643252695
TRIM7.2	-0.485386609	5.52965783	25.13900975	5.33E-07	2.84E-05
IMPA1	-0.313031478	5.530069093	12.54984628	0.000396239	0.006082193
MED12L	-0.367957935	5.530265021	22.4806506	2.12E-06	8.90E-05
PLPP5	0.030323011	5.530644432	0.215495747	0.642493576	0.809078732
OSTC	-0.053069144	5.530696174	0.715135855	0.397744087	0.630102912
CYB5A	0.049801198	5.530741982	0.577715229	0.447209274	0.670752767
SLITRK2	0.212431748	5.532521437	12.57824101	0.000390264	0.006006004
KEAP1	0.260351336	5.532648165	7.954917794	0.004795689	0.037188057
CARNMT1	0.286234998	5.533285924	7.481746105	0.00623276	0.044717367
PPP1R13B	-0.140723214	5.533733816	3.825989688	0.050463537	0.180988995
C5H14ORF169	0.103786934	5.533892885	2.093896083	0.147888496	0.357979712
FAM175A	0.02980248	5.533898851	0.105169164	0.745712467	0.871333755
PPP4R1L	-0.14695778	5.534469751	3.81839581	0.05069275	0.181494606
IPPK	-0.177323922	5.534545937	5.59660949	0.01799527	0.092737919
TRAF3IP1	0.033724948	5.534685983	0.273678642	0.600874715	0.782591357
C20H20ORF24	0.074625742	5.535186617	1.446044506	0.229163707	0.459976677
SIRT6	-0.047768889	5.535661914	0.531911847	0.465804187	0.686100024
USP46	0.013877994	5.536282714	0.041857676	0.837891308	0.923600045
DGKD	-0.138489462	5.536623055	2.588659995	0.10763136	0.292160018
P3H4	0.030247387	5.53706689	0.053246873	0.817506822	0.91379626
SRRM4	-0.298083973	5.537357871	3.328592556	0.068085114	0.219439538
APOPT1	0.186247558	5.537390516	1.220148033	0.269331603	0.504055228
TOP1MT	-0.314522712	5.537473694	23.06061385	1.57E-06	6.91E-05
NOTUM	-0.211675212	5.538011954	4.338264005	0.037264901	0.148842797
RGS10	-0.135329127	5.538055174	2.381863012	0.12275108	0.317489629
PREX2	-0.055316345	5.538111848	0.274345287	0.6004317	0.782582465
DYNLL2	0.084812818	5.538619671	1.127705662	0.288265255	0.525003295
LSM5	-0.016486255	5.538784767	0.039933205	0.841611236	0.925494032
CRELD1	0.222814975	5.539258572	5.673874543	0.017219383	0.090294756

YTHDC2	0.081447368	5.539384496	0.640778491	0.423429035	0.652756677
AKR7A2	-0.183280632	5.539472849	2.598874205	0.106939655	0.291098762
CRISPLD1	-0.039419584	5.539565127	0.168065601	0.681836723	0.833288668
COL2A1L	0.603103801	5.539568419	12.4530939	0.0004173	0.006311279
TRMT6	0.235014419	5.539812677	11.46227159	0.000710234	0.009372017
FAM126B	-0.03505493	5.540064955	0.145139739	0.703223953	0.846389149
RGS12	-0.06029309	5.540943628	0.95815291	0.32765269	0.565526033
SNAPC4	-0.193941367	5.541221376	7.392661342	0.006549052	0.04623108
NDUFC2	-0.062702293	5.541785358	0.638649602	0.424200219	0.65309226
MIS18A	0.078025066	5.541864586	1.626118882	0.202240703	0.429146995
MOB1A	0.094050684	5.54248331	2.528288854	0.111821419	0.29962006
DHTKD1	0.398700756	5.543292121	12.44708733	0.000418644	0.006325794
STX2	0.045055434	5.543559822	0.529886485	0.466654588	0.686588094
PRTFDC1	0.012612183	5.543681239	0.025582804	0.872923412	0.94177516
WDYHV1	-0.758020074	5.543903918	41.40299893	1.24E-10	1.84E-08
IMP3	0.135517523	5.544808466	1.249171272	0.263710819	0.498834524
DIABLO	0.069556432	5.545184825	1.15062121	0.283419146	0.519396598
TGFB2	0.305744448	5.545262071	8.615294653	0.003333519	0.028509901
BMPR1B	0.15448447	5.545512982	2.178763508	0.139926842	0.345927911
ASTN2	0.048353672	5.545801271	0.247812002	0.618619945	0.794077342
CREB3L1	0.012597264	5.545802062	0.017597883	0.894464642	0.951827123
FOXD1	0.269433084	5.54586486	3.308226826	0.068933835	0.221241712
AMMECR1L	0.01246922	5.546385078	0.036470989	0.848546126	0.929382324
MICALL2	-0.073599437	5.546423842	0.748243058	0.38703306	0.621219187
PCCA	0.435653044	5.546618623	46.88071512	7.54E-12	1.50E-09
REV1	0.207902881	5.546808851	7.290823098	0.006930772	0.04799377
ALG9	-0.049875494	5.547429798	0.478957416	0.488894944	0.702354313
LRRC75B	-0.103315371	5.547442896	1.484587623	0.223058123	0.452356596
OSBPL5	-0.013227544	5.547773355	0.048183933	0.826254045	0.917837275
MYOM1	-0.13372891	5.548000264	0.512031208	0.474260881	0.69207548
DHX40	0.004305574	5.548779889	0.004423922	0.946969753	0.977487125
NLE1	0.195241112	5.54878885	6.542472394	0.010532887	0.064263496

TTC21B	0.167105053	5.548972299	5.586456158	0.018099879	0.092927773
BRCC3	-0.12079857	5.54920249	3.684559491	0.054918477	0.190718017
TSPAN13	0.258972781	5.54985	13.78103316	0.000205399	0.003653108
UNC5B	-0.284383956	5.549961965	7.979373065	0.004731332	0.036810437
FAAH2	-0.244912147	5.550332083	14.7205395	0.000124681	0.002451158
TRIM37	0.038033757	5.550873837	0.31428115	0.575064906	0.765492399
TAOK3	-0.099529168	5.551283197	1.817557253	0.177604319	0.398320713
NCEH1	0.034611282	5.551352131	0.329244876	0.566104071	0.759683778
PAPD5	-0.074142587	5.551493576	1.109304639	0.292233154	0.529417214
LOC107049717	0.144134175	5.55202341	4.416483123	0.035593325	0.144650075
CPPED1	0.427530579	5.552588802	30.38603425	3.54E-08	2.71E-06
ADGRA2	-0.289023378	5.552852445	7.10468755	0.007688263	0.051742479
GRN	0.089455945	5.553117933	0.923648184	0.336518898	0.573825748
CCDC92	-0.213108037	5.554177663	7.593446735	0.005858084	0.042652557
STX7	-0.088832318	5.555323413	1.733947071	0.187907604	0.411143602
LRRC45	-0.243098039	5.556272124	7.09986132	0.007708992	0.051839582
TNFAIP8L1	0.054061405	5.557814421	0.50478402	0.477405574	0.693818648
TOMM34	-0.087046976	5.557851682	1.577337222	0.209144663	0.43679089
CLCN6	0.070670647	5.558098235	1.192537783	0.274818252	0.509715719
SSBP1	-0.12779897	5.559161851	3.416922059	0.064531225	0.21237226
ZDHHC3	0.068672011	5.559197251	0.935041123	0.333556409	0.571379421
ALG8	0.224204113	5.559236348	10.06045838	0.001514855	0.016282355
JMY	-0.071179313	5.559338696	0.806672505	0.369105858	0.603921338
SDF4	-0.017984824	5.559455031	0.083105006	0.773132884	0.887610344
SLC2A13	0.038052157	5.559708896	0.337635418	0.561197294	0.75591721
GLB1	-0.028389973	5.560010785	0.1060599	0.744675262	0.870453347
DYRK2	-0.193022095	5.560392329	6.009468364	0.014229314	0.079236332
TMEM106B	-0.413467361	5.562948201	21.12959313	4.29E-06	0.000162002
NCKIPSD	-0.245979716	5.563109212	8.30252099	0.003959008	0.032220766
HGSNAT	-0.186434759	5.563146698	10.82592046	0.001000889	0.012043138
ADPRHL2	-0.036596404	5.563517665	0.412947073	0.520477465	0.725779864
MTAP	0.129338351	5.563539581	1.79300537	0.180560417	0.402481937

SLC15A4	-0.128151957	5.563930344	4.10750603	0.042693276	0.162808308
THBS4	-0.422195271	5.564572329	20.12939324	7.24E-06	0.000250198
MVD	-0.115863672	5.564602303	1.64757405	0.199289438	0.425111187
POLR3D	0.071336734	5.564919414	1.196401478	0.274042095	0.508957412
LDLRAP1	0.176096168	5.565096342	5.571207455	0.018258166	0.093383174
CAST	-0.037777115	5.565485908	0.196776462	0.657335472	0.817261442
ZNF346	-0.011229217	5.565548947	0.034729297	0.852164041	0.931269647
SDHD	-0.159793609	5.565882154	4.030112209	0.044694968	0.166867109
TBC1D31	-0.089136998	5.566301058	1.633069983	0.201278952	0.427912811
PSMC3IP	-0.001064695	5.566413606	0.000209129	0.98846197	0.994809891
ARF6	-0.008778057	5.566417807	0.020319508	0.886648345	0.948991708
SWAP70	0.045971055	5.566511443	0.531835585	0.465836162	0.686100024
CCDC59	0.135633368	5.566766254	3.700050117	0.054410834	0.189809258
TPRG1L	0.027148909	5.567353382	0.191882532	0.661354399	0.820458761
CDK19	-0.156179	5.567698978	2.380683334	0.122843802	0.317579695
MRPL57	0.07611374	5.568039048	1.441071049	0.2299661	0.46119344
CNTN2	-0.008645682	5.568189445	0.00154949	0.968600532	0.987147683
MVP	-0.078128119	5.56861783	0.456569105	0.499231787	0.710379765
VPS33A	-0.169792386	5.568693575	7.125702049	0.007598666	0.051307362
DDAH1	0.061086274	5.569061983	0.726867023	0.393900597	0.627153781
MPP7	-0.0586296	5.569115092	0.637356256	0.42466976	0.653322821
UBE2E1	-0.044733061	5.569403495	0.564448271	0.452473498	0.67549848
ISLR2	0.13014794	5.569906694	0.703484153	0.401615439	0.634066049
PPFIBP2	0.000476588	5.570291326	2.06E-05	0.996376289	0.998524363
APEH	-0.045127505	5.570892114	0.411481504	0.521218509	0.726233326
MRPL18	0.173028924	5.571080015	6.93990526	0.008429358	0.055195019
TMEM39A	0.019945643	5.57121638	0.111654657	0.73826814	0.866738173
SYVN1	0.398356131	5.573801938	13.71345392	0.000212924	0.003763329
ZNRF3	0.048605049	5.573805421	0.368664742	0.543732999	0.744043784
CYTH3	-0.124716096	5.573819357	2.570700252	0.108859534	0.294440683
NUS1	0.010790681	5.574130163	0.028092121	0.86689243	0.93840635
MINA	-0.011229665	5.57453608	0.026271915	0.871238024	0.940731028

SPOCK2	0.013867083	5.574654591	0.026837341	0.86987201	0.940085638
NINL	-0.209020331	5.57493298	4.987511235	0.025530903	0.116705787
GPBP1	0.16847429	5.57510501	4.2014417	0.040389628	0.15741623
COL24A1	0.037579792	5.57546829	0.063420369	0.801169367	0.903085487
PWWP2A	-0.156051147	5.575521811	5.049425083	0.024634111	0.113863565
LOC107050443	0.031177191	5.576477938	0.193072173	0.660371881	0.819884957
CLCN7	-0.183286068	5.577056949	8.814252882	0.002988866	0.026356798
LOC107049862	0.161461124	5.577229047	3.06039473	0.080222179	0.242746589
CLK4	-0.254877489	5.577429768	5.655158593	0.017404101	0.090912924
LMTK2	-0.182676953	5.577520076	3.612453713	0.057348448	0.196541945
SERPINF1	-0.055709445	5.577788493	0.441449739	0.506423557	0.715978975
MSANTD2	-0.039502387	5.578469757	0.165136736	0.684470615	0.835303382
CUEDC1	0.031495258	5.578474263	0.261642318	0.608994099	0.787473436
FRS2	-0.015501188	5.578766622	0.050515286	0.822169051	0.916392801
CXorf23	-0.111820888	5.578842604	3.711756282	0.054030511	0.189124029
SPATA20	0.331922777	5.57900768	18.23007956	1.96E-05	0.000555392
UBAC1	0.036307529	5.579192454	0.303673399	0.581588642	0.770401119
KAT5	0.339127345	5.579347052	34.56112998	4.13E-09	4.00E-07
TLCD1	0.015882863	5.579402019	0.060054314	0.806410115	0.90650898
NDUFB2	0.301569825	5.579530067	8.559455358	0.003437322	0.029110211
MYSM1	-0.115024605	5.57970633	2.47440924	0.115712913	0.305948797
CHUK	-0.043395539	5.580342036	0.518709012	0.471392901	0.690165511
PIGY	-0.024881782	5.580407767	0.17330265	0.677193164	0.830158199
SAR1B	-0.121326124	5.580531276	2.586190606	0.107799323	0.292504041
TMLHE	0.081571384	5.581047119	1.520189478	0.217590822	0.446496692
SLC25A14	-0.082473061	5.581745753	1.67388443	0.195739054	0.420677293
GPR161	-0.173499777	5.582122868	4.940036945	0.026241476	0.118692546
AFMID	-0.395342053	5.582318391	22.09096021	2.60E-06	0.000106174
B4GALT1	-0.001007137	5.582378158	9.19E-05	0.992351428	0.996459368
FAM160A2	0.106972763	5.582462625	2.436429998	0.118545746	0.310692768
TBX2	-0.034088833	5.582556923	0.025057238	0.87422452	0.942721236
MCCC2	0.024456989	5.582563887	0.073599344	0.786166467	0.894763475

REXO2	0.089154136	5.582685214	1.75465587	0.185292494	0.407836809
ZSWIM8	0.101327068	5.582755269	1.786792395	0.181317467	0.40334986
SLC2A8	-0.10108766	5.582774011	2.409046638	0.120635814	0.314024958
SLC35F5	-0.101734202	5.582791042	2.271500408	0.131772274	0.333125329
DPP7	0.191712548	5.582891394	7.74625791	0.005382396	0.040190968
APPL2	-0.081078069	5.58295628	1.618096177	0.203357453	0.430265966
PYGB	0.072875386	5.583187299	1.147978622	0.283972695	0.520055692
CNKSR3	0.001735244	5.583741348	0.000256683	0.987217372	0.993982859
LNPK	-0.228656156	5.583904	13.24380463	0.000273482	0.004587309
ZC3H12B	-0.07442042	5.584509106	1.099383839	0.294401366	0.531297924
AFF2	0.102981433	5.585147086	1.355575496	0.244305761	0.477497481
SNCAIP	-0.252996848	5.585368652	5.68152128	0.017144496	0.090103062
BLOC1S5	0.127916503	5.5857711	2.808691097	0.093754843	0.268161991
IFT140	0.292201036	5.585834844	18.07422298	2.12E-05	0.000589545
DCP1A	-0.1111513	5.58594111	1.868046844	0.171698945	0.390367969
ERCC6L2	0.077605699	5.585996405	0.687293921	0.407086346	0.637801433
PDHX	0.077813967	5.586332429	1.396374757	0.237331499	0.469668047
CDC25A	-0.083024255	5.586643701	0.942740425	0.33157407	0.569374086
MAT2B	-0.424007176	5.587615338	40.29894117	2.18E-10	3.03E-08
ST7L	-0.162885191	5.587718227	7.777234871	0.005290872	0.039772178
MGARP	-0.222870026	5.587838924	8.18715315	0.004218808	0.033764824
CNPY3	0.178397537	5.588021435	3.199906114	0.073642498	0.22989704
MSH4	-0.459827288	5.588101383	21.49232878	3.55E-06	0.000138194
PRDM10	-0.003090084	5.588435395	0.001493658	0.969171133	0.987352092
RRS1	0.041078558	5.589161636	0.13610802	0.71218124	0.850431227
CNKSR2	0.011634926	5.589463771	0.013022127	0.909147001	0.95829261
ASB6	-0.202809357	5.58971019	7.680416052	0.00558233	0.041210065
ZNF710	0.150056562	5.589721714	4.434422947	0.035221124	0.143669707
PBX3	0.154862648	5.589926919	3.357168622	0.066913022	0.217030217
TMEM39B	-0.019007617	5.590031001	0.083730348	0.772304397	0.887015345
CPOX	0.079269934	5.590929966	0.909996881	0.340115246	0.577335279
RAB11A	-0.039365735	5.591102003	0.447900222	0.503333796	0.713873264

SNX7	0.137334476	5.591805988	4.63285433	0.031365341	0.133294601
FCHO2	0.051320451	5.592291393	0.442938717	0.505707462	0.715440088
MYT1	-0.604021722	5.592666141	5.97593254	0.014502411	0.080322171
MLF1	0.048766705	5.593415675	0.387603607	0.533561745	0.735495698
PTGES2	0.071474704	5.593482653	1.205639967	0.272197338	0.506961764
CNNM2	0.106083062	5.593504746	2.410319573	0.120537757	0.31390391
ELMOD2	-0.033300844	5.593785938	0.172267724	0.678104224	0.830532564
MRPL54	-0.088908884	5.594085907	1.638959733	0.200468257	0.42687655
GPN3	0.011507789	5.594146642	0.025006626	0.874350551	0.942721236
PHLPP1	0.064798335	5.59416418	0.515621185	0.472715561	0.691022409
DOHH	0.196400131	5.594561731	4.696434258	0.030225263	0.12989206
TARBP1	-0.182213648	5.595115575	2.798316928	0.094363315	0.269024818
INIP	0.120510624	5.595208777	1.532044006	0.215805935	0.444608321
TRIP11	-0.120400853	5.595562686	2.374877355	0.123301281	0.318327342
DNAJC16	-0.133147072	5.595956651	3.279753402	0.070139467	0.223628159
ACSF2	0.35235603	5.596602842	25.35484306	4.77E-07	2.57E-05
PAM	0.025235943	5.596760584	0.107794843	0.742668779	0.869455911
TMCO1	0.01669708	5.596772599	0.066689896	0.796218702	0.900610289
SSU72	-0.049822565	5.596993679	0.49783964	0.480450907	0.696466157
B4GALT7	-0.110365189	5.597222359	2.52910405	0.11176366	0.299579077
CUTC	-0.022255274	5.597720076	0.126290346	0.722309777	0.856852067
IP6K2	-0.083342623	5.598996306	1.442845019	0.229679509	0.460787164
PABPC4	0.121924879	5.599002532	3.681413659	0.055022181	0.191010545
PHF10	-0.087768322	5.599464385	1.521449483	0.217400275	0.446386678
GPATCH1	-0.264451493	5.599870116	13.97989283	0.000184776	0.003352251
RANBP10	-0.072725918	5.600015474	0.716104227	0.397424774	0.629960954
TCAIM	-0.126734657	5.600186056	3.859952108	0.04945174	0.178292809
UBQLN1	0.200061589	5.60019708	4.209496165	0.040198275	0.157042407
PIAS1	-0.06099122	5.600386748	0.721633666	0.395608559	0.628430253
ENG	0.003699058	5.60095082	0.001389421	0.970265792	0.987977946
ZDBF2	0.000150478	5.601059777	5.02E-06	0.998212086	0.99928772
PAXIP1	-0.192221002	5.60140241	6.444059019	0.011132399	0.066953081

EBAG9	0.003020751	5.601702154	0.002216675	0.962448208	0.983899168
DHODH	0.06158034	5.601915992	0.432068617	0.510975651	0.718764262
HECW2	0.006986212	5.601919788	0.00991209	0.920694065	0.963741784
GAN	-0.290370047	5.602407842	4.766494777	0.029018851	0.1266592
HID1	-0.070349565	5.602681611	0.784988637	0.375619766	0.61015037
USP6NL	0.082000428	5.603271827	1.521440913	0.21740157	0.446386678
LAMTOR1	0.233863184	5.603300214	9.319642852	0.002267102	0.021854224
SFXN2	0.207248594	5.603346086	6.038783462	0.013994925	0.078480313
SNRPB2	0.118421922	5.603691486	1.886655392	0.169579643	0.388065135
S1PR3	0.166725534	5.604671984	2.939400289	0.086442822	0.254776398
MRPL1	0.055487135	5.60499729	0.599974131	0.438587897	0.664069883
RNF2	-0.019117062	5.605188581	0.040999741	0.839538423	0.923898005
NEUROD4	-0.237347475	5.605192782	2.503487915	0.113594468	0.302459056
MTMR14	0.201446784	5.605249709	8.659138532	0.003254251	0.02803597
AZI2	-0.081497536	5.605619392	1.782350648	0.181860945	0.404038672
NIF3L1	-0.152917144	5.606739381	5.004836254	0.025276594	0.115869639
TRAPPC11	-0.111016747	5.606975887	2.826640254	0.092712131	0.266151102
ACOT7	-0.017736889	5.607179815	0.026903541	0.869713046	0.939975578
LRRC8D	-0.13108246	5.607559157	3.370937954	0.066355967	0.216110872
GLIPR2	0.337700271	5.607621371	19.17630671	1.19E-05	0.000373695
PDE3A	-0.1566467	5.607730384	3.533945163	0.060124567	0.202569563
NSMAF	0.248790122	5.607821767	13.26078795	0.000271016	0.004583313
LOC112531359	0.077649383	5.607945299	0.973163437	0.323892492	0.56166624
AKAP17A	-0.057497563	5.608230087	0.745278034	0.387975364	0.621932256
CDAN1	0.113622967	5.608784491	3.591592177	0.058072579	0.198171773
PYGO2	-0.000792657	5.608827133	0.000179425	0.989312691	0.995057477
GPRIN3	-0.070407597	5.609127783	0.808655692	0.368517994	0.603219096
SYNPO	-0.139468052	5.609728757	1.048037097	0.305959594	0.544100846
AFF3	0.177327354	5.609900685	2.416624658	0.12005336	0.313021397
GATB	0.149367586	5.610356761	3.898960598	0.048315991	0.175408016
LOC101750690	0.099376931	5.61042819	2.127545044	0.144672299	0.353434606
EPAS1	-0.285793449	5.610592739	2.973291913	0.084649439	0.251291092

AP5M1	0.118545129	5.610703443	2.748301502	0.097357801	0.273597379
IAH1	0.072300163	5.61133974	0.85771452	0.354379058	0.591170661
SLC29A1	0.048324327	5.612131884	0.388419409	0.533131399	0.73534595
SUPT4H1	-0.138393497	5.612486198	4.495950895	0.033975213	0.140432588
EFCC1	-0.029822872	5.612538144	0.144226746	0.704114695	0.846723552
COQ4	0.141655658	5.612871263	2.969725748	0.084836236	0.251493697
VAMP7	-0.057834831	5.613100225	1.004667182	0.316183816	0.553730437
MPP5	-0.009647246	5.613512785	0.019661117	0.888487666	0.949552787
PIK3C3	0.147556072	5.614090806	2.607087991	0.106386956	0.290287555
LRRC8C	0.080093607	5.614208576	0.767652009	0.380944404	0.615531007
HMOX2	0.027393369	5.614278818	0.147916455	0.700534474	0.844482927
SEC61G	0.04856731	5.614702923	0.239758961	0.62438026	0.798242342
GPD1L	-0.022049552	5.615202601	0.118055792	0.731152781	0.862328105
EEF1E1	-0.119990581	5.615757614	2.460054314	0.116774744	0.308134768
TIMM22	-0.033044635	5.615984467	0.394489921	0.5299487	0.733281956
DHRS4	0.043119242	5.616878781	0.173019173	0.677442395	0.830340008
RFX4	0.18849531	5.617029505	3.021791975	0.082152643	0.246148666
FOXK1	0.022343759	5.618123916	0.08810709	0.766597534	0.883544332
LOC100858388	0.355866703	5.618140353	7.648713109	0.005681284	0.041753247
KLHDC8A	0.160582375	5.619233765	4.252031051	0.039203418	0.154217605
GJC1	-0.08471347	5.619321284	1.67906848	0.195048276	0.420031382
C14H7orf50	0.34518421	5.619800262	22.24157338	2.40E-06	9.91E-05
SIAH1	-0.011481354	5.620243592	0.026453886	0.870796768	0.940547786
MORC3	0.084663108	5.620308428	1.734610766	0.18782313	0.411143602
LOC107050785	0.149626788	5.620721513	1.941557394	0.163499534	0.380007306
SPRED1	0.070963885	5.620946811	0.989807375	0.319789458	0.557135546
BTBD10	-0.093663242	5.62137298	1.895759976	0.168553681	0.386836922
GNG12	-0.032212235	5.621989519	0.21984046	0.639161506	0.806858657
DSCR3	0.107204288	5.622038797	3.536551704	0.06003014	0.202334279
HOMER2	-0.031405282	5.622313783	0.086373363	0.768839319	0.884764739
RNF185	-0.041040016	5.622340705	0.548298997	0.459013557	0.681397294
PRELID3B	-0.081233299	5.622808731	1.422066819	0.233063514	0.464685826

LIN54	-0.089247989	5.623127653	1.481851979	0.223484988	0.452904577
FAM76A	0.062761599	5.623133609	1.125630922	0.288709193	0.525172427
ARHGEF10	0.022277434	5.623316103	0.103550716	0.74760954	0.872702382
MTBP	-0.043202042	5.623703574	0.400812736	0.526669824	0.730729536
PLBD2	0.201773995	5.624231488	4.442040659	0.035064312	0.143174449
ERCC6	-0.114527569	5.625623928	2.962337656	0.085224645	0.25228531
PPM1D	-0.0056032	5.626599044	0.007054402	0.933063997	0.970823147
DLL1	0.252521585	5.626697737	7.290266383	0.00693292	0.04799377
MEGF9	-0.148987057	5.626715853	3.114999983	0.07757448	0.23788447
PCK2	-0.352362132	5.626963894	15.40477038	8.68E-05	0.001851859
SLC35D1	-0.081040407	5.627452546	1.892524676	0.168917438	0.38738434
MGST1	0.065800231	5.627862957	1.08777513	0.296964694	0.533932916
CCDC61	0.07770126	5.628263398	0.972085729	0.324160554	0.562071857
ST6GALNAC3	-0.090956916	5.629232962	1.33008486	0.248790122	0.482992149
SBF2	-0.067017415	5.629743316	0.838838189	0.359729261	0.596167286
ATAD5	-0.228430807	5.630046635	6.635917891	0.009994269	0.061922705
ORC1	0.098026281	5.630133293	1.287144625	0.256574827	0.491380212
LOC107056600	-0.092264894	5.630683954	1.419446501	0.233494528	0.464982749
EXD2	-0.083507444	5.63075386	1.720395115	0.189642193	0.41314838
GTF2E2	0.179732713	5.631248792	8.823478921	0.002973792	0.026269859
GTF3C4	-0.074257849	5.631504509	1.028378609	0.31053979	0.548336682
TMEM9B	-0.084831544	5.632161103	2.198764879	0.138121369	0.343062208
ERAL1	0.006290217	5.632273009	0.007064519	0.933016125	0.970823147
L3MBTL3	-0.111080082	5.632557944	1.311255434	0.252167478	0.486934484
SRFBP1	-0.022363731	5.632566245	0.065754243	0.79762199	0.901282145
OSBPL1A	-0.104588783	5.632659124	2.805977233	0.093913604	0.268569406
EIF2B1	-0.160556025	5.632705744	5.609572193	0.017862623	0.092198704
PFDN4	0.182867776	5.632824521	4.771672595	0.028931705	0.126457074
UST	0.024468547	5.633451795	0.108272479	0.74211953	0.869036926
PGS1	0.084333253	5.633501206	1.342480655	0.246596973	0.480207478
VPS41	0.583702741	5.633698349	52.79102093	3.71E-13	1.03E-10
ATG101	0.058850227	5.634056979	0.873976757	0.349856722	0.586480476

SNF8	-0.008915156	5.634451263	0.019746878	0.888246318	0.949552787
SEMA6D	-0.191294364	5.634493999	4.992359218	0.025459475	0.116565291
LOC107051846	0.165785126	5.635448766	3.470234387	0.062482457	0.208060871
PPP6C	-0.046372164	5.635585851	0.695629192	0.404256258	0.635646128
POLR1B	-0.08845467	5.635821884	1.9471323	0.162896208	0.379345755
ADAMTSL2	-0.16319679	5.63610856	1.228849131	0.267630971	0.503053957
TRNAU1AP	0.049447021	5.636718454	0.374048623	0.54080565	0.741504513
PANX2	-0.213104719	5.636774189	7.726813107	0.00544067	0.040476061
WDR92	0.040129433	5.637142511	0.463195136	0.496134496	0.707872464
LOC107056693	-0.127639188	5.637197179	2.825181932	0.092796376	0.266209853
AGPAT4	0.048298407	5.637726885	0.572644409	0.449210008	0.672588779
PAX7	0.086488769	5.637890954	0.661154733	0.416152575	0.646383861
IPMK	-0.081045405	5.638049337	1.385261313	0.239206983	0.47181432
NEK2	-0.132678161	5.638066774	2.265979122	0.132242609	0.333699147
DAGLA	0.065673974	5.638165364	0.759788605	0.383394686	0.618081666
GPX3	-0.026280685	5.638593531	0.060753504	0.805308935	0.905731091
DDX31	0.28790453	5.638806691	23.06700511	1.56E-06	6.90E-05
ING4	-0.007068662	5.638839217	0.010511464	0.918339629	0.962372833
SLC4A8	0.069554408	5.639554192	0.705424614	0.400966927	0.63362247
GALNT18	-0.097529184	5.640038473	1.420847869	0.233263898	0.464849933
SMIM14	-0.180667999	5.640980607	7.579925238	0.005902189	0.042878815
MRPS6	0.17638969	5.640999626	4.947812084	0.026123709	0.118420283
DTD1	0.23277218	5.64117937	12.40036698	0.000429249	0.006456399
ARHGAP39	-0.162413648	5.641388935	3.91855645	0.04775587	0.174085699
PLEKHA7	-0.292814542	5.64262351	8.628622565	0.003309217	0.028375803
CNOT10	-0.101708065	5.642789187	1.814448816	0.177975473	0.398874371
GTF2H1	-0.004536574	5.642874514	0.003729934	0.951300924	0.979639321
PSKH1	-0.22127749	5.642877537	9.65969263	0.001883551	0.019262795
KBTBD11	-0.17771749	5.644508278	3.238104556	0.071943826	0.226571418
CSRNP2	-0.052810011	5.644597919	0.682370853	0.408771523	0.639203213
LRIF1	0.032657995	5.64466948	0.157764246	0.691223318	0.838989066
MLLT3	0.18690612	5.644756339	4.336686867	0.037299438	0.148899141

RBP5	0.187336771	5.644781498	4.5427717	0.033058023	0.13830912
NCAPG2	-0.015753757	5.645536705	0.041574809	0.838432413	0.923661189
SEC61A2	0.062317838	5.645624873	1.133060754	0.287123415	0.523619173
LOC107049580	0.17236117	5.645856822	2.704375968	0.100073233	0.278206631
IFT80	0.060901518	5.646027355	0.48084472	0.488039942	0.702056988
LRRFIP1	0.130862386	5.64654959	2.850308823	0.091356387	0.263889312
ETV1	-0.027436835	5.646843263	0.202742133	0.65251652	0.814655489
SPTBN2	0.499298306	5.647612119	17.87282768	2.36E-05	0.000647694
HS2ST1	0.061260909	5.648270765	0.944754755	0.331058033	0.568878379
BTBD6	-0.032699422	5.649912011	0.161160266	0.688090527	0.837680081
SCFD1	0.069244674	5.649963056	1.396802647	0.237259646	0.469624971
ARHGAP12	-0.104749742	5.650020148	2.070537628	0.150168576	0.361300171
ASPG	-0.102792368	5.650489084	1.687969174	0.193868893	0.418595019
SLC38A2	-0.185345833	5.65090941	3.75046752	0.052792743	0.186176996
ZNF330	-0.051027421	5.651614275	0.524615855	0.468879304	0.688269654
C2H5ORF22	-0.042472689	5.651870442	0.348199234	0.555134274	0.752697304
COX8A	0.078846998	5.651966143	1.160427322	0.281376935	0.517209279
NRF1	-0.13870569	5.652653149	3.815162709	0.050790672	0.181568652
CAMK2D	-0.347361334	5.652665493	4.129415449	0.042143906	0.161461693
SLC25A15	0.191517781	5.65290325	8.137858911	0.004335044	0.034360863
MRPL40	0.124295156	5.652937932	3.796241391	0.051367769	0.182758193
FAM155B	-0.206586621	5.653226064	5.362956084	0.020568879	0.101002957
CDC123	0.026444835	5.653475047	0.212095483	0.645129921	0.81068921
RAB41	-0.154751549	5.653836252	3.567293892	0.058928269	0.19993086
HMCES	0.21487818	5.654248783	8.142802197	0.004323242	0.034316908
TMEM254	-0.078446532	5.654643987	1.057898467	0.303694986	0.541771573
PEX16	-0.125885506	5.654789103	2.871290958	0.090172513	0.262036199
NUMB	0.090507164	5.655281402	1.746551995	0.186310782	0.409122572
SP4	-0.080038832	5.655500015	1.322428846	0.250156614	0.484387747
ROBO2	-0.036590848	5.655627275	0.094982826	0.757935602	0.878359766
MRPL46	-0.070352547	5.65645544	0.773500758	0.379136239	0.613681567
ANKLE1	0.334586638	5.657947887	10.5675442	0.001150905	0.013292521

TMUB1	0.007586634	5.658277515	0.011372816	0.915071883	0.961017703
FAM199X	0.054448436	5.658841203	0.550904531	0.457948339	0.68065576
TMCO7	-0.061514597	5.658869423	0.36207871	0.547354033	0.746757539
CHCHD6	0.251816274	5.6591547	11.24534974	0.000798228	0.010171186
DHX34	-0.117410972	5.65973892	2.566115397	0.10917553	0.295037502
JAKMIP2	0.280005291	5.660080742	14.40239859	0.000147614	0.002801605
MED10	-0.198839023	5.660405737	7.049091271	0.007930547	0.052881874
TPRA1	-0.09902688	5.660559007	2.594696154	0.107222	0.291626119
RHOJ	-0.275633594	5.660853449	6.460161452	0.011031962	0.066568
LOC107049257	0.15662626	5.661974269	4.170289509	0.04113877	0.159204716
PYCRL	-0.054187697	5.661993343	0.65228462	0.419297051	0.649012971
TMEM185A	-0.095623118	5.66203458	2.277367176	0.131274553	0.332071141
MSANTD4	0.000428368	5.662661963	3.91E-05	0.995009178	0.99763501
AURKAIP1	0.028398908	5.663238447	0.156668854	0.692242125	0.839288299
ZBTB37	-0.072819749	5.664069687	0.79444634	0.372759027	0.607362095
SDAD1	0.055707711	5.664502714	0.563474866	0.452863544	0.675632701
UBTD2	0.017027482	5.664773124	0.081548512	0.775209781	0.888893846
C1D	-0.183099969	5.665060746	5.403690099	0.020094223	0.099346821
FAM107B	-0.029820733	5.66512361	0.18417884	0.667806337	0.824166458
GALNT11	0.296009903	5.665543474	25.81164938	3.76E-07	2.13E-05
ZGPAT	-0.01373959	5.665736562	0.05132302	0.820776907	0.915860806
SLC7A1	-0.160172636	5.665855898	3.46227393	0.062783917	0.208751134
01/mar	0.164537934	5.665895626	2.227394011	0.135582356	0.338698598
PPM1K	-0.018480543	5.66617098	0.059891249	0.806667908	0.906612966
ACTA1	-0.154903194	5.666252116	0.882151057	0.347613198	0.584088143
PAX2	0.441296102	5.66638433	11.17293051	0.000829994	0.01050352
ORAI2	0.084535586	5.6668491	1.599603387	0.205959426	0.432885726
C33H12orf10	0.155806081	5.666910175	3.661939894	0.055668781	0.192449429
ZFYVE1	0.013265562	5.667590948	0.041288087	0.838982853	0.923751278
TEAD4	0.142874306	5.667988756	4.15885374	0.041417424	0.159741682
CWC27	0.204305204	5.668195766	4.962563124	0.025901788	0.117673638
LOC107050018	0.170358815	5.669139122	3.116722946	0.077492483	0.237721627

HERC3	0.133061389	5.669276703	4.032196926	0.044639775	0.166715272
ABHD13	0.029990057	5.669290075	0.24917644	0.617655569	0.793398483
MRPS35	0.098275795	5.669454989	2.067347657	0.15048303	0.361805708
BICC1	-0.061997778	5.670386657	1.058055142	0.303659181	0.541766435
SMARCAL1	0.028636621	5.670555943	0.155048654	0.69375663	0.83995036
PAK4	-0.036969874	5.670796583	0.296917206	0.585821542	0.772411336
CRIPT	-0.013075953	5.670801907	0.033785268	0.854164321	0.932178863
CHPF	0.078569337	5.671132464	1.592756284	0.206932779	0.434352468
FAM179B	0.003890857	5.671514697	0.004013672	0.949484979	0.978504123
ALAS1	-0.270413338	5.672298439	7.443275969	0.006367388	0.045416287
ANXA4	-0.030127629	5.672355124	0.202545859	0.652673702	0.814728097
PHPT1	0.195500199	5.673168981	5.168144645	0.023004773	0.108818545
TNNI1	-0.087323376	5.673304665	0.183834738	0.66809823	0.824166458
GZF1	-0.204992006	5.673348356	7.876588139	0.005007887	0.038274401
ADIPOR2	-0.059776164	5.673681534	0.853432321	0.355583161	0.592237747
CDK5	-0.078015262	5.674414336	1.146637451	0.284254157	0.520218204
MNR2	-0.496139084	5.676774738	7.452533998	0.006334721	0.045281419
EMG1	0.016203233	5.676967858	0.029942109	0.862621651	0.936001285
CASKIN2	-0.147047603	5.677053682	2.303261193	0.129102682	0.328293098
ARAP3	-0.087625513	5.677408279	1.105575444	0.293045775	0.530369636
DPH1	0.005700112	5.678189842	0.007497052	0.931000979	0.969780408
STON2	0.150323722	5.678489929	1.834876371	0.175552657	0.395281742
NDUFAB1	0.133748552	5.678629921	4.873419398	0.027273407	0.121841837
HJURP	0.188604404	5.67882614	7.631358051	0.005736209	0.041969465
LOC107050028	0.033251527	5.67946485	0.217352779	0.641064413	0.808082957
URB2	0.11096329	5.679580445	2.690513368	0.100947286	0.27992713
CASD1	-0.188032763	5.679870929	11.05711803	0.000883474	0.010946957
NTAN1	0.043858496	5.680595887	0.449771494	0.502443488	0.713233384
ABHD18	0.014082429	5.680932577	0.016912054	0.896529754	0.95275104
RNF34	-0.008171447	5.681825132	0.016673407	0.897258307	0.953032884
RNF25	-0.189521708	5.681855102	9.463748394	0.002095724	0.020835945
XPC	0.100480203	5.681968054	2.10305301	0.147005353	0.356570325

ATP9A	-0.109562831	5.682143717	1.499993187	0.22067241	0.450069969
BACE2	0.044769674	5.68312467	0.437309873	0.508423731	0.717589112
FLI1	-0.072841558	5.683332016	0.677628739	0.410404457	0.640661251
SLC6A8	0.074403381	5.683613986	1.143229686	0.284970912	0.520848201
BTBD2	0.195881447	5.684526706	6.250621251	0.012414976	0.072365719
HOXA6	0.192758128	5.68487745	7.726675411	0.005441085	0.040476061
USP31	-0.199616965	5.684986845	7.158490221	0.007460997	0.050583386
GEMIN2	-0.055382989	5.685451346	0.815844307	0.366398001	0.601466431
SCO1	0.335772481	5.685924266	16.7927234	4.17E-05	0.001034611
MTSS1	0.087482503	5.686129488	1.310826177	0.252245126	0.486934484
LZTR1	0.268067917	5.686140565	20.52296813	5.89E-06	0.000212151
ARMC9	0.001955781	5.686264484	0.000566394	0.981012918	0.991828264
HES1	-0.233497022	5.686382935	5.522891174	0.018769214	0.095070641
RWDD1	0.058623533	5.686795546	0.625334243	0.429071927	0.656474064
MFSD2A	-0.06060897	5.687633278	0.942038795	0.331754067	0.56937808
RAD54L	0.191776807	5.688090133	8.126671745	0.004361875	0.034540253
MOXD1	0.10780161	5.688133538	1.481741881	0.223502188	0.452904577
PCYT2	-0.12507466	5.688474288	2.529781817	0.111715662	0.299578911
PBDC1	-0.157182143	5.688597361	4.912780868	0.026658688	0.120183209
FOXO3	0.131308645	5.688739797	3.66546764	0.055551052	0.192197764
UBE2M	0.119439278	5.6895123	2.909755222	0.088045237	0.257470121
NCBP3	-0.083543547	5.689596435	1.823109486	0.176943592	0.397217844
FBXW2	-0.083821375	5.689935078	1.725204328	0.18902451	0.412574387
CEP192	0.005653775	5.690163834	0.005151361	0.942782572	0.975445625
MED20	0.032536295	5.690206741	0.334973111	0.56274531	0.757276257
DHCR7	-0.146511249	5.690509801	3.160595127	0.075435679	0.233768701
LOC101748694	-0.251923715	5.69153723	11.70025296	0.000624916	0.008540692
GOLGA3	-0.213481339	5.692784072	9.212689435	0.002403434	0.022697352
NT5C3A	0.086232265	5.69280916	1.751229019	0.185722302	0.408345869
KIF1BP	0.205033181	5.693175965	12.0310376	0.000523219	0.00749963
SHOC2	0.160814067	5.693255768	4.825073359	0.028048672	0.124192137
COG7	-0.080080109	5.694012833	1.400583709	0.236625863	0.469004888

VPS13B	-0.044986276	5.694500058	0.185044948	0.667073066	0.823888674
POLR2D	-0.075446579	5.69450701	1.08071828	0.298536927	0.535472546
NAA10L	0.139442547	5.694851463	4.146725351	0.041715123	0.160433183
RIOK2	0.063537406	5.69568371	0.465100448	0.495249869	0.707178212
IRX3	-0.127325043	5.696367905	1.12938587	0.287906372	0.524698123
TMEM50A	0.111797779	5.696755777	2.661207181	0.102822671	0.283723182
GOSR1	-0.166721823	5.697918705	6.854218176	0.008843224	0.05688634
ADIPOR1	0.020568947	5.697921897	0.103296174	0.747909389	0.872702382
NDUFS4	0.08475585	5.698896963	0.949316789	0.329893264	0.56752678
SCG3	0.002872695	5.698909816	0.000814685	0.977229316	0.990525896
CRCP	0.220274851	5.698916352	9.655001338	0.001888368	0.019300055
GALNT10	-0.044676638	5.698988694	0.290954255	0.589609669	0.77529384
CETN2	0.020730689	5.699046245	0.081755435	0.774932446	0.888854969
ZBTB8OS	0.073404443	5.699544075	1.386098664	0.239065048	0.471812538
ZBTB47	-0.219502825	5.699544616	8.820324789	0.002978936	0.026297424
NHP2	0.023158214	5.699861991	0.088529008	0.766055614	0.883053012
PCDH19	0.046928966	5.700114458	0.443421345	0.505475723	0.715440088
SNX13	0.005209384	5.70086065	0.006174357	0.937369023	0.972841834
CMTM4	-0.184570298	5.701162229	3.949451775	0.04688664	0.172329611
CNPPD1	0.017807865	5.70160817	0.079106501	0.778511924	0.890292148
PPP1R12C	0.187685353	5.701627618	5.342367742	0.020813185	0.101807658
MYLK	0.081832364	5.701664091	0.510847209	0.474772336	0.692265465
LOC776992	0.118467116	5.70172913	2.635278235	0.10451375	0.286868017
HDX	0.051487029	5.702324529	0.505726079	0.47699488	0.693600685
FAM13B	0.066702957	5.702357576	1.219594884	0.269440171	0.504156042
SFT2D2	0.012721903	5.702364727	0.033112549	0.855607415	0.932779908
DCDC2	-0.036227777	5.702381649	0.130951064	0.717447974	0.853808231
MLLT11	0.24106972	5.702382106	5.663063302	0.017325837	0.090737315
ARHGAP26	0.019958892	5.702498897	0.102553351	0.748786766	0.873168892
MAML3	0.174642838	5.702809283	4.679622853	0.030522449	0.130805141
GCDH	0.028287553	5.703085141	0.156132798	0.692742205	0.839401545
TRIAP1	0.100342399	5.703092944	2.117761023	0.145599259	0.354780957

FAM96B	0.110663471	5.703943595	3.137967151	0.076489087	0.23605175
SH3BP5	0.14866742	5.704069957	4.652673908	0.031005215	0.13235142
TRANK1	-0.21530127	5.704576167	5.293882984	0.021400452	0.103862114
HHIP	-0.074487239	5.704816797	0.346028651	0.55636987	0.753031308
ECHS1	-0.006861418	5.7059877	0.008126329	0.928171072	0.968119612
MRPS25	0.033263557	5.706147891	0.245675198	0.620136902	0.795058816
DCLRE1A	0.196049545	5.706241422	7.166073076	0.007429523	0.050392748
GIPC1	0.019178335	5.706414541	0.098977297	0.753060386	0.874832943
ZNF827	-0.00919065	5.70648597	0.011779907	0.913571097	0.960378994
LOC419409	-0.008535208	5.706756084	0.012767124	0.910037135	0.958869249
SPTLC1	0.141815054	5.706828519	2.27093655	0.131820221	0.333195351
ANK2	-0.160319965	5.706891242	1.719272619	0.189786702	0.413196637
C2CD2L	-0.088836517	5.706954685	0.83304993	0.36139209	0.597823318
TTBK1	-0.363988105	5.70799523	19.37127245	1.08E-05	0.000345182
TRIP13	0.220292308	5.708139871	8.6998875	0.003182297	0.027522555
C25H1ORF43	0.342401466	5.708245714	23.02032432	1.60E-06	7.00E-05
BCCIP	0.385257178	5.708404064	40.5941439	1.87E-10	2.68E-08
PODXL2	-0.162524373	5.708576097	3.570274962	0.05882257	0.199860705
TMEM43	0.222190275	5.709040332	10.41066159	0.001252898	0.014072659
ZXDC	-0.009455848	5.709261632	0.018207287	0.892663757	0.951217859
DNAJB2	0.037087971	5.709437015	0.117172434	0.73212168	0.862661099
TXNIP	-0.351967314	5.709630909	6.148001361	0.01315607	0.075324678
CPT2	-0.066759216	5.709741573	1.342199016	0.246646539	0.480247167
CIAO1	0.038586894	5.710622483	0.222997949	0.636765065	0.805440633
LOC101747338	0.036498299	5.710891257	0.177861456	0.67321755	0.827473733
PDCD10	-0.015824292	5.711224029	0.043699362	0.834413949	0.921720365
FAM53A	0.091775211	5.711843121	1.485807561	0.222868081	0.452323547
FAM43A	0.322819047	5.711845122	12.93952987	0.000321716	0.005185773
WDR59	-0.130454308	5.712142037	2.391050061	0.122031635	0.316524361
NHS	-0.040271207	5.712195346	0.38910914	0.532768048	0.735091248
UBL3	0.162389387	5.712650038	8.309216731	0.00394444	0.032188403
MRPS22	0.273894994	5.712886369	13.5619623	0.000230816	0.004023381

MLH3	0.151939143	5.713142848	5.128648879	0.023534041	0.110580422
PCCB	0.292847389	5.713874587	16.01881668	6.27E-05	0.001441331
DCUN1D1	-0.136548347	5.71388482	4.172981166	0.041073469	0.159026807
CCDC30	0.168971171	5.714021247	7.84731601	0.005089621	0.038629944
LMO7	-0.025537679	5.714042788	0.076837829	0.781629325	0.892368733
CSRNP1	-0.055113642	5.714200826	0.314026929	0.57521955	0.76554818
MKNK2	0.299725184	5.714575211	13.89968676	0.000192831	0.00346779
KIAA1468	-0.144590324	5.714607872	5.560200082	0.018373314	0.093729893
THG1L	0.07852252	5.714842686	1.140045084	0.285642801	0.52190232
LOC107051757	-0.144738521	5.71565637	2.434862024	0.118664336	0.310927013
MAGI3	-0.123760869	5.715725221	3.332548783	0.067921542	0.219170974
RRP15	0.165087201	5.715775667	5.189596126	0.022722496	0.107876133
ATP6AP1	0.157431281	5.715915541	5.613062113	0.017827084	0.092131151
RPE	0.064112615	5.716155917	1.227803713	0.267834589	0.503053957
TBC1D16	0.16679583	5.716667974	5.063085955	0.024440681	0.11328772
ZNF767	-0.027803472	5.717298818	0.167171251	0.682638141	0.833665624
RPAP3	0.077778467	5.717325219	1.470366625	0.225287857	0.455139557
SMCR8	-0.037894443	5.717820859	0.215512335	0.642480777	0.809078732
LARGE2	0.016700732	5.718720784	0.052910763	0.818073589	0.914062667
CLMN	-0.581682042	5.718834446	12.52376982	0.000401807	0.006156183
KCMF1	0.108355269	5.719659022	1.655072648	0.19826992	0.423935622
ABTB1	-0.070345341	5.720025575	1.255641209	0.262477766	0.497588898
ITPK1	-0.072564767	5.720324177	0.819661384	0.365279188	0.60086122
PEX14	0.061722738	5.720596409	1.057103075	0.303876838	0.541885862
PACSIN2	0.236057548	5.720708478	15.68333595	7.49E-05	0.001658367
CRYM	0.139526692	5.72133074	3.271789112	0.07048072	0.224194905
AGA	-0.251639194	5.721396381	12.87591798	0.000332837	0.00531732
DTL	0.023548776	5.721653758	0.114754039	0.734795317	0.864635078
MTMR6	0.059607867	5.721731692	0.919955863	0.337486562	0.574603067
SLAIN2	-0.118472362	5.722429	3.148440351	0.075999572	0.235104578
SMPD4	-0.050172955	5.723041583	0.736688772	0.390723651	0.624148887
SCAI	-0.031641	5.723164318	0.287407684	0.591886556	0.776980894

NCOA1	0.124125133	5.723979621	3.539701714	0.059916235	0.202033125
RRM2B	-0.012676973	5.724021878	0.019030199	0.890279943	0.950652593
DUSP7	0.005386548	5.724080716	0.006002862	0.938243182	0.973257994
NCDN	0.086184922	5.724196976	1.820160961	0.177294118	0.397841909
DNAJC1	0.040834793	5.724911878	0.393982464	0.530213443	0.733349769
ARHGAP23	-0.11411478	5.72540727	2.321943344	0.127560573	0.325528727
RYBP	-0.144777488	5.725554358	4.807835388	0.02833061	0.124748861
SOWAHC	0.085905229	5.725931164	1.603853418	0.205357979	0.432228901
INTS9	-0.116905196	5.726254027	3.257359376	0.071103541	0.225216689
ERI1	0.00739324	5.72629588	0.011131944	0.915972701	0.96163855
NDUFB3	-0.165256159	5.72630113	4.413186246	0.035662172	0.144864108
MAST4	-0.111953983	5.726478686	1.323003119	0.250053795	0.484356521
ACY1	0.021863353	5.728231284	0.078841674	0.778873318	0.890625824
COIL	0.063996462	5.729159472	0.697952748	0.403472457	0.635202763
RSU1	-0.172330349	5.729473213	6.049750583	0.013908261	0.078259853
MTCL1	0.226358619	5.730007123	3.36217756	0.066709803	0.216810155
VMA21	0.009233745	5.730643606	0.020535829	0.886050669	0.948791653
IRAK2	0.387516034	5.730677265	33.83439966	6.00E-09	5.58E-07
ELK4	-0.168567326	5.73112461	5.029232395	0.024922942	0.114806842
SOX5	0.012480792	5.731999592	0.020734955	0.885503334	0.9486918
NBEAL2	-0.227733605	5.732246287	11.61805797	0.000653146	0.008831043
NOA1	0.156174122	5.732748814	6.850462114	0.008861835	0.056939278
SLC43A2	0.111212571	5.733317766	1.345116012	0.24613376	0.47986029
PPM1E	0.013338873	5.733508487	0.031432301	0.859279371	0.934098041
ZFPM1	-0.117059579	5.733684455	0.896923635	0.343607939	0.580619084
ARFGEF2	-0.13189561	5.73386627	2.924826074	0.087226624	0.256077448
RB1CC1	-0.185573371	5.734435996	8.415261771	0.003720842	0.030976225
KIAA1143	-0.433308428	5.734583	22.28794468	2.35E-06	9.73E-05
TMEM161A	0.050138708	5.734817756	0.428415576	0.512767427	0.720237986
C7orf73	-0.007344892	5.734892855	0.008597692	0.926123037	0.967021008
TERF2IP	-0.131626125	5.735048916	4.312748605	0.037827795	0.150388107
UBLCP1	0.039889095	5.735244387	0.456287396	0.499364194	0.710379765

EPCAM	-0.167767918	5.735600992	3.047952962	0.080838974	0.243895366
KIAA2026	0.022090527	5.736356922	0.061218899	0.804579691	0.905502962
SOX4	0.025720796	5.736851033	0.055827975	0.813215838	0.910675556
MKRN2	0.057020687	5.73710889	0.631322827	0.426870507	0.654871047
PDZRN3	-0.027823166	5.737138423	0.114357317	0.7352369	0.864745668
ELMSAN1	0.070181849	5.738036665	0.486571697	0.485460591	0.700109905
ZC3H3	0.054512411	5.738512703	0.724160963	0.39478242	0.627589842
VIPAS39	0.116435278	5.738561264	3.878829844	0.048898627	0.176802221
SRSF7L	-0.112419026	5.738611126	2.146590081	0.142886927	0.350612047
AGPAT5	-0.117887306	5.738868615	3.580147915	0.058473944	0.199087266
RAD18	0.230036966	5.739371745	10.82531562	0.001001216	0.012043138
MIPOL1	0.228656253	5.739499693	6.725401466	0.009504933	0.059855981
PATL1	0.083889194	5.739567401	1.724352582	0.189133735	0.412641691
BUD13	0.323751624	5.73985678	26.70937569	2.36E-07	1.39E-05
POLR2F	0.068701034	5.739936676	0.947379525	0.330387215	0.568079584
LOC107049496	0.254987174	5.740385368	4.729958922	0.029641593	0.128187232
USP28	0.038142825	5.740720382	0.36395221	0.546319431	0.746341738
ZBTB26	-0.22243601	5.741014634	8.788402649	0.003031516	0.026604583
NTN3	0.026016816	5.741084629	0.062350501	0.802818721	0.904325168
SLBP	-0.0035059	5.741754179	0.00129538	0.971289223	0.988591833
DSG2	-0.074602077	5.742024279	0.331216869	0.564943429	0.758868908
PRKAG1	0.110974627	5.742221327	3.814217149	0.050819348	0.181580292
ACSS2	-0.10764321	5.742611346	3.495340745	0.061541742	0.206078422
PDDC1	0.02848851	5.742914747	0.232421131	0.629734378	0.801165857
ANKRA2	0.123918592	5.743034653	2.153970711	0.142201709	0.34945178
MYO10L	-0.059542907	5.74415574	0.523088694	0.469527099	0.688659275
XKR6	-0.534570076	5.745560823	14.66839102	0.000128178	0.002510913
CTDP1	0.294678761	5.745943816	16.91435875	3.91E-05	0.000976522
NHLH1	-0.185771647	5.745959381	1.006641295	0.315708828	0.553296004
LMBR1	0.144232838	5.746379969	3.946078297	0.046980735	0.172559821
CEP85	-0.120000788	5.746587175	1.725814311	0.188946333	0.412567928
FAAHL	0.213797107	5.746812899	7.026880687	0.00802951	0.053336266

LYSMD4	-0.047704527	5.746912617	0.224401454	0.635706503	0.804781544
ECI1	0.001724555	5.746970063	0.000661894	0.979474847	0.99116036
RFWD2	0.078905062	5.747664626	1.390214921	0.238368808	0.470995489
TIAL1	0.165240805	5.748648554	8.260840205	0.004050938	0.032707643
TIE1	0.151826491	5.748762994	3.978495636	0.046084705	0.17025581
MYO1C	0.08434905	5.749151764	1.856620295	0.173015374	0.392360526
CLCC1	0.231230075	5.749855327	9.616591232	0.001928273	0.019598353
PIM3	-0.026827156	5.750439032	0.100284939	0.751487964	0.874274211
MGST3	0.164600306	5.750809993	4.308549443	0.037921283	0.15049741
GOLGA4	-0.135832288	5.751909582	3.418890286	0.064454326	0.212161621
LGR5	0.003327358	5.751956122	0.001684635	0.967260572	0.986598106
MGAT2	-0.073591712	5.751968829	1.083141798	0.297995776	0.534872864
TOE1	-0.131774485	5.752067376	3.996162417	0.045603986	0.169240772
FRMD6	-0.187736512	5.752438711	4.066245206	0.043748419	0.164958806
PPHLN1	-0.129068613	5.752475505	3.809989564	0.050947768	0.181893148
SH3BGRL3	0.088831092	5.752632755	2.398754009	0.121431935	0.315600463
SNX3	0.139981256	5.753295177	4.587837898	0.032199608	0.135766182
TYW1	-0.007939789	5.753305444	0.013663294	0.906947148	0.957377047
WRB	0.13355426	5.753313315	3.564271543	0.059035638	0.200200812
PHGDH	0.257907714	5.753543287	4.203496693	0.040340716	0.15741623
STX18	-0.057521507	5.753848756	0.698806307	0.403185083	0.634993352
ZCCHC6	-0.266469454	5.753883954	6.350644559	0.011733866	0.069426128
POLR3E	0.067819924	5.754026299	1.097608884	0.294791454	0.531768622
TRAIL-LIKE	-0.107403154	5.754510852	2.299763709	0.129393682	0.328931413
GLB1L2	0.45268071	5.754617907	46.32639914	1.00E-11	1.94E-09
PLEKHH2	-0.115772544	5.754698396	1.385318438	0.239197297	0.47181432
STX6	-0.03916513	5.75568177	0.392296279	0.531094843	0.733723117
JAG2	0.068693792	5.75679412	0.660938148	0.416228937	0.64644145
SOX9	-0.128061162	5.756982	0.654528282	0.418498338	0.648252861
TENM2	1.64E-05	5.757075616	2.55E-08	0.999872573	0.999872573
CEP78	0.22388289	5.757288478	5.654538888	0.017410252	0.090912924
PEX6	0.584424836	5.75744209	40.03471309	2.49E-10	3.34E-08

SIX1	-0.001414009	5.757608824	0.00028862	0.986445535	0.993448882
LARS2	-0.022081717	5.758103194	0.124892221	0.723787885	0.857408332
TMEM127	-0.097051545	5.758113032	2.372211674	0.123511955	0.318606243
C6H10orf76	0.077113055	5.758129619	1.529236455	0.216227075	0.44508588
ENTPD5	0.022000067	5.75814896	0.070914773	0.790009498	0.896648248
TRPC1	-0.290101367	5.758185227	20.69395954	5.39E-06	0.000197481
TRIM2	-0.078541383	5.758293447	0.744280924	0.388292987	0.622198958
LSAMP	-0.035931263	5.758325331	0.179758226	0.6715811	0.826344165
LOC101749950	-0.090067391	5.758725907	2.02584604	0.154642779	0.367509705
ANAPC5	-0.318547195	5.758833446	14.8581626	0.000115905	0.00230341
LOC112531360	-0.037538167	5.759078858	0.301778762	0.58276944	0.770808073
PTPN21	0.104152061	5.759113057	2.129008242	0.144534245	0.35338945
THAP9	0.031808343	5.759442777	0.316399042	0.573779768	0.764869254
CHST2	0.144612746	5.759683054	4.955891681	0.026001912	0.118095905
CHMP3	0.009901459	5.759780452	0.012125577	0.912317223	0.959684907
ACAP2	0.072605726	5.760473473	1.322818408	0.25008686	0.484356521
ACSL4	0.043059373	5.761016523	0.365764624	0.545322011	0.745408895
HYPK	0.226898884	5.761162034	11.80249495	0.000591514	0.008186175
SAT1	-0.642088967	5.761271971	76.73825751	1.95E-18	1.19E-15
ASTN1	0.268828285	5.761449398	4.769542684	0.028967519	0.126535846
ZRSR2	-0.036600688	5.761601782	0.360649379	0.548145808	0.747409618
TCEB3	-0.164924791	5.761733218	8.375095065	0.003803977	0.031328546
GPC3	0.107849411	5.762118411	0.201555209	0.653468444	0.815062002
C9H21orf2	0.135638536	5.762387975	2.932657992	0.086804471	0.255293578
SPCS3	0.122575144	5.762695788	3.69613796	0.054538568	0.190053395
ZC2HC1A	-0.21808042	5.762781742	12.3527223	0.000440344	0.006587144
ELN	-0.012382453	5.76304122	0.009635062	0.921806555	0.964486558
MOCS1	-0.080434122	5.763147186	1.538220496	0.214882875	0.444040903
SMIM7	0.063700941	5.764408022	1.030989628	0.309926346	0.547741793
LIMK2	-0.174001769	5.764446767	7.333244046	0.006769077	0.047177115
CRELD2	0.308253083	5.765288168	14.65524372	0.000129075	0.002522482
CDC16	0.20054975	5.765695665	10.29548352	0.001333562	0.014776945

KCTD9	-0.073495141	5.765915246	1.479163999	0.223905368	0.453292671
TCTN2	-0.071732284	5.766325523	1.51848279	0.217849237	0.446915496
CDH6	0.3604136	5.766722066	11.76177205	0.000604596	0.008338694
MCC	0.194766293	5.76727731	5.255549911	0.021876887	0.105197013
BCAP29	-0.052567114	5.767486456	0.754415599	0.385081817	0.619718521
APPL1	-0.287770435	5.767843881	11.55929629	0.000674114	0.009047748
SLC25A17	0.099451556	5.76819147	2.352504558	0.125081902	0.321346245
TRMT61A	-0.226891051	5.768441999	9.519564756	0.002032928	0.020370105
FAM135A	-0.03283215	5.768474199	0.297147579	0.585676183	0.772343452
CEP152	0.146596332	5.768943963	3.559428119	0.059208134	0.200531809
WARS	0.056561012	5.768983401	0.810074732	0.368098157	0.60281202
TMEM8A	-0.096677483	5.769142885	1.984070875	0.158962205	0.37396064
SCNM1	0.00333569	5.769819851	0.002535475	0.959840688	0.982787361
FBXW7	-0.053367458	5.770608491	0.641173331	0.423286236	0.652625553
RNF219	-0.049412886	5.771064585	0.25423947	0.61410563	0.791006508
LOC107049046	0.203359362	5.771575369	0.106489162	0.744177135	0.870074943
LIPA	-0.179946207	5.771904934	8.815134585	0.002987422	0.026356798
LLGL2	-0.103576801	5.772084227	1.290897852	0.255882554	0.490715523
HEATR5B	0.11520557	5.772121764	1.509475798	0.219219092	0.448662956
FOXN2	-0.221108599	5.774916802	7.985398331	0.004715612	0.036705484
HDAC9	0.103137253	5.774917363	1.363187079	0.242985896	0.476013219
FHL3	0.143828293	5.775376318	3.213424767	0.07303646	0.228860693
FANCI	0.260104904	5.776135004	15.72412681	7.33E-05	0.001631771
FRRS1L	-0.534601604	5.776169578	39.36388566	3.52E-10	4.45E-08
AKNA	0.152186685	5.776551965	3.941207888	0.047116935	0.172801769
ARID4A	-0.166295323	5.776680658	5.628329502	0.017672465	0.091706216
RAP2C	-0.063235234	5.776719537	1.263941587	0.260906319	0.495752133
PSD3	0.033430449	5.77689491	0.110052635	0.740084219	0.867860967
SYF2	-0.032104901	5.776976136	0.227125929	0.633663146	0.80403489
ITPR3	0.03088514	5.777165066	0.120144372	0.728877959	0.860749736
SYNE1	-0.259540426	5.777181779	9.540435199	0.002009941	0.020203773
SNAI2	0.215884455	5.777188008	3.710432214	0.054073387	0.189191019

MIER2	-0.163398382	5.777736302	8.951170359	0.002772919	0.024974482
TADA3	0.064309399	5.777759786	0.958056363	0.327677062	0.565526033
RELT	0.071870439	5.777839388	1.227635128	0.267867443	0.503053957
CDC7	0.153199901	5.777845847	5.672068412	0.01723712	0.090358971
WASF1	-0.084775067	5.778050645	1.184672301	0.276406857	0.511559422
TNKS2	-0.13744178	5.778148964	3.054686196	0.080504545	0.243243167
BLOC1S4	0.139010419	5.778339848	4.120932803	0.042355718	0.162046812
PECAM1	1.251728147	5.778343923	75.13973057	4.39E-18	2.58E-15
UQCC1	0.198821068	5.778376467	9.762630389	0.001780949	0.018442743
UAP1	0.185013663	5.779228551	6.964401835	0.008314722	0.054640079
KRIT1	-0.269944512	5.779662701	22.76329265	1.83E-06	7.83E-05
TMEM198	-0.018368529	5.779674993	0.054621786	0.815207762	0.912285346
DNAJB12	-0.031813538	5.779945798	0.29248477	0.588632614	0.774756996
TMEM258	0.08721492	5.780405516	1.694092447	0.193062372	0.417328557
SLC8A3	-0.089499623	5.781304394	0.951325969	0.329382013	0.567121588
NCAN	0.018118384	5.781775602	0.018112305	0.892942404	0.951217859
PTPN23	0.117413957	5.782231913	2.476074515	0.115590424	0.305941842
ADAMTSL2L	-0.019162697	5.782846967	0.080110337	0.777147942	0.889536847
MRPS30	0.158561858	5.782967542	2.346227314	0.125586617	0.322171313
RHOBTB2	-0.118049003	5.783057328	3.349027831	0.067244716	0.21763368
ARHGEF18	-0.063528404	5.783255569	1.180598389	0.277234209	0.512364651
PFDN1	0.040605576	5.78328489	0.369653531	0.543193184	0.74336692
SFRP2	0.099289696	5.783842953	1.370099364	0.241794812	0.474797763
INSIG2	-0.043348765	5.784047239	0.326944473	0.567463857	0.760729568
VPS37C	0.080950046	5.784676061	1.420831086	0.233266659	0.464849933
ATE1	0.02122368	5.785334695	0.142906324	0.705408658	0.847437973
WNT5A	-0.242268148	5.785663427	1.733450869	0.187970789	0.411201719
ORC6	0.257761179	5.786187032	10.90512022	0.000958988	0.011654468
TXNDC16	-0.052507032	5.786318184	0.521867739	0.470046043	0.689065646
ABCB7	0.087989936	5.786666278	1.582023527	0.208469461	0.436100304
CENPK	0.33588711	5.786693898	11.28427193	0.000781665	0.010014937
NEBL1	0.029902675	5.786908524	0.099873187	0.751981868	0.874401564

SLC25A28	0.106314583	5.787115738	3.509632597	0.061012985	0.204724441
ERN1	-0.030833315	5.787534733	0.192466742	0.66087145	0.820108576
GNAO1	0.185356484	5.787842866	2.677488446	0.101776129	0.281513901
ATP6V1D	0.051456868	5.788107924	0.578563617	0.446875889	0.670621395
SPCS2	0.039381932	5.788428084	0.338615719	0.560629347	0.755726707
SLC7A6OS	-0.076021267	5.788446982	0.927141581	0.335606787	0.572942255
CYP20A1	-0.117022411	5.78947641	2.826193837	0.092737911	0.266178673
WFDC1	-0.288951687	5.789702841	6.935487109	0.008450205	0.055265548
DFNA5	-0.275591854	5.790204817	8.603293224	0.003355557	0.028660467
MED31	0.122360253	5.790309488	4.151490527	0.041597892	0.160152856
HYAL2	0.125928758	5.79038524	4.343383308	0.037153025	0.148542664
TM9SF2L	-0.122927388	5.791235775	3.459767604	0.062879152	0.208973648
GLUD1	-0.265671706	5.791469807	21.71231217	3.17E-06	0.000124992
DCK	0.253521897	5.79197046	16.842566	4.06E-05	0.001009474
TAF12	0.002390772	5.792463516	0.001849038	0.965701179	0.985815656
GPN2	0.025148155	5.792504848	0.158869667	0.690199326	0.838473731
ADO	0.049661545	5.792567764	0.658701139	0.417018865	0.64711858
PRICKLE1	0.034708942	5.793001217	0.136153667	0.712135132	0.850431227
BECN1	-0.037878153	5.793140659	0.494139347	0.482086624	0.697256981
KALRN	-0.187257235	5.794644221	5.124808303	0.023586176	0.110762135
GAK	-0.131359989	5.79499945	1.982023816	0.159177364	0.374203142
TCF7L1	0.053629892	5.795397963	0.603559584	0.437223098	0.663330972
PEF1	-0.089678446	5.795520265	1.37679397	0.240648007	0.473216236
GRIPAP1	0.127733535	5.795674724	2.852629273	0.091224634	0.263674925
MRPL15	0.026387951	5.795697793	0.10666112	0.743977902	0.869965632
RFX3	0.275056634	5.795805669	7.977920168	0.004735131	0.036822582
SOD2	0.037333645	5.795931788	0.258903859	0.61087422	0.788758262
SLC25A12	-0.051046887	5.795941728	0.669033248	0.413388864	0.643609969
ACADSB	-0.408492262	5.796067686	20.20589876	6.95E-06	0.000242067
NRK	-0.152354407	5.796069511	3.743442813	0.053015104	0.18668169
BCAS2	-0.044475634	5.796242276	0.536747694	0.463783722	0.684929547
NDUFA5	0.350009805	5.796273468	16.4885473	4.89E-05	0.001172325

SDF2L1		0.237434393	5.797074511	10.11129081	0.001473637	0.015958825
UNK		-0.156682147	5.797110012	3.666950734	0.055501637	0.192147998
	06/mar	-0.205264283	5.797197009	8.512494848	0.003527162	0.029687695
SQLE		-0.062926157	5.797527785	0.443018751	0.50566902	0.715440088
NKIRAS2		0.303241986	5.798327075	20.58810826	5.69E-06	0.000207321
MRPL4		0.043766082	5.798361432	0.394288192	0.530053915	0.733281956
PRMT9		-0.115451138	5.799084152	2.830381637	0.092496377	0.265821665
MUM1		0.198976362	5.799298938	9.250570843	0.00235422	0.022470241
GBE1		0.089100016	5.799384271	1.674704729	0.195629558	0.42063039
NEMP2		-0.095998363	5.799855367	2.071937908	0.150030777	0.361281572
BTG2		-0.26451343	5.80005548	3.070241737	0.079737614	0.241858514
SLC11A2		0.043028344	5.800921438	0.204683352	0.650966843	0.813770371
GPAM		0.015890653	5.80181622	0.04789607	0.826765566	0.917915823
SLF1		0.224795376	5.802193786	4.078217155	0.04343947	0.164493894
HDDC2		-0.065488316	5.802280112	1.12728672	0.288354827	0.525060454
TMTC3		-0.053528324	5.803083338	0.645980173	0.421553549	0.650779966
CDC42SE2		0.120068371	5.803262491	1.423168778	0.232882541	0.464382236
MAN1B1		-0.237909827	5.803316246	8.684784019	0.003208776	0.027687682
APELA		-0.254536935	5.803378978	5.345487284	0.020775976	0.101716359
ZNF395		-0.146236366	5.803748393	3.829887496	0.050346312	0.18068671
PLCH1		-0.114258599	5.804531447	1.763852589	0.184144703	0.406794321
RBM45		-0.031116699	5.804768123	0.305427783	0.580499528	0.769587474
LZTS2		0.055763962	5.80515585	0.734674584	0.391372157	0.624820884
FOXK2		-0.067498207	5.805217709	0.968206142	0.32512797	0.563215153
COL7A1L		-0.363550648	5.806735256	4.160780796	0.041370329	0.159710841
AKR1D1		-0.945917382	5.807107113	47.19676391	6.42E-12	1.34E-09
DPYSL5L		-0.254214157	5.807844314	2.74530939	0.097540196	0.273831189
LRRC40		0.154482837	5.808751964	6.870838669	0.008761353	0.05658087
C1QTNF3		0.153818115	5.809159604	0.573707131	0.44878955	0.672142719
NBEA		-0.185417277	5.809181716	6.640564305	0.009968235	0.061913716
SPOPL		-0.10937519	5.809635406	2.12454189	0.144956115	0.353842587
DYRK3		-0.079238132	5.809679995	1.028355128	0.310545314	0.548336682

COMMD10	0.215130351	5.809686136	7.617722574	0.005779743	0.042212905
SEC24B	-0.004297917	5.809854882	0.00466349	0.945554982	0.97671734
BRMS1L	0.055862279	5.810479203	0.996671988	0.318117132	0.555927931
MADD	-0.110971197	5.810960986	1.872092443	0.171235622	0.389828743
BNC1	0.218286624	5.811620294	5.429878634	0.019795055	0.098556014
NCSTN	-0.070138601	5.811935348	1.280086317	0.257882987	0.492541889
MAD2L1BP	0.01197845	5.81245647	0.019345385	0.889380858	0.950116515
DSN1	0.111289209	5.812894744	2.789180598	0.094902743	0.269803843
ABCA5	-0.034749635	5.813134452	0.15733489	0.691622167	0.839091293
ATP11A	0.059097164	5.813270393	0.755926858	0.384606216	0.619306711
TBC1D22A	-0.180475829	5.813322048	5.573607896	0.018233154	0.093320856
ARRDC3	0.248571908	5.813443915	3.896182001	0.048395973	0.175602147
ARL8A	0.022766776	5.813484691	0.092015111	0.761630822	0.88065738
SMIM19	-0.0947501	5.814492664	2.611629764	0.10608269	0.289868924
CASP2	0.156754231	5.81472844	5.736642301	0.016614525	0.088361992
GRB10	0.191914757	5.814805138	6.968087852	0.008297612	0.054593043
PI4K2B	0.007464158	5.814999401	0.015404368	0.901224747	0.954933316
ZNF622	-0.092882949	5.815077824	2.118933493	0.145487824	0.354656516
CTU2	-0.017418309	5.815597872	0.063886372	0.800455584	0.902713771
BRPF1	-0.116081707	5.816591461	2.868406775	0.090334255	0.262114296
MAPKBP1	-0.043919223	5.817024094	0.258770388	0.610966176	0.788815098
CCNH	0.208753352	5.81745851	5.458087769	0.019477951	0.09771631
NUDCD2	0.095874624	5.817542513	2.630350259	0.104838584	0.287471904
SELENOS	-0.261633841	5.817738214	10.85876591	0.000983292	0.011888369
TSHZ2	-0.162500142	5.817791879	1.693628775	0.193123307	0.417330711
MVK	-0.237874985	5.818107457	12.42975615	0.000422547	0.00637891
ASB9	-0.1726908	5.818118147	1.824600086	0.17676669	0.397085792
PKD2	-0.035027502	5.818304579	0.355827585	0.55083267	0.749396575
LSM3	-0.042209424	5.818703024	0.287719419	0.591685701	0.776938793
SLC37A3	0.058344628	5.818762626	0.930656791	0.334692316	0.572087272
L3MBTL2	0.046599213	5.818823436	0.507918678	0.476041235	0.693086049
TOLLIP	0.075081091	5.81916054	1.359615659	0.243604102	0.47663078

PRPSAP1	0.103524757	5.819430725	1.971307359	0.160309162	0.375840233
ABCA1	-0.475671062	5.819567084	6.594486014	0.010229508	0.063020053
MRPL35	0.059852907	5.819901449	0.505127898	0.477255592	0.693810475
DUSP14	0.077930079	5.819950273	1.187387704	0.275857121	0.510915379
CHRAC1	0.090863886	5.820008795	1.970411285	0.160404213	0.375883128
FBXL5	0.005451672	5.82008818	0.00844559	0.926777576	0.967217925
VAC14	-0.103200708	5.820206143	1.83380544	0.175678726	0.395349213
ZER1	-0.09858235	5.82023156	2.597386096	0.107040125	0.291188417
RFXAP	-0.01579626	5.821589214	0.065009982	0.798745845	0.901588892
PNO1	-0.004726292	5.821949981	0.003311803	0.954108457	0.980523198
MAML1	-0.25337388	5.822077795	10.09528965	0.001486488	0.01608163
CD99L2	0.067354938	5.822196121	0.547605926	0.459297565	0.681523442
CAPN5	0.164774004	5.822835946	2.12280167	0.145120864	0.354034665
B3GLCT	-0.046007052	5.822875324	0.531089425	0.466149203	0.686212661
LANCL1	0.089248996	5.822931545	2.071639928	0.150060089	0.361281572
CCDC15	0.12227176	5.823283672	3.3067282	0.068996734	0.221280185
RAB5B	0.032777534	5.823618671	0.301763961	0.582778683	0.770808073
NDUFA13	-0.048850121	5.823912475	0.336676809	0.561753741	0.756313026
PMS2	0.025716116	5.824554696	0.212797342	0.644583655	0.810532176
CC2D1B	-0.062076159	5.825842596	1.100859832	0.294077485	0.531081633
PPM1L	-0.152008661	5.82685759	2.788896672	0.094919561	0.269805039
YEATS4	-0.038542278	5.826891647	0.365686757	0.545364794	0.745408895
ORMDL3	0.022832707	5.82840018	0.144981961	0.703377655	0.846389149
POLR2L	0.046571279	5.828763146	0.36308647	0.546797066	0.746560382
VPS25	-0.023082994	5.829013176	0.149573678	0.6989431	0.843494384
RRN3	0.211906999	5.829225654	10.63808065	0.001107826	0.012919401
ISY1	-0.017023632	5.829238847	0.066629716	0.796308643	0.900629509
CDR2	0.026603391	5.829975592	0.210064894	0.646716545	0.811526992
KCNS2	-0.083254292	5.830461871	1.615175797	0.203765768	0.430597946
NDST3	0.018821023	5.830802993	0.079485939	0.777995263	0.889948343
CEP70	-0.059645948	5.831470817	0.813593195	0.36706005	0.602190739
LSM12	0.091109809	5.831489354	2.835271714	0.092215202	0.265492444

CHST10	-0.076228757	5.831605644	1.857251543	0.172942347	0.392303049
MAP2K4	-0.109165386	5.831959589	2.395034213	0.121721087	0.31581843
MRPS7	-0.023566655	5.832717804	0.12709093	0.721467538	0.856238611
DIS3L2	-0.071592644	5.832852104	1.65437053	0.198365121	0.4239639
TMEM161B	-0.094289119	5.833020842	1.340988156	0.24685978	0.480491801
B9D1	-0.031387647	5.833289862	0.289538133	0.590516658	0.77617634
UBE2F	0.13275341	5.833357193	4.168028529	0.041193706	0.15927139
PICK1	0.062033464	5.834101834	0.990030791	0.319734849	0.557135546
UBE2Q2	0.000333581	5.834179718	3.63E-05	0.995193127	0.99763501
KDM7A	-0.088101048	5.83620787	1.947868744	0.162816699	0.379345755
CLEC16A	0.061134436	5.836493954	0.60079644	0.43827431	0.663886934
CZH5ORF42	-0.036538517	5.836767105	0.115412134	0.734064677	0.864146105
LTV1	0.126086378	5.837120365	3.800872117	0.051225895	0.182450671
LOC101747302	2.024867193	5.837883537	119.0718108	1.01E-27	1.66E-24
KLHL25	0.043281891	5.838588318	0.1851694	0.666967869	0.82382197
SCYL3	-0.119244299	5.83883121	3.703708437	0.054291674	0.189634791
RHPN1	-0.002690771	5.83914712	0.001640154	0.967695448	0.986817198
MORN4	0.009284843	5.839570528	0.017862992	0.893677363	0.951501845
PRIM2	0.029961358	5.839754082	0.153463881	0.695246886	0.841135763
LOC419390	0.043533859	5.840182719	0.101638563	0.749872085	0.873444126
LOC100859302	0.099101172	5.840299106	1.0752619	0.299759925	0.537080745
UBA5	-0.045605412	5.840368884	0.282680923	0.594949405	0.77845837
RNF149	-0.257138632	5.840370473	15.77692043	7.13E-05	0.001597672
BORA	-0.154136218	5.840506529	3.108795347	0.077870536	0.238215389
C20H20ORF112	-0.086102758	5.840636517	0.846248911	0.357615673	0.59400061
ARL6IP4	-0.207742567	5.840679447	13.85734783	0.000197224	0.003535209
LOC416530	0.043203023	5.840780264	0.385339107	0.534759597	0.736480513
CC2D2A	-0.082518776	5.840883349	1.207868092	0.271754752	0.506602963
RNF32	0.368466861	5.841182101	26.8227966	2.23E-07	1.33E-05
C1H12ORF45	0.014336718	5.841455738	0.042953118	0.835813573	0.922298307
ZDHHC17	-0.023150734	5.841630126	0.125995432	0.722620792	0.856989199
RALGAPA2	0.027444068	5.841903465	0.175695108	0.675099201	0.828859984

SRBD1	0.170300535	5.842401776	4.642904833	0.031182179	0.132873572
EMC6	0.086216258	5.842404918	1.556904014	0.212119109	0.440017739
PARP16	-0.110707258	5.842571752	1.719560322	0.189749651	0.413170638
FAM172A	0.138548659	5.842730217	2.091503191	0.148120265	0.358220275
ARHGEF6	0.01481916	5.8428831	0.044294343	0.833306942	0.921086197
NDNF	-0.293411698	5.843080888	4.484790402	0.03419774	0.141139655
TYK2	-0.032546146	5.843126592	0.335504645	0.562435591	0.756921375
SPAG6	0.179751657	5.843151702	4.146549643	0.041719452	0.160433183
AMPD2	-0.028189935	5.843352194	0.180415832	0.671016124	0.826032043
CCNT1	-0.093287095	5.843721212	2.922568043	0.087348748	0.256233851
METTL9	0.02323633	5.843959572	0.098973393	0.753065098	0.874832943
RNF111	-0.036131848	5.844011608	0.276667569	0.598893776	0.781613119
HMGXB3	-0.139436321	5.844224254	4.628563764	0.031443875	0.133490445
UBE2D1	-0.036497913	5.844387762	0.284578468	0.593715909	0.77771018
ZIC1	-0.052408517	5.844440433	0.109758893	0.7404188	0.867900794
PRCP	0.022050056	5.845269458	0.068964346	0.792850702	0.898200227
UBA6	0.061073192	5.845367079	1.101627216	0.293909276	0.530933926
RAF1	-0.030675683	5.845391397	0.332964632	0.563918598	0.758108965
TJAP1	0.040240257	5.845591445	0.422739886	0.515573051	0.722206057
MIEF1	0.158855974	5.846867387	2.767317695	0.096207254	0.271821836
NPC1	-0.072376137	5.847045042	1.386754587	0.238953938	0.471800701
ASCC2	-0.455002938	5.847184047	53.11901925	3.14E-13	9.39E-11
COG6	0.210257361	5.847307429	10.82088459	0.001003615	0.012063177
COPS7A	0.120215208	5.847391114	3.15777116	0.075566288	0.234081941
GLIS2	0.164268328	5.847485616	3.793668548	0.051446775	0.182881114
RSRP1	0.030481646	5.847835133	0.007281142	0.931999355	0.970083458
MAN2A2	0.155577618	5.848282271	3.963201505	0.046505176	0.171195228
UQCRQ	0.025038344	5.848385067	0.160158087	0.689011012	0.83810791
MRPL10	-0.187331898	5.848431208	3.66357583	0.055614154	0.192335203
KIF16B	0.063751136	5.848880733	0.711922167	0.398806437	0.631178216
NAPRT	0.144908062	5.848966797	4.199717757	0.040430708	0.157539024
GPR162	-0.07248526	5.849200553	1.501235699	0.220481327	0.450014914

SH3RF1	-0.029700337	5.849404765	0.137108843	0.7111723	0.849781439
METRN	0.196461767	5.849744061	4.529165702	0.033321859	0.13895956
NAT8L	-0.010942935	5.84995334	0.011361732	0.91511312	0.961017703
TBCK	0.04055189	5.850009532	0.367414188	0.544417142	0.744408787
LOC423474	-0.068809208	5.850257832	0.247265312	0.619007272	0.794312827
PIGT	0.089285202	5.850714673	2.339731926	0.126111255	0.323233753
CEP164	0.117789113	5.85096485	3.168512868	0.075070772	0.233161487
ATG4B	-0.111242074	5.851042254	2.387974276	0.12227198	0.316820286
MPPED1	0.147265097	5.852307174	4.538800607	0.033134801	0.138454329
TGFBR1	-0.159884512	5.853113537	5.258206613	0.021843516	0.105159466
CDS2	-0.070849534	5.853252953	1.217770187	0.269798698	0.504595753
CIPC	-0.081754826	5.853931799	1.229427007	0.2675185	0.503030159
TIPIN	0.232565108	5.85431212	10.20105141	0.001403606	0.0153873
ARID4B	-0.029924741	5.8550701	0.151792051	0.696828659	0.842201708
ZCCHC11	-0.077721667	5.85537019	1.638414393	0.200543159	0.426900087
ADAMTS3	-0.417269997	5.855921196	24.57266768	7.16E-07	3.65E-05
UBE3C	-0.010656199	5.855947312	0.029168172	0.864391315	0.937302082
CNN2	0.004795101	5.856299839	0.004030158	0.949381475	0.978504123
TMEM47	0.11123699	5.856654886	1.943941088	0.163241255	0.379719374
FJX1	-0.056765128	5.85669493	0.239086051	0.624866998	0.798430381
R3HDM2	0.056115254	5.856860471	0.619091283	0.431385171	0.658562876
CAMK2G	0.102303481	5.857001986	2.860530337	0.090777565	0.262844418
DUS3L	-0.04614221	5.857016169	0.509022602	0.475562271	0.692572543
FAM122B	-0.000839737	5.857061666	0.000196496	0.988815864	0.994861819
MRPS23	0.036999209	5.857247314	0.411837613	0.521038276	0.726152692
ZNF639	-0.080393283	5.857521719	1.695885285	0.192826972	0.417197567
SLC12A9	-0.124993353	5.857661702	2.796009232	0.09449925	0.269205523
ABI2	-0.063232699	5.858232226	0.595183483	0.440421669	0.6651417
DPY19L3	-0.179150468	5.858696147	4.9788802	0.025658586	0.116956243
GORASP1	0.096103915	5.858891595	2.794219371	0.09460483	0.269329147
GCAT	0.119481038	5.859994978	1.414354371	0.234334886	0.466204878
ZBTB18	0.039992606	5.860286868	0.203068937	0.652255009	0.814579419

JTB	-0.330521329	5.860618924	6.513232803	0.010707467	0.065013228
DCTN5	0.142067878	5.861102919	5.625758451	0.017698406	0.091725124
VPS51	0.069925474	5.862228978	1.034834632	0.30902585	0.546988236
AAR2	0.049426678	5.862327285	0.703011202	0.401773733	0.634166597
LOC107051323	0.171955654	5.862339341	5.89899459	0.01514953	0.08262488
EIF2B4	0.285440649	5.862535574	12.80308755	0.000346048	0.005475208
SPINT1	-0.229502813	5.862553441	9.362229966	0.002215025	0.021579772
SMURF2	-0.151793544	5.862952146	4.392661034	0.036093937	0.146157165
TMTC2	0.20149342	5.863053931	9.232149128	0.002378024	0.022564277
SOX13	-0.037318406	5.863247533	0.131844316	0.716527401	0.853144601
PLD1	0.095931069	5.86328709	1.609361324	0.204581603	0.431327134
LOC112531692	-0.110066434	5.863467778	3.283335533	0.069986558	0.22331371
FASTKD2	0.052520084	5.863637028	0.478337336	0.489176402	0.70269731
EIF2AK1	0.045024215	5.863846788	0.497060572	0.480794537	0.696677888
KIFC3	-0.168565679	5.863964265	2.670222038	0.102241759	0.282422048
PIGV	-0.046281335	5.864309665	0.392703537	0.53088172	0.733558252
ATP6AP2	-0.103878996	5.864478289	1.835738563	0.175451236	0.395161523
INPP5E	0.027785409	5.865073568	0.211989163	0.645212766	0.81068921
LPCAT3	0.010245269	5.865830832	0.039110865	0.843229188	0.926094935
CSTF1	-0.069737802	5.866633712	0.980045355	0.322187609	0.559831607
CAPN10	0.238807682	5.86666143	11.7324727	0.000614189	0.008443179
PIK3R4	0.043949011	5.867360033	0.465221759	0.495193635	0.707178212
NT5C3B	0.138136003	5.867460243	3.775042197	0.052022589	0.184251334
TBC1D15	0.022609584	5.867561154	0.126216676	0.72238743	0.856852067
SEC62	0.072417602	5.868192226	1.592201077	0.207011943	0.434352468
LOC101750687	0.325052885	5.868463628	17.98885555	2.22E-05	0.00061451
CWF19L1	-0.127607062	5.868855966	3.486461226	0.061872715	0.206934049
RARB	-0.037780272	5.869307691	0.133402225	0.714930251	0.852353085
FAM117B	-0.02546649	5.869469714	0.138299449	0.709977477	0.849641566
MSL3	-0.135701613	5.870012543	5.370488807	0.020480237	0.100778202
NSMCE3	0.038682814	5.870114067	0.159577266	0.689546016	0.83825922
MRPL48	0.00354167	5.870448601	0.002614429	0.959220749	0.982445999

PANK1	0.028203164	5.870539207	0.170376693	0.679777245	0.832086184
LOC395159	-0.101552648	5.870725608	0.431914614	0.511050969	0.718808761
SLC45A4	0.015217745	5.871324509	0.025749512	0.872513573	0.94163263
CLN8	-0.004924013	5.87141807	0.00556364	0.94054108	0.974411302
TUSC5	0.292608853	5.871508523	8.823217753	0.002974217	0.026269859
HERC4	0.002458022	5.87188955	0.001712685	0.966989293	0.986411805
UTP11	-0.01505151	5.872830839	0.030777541	0.860737612	0.93500379
ATP6V1E1	-0.168969638	5.872992735	7.509461875	0.006137571	0.044214145
PPP1R15B	0.046125897	5.873892176	0.450362326	0.502162942	0.71305564
SETD3	-0.03468391	5.874798334	0.39185799	0.531324377	0.733850867
MICALL1	-0.078552441	5.875037218	1.319389093	0.250701719	0.48498669
PEX5	0.078882759	5.875194734	2.093870414	0.14789098	0.357979712
VEZT	-0.092940777	5.875397548	2.258868466	0.1328511	0.334670064
KIF13A	0.011897936	5.875928271	0.027027165	0.869416731	0.939840514
KCTD15	0.06830015	5.876107929	0.852254564	0.355915314	0.59235223
MRPS33	-0.081114768	5.876151744	1.493092806	0.221737213	0.451179047
IFT27	-0.142516114	5.876363544	4.921814943	0.026519644	0.119720367
THAP5	-0.003951568	5.876503121	0.00381229	0.950766906	0.979334551
PSEN2	0.070018679	5.87661854	1.505912677	0.219763837	0.448996018
CHMP2B	-0.120582015	5.876688861	3.251541048	0.07135634	0.225714836
RASA2	-0.118204971	5.876921349	2.382045932	0.12273671	0.317489629
CBR1	0.176647034	5.877044848	7.416160702	0.00646406	0.045954099
ARL4A	-0.055206249	5.877363409	0.372179867	0.541818445	0.742288112
NKX3-2	0.418415415	5.877456877	13.26350607	0.000270623	0.004583313
WNT4	0.227976926	5.877477754	2.863141379	0.090630346	0.262637775
TAF7	0.145112972	5.877759398	6.095662379	0.013551412	0.076843124
GLT8D1	0.160640868	5.877796873	8.062346248	0.004519462	0.035514684
LOC107057363	0.223532653	5.877801505	4.371212045	0.036550985	0.147261539
LRRC59	0.015030043	5.877924506	0.053320375	0.817383129	0.913725502
PPP3CA	-0.097048267	5.878063349	1.824011613	0.176836505	0.397085792
SETDB2	0.094458408	5.878239373	3.208945391	0.073236674	0.229108265
GMNN	0.337150548	5.878679686	17.32179107	3.16E-05	0.000816405

HMGA2	0.127699469	5.878686654	2.146928508	0.142855427	0.350587031
PARD6G	0.066307271	5.87873755	1.093844698	0.295620915	0.532447697
SMIM15	-0.067808944	5.878976341	0.694614743	0.404599157	0.636032596
C11H16orf70	-0.026187887	5.879165895	0.207648036	0.648617128	0.812444812
OSBPL11	0.015454721	5.880236408	0.074760378	0.784527736	0.894188813
RPA3	0.243185637	5.880402402	12.04083083	0.000520478	0.007473351
ZNF654	-0.03681896	5.880564118	0.280106189	0.596631625	0.779853315
RSG1	0.123212432	5.88095075	2.636226501	0.104451371	0.286823373
LOC107051537	0.132945459	5.881103701	3.246150823	0.071591398	0.226197475
PAK1IP1	0.114173928	5.88128611	2.922485276	0.087353228	0.256233851
TRIOBP	-0.093425546	5.881359346	1.854691863	0.173238688	0.392542358
CDC42EP4	-0.031415748	5.882375933	0.168398544	0.681539012	0.83319824
MSL2	-0.085453784	5.882844871	1.29382239	0.255344726	0.490348661
INTS10	0.028725517	5.883259942	0.173980909	0.676597813	0.830023971
TOP3A	0.406134307	5.88343129	12.3234403	0.000447306	0.00666704
DECR1	-0.056459768	5.883561024	0.367841127	0.544183397	0.744387837
KPNA6	0.069590417	5.88390431	1.063656929	0.302382604	0.540191699
TMEM237	-0.078987991	5.883972612	0.54056859	0.46219718	0.683472595
MRPL13	-0.017961956	5.884472112	0.073804505	0.785875889	0.894610014
FCRL4	-0.176346241	5.884668282	3.753030089	0.052711873	0.1859721
CHN1	0.019672139	5.884866705	0.080283445	0.776913662	0.889536847
CDK5RAP1	-0.011339056	5.885389465	0.031269701	0.85964003	0.934304934
NDUFB7	0.1070502	5.88546868	0.760680542	0.383115633	0.61781338
CCND2	-0.069384673	5.886218057	1.009426226	0.315040337	0.552546231
MTIF2	0.116808571	5.886218849	3.380234228	0.065982677	0.215420225
RBP14L	0.12383062	5.886228666	2.369914168	0.123693852	0.318775621
USP33	-0.006215602	5.886530209	0.0116769	0.913948329	0.960591451
PPA2	0.1958818	5.88665376	4.703598538	0.030099533	0.129589207
SPOP	-0.008680419	5.887108238	0.024997524	0.87437323	0.942721236
TBC1D2B	0.142058504	5.887473331	5.620177426	0.017754851	0.091921032
ZNF451	0.015695731	5.887687171	0.065865926	0.797453931	0.901181542
GCOM1	-0.057891761	5.888280091	0.800343282	0.370990754	0.605499391

SENP1	-0.046174922	5.88857259	0.438455643	0.507868794	0.717152751
AKAP11	-0.074498155	5.888902231	1.055387438	0.304269566	0.54215005
TTC17	-0.234314795	5.889022576	12.0218087	0.000525817	0.007530297
KIF18A	-0.007497472	5.890089778	0.010468627	0.918505613	0.962372833
LRIG2	-0.13805537	5.890405682	3.284053161	0.069955967	0.223259395
DAP	-0.291261323	5.891044106	18.82220602	1.43E-05	0.000434021
WDR24	0.363403383	5.891050633	24.10637206	9.12E-07	4.45E-05
PIGK	-0.067894395	5.891258715	0.415709812	0.519085565	0.7245973
GPHN	0.213740629	5.891267024	10.43515054	0.001236395	0.013976095
RIN2	-0.017203197	5.891346026	0.090260134	0.76384671	0.881608866
AHSA2	0.171672024	5.891352831	6.91168035	0.008563448	0.055740321
SEC22A	0.073262744	5.891906959	1.430285212	0.231717897	0.46346396
SLAINL	-0.032942131	5.892008555	0.186685798	0.665689434	0.823154425
PHACTR4	0.073261095	5.892229263	1.177643239	0.277836308	0.513166062
GTF3C2	0.201592679	5.892329965	10.72955255	0.001054379	0.012501579
RPS6KA6	-0.153316485	5.893191838	6.093755313	0.013566045	0.076843124
CTSB	-0.303093774	5.893608147	14.05885858	0.000177177	0.003239391
LANCL2	0.063531825	5.893631339	1.223414241	0.268691645	0.503682048
NFKBIB	-0.00206112	5.894262638	0.000742221	0.978265341	0.990904465
TMEM209	-0.1799056	5.894848308	8.433413293	0.003683882	0.030739491
MID1IP1	-0.009468209	5.895338229	0.015236357	0.901762136	0.955177611
STX12	-0.029511538	5.895391315	0.226399435	0.634206541	0.80403489
CENPQ	0.961491513	5.895739526	180.1378513	4.52E-41	2.48E-37
BMP7	-0.225764395	5.896210149	10.04605902	0.001526742	0.016366476
SEMA5A	-0.784896741	5.896589604	17.7691673	2.49E-05	0.000674855
PDK3	0.054971817	5.896600787	0.733517814	0.391745299	0.625295266
PPIL4	-0.02315335	5.896757216	0.143355759	0.704967465	0.847186196
MRPL37	0.135008046	5.89744839	4.736662624	0.029526296	0.127899143
TXNDC17	0.018153352	5.897497954	0.053755246	0.81665314	0.9133936
LOC107050762	0.086696785	5.897695703	2.052824658	0.151924098	0.363729234
STAGL	-0.333007149	5.898564313	20.44559788	6.14E-06	0.000219524
LOC107051531	0.308225502	5.898694978	18.5043184	1.70E-05	0.000491966

WIPI2	-0.112254618	5.899153134	4.307184468	0.037951725	0.15049741
GMPPB	0.125170838	5.899203152	2.059311755	0.151278484	0.362972935
RBM34	-0.057350498	5.899302246	0.595862189	0.440161158	0.665015477
LOC107057188	-1.318872589	5.899385956	48.91066416	2.68E-12	6.12E-10
RUNX1T1	-0.087257644	5.899694434	1.539243659	0.21473042	0.44383734
CD99	0.025089093	5.90007435	0.101125265	0.750483428	0.873723277
RUFY1	0.064991035	5.900627909	0.905873557	0.341211661	0.578292088
RAC3	-0.078311703	5.900664759	1.220635411	0.269235988	0.504027293
HDHD3	0.095635669	5.901041601	1.34185745	0.246706669	0.48030741
TK1	0.078807327	5.901056913	0.785667779	0.375413317	0.610055909
SPICE1	0.236121426	5.90206576	15.46306876	8.41E-05	0.001814074
SNRPD1	0.045824688	5.902524965	0.359799806	0.548617439	0.747742894
SEPW1	0.206760876	5.903807128	7.093061397	0.007738295	0.051960495
MIOS	-0.302022359	5.904478843	13.15621045	0.000286568	0.004758299
UBN1	-0.104164517	5.904616368	2.859043885	0.090861491	0.262937133
NGLY1	-0.085644636	5.904975557	1.99802129	0.157504704	0.372055685
DNAJB4	0.108168992	5.905299262	2.202937549	0.137748016	0.34234158
RAB12	0.065525684	5.905668182	0.763500978	0.382235116	0.616769322
LUZP1	-0.145754497	5.906064569	5.015189841	0.025125874	0.115487782
ACOT9	0.434181318	5.906913897	39.31191809	3.61E-10	4.54E-08
HIKESHI	0.007064508	5.907094995	0.014079412	0.905547345	0.956882345
REXO5	-0.07693433	5.907396703	1.119397191	0.290048291	0.526909322
LRBA	-0.24243251	5.907499475	5.679732984	0.017161979	0.090137367
TRAPPC9	-0.175063811	5.907651497	6.959962162	0.00833538	0.05475396
PCYT1A	0.021532033	5.907928023	0.148858277	0.699628826	0.843779396
FKBP8	0.190967575	5.908289725	7.04743022	0.007937905	0.052881874
RPF1	0.19206104	5.909037042	7.446310572	0.006356662	0.045379117
SPOCK1	-0.169055732	5.909373782	1.758515956	0.18480973	0.407359715
MED29	0.245711261	5.91067387	6.747921424	0.009385696	0.059263866
COL8A2	-0.040228195	5.911238541	0.220165503	0.638913842	0.806855508
SPATS2L	0.115966964	5.911256507	2.161738407	0.141484543	0.348470012
KIF18B	0.202595933	5.911592596	11.84269789	0.000578879	0.008086123

TET1	-0.184480504	5.911667101	1.56201377	0.211370609	0.438932783
DMD	0.10794702	5.911986349	2.104633795	0.146853497	0.356412138
HDAC10	0.186391439	5.912402449	4.092326789	0.043078301	0.163594146
IGF1R	0.02826418	5.912577879	0.031713058	0.858658889	0.933917114
ESF1	0.002131002	5.912615041	0.001173285	0.972675202	0.989145939
ILDR2	-0.205314071	5.912721402	6.688196236	0.009705335	0.060800697
MESDC1	0.00177145	5.912944952	0.000952017	0.975385362	0.990018742
ANKRD52	-0.383217647	5.912973235	18.53114892	1.67E-05	0.000488536
HIF1AN	-0.126438127	5.913297566	4.017272039	0.045036504	0.167778055
TA3	0.115249984	5.9137731	2.853045155	0.091201042	0.263653048
WIPI1	0.094722725	5.913853426	2.062854989	0.150927163	0.362396975
HSF3	-0.062919046	5.914100224	1.089182812	0.296652342	0.533680327
PRDM16	-0.1884724	5.914521448	1.082622214	0.298111689	0.534894621
IFT20	0.092320973	5.915084023	1.246102346	0.264298216	0.499486292
RAB23	-0.100448949	5.915125381	2.006275306	0.156649505	0.370940798
PSMD5	0.02305632	5.915516378	0.127420705	0.721121474	0.856013119
MTFR2	0.134682397	5.915814787	3.177741102	0.07464786	0.232444675
QSOX1	-0.162990618	5.915929087	5.787265557	0.016142679	0.086664858
ACADS	-0.109086778	5.916344768	1.614912201	0.20380267	0.430597946
STIM2	0.091335225	5.916785806	2.770472136	0.096017836	0.271613116
SLC25A4	0.267729301	5.917562794	10.58524546	0.001139938	0.013218938
MIA2	-0.06837985	5.917797922	0.845243238	0.357901495	0.594215427
ANKLE2	0.028757426	5.918825023	0.215055534	0.642833459	0.809259013
KIAA1522	-0.078997164	5.919156648	1.322683287	0.250111052	0.484356521
FANCM	0.016738217	5.919199897	0.082880185	0.773431563	0.887781347
CLDND1	0.050244903	5.919778061	0.878145694	0.348710062	0.585333477
TEAD3	-0.15150216	5.920053849	6.81812593	0.009023721	0.057709029
ACSL1	-0.198668509	5.920311894	7.669613013	0.00561585	0.041420355
RPIA	-0.072465493	5.920421034	1.661377192	0.197417481	0.423092556
SPCS1	0.080407559	5.920544851	1.752300013	0.18558785	0.4081865
NKRF	0.00452036	5.920623267	0.003075108	0.955777052	0.981041173
SGO1	0.067787733	5.921576054	0.818407652	0.365646143	0.600949588

NUDCD3	0.138702225	5.923058091	4.212550542	0.04012596	0.156957222
FEZ2	0.046972967	5.923274424	0.579086981	0.446670418	0.670621395
NPHS2	-2.71261099	5.92358938	19.79444519	8.62E-06	0.000290771
PISD	-0.201927427	5.924153322	7.835552684	0.005122849	0.038828409
VPS4B	-0.194361369	5.925370084	8.623318313	0.003318867	0.028443727
TLE1	0.244910661	5.92588502	8.543192302	0.003468169	0.029296054
TF	0.596495082	5.926126629	4.894421871	0.026943593	0.120970485
MRPL24	0.004075424	5.926858057	0.002221844	0.962404484	0.983899168
ANKRD28	-0.056528227	5.926880234	0.805890685	0.369337967	0.604000819
SUFU	0.171713777	5.92753777	7.374871847	0.00661415	0.04653862
GOPC	-0.076957522	5.927558678	1.659609178	0.1976561	0.42338338
CENPI	-0.201390149	5.927945245	10.91425008	0.000954273	0.011605742
MAZ	0.151071813	5.928031453	6.065243831	0.013786772	0.077865936
FN3KRP	0.196564912	5.928233583	4.8464946	0.027702376	0.123267332
BLOC1S3	-0.277535643	5.928257965	8.592033708	0.003376367	0.028756793
GRK2	-0.062747875	5.928325174	1.162421868	0.280963832	0.51662307
FAM208B	-0.16212695	5.928365011	4.235671605	0.03958296	0.155487612
GALE	0.018942761	5.92854952	0.085499105	0.769979051	0.88527147
SUCLA2	0.094565001	5.92937789	2.559465591	0.109635638	0.295670298
ISYNA1	0.013802953	5.929714068	0.027789675	0.867604253	0.938868077
RHOT1	0.177727608	5.929997147	10.34640814	0.001297269	0.014472241
MRPL2	0.049106194	5.930107735	0.325905586	0.568080032	0.760970118
PLEKHA5	0.132903752	5.930898783	4.370872525	0.036558268	0.147261539
FUNDC1	-0.087163144	5.93119625	1.951163235	0.162461556	0.378860143
RPS5	0.209353148	5.932363855	4.58287002	0.032293084	0.136033958
GOLGA1	-0.118307707	5.932617606	2.169128129	0.140806045	0.347551822
DISP3	0.076911038	5.932817435	0.606463848	0.436122349	0.662345819
GALNT7	0.084697547	5.933502675	1.520620152	0.21752567	0.446474354
EFNA5	0.214974093	5.934385802	4.012960838	0.045151793	0.168055361
FUK	0.187468574	5.934771936	6.932693964	0.008463411	0.055285999
ATRNL1	0.088205832	5.935627243	1.378354324	0.240381665	0.473030411
MTX2	0.082536166	5.936426207	1.874130999	0.1710027	0.389793886

DDRGK1	-0.023962579	5.936664873	0.139186025	0.709091558	0.849542309
ITGA3	0.004739604	5.937046897	0.003228476	0.954688836	0.980541221
IMP4	-0.030698796	5.937077013	0.105873495	0.74489192	0.87060136
MAPK8	-0.011426299	5.937314985	0.036438157	0.848613489	0.929382324
TOR1A	0.038541331	5.937494608	0.470423025	0.492792622	0.705297727
NSG1	0.011162461	5.937717174	0.014477038	0.904229214	0.956301286
TSKU	-0.023299798	5.937889356	0.063033768	0.801763649	0.903384062
BET1L	-0.049282788	5.937911247	0.696033987	0.40411955	0.635646128
PRKAR1B	0.114505831	5.93797099	3.200725454	0.073605614	0.229825497
NAA35	0.239061978	5.938297445	6.803145606	0.009099741	0.058014817
LSM4	0.041108846	5.938423978	0.431712019	0.51115008	0.71887419
HCFC2	-0.127419415	5.938449243	1.863122795	0.172264817	0.391322595
LOC112531189	-0.02335061	5.938757753	0.05069419	0.821859704	0.916364103
DHX57	0.091941261	5.93892451	2.735908433	0.098115694	0.274842017
NR1H3	0.007961929	5.939407457	0.015971806	0.899431442	0.954232391
TMEM55A	-0.180516793	5.939477827	5.403537517	0.02009598	0.099346821
RLF	-0.054359073	5.93949195	0.60173195	0.437917971	0.663807274
FLT4	-0.015195842	5.940104872	0.031355544	0.859449504	0.934196556
VPS13D	0.044574256	5.940191293	0.289872698	0.59030212	0.776026179
SORT1	0.072991851	5.940298138	0.841209781	0.359051002	0.595343031
THADA	-0.094864694	5.940686926	1.480373873	0.223716036	0.453072564
GTF2A2	0.000712313	5.940866081	0.00011418	0.991474377	0.996014095
NXT2	-0.040371602	5.941199521	0.328699774	0.566425713	0.759991448
NBAS	-0.013913171	5.941371811	0.03532889	0.850908158	0.930592432
BRD9	0.142247139	5.941462865	5.777518999	0.016232438	0.086959286
RNF38	0.109445137	5.941571433	1.631564482	0.201486795	0.428133421
MED22	0.048129086	5.942194638	0.589824506	0.442486989	0.667258377
COL9A3	-0.115686515	5.942238212	0.665982636	0.414455759	0.644904919
PRKCA	0.012238133	5.943082116	0.017449346	0.894908382	0.951827123
DTYMK	-0.056269725	5.943632903	0.925828487	0.335949241	0.57321078
EBF3	-0.213967853	5.943724066	2.681071678	0.10154737	0.281117424
IGBP1	0.012143793	5.94405801	0.042282218	0.83708275	0.923141657

BSDC1	-0.115198031	5.944484822	4.573832038	0.032463869	0.136484247
LOC420374	-0.061806289	5.944913229	0.620708084	0.430784279	0.658237099
ALS2	-0.101413155	5.944961603	2.708935269	0.099787573	0.277553164
OLFML2A	-0.073706688	5.944966068	1.095677214	0.295216736	0.532185736
PTP4A3	0.147863452	5.94517645	4.63326699	0.031357799	0.133294601
SCFD2	-0.19316095	5.945947786	7.924565001	0.004876807	0.037575148
BRCA2	0.067415496	5.946522834	1.310030155	0.252389196	0.486934484
MRPL17	0.138559317	5.946697619	1.289421675	0.256154556	0.490864503
TDP1	0.002924305	5.946960191	0.001957542	0.964709812	0.985185872
CDC40	0.068903438	5.947165115	1.527844619	0.216436217	0.445460658
WDR18	0.016354648	5.94738039	0.069474596	0.792103309	0.897745396
STUB1	-0.004978123	5.947760878	0.008455913	0.926732969	0.967217925
TBP	-0.01881067	5.947817979	0.062431586	0.802693193	0.90424567
AGO3	-0.11520054	5.948189882	1.006805776	0.315669294	0.553295509
KMT5B	0.02389108	5.948221817	0.18664104	0.665727079	0.823154425
EDRF1	-0.062428719	5.948538043	0.994430021	0.318662044	0.556348428
GSS	0.067193249	5.949266664	0.961951187	0.326695767	0.564326983
CAMTA1	0.116975576	5.949418836	4.114249512	0.042523388	0.162368316
ZC2HC1C	0.113756762	5.950104177	3.706676062	0.054195216	0.189458737
RNF11	-0.003927764	5.950372196	0.003694401	0.951533154	0.979652938
C9ORF58	-0.006167031	5.95134002	0.004882492	0.944293287	0.976060582
ERBB2	0.161141665	5.951438625	5.508594485	0.018923248	0.095662686
ITCH	0.031407041	5.95151636	0.214434684	0.643313528	0.809491788
SMC6	0.250201977	5.951756094	14.54530381	0.000136829	0.002633363
FANCL	0.011958479	5.953226661	0.025050323	0.874241732	0.942721236
CCDC25	0.079386199	5.953320085	1.788473797	0.181112226	0.403274923
TRIM24	0.125564682	5.953399952	2.109973054	0.146341894	0.35556918
PLCD1	-0.294717732	5.95434904	9.140718386	0.002499829	0.023239939
CHFR	-0.147869466	5.954664054	6.800906423	0.009111161	0.058063544
RNF6	-0.142023414	5.955596442	2.870590847	0.090211746	0.262036199
AP5Z1	0.023966314	5.955633679	0.130828706	0.717574351	0.853896872
LRRFIP2	-0.110078912	5.955638735	4.324813372	0.037560533	0.149614536

PMM2	0.068937094	5.955997691	1.049217359	0.305687405	0.54382671
GOSR2	0.058301613	5.95657109	1.010874058	0.314693533	0.552291178
SCHIP1	0.20019119	5.957428177	8.750457135	0.003095245	0.027019761
PIP5K1C	0.098529127	5.957833943	2.513856193	0.112849488	0.301157691
PDPR	-0.320311396	5.958137571	19.84939535	8.38E-06	0.000284279
HBS1L	0.105418339	5.958268605	3.589926965	0.058130798	0.1982882
TRAPPC10	-0.112424286	5.958443063	3.301936894	0.069198244	0.221567349
WDR70	0.361532767	5.958547342	13.62900019	0.000222718	0.00390291
LOC107049075	0.435632844	5.958553132	17.52910254	2.83E-05	0.000744933
CIC	0.21424801	5.958791264	3.939737067	0.047158148	0.172864184
WDR61	0.001729019	5.958799128	0.000610583	0.980286291	0.991676559
LOC419404	0.024224305	5.959345276	0.208387209	0.64803444	0.812187803
ANTXRL	0.0432192	5.960686857	0.286683861	0.592353469	0.77703893
PLCE1	0.009086743	5.960726833	0.007602089	0.93052052	0.969586826
BORCS7	-0.08329576	5.960898223	1.112311167	0.291580103	0.528791006
CCDC43	-0.102538335	5.961064312	2.326778606	0.127164797	0.325073284
SRC	-0.123385582	5.961164297	2.307878748	0.128719606	0.327927097
STAT1	-0.006356991	5.961298092	0.009893518	0.920768151	0.963742606
YIPF3	-0.203521672	5.961393788	13.16007252	0.000285978	0.004757961
GPATCH4	-0.192099091	5.961521849	7.202851294	0.007278784	0.049595195
GTF2B	-0.164765369	5.961594337	4.119661908	0.042387549	0.162100519
SAMD11	-0.008661163	5.96165725	0.006131698	0.937585313	0.972943578
NTMT1	0.243410126	5.961741294	9.908442399	0.001645223	0.017331719
ZNF106	0.011438356	5.962145793	0.01465536	0.903644046	0.955902743
CHMP2A	-0.083241186	5.962883376	2.154949141	0.142111149	0.349381997
DPYD	-0.148300789	5.963277778	3.646774514	0.056177908	0.193552656
LONP2	0.18097409	5.963570736	6.127482061	0.013309629	0.076018726
ETS2	-0.051192326	5.963698145	0.352256366	0.552838627	0.751194021
GTF3C6	-0.067067783	5.964048599	0.853136423	0.35566657	0.592237747
PDK1	0.013855444	5.964470215	0.050281235	0.822574625	0.916456624
AXIN1	0.143405981	5.964604038	3.910815929	0.047976299	0.174695731
COQ7	-0.134637066	5.964627807	4.010279018	0.045223666	0.168242723

GNAQ	0.148506025	5.964767061	4.051994419	0.044119192	0.165887453
PPP1R8	-0.021235931	5.965499877	0.128407433	0.720089015	0.855589916
FGFRL1	0.066785305	5.965510545	0.433665682	0.510195714	0.718341124
GNPDA1	0.122238594	5.965744151	3.30401325	0.069110841	0.221464242
TRAPPC2	0.341863222	5.966546845	25.15804581	5.28E-07	2.82E-05
ACAP3	-0.088530314	5.966674396	0.725995467	0.394184301	0.627362673
TMEM132E	0.123710529	5.96668245	0.939060693	0.332519525	0.570137415
SETD6	-0.095853556	5.96677709	1.623526745	0.202600734	0.429233617
LOC420992	-0.21848353	5.967424871	11.25036968	0.000796072	0.010162419
ZNF648	-0.03618142	5.967484637	0.308102958	0.578846626	0.768285864
DIEXF	0.09998266	5.96759016	3.207966079	0.073280525	0.229158311
SLC45A1	-0.188285753	5.967701185	5.248615813	0.021964237	0.105462945
SLC6A15	-0.091350428	5.967715875	1.770861015	0.183275558	0.40570577
HEATR5A	0.081687099	5.968389199	1.147848308	0.284000027	0.520055692
TBCC	0.012489967	5.968480116	0.032702528	0.856494419	0.933075655
SNX27	0.026617008	5.968543512	0.179711604	0.671621201	0.826344165
ARL15	0.190755984	5.96860566	6.183631819	0.012893736	0.074256743
KLHL7	-0.026920821	5.968684578	0.235617829	0.627389222	0.799999777
MTA3	-0.032202189	5.96888205	0.260429904	0.609824959	0.787717486
KIAA1671	-0.160876762	5.969477443	2.830714075	0.092477232	0.265821665
E2F8	-0.002010502	5.970348079	0.000532719	0.981585897	0.991828264
MRPS9	0.132039402	5.970363351	4.392474792	0.03609788	0.146157165
RGMB	-0.022666235	5.971715386	0.029701997	0.863168138	0.93634595
LOC107050768	-0.864408256	5.972316841	17.01735417	3.70E-05	0.000935443
FOXC2	0.102270992	5.972770964	0.757674966	0.384057117	0.618725265
INTS6	-0.128314886	5.972951121	4.227033013	0.03978493	0.156094666
UGGT2	-0.051988342	5.973044748	0.437406316	0.50837698	0.717589112
TXNDC9	0.055775543	5.97334	0.778861023	0.377489706	0.612341592
C2H6orf52	0.133620811	5.973353842	4.610137298	0.031783488	0.134578679
APOO	0.108002944	5.97345869	2.196416669	0.138331976	0.343481616
SCD	-0.046006897	5.974090342	0.205787901	0.650089029	0.81335272
ADGRD2	0.148140028	5.974290459	1.694008543	0.193073397	0.417328557

MRI1	0.109325842	5.974727687	1.894121339	0.168737807	0.387094976
CEP250	0.023742878	5.974728634	0.102669423	0.748649438	0.873168892
LOC423967	-0.030081171	5.974925457	0.263872382	0.607472171	0.787083037
RTEL1	0.286803988	5.975059424	15.19490399	9.70E-05	0.00201969
MFSD14A	0.003127767	5.975423352	0.002218807	0.96243017	0.983899168
SESN1	-0.104854376	5.975886793	2.266749739	0.132176851	0.33363554
DDX20	0.04672428	5.97640835	0.37924964	0.53800509	0.739217915
E2F7	0.114775393	5.97673066	2.195586509	0.138406518	0.343563019
COPZ1	0.005800945	5.97706365	0.008551643	0.92632058	0.967109012
RNF220	0.065311822	5.978641052	1.308121668	0.252735019	0.487280718
SDC3	-0.089354316	5.978874056	1.186237526	0.276089808	0.511145116
DUSP6	-0.01923559	5.981100028	0.013943736	0.906001421	0.957006893
MXRA7	-0.135795607	5.981419662	2.729593805	0.098504335	0.275473969
LOC107050199	0.084995544	5.981459646	0.75736836	0.384153345	0.61879414
PSEN1	-0.442703514	5.981549327	41.92519909	9.48E-11	1.44E-08
NSL1	-0.028878841	5.981903139	0.245561219	0.620218049	0.795058816
EPHB6	0.062992228	5.981916774	0.955911383	0.328219156	0.565717803
MAD1L1	0.164427719	5.982027811	3.247138728	0.071548255	0.226191458
NCK2	0.042233229	5.98354602	0.535719181	0.464212272	0.685261768
PHKA1	-0.116298063	5.98446985	1.979148848	0.159480103	0.374517638
PLEKHB2	-0.083195844	5.984611311	2.229593329	0.135389479	0.33839038
CDC42SE1	0.012738495	5.986488425	0.041375074	0.838815649	0.923690792
WDR45B	-0.027858184	5.987275786	0.218534887	0.640158535	0.807491962
TMEM168	-0.026444259	5.987615954	0.180063418	0.671318745	0.826342756
TMX4	-0.281227991	5.98787899	12.8237185	0.000342253	0.005441324
ASNS	0.098558107	5.987922719	1.349219037	0.245414692	0.47898218
PFKFB3	-0.051958098	5.987975813	0.286062481	0.592754905	0.777327412
ANKRD10	-0.087573792	5.9882192	1.897651148	0.168341466	0.386609745
MRPL22	0.099432273	5.988700767	2.415487611	0.120140555	0.313099911
WDR20	-0.087952057	5.98895236	1.389132433	0.238551665	0.471215677
EVI5L	0.023232709	5.989634805	0.08540936	0.770096404	0.885344534
DCPS	0.201712697	5.990749644	10.32511839	0.001312318	0.014600541

RIC1	-0.192553028	5.99118606	4.765523523	0.029035228	0.126697076
SELENOH	0.012038817	5.991287265	0.013138522	0.908743638	0.95829261
ARF3	0.067134663	5.991957978	1.206242334	0.272077597	0.506853488
PIP5K1A	-0.015914337	5.992197208	0.061826984	0.803631278	0.905054594
DSCC1	-0.121361375	5.99295569	2.782596684	0.095293553	0.270386182
PPP2R3A	-0.031122248	5.992957015	0.268233837	0.604518998	0.785137502
KIAA0319L	-0.049640221	5.993011942	0.7751279	0.378635355	0.613278882
RNGTT	0.214893277	5.993272423	8.7467254	0.003101585	0.027043327
TDRD3	0.010343842	5.993446457	0.022012872	0.882052862	0.947277107
TMEM132D	0.032286555	5.994853915	0.201384899	0.65360531	0.815062002
CHST6	-0.088059791	5.994895932	1.618270045	0.203333174	0.430265966
DCUN1D5	-0.114947496	5.995447982	3.658408285	0.055786905	0.19262599
DCAF15	0.141035606	5.997313932	4.009382816	0.045247711	0.168242723
SNX2	0.130700949	5.997533401	2.709582125	0.099747117	0.277547145
UBASH3B	-0.081632613	5.997592283	1.601116335	0.205745081	0.43265627
PKNOX2	-0.000474466	5.997633604	6.37E-05	0.9936308	0.99720632
TXNRD1	0.055262279	5.997938057	0.715409403	0.397653849	0.630081279
ARID2	-0.007346057	5.999152035	0.011190167	0.91575406	0.961568059
MID1	-0.10476911	5.999257718	1.499405515	0.220762856	0.450182768
SMARCD3	0.169725693	5.999570262	6.859947669	0.008814913	0.056793028
N4BP2	0.330172103	5.999592023	15.93590742	6.55E-05	0.001493344
WT1	0.082517329	6.000188965	0.61533728	0.432785278	0.659478488
SLC29A4	0.030134854	6.000568101	0.203682325	0.651764854	0.814211257
RBMX2	0.102726269	6.00074093	3.166180701	0.075178058	0.233335674
NUFIP1	-0.121373699	6.001323994	2.064549626	0.150759462	0.362205716
HLCS	0.501115256	6.001528092	43.10291851	5.19E-11	8.38E-09
UBE3B	-0.214559151	6.002365433	10.16428244	0.001431876	0.015645103
PGPEP1	-0.147556745	6.002837094	1.238968281	0.265669984	0.500870713
LOC425607	-0.082262579	6.003095774	1.049632925	0.305591643	0.543799122
ERI3	0.083683026	6.003108314	1.994125646	0.157910174	0.372585592
ZFYVE16	-0.054676403	6.003287527	0.408752335	0.522603451	0.727348371
TMEM222	-0.10210237	6.003841974	3.333560547	0.067879778	0.219098029

GLDC	0.070228386	6.004542425	0.601115511	0.438152724	0.663886934
DNAJA3	0.005280319	6.004654228	0.006670971	0.934904353	0.971447144
GLOD4	-0.054475748	6.005012151	0.813677695	0.367035168	0.602190739
SLC1A2	-0.19578174	6.005579692	3.685437248	0.054889578	0.190718017
YIPF6	-0.150162874	6.00574776	5.439148364	0.019690268	0.098259383
INO80C	-0.216461548	6.00603215	10.2132944	0.001394319	0.015295677
DNTTIP1	-0.10507096	6.006088836	1.767830708	0.183650774	0.406124645
EVL	0.01653971	6.006509436	0.073464223	0.786358081	0.894849393
INO80	-0.040049477	6.006662408	0.343832407	0.557625393	0.753529264
TOPBP1	0.038043097	6.006708324	0.375340158	0.540107717	0.740993203
ARHGAP29	0.013570235	6.008083155	0.025510173	0.873102399	0.941906508
IFT172	0.122320907	6.00813494	3.473063221	0.062375701	0.207940065
CHST3	-0.082993223	6.008521453	1.124232387	0.289008933	0.525485304
ZFAND6	0.009848537	6.008640776	0.028145666	0.866766819	0.93840635
TBC1D1	-0.025650468	6.008773322	0.164213893	0.685306152	0.835563601
SLC38A7	-0.138417782	6.008888141	3.004306236	0.083043522	0.248033218
CTDSP1	-0.054220281	6.009180294	0.58918577	0.442734149	0.667447588
FNDC3B	-0.049386704	6.009591311	0.276615793	0.598927974	0.781613119
RB1	0.125310229	6.009843328	4.94954769	0.026097495	0.118334055
SMC5	0.056528819	6.010434203	0.44286518	0.505742787	0.715440088
CREB3	-0.151546673	6.010857223	2.929416149	0.086978943	0.255669533
GPAT4	-0.052354797	6.010962431	0.793615595	0.373009083	0.607528899
TWF1	-0.02468055	6.011231201	0.206042686	0.649886947	0.813173579
IGF2R	-0.07112131	6.011484491	1.104132646	0.293360947	0.530584126
FAM72A	-0.013674746	6.012313946	0.034912743	0.85177862	0.93117308
MED6	-0.017679001	6.01242412	0.103387549	0.747801703	0.872702382
PTPN1	0.023085925	6.012594457	0.129554229	0.718894678	0.854788057
SNX9	-0.022425051	6.013279182	0.129696403	0.718747027	0.854724131
SPDL1	0.258797941	6.013537924	22.74504431	1.85E-06	7.89E-05
VWA9	-0.025368208	6.013661985	0.147467159	0.700967679	0.844765135
RNF212B	0.061552746	6.013703652	1.100282438	0.294204129	0.531116725
MMADHC	-0.081935718	6.01445263	1.898405769	0.168256873	0.386469408

PCASP1	-0.082901263	6.014842475	0.963577094	0.326287274	0.564154365
LTN1	-0.005509302	6.015129992	0.006064634	0.937926879	0.973175282
RP11-762I7.5	-0.006270163	6.015328032	0.011382882	0.915034446	0.961017703
GBA2	0.139566225	6.016292298	3.275833925	0.070307186	0.223902604
E2F1	-0.002540675	6.0176332	0.001146435	0.972989546	0.989268858
NOL7	-0.484307172	6.017912664	45.52311728	1.51E-11	2.82E-09
NDUFS8	0.041282622	6.018064065	0.545235677	0.460270955	0.68238923
PPP2R5E	0.133702064	6.019401653	5.674795184	0.017210349	0.090294756
KRR1	0.099133145	6.019441485	1.904791587	0.167542967	0.385423663
FBXO42	0.099243337	6.019750692	2.628875729	0.104935994	0.287691066
NVL	0.188096286	6.019914738	11.11297494	0.000857259	0.010727143
HES5	0.104716831	6.021740693	0.581377417	0.445772924	0.670322942
KCTD12	-0.242633325	6.021905512	4.669447214	0.030703809	0.131331211
SIGMAR1	-0.066791258	6.022039764	0.52866308	0.467169471	0.686899055
RICTOR	0.11137539	6.022315636	1.380954766	0.239938583	0.472384468
FEZ1	0.100935296	6.022758358	1.297435682	0.254682157	0.489578844
TFIP11	-0.101280828	6.022817761	3.380750403	0.065962016	0.215420225
TRNT1	0.088793843	6.022861005	1.718815993	0.189845524	0.413257267
DPH7	0.172118653	6.022884974	6.732819055	0.009465489	0.059691561
YES1	-0.041531369	6.022930445	0.435996776	0.509060997	0.717892027
TMEM70	-0.072051493	6.022982318	0.967439715	0.325319538	0.563428375
NUFIP2	-0.069437425	6.023028491	0.647069449	0.421162381	0.650664442
GNAT2	0.115596277	6.023073736	3.986314554	0.045871294	0.169735134
SLC9A8	-0.067624644	6.02317581	1.414378997	0.234330813	0.466204878
GSR	0.062022626	6.023450019	0.940887561	0.332049688	0.569509862
FLCN	-0.062198431	6.023697349	1.36272887	0.243065104	0.476013219
TARSL2	0.201551134	6.023958465	12.42363527	0.000423934	0.00638813
HOXD4	0.354335084	6.024529135	12.98055668	0.000314742	0.005112622
LOC107049485	0.000644287	6.024709117	0.000119437	0.991280321	0.995986157
B4GALT3	0.107908737	6.025100528	3.821714555	0.050592443	0.181174895
RANBP9	0.03299877	6.025118396	0.348668082	0.554868067	0.752584408
GNG5	0.202983601	6.025370103	9.285271009	0.002310037	0.022102937

SUPV3L1	-0.059422721	6.026473578	0.940970253	0.332028442	0.569509862
CLDN12	-0.073044364	6.026530362	1.046684802	0.306271844	0.544479602
LBH	-0.106611763	6.027275388	1.774816775	0.182787087	0.405249463
MCL1	-0.103195261	6.027747296	2.570617702	0.108865215	0.294440683
PNPLA3	0.339050322	6.027908401	12.66205007	0.000373153	0.00580362
ICK	-0.253483792	6.02807126	15.15448664	9.91E-05	0.002045264
RNF170	0.165945404	6.028674203	3.911174053	0.047966077	0.174695731
C18H17orf75	0.166456412	6.029347346	5.370745668	0.020477222	0.100778202
DPP8	-0.022717252	6.029705778	0.160901246	0.688328113	0.837754371
NPTN	0.00921591	6.029965841	0.020888998	0.885081757	0.9486918
QDPR	0.280623916	6.030600347	13.73742008	0.000210224	0.003723613
GATAD2B	-0.002091729	6.031102046	0.001009878	0.974648627	0.989871494
BTBD3	0.036068297	6.031164628	0.297287315	0.585588049	0.772343452
NUDT1	-0.328588845	6.031349226	18.43774257	1.76E-05	0.000505887
KLHL9	-0.086571645	6.031724109	2.037201971	0.153491757	0.366150603
CD2AP	0.204593174	6.031946762	7.744402478	0.005387929	0.040207875
DYNC1LI2	-0.082787182	6.032299353	1.230856156	0.267240601	0.502710277
RASA3	0.054880497	6.032998275	0.737252976	0.390542271	0.624040889
ACBD3	0.014744598	6.033074757	0.070157099	0.791108182	0.897338191
NOX4	0.06520362	6.033335303	0.509269102	0.475455427	0.692572543
PQLC1	-0.040210967	6.033647273	0.347349748	0.555617221	0.752912164
LOC107049545	-0.271164874	6.033753234	10.48966341	0.001200442	0.013660636
IFT52	0.139906643	6.033832563	4.025184964	0.044825704	0.167123725
LMO2	0.262548604	6.03393729	10.68087474	0.001082489	0.012714026
TCEA2	-0.272641003	6.033968384	18.12061525	2.07E-05	0.00057828
ABCC1	0.106632217	6.034361497	2.532229172	0.111542538	0.299369182
TBC1D10A	-0.044181932	6.035496073	0.520524518	0.470618023	0.689405693
CMIP	0.117121706	6.036797536	1.355770385	0.244271858	0.477487933
FAM76B	-0.026882465	6.036949545	0.190450014	0.662542338	0.820943839
TTF2	0.141362706	6.037073476	6.066064331	0.013780369	0.077865936
JOSD1	-0.023009091	6.037129591	0.151935838	0.696692225	0.842141376
USP20	-0.223936655	6.037477697	11.48651316	0.00070103	0.009265425

INTS7	-0.270562698	6.037483667	11.89781759	0.000561998	0.007890503
CECR5	-0.082677915	6.037636637	1.390293783	0.238355493	0.470995489
RNF216	-0.026119221	6.037744249	0.248668053	0.618014508	0.793494751
DPY19L4	0.004177666	6.038901338	0.004337845	0.947487438	0.977802274
BHMT	0.284220296	6.039121187	3.693595866	0.054621739	0.190222373
TUBGCP2	0.174712205	6.039238289	6.643440349	0.009952156	0.061890677
C1H12ORF57	-0.165248896	6.039401895	3.987822246	0.045830263	0.169621451
LOC100859276	0.145773656	6.039477696	3.434688727	0.063840614	0.211027985
WASHC4	-0.187817011	6.040339812	11.68344708	0.000630586	0.008582541
ECD	-0.11765214	6.040521322	2.969654766	0.084839958	0.251493697
CLVS2	0.014232883	6.041139991	0.014304605	0.904798546	0.95651142
KMT5C	0.167110298	6.041694392	5.587328641	0.018090865	0.092927773
NEDD9	0.141462331	6.041990541	2.115238231	0.145839358	0.355022129
ACD	-0.200606991	6.042103589	6.216874684	0.012653829	0.073372
TGS1	-0.113891485	6.042339549	3.555281923	0.059356224	0.200885782
RAB35	-0.094024036	6.042447324	2.576713767	0.108446595	0.293743001
NOL10	0.127248309	6.042637835	4.197008621	0.040495353	0.157678902
WASHC5	0.126939459	6.043183392	5.368368897	0.020505144	0.100810319
MYO7L2	-0.200518488	6.043274992	0.564157043	0.452590139	0.67549848
ETAA1	0.058606658	6.044301319	0.872537945	0.350253655	0.586825349
TRIM59	0.108858663	6.04478794	2.098140241	0.147478424	0.35740169
CACNA2D2	0.154407331	6.045659724	3.30632005	0.069013876	0.221282799
OGFOD1	-0.056960795	6.045851318	0.576293315	0.447768902	0.671102767
ANKRD54	0.091273683	6.045907895	2.837496919	0.092087563	0.265283762
POLR1C	-0.031588635	6.046000347	0.225767172	0.634680325	0.804224916
NRBP1	-0.12346139	6.046017469	3.70146287	0.054364784	0.189709631
TBC1D22B	-0.002483506	6.046071177	0.002174166	0.962809755	0.983978295
CCNDBP1	-0.060640701	6.046517914	0.993937192	0.318781991	0.556439764
PIK3R3	-0.075176983	6.046562206	0.81734662	0.365957096	0.60110042
NEDD4	-0.043971418	6.046615228	0.619389938	0.431274079	0.65855744
AGL	0.07477215	6.046636605	1.337850583	0.247413378	0.481398503
ASXL3	-0.006163858	6.048553161	0.008468208	0.926679872	0.967217925

FBN2	0.218346308	6.049105703	5.313135514	0.021165231	0.103161692
BACH1	0.026915496	6.049175661	0.18518762	0.666952471	0.82382197
KLHDC10	-0.057257904	6.049388984	0.688706465	0.406604709	0.637633886
SIKE1	0.128596383	6.049773235	3.975408908	0.046169242	0.170404892
SF3B5	-0.038796766	6.049778065	0.438827937	0.507688703	0.717083057
TBC1D24	0.133850133	6.049833653	3.616880298	0.057196032	0.196197771
WDR47	-0.085888424	6.04990076	1.722206073	0.189409322	0.41297607
GINS2	-0.115190204	6.050102146	2.219856511	0.136245722	0.339685356
ANKS1A	-0.023704609	6.051405622	0.121772211	0.727120276	0.859537653
NUTF2	0.005864334	6.051695632	0.007920113	0.92908587	0.968521254
GOLT1B	0.041190554	6.051906533	0.516721599	0.472243515	0.690861219
CCND1	0.229851559	6.051945608	7.55116795	0.005997119	0.043344549
ARMT1	0.105931506	6.052069897	2.125893	0.14482835	0.353635628
XRCC6	-0.018742688	6.052275128	0.068857989	0.79300686	0.898315289
PPIL2	-0.094011108	6.052629145	2.548452347	0.110402354	0.29723016
LOC112531894	-0.945837152	6.054306955	167.499115	2.60E-38	1.07E-34
LOC107050717	-0.175128261	6.05463828	7.725042078	0.005446009	0.040481464
SIPA1L1	0.133663505	6.054716538	2.953764991	0.085677742	0.252974562
TOMM7	-0.049284447	6.054818633	0.513420323	0.473661961	0.691702838
ZFX	-0.043128306	6.054983299	0.443532567	0.505422345	0.715440088
ATM	0.227936408	6.055216249	11.37771699	0.000743304	0.00967648
CLAPS2	0.109362797	6.055313119	2.113406398	0.146013977	0.355160382
REEP2	0.108274526	6.056152164	2.437023412	0.118500898	0.310692768
FAT3	-0.06633966	6.056357991	0.302395163	0.58238475	0.77051357
ABCB8	0.145078525	6.056804069	2.549030395	0.110361966	0.297170045
CTGF	0.416836208	6.056964209	22.04502206	2.66E-06	0.000107677
MGAT5	-0.068513735	6.057505412	0.564170242	0.452584852	0.67549848
ANKRD31	0.052994222	6.057850691	0.266131589	0.60593862	0.786214274
ARIH2	-0.066761609	6.058680343	1.582355112	0.208421785	0.436100304
CLPX	-0.072837655	6.059261326	1.707398664	0.191323229	0.415364966
PPTC7	-0.058094391	6.059592492	0.874950458	0.349588448	0.586150185
RNF141	0.041898461	6.05964093	0.53665884	0.463820719	0.684929547

PAPD7	0.046673646	6.06030747	0.273487649	0.601001765	0.782591357
NCOA2	-0.062679403	6.061462374	0.75444178	0.38507357	0.619718521
CDKN1B	-0.080185984	6.061733473	1.511511312	0.218908618	0.448199473
CDK8	0.106907169	6.061741857	2.429559629	0.119066343	0.31158344
HTRA2	0.095948922	6.061832118	1.760170271	0.184603279	0.407136705
ZBTB2	-0.068075917	6.061980175	0.966845259	0.325468224	0.563507958
SCP2	0.087805957	6.062833144	2.795082887	0.094553877	0.269230671
SRP9	0.064662255	6.062870941	0.914461634	0.338933381	0.575913753
AGO4	-0.05664512	6.06330665	0.563684858	0.452779356	0.675632701
LOC107054982	-0.032577414	6.063556595	0.261398033	0.60916131	0.787473436
HMCN1	-0.237211616	6.063699602	6.150346093	0.013138639	0.075276397
NDUFS7	-0.021331801	6.063784971	0.101695702	0.749804138	0.873444126
GUCY1A2	-0.007552511	6.063846431	0.005596176	0.940367798	0.974411302
CYFIP2	-0.04322769	6.066832678	0.319327135	0.572012293	0.763812568
MICU2	-0.415953544	6.067638532	59.86666889	1.02E-14	3.88E-12
SCAMP2	-0.145225363	6.067792246	5.458815863	0.019469837	0.097705448
CROCC	0.186786123	6.068355363	6.700991276	0.009635932	0.060491209
IRS2	0.010686948	6.068382908	0.021800427	0.882619248	0.947489154
PLEKHA1	0.104484355	6.06849401	2.239452611	0.134528597	0.337397967
VANGL1	0.031435373	6.068564364	0.248709104	0.617985507	0.793494751
LCMT2	-0.03329262	6.068720172	0.265636705	0.606273841	0.786447764
NDE1	0.236817491	6.06872524	11.89478814	0.000562912	0.007896609
DDX49	-0.049026336	6.069241075	0.804744513	0.369678613	0.604257632
ZPR1	0.063877641	6.069374835	1.350692413	0.245157105	0.478649758
AATF	0.168778936	6.069578973	4.72398978	0.029744651	0.128531574
NEDD8	-0.001514123	6.069761667	0.000561093	0.98110196	0.991828264
SMUG1	0.236014638	6.069920579	9.186206118	0.002438458	0.022876183
LOC776275	-0.070095248	6.070269732	1.337526391	0.247470666	0.481416217
WBP2NL	-0.010255306	6.070362389	0.016930113	0.896474836	0.95275104
KDSR	-0.099359826	6.070473591	2.330944839	0.126824877	0.324456368
KNOP1	0.052961748	6.070763286	0.526511473	0.468077212	0.687574588
SELENOM	-0.554004238	6.07080025	46.87193846	7.58E-12	1.50E-09

TMEM41B	0.174424721	6.071072238	5.418793554	0.01992112	0.098914314
REXO1	-0.026578534	6.071146349	0.207251162	0.648930499	0.812579461
ATG9A	-0.130366096	6.072243224	4.390608974	0.036137403	0.146247165
NAA20	0.040043272	6.072639314	0.467737915	0.494029678	0.70621652
RNF126	0.069174647	6.072714566	1.584564275	0.20810447	0.43586316
RIOK1	0.013767097	6.072826662	0.039143452	0.843164738	0.926094935
KMT2A	-0.080902738	6.073962638	0.401876279	0.526121856	0.730515158
LOC423752	-0.131203275	6.075474935	1.607758567	0.204807165	0.431676944
DNAJB14	0.083976731	6.075570922	2.311260979	0.128439817	0.32741707
SCRN1	-0.023560375	6.075576677	0.116864598	0.732460284	0.86296537
PPIP5K2	0.00878113	6.075737287	0.010741376	0.91745456	0.96231374
SMG5	0.017756417	6.076257268	0.080452839	0.776684673	0.889376916
ST3GAL3	0.121626347	6.076296627	3.680154542	0.055063746	0.191114521
CA2	0.274252667	6.076868655	6.969973268	0.008288873	0.054576529
NENF	-0.010250867	6.077004065	0.018663539	0.891335463	0.951008202
CMTR1	0.105708082	6.077183652	2.558272213	0.109718435	0.295824488
GMDS	0.103833592	6.077858206	2.919337462	0.087523794	0.256476892
CCDC85A	0.057443816	6.078017358	0.201244906	0.653717865	0.815062002
ST3GAL6	0.096688706	6.078307609	1.485464513	0.222921502	0.452323547
ITFG1	0.025407298	6.078460671	0.114409072	0.735179244	0.864745668
KLHL15	-0.02954716	6.078640431	0.231279503	0.630576715	0.801741605
ACTR10	0.236914208	6.078686848	18.5348129	1.67E-05	0.000488465
SVBP	-0.035642289	6.078696698	0.198882189	0.655624628	0.816258385
ESYT2	0.020422357	6.078750214	0.122710442	0.726113189	0.858964236
FAM91A1	-0.081689284	6.079090356	2.001558493	0.157137566	0.371561812
GIT1	0.107941237	6.080425747	2.61564351	0.105814592	0.289280463
MALSU1	-0.076940553	6.080802011	1.798807956	0.179856674	0.40156602
TPBG	0.082211662	6.081012998	1.223892834	0.268598033	0.503621311
SLU7	0.058806531	6.081654845	0.708569591	0.399919082	0.632391745
PARN	0.04529291	6.082014274	0.512917904	0.473878439	0.691896159
COX7A2L	0.017734578	6.08240421	0.076182818	0.78253858	0.892887345
NUAK1	0.273167431	6.082455923	11.83838549	0.000580221	0.008097997

RAE1	-0.030478461	6.082643553	0.204605435	0.651028874	0.813786092
LDB2	0.066494887	6.08264448	0.892708212	0.344744462	0.581524359
ABL2	-0.163820372	6.08267077	5.127177315	0.023554003	0.110642624
C2CD2	0.236514837	6.082927572	16.19395734	5.72E-05	0.001334652
MIPEP	0.060894798	6.08303762	0.847161877	0.357356471	0.593806442
ACP1	0.027478173	6.083131147	0.161087282	0.688157449	0.837680081
DHFR	0.254485108	6.083201389	5.626897947	0.017686904	0.091711634
GCNA	-0.047491129	6.08387975	0.402745253	0.525674889	0.730140989
USP37	-0.071720127	6.08458039	0.903857243	0.341749544	0.578667292
NOP16	-0.05238918	6.08465666	0.480682102	0.488113515	0.702056988
NOS1AP	0.056765697	6.084830769	0.650028418	0.42010252	0.649636966
ARFGAP3	-0.028114132	6.08483718	0.10535751	0.745492747	0.871179827
CTNND2	0.226723718	6.08532437	11.06677285	0.000878885	0.010906527
BRF1	-0.047572809	6.085366439	0.44246437	0.505935401	0.715650909
STRN	-0.063425535	6.086310929	1.052289221	0.304980447	0.542946366
HIBCH	0.068366908	6.086579941	1.279738547	0.257947654	0.492541889
FREM2	-0.005942473	6.086643145	0.003641328	0.951882122	0.979652938
SAPCD2	-0.16168781	6.086990508	5.578274037	0.018184635	0.093159457
FAM83D	-0.075449285	6.087500446	0.599783445	0.438660664	0.664105366
PCDH1	0.256837248	6.087769082	8.975255284	0.002736603	0.024728616
FIGN	-0.032288651	6.088297545	0.310431519	0.577415509	0.76705002
FAM129B	-0.059843315	6.088425573	0.408827533	0.522565204	0.727348371
MAPK11	-0.012529404	6.088588989	0.028663163	0.865559112	0.937950292
GRAMD4	-0.094965315	6.088629908	2.004052686	0.15687927	0.371217773
FAM160B1	-0.196792729	6.089020204	11.90709917	0.000559204	0.007882433
GTF2I	0.003236804	6.089522574	0.003019298	0.956179783	0.981041173
LOC415641	-0.182523909	6.089589488	6.759860801	0.009323103	0.058981801
CBLL1	0.116807315	6.089878975	3.168117944	0.075088928	0.233173866
USP38	-0.09747411	6.091485096	1.847977106	0.174018852	0.393551431
HEYL	-0.006035766	6.091814332	0.006361794	0.936427457	0.972171218
CHIC2	-0.166414545	6.092087091	4.89897612	0.026872624	0.12073464
MIER1	-0.126072043	6.093172969	3.629349048	0.056769016	0.195081258

TNS1	-0.274870962	6.09353616	6.331057676	0.011864154	0.069897836
SKA3	-0.032515816	6.093671735	0.20313281	0.652203927	0.814579419
BTBD1	0.009780493	6.093818586	0.024387689	0.875902505	0.943751684
NAA16	0.103550594	6.09459621	2.730645476	0.098439492	0.275339427
TAF5L	-0.011004628	6.095072756	0.027363543	0.868613968	0.939356373
RPAP1	-0.037386375	6.095986211	0.381303941	0.536906214	0.738139507
LOC107050604	0.008034798	6.09631186	0.015633323	0.900497198	0.95453991
CETN1	-0.040073619	6.096358372	0.381979196	0.536545903	0.737836448
SALL3	0.219499736	6.096568965	3.701279909	0.054370746	0.189709631
SOAT1	0.028959683	6.097277534	0.128463435	0.720030552	0.855582231
RRAGC	0.034118014	6.097309459	0.293693627	0.587863232	0.774114284
OST4	0.127491954	6.097472454	0.995924147	0.318298759	0.556048026
FZD3	0.028687241	6.097882774	0.141482519	0.706811613	0.848204864
ARFIP2	-0.180927353	6.098074055	10.37290992	0.001278778	0.01433399
VTA1	0.131631443	6.098174759	4.464239017	0.034611504	0.142240832
POLR2H	-0.056281841	6.0986543	0.673621296	0.4117919	0.642034842
GALNT2	0.347440215	6.098824571	30.77469055	2.90E-08	2.26E-06
ZFP64	0.04748356	6.098876749	0.834316208	0.361027414	0.597476225
BCAP31	0.036313674	6.099378158	0.226233854	0.63433054	0.80403489
CHMP5	0.019850083	6.099403087	0.128022189	0.72049158	0.855792151
ABCD3	-0.037691698	6.099676344	0.448166085	0.50320714	0.71381668
PNPT1	-0.085850384	6.099817731	1.860542522	0.172562198	0.391818818
WDR48	-0.040697809	6.100371796	0.538690085	0.462976105	0.684057976
QPCT	-0.026466432	6.100756224	0.160033125	0.689126021	0.83810791
GATSL2	0.079543955	6.100841391	1.032992582	0.309456831	0.547421217
EMSY	-0.019619907	6.101273267	0.133581845	0.714746788	0.852264423
PAX1	0.42746464	6.101678024	5.343362711	0.02080131	0.101779825
SMOC1	-0.101679142	6.101860346	0.528590865	0.467199892	0.686899055
TNNT2	-0.170764953	6.102087462	0.3637793	0.546414764	0.746408774
LOXL1	0.077614013	6.103045512	1.042140864	0.307324086	0.545348629
NEBL	0.165823367	6.103191959	3.573304751	0.058715351	0.199620061
UCHL1	-0.229628675	6.103538031	6.883438408	0.008699804	0.056293856

PPIE	0.013019423	6.103658854	0.047052481	0.828273935	0.918593819
ABI1	0.142380663	6.103903476	5.529472196	0.018698745	0.09484829
HMBS	0.192793875	6.104097094	9.10009628	0.00255596	0.023641556
RSPRY1	-0.065729458	6.10487379	0.90569758	0.341258561	0.57831201
AHCYL2	0.062994802	6.105014559	1.312062176	0.252021626	0.486796086
ATAD1	-0.016883532	6.105106395	0.046848619	0.828640567	0.918759906
CHURC1	0.137669784	6.105584581	2.158211036	0.141809708	0.348957492
ETV5	0.059071167	6.105745021	0.578456627	0.446917911	0.670621395
IRS4	0.00786618	6.106235079	0.00949749	0.922365016	0.964657225
FRZB	0.206823059	6.106319605	4.89873417	0.026876389	0.12073464
HOXB8	0.112197939	6.106621486	1.742064768	0.18687742	0.409899752
ENY2	0.025286013	6.106672763	0.137341087	0.710938771	0.849781439
SIN3B	-0.204225162	6.106991003	8.855578466	0.002921945	0.02594744
PTPRU	0.30952597	6.107420928	6.840532001	0.00891123	0.05715526
ZBTB33	0.164614811	6.108038503	10.13543116	0.001454462	0.015797475
UNG	0.007899924	6.108381956	0.008492684	0.926574285	0.967217925
THSD4	0.165172913	6.108482879	2.607646015	0.106349521	0.290287555
NUDT3	0.207846382	6.109244422	5.878856733	0.015323769	0.083136371
C4H4ORF46	-0.07602297	6.109966136	1.28357962	0.257234525	0.492128137
NAA30	-0.098204066	6.111037055	2.536849298	0.111216516	0.298821132
RUNDC1	-0.116480162	6.111261846	2.37370854	0.123393606	0.318433997
ZMYM3	0.101245664	6.11171547	1.941659148	0.1634885	0.380007306
LOC107051192	-0.122535071	6.112808353	3.062348958	0.080125761	0.242578156
RNF123	0.150732978	6.113338796	6.469410243	0.010974695	0.066319721
AMFR	-0.115118944	6.113453653	2.495199399	0.114193912	0.303152969
THUMPD1	0.009081062	6.114416808	0.012574211	0.910716532	0.959032416
SNRNP27	0.151015728	6.115077457	6.564774097	0.010401694	0.063746695
TBCB	0.316387178	6.115122288	17.01604647	3.71E-05	0.000935443
FRG1	-0.158178458	6.115457001	5.203116067	0.022546436	0.107318946
NNF1	0.280655376	6.115530687	9.054402524	0.00262063	0.024023661
IDI1	-0.225615066	6.116485816	7.4677145	0.006281524	0.044959759
INTS11	0.223966824	6.116855212	12.77871089	0.000350586	0.005525766

MINOS1	0.055609529	6.117445473	0.626107622	0.428786669	0.656275148
TBX3	-0.326388382	6.118431965	2.236155807	0.13481578	0.337831175
DACH2	0.218127235	6.119264508	7.385228952	0.00657617	0.046362842
IRF2BP2	0.106625571	6.119862243	2.16354635	0.141318205	0.348249245
LARP7	0.17647934	6.119995714	6.975461575	0.008263491	0.054499294
SCYL2	-0.018505974	6.121117435	0.106972091	0.743618056	0.869767312
DIAPH2	0.122606318	6.122752276	3.935888744	0.047266159	0.172997432
CHP1	-0.068478136	6.123324244	1.136841091	0.286320812	0.522676831
UFL1	-0.018194386	6.123681001	0.069283548	0.792382803	0.897798812
TTC4	-0.067840322	6.124637169	0.638534253	0.424242064	0.65309226
TPCN1	-0.116268451	6.125120663	2.952405338	0.085749843	0.253142029
ANAPC7	-0.064912267	6.125265455	1.352585216	0.244826674	0.478288368
FLRT2	-0.236496288	6.125579517	3.773373906	0.052074494	0.184355808
MRPL19	0.037879228	6.126171191	0.401321754	0.526407437	0.730603439
MED17	0.016912209	6.126706451	0.085698762	0.76971821	0.88505745
NDUFS3	0.062023864	6.12689913	1.25163494	0.263240442	0.498460469
LUC7L	-0.037832617	6.127601173	0.442864984	0.505742881	0.715440088
GMPPA	-0.164166845	6.127661312	8.772988926	0.003057241	0.026758985
FAM168A	0.153417038	6.127911553	8.131099347	0.004351236	0.034472596
ES1ML1	0.093798785	6.128109649	1.506845751	0.219621028	0.448927208
FNTB	0.066516132	6.128807477	1.194802346	0.274363002	0.509389401
DHCR24	-0.332358311	6.12915371	18.47697095	1.72E-05	0.000497322
TRAF7	0.152141894	6.129174876	7.986100139	0.004713784	0.036705484
LOC107049953	0.133655825	6.129317351	2.50390394	0.113564471	0.302428124
GRPEL1	0.018666125	6.130383986	0.107474497	0.743037911	0.869560491
LOC112532833	0.075608752	6.130661447	1.535759662	0.215250076	0.444471699
GRAMD1B	-0.118611435	6.130799898	2.706591386	0.099934316	0.277867383
STAT5B	0.0685856	6.131744956	1.105224457	0.293122407	0.530432584
MAT2A	0.12711744	6.132325235	2.39640907	0.121614126	0.315675937
LRRC8A	-0.188100093	6.132554089	6.122850928	0.013344541	0.076138845
VASN	-0.306511731	6.132754298	17.85648034	2.38E-05	0.000652195
DROSHA	-0.028403748	6.132830861	0.267600487	0.604945947	0.785479804

KBTBD2		0.081892151	6.133058222	1.680569095	0.19484885	0.419664638
TPST1		0.182277653	6.133096724	5.69120512	0.017050142	0.089807965
NIP7		0.023196478	6.133308912	0.071580752	0.78904896	0.89623805
GJA5		0.04641442	6.133597555	0.158078289	0.690931988	0.838937858
ULK2		0.041674779	6.133919924	0.464698905	0.495436082	0.707243926
NANS		0.431042357	6.134239019	35.32013755	2.80E-09	2.82E-07
CPZ		0.416130464	6.134549644	17.29712475	3.20E-05	0.000822232
SNX14		0.031927555	6.135439807	0.373134107	0.541300848	0.741887844
PHC1		0.035464692	6.135735201	0.442908627	0.505721915	0.715440088
PTTG1		0.146764749	6.136308862	4.500596587	0.03388303	0.14015718
KDR		0.036991209	6.13683144	0.215198294	0.642723189	0.809182103
FAM193A		0.070160581	6.137626654	1.683973408	0.194397316	0.418911642
	05/mar	-0.161509551	6.138060926	4.814190555	0.028226326	0.124620508
ASH2L		0.047818294	6.138260939	0.870684757	0.350765809	0.587324724
ENC1		-0.006263959	6.138261913	0.003169349	0.955105233	0.980856058
NME3		0.055453229	6.138557709	0.652152958	0.419343991	0.649012971
BAG3		0.225455645	6.138648096	11.17045111	0.000831104	0.010503701
SLC25A1		0.103889003	6.138957169	2.652086548	0.103414076	0.28479977
LPIN2		0.02161613	6.139126885	0.117811947	0.731419833	0.862328105
SIRT2		-0.006918333	6.14004046	0.008155028	0.928044694	0.968067662
RRAS2		0.10034919	6.140173184	3.203500874	0.07348082	0.229566528
STK35		0.043666315	6.141235178	0.316543551	0.573692286	0.764869254
MRPL38		0.125838102	6.14123864	1.834537047	0.175592591	0.395317565
TBCA		0.09650223	6.14142357	0.908431535	0.340530921	0.577554763
SLC9A1		0.136425371	6.141666363	4.398151907	0.035977898	0.145852751
RSL24D1		0.143968356	6.141799208	4.856377096	0.027544117	0.122729069
INO80D		0.01917533	6.142116283	0.086720196	0.768388905	0.884493839
T		-0.051474574	6.143230248	0.215549554	0.642452061	0.809078732
CECR2		0.04903028	6.14430957	0.329497926	0.565954877	0.75954551
C4HXorf56		-0.244009709	6.144525711	12.97821831	0.000315136	0.005113963
ITGA8		0.191396633	6.14522699	6.60174407	0.010187893	0.062810705
PNPLA7		-0.013012127	6.145320838	0.058608016	0.80870971	0.907749244

CERK	-0.011111425	6.145860576	0.02570632	0.872619628	0.94163263
IGFBP4	-0.105829081	6.146769769	1.663746051	0.197098298	0.422518563
SHROOM3	-0.073757081	6.146915991	0.393289037	0.530575591	0.733450191
SMAD3	-0.085201607	6.147133323	1.385116892	0.239231474	0.47181432
PRMT3	0.144893911	6.147642938	5.957098488	0.014658145	0.080768714
PRKAG2	-0.003077382	6.147664553	0.002460764	0.960436294	0.983096197
LEMD3	-0.120517751	6.148021031	4.508842739	0.033720048	0.140010949
CAAP1	0.139305713	6.148055669	2.382830032	0.122675131	0.317392968
EMC2	0.095224196	6.148589623	1.621797976	0.202841268	0.42951397
TUBG1	0.079766033	6.148718131	2.006709131	0.156604703	0.370940798
SIX4	-0.171852017	6.149945678	4.582757223	0.032295209	0.136033958
MFSD1	-0.080145364	6.149957335	1.879970424	0.170337509	0.389184076
GFPT2	-0.147073772	6.150259754	4.736334478	0.029531929	0.127899143
RSBN1L	-0.053516564	6.150936914	0.704483421	0.401281288	0.633814897
RBM6	0.164144378	6.151534441	4.911653448	0.026676093	0.120228737
DCTN3	0.149264646	6.151618032	2.832767805	0.092359058	0.265645587
APBA2	0.015178177	6.151855436	0.050548245	0.822112018	0.916392801
MRPL45	0.134792707	6.152338849	4.189561847	0.040673608	0.158073742
OCIAD1	-0.082289446	6.152460069	1.243188419	0.264857451	0.499968953
PIM1	0.091015199	6.153834188	1.10262083	0.29369166	0.530848821
APOA1	0.977913393	6.154987285	22.21712023	2.43E-06	9.99E-05
MED16	-0.127918466	6.155005777	5.09672555	0.023971054	0.111930673
RIC8A	-0.025753785	6.155468759	0.224531279	0.635608791	0.80476003
LRP11	-0.147272264	6.155683008	1.594593045	0.206671141	0.434057058
TIMELESS	0.076066202	6.155946164	1.486083105	0.222825184	0.452323547
TIMMDC1	0.186686828	6.156404091	9.195032173	0.002426728	0.022805148
SLC35C1	-0.010636271	6.156867199	0.025211928	0.873840125	0.94264057
TET2	-0.172252041	6.156884596	5.225607174	0.022256675	0.106556178
CHCHD7	0.096905763	6.156991508	3.357022424	0.066918963	0.217030217
FPGS	0.155147216	6.157212458	5.486274035	0.019166347	0.096506193
POMGNT1	0.005466801	6.157568029	0.007362398	0.931621894	0.969997359
DMXL1	-0.257252642	6.157607016	6.998043972	0.008159883	0.053880769

SMOC2	0.422002094	6.157615144	19.87544364	8.27E-06	0.000281593
CERS5	-0.012870314	6.157699151	0.046461611	0.82933888	0.918867551
PCSK5	0.272979181	6.157813999	5.265958246	0.021746448	0.10493777
ITPR2	-0.288047003	6.158216372	7.286013381	0.006949352	0.048015912
SENP5	-0.041008174	6.158270361	0.515105318	0.472937116	0.691098524
RCBTB1	-0.062884838	6.15885313	0.651422817	0.419604444	0.649223958
NOL8	-0.146695529	6.158991688	4.975738517	0.025705227	0.117103962
ADCY9	0.0665228	6.159391331	1.311442957	0.252133566	0.486934484
C18H17orf62	0.232365976	6.15960079	11.5189089	0.000688918	0.009171641
GOLM1	0.03613415	6.159762921	0.14620363	0.70219003	0.845693564
FOCAD	0.224227232	6.160093856	4.374005944	0.036491109	0.147108386
SIRT1	0.117202336	6.160392361	3.74510618	0.052962362	0.186535888
RBM7	-0.011750652	6.160524744	0.028526202	0.865877646	0.938048368
EIF2B3	-0.11937065	6.160773167	3.234880062	0.072085581	0.226930982
LOC107056305	-0.119839087	6.160931192	1.895722148	0.168557929	0.386836922
SEMA3A	-0.052356207	6.161260315	0.261597656	0.609024663	0.787473436
TOMM40L	-0.039656207	6.161933933	0.479868331	0.488481963	0.702128818
UBE2V2	0.121561301	6.16205761	4.779765478	0.028796042	0.126061116
MFSD11	-0.004053807	6.162145965	0.002905209	0.957014847	0.981347249
AP1AR	-0.062853444	6.162279879	0.778112465	0.377719037	0.612609974
CDK9	-0.033767404	6.162403803	0.307841382	0.57900783	0.768307327
RRBP1	0.145371589	6.162406409	3.686018201	0.05487046	0.190718017
MCFD2	0.051820019	6.163275186	0.461701477	0.496829858	0.708495997
LOC422214	0.194349621	6.163535617	8.038114882	0.004580318	0.03587298
PAPPA	-0.113933422	6.163835341	1.113483123	0.291326046	0.528588609
AMBRA1	0.034708506	6.164326787	0.344871444	0.557030739	0.753115441
METTL14	0.08016192	6.165163225	1.673211405	0.195828945	0.420708925
RNF41	0.057936529	6.165579495	0.941927725	0.331782573	0.56937808
DNAJA4	0.674464827	6.165635044	29.47626824	5.66E-08	4.14E-06
DENND5A	0.093248606	6.166034308	1.702887137	0.191910835	0.416137874
ST3GAL2	-0.033448006	6.166065524	0.196506062	0.657555957	0.817412048
CSNK1G1	0.004842695	6.166114195	0.006489612	0.935793365	0.972003524

PTBP2	-0.026271925	6.166298493	0.161793772	0.687510376	0.837192573
TMEM87A	-0.09438374	6.166881194	2.192558088	0.138678828	0.344031377
TNFSF10	-0.083699398	6.167082975	1.729633132	0.188457757	0.411993143
PRRC1	0.132841261	6.167227608	3.329863053	0.068032539	0.219418939
PDXK	-0.127936965	6.167561839	2.993277901	0.083610764	0.249154317
DRG1	0.131343089	6.167875454	4.302739257	0.038051038	0.1506931
DENND1A	-0.04526173	6.168145202	0.630211905	0.427277596	0.655007718
FAM110B	0.199841581	6.16830558	7.41274731	0.006476335	0.045954099
UBR3	-0.095880142	6.169119239	1.904845812	0.16753692	0.385423663
DDX51	0.048448476	6.169164623	0.38077954	0.537186335	0.738339554
PRPF4	-0.166606593	6.169336806	9.131309892	0.002512718	0.023320229
MON2	0.043312471	6.169700236	0.547771901	0.459229526	0.681513515
EXOSC9	0.177780264	6.169763512	8.368053458	0.003818744	0.031387332
DDX41	-0.009735476	6.170081228	0.020811864	0.885292654	0.9486918
WDR55	0.245966117	6.170196191	10.47576944	0.001209504	0.013754243
CIR1	0.204196681	6.170220705	10.96727362	0.000927351	0.011379239
SLC39A14	0.191725436	6.170394966	8.053924222	0.00454052	0.035612984
PSAT1	0.130449453	6.170780155	2.133987715	0.144065537	0.352738591
TXLNG	-0.115724271	6.170913522	3.900044422	0.048284831	0.175353541
ZMPSTE24	-0.045212848	6.171471845	0.679888506	0.409625119	0.640051404
NAB1	0.092938789	6.171540427	2.221692776	0.13608378	0.339435896
RNF44	-0.02359375	6.171671968	0.146665682	0.701742336	0.845451028
PTDSS1	-0.243104057	6.171873362	16.03569076	6.22E-05	0.001432544
LOC422051	0.040668231	6.172666467	0.345050699	0.55692827	0.753115441
BUD31	0.149967891	6.172672338	5.16390381	0.023061007	0.108948856
HOXA2	-0.022149957	6.173183899	0.069280513	0.792387247	0.897798812
RMND5A	0.013066292	6.173565678	0.049410771	0.824091767	0.917134318
MCOLN1	0.037399907	6.174007323	0.464368453	0.495589416	0.707281655
RASAL2	-0.073673641	6.174123526	1.168707163	0.279667043	0.514954761
THOC3	-0.02794161	6.174636116	0.253746011	0.61444966	0.791137476
LRP4	0.168275385	6.174686275	5.192199462	0.022688484	0.107745746
TFAP4	-0.059399024	6.174886332	0.942678684	0.331589904	0.569374086

SRP54	-0.036149217	6.17584164	0.436845528	0.508648928	0.717761607
CENPX	0.13208285	6.175844861	4.033747062	0.044598781	0.166713525
NDUFV2	-0.010072492	6.176184113	0.020176938	0.887044029	0.949086044
CTNNAL1	0.468123204	6.176448566	47.3322605	5.99E-12	1.28E-09
SLC9A3R2	0.014604712	6.176563624	0.051240948	0.820917831	0.915934561
DGCR6L	0.169190688	6.176692551	6.064112146	0.013795609	0.077865936
HEY1	-0.201509493	6.176740817	7.779485467	0.005284285	0.039740815
SHANK3	0.074713849	6.177283997	1.318242996	0.250907621	0.485270911
YPEL1	-0.157100252	6.178433937	4.615340707	0.0316872	0.134211808
MLX	0.042773698	6.178468096	0.575211702	0.448195325	0.671427866
EXT1	-0.083390163	6.178669601	1.658090181	0.197861381	0.4235471
SIK1	0.487147635	6.178992851	27.44545954	1.62E-07	1.03E-05
TERAL2	0.346227343	6.17936867	0.32184813	0.570499098	0.762658433
VPS11	0.096376178	6.179650985	1.822057283	0.177068588	0.397444225
MAP2K5	0.016469346	6.17982448	0.081643263	0.775082741	0.888893846
PSMG1	0.109711173	6.179999197	3.48157184	0.062055769	0.207293479
YOD1	-0.218609052	6.180180179	10.74391044	0.00104623	0.012430121
GTSE1	0.097647606	6.180326721	2.573195335	0.108687992	0.294154753
INPP5A	0.072287036	6.180349558	1.490167141	0.222190529	0.451822189
MRPL23	-0.124578492	6.180728131	3.472503998	0.06239679	0.207968235
CEBPG	0.157057123	6.180782713	4.288765928	0.038365003	0.151618459
SEC31B	-0.042521422	6.181275835	0.539943672	0.462456072	0.683627365
CLK1	-0.171780105	6.181372983	3.410086631	0.064799045	0.212955518
SRP19	0.083322092	6.181746521	0.820710674	0.364972463	0.600737409
PSMG3	0.193904376	6.181797475	7.057223475	0.007894626	0.05272162
GPN1	-0.108818771	6.181846488	2.785068423	0.09514663	0.270217085
TRUB2	-0.048464687	6.181933298	0.700698093	0.402549224	0.634660102
INTS6L	-0.243459229	6.182326332	13.49555487	0.000239129	0.00414197
SELENOT	0.198142964	6.182474259	9.937549304	0.00161941	0.017136587
RNF8	-0.052891059	6.182772148	0.687543307	0.407001252	0.637801433
DBF4	-0.161066798	6.183353944	6.629164961	0.010032228	0.062130342
KIAA0232	-0.114272153	6.183917095	1.705711265	0.191542759	0.415657101

USP12-like	-0.08590699	6.184094752	2.233119518	0.135080877	0.338231353
INPP4A	-0.023282209	6.184946317	0.139505984	0.708772625	0.849504957
ABHD2	-0.147405907	6.185768665	5.507537469	0.018934688	0.095691121
ATP6V0A2	0.134568123	6.186126329	5.48178471	0.01921563	0.096665606
MTMR10	-0.052734045	6.186935402	0.589942388	0.442441398	0.667250774
GFOD2	-0.056187344	6.187060305	0.767627009	0.380952159	0.615531007
MKKS	0.197828749	6.187786617	6.843721205	0.008895335	0.057109926
SUGP1	-0.121863064	6.188511397	4.32415481	0.03757507	0.149614536
MURC	-0.089142317	6.188543186	0.603456372	0.437262294	0.663330972
SLMAP	0.10113742	6.188567562	3.144075886	0.076203156	0.235478484
PLEKHO1	-0.196850848	6.188578721	8.570461608	0.003416605	0.02899445
B4GALT5	0.096174014	6.188838418	2.969087294	0.084869725	0.251536622
PCDH18	-0.207450515	6.189127955	3.89494788	0.048431542	0.175612611
CDC73	0.032035153	6.189611347	0.303155118	0.581911173	0.770465751
TMF1	-0.53681473	6.189734937	82.09656124	1.30E-19	9.69E-17
ICMT	0.141614868	6.189774715	5.503544232	0.018977971	0.095739139
CUX2	-0.191374105	6.1899389	2.724696898	0.098806877	0.275898043
SLCO5A1	-0.15651732	6.191631545	2.485975598	0.114865103	0.304424812
TRAK1	0.015681898	6.19198041	0.064309816	0.799809387	0.902345328
PPWD1	0.234255618	6.1928946	7.411796273	0.00647976	0.045954099
TERF1	0.152596534	6.194140567	6.060107352	0.013826928	0.077971932
ILKAP	0.121149572	6.194438852	4.160372163	0.041380311	0.159710841
TMEM165	-0.05848152	6.194476161	0.906470938	0.34105252	0.57809447
ZFAND3	0.148754061	6.195039241	6.828143239	0.00897325	0.057475604
LNPEP	-0.162039585	6.195588735	4.638410049	0.031263955	0.133130044
DISP1	0.08698722	6.196063416	1.088772607	0.29674332	0.533680327
NCOA5	0.031294245	6.196521747	0.337530106	0.561258373	0.75591721
GEMIN4	0.128007789	6.196750985	3.066354705	0.079928514	0.24221431
FRS3	0.32755642	6.197064455	19.70764846	9.02E-06	0.00029933
LOC107050614	0.097540462	6.198920995	2.253587305	0.133305057	0.335198058
ORC4	-0.062937723	6.199056261	0.965369998	0.325837609	0.563899329
MMP17	-0.026896564	6.19907405	0.110070714	0.740063643	0.867860967

CDCA2	0.044071138	6.199209853	0.603872759	0.437104196	0.663219743
SUV39H2	0.022986728	6.199758468	0.119283938	0.729812417	0.861397607
CNOT2	-0.012912724	6.199795294	0.061617246	0.803957837	0.905174561
PDZD11	-0.085326762	6.200916028	1.806884865	0.178882372	0.399933346
URB1	0.29539818	6.201222967	14.39244583	0.000148396	0.002809969
SGCD	-0.099731768	6.201434542	2.016615751	0.155585566	0.368993436
PUS7	-0.150686971	6.202220614	4.27957618	0.038572965	0.152208497
NDUFA8	0.072153835	6.202297771	0.920385492	0.337373775	0.57457428
CDKN1C	0.021026096	6.202324887	0.035352517	0.850858895	0.930592432
SPPL3	-0.030971867	6.202822403	0.333733622	0.563468823	0.757622344
SLC6A6	0.138183403	6.202864269	2.809043386	0.093734255	0.268149716
FIP1L1	0.213992292	6.20385021	11.32777656	0.000763563	0.009851805
GINS3	0.118302073	6.204049193	3.427531489	0.064117871	0.211604407
RFTN2	-0.263509761	6.20424161	9.411778862	0.00215596	0.02117989
MCTS1	0.064358387	6.204869299	1.054153868	0.30455235	0.542507284
SHF	-0.018705207	6.205055598	0.087887019	0.766880758	0.883808857
COL9A2	-0.103400242	6.205870207	0.699588237	0.402922087	0.634882978
LOC107050254	0.029062877	6.206124534	0.206868901	0.649232675	0.81275402
TM9SF4	0.097501885	6.207245013	3.469901603	0.062495028	0.208060871
TNIP1	0.015622598	6.207398703	0.047839313	0.82686661	0.917915823
HSPA12A	-0.110018451	6.207412457	1.571686464	0.209962267	0.437998112
FANCC	0.052918717	6.207423137	0.329518695	0.565942635	0.75954551
AGFG1	0.072208861	6.207643691	1.653530917	0.198479035	0.424097198
OAT	0.140528788	6.207885343	6.466151397	0.010994838	0.066392683
SHROOM4	0.004161314	6.207929545	0.005389634	0.941476579	0.974802494
NTN1	0.224772008	6.208232029	3.445676763	0.063417445	0.210008866
GNG2	0.159284738	6.20879629	3.133894993	0.076680333	0.236375962
PRKAB1	-0.27054103	6.208975934	18.21375985	1.97E-05	0.000558246
EIF2B5	0.011362048	6.209156293	0.028964945	0.864860001	0.937563167
CRY2	0.135291813	6.209548454	5.924538087	0.014931452	0.081844451
SMARCA1	0.096719361	6.209564944	2.418906953	0.119878551	0.312763843
LEMD2	0.046769667	6.209876613	0.773853316	0.379027631	0.613681567

MYCL	-0.004250609	6.210002029	0.004954477	0.943884806	0.975921243
RASSF3	-0.243463693	6.210150235	3.806355789	0.051058424	0.182071677
SIK2	-0.131383091	6.210310463	4.180986132	0.040879908	0.15865068
PKDCCA	0.353725974	6.210572862	11.34095529	0.000758163	0.009807842
UCK1	0.045663504	6.210722878	0.51077282	0.474804499	0.692265465
GJC2	-0.006535053	6.210799365	0.008943529	0.924656192	0.966229608
KIAA0922	0.070403102	6.211113425	1.522995388	0.217166764	0.446236333
DDIT4	0.300354059	6.21122429	2.324868546	0.127320976	0.325219909
GPR27	0.193291899	6.211251293	6.869950012	0.00876571	0.056586804
ARSI	-0.461395733	6.211454266	13.17598804	0.000283559	0.00472413
HMMR	0.161076904	6.211633655	7.088370631	0.007758575	0.052035633
NKX6-1	0.141373942	6.211940812	1.101993712	0.293828984	0.530848821
RNF19A	0.153089133	6.211967005	5.923851463	0.014937272	0.081849086
MCU	0.100466222	6.212048817	3.301010653	0.069237272	0.221567349
CHL1	0.09276354	6.212555669	0.418441811	0.517715576	0.723314877
PTPN14	0.060576492	6.212618407	0.479429252	0.488680956	0.702230821
FNDC3A	0.023270232	6.212986848	0.119246785	0.729852852	0.861397607
FBXO31	-0.066212004	6.213143711	0.646527017	0.421357105	0.650673858
MBOAT2	0.005841979	6.213518252	0.008224929	0.927737809	0.967994498
RRAGA	-0.012205439	6.213661972	0.041758316	0.83808116	0.923661189
KIAA1109	-0.003551079	6.214257594	0.00221978	0.962421942	0.983899168
PAQR3	-0.061308956	6.21430428	0.752109483	0.385809177	0.620333203
CLSTN3	-0.12742894	6.214506337	4.096537645	0.042971127	0.163356737
REEP3	-0.041521696	6.214727157	0.329775375	0.565791385	0.759507887
RNF214	-0.066973271	6.215732564	1.01750919	0.313110566	0.551100049
PRELP	0.304889866	6.21652654	6.708992964	0.009592788	0.06027084
INTS4	0.01874174	6.216788239	0.105593583	0.745217656	0.870920208
ZBTB39	-0.17254229	6.217012994	6.102498514	0.013499089	0.076701489
RBM14	0.14661265	6.219135837	3.269021473	0.070599723	0.224327772
BRI3BP	-0.054296787	6.220018865	1.118546686	0.290231605	0.52706777
LOC101748916	-1.06185401	6.221482946	88.32709696	5.55E-21	5.07E-18
SULF1	-0.048922293	6.221721871	0.347397545	0.555590027	0.752912164

CHM	0.199295751	6.221839721	9.198618179	0.002421979	0.022786542
MTSS1L	-0.04606327	6.221935557	0.143922271	0.704412465	0.846807942
MEGF6L	-0.171571577	6.222403361	4.762314278	0.02908941	0.126765425
HDGFRP3	0.063641187	6.222462296	1.188322677	0.275668154	0.510824264
RBM15B	0.072281425	6.222476153	0.641450589	0.423186005	0.65260216
NDUFS5	0.162777416	6.222596152	5.167978656	0.023006972	0.108818545
VPS29	-0.013017394	6.222742525	0.048924183	0.824945962	0.917552618
AREL1	0.21391608	6.223410881	6.517448771	0.010682113	0.0649332
MAP1A	-0.309135111	6.223556643	5.456114135	0.019499966	0.097736948
LRP6	-0.02436935	6.22426689	0.105031934	0.745872693	0.871376299
EIF4ENIF1	-0.052424743	6.225136702	0.721135964	0.395771545	0.628551587
TARS2	0.11004799	6.226147623	3.902485901	0.048214716	0.175137561
DET1	-0.070151083	6.226331806	1.333716498	0.248145124	0.482309002
ZNF593	-0.077299018	6.226437123	0.653715203	0.418787521	0.648558238
PIN1	0.096813037	6.226444143	2.241031679	0.134391287	0.337173706
CCNC	0.046108846	6.226911545	0.510877045	0.474759436	0.692265465
RAB43	0.007499134	6.22784606	0.00708867	0.932901996	0.970777357
ASB1	-0.06565824	6.227846373	1.242162003	0.26505479	0.500169331
AP4E1	-0.052619756	6.22824133	0.749796446	0.386540685	0.620979506
ZNF652	-0.093189962	6.22898943	2.526911799	0.111919063	0.299695393
NOC3L	0.00829039	6.2290324	0.014989348	0.902557693	0.955420212
RRP36	0.403567471	6.230165045	13.24826713	0.000272832	0.004586857
TNFRSF19	0.145666498	6.230450364	3.702793639	0.054321445	0.189680416
LRRC47	0.080870971	6.230787418	2.111478952	0.146197964	0.355450281
SLC44A5	-0.020524305	6.231124536	0.125526188	0.723116502	0.857066913
SVEP1	0.210961389	6.231445216	3.664338114	0.055588718	0.192287652
EVI5	0.206195695	6.231574796	14.61247407	0.000132038	0.002562122
RHBDL3	-0.005086033	6.232534674	0.001645909	0.967638854	0.986817198
MLST8	0.079339747	6.232728556	2.044264232	0.152780835	0.365142868
HIVEP1	-0.083150308	6.233506669	1.170614508	0.279275012	0.514618765
AFF1	0.076757079	6.233596807	1.304370073	0.25341652	0.48783004
ATP6V0A1	-0.134511676	6.234155618	4.07799502	0.043445182	0.164493894

MRPS17	0.241326869	6.234404264	11.94654397	0.000547488	0.007773013
VPS26A	-0.071821676	6.234652889	1.226498105	0.268089156	0.503183194
PPIH	-0.049404424	6.235105523	0.611700657	0.4341482	0.660738868
MAN1A2	0.076794912	6.236552005	2.158474645	0.141785379	0.348949807
NPTXR	0.063208807	6.236589245	0.834012965	0.361114699	0.59750049
NDUFB10	0.085932508	6.236910667	1.587079134	0.207743941	0.435301993
POLR1E	0.216025962	6.237000648	9.321578969	0.002264708	0.021845683
CEBPZ	0.109428526	6.237033773	3.659496934	0.055750464	0.192604216
SF3B6	0.06569557	6.237225718	1.349446499	0.245374904	0.478972682
OCRL	-0.010573231	6.237829653	0.031210478	0.859771633	0.93438625
ATP5J2	0.004036486	6.238064874	0.002808591	0.957734979	0.981779783
ZCCHC2	-0.074361631	6.23814889	1.811954669	0.178273927	0.399115301
FDPS	-0.034155963	6.238858299	0.148506002	0.699967184	0.844053936
MRPS5	0.057820123	6.238860219	0.896483911	0.343726257	0.580746753
REEP1	0.076497528	6.239634632	0.543514065	0.460980022	0.68269519
IPO11	0.233777482	6.239698069	6.887342264	0.008680824	0.056239599
CBX6	0.029456992	6.239904407	0.132425298	0.715930542	0.852989434
ELAC2	-0.120126875	6.240121104	2.429020551	0.119107298	0.311591509
DEGS1	0.049487315	6.24115906	0.824912125	0.363747879	0.599566398
MYO9B	-0.003902418	6.241331565	0.003285574	0.954290344	0.980523198
TTLL5	0.086271314	6.241649308	1.600605133	0.205817476	0.432697913
BPGM	-0.027183131	6.241794131	0.221105213	0.638199089	0.806386086
KDELC1	0.059966195	6.2422222	1.070329006	0.300871143	0.538455175
NAPG	0.120406614	6.242350607	3.717532643	0.053843881	0.188751825
SECISBP2	0.012805574	6.243151873	0.022664274	0.8803334	0.946351332
SACM1L	-0.040592202	6.243176093	0.644691094	0.422017171	0.651251295
TOMM6	0.075932663	6.24327342	0.872455476	0.350276425	0.586825349
DHRS7	0.179977706	6.243329106	6.073226949	0.013724599	0.077607657
AK6	-0.011005125	6.243800332	0.010562008	0.918144221	0.962365851
TMEM123	-0.119381249	6.243809954	2.863614835	0.090603679	0.262618204
ABHD12	-0.132441099	6.244227783	4.736090778	0.029536113	0.127899143
RNF26	0.032969293	6.244364455	0.353760272	0.551992211	0.750601754

BIRC2	-0.141288337	6.244378248	6.333904032	0.011845129	0.069810743
FLVCR1	0.103516922	6.244620866	1.82489466	0.176731755	0.397067317
SMTNL2	-0.0149838	6.244786326	0.0574155	0.810628489	0.909184251
PER2	0.087633191	6.244905138	2.161136318	0.141539986	0.34850224
LOC107051565	0.02405065	6.245225029	0.149221188	0.699280732	0.843658952
PDE4DIP	0.112183067	6.245227871	0.9668762	0.325460483	0.563507958
AIMP2	0.096458608	6.245983553	3.006197237	0.082946676	0.247845934
LOC107049146	0.015635722	6.246075332	0.049185903	0.824485966	0.917226694
SFT2D1	0.019028644	6.246257642	0.0909434	0.762981226	0.881102924
SERTAD4	-0.034013461	6.246795936	0.422866493	0.515510175	0.722197865
MED15	-0.120306635	6.247124317	3.909320056	0.048019021	0.174743055
CACFD1	-0.215923962	6.247351543	6.196845819	0.012797818	0.073864644
AGAP3	-0.086648434	6.247478984	1.868028486	0.17170105	0.390367969
BMP2K	-0.027468496	6.247678267	0.184077557	0.667892219	0.824166458
WWC2	-0.061863896	6.248983595	0.771920519	0.379623579	0.61405408
AAMP	0.012083529	6.249395628	0.039666151	0.842134749	0.925549512
MYO9A	0.056300406	6.250008867	0.631557863	0.426784455	0.654800765
TSPAN7	-0.086733965	6.250144405	1.030941503	0.309937638	0.547741793
DAP3	0.048797356	6.250367943	0.505824859	0.47695185	0.693600685
GTF2E1	0.087870121	6.251267844	2.144130291	0.143116119	0.351069729
DGKZ	-0.007309287	6.25157936	0.012400886	0.911331461	0.959365561
RER1	-0.008603599	6.251717724	0.023694913	0.877663732	0.944829372
CLSPN	0.061415828	6.252008661	0.882416345	0.347540715	0.584085637
HSD17B4	0.305731327	6.252557575	11.86001332	0.000573521	0.008038581
PMPCB	0.300037976	6.252930563	17.32627048	3.15E-05	0.000815765
CARMIL1	0.094254873	6.253217035	2.12621244	0.144798161	0.353614387
SLC5A6	0.039515758	6.253452498	0.352958007	0.552443432	0.750843039
LSS	-0.202222111	6.254047254	5.891317792	0.01521571	0.082823192
FBXO7	-0.067977559	6.255214474	1.313941308	0.251682298	0.486369021
UFM1	0.165899364	6.255671898	3.366856426	0.066520572	0.216452808
WDR76	0.174923777	6.256391543	7.79504269	0.005238977	0.039472239
SUN2	-0.069024465	6.256509187	0.864874175	0.352378273	0.58884782

KLHL12	-0.060330398	6.2580639	1.0367136	0.308587035	0.546470046
NLRP1L	0.115066566	6.258092225	3.838250858	0.050095756	0.179944482
LIMD1	0.11449489	6.259603953	3.907362356	0.048074994	0.174835091
TANC1	-0.216761233	6.259638326	9.670619258	0.001872382	0.019184336
SLC27A4	-0.027364649	6.25982113	0.159469471	0.689645432	0.838303582
MAP3K1	0.118575175	6.260440948	3.114455988	0.077600388	0.23791958
RFC2	-0.213317949	6.261370481	9.474865974	0.002083061	0.02074865
IBTK	0.079530288	6.261505361	2.613004964	0.10599075	0.289665801
KLF12	0.074007532	6.261537838	0.977627602	0.322785216	0.56028602
PPP2R2D	-0.025722172	6.261902184	0.189430869	0.663390723	0.821350448
SAMD4B	0.000619805	6.262130677	0.000117156	0.991363973	0.995986157
ETV4	-0.054887101	6.262156021	0.285655863	0.5930179	0.777327412
ZZZ3	0.121764533	6.26229863	4.173205688	0.041068027	0.159026807
NAE1	0.035035752	6.262478646	0.257492014	0.611848434	0.789305554
DENND6A	0.022512408	6.262572838	0.122972709	0.725832447	0.858817352
EXOC2	0.057749596	6.263000072	0.723590123	0.394968803	0.627700565
PHLDB1	-0.051965209	6.263757073	0.44083167	0.506721317	0.716085474
MAP4K5	-0.140239704	6.264345809	5.949127411	0.014724573	0.081061509
ZNF740	0.003026716	6.264907355	0.003113925	0.955499098	0.981041173
PGM2L1	-0.102122674	6.265320272	2.096035536	0.147681621	0.357736063
PLA2G15	0.071133825	6.265345075	0.902281137	0.34217079	0.579261353
MTMR1	-0.042147326	6.26561092	0.584175254	0.444680393	0.669522908
SMARCD2	0.206322082	6.266428083	7.092758376	0.007739603	0.051960495
SETBP1	0.062145685	6.266647091	0.509078045	0.475538236	0.692572543
ZC3H14	0.110369762	6.266728652	3.379948265	0.065994127	0.215420225
PXN	0.246952911	6.267026196	11.42451889	0.000724812	0.009518145
SMG6	-0.101161285	6.267052633	2.801642291	0.094167807	0.268763511
PDGFRB	0.097831631	6.267955258	0.895619849	0.343958912	0.580854259
MAP3K3	-0.012053178	6.268178059	0.031179535	0.859840444	0.934387716
CLIP1	-0.12060354	6.269160195	3.325637474	0.068207568	0.219520112
SEMA4B	-0.019658085	6.269347678	0.08915646	0.765252293	0.882426523
SLC37A4	-0.150396884	6.269753281	6.058104085	0.013842622	0.078033689

CFAP20	0.081044081	6.269954572	1.761634492	0.184420774	0.407006953
NUR2L	-0.087560534	6.270114665	1.368703933	0.242034691	0.474863579
ARHGEF7	-0.101372189	6.270294188	2.709688764	0.099740449	0.277547145
LRP12	-0.042578506	6.270605698	0.312834782	0.575945837	0.765833434
SBDS	-0.074523876	6.270809641	1.760777656	0.184527547	0.407078803
NUP54	0.046107113	6.270875282	0.469314602	0.493302653	0.705551458
KIF7	-0.008663333	6.271052438	0.028994169	0.864792501	0.937551759
MRPL9	0.056577341	6.271098517	0.737529968	0.390453268	0.624019865
FAAP100	0.086249725	6.271207342	1.175424022	0.278289546	0.513657261
LRCH2	0.035494149	6.271791208	0.291936899	0.588981985	0.774881119
NKD1	-0.102834183	6.272496707	1.494882674	0.221460428	0.451003135
KAT6B	-0.07798196	6.272721776	0.913491476	0.339189722	0.576114424
DUS1L	-0.111034802	6.2729258	2.555063532	0.109941395	0.296265798
WWC1	0.225352163	6.274755741	14.34219041	0.000152411	0.002862918
CSNK1G2	0.112464248	6.274779477	2.648127886	0.103671924	0.28504178
TUT1	-0.130890101	6.275047407	3.989538573	0.045783601	0.169559788
SEC23IP	-0.053094652	6.275586048	0.887023244	0.346285257	0.582689836
PTPN12	0.087073186	6.275993661	2.610053079	0.106188208	0.290060917
SH3PXD2B	0.096137278	6.276014179	1.38644188	0.239006901	0.471810749
NOX5	-0.284038321	6.276689365	8.262561543	0.004047098	0.032707643
MAPKAPK5	-0.218559781	6.277297746	12.98816591	0.000313466	0.005106216
KIF3B	0.042056698	6.277789687	0.539320433	0.462714498	0.683909733
FNDC1	-0.024082714	6.277918184	0.021770553	0.882699119	0.947489154
PPP2R5C	0.146626842	6.277979853	4.71221082	0.029949113	0.129177628
MMGT1	0.022571331	6.278169263	0.120866926	0.728096134	0.860073359
UBXN4	-0.117655631	6.278281451	5.266668179	0.02173758	0.10493777
NECTIN3	0.07543522	6.278421384	1.220636439	0.269235787	0.504027293
CCNK	-0.023539666	6.278580807	0.178963765	0.672265281	0.826580378
ATG16L1	-0.001791396	6.278791139	0.000856953	0.976646253	0.990307746
FDFT1	-0.179747734	6.27970077	3.96565212	0.046437532	0.171022736
RIMKLB	0.184764065	6.279897832	8.243936682	0.004088835	0.032981268
GLI1	-0.151012607	6.28001137	1.715256397	0.190304798	0.413831828

FCF1	-0.103308411	6.28021575	1.921021412	0.165744104	0.383373522
DENR	0.135269461	6.280222098	4.900003827	0.026856636	0.120711812
DHX8	-0.052083034	6.280560484	0.725874961	0.394223551	0.627364461
ABCC3	-0.347106593	6.281371535	13.15823518	0.000286258	0.004757961
SLC44A1	0.077911125	6.281778995	0.630672218	0.427108847	0.654871047
GNE	-0.101150803	6.281892707	1.541713928	0.214362871	0.443411821
TMEM59	0.011536434	6.282360259	0.044931155	0.832130671	0.920518332
CEP350	-0.2786961	6.283560633	13.2323905	0.000275153	0.004605938
PHAX	0.039320246	6.28381959	0.158306272	0.690720702	0.838773544
PIEZO1	-0.040371422	6.283949431	0.177273677	0.673726745	0.827869219
NDUFB8	-0.015890727	6.283996419	0.058249521	0.809284346	0.908188905
MKL2	0.106629448	6.284813149	2.455623626	0.117104651	0.308693362
LOC112530216	-0.091222497	6.284873054	1.754807987	0.185273441	0.407836809
TSC22D2	-0.043602139	6.284972355	0.542972506	0.461203428	0.682804855
MPHOSPH6	0.000862946	6.285200829	0.000129621	0.990916183	0.995986157
TYRO3	0.228813353	6.285251465	8.617904684	0.003328745	0.028483882
FHOD3	0.026637113	6.285704561	0.079127287	0.778483586	0.890292148
LOC107049542	0.108028843	6.286010085	2.023082386	0.154924378	0.367914655
SYNE3	-0.213975139	6.286260999	9.344331445	0.002236762	0.02171441
CTNNBIP1	-0.085469806	6.286487915	1.888464822	0.169375176	0.387846997
TWIST1	-0.060361987	6.286809057	0.249584722	0.61736764	0.793345139
BAK1	0.07755775	6.287389603	1.530252483	0.216074556	0.444938908
CCDC71	-0.162526686	6.288880323	3.536109886	0.060046134	0.202346742
RMDN3	0.080287996	6.289690715	1.251131427	0.263336491	0.498485308
ERCC3	0.015735521	6.290198548	0.081287201	0.775560556	0.888893846
ATP5E	0.079516743	6.290581608	1.17679408	0.278009625	0.513313328
TGOLN2	0.055783033	6.290648038	0.648694703	0.420579744	0.650239722
EXOC4	0.05877787	6.290661449	0.62902773	0.427712173	0.655213795
UNC5C	0.13531452	6.291452713	1.440554189	0.230049682	0.461248631
KIAA0753	0.086059234	6.292099321	1.616813813	0.203536629	0.430292477
INPP5K	0.043093649	6.292160273	0.481481593	0.487751981	0.701999375
RNF130	-0.056445546	6.292555762	0.995832521	0.318321021	0.556048026

FKBP15	-0.202267268	6.29283167	13.06320125	0.000301154	0.004940672
USP15	0.051642158	6.292835388	0.801986553	0.370500086	0.604999396
KLHL2	0.181039791	6.293483659	9.04412572	0.002635402	0.024091969
CHKA	-0.037426539	6.293576467	0.400786894	0.526683152	0.730729536
SHISA5	0.064666337	6.293589611	1.625967577	0.202261698	0.429146995
USP8	0.047619706	6.294523246	0.686459191	0.407371359	0.637865574
COPS5	0.012276888	6.294798989	0.05026026	0.82261102	0.916456624
LOC112530941	0.085937497	6.29506513	0.675304491	0.41120831	0.641368033
ORAOV1	0.341386892	6.295194854	24.10758401	9.11E-07	4.45E-05
GFRA4	0.105047976	6.295346268	0.698081703	0.403429022	0.635202763
DNMBP	-0.024354382	6.296177891	0.164210299	0.685309411	0.835563601
GABPB1	0.097045879	6.296465472	2.197925564	0.138196605	0.343197273
ACKR3	0.064385957	6.296562724	0.348916354	0.554727198	0.752498563
IPO13	0.226234667	6.296620075	9.736640273	0.001806308	0.018634978
PDCL3	0.058716066	6.298113835	1.022522102	0.311921506	0.549771677
ZWILCH	0.392864138	6.299130901	32.45825492	1.22E-08	1.04E-06
ENOPH1	-0.033918342	6.299316471	0.330494665	0.565367957	0.759194353
MAP3K4	-0.116474638	6.300560811	2.264394623	0.132377932	0.333989401
BEAN1	-0.093667758	6.301047974	1.207316729	0.271864189	0.506705893
PKNOX1	0.186252651	6.301353431	7.729059617	0.005433904	0.040459229
MRPS15	0.067977598	6.3015209	1.129231227	0.287939379	0.524700164
DDR2	-0.10039529	6.301952301	1.484753307	0.223032302	0.452356596
CNIH1	0.043589345	6.302003341	0.465946475	0.494857914	0.706915718
TRAPPC8	-0.004368293	6.302220698	0.005372673	0.941568571	0.974802494
NOB1	-0.014959933	6.302381328	0.033492553	0.854790403	0.932421351
CYYR1	-0.185202345	6.302663303	3.142016222	0.076299434	0.23564324
EXT2	0.061092827	6.302733888	0.957929455	0.327709102	0.565526033
PLEKHA8	0.099885318	6.303315952	2.431358887	0.118929762	0.31136967
ZBTB1	0.001041675	6.303444582	0.000367706	0.984700979	0.992968171
ODF3L15	-0.353588346	6.304775494	9.598697823	0.001947154	0.019741479
RTKN	0.166561789	6.305007613	4.475826399	0.034377574	0.141562306
UBR1	-0.108715277	6.305281835	2.905637165	0.088270365	0.257990916

GON4L	-0.408915408	6.305751859	23.03143763	1.59E-06	6.97E-05
RAP2B	-0.1515418	6.305921652	4.461468032	0.034667691	0.14237805
LY6E	-0.015676109	6.306710661	0.079639271	0.777786856	0.889865642
P3H1	0.155996756	6.307348131	6.023974616	0.014112828	0.078988636
HIC1	-0.049079033	6.30740495	0.359397526	0.548841025	0.747861797
MRPL51	0.000946685	6.307520684	0.000203557	0.988616698	0.994817358
RARA	0.301848589	6.307904914	9.747026611	0.00179613	0.018576568
JUND	0.188047561	6.309468021	5.639979088	0.017555416	0.091346954
HMGXB4	0.090852947	6.310538003	2.47426942	0.115723203	0.305948797
MAK16	0.10194076	6.31075859	2.137930146	0.143695657	0.352123908
TUBGCP3	0.017385592	6.310915351	0.060784952	0.805259566	0.905731091
REPS1	0.053362433	6.311194061	0.866055099	0.352049744	0.588551802
UTP6	0.00813393	6.311378714	0.015785221	0.900017492	0.954423396
TMA7	0.022817045	6.31158966	0.115848296	0.733581714	0.863886574
SIPA1L2	-0.078771796	6.312001428	0.750984653	0.386164663	0.620662192
EIF4E2	0.019756643	6.312123518	0.109178746	0.741081066	0.868429635
HELZ	-0.036318046	6.312162597	0.190721547	0.662316758	0.820794617
ANKRD46	0.238041302	6.312895098	17.56495425	2.78E-05	0.000734492
DBI	0.061236318	6.313534055	1.009631431	0.314991153	0.552546231
PLAA	0.244148063	6.313914549	9.224315045	0.00238822	0.02260647
ASF1A	-0.03694735	6.314120385	0.267409788	0.605074624	0.785576327
IDH3A	-0.034925337	6.314163762	0.291665672	0.5891551	0.77500577
EIF2A	-0.023950244	6.314303497	0.129848886	0.71858877	0.854609585
PAN2	0.035096576	6.315230356	0.363169236	0.546751369	0.746559936
WDR4	-0.100848413	6.315961004	3.826040476	0.050462008	0.180988995
ATAD2B	0.066073539	6.316163761	0.853140424	0.355665442	0.592237747
ZMYND11	-0.106248445	6.316287965	3.092413954	0.078658039	0.239688525
SLC19A1	-0.153879684	6.316504074	5.683769294	0.017122545	0.090018266
PROSER1	-0.001461996	6.316586729	0.000290673	0.986397427	0.993448882
SOS1	-0.077285846	6.316961164	1.52174314	0.217355895	0.446386678
MRPS34	0.372437305	6.317219646	39.0150208	4.21E-10	5.16E-08
SZT2	0.026043122	6.317647464	0.146226885	0.702167478	0.845693564

STRIP1	0.140331883	6.318771694	5.092123701	0.024034741	0.112185069
DDX47	0.050099491	6.319607632	0.58179412	0.445609941	0.670247859
CDH4	-0.165647422	6.322022703	3.126668962	0.077020966	0.237108726
FAM149B1	0.228573881	6.322582117	13.66306338	0.000218714	0.003849022
SMCHD1	-0.170243608	6.323151633	3.439389819	0.063659199	0.210597532
LOC101748706	-0.526754214	6.323436279	26.55235694	2.56E-07	1.49E-05
HEATR3	0.0190236	6.323482843	0.07302898	0.786976586	0.895243672
RABGAP1L	-0.047372208	6.324082863	0.763706125	0.382171183	0.616769322
CRKL	0.025526178	6.324222792	0.175365296	0.675386865	0.828968937
HTATSF1	-0.065665754	6.324542403	0.787130838	0.374969109	0.609574856
PXMP4	0.125459944	6.324868389	5.637716043	0.017578092	0.091389414
DAD1	0.009674555	6.324869106	0.022121973	0.881763081	0.947093053
ZBTB5	0.016387146	6.325064423	0.051741491	0.820060192	0.91542069
WASHC2C	0.132821657	6.325268401	5.021168165	0.025039271	0.115250687
IQGAP2	0.011202959	6.325295212	0.012488044	0.911021698	0.959169623
TNKS	-0.157515808	6.325384468	3.231296632	0.072243468	0.227254112
CLDN1	-0.048165348	6.325508181	0.112615166	0.737186226	0.865900446
PEAK1	0.092338975	6.326330037	1.305456083	0.253219007	0.487812239
ZBTB46	0.425276108	6.326496008	27.37040617	1.68E-07	1.06E-05
WDFY1	-0.021980093	6.326580268	0.068363198	0.79373504	0.898830782
PDLIM7	0.134335572	6.326836275	3.620631193	0.057067218	0.195919273
MAN2A1	0.049464431	6.32781505	0.462739844	0.496346279	0.7079291
HDAC4	0.020184709	6.327864318	0.08185175	0.774803486	0.88883335
EPS15L1	0.023433604	6.32872254	0.183526331	0.668360119	0.824242356
HIST1H2B5L	-0.275735307	6.329305018	17.46245301	2.93E-05	0.000767826
PTTG1IP	-0.029906652	6.329330846	0.266127151	0.605941624	0.786214274
GAPVD1	-0.019441377	6.330477342	0.069368001	0.792259202	0.897777368
ERP44	0.060682505	6.330498306	1.160013168	0.281462809	0.517251567
NFASC	-0.015184894	6.330645113	0.017471861	0.894840997	0.951827123
C7H2ORF47	0.102863099	6.330720607	2.402152227	0.121168446	0.315179699
HDAC3	0.032501501	6.33114722	0.347066526	0.555778415	0.752912164
NR2C2	-0.162396772	6.331709305	5.64221864	0.017533007	0.091299248

BRPF3	-0.159089522	6.331717014	6.850577349	0.008861263	0.056939278
FADD	0.081901707	6.331720761	1.887080309	0.169531602	0.388043192
SMAD1	-0.033648406	6.331996373	0.251624371	0.615933623	0.792054659
ATXN3	0.018886143	6.332489265	0.065425697	0.798117263	0.901497773
CDK17	-0.041199681	6.333240469	0.599471253	0.438779838	0.664163576
NHLRC2	-0.021310248	6.333846043	0.12476039	0.723927737	0.857408332
XRN1	0.072694801	6.335130512	1.112227399	0.291598274	0.528791006
DDX10	0.006820184	6.33546468	0.008981699	0.924496064	0.966229608
SCAF4	-0.01432948	6.335682088	0.04961616	0.823732534	0.91690482
UBE2R2	0.167235375	6.335833926	3.348472931	0.06726739	0.21763368
RCAN2	0.567362732	6.336032825	36.37278808	1.63E-09	1.75E-07
FAM32A	0.044739679	6.336127574	0.547388924	0.459386545	0.681523442
NRAS	-0.165955693	6.336245399	9.453471288	0.002107499	0.020869886
CAMKK2	0.272338253	6.336498729	11.1213221	0.000853409	0.01069524
FANCD2	-0.024359292	6.336517382	0.13715748	0.711123376	0.849781439
UBFD1	-0.10582392	6.336532067	3.148201089	0.076010717	0.235104578
PDLIM5	-0.165065291	6.336540606	4.454997944	0.034799258	0.142543309
AHNAK2	0.544466164	6.336967552	4.14155961	0.041842601	0.160794023
EIF2D	-0.120486072	6.337395377	4.669989583	0.030694114	0.131323879
WDTC1	0.001140844	6.337493351	0.000415886	0.983729651	0.992657841
ID1	-0.121715658	6.338518485	1.561110003	0.211502769	0.439096399
NEDD4L	-0.152131356	6.3386871	4.953794902	0.026033463	0.118190772
ZW10	-0.042893588	6.339177863	0.473241444	0.49149972	0.704487482
LEF1	-0.029855286	6.340298039	0.133532899	0.714796768	0.852264423
KNSTRN	0.216299499	6.340510741	12.05802694	0.000515698	0.007413349
USP36	-0.004392245	6.34079917	0.003061057	0.955878098	0.981041173
USP48	0.157775794	6.341038445	7.813763259	0.005184982	0.03915506
MDFIC	0.149015491	6.34114118	4.132811525	0.042059418	0.161260659
USP42	-0.098000092	6.341387956	2.858548478	0.090889481	0.262937133
METAP1	0.050250673	6.342094822	0.675593801	0.411108125	0.641333351
LTBP2	-0.233658514	6.342095693	5.035076437	0.024838991	0.114470733
UBR2	-0.045475178	6.342292275	0.60633758	0.436170118	0.662345819

PFKL	-0.063935223	6.342658884	1.08848991	0.296806038	0.533705973
CACYBP	0.147886873	6.343186977	5.321641796	0.021062158	0.102781082
IFT122	0.123766996	6.343671568	5.639084803	0.017564373	0.091346954
NADK2	0.128303164	6.343719443	2.888217925	0.089229574	0.260150124
ARMC6	0.142798987	6.343854252	6.635798572	0.009994938	0.061922705
TSHZ1	0.062961408	6.343917168	1.173155487	0.27875382	0.514181965
EMILIN2	-0.083133689	6.344593895	1.03421206	0.309171425	0.54712396
TAZ	0.096640489	6.345381084	1.564030839	0.211076003	0.438848349
TMCC1	0.013406904	6.345554985	0.055520997	0.813720615	0.911019232
NPR2	-0.047470306	6.347316186	0.212661382	0.644689389	0.810541251
SPRED2	-0.14248794	6.347855868	4.573136374	0.032477054	0.136484247
GNA11	-0.148642269	6.348294738	7.447901073	0.006351047	0.045374963
WBP4	0.00883602	6.348659169	0.03069545	0.860921565	0.935018438
RPTOR	-0.076998154	6.348677736	1.22805702	0.267785235	0.503053957
AP3B1	0.025427674	6.349655601	0.105093936	0.745800287	0.871353573
AMOTL2	0.193541638	6.349916687	6.151501548	0.013130058	0.075254302
LDHA	0.644036417	6.349989602	9.526519719	0.002025238	0.020320301
PDIA5	-0.215841388	6.350045035	12.68816748	0.000367977	0.005744836
PIAS2	0.064216317	6.350327281	0.587976736	0.443202571	0.667970169
SMPD3	0.007712222	6.350629382	0.002754005	0.958147337	0.982056949
HOXA5	0.235062134	6.350826325	6.367698935	0.01162162	0.069112307
PHKB	0.431073929	6.350848764	19.73414752	8.90E-06	0.000297719
LAPTM4A	0.014052602	6.350992913	0.055952297	0.813011827	0.91050906
PITX2	0.165552068	6.351117867	1.29759981	0.254652112	0.48957828
TMEM184C	0.15349793	6.35125634	8.37204689	0.003810362	0.031356359
CTSD	-0.196331591	6.351618631	8.978251143	0.00273212	0.024701669
CENPT	0.115380413	6.351788824	2.05887914	0.151321442	0.362972935
DAB2IP	-0.105602459	6.351790318	1.791810065	0.18070578	0.402696859
SKIV2L	0.157226389	6.351828599	5.639386787	0.017561348	0.091346954
SDE2	0.13650627	6.351890251	5.964178163	0.014599404	0.080750652
NAMPT	0.019817633	6.353289421	0.117237482	0.732050193	0.862638637
ERC1	0.133226292	6.353290515	1.887409037	0.169494446	0.388012119

SSBP2	0.007726855	6.354763544	0.006706916	0.934729603	0.971447144
APPBP2	-0.079952993	6.354973613	1.971127942	0.160328188	0.375840233
ZDHHC7	-0.097237191	6.355087634	2.429050375	0.119105032	0.311591509
ITGA4	-0.176742378	6.355138427	6.362632917	0.011654847	0.06923484
PHACTR2	-0.193575422	6.355340236	8.943128584	0.002785153	0.025070946
WDR44	-0.030996358	6.355610953	0.331730973	0.564641602	0.758587325
NPEPL1	-0.026802087	6.355904476	0.25413963	0.614175202	0.791034201
AUP1	-0.03588501	6.3560603	0.371527634	0.542172752	0.742526229
SURF4	0.083800926	6.356227967	2.650586995	0.103511667	0.284879329
IL17RD	-0.08539804	6.357142329	0.559279054	0.454550868	0.677378995
LOC107055293	0.167863479	6.357364415	5.009429847	0.025209609	0.115702211
ZNF384	-0.125089085	6.357559086	4.168358744	0.041185678	0.15927139
MANF	0.142850682	6.358102139	4.45696823	0.034759138	0.142491683
PRKDC	-0.025775778	6.358352933	0.118106688	0.73109708	0.862328105
POLE2	0.015040165	6.358450967	0.053216778	0.817557492	0.91379626
RALGAPA1	0.000271442	6.359253442	1.82E-05	0.996597734	0.998539591
LOC416924	0.099088352	6.360248017	2.545300887	0.110622835	0.297628965
ATXN7L3	0.012267652	6.360337794	0.053919549	0.816378147	0.913160384
MACROD2	-0.096118079	6.360491931	1.23817793	0.2658225	0.500986054
ESD	0.224693874	6.360575053	12.13661341	0.000494415	0.007206019
SREBF1	-0.178841961	6.360632172	4.406478948	0.035802668	0.145357241
KHDRBS3	0.048849452	6.360743156	0.418049572	0.517911878	0.723449911
TPP1	-0.140771576	6.361259641	2.956871224	0.085513265	0.252715658
TRPM7	-0.026149574	6.361306242	0.15067619	0.697890002	0.842758423
DNA2	0.075117253	6.361507195	1.126609908	0.288499608	0.525139497
DLG3	-0.12345169	6.361654174	4.831262569	0.027948157	0.123992158
CCNL1	-0.044366803	6.361814319	0.448739698	0.50293406	0.713675402
BACH2	0.006754259	6.362689142	0.008150996	0.928062435	0.968067662
TSNAX	0.013609819	6.362741963	0.057403599	0.810647745	0.909184251
RPRD1A	0.041730616	6.362772017	0.45201092	0.501381544	0.712147891
RAB3GAP2	-0.029182023	6.363268118	0.268264753	0.604498173	0.785137502
TMEM169	-0.326901046	6.363408287	16.07951521	6.07E-05	0.001405676

YTHDF3	-0.028218621	6.364815537	0.233170383	0.629182937	0.80077386
LOC421419	0.099095877	6.364927727	3.167157785	0.075133089	0.233266979
VPS37B	-0.254036627	6.365085288	18.21781621	1.97E-05	0.000558017
ACTR8	0.114446301	6.365113229	3.696535017	0.054525589	0.190053395
CGGBP1	-0.094381064	6.3651857	2.96361003	0.085157616	0.252254934
GTF2H3	0.096414695	6.365219432	2.929128303	0.086994452	0.255669533
BIN3	0.23027673	6.365695636	11.69516903	0.000626626	0.008542773
MECOM	-0.152956569	6.366207431	2.650006453	0.103549476	0.284879329
EIF2AK4	-0.135181122	6.366385907	4.367513858	0.036630398	0.147395721
RNF217	-0.261697005	6.3670958	12.05748521	0.000515848	0.007413349
FAM65A	-0.224692854	6.367460308	6.68794916	0.009706681	0.060800697
ALDH3A2	-0.017948975	6.368681263	0.090875152	0.763067515	0.881102924
HSPA14	-0.057928753	6.369066211	0.512317672	0.474137271	0.692028455
C14H16orf62	0.059856021	6.369774708	0.829149477	0.362518582	0.59885988
TIMM44	0.103887744	6.369961095	2.620934435	0.105462319	0.288590399
RAB8A	-0.02182389	6.371543106	0.17362056	0.676913944	0.830079538
PIKFYVE	-0.235986448	6.371906078	9.893314375	0.001658803	0.017446238
PSMD9	0.180172025	6.372147719	6.660172857	0.009859135	0.061521452
GGA3	-0.122566772	6.37239197	2.760097703	0.096642335	0.272638555
MBD5	0.033146261	6.372628128	0.271179136	0.602541893	0.783718824
MRPL44	-0.053777782	6.37269348	0.69051474	0.405989354	0.637092773
ZNF629L	0.06419031	6.373152424	0.416817845	0.518529164	0.724065331
PPM1A	0.102165205	6.373248958	4.119585698	0.042389458	0.162100519
ETV3	-0.098172089	6.373474394	3.055648626	0.080456864	0.243233089
CNOT11	0.120020515	6.373636051	4.876092351	0.0272312	0.121730346
MARK2	0.054727894	6.373770976	1.066676748	0.301697298	0.539435949
FYN	-0.064889871	6.375200421	1.028190963	0.310583938	0.548336682
PDHA1	0.016342413	6.375228215	0.072402605	0.787870182	0.895481757
SCML2	0.056876896	6.375484658	0.990129552	0.319710713	0.557135546
TSPAN4	0.184832099	6.375623786	10.69401552	0.001074827	0.012633051
POMT2	-0.035155666	6.377867964	0.264639083	0.606950806	0.786666377
ERCC6L	0.126935701	6.378594208	3.149905327	0.075931368	0.234991662

RBPMS	-0.095183347	6.379209232	1.863057712	0.172272311	0.391322595
MPP1	-0.148963346	6.379555534	6.912531862	0.008559371	0.05573583
TMEM65	0.004430915	6.379584214	0.006459087	0.935944224	0.972046586
DPM1	0.110183936	6.379755086	2.926270083	0.087148623	0.255894109
PTGR1	0.044587836	6.379969494	0.318053421	0.572779823	0.764589274
FAF2	0.006011971	6.380522668	0.012182503	0.912112473	0.959684907
NCOA3	-0.078627596	6.381229996	0.595564379	0.440275439	0.66508146
ROCK2	0.004746941	6.381472209	0.00653406	0.935574343	0.971960086
ATAD2	0.079262423	6.381674502	1.228020445	0.26779236	0.503053957
SMARCAD1	0.0839735	6.382011308	2.756913557	0.096834894	0.272657543
DTNA	0.111563886	6.382752594	3.612627897	0.057342442	0.196541945
SEC11A	0.023433842	6.382905717	0.201900847	0.653190892	0.815005705
GOLGB1	-0.021255854	6.383304784	0.071643418	0.788958824	0.896197532
GPS1	-0.02076132	6.383508626	0.091546358	0.76222041	0.880756463
MED14	-0.07886736	6.383817056	1.527435389	0.216497756	0.445531587
TXNL1	0.110322474	6.384954144	1.232538955	0.266913839	0.502370343
ARFGAP2	0.027965661	6.385160124	0.343360358	0.557895952	0.753709187
TMEM132C	-0.05520759	6.38553336	0.428237561	0.51285502	0.72023806
SPC25	0.161641138	6.385554265	4.941468928	0.026219745	0.118659488
ARG2	0.378504357	6.385958966	33.32829533	7.78E-09	6.95E-07
CNTRL	-0.018008806	6.3862481	0.087055596	0.767954268	0.884302833
GLCE	0.128099267	6.386548049	5.445234138	0.019621785	0.098049338
PNRC1	-0.012088297	6.387213115	0.028092902	0.866890598	0.93840635
TMEM57	-0.150272542	6.387444216	10.30071197	0.001329789	0.014764964
AKAP2	0.034729487	6.387593435	0.187670534	0.664862525	0.822332596
TACC2	0.072525872	6.387609808	1.217022931	0.269945695	0.504595753
ARHGAP42	0.085274957	6.389186681	1.092242216	0.29597494	0.532793746
NSF	0.191062912	6.390855341	7.848500021	0.005086289	0.038622467
VEZF1	-0.046512541	6.391141827	0.561051383	0.453836934	0.676684193
CDK13	-0.103524136	6.391754164	2.52409801	0.112118875	0.299888832
C14orf2	0.087539777	6.391855621	1.324173907	0.249844337	0.484181905
ZDHHC9	0.117383747	6.39235294	3.858463061	0.049495649	0.178407601

ARPC3	0.075052153	6.392490804	1.881617921	0.170150374	0.388972549
FAM214A	-0.017895954	6.392515158	0.106027953	0.74471238	0.870453347
PHB	0.253267373	6.392675216	17.63992587	2.67E-05	0.000715351
PAN3	0.142676068	6.393928995	6.776956483	0.009234221	0.058593149
COMMD5	0.069593191	6.394073678	1.30740205	0.252865568	0.487396382
CD69L	-1.986714278	6.394111973	36.3385817	1.66E-09	1.77E-07
SURF6	0.027511317	6.39413201	0.175201691	0.67552968	0.829045412
LIN52	0.098405244	6.395556397	2.87775603	0.089811098	0.261518602
UFD1L	-0.057404204	6.396313941	1.234803844	0.266474835	0.501813162
UNC45A	0.033487176	6.396406798	0.496446542	0.481065656	0.696704901
ODF2	0.039121753	6.39696563	0.486063244	0.485688677	0.700316086
MAML2	-0.01696625	6.396998489	0.021427074	0.883621498	0.947979469
ZFYVE19	-0.349218013	6.397931631	15.03900054	0.000105312	0.002147351
PAWR	0.248170227	6.398256746	8.712002172	0.003161218	0.027406663
RABGAP1	-0.030123743	6.399152579	0.287309139	0.59195008	0.776980894
COMMD6	0.279143242	6.399387034	15.87071646	6.78E-05	0.001535046
KHDC4	-0.029081266	6.399959256	0.237225974	0.626216881	0.799103476
SLF2	-0.086396138	6.400232597	1.269989209	0.259768704	0.494318997
CHEK1	0.129027614	6.400823743	3.335094289	0.06781652	0.218936793
ELOC	0.049229976	6.401161567	0.610424886	0.434627878	0.661129662
ADAMTS1	-0.048316873	6.401992116	0.097873903	0.754396107	0.875726752
NEK4	-0.072155089	6.402699192	0.594914301	0.440525056	0.6651417
UBA3	0.163043712	6.403878025	7.531571264	0.006062701	0.043721152
UBXN7	-0.027858972	6.404162226	0.179611727	0.671707129	0.826371731
IDH3B	0.131100345	6.40421288	4.075178057	0.043517679	0.164546302
ZCCHC8	-0.00938919	6.404400636	0.020142353	0.887140227	0.949086044
ST6GAL2	-0.069176411	6.404601479	0.807113526	0.368975016	0.603767292
CCDC173	-0.26656659	6.406334051	12.48680816	0.000409836	0.006238528
ANAPC4	-0.0685369	6.406540761	0.900325997	0.342694314	0.579842456
SOS2	0.043235812	6.406703606	0.705100335	0.401075196	0.633652443
TSG101	0.057210406	6.407084093	1.107295626	0.292670576	0.529917949
ARL1	-0.133029061	6.407107142	6.659023249	0.009865498	0.061537818

BLM	0.038574722	6.407206758	0.193002304	0.660429485	0.819884957
NAXD	0.035916815	6.407501595	0.390617386	0.531975056	0.734393751
ANKRD44	-0.126348088	6.407606514	3.979106982	0.046067981	0.170233242
E2F4	-0.199682099	6.407790675	11.49866574	0.000696462	0.009234711
CNPY2	0.008091582	6.407971223	0.013665958	0.906938118	0.957377047
PALM	0.089238811	6.408891853	1.487565532	0.222594566	0.452140918
MELK	0.0540875	6.408929005	0.363462001	0.546589787	0.746525145
TIAM1	-0.277897522	6.409521474	9.703551921	0.001839122	0.018937498
MRPL20	0.146229532	6.410452821	8.275220499	0.004018978	0.032593537
ACAT2	-0.002071797	6.411775359	0.001163353	0.972791058	0.989202624
LOC101750998	0.506155318	6.412386774	9.455982551	0.002104615	0.020862316
LSG1	-0.077154363	6.412457071	1.810515071	0.178446455	0.399368012
PPID	0.255347499	6.412487678	17.33254372	3.14E-05	0.000814359
ZDHHC6	0.022997593	6.412739884	0.14325869	0.705062686	0.847186196
P4HA1	0.038838412	6.413082001	0.158459633	0.690578672	0.83869452
QTRT2	-0.042308559	6.414021047	0.600047326	0.43855997	0.664069883
CPT1A	-0.123167159	6.414085906	2.233049557	0.135086992	0.338231353
CHCHD5	-0.203315471	6.415197216	10.49299254	0.001198281	0.013652459
TBC1D9	-0.063397645	6.415245269	1.039388078	0.307963831	0.545966429
IPO8	0.017761391	6.415260005	0.09173229	0.761986351	0.880756463
WASL	0.138042521	6.415856711	5.360255056	0.020600761	0.101008794
OPHN1	-0.007548624	6.416139692	0.012047994	0.912597052	0.959845606
ZFP36L2	-0.012763428	6.416951517	0.020382372	0.886474322	0.948867168
RYK	0.142870053	6.418224066	9.000654384	0.00269883	0.024478493
LGMN	0.142565476	6.419230804	5.908730203	0.015066028	0.082253314
FADS1	0.315013632	6.419882568	36.76941457	1.33E-09	1.48E-07
DMAP1	-0.071193728	6.420446257	1.520297746	0.217574441	0.446496692
TCF25	-0.015094785	6.420449068	0.049927372	0.823189701	0.916737351
SCOC	0.186295384	6.420497801	8.483650479	0.003583523	0.030100496
COMMD2	-0.035120085	6.420960974	0.397249578	0.528513118	0.732173853
TIPRL	0.07892283	6.42101797	1.974162672	0.16000671	0.375379301
PCID2	-0.042318192	6.42175332	0.510539034	0.474905605	0.692302165

CWC22	-0.088032223	6.421919581	2.36498496	0.124085109	0.319583733
RFC3	0.051939052	6.422265609	0.511934324	0.474302699	0.69207548
TOP3B	0.068734736	6.42280105	1.159307895	0.281609123	0.517377633
WDHD1	0.21617604	6.423266647	12.15839828	0.000488673	0.007147661
KLHL21	-0.011113183	6.423704941	0.038516252	0.844410129	0.926750395
CRY1	0.107516279	6.424100926	2.804395386	0.094006278	0.268624477
RTFDC1	0.244911643	6.425547291	17.37972355	3.06E-05	0.000796906
SLX4	-0.410450847	6.425689531	23.9059849	1.01E-06	4.82E-05
CFDP1	0.103425928	6.425930755	2.58696138	0.107746865	0.292425313
PRKG1	0.236768221	6.426271079	8.400065453	0.003752075	0.031051952
SESN3	0.112789186	6.426727957	1.015498738	0.313589106	0.55158832
ASAP1	0.046924022	6.426786507	0.444439122	0.504987626	0.715355663
SUZ12	-0.022985317	6.426903127	0.092974961	0.760428638	0.879947485
MAPK14	-0.009459207	6.426952253	0.033505297	0.854763085	0.932421351
AGPAT3	0.052926902	6.427225576	0.787159844	0.37496031	0.609574856
DLG5	-0.015024882	6.427486045	0.051311856	0.820796068	0.915860806
PHLPP2	-0.082314915	6.427491177	1.11143221	0.29177083	0.52883963
SPOCK3	-0.270343124	6.427845739	12.81233267	0.000344342	0.00545803
ATRIP	-0.054982464	6.429262427	0.601939324	0.437839041	0.66377754
CENPH	0.258186544	6.429284411	7.870243137	0.00502549	0.038292634
JUN	0.357649336	6.429529651	9.390297052	0.002181369	0.021373879
TRAIP	-0.102779256	6.429542762	2.192036254	0.138725812	0.344086088
CCDC6	-0.037230326	6.4302518	0.435049552	0.509521555	0.717892027
PARD3	-0.02942626	6.430991302	0.11423203	0.735376532	0.864745668
KLHL36	-0.104809454	6.431020334	2.136243652	0.143853754	0.352353902
TBC1D5	0.045187127	6.431182667	0.473698197	0.491290727	0.704487482
JMJD4	-0.413839717	6.431342865	43.81554331	3.61E-11	6.12E-09
LIN7C	0.070281594	6.431513721	1.340361308	0.246970261	0.480649987
COTL1	0.035358474	6.4315999	0.186548487	0.665804943	0.823156351
NISCH	0.091358996	6.431681849	2.935887377	0.086631046	0.254978044
VRK1	-0.015337031	6.432258851	0.051330824	0.820763513	0.915860806
ZCCHC14	-0.078617987	6.432714225	1.115781421	0.29082864	0.527918949

EXOC5	0.092232165	6.432768455	3.03838894	0.081316583	0.24464664
MIS18BP1	0.093419141	6.433648557	2.732862818	0.098302931	0.275170223
YTHDF2	-0.073545731	6.433675249	1.539022696	0.214763334	0.443849617
TUBB2A	-0.048936609	6.433874665	0.262447724	0.608443501	0.787473436
SMG8	0.002452582	6.43399985	0.001762229	0.966515508	0.986147777
PITRM1	0.119645001	6.434030383	4.989130418	0.025507024	0.116705787
ARHGEF10L	0.054624937	6.434339524	0.775399767	0.378551757	0.613271837
SLC25A13	-0.030348448	6.434368102	0.32308028	0.569762363	0.762434642
SMTN	-0.100010872	6.434902238	1.753241162	0.185469791	0.4080633
MAP2K2	-0.070777587	6.434939734	1.512742894	0.218721022	0.448033663
OGFR	0.01541103	6.435882389	0.074191591	0.785328823	0.894420389
UGP2	0.052944565	6.436049721	0.860541744	0.353587133	0.590268466
RBM4B	0.161374645	6.436098192	7.936158907	0.004845658	0.03741685
MTO1	0.021933077	6.436576178	0.148889818	0.699598554	0.843779396
GGH	-0.10025307	6.436588002	2.436558053	0.118536066	0.310692768
LRPAP1	0.073823619	6.437137093	1.676296395	0.195417302	0.420319684
TPD52	-0.031191821	6.437642244	0.147755454	0.700689621	0.844553744
UTP15	0.092050252	6.437722689	1.103611032	0.293474997	0.530673745
CKAP2L	-0.040808431	6.438065613	0.358022498	0.549606546	0.748347183
NOL11	-0.056995073	6.438271344	0.504728325	0.477429872	0.693818648
PPP6R3	-0.137526478	6.438938465	6.407549166	0.011363616	0.06794633
MEF2A	-0.023769327	6.438972412	0.183671102	0.668237153	0.824214253
POLR3B	0.079443246	6.439128845	1.69356897	0.193131168	0.417330711
OSBPL9	0.093730228	6.439413008	3.34973303	0.067215914	0.21763368
KIAA1462	0.09879995	6.439745156	1.223219794	0.268729691	0.503695986
CTNNA2	-0.052128604	6.439765821	0.543647561	0.460924978	0.682675113
UBL5	0.087798048	6.440715648	1.798003139	0.179954093	0.401619945
FAM103A1	0.080053001	6.441060966	1.324719891	0.249746731	0.48404976
ARL5B	-0.05881372	6.441440701	1.004515739	0.316220293	0.553730437
PYGL	-0.074415017	6.441698039	1.379441004	0.240196388	0.472831035
LOC425117	-0.283475149	6.441772509	15.50163243	8.24E-05	0.001787157
ALDH18A1	0.234901948	6.441833064	10.80582643	0.001011811	0.012126262

RAD50	0.326469365	6.442369286	18.51562123	1.69E-05	0.000489921
USMG5	0.114178672	6.442964027	2.828671592	0.092594922	0.265995181
PBK	0.121350936	6.443646646	5.232932904	0.02216313	0.106170104
VPS36	0.094165527	6.443788858	3.092989646	0.078630219	0.23964813
SH3BP4	0.165467665	6.444103727	6.109953364	0.013442268	0.076484271
PARL	0.043681846	6.44473076	0.448856519	0.502878476	0.71365807
ZEB2	0.211623882	6.444750641	6.616560219	0.010103479	0.062383767
SDC2	-0.243743434	6.445696405	10.71096848	0.001065023	0.012562687
CCNJ	0.029359496	6.445933409	0.139251802	0.709025958	0.849542309
MRPL28	-0.133233693	6.446411242	3.604539649	0.05762202	0.197247834
RAI1	-0.129905821	6.446426113	3.117286916	0.077465664	0.237687384
LOC112529925	-0.090478505	6.446492117	1.917896518	0.166088732	0.38379302
CBL	-0.231281161	6.446545071	14.98449678	0.000108398	0.002177888
FAM49B	0.061199219	6.446580393	1.493303524	0.221704606	0.451179047
DAAM2	0.094678999	6.447031049	1.476312288	0.224352392	0.453917322
HPF1	0.209571086	6.447031304	14.37191197	0.000150024	0.002834257
HDAC8	0.075564186	6.447965928	1.783776158	0.181686319	0.403788274
C2CD5	-0.119472509	6.448167821	3.93346549	0.047334307	0.173124253
ATPIF1	0.080817235	6.448947231	1.111815946	0.291687543	0.528815967
CTSC	0.004596586	6.44925884	0.007834939	0.929467212	0.968796033
VAV2	-0.046720555	6.449283846	0.747952906	0.387125128	0.621284893
FOXN3	0.06388341	6.449824178	0.517433142	0.471938689	0.690534513
COG4	-0.160270593	6.450225555	4.317563517	0.037720896	0.150108184
MSANTD3	-0.094763035	6.450296936	2.813200865	0.093491665	0.267735006
DLAT	0.098383027	6.450319259	3.592085965	0.058055327	0.198171773
PPAT	0.075763336	6.450723555	1.203574737	0.272608372	0.507375894
PIGS	0.004554176	6.450765029	0.004484133	0.946610627	0.977439625
LOC107050871	-0.139684783	6.450894839	2.294925864	0.129797408	0.32942409
CARS	-0.031613305	6.451251296	0.213642577	0.643927248	0.809954354
LOC107056441	0.035409925	6.452837759	0.272689455	0.601533343	0.783087908
LOC112530170	-0.024299226	6.453036548	0.163487286	0.685965943	0.836116266
NET1	-0.018370893	6.453159909	0.087204595	0.767761476	0.884179034

BYSL	0.079557584	6.454511376	1.346479152	0.245894581	0.479484146
SLC7A3	0.074140766	6.454646342	1.207106208	0.271905988	0.506705893
LOC107050352	0.091608927	6.455715164	1.789340953	0.181006482	0.403134632
CXXC5	0.133330536	6.456993266	4.163082933	0.041314141	0.159620614
EDEM3	-0.001619516	6.457294742	0.00096556	0.975210954	0.990018742
ZNF518B	0.036861374	6.457559168	0.451196628	0.501767242	0.712634211
TUBGCP4	-0.023456883	6.457805175	0.186829804	0.665568347	0.823081854
SEMA6A	0.105289963	6.458044634	1.393562921	0.237804328	0.470490587
MPI	0.15687663	6.458094218	6.288569032	0.012151912	0.071210725
RNF14	0.183959804	6.458197516	8.093310925	0.004442892	0.035114215
RAB30	-0.025099592	6.45844742	0.162172188	0.68716446	0.837081076
MYL12A	0.095992972	6.459304265	1.634022487	0.201147584	0.427744054
MYDGF	0.138442919	6.459316074	6.946756443	0.008397135	0.055071683
OAZ2	0.198158998	6.459858374	13.09922386	0.000295418	0.004870847
JAG1	0.038462526	6.459861184	0.183700728	0.668211995	0.824214253
FRYL	-0.148200049	6.460293817	4.215658239	0.040052523	0.15680554
WSB2	-0.106003105	6.460535979	2.702071913	0.100217932	0.278467758
SLC35E3	0.138805644	6.460920905	4.92507149	0.026469707	0.119566735
USP54	-0.017924877	6.461589034	0.06995888	0.791396659	0.897586603
VGLL4	0.044218318	6.461770816	0.386488055	0.53415123	0.73595064
GSE1	-0.059929098	6.462363465	0.443937163	0.50522825	0.715440088
DENND2A	0.042894727	6.462769298	0.585342854	0.44422568	0.669144413
PARK7	0.042734955	6.462820512	0.676740023	0.410711551	0.640927017
CAMSAP1	-0.221982482	6.463143831	10.36789509	0.001282257	0.014363194
CTSV	-0.030654433	6.463157927	0.220656047	0.638540497	0.806602965
CA9	0.225568045	6.463664315	5.196521222	0.022632138	0.10750919
RAPH1	0.016443202	6.463942107	0.080571147	0.776524897	0.889376916
FAM234A	-0.037955925	6.464059911	0.497667132	0.480526961	0.696466157
GPR107	-0.105338132	6.464353944	4.287833364	0.038386053	0.151618459
NDUFB5	-0.000723179	6.464801168	0.000181241	0.989258754	0.995057477
PRORSD1P	-0.00227205	6.464903855	0.001844219	0.965745875	0.985815656
TCEA1	0.115318246	6.465004061	3.040730246	0.081199382	0.244534238

USP4	0.066697674	6.465435657	1.437378547	0.23056402	0.461942159
ANAPC2	0.155235251	6.466083358	6.921309124	0.00851746	0.055506853
WDR90	0.429071625	6.466283023	39.52832892	3.23E-10	4.12E-08
MTMR3	-0.044406353	6.466704291	0.64044797	0.423548627	0.652879875
SLC30A5	0.074860288	6.466850503	0.71683353	0.397184537	0.629797549
IDE	-0.095689329	6.467330037	1.880694214	0.170255267	0.389158274
CKS1B	0.154175792	6.467503015	4.552307215	0.03287442	0.137716032
RAD51	0.00360622	6.467551943	0.001819111	0.965979709	0.985879339
BIVM	0.100728472	6.467589531	4.519769433	0.033505346	0.139436135
CAPN11	0.035877055	6.467681999	0.244868621	0.620711643	0.79534419
BCL2L1	-0.024513489	6.468209307	0.164393055	0.685143726	0.835563601
NGFR	0.088531532	6.468562023	0.320464781	0.571328462	0.763271077
PRSS23	-0.051544006	6.469649349	0.237212424	0.626226739	0.799103476
DAAM1	-0.097761299	6.469725938	2.868737054	0.090315718	0.262106726
APOOL	0.302194991	6.469951829	20.97974072	4.64E-06	0.000173194
SEMA3C	-0.214224973	6.470115092	6.111506456	0.013430461	0.076469976
NELFCD	0.159200664	6.470267308	6.61497087	0.010112499	0.062416045
MDM2	-0.225099572	6.470314317	8.871803607	0.002896089	0.025801375
PMPCA	-0.08755415	6.471579239	2.777761839	0.095581656	0.270889262
TSC1	-0.068766095	6.472071937	1.167728859	0.279868391	0.515184514
TAF5	-0.031156204	6.472227066	0.340874291	0.559324999	0.754710362
DCAF10	0.049274322	6.472811276	0.494813776	0.481787811	0.697173002
NDUFB4	0.167527431	6.473586593	7.400633242	0.006520092	0.046066172
BDP1L	0.014915954	6.473811994	0.031543592	0.859033073	0.933987549
XBP1	0.286036803	6.474200774	21.02511133	4.53E-06	0.000169911
ZNF516	0.071297941	6.474327228	1.182152444	0.276918236	0.512102672
C1H21ORF33	-0.077676052	6.474369449	1.750193398	0.185852421	0.408503379
NUP37	0.102009872	6.475681103	2.4950664	0.114203559	0.303152969
MOB4	-0.033664687	6.475745347	0.424403726	0.51474781	0.721735682
ENPP2	0.124809622	6.476089227	2.379446908	0.122941067	0.317681417
FNIP2	0.033521588	6.476520498	0.401098355	0.526522565	0.730639974
SKAP2	0.194777189	6.476869133	5.708401611	0.016883906	0.089275279

ADSL	-0.056835629	6.477145357	0.822784031	0.364367436	0.600046653
DR1	-0.025073039	6.477486793	0.190061813	0.662865176	0.821016009
PLVAP	-0.025908433	6.478238618	0.103484632	0.747687348	0.872702382
PSMF1	-0.045289509	6.478529854	0.679495336	0.409760558	0.640132877
CTSA	0.062882988	6.478743783	0.956546682	0.328058475	0.565717803
DESI1	0.028591903	6.479713841	0.228791653	0.63242125	0.803057713
LOC107050411	0.042186698	6.479728161	0.443812718	0.505287936	0.715440088
RTKN2	0.041072456	6.480141279	0.204906175	0.65078953	0.813672344
TMEM214	0.054856231	6.480279245	1.082820784	0.298067383	0.534894621
CTNNBL1	0.152762336	6.480734655	7.625653932	0.00575438	0.042072287
ETFDH	-0.067374196	6.480910294	0.884518597	0.346967052	0.58353872
COPS7B	0.005071573	6.481930511	0.005530709	0.940716982	0.974411302
BIRC5	0.109560156	6.484335329	2.452397318	0.117345529	0.308947308
ZRANB1	0.014686802	6.485493259	0.077988066	0.780042665	0.891423158
KDM2A	-0.130143764	6.485990669	4.039138609	0.044456508	0.166522158
PLIN4	0.135869169	6.486129145	4.642720947	0.03118552	0.132873572
ATP6V0D1	-0.146077892	6.48680645	3.219547685	0.072763734	0.228323272
SASS6	-0.142247875	6.487190917	4.953549095	0.026037165	0.118190772
AP3S1	0.178838866	6.487832051	3.323500591	0.068296263	0.219752642
FUNDC2	0.225840024	6.487856068	21.21341461	4.11E-06	0.000156143
TMEM2	0.234742938	6.489935764	6.879861172	0.008717233	0.056362309
NDUFA9	0.003699198	6.489989594	0.004326498	0.947556066	0.977802274
HRAS	0.157790596	6.489994044	5.674367626	0.017214544	0.090294756
KIAA1524	0.056796084	6.490919282	0.759038942	0.383629451	0.618392982
SRM	0.207679705	6.491335569	14.51409115	0.000139115	0.002667993
SLC25A11	0.284699507	6.491623911	15.22738367	9.53E-05	0.001995353
NEDD1	-0.02780514	6.492124468	0.224954346	0.63529061	0.804626461
ACO1	0.111030646	6.492475244	2.316194179	0.12803294	0.326632872
RBM18	0.068330226	6.492765481	1.740910125	0.187023549	0.410088099
CHMP6	0.066667961	6.49283607	0.936733104	0.333119422	0.570880873
LOC107051263	0.098928046	6.493817367	2.080915922	0.149150656	0.360101426
WDR81	0.017202979	6.494290045	0.106524358	0.744136341	0.870074943

BAZ1A	-0.152328003	6.495700648	9.248810841	0.002356484	0.022473132
TTC3	0.003367659	6.498633825	0.002975856	0.956495851	0.981184588
LOC100859273	0.059845838	6.499463992	0.540176591	0.462359551	0.683620118
PTPN13	0.144831902	6.499762749	4.233931194	0.039623564	0.155572834
MAP2K1	0.154319257	6.500211473	7.447256356	0.006353322	0.045374963
SNAP23	0.074721993	6.500414689	1.905469864	0.167467343	0.385409109
DLC1	0.001768049	6.500573999	0.000329839	0.985510038	0.993236215
MPHOSPH10	0.042206647	6.501261311	0.395766354	0.529283828	0.73304986
NAV2	-0.102366154	6.501272768	2.026738539	0.154551963	0.367433368
RAB5C	-0.119652955	6.501492525	5.62141469	0.017742322	0.09189484
FARP2	-0.061614325	6.502728299	1.522742055	0.21720501	0.446236333
SNED1	-0.154043064	6.503402048	1.908954958	0.16707939	0.385054812
EPC1	-0.055110983	6.503466317	0.59500147	0.440491572	0.6651417
NFS1	-0.032272412	6.503527184	0.349453288	0.554422774	0.752290487
FBXO9	-0.014053568	6.503553587	0.07729019	0.781003812	0.891964029
CROT	-0.074376109	6.503554555	2.229504292	0.135397281	0.33839038
MRTO4	0.289656885	6.503786673	19.66267393	9.24E-06	0.000304051
FAM98A	0.153954746	6.504191099	8.564454773	0.003427896	0.029070953
CDCA3	0.099741957	6.504293311	2.244020461	0.134131823	0.336813543
CEP295	0.199001616	6.504384298	9.367029115	0.002209233	0.021548861
NSUN2	0.049326882	6.504660073	0.33112553	0.564997085	0.758879034
ENOX2	0.118916221	6.505106608	4.368222007	0.036615178	0.147383256
UTP4	0.002435165	6.505253092	0.000945632	0.975468015	0.990018742
RAB5A	-0.102226107	6.505879056	2.564915444	0.109258399	0.295071008
RPF2	0.134551672	6.506319217	3.995379432	0.045625179	0.169262559
FOXP2	-0.119334659	6.506354883	4.01420793	0.045118411	0.168045147
GNAS	0.119337576	6.507012737	4.488150633	0.034130581	0.140933177
CRNKL1	0.125761042	6.50717969	6.325290826	0.011902797	0.070021151
YIPF5	0.122072421	6.507309116	5.259622398	0.021825754	0.105137513
CHSY1	0.119654904	6.507724873	4.429264286	0.035327733	0.143987371
MGAT4B	0.02272585	6.509100962	0.110060639	0.740075109	0.867860967
FAM63A	0.237139441	6.509409632	13.60696251	0.000225348	0.003936417

RHOT2	0.067630874	6.509726	1.063407742	0.302439243	0.540234232
NDST2	-0.025715666	6.510157295	0.137733917	0.710544279	0.849781439
LOC101748353	-0.002091613	6.510489515	0.001439734	0.969732465	0.987658702
DENND4A	-0.198626575	6.510670562	8.138553842	0.004333383	0.034360863
UTP18	0.114878906	6.511394607	2.634031685	0.104595814	0.286901837
USF1	0.067451365	6.514446447	1.319498534	0.250682068	0.48498669
ZEB1	-0.154579513	6.514955274	4.182483783	0.040843801	0.158588622
PLEKHJ1	-0.032696185	6.515399352	0.371023901	0.542446682	0.742839561
LARP4B	0.035397192	6.515759142	0.206645718	0.649409256	0.812811235
BCL9L	-0.123427211	6.51600763	2.068958733	0.150324124	0.361529299
PRIM1	0.112336148	6.516109269	2.801846413	0.09415582	0.268763511
H2A2BL	0.248589953	6.517453094	10.06323653	0.001512573	0.016278211
STX16	-0.079610682	6.517667446	1.815425862	0.177858715	0.398728223
ANAPC16	0.034364458	6.518173782	0.218118898	0.640476976	0.807749496
SH3GLB1	-0.007378073	6.518235795	0.014354351	0.904633941	0.956421967
DYNLRB1	-0.022039158	6.519716347	0.123147795	0.725645215	0.858657559
DIS3	-0.054173728	6.520329453	0.549955409	0.458335915	0.680869279
ST8SIA2	0.121765968	6.520415757	2.876637525	0.089873513	0.261607758
MAP6	-0.090319872	6.521035104	0.473044286	0.491589978	0.704487482
UQCR10	-0.05176807	6.521431548	0.346190489	0.556277564	0.753031308
UQCRB	0.125282497	6.521765477	3.808695584	0.050987143	0.181989717
PICALM	0.037632809	6.522126239	0.48015504	0.488352098	0.702064807
MBNL3	-0.055320286	6.522768956	0.572781991	0.44915554	0.672568424
MYCN	0.248077912	6.52393673	4.887690021	0.027048853	0.121226639
PEPD	0.137289682	6.524088775	3.110952982	0.077767446	0.238215389
TAF1D	0.059553536	6.524267256	0.615517044	0.432718075	0.659478488
MAP4K3	0.043285697	6.524385236	0.714678155	0.397895139	0.630220859
CSTF3	-0.121591337	6.524755867	6.063115111	0.013803399	0.077865936
RBSN	-0.083762306	6.524816952	2.5504397	0.110263565	0.297002285
CCDC12	0.045842584	6.524932522	0.685035456	0.407858157	0.638408775
KANK4	0.090267872	6.52566725	1.99522877	0.157795238	0.372474628
ZNF512B	-0.254887263	6.525673471	25.41570942	4.62E-07	2.51E-05

MARK1	-0.088731372	6.525965544	2.229595807	0.135389261	0.33839038
SNX17	0.069234871	6.526064566	1.429175414	0.231899058	0.4635017
GCLM	0.24570096	6.526183089	4.888306886	0.02703919	0.121226639
RC3H2	-0.192451121	6.526244198	5.634907543	0.017606274	0.091483473
RFC5	0.080762401	6.526811954	2.055483658	0.15165909	0.363412018
PRDM11	-0.124594944	6.526817728	2.519316968	0.112459286	0.300257264
ADPGK	-0.047579292	6.527254064	0.920877005	0.337244805	0.574527721
GARNL3	0.118689962	6.527299917	4.103910035	0.042784161	0.163008754
MYL4	0.046418647	6.52808343	0.127719617	0.720808234	0.855792151
CCDC124	-0.100205243	6.529152214	4.124227122	0.042273326	0.161851083
STK26	-0.165472683	6.529221244	6.812238571	0.00905352	0.057805724
SPAST	0.00320527	6.529662531	0.004424372	0.94696706	0.977487125
CWC15	0.091607581	6.529949665	2.756579069	0.096855146	0.272657543
TMEM115	-0.071952205	6.530413401	1.563836617	0.211104349	0.438848349
WBP1	0.01122932	6.530429015	0.022538549	0.880663281	0.946402448
LAP3	-0.072451851	6.530560189	0.810540971	0.367960361	0.60268481
LOC101751286	-0.088614283	6.530575886	1.83422257	0.17562961	0.395346816
AKR1A1	0.188501022	6.530817644	10.51714317	0.001182722	0.013524453
UCHL3	0.064771885	6.53084675	0.810840395	0.367871904	0.60268142
PAIP1	0.001642731	6.530986806	0.000377392	0.984500815	0.992968171
IQSEC1	0.066706647	6.532349305	1.043317714	0.307051113	0.545040568
RAP2A	0.109127334	6.532364783	5.051800782	0.024600359	0.11381102
EPB41L2	0.187777115	6.532535161	8.455340463	0.003639731	0.030463772
PAX3	0.07168882	6.533559868	0.588801407	0.442882982	0.667610797
PPRC1	-0.039438203	6.533952365	0.332268422	0.564326402	0.758411544
ARHGAP18	-0.004652537	6.534728619	0.005444942	0.941177606	0.974643014
PLEKHM2	-0.047232643	6.534890653	0.921110124	0.337183659	0.57448303
SPTBB4L	-0.056135866	6.534973285	0.302998905	0.582008456	0.770499657
TIMM50	0.035323041	6.535684732	0.253063602	0.614926114	0.791505727
BTAF1	-0.125244285	6.536146356	4.055728014	0.044021733	0.165572028
AFF4	-0.079340501	6.536326248	1.255727216	0.262461423	0.497588898
AIMP1	0.032666717	6.536637902	0.321222459	0.570873916	0.763014502

DIP2A	0.094543719	6.536793053	2.240952169	0.134398197	0.337173706
KIAA0100	0.020835044	6.538420957	0.159564275	0.689557995	0.83825922
HGS	0.014392811	6.539478196	0.047685282	0.82714115	0.918022907
WNK2	0.160927941	6.539855621	3.279780473	0.07013831	0.223628159
DCAF1	-0.001585991	6.540191354	0.001015898	0.974573205	0.989871494
MORC2	-0.344572328	6.540793874	20.89355823	4.86E-06	0.000180754
YY2	0.263960766	6.541082171	19.79912594	8.60E-06	0.000290655
MICAL3	0.027046647	6.541389373	0.145975857	0.702411023	0.845761261
NEMF	0.02178856	6.541614082	0.146139726	0.702252012	0.845693564
RNASEH2B	-0.071482103	6.542039008	0.616663636	0.432289809	0.659313078
LOC112531093	0.002641935	6.542359599	0.00092457	0.975742668	0.990018742
CNOT8	-0.015702973	6.542930561	0.093656723	0.759578865	0.879458924
RASSF8	-0.078463048	6.543332863	1.741586267	0.186937962	0.409977897
TTC27	-0.027378953	6.543545183	0.183883406	0.668056927	0.824166458
EPS15	-0.001903879	6.54378541	0.001338821	0.970811989	0.988350633
SLC39A10	0.128137285	6.54395783	4.159982097	0.041389841	0.159710841
RAPGEF2	-0.09055642	6.544068722	2.02633365	0.154593155	0.367446246
FAM174B	0.084960426	6.544139655	1.174015164	0.278577766	0.514073919
SGF29	-0.186157863	6.544281188	10.44529942	0.00122962	0.013915676
TGFBI	0.122288223	6.544865038	1.474075507	0.22470377	0.454294204
DICER1	-0.264113618	6.544901468	5.128896381	0.023530685	0.110580422
ZNF598	-0.12306785	6.545065391	3.814325757	0.050816053	0.181580292
SOX10	-0.061254911	6.545668026	0.183384686	0.668480487	0.824270542
DPAGT1	0.092977586	6.54628712	3.998334791	0.04554524	0.169060891
SLC31A1	0.140122646	6.546736958	5.209789994	0.022460047	0.107109491
TAF3	-0.000365504	6.547663725	4.15E-05	0.994860689	0.997594138
LZIC	0.002137916	6.547829486	0.001752228	0.966610604	0.986147777
STAM	0.061701024	6.548499086	1.000203705	0.317261222	0.555139665
NFRKB	-0.065613086	6.54966875	1.463672895	0.226346628	0.456254766
UBE2H	0.023970301	6.549925999	0.164525308	0.685023894	0.835563601
ANO5	-0.219067982	6.550051773	14.17071897	0.000166948	0.003093622
SMAP2	-0.135494303	6.550154305	7.251596132	0.007083802	0.048629103

LEO1	-0.047643069	6.550734144	0.828979637	0.362567744	0.59888097
HAPLN1	0.072580008	6.550906529	0.580710287	0.446034046	0.670518018
DNAJC11	0.012938424	6.553090509	0.050036604	0.822999592	0.916703329
PLEKHG1	0.044627571	6.55341793	0.268582138	0.604284476	0.785137502
PLXNC1	0.150454706	6.553482921	4.987388441	0.025532715	0.116705787
08/set	0.029954773	6.553645601	0.324059761	0.569178031	0.762061813
CUL2	0.109474978	6.553891313	4.192890101	0.040593838	0.157912907
TUBB6	0.344465728	6.553923476	10.59775112	0.001132253	0.013166942
SRPK2	-0.00664578	6.554022902	0.010454746	0.918559471	0.962372833
ESYT1	0.150186849	6.554028196	5.219431624	0.02233585	0.106810932
BCOR	-0.082366078	6.554792832	2.026821848	0.154543489	0.367433368
UHRF1BP1	0.063691188	6.554825957	0.455709151	0.499636165	0.710527447
RNF151	0.033075235	6.55547962	0.249027148	0.617760927	0.793398483
MMS19	0.164233757	6.555873124	6.244335419	0.012459112	0.072571572
TBC1D12	-0.106517331	6.556525183	3.67849843	0.055118468	0.191264106
CAPN15	-0.021467662	6.557254081	0.183068325	0.668749523	0.824382304
CELSR3	-0.26186777	6.557421028	12.29270967	0.000454731	0.006739878
TOR1AIP1	-0.023667157	6.558265922	0.22339103	0.636468182	0.805250188
FBXO3	0.021315275	6.558373524	0.184588115	0.66745958	0.824120011
HDAC7	-0.009693193	6.558841023	0.026625467	0.870382129	0.940243379
CLCN3	-0.167281676	6.559078614	10.22951218	0.001382112	0.015182013
CD151	0.087782296	6.559547305	2.436293616	0.118556055	0.310692768
DHX33	0.055100514	6.559695966	0.737127554	0.390582582	0.624044701
WDR11	0.033245128	6.55975395	0.258200973	0.611358813	0.788982376
FREM1	-0.056151077	6.559916012	0.211039844	0.645953603	0.811077187
PTDSS2	0.021205529	6.560043809	0.110958005	0.739056085	0.867283451
FBF1	0.114915944	6.560242405	1.87067562	0.17139772	0.390089808
KIAA1217	-0.062095407	6.561302588	0.671394567	0.41256582	0.642936885
STAG1	0.118814729	6.561638773	3.919441201	0.047730743	0.174032656
SCAMP4	0.114871186	6.56172858	5.202575288	0.022553451	0.107321292
LOC100859084	0.00126214	6.561766877	0.00030482	0.986070366	0.993435858
ZNF292	0.012424399	6.562056067	0.034359771	0.852943631	0.931394748

PACS1	0.052097502	6.562725126	0.882218239	0.347594841	0.584088143
ADNP2	-0.132274028	6.563926608	3.523978097	0.060487102	0.203499338
GLE1	-0.044292149	6.563961646	0.523955192	0.469159369	0.688269654
ASXL1	-0.090799382	6.564077291	2.253349821	0.133325512	0.335198058
SUN1	-0.008116986	6.566185363	0.026044923	0.871790658	0.941046659
USP16	0.256138963	6.566190406	17.82950934	2.42E-05	0.000657562
SDHC	-0.037094902	6.566211741	0.409854252	0.522043491	0.726876429
BCL7A	0.157745953	6.566477151	8.701531825	0.003179428	0.027521031
MCM10	-0.003958729	6.566826254	0.004741761	0.945100705	0.976598148
FOXO4	-0.068192937	6.567749853	0.961218794	0.326879995	0.564408217
RAB2A	-0.089737675	6.568722697	2.415569392	0.120134282	0.313099911
CPSF3	0.224637225	6.569826098	14.39729589	0.000148015	0.00280597
PCMT1	0.069146037	6.570427724	1.967148851	0.160750821	0.376111802
BRWD3	-0.153664862	6.571298901	6.57196999	0.010359722	0.063571467
LOC107050363	0.137768004	6.5713985	3.302673304	0.069167232	0.221567349
RBMS3	0.13155722	6.573219495	3.959040846	0.04662026	0.171531198
EIF2B2	-0.010621027	6.573237399	0.041594255	0.838395154	0.923661189
FARSA	0.184650899	6.573596162	8.351676605	0.003853316	0.031608333
CELSR1	-0.004412528	6.573885849	0.002744771	0.958217491	0.982056949
GMFB	0.038194057	6.574223268	0.389475231	0.532575371	0.734892189
OPA1	-0.103384119	6.575090148	3.043154404	0.081078226	0.244437927
ZC4H2	0.169906191	6.575205783	6.571935371	0.010359923	0.063571467
CAMSAP2	-0.109813137	6.575630735	2.88566346	0.089371186	0.260421971
PPP3CB	-0.063906506	6.576460683	1.268006644	0.260140966	0.494754922
KDM4B	0.12484689	6.576872209	3.040179404	0.08122694	0.244534238
RELN	0.11054059	6.577263431	0.393774736	0.530321884	0.733379515
DENND5B	0.003132786	6.577741414	0.001258994	0.971695155	0.988743849
RAVER2	-0.059481386	6.578030102	1.182939961	0.27675829	0.511921949
GNAI1	-0.053114321	6.578248121	0.932613379	0.33418476	0.571985858
MFAP1	-0.022589285	6.579176039	0.183844887	0.668089616	0.824166458
RBM12B	0.21355367	6.579513184	15.60807757	7.79E-05	0.001716453
SUPT20H	0.088814434	6.57962206	2.662384418	0.102746606	0.283626136

PI4KB	0.181033595	6.579728485	8.965952349	0.002750573	0.024827579
RGMA	0.254753446	6.579804813	8.274245078	0.004021138	0.032594987
WDR91	0.022763971	6.581012471	0.167435593	0.682401007	0.833499745
SCCPDH	-0.052286652	6.582609125	0.618752248	0.431511337	0.658653601
LRIG3	0.003972107	6.58275429	0.002661331	0.958856907	0.982278445
TMOD3	0.044718482	6.583600818	0.458957903	0.498111402	0.70960986
ADGRA3L	-0.090149126	6.584986252	2.743399615	0.097656808	0.273991768
TMEM259	-0.021601699	6.585098516	0.154074222	0.694671899	0.840749198
PTCD3	-0.120577507	6.58555692	1.981559861	0.159226175	0.374241781
EXO1	0.034390852	6.585729737	0.152974465	0.695708906	0.841440815
GNA13	-0.182721314	6.585750773	6.335276278	0.011835968	0.069781745
IARS2	0.046158642	6.586881393	0.631157017	0.42693123	0.654871047
CPSF2	0.056278528	6.586957456	1.183484494	0.276647762	0.511832575
RNF166	-0.078508567	6.587124849	1.179031215	0.277553309	0.512700909
TMEM248	-0.211754403	6.587867296	11.99610561	0.000533118	0.007608381
DACT1	-0.223423844	6.58818243	3.750114569	0.052803892	0.186176996
ACAA2	0.095431616	6.589080154	0.962733935	0.326499025	0.564254007
DCAF12	0.063208342	6.589324644	0.710664712	0.399223232	0.631594874
ATR	-0.324238974	6.589372667	15.44670373	8.49E-05	0.001823043
AHCYL1	0.086927346	6.589788252	1.683972735	0.194397405	0.418911642
ANKRD50	-0.002667463	6.590615432	0.001627118	0.967824013	0.986817198
PHF21A	-0.024760101	6.59078668	0.168333467	0.681597175	0.83319824
KANSL3	0.145009177	6.592288868	6.249169591	0.012425154	0.072399403
SUGT1	0.178161098	6.594087626	11.15275151	0.000839071	0.010580014
CHMP4B	0.06212259	6.594692857	1.435875338	0.230807968	0.462262064
XYLT1	-0.025871103	6.59502918	0.100657925	0.751041522	0.874063812
ZNF318	-0.040041333	6.595721837	0.350533338	0.553811376	0.75180646
EFR3A	-0.113105986	6.596418344	3.033699162	0.081551893	0.245147314
KIAA0430	-0.098040965	6.596591166	1.101989513	0.293829904	0.530848821
ALCAM	-0.337813044	6.597696165	8.563787172	0.003429153	0.029070953
KIF5C	-0.303819569	6.597840064	13.50540334	0.000237878	0.004124631
MPLKIP	0.011537801	6.597917774	0.047023815	0.828325438	0.918593819

NT5C2	0.168907861	6.597956495	9.965178093	0.001595287	0.01696862
ZNF280D	-0.063610359	6.598946158	0.492712641	0.482719747	0.697663204
GEN1	-0.011346796	6.59896737	0.03337616	0.855040138	0.932526733
ADAM9	-0.039654711	6.598995728	0.543354017	0.461046028	0.682731496
POLR1A	0.19743563	6.59942627	9.843658314	0.001704182	0.017793351
ATP5I	0.02970027	6.599896001	0.101745952	0.749744399	0.873444126
CUL5	-0.112608623	6.600759743	4.346965564	0.037074948	0.14840849
LYAR	-0.021672895	6.6008148	0.096471751	0.756105472	0.877026984
TWSG1	0.207037988	6.601124241	10.92533808	0.000948579	0.011562123
WDR12	-0.080216279	6.601395057	1.314423893	0.251595245	0.486257899
LOC107056412	-0.06916259	6.601978649	1.247757904	0.263981138	0.499116354
KAT14	0.03918399	6.601983588	0.583681357	0.444872954	0.669687945
LOC107051082	-1.079342631	6.602069866	54.27418554	1.74E-13	5.74E-11
UTP3	0.096513541	6.602878874	1.220053236	0.269350205	0.504055228
OSBPL8	0.068295413	6.602940505	1.083088961	0.298007561	0.534872864
SLC38A6	0.151204686	6.603654937	5.084569419	0.02413967	0.112494555
MAGI1	0.08538419	6.60367509	1.678903759	0.195070181	0.420031382
LOC107055284	-0.260436745	6.60413852	3.063877452	0.080050435	0.242449825
TYMS	0.298518386	6.604249162	25.20964623	5.14E-07	2.76E-05
GPC2	-0.081463677	6.604426447	1.878282494	0.170529482	0.389244365
KANSL2	0.356128393	6.605124342	26.74517015	2.32E-07	1.37E-05
COX7A2	-0.039571493	6.605224139	0.261112512	0.609356873	0.787540633
PTPRK	-0.005681986	6.605768898	0.006385039	0.936311667	0.972171218
ZBED4	-0.031272697	6.606597733	0.276306742	0.59913219	0.781804074
NDUFB1	0.087933864	6.60664101	1.592012295	0.207038868	0.434352468
EMILIN1	-0.014378308	6.606816192	0.021330443	0.883882348	0.948004435
FLOT2	0.106419291	6.607010277	3.270717175	0.070526786	0.224259874
SORBS1	-0.125940813	6.607558276	3.381800051	0.065920023	0.215349212
WAC	0.045203837	6.607665982	0.727879612	0.393571353	0.626750858
SUDS3	0.106321452	6.609140364	4.102816845	0.04281183	0.163033711
KIF20B	-0.068409901	6.609576385	0.89221228	0.344878504	0.581631217
APBB2	0.09822504	6.610054524	2.549253163	0.110346405	0.297170045

QSOX2	0.023290939	6.610372171	0.145514131	0.702859613	0.846239477
TBCD	0.043737002	6.611223745	0.377086539	0.539166611	0.740257537
TMED8	0.011356361	6.611382763	0.046511944	0.829247889	0.918867551
SMAP1	0.042912886	6.61235804	0.612646876	0.433792952	0.660320354
LOC426097	0.072203337	6.612664432	1.486861692	0.222704025	0.452195797
HINT1Z	-0.016789017	6.613928846	0.025718623	0.872589409	0.94163263
DCTD	0.172658177	6.614486707	4.118425684	0.042418535	0.162174022
RAB21	-0.080754363	6.61486275	2.399153577	0.121400921	0.315584858
BTRC	0.072368815	6.615890598	2.046940762	0.152512378	0.364656375
SKA2	0.169863749	6.617834986	8.003054724	0.00466985	0.036385377
SLC2A1	0.060317599	6.618088101	0.596256082	0.440010076	0.664967091
LOC112531804	0.102444242	6.619722932	1.981932267	0.159186994	0.374203142
FAM49A	-0.16387737	6.619912363	5.789929793	0.016118233	0.086590115
STK38	-0.070587375	6.620184269	1.571249292	0.210025679	0.438045609
MED13L	0.086074005	6.620348043	0.600976985	0.438205505	0.663886934
MEMO1	0.138121652	6.620886079	7.410021668	0.006486154	0.045954099
DHRS11	0.059088596	6.621187293	0.859644244	0.353838267	0.590507979
EPHB1	0.001201651	6.621588358	0.000290016	0.986412804	0.993448882
PRPF38B	0.014051987	6.62170071	0.065035738	0.798706839	0.901588892
GFM1	0.106747072	6.622366207	4.081675032	0.043350664	0.164249407
MTMR4	0.056639826	6.622379662	0.873360064	0.350026777	0.58664602
MYO5A	-0.08135496	6.623509978	0.702453813	0.401960404	0.634366465
SLC30A4	-0.11843145	6.623581959	4.206940992	0.040258876	0.157241824
KANK1	0.071727073	6.623644487	0.584676355	0.444485155	0.669412705
DBR1	0.047683109	6.624304802	0.882679262	0.3474689	0.584085637
SPG11	-0.056908459	6.624948178	0.895902274	0.343882844	0.580845021
DGKE	-0.163337143	6.626028141	2.69214557	0.100843942	0.279687689
PITPNA	0.056914639	6.626146499	1.121631599	0.289567399	0.526210001
CXCL12	0.105850448	6.626152462	0.357796607	0.549732497	0.748398787
MPZL1	0.074573858	6.62631509	1.289800802	0.256084664	0.490864503
FOXJ2	-0.035313744	6.626966658	0.414714805	0.519586105	0.725172974
PHF20L1	0.109076962	6.627790054	4.758328379	0.029156852	0.126959748

SLC1A4	-0.00652445	6.628296192	0.007366756	0.931601712	0.969997359
RCC1	0.173705221	6.629138273	5.722178158	0.016751936	0.08880577
SEPHS1	0.022098575	6.629267455	0.161078767	0.688165259	0.837680081
HERPUD2	-0.056237293	6.629440166	0.875619727	0.349404214	0.586080157
HAT1	0.042109636	6.630426866	0.480561209	0.488168222	0.702056988
CLPTM1L	0.132811469	6.630585148	5.558989802	0.018386021	0.093753322
WWP2	-0.231981238	6.630716093	3.853304847	0.049648073	0.178843922
HTT	-0.03164548	6.631366984	0.219849882	0.639154325	0.806858657
SAV1	0.069464568	6.632941037	1.601843334	0.205642179	0.432512738
SUCO	-0.158347618	6.632957322	6.550917178	0.010483011	0.064077987
PPP2R5A	0.087827928	6.633781851	1.787438127	0.181238613	0.403338029
CIT	-0.02518844	6.634005257	0.189396289	0.663419556	0.821350448
CPSF4	0.037007294	6.634429162	0.455957127	0.499519501	0.710422938
NFATC3	-0.11535872	6.634429506	1.848556771	0.173951344	0.393551431
STK40	0.113536339	6.634461384	3.850711293	0.049724899	0.179003109
LOC107050835	-0.02089984	6.635240542	0.137246221	0.711034135	0.849781439
EPB41L1	-0.168597384	6.635318011	9.519056808	0.002033491	0.020370105
CD63	0.041166338	6.635690531	0.356474833	0.550470571	0.749027805
RAP1B	0.053987454	6.635745412	1.187949161	0.275743625	0.510906582
NARF	0.113402638	6.636538804	1.641943899	0.200058966	0.426200192
DPYSL4	-0.010312262	6.636625899	0.010836379	0.917091633	0.96217437
SAMD4A	0.138922579	6.636855903	3.985560086	0.045891841	0.169772987
TXNDC12	-0.018476017	6.63687262	0.086493094	0.768683721	0.884709423
DNTTIP2	-0.021858648	6.638038432	0.043182313	0.835382365	0.92213167
RAB11FIP3	0.304110062	6.638886111	21.89652511	2.88E-06	0.000115207
ELOVL5	0.112798903	6.640255911	4.632947506	0.031363638	0.133294601
SNX12	-0.052870342	6.640812647	1.049690271	0.305578431	0.543799122
KANSL1	0.098116568	6.640854147	1.97792972	0.159608676	0.374636459
NABP1	0.493672021	6.641569373	45.11201977	1.86E-11	3.40E-09
GPSM2	0.02253335	6.641639821	0.208158524	0.648214578	0.812282164
STOX2	-0.103634642	6.641828721	1.649250588	0.199060961	0.424844113
LOC101749138	-0.015929106	6.642162169	0.085966537	0.769368897	0.884879094

NOP14	-0.12678949	6.642206777	3.172082139	0.074906895	0.232872277
IP6K1	-0.082155628	6.642581373	2.459798356	0.116793775	0.308135572
NUP62	0.088427911	6.642778019	2.163754878	0.141299034	0.348249245
CASP6	0.097349918	6.642943414	3.002143962	0.08315441	0.248241258
STRBP	0.084143682	6.643599548	2.326442855	0.127192235	0.32509292
CYP27C1	-0.052216952	6.643912789	0.666690351	0.414207887	0.644580175
HOXB6	0.190231145	6.64464392	4.474050681	0.034413315	0.141638594
KIF21A	-0.367499934	6.645040255	25.44336942	4.56E-07	2.49E-05
C15H12orf49	-0.043193648	6.645774695	0.639867518	0.423758775	0.65308145
TAB3	-0.003585512	6.646735576	0.004037692	0.949334247	0.978504123
CSK	0.007416855	6.646891822	0.020156865	0.887099853	0.949086044
DECR2	0.049127262	6.647266447	0.432330359	0.510847687	0.718764262
NCAPG	0.1599568	6.647401605	6.929576347	0.008478177	0.055316575
INSR	-0.047315494	6.64852376	0.636440523	0.425002682	0.653554554
SLC12A2	-0.146039724	6.648913887	4.514396546	0.033610741	0.139733385
PDPK1	-0.049221802	6.649465725	0.562491653	0.453258056	0.676066109
COLEC12	0.117400685	6.649961172	1.987319162	0.158621467	0.37362099
RAPGEF1	0.018048946	6.650046482	0.095693182	0.757060513	0.877716532
CYP51A1	-0.195177784	6.6507396	6.619409423	0.010087328	0.062354237
PWP1	0.200463283	6.651379931	11.06321951	0.000880571	0.010919214
CAT	-0.052468247	6.651458424	1.089535753	0.296574094	0.533680327
TSPAN6	-0.063676341	6.651809661	0.935344547	0.333477988	0.571376385
KDM2B	-0.037676223	6.652037562	0.491364837	0.483319113	0.698061617
TANC2	0.094528317	6.652882986	1.909740221	0.166992118	0.384961516
ZDHHC20	-0.242705096	6.653505185	19.09778381	1.24E-05	0.000384122
DEDD	-0.106304457	6.653636005	3.340955143	0.067575372	0.218415389
TBC1D9B	-0.085891997	6.654401684	2.811911463	0.093566829	0.267810431
KIFAP3	0.137689412	6.654446027	4.678140934	0.030548791	0.130872263
LOC107049719	0.193857041	6.655339768	6.587044909	0.010272355	0.063191969
NCAPH2	0.126810498	6.656697129	4.664650977	0.030789681	0.131630085
TTC37	0.217150517	6.657545492	7.33943382	0.006745808	0.047114718
KIAA2013	0.004089429	6.659303951	0.004876748	0.944326008	0.976060582

TNS2	0.194302592	6.659350694	6.379253602	0.011546198	0.068788083
ANKRD13A	-0.120937129	6.659506764	3.957127612	0.046673281	0.171660448
NOVA1	0.01808559	6.659552264	0.077475764	0.780747777	0.891855827
FAR1	0.171251369	6.659597982	10.92831888	0.000947054	0.011552092
GREB1	0.297856431	6.65964458	13.80393939	0.00020291	0.003619311
MRE11	0.182577857	6.6601599	9.89293786	0.001659143	0.017446238
MTOR	-0.022068531	6.661876217	0.093742338	0.759472392	0.879397523
CYB5R3	-0.038083092	6.662697408	0.434610124	0.509735458	0.718063262
EHBP1	0.072618988	6.662844084	1.798866317	0.179849612	0.40156602
TNIK	0.14016908	6.663194166	6.83312896	0.008948238	0.05736005
BEND3	-0.001175269	6.663887485	0.000330545	0.985494544	0.993236215
USP47	-0.093093164	6.664291447	1.895788892	0.168550434	0.386836922
NLK	0.069728919	6.664366114	1.733911243	0.187912165	0.411143602
ADK	0.059791955	6.664545408	0.935305824	0.333487995	0.571376385
LOC107057377	-0.010304475	6.66461964	0.022317642	0.881245189	0.946842285
RYR1L1	-0.030587143	6.665812215	0.110705529	0.739342326	0.867380435
ATP13A1	0.06815609	6.665876124	1.654502357	0.198347242	0.4239639
ACVR1	0.063927291	6.665893131	1.534007031	0.215512056	0.444540058
ATL2	-0.07709078	6.667069623	2.008623287	0.156407196	0.37052698
SRF	0.013279528	6.667504816	0.048843577	0.825087894	0.917552618
SGPL1	-0.143032944	6.667846793	7.373037628	0.0066209	0.04653862
BOD1	-0.050816967	6.668897112	0.692526644	0.405306303	0.636520766
BMI1	0.003403815	6.669256712	0.00362189	0.952010568	0.979652938
GNS	-0.213887798	6.669625592	15.42421039	8.59E-05	0.001840134
GAR1	0.105743436	6.670258731	2.780460625	0.095420721	0.270575214
NCOA6	0.103387993	6.67113991	3.099554557	0.07831372	0.238816207
NSFL1C	-0.043009711	6.671422513	0.422549536	0.515667609	0.722215551
AGAP1	-0.134478917	6.67232454	3.041583994	0.081156691	0.244534238
TRIM3	-0.098095882	6.672607512	1.748992459	0.186003445	0.408690971
PPP4R3A	0.007095949	6.673688276	0.016858591	0.896692511	0.952793351
RGI3L1	0.185040882	6.673733383	6.380134595	0.011540468	0.068778848
SNRPD2	-0.111272801	6.673853604	2.737009631	0.098048091	0.274842017

SPPL2B	-0.264547617	6.674902952	8.086764937	0.004458969	0.035221456
HOXB1	0.523164752	6.675868797	28.50349421	9.35E-08	6.36E-06
HADH	0.007980749	6.676154541	0.019871776	0.887895786	0.949400556
CMPK1	0.121364755	6.676251636	5.535384594	0.018635669	0.094674262
ZFC3H1	-0.141001423	6.678770204	2.242647177	0.134250972	0.337009877
BRIX1	0.225086378	6.678794363	4.459755351	0.034702467	0.142421116
SP1	-0.037395515	6.679076575	0.455290203	0.499833369	0.710623646
LOC101748058	-0.016445996	6.679429397	0.024208234	0.876356245	0.943959209
ADAR	0.233717817	6.680162134	21.26386372	4.00E-06	0.000152793
FZD4	-0.22443577	6.680330975	6.732356959	0.009467941	0.059691561
UBN2	0.105448914	6.680993084	2.649829602	0.103560997	0.284879329
SUPT7L	0.099113136	6.682897771	2.574686774	0.108585593	0.293970224
PRPSAP2	-0.050313776	6.684000697	0.844642445	0.358072396	0.594395831
FARSB	0.176044101	6.684033142	6.382618466	0.011524329	0.068707547
CPNE1	-0.118748063	6.685069758	4.139403163	0.041895939	0.160923826
ADI1	0.155478282	6.685124381	4.752129358	0.029262063	0.127226739
ATF7IP	-0.237011668	6.685722952	11.95907906	0.000543817	0.00773424
CST3	-0.100350289	6.686026476	1.989003314	0.158445131	0.373419454
KDM4A	-0.050732845	6.686084018	0.646249883	0.421456644	0.650750442
NDUFB9	-0.131444466	6.686706258	3.634595009	0.056590371	0.194626589
CEP170B	-0.079518782	6.686815394	1.39254336	0.237976056	0.470670135
MTFR1	0.112136677	6.687171129	4.032563205	0.044630085	0.166715272
LOC107053499	0.005068043	6.687392917	0.001448477	0.969640752	0.987658702
OXSR1	0.043775083	6.687588392	0.428241541	0.512853061	0.72023806
LCOR	-0.158841708	6.68776397	5.812157271	0.015915759	0.085782446
CCNT2	-0.129766249	6.687909721	2.986475158	0.083962749	0.249899506
TULP4	0.296288499	6.688133643	16.49445551	4.88E-05	0.00117209
ANTXR2	0.019758652	6.688307704	0.131108978	0.717284972	0.853728044
ZC3H18	-0.11971768	6.6886091	1.796701862	0.180111736	0.401753912
DPP9	-0.134551931	6.68864354	6.063649121	0.013799226	0.077865936
KIF3A	-0.128460278	6.689633886	3.846114183	0.049861383	0.179259953
TIMM8A	0.033622897	6.690001498	0.24086374	0.623582971	0.797656856

SKP2	0.115627889	6.691052014	2.232052013	0.135174219	0.338271869
MIDN	-0.109716602	6.691184566	1.616935175	0.203519664	0.430292477
ABR	-0.039956096	6.692006666	0.472660902	0.49176557	0.704555909
GOLGA2	-0.136295208	6.692039764	6.64055837	0.009968269	0.061913716
NCS1	-0.170567277	6.692874756	5.912183844	0.01503652	0.082201308
LOC771811	-0.098752217	6.693848011	3.101901625	0.078200899	0.238646021
LMAN2	0.027879144	6.694688014	0.272807258	0.601454826	0.783047643
XXYLT1	-0.051072085	6.694972195	0.843409235	0.358423545	0.594782113
COL14A1	-0.058113867	6.695286519	0.348511253	0.554957086	0.75264311
HECA	0.027023678	6.695541265	0.225386072	0.634966294	0.804339521
BRD1	-0.096029741	6.695762704	2.114451645	0.14591431	0.355031785
ZFYVE9	0.066518679	6.696203261	1.212177829	0.270901237	0.505979552
ACER3	-0.057995526	6.696252562	0.736895046	0.390657325	0.624103522
G2E3	-0.074019621	6.696521956	1.16751833	0.279911744	0.515206683
CHD1Z	-0.053822036	6.696699868	0.411447153	0.5212359	0.726233326
BTBD7	-0.103844701	6.697115006	2.736689371	0.098067747	0.274842017
LOC112530674	0.164031118	6.697212107	1.544278903	0.213982021	0.442791299
ADAMTS9	-0.005631841	6.697655155	0.003617207	0.952041565	0.979652938
PXYLP1	-0.01151052	6.698241506	0.031529512	0.859064209	0.933987549
RBM15	-0.230889495	6.698250164	8.864930141	0.002907014	0.02584274
HSF2	0.02059397	6.698926106	0.122492156	0.726347108	0.859117428
RECK	-0.024751234	6.699115054	0.163005084	0.686404745	0.836341361
DCAF7	-0.092302021	6.699330401	2.17386529	0.140373018	0.346874608
COPS2	0.040002671	6.699560745	0.441489813	0.506404261	0.715978975
AKR1B10L1	0.002614726	6.700221535	0.002854298	0.957392786	0.981573571
SZRD1	-0.083972202	6.700785991	1.909853975	0.16697948	0.384961516
LIN28A	0.518135316	6.701209822	8.265780609	0.004039929	0.032673977
MKL1	0.206682554	6.701392737	10.72175014	0.001058835	0.012534623
DNAJC13	0.152388124	6.70244627	6.467827701	0.010984472	0.066354436
KLHDC4	0.07823923	6.702803964	0.750854132	0.386205942	0.620667915
ABHD17A	0.02860515	6.703217091	0.254336795	0.614037826	0.790981089
TMEM132A	-0.014308412	6.703372515	0.0446722	0.832607935	0.920860571

FBXW5	-0.040091547	6.704169991	0.585481182	0.444171857	0.669129516
ASNA1	0.027630232	6.705789859	0.209943403	0.646811767	0.811526992
FBN1	-0.141945922	6.706096314	2.850193251	0.091362954	0.263889312
TMEM164	0.042129335	6.706510972	0.381432895	0.536837371	0.738106529
CDC23	-0.031223968	6.706915802	0.341075129	0.559209294	0.754678006
TET3	0.113170124	6.707857387	2.360595077	0.124434714	0.320131196
LDB1	-0.055804402	6.708116931	1.070171027	0.300906818	0.538455175
SEH1L	0.016114376	6.709496969	0.081315778	0.775522166	0.888893846
CCDC80	-0.307338487	6.709553075	15.09397607	0.000102289	0.002103961
C5H14orf37	-0.098802078	6.7099836	1.523964453	0.217020538	0.446105303
KNTC1	0.240210312	6.710994149	11.4180183	0.000727352	0.009521546
LPCAT1	0.139381951	6.711024731	5.217066165	0.022366255	0.106863159
POLRMT	0.052009892	6.711491711	0.66849408	0.413577132	0.643781261
LSM11	0.037997072	6.711864512	0.31200327	0.576453493	0.766303478
USP44	-0.174027569	6.712421789	7.263395459	0.007037411	0.048411624
ACADL	0.14172618	6.713425229	7.13399985	0.007563582	0.051133418
COPS3	0.076444288	6.713527329	1.465066292	0.226125738	0.456216162
POLR2E	0.204104854	6.716102767	12.38725333	0.000432275	0.006495963
PDE5A	0.029848351	6.716957197	0.101183111	0.750414448	0.873704786
BABAM1	0.100280038	6.717113099	1.696815163	0.192705012	0.41714129
HOXB7	0.256590641	6.717750001	13.12439151	0.000291476	0.004825189
MGRN1	0.123215454	6.718367525	4.883255093	0.027118432	0.121424164
LRWD1	0.183436858	6.719195952	8.708038951	0.003168098	0.027437399
CSTF2	0.023403769	6.719330169	0.148119537	0.700338912	0.84438535
TSSC4	-9.52E-05	6.719792978	2.55E-06	0.998726554	0.999310367
HIC2	-0.016005071	6.720731398	0.086136945	0.769146907	0.884809309
AKT1	0.013029187	6.721132163	0.072386412	0.787893338	0.895481757
ATP5L	-0.098357515	6.721701446	1.239640109	0.265540425	0.50073011
HOXB5	0.034191995	6.722138413	0.101749506	0.749740175	0.873444126
RCHY1	0.011072191	6.722437471	0.048063486	0.82646788	0.917837275
ZSWIM5	0.013119999	6.722670919	0.037830254	0.845784388	0.927768873
CLUH	0.01761828	6.723052693	0.102462575	0.748894225	0.873205161

SLC22A5	-0.180767746	6.72464141	11.42293792	0.000725429	0.009518145
SELENOF	0.132437214	6.724765817	5.740861048	0.016574665	0.088264115
PITPNC1	0.022538439	6.72520788	0.11655656	0.732799609	0.863216914
COL6A3	-0.282513557	6.72544009	5.618121959	0.017775687	0.091922981
CHAF1A	0.037168106	6.72595153	0.346566007	0.556063497	0.752930284
COL8A1	-0.225301509	6.726893883	4.05986685	0.043913962	0.165431374
CDCA4	-0.014774084	6.727084019	0.021825934	0.882551098	0.947489154
UPF3B	0.045456228	6.727284173	0.682435412	0.408749358	0.639203213
SNRPG	0.092980744	6.727561965	2.004387078	0.156844678	0.371214577
POLR2C	-0.068673704	6.727975125	1.100812489	0.294087866	0.531081633
DIXDC1	-0.098983883	6.728824119	2.386073302	0.122420787	0.316984114
KCTD20	-0.105322169	6.728867186	3.511401055	0.060947894	0.20464218
FOXM1	0.057548866	6.729758306	1.209379948	0.271454956	0.50640736
NUP58	-0.040169724	6.730678708	0.40340969	0.525333584	0.729975014
FZD2	-0.061479867	6.73083837	0.963940247	0.326196128	0.564154365
RAPGEF6	0.126780711	6.731079465	4.773647488	0.028898538	0.126376774
EIF4EBP2	-0.015750528	6.731572967	0.058498919	0.808884385	0.907863895
RBM17	0.031129352	6.731630086	0.322910484	0.569863778	0.762434642
DRG2	0.006334453	6.732314468	0.008804428	0.925242678	0.966535568
FAM193B	0.041557015	6.73351341	0.529100775	0.466985158	0.686706056
MN1	-0.023648894	6.734055423	0.090557244	0.763469927	0.88142129
PCGF3	-0.086587929	6.734697737	1.163846796	0.280669175	0.516358387
ATP6V1G1	0.111370037	6.734759154	3.483730603	0.061974876	0.207115138
SEC61B	0.165025858	6.734836553	5.888122086	0.015243347	0.082863984
DHRS3	0.047808299	6.736477204	0.285764704	0.592947479	0.777327412
HUNK	-0.092165371	6.737304392	2.122496086	0.145149816	0.354052804
FAM171A1	0.158537613	6.737475931	9.157336622	0.002477229	0.023095068
SNRNP40	-0.042983461	6.738163023	0.388905211	0.532875431	0.735103154
TPP2	0.10940686	6.738418594	3.673731153	0.055276312	0.191580742
EXOG	0.322980246	6.738972363	26.7736355	2.29E-07	1.36E-05
SLC35B1	0.104568528	6.739210348	4.056732957	0.04399554	0.165511342
HIPK2	-0.008444352	6.739506721	0.011506496	0.914576102	0.960982957

CERS2	0.087258749	6.740019083	2.751474239	0.097164802	0.27321374
AP3M2	-0.064650756	6.74013684	0.97664773	0.323027831	0.560580359
PLK4	0.012573621	6.740528142	0.030589375	0.861159636	0.935139468
PARD3B	0.068444963	6.74105783	0.4971984	0.480733714	0.696651103
FBXO22	0.101722326	6.741555922	3.547132875	0.059648434	0.20141904
FAF1	0.046894771	6.741865559	0.496312283	0.48112497	0.696704901
HOMER3	-0.058401237	6.742501097	0.707067676	0.400418994	0.632938958
RSRC2	-0.109200948	6.742982498	4.113772419	0.042535384	0.162368316
LOC107050371	0.078934443	6.743000949	1.570040291	0.210201163	0.438045609
SLC16A6	0.010099387	6.743582369	0.013316027	0.90813197	0.957904588
COX7B	0.054482755	6.743924427	0.679231236	0.409851571	0.640162088
NELFB	0.046615361	6.744834945	0.693142682	0.405097491	0.63642154
RPA2	0.005598108	6.745828498	0.008584979	0.92617752	0.967021008
GAS1	0.143121376	6.745860839	1.754303516	0.185336634	0.407879405
TMEM200A	-0.086068658	6.74610658	1.642634254	0.199964421	0.42610911
ARL5A	-0.118158587	6.746973342	4.379618553	0.036371136	0.146850563
AHCTF1	-0.057617362	6.748021479	0.857537043	0.354428852	0.591193792
HEG1	-0.040144303	6.748106959	0.060083834	0.806363487	0.90650898
ATF1	-0.041902351	6.748235957	0.533807924	0.465010314	0.685640208
RAB18	-0.004237936	6.748526408	0.005286484	0.942038315	0.97516611
DSP	0.294130725	6.74914896	3.321662409	0.068372659	0.219955447
ARHGDIB	0.070657111	6.749522246	1.180078139	0.277340089	0.512422093
BMPR1A	-0.066580267	6.749681261	0.687843218	0.406898952	0.637801433
PDCL	0.050545526	6.751239899	0.689454356	0.406350037	0.63741562
RNF4	0.002132467	6.751476067	0.002093815	0.963502953	0.984418122
STXBP4	-0.064832446	6.752751198	0.469422534	0.49325295	0.705551458
CHST14	-0.107522143	6.75332711	4.0670905	0.043726529	0.164914059
SEC23A	0.296276485	6.753344723	27.45438352	1.61E-07	1.03E-05
METRNL	-0.107667046	6.753439916	1.784526525	0.181594476	0.403693205
LOC107050463	-0.542876058	6.754134433	27.84732651	1.31E-07	8.64E-06
MTMR8	-0.069468851	6.754459521	1.51144826	0.218918228	0.448199473
RND3	-0.131777689	6.754536376	2.525361109	0.112029132	0.29977185

PPP3R1	0.075337822	6.754676386	2.292895717	0.129967245	0.329676434
ARPC4	-0.010642462	6.755147529	0.035106235	0.851373223	0.930961329
URI1	0.074763405	6.75520816	1.075269432	0.299758232	0.537080745
NEFL	-0.318829158	6.755989764	3.947385896	0.046944239	0.172502783
SMN	0.193347735	6.756017978	4.950217922	0.02608738	0.118320793
ECT2	0.193666673	6.756098383	11.03108923	0.000895965	0.011056423
PUS1	0.081970995	6.756972432	2.276376362	0.131358464	0.332232327
EXOSC10	-0.03158092	6.757242333	0.28423931	0.59393599	0.777936536
HMGCR	-0.018485835	6.758738061	0.059616067	0.807103794	0.906917026
ZNF532	0.006197829	6.758808279	0.005049222	0.943351691	0.975792553
NAPA	0.228929547	6.758836863	12.01170757	0.000528674	0.007558063
SACS	-0.141997544	6.758949843	2.902664118	0.088433285	0.258421187
VPS4A	-0.031665563	6.759151438	0.174021549	0.676562184	0.830023971
RAD54L2	-0.066518279	6.759824098	1.534136743	0.215492654	0.444540058
ERGIC2	0.008232639	6.759858811	0.028607246	0.865689066	0.937967577
C26H6orf106	0.093201044	6.759972626	3.040137074	0.081229058	0.244534238
CCDC85C	0.112383085	6.760052559	1.071107195	0.300695491	0.538229555
HK2	0.181971266	6.760949846	4.04936931	0.044187851	0.166007097
FAM208A	-0.007011797	6.761230233	0.016758043	0.896999322	0.952819305
OLFM1	0.264405296	6.761256064	5.554121272	0.018437226	0.093956195
KIAA1551	0.075396265	6.761777376	0.658228025	0.417186215	0.647256191
LOC101751423	-0.479566814	6.761894128	23.95385014	9.87E-07	4.76E-05
CELSR2	0.244860799	6.762259178	4.572936372	0.032480845	0.136484247
MIA3	-0.176304074	6.762275777	11.92984792	0.000552417	0.007836224
TNFAIP1	-0.127599872	6.76231567	4.583504366	0.032281132	0.136033958
ARHGAP11A	-0.051456632	6.762697706	0.496251339	0.481151899	0.696704901
SNU13	0.20435389	6.763296831	11.54211586	0.000680372	0.009094651
TROVE2	-0.06506807	6.763410616	1.001661997	0.316908687	0.55469976
TLE3	0.025360376	6.763475531	0.077398113	0.780854871	0.891855827
TMEM201	0.035158077	6.764167508	0.32072665	0.571171282	0.763140471
TMTC4	-0.063705459	6.765173294	0.991000897	0.31949787	0.557135546
ZC3H6	-0.038868217	6.765649005	0.320124261	0.571532978	0.763358373

EXOC7	0.112230555	6.765957166	2.768482686	0.096137252	0.271780574
GSN	0.044793094	6.766457973	0.426677725	0.513623657	0.720825354
CHMP7	-0.043132408	6.767353066	0.647862569	0.420877905	0.650469233
SHC1	0.010855855	6.768157221	0.029433262	0.863782476	0.936765349
MAD2L1	0.100247028	6.76823952	2.700831503	0.100295926	0.278590389
XPO4	-0.04543817	6.76838039	0.636823101	0.424863545	0.653441581
LGR4	0.136733979	6.768653607	5.457023762	0.019489816	0.09771631
NUSAP1	0.072432356	6.769686977	1.484603191	0.223055697	0.452356596
PGAM5	-0.005821588	6.770355981	0.012175334	0.912138231	0.959684907
POLE	0.061727639	6.770579929	1.267507337	0.260234823	0.494819045
UHRF2	0.134812502	6.771527189	3.344934199	0.067412174	0.217974116
SRP68	-0.077814158	6.772055933	1.41965163	0.233460752	0.464979011
RIC8B	-0.049924379	6.773064126	0.688636446	0.406628563	0.637633886
FAM134A	0.035171138	6.773301615	0.329702143	0.56583453	0.759507887
SDHA	0.001051736	6.773381315	0.000325913	0.985596533	0.993262552
ANGEL1	-0.093120659	6.775388023	3.077974025	0.079359324	0.241066582
SDK2	-0.182336998	6.775403397	3.994294933	0.04565455	0.169305225
CD276	0.024072292	6.77602568	0.199102892	0.655445942	0.816217216
SLC4A7	-0.146301283	6.776545787	3.652693586	0.05597861	0.193149094
VASH2	0.203980974	6.777927926	8.64240954	0.003284268	0.028235437
CDT1	0.054457645	6.778018157	0.38540869	0.534722717	0.736480513
DES	0.093782226	6.778087226	0.630699414	0.42709888	0.654871047
HMG20A	-0.043384573	6.77836101	0.652857483	0.419092904	0.648868435
SLC20A1	-0.046819629	6.778544395	0.775539403	0.37850883	0.613269279
CERCAM	0.051449945	6.778847416	0.370306868	0.542837046	0.743026497
NCLN	-0.24091476	6.779027969	16.75016114	4.26E-05	0.001051902
ZC3H7A	-0.042070053	6.77918845	0.531384147	0.466025516	0.686100024
OAF	-0.076293145	6.780110232	2.121160413	0.145276437	0.354309139
CISD1	0.272047366	6.780144801	24.16472347	8.84E-07	4.34E-05
USP19	-0.071659537	6.780993843	2.31454656	0.128168671	0.326827132
AURKA	0.090392879	6.781462827	1.987989409	0.158551263	0.373562577
FZR1	-0.087755775	6.781883233	2.962571887	0.085212301	0.25228531

SKI	-0.164045605	6.781964247	3.554827568	0.059372476	0.200899465
RCAN3	-0.039273526	6.782767358	0.536381447	0.463936253	0.68503868
SLC25A39	0.075008518	6.783079196	1.451682769	0.22825813	0.458718555
GMPS	0.017804203	6.783087759	0.117839228	0.731389941	0.862328105
FASTK	0.15210336	6.783683983	5.792523714	0.016094468	0.086518939
POLDIP2	-0.072219042	6.785109495	1.561507471	0.211444634	0.439031098
PTP4A2	0.188401803	6.78515582	9.693975412	0.001848731	0.01898931
GNPAT	0.125684785	6.785181065	4.505182758	0.033792285	0.140028216
SMARCC2	0.084728475	6.786303996	1.064789998	0.302125234	0.539918567
DNAJC9	-0.145406402	6.786376401	6.573761014	0.010349302	0.063567662
NARS	0.036416342	6.786714265	0.317566803	0.573073589	0.764693721
COPS8	-0.010998149	6.787581679	0.03806685	0.845308966	0.927489433
EGLN1	0.079709315	6.788115085	1.938599451	0.163820684	0.380421868
SH3KBP1	-0.040362115	6.788232464	0.456073881	0.499464589	0.710406242
ACSL3	0.035568281	6.788271514	0.42208059	0.515900692	0.722359455
LOC107051857	-0.074203745	6.788471718	1.085874402	0.297387119	0.534400463
KRAS	-0.062086665	6.78860161	1.17312533	0.278759999	0.514181965
TNC	-0.190319508	6.788800966	3.126407582	0.077033318	0.237108726
CCM2	0.052353569	6.788933598	0.84239017	0.358714074	0.595084191
RPS6KA1L	0.061832364	6.789443329	1.289650915	0.256112293	0.490864503
IQCE	-0.194783346	6.789975031	10.27035365	0.001351849	0.01487938
MAP3K7	-0.076054764	6.790976702	2.504339255	0.113533094	0.302406036
NIFK	0.110239024	6.791040479	3.196714705	0.073786354	0.230258761
VPS72	-0.089022763	6.791394684	1.432564228	0.231346411	0.462889736
STRADA	-0.172600565	6.791453109	8.621089877	0.00332293	0.028463721
UBE3A	-0.003457423	6.791798228	0.003936724	0.949970901	0.978820988
NPLOC4	-0.213506713	6.792416953	9.04854404	0.002629041	0.02407394
NEK1	0.077922198	6.792456344	1.789191336	0.181024722	0.403134632
ARHGEF11	-0.075201311	6.792600345	2.190011126	0.138908312	0.344313238
ATP5A1W	0.279115133	6.792914089	0.237435799	0.626064281	0.799103476
MYL9	0.259917179	6.793795029	13.80296361	0.000203016	0.003619311
PRDX4	0.027648908	6.794044574	0.243642295	0.621587746	0.795959747

COL12A1	-0.124482915	6.794163024	1.192550818	0.274815629	0.509715719
WDR26	-0.201644906	6.794345627	15.25565242	9.39E-05	0.001968214
CHTF18	0.034072512	6.794939897	0.317770567	0.572950543	0.764693096
PAG1	-0.069885241	6.795097168	0.518292791	0.471570839	0.690241785
KIAA1429	-0.017282161	6.796634045	0.085260034	0.770291817	0.885380276
C13H5orf15	-0.025570355	6.797038936	0.216010902	0.642096367	0.808826129
ECE1	0.214252747	6.797564779	11.90609187	0.000559507	0.007882433
RCL1	-0.003001433	6.798110122	0.001065761	0.973956894	0.989715351
TERF2	0.013724774	6.799112282	0.044363398	0.833178963	0.921086197
ZCCHC24	0.176722092	6.799749578	8.630394014	0.003306001	0.028363003
FKBP10	0.02692422	6.800166156	0.19778417	0.656515377	0.816612028
WASF2	-0.020561702	6.800382692	0.129289866	0.719169469	0.85499123
DNAJC3	0.136321637	6.801507255	6.363739786	0.011647579	0.069216652
ADGRL3	-0.150082139	6.802448331	3.622818228	0.056992253	0.195702738
ZADH2	0.022640133	6.802839219	0.115922035	0.733500163	0.863886574
SULF2	0.17443591	6.803289	5.008796775	0.025218829	0.115702211
MYO1B	-0.025788199	6.804491735	0.120031553	0.72900027	0.860832396
TOMM5	-0.021071078	6.805688767	0.037321108	0.84681274	0.928334686
NUF2	0.015822564	6.805882578	0.053529431	0.817031811	0.913581031
ZNF217L	-0.014994366	6.806528631	0.066513494	0.796482463	0.900763967
ISCU	-0.249782351	6.806547031	15.74878965	7.23E-05	0.0016172
LAS1L	0.17882903	6.806557447	13.69746659	0.000214744	0.003791429
SMNDC1	-0.073896037	6.807903218	1.663978084	0.197067066	0.422518563
DYNC1LI1	0.014018174	6.808247807	0.069856944	0.791545184	0.897601674
MAP1LC3B	0.008126078	6.809016696	0.021971516	0.8821629	0.947322527
LOC417113	-0.077198518	6.809976903	1.078608021	0.29900916	0.536085828
ARHGEF12	0.005806348	6.81039841	0.006994266	0.933349241	0.970935754
DDX54	0.033330393	6.810575638	0.352300419	0.552813798	0.751194021
ATP13A2	0.15166607	6.811127337	3.640251637	0.056398409	0.194068555
UMPS	-0.193224567	6.81277499	6.777558237	0.009231109	0.058593149
RFC4	-1.90E-05	6.813725351	5.39E-08	0.999814797	0.999872573
ITGB5	0.081491907	6.813909637	0.612429093	0.433874677	0.660383666

SOX11	-0.338419513	6.814788872	19.26977411	1.13E-05	0.000361211
DAB1	0.018861947	6.814815666	0.077155014	0.781190524	0.891991539
RAD23A	0.039221262	6.814839442	0.38346168	0.535756402	0.737359618
THOC1	0.011057059	6.81520975	0.032067635	0.857879295	0.933748101
PDE7A	0.021762345	6.815276421	0.181683011	0.669930859	0.82519211
HMGA1	0.307338657	6.816317085	25.63604002	4.12E-07	2.28E-05
PELI1	-0.00037266	6.816510083	5.57E-05	0.994043773	0.997413991
PCBP4	0.196148931	6.817287391	11.48998365	0.000699723	0.009255574
RRP9	0.121845972	6.817424406	3.487149809	0.061846981	0.206890033
MTHFD1L	-0.025583399	6.8179066	0.155912004	0.692948469	0.839553694
PUM3	0.084970317	6.818468311	0.826983642	0.363146193	0.599097263
DNM2L	-0.030523632	6.818912411	0.27409269	0.600599481	0.782591357
TJP2	-0.03723404	6.818943273	0.139234149	0.709043562	0.849542309
GLYR1	0.08267906	6.819516766	2.107498418	0.146578761	0.355876056
TAF6	-0.016441111	6.819561629	0.071725149	0.788841329	0.896167916
PLXNB1	-0.182868042	6.820329144	5.446725052	0.019605045	0.097995449
RALGDS	-0.106682975	6.820657365	1.701761097	0.192057825	0.416213817
PREX1	0.152816597	6.820922439	4.75608792	0.029194832	0.127090201
KLHL11	-0.082012463	6.82209341	1.235758817	0.266290002	0.501637318
ST5	0.011781692	6.822288958	0.028886698	0.865040908	0.937625632
PRCC	-0.150704103	6.822315699	7.401239384	0.006517896	0.046066172
LPAR1	-0.105442241	6.824652043	1.072712779	0.300333496	0.53775933
MDC1	0.273450689	6.824733352	16.61085458	4.59E-05	0.001115334
NUP88	0.041670026	6.824984727	0.570785415	0.449946982	0.673300375
TLK2	0.027492264	6.825554078	0.275806329	0.599463165	0.781939467
CELF1	-0.158878554	6.82576196	4.45258061	0.034848548	0.142552294
CTBP2	-0.08790393	6.827217992	1.5827935	0.208358771	0.436100304
BRD8	0.082016347	6.827261966	2.110312844	0.146309404	0.355567069
H2AFY	0.133681066	6.827361606	4.83062203	0.027958542	0.124004799
FBLN1	-0.509793844	6.828447686	29.34483454	6.06E-08	4.37E-06
ANXA5	0.308924123	6.828473585	23.82506729	1.05E-06	4.97E-05
MPDZ	0.164961728	6.828529355	3.645602103	0.056217473	0.193648425

SPATA13	-0.148728213	6.828725005	2.173376417	0.140417637	0.346932766
DDX24	0.069094253	6.8296805	1.448214365	0.228814689	0.459556415
SCUBE2	0.029413487	6.829790649	0.045622873	0.83086281	0.919610381
ADAM17	-0.130847423	6.831575135	7.044523567	0.007950797	0.052946324
CD81	0.017014404	6.832066045	0.088346768	0.766289514	0.883251187
MED13	-0.050164782	6.8324896	0.53498969	0.464516611	0.68540359
RASA1	0.145983083	6.832645738	2.851658673	0.091279718	0.263787806
CLNS1A	0.009452096	6.833726606	0.035794866	0.849939742	0.930275273
PKN2	-0.071360225	6.834166953	1.770572436	0.183311251	0.40570577
GNA12	-0.145649007	6.835814891	5.075911102	0.024260519	0.112764397
AIDA	0.099336392	6.836475233	3.404257147	0.065028388	0.213483102
AFG3L2	-0.208575116	6.83749343	17.64227527	2.67E-05	0.000715351
TICRR	-0.049575796	6.838309245	0.37382409	0.540927154	0.74156097
UHMK1	0.006371618	6.838612714	0.013949761	0.90598121	0.957006893
BOD1L1	-0.196937622	6.83866126	7.809779043	0.005196426	0.03922348
BCL2L13	-0.006457923	6.838749708	0.012918862	0.909506395	0.958432772
TMEM33	-0.056886763	6.839576732	1.240857306	0.265305894	0.500413628
CCNG1	-0.511800376	6.839602104	13.79742209	0.000203615	0.003626073
TBC1D14	-0.109954096	6.840421798	4.129478006	0.042142348	0.161461693
ABCF3	0.096073244	6.840702591	4.17784203	0.040955818	0.158795473
SALL1	0.296400783	6.84149503	9.461802319	0.002097948	0.020835945
CHAF1B	0.080165187	6.842345999	1.55100903	0.212986542	0.441341589
MRPL12	0.114671953	6.842371896	2.858683362	0.09088186	0.262937133
ZFP36L1	0.031149461	6.84248871	0.133336478	0.71499744	0.852371432
PHF14	0.242231761	6.842834341	13.25160412	0.000272347	0.004586857
UBE2C	0.039190282	6.843632339	0.320158415	0.571512459	0.763358373
RSF1	-0.085410986	6.844442454	1.778784848	0.182298611	0.404493483
MCMBP	0.16254277	6.844609421	9.416767866	0.002150102	0.02113496
KPNA1	-0.017428651	6.84465238	0.113067169	0.736678868	0.865613451
SLC39A9	-0.157126691	6.845743972	8.436056268	0.003678531	0.030726008
PFDN5	0.100264102	6.846314466	1.609739354	0.204528445	0.431327134
HOXD3	0.114068471	6.847153607	1.852649566	0.173475548	0.392862668

MPC1	0.070437058	6.847267448	0.832794638	0.361465673	0.597823318
INTS8	-0.025143073	6.848380969	0.211273697	0.64577092	0.81097157
MRPL3	0.038471086	6.848498442	0.444752471	0.504837514	0.71526617
DUSP22	-0.093038798	6.849169274	1.73514029	0.187755764	0.41105922
DESI2	-0.048524999	6.850236789	0.922946005	0.336702635	0.573918636
UCHL5	0.08592058	6.850277265	1.522944594	0.217174432	0.446236333
ATP2C1	0.207312569	6.85174043	16.53784426	4.77E-05	0.001148967
TXLNA	-0.072636575	6.852038852	1.915143628	0.166393014	0.384172449
PCNX1	-0.02282883	6.852377036	0.135922185	0.712369046	0.850434818
COPS4	0.154580594	6.852425668	9.098103581	0.002558747	0.023654031
ROCK1	0.025773533	6.852474102	0.22559496	0.634809512	0.804262718
HEXIM1	0.232579265	6.853359982	11.14049221	0.000844635	0.010627705
MTMR9	0.056682944	6.853372821	1.182584133	0.276830545	0.511998046
LOC101749223	-0.05682893	6.853552423	0.84063027	0.359216575	0.595497557
LOC107053919	-0.018539903	6.854281901	0.073865866	0.785789066	0.894573065
UACA	0.218566036	6.854440578	13.84665484	0.00019835	0.003551514
R3HCC1	0.038601051	6.854535968	0.532151558	0.465703702	0.686047844
ARFGEF1	-0.08087158	6.854850841	1.745461893	0.186448254	0.409285755
ELK3	-0.154005606	6.855104458	5.019381176	0.025065125	0.115289749
MAEA	0.130978731	6.855152371	6.705928617	0.009609287	0.06035146
TMX3	0.02347658	6.85544509	0.201130096	0.653810207	0.81509561
PSMD14	0.035481745	6.856436845	0.380428424	0.53737404	0.73847418
PDS5A	0.051805077	6.857058339	0.934558899	0.333681093	0.571533506
POMP	0.059222604	6.857115876	0.60941598	0.435007789	0.661252025
FAM126A	0.24546087	6.857374106	19.64712756	9.31E-06	0.000305923
KRT18	0.186145263	6.85811362	0.526004527	0.4682915	0.687705188
GLS	0.091386156	6.858273102	2.92730979	0.087092508	0.255820637
AGPS	0.018936308	6.858470671	0.15000789	0.698527819	0.843245195
GNL2	0.091467078	6.859158512	2.417184271	0.120010471	0.313008765
GLTSCR2	-0.048470987	6.859505278	0.703678581	0.401550391	0.634066049
ABCC5	-0.004618569	6.859790209	0.003610857	0.952083627	0.979652938
EEA1	-0.153526565	6.860439816	5.412129725	0.019997305	0.099042604

SCUBE3	-0.430308357	6.860659276	12.00430929	0.000530777	0.007581538
HSPA2	0.201243166	6.860929259	2.707932007	0.099850355	0.277680851
CABIN1	0.089266734	6.861252393	1.816427889	0.177739064	0.398568588
TRABD	-0.103301187	6.861586647	2.402822838	0.121116523	0.315094448
DNPEP	-0.109928279	6.862073121	4.698349488	0.030191598	0.129781281
SNRPF	-0.017958669	6.862202488	0.082707061	0.77366186	0.887954657
PPP2CB	-0.028102045	6.862782501	0.298956523	0.584537328	0.771459876
PYCR1	0.573424755	6.862957997	45.37151988	1.63E-11	3.01E-09
ANXA2	0.040038312	6.863907742	0.262455139	0.608438437	0.787473436
ACTR3	-0.017101053	6.864331285	0.138998641	0.709278535	0.849591042
MLXIP	-0.240450994	6.865690623	6.224001622	0.012602995	0.07323051
GCSH	-0.12599248	6.865763901	2.866549432	0.09043858	0.262232041
PPP1R12A	0.051735785	6.866001465	1.171101887	0.279174948	0.514618765
SHROOM2	0.044637647	6.866222986	0.370246987	0.54286967	0.743026497
ZBTB10	0.014015137	6.866620347	0.048199271	0.826226836	0.917837275
SLC35F1	-0.028986052	6.866675691	0.156733447	0.692181934	0.839288299
TBC1D13	0.185072232	6.866904731	10.27745582	0.001346655	0.014871953
HCCS	-0.014347624	6.86716837	0.03766125	0.846124935	0.9279516
MEX3A	-0.106948666	6.867209256	2.529855519	0.111710445	0.299578911
SEL1L	0.025135309	6.868731757	0.222192774	0.63737419	0.805776912
SETX	-0.03935099	6.868897042	0.254029242	0.614252146	0.791071383
DERL1	0.14720004	6.870546462	10.15857404	0.001436317	0.01566242
RFX7	-0.183055933	6.870669516	4.716044555	0.029882406	0.128923698
VEGFD	-0.552562328	6.870901568	12.24251503	0.000467128	0.006881464
MXRA5	-0.265196315	6.871096472	5.276866851	0.021610602	0.104527473
GIGYF2	-0.049293976	6.871748476	1.072369556	0.300410832	0.537832134
PPP1R9B	0.117828998	6.872371504	4.826540029	0.028024818	0.124170626
GPX4	0.091314492	6.872513869	1.327355863	0.249276158	0.483479394
RPS6KB1	-0.058894825	6.872540184	1.190861425	0.275155866	0.510047287
NELFA	-0.011578731	6.873326305	0.039035841	0.843377674	0.92611142
ALMS1	-0.247416229	6.87355991	12.12667114	0.000497058	0.007234003
RAB3GAP1	-0.039781262	6.87408098	0.671074405	0.412677271	0.642988779

GTF3C1		-0.039705016	6.874114918	0.5553406	0.456143702	0.67858644
SF3A2		0.028626534	6.87475948	0.272066548	0.601948871	0.783442907
TLK1		0.020965383	6.875229769	0.153026703	0.695659551	0.841440815
FBXO45		-0.100609097	6.875512333	3.254095035	0.071245254	0.225536872
IDH1		0.012567214	6.87628815	0.046697209	0.828913408	0.918867551
KAT2A		0.191628584	6.876697009	11.844113	0.000578439	0.008086123
NOC4L		0.151135709	6.877380418	4.672315524	0.030652573	0.131180259
CACTIN		-0.06994234	6.877915098	1.667393812	0.196607976	0.421687205
	07/mar	0.009108132	6.877940316	0.021375693	0.883760122	0.947996924
OTUD4		-0.014164532	6.87836429	0.07035312	0.790823331	0.897267524
PRPF3		-0.023564904	6.878380727	0.22672369	0.633963877	0.80403489
MLLT10		0.090548037	6.878852593	1.562472698	0.211303537	0.438848883
ILK		0.087468682	6.878972587	2.983517704	0.084116272	0.250204854
ROBO1		-0.268840618	6.87906166	14.06323549	0.000176765	0.003239391
MKLN1		-0.046171913	6.879208898	0.510520053	0.474913816	0.692302165
PROS1		-0.231492315	6.879239671	16.15890109	5.82E-05	0.001351775
ZNF638		0.018587782	6.879410149	0.086247741	0.7690027	0.884767126
PDHB		-0.039880474	6.879833443	0.594787021	0.440573955	0.6651417
MFGE8		0.158002727	6.880264444	6.01904086	0.014152336	0.079048435
SEC22B		0.017539917	6.880312033	0.115139923	0.734366613	0.864254228
RAP1GDS1		-0.060189426	6.880488639	1.533482626	0.215590516	0.444540058
SERPINE2		-0.164337155	6.880553537	6.923633145	0.008506398	0.055456729
CTR9		0.04191862	6.880700301	0.52438825	0.468975759	0.688269654
SNW1		0.049881401	6.880810158	0.870602159	0.350788659	0.587324724
AMMECR1		-0.098720031	6.881002789	2.333192726	0.126641894	0.32408887
NDST1		-0.138186317	6.881198864	7.261433335	0.007045104	0.048444291
HERC1		0.067826313	6.881942142	0.596317939	0.439986358	0.664967091
HOOK3		0.074425868	6.882858235	0.664170369	0.415091495	0.645466883
CDH3		-0.093309645	6.886566066	0.751422845	0.386026123	0.620596444
SKIV2L2		0.037950335	6.886990736	0.181940806	0.669710621	0.825134152
DACT2		0.172721429	6.888698608	2.520276199	0.112390898	0.300174668
GPX1		-0.033491541	6.888719961	0.098958085	0.753083573	0.874832943

ATXN1L	-0.165167593	6.888977919	4.775429245	0.028868649	0.126305136
TIMM23B	-0.066047803	6.889139661	1.265009257	0.260705032	0.495541331
MBNL1	-0.092682982	6.889951538	3.121128432	0.077283247	0.237584212
ATP8B2	0.014998013	6.890370327	0.069132315	0.792604343	0.897982957
YARS	-0.033455209	6.890776909	0.393449443	0.530491778	0.733450191
SCD5	-0.071146999	6.890868291	1.687083787	0.193985837	0.418684257
RABEP1	0.012127421	6.891306085	0.054738557	0.815013917	0.91213045
STOML2Z	0.114507003	6.892541287	2.006394438	0.1566372	0.370940798
GPCPD1	-0.186578382	6.892743413	6.753335959	0.009357257	0.059175121
PAXBP1	-0.004032565	6.892946992	0.005377791	0.941540793	0.974802494
POLD3	0.108122393	6.89323952	3.476842814	0.062233368	0.207718068
KCTD3	0.192133497	6.893289472	10.42640159	0.001242265	0.014020218
CRTC1	-0.099291879	6.894139045	1.87718847	0.170654041	0.389366645
XRCC5	0.087304814	6.894774483	2.280646966	0.130997219	0.331522491
NMT1	0.107291021	6.894848579	4.52398115	0.033422971	0.139198934
HOXA7	0.226680116	6.895358217	3.73471491	0.05329276	0.187258675
LCLAT1	0.02803487	6.896369955	0.278171274	0.597902341	0.780841953
ANKRD13C	0.029420256	6.896970667	0.392902681	0.530777562	0.733558252
KCTD10	-0.132794868	6.897355833	8.058323692	0.004529507	0.035576631
DNAJB11	-0.007854124	6.898049906	0.024762303	0.874960801	0.94316934
TGFB3	0.041683055	6.898569251	0.426736512	0.513594652	0.720825354
SEMA3F	-0.062472478	6.898605609	0.655852492	0.418028	0.647768221
COX7C	0.163738467	6.899077207	2.675244441	0.101919676	0.281768862
EDC3	-0.011967798	6.899118382	0.046500612	0.82926837	0.918867551
KLC1	-0.247899235	6.899293837	6.175920527	0.012950052	0.074430003
WDR82	-0.131257669	6.9000081	4.843106367	0.027756852	0.123439363
BCR	-0.04445583	6.900118541	0.481850041	0.487585517	0.701827677
TES	-0.082392838	6.901267766	1.724181731	0.189155653	0.412641691
EIF6	-0.016852895	6.902787598	0.077450462	0.780782667	0.891855827
CSNK1E	0.060086251	6.90302794	1.037586234	0.308383515	0.54628601
ZFAND5	0.039710924	6.903349134	0.156442628	0.692453048	0.839401545
ETFA	0.016297473	6.904257428	0.092585651	0.760915416	0.880263159

MSL1	0.151037499	6.904277309	6.527336429	0.010622891	0.064716652
SLC9A3R1	0.140708737	6.905374398	2.92276976	0.087337831	0.256233851
GTF2H5	-0.156863224	6.905864106	7.740825513	0.005398612	0.040251089
HNRNPKL	0.313469042	6.906270821	0.295743456	0.58656328	0.773141522
NUP133	-0.155631064	6.906777934	7.412347961	0.006477773	0.045954099
HSDL2	0.07908465	6.907360212	0.716878482	0.397169736	0.629797549
MLEC	0.001233845	6.908048516	0.000379083	0.984466136	0.992968171
SNX4	0.065233209	6.908858038	1.674136971	0.195705336	0.420677293
CD47	0.047697217	6.90935124	0.701576742	0.402254393	0.634499236
PFKFB4L	0.142971467	6.909371332	5.263986424	0.021771097	0.105025916
GUSB	0.096284715	6.910921939	3.429923006	0.064025086	0.211466004
PSMB3	0.146372133	6.910987574	4.868823571	0.027346137	0.122045207
RBBP5	0.061419761	6.911383093	1.379285573	0.240222878	0.472831035
INTS2	-0.095496404	6.911629788	2.497298258	0.114041789	0.303012698
PELI2	0.089211609	6.911634504	0.564354156	0.452511188	0.67549848
B3GNT5	0.053714689	6.913171644	1.17725799	0.277914922	0.51321028
ASH1L	-0.067051505	6.913353236	0.604258972	0.436957634	0.663113332
NXF1	0.097109598	6.913799286	2.520033989	0.112408162	0.300174668
SNX1	0.133945752	6.914619758	4.52721852	0.033359796	0.139041401
RDH10	0.206902719	6.915020441	2.039336514	0.153276489	0.365854976
TOM1L2	-0.098335569	6.915498419	3.596908618	0.057887121	0.197908285
RUFY3	0.004083012	6.915975403	0.003499302	0.95282873	0.979802073
CLTA	0.23327286	6.916026555	6.336612457	0.011827055	0.069779198
TAB2	0.247664201	6.916156496	15.18080518	9.77E-05	0.002029687
GGA1	0.002223253	6.917161919	0.001800061	0.966158205	0.985930745
RLIM	-0.239754996	6.917207708	16.95349581	3.83E-05	0.00096088
GOLPH3	0.084880126	6.917453628	1.360332939	0.243479789	0.476449034
GLRX5	0.062593671	6.917753453	1.102331646	0.293754974	0.530848821
LAMP2	0.085836871	6.918259097	1.775395578	0.182715741	0.405180824
THUMPD3	-0.015387417	6.918843281	0.092044082	0.761594437	0.88065738
THOC7	0.00267983	6.918901216	0.002212711	0.962481776	0.983899168
THBS1	0.187354799	6.918920321	2.483677758	0.115032986	0.304741342

MBTPS1	-0.031488444	6.92107612	0.286585441	0.592417015	0.777060342
SELENON	0.189015285	6.921796892	11.84912886	0.000576883	0.008071951
DAPK3	0.297490656	6.922710506	17.97167496	2.24E-05	0.000619041
EXOSC2	0.067324708	6.92331966	1.272344986	0.259327223	0.493892297
VPS8	0.015215976	6.924044142	0.051751488	0.820043106	0.91542069
WDR36	0.213116528	6.924379972	4.662008474	0.030837099	0.131764339
BMPR2	0.094221969	6.924466595	1.941890824	0.163463378	0.380007306
UTP14A	-0.000178128	6.925782174	1.04E-05	0.997428799	0.998885697
HACD3	0.066920287	6.926003749	1.351827883	0.244958818	0.478376139
CDK5RAP2	-0.104491376	6.926191511	1.905595173	0.167453376	0.385409109
KDM6A	-0.032769501	6.926775872	0.194893544	0.658874593	0.818680165
AES	-0.061690314	6.926873745	0.573894524	0.448715473	0.672092947
CUL1	0.033687764	6.927960592	0.470449523	0.49278044	0.705297727
LOC107051197	0.091092688	6.927970011	1.880083223	0.170324689	0.389184076
WDR75	0.071477353	6.928559786	2.016446799	0.155602884	0.368993436
NRP1	0.022741467	6.92903598	0.072777829	0.787334386	0.895403091
NCAPD3	0.061894976	6.929060463	0.783020872	0.376218839	0.61076174
DPYSL3	0.052698266	6.929736775	0.535367882	0.464358792	0.685317669
USP34	-0.025401958	6.930455493	0.140027504	0.708253672	0.848944796
UGGT1	-0.214386381	6.930877961	8.26799995	0.004034993	0.032673977
AHSA1	0.208248206	6.931208849	8.592316885	0.003375842	0.028756793
LSP1	0.033336248	6.931393262	0.314974123	0.574643784	0.765215138
SEC24A	0.0305116	6.93157573	0.268255452	0.604504438	0.785137502
CTDSPL2	0.040700103	6.931675115	0.615007115	0.432908748	0.659478488
HOXA4	0.046058894	6.932564488	0.575671532	0.448013961	0.671347758
NDUFAF1	0.03754201	6.932570329	0.40350564	0.525284329	0.729968216
ATP5D	-0.077939021	6.933064662	0.969369585	0.324837453	0.562889668
GNPTAB	-0.072263595	6.933327895	1.493844556	0.221620912	0.45116567
LRCH3	0.01069848	6.9335788	0.040154664	0.841178483	0.925252652
DHX29	0.106799484	6.934345179	1.444332551	0.229439524	0.460417972
EPB41L3	-0.058802235	6.934398237	0.688883719	0.406544329	0.637633886
ELF2	0.019096661	6.935383529	0.107463892	0.743050141	0.869560491

RALA	0.036756462	6.935518469	0.358084535	0.549571965	0.748347183
CYB5B	0.021241228	6.93610666	0.124780378	0.723906527	0.857408332
RBM8A	0.045036047	6.937268094	0.635595727	0.425310163	0.653696874
ATP5J	0.007890623	6.93799084	0.010571204	0.918108722	0.962365851
R3HDM1	0.137899387	6.938303122	6.653423944	0.009896547	0.061651442
HSPBP1	0.167804791	6.938446238	5.653009506	0.017425442	0.090940578
SP3	-0.108664453	6.939137784	3.619272014	0.05711386	0.196038499
BCAT1	0.428056013	6.93915016	30.24281827	3.81E-08	2.88E-06
FGD3	-0.141057464	6.940463257	6.653036272	0.0098987	0.061651442
SUCLG2	-0.203878559	6.941057546	8.399419644	0.003753408	0.031051952
MAPKAPK2	0.256483621	6.941084962	23.87963692	1.03E-06	4.87E-05
IER5L	0.312947127	6.941784972	11.68348916	0.000630572	0.008582541
PTPRA	0.040992626	6.942071205	0.751181812	0.38610232	0.620622611
LYPLA1	0.053154609	6.942352654	1.135016048	0.28670793	0.523035365
REV3L	-0.076250293	6.942865481	0.823185821	0.36425035	0.599973925
MAGT1	-0.014448801	6.943222534	0.06428008	0.799854691	0.902345328
MTF2	0.009759079	6.943349024	0.036110129	0.849288245	0.929809585
PLIN2	-0.001450113	6.944840018	0.000444965	0.983170508	0.992467303
UBE2E3	-0.085003121	6.945364606	2.991188159	0.083718721	0.24935223
GPD2	0.015761655	6.946878689	0.114292242	0.735309415	0.864745668
ATAD3A	-0.061169907	6.947345211	0.637090591	0.424766304	0.653410259
CRLF3	-0.14757065	6.947415801	6.522943361	0.010649162	0.06483625
PPP1R7	-0.040814723	6.947937205	0.520039706	0.470824745	0.689579099
RALGPS2	0.12387352	6.948301049	4.218057781	0.039995917	0.15669829
COQ8A	0.063133182	6.948567622	0.488473677	0.484608953	0.699310736
FAT4	0.132700929	6.949054808	1.014681068	0.313784006	0.551695247
PSME4	0.155119831	6.949388005	8.03177583	0.004596375	0.035964506
VPS16	0.028010808	6.949459962	0.34827538	0.555091023	0.752697304
NAA40	0.106067965	6.94971945	3.388756256	0.065642447	0.214698165
SAMM50	0.030959987	6.950635134	0.261824969	0.60886914	0.787473436
PHTF1	-0.055421846	6.951488913	0.98504811	0.320955679	0.558397726
C1GALT1	-0.003468755	6.951902572	0.004925951	0.944046318	0.975973541

HOXA3	0.040772586	6.952131454	0.241911506	0.622828921	0.79687815
DNAJC10	0.143933294	6.952160179	8.125501239	0.004364692	0.034545935
ATP1B1	-0.097806202	6.952610571	1.917412941	0.166142137	0.383862519
PAF1	0.019068428	6.952673259	0.117653769	0.73159323	0.862408819
CLTB	0.027782044	6.953506365	0.279493561	0.597033347	0.780192481
PKP4	0.100269439	6.954049387	3.193293042	0.073940924	0.230566212
TMEM30A	0.0571248	6.954501889	1.330805038	0.248662051	0.482857453
KERA	0.382844255	6.955729127	6.73064138	0.009477052	0.059703249
RAB8B	0.181906452	6.95609446	14.89990448	0.000113369	0.002255722
SEC23B	0.016564409	6.956489206	0.13282534	0.715520429	0.852809551
TRIL	0.049120877	6.958504594	0.435107636	0.509493293	0.717892027
TARDBP	0.14337667	6.958873007	7.269226985	0.007014599	0.048295072
TCTN3	-0.08943712	6.958882579	3.023779891	0.082052016	0.246021488
IL6ST	-0.02348963	6.959824855	0.108485373	0.74187515	0.868980128
WWC3	0.138904488	6.960041888	3.937739781	0.047214173	0.172938715
NDC80	0.155255898	6.960941633	6.999025092	0.008155412	0.053872864
PHF3	-0.043914417	6.961317679	0.66206594	0.415831535	0.64612917
POLDIP3	0.045227601	6.961681246	0.915198414	0.338738878	0.57576162
RHOU	-0.156783613	6.961726352	4.056873455	0.043991879	0.165511342
TFDP1	-0.075506916	6.961993136	1.35219635	0.244894515	0.478364107
SEC16A	-0.111292312	6.962122416	2.259956991	0.132757748	0.334537327
PIK3C2A	-0.075868065	6.962179249	1.872218002	0.171221265	0.389828743
KRT19	-0.091208822	6.963656374	0.242941549	0.622089599	0.796365672
PIK3IP1	-0.534953263	6.964039554	50.44032341	1.23E-12	3.02E-10
GTPBP4	0.079539231	6.964050683	1.000246161	0.317250951	0.555139665
CHD9	-0.034538536	6.964660921	0.154427018	0.694340139	0.840502295
POR	0.099944433	6.965089143	2.775356143	0.095725362	0.271112019
TP53INP1	-0.013434179	6.965187481	0.05871643	0.808536299	0.907749244
TFAM	-0.04638752	6.965507608	0.678523685	0.410095552	0.640360818
REST	-0.030827944	6.966634803	0.242768097	0.622213959	0.796462909
EPHB3	-0.128482325	6.96841651	3.974647207	0.046190128	0.170404892
PLPP3	0.046812334	6.968572822	0.467963527	0.493925536	0.706154037

XPNPEP1	0.196732594	6.969131084	13.55910076	0.000231168	0.004023954
PPP2CA	0.007913659	6.969667641	0.023608682	0.877884788	0.94495939
MAP2	-0.055765647	6.969734631	0.498778551	0.480037308	0.696439244
ERBIN	-0.001613185	6.972063563	0.000485267	0.982425005	0.992167118
TIMM17A	-0.030573522	6.97208305	0.271445578	0.602363711	0.783672902
LOC107050177	0.118223115	6.973830002	3.212444976	0.073080203	0.228879852
MTCH2	0.077701816	6.974263091	1.194650945	0.274393409	0.509389401
TAF2	0.000912705	6.977877801	0.000271913	0.986843647	0.993788997
BAZ2A	0.087013325	6.977975807	1.673324627	0.195813819	0.420708925
POFUT1	-0.128269756	6.978426968	3.967201126	0.046394828	0.170903715
MTPAP	0.082734195	6.978671995	2.348184712	0.125428992	0.321835968
ATP11B	-0.034770334	6.978970965	0.247685467	0.618709547	0.794077342
ARNT2	-0.081977465	6.979780588	2.000904642	0.157205358	0.371668702
CASP3	0.184735069	6.979796611	12.41386961	0.000426157	0.006415747
USP51	0.174997606	6.979916869	10.76146257	0.001036355	0.012348453
MFAP3	-0.069133498	6.980206252	2.072068602	0.150017923	0.361281572
RCOR3	0.196464475	6.980258917	17.70702589	2.58E-05	0.000695084
ELAVL4	-0.112378529	6.980850719	1.149186368	0.283719536	0.519831307
AAK1	-0.011146024	6.980910756	0.04256596	0.836544709	0.922733824
FAM109B	0.142741287	6.981268317	8.287738625	0.003991366	0.032401545
FAM18B1	-0.111241382	6.982846577	3.431596342	0.06396025	0.211296107
MCRIP1	0.090959403	6.9833205	1.480848654	0.22364179	0.452981985
PCIF1	-0.00762291	6.983586287	0.017320877	0.895293723	0.952048632
FAM192A	0.003621243	6.983791753	0.003501932	0.95281103	0.979802073
STAT3	0.008399225	6.98391217	0.028780527	0.865286779	0.937716936
COX6A1	0.046937267	6.984267039	0.457870115	0.498621064	0.709942858
CDKN2AIP	-0.109724796	6.984448453	3.316136206	0.068602883	0.220523627
LTA4H	-0.006423861	6.985090483	0.016444736	0.897961395	0.95334891
PAPSS1	0.22414051	6.985284185	11.0342843	0.000894422	0.011049335
VPS33B	0.125520759	6.985880768	5.464495118	0.01940666	0.097418119
SEM1	0.257139157	6.986869453	10.11062854	0.001474167	0.015958825
EIF1B	-0.090013609	6.987108495	1.682844799	0.194546874	0.419178842

MAP7D3	0.075648805	6.987568357	1.930497476	0.164704029	0.381697263
MRPS26	-0.00117128	6.988996223	0.000535036	0.981545907	0.991828264
SUSD6	0.014327012	6.989143939	0.063273609	0.801394739	0.903215783
PTPN11	0.000140703	6.98962956	7.61E-06	0.997799155	0.998973634
RTN3	-0.002642791	6.990466015	0.001904685	0.965189215	0.98555312
DCTN2	0.018546718	6.991293577	0.107242732	0.743305351	0.869735444
CRK	0.021187025	6.991336695	0.182575723	0.669168986	0.824683618
NDC1	0.179384081	6.991525376	8.016519808	0.004635256	0.036199874
GRSF1	0.278584833	6.992285632	17.82826285	2.42E-05	0.000657562
GOT1	-0.041707814	6.992821591	0.497583671	0.480563764	0.696466157
LMO4	0.052433977	6.99283961	0.817976047	0.365772587	0.60103734
FAM219A	-0.224039501	6.993362145	5.03486711	0.024841993	0.114470733
SLC25A36	-0.028167103	6.99347909	0.179446082	0.671849701	0.826371731
FBXW11	-0.044447134	6.994016526	0.746797562	0.38749204	0.62171007
HSBP1	0.014625224	6.994221454	0.075349002	0.78370215	0.893619214
CYP1B1	-0.163443882	6.994238407	1.254246431	0.262742979	0.497862243
NEURL1	-0.001384765	6.996259813	0.000372213	0.984607516	0.992968171
ATP13A3	-0.082474197	6.997217563	1.22442488	0.268494012	0.503585025
FMNL2	-0.168901032	6.997770209	8.699514174	0.003182949	0.027522555
SUCLG1	0.049881161	6.998780585	1.126094845	0.288609851	0.525165885
CDH5	0.026933078	6.999275664	0.113212224	0.736516288	0.865484219
MAU2	-0.048072553	6.999925979	0.889761932	0.34554183	0.582212863
GREB1L	0.24219803	7.000015201	8.01831156	0.004630672	0.036198437
CDC45	0.044070103	7.000110428	0.598676413	0.439083481	0.664502993
RAB39B	-0.043729468	7.001389892	0.615372599	0.432772073	0.659478488
TFRC	0.143155455	7.001870147	2.887256537	0.089282842	0.260256717
ZNF821	-0.185408022	7.002247654	1.911391057	0.16680882	0.384754574
ARPC1A	0.108922222	7.002547181	5.151057171	0.023232225	0.109557483
OLFML3	-0.1090259	7.002664636	1.756579829	0.18505169	0.40768852
SDK1	0.117693182	7.003208109	2.22915736	0.135427688	0.338414974
IGDCC4	-0.183060744	7.003507271	7.260228428	0.007049832	0.048456554
FBRSL1	0.085871788	7.003762804	1.221382758	0.269089456	0.504027293

RALGAPB	0.159634751	7.004725835	8.402598958	0.003746849	0.031033114
FH	-0.026118556	7.007210716	0.215378744	0.642583871	0.809098003
SYNRG	0.030171369	7.007319962	0.401658597	0.526233929	0.730547482
NUCB2	0.055852856	7.008553037	1.471862782	0.225052019	0.454830627
WAPL	-0.052796034	7.008638664	0.623411558	0.429782348	0.657247082
ARFGAP1	-0.046126878	7.009123794	0.541201073	0.461935389	0.683433449
PTPA	-0.029031529	7.009488802	0.412218977	0.520845384	0.725945349
JARID2	-0.49876713	7.010350488	13.3238934	0.000262046	0.004463726
CDC42BPA	0.130527756	7.010787774	4.376662008	0.036434283	0.1469787
MAGOH	0.030133573	7.013318905	0.206738814	0.649335585	0.81278085
CTCF	0.012112218	7.014048373	0.043119343	0.835500718	0.922151373
BACE1	-0.02843344	7.014129998	0.315928601	0.574064742	0.765001241
IKBKAP	0.547766081	7.014228978	28.80137897	8.02E-08	5.57E-06
CDK14	0.128690794	7.01426439	2.193464517	0.138597261	0.34388087
RC3H1	-0.043730871	7.01441848	0.61721715	0.432083294	0.659120293
HACD2	-0.096811957	7.018140904	2.75732643	0.096809902	0.272657543
TSR1	0.075997925	7.019071284	1.848819081	0.173920805	0.393551431
LOXL2	0.083310624	7.020134932	1.864674495	0.172086265	0.391115951
CDC42EP1	-0.222423976	7.020390147	13.1934315	0.000280932	0.004693134
FAM171B	-0.003369274	7.020557926	0.002133617	0.96315794	0.98421188
LMAN2L	-0.046503145	7.020633114	0.964695997	0.326006554	0.564056745
DBNL	-0.024251339	7.020812297	0.150907517	0.697669607	0.842758423
TOMM70	-0.064057613	7.02137539	0.845848973	0.357729302	0.59400061
C3H1ORF198	-0.195390286	7.021382966	13.92373933	0.000190379	0.003438729
NSA2	0.217220775	7.022365254	5.01907772	0.025069518	0.115289749
ATP6V1B2	-0.03420667	7.023188604	0.507354797	0.476286192	0.693258672
SMG7	0.031745301	7.023276329	0.39169053	0.531412123	0.733850867
05/set	-0.013970891	7.023285642	0.044047731	0.833764834	0.921335057
NPR3	0.416848718	7.023658972	8.640193092	0.003288266	0.028243841
PSMA6	0.012523152	7.023723609	0.056033784	0.812878237	0.910421412
GXYLT1	0.180547128	7.023805878	3.154284044	0.075727904	0.234450171
RNF145	0.120190284	7.024585811	5.750089066	0.016487821	0.087886975

MVB12B	-0.162227383	7.026356216	11.90029597	0.00056125	0.00788956
RNH1	0.114731133	7.026517677	4.943459022	0.026189575	0.118568272
AP1S2	-0.012758715	7.026594111	0.022217829	0.88150908	0.94696347
FAM98B	-0.170191937	7.027682435	7.902067705	0.004937832	0.037954122
COPG2	0.135961214	7.028045092	7.232203799	0.007160725	0.049014032
HK1	0.102632645	7.02956249	2.255068038	0.133177602	0.335082178
ACTR6	0.191836447	7.029799438	7.882330606	0.004992011	0.038241869
LEPROT	0.028232618	7.030046036	0.344564059	0.557206534	0.753148802
OXCT1	-0.051009039	7.030995663	0.302657505	0.58222118	0.770504224
DDX18	0.092325073	7.031267413	2.560599626	0.109557023	0.295660128
PWP2	0.018421699	7.031366231	0.064123576	0.800093318	0.902490783
IFRD1	0.025028256	7.032601578	0.209430229	0.647214351	0.811669244
RNF13	-0.038892721	7.033545135	0.661539134	0.416017096	0.646356465
ARL3	0.27456273	7.03367394	19.12332827	1.23E-05	0.000379729
NIN	-0.132586509	7.034130417	2.224361128	0.13584884	0.339004045
DNAJB6	0.097062959	7.034317927	3.658496838	0.05578394	0.19262599
JADE3	-0.013809047	7.034697238	0.067080383	0.79563616	0.900396293
DGUOK	0.106619068	7.035287902	2.510772765	0.113070474	0.301600688
PDS5B	0.118805704	7.035454593	5.30490337	0.021265479	0.103497031
RBM19	0.034075369	7.036152804	0.40775728	0.523110022	0.727807171
AP1B1	0.087928448	7.036901723	1.754789846	0.185275713	0.407836809
GET4	-0.027263436	7.037789827	0.171845508	0.678476828	0.830803409
CDK2	0.11283584	7.039227816	3.505902288	0.061150527	0.205073304
BOC	-0.023804026	7.040077168	0.060738292	0.805332823	0.905731091
PRDX3	0.045773193	7.040133849	0.579596822	0.446470397	0.670621395
SSR2	-0.159208212	7.040956016	5.926714176	0.014913024	0.081797935
DLG1	0.149569567	7.041495718	5.135712317	0.023438467	0.110257284
ARL6IP1	0.050064173	7.043143819	0.804608645	0.369719023	0.604263659
BMF	0.171110353	7.043344898	3.564647886	0.059022257	0.200200812
LIG3	0.036177508	7.044697332	0.406117877	0.523946524	0.728478246
EBNA1BP2	0.01158691	7.046261021	0.029894162	0.862730596	0.936001285
SCAF8	0.06445405	7.046635963	1.045008416	0.306659501	0.544776458

MAP7D1	0.041050336	7.047104139	0.473848244	0.491222103	0.704467466
SMARCA2	0.105596199	7.048725262	1.677319355	0.195281028	0.42026541
NDFIP1	-0.055177149	7.050260471	0.879035327	0.348466032	0.585043215
PHYKPL	0.165377505	7.051049246	4.573130353	0.032477168	0.136484247
MEAF6	0.032665748	7.051686703	0.483272666	0.486943661	0.701326735
BRE	0.015800219	7.051803376	0.050818929	0.821644355	0.916282016
SNRPC	0.08758115	7.052745825	2.876205191	0.089897651	0.261631737
SCARF2	-0.273698359	7.053882003	15.06180282	0.000104048	0.002132133
MRPS31	0.156815229	7.053898901	4.385169988	0.036252877	0.146512375
CBFB	0.201265494	7.053926308	11.53065138	0.00068458	0.009136065
CIZ1	-0.047252245	7.054519341	0.79943859	0.371261275	0.605641348
TK2	-0.26864988	7.055880422	10.3189062	0.001316743	0.014639869
RPRD2	-0.035297096	7.056985073	0.412590287	0.520657697	0.72580671
GLI3	0.034680174	7.057244987	0.138572456	0.709704328	0.849639454
AP1M1	-0.143866098	7.057640478	6.235288552	0.012522919	0.072917419
UPF2	0.068972707	7.059084356	1.392477014	0.237987236	0.470670135
SEC14L1	0.043140642	7.059086889	0.285247942	0.593281979	0.777327412
ANK3	0.140034685	7.059494443	2.992415601	0.083655292	0.249239152
WFIKKN1	0.041385369	7.059545195	0.358574655	0.549298903	0.748237869
TWNK	0.155303992	7.060124718	5.962708763	0.014611576	0.080754108
ABCA2	0.05228288	7.060277781	0.521747281	0.470097292	0.689065646
NUDCD1	0.046086543	7.062005217	0.559793601	0.454343416	0.677193923
BRD3	-0.059560303	7.062021085	1.278438992	0.25818948	0.492671516
TAX1BP1	0.068536142	7.063030594	1.921895921	0.165647806	0.383258527
WEE1	-0.024546848	7.063199752	0.106170015	0.744547376	0.870384134
M6PR	0.030630801	7.064732068	0.458921505	0.498128441	0.70960986
DCHS1	-0.107008409	7.064893815	1.755657111	0.185167132	0.407836809
ITM2B	-0.131846059	7.065195877	4.700098474	0.030160889	0.129695607
PGD	0.062989595	7.065651558	1.108960732	0.292307974	0.529494464
ZC3H7B	0.001122958	7.065670434	0.000295689	0.986280554	0.993448882
MYO3AL	0.168444167	7.066826346	1.677782072	0.195219424	0.420233023
DNAJC2	0.099836629	7.067175664	1.960444123	0.161465828	0.37728194

NCAPH	0.113879815	7.068100782	4.035855275	0.044543092	0.16669233
HIRA	0.02964556	7.068318572	0.402555654	0.525772353	0.730214726
TBL1XR1	0.038432445	7.069611293	0.532369161	0.465612514	0.686036347
UBE2S	0.194573246	7.069692074	10.15179426	0.001441609	0.015699465
C5H14orf166	-0.065775864	7.070296526	1.017594834	0.313090201	0.551100049
KPNA3	0.048751133	7.070316743	1.006099452	0.315839106	0.553298466
ALG12	0.249097045	7.070422055	17.79263895	2.46E-05	0.000667781
TEX10	0.109760322	7.070575983	3.67161738	0.055346452	0.191772134
KMT2C	0.002294263	7.07152416	0.000692902	0.978999688	0.991002256
ATP2B1	0.123335508	7.072993915	3.496426469	0.061501403	0.206069147
NDUFA4	-0.005476842	7.073505045	0.006543465	0.935528093	0.971960086
TSPAN5	-0.03432807	7.074139366	0.303635462	0.581612238	0.770401119
ZMYM2	0.017079952	7.074337378	0.112866648	0.736903806	0.865692306
LOC107057078	0.199992754	7.075442536	9.568727606	0.001979199	0.020004743
ZNF362	0.035474566	7.075507568	0.315156843	0.574532846	0.765147438
USP14	0.051424964	7.075674014	0.769896767	0.38024899	0.614757038
CCNG2	-0.178617386	7.076064524	6.358466802	0.011682245	0.069288863
DDX42	0.015055977	7.076434775	0.10138915	0.750168926	0.873555895
RSL1D1	0.16166313	7.076503592	5.054186857	0.024566507	0.11374279
SAR1A	-0.01790709	7.076797557	0.117475859	0.731788414	0.862408819
ZRANB2	0.311722962	7.078135385	22.06781314	2.63E-06	0.000106931
PCNT	-0.099178805	7.078606662	1.835396342	0.175491484	0.39519808
CREB3L2	-0.004964403	7.079164524	0.003986608	0.949655347	0.978557126
RFC1	-0.02641302	7.079428922	0.198245613	0.656140678	0.816485578
CLINT1	0.022603583	7.079790092	0.170220184	0.679916197	0.832132271
AUTS2	0.025791839	7.079984869	0.098263245	0.753923847	0.875422686
TMEM263	0.1270391	7.080220329	7.02555661	0.008035449	0.053336266
STK4	0.042236317	7.080255404	0.696048514	0.404114645	0.635646128
ATP6V1A	0.106184521	7.080948653	4.373905956	0.03649325	0.147108386
SSBP4	0.118429511	7.081949045	2.753943404	0.097014889	0.272978797
KIF21B	-0.00256979	7.083156798	0.001546391	0.968631934	0.987147683
KRT8	0.110019535	7.083404721	0.509577584	0.475321774	0.692572543

MFN1	-0.262332154	7.083636721	18.16370961	2.03E-05	0.000569199
SLIT2	0.013756928	7.083913801	0.031732226	0.858616631	0.933917114
ATG13	-0.0638838	7.083959282	1.294593298	0.255203187	0.49032844
HMGCS1	-0.109387097	7.084355236	1.919811339	0.16587746	0.383520247
ZMYND8	0.117311614	7.085299303	1.453015975	0.22804463	0.458364104
PTPRZ1	0.28938185	7.086377443	5.829723846	0.015757597	0.085097229
UNC119B	0.122373104	7.087142356	5.326242509	0.021006626	0.102570931
UBE2Q1	0.054993197	7.087320115	0.876201219	0.34924425	0.585991041
RALBP1	0.078218063	7.088364353	2.307332109	0.12876489	0.327940916
GNAI3	0.078838329	7.088609738	2.781385434	0.095365641	0.27051226
KIF2C	0.027511801	7.088886865	0.19002572	0.662895212	0.821016009
ZNF628	-0.032815521	7.089200399	0.130500868	0.717913286	0.854176517
CREB1	-0.153693791	7.089267658	6.098033195	0.013533242	0.076804448
RANBP3	-0.028506135	7.090231796	0.361015892	0.547942576	0.747256347
RAB3IL1	-0.014744512	7.092235527	0.081693823	0.775014984	0.888887681
PTPN2	-0.06876716	7.092299611	1.026910779	0.310885343	0.548532953
TP53BP1	0.044881593	7.095661065	0.798497921	0.371542846	0.605860424
GORASP2	0.027203908	7.096800538	0.308988945	0.578301273	0.767848579
SETD5	-0.057959124	7.096915156	1.116627392	0.290645824	0.527703524
PLEKHG4	-0.076593667	7.097466496	0.828151058	0.362807715	0.599097263
GTF2A1	0.061193943	7.097845466	1.284017136	0.257153451	0.492030236
PITPNB	0.167288189	7.098193061	13.01103938	0.00030966	0.005060035
RCN1L	0.207377604	7.098291537	18.1993441	1.99E-05	0.000561523
GLI2	-0.074271483	7.099420181	0.83506912	0.360810824	0.597237914
AMOT	-0.086373752	7.100548846	1.648090911	0.199218968	0.425071073
ZNF335	-0.138628753	7.101287953	5.450741432	0.019560024	0.09785959
NAA25	-0.025534087	7.101800089	0.205027809	0.650692788	0.813613209
TTK	0.067664959	7.101829986	1.126738939	0.288472	0.525139497
NUP85	-0.087247694	7.102358197	1.924212304	0.165393041	0.382814912
PTGFRN	-0.151349807	7.10248685	5.115865157	0.023708042	0.111239189
ZYX	0.318093973	7.10454972	27.2789473	1.76E-07	1.11E-05
SAAL1	0.098250087	7.10467326	2.935804394	0.086635498	0.254978044

CCNF	-0.08804559	7.105459876	1.709016249	0.191113057	0.415159753
TMEM184B	-0.028707737	7.106292353	0.359198237	0.548951852	0.747916769
CXCL14	-0.044696819	7.106566343	0.302756791	0.5821593	0.770499657
LOC428335	-0.141592373	7.10724813	8.730890203	0.003128637	0.027195839
NOL6	0.033503435	7.108568037	0.155419856	0.693408839	0.839776437
PLXND1	0.053776633	7.108725421	0.443999807	0.50519821	0.715440088
LIMD2	0.008228489	7.109099846	0.00704177	0.93312381	0.970823994
SESTD1	-0.041534993	7.109165998	0.62589248	0.428865994	0.656281032
PATZ1	0.063780575	7.109579492	1.02084897	0.312317716	0.550288717
SBNO1	-0.07977268	7.110259364	2.123545148	0.145050452	0.354020349
FAM133B	0.116362422	7.110285188	4.085371545	0.04325594	0.164003801
RNASEK	-0.87365125	7.111135405	139.7588773	3.01E-32	8.24E-29
OLA1	0.024400745	7.111894174	0.190715057	0.662322147	0.820794617
SSR3	-0.006969615	7.113224705	0.021276054	0.884029435	0.948083049
HAUS6	0.184993747	7.113682034	5.257672993	0.021850215	0.105160948
ZNF212	0.019281051	7.113909349	0.132191952	0.716170087	0.853085451
RAI14	0.036339743	7.114269688	0.226949075	0.63379533	0.80403489
AIFM1	0.178119671	7.114762988	11.31442746	0.000769072	0.009898861
PLRG1	0.063788828	7.114890114	1.497807778	0.221008979	0.450421446
PRKACB	-0.050156407	7.115024277	1.002811454	0.316631173	0.554331945
DLGAP4	0.085763882	7.11671323	3.251859702	0.07134247	0.225714352
AMER1	-0.141660861	7.116942586	5.406252971	0.02006474	0.099297835
HECTD4	0.065742556	7.117120939	0.95538667	0.328351946	0.565814483
FAM89A	0.028360489	7.12072718	0.229647055	0.631785655	0.802844463
LOC107052011	0.090742761	7.122113359	3.882422949	0.048794092	0.176579455
QRICH1	-0.060776003	7.12217343	1.013649035	0.314030228	0.551873406
PTPRN2	0.014163333	7.12220721	0.031743502	0.858591777	0.933917114
EP400	-0.060029761	7.122720434	0.764494494	0.381925632	0.616619532
NCOR2	0.036427451	7.123030053	0.274066482	0.600616894	0.782591357
MYCBP2	0.078548379	7.123477744	1.275206712	0.258792172	0.493272928
FNBP1	-0.075781218	7.124068523	1.369608512	0.241879158	0.474797763
PHLDB2	0.078088874	7.124107505	1.773107168	0.182998009	0.405516403

HNRNPLL	0.209400541	7.124236684	15.59176848	7.86E-05	0.001726699
POLA2	0.074334495	7.125281736	1.859374622	0.172696995	0.3920167
SAE1	0.159720456	7.12681002	8.40558748	0.003740695	0.031033114
GLRX3	0.244606995	7.12783168	15.57941104	7.91E-05	0.001735703
RBM27	-0.093781865	7.128269209	3.037974722	0.081337337	0.24464664
ELMO2	0.164571697	7.129605033	9.183841415	0.00244161	0.022880678
TMEM94	-0.00467418	7.129626764	0.004787277	0.94483826	0.976406052
PPP4R1	0.103729652	7.129748979	2.261840511	0.132596389	0.334233087
COX6C	-0.103935913	7.131226474	1.787447851	0.181237426	0.403338029
AKAP8L	-0.0621343	7.131958281	1.274703885	0.258886086	0.493394781
NDUFV1	0.134231084	7.131975689	6.999665052	0.008152497	0.053872864
FLRT3	-0.165609566	7.132169673	2.969910593	0.084826542	0.251493697
NR2F1	0.371715864	7.132556518	18.3627529	1.83E-05	0.000522824
CTPS1	0.040706657	7.133033021	0.494002794	0.482147162	0.697256981
POLR2B	-0.093260549	7.133580224	3.066570078	0.079917924	0.24221431
LAMP1	0.03161783	7.134438391	0.43616255	0.508980468	0.717892027
GST2L	0.146308737	7.134567808	3.799434778	0.051269887	0.182528341
ANXA6	-0.126872789	7.135036318	1.787782025	0.181196635	0.403338029
PPFIA1	-0.104921974	7.135325078	4.107835502	0.042684959	0.162808308
DYSF	0.163433174	7.135390068	4.986038658	0.025552641	0.116732013
TSC2	-0.026466518	7.135730186	0.225531961	0.634856787	0.804262718
SLC25A20	-0.175995703	7.136285851	10.64433585	0.001104085	0.012894054
CASC3	0.073095521	7.13733205	1.825012979	0.176717725	0.397067317
DNAJC8	0.042009735	7.137676919	0.664506789	0.414973372	0.645405182
NAT10	0.239639724	7.138311972	12.75044219	0.000355925	0.005593836
SLC12A4	0.017486062	7.139503102	0.059091237	0.807938085	0.907527084
WHSC1L1	0.006588437	7.141595362	0.008342844	0.927223094	0.967560151
STRAP	-0.058905111	7.14350601	1.482880851	0.22332433	0.452729068
HARS	0.112405589	7.144488679	4.536931319	0.033171006	0.138567052
GPBP1L1	0.051282098	7.144533287	0.79993268	0.371113498	0.605520338
JMJD1C	-0.089974438	7.14487659	1.784656782	0.181578539	0.403693205
SASH1	-0.054395062	7.145820897	0.6556082	0.418114709	0.647841576

C4BPA	-0.151274911	7.146644122	3.902664392	0.048209594	0.175137561
AAGAB	-0.159164843	7.14667708	6.357053311	0.011691556	0.069288863
BAZ2B	-0.272342314	7.14717014	12.31548004	0.000449217	0.006689474
URM1	-0.180365499	7.147397097	1.847622504	0.174060165	0.393575338
ZMAT2	0.066865752	7.148471748	1.765449537	0.183946239	0.406559484
MED12	-0.024695082	7.148622022	0.188662924	0.664031792	0.821860352
CALD1	0.019519886	7.149541281	0.053137816	0.817690511	0.913838618
TTLL12	0.194233368	7.150425489	11.57741486	0.000667577	0.008995963
CEP170	0.03995464	7.152160296	0.459171361	0.498011494	0.709566121
WDR3	0.058889778	7.152488202	0.850373117	0.356446806	0.592942775
YEATS2	-0.119292634	7.152488552	4.396876832	0.03600481	0.145925897
NUDT21	0.047542347	7.152873915	0.481966563	0.487532892	0.701827677
RANGAP1	0.097754526	7.153215056	3.063535149	0.080067297	0.24245627
ATP2B4	-0.005581617	7.15331427	0.006397806	0.936248165	0.972169089
DCAF13	-0.055404156	7.154336469	0.943729276	0.331320609	0.569160583
CCDC88A	-0.021079683	7.155716593	0.0739156	0.785718723	0.894554873
PI4KA	0.084326716	7.156139276	1.989361582	0.158407648	0.373384593
UBE4A	0.105931414	7.156191335	4.527282793	0.033358543	0.139041401
FEM1B	-0.010746092	7.157359172	0.046785205	0.828754783	0.918760356
LDLRAD3	-0.24388306	7.157467601	7.209843647	0.007250481	0.049443293
CCNL2	-0.031211132	7.158362082	0.203366908	0.652016791	0.814464154
COL26A1	0.006968403	7.158968801	0.007939813	0.928997963	0.96849097
PHF12	-0.1435537	7.159703712	7.127505088	0.007591028	0.051297892
FLII	-0.017599498	7.160535634	0.127787411	0.720737249	0.855792151
ZNF512	0.019650134	7.161944217	0.134301812	0.714012815	0.851753
PXDN	-0.139540177	7.162278915	3.366362844	0.066540507	0.216452808
MYBL2	0.108034028	7.162732748	3.58406487	0.058336241	0.19889328
SRPRB	-0.068082847	7.164000849	1.491111967	0.222044012	0.451580054
MROH1L1	-0.108395613	7.16542422	3.147739342	0.076032232	0.235126926
JUP	-0.051418328	7.166304762	0.673423001	0.411860733	0.642081322
GTPBP1	-0.008209744	7.166443028	0.032122981	0.857758011	0.933726046
ATP5O	0.093401944	7.167562318	2.533635825	0.111443165	0.299248904

SCAP	-0.151523453	7.168647186	7.646026675	0.00568975	0.041755635
GEMIN5	0.204872995	7.168993392	11.89963003	0.000561451	0.00788956
NFYA	0.319100067	7.169125587	19.23455447	1.16E-05	0.000365813
PDCD4	0.098983807	7.1694249	2.69482659	0.100674442	0.279311741
MGA	-0.011102222	7.170887858	0.024418785	0.875824055	0.943751684
YBX3	0.045967123	7.174808992	0.795992385	0.372294288	0.606725019
RACGAP1	0.154236665	7.174957623	6.891793354	0.008659235	0.056173972
FMR1	0.130836985	7.175232013	6.666121477	0.00982628	0.061386271
SLTM	0.026151652	7.175508072	0.221120202	0.638187703	0.806386086
STAU2	0.050040371	7.175715057	1.014140836	0.313912863	0.551828937
SAP130	0.025432152	7.176620932	0.152634218	0.696030609	0.841712624
PSMD6	0.091292876	7.176815584	2.170739013	0.140658625	0.34739544
FEM1A	0.118971641	7.177500411	6.020295061	0.014142282	0.079034921
QSER1	0.057872159	7.177579378	0.835878618	0.360578155	0.597143069
CHCHD3	0.125791811	7.178003756	3.889288659	0.048595002	0.176052566
IWS1	0.10443601	7.17879028	3.9375137	0.047220519	0.172938715
SYNGR1	-0.084693148	7.179362513	1.882142673	0.170090819	0.388944472
SGTA	-0.030710798	7.179384659	0.443382458	0.505494389	0.715440088
ATP5H	0.033002038	7.180182492	0.230095184	0.63145326	0.802608017
USPL1	0.178059477	7.181068201	8.085686331	0.004461623	0.035221456
TENM3	-0.072097473	7.181929281	0.929399107	0.335019115	0.572334667
XPO6	-0.018098007	7.182210549	0.159224729	0.689871295	0.83840108
MCAM	0.15361407	7.183619456	4.807351054	0.028338574	0.124748861
DDA1	0.031251813	7.184025903	0.441349329	0.50647191	0.715978975
ATXN2	-0.068284246	7.184593363	1.886578349	0.169588355	0.388065135
YTHDF1	0.058195314	7.185166759	0.83904214	0.359670863	0.596167286
PSMD12	0.035540026	7.186157681	0.331596795	0.564720347	0.758631179
CDCA7	0.040273014	7.188696207	0.439314643	0.507453434	0.716891343
PRRX1	-0.430231714	7.188744813	2.427990137	0.119185625	0.311642197
BLCAP	0.056430399	7.190670095	1.343679469	0.246386127	0.479919939
TTYH2	-0.05713735	7.190852664	0.614535333	0.433085269	0.659609229
TMEFF1	0.037835297	7.191151213	0.229242993	0.632085706	0.803037455

RHOB	0.040424094	7.192075562	0.205254822	0.650512325	0.813573027
ZNF608	0.104622406	7.192276559	1.392344952	0.238009492	0.470670135
MED24	-0.016598714	7.192399185	0.098961199	0.753079815	0.874832943
BID	0.290402251	7.192565384	25.97113046	3.47E-07	1.99E-05
TXNRD3	0.152894247	7.192617732	8.163353798	0.004274525	0.034028696
PABPN1	0.022011683	7.192680501	0.123335734	0.725444404	0.858483911
LIMA1	0.013025314	7.193353333	0.046412047	0.829428533	0.918867551
LIG1	0.018452582	7.19349311	0.069482212	0.792092175	0.897745396
DYNC1I2	0.024330742	7.195459476	0.210361866	0.646483925	0.811495384
ITGA6	0.095601618	7.195772107	1.71182147	0.190749215	0.414524343
PGLS	-0.123107445	7.195920852	5.423496394	0.019867536	0.098737634
GBAS	-0.023694823	7.196746737	0.179829351	0.671519935	0.826344165
ZNF711	-0.154717157	7.197674148	6.30602109	0.012032859	0.070613657
CSNK2A2	-0.067225364	7.198580559	1.193025863	0.274720053	0.509698778
PTCH1	0.267531106	7.198687565	5.255597884	0.021876284	0.105197013
ETF1	0.082012305	7.199574509	2.348735285	0.125384695	0.321822673
SRGAP1	-0.033518127	7.199664213	0.33775204	0.56112967	0.75591721
ABCA3	-0.109453409	7.199828282	3.548125298	0.059612766	0.201339914
LSM7	-0.039798708	7.200517283	0.312858247	0.575931524	0.765833434
CHD2	-0.148341507	7.200795787	3.007266597	0.082891964	0.247817455
LOC101750560	0.058575027	7.201083571	1.102949856	0.293619645	0.530818639
ZNF644	-0.024814394	7.201152743	0.226170666	0.634377874	0.80403489
SRP72	0.026235109	7.202152875	0.291278126	0.589402639	0.775145494
GTF2F2	-0.236616222	7.203492323	15.2815122	9.26E-05	0.001951394
HNRNPD	-0.061973459	7.203799509	0.804418101	0.369775704	0.604296277
AK2	0.065938016	7.205362496	1.837456762	0.175249323	0.394944042
SLC35B4	0.062804506	7.206067211	1.23853351	0.265753869	0.500971465
TWF2	-0.166663274	7.206317027	7.666872703	0.005624385	0.041464723
ARHGEF17	0.070717021	7.20651591	0.962249821	0.326620687	0.564315771
RGL1	-0.147995993	7.206610949	8.878148718	0.00288604	0.025771652
UBP1	-0.077565047	7.208460707	2.299765863	0.129393502	0.328931413
NOC2L	-0.038355909	7.208876418	0.340264219	0.559676749	0.755123066

SRGAP2	0.201026358	7.209174438	9.642688914	0.001901067	0.019414044
FNDC3AL	0.034572304	7.210564836	0.307459956	0.579243057	0.76846896
ZFR	0.108989413	7.210644362	3.360822289	0.066764723	0.216817351
XPOT	0.072696345	7.211792097	1.707285217	0.191337979	0.415364966
TRIM1BL	-0.012292662	7.212686965	0.041002921	0.839532283	0.923898005
WDR33	-0.059388377	7.21289919	1.249003064	0.263742972	0.498838001
AMD1	0.070961764	7.21310374	1.086574988	0.297231329	0.534295555
ATP1B3	-0.001615935	7.21313368	0.000997224	0.974807907	0.989932647
CASK	0.029373446	7.213427896	0.260338539	0.609887669	0.787717486
STRN3	-0.041215272	7.214682218	0.57154956	0.449643819	0.673115815
NPM3	0.048840858	7.215388491	0.295038411	0.587009742	0.773482167
DPY19L1	-0.022531343	7.216101488	0.171366359	0.678900325	0.831198278
PPM1B	0.027450941	7.217237182	0.273476421	0.601009236	0.782591357
UBE2L3	0.087763769	7.218497417	1.992786487	0.158049832	0.372754764
KIFC1	0.029091767	7.21938633	0.057150626	0.81105753	0.909337575
CRB2	0.217633178	7.219932765	6.81530026	0.009038011	0.0577331
NCOR1	0.009891158	7.220102081	0.030982988	0.860278346	0.934628297
LOC107056274	0.124028027	7.221473678	3.274196277	0.070377391	0.223996125
RCN2	0.039091979	7.221727588	0.471557819	0.49227137	0.704866464
HMGN3	0.087938433	7.222223668	2.828816962	0.09258654	0.265995181
NECAP1	-0.087734317	7.222284849	3.254642045	0.071221486	0.225505013
ICE1	-0.109044113	7.222772894	2.353094153	0.125034612	0.321282385
WBP11	0.017404143	7.22307527	0.082243985	0.774279154	0.88847723
CENPF	-0.012674576	7.223567111	0.039830601	0.841812159	0.925494032
ARHGAP21	0.079298037	7.224457376	1.818905369	0.177443629	0.398014574
KLHDC2	0.094895052	7.224753117	2.821125969	0.093031122	0.266694618
GPATCH8	-0.056599172	7.224848385	0.410242962	0.521846213	0.726724732
EYA3	0.029267766	7.225093217	0.305101328	0.58070188	0.769731709
PPP1R2	0.046805257	7.226604039	0.790041164	0.374087679	0.608803556
LATS1	-0.089584424	7.226782735	2.029666985	0.154254404	0.36713461
RAB10	0.057546683	7.227711631	1.569618805	0.210262381	0.438068813
DCTN4	-0.102124387	7.228918784	3.133997494	0.076675513	0.236375962

GOLGA7	0.068516616	7.229886268	1.827774118	0.176390682	0.396589338
WDFY3	-0.093945556	7.23110873	1.524519043	0.216936906	0.446100574
ARID3A	0.039390179	7.231612749	0.522284798	0.469868676	0.688976035
LOC107051290	-0.320714692	7.233011964	9.730026604	0.001812819	0.018690433
CCNYL1	0.113523803	7.233046847	4.435325036	0.035202516	0.143637893
EZH2	0.143113626	7.233313573	7.404995055	0.006504302	0.046018535
KAT7	-0.029074801	7.233391257	0.087703789	0.767116864	0.883957143
TBL3	-0.256689053	7.234911021	18.54386032	1.66E-05	0.000487865
GALNT1	0.161499544	7.23503615	9.641903662	0.00190188	0.019414044
LOC107050163	0.346761762	7.235556282	20.348979	6.45E-06	0.000228344
CEP131	0.009778749	7.235768787	0.012658988	0.910417322	0.95894146
ANKFY1	0.043076627	7.235777366	0.469211693	0.493350051	0.705551458
PLXNB2	-0.010361248	7.2359168	0.02783456	0.867498364	0.93881523
MLF2	-0.017038457	7.236106231	0.122098715	0.726769317	0.859246236
CORO1C	-0.010112868	7.237227439	0.043373697	0.835023208	0.921947852
SDC1	-0.013632584	7.237500124	0.013070553	0.908978962	0.95829261
CDK12	-0.02723786	7.238533873	0.275014496	0.599987669	0.782375552
ANLN	-0.138214168	7.238830753	2.861650829	0.090714356	0.26275387
TCF20	0.067464309	7.239199769	1.169222848	0.279560982	0.514855494
ADRM1	-0.02005862	7.23934499	0.127697583	0.72083131	0.855792151
SALL4	0.092841418	7.242268695	0.875103601	0.34954628	0.586139207
SAP18	0.017234928	7.242384231	0.099886083	0.751966382	0.874401564
VPS35	0.071686405	7.242808075	1.576450455	0.209272718	0.437002866
SIN3A	-0.241777655	7.24550974	16.32722589	5.33E-05	0.001258152
ARHGAP5	0.010701427	7.245853589	0.026258893	0.871269663	0.940731028
CDK11A	0.125684315	7.245926992	5.086069111	0.024118802	0.112429145
AP1G1	0.070993783	7.246516073	0.83270518	0.361491463	0.597823318
XPA	-0.137821148	7.247490543	1.677624187	0.195240441	0.420233023
HH3L	0.052369516	7.247740169	0.629180253	0.427656162	0.655213795
PFKP	0.003953975	7.24872654	0.00304251	0.956011828	0.981041173
NDUFS2	0.083735767	7.248777719	1.750331401	0.185835076	0.408503379
NXN	0.032778957	7.249115027	0.110459141	0.739622015	0.8676467

UBE2I	0.101925783	7.249394827	3.027073235	0.081885602	0.245804295
RCOR1	-0.027592022	7.249798619	0.307745408	0.579066999	0.768307327
MYH15	-0.351550155	7.252563262	3.474687849	0.062314478	0.207858446
OTUD6B	-0.017462403	7.256789114	0.100810459	0.75085921	0.873913447
FRAS1	-0.034833197	7.259488098	0.135900493	0.712390977	0.850434818
PRKCI	0.005500838	7.259579153	0.014853177	0.902999115	0.955614537
ADSS	-0.026445272	7.261034989	0.175948012	0.674878832	0.828679291
FRMD4A	-0.055888875	7.262142962	0.70194276	0.402131668	0.634366465
GLO1	0.151462874	7.262413658	4.134368212	0.04202075	0.161215073
VANGL2	0.064172161	7.263096406	0.629357642	0.427591033	0.655213795
LONP1	-0.138539879	7.263859321	5.867281201	0.015424856	0.083547074
DNM1	-0.076911327	7.264204565	1.015635445	0.313556536	0.55158832
LOC407092	0.412861455	7.265305706	19.71362009	9.00E-06	0.000299042
CUL3	-0.000648187	7.265442676	0.000130275	0.990893303	0.995986157
PRC1	0.001302433	7.26569452	0.000491552	0.982311572	0.992139511
MFN2	-0.066361288	7.265827782	1.687633092	0.193913274	0.418635912
IMPAD1	-0.078999524	7.26778122	1.876520968	0.17073009	0.389486154
ROR1	-0.127141637	7.267913937	1.685938582	0.19413722	0.418721657
SENP6	0.064715091	7.26867614	1.636793159	0.200766028	0.427208714
MARK3	0.069972559	7.269142795	2.091181107	0.148151493	0.358241413
SFSWAP	-0.040531513	7.269526664	0.695688513	0.40423622	0.635646128
RAB11FIP4	-0.0917892	7.270218539	0.622426124	0.430147149	0.657590258
NUP107	0.063889878	7.270372945	1.048779882	0.305788258	0.543913716
VAPA	0.224833652	7.270480774	18.00003669	2.21E-05	0.00061194
EDC4	-0.062218274	7.270490182	0.745030676	0.388054123	0.621997915
HSPH1	0.400154876	7.270784931	15.3829871	8.78E-05	0.001870904
COL11A1	-0.074574117	7.271735928	0.481172979	0.487891485	0.702056988
AAAS	0.306289994	7.272044086	25.09461354	5.46E-07	2.89E-05
NUP93	0.105933363	7.273059222	2.641320881	0.104116946	0.286065178
ZC3H11A	0.080065579	7.273373301	1.977962835	0.159605182	0.374636459
NKTR	0.092401949	7.275092408	2.224473306	0.135838973	0.339004045
DDX21	0.068459539	7.276002678	1.28166409	0.257589855	0.492464397

TPD52L2	-0.012075139	7.276993541	0.065654334	0.797772459	0.901282145
KDM3B	0.013271272	7.277848069	0.070312519	0.790882295	0.897267524
SOD1	0.11840539	7.279639405	4.035076814	0.044563647	0.166695796
RBM22	0.03730061	7.280953411	0.565107602	0.452209601	0.67549848
AKAP9	0.044511844	7.281552812	0.360464516	0.548248369	0.747425593
PTCH2	0.130460667	7.28263966	1.209310023	0.271468813	0.50640736
NBR1	-0.039581658	7.284396595	0.547873021	0.459188081	0.681513473
HSPA4L	0.256791309	7.286810369	14.40789248	0.000147184	0.002796669
CLIP2	-0.209657333	7.287106337	9.369401399	0.002206376	0.021546535
PCF11	-0.180747798	7.287432702	8.317785058	0.003925876	0.032075618
PSMA5	-0.029530803	7.287471483	0.279369155	0.597114993	0.780221347
ACTR2	-0.033903586	7.287785999	0.427434874	0.513250308	0.720608688
RPL22L1	0.238672467	7.288599072	3.493092278	0.061625372	0.20628511
RAB11FIP2	-0.098003767	7.289103075	2.071840835	0.150040325	0.361281572
TSN	-0.139231233	7.290154733	7.341433199	0.00673831	0.047102329
BTG1	-0.179347416	7.291273812	11.25147238	0.000795599	0.010162419
LOC427439	0.024986909	7.291821619	0.108447534	0.741918566	0.868980128
BDH1A	0.129187822	7.291999312	6.231149171	0.012552225	0.073010557
POU2F1	-0.053270965	7.292181431	0.431267965	0.511367432	0.71900804
SMG1	-0.005016457	7.29430717	0.005259907	0.942183936	0.975255499
POGZ	-0.063995444	7.295912471	1.89696085	0.168418891	0.386679623
HBE	0.594628165	7.297557066	4.565793092	0.032616569	0.136914704
TMSB15B	0.282731708	7.297833306	8.931364448	0.002803149	0.025177844
LOC776273	-0.209138973	7.300009954	15.30732441	9.14E-05	0.001931576
CDC27	0.018426871	7.300220193	0.135691652	0.712602226	0.850454908
SNRPE	0.069490087	7.300579682	1.219466621	0.269465353	0.504156042
TNRC6C	-0.227717225	7.300799205	6.771227761	0.009263908	0.058742818
SETDB1	-0.120412084	7.301664343	5.565854176	0.018314073	0.093589465
NAV1	-0.061694636	7.302149408	0.900196925	0.342728914	0.579842456
ITSN1	0.173063335	7.302736089	10.49524339	0.001196822	0.013647757
GIT2	-0.014340002	7.303261516	0.089940084	0.76425334	0.881657566
TBRG4	0.151465771	7.304755284	5.42912611	0.019803587	0.098568669

PHF6	0.084860662	7.304867793	2.614187545	0.105911757	0.289498
TRAPPC3	0.084206628	7.305461324	2.56018723	0.109585604	0.295660128
ARPC2	-0.037105357	7.305526193	0.505669205	0.477019658	0.693600685
TNRC6B	0.012777833	7.306577112	0.028184225	0.866676441	0.93840635
ADAM10	0.085852871	7.307232455	2.775810415	0.095698208	0.271081771
C5H14orf4	-0.144845311	7.307328906	3.469292701	0.062518037	0.208077327
MSI2	0.122965767	7.307330664	7.694256523	0.005539682	0.040950348
WHSC1	-0.079743677	7.307435513	1.592573747	0.206958802	0.434352468
ERLIN1	-0.053380223	7.308561345	0.794091513	0.372865802	0.607475918
GART	-0.153166723	7.309088175	3.57624491	0.058611501	0.199431814
NOMO3	0.149183538	7.313476711	7.443935293	0.006365056	0.045416287
KDELR2	0.052028234	7.314295468	1.279352225	0.258019513	0.492541889
USP3	-0.02458433	7.317139572	0.185746702	0.666480432	0.823590831
PRPF4B	0.020600335	7.31759472	0.150645012	0.697919722	0.842758423
TRMT1L	0.044766583	7.318230645	0.559505264	0.454459648	0.677305815
CACHD1	0.065357528	7.318956579	1.447538575	0.22892332	0.459718481
PSMA2	0.110615853	7.321047729	3.689009377	0.054772138	0.190504233
OTUB1	0.009506146	7.321176353	0.028570842	0.865773741	0.937997557
LMNB1	0.305475877	7.321893538	12.50829079	0.00040515	0.006189294
LOC107057625	0.061611328	7.323874654	1.286758173	0.256646239	0.491406353
TGIF2	-0.035735724	7.326331573	0.509235856	0.475469835	0.692572543
ATP5G1	0.246200653	7.326717524	14.29752341	0.00015607	0.002924981
FGFR2	-0.049822031	7.327216351	0.900078306	0.342760715	0.579842456
VAT1	-0.126797571	7.327287758	3.024499108	0.082015643	0.245957245
PIP4K2B	-0.038474896	7.327312653	0.52842782	0.467268587	0.686938676
SEPT2L	0.058100519	7.327547292	1.316550161	0.251212129	0.485797673
UBE4B	0.299125798	7.329020609	34.47324119	4.32E-09	4.13E-07
RARS	0.18709819	7.331142895	8.362142741	0.003831185	0.031458162
ID3	0.075142268	7.333606175	0.921607411	0.337053272	0.574320348
ANAPC1	0.112823607	7.333683347	2.728967743	0.098542958	0.275535154
IGSF3	0.163751889	7.333998842	7.625341737	0.005755376	0.042072287
UQCRH	0.004065733	7.334582033	0.002906254	0.957007122	0.981347249

UBE2K	0.055497131	7.334593289	1.502896097	0.220226287	0.449717493
CTDSPL	0.060203975	7.335487057	1.291976454	0.255684035	0.490546484
KAT6A	-0.073126545	7.336457684	1.033248504	0.309396907	0.547374057
TARS	0.27007425	7.336797425	8.985940566	0.002720647	0.024611462
ASPH	0.062785733	7.338713886	0.636816108	0.424866088	0.653441581
TRIB2	-0.113146572	7.338780441	4.136965343	0.041956321	0.161005425
PREP	-0.034450142	7.340548962	0.435956089	0.509080765	0.717892027
FGFR3	0.058914228	7.340578389	0.45051271	0.502091577	0.713033304
SMU1	0.119370175	7.341310161	2.848657313	0.091450283	0.264002528
WDR5	0.142714899	7.342812997	6.783675845	0.009199525	0.058469749
ARHGAP17	0.031226031	7.344374425	0.481289267	0.487838911	0.702056988
TNPO3	0.015623234	7.344633694	0.096438672	0.756145963	0.877026984
EXTL3	-0.00512014	7.345841148	0.00691562	0.933724154	0.971202968
SMS	0.0246683	7.346019824	0.17538553	0.675369207	0.828968937
KIF2A	-0.021170226	7.346326884	0.102570323	0.748766681	0.873168892
INTS3	0.033513454	7.346417052	0.472412007	0.49187962	0.704568538
ZNF521	0.148301614	7.347417647	3.12111041	0.077284102	0.237584212
FOXP1	-0.045686313	7.350903845	0.370850637	0.542540963	0.742906844
VEGFRKDRL	-0.00309815	7.351095672	0.001155017	0.972888677	0.989240757
GMSRAL	0.205673605	7.351475951	5.738913504	0.016593054	0.088321386
COPE	0.10343951	7.352009904	4.629904009	0.031419321	0.133420628
DNMT1	0.113600931	7.352482029	2.833316039	0.09232754	0.265645587
BTF3	0.155824072	7.353050781	3.382058369	0.065909693	0.215349212
STRA6	0.228492529	7.353107866	7.851966511	0.005076546	0.038573365
PRDX6	0.12156906	7.353773618	5.208801006	0.022472827	0.107109491
CCDC88C	0.110857506	7.35398304	1.988126355	0.158536923	0.373562577
OSBP	0.08384975	7.354158328	2.732297455	0.098337731	0.275195129
BPTF	-0.102008068	7.354187397	1.125682428	0.288698162	0.525172427
ZBTB44	-0.075517647	7.354262968	0.98051773	0.32207102	0.559752719
FAM122A	-0.039841977	7.355204174	0.310468116	0.577393073	0.76705002
UBE2G1	0.134655546	7.356387275	7.211689649	0.007243028	0.049412944
AKIRIN2	0.144862292	7.357053959	8.305052378	0.003953494	0.032205321

NFYC	0.022458723	7.35723357	0.159631257	0.689496237	0.83825922
ZNF503	0.242295527	7.357636648	3.783446038	0.051761953	0.183525736
POLE3	0.297494245	7.360743344	32.64131608	1.11E-08	9.55E-07
DDR1	0.172466757	7.361266387	8.156901349	0.00428976	0.034116967
IK	-0.052697902	7.362642257	1.227754079	0.267844261	0.503053957
UBE2V1	0.081761762	7.363105073	2.235096153	0.134908232	0.337938035
DVL3	-0.046328726	7.364102605	0.726227226	0.394108832	0.627342312
JAK1	-0.008068308	7.364193874	0.023068799	0.879278299	0.94602618
PHC2	0.034312559	7.364472348	0.401416473	0.526358637	0.730603439
FXR1	0.079353753	7.364620006	2.508930324	0.113202747	0.301904571
PPM1G	0.076482815	7.366586419	1.659702137	0.197643546	0.42338338
CREBBP	0.105572222	7.368168403	1.784299753	0.181622227	0.403700357
ARHGAP19	0.092735508	7.368270618	3.666154266	0.055528169	0.192171483
BICD2	0.004229778	7.369569918	0.005921111	0.938664313	0.973510732
CDC37	0.100328047	7.371538116	3.42902157	0.064060043	0.211472861
SUMO1	0.139029748	7.371753615	6.581154875	0.0103064	0.063351444
PAIP2	0.118947407	7.371775092	4.288435344	0.038372463	0.151618459
PAK3	0.091575415	7.37190786	2.528168232	0.111829969	0.29962006
DOCK1	-0.018982882	7.372918674	0.081525573	0.775240549	0.888893846
DHX30	0.036303823	7.373355965	0.313854811	0.575324297	0.765607847
KDM5A	-0.040129506	7.373641106	0.426840394	0.513543405	0.720825354
ARL8B	-0.027013969	7.373895319	0.29158249	0.589208213	0.775013681
HSD17B12	-0.022274071	7.374645133	0.190875079	0.662189293	0.820794617
CNDP2	0.007019892	7.374911035	0.016759628	0.896994479	0.952819305
RAB6A	0.020641004	7.376367327	0.100918739	0.750729884	0.873886548
KLC4	-0.050573216	7.377069681	0.811075129	0.367802579	0.602627845
SART3	-0.004583782	7.377880942	0.006772154	0.934413643	0.971393237
SPPL2A	0.124259943	7.378423321	5.62459697	0.017710138	0.091757026
PSMD4	0.099608867	7.378590295	3.081411801	0.079191757	0.240824314
B4GALT2	-0.104713875	7.380101251	4.861741251	0.02745861	0.122414366
CAPZA2	0.14460598	7.380148982	5.042917272	0.024726816	0.114127827
JAM3	-0.033678277	7.380864364	0.274580816	0.600275343	0.782502636

LYPLA2	-0.045889248	7.380939944	0.602404641	0.437662015	0.663631446
IGSF9	0.205533886	7.381024842	12.24809553	0.000465733	0.00687322
MMP2	-0.100090901	7.381587868	2.657660032	0.103052236	0.284088548
LARP4	-0.155191476	7.38160375	6.20441113	0.012743234	0.073652936
FBXO11	-0.003813344	7.383322523	0.00600296	0.938242681	0.973257994
PTPRG	0.123032257	7.38345781	3.398872037	0.065241018	0.213852779
HOXB9	0.315569326	7.384002214	5.888856714	0.015236989	0.0828603
CAB39	-0.028443392	7.384997536	0.279170985	0.597245098	0.780221347
ZC3H15	0.057434295	7.386005308	1.201410419	0.273039963	0.507692808
DNAJA1	0.681954638	7.386494757	21.42788535	3.67E-06	0.000141578
RTCB	-0.023118341	7.3865264	0.219285712	0.63958471	0.807117561
IVNS1ABP	0.036222708	7.386571153	0.475011068	0.490690831	0.703969418
SLC7A5	-0.088696362	7.386968055	0.681444992	0.40908959	0.639579022
APC	-0.070565645	7.387645478	0.702152455	0.402061382	0.634366465
RALB	0.098596734	7.387915002	3.882563497	0.048790008	0.176579455
USO1	0.064761743	7.388193801	1.4972265	0.221098604	0.4505483
DLD	-0.058569068	7.388510465	1.301396541	0.253958287	0.488529765
TOMM20	-0.089919829	7.388514021	2.585424053	0.107851521	0.292564585
ZMYM4	-0.033316466	7.388672157	0.380627657	0.537267517	0.738389458
UTRN	0.029487952	7.389297629	0.09298729	0.760413241	0.879947485
VPS26B	0.032487704	7.390564981	0.410131196	0.521902923	0.726742202
UQCRFS1	0.064281425	7.390569297	0.893538894	0.344520099	0.581324676
SRPRA	0.031395905	7.391391943	0.376827029	0.539306268	0.740387505
BAP1	-0.061596875	7.391697797	1.785731113	0.181447151	0.403583788
LOC101750015	0.031470125	7.392129471	0.200306044	0.654473929	0.815640639
RPS6KA3	0.111744605	7.392337924	4.102936165	0.042808809	0.163033711
WTAP	0.026159259	7.392947112	0.261794846	0.608889745	0.787473436
CUX1	0.132628613	7.39338268	3.182786095	0.074417741	0.231877281
STK25	0.07509817	7.395708395	2.5359884	0.111277186	0.298900767
BIRC6	-0.173083597	7.396087685	5.150742154	0.02323644	0.109557483
BCAR1	0.021370842	7.396557153	0.059077538	0.807959914	0.907527084
CHAMP1	-0.110928155	7.396701132	3.595340542	0.057941756	0.198004626

RAB14	0.032076791	7.397302143	0.422031448	0.515925128	0.722359455
SARS	0.023433915	7.398344726	0.156633867	0.692274734	0.839288299
FADS2	0.055523064	7.39845314	0.441172553	0.506557056	0.716037829
ZFHX4	0.025164005	7.399075762	0.07551722	0.783466851	0.893412823
C5H11orf58	-0.06187795	7.399151788	1.162899714	0.280864976	0.516583487
CD164	0.094150329	7.400183011	3.026987072	0.081889951	0.245804295
RPA1	0.104072139	7.400558409	2.812244076	0.093547433	0.267801499
PRELID1	0.036760577	7.400742687	0.273174303	0.601210328	0.78279126
NDFIP2	0.005666369	7.401947314	0.01212269	0.912327621	0.959684907
VDAC3	0.018558746	7.402125426	0.139163969	0.709113559	0.849542309
ZFP91	0.110524491	7.403000321	5.909471625	0.015059688	0.08224599
CCNI	0.072039561	7.403428161	1.603129599	0.205460264	0.432388879
TFG	0.123769609	7.404799569	6.271187514	0.012271687	0.071759279
PPP2R5D	-0.037111203	7.405072099	0.538678102	0.462981081	0.684057976
KMT2E	-0.048285487	7.405109094	0.293689553	0.587865822	0.774114284
EDF1	0.115140113	7.406045413	5.825058337	0.015799444	0.085248892
KNL1	-0.029648121	7.40735416	0.228232302	0.632837659	0.80349874
MTDH	0.019967017	7.407597246	0.201829395	0.653248246	0.815012502
CENPE	0.233124731	7.40759755	17.81938372	2.43E-05	0.000659547
PPA1	0.101084282	7.408012253	3.285099035	0.069911411	0.223160477
HM13	-0.043705399	7.408671418	0.849353121	0.356735401	0.593297051
SF3B4	0.090650458	7.412739889	3.270362601	0.07054203	0.224259874
HSPA4	0.183817735	7.413200922	8.07521387	0.004487481	0.035330862
ZNF706	0.210299098	7.413457332	11.10054265	0.000863025	0.010774712
MBTD1	-0.141779153	7.415514371	3.509009426	0.061035939	0.20475971
HNRNPUL1	-0.01567649	7.415860945	0.074239331	0.785261459	0.894405573
LSM14A	-0.049009607	7.417600487	0.506658571	0.476588924	0.693600685
UTP20	0.115361648	7.417645629	3.129323024	0.076895666	0.236862258
DDI2	-0.049912577	7.418500027	0.745351089	0.387952107	0.621932256
ATP6V0C	0.010497366	7.419608567	0.051183184	0.821017088	0.915983198
MAP1B	-0.080350662	7.420267493	0.361346953	0.547759122	0.747130003
MDM4	-0.212921937	7.422609479	10.78879584	0.001021163	0.012229429

GSPT1	0.098973177	7.423813557	4.155788644	0.041492447	0.159821681
GABARAPL1	-0.021964244	7.423822442	0.169731908	0.680350179	0.832415957
AKAP1	0.084396239	7.424311743	2.956135713	0.08555218	0.252739878
GNG7	0.06633568	7.424619709	1.261962575	0.261279929	0.49617556
LOC107052389	0.144623442	7.425001509	2.252628164	0.133387689	0.33530315
YTHDC1	-0.127152453	7.425162188	6.130560389	0.013286475	0.075939197
ACAD9	0.134108883	7.426045392	4.925707429	0.026459967	0.119566735
CCNA2	0.050567051	7.426428222	0.769155573	0.380478409	0.615067514
HOXB4	0.33578675	7.428512664	12.70385932	0.000364902	0.005707659
LIMS2	0.005215775	7.428830966	0.013696007	0.906836328	0.957377047
YLPM1	0.184599306	7.428832931	6.275316724	0.012243124	0.071658127
AFDN	0.043913921	7.428886213	0.387868429	0.533421979	0.735495698
TLN2	0.065652206	7.429204695	0.848925104	0.356856598	0.593318715
GTF2IRD1	-0.099846923	7.431435495	2.726770195	0.098678661	0.27564743
TRAM1	0.006758335	7.432151564	0.015920984	0.899590722	0.954278322
CLASP2	0.109518861	7.432344499	4.074737784	0.043529022	0.164546302
ACTR1A	0.159482344	7.432693068	9.994636979	0.001569968	0.016731748
DAZAP1	0.074939309	7.433124478	1.960503529	0.161459477	0.37728194
PHF20	-0.034210046	7.434352912	0.558159586	0.455002725	0.67768554
LOC107049885	0.250818193	7.434783335	13.83339762	0.000199754	0.003572771
MTHFD1	0.152106476	7.434939245	4.637191264	0.031286167	0.133130044
MPP6	0.01177438	7.435089532	0.022595324	0.880514197	0.946351332
NRARP	-0.045032429	7.435836181	0.138256019	0.710020958	0.849641566
PHRF1	-0.097685403	7.435872054	3.26662671	0.070702867	0.224487636
CNOT6	-0.084401518	7.436006903	2.188632278	0.139032725	0.344390109
CTH	0.128946463	7.436390645	4.738293858	0.029498312	0.127899143
CDON	-0.051110949	7.437780686	0.737966905	0.39031293	0.623951561
TALDO1	0.125379264	7.437990247	3.287678745	0.069801639	0.222896558
SMAD5	0.147829086	7.438348081	7.470201089	0.006272854	0.044936796
INTS1	-0.014968051	7.440934791	0.073413721	0.786429746	0.89486906
CIAPIN1	0.075682953	7.443737865	1.059310646	0.303372463	0.54137229
CPD	0.059838928	7.444295206	1.273778276	0.259059073	0.493552975

NHSL1	-0.117219046	7.445329293	2.645379895	0.103851329	0.285478385
RUVBL1	0.401612664	7.446050925	24.43298438	7.69E-07	3.84E-05
FZD1	-0.164294516	7.4481171	7.768103322	0.005317686	0.039858242
PSMA3	0.117995927	7.44885524	4.863070733	0.02743746	0.122386393
RBBP6	-0.058386967	7.45039215	0.595134155	0.440440612	0.6651417
TM9SF2	-0.037150437	7.450923658	0.570620097	0.450012611	0.673300375
HEATR1	0.073113582	7.451401343	1.271936037	0.259403795	0.493923796
STAG2	0.101252154	7.451941463	3.463457943	0.062738981	0.208643883
NR6A1	0.117573397	7.452869915	1.117455474	0.290467017	0.527437073
IGF2BP2	0.168356321	7.452888108	7.050189382	0.007925687	0.052881874
FYTTD1	-0.081403318	7.453494957	2.136512214	0.143828565	0.352344653
KIFC1L	0.665161277	7.455760892	43.02051769	5.42E-11	8.65E-09
UBE2N	0.159551281	7.457310295	9.132284006	0.00251138	0.023320229
SLC39A6	0.18947049	7.459052001	1.483334737	0.2232535	0.452697023
C16orf72	-0.053149893	7.459501541	0.772726926	0.379374776	0.614007272
DST	-0.203159625	7.460068627	6.350869515	0.011732378	0.069426128
ARID1B	-0.043347299	7.46099974	0.430180841	0.511900226	0.719326918
BCL9	-0.029834496	7.461009351	0.31051092	0.577366834	0.76705002
CDCA7L	0.064444278	7.461245172	1.143255221	0.284965532	0.520848201
PGRMC1	-0.051792191	7.462448347	0.797071177	0.371970485	0.606437563
LMF2	0.030266134	7.463470028	0.418599732	0.517636579	0.723314877
MYEF2	0.212326483	7.464481827	17.50185785	2.87E-05	0.000753274
TNKS1BP1	0.067021464	7.467985897	1.066479818	0.301741927	0.539448158
EIF3J	0.023637755	7.468503923	0.193596617	0.659939892	0.819543887
LENG8	-0.006873717	7.468637858	0.013743735	0.906674881	0.957349536
PSIP1	0.212636299	7.468815085	8.795223164	0.003020203	0.026547778
CCT3	0.213305346	7.469629555	8.26706313	0.004037076	0.032673977
NDUFS1	0.095001699	7.471369014	3.59170084	0.058068782	0.198171773
MCRS1	0.028253293	7.472500423	0.275119149	0.599918293	0.782347084
KCTD5	-0.090934637	7.475389615	2.747094737	0.097431319	0.273636511
AN1ZNF5L	-0.030537041	7.475782206	0.424271786	0.514813166	0.721736766
PLXNA2	0.207338613	7.47671178	5.266011751	0.021745779	0.10493777

NCOA4	-0.035109319	7.477185304	0.426244172	0.513837654	0.720949939
TDG	-0.010404276	7.479003899	0.02434879	0.876000713	0.9437957
DDX27	0.137110698	7.479664068	4.813279158	0.028241256	0.124620508
RERE	0.035236895	7.480371869	0.286901365	0.592213085	0.776980894
ESPL1	0.159884289	7.480659571	7.291874065	0.006926718	0.047991221
RBL1	-0.113821026	7.484153546	2.452880087	0.11730945	0.308947308
XPO7	-0.005472957	7.484757007	0.012922358	0.909494202	0.958432772
KIF1A	-0.149108218	7.486017907	1.773877137	0.18290298	0.405436365
SPG7	-0.092292661	7.486864962	2.285131107	0.130619104	0.330842759
LLGL1	0.098315918	7.487060683	3.40921613	0.064833237	0.212982814
ACTN1	0.192651207	7.487973404	14.05934811	0.000177131	0.003239391
NELL2	-0.085317717	7.489631859	1.156384261	0.282216677	0.517783055
AQR	0.021539573	7.489640434	0.134769154	0.713537576	0.851309514
EPHA4	-0.083392326	7.490850599	0.481107397	0.487921139	0.702056988
CYFIP1	0.144855596	7.491804025	10.56257246	0.001154005	0.013297724
HBM	0.237500657	7.494206127	0.816199133	0.366293799	0.601466431
TNPO1	0.014785987	7.494405447	0.047047552	0.82828279	0.918593819
CPAMD8	0.061166924	7.495438645	0.893356784	0.344569269	0.581348029
SF3A3	0.083710694	7.497726901	2.527346969	0.111888196	0.299661502
PPP4R2	0.21084077	7.49860363	17.85313526	2.39E-05	0.000652257
PSMB1	0.204636258	7.49921903	12.27477511	0.000459122	0.006793932
SLIT1	-0.058091306	7.499230843	0.300409979	0.583625515	0.770936649
RRP1B	-0.106108683	7.499715361	2.420663895	0.119744173	0.312585525
CAPZB	0.051255537	7.499740382	0.941684899	0.331844905	0.56937808
FUBP3	-0.101916568	7.499805283	4.522255972	0.033456688	0.139304098
SNX6	0.055664488	7.500133137	1.056017261	0.304125316	0.541951709
FZD7	0.104286774	7.502279475	3.961733592	0.046545744	0.171306245
PSMA1	-0.064449009	7.503228906	1.329470366	0.248899462	0.483090428
BUB1	0.151880857	7.503343358	8.198798064	0.004191815	0.033597816
SPIN1Z	0.089761127	7.503637272	1.366078092	0.242486866	0.475297366
RRP12	0.113709496	7.503869179	3.603992565	0.057640983	0.197271708
KIF26A	0.365534544	7.5044052	11.73044552	0.000614859	0.008445326

SUGP2	-0.036004445	7.504855859	0.313008418	0.575839941	0.765833434
CSNK1D	-0.063031957	7.505323232	1.08626872	0.297299421	0.534359582
IST1	-0.018091777	7.505884382	0.100976716	0.75066067	0.873867797
EIF4EBP1	0.070043369	7.506434262	1.31097923	0.252217437	0.486934484
CHERP	0.02953437	7.50651058	0.285316551	0.593237546	0.777327412
PLOD1	0.15869678	7.506641333	3.401659778	0.065130852	0.213625062
NUP50	0.006720723	7.508285149	0.009401226	0.922758224	0.964920347
SCUBE1	-0.171748731	7.50933277	2.005059159	0.156775178	0.371184972
NOP2	-0.147324322	7.510263175	6.548622291	0.010496541	0.064126023
LARS	0.244248491	7.510891732	22.93560498	1.68E-06	7.27E-05
SAFB2	-0.102409059	7.512053037	3.514100209	0.060848689	0.204535173
KTN1	0.081714517	7.51211359	2.888201845	0.089230465	0.260150124
PDAP1	0.063811472	7.513392361	1.681725641	0.194695313	0.419443752
MAPK8IP3	-0.067040624	7.513625831	0.889785848	0.345535348	0.582212863
FKBP3	0.039645672	7.513957571	0.521826085	0.470063764	0.689065646
PHLDA1	-0.098576654	7.513969756	0.826738453	0.363217338	0.599097263
LOC424740	0.257257228	7.514165583	12.07008952	0.000512372	0.007388296
NUP188	0.248292365	7.514890502	15.46722161	8.39E-05	0.001812825
MAPRE2	0.065782381	7.515383584	1.128041322	0.288193514	0.52498885
DIP2B	-0.042084256	7.515580159	0.456277928	0.499368645	0.710379765
ACTC1	-0.030159151	7.516247321	0.009167313	0.923722237	0.965621588
PHIP	0.147230038	7.516366846	5.578880883	0.018178335	0.093156184
HERC2	-0.010560818	7.516525934	0.020208449	0.88695645	0.949086044
DARS	0.137643777	7.517655564	7.833548014	0.005128533	0.038853598
GLCCI1	-0.090407362	7.518139185	1.423248055	0.232869528	0.464382236
TRAP1	-0.019635506	7.519386882	0.120593933	0.728391212	0.860298406
COL4A2	0.000974473	7.520454709	0.000126515	0.991025656	0.995986157
EPN2	0.072567355	7.521664537	2.297202846	0.129607216	0.329223095
CMTM3	-0.130476418	7.522608478	3.371441057	0.066335707	0.216106526
OGDH	0.024117675	7.522767747	0.133967082	0.714353779	0.852097965
SEC13	0.125165815	7.524254406	7.240271718	0.007128619	0.048855238
EPB41L5	0.050872527	7.525697285	0.661226453	0.416127293	0.646383861

RPS21		-0.021393723	7.525944181	0.0762434	0.782454306	0.892887345
KIF23		0.119876502	7.526513322	4.988895663	0.025510485	0.116705787
KPNA4		0.035638582	7.528480775	0.484915627	0.486204136	0.700617701
CLMP		-0.138533172	7.528736441	3.67105445	0.055365147	0.191796526
MLLT1		-0.116478865	7.529008092	4.211081696	0.040160719	0.156992849
RABL6		-0.037479163	7.529076596	0.464422358	0.495564399	0.707281655
TRIO		-0.113691667	7.529135834	2.341675988	0.125953976	0.322880928
RBMS1		0.050377635	7.52924944	1.023775062	0.311625225	0.549426023
CBS		0.303669418	7.53063655	15.0203442	0.000106358	0.002155577
	06/set	-0.022987655	7.531528091	0.226251712	0.634317164	0.80403489
TUBB4B		0.187301874	7.532319103	12.90643262	0.000327455	0.005251724
LOC101749469		-0.227429762	7.532940767	9.243987596	0.002362699	0.022498964
VAPB		-0.056193798	7.533101514	1.362305282	0.243138355	0.476034404
PLS3		-0.053015208	7.533471501	1.166173644	0.280188851	0.51560138
NR2F2		0.051793544	7.534343834	0.970144356	0.32464418	0.562732537
NFE2L1		0.044780996	7.53458987	0.591732755	0.441749854	0.666452173
TNS3		0.061332655	7.534831546	0.432080048	0.510970061	0.718764262
SREBF2		-0.118742261	7.535050355	4.89366002	0.026955484	0.12099086
GABPA		0.038181882	7.536937702	0.400552959	0.526803824	0.730729536
TRPC4AP		-0.024980686	7.537109713	0.209661062	0.64703319	0.811526992
LASP1		0.123885565	7.537374469	4.42544686	0.035406841	0.144141407
H2AFY2		0.094838657	7.537958587	3.269679717	0.0705714	0.224309908
PTEN		0.016913906	7.538569123	0.117488936	0.731774061	0.862408819
IQGAP3		0.00448419	7.53862993	0.006422453	0.936125742	0.972120443
NRIP1		0.046663582	7.538939935	0.370238599	0.54287424	0.743026497
PSMB2		0.092929923	7.539170265	2.812374796	0.093539812	0.267801499
FARP1		0.031039724	7.539461334	0.343527358	0.557800206	0.75364171
CHD6		-0.121056597	7.539490711	3.037906946	0.081340733	0.24464664
ACTL6A		0.127788361	7.541439282	5.95450118	0.014679756	0.080841828
G3BP2		-0.048615641	7.541904715	0.989878644	0.319772037	0.557135546
MYBBP1A		-0.04090497	7.542638642	0.384036925	0.535450622	0.7371794
TXNDC5		0.017818797	7.544284622	0.098229473	0.753964771	0.875422686

PNRC2	0.093821591	7.545445828	2.443064821	0.118045385	0.310293421
UBE2J2	-0.074108833	7.546480962	1.464230706	0.226258169	0.456254766
MEX3B	-0.056055531	7.549373647	0.338859843	0.560488083	0.755598182
BRD7	-0.002262343	7.549534325	0.001081278	0.973768059	0.98970682
SRGAP3	-0.089275026	7.5498295	1.144323313	0.28474064	0.520658654
RNF20	-0.00415883	7.550378251	0.002754056	0.958146951	0.982056949
PPME1	-0.038428207	7.550437204	0.585574121	0.4441357	0.669129516
KCTD2	-0.046239486	7.551680013	0.36487919	0.545808866	0.745830003
BRCA1	-0.023098075	7.55224068	0.125508793	0.723134899	0.857066913
KIAA1549	-0.027091579	7.552463723	0.264478122	0.607060182	0.786735079
EIF3F	0.001725776	7.553344425	0.001272461	0.97154424	0.988729078
KDM3A	0.089407867	7.553837131	2.46624067	0.116315827	0.307120817
PSMD11	0.007107602	7.5542585	0.018940792	0.890536362	0.950676503
HDAC1	0.120377749	7.554511839	5.035572096	0.024831884	0.114470733
RBM26	0.048309454	7.55599239	0.798998147	0.371393076	0.60567622
TFDP2	-0.144485762	7.557479866	4.385006242	0.036256359	0.146512375
BOP1	0.05178097	7.557743098	0.614347081	0.433155736	0.659655497
ACTA2	-0.489121537	7.55844741	5.702185288	0.016943804	0.089505714
MAF1	0.124736464	7.558756546	4.198101654	0.040469258	0.157645807
DLST	0.121251033	7.558987425	5.719311603	0.016779308	0.088877503
CBX5	-0.046302666	7.559237887	0.754029766	0.385203374	0.619731944
GBF1	-0.060333953	7.559517638	0.85036071	0.356450315	0.592942775
PDCD11	0.064557495	7.559953922	0.753475858	0.38537798	0.619942777
RNPS1	0.02028491	7.559960594	0.169066572	0.68094271	0.832955122
mesd	0.071519568	7.561312182	2.400001112	0.121335164	0.31551361
C2H6ORF62	0.16084954	7.562204573	3.634589515	0.056590558	0.194626589
MDN1	0.082953366	7.5625311	1.095359658	0.295286725	0.532205993
MCM3AP	-0.14117333	7.562725379	6.439098941	0.011163526	0.067091241
PTK2	0.164930315	7.562959994	8.718200861	0.003150488	0.027356875
METAP2	0.043485464	7.563427417	0.534830113	0.464583228	0.685414775
ID2	0.072159621	7.563975401	0.706759983	0.400521522	0.633035951
CAPZA1	0.100556371	7.564926174	3.395426913	0.065377437	0.214257266

LOC107050599	0.056199435	7.565380949	0.527674229	0.467586307	0.687221568
MAPK1	0.014230946	7.566356782	0.069476559	0.792100439	0.897745396
RPRD1B	-0.006306369	7.567002251	0.009369497	0.92288827	0.964995011
WDR43	-0.056670228	7.567317857	0.509124051	0.475518294	0.692572543
RBM33	-0.106315101	7.568550384	2.097060567	0.147582621	0.357601536
EPHA3	0.058933556	7.570468338	0.434735083	0.509674615	0.718039023
DEK	0.006580941	7.570948832	0.011340024	0.915193946	0.961041249
ALDH7A1	0.033588984	7.571034555	0.175948775	0.674878167	0.828679291
SSB	0.061518433	7.572928082	0.933255687	0.334018363	0.571813584
CNOT9	-0.048957987	7.573051525	1.049135408	0.305706294	0.54382671
RNMT	-0.042109613	7.573629066	0.558257123	0.454963328	0.67768554
BNIP3L	0.100798209	7.574405077	3.77137571	0.052136735	0.184468767
LSM14B	-0.097511482	7.575567138	3.837007803	0.050132913	0.180023331
POM121CL	-0.065299107	7.577687714	1.170629692	0.279271894	0.514618765
HECTD1	-0.031297489	7.577917504	0.27475692	0.600158493	0.782412296
CASC4	-0.003859255	7.578731185	0.006507121	0.935706999	0.972003524
CD24	-0.071433066	7.579650446	0.852837855	0.35575076	0.592239624
SMO	0.008867782	7.580104024	0.026273549	0.871234055	0.940731028
FRMD1	0.008054292	7.585349168	0.007321243	0.93181281	0.970075727
DHX38	-0.044414169	7.586254861	0.397964626	0.528142285	0.731816057
NEFM	-0.432198241	7.587791596	6.841661739	0.008905596	0.057153502
GJA1	-0.194535977	7.588004617	3.377118837	0.066107525	0.215662037
TEAD1	0.026462654	7.58810534	0.13496096	0.7133428	0.851138842
TENM4	-0.110985974	7.591250027	2.545435993	0.110613373	0.297628965
MSH6	0.188301866	7.591654066	10.53939355	0.001168568	0.013409191
PLOD2	-0.197693129	7.592243311	11.08461977	0.000870467	0.01083699
SRSF7	0.169307316	7.596185461	6.857256226	0.008828201	0.056834133
EP300	-0.020472059	7.596873749	0.120956213	0.727999705	0.860021189
THOC5	0.174314197	7.597158772	10.11834037	0.001468011	0.015913126
APCDD1	-0.624693383	7.597365289	8.224024208	0.004133943	0.033263583
SPEN	-0.07680388	7.599402298	0.758932001	0.383662957	0.618392982
ACAT1	0.000840373	7.601561296	0.000260962	0.987111269	0.993936846

ETS1	0.154547373	7.601826608	5.046775554	0.02467181	0.1139418
DNAJC7	-0.062718427	7.602051258	1.577887638	0.209065225	0.43679089
DDX23	0.045432886	7.603163256	0.767445522	0.381008462	0.615561537
LMAN1	-0.007283224	7.603547677	0.007219851	0.932285474	0.970319891
ACVR2A	-0.075083607	7.604040329	1.692545504	0.193265755	0.417457076
NSD1	-0.120811674	7.609838778	3.313572465	0.068709971	0.220781601
BUB3	0.037472545	7.611407995	0.49458678	0.48188835	0.697173002
CCNB3	0.138152198	7.611580944	4.883806025	0.027109779	0.121418456
STT3A	0.214197263	7.614851121	19.15268458	1.21E-05	0.000376058
PLCG1	-0.061006988	7.616028253	1.046239893	0.306374665	0.544521108
SMARCB1	-0.006611368	7.617340137	0.017639724	0.894339988	0.951827123
IGF2BP1	-0.116155536	7.61837423	3.49716619	0.061473935	0.206019063
TTC28	-0.079687804	7.619597309	1.293844489	0.255340667	0.490348661
UBE2O	-0.087576822	7.622424479	1.545908155	0.213740526	0.442458216
DHX37	-0.011110629	7.623469513	0.050660068	0.821918662	0.916367747
PCM1	-0.021303521	7.62661687	0.172651621	0.6777659	0.830489082
POLA1	0.049982773	7.627230653	0.4917464	0.483149308	0.697948254
MED1	-0.10585578	7.627356256	2.819445278	0.093128583	0.266881024
AP2M1	0.001282113	7.628436944	0.000726873	0.978491179	0.990912186
HIF1A	0.018460676	7.631888752	0.053375833	0.817289861	0.913683311
MPRIP	0.01028765	7.632297284	0.034438916	0.852776295	0.931394748
ARPC5	-0.005405348	7.634209537	0.013201903	0.908524754	0.958196054
SUPT6H	0.114335181	7.634298877	4.147329252	0.041700247	0.160433183
SPON1	0.025468972	7.635140701	0.093861407	0.759324401	0.879311307
PUF60	0.001211525	7.636510221	0.000637739	0.979852762	0.99148181
RPL7L1	-0.079420948	7.636639066	1.717919113	0.189961121	0.413388669
GNAI2	0.02804572	7.636756075	0.255899985	0.612951017	0.790261455
HMGN1	0.172989962	7.637349343	6.695523292	0.00966553	0.06063526
SLIT3	-0.131992089	7.638131083	2.896709887	0.088760551	0.259147422
B4GALNT4	-0.058431277	7.639425455	0.799236863	0.371321633	0.60567622
CYCS	0.061861039	7.639937532	0.974754717	0.323497222	0.561158211
RNF10	0.036777479	7.639940725	0.46732899	0.494218531	0.706269033

HIP1	0.290316558	7.642228993	16.52612773	4.80E-05	0.001154359
SPAG9	0.006232573	7.642526899	0.004237802	0.94809565	0.978142017
PSMD7	-0.040831381	7.64306828	0.440905643	0.506685664	0.716085474
TSC22D1	0.006159428	7.64362245	0.016114276	0.898986291	0.953944632
NF2	0.163485175	7.646399972	9.389928699	0.002181807	0.021373879
TSPAN3	0.042295107	7.646448558	0.467523524	0.494128675	0.706269033
PSMA4	0.263647117	7.648279198	19.30543871	1.11E-05	0.000356595
CDC5L	0.111578294	7.649724558	4.597778198	0.032013418	0.135293225
ADD3	-0.012940717	7.649824825	0.039054175	0.843341375	0.92611142
COPB1	0.029983053	7.650214192	0.326674571	0.567623813	0.760792527
PRPF40A	0.069000987	7.651860196	1.787116632	0.181277868	0.40334986
LAMA1	0.101299356	7.654057963	1.680659856	0.194836796	0.419664638
PSMC1	-0.123402272	7.657766366	5.41624346	0.019950239	0.098983362
AP3M1	0.002957264	7.658074413	0.003492529	0.952874351	0.979802073
FNBP1L	0.030650695	7.659381314	0.34468722	0.557136084	0.753115441
IARS	0.201329305	7.660788743	10.50863992	0.001188177	0.013567973
MSI1	0.006910612	7.661364912	0.014065814	0.905592753	0.956882345
SUPT5H	0.025799471	7.661923305	0.223474823	0.636404937	0.805232083
AP2B1	0.099945541	7.662929515	2.836693391	0.092133632	0.265370018
IREB2	-0.180293008	7.663165233	11.41493407	0.000728561	0.009529784
RASAL3	0.18725901	7.663230617	9.444506803	0.002117824	0.020892566
NCBP1	0.09792265	7.667266296	1.904560458	0.167568746	0.385426854
U2SURP	-0.047797042	7.66727819	1.04346111	0.307017873	0.545040361
PCNP	0.070638229	7.669270314	1.890144565	0.169185617	0.387628701
USP1	-0.026113221	7.670543941	0.139222505	0.709055174	0.849542309
THOP1	0.064694723	7.670679484	1.224217957	0.268534462	0.503585025
LRRN1	0.08738056	7.670787135	1.061885612	0.302785514	0.540559362
DNM1L	-0.062148653	7.672656203	0.94973084	0.329787819	0.567404701
LOC100857358	0.07608983	7.675357844	1.702278949	0.19199021	0.416176907
WNK1	-0.001652505	7.676038488	0.000770207	0.977859468	0.990744261
LOC107051177	0.120960438	7.676712885	4.07645879	0.043484702	0.164546302
PPP1CB	0.093444117	7.677018501	3.088613563	0.078841958	0.239970995

PIK3C2B	0.168200358	7.677341706	7.095124065	0.007729394	0.051955548
SLC25A5	-0.009052198	7.678040686	0.032175262	0.857643541	0.933726046
UQCRC2	0.095956159	7.679086881	4.233157496	0.039641629	0.155606632
CNOT7	0.021354733	7.68186553	0.110361657	0.739732769	0.867714765
TMEM131	-0.034430186	7.682366115	0.449245299	0.502693568	0.713518732
ADGRL2	0.093878318	7.683489204	1.72315997	0.189286795	0.412769276
TRIM71	0.261234539	7.684213922	4.90992231	0.026702842	0.120316338
PLAGL2	-0.018209125	7.684346583	0.102195189	0.749211058	0.873205803
C13H5orf24	-0.005195047	7.68608804	0.013103379	0.908865234	0.95829261
RPS28	0.149899933	7.687041237	2.000144695	0.157284192	0.371801663
SOGA1	0.046882439	7.687313641	0.615661538	0.43266407	0.659478488
GATAD2A	-0.168511109	7.687427858	10.94024956	0.000940975	0.011486456
EHMT1	-0.03045745	7.689058579	0.207640901	0.648622758	0.812444812
CTBP1	0.045628517	7.689620156	0.852621929	0.355811664	0.592239624
RAD23B	0.130152225	7.692323593	2.809207839	0.093724646	0.268149716
ARL4C	0.148546711	7.693340623	4.45527163	0.034793682	0.142543309
ANKHD1	0.019535078	7.6946062	0.112149344	0.737710283	0.866307619
PAK2	0.001510852	7.694946633	0.000617441	0.980175923	0.991676559
CLASP1	0.060796436	7.697467731	0.805697824	0.369395255	0.60402889
CDC20	0.314848024	7.698153698	27.13538258	1.90E-07	1.17E-05
SEC63	-0.014353003	7.698444706	0.083963176	0.77199679	0.886785841
API5	0.029896539	7.699103005	0.397909965	0.528170617	0.731816057
PDGFRA	0.016394596	7.700021604	0.037902539	0.845638973	0.927727802
TAX1BP3	0.130961138	7.701034402	7.735359924	0.005414977	0.040336552
KIF15	0.379259973	7.702245204	29.75510865	4.90E-08	3.66E-06
UBE2D2	0.09854463	7.702425302	4.045179315	0.044297672	0.166154137
CHTOP	-0.032464285	7.703308788	0.38472946	0.535082915	0.736802457
EIF3G	0.097014682	7.704819812	3.238966266	0.071905994	0.226571418
MSH2	0.107805213	7.709107084	3.858137201	0.049505263	0.178407601
NUP214	-0.099239144	7.713436323	2.279605985	0.131085172	0.331694065
EML4	0.054227525	7.715117508	1.028880747	0.310421693	0.548336682
XRN2	-0.048568428	7.717583162	0.871491698	0.350542674	0.587126307

NAP1L4	0.070130946	7.718850417	1.571099962	0.210047345	0.438045609
DCBLD2	0.087135954	7.718971318	3.080520926	0.079235144	0.240833472
DLGAP5	0.018532189	7.719173975	0.109322548	0.740916727	0.868298892
NUP98	-0.091356995	7.722682125	3.275513888	0.0703209	0.223902944
IMMT	-0.086366542	7.723285446	2.819459038	0.093127785	0.266881024
DOT1L	-0.016226652	7.72341436	0.058780838	0.808433356	0.907749244
EPHB2	0.106087355	7.723723371	2.86572437	0.090484965	0.262320313
PSMC6	0.111558319	7.725100036	4.077138418	0.043467214	0.164539453
SERP1	-0.002432743	7.726335929	0.002188155	0.962690388	0.983917412
PRPF6	0.106930112	7.729323778	4.294290033	0.038240559	0.151298004
ARHGAP28	0.147811079	7.730515205	6.324759747	0.011906362	0.070021151
ZNF326	-0.014320002	7.731792525	0.073727933	0.78598429	0.89467152
TRAM2	-0.062582222	7.731901531	1.019477589	0.312642959	0.550630406
SYNE2	0.062641336	7.732690669	0.717851924	0.396849422	0.629534102
MKRN1	-0.071687227	7.7332425	2.116180199	0.145749657	0.354989728
RIT1	-0.193463295	7.733305359	8.788822139	0.003030819	0.026604583
EIF2S2	0.13438013	7.736271858	4.963468991	0.025888223	0.117644495
AZIN1	0.038091469	7.73654873	0.418438453	0.517717256	0.723314877
ALDH9A1	0.176365942	7.736557354	12.89523842	0.000329419	0.005272949
MMD	0.038156793	7.73655827	0.305559667	0.58041782	0.769541151
SYT11	-0.201012293	7.736699066	11.14440697	0.000842854	0.010619575
ARVCF	0.021727215	7.737238869	0.0979291	0.754329092	0.875722111
GPC1	-0.181249008	7.737248747	4.338637639	0.037256724	0.148842797
PSMC2	0.093564039	7.739188209	3.228911435	0.072348765	0.227375706
NEK6	-0.054415851	7.74175104	1.100640393	0.294125608	0.531091504
PAFAH1B2	-0.06491699	7.743943559	1.652389102	0.198634073	0.424263198
YAP1	0.05582437	7.746901385	0.638151827	0.424380841	0.65312259
GLG1	-0.124720386	7.747571996	1.508379668	0.219386501	0.448737316
NDUFA10	-0.009721464	7.750720525	0.037208554	0.847041052	0.928523117
NCBP2	0.173904325	7.753056883	12.93923261	0.000321767	0.005185773
EIF2S1	0.021130256	7.755303775	0.178286782	0.672849705	0.827175338
NF1	-0.047220797	7.7555756	0.595783512	0.440191345	0.665015477

PSMA7	0.142059241	7.755941194	5.70625619	0.016904554	0.089355743
SVIL	0.077175096	7.757146144	1.694177574	0.193051187	0.417328557
UBQLN4	0.017253906	7.757961759	0.126028392	0.722586012	0.856989199
UBA2	0.07215466	7.758235733	1.10703997	0.2927263	0.52996053
SAFB	0.084292172	7.759523701	2.862928253	0.090642353	0.262637775
PSMC5	-0.05799602	7.76035244	0.785265881	0.375535469	0.610073664
C5H11ORF9	0.026185046	7.762544521	0.059990227	0.806511387	0.906551274
BIN1	0.05561946	7.76318342	1.012304007	0.314351502	0.55198527
PIK3R2	-0.08177661	7.763660439	2.237062702	0.134736713	0.337765479
SMARCD1	0.068939306	7.765493003	2.362406004	0.124290362	0.31992524
HIPK1	-0.055959776	7.765530326	0.770028964	0.380208092	0.614751317
XPR1	0.026496113	7.766913343	0.199737408	0.654932886	0.815920147
SH3GL1	0.064208037	7.767439993	1.910456294	0.166912582	0.384939948
STAU1	0.038092641	7.767987411	0.540535065	0.462211063	0.683472595
SMC4	0.195613654	7.769062042	18.71706589	1.52E-05	0.000454444
EI24	0.068928587	7.777002075	1.517465329	0.218003468	0.447176149
NID2	0.176312721	7.779136232	8.710292413	0.003164184	0.027417934
TIA1	0.034090551	7.779908124	0.42184016	0.516020266	0.722402031
ARNT	0.109164665	7.782507817	3.926493801	0.047530945	0.17353488
WDR77	0.135969141	7.783567103	3.466802353	0.062612237	0.208306584
LUC7L2	0.182004692	7.784786788	11.19820507	0.000818765	0.010411729
GPM6B	0.114450215	7.787326631	1.536650847	0.215117009	0.444357317
FERMT2	0.130332209	7.7873323	5.180935196	0.02283603	0.108227784
SLK	-0.057846906	7.787900848	0.648607839	0.420610854	0.650239722
DCN	0.092176868	7.787942351	0.897716086	0.343394851	0.58056982
GNL3	0.212678572	7.790887113	11.2451911	0.000798296	0.010171186
FNBP4	-0.100626818	7.791555976	3.805804731	0.051075227	0.182071677
ERP29	0.124417852	7.791788267	4.789849924	0.028627921	0.125652824
NCAPD2	-0.01810344	7.793245501	0.081324594	0.775510324	0.888893846
EWSR1	0.009122188	7.793461211	0.039563668	0.842336136	0.925585383
BUB1B	0.226195887	7.795710398	9.999852536	0.001565528	0.016695241
FTSJ3	0.129837598	7.797732526	3.243064567	0.071726357	0.226319695

TTL	-0.129703607	7.798858454	5.086325105	0.024115241	0.112429145
RANBP1	0.082739343	7.800012886	1.56272784	0.211266259	0.438848883
AP3D1	-0.006366209	7.806129875	0.014425151	0.90440017	0.956359155
ATRN	-0.135442204	7.806699656	3.449046708	0.063288262	0.209722827
PSMB4	-0.000390044	7.806730983	4.63E-05	0.994568393	0.997544036
UBE2D3	0.095116503	7.806736554	3.474047912	0.062338586	0.207858446
SUMO3	-0.056223939	7.806944619	1.028090878	0.310607488	0.548336682
IDH2	-0.085333097	7.807515921	1.471258532	0.225147231	0.454911306
DDX1	0.0177254	7.812607483	0.087507254	0.767370412	0.884179034
FAM168B	-0.131809461	7.813562645	6.182532372	0.01290175	0.074256743
YWHAH	0.335146263	7.814142165	24.55200584	7.23E-07	3.67E-05
SRSF5	0.074737348	7.816876324	1.968204291	0.160638595	0.376057489
ARHGAP32	0.029724468	7.8190219	0.207826248	0.648476529	0.812444812
SFRP1	-0.050435117	7.820344334	1.044674514	0.30673679	0.544776458
DGCR2	-0.046780712	7.821131305	0.991033612	0.319489882	0.557135546
DAG1	-0.02021232	7.821649366	0.107557022	0.742942759	0.869560491
BMS1	0.034416467	7.825331972	0.271346328	0.602430071	0.78367454
MTA1	0.079276204	7.825959051	2.42693077	0.119266212	0.311660395
POSTN	-0.437231883	7.826274199	21.72682147	3.14E-06	0.000124348
LFNG	-0.009905463	7.827134035	0.012310192	0.911654961	0.959545932
COPG1	-0.034418548	7.82721135	0.545022341	0.460358726	0.68238923
WSB1	-0.02323584	7.827923447	0.17100827	0.679217277	0.831524461
SREK1	0.105809557	7.830390408	2.409885563	0.12057118	0.313923855
NOL9	0.044698448	7.830509375	0.703931437	0.401465819	0.634045499
COL4A1	0.030009575	7.831800487	0.136513015	0.711772454	0.850204099
NRDC	0.042684668	7.832937532	0.65623594	0.417891951	0.647686055
SS18	-0.0260756	7.83368625	0.231634075	0.630314825	0.801583732
TPM2	0.062531231	7.835019985	0.302861293	0.582094183	0.770499657
KIF11	0.08135731	7.835089595	1.412794235	0.234593088	0.46656279
ZMIZ1	-0.034082549	7.836103509	0.263193339	0.607934727	0.787414637
NPEPPS	0.089429317	7.837617743	3.6309931	0.056712966	0.194947118
PARG	0.000255963	7.838159638	2.11E-05	0.99633121	0.998524363

COX4I1	-0.004944352	7.840033725	0.007586438	0.930591898	0.969599803
UGDH	0.064902407	7.84003783	1.074339672	0.29996727	0.537276742
TRMT2A	0.080786097	7.841686675	2.182130679	0.139621051	0.34532758
TLN1	0.067664889	7.842013065	0.761276204	0.382929433	0.617573637
TCOF1	0.011191034	7.842432767	0.045087696	0.831842857	0.920323688
FEN1	0.075915216	7.842785348	1.739621431	0.187186801	0.410108655
LOC107051050	-0.030333791	7.845586874	0.296767411	0.585916097	0.772448047
PRPF39	0.137659646	7.846925693	5.208017819	0.022482953	0.107109491
EMC1	-0.020201095	7.847836704	0.157916236	0.691082278	0.838989066
NFAT5	0.0879878	7.848013376	0.933291334	0.334009132	0.571813584
EIF4G3	0.282193523	7.848660273	15.63461953	7.68E-05	0.001697073
RBM25	-0.058971455	7.84920273	0.965630308	0.325772391	0.563899329
CDK6	-0.166773791	7.850915644	4.943273678	0.026192383	0.118568272
H2AFV	0.153286572	7.851769176	4.761445098	0.029104103	0.126795873
TAOK1	-0.020339095	7.853085226	0.123635125	0.725124863	0.858350451
CXADR	-0.008686919	7.853393077	0.015338328	0.901435622	0.954942584
SH3BGRL	-0.023871306	7.854841171	0.284593075	0.593706434	0.77771018
PCDHGC3	-0.053392735	7.859082539	0.732280828	0.392144881	0.625811659
PPFIBP1	-0.084221917	7.860777128	2.114413396	0.145917956	0.355031785
GARS	0.211602929	7.862671794	15.21601065	9.59E-05	0.002004855
MARS	0.07902645	7.863651762	2.226325469	0.135676177	0.338778678
UBAP2L	-0.043764939	7.864598814	0.746551023	0.387570399	0.62171007
NECTIN1	0.131457371	7.866547319	3.365068948	0.066592797	0.216472633
LUC7L3	0.025134129	7.868096288	0.163843646	0.685642139	0.83583947
GRB2	-0.010324638	7.869502332	0.032846439	0.856182444	0.93289505
CNMD	-0.178448799	7.870464525	2.756716559	0.096846821	0.272657543
MORF4L1	0.048075165	7.87119661	0.93049564	0.334734166	0.572087272
MYO18A	0.173287282	7.871339904	6.325419818	0.011901931	0.070021151
USP24	-0.008588108	7.871573048	0.01699831	0.896267715	0.952718685
LOC112530429	0.167933589	7.871955585	7.311369823	0.006851968	0.047592415
TNRC6A	-0.140689541	7.875082289	4.578208957	0.032381044	0.136273676
SETD7	0.136988655	7.875448131	5.738202545	0.016599772	0.088321386

GCN1	0.091425985	7.875670845	2.103153445	0.1469957	0.356570325
ABCF2	-0.027548378	7.875881878	0.149524117	0.698990545	0.843494384
TMBIM6	0.006462968	7.876135808	0.017461948	0.894870659	0.951827123
RBM12	0.121918352	7.877989921	4.165479366	0.041255737	0.159432399
PUM2	-0.027685413	7.878252432	0.304245085	0.581233291	0.770125921
LOC107052650	0.098800298	7.881548845	3.243190568	0.071720842	0.226319695
NEK9	0.117778779	7.883705766	3.618835955	0.057128832	0.196048995
LOC423693	0.051070403	7.884044043	0.599950614	0.43859687	0.664069883
UQCRC1	-0.016456586	7.885654974	0.094782639	0.758182858	0.878460705
FABP7	0.06519832	7.88811733	0.356529073	0.550440248	0.749027805
EIF4E	0.079648014	7.888459062	2.229872991	0.135364975	0.33839038
FBLN2	-0.053043379	7.888592707	0.434115095	0.509976611	0.718279992
TMED2	0.013894555	7.888819356	0.076603394	0.781954275	0.892677785
NNT	0.099639518	7.889157062	1.53945963	0.214698256	0.44383734
ATIC	0.103127249	7.889814595	2.214066761	0.136757738	0.340725525
DNAJA2	-0.005847024	7.890691898	0.006763946	0.934453313	0.971393237
ERH	0.079824998	7.892717053	2.262929651	0.132503184	0.334100506
ANP32A	0.039032679	7.893346127	0.494941745	0.481731148	0.697173002
RAC1	0.014936804	7.893348042	0.108303942	0.742083398	0.869036926
PPIG	0.05177716	7.893381227	0.579071304	0.446676571	0.670621395
HOXB3	0.104245613	7.897448481	1.984617433	0.158904814	0.37396064
LOC772071	-0.021509322	7.898010176	0.188246635	0.664379952	0.821982865
VASH1	0.15188427	7.898074167	5.070693774	0.024333643	0.113014421
PES1	0.022301076	7.898584446	0.166945489	0.682840839	0.833770356
PGPEP1L	0.135959833	7.899666863	6.895334414	0.008642099	0.056119079
IGFBP2	-0.053155686	7.900862447	0.773691687	0.379077417	0.613681567
ERGIC1	-0.027954353	7.901337041	0.292395585	0.588689457	0.774764858
NAA50	0.033997681	7.902017338	0.448217499	0.503182654	0.71381668
DUT	0.013117893	7.902636886	0.030514746	0.861327387	0.935139468
MFAP2	-0.219421777	7.902810426	4.22590834	0.039811305	0.15616091
CUL4B	-0.010518929	7.90489769	0.037015903	0.847432673	0.928742508
PSMC3	0.155192145	7.905108064	9.452545323	0.002108563	0.020869886

ITM2A	0.035791313	7.905796551	0.430810872	0.511591334	0.719032808
WDR1	0.076144415	7.907172184	2.306980336	0.12879404	0.327964396
EIF3H	0.006829375	7.907438286	0.016867724	0.896664688	0.952793351
DHX36	-0.043364061	7.907629483	0.590755641	0.442127066	0.66689897
ANKRD17	0.04423554	7.907692441	0.41137453	0.521272672	0.726233326
CSNK2A1	-0.039913136	7.909344005	0.617710786	0.431899246	0.658961715
BORL4	-0.112902317	7.910654129	1.478139958	0.22406577	0.4535616
EPB41	-0.05489428	7.912861457	0.770156106	0.380168764	0.614751317
SRPK1	-0.101563783	7.91548925	3.475794593	0.062272808	0.207801968
NT5DC2	0.143641966	7.919453459	6.748417711	0.009383086	0.059263866
MDH2	0.095815174	7.919608017	2.961951648	0.085244991	0.25228531
HELLS	0.048971577	7.921150008	0.433309961	0.510369254	0.718341124
SCRIB	0.039220176	7.921320866	0.423284715	0.515302569	0.722011561
HSPE1	0.170353375	7.921721934	4.045267843	0.044295349	0.166154137
RPL38	-0.092657147	7.92214734	1.439202834	0.230268383	0.461574642
MTA2	0.003201925	7.922664303	0.00370121	0.951488568	0.979652938
SINHCAF	0.099993515	7.922874514	2.497793974	0.114005893	0.302966241
ACACA	-0.037454235	7.923729801	0.32195625	0.570434375	0.762633868
NUDC	0.096505564	7.928228814	2.678328271	0.101722462	0.281449149
NUP153	0.078308048	7.928496673	1.598771296	0.206077423	0.433078416
PPP2R2A	0.071068893	7.931548387	1.769326287	0.183465478	0.405878522
ZFHX3	-0.094308691	7.932227794	0.992871698	0.319041518	0.55677465
PSMD13	-0.016893596	7.933293045	0.106780745	0.743839407	0.869927323
KIF5B	-0.089956703	7.934735286	3.573310954	0.058715131	0.199620061
MCM4	0.137866552	7.935740345	4.637932155	0.031272662	0.133130044
CRABP1	-0.305449304	7.940688842	2.836034648	0.092171418	0.265432392
ATP5C1	0.121431938	7.942158319	5.627188925	0.017683968	0.091711634
USP7	0.01575975	7.944202175	0.086781308	0.768309643	0.884464473
RPS29	0.077455849	7.94423811	0.818904877	0.365500549	0.600890352
CLTCL1	0.109734185	7.949142767	5.66476576	0.017309028	0.090678147
PSMB7	0.046543175	7.949504261	0.554333668	0.456552346	0.6790102
CDK1	0.230747802	7.949593252	10.8526026	0.00098657	0.011919244

KIDINS220	-0.047948981	7.950250206	0.533583172	0.465104303	0.685717347
PNISR	-0.086363606	7.951442904	2.798013784	0.094381159	0.269024818
DDOST	0.07335288	7.952120516	2.374935466	0.123296693	0.318327342
QARS	0.014741703	7.953116453	0.06630111	0.796800519	0.900876222
TP53BP2	-0.115301759	7.954906273	5.661020791	0.017346025	0.090756386
BANF1	-0.095255206	7.957180295	2.115439325	0.145820203	0.355022129
GFPT1	0.05878886	7.962005052	1.088700376	0.296759343	0.533680327
CDV3	0.039708477	7.96726726	0.576990358	0.447494428	0.670925335
FAM120A	0.018810332	7.968539488	0.1027072	0.748604761	0.873168892
BAHCC1	0.001487241	7.976981304	0.000309813	0.985956757	0.993435858
SEC24C	-0.027793693	7.977535997	0.331733966	0.564639846	0.758587325
IPO9	-0.002009957	7.980171544	0.000964113	0.975229536	0.990018742
ARHGAP1	-0.015921518	7.982143476	0.0914509	0.762340677	0.880756463
SARNP	-0.010136487	7.986665841	0.040148818	0.841189892	0.925252652
ACO2	0.240294482	7.986857152	15.40479887	8.68E-05	0.001851859
COPB2	0.228094832	7.987434573	20.01227148	7.69E-06	0.000264334
NUP205	0.203159022	7.98813415	12.22983675	0.000470313	0.006915999
LUM	0.02030908	7.989336466	0.018160307	0.892801489	0.951217859
KIF1B	-0.151286807	7.989841681	6.233162025	0.012537966	0.072979209
MBD3	0.128439184	7.991637285	5.562560981	0.018348554	0.093707466
BLMH	-0.015285816	7.992125826	0.076135346	0.782604641	0.892887345
PRKAR2A	0.076189885	7.994465438	2.163402663	0.141331417	0.348249245
CCNB2	0.089633702	7.994690184	1.766112265	0.18386395	0.406459052
CHCHD2	-0.079559488	7.996943166	1.373479367	0.241214979	0.474046635
STIP1	0.21242723	7.997068464	9.248212036	0.002357255	0.022473132
PRKAR1A	-0.100723374	7.997749023	2.042143708	0.152993905	0.365439789
YME1L1	-0.056911175	7.998889051	1.260636302	0.261530682	0.496537137
EHD3	0.005501435	8.000971817	0.010221755	0.919468938	0.962786027
LRIG1	0.425214155	8.003621658	12.33197076	0.000445266	0.006642659
DOCK7	-0.040792409	8.006000007	0.54009365	0.462393918	0.683620118
CRYBA4	0.197631815	8.006218504	4.090435428	0.043126531	0.163739516
BRD4L	0.140657043	8.006230271	4.814112577	0.028227603	0.124620508

MCM6	-0.0568432	8.007199767	1.168639367	0.27968099	0.514954761
PNN	-0.193847821	8.008049201	10.18188444	0.001418272	0.015527385
DYL1	0.16542105	8.008695002	5.013358707	0.025152462	0.115513193
CTTN	0.000448037	8.009008236	8.93E-05	0.992460744	0.996459368
ARID3B	0.078680998	8.012435326	2.027835879	0.154440386	0.367348446
AGO1	0.014567151	8.013595474	0.047261786	0.827898376	0.918504892
CS	0.032917888	8.017340075	0.310855432	0.577155732	0.767008365
NR2F6	0.10846372	8.018907223	3.422102481	0.064329037	0.212003664
TGM2	0.088219626	8.019540632	2.094579222	0.147822404	0.357971694
LIN28B	0.151814468	8.020302567	2.310118023	0.12853429	0.327607148
PAICS	-0.00681759	8.020340512	0.011896863	0.913144797	0.960114864
PODXL	0.119616997	8.022334326	1.890241362	0.169174701	0.387628701
TRRAP	0.170087832	8.023185232	5.723380578	0.016740469	0.088773577
FAT1	-0.02493834	8.025662836	0.099855457	0.75200316	0.874401564
PSPC1	-0.006614476	8.027789623	0.016361925	0.898217236	0.953465464
CHD7	0.141698849	8.029392398	4.060278336	0.043903262	0.16542894
ZNF609	0.002431339	8.030302848	0.002248131	0.962182905	0.983899168
NUP155	0.125572483	8.031137414	2.091495805	0.148120981	0.358220275
EPFIP1L	0.025507886	8.032545076	0.223280298	0.636551783	0.80529404
JPT1	-0.055098587	8.035578631	0.638782954	0.424151851	0.65309226
LMNB2	-0.009037708	8.035881286	0.011395174	0.91498876	0.961017703
ALYREF	0.022381213	8.036688284	0.097310801	0.755080961	0.876347666
POGLUT1	-0.0433384	8.039352724	0.502934667	0.478213485	0.694589363
KIF20A	0.117041878	8.042493501	5.249627493	0.02195147	0.105462945
SEMA5B	-0.251792859	8.042946139	14.9919669	0.00010797	0.002174594
ATN1	-0.04802721	8.043820169	0.543025309	0.461181638	0.682804855
UBR7	0.062084764	8.048809528	1.634884973	0.201028717	0.427601802
TMED10	0.003273638	8.049310511	0.0039212	0.950069513	0.97884497
SBF1	-0.010998879	8.050071159	0.039786033	0.841899516	0.925494032
GSK3B	0.063417604	8.05011419	1.027350467	0.31078178	0.548467846
RPS27	-0.058332125	8.057059874	0.641384693	0.423209824	0.65260216
USP9X	-0.086062626	8.057455159	1.812089891	0.178257731	0.399115301

CAP1	0.052476214	8.060074969	1.268256797	0.260093958	0.494722701
PPP4R3B	-0.063755458	8.060622578	1.848369855	0.173973109	0.393551431
RBM39	0.053700208	8.061220103	1.048186811	0.30592505	0.544098217
DCTN1	0.15409651	8.062303598	7.581116315	0.00589829	0.042878815
FIGNL2	0.063749959	8.063099227	0.589603998	0.442572291	0.667325855
DDB1	-0.051213862	8.064841459	0.496769427	0.480923057	0.69668007
LOC107049011	-0.063615572	8.071388224	1.735525344	0.187706795	0.411006696
MRC2	0.040753438	8.073173042	0.274851414	0.600095813	0.782392568
NUDT4	-0.088246943	8.07337753	3.389385983	0.065617381	0.214698165
MAST2	-0.078581052	8.073486571	1.346419758	0.245904996	0.479484146
SNRPL	0.074015577	8.07441482	1.947403621	0.16286691	0.379345755
PDCD6IP	-0.088829833	8.078545896	1.686255024	0.194095376	0.418721657
PBRM1	-0.009854977	8.083270331	0.031072154	0.86007951	0.934473974
ARID1A	-0.036654702	8.0841034	0.517883082	0.471746099	0.690375494
PUM1	0.104225214	8.086834408	3.146322462	0.076098289	0.235242786
MAP1S	0.018485836	8.087715839	0.081450774	0.775340909	0.888893846
NUP160	0.202183332	8.089755165	12.12431071	0.000497687	0.007234491
THOC2	-0.026322586	8.092110302	0.264626488	0.606959363	0.786666377
TRIP12	-0.022308571	8.094761147	0.20164344	0.653397566	0.815062002
TXN	0.257693714	8.095011122	5.415344166	0.019960518	0.098983362
EIF5B	0.080818963	8.095444401	2.5025758	0.113660264	0.302558503
PEBP1	0.1405066	8.095473363	6.522078201	0.010654343	0.06483625
STT3B	0.065668112	8.097752258	1.721013411	0.189562649	0.413123016
SF3A1	0.012815565	8.100346829	0.044526317	0.832877439	0.921010621
RAB11B	-0.006126207	8.101114125	0.018921697	0.890591206	0.950676503
GHITM	-0.006720321	8.101861887	0.016064615	0.899141229	0.953986003
SEC61A1	-0.180063247	8.10822866	9.617202429	0.001927631	0.019598353
TRA2B	-0.084933992	8.110578306	2.189958526	0.138913056	0.344313238
SDCBP	-0.01262334	8.112528857	0.066003408	0.797247259	0.901033555
NOTCH2	0.009261874	8.114787238	0.018675487	0.891300905	0.951008202
APLP2	0.116796081	8.116966162	4.755044596	0.029212536	0.127110744
KHDRBS1	0.00868156	8.118381176	0.033789432	0.854155436	0.932178863

UBR5	0.041536916	8.119514035	0.337605322	0.561214747	0.75591721
ASPM	0.157736741	8.120047103	6.411512438	0.01133828	0.067868823
SNRPD3	0.061948042	8.121521268	1.326756451	0.249383069	0.483598593
YWHAG	-0.023287242	8.12266385	0.157018931	0.691916077	0.839182338
SSR1	-0.066870936	8.124533127	1.71675617	0.190111133	0.413465331
PANK3	0.070515303	8.125047586	1.57075979	0.210096708	0.438045609
PARVB	0.045141368	8.125678233	0.444196506	0.505103904	0.715397207
TUBB3	-0.243605845	8.127971318	2.702821779	0.100170814	0.278388481
DCX	-0.089351133	8.129495255	0.48034628	0.488265508	0.702056988
PPP1CC	0.090698649	8.13139099	3.304274055	0.069099871	0.221464242
PTPRF	0.074187523	8.131901894	1.146559238	0.284270582	0.520218204
TMED5	0.094129111	8.134685878	3.923440627	0.047617332	0.173773165
CCAR1	0.003115117	8.134736601	0.002998237	0.956332725	0.981136915
TFCP2	0.089819241	8.134793644	2.601051611	0.106792833	0.290891585
SERINC1	-0.089382378	8.14241493	2.372917114	0.123456164	0.318512258
SCAF11	-0.112099921	8.143788377	2.796815754	0.094451718	0.269126063
ARCN1	0.125135518	8.143816348	6.638798722	0.00997812	0.061913716
TM9SF3	-0.020502345	8.145128512	0.133569955	0.714758929	0.852264423
PPP1CA	0.057845731	8.145565329	1.397427763	0.237154722	0.469544092
MAPK1IP1L	0.022915475	8.149997757	0.173887034	0.676680132	0.830023971
CPSF6	0.009904116	8.151748251	0.026211892	0.871383916	0.940731028
PDIA4	0.110509244	8.157371256	2.777604066	0.095591073	0.270889262
RIF1	0.027379912	8.160592132	0.14989185	0.698638732	0.843317219
RPL36	-0.025666162	8.161275992	0.098957156	0.753084694	0.874832943
LOC107050620	-0.055324411	8.162019282	0.449759495	0.502449188	0.713233384
KMT2D	-0.045961082	8.164032771	0.414043215	0.51992443	0.725544277
RTF1	-0.063941937	8.164400311	1.323698477	0.249929367	0.484259537
SPARC	-0.053429934	8.166818197	0.631564165	0.426782148	0.654800765
CAND1	-0.030650725	8.167907233	0.262696721	0.608273495	0.78745769
FKBP1C	-0.012380879	8.170057567	0.041910222	0.837791001	0.923551345
ABL1	-0.140463489	8.171522016	5.728418337	0.01669251	0.088638166
ARGLU1	0.065933962	8.172459319	1.696300638	0.192772484	0.417158237

UHRF1	-0.050467983	8.173293129	0.473017129	0.491602413	0.704487482
ATP11C	-0.134659643	8.173838934	3.480390917	0.062100068	0.207399355
FGFR1	-0.010955884	8.176269592	0.037973708	0.845495943	0.927632734
ATP5F1	0.006184234	8.178515016	0.010446737	0.918590563	0.962372833
EIF1AY	-0.028478133	8.182245147	0.22018535	0.638898727	0.806855508
NOP58	0.069637978	8.184560494	1.012618999	0.314276224	0.551930556
EIF4B	0.028204975	8.186918048	0.28760756	0.591757757	0.776938793
CDH2	0.181359948	8.188440208	5.586508333	0.01809934	0.092927773
TJP1	-0.050829943	8.18995659	0.700394765	0.402651079	0.634699062
TKT	0.157386924	8.193196852	7.106821303	0.007679116	0.051702068
MGEA5	-0.166819244	8.194987718	4.137562503	0.041941522	0.160986176
CFL2	0.066358511	8.196713634	1.362225341	0.243152183	0.476034404
EIF3I	0.120301366	8.198016933	4.914372813	0.026634131	0.120167111
SERPINH1	0.569174925	8.199463183	10.06362476	0.001512254	0.016278211
TAF15	0.054712794	8.199506609	1.277564871	0.258352298	0.492776986
DHX15	0.064453642	8.200899959	1.563682902	0.211126786	0.438848349
AP2A2	0.011940967	8.201577155	0.058882535	0.808270936	0.907716078
SETD2	-0.090890906	8.202235853	2.97013759	0.08481464	0.251493697
TACC3	-0.034131519	8.20435533	0.27709711	0.598610217	0.781392287
U2AF2	0.010436902	8.205200648	0.048788578	0.825184806	0.917552618
NAA15	0.101069201	8.206690115	2.48702245	0.114788708	0.304404099
ACTN4	0.247113347	8.209826843	13.57041803	0.000229778	0.004009544
ELAVL1	0.06126535	8.210198916	1.767425031	0.183701073	0.406181289
CLIC4	0.067795883	8.21369537	1.842369704	0.174673457	0.394160332
SPAG5	-0.080942228	8.215236718	1.86826266	0.171674192	0.390367969
ADD1	0.124082086	8.217880669	6.294118145	0.012113927	0.071064053
MDH1	0.088928586	8.218291406	2.208885967	0.13721773	0.341618515
ADH5	0.110073747	8.219988271	4.752847891	0.029249848	0.127226739
ATP5G3	-0.036499933	8.221674178	0.342625856	0.558317435	0.753969093
FKBP9	-0.305718527	8.221934132	16.32968295	5.32E-05	0.001258152
CDC42BPB	-0.07026003	8.224356145	1.416182237	0.234032812	0.465885545
ATRX	0.030940525	8.227992624	0.294094956	0.587608258	0.773960928

NUCKS1	0.023774938	8.228017291	0.129061371	0.719407233	0.855189331
COL3A1	0.001133837	8.229452078	5.50E-05	0.994080186	0.997413991
TBL1X	0.052604116	8.229516463	0.718261595	0.39671473	0.629433501
RPL29	0.014890489	8.2310391	0.027258237	0.868864732	0.939552419
IMPDH2	0.053250065	8.237962971	0.780751786	0.376911316	0.611748295
ENAH	0.099723319	8.239176602	3.940727281	0.047130397	0.172801769
ARHGAP35	-0.136791927	8.239659728	4.862081346	0.027453198	0.122414366
SPECC1L	-0.029135083	8.239925844	0.16859189	0.681366283	0.83319824
SSBP3	-0.018599647	8.241069301	0.119686618	0.729374627	0.861089072
TRA2A	0.137371901	8.246855425	6.436188266	0.011181833	0.06717673
C4H20ORF194	-0.199446318	8.248651638	3.675427391	0.055220095	0.191495608
USP5	-0.029614147	8.249730708	0.314591002	0.574876533	0.765401193
MARCKSL1	0.121931203	8.250950833	3.205308874	0.073399648	0.229400041
SRSF5A	0.062887068	8.254460223	1.696553644	0.192739303	0.41714129
MARCKS	0.094917917	8.254838754	1.126449403	0.288533957	0.525143928
MCM3	0.112186233	8.255280532	3.582972058	0.058374625	0.19889328
USP10	0.014427023	8.256432593	0.049161645	0.824528548	0.917226694
HBA1	0.228115084	8.256897741	0.724958546	0.394522217	0.627445382
CTNND1	-0.040347235	8.262433693	0.62796989	0.428100951	0.655598059
MIF	0.094279026	8.2689271	2.204606916	0.137598965	0.342177869
NEO1	-0.009165476	8.271048531	0.020608892	0.88584953	0.948791653
EIF3M	0.033312033	8.275099702	0.303438509	0.581734771	0.770421418
HDGFRP2	0.059297405	8.276339653	1.291692968	0.255736192	0.490573449
MTPN	0.020856552	8.278627589	0.145041084	0.703320048	0.846389149
MSN	0.058309117	8.283892883	1.157299848	0.282026233	0.517708798
PALLD	-0.115955428	8.285492966	5.320287587	0.021078532	0.102830492
AARS	0.173896087	8.287582219	3.327453761	0.068132276	0.219439538
RPS13	0.129371376	8.289957606	5.809505695	0.015939774	0.085828988
NID1	-0.039245303	8.29378353	0.210262727	0.646561559	0.811526992
PSME3	0.023208092	8.298497871	0.179249074	0.672019371	0.826403425
TCERG1	-0.044782675	8.299457193	0.710820287	0.399171631	0.63157396
PAPOLA	0.109607908	8.29974443	4.570301053	0.032530848	0.136589718

INCENP	0.010598867	8.301600987	0.034816561	0.851980568	0.931269647
PLXNA1	-0.092512786	8.303010825	2.911888455	0.087928861	0.257175507
ATP2A2	0.040872	8.305422792	0.43082529	0.511584268	0.719032808
RPS26	0.060523813	8.311020413	0.912632241	0.339416971	0.576437843
BZW2	0.063663733	8.313721512	1.469608907	0.225407409	0.455269292
PSMD3	0.032318782	8.315159831	0.262072851	0.608699642	0.787473436
CSE1L	0.16462394	8.316027787	8.836467266	0.002952702	0.026150002
RPL22	0.00026973	8.31783917	1.95E-05	0.996480774	0.998533498
HADHA	0.068088399	8.317968891	1.44866954	0.228741556	0.459465614
ARF4	0.089398731	8.31863753	2.955685176	0.085576027	0.252764947
SMC3	0.044552202	8.319080208	0.663562859	0.415304931	0.645642722
MCM5	0.156423827	8.321231047	5.51122026	0.01889486	0.095548531
MAP4K4	0.066752328	8.321547884	1.046154238	0.306394466	0.544521108
RBMX	0.155227812	8.32258931	6.205661327	0.012734236	0.073626795
SKP1	0.002717828	8.324579272	0.002963824	0.956583805	0.981210978
SUMO2	0.037308916	8.337711348	0.387594401	0.533566605	0.735495698
MEIS2	0.130329025	8.340386593	2.793877489	0.094625011	0.269340002
GOT2	0.001945855	8.344100814	0.000592958	0.980572848	0.991750505
NCKAP1	0.065835086	8.34599	1.607452868	0.20485022	0.431712394
CKAP4	-0.030699801	8.351844317	0.399195907	0.527504817	0.731324607
CLSTN1	-0.013124048	8.35669437	0.041994104	0.837631011	0.923498713
RPN2	-0.094799312	8.360844264	3.312605052	0.068750427	0.220868464
ABCE1	0.103973538	8.362949458	2.255230284	0.133163645	0.335082178
EIF1	-0.076900195	8.36482466	1.153286844	0.282862154	0.518549102
SRSF11	0.100227872	8.367320846	3.524512921	0.06046759	0.203475294
HYOU1	0.10575029	8.368718303	3.161063633	0.075414034	0.233768701
LOC112533601	0.350823411	8.374994284	1.449229117	0.228651688	0.459397255
NUMA1	0.181207096	8.375833323	5.068863958	0.024359343	0.113101859
RHOGL	0.003917982	8.37840909	0.006849404	0.93404148	0.971309744
ANKRD11	-0.096650311	8.380607049	1.613759676	0.203964112	0.430812852
ZDHHC8	0.004859275	8.380795251	0.007298522	0.931918443	0.970083458
KARS	0.016414413	8.385316854	0.065989457	0.79726822	0.901033555

TUBA1C	0.318726206	8.385342394	2.440696049	0.118223756	0.310434308
OAZ1	0.091117236	8.386982172	1.509715722	0.21918247	0.448643804
RDX	-0.005545806	8.387739416	0.014015684	0.90576035	0.956948586
PTP4A1	-0.102989814	8.391058218	3.239028649	0.071903256	0.226571418
PAFAH1B1	-0.000703721	8.396145377	0.000227239	0.98797279	0.994500046
TOP1	0.159718506	8.396882317	8.884579393	0.002875893	0.025718923
PRTG	0.138124369	8.399996911	0.796799372	0.372052031	0.606489157
CTBPL	0.085167008	8.403295312	2.051325881	0.152073703	0.363981496
RAB1B	0.011605793	8.404313341	0.058728885	0.808516386	0.907749244
EIF3E	0.080648678	8.404736068	1.967380797	0.16072615	0.376111802
TCF3	0.054249382	8.407455569	0.931045642	0.334591363	0.572087272
MMP15	-0.158791874	8.410957657	3.213464786	0.073034674	0.228860693
HNRNPA0	0.059144153	8.411289118	1.173034112	0.278778688	0.514181965
GNB4	0.139540731	8.411667376	7.644910104	0.005693273	0.041755635
RBM5	0.114399846	8.412472592	0.862846452	0.352943356	0.589542462
SLC38A1	-0.012246084	8.412574878	0.048467605	0.825751536	0.917656617
U2AF1	0.007453638	8.413078054	0.016551743	0.89763177	0.953183452
ALDH1A2	0.437858558	8.41806284	31.17864834	2.35E-08	1.89E-06
PTMS	-0.011418707	8.422524584	0.042694125	0.836302292	0.922651976
EIF4A3	0.045809802	8.423040677	0.763227515	0.382320364	0.616769322
HDGF	0.033266761	8.42884877	0.377720081	0.538825943	0.739913285
FSCN1	-0.088065299	8.431975059	1.813431579	0.178097127	0.398882296
PSAP	-0.080834897	8.433770478	2.38285753	0.122672972	0.317392968
LAMA5	0.028180023	8.434053685	0.140726978	0.707559364	0.848656708
ZNF423	-0.043842023	8.438598706	0.407165675	0.523411614	0.727919042
RTN4	-0.074717125	8.439353663	1.625138809	0.20237674	0.429146995
PPIB	0.005060704	8.441987274	0.007408112	0.931410463	0.96989996
NOP56	0.093342987	8.448520603	2.165077843	0.141177474	0.348096246
CBX1	-0.020392934	8.449821275	0.106944699	0.74364973	0.869767312
SLC16A1	-0.007230826	8.450851903	0.00960949	0.921910055	0.964486558
KIRREL	0.212236217	8.453646502	13.00735742	0.000310269	0.005064965
TMPO	0.130810414	8.457886175	4.158178907	0.041433929	0.159745855

COL5A1	-0.102156799	8.458006015	1.779866505	0.18216572	0.404441654
SRSF2	-0.060528868	8.461386587	1.045508376	0.306543821	0.544727708
UPF1	-0.066095846	8.461447669	1.575785557	0.209368796	0.437148019
MYL6	0.038558116	8.464241189	0.205482664	0.650331324	0.813532153
AHCY	-0.063222327	8.464846329	0.607162054	0.435858352	0.662239075
HNRNPDL	-0.034897744	8.466775919	0.459238679	0.497979993	0.709566121
C1QBP	0.178914871	8.468529527	7.965791046	0.004766965	0.037035135
RAD21	-0.003109803	8.468785133	0.002949009	0.956692344	0.981261144
BCLAF1	-0.094493837	8.470449605	2.725969312	0.098728167	0.27572505
SHISA2	0.002638539	8.474510685	0.001180835	0.972587461	0.989117842
RPL35A	0.098088842	8.47611947	1.217476797	0.269856401	0.504595753
CTNNA1	0.059846682	8.478596272	1.507917908	0.21945707	0.448737316
TTYH3	-0.212331227	8.482378616	11.83352718	0.000581737	0.008105403
CKAP2	0.205631225	8.485118471	10.27348066	0.00134956	0.014877209
SRSF10	0.109796788	8.488788931	3.296237348	0.069438772	0.222057458
COL4A6	0.089537945	8.489663437	3.214178921	0.073002809	0.228860693
EFTUD2	-0.008664709	8.491523059	0.02500909	0.874344414	0.942721236
H1FX	0.33882941	8.496962971	14.84146716	0.000116936	0.002321086
ERLIN2	-0.008512909	8.498111707	0.02861617	0.865668317	0.937967577
EPRS	0.111172916	8.498172064	3.112071642	0.077714055	0.238179322
EIF2S3	0.091222661	8.498375324	2.96156926	0.085265151	0.252299598
SYNCRIP	-0.050433918	8.499408244	0.815715655	0.366435793	0.601466431
MKI67	-0.022712299	8.502443518	0.09861034	0.753503697	0.87513434
ST13	0.061282358	8.50832692	1.044471866	0.30678371	0.544790871
LTBP1	0.18158541	8.50873067	7.886655887	0.004980086	0.038203877
RPL35	0.108573397	8.50922726	2.665584667	0.102540138	0.283198721
KDM5B	0.178303475	8.509587385	5.884556738	0.015274242	0.082977106
UBA52	0.010270274	8.513342169	0.021348756	0.883832867	0.948004435
NOLC1	0.15043764	8.513991889	3.170975624	0.074957658	0.232906818
EIF3L	-0.003147119	8.51693993	0.002975501	0.956498449	0.981184588
CRMP1	0.060037312	8.517940583	0.534543165	0.464703056	0.685494288
TPR	0.063491887	8.524208585	1.066786726	0.301672378	0.539435949

MAPRE1	0.121510951	8.524834571	6.125909707	0.013321472	0.076059966
RPL34	-0.041715529	8.525691967	0.264400058	0.607113243	0.786741882
MCM2	0.090475434	8.525881015	2.133164945	0.144142864	0.352809999
PSMD1	0.035259229	8.53245239	0.395951321	0.529187606	0.733039991
LRPPRC	0.156550852	8.540917053	6.202513698	0.012756901	0.073706043
BAZ1B	0.13489158	8.541196453	5.440501119	0.019675024	0.098225885
CNN3	0.251559594	8.541933089	23.12354906	1.52E-06	6.74E-05
EFNB1	0.079136418	8.545677903	1.455461271	0.227653663	0.457727398
MANBAL1	-0.295187723	8.548231938	4.478489966	0.034324035	0.141480755
VCL	0.080354796	8.549280164	2.015504149	0.155699545	0.369116269
PTGES3	0.15489355	8.549749403	7.385601101	0.006574809	0.046362842
RPN1	-0.063031602	8.552379775	1.532055565	0.215804203	0.444608321
RRM2	0.061636911	8.553628739	0.715149622	0.397739545	0.630102912
KRT7	0.074355816	8.55739325	0.072686016	0.787465352	0.895442461
PRPF19	0.063740009	8.575200606	1.703355549	0.191849728	0.416091641
SMARCA5	0.017768763	8.579264377	0.076345292	0.78231265	0.892887345
LOC112531056	0.090009409	8.580209036	0.762993833	0.382393231	0.616769322
RCC2	0.044062484	8.581556414	0.609416427	0.43500762	0.661252025
RBFOX2	-0.136349313	8.584587085	4.75831083	0.029157149	0.126959748
COL4A5	0.052490659	8.590227624	0.685350306	0.407750431	0.63839518
RPL36A	-0.017271219	8.592689537	0.065668689	0.797750832	0.901282145
BTF3L4	0.020984139	8.600249422	0.133903809	0.714418285	0.85211314
PLK1	0.153347944	8.600629741	7.373734384	0.006618335	0.04653862
JPT2	0.315663403	8.605992452	26.8971357	2.15E-07	1.30E-05
DDX19B	-0.015659408	8.608561845	0.080184195	0.77704795	0.889536847
INTS13	-0.007398596	8.61300487	0.015160496	0.902005767	0.955177611
BZW1	0.039960597	8.614202791	0.656604427	0.417761274	0.647659861
PTN	0.01607891	8.622367363	0.009842034	0.920973896	0.963850757
DKC1	0.003627319	8.626944716	0.003223384	0.954724548	0.980541221
UBTF	0.040984808	8.627747828	0.426756759	0.513584663	0.720825354
NUDT16L1	0.011907779	8.632000272	0.031972642	0.858087715	0.933773189
RPL37	0.060595735	8.632175184	0.355047629	0.551269604	0.749867019

NOTCH1		-0.206768794	8.635384426	5.304008149	0.021276411	0.103502261
CDH11		-0.14512156	8.637956677	3.248965301	0.07146856	0.225982928
CKB		0.114801754	8.639349714	1.3283979	0.249090433	0.483290069
COL5A2		0.013499689	8.639810752	0.021570717	0.883234847	0.947866002
FKBP4		0.225615282	8.641145726	13.92515092	0.000190236	0.003438729
GNB1		0.034318424	8.648071169	0.550456928	0.458131054	0.680801379
ATF4		-0.019368142	8.648768951	0.117462792	0.731802755	0.862408819
H2AFZ		0.069873866	8.65029514	1.501868154	0.22038414	0.44987235
CADM1		0.08385639	8.653256372	1.201346864	0.27305265	0.507692808
VCAN		0.123020286	8.653585592	1.676539128	0.195384956	0.420319684
VCP		0.105082233	8.653667905	1.305398121	0.253229544	0.487812239
PALD1		0.085245239	8.658125223	2.975095645	0.084555129	0.251101723
LOC101749377		0.146532863	8.658990392	6.473453732	0.010949754	0.066217639
BRD2		0.077068785	8.660164201	2.002373092	0.157053154	0.371415586
GPI		-0.067033657	8.661765108	0.95695874	0.327954312	0.565610335
CDC42		0.111537173	8.66194105	5.613056623	0.01782714	0.092131151
SUB1		0.011061523	8.665534448	0.01658586	0.897526904	0.953133605
PHB2		-0.033603114	8.668639487	0.427969724	0.512986859	0.720361731
HNRNPR		0.069138536	8.670295591	1.507631461	0.21950086	0.448737316
TOP2B		-0.031012671	8.673678883	0.307396476	0.579282223	0.76846896
RRM1		-0.003894098	8.677724225	0.003579401	0.952292551	0.979802034
EIF3D		0.188085955	8.679966559	9.621185728	0.001923455	0.019585677
CCDC50		0.005521032	8.684697893	0.01022295	0.919464247	0.962786027
PDIA6		0.146085068	8.69392889	6.483073632	0.01089065	0.065951574
	02/set	-0.042119457	8.695102687	0.486761207	0.485375624	0.700048724
MAP4		-0.075009059	8.695116351	1.711060534	0.190847829	0.414683881
LOC107050721		0.05301731	8.697762504	0.529403411	0.466857786	0.686608642
CKAP5		0.021563096	8.697825757	0.13174537	0.716629199	0.853204072
AGRN		-0.000238684	8.701297774	7.46E-06	0.997820154	0.998973634
NRP2		0.222232254	8.711593136	8.38615469	0.003780899	0.031247966
FOXP4L		-0.07287642	8.714122854	1.064009688	0.302302447	0.54010714
ETNK1		0.040404677	8.714897602	0.471952496	0.492090298	0.704729839

RPL31	-0.050403804	8.717670854	0.476367147	0.490072474	0.70373877
TNPO2	0.115422638	8.719812551	3.786455208	0.051668963	0.183392983
FAU	0.156143278	8.722758118	4.735016888	0.029554558	0.127936597
CANX	-0.065034248	8.724078052	1.020302911	0.312447169	0.550344483
XPO1	0.409538803	8.724315935	33.67098625	6.53E-09	5.97E-07
ARHGDIA	-0.049207914	8.724738368	0.584049591	0.444729374	0.669535393
PGK1	0.222810483	8.725437677	6.368762207	0.011614658	0.069095879
PRPS1	0.002760135	8.725632032	0.002430419	0.960680792	0.9832549
WLS	-0.139060821	8.7285714	4.590447418	0.032150621	0.135650888
LOC426023	0.075648309	8.729060128	1.241718863	0.265140044	0.500215481
SMARCE1	0.007436306	8.729120921	0.018180239	0.89274303	0.951217859
SSRP1	0.105126969	8.731254309	3.822525202	0.050567973	0.181174895
PCNA	0.041512295	8.736835489	0.326909838	0.567484378	0.760729568
EIF3B	0.04710159	8.737605666	0.745738088	0.387828941	0.621879285
COPA	0.024217647	8.73940064	0.215535803	0.64246267	0.809078732
MIB1	-0.119697964	8.739416076	2.495926404	0.114141194	0.303152969
RPS15	0.052178355	8.740846846	0.274507372	0.60032409	0.782504191
QKI	0.075489	8.75511587	1.727664373	0.188709454	0.412324268
MEIS1	0.079090271	8.755820379	0.932018366	0.334339003	0.572064915
ZNF207	0.07139097	8.758398167	1.381523096	0.239841879	0.472323431
OIP5-AS1	-0.004904628	8.765766938	0.006371387	0.936379645	0.972171218
NREP	-0.256371711	8.770199514	3.079103972	0.079304205	0.240988124
07/set	0.075019712	8.773066299	1.61281504	0.204096545	0.430927383
ATP1A1	0.045486608	8.781402754	0.575692421	0.448005725	0.671347758
SRRM1	-0.042504753	8.78147278	0.709331364	0.399665872	0.63209203
H3F3C	0.016072872	8.785173885	0.080465051	0.776668175	0.889376916
CBX3	0.075812636	8.786302667	1.676738697	0.195358367	0.420319684
BASP1	-0.0880916	8.790504391	1.208433944	0.271642497	0.506501676
PSMD2	0.018814441	8.793799698	0.140156381	0.708125598	0.848914958
FTH1	-0.048830007	8.794457125	0.585054799	0.444337794	0.669252027
CRABP2	-0.115481075	8.799838636	1.167976201	0.279817467	0.515148403
SNRNP200	-0.102433506	8.79990606	2.948062348	0.08598059	0.253686679

	09/set	-0.129091641	8.801046392	3.372909823	0.066276599	0.216067281
TNRC18		-0.00666748	8.803407157	0.012386496	0.911382707	0.959365561
CAD		0.183090152	8.805482264	8.152861775	0.004299326	0.034176527
CCT8		0.180207001	8.809778309	7.649275617	0.005679512	0.041753247
DNMT3B		0.104292197	8.812034381	3.568009577	0.058902875	0.199896572
RPS25		-0.04574932	8.812974791	0.556325253	0.455744655	0.678299412
IPO5		0.063371048	8.81751048	1.398512326	0.236972815	0.469296867
TUBB2B		0.171760904	8.825538143	5.076693765	0.024249569	0.112764397
HP1BP3		-0.025165773	8.834282759	0.167925931	0.681961715	0.833288668
VDAC2		0.02027336	8.835476699	0.132590112	0.715761494	0.852973304
ZC3H13		-0.048090182	8.838486294	0.652152867	0.419344023	0.649012971
DDX6		0.005638277	8.840204325	0.007604868	0.930507854	0.969586826
THRAP3		0.003518363	8.84478019	0.004445047	0.946843471	0.977487125
CSNK1A1		0.004762551	8.847409721	0.008460853	0.926711632	0.967217925
	11/set	-0.114695415	8.851515449	1.462116515	0.226593661	0.456544471
DNMT3A		-0.036910152	8.855829292	0.251525752	0.616002792	0.792054659
CCT2		0.116324058	8.859164572	2.817006792	0.093270186	0.267193753
ACLY		0.076026695	8.870310015	1.359338977	0.243652075	0.47663078
RPL30		-0.029550585	8.876135779	0.189473596	0.663355101	0.821350448
SMC2		0.48326434	8.876831585	28.52460171	9.25E-08	6.32E-06
CCT6A		0.180468604	8.880164317	8.078695479	0.004478868	0.035279926
TUBA3E		0.103125092	8.888000183	2.25535005	0.133153343	0.335082178
RPL24		0.018866402	8.893437179	0.086013381	0.769307849	0.884870729
SLC38A10		-0.188852672	8.893573014	5.670085472	0.017256615	0.090432358
CNOT1		-0.100263541	8.894577833	1.262613935	0.261156888	0.496056399
MACF1		-0.072677008	8.897860826	0.933644528	0.333917684	0.571760197
PRRC2B		0.060774633	8.907393255	0.472839978	0.491683539	0.704515207
DBN1		0.030819746	8.908648233	0.233622024	0.628851062	0.800641717
KIF4A		-0.018065829	8.912364039	0.074564653	0.784803027	0.894316745
TPX2		0.07952839	8.91745187	1.658938627	0.197746689	0.423467175
NCAM1		0.191557255	8.926306125	4.098269591	0.042927127	0.163356737
COL18A1		0.144633492	8.938069547	5.435612347	0.019730172	0.098411633

ILF2	0.097947161	8.940674312	3.117261426	0.077466876	0.237687384
HBBR	0.218762063	8.941050514	0.69933508	0.403007207	0.63489551
SEC31A	0.178107653	8.946552088	7.531392015	0.006063304	0.043721152
HSPG2	0.108000205	8.950560485	1.504871272	0.219923356	0.44921038
ADNP	-0.027925862	8.954959007	0.303359325	0.58178405	0.770421418
SPTAN1	0.126020041	8.956149493	4.57176212	0.032503116	0.136508109
PTK7	0.094466398	8.95674491	3.003417019	0.083089104	0.24809131
MAPK6	0.012417125	8.965117891	0.057935677	0.809788947	0.90863124
KPNA2	0.208193038	8.968335109	11.52232556	0.000687653	0.009162212
CLTC	0.002059152	8.976467072	0.001015045	0.974583882	0.989871494
SF3B1	0.052592629	8.987715092	1.03548815	0.308873136	0.546917836
RPLP2	-0.035447793	8.988627534	0.346541597	0.556077407	0.752930284
ARF1	0.079635299	8.990266029	2.444432948	0.117942499	0.310122056
RPL27	0.012713878	8.994447505	0.039836624	0.841800356	0.925494032
RPS15A	0.006574308	8.99497606	0.007167716	0.932529816	0.970512816
HMGB3	0.046154704	8.996732702	0.59668071	0.439847295	0.664967091
CCT4	0.248663608	8.998251516	15.70785173	7.39E-05	0.001643648
DDX17	0.205958895	8.998922164	8.60174302	0.003358414	0.02866323
GPC4	0.036362773	8.9996426	0.360661139	0.548139285	0.747409618
LOC776067	-0.136527889	9.002212423	2.397964401	0.12149325	0.315600463
TCF12	0.045793737	9.003578066	0.424955292	0.514474749	0.721527487
ODC1	0.062159977	9.005924427	0.958706564	0.327512973	0.565382499
TPM1	0.198717452	9.010332051	12.66974229	0.000371621	0.00578937
RPL37A	-0.11123376	9.016086247	2.180946998	0.139728462	0.345541304
RPS16	-0.114060587	9.01761999	2.63308666	0.104658074	0.287024769
RPL11	0.080529987	9.024424455	1.575266056	0.2094439	0.437249349
ACTG1	0.080535216	9.0271373	1.725296841	0.189012651	0.412574387
DTX4	0.255419531	9.027441688	6.861913029	0.008805223	0.056752818
FASN	-0.063948075	9.029124718	0.346951078	0.555844146	0.752912164
OGT	0.120360083	9.034560767	3.890884846	0.048548839	0.175924057
NACA	0.056578054	9.035590921	1.192518603	0.274822112	0.509715719
EIF4G1	0.019436479	9.044146362	0.099276632	0.752699436	0.874740262

HNRNPUL2	0.12774777	9.049558193	4.663242364	0.030814948	0.131703889
DPYSL2	0.012468989	9.052100048	0.039614	0.842237196	0.925580035
RPL39	0.010250761	9.055208212	0.026446739	0.87081407	0.940547786
LBR	0.062741321	9.057578532	0.981525769	0.321822409	0.559491572
RPS17	0.068479267	9.059216816	0.62814282	0.428037359	0.655561685
HDAC2	0.062640518	9.067692188	1.244893845	0.264529971	0.499779071
ITGB1	0.049612132	9.069991658	0.729148818	0.393159229	0.62615516
FLNA	0.188715352	9.077839258	3.86796667	0.049216112	0.177598932
LAMC1	0.076080274	9.087419666	0.855727001	0.354937229	0.591551807
EIF5	0.07488296	9.090722554	1.723727559	0.189213933	0.412714112
ARPP19	0.069539972	9.094486578	1.399093912	0.236875338	0.46916029
COL1A1	-0.281896819	9.105275739	4.54174334	0.033077888	0.138357052
SRSF3	0.058329856	9.10827933	1.136466822	0.286400146	0.52276366
KDM1A	-0.010090009	9.109556664	0.031129761	0.859951204	0.934396267
LRP1	0.017784239	9.113598884	0.048024694	0.826536809	0.917847428
HSPA9	0.056977922	9.114628365	0.948886845	0.330002803	0.567655878
RPL23A	0.016730425	9.132629703	0.048698806	0.825343116	0.917552618
SPTBN1	-0.079451281	9.139534755	1.135505852	0.286603971	0.522903685
CCT5	0.110177899	9.147326156	3.124931746	0.077103101	0.237279132
SF3B3	0.110116129	9.152596218	2.541708295	0.110874769	0.298209272
CALM2	0.080347041	9.155032713	1.846820741	0.174153614	0.393585733
PFN2	-0.020861474	9.15847728	0.136248909	0.712038954	0.850384743
RPL12	-0.071015992	9.159028862	1.047087314	0.30617886	0.544431936
PARP1	0.081240689	9.169324581	1.841000191	0.174833764	0.394310525
LARP1	0.005409622	9.169514377	0.008962741	0.924575554	0.966229608
RPL27A	0.04513601	9.172005543	0.397240484	0.528517838	0.732173853
RPS11	0.093715311	9.176106462	2.425687897	0.119360835	0.311808626
IGF2BP3	0.088517122	9.176933679	2.285024236	0.130628101	0.330842759
BSG	0.059549615	9.181369453	0.915212755	0.338735094	0.57576162
RPS14	-0.049561752	9.185284925	0.505555661	0.477069131	0.693600685
G3BP1	0.114470505	9.193574585	3.767181859	0.052267625	0.1847216
GDI2	0.063741876	9.20005266	1.316183931	0.251278066	0.48581607

EIF4A2	0.38024517	9.204211653	12.92457447	0.000324296	0.005214537
HSPA5	0.506183164	9.208575069	25.37408825	4.72E-07	2.56E-05
RPL26L1	-0.061636125	9.209436831	0.607428818	0.435757551	0.662208211
SON	-0.0843147	9.209539331	1.198626092	0.273596451	0.508416669
CALM1	-0.140899718	9.220194376	6.926836814	0.008491174	0.055379415
RPS19	0.006133581	9.226771511	0.006796173	0.934297701	0.971393237
ANP32E	0.082147861	9.237156689	2.182440767	0.139592928	0.34532758
CCT7	0.165986848	9.241253664	6.730967301	0.00947532	0.059703249
ILF3	0.045352223	9.243713806	0.723947638	0.394852057	0.627589842
P4HB	-0.039981793	9.248335795	0.273840178	0.600767304	0.782591357
IPO7	-0.00394619	9.253681371	0.004150159	0.948634427	0.978362864
HNRNPK	0.060887722	9.255057434	1.012018185	0.314419829	0.552020128
PTPRS	0.080574631	9.259242924	1.997363213	0.157573115	0.372092155
RPS27A	-0.067200911	9.263869742	0.83285372	0.361448642	0.597823318
RPL23	0.091505901	9.272100272	1.786842299	0.181311371	0.40334986
SMARCA4	0.106319051	9.274624783	2.024237771	0.154806581	0.367794151
APP	-0.075327186	9.282063491	1.585677039	0.207944853	0.435667502
RPS23	0.129042446	9.282911772	1.323620848	0.249943254	0.484259537
TCP1	0.107658675	9.284109567	3.032938174	0.081590145	0.245217503
LAMB1	0.037442438	9.302508367	0.409590929	0.522177207	0.727001094
ALDOC	0.04272923	9.308581885	0.438654374	0.507772648	0.717091444
HINTW	0.399813799	9.322956713	0.310362713	0.577457695	0.76705002
TPI1	0.119545346	9.329430157	3.770915145	0.052151093	0.184468767
EEF1D	-0.005498877	9.333152891	0.007016037	0.933245832	0.970889559
HMGN5	0.026540505	9.337006243	0.179011794	0.672223868	0.826580378
RPL9	0.027182633	9.342911399	0.157269929	0.691682567	0.839091293
RPS20	0.037496616	9.349809335	0.276110295	0.599262075	0.781804074
UBB	0.238982093	9.350111975	7.300220677	0.006894614	0.047809051
PTBP1	0.010886059	9.350671053	0.030494032	0.861373985	0.935139468
MYH9	-0.118444643	9.35092551	3.210173499	0.073181723	0.229067006
NAP1L1	0.16415621	9.378036755	7.257871801	0.00705909	0.048485265
PDIA3	0.066905738	9.382032476	1.530469674	0.216041969	0.444927484

FLNB	0.0168227	9.384359609	0.040540112	0.84042823	0.92472394
TPT1	-0.052040528	9.393159635	0.847126656	0.357366466	0.593806442
FUS	0.192303132	9.399092816	9.701291569	0.001841385	0.018937498
EIF4H	0.017127249	9.399452136	0.1036635	0.747476811	0.872702382
YWHAB	0.06376009	9.401210662	1.362622448	0.243083505	0.476013219
RPL32	0.000445389	9.405375666	4.14E-05	0.994865987	0.997594138
HMGB2	0.088885441	9.413634556	1.889147324	0.169298126	0.387724519
CNBP	-0.02707782	9.419616041	0.143451079	0.704873994	0.847177093
ARIH1	-0.143604908	9.420687579	2.363200295	0.124227106	0.319849324
RPS7	0.027297522	9.425932887	0.131454127	0.716929089	0.853499361
XPO5	-0.039364009	9.436470149	0.327454753	0.567161679	0.760668794
EEF1B2	0.106014803	9.450310221	2.941805395	0.086314209	0.254534107
SMARCC1	0.016819468	9.453738497	0.071178448	0.789628627	0.89658489
PA2G4	0.046381335	9.456338643	0.454735747	0.500094561	0.710749352
AKAP12	-0.124937124	9.460982482	3.176687483	0.074696016	0.232524204
NME2	0.016536077	9.467216147	0.05411541	0.816050911	0.912856407
PGAM1	0.172322675	9.469509342	6.47548364	0.010937255	0.06616637
YWHAZ	0.056879395	9.469721563	1.172514943	0.278885089	0.514237354
RBBP4	0.071122954	9.477722474	1.276955919	0.258465799	0.492901839
RPL15	-0.026530934	9.487029054	0.15527856	0.693541167	0.839813077
PBX1	0.109649088	9.495724234	4.145574828	0.04174348	0.160488075
SLC25A3	0.027479134	9.496230656	0.238321772	0.625420856	0.798796471
RPS10	0.03587127	9.49822323	0.319329243	0.572011025	0.763812568
TP53I11	-0.19593869	9.498302532	3.903387672	0.048188846	0.175120905
MATR3	0.073907658	9.498639871	1.717613601	0.190000517	0.413388669
HNRNPM	0.042422697	9.523167609	0.499414822	0.479757357	0.696094464
RAN	0.123572579	9.538296787	3.389142609	0.065627067	0.214698165
ITGB1BP3	0.102520227	9.54477581	3.171364797	0.0749398	0.232906818
SRSF6	0.05611464	9.569002593	0.957611985	0.327789271	0.565526033
PRPF8	-0.037968461	9.569414627	0.254482976	0.613936016	0.790936713
CAPRIN1	0.125582918	9.572834291	3.998619246	0.045537554	0.169060891
TPM3	0.100442806	9.575422883	3.265135161	0.070767191	0.224585173

PRDX1	0.119120432	9.578124991	2.401111567	0.121249069	0.315339564
RHOA	0.068726743	9.587463673	1.37527966	0.240906832	0.473610744
IGDCC3	-0.130981543	9.588456067	2.616152102	0.105780674	0.28923579
RPL17	0.125626646	9.590375424	1.578655578	0.208954453	0.436670755
RPLP1	-0.181474722	9.595094351	6.063520253	0.013800233	0.077865936
RPL19	0.096564893	9.600741947	2.263540242	0.132450964	0.334020017
H3F3B	-0.022163063	9.603271759	0.090835973	0.763117067	0.881102924
ATP5A1Z	0.098992144	9.617518508	1.216754487	0.269998527	0.504637183
EIF3A	-0.011793817	9.620021804	0.036561784	0.848360005	0.929334244
RPL10A	0.056896601	9.62642754	0.566163732	0.451787387	0.675157701
RPL21	0.041651416	9.626775473	0.312872643	0.575922743	0.765833434
CHD4	-0.044274399	9.627500187	0.521321145	0.470278662	0.68920871
SET	0.010136559	9.62954206	0.02621595	0.871374047	0.940731028
RPS12	0.102674406	9.64223998	1.850847763	0.173684827	0.393282486
HSPD1	0.159090938	9.676151026	5.308013304	0.02122755	0.103346466
HNRNPU	0.147321932	9.681574666	6.255928985	0.012377832	0.072225967
KPNB1	-0.024446608	9.704262026	0.14397402	0.704361831	0.846807942
RPS24	0.02074228	9.713454973	0.07688189	0.781568311	0.892360988
DYNC1H1	-0.090544059	9.725137413	1.088781424	0.296741364	0.533680327
COL1A2	0.026862281	9.737760977	0.064480161	0.799550069	0.902345328
HBZ	0.261957734	9.742072485	0.938401263	0.332689335	0.570309721
RPS3	-0.118079427	9.750170589	2.59998608	0.106864654	0.290942732
SLC25A6	-0.029761313	9.750477223	0.265579298	0.606312752	0.786447764
PRRC2C	-0.016475384	9.755873058	0.052702135	0.818426344	0.914146449
NASP	0.096333748	9.76653922	1.599791747	0.205932726	0.4328849
FUBP1	0.043860349	9.797551172	0.526862037	0.46792912	0.687479792
HSP90B1	0.32542125	9.798872787	11.59500351	0.000661292	0.008919318
RPL13	-0.058568806	9.819016176	0.518571232	0.471451791	0.690190322
SERBP1	0.089931306	9.825060354	2.591617229	0.107430593	0.291842445
TUBA1A	0.004284377	9.856613372	0.001542585	0.968670535	0.987147683
HMGB1	0.027884218	9.864763162	0.216391655	0.641803159	0.808610358
MYH10	-0.043197793	9.866889761	0.228888641	0.632349111	0.803057713

NONO	0.029964679	9.879677259	0.223712463	0.636225651	0.80506714
UBC	0.019508788	9.886834569	0.096956072	0.755513505	0.876540558
YWHAQ	0.0446107	9.89664673	0.683027107	0.408546296	0.638972464
HMGN4	0.077143148	9.910448636	1.658049657	0.197866861	0.4235471
HNRNPH3	0.090586901	9.918210531	2.15363208	0.142233066	0.349476647
VIM	-0.049733171	9.921586457	0.433615455	0.510220211	0.718341124
LDHB	0.094769883	9.925323234	1.618424152	0.203311657	0.430265966
SRSF1	0.100861711	9.949502852	2.75427076	0.096995033	0.272969604
FN1	-0.162974907	9.95452371	3.326649464	0.068165606	0.219460976
RPL7	0.071292431	9.965205367	0.892911908	0.344689426	0.581491133
DSTN	0.010348947	10.01764491	0.030870003	0.860530723	0.934840764
TMSB4X	0.098464057	10.0181931	1.488835813	0.222397178	0.452018968
TUBA1A1	-0.099422545	10.04156996	1.610618468	0.204404886	0.431179727
CALR	0.124427935	10.04356445	2.689745524	0.100995943	0.279967694
RACK1	0.115016904	10.050814	3.057629172	0.08035884	0.242981389
FBN3	0.055602738	10.08749511	0.41343297	0.520232188	0.725779864
TOP2A	0.028258679	10.09152179	0.162056741	0.687269942	0.837147597
MDK	-0.185517542	10.10133681	3.252697526	0.071306016	0.225685804
RPS8	0.070569458	10.11612696	0.988754656	0.320046938	0.557465054
RPS4X	0.040310873	10.11652367	0.277922356	0.598066222	0.780984024
ATP5F1B	0.04410976	10.12497505	0.474215529	0.491054194	0.704288047
HNRNPAB	0.079843058	10.13809169	2.096257723	0.147660155	0.357736063
LOC112533603	0.337885746	10.16123085	1.344950384	0.246162841	0.47986029
CTNNB1	0.065148033	10.16664083	1.293370343	0.255427767	0.490361188
SFPQ	0.035150991	10.16824174	0.350267491	0.553961749	0.751850922
RPL5	0.064426954	10.17004781	0.897202716	0.343532875	0.580582484
PABPC1	0.052006561	10.18495987	0.614762929	0.433000099	0.659540556
CSDE1	0.060096587	10.19050371	1.039357904	0.307970853	0.545966429
YWHAE	0.052187022	10.19710844	0.731753446	0.392315418	0.625832839
HNRNPA1	0.013249337	10.19740911	0.042172626	0.837291066	0.923303231
RPL8	0.038796872	10.1997858	0.235525711	0.627456525	0.799999777
ANP32B	-0.02534166	10.20355173	0.161927888	0.687387725	0.837167124

RPL6	0.097890035	10.20869438	2.226696701	0.135643573	0.338760584
STMN1	-0.001525009	10.22626485	0.0006065	0.980352305	0.991682374
HNRNPA2B1	0.047069708	10.23511656	0.650755465	0.419842711	0.649357252
EIF5A2	0.050533922	10.24144959	0.490274767	0.483804763	0.698351687
DDX5	-0.126509218	10.28018522	3.803688137	0.051139822	0.182222989
DDX3X	-0.048079159	10.28026002	0.388030227	0.533336619	0.735495698
RPLP0	0.09359794	10.28166727	2.102745551	0.147034909	0.356589452
FSTL1	-0.048452582	10.2838989	0.544159322	0.460714062	0.682585354
RPL7A	-0.044145881	10.3311558	0.367350317	0.544452127	0.744408787
RPS3A	0.042145048	10.33592326	0.402332143	0.525887292	0.730312718
COL2A1	0.087956455	10.34937841	0.606412535	0.43614176	0.662345819
HDLBP	-0.002047614	10.38099037	0.001053655	0.97410517	0.989804901
HSP90AB1	0.069169595	10.41412215	0.848953665	0.356848509	0.593318715
NES	-0.005162108	10.42386349	0.002550073	0.959725344	0.982779298
PTMA	0.041875892	10.44861268	0.298673575	0.584715168	0.771586017
HNRNPH2	0.017259376	10.50473371	0.081850197	0.774805565	0.88883335
CIRBP	-0.090083613	10.52936301	1.371900279	0.241485658	0.474295357
RPS2	0.079465979	10.64655777	1.072707233	0.300334746	0.53775933
RPS6	0.135643376	10.65907852	2.047898877	0.152416409	0.364536629
HSP90AA1	0.834944785	10.68308876	19.27042331	1.13E-05	0.000361211
LOC112533602	0.326527447	10.71620499	1.210575252	0.271218225	0.506356841
RPL3	0.113561903	10.71912643	2.53292989	0.111493023	0.299285106
RPL4	0.062122048	10.79476869	0.755561165	0.384721225	0.619431287
PKM	0.203257496	10.80797199	8.174378605	0.004248622	0.033904499
HSPA8	0.594582483	10.88731401	18.49837079	1.70E-05	0.000492635
LOC112530942	-0.167648115	10.90152005	2.522438498	0.112236904	0.299960737
YBX1	-0.022741739	10.93190978	0.114119995	0.735501467	0.864785755
HNRNPA3	0.044677969	10.96323204	0.484085673	0.48657748	0.700983403
PPIA	0.0698898	11.04293393	0.808615061	0.368530025	0.603219096
NCL	0.066044717	11.12834934	0.729544245	0.393030956	0.62615185
RPSA	0.107724428	11.13082002	1.890179643	0.169181661	0.387628701
NPM1	0.008941998	11.19779529	0.013500044	0.907502208	0.957538022

ENO1	0.269756949	11.20888214	8.742953701	0.003108007	0.027073717
EEF2	-0.063453675	11.23615704	0.876244602	0.34923232	0.585991041
EIF4G2	0.018119972	11.59030741	0.075048829	0.784122728	0.893912948
ACTC2L	0.090084629	11.81522127	1.367881068	0.242176279	0.475028094
GAPDH	0.152273405	11.84275074	2.783911582	0.095215363	0.270318978
TUBB	-0.066713638	11.94221872	0.656660947	0.417741236	0.647659861
ACTB	0.086345474	12.2484751	1.494568732	0.221508946	0.451003135
LOC101750014	-0.205548857	12.59860633	3.919617724	0.047725731	0.174032656
EEF1A1	0.04598152	13.02300614	0.378233339	0.538550243	0.739658146
LOC112533599	0.153676865	13.25386423	0.167173368	0.682636241	0.833665624
