# Supplementary material for: Phenotypic divergence between broiler and layer chicken lines is regulated at the molecular level during development
Source: BMC Genomics. 2024 Feb 12;25:168. doi: 10.1186/s12864-024-10083-x (PMC10863267; doi:10.1186/s12864-024-10083-x)
Supplement: Supplementary file 2 — Supplementary Material 2 [file 12864_2024_10083_MOESM2_ESM.pdf]

**Table S2 - Information about DEGs showing the gene name and Ensembl ID, description of annotated gene, LogFoldChange, logCPM, LR, Pvalue and FDR**

| Gene name | Gene synonym | Gene stable ID | Gene description | log2FC | logCPM | LR | PValue | FDR |
|---|---|---|---|---|---|---|---|---|
| LOC107049387 | | - - - - - | coiled-coil domain-containing protein 81-like | 5.46 | 1.97062 | 40.28884 | 2.19E-10 | 3.03E-08 |
| LOC107049379 | | - - - - - | coiled-coil domain-containing protein 81-like | 4.84 | 3.65201 | 52.31400 | 4.73E-13 | 1.28E-10 |
| BTN3A2 | - - - - - | ENSGALG00010006046 | butyrophilin, subfamily 3, member A2 | 4.59 | -0.54104 | 96.64486 | 8.29E-23 | 8.53E-20 |
| LOC107052718 | | - - - - - | endogenous retrovirus group K member 11 Pol protein | 4.02 | 4.49362 | 478.12181 | 5.48E-106 | 9.01E-102 |
| LOC107052719 | gag; SE21Q1b | - - - - - | uncharacterized LOC107052719 | 3.31 | 3.31605 | 205.06580 | 1.64E-46 | 1.35E-42 |
| GBP | - - - - - | ENSGALG00010028163 | guanylate binding protein | 2.35 | 1.72085 | 22.23495 | 2.41E-06 | 9.92E-05 |
| HEATR7B2L2 | MROH2B; HEATR7B2L | - - - - - | maestro heat-like repeat-containing protein family member 2B | 2.33 | 0.28425 | 42.36640 | 7.57E-11 | 1.17E-08 |
| MOGL4 | MOGL; BG4 | - - - - - | myelin oligodendrocyte glycoprotein like | 2.33 | 1.48422 | 59.72453 | 1.09E-14 | 4.08E-12 |
| LOC112531062 | | - - - - - | zinc finger protein 519-like | 2.30 | -0.21369 | 24.70611 | 6.68E-07 | 3.47E-05 |
| LOC428505 | | ENSGALG00000046353 | SUN domain-containing protein 3-like | 2.20 | -0.64518 | 28.24581 | 1.07E-07 | 7.17E-06 |
| OR11A1 | - - - - - | ENSGALG00000041229 | olfactory receptor 1020-like | 2.19 | 0.50449 | 12.06927 | 5.13E-04 | 7.39E-03 |
| BTN3A3L1 | BG2 | - - - - - | butyrophilin subfamily 3 member A3-like 1 | 2.17 | 1.81418 | 32.06362 | 1.49E-08 | 1.25E-06 |
| LOC107056757 | | - - - - - | uncharacterized LOC107056757 | 2.16 | 0.65034 | 53.72986 | 2.30E-13 | 7.42E-11 |
| LOC112532660 | | - - - - - | monocarboxylate transporter 9-like | 2.15 | 1.38082 | 89.29726 | 3.40E-21 | 3.29E-18 |
| LOC101749199 | | - - - - - | uncharacterized LOC101749199 | 2.07 | 0.00455 | 40.87006 | 1.63E-10 | 2.36E-08 |
| LOC101751234 | | - - - - - | uncharacterized LOC101751234 | 2.06 | -0.25751 | 21.52971 | 3.48E-06 | 1.36E-04 |
| LOC101747302 | | - - - - - | uncharacterized LOC101747302 | 2.02 | 5.83788 | 119.07181 | 1.01E-27 | 1.66E-24 |
| C4 | C4-2 | ENSGALG00000032231 | complement 4 | 2.02 | 2.73485 | 62.11648 | 3.24E-15 | 1.30E-12 |
| LOC107054918 | | - - - - - | uncharacterized LOC107054918 | 1.95 | -0.34757 | 29.00461 | 7.22E-08 | 5.10E-06 |
| LOC107050939 | | - - - - - | zinc finger protein 135-like | 1.94 | 1.47057 | 11.92164 | 5.55E-04 | 7.86E-03 |
| MHCIA9 | MHCY6 | - - - - - | major histocompatibility complex Y, class I heavy chain 6 | 1.88 | 3.28359 | 28.77669 | 8.12E-08 | 5.59E-06 |
| LOC107051303 | | - - - - - | zinc finger protein 160-like | 1.84 | 1.78809 | 55.81257 | 7.97E-14 | 2.68E-11 |
| ACKR1 | - - - - - | - - - - - | atypical chemokine receptor 1 (Duffy blood group) | 1.84 | 0.66674 | 63.10464 | 1.96E-15 | 8.72E-13 |
| ADCY8 | - - - - - | ENSGALG00000030251 | adenylate cyclase 8 | 1.82 | 1.24948 | 43.00156 | 5.47E-11 | 8.65E-09 |
| LOC101750974 | | - - - - - | uncharacterized LOC101750974 | 1.77 | -0.68164 | 21.92255 | 2.84E-06 | 1.14E-04 |
| COLEC10 | CL-1 | ENSGALG00000033144 | collectin subfamily member 10 | 1.77 | 3.52940 | 10.69550 | 1.07E-03 | 1.26E-02 |
| C2orf50 | - - - - - | - - - - - | chromosome 2 open reading frame 50 | 1.76 | -0.63290 | 22.80235 | 1.80E-06 | 7.73E-05 |
| LOC112531790 | | - - - - - | uncharacterized LOC112531790 | 1.75 | -0.39584 | 15.17031 | 9.82E-05 | 2.04E-03 |
| LOC112533394 | | - - - - - | protocadherin beta-15-like | 1.70 | -0.51149 | 14.10428 | 1.73E-04 | 3.19E-03 |
| OR52R1L2 | - - - - - | ENSGALG00000053623 | olfactory receptor 52R1-like | 1.67 | 0.06438 | 30.25804 | 3.78E-08 | 2.87E-06 |
| GM2A | - - - - - | ENSGALG00000027534 | GM2 ganglioside activator | 1.65 | 4.44794 | 135.27486 | 2.87E-31 | 6.76E-28 |
| ADGRG5 | - - - - - | ENSGALG00000001149 | adhesion G protein-coupled receptor G5 | 1.64 | -0.58325 | 25.77582 | 3.83E-07 | 2.16E-05 |
| LOC107051154 | | - - - - - | protocadherin beta-8-like | 1.60 | 0.72921 | 18.54055 | 1.66E-05 | 4.88E-04 |
| LYG2 | LYG1 | ENSGALG00000016761 | lysozyme g2 | 1.60 | -0.21761 | 15.95552 | 6.48E-05 | 1.48E-03 |
| AKR1B1L | AKR1E2 | ENSGALG00000053854 | aldo-keto reductase family 1 member E2 | 1.59 | 3.23802 | 12.89178 | 3.30E-04 | 5.28E-03 |
| MROH2B1 | - - - - - | - - - - - | maestro heat-like repeat family member 2B 1 | 1.59 | -0.74565 | 19.18100 | 1.19E-05 | 3.74E-04 |
| VSIG1 | CHT1 | ENSGALG00000008290 | V-set and immunoglobulin domain containing 1 | 1.58 | -0.64250 | 14.28824 | 1.57E-04 | 2.93E-03 |
| LOC100859910 | | - - - - - | uncharacterized LOC100859910 | 1.51 | 2.19170 | 49.91625 | 1.60E-12 | 3.83E-10 |
| BF2 | B-F | ENSGALG00000041380 | Major histocompatibility complex class I antigen BF2 | 1.48 | 2.53136 | 62.62506 | 2.50E-15 | 1.03E-12 |
| DHRS13 | - - - - - | ENSGALG00000004009 | dehydrogenase/reductase 13 | 1.48 | 1.97704 | 68.54611 | 1.24E-16 | 6.37E-14 |
| LOC107053759 | | - - - - - | uncharacterized LOC107053759 | 1.47 | 1.17686 | 28.35526 | 1.01E-07 | 6.84E-06 |
| LOC100858315 | ARHGAP33L1 | - - - - - | Rho GTPase-activating protein 33 like 1 | 1.47 | 0.09050 | 16.14343 | 5.87E-05 | 1.36E-03 |

| | | | | | | | | |
|---|---|---|---|---|---|---|---|---|
| LOC112533400 | | ----- | uncharacterized LOC112533400 | 1.45 | -0.99113 | 12.94309 | 3.21E-04 | 5.19E-03 |
| BTN3A3L2 | B-G; BG5; BG11 | ----- | butyrophilin subfamily 3 member A3-like 2 | 1.43 | 4.68356 | 29.09217 | 6.90E-08 | 4.92E-06 |
| SAMD7 | ----- | ENSGALG00000009383 | sterile alpha motif domain containing 7 | 1.40 | 1.89607 | 13.13568 | 2.90E-04 | 4.80E-03 |
| LOC107052398 | | ----- | uncharacterized LOC107052398 | 1.39 | 0.77701 | 37.73894 | 8.09E-10 | 9.31E-08 |
| OR5AS1 | ----- | ENSGALG00000043910 | olfactory receptor family 5 subfamily AS member 1 | 1.37 | 0.10685 | 15.34482 | 8.96E-05 | 1.90E-03 |
| BF1 | B-F | ENSGALG00000033932 | MHC BF1 class I | 1.37 | 2.63024 | 36.19169 | 1.79E-09 | 1.89E-07 |
| LOC107053999 | | ----- | uncharacterized LOC107053999 | 1.36 | 1.03996 | 32.25657 | 1.35E-08 | 1.15E-06 |
| LOC112531075 | | ----- | zinc finger protein 501-like | 1.36 | 0.45089 | 17.17720 | 3.40E-05 | 8.67E-04 |
| LOC112533561 | | ----- | uncharacterized LOC112533561 | 1.33 | 1.10952 | 18.17183 | 2.02E-05 | 5.68E-04 |
| LOC101748371 | | ----- | uncharacterized LOC101748371 | 1.33 | 2.62172 | 21.03708 | 4.50E-06 | 1.69E-04 |
| LOC107053444 | | ----- | uncharacterized LOC107053444 | 1.33 | 0.89736 | 30.94816 | 2.65E-08 | 2.09E-06 |
| LOC112530181 | | ----- | uncharacterized LOC112530181 | 1.33 | 1.79082 | 10.78328 | 1.02E-03 | 1.22E-02 |
| LOC107053612 | | ----- | translation initiation factor IF-2-like | 1.32 | -0.81959 | 12.30254 | 4.52E-04 | 6.72E-03 |
| LOC107054467 | | ----- | protocadherin beta-15-like | 1.31 | 0.21127 | 9.74161 | 1.80E-03 | 1.86E-02 |
| LOC112532499 | | ----- | uncharacterized LOC112532499 | 1.30 | 0.32758 | 20.44867 | 6.13E-06 | 2.20E-04 |
| SLC2A2 | GLUT2 | ENSGALG00000009306 | solute carrier family 2 member 2 | 1.30 | 1.34307 | 8.41145 | 3.73E-03 | 3.10E-02 |
| LOC112529957 | | ----- | sperm acrosome membrane-associated protein 6 | 1.30 | 3.17608 | 77.79166 | 1.15E-18 | 7.85E-16 |
| TTR | ----- | ENSGALG00000015143 | transthyretin | 1.29 | 3.12347 | 7.22748 | 7.18E-03 | 4.91E-02 |
| LOC107054533 | | ----- | uncharacterized LOC107054533 | 1.29 | -0.15858 | 10.78192 | 1.02E-03 | 1.22E-02 |
| LOC101749307 | | ----- | uncharacterized LOC101749307 | 1.28 | -0.35228 | 8.51975 | 3.51E-03 | 2.96E-02 |
| CDHR1 | PCDH21 | ENSGALG00000002314 | cadherin related family member 1 | 1.28 | -0.66578 | 10.56603 | 1.15E-03 | 1.33E-02 |
| LOC107054129 | | ----- | uncharacterized LOC107054129 | 1.27 | -0.32016 | 14.97645 | 1.09E-04 | 2.18E-03 |
| LOC101750503 | | ----- | uncharacterized LOC101750503 | 1.27 | 1.53048 | 40.86040 | 1.64E-10 | 2.36E-08 |
| TAP2 | ----- | ----- | transporter 2, ATP binding cassette subfamily B | 1.26 | 2.05204 | 53.40004 | 2.72E-13 | 8.45E-11 |
| PECAM1 | ----- | ENSGALG00000046125 | platelet and endothelial cell adhesion molecule 1 | 1.25 | 5.77834 | 75.13973 | 4.39E-18 | 2.58E-15 |
| SYPL1 | ----- | ENSGALG00000029006 | synaptophysin like 1 | 1.24 | -0.02826 | 19.70023 | 9.06E-06 | 2.99E-04 |
| SH2D1B | ----- | ENSGALG00000028448 | SH2 domain containing 1B | 1.24 | 0.74777 | 10.95612 | 9.33E-04 | 1.14E-02 |
| LOC431499 | ARHGAP33L2 | ----- | Rho GTPase activating protein 33 like 2 | 1.24 | 2.78730 | 37.71847 | 8.17E-10 | 9.34E-08 |
| CRP | APCS | ENSGALG00000022137 | C-reactive protein, pentraxin-related | 1.22 | -0.38563 | 16.73408 | 4.30E-05 | 1.06E-03 |
| LOC107050915 | | ----- | uncharacterized LOC107050915 | 1.22 | 1.38717 | 10.98934 | 9.16E-04 | 1.13E-02 |
| MUC4 | ----- | ENSGALG00000048048 | mucin 4, cell surface associated | 1.21 | -0.36391 | 7.96281 | 4.77E-03 | 3.71E-02 |
| LOC112533543 | | ----- | zinc finger protein 135-like | 1.20 | 3.97705 | 46.62441 | 8.60E-12 | 1.68E-09 |
| LOC776376 | CRPL2 | ----- | C-reactive protein like 2 | 1.20 | 0.91163 | 31.38050 | 2.12E-08 | 1.73E-06 |
| WNT11B | cWnt11b | ENSGALG00000004401 | wingless-type MMTV integration site family, member 11b | 1.20 | -0.49402 | 10.28950 | 1.34E-03 | 1.48E-02 |
| VWA5A | ----- | ENSGALG00000028815 | von Willebrand factor A domain containing 5A | 1.19 | 0.21225 | 23.45323 | 1.28E-06 | 5.84E-05 |
| LOC101749621 | | ----- | uncharacterized LOC101749621 | 1.16 | -0.65184 | 10.34655 | 1.30E-03 | 1.45E-02 |
| NKX2-1 | NKX2.1 | ENSGALG00000037632 | NK2 homeobox 1 | 1.16 | -0.23795 | 11.92039 | 5.55E-04 | 7.86E-03 |
| LOC107050034 | | ----- | uncharacterized LOC107050034 | 1.16 | 1.65606 | 38.42395 | 5.69E-10 | 6.69E-08 |
| CNGA2 | ----- | ENSGALG00000007282 | cyclic nucleotide gated channel alpha 2 | 1.16 | 0.06504 | 14.29413 | 1.56E-04 | 2.93E-03 |
| LOC101747293 | | ----- | PHD finger protein 7-like | 1.15 | -0.36903 | 9.57152 | 1.98E-03 | 2.00E-02 |
| LOC107051395 | | ----- | uncharacterized threonine-rich GPI-anchored glycoprotein PJ4664.02-like | 1.14 | 0.34103 | 14.16702 | 1.67E-04 | 3.10E-03 |
| DPF1 | ----- | ENSGALG00000052924 | D4, zinc and double PHD fingers family 1 | 1.13 | 0.68293 | 15.74443 | 7.25E-05 | 1.62E-03 |
| MC3R | ----- | ENSGALG00000027260 | melanocortin 3 receptor | 1.12 | -0.95275 | 9.20962 | 2.41E-03 | 2.27E-02 |
| HS3ST4 | ----- | ENSGALG00000006290 | heparan sulfate-glucosamine 3-sulfotransferase 4 | 1.10 | 0.71301 | 9.28501 | 2.31E-03 | 2.21E-02 |

| | | | | | | | | |
|---|---|---|---|---|---|---|---|---|
| LOC101750341 | | ----- | uncharacterized LOC101750341 | 1.10 | 2.66444 | 47.13346 | 6.63E-12 | 1.36E-09 |
| ALPI | ALPP | ENSGALG00000007814 | alkaline phosphatase, placental | 1.09 | 1.40649 | 22.69775 | 1.90E-06 | 8.04E-05 |
| TRNP1 | ----- | ----- | TMF1-regulated nuclear protein | 1.08 | -0.48361 | 12.13908 | 4.94E-04 | 7.20E-03 |
| ACSBG1 | ----- | ENSGALG00000003286 | acyl-CoA synthetase bubblegum family member 1 | 1.08 | 2.08165 | 36.96110 | 1.21E-09 | 1.35E-07 |
| GIMAP8L1 | ----- | ----- | GTPase IMAP family member 7-like 5 | 1.08 | -0.39483 | 8.95774 | 2.76E-03 | 2.49E-02 |
| CFAP161 | ----- | ENSGALG00000021183 | cilia and flagella associated protein 161 | 1.08 | -0.71654 | 9.03625 | 2.65E-03 | 2.42E-02 |
| MIR1748 | ----- | ENSGALG00000025323 | gga-mir-1748 | 1.07 | -0.89483 | 8.37801 | 3.80E-03 | 3.13E-02 |
| LOC112531929 | | ----- | uncharacterized LOC112531929 | 1.06 | 1.01669 | 15.56254 | 7.98E-05 | 1.75E-03 |
| MCCC2L | ----- | ENSGALG00000011255 | methylcrotonoyl-Coenzyme A carboxylase 2-like | 1.05 | 3.81776 | 40.13540 | 2.37E-10 | 3.21E-08 |
| OTX5 | CRX | ENSGALG00000039336 | orthodenticle-related homeobox 5 | 1.05 | 2.13916 | 7.55699 | 5.98E-03 | 4.33E-02 |
| LOC107055278 | | ----- | uncharacterized LOC107055278 | 1.05 | 1.42381 | 24.98955 | 5.76E-07 | 3.03E-05 |
| RBM11 | ----- | ENSGALG00000044391 | RNA binding motif protein 11 | 1.04 | 1.15019 | 21.15159 | 4.24E-06 | 1.61E-04 |
| COMTD1 | ----- | ENSGALG00000005000 | catechol-O-methyltransferase domain containing 1 | 1.04 | 4.20873 | 36.10497 | 1.87E-09 | 1.93E-07 |
| TDRD6 | ----- | ENSGALG00000044279 | tudor domain containing 6 | 1.02 | 0.52122 | 11.85547 | 5.75E-04 | 8.05E-03 |
| LOC107053729 | | ----- | uncharacterized LOC107053729 | 1.02 | -0.00903 | 7.40616 | 6.50E-03 | 4.60E-02 |
| LOC107050073 | | ----- | uncharacterized LOC107050073 | 1.02 | 2.85859 | 34.60588 | 4.04E-09 | 3.93E-07 |
| NKX2-8 | ----- | ENSGALG00000028483 | NK2 homeobox 8 | 1.02 | 2.23816 | 31.14889 | 2.39E-08 | 1.90E-06 |
| LCAT | ----- | ENSGALG00000028928 | lecithin-cholesterol acyltransferase | 1.01 | 1.51001 | 21.26947 | 3.99E-06 | 1.53E-04 |
| LOC107049645 | | ----- | uncharacterized LOC107049645 | 1.01 | 1.52053 | 18.61441 | 1.60E-05 | 4.74E-04 |
| GALNT15 | ----- | ENSGALG00000011235 | polypeptide N-acetylgalactosaminyltransferase 15 | 1.00 | 2.11982 | 23.66561 | 1.15E-06 | 5.31E-05 |
| SP7 | ----- | ENSGALG00000050811 | Sp7 transcription factor | 1.00 | 1.14687 | 11.27482 | 7.86E-04 | 1.01E-02 |
| LOC419830 | | ----- | uncharacterized LOC419830 | 0.99 | 1.73168 | 24.87850 | 6.11E-07 | 3.19E-05 |
| LOC101747645 | | ----- | translation initiation factor IF-2-like | 0.99 | -0.43579 | 9.01145 | 2.68E-03 | 2.44E-02 |
| SPATA17 | ----- | ENSGALG00000009616 | spermatogenesis associated 17 | 0.99 | 2.34282 | 39.69025 | 2.98E-10 | 3.83E-08 |
| APOA5 | ----- | ENSGALG00010024749 | apolipoprotein A5 | 0.98 | 1.48780 | 28.78212 | 8.10E-08 | 5.59E-06 |
| RASGRF1 | ----- | ENSGALG00000008262 | Ras protein specific guanine nucleotide releasing factor 1 | 0.98 | 0.00922 | 12.29136 | 4.55E-04 | 6.74E-03 |
| APOA1 | ----- | ENSGALG00000007114 | apolipoprotein A1 | 0.98 | 6.15499 | 22.21712 | 2.43E-06 | 9.99E-05 |
| CKMT2 | MIBCK | ENSGALG00000015602 | creatine kinase, mitochondrial 2 | 0.98 | -0.12810 | 12.23836 | 4.68E-04 | 6.89E-03 |
| LOC112531211 | | ----- | rho GTPase-activating protein 33-like | 0.97 | 1.49540 | 15.03944 | 1.05E-04 | 2.15E-03 |
| TGM6 | ----- | ENSGALG00000021232 | transglutaminase 6 | 0.97 | 0.84085 | 16.18187 | 5.75E-05 | 1.34E-03 |
| TAP1 | ----- | ENSGALG00000035075 | transporter 1, ATP-binding cassette, sub-family B (MDR/TAP) | 0.96 | 1.77047 | 16.58600 | 4.65E-05 | 1.12E-03 |
| LOC107054756 | | ----- | uncharacterized LOC107054756 | 0.96 | 0.44852 | 16.49028 | 4.89E-05 | 1.17E-03 |
| LOC112530484 | | ----- | uncharacterized LOC112530484 | 0.96 | -0.32230 | 9.51867 | 2.03E-03 | 2.04E-02 |
| CENPQ | ----- | ENSGALG00000016692 | centromere protein Q | 0.96 | 5.89574 | 180.13785 | 4.52E-41 | 2.48E-37 |
| LOC100859067 | | ----- | uncharacterized LOC100859067 | 0.96 | 2.18051 | 16.04494 | 6.19E-05 | 1.43E-03 |
| LOC112529979 | | ----- | uncharacterized LOC112529979 | 0.96 | 1.10623 | 12.31106 | 4.50E-04 | 6.70E-03 |
| LOC112532478 | | ----- | uncharacterized LOC112532478 | 0.96 | 2.10645 | 21.99277 | 2.74E-06 | 1.10E-04 |
| ACOT12 | ----- | ENSGALG00000015616 | acyl-CoA thioesterase 12 | 0.96 | -0.18415 | 9.93247 | 1.62E-03 | 1.72E-02 |
| UCP3 | UCP | ENSGALG00000017316 | uncoupling protein 3 | 0.95 | 1.24409 | 15.72881 | 7.31E-05 | 1.63E-03 |
| AGBL2 | ----- | ENSGALG00010021737 | ATP/GTP binding protein like 2 | 0.95 | 1.61916 | 13.31954 | 2.63E-04 | 4.47E-03 |
| LOC107050476 | | ----- | uncharacterized LOC107050476 | 0.95 | -0.27192 | 8.04157 | 4.57E-03 | 3.58E-02 |
| F8 | ----- | ENSGALG00000005077 | coagulation factor VIII | 0.95 | 1.64998 | 15.47932 | 8.34E-05 | 1.80E-03 |
| HAO1 | ----- | ENSGALG00000008845 | hydroxyacid oxidase 1 | 0.94 | 0.08315 | 13.23276 | 2.75E-04 | 4.61E-03 |
| AKR1B10L4 | ----- | ----- | discontued | 0.94 | 2.61349 | 12.50507 | 4.06E-04 | 6.19E-03 |

| | | | | | | | | |
|---|---|---|---|---|---|---|---|---|
| SPAG16 | ----- | ENSGALG00000003148 | sperm associated antigen 16 | 0.94 | 2.90366 | 38.94855 | 4.35E-10 | 5.30E-08 |
| GEM | ----- | ENSGALG00000032506 | GTP binding protein overexpressed in skeletal muscle | 0.94 | 1.03292 | 14.42113 | 1.46E-04 | 2.78E-03 |
| LOC112533263 | | ----- | uncharacterized LOC112533263 | 0.93 | 1.62725 | 7.25074 | 7.09E-03 | 4.86E-02 |
| LOC101747452 | | ----- | serine/arginine repetitive matrix protein 2-like | 0.93 | -0.03893 | 11.08424 | 8.71E-04 | 1.08E-02 |
| AvBD14 | DEFB1 | ENSGALG00000052020 | avian beta-defensin 14 | 0.93 | -0.19083 | 11.80476 | 5.91E-04 | 8.18E-03 |
| NT5DC4 | ----- | ENSGALG00000041161 | 5'-nucleotidase domain containing 4 | 0.93 | 2.55384 | 17.89435 | 2.34E-05 | 6.41E-04 |
| LOC107049688 | | ----- | uncharacterized LOC107049688 | 0.92 | 2.79743 | 27.47352 | 1.59E-07 | 1.02E-05 |
| LOC101750700 | | ----- | uncharacterized LOC101750700 | 0.92 | 5.03911 | 51.73216 | 6.36E-13 | 1.59E-10 |
| KCNK4 | ----- | ENSGALG00000012021 | potassium channel, subfamily K, member 4 | 0.91 | -0.29429 | 7.63746 | 5.72E-03 | 4.19E-02 |
| LOC101748003 | | ----- | uncharacterized LOC101748003 | 0.91 | 0.39861 | 12.78459 | 3.49E-04 | 5.51E-03 |
| FBXL13 | ----- | ----- | F-box and leucine rich repeat protein 13 | 0.90 | 1.16175 | 14.71798 | 1.25E-04 | 2.45E-03 |
| NTRK1 | CTRKA | ENSGALG00000019019 | neurotrophic receptor tyrosine kinase 1 | 0.90 | 0.33692 | 11.69628 | 6.26E-04 | 8.54E-03 |
| LOC107054526 | | ----- | uncharacterized LOC107054526 | 0.90 | -0.56038 | 9.03437 | 2.65E-03 | 2.42E-02 |
| CPLX1 | ----- | ENSGALG00000015332 | complexin 1 | 0.90 | 4.09625 | 23.05199 | 1.58E-06 | 6.92E-05 |
| LOC101748847 | | ----- | uncharacterized LOC101748847 | 0.90 | -0.06386 | 7.76404 | 5.33E-03 | 3.99E-02 |
| DNAJC28 | ----- | ENSGALG00000037414 | DnaJ heat shock protein family (Hsp40) member C28 | 0.90 | 2.22270 | 34.77439 | 3.70E-09 | 3.65E-07 |
| LOC101751275 | | ----- | uncharacterized LOC101751275 | 0.90 | 0.19475 | 8.76005 | 3.08E-03 | 2.69E-02 |
| LOC112530112 | | ----- | uncharacterized LOC112530112 | 0.90 | 1.01634 | 10.71463 | 1.06E-03 | 1.26E-02 |
| LOC107049643 | | ----- | uncharacterized LOC107049643 | 0.89 | 0.57253 | 11.70873 | 6.22E-04 | 8.51E-03 |
| AASDHPPT | ----- | ENSGALG00000027981 | aminoadipate-semialdehyde dehydrogenase-phosphopantetheinyl transferase | 0.89 | 2.04872 | 31.66932 | 1.83E-08 | 1.52E-06 |
| LOC107055878 | | ----- | acid-sensing ion channel 1-like | 0.89 | 1.37386 | 12.61670 | 3.82E-04 | 5.91E-03 |
| CD79B | ----- | ENSGALG00000000251 | CD79b molecule | 0.89 | -0.18686 | 8.87250 | 2.89E-03 | 2.58E-02 |
| LOC112530977 | | ----- | fibrinogen alpha-1 chain-like | 0.89 | 1.32634 | 15.07318 | 1.03E-04 | 2.12E-03 |
| LOC112532935 | | ----- | uncharacterized LOC112532935 | 0.89 | 1.49744 | 22.53652 | 2.06E-06 | 8.70E-05 |
| LOC107050328 | | ----- | uncharacterized LOC107050328 | 0.88 | 2.30709 | 8.33768 | 3.88E-03 | 3.18E-02 |
| LY9 | ----- | ----- | ----- | 0.88 | 0.55941 | 9.03211 | 2.65E-03 | 2.42E-02 |
| LOC112532241 | | ----- | small nucleolar RNA SNORA33 | 0.88 | 0.20526 | 15.65747 | 7.59E-05 | 1.68E-03 |
| LOC107053429 | | ----- | uncharacterized LOC107053429 | 0.87 | 1.11474 | 22.44910 | 2.16E-06 | 9.01E-05 |
| LOXHD1 | ----- | ENSGALG00000037829 | lipoxygenase homology domains 1 | 0.87 | -0.12773 | 7.47385 | 6.26E-03 | 4.49E-02 |
| EPB42 | ----- | ENSGALG00000021230 | erythrocyte membrane protein band 4.2 | 0.87 | 1.92796 | 7.69826 | 5.53E-03 | 4.09E-02 |
| LOC107055363 | | ----- | uncharacterized LOC107055363 | 0.86 | -0.47308 | 8.00882 | 4.66E-03 | 3.63E-02 |
| LOC107051705 | | ----- | uncharacterized LOC107051705 | 0.85 | 4.46738 | 37.43035 | 9.47E-10 | 1.07E-07 |
| KCTD16 | ----- | ENSGALG00000012322 | potassium channel tetramerization domain containing 16 | 0.85 | 0.78043 | 15.92212 | 6.60E-05 | 1.50E-03 |
| PIH1D3 | DNAAF6 | ENSGALG00000004870 | PIH1 domain containing 3 | 0.85 | 4.65772 | 115.29342 | 6.79E-27 | 9.31E-24 |
| LOC107051973 | | ----- | uncharacterized LOC107051973 | 0.85 | -0.10831 | 8.96095 | 2.76E-03 | 2.49E-02 |
| IFT81 | CDV1 | ENSGALG00000003854 | intraflagellar transport 81 | 0.84 | 4.81819 | 104.60862 | 1.49E-24 | 1.63E-21 |
| KCNE4 | ----- | ENSGALG00000031350 | potassium voltage-gated channel subfamily E regulatory subunit 4 | 0.84 | 1.29570 | 12.82156 | 3.43E-04 | 5.44E-03 |
| ULK4 | ----- | ----- | unc-51 like kinase 4 | 0.84 | 3.65895 | 43.54491 | 4.14E-11 | 6.82E-09 |
| LOC107050901 | | ----- | keratin-associated protein 10-7-like | 0.84 | 3.45449 | 23.59477 | 1.19E-06 | 5.48E-05 |
| LOC107053559 | | ----- | uncharacterized LOC107053559 | 0.83 | 0.29674 | 11.71815 | 6.19E-04 | 8.49E-03 |
| HSP90AA1 | ----- | ENSGALG00000033212 | heat shock protein 90 alpha family class A member 1 | 0.83 | 10.68309 | 19.27042 | 1.13E-05 | 3.61E-04 |
| TDRKH | ----- | ENSGALG00000031812 | tudor and KH domain containing | 0.83 | 3.39539 | 26.74730 | 2.32E-07 | 1.37E-05 |
| BBOX1 | ----- | ENSGALG00000013297 | gamma-butyrobetaine hydroxylase 1 | 0.83 | 4.92430 | 20.58288 | 5.71E-06 | 2.07E-04 |
| IMPG2 | ----- | ENSGALG00000015322 | interphotoreceptor matrix proteoglycan 2 | 0.83 | 1.25076 | 10.81653 | 1.01E-03 | 1.21E-02 |

| | | | | | | | | |
|---|---|---|---|---|---|---|---|---|
| SLC27A6 | - - - - - | ENSGALG00000000184 | solute carrier family 27 (fatty acid transporter), member 6 | 0.82 | 1.09356 | 18.24623 | 1.94E-05 | 5.52E-04 |
| RMI2 | BLAP18 | ENSGALG00000007155 | RecQ mediated genome instability 2 | 0.82 | 4.31898 | 53.04169 | 3.27E-13 | 9.60E-11 |
| RXFP2 | - - - - - | ENSGALG00000029764 | relaxin/insulin-like family peptide receptor 2 | 0.82 | 2.58674 | 12.98660 | 3.14E-04 | 5.11E-03 |
| AOX2 | - - - - - | ENSGALG00000020876 | aldehyde oxidase 2 | 0.82 | 1.91706 | 24.18121 | 8.77E-07 | 4.32E-05 |
| HIST1H4B | - - - - - | - - - - - | histone cluster 1, H4b | 0.82 | 0.27428 | 9.78427 | 1.76E-03 | 1.83E-02 |
| SPHKAP | 4930544G21RIK | ENSGALG00000002974 | SPHK1 interactor, AKAP domain containing | 0.81 | 2.18268 | 14.90971 | 1.13E-04 | 2.25E-03 |
| LOC101751352 | | - - - - - | translation initiation factor IF-2-like | 0.81 | 2.35421 | 23.76012 | 1.09E-06 | 5.09E-05 |
| LOC101750502 | | - - - - - | uncharacterized LOC101750502 | 0.81 | 4.06218 | 21.55026 | 3.45E-06 | 1.35E-04 |
| LOC107054466 | | - - - - - | protocadherin beta-15-like | 0.80 | 0.15285 | 7.37822 | 6.60E-03 | 4.65E-02 |
| LOC107054904 | | - - - - - | uncharacterized LOC107054904 | 0.80 | 0.26599 | 10.42163 | 1.25E-03 | 1.40E-02 |
| LOC112530086 | | - - - - - | fibrinogen-like protein 1-like protein | 0.80 | 2.21999 | 11.75936 | 6.05E-04 | 8.34E-03 |
| LOC112533519 | | - - - - - | uncharacterized LOC112533519 | 0.80 | 0.73163 | 12.33210 | 4.45E-04 | 6.64E-03 |
| GP1BA | - - - - - | - - - - - | ----- | 0.80 | 1.58181 | 14.60382 | 1.33E-04 | 2.57E-03 |
| LOC112530939 | | - - - - - | uncharacterized LOC112530939 | 0.80 | 0.91143 | 9.62481 | 1.92E-03 | 1.96E-02 |
| LYGL | - - - - - | ENSGALG00000043257 | lysozyme g-like | 0.79 | 3.02916 | 13.24720 | 2.73E-04 | 4.59E-03 |
| TKFC | - - - - - | ENSGALG00000037854 | triokinase and FMN cyclase | 0.79 | 4.90513 | 23.80200 | 1.07E-06 | 5.01E-05 |
| DAW1 | - - - - - | ENSGALG00000002992 | dynein assembly factor with WD repeats 1 | 0.79 | 0.54092 | 14.55476 | 1.36E-04 | 2.62E-03 |
| LOC101749472 | | - - - - - | uncharacterized LOC101749472 | 0.78 | -0.04365 | 7.45340 | 6.33E-03 | 4.53E-02 |
| LOC107052084 | | - - - - - | uncharacterized LOC107052084 | 0.78 | 0.14793 | 11.72186 | 6.18E-04 | 8.48E-03 |
| LOC112530017 | | - - - - - | uncharacterized LOC112530017 | 0.77 | 1.52450 | 7.60983 | 5.81E-03 | 4.23E-02 |
| LOC112533567 | | - - - - - | myelin-oligodendrocyte glycoprotein-like | 0.77 | 0.34967 | 8.05651 | 4.53E-03 | 3.56E-02 |
| CCNO | - - - - - | ENSGALG00000028595 | cyclin O | 0.77 | -0.23339 | 8.67088 | 3.23E-03 | 2.79E-02 |
| TMEM178B | - - - - - | ENSGALG00000028444 | transmembrane protein 178B | 0.77 | 1.83884 | 21.99521 | 2.73E-06 | 1.10E-04 |
| OTOP1 | - - - - - | ENSGALG00000030356 | otopetrin 1 | 0.77 | 0.54405 | 7.72480 | 5.45E-03 | 4.05E-02 |
| WNT2B | - - - - - | ENSGALG00000034196 | Wnt family member 2B | 0.76 | 2.19330 | 9.94965 | 1.61E-03 | 1.71E-02 |
| LOC418114 | | - - - - - | pseudouridine-5'-phosphate glycosidase-like | 0.76 | 3.24680 | 38.09977 | 6.72E-10 | 7.79E-08 |
| CYR61 | CCN1 | ENSGALG00000008661 | cysteine rich angiogenic inducer 61 | 0.76 | 4.71668 | 23.34345 | 1.36E-06 | 6.11E-05 |
| LOC107049250 | | - - - - - | inhibin beta C chain-like | 0.76 | 0.37779 | 9.05921 | 2.61E-03 | 2.40E-02 |
| LOC112532772 | | - - - - - | uncharacterized LOC112532772 | 0.76 | 0.12690 | 9.28508 | 2.31E-03 | 2.21E-02 |
| INSC | - - - - - | ENSGALG00000032558 | inscuteable homolog (Drosophila) | 0.76 | 0.23172 | 9.29322 | 2.30E-03 | 2.21E-02 |
| GBP4L | - - - - - | ENSGALG00000026152 | guanylate-binding protein 4-like | 0.75 | 1.96365 | 10.87515 | 9.75E-04 | 1.18E-02 |
| NTHL1 | CHNTHL1 | ENSGALG00000005617 | nth like DNA glycosylase 1 | 0.75 | 4.07337 | 41.82734 | 9.97E-11 | 1.51E-08 |
| LOC107054327 | | - - - - - | uncharacterized LOC107054327 | 0.75 | -0.15975 | 7.49760 | 6.18E-03 | 4.44E-02 |
| LOC107050538 | MHCY11 | - - - - - | major histocompatibility complex Y, class I heavy chain 11 | 0.75 | 0.23562 | 7.27512 | 6.99E-03 | 4.82E-02 |
| FAM161A | - - - - - | ENSGALG00000008810 | family with sequence similarity 161 member A | 0.75 | 2.14563 | 17.10501 | 3.54E-05 | 8.98E-04 |
| LOC101752186 | | - - - - - | uncharacterized LOC101752186 | 0.75 | 0.92036 | 9.24568 | 2.36E-03 | 2.25E-02 |
| LOC112532059 | | - - - - - | uncharacterized LOC112532059 | 0.75 | 2.22683 | 11.19059 | 8.22E-04 | 1.04E-02 |
| LOC107050418 | | - - - - - | uncharacterized LOC107050418 | 0.74 | 0.88404 | 8.65688 | 3.26E-03 | 2.81E-02 |
| FAM132A | - - - - - | ENSGALG00000001857 | family with sequence similarity 132 member A | 0.74 | 2.85385 | 9.16953 | 2.46E-03 | 2.30E-02 |
| ASTL | - - - - - | ENSGALG00000037220 | astacin like metalloendopeptidase | 0.74 | 4.00195 | 11.99072 | 5.35E-04 | 7.62E-03 |
| LOC101751045 | | - - - - - | uncharacterized LOC101751045 | 0.74 | 0.22711 | 7.18602 | 7.35E-03 | 5.00E-02 |
| LMOD2 | C-LMOD | ENSGALG00000008805 | leiomodin 2 | 0.73 | 1.27139 | 7.64537 | 5.69E-03 | 4.18E-02 |
| STC2 | - - - - - | ENSGALG00000002893 | stanniocalcin 2 | 0.73 | 4.92372 | 13.77931 | 2.06E-04 | 3.65E-03 |
| LOC107054649 | | - - - - - | uncharacterized LOC107054649 | 0.73 | 1.57897 | 9.77790 | 1.77E-03 | 1.83E-02 |

| | | | | | | | | |
|---|---|---|---|---|---|---|---|---|
| LOC112533499 | | - - - - - | 2-acylglycerol O-acyltransferase 2-like | 0.73 | 0.83955 | 9.69241 | 1.85E-03 | 1.90E-02 |
| OXNAD1 | - - - - - | ENSGALG00000011239 | oxidoreductase NAD binding domain containing 1 | 0.73 | 4.28702 | 50.10715 | 1.46E-12 | 3.52E-10 |
| NHEJ1 | - - - - - | ENSGALG00000011335 | non-homologous end joining factor 1 | 0.72 | 2.19453 | 20.72699 | 5.30E-06 | 1.95E-04 |
| HLX | - - - - - | ENSGALG00000037257 | H2.0 like homeobox | 0.72 | 3.18949 | 12.81210 | 3.44E-04 | 5.46E-03 |
| CFAP70 | - - - - - | ENSGALG00000002551 | cilia and flagella associated protein 70 | 0.72 | 2.10334 | 20.27949 | 6.69E-06 | 2.34E-04 |
| MFSD2B | - - - - - | ENSGALG00000016498 | major facilitator superfamily domain containing 2B | 0.72 | 1.95886 | 8.31922 | 3.92E-03 | 3.21E-02 |
| SAMD15 | - - - - - | - - - - - | sterile alpha motif domain containing 15 | 0.72 | 0.70312 | 9.22364 | 2.39E-03 | 2.26E-02 |
| GTSF1 | - - - - - | ENSGALG00000001109 | gametocyte specific factor 1 | 0.72 | 3.88663 | 13.34087 | 2.60E-04 | 4.43E-03 |
| LOC107052732 | | - - - - - | uncharacterized LOC107052732 | 0.71 | 0.46855 | 9.18362 | 2.44E-03 | 2.29E-02 |
| LOC771917 | | - - - - - | uncharacterized LOC771917 | 0.71 | 0.64828 | 10.53516 | 1.17E-03 | 1.34E-02 |
| LOC107052318 | | - - - - - | uncharacterized LOC107052318 | 0.71 | 1.52420 | 8.00297 | 4.67E-03 | 3.64E-02 |
| LOC107055288 | | - - - - - | uncharacterized LOC107055288 | 0.71 | 3.45704 | 23.30045 | 1.39E-06 | 6.21E-05 |
| SLC17A9 | C20orf59 | ENSGALG00000028225 | solute carrier family 17 member 9 | 0.71 | 2.57672 | 23.92389 | 1.00E-06 | 4.79E-05 |
| NOXA1 | - - - - - | ENSGALG00000008952 | NADPH oxidase activator 1 | 0.70 | 1.97992 | 12.20246 | 4.77E-04 | 6.99E-03 |
| VWA5B1 | - - - - - | - - - - - | von Willebrand factor A domain containing 5B1 | 0.70 | 4.43046 | 38.65580 | 5.06E-10 | 6.07E-08 |
| LOC100857609 | | - - - - - | phosphatidylinositol-4,5-bisphosphate phosphodiesterase gamma-1-like | 0.70 | 2.75183 | 18.13278 | 2.06E-05 | 5.78E-04 |
| LOC101747364 | | - - - - - | uncharacterized LOC101747364 | 0.70 | 1.84240 | 14.02125 | 1.81E-04 | 3.29E-03 |
| SLC6A4 | SERT | ENSGALG00000004246 | solute carrier family 6 member 4 | 0.70 | 3.76234 | 13.26270 | 2.71E-04 | 4.58E-03 |
| IL18 | ChIL-18 | ENSGALG00000007874 | interleukin 18 | 0.70 | 3.63024 | 44.26565 | 2.87E-11 | 4.97E-09 |
| MAK | - - - - - | - - - - - | male germ cell associated kinase | 0.70 | 2.03241 | 16.91418 | 3.91E-05 | 9.77E-04 |
| ADORA2B | A2B-AR | ENSGALG00000014182 | adenosine A2b receptor | 0.69 | 1.62156 | 10.14597 | 1.45E-03 | 1.57E-02 |
| LOC101748818 | | - - - - - | uncharacterized LOC101748818 | 0.69 | 0.95049 | 10.41983 | 1.25E-03 | 1.40E-02 |
| SCO2 | - - - - - | ENSGALG00000046885 | SCO2, cytochrome c oxidase assembly protein | 0.69 | 3.59986 | 31.09835 | 2.45E-08 | 1.94E-06 |
| SULT1E1 | - - - - - | ENSGALG00000011812 | sulfotransferase family 1E member 1 | 0.69 | 1.50363 | 12.06869 | 5.13E-04 | 7.39E-03 |
| DNAJC24 | DPH4 | ENSGALG00000012130 | DnaJ heat shock protein family (Hsp40) member C24 | 0.69 | 3.10272 | 29.10887 | 6.84E-08 | 4.90E-06 |
| LOC107052946 | | - - - - - | uncharacterized LOC107052946 | 0.68 | 0.63573 | 10.65933 | 1.10E-03 | 1.28E-02 |
| DNAJA1 | - - - - - | ENSGALG00000023066 | DnaJ heat shock protein family (Hsp40) member A1 | 0.68 | 7.38649 | 21.42789 | 3.67E-06 | 1.42E-04 |
| MANBAL7 | - - - - - | - - - - - | ----- | 0.68 | 5.18503 | 11.29648 | 7.77E-04 | 9.98E-03 |
| SLC39A8 | - - - - - | ENSGALG00000012298 | solute carrier family 39 member 8 | 0.68 | 3.97478 | 13.63476 | 2.22E-04 | 3.90E-03 |
| LOC107057116 | ZNFY4 | - - - - - | MHCY region zinc finger protein 4 | 0.68 | 3.04191 | 11.42903 | 7.23E-04 | 9.50E-03 |
| LOC418108 | | - - - - - | poly | 0.67 | 1.90963 | 10.67551 | 1.09E-03 | 1.27E-02 |
| DNAJA4 | - - - - - | ENSGALG00000036850 | DnaJ heat shock protein family (Hsp40) member A4 | 0.67 | 6.16564 | 29.47627 | 5.66E-08 | 4.14E-06 |
| TMEM109 | - - - - - | - - - - - | transmembrane protein 109 | 0.67 | 2.67533 | 16.42898 | 5.05E-05 | 1.20E-03 |
| LOC112531022 | | - - - - - | uncharacterized LOC112531022 | 0.67 | 0.21959 | 8.80203 | 3.01E-03 | 2.65E-02 |
| VWC2L | - - - - - | ENSGALG00000003472 | von Willebrand factor C domain containing protein 2 like | 0.67 | 3.56223 | 25.86107 | 3.67E-07 | 2.09E-05 |
| LYPD6 | - - - - - | ENSGALG00000012470 | LY6/PLAUR domain containing 6 | 0.67 | 1.32156 | 8.87688 | 2.89E-03 | 2.58E-02 |
| CALCB | - - - - - | - - - - - | ----- | 0.67 | 4.57919 | 24.49675 | 7.44E-07 | 3.75E-05 |
| ENKUR | - - - - - | ENSGALG00000007680 | enkurin, TRPC channel interacting protein | 0.67 | 1.93364 | 10.15902 | 1.44E-03 | 1.57E-02 |
| TMEM59L | - - - - - | ENSGALG00000021620 | transmembrane protein 59 like | 0.67 | 1.98147 | 12.78525 | 3.49E-04 | 5.51E-03 |
| LOC100857706 | | - - - - - | uncharacterized LOC100857706 | 0.67 | 2.61569 | 13.39398 | 2.52E-04 | 4.33E-03 |
| SLC18A2 | - - - - - | ENSGALG00000009289 | solute carrier family 18 member A2 | 0.67 | 1.51114 | 10.47368 | 1.21E-03 | 1.38E-02 |
| KIFC1L | - - - - - | - - - - - | ----- | 0.67 | 7.45576 | 43.02052 | 5.42E-11 | 8.65E-09 |
| CFAP58 | - - - - - | ENSGALG00000008417 | cilia and flagella associated protein 58 | 0.67 | 0.93828 | 11.75720 | 6.06E-04 | 8.34E-03 |
| STN1 | - - - - - | ENSGALG00000008304 | STN1, CST complex subunit | 0.66 | 5.29576 | 52.66869 | 3.95E-13 | 1.08E-10 |

| | | | | | | | | |
|---|---|---|---|---|---|---|---|---|
| ST6GALNAC2L | - - - - - | - - - - - | ST6 N-acetylgalactosaminide alpha-2,6-sialyltransferase 2 like | 0.66 | 2.38842 | 14.82561 | 1.18E-04 | 2.34E-03 |
| VWA3A | - - - - - | ENSGALG00000049654 | von Willebrand factor A domain containing 3A | 0.65 | 1.09276 | 12.83956 | 3.39E-04 | 5.41E-03 |
| LOC112529982 | | - - - - - | syntaxin-4-like | 0.65 | 2.71443 | 17.91028 | 2.32E-05 | 6.37E-04 |
| RAB19 | - - - - - | ENSGALG00000012850 | RAB19, member RAS oncogene family | 0.65 | 1.27640 | 11.17244 | 8.30E-04 | 1.05E-02 |
| CRYGN | - - - - - | ENSGALG00000006189 | crystallin gamma N | 0.65 | 1.12924 | 10.27383 | 1.35E-03 | 1.49E-02 |
| GIMAP5L | - - - - - | - - - - - | ----- | 0.65 | 2.57118 | 9.82575 | 1.72E-03 | 1.79E-02 |
| NUP210L | - - - - - | - - - - - | nucleoporin 210 like | 0.65 | 2.44187 | 15.27203 | 9.31E-05 | 1.96E-03 |
| LAMC3 | - - - - - | - - - - - | laminin subunit gamma 3 | 0.65 | 4.04705 | 19.87715 | 8.26E-06 | 2.82E-04 |
| LOC100857928 | | - - - - - | uncharacterized LOC100857928 | 0.65 | 1.51683 | 11.92288 | 5.54E-04 | 7.86E-03 |
| LDHA | - - - - - | ENSGALG00000006300 | lactate dehydrogenase A | 0.64 | 6.34999 | 9.52652 | 2.03E-03 | 2.03E-02 |
| ALG14 | - - - - - | ENSGALG00000005583 | ALG14, UDP-N-acetylglucosaminyltransferase subunit | 0.64 | 2.89310 | 25.75054 | 3.89E-07 | 2.18E-05 |
| LOC107050412 | | - - - - - | uncharacterized LOC107050412 | 0.64 | 1.62295 | 13.72154 | 2.12E-04 | 3.75E-03 |
| MPL | c-mpl | ENSGALG00000009965 | MPL proto-oncogene, thrombopoietin receptor | 0.64 | 2.76289 | 13.81857 | 2.01E-04 | 3.60E-03 |
| LYPD1 | - - - - - | ENSGALG00000050403 | LY6/PLAUR domain containing 1 | 0.64 | 0.96712 | 7.25767 | 7.06E-03 | 4.85E-02 |
| POLG2 | - - - - - | ENSGALG00000003520 | DNA polymerase gamma 2, accessory subunit | 0.63 | 3.48036 | 24.57094 | 7.16E-07 | 3.65E-05 |
| CCDC27 | - - - - - | ENSGALG00000043926 | coiled-coil domain containing 27 | 0.63 | 2.30130 | 16.30740 | 5.39E-05 | 1.27E-03 |
| SLC13A3 | - - - - - | ENSGALG00000054473 | solute carrier family 13 member 3 | 0.63 | 3.21238 | 26.06317 | 3.30E-07 | 1.90E-05 |
| TLR4 | - - - - - | ENSGALG00000007001 | toll like receptor 4 | 0.63 | 1.28219 | 7.28828 | 6.94E-03 | 4.80E-02 |
| FOXF1 | - - - - - | ENSGALG00000034111 | forkhead box F1 | 0.63 | 4.40096 | 18.56196 | 1.64E-05 | 4.84E-04 |
| CASP7 | - - - - - | ENSGALG00000008933 | caspase 7 | 0.63 | 1.86049 | 12.53488 | 3.99E-04 | 6.13E-03 |
| NR2C1 | - - - - - | ENSGALG00000011327 | nuclear receptor subfamily 2, group C, member 1 | 0.63 | 4.90645 | 36.29898 | 1.69E-09 | 1.80E-07 |
| FAIM | - - - - - | ENSGALG00000012082 | Fas apoptotic inhibitory molecule | 0.63 | 4.63998 | 34.91467 | 3.44E-09 | 3.44E-07 |
| VSNL1 | - - - - - | ENSGALG00000016464 | visinin like 1 | 0.63 | 2.28756 | 10.46173 | 1.22E-03 | 1.38E-02 |
| LOC112530784 | | - - - - - | small nucleolar RNA SNORD58 | 0.62 | 0.79690 | 7.49955 | 6.17E-03 | 4.44E-02 |
| LOC107052236 | | - - - - - | uncharacterized LOC107052236 | 0.62 | 1.28724 | 10.06027 | 1.52E-03 | 1.63E-02 |
| MATK | - - - - - | ENSGALG00000000736 | megakaryocyte-associated tyrosine kinase | 0.62 | 1.56424 | 10.95737 | 9.32E-04 | 1.14E-02 |
| BMP3 | - - - - - | ENSGALG00000035052 | bone morphogenetic protein 3 | 0.61 | 2.29164 | 7.21704 | 7.22E-03 | 4.93E-02 |
| LOC101750197 | | - - - - - | uncharacterized LOC101750197 | 0.61 | 0.59503 | 8.11375 | 4.39E-03 | 3.48E-02 |
| LOC112532382 | | - - - - - | uncharacterized LOC112532382 | 0.61 | 1.05164 | 9.00756 | 2.69E-03 | 2.44E-02 |
| LOC112532614 | | - - - - - | uncharacterized LOC112532614 | 0.61 | 4.13200 | 15.04094 | 1.05E-04 | 2.15E-03 |
| FIBCD1 | - - - - - | ENSGALG00000003866 | ficolin 3 | 0.60 | 1.46701 | 8.22506 | 4.13E-03 | 3.33E-02 |
| COL2A1L | - - - - - | - - - - - | ----- | 0.60 | 5.53957 | 12.45309 | 4.17E-04 | 6.31E-03 |
| HTR2B | - - - - - | ENSGALG00000007681 | 5-hydroxytryptamine receptor 2B | 0.60 | 1.07254 | 8.48864 | 3.57E-03 | 3.00E-02 |
| EGLN3 | - - - - - | ENSGALG00000010001 | egl-9 family hypoxia inducible factor 3 | 0.60 | 2.97052 | 12.12140 | 4.98E-04 | 7.24E-03 |
| LOC107056270 | | - - - - - | BCL2/adenovirus E1B 19 kDa protein-interacting protein 3-like | 0.60 | 3.11793 | 10.35408 | 1.29E-03 | 1.44E-02 |
| LOC107054458 | | - - - - - | PHD finger protein 7-like | 0.60 | 2.03444 | 13.91955 | 1.91E-04 | 3.44E-03 |
| MDH1B | - - - - - | ENSGALG00000008548 | malate dehydrogenase 1B | 0.60 | 1.85184 | 13.02038 | 3.08E-04 | 5.04E-03 |
| CYP26B1 | - - - - - | ENSGALG00000016102 | cytochrome P450 family 26 subfamily B member 1 | 0.60 | 4.99141 | 26.98388 | 2.05E-07 | 1.25E-05 |
| HSPA8 | HSC70 | ENSGALG00000006512 | heat shock 70kDa protein 8 | 0.59 | 10.88731 | 18.49837 | 1.70E-05 | 4.93E-04 |
| FSIP1 | - - - - - | ENSGALG00000009624 | fibrous sheath interacting protein 1 | 0.59 | 1.82442 | 12.33437 | 4.45E-04 | 6.64E-03 |
| LOC112532778 | | - - - - - | uncharacterized LOC112532778 | 0.59 | 1.00040 | 9.19169 | 2.43E-03 | 2.28E-02 |
| PLPP4 | - - - - - | ENSGALG00000029020 | phospholipid phosphatase 4 | 0.59 | 5.04021 | 27.09565 | 1.94E-07 | 1.19E-05 |
| PBLD | - - - - - | ENSGALG00000003935 | phenazine biosynthesis like protein domain containing | 0.59 | 3.27521 | 27.57730 | 1.51E-07 | 9.74E-06 |
| ZFPM2 | - - - - - | ENSGALG00000040079 | zinc finger protein, FOG family member 2 | 0.59 | 3.07736 | 8.85605 | 2.92E-03 | 2.59E-02 |

| | | | | | | | | |
|---|---|---|---|---|---|---|---|---|
| PEX6 | ----- | ENSGALG00000045514 | peroxisomal biogenesis factor 6 | 0.58 | 5.75744 | 40.03471 | 2.49E-10 | 3.34E-08 |
| VPS41 | ----- | ENSGALG00000031635 | VPS41, HOPS complex subunit | 0.58 | 5.63370 | 52.79102 | 3.71E-13 | 1.03E-10 |
| LOC107050006 | | ----- | uncharacterized LOC107050006 | 0.58 | 2.02232 | 12.14186 | 4.93E-04 | 7.20E-03 |
| LOC421506 | | ----- | uncharacterized LOC421506 | 0.58 | 2.97125 | 11.04376 | 8.90E-04 | 1.10E-02 |
| SHC4 | ----- | ----- | SHC adaptor protein 4 | 0.58 | 3.53231 | 27.34973 | 1.70E-07 | 1.07E-05 |
| PYCR1 | PYCR2 | ENSGALG00000007300 | pyrroline-5-carboxylate reductase 1 | 0.57 | 6.86296 | 45.37152 | 1.63E-11 | 3.01E-09 |
| RRP8 | ----- | ----- | ribosomal RNA processing 8, methyltransferase, homolog (yeast) | 0.57 | 4.54959 | 27.95926 | 1.24E-07 | 8.19E-06 |
| PMFBP1 | ----- | ----- | polyamine modulated factor 1 binding protein 1 | 0.57 | 3.08059 | 19.25710 | 1.14E-05 | 3.63E-04 |
| RBFA | C18orf22 | ENSGALG00000036067 | ribosome binding factor A (putative) | 0.57 | 4.60691 | 48.83635 | 2.78E-12 | 6.27E-10 |
| SERPINH1 | HSP47 | ENSGALG00000011214 | serpin family H member 1 | 0.57 | 8.19946 | 10.06362 | 1.51E-03 | 1.63E-02 |
| LOC101749936 | | ----- | uncharacterized LOC101749936 | 0.57 | 1.36136 | 8.99986 | 2.70E-03 | 2.45E-02 |
| RCAN2 | ----- | ENSGALG00000016709 | regulator of calcineurin 2 | 0.57 | 6.33603 | 36.37279 | 1.63E-09 | 1.75E-07 |
| CARD8 | ----- | ENSGALG00000007698 | caspase recruitment domain family, member 8 | 0.57 | 1.01472 | 10.13266 | 1.46E-03 | 1.58E-02 |
| LOC107054051 | | ----- | uncharacterized LOC107054051 | 0.56 | 2.24173 | 12.86856 | 3.34E-04 | 5.33E-03 |
| RNF207 | ----- | ENSGALG00000050086 | ring finger protein 207 | 0.56 | 4.71911 | 27.59626 | 1.49E-07 | 9.68E-06 |
| TRIM63 | ----- | ENSGALG00000034107 | tripartite motif containing 63 | 0.56 | 3.52746 | 10.83191 | 9.98E-04 | 1.20E-02 |
| LOC770653 | | ----- | uncharacterized LOC770653 | 0.56 | 3.41825 | 10.78700 | 1.02E-03 | 1.22E-02 |
| UCKL1 | ZFP | ENSGALG00000006040 | uridine-cytidine kinase 1 like 1 | 0.56 | 3.21778 | 12.12063 | 4.99E-04 | 7.24E-03 |
| SPTBN4L | ----- | ----- | ----- | 0.55 | 3.07086 | 8.55723 | 3.44E-03 | 2.91E-02 |
| RSPH14 | ----- | ENSGALG00000006712 | radial spoke head 14 homolog | 0.55 | 2.79561 | 12.22336 | 4.72E-04 | 6.93E-03 |
| KLHL6 | ----- | ENSGALG00000002263 | kelch like family member 6 | 0.55 | 1.69701 | 8.78373 | 3.04E-03 | 2.66E-02 |
| OTOG | ----- | ----- | otogelin | 0.55 | 3.19016 | 7.90105 | 4.94E-03 | 3.80E-02 |
| IKBKAP | ----- | ENSGALG00000001947 | inhibitor of kappa light polypeptide gene enhancer in B-cells | 0.55 | 7.01423 | 28.80138 | 8.02E-08 | 5.57E-06 |
| MYH7 | AMHC1 | ENSGALG00000035594 | myosin, heavy chain 7, cardiac muscle, beta | 0.55 | 2.72866 | 7.49301 | 6.19E-03 | 4.45E-02 |
| UGT8 | ----- | ENSGALG00000012018 | UDP glycosyltransferase 8 | 0.55 | 2.16468 | 7.63432 | 5.73E-03 | 4.19E-02 |
| MILR1 | ----- | ENSGALG00000046212 | mast cell immunoglobulin like receptor 1 | 0.55 | 1.36625 | 7.94021 | 4.83E-03 | 3.74E-02 |
| LOC107052132 | | ----- | uncharacterized LOC107052132 | 0.54 | 2.07725 | 10.56459 | 1.15E-03 | 1.33E-02 |
| CBLN1 | ----- | ENSGALG00000042664 | cerebellin 1 precursor | 0.54 | 3.47299 | 7.28504 | 6.95E-03 | 4.80E-02 |
| NOM1 | ----- | ENSGALG00000006437 | nucleolar protein with MIF4G domain 1 | 0.54 | 4.68894 | 40.50798 | 1.96E-10 | 2.77E-08 |
| SIM1 | ----- | ENSGALG00000015466 | SIM bHLH transcription factor 1 | 0.54 | 4.92844 | 24.05842 | 9.35E-07 | 4.55E-05 |
| DAO | ----- | ENSGALG00000043053 | D-amino acid oxidase | 0.54 | 3.35977 | 23.41805 | 1.30E-06 | 5.93E-05 |
| ASCC1 | ----- | ENSGALG00000026019 | activating signal cointegrator 1 complex subunit 1 | 0.54 | 5.38196 | 27.25840 | 1.78E-07 | 1.11E-05 |
| LOC112533544 | | ----- | E3 ubiquitin-protein ligase TRIM7-like | 0.53 | 5.22062 | 48.46844 | 3.36E-12 | 7.46E-10 |
| MISP | ----- | ENSGALG00000044985 | mitotic spindle positioning | 0.53 | 2.47867 | 13.13857 | 2.89E-04 | 4.80E-03 |
| VRTN | ----- | ENSGALG00000010232 | vertebrae development associated | 0.53 | 2.96244 | 18.12469 | 2.07E-05 | 5.78E-04 |
| SLC3A1 | ----- | ENSGALG00000009973 | solute carrier family 3 member 1 | 0.53 | 1.38265 | 7.82584 | 5.15E-03 | 3.89E-02 |
| ACOX3 | ----- | ENSGALG00000015591 | acyl-CoA oxidase 3, pristanoyl | 0.53 | 3.39689 | 14.90141 | 1.13E-04 | 2.26E-03 |
| DNAH12 | ----- | ENSGALG00000005601 | dynein, axonemal, heavy chain 12 | 0.53 | 1.93993 | 9.08794 | 2.57E-03 | 2.37E-02 |
| PROCA1 | ----- | ENSGALG00000030512 | protein interacting with cyclin A1 | 0.53 | 4.17809 | 31.39498 | 2.11E-08 | 1.72E-06 |
| LOC107056831 | | ----- | uncharacterized LOC107056831 | 0.53 | 1.30912 | 7.31890 | 6.82E-03 | 4.74E-02 |
| C26H1orf74 | ----- | ----- | ----- | 0.53 | 2.50791 | 10.71670 | 1.06E-03 | 1.26E-02 |
| GAA | ----- | ENSGALG00000038853 | alpha glucosidase | 0.53 | 4.10108 | 12.99324 | 3.13E-04 | 5.10E-03 |
| LOC112533501 | | ----- | protein NDRG2-like | 0.52 | 1.24904 | 7.74832 | 5.38E-03 | 4.02E-02 |
| LOC426385 | | ENSGALG00000026301 | serine/threonine kinase 35-like | 0.52 | 4.78196 | 34.63907 | 3.97E-09 | 3.89E-07 |

| | | | | | | | | |
|---|---|---|---|---|---|---|---|---|
| HOXB1 | Ghox-lab | ENSGALG00000013454 | homeobox B1 | 0.52 | 6.67587 | 28.50349 | 9.35E-08 | 6.36E-06 |
| AAREL | - - - - - | ENSGALG00000033303 | acylamino-acid-releasing enzyme-like | 0.52 | 2.65371 | 8.01666 | 4.63E-03 | 3.62E-02 |
| TRPV1 | - - - - - | ENSGALG00000004649 | transient receptor potential cation channel subfamily V member 1 | 0.52 | 2.74528 | 7.51332 | 6.12E-03 | 4.41E-02 |
| ST8SIA1 | SIAL-T2 | ENSGALG00000013211 | ST8 alpha-N-acetyl-neuraminide alpha-2,8-sialyltransferase 1 | 0.52 | 3.86006 | 16.72555 | 4.32E-05 | 1.06E-03 |
| COLEC11 | - - - - - | ENSGALG00000050002 | collectin subfamily member 11 | 0.52 | 3.58007 | 12.68368 | 3.69E-04 | 5.75E-03 |
| LOC100858737 | | - - - - - | uncharacterized LOC100858737 | 0.52 | 1.88723 | 10.52930 | 1.17E-03 | 1.35E-02 |
| SEMA3BL | - - - - - | - - - - - | ----- | 0.52 | 4.90530 | 13.88518 | 1.94E-04 | 3.49E-03 |
| LIN28A | LIN28 | ENSGALG00000036022 | lin-28 homolog A | 0.52 | 6.70121 | 8.26578 | 4.04E-03 | 3.27E-02 |
| GSTK1 | - - - - - | ENSGALG00000014708 | glutathione S-transferase kappa 1 | 0.52 | 5.31156 | 53.21372 | 2.99E-13 | 9.12E-11 |
| IKBKE | - - - - - | ENSGALG00000013356 | inhibitor of kappa light polypeptide gene enhancer in B-cells | 0.52 | 2.45355 | 9.94483 | 1.61E-03 | 1.71E-02 |
| TRIM45 | - - - - - | ENSGALG00000014818 | tripartite motif containing 45 | 0.51 | 3.52653 | 19.71437 | 8.99E-06 | 2.99E-04 |
| BCO2 | - - - - - | ENSGALG00000007868 | beta-carotene oxygenase 2 | 0.51 | 2.47314 | 11.03559 | 8.94E-04 | 1.10E-02 |
| LOC107053557 | | - - - - - | uncharacterized LOC107053557 | 0.51 | 2.59617 | 10.56924 | 1.15E-03 | 1.33E-02 |
| LSM10 | - - - - - | ENSGALG00000002127 | LSM10, U7 small nuclear RNA associated | 0.51 | 2.09851 | 10.27309 | 1.35E-03 | 1.49E-02 |
| LOC112532559 | | - - - - - | translation initiation factor IF-2-like | 0.51 | 3.19000 | 21.61043 | 3.34E-06 | 1.31E-04 |
| HSPA5 | GRP78 | ENSGALG00000001000 | heat shock 70kDa protein 5 (glucose-regulated protein, 78kDa) | 0.51 | 9.20858 | 25.37409 | 4.72E-07 | 2.56E-05 |
| LOC101750998 | | - - - - - | uncharacterized LOC101750998 | 0.51 | 6.41239 | 9.45598 | 2.10E-03 | 2.09E-02 |
| GCNT7 | - - - - - | ENSGALG00000007717 | glucosaminyl (N-acetyl) transferase family member 7 | 0.50 | 2.26302 | 9.44583 | 2.12E-03 | 2.09E-02 |
| LOC101749541 | | - - - - - | translation initiation factor IF-2-like | 0.50 | 2.16796 | 7.22148 | 7.20E-03 | 4.92E-02 |
| RSPH10B | - - - - - | - - - - - | radial spoke head 10 homolog B | 0.50 | 3.66384 | 17.03584 | 3.67E-05 | 9.30E-04 |
| 2KTGRL | - - - - - | ENSGALG00000046360 | probable 2-ketogluconate reductase-like | 0.50 | 3.39609 | 16.99252 | 3.75E-05 | 9.44E-04 |
| HLCS | - - - - - | ENSGALG00000016047 | holocarboxylase synthetase | 0.50 | 6.00153 | 43.10292 | 5.19E-11 | 8.38E-09 |
| CYP46A1 | - - - - - | ENSGALG00000011162 | cytochrome P450 family 46 subfamily A member 1 | 0.50 | 4.24970 | 23.11713 | 1.52E-06 | 6.74E-05 |
| FRMD5 | - - - - - | ENSGALG00000030858 | FERM domain containing 5 | 0.50 | 4.16972 | 15.79369 | 7.06E-05 | 1.59E-03 |
| SNAP47 | - - - - - | ENSGALG00000005412 | synaptosome associated protein 47 | 0.50 | 4.65215 | 30.27263 | 3.75E-08 | 2.86E-06 |
| SPTBN2 | - - - - - | - - - - - | spectrin beta, non-erythrocytic 2 | 0.50 | 5.64761 | 17.87283 | 2.36E-05 | 6.48E-04 |
| FUT10 | - - - - - | ENSGALG00000015399 | fucosyltransferase 10 | 0.49 | 2.72276 | 9.27511 | 2.32E-03 | 2.22E-02 |
| ADCY5 | ADCY6 | ENSGALG00000031244 | adenylate cyclase 6 | 0.49 | 4.99531 | 27.03729 | 2.00E-07 | 1.22E-05 |
| NABP1 | - - - - - | ENSGALG00000025884 | nucleic acid binding protein 1 | 0.49 | 6.64157 | 45.11202 | 1.86E-11 | 3.40E-09 |
| C20H20orf96 | - - - - - | - - - - - | ----- | 0.49 | 1.88788 | 7.89605 | 4.95E-03 | 3.80E-02 |
| MUT | - - - - - | ENSGALG00000016697 | methylmalonyl-CoA mutase | 0.49 | 4.87546 | 20.99622 | 4.60E-06 | 1.72E-04 |
| LOC768735 | CTC-575C13.4 | - - - - - | uncharacterized protein LOC768735 | 0.49 | 2.70950 | 9.12314 | 2.52E-03 | 2.34E-02 |
| SIK1 | SIK2 | ENSGALG00000041988 | salt inducible kinase 1 | 0.49 | 6.17899 | 27.44546 | 1.62E-07 | 1.03E-05 |
| LOC101747556 | | - - - - - | uncharacterized LOC101747556 | 0.49 | 4.53877 | 19.41025 | 1.05E-05 | 3.40E-04 |
| LOC101747742 | | - - - - - | uncharacterized LOC101747742 | 0.48 | 2.90483 | 17.56204 | 2.78E-05 | 7.34E-04 |
| SMC2 | SCII | ENSGALG00000015691 | structural maintenance of chromosomes 2 | 0.48 | 8.87683 | 28.52460 | 9.25E-08 | 6.32E-06 |
| N4BP2L1 | - - - - - | ENSGALG00000017072 | NEDD4 binding protein 2 like 1 | 0.48 | 3.98943 | 12.42733 | 4.23E-04 | 6.38E-03 |
| LOC424430 | | - - - - - | quinone oxidoreductase-like 2 | 0.48 | 2.65388 | 12.63342 | 3.79E-04 | 5.88E-03 |
| PDE1C | - - - - - | ENSGALG00000039935 | phosphodiesterase 1C | 0.48 | 4.06600 | 19.89312 | 8.19E-06 | 2.80E-04 |
| LOC101747934 | | - - - - - | uncharacterized LOC101747934 | 0.48 | 3.28191 | 14.60282 | 1.33E-04 | 2.57E-03 |
| MAOB | - - - - - | ENSGALG00000032836 | monoamine oxidase B | 0.48 | 3.43523 | 8.94008 | 2.79E-03 | 2.51E-02 |
| PLCD3 | - - - - - | ENSGALG00000045212 | phospholipase C delta 3 | 0.48 | 3.17572 | 13.62375 | 2.23E-04 | 3.91E-03 |
| ATP2A3 | SERCA | ENSGALG00000001564 | ATPase sarcoplasmic/endoplasmic reticulum Ca2+ transporting 3 | 0.47 | 4.71583 | 14.63372 | 1.31E-04 | 2.55E-03 |
| GRHPR | - - - - - | ENSGALG00000005423 | glyoxylate and hydroxypyruvate reductase | 0.47 | 3.38355 | 14.02218 | 1.81E-04 | 3.29E-03 |

| | | | | | | | | |
|---|---|---|---|---|---|---|---|---|
| SOCS2 | - - - - - | ENSGALG00000050619 | suppressor of cytokine signaling 2 | 0.47 | 4.14594 | 10.65962 | 1.09E-03 | 1.28E-02 |
| LOC101747310 | | - - - - - | uncharacterized LOC101747310 | 0.47 | 1.31047 | 7.39135 | 6.55E-03 | 4.62E-02 |
| KLF4 | - - - - - | - - - - - | Kruppel like factor 4 | 0.47 | 2.34432 | 9.94298 | 1.61E-03 | 1.71E-02 |
| CTNNAL1 | - - - - - | ENSGALG00000013207 | catenin alpha like 1 | 0.47 | 6.17645 | 47.33226 | 5.99E-12 | 1.28E-09 |
| TRAPPC12 | - - - - - | ENSGALG00000016387 | trafficking protein particle complex 12 | 0.47 | 4.88611 | 18.38055 | 1.81E-05 | 5.19E-04 |
| CERS4 | - - - - - | ENSGALG00000043352 | ceramide synthase 4 | 0.46 | 5.51261 | 29.00829 | 7.21E-08 | 5.10E-06 |
| LOC107051305 | | - - - - - | sodium channel subunit beta-1-like | 0.46 | 3.59859 | 11.83074 | 5.83E-04 | 8.11E-03 |
| EDEM2 | - - - - - | ENSGALG00000003128 | ER degradation enhancing alpha-mannosidase like protein 2 | 0.46 | 5.27678 | 43.29733 | 4.70E-11 | 7.66E-09 |
| LOC112533506 | | - - - - - | uncharacterized LOC112533506 | 0.46 | 5.04495 | 25.49525 | 4.43E-07 | 2.43E-05 |
| LOC112533366 | | - - - - - | uncharacterized LOC112533366 | 0.46 | 2.86910 | 15.16406 | 9.86E-05 | 2.04E-03 |
| TTC32 | - - - - - | ENSGALG00000016476 | tetratricopeptide repeat domain 32 | 0.46 | 3.01363 | 8.54752 | 3.46E-03 | 2.92E-02 |
| LRRC75A | - - - - - | ENSGALG00000002631 | leucine rich repeat containing 75A | 0.45 | 3.85241 | 12.38095 | 4.34E-04 | 6.51E-03 |
| STRADB | - - - - - | ENSGALG00000008373 | STE20-related kinase adaptor beta | 0.45 | 5.51091 | 28.88958 | 7.66E-08 | 5.39E-06 |
| GLB1L2 | - - - - - | ENSGALG00000001604 | galactosidase beta 1 like 2 | 0.45 | 5.75462 | 46.32640 | 1.00E-11 | 1.94E-09 |
| DTD2 | - - - - - | ENSGALG00000009978 | D-tyrosyl-tRNA deacylase 2 (putative) | 0.45 | 3.06930 | 12.50638 | 4.06E-04 | 6.19E-03 |
| ASL2 | ASL | ENSGALG00000002576 | argininosuccinate lyase 2 | 0.45 | 2.03955 | 10.62732 | 1.11E-03 | 1.30E-02 |
| BTD | - - - - - | ENSGALG00000011216 | biotinidase | 0.45 | 3.02549 | 8.79793 | 3.02E-03 | 2.65E-02 |
| PARP14 | - - - - - | ENSGALG00000026422 | poly(ADP-ribose) polymerase family member 14 | 0.45 | 2.61485 | 9.16801 | 2.46E-03 | 2.30E-02 |
| VDAC1 | - - - - - | ENSGALG00000006503 | voltage dependent anion channel 1 | 0.45 | 3.78611 | 10.81799 | 1.01E-03 | 1.21E-02 |
| SLC16A3 | MCT4 | ENSGALG00000002728 | solute carrier family 16 member 3 | 0.45 | 5.24705 | 10.65672 | 1.10E-03 | 1.28E-02 |
| PTGR2 | ZADH1 | ENSGALG00000009269 | prostaglandin reductase 2 | 0.45 | 5.36727 | 24.48681 | 7.48E-07 | 3.75E-05 |
| ACYP2 | acylphosphatase-2 | ENSGALG00000027632 | acylphosphatase 2 | 0.44 | 3.46013 | 14.23317 | 1.61E-04 | 3.00E-03 |
| GDF10 | - - - - - | ENSGALG00000005985 | growth differentiation factor 10 | 0.44 | 4.45645 | 8.30699 | 3.95E-03 | 3.22E-02 |
| PAX2 | PAX-2 | ENSGALG00000005689 | paired box 2 | 0.44 | 5.66638 | 11.17293 | 8.30E-04 | 1.05E-02 |
| LOC107053581 | | - - - - - | uncharacterized LOC107053581 | 0.44 | 2.82782 | 11.40726 | 7.32E-04 | 9.55E-03 |
| METTL25 | - - - - - | ENSGALG00000042269 | methyltransferase like 25 | 0.44 | 3.07185 | 9.53150 | 2.02E-03 | 2.03E-02 |
| HSD11B1L | 11-BETA-HSD3 | ENSGALG00000012860 | hydroxysteroid 11-beta dehydrogenase 1 like | 0.44 | 4.77915 | 29.69965 | 5.04E-08 | 3.74E-06 |
| FAM13AL | - - - - - | - - - - - | ----- | 0.44 | 3.07443 | 8.71579 | 3.15E-03 | 2.74E-02 |
| TMEM116 | - - - - - | ENSGALG00000004760 | transmembrane protein 116 | 0.44 | 1.51957 | 7.27106 | 7.01E-03 | 4.83E-02 |
| TMEM35B | - - - - - | ENSGALG00000002517 | transmembrane protein 35B | 0.44 | 3.44676 | 8.03220 | 4.60E-03 | 3.60E-02 |
| ALDH1A2 | RALDH2 | ENSGALG00000004270 | aldehyde dehydrogenase 1 family member A2 | 0.44 | 8.41806 | 31.17865 | 2.35E-08 | 1.89E-06 |
| OLIG3 | - - - - - | - - - - - | oligodendrocyte transcription factor 3 | 0.44 | 2.83127 | 7.78109 | 5.28E-03 | 3.97E-02 |
| PCCA | - - - - - | ENSGALG00000016872 | propionyl-CoA carboxylase alpha subunit | 0.44 | 5.54662 | 46.88072 | 7.54E-12 | 1.50E-09 |
| LOC107049075 | DNAJB1 | - - - - - | DnaJ heat shock protein family (Hsp40) member B1 | 0.44 | 5.95855 | 17.52910 | 2.83E-05 | 7.45E-04 |
| ACOT9 | - - - - - | ENSGALG00000016351 | acyl-CoA thioesterase 9 | 0.43 | 5.90691 | 39.31192 | 3.61E-10 | 4.54E-08 |
| GREM2 | - - - - - | ENSGALG00000055021 | gremlin 2, DAN family BMP antagonist | 0.43 | 3.80495 | 11.41089 | 7.30E-04 | 9.54E-03 |
| DMGDH | - - - - - | ENSGALG00000004491 | dimethylglycine dehydrogenase | 0.43 | 3.58406 | 8.78358 | 3.04E-03 | 2.66E-02 |
| PIGC | - - - - - | ENSGALG00000027608 | phosphatidylinositol glycan anchor biosynthesis class C | 0.43 | 3.84211 | 14.98598 | 1.08E-04 | 2.18E-03 |
| C4H4orf33 | - - - - - | - - - - - | ----- | 0.43 | 3.44109 | 9.94042 | 1.62E-03 | 1.71E-02 |
| PHKB | - - - - - | ENSGALG00000004004 | phosphorylase kinase regulatory subunit beta | 0.43 | 6.35085 | 19.73415 | 8.90E-06 | 2.98E-04 |
| NANS | - - - - - | ENSGALG00000015320 | N-acetylneuraminate synthase | 0.43 | 6.13424 | 35.32014 | 2.80E-09 | 2.82E-07 |
| MUS81 | - - - - - | - - - - - | ----- | 0.43 | 3.76691 | 18.90877 | 1.37E-05 | 4.18E-04 |
| WDR90 | - - - - - | ENSGALG00000028172 | WD repeat domain 90 | 0.43 | 6.46628 | 39.52833 | 3.23E-10 | 4.12E-08 |
| RACGAP1L | - - - - - | - - - - - | ----- | 0.43 | 5.40665 | 21.24319 | 4.05E-06 | 1.54E-04 |

| | | | | | | | | |
|---|---|---|---|---|---|---|---|---|
| BCAT1 | - - - - - | ENSGALG00000013177 | branched chain amino acid transaminase 1 | 0.43 | 6.93915 | 30.24282 | 3.81E-08 | 2.88E-06 |
| CPPED1 | - - - - - | ENSGALG00000040854 | calcineurin like phosphoesterase domain containing 1 | 0.43 | 5.55259 | 30.38603 | 3.54E-08 | 2.71E-06 |
| DPF2 | - - - - - | - - - - - | double PHD fingers 2 | 0.43 | 2.67743 | 10.15178 | 1.44E-03 | 1.57E-02 |
| C20orf196 | - - - - - | - - - - - | ----- | 0.43 | 2.74769 | 9.57720 | 1.97E-03 | 1.99E-02 |
| LOC101750925 | | - - - - - | mucin-5AC-like | 0.43 | 3.29281 | 7.41225 | 6.48E-03 | 4.60E-02 |
| NPTX2 | - - - - - | ENSGALG00000003562 | neuronal pentraxin 2 | 0.43 | 1.95819 | 8.14313 | 4.32E-03 | 3.43E-02 |
| ZBTB46 | - - - - - | ENSGALG00000006062 | zinc finger and BTB domain containing 46 | 0.43 | 6.32650 | 27.37041 | 1.68E-07 | 1.06E-05 |
| LRIG1 | - - - - - | ENSGALG00000007483 | leucine rich repeats and immunoglobulin like domains 1 | 0.43 | 8.00362 | 12.33197 | 4.45E-04 | 6.64E-03 |
| LOC422171 | ARHGAP20B | - - - - - | Rho GTPase activating protein 20B | 0.42 | 4.91541 | 19.86641 | 8.30E-06 | 2.82E-04 |
| SMOC2 | - - - - - | ENSGALG00000011205 | SPARC related modular calcium binding 2 | 0.42 | 6.15762 | 19.87544 | 8.27E-06 | 2.82E-04 |
| COX17 | - - - - - | ENSGALG00000014981 | COX17, cytochrome c oxidase copper chaperone | 0.42 | 4.00233 | 16.70557 | 4.37E-05 | 1.07E-03 |
| LMBR1L | - - - - - | ENSGALG00000037665 | limb development membrane protein 1 like | 0.42 | 4.62112 | 26.47824 | 2.67E-07 | 1.54E-05 |
| GFM2 | - - - - - | ENSGALG00000014939 | G elongation factor mitochondrial 2 | 0.42 | 5.22475 | 16.42403 | 5.06E-05 | 1.21E-03 |
| NME7 | - - - - - | ENSGALG00000015230 | NME/NM23 family member 7 | 0.42 | 4.13936 | 15.13342 | 1.00E-04 | 2.07E-03 |
| LOC107051884 | | - - - - - | uncharacterized LOC107051884 | 0.42 | 1.98471 | 7.87139 | 5.02E-03 | 3.83E-02 |
| ATL1 | - - - - - | ENSGALG00000012339 | atlastin GTPase 1 | 0.42 | 4.93239 | 28.12102 | 1.14E-07 | 7.59E-06 |
| ADAM22 | - - - - - | ENSGALG00000008976 | ADAM metallopeptidase domain 22 | 0.42 | 4.44794 | 16.40470 | 5.12E-05 | 1.21E-03 |
| NKX3-2 | BAPX1 | ENSGALG00000031859 | NK3 homeobox 2 | 0.42 | 5.87746 | 13.26351 | 2.71E-04 | 4.58E-03 |
| LRRC4C | - - - - - | ENSGALG00000007948 | leucine rich repeat containing 4C | 0.42 | 3.70878 | 8.74055 | 3.11E-03 | 2.71E-02 |
| NPR3 | - - - - - | ENSGALG00000017405 | natriuretic peptide receptor 3 | 0.42 | 7.02366 | 8.64019 | 3.29E-03 | 2.82E-02 |
| CTGF | CCN2 | ENSGALG00010004251 | cellular communication network factor 2 | 0.42 | 6.05696 | 22.04502 | 2.66E-06 | 1.08E-04 |
| TLE4Z1 | - - - - - | - - - - - | ----- | 0.42 | 5.35651 | 17.84292 | 2.40E-05 | 6.55E-04 |
| CPZ | - - - - - | ENSGALG00000030209 | carboxypeptidase Z | 0.42 | 6.13455 | 17.29712 | 3.20E-05 | 8.22E-04 |
| LRRC46 | - - - - - | - - - - - | leucine rich repeat containing 46 | 0.42 | 1.79293 | 8.54770 | 3.46E-03 | 2.92E-02 |
| CDH7 | cadherin-7 | ENSGALG00000013782 | cadherin 7 | 0.41 | 4.39787 | 14.76364 | 1.22E-04 | 2.40E-03 |
| LOC407092 | | - - - - - | ubiquitin associated protein 2 | 0.41 | 7.26531 | 19.71362 | 9.00E-06 | 2.99E-04 |
| LOC101752064 | | - - - - - | uncharacterized LOC101752064 | 0.41 | 2.44896 | 7.87445 | 5.01E-03 | 3.83E-02 |
| XPO1 | exportin-1 | ENSGALG00000029476 | exportin 1 | 0.41 | 8.72432 | 33.67099 | 6.53E-09 | 5.97E-07 |
| ARHGAP39L | - - - - - | ENSGALG00000001536 | Rho GTPase activating protein 39 | 0.41 | 4.81529 | 18.39244 | 1.80E-05 | 5.17E-04 |
| KLF9 | - - - - - | ENSGALG00000027374 | Kruppel like factor 9 | 0.41 | 2.64361 | 7.45681 | 6.32E-03 | 4.52E-02 |
| THY1 | - - - - - | ENSGALG00000006751 | Thy-1 cell surface antigen | 0.41 | 2.09143 | 7.55679 | 5.98E-03 | 4.33E-02 |
| LOC107053100 | | - - - - - | basic proline-rich protein-like | 0.41 | 3.46959 | 10.94497 | 9.39E-04 | 1.15E-02 |
| EPHX2 | - - - - - | ENSGALG00000016567 | epoxide hydrolase 2 | 0.41 | 4.78213 | 19.12484 | 1.22E-05 | 3.80E-04 |
| TOP3A | - - - - - | ENSGALG00000005028 | topoisomerase (DNA) III alpha | 0.41 | 5.88343 | 12.32344 | 4.47E-04 | 6.67E-03 |
| ITGB3BP | CENP-R | ENSGALG00000010995 | integrin subunit beta 3 binding protein | 0.41 | 5.28617 | 25.50565 | 4.41E-07 | 2.43E-05 |
| ADCY2 | - - - - - | ENSGALG00000013060 | adenylate cyclase 2 | 0.40 | 2.93028 | 7.87730 | 5.01E-03 | 3.83E-02 |
| LOC107051471 | | - - - - - | fermitin family homolog 3-like | 0.40 | 5.15465 | 9.36962 | 2.21E-03 | 2.15E-02 |
| TPK1 | - - - - - | ENSGALG00000040298 | thiamin pyrophosphokinase 1 | 0.40 | 4.67878 | 18.66951 | 1.55E-05 | 4.64E-04 |
| STK10 | - - - - - | ENSGALG00000002816 | serine/threonine kinase 10 | 0.40 | 4.34186 | 11.38747 | 7.39E-04 | 9.65E-03 |
| RRP36 | - - - - - | ENSGALG00000028056 | ribosomal RNA processing 36 | 0.40 | 6.23017 | 13.24827 | 2.73E-04 | 4.59E-03 |
| DERL3 | - - - - - | ENSGALG00000005994 | derlin 3 | 0.40 | 3.80814 | 11.11914 | 8.54E-04 | 1.07E-02 |
| LOC428824 | | - - - - - | SITS-binding protein-like | 0.40 | 2.94977 | 9.74368 | 1.80E-03 | 1.86E-02 |
| RUVBL1 | - - - - - | ENSGALG00000005931 | RuvB like AAA ATPase 1 | 0.40 | 7.44605 | 24.43298 | 7.69E-07 | 3.84E-05 |
| HSPH1 | - - - - - | ENSGALG00000017077 | heat shock protein family H (Hsp110) member 1 | 0.40 | 7.27078 | 15.38299 | 8.78E-05 | 1.87E-03 |

| | | | | | | | | |
|---|---|---|---|---|---|---|---|---|
| LOC107054036 | | - - - - - | endogenous retrovirus group K member 9 Pol protein-like | 0.40 | 3.61008 | 11.32776 | 7.64E-04 | 9.85E-03 |
| LOC112532692 | | - - - - - | uncharacterized LOC112532692 | 0.40 | 2.75564 | 9.22109 | 2.39E-03 | 2.26E-02 |
| HAUS1 | CCDC5 | ENSGALG00000040397 | HAUS augmin like complex subunit 1 | 0.40 | 4.88655 | 10.64963 | 1.10E-03 | 1.29E-02 |
| DHTKD1 | - - - - - | ENSGALG00000003674 | dehydrogenase E1 and transketolase domain containing 1 | 0.40 | 5.54329 | 12.44709 | 4.19E-04 | 6.33E-03 |
| SYVN1 | - - - - - | - - - - - | synoviolin 1 | 0.40 | 5.57380 | 13.71345 | 2.13E-04 | 3.76E-03 |
| P2RX4 | - - - - - | ENSGALG00000003901 | purinergic receptor P2X 4 | 0.40 | 3.59149 | 17.57099 | 2.77E-05 | 7.33E-04 |
| GFRA2 | NTNRALPHA | ENSGALG00000032856 | GDNF family receptor alpha 2 | 0.40 | 4.90299 | 9.21209 | 2.40E-03 | 2.27E-02 |
| RARRES1 | OCX32 | ENSGALG00000009594 | retinoic acid receptor responder 1 | 0.40 | 4.19737 | 7.26996 | 7.01E-03 | 4.83E-02 |
| SOX3 | CH3 | ENSGALG00000054923 | SRY-box 3 | 0.39 | 4.74932 | 8.50563 | 3.54E-03 | 2.98E-02 |
| CIART | - - - - - | - - - - - | circadian associated repressor of transcription | 0.39 | 2.57972 | 7.93921 | 4.84E-03 | 3.74E-02 |
| ZWILCH | - - - - - | ENSGALG00000007736 | zwilch kinetochore protein | 0.39 | 6.29913 | 32.45825 | 1.22E-08 | 1.04E-06 |
| TRIM47 | - - - - - | ENSGALG00000028301 | tripartite motif containing 47 | 0.39 | 3.23443 | 7.40997 | 6.49E-03 | 4.60E-02 |
| SDHAF1 | - - - - - | - - - - - | succinate dehydrogenase complex assembly factor 1 | 0.39 | 5.07331 | 22.70151 | 1.89E-06 | 8.04E-05 |
| LOC112530910 | | - - - - - | REST corepressor 2-like | 0.39 | 5.28041 | 12.71877 | 3.62E-04 | 5.67E-03 |
| RPS6KB2 | - - - - - | ENSGALG00000031629 | ribosomal protein S6 kinase B2 | 0.39 | 5.38109 | 24.84482 | 6.21E-07 | 3.24E-05 |
| CFAP43 | - - - - - | ENSGALG00000036896 | cilia and flagella associated protein 43 | 0.39 | 4.06222 | 9.45765 | 2.10E-03 | 2.09E-02 |
| IRAK2 | - - - - - | ENSGALG00000008407 | interleukin 1 receptor associated kinase 2 | 0.39 | 5.73068 | 33.83440 | 6.00E-09 | 5.58E-07 |
| ENOX1 | - - - - - | ENSGALG00000016967 | ecto-NOX disulfide-thiol exchanger 1 | 0.39 | 4.42873 | 8.38293 | 3.79E-03 | 3.13E-02 |
| PSTK | - - - - - | ENSGALG00000009610 | phosphoseryl-tRNA kinase | 0.39 | 3.95351 | 10.28026 | 1.34E-03 | 1.49E-02 |
| PDE6G | - - - - - | ENSGALG00000004514 | phosphodiesterase 6G | 0.39 | 3.98057 | 13.88633 | 1.94E-04 | 3.49E-03 |
| BCCIP | - - - - - | ENSGALG00000006617 | BRCA2 and CDKN1A interacting protein | 0.39 | 5.70840 | 40.59414 | 1.87E-10 | 2.68E-08 |
| PUDP | - - - - - | ENSGALG00000040388 | pseudouridine 5'-phosphatase | 0.38 | 3.05320 | 8.36926 | 3.82E-03 | 3.14E-02 |
| LOC426743 | | - - - - - | probable beta-D-xylosidase 5-like | 0.38 | 3.57174 | 12.59570 | 3.87E-04 | 5.97E-03 |
| LOC419584 | | - - - - - | discoidin, CUB and LCCL domain-containing protein 1-like | 0.38 | 3.87216 | 9.06319 | 2.61E-03 | 2.39E-02 |
| IFT22 | - - - - - | ENSGALG00000021653 | intraflagellar transport 22 | 0.38 | 4.02489 | 16.87648 | 3.99E-05 | 9.93E-04 |
| IVD | - - - - - | ENSGALG00000040267 | isovaleryl-CoA dehydrogenase | 0.38 | 5.28536 | 29.37212 | 5.97E-08 | 4.33E-06 |
| EIF4A2 | EIF4A1 | ENSGALG00000008684 | eukaryotic translation initiation factor 4A2 | 0.38 | 9.20421 | 12.92457 | 3.24E-04 | 5.21E-03 |
| NDRG1 | - - - - - | ENSGALG00000054783 | N-myc downstream regulated 1 | 0.38 | 5.28635 | 19.23987 | 1.15E-05 | 3.65E-04 |
| KIF15 | - - - - - | ENSGALG00000036645 | kinesin family member 15 | 0.38 | 7.70225 | 29.75511 | 4.90E-08 | 3.66E-06 |
| APTX | FHA-HIT | ENSGALG00000001954 | aprataxin | 0.38 | 4.71755 | 9.34086 | 2.24E-03 | 2.17E-02 |
| C1H11ORF70 | C11orf70 | ENSGALG00000017189 | chromosome 1 open reading frame, human C11orf70 | 0.38 | 2.02884 | 8.37166 | 3.81E-03 | 3.14E-02 |
| ARG2 | - - - - - | ENSGALG00000009519 | arginase 2 | 0.38 | 6.38596 | 33.32830 | 7.78E-09 | 6.95E-07 |
| POLE4 | - - - - - | ENSGALG00000031679 | DNA polymerase epsilon 4, accessory subunit | 0.38 | 5.25114 | 24.69622 | 6.71E-07 | 3.47E-05 |
| ZNF624 | - - - - - | - - - - - | zinc finger protein 624 | 0.38 | 4.20296 | 10.52695 | 1.18E-03 | 1.35E-02 |
| ERN2 | - - - - - | ENSGALG00000027592 | endoplasmic reticulum to nucleus signaling 2 | 0.38 | 3.48310 | 11.77936 | 5.99E-04 | 8.27E-03 |
| SOX1 | - - - - - | ENSGALG00000030353 | SRY-box 1 | 0.38 | 4.02537 | 7.58830 | 5.87E-03 | 4.27E-02 |
| ABCB6 | - - - - - | ENSGALG00000029672 | ATP binding cassette subfamily B member 6 (Langereis blood group) | 0.38 | 4.22938 | 12.20211 | 4.77E-04 | 6.99E-03 |
| HYLS1 | - - - - - | ENSGALG00000036796 | HYLS1, centriolar and ciliogenesis associated | 0.37 | 3.80127 | 12.74268 | 3.57E-04 | 5.60E-03 |
| NPC2 | - - - - - | ENSGALG00000010237 | Niemann-Pick disease, type C2 | 0.37 | 5.52158 | 9.47869 | 2.08E-03 | 2.07E-02 |
| MRPS34 | - - - - - | ENSGALG00000001792 | mitochondrial ribosomal protein S34 | 0.37 | 6.31722 | 39.01502 | 4.21E-10 | 5.16E-08 |
| NR2F1 | - - - - - | ENSGALG00000027907 | nuclear receptor subfamily 2 group F member 1 | 0.37 | 7.13256 | 18.36275 | 1.83E-05 | 5.23E-04 |
| FOXO6 | - - - - - | ENSGALG00000026153 | forkhead box O6 | 0.37 | 5.09217 | 17.40343 | 3.02E-05 | 7.89E-04 |
| SLC26A8 | - - - - - | ENSGALG00000040498 | solute carrier family 26 member 8 | 0.37 | 3.19351 | 10.43476 | 1.24E-03 | 1.40E-02 |
| RDH5 | - - - - - | ENSGALG00000034988 | retinol dehydrogenase 5 | 0.37 | 3.71502 | 13.67097 | 2.18E-04 | 3.84E-03 |

| | | | | | | | | |
|---|---|---|---|---|---|---|---|---|
| MATN2 | - - - - - | ENSGALG00000044675 | matrilin 2 | 0.37 | 3.65444 | 10.27089 | 1.35E-03 | 1.49E-02 |
| PIF1 | - - - - - | ENSGALG00000009032 | PIF1 5'-to-3' DNA helicase | 0.37 | 4.89973 | 9.28543 | 2.31E-03 | 2.21E-02 |
| FAM177A1 | - - - - - | ENSGALG00000010046 | family with sequence similarity 177 member A1 | 0.37 | 5.40244 | 35.62676 | 2.39E-09 | 2.44E-07 |
| RNF32 | - - - - - | ENSGALG00000006387 | ring finger protein 32 | 0.37 | 5.84118 | 26.82280 | 2.23E-07 | 1.33E-05 |
| KIF26A | - - - - - | ENSGALG00000011581 | kinesin family member 26A | 0.37 | 7.50441 | 11.73045 | 6.15E-04 | 8.45E-03 |
| LPAR2 | EDG4 | ENSGALG00000042767 | lysophosphatidic acid receptor 2 | 0.37 | 4.74568 | 12.69078 | 3.67E-04 | 5.74E-03 |
| CLN5 | - - - - - | ENSGALG00000016917 | ceroid-lipofuscinosis, neuronal 5 | 0.36 | 3.52719 | 10.16593 | 1.43E-03 | 1.56E-02 |
| WDR24 | - - - - - | ENSGALG00000002450 | WD repeat domain 24 | 0.36 | 5.89105 | 24.10637 | 9.12E-07 | 4.45E-05 |
| TDRD7 | - - - - - | ENSGALG00000012350 | tudor domain containing 7 | 0.36 | 4.10349 | 10.86841 | 9.78E-04 | 1.19E-02 |
| WDR70 | - - - - - | ENSGALG00000003708 | WD repeat domain 70 | 0.36 | 5.95855 | 13.62900 | 2.23E-04 | 3.90E-03 |
| RHBDD1 | - - - - - | ENSGALG00000004957 | rhomboid domain containing 1 | 0.36 | 3.51853 | 9.10744 | 2.55E-03 | 2.36E-02 |
| FOXC1 | CFKH-1 | ENSGALG00000030442 | forkhead box C1 | 0.36 | 4.32065 | 8.34429 | 3.87E-03 | 3.17E-02 |
| CDH6 | c-cad6B | ENSGALG00000012917 | cadherin 6 | 0.36 | 5.76672 | 11.76177 | 6.05E-04 | 8.34E-03 |
| ENTPD2 | - - - - - | ENSGALG00000039696 | ectonucleoside triphosphate diphosphohydrolase 2 | 0.36 | 3.64873 | 8.16501 | 4.27E-03 | 3.40E-02 |
| SMCO4 | C11ORF75 | ENSGALG00000026639 | single-pass membrane protein with coiled-coil domains 4 | 0.36 | 3.88423 | 11.82136 | 5.86E-04 | 8.12E-03 |
| PRSS35 | - - - - - | ENSGALG00000055053 | serine protease 35 | 0.36 | 4.77094 | 8.41002 | 3.73E-03 | 3.10E-02 |
| COPS9 | MYEOV2 | ENSGALG00000052051 | COP9 signalosome subunit 9 | 0.36 | 5.31211 | 26.86142 | 2.19E-07 | 1.32E-05 |
| JUN | - - - - - | ENSGALG00000010870 | Jun proto-oncogene, AP-1 transcription factor subunit | 0.36 | 6.42953 | 9.39030 | 2.18E-03 | 2.14E-02 |
| LOC107051855 | | - - - - - | uncharacterized LOC107051855 | 0.36 | 4.39049 | 8.72816 | 3.13E-03 | 2.72E-02 |
| SLC22A23 | - - - - - | ENSGALG00000012816 | solute carrier family 22 member 23 | 0.36 | 4.83495 | 16.40440 | 5.12E-05 | 1.21E-03 |
| KANSL2 | - - - - - | ENSGALG00000033877 | KAT8 regulatory NSL complex subunit 2 | 0.36 | 6.60512 | 26.74517 | 2.32E-07 | 1.37E-05 |
| LOC100858388 | | - - - - - | 78 kDa glucose-regulated protein-like | 0.36 | 5.61814 | 7.64871 | 5.68E-03 | 4.18E-02 |
| TEX2L | - - - - - | - - - - - | testis expressed 2 | 0.36 | 2.28410 | 7.19154 | 7.32E-03 | 4.98E-02 |
| RNF213 | - - - - - | ENSGALG00000039269 | ring finger protein 213 | 0.36 | 3.21741 | 8.14427 | 4.32E-03 | 3.43E-02 |
| TSEN15 | - - - - - | ENSGALG00000004782 | tRNA splicing endonuclease subunit 15 | 0.36 | 2.64495 | 7.94237 | 4.83E-03 | 3.74E-02 |
| LOC107053815 | | - - - - - | uncharacterized LOC107053815 | 0.36 | 3.53029 | 8.00443 | 4.67E-03 | 3.64E-02 |
| COQ2 | - - - - - | ENSGALG00000041294 | coenzyme Q2, polyprenyltransferase | 0.36 | 2.95418 | 8.18851 | 4.22E-03 | 3.38E-02 |
| MUTYH | - - - - - | ENSGALG00000010226 | mutY DNA glycosylase | 0.35 | 5.32423 | 15.20812 | 9.63E-05 | 2.01E-03 |
| HOXD4 | Chox-a | ENSGALG00000023419 | homeobox D4 | 0.35 | 6.02453 | 12.98056 | 3.15E-04 | 5.11E-03 |
| LHFPL4 | - - - - - | ENSGALG00000048164 | lipoma HMGIC fusion partner-like 4 | 0.35 | 3.13048 | 9.46303 | 2.10E-03 | 2.08E-02 |
| PKDCCA | - - - - - | ENSGALG00000042511 | protein kinase domain containing, cytoplasmic A | 0.35 | 6.21057 | 11.34096 | 7.58E-04 | 9.81E-03 |
| CRYL1 | - - - - - | ENSGALG00000017135 | crystallin lambda 1 | 0.35 | 3.49866 | 11.11022 | 8.59E-04 | 1.07E-02 |
| ACSF2 | - - - - - | ENSGALG00000019768 | acyl-CoA synthetase family member 2 | 0.35 | 5.59660 | 25.35484 | 4.77E-07 | 2.57E-05 |
| MYO19 | - - - - - | ENSGALG00000005374 | myosin XIX | 0.35 | 5.36976 | 16.58873 | 4.64E-05 | 1.12E-03 |
| RCCD1 | - - - - - | - - - - - | RCC1 domain containing 1 | 0.35 | 3.40229 | 9.45119 | 2.11E-03 | 2.09E-02 |
| C1HXORF36 | - - - - - | - - - - - | ----- | 0.35 | 4.41523 | 13.08025 | 2.98E-04 | 4.91E-03 |
| LOC101748153 | | - - - - - | laminin subunit beta-2-like | 0.35 | 5.42171 | 9.73861 | 1.80E-03 | 1.86E-02 |
| BRIP1 | - - - - - | ENSGALG00000005279 | BRCA1 interacting protein C-terminal helicase 1 | 0.35 | 4.57509 | 16.73027 | 4.31E-05 | 1.06E-03 |
| NDUFA5 | - - - - - | ENSGALG00000008821 | NADH:ubiquinone oxidoreductase subunit A5 | 0.35 | 5.79627 | 16.48855 | 4.89E-05 | 1.17E-03 |
| LYRM7 | - - - - - | ENSGALG00000037299 | LYR motif containing 7 | 0.35 | 3.70271 | 8.35611 | 3.84E-03 | 3.15E-02 |
| RASL11AL | - - - - - | ENSGALG00000007710 | ras-like protein family member 11A-like | 0.35 | 3.34609 | 8.40235 | 3.75E-03 | 3.10E-02 |
| GALNT2 | - - - - - | ENSGALG00000011106 | polypeptide N-acetylgalactosaminyltransferase 2 | 0.35 | 6.09882 | 30.77469 | 2.90E-08 | 2.26E-06 |
| CNP | - - - - - | ENSGALG00000003457 | 2',3'-cyclic nucleotide 3' phosphodiesterase | 0.35 | 4.70616 | 15.03148 | 1.06E-04 | 2.15E-03 |
| LOC107050163 | | - - - - - | probable very-long-chain enoyl-CoA reductase art-1 | 0.35 | 7.23556 | 20.34898 | 6.45E-06 | 2.28E-04 |

| | | | | | | | | |
|---|---|---|---|---|---|---|---|---|
| LOC107055019 | | ----- | uncharacterized LOC107055019 | 0.35 | 4.64229 | 9.18885 | 2.43E-03 | 2.29E-02 |
| EFHD2 | ----- | ENSGALG00000038313 | EF-hand domain family member D2 | 0.35 | 3.71877 | 7.65465 | 5.66E-03 | 4.17E-02 |
| C14H7orf50 | ----- | ENSGALG00000004055 | chromosome 14 C7orf50 homolog | 0.35 | 5.61980 | 22.24157 | 2.40E-06 | 9.91E-05 |
| HR | ----- | ----- | ----- | 0.34 | 3.75471 | 11.21321 | 8.12E-04 | 1.03E-02 |
| TUBB6 | ----- | ENSGALG00000031275 | tubulin beta 6 class V | 0.34 | 6.55392 | 10.59775 | 1.13E-03 | 1.32E-02 |
| C25H1ORF43 | C1orf43 | ENSGALG00000034258 | chromosome 25 open reading frame, human C1orf43 | 0.34 | 5.70825 | 23.02032 | 1.60E-06 | 7.00E-05 |
| SLC41A2 | ----- | ENSGALG00000012697 | solute carrier family 41 member 2 | 0.34 | 3.71809 | 8.18363 | 4.23E-03 | 3.38E-02 |
| DYNC2LI1 | ----- | ENSGALG00000009954 | dynein cytoplasmic 2 light intermediate chain 1 | 0.34 | 4.50562 | 13.38529 | 2.54E-04 | 4.35E-03 |
| TRAPPC2 | ----- | ENSGALG00000016578 | trafficking protein particle complex 2 | 0.34 | 5.96655 | 25.15805 | 5.28E-07 | 2.82E-05 |
| ORAOV1 | LTO1 | ENSGALG00000007556 | oral cancer overexpressed 1 | 0.34 | 6.29519 | 24.10758 | 9.11E-07 | 4.45E-05 |
| MRPL49 | ----- | ----- | mitochondrial ribosomal protein L49 | 0.34 | 4.75569 | 16.33090 | 5.32E-05 | 1.26E-03 |
| KIAA1210 | ----- | ENSGALG00000013771 | KIAA1210 | 0.34 | 4.67725 | 12.24603 | 4.66E-04 | 6.87E-03 |
| BRK1 | ----- | ----- | BRICK1, SCAR/WAVE actin nucleating complex subunit | 0.34 | 3.93947 | 11.30780 | 7.72E-04 | 9.92E-03 |
| KAT5 | ----- | ----- | lysine acetyltransferase 5 | 0.34 | 5.57935 | 34.56113 | 4.13E-09 | 4.00E-07 |
| PNPLA3 | ----- | ENSGALG00000029308 | patatin like phospholipase domain containing 3 | 0.34 | 6.02791 | 12.66205 | 3.73E-04 | 5.80E-03 |
| H1FX | ----- | ----- | H1 histone family member X | 0.34 | 8.49696 | 14.84147 | 1.17E-04 | 2.32E-03 |
| PEX11B | ----- | ENSGALG00000052329 | peroxisomal biogenesis factor 11 beta | 0.34 | 2.95346 | 9.00077 | 2.70E-03 | 2.45E-02 |
| GLIPR2 | ----- | ENSGALG00000028821 | GLI pathogenesis related 2 | 0.34 | 5.60762 | 19.17631 | 1.19E-05 | 3.74E-04 |
| GMNN | ----- | ENSGALG00000013626 | geminin, DNA replication inhibitor | 0.34 | 5.87868 | 17.32179 | 3.16E-05 | 8.16E-04 |
| MTHFD2 | ----- | ENSGALG00000034344 | methylenetetrahydrofolate dehydrogenase 2, cyclohydrolase | 0.34 | 4.99082 | 13.17540 | 2.84E-04 | 4.72E-03 |
| CENPK | CENP-K | ENSGALG00000014753 | centromere protein K | 0.34 | 5.78669 | 11.28427 | 7.82E-04 | 1.00E-02 |
| LOC101748987 | | ----- | FCH domain only protein 1-like | 0.34 | 3.96389 | 9.27201 | 2.33E-03 | 2.22E-02 |
| HOXB4 | Chox-Z | ENSGALG00000000284 | homeobox B4 | 0.34 | 7.42851 | 12.70386 | 3.65E-04 | 5.71E-03 |
| SCO1 | ----- | ENSGALG00000030475 | SCO1, cytochrome c oxidase assembly protein | 0.34 | 5.68592 | 16.79272 | 4.17E-05 | 1.03E-03 |
| ZNF414 | ----- | ENSGALG00000024378 | zinc finger protein 414 | 0.34 | 3.99504 | 8.90065 | 2.85E-03 | 2.55E-02 |
| YWHAH | ----- | ENSGALG00000006743 | tyrosine 3-monooxygenase/tryptophan 5-monooxygenase activation protein eta | 0.34 | 7.81414 | 24.55201 | 7.23E-07 | 3.67E-05 |
| MRPS24 | ----- | ENSGALG00000052064 | mitochondrial ribosomal protein S24 | 0.33 | 4.33327 | 11.55702 | 6.75E-04 | 9.05E-03 |
| LOC770165 | | ----- | BUD13 homolog | 0.33 | 3.98230 | 12.21358 | 4.74E-04 | 6.96E-03 |
| NFKB1 | NF-KB1 | ENSGALG00000012304 | nuclear factor kappa B subunit 1 | 0.33 | 4.98983 | 18.44768 | 1.75E-05 | 5.04E-04 |
| ANKLE1 | ----- | ENSGALG00000051336 | ankyrin repeat and LEM domain containing 1 | 0.33 | 5.65795 | 10.56754 | 1.15E-03 | 1.33E-02 |
| HOXB2 | ----- | ENSGALG00000025774 | homeobox B2 | 0.33 | 5.32757 | 14.71410 | 1.25E-04 | 2.45E-03 |
| TGFBR2 | ----- | ENSGALG00000011442 | transforming growth factor beta receptor 2 | 0.33 | 4.78532 | 14.42067 | 1.46E-04 | 2.78E-03 |
| VEGFC | ----- | ENSGALG00000010847 | vascular endothelial growth factor C | 0.33 | 4.22964 | 11.67180 | 6.35E-04 | 8.62E-03 |
| SPATA20 | ----- | ENSGALG00000006857 | spermatogenesis associated 20 | 0.33 | 5.57901 | 18.23008 | 1.96E-05 | 5.55E-04 |
| TMEM132B | ----- | ENSGALG00000002742 | transmembrane protein 132B | 0.33 | 4.36156 | 8.23207 | 4.12E-03 | 3.31E-02 |
| N4BP2 | ----- | ----- | NEDD4 binding protein 2 | 0.33 | 5.99959 | 15.93591 | 6.55E-05 | 1.49E-03 |
| TBC1D2 | ----- | ENSGALG00000001944 | TBC1 domain family member 2 | 0.33 | 4.12846 | 7.57728 | 5.91E-03 | 4.29E-02 |
| ARL14EP | ARF7EP | ENSGALG00000012139 | ADP ribosylation factor like GTPase 14 effector protein | 0.33 | 5.18156 | 15.37501 | 8.81E-05 | 1.88E-03 |
| PLA2R1 | ----- | ENSGALG00000011149 | phospholipase A2 receptor 1 | 0.33 | 5.48317 | 9.27855 | 2.32E-03 | 2.22E-02 |
| FRS3 | ----- | ENSGALG00000003447 | fibroblast growth factor receptor substrate 3 | 0.33 | 6.19706 | 19.70765 | 9.02E-06 | 2.99E-04 |
| ZNF252L | ----- | ----- | ----- | 0.33 | 4.60149 | 9.89162 | 1.66E-03 | 1.74E-02 |
| RAD50 | ----- | ENSGALG00000006794 | RAD50 double strand break repair protein | 0.33 | 6.44237 | 18.51562 | 1.69E-05 | 4.90E-04 |
| LOC101752260 | | ----- | uncharacterized LOC101752260 | 0.33 | 5.52499 | 20.67906 | 5.43E-06 | 1.99E-04 |
| TMEM106C | ----- | ENSGALG00000034069 | transmembrane protein 106C | 0.33 | 3.80079 | 11.51181 | 6.92E-04 | 9.19E-03 |

| | | | | | | | | |
|---|---|---|---|---|---|---|---|---|
| HSP90B1 | hsp108 | ENSGALG00000033789 | heat shock protein 90 beta family member 1 | 0.33 | 9.79887 | 11.59500 | 6.61E-04 | 8.92E-03 |
| GPR155 | ----- | ENSGALG00000014209 | G protein-coupled receptor 155 | 0.33 | 5.15871 | 24.45376 | 7.61E-07 | 3.81E-05 |
| LOC101750687 | | ----- | uncharacterized LOC101750687 | 0.33 | 5.86846 | 17.98886 | 2.22E-05 | 6.15E-04 |
| HIBADH | ----- | ENSGALG00000033207 | 3-hydroxyisobutyrate dehydrogenase | 0.32 | 5.10040 | 19.67409 | 9.18E-06 | 3.03E-04 |
| NDUFAF4 | ----- | ENSGALG00000019921 | NADH:ubiquinone oxidoreductase complex assembly factor 4 | 0.32 | 4.95405 | 12.30541 | 4.52E-04 | 6.71E-03 |
| LMCD1 | ----- | ENSGALG00000008349 | LIM and cysteine rich domains 1 | 0.32 | 5.44100 | 9.31629 | 2.27E-03 | 2.19E-02 |
| VASP | ----- | ----- | vasodilator-stimulated phosphoprotein | 0.32 | 4.07252 | 9.08617 | 2.58E-03 | 2.37E-02 |
| BUD13 | ----- | ENSGALG00000025882 | BUD13 homolog | 0.32 | 5.73986 | 26.70938 | 2.36E-07 | 1.39E-05 |
| SHCBP1 | ----- | ENSGALG00000004444 | SHC binding and spindle associated 1 | 0.32 | 5.34029 | 18.18546 | 2.00E-05 | 5.65E-04 |
| COA5 | ----- | ENSGALG00000036791 | cytochrome c oxidase assembly factor 5 | 0.32 | 5.49248 | 23.57878 | 1.20E-06 | 5.51E-05 |
| EXOG | ----- | ENSGALG00000006153 | exo/endonuclease G | 0.32 | 6.73897 | 26.77364 | 2.29E-07 | 1.36E-05 |
| FAM43A | ----- | ENSGALG00000041898 | family with sequence similarity 43 member A | 0.32 | 5.71185 | 12.93953 | 3.22E-04 | 5.19E-03 |
| PITPNM2 | ----- | ----- | phosphatidylinositol transfer protein membrane associated 2 | 0.32 | 5.05450 | 15.27229 | 9.31E-05 | 1.96E-03 |
| SHC2 | ----- | ENSGALG00000001289 | SHC (Src homology 2 domain containing) transforming protein 2 | 0.32 | 4.74605 | 8.08370 | 4.47E-03 | 3.52E-02 |
| TMEM177 | ----- | ENSGALG00000011593 | transmembrane protein 177 | 0.32 | 4.62737 | 15.62560 | 7.72E-05 | 1.70E-03 |
| LOC107052842 | | ----- | uncharacterized LOC107052842 | 0.32 | 4.52545 | 12.78823 | 3.49E-04 | 5.51E-03 |
| CAMK4L | ----- | ----- | calcium/calmodulin-dependent protein kinase IV-like | 0.32 | 4.49034 | 10.01079 | 1.56E-03 | 1.66E-02 |
| NFYA | ----- | ENSGALG00000003247 | nuclear transcription factor Y subunit alpha | 0.32 | 7.16913 | 19.23455 | 1.16E-05 | 3.66E-04 |
| MRPL55 | ----- | ENSGALG00000038820 | mitochondrial ribosomal protein L55 | 0.32 | 4.08981 | 7.47926 | 6.24E-03 | 4.48E-02 |
| TTC30B | TTC30A | ENSGALG00000009224 | tetratricopeptide repeat domain 30B | 0.32 | 4.92755 | 11.67842 | 6.32E-04 | 8.59E-03 |
| ZYX | ----- | ENSGALG00000014688 | zyxin | 0.32 | 7.10455 | 27.27895 | 1.76E-07 | 1.11E-05 |
| TBCB | ----- | ----- | tubulin folding cofactor B | 0.32 | 6.11512 | 17.01605 | 3.71E-05 | 9.35E-04 |
| PRR7 | ----- | ENSGALG00000041981 | proline rich 7, synaptic | 0.32 | 5.27858 | 20.09519 | 7.37E-06 | 2.54E-04 |
| JPT2 | ----- | ----- | ----- | 0.32 | 8.60599 | 26.89714 | 2.15E-07 | 1.30E-05 |
| BROX | ----- | ENSGALG00000009413 | BRO1 domain and CAAX motif containing | 0.32 | 5.20128 | 14.80665 | 1.19E-04 | 2.35E-03 |
| FADS1 | ----- | ENSGALG00000007127 | fatty acid desaturase 1 | 0.32 | 6.41988 | 36.76941 | 1.33E-09 | 1.48E-07 |
| CDC20 | ----- | ENSGALG00000009971 | cell division cycle 20 | 0.31 | 7.69815 | 27.13538 | 1.90E-07 | 1.17E-05 |
| USP43 | ----- | ENSGALG00000037812 | ubiquitin specific peptidase 43 | 0.31 | 5.37032 | 7.69824 | 5.53E-03 | 4.09E-02 |
| TMEM199 | ----- | ENSGALG00000023757 | transmembrane protein 199 | 0.31 | 4.97420 | 15.85585 | 6.84E-05 | 1.55E-03 |
| NMRK2 | ----- | ENSGALG00000025751 | nicotinamide riboside kinase 2 | 0.31 | 4.82557 | 15.36900 | 8.84E-05 | 1.88E-03 |
| LOC107053944 | | ----- | uncharacterized LOC107053944 | 0.31 | 5.38288 | 8.13818 | 4.33E-03 | 3.44E-02 |
| SATB1 | ----- | ENSGALG00000011254 | SATB homeobox 1 | 0.31 | 4.21084 | 8.45566 | 3.64E-03 | 3.05E-02 |
| DTNB | ----- | ----- | dystrobrevin beta | 0.31 | 5.47033 | 21.29962 | 3.93E-06 | 1.51E-04 |
| IER5L | ----- | ENSGALG00000053552 | immediate early response 5 like | 0.31 | 6.94178 | 11.68349 | 6.31E-04 | 8.58E-03 |
| SS18L2 | ----- | ENSGALG00000004943 | SS18 like 2 | 0.31 | 4.48349 | 15.50999 | 8.21E-05 | 1.78E-03 |
| LOC107056278 | | ----- | probable N-acetyltransferase camello | 0.31 | 5.04002 | 8.61021 | 3.34E-03 | 2.86E-02 |
| NSDHL | ----- | ENSGALG00000007493 | NAD(P) dependent steroid dehydrogenase-like | 0.31 | 4.37155 | 7.88355 | 4.99E-03 | 3.82E-02 |
| ZRANB2 | ZNF265 | ENSGALG00000011340 | zinc finger RANBP2-type containing 2 | 0.31 | 7.07814 | 22.06781 | 2.63E-06 | 1.07E-04 |
| IFI35 | ----- | ENSGALG00000002832 | interferon-induced protein 35 | 0.31 | 3.99079 | 8.74597 | 3.10E-03 | 2.70E-02 |
| CTSH | ----- | ENSGALG00000033557 | cathepsin H | 0.31 | 5.52223 | 16.90103 | 3.94E-05 | 9.82E-04 |
| ANXA5 | ANX5 | ENSGALG00000011885 | annexin A5 | 0.31 | 6.82847 | 23.82507 | 1.05E-06 | 4.97E-05 |
| CRELD2 | ----- | ENSGALG00000008551 | cysteine rich with EGF like domains 2 | 0.31 | 5.76529 | 14.65524 | 1.29E-04 | 2.52E-03 |
| LOC107051531 | PPP1R14CL | ----- | protein phosphatase 1 regulatory subunit 14C like | 0.31 | 5.89869 | 18.50432 | 1.70E-05 | 4.92E-04 |
| CREB5 | ----- | ENSGALG00000038265 | cAMP responsive element binding protein 5 | 0.31 | 4.29376 | 9.85734 | 1.69E-03 | 1.77E-02 |

| | | | | | | | | |
|---|---|---|---|---|---|---|---|---|
| TGFB1 | ----- | ENSGALG00000031325 | transforming growth factor beta 1 | 0.31 | 4.83647 | 15.26530 | 9.34E-05 | 1.96E-03 |
| HMGA1 | ----- | ENSGALG00000030396 | high mobility group AT-hook 1 | 0.31 | 6.81632 | 25.63604 | 4.12E-07 | 2.28E-05 |
| DUSP8 | ----- | ENSGALG00000006647 | dual specificity phosphatase 8 | 0.31 | 4.98313 | 11.49978 | 6.96E-04 | 9.23E-03 |
| LOC112531536 | | ----- | uncharacterized LOC112531536 | 0.31 | 5.13370 | 12.50949 | 4.05E-04 | 6.19E-03 |
| AAAS | ----- | ENSGALG00000032843 | aladin WD repeat nucleoporin | 0.31 | 7.27204 | 25.09461 | 5.46E-07 | 2.89E-05 |
| RFXANK | ----- | ENSGALG00000037261 | regulatory factor X associated ankyrin containing protein | 0.31 | 4.64332 | 16.62770 | 4.55E-05 | 1.11E-03 |
| GAS2L3 | ----- | ENSGALG00000028373 | growth arrest specific 2 like 3 | 0.31 | 4.72986 | 9.61278 | 1.93E-03 | 1.96E-02 |
| TGFB2 | TGF-beta-2 | ENSGALG00000009612 | transforming growth factor beta 2 | 0.31 | 5.54526 | 8.61529 | 3.33E-03 | 2.85E-02 |
| HSD17B4 | ----- | ENSGALG00000002187 | hydroxysteroid 17-beta dehydrogenase 4 | 0.31 | 6.25256 | 11.86001 | 5.74E-04 | 8.04E-03 |
| LMNB1 | lamin-B1 | ENSGALG00000014692 | lamin B1 | 0.31 | 7.32189 | 12.50829 | 4.05E-04 | 6.19E-03 |
| CCDC189 | ----- | ENSGALG00000041514 | coiled-coil domain containing 189 | 0.31 | 4.21516 | 11.13782 | 8.46E-04 | 1.06E-02 |
| EXOSC1 | ----- | ENSGALG00000039917 | exosome component 1 | 0.30 | 4.92820 | 16.44001 | 5.02E-05 | 1.20E-03 |
| ORC5 | ORC5L | ENSGALG00000008175 | origin recognition complex subunit 5 | 0.30 | 5.18173 | 10.84889 | 9.89E-04 | 1.19E-02 |
| RAB11FIP3 | ----- | ENSGALG00000002666 | RAB11 family interacting protein 3 | 0.30 | 6.63889 | 21.89653 | 2.88E-06 | 1.15E-04 |
| CBS | ----- | ----- | ----- | 0.30 | 7.53064 | 15.02034 | 1.06E-04 | 2.16E-03 |
| NKIRAS2 | ----- | ENSGALG00000028971 | NFKB inhibitor interacting Ras like 2 | 0.30 | 5.79833 | 20.58811 | 5.69E-06 | 2.07E-04 |
| GTF3C3 | ----- | ENSGALG00000007958 | general transcription factor IIIC subunit 3 | 0.30 | 4.96835 | 17.27875 | 3.23E-05 | 8.27E-04 |
| APOOL | ----- | ENSGALG00000006978 | apolipoprotein O like | 0.30 | 6.46995 | 20.97974 | 4.64E-06 | 1.73E-04 |
| LOC421106 | | ----- | KIAA0146 | 0.30 | 5.02463 | 13.44480 | 2.46E-04 | 4.24E-03 |
| RARA | RAR-ALPHA1 | ENSGALG00000037935 | retinoic acid receptor alpha | 0.30 | 6.30790 | 9.74703 | 1.80E-03 | 1.86E-02 |
| GALNT6 | ----- | ENSGALG00000041101 | polypeptide N-acetylgalactosaminyltransferase 6 | 0.30 | 4.09825 | 9.19900 | 2.42E-03 | 2.28E-02 |
| NDUFB2 | ----- | ENSGALG00000032595 | NADH:ubiquinone oxidoreductase subunit B2 | 0.30 | 5.57953 | 8.55946 | 3.44E-03 | 2.91E-02 |
| CCDC34 | ----- | ----- | coiled-coil domain containing 34 | 0.30 | 3.72225 | 7.86986 | 5.03E-03 | 3.83E-02 |
| OSGEPL1 | ----- | ENSGALG00000037727 | O-sialoglycoprotein endopeptidase like 1 | 0.30 | 4.83170 | 14.97034 | 1.09E-04 | 2.19E-03 |
| ALPL | ----- | ENSGALG00000023542 | alkaline phosphatase, liver/bone/kidney | 0.30 | 5.30991 | 14.06151 | 1.77E-04 | 3.24E-03 |
| PMPCB | ----- | ENSGALG00000008280 | peptidase, mitochondrial processing beta subunit | 0.30 | 6.25293 | 17.32627 | 3.15E-05 | 8.16E-04 |
| RASGEF1B | ----- | ENSGALG00000010903 | RasGEF domain family member 1B | 0.30 | 4.42683 | 8.58402 | 3.39E-03 | 2.88E-02 |
| SDCCAG8 | ----- | ENSGALG00000010713 | serologically defined colon cancer antigen 8 | 0.30 | 4.79484 | 14.34328 | 1.52E-04 | 2.86E-03 |
| MKNK2 | ----- | ENSGALG00000003845 | MAP kinase interacting serine/threonine kinase 2 | 0.30 | 5.71458 | 13.89969 | 1.93E-04 | 3.47E-03 |
| UBE4B | ----- | ENSGALG00000002649 | ubiquitination factor E4B | 0.30 | 7.32902 | 34.47324 | 4.32E-09 | 4.13E-07 |
| IL12RB2 | ----- | ENSGALG00000011222 | interleukin 12 receptor subunit beta 2 | 0.30 | 5.14765 | 14.74355 | 1.23E-04 | 2.43E-03 |
| TYMS | ----- | ----- | thymidylate synthetase | 0.30 | 6.60425 | 25.20965 | 5.14E-07 | 2.76E-05 |
| PQLC2L | ----- | ENSGALG00000020626 | PQ loop repeat containing 2 like | 0.30 | 5.36030 | 10.06913 | 1.51E-03 | 1.63E-02 |
| GREB1 | ----- | ENSGALG00000016455 | growth regulating estrogen receptor binding 1 | 0.30 | 6.65964 | 13.80394 | 2.03E-04 | 3.62E-03 |
| POLE3 | ----- | ENSGALG00000008873 | DNA polymerase epsilon 3, accessory subunit | 0.30 | 7.36074 | 32.64132 | 1.11E-08 | 9.55E-07 |
| DAPK3 | ----- | ENSGALG00000037733 | death-associated protein kinase 3 | 0.30 | 6.92271 | 17.97167 | 2.24E-05 | 6.19E-04 |
| IFT74 | ----- | ENSGALG00000001801 | intraflagellar transport 74 | 0.30 | 5.18672 | 9.28949 | 2.30E-03 | 2.21E-02 |
| JPH1 | ----- | ENSGALG00000035845 | junctophilin 1 | 0.30 | 4.19089 | 9.89173 | 1.66E-03 | 1.74E-02 |
| PDZD2 | ----- | ENSGALG00000002854 | PDZ domain containing 2 | 0.30 | 5.38628 | 8.20712 | 4.17E-03 | 3.35E-02 |
| SALL1 | SAL1 | ENSGALG00000039238 | spalt like transcription factor 1 | 0.30 | 6.84150 | 9.46180 | 2.10E-03 | 2.08E-02 |
| TULP4 | ----- | ENSGALG00000037377 | tubby like protein 4 | 0.30 | 6.68813 | 16.49446 | 4.88E-05 | 1.17E-03 |
| SEC23A | ----- | ENSGALG00000010150 | Sec23 homolog A, coat complex II component | 0.30 | 6.75334 | 27.45438 | 1.61E-07 | 1.03E-05 |
| GALNT11 | ----- | ENSGALG00000006233 | polypeptide N-acetylgalactosaminyltransferase 11 | 0.30 | 5.66554 | 25.81165 | 3.76E-07 | 2.13E-05 |
| PPM1M | ----- | ENSGALG00000033277 | protein phosphatase, Mg2+/Mn2+ dependent 1M | 0.30 | 4.59023 | 12.36938 | 4.36E-04 | 6.54E-03 |

| | | | | | | | | |
|---|---|---|---|---|---|---|---|---|
| URB1 | - - - - - | ENSGALG00000015887 | URB1 ribosome biogenesis 1 homolog (S. cerevisiae) | 0.30 | 6.20122 | 14.39245 | 1.48E-04 | 2.81E-03 |
| CTDP1 | - - - - - | ENSGALG00000031461 | CTD phosphatase subunit 1 | 0.29 | 5.74594 | 16.91436 | 3.91E-05 | 9.77E-04 |
| IBA57 | C1orf69 | ENSGALG00000005328 | IBA57 homolog, iron-sulfur cluster assembly | 0.29 | 5.45724 | 16.91874 | 3.90E-05 | 9.77E-04 |
| MCPH1 | - - - - - | ENSGALG00000016332 | microcephalin 1 | 0.29 | 5.06891 | 10.39962 | 1.26E-03 | 1.41E-02 |
| PCCB | - - - - - | ENSGALG00000037238 | propionyl-CoA carboxylase beta subunit | 0.29 | 5.71387 | 16.01882 | 6.27E-05 | 1.44E-03 |
| RBKS | - - - - - | ENSGALG00000010043 | ribokinase | 0.29 | 4.63306 | 11.83503 | 5.81E-04 | 8.11E-03 |
| TUSC5 | - - - - - | ENSGALG00000047441 | tumor suppressor candidate 5 | 0.29 | 5.87151 | 8.82322 | 2.97E-03 | 2.63E-02 |
| IFT140 | - - - - - | ENSGALG00000009318 | intraflagellar transport 140 | 0.29 | 5.58583 | 18.07422 | 2.12E-05 | 5.90E-04 |
| EED | - - - - - | ENSGALG00000014060 | embryonic ectoderm development | 0.29 | 5.18224 | 16.24889 | 5.55E-05 | 1.31E-03 |
| PNPLA6 | NTE | ENSGALG00000039408 | patatin like phospholipase domain containing 6 | 0.29 | 4.89315 | 7.57541 | 5.92E-03 | 4.29E-02 |
| BID | - - - - - | ENSGALG00000013039 | BH3 interacting domain death agonist | 0.29 | 7.19257 | 25.97113 | 3.47E-07 | 1.99E-05 |
| HIP1 | - - - - - | ENSGALG00000040908 | huntingtin interacting protein 1 | 0.29 | 7.64223 | 16.52613 | 4.80E-05 | 1.15E-03 |
| SRSF4 | - - - - - | ENSGALG00000050314 | serine and arginine rich splicing factor 4 | 0.29 | 5.51476 | 10.05646 | 1.52E-03 | 1.63E-02 |
| LOC112532285 | | - - - - - | uncharacterized LOC112532285 | 0.29 | 4.54346 | 11.28328 | 7.82E-04 | 1.00E-02 |
| MRTO4 | - - - - - | ENSGALG00000003991 | MRT4 homolog, ribosome maturation factor | 0.29 | 6.50379 | 19.66267 | 9.24E-06 | 3.04E-04 |
| EPS8 | - - - - - | ENSGALG00000013091 | epidermal growth factor receptor pathway substrate 8 | 0.29 | 5.00849 | 9.07900 | 2.59E-03 | 2.38E-02 |
| DDX31 | - - - - - | ENSGALG00000041473 | DEAD-box helicase 31 | 0.29 | 5.63881 | 23.06701 | 1.56E-06 | 6.90E-05 |
| FKBP11 | - - - - - | ENSGALG00000035060 | FK506 binding protein 11 | 0.29 | 3.80476 | 9.00258 | 2.70E-03 | 2.45E-02 |
| ACAD11 | - - - - - | ENSGALG00000035949 | acyl-CoA dehydrogenase family member 11 | 0.29 | 5.08534 | 9.44017 | 2.12E-03 | 2.09E-02 |
| ATXN10 | - - - - - | ENSGALG00000014234 | ataxin 10 | 0.29 | 4.20060 | 12.62485 | 3.81E-04 | 5.90E-03 |
| TMEM135 | - - - - - | ENSGALG00000047269 | transmembrane protein 135 | 0.29 | 4.86787 | 10.81794 | 1.01E-03 | 1.21E-02 |
| RTEL1 | - - - - - | ENSGALG00000006123 | regulator of telomere elongation helicase 1 | 0.29 | 5.97506 | 15.19490 | 9.70E-05 | 2.02E-03 |
| SLC25A22 | - - - - - | ENSGALG00000039335 | solute carrier family 25 member 22 | 0.29 | 4.44918 | 11.54281 | 6.80E-04 | 9.09E-03 |
| CARNMT1 | - - - - - | ENSGALG00000015155 | carnosine N-methyltransferase 1 | 0.29 | 5.53329 | 7.48175 | 6.23E-03 | 4.47E-02 |
| XBP1 | - - - - - | ENSGALG00000005796 | X-box binding protein 1 | 0.29 | 6.47420 | 21.02511 | 4.53E-06 | 1.70E-04 |
| EIF2B4 | - - - - - | ENSGALG00000033575 | eukaryotic translation initiation factor 2B subunit delta | 0.29 | 5.86254 | 12.80309 | 3.46E-04 | 5.48E-03 |
| MYCBPAP | - - - - - | ENSGALG00000007661 | radical S-adenosyl methionine domain containing 1 | 0.28 | 4.81373 | 7.85339 | 5.07E-03 | 3.86E-02 |
| GALK2 | - - - - - | ENSGALG00000036201 | galactokinase 2 | 0.28 | 4.45339 | 7.54837 | 6.01E-03 | 4.34E-02 |
| SLC25A11 | - - - - - | ENSGALG00000029383 | solute carrier family 25 member 11 | 0.28 | 6.49162 | 15.22738 | 9.53E-05 | 2.00E-03 |
| EVC | DWF-1 | ENSGALG00000015037 | EvC ciliary complex subunit 1 | 0.28 | 4.93513 | 12.08868 | 5.07E-04 | 7.34E-03 |
| INTU | - - - - - | - - - - - | inturned planar cell polarity protein | 0.28 | 4.80982 | 10.56476 | 1.15E-03 | 1.33E-02 |
| TMSB15B | - - - - - | ENSGALG00000031593 | thymosin beta 15B | 0.28 | 7.29783 | 8.93136 | 2.80E-03 | 2.52E-02 |
| EPHA7 | CEPHA7 | ENSGALG00000015593 | EPH receptor A7 | 0.28 | 4.60149 | 8.33877 | 3.88E-03 | 3.18E-02 |
| EIF4G3 | - - - - - | ENSGALG00000010084 | eukaryotic translation initiation factor 4 gamma 3 | 0.28 | 7.84866 | 15.63462 | 7.68E-05 | 1.70E-03 |
| FASTKD1 | - - - - - | ENSGALG00000009841 | FAST kinase domains 1 | 0.28 | 4.44420 | 11.35756 | 7.51E-04 | 9.75E-03 |
| NRG4 | - - - - - | ENSGALG00000002952 | neuregulin 4 | 0.28 | 3.72710 | 8.92899 | 2.81E-03 | 2.52E-02 |
| NNF1 | PMF1 | ENSGALG00000046965 | Nnf1 protein | 0.28 | 6.11553 | 9.05440 | 2.62E-03 | 2.40E-02 |
| QDPR | - - - - - | ENSGALG00000030070 | quinoid dihydropteridine reductase | 0.28 | 6.03060 | 13.73742 | 2.10E-04 | 3.72E-03 |
| KATNAL1 | - - - - - | ENSGALG00000017083 | katanin catalytic subunit A1 like 1 | 0.28 | 4.84051 | 15.03453 | 1.06E-04 | 2.15E-03 |
| JAKMIP2 | - - - - - | ENSGALG00000007593 | janus kinase and microtubule interacting protein 2 | 0.28 | 5.66008 | 14.40240 | 1.48E-04 | 2.80E-03 |
| COMMD6 | - - - - - | ENSGALG00000016922 | COMM domain containing 6 | 0.28 | 6.39939 | 15.87072 | 6.78E-05 | 1.54E-03 |
| FAM114A1 | - - - - - | ENSGALG00000013583 | family with sequence similarity 114 member A1 | 0.28 | 3.79904 | 7.64557 | 5.69E-03 | 4.18E-02 |
| C1H12orf29 | - - - - - | - - - - - | ----- | 0.28 | 4.85159 | 13.45244 | 2.45E-04 | 4.22E-03 |
| GRSF1 | - - - - - | ENSGALG00000031160 | G-rich RNA sequence binding factor 1 | 0.28 | 6.99229 | 17.82826 | 2.42E-05 | 6.58E-04 |

| | | | | | | | | | |
|---|---|---|---|---|---|---|---|---|---|
| APBA3 | - - - - - | ENSGALG00000000740 | amyloid beta (A4) precursor protein-binding, family A, member 3 | 0.28 | 5.11250 | 14.24789 | 1.60E-04 | 2.99E-03 |
| TMEFF2 | - - - - - | ENSGALG00000007759 | transmembrane protein with EGF like and two follistatin like domains 2 | 0.28 | 3.94017 | 8.38434 | 3.78E-03 | 3.13E-02 |
| USP21 | - - - - - | ENSGALG00000051012 | ubiquitin specific peptidase 21 | 0.28 | 4.86595 | 11.29001 | 7.79E-04 | 9.99E-03 |
| FIGNL1 | - - - - - | ENSGALG00000026363 | fidgetin like 1 | 0.28 | 4.65917 | 10.08524 | 1.49E-03 | 1.61E-02 |
| RFX3 | - - - - - | ENSGALG00000010179 | regulatory factor X3 | 0.28 | 5.79581 | 7.97792 | 4.74E-03 | 3.68E-02 |
| SEL1L3 | - - - - - | ENSGALG00000039549 | SEL1L family member 3 | 0.27 | 5.26640 | 15.97727 | 6.41E-05 | 1.47E-03 |
| ARL3 | - - - - - | ENSGALG00000008049 | ADP ribosylation factor like GTPase 3 | 0.27 | 7.03367 | 19.12333 | 1.23E-05 | 3.80E-04 |
| MRPS22 | - - - - - | ENSGALG00000005367 | mitochondrial ribosomal protein S22 | 0.27 | 5.71289 | 13.56196 | 2.31E-04 | 4.02E-03 |
| MDC1 | - - - - - | - - - - - | ----- | 0.27 | 6.82473 | 16.61085 | 4.59E-05 | 1.12E-03 |
| NUAK1 | - - - - - | ENSGALG00000012662 | NUAK family kinase 1 | 0.27 | 6.08246 | 11.83839 | 5.80E-04 | 8.10E-03 |
| GUF1 | - - - - - | ENSGALG00000014217 | GUF1 homolog, GTPase | 0.27 | 4.50261 | 7.61061 | 5.80E-03 | 4.23E-02 |
| CALML4 | - - - - - | ENSGALG00000007979 | calmodulin like 4 | 0.27 | 3.79979 | 7.29519 | 6.91E-03 | 4.79E-02 |
| DYM | - - - - - | ENSGALG00000037426 | dymeclin | 0.27 | 5.32546 | 11.18446 | 8.25E-04 | 1.05E-02 |
| CAMKK2 | - - - - - | ENSGALG00000040131 | calcium/calmodulin dependent protein kinase kinase 2 | 0.27 | 6.33650 | 11.12132 | 8.53E-04 | 1.07E-02 |
| CISD1 | ZCD1 | ENSGALG00000002705 | CDGSH iron sulfur domain 1 | 0.27 | 6.78014 | 24.16472 | 8.84E-07 | 4.34E-05 |
| B4GALT6 | - - - - - | ENSGALG00000015151 | beta-1,4-galactosyltransferase 6 | 0.27 | 5.18628 | 10.08057 | 1.50E-03 | 1.62E-02 |
| TARS | - - - - - | ENSGALG00000003288 | threonyl-tRNA synthetase | 0.27 | 7.33680 | 8.98594 | 2.72E-03 | 2.46E-02 |
| PARP9 | - - - - - | ENSGALG00000045511 | poly(ADP-ribose) polymerase family member 9 | 0.27 | 3.94174 | 8.84422 | 2.94E-03 | 2.61E-02 |
| ENO1 | - - - - - | ENSGALG00000002377 | enolase 1, (alpha) | 0.27 | 11.20888 | 8.74295 | 3.11E-03 | 2.71E-02 |
| ABCC6 | - - - - - | ENSGALG00000038152 | ATP binding cassette subfamily C member 6 | 0.27 | 4.21813 | 7.74100 | 5.40E-03 | 4.03E-02 |
| PLEKHB1 | - - - - - | ENSGALG00000017321 | pleckstrin homology domain containing B1 | 0.27 | 4.64795 | 8.40456 | 3.74E-03 | 3.10E-02 |
| CHST13 | - - - - - | ENSGALG00000029632 | carbohydrate sulfotransferase 13 | 0.27 | 5.52157 | 10.74483 | 1.05E-03 | 1.24E-02 |
| BCAS3 | - - - - - | ENSGALG00000005295 | BCAS3, microtubule associated cell migration factor | 0.27 | 4.97406 | 8.92893 | 2.81E-03 | 2.52E-02 |
| DACH1 | DACH | ENSGALG00000037015 | dachshund family transcription factor 1 | 0.27 | 5.22275 | 10.34386 | 1.30E-03 | 1.45E-02 |
| PSRC1 | - - - - - | - - - - - | proline and serine rich coiled-coil 1 | 0.27 | 5.22166 | 15.16369 | 9.86E-05 | 2.04E-03 |
| LZTR1 | - - - - - | ENSGALG00000031175 | leucine zipper like transcription regulator 1 | 0.27 | 5.68614 | 20.52297 | 5.89E-06 | 2.12E-04 |
| FAM53B | - - - - - | ENSGALG00000009718 | family with sequence similarity 53 member B | 0.27 | 5.37546 | 7.97274 | 4.75E-03 | 3.69E-02 |
| ELP2 | - - - - - | ENSGALG00000039034 | elongator acetyltransferase complex subunit 2 | 0.27 | 5.18873 | 15.54207 | 8.07E-05 | 1.76E-03 |
| SLC25A4 | - - - - - | ENSGALG00000010614 | solute carrier family 25 member 4 | 0.27 | 5.91756 | 10.58525 | 1.14E-03 | 1.32E-02 |
| LOC107052085 | | - - - - - | uncharacterized LOC107052085 | 0.27 | 5.28063 | 8.21981 | 4.14E-03 | 3.33E-02 |
| TRMT10A | - - - - - | ENSGALG00000012264 | tRNA methyltransferase 10A | 0.27 | 4.17803 | 8.41254 | 3.73E-03 | 3.10E-02 |
| CTSZ | - - - - - | ENSGALG00000007462 | cathepsin Z | 0.27 | 5.29071 | 13.07762 | 2.99E-04 | 4.91E-03 |
| MRPS14 | - - - - - | ENSGALG00000030028 | mitochondrial ribosomal protein S14 | 0.27 | 5.20393 | 10.43333 | 1.24E-03 | 1.40E-02 |
| CCDC58 | - - - - - | ENSGALG00000014413 | coiled-coil domain containing 58 | 0.26 | 4.80765 | 7.63590 | 5.72E-03 | 4.19E-02 |
| YY2 | NF-E1 | ENSGALG00000017388 | YY2 transcription factor | 0.26 | 6.54108 | 19.79913 | 8.60E-06 | 2.91E-04 |
| PC | PCX (retired) | ENSGALG00000053095 | pyruvate carboxylase | 0.26 | 5.11775 | 8.20806 | 4.17E-03 | 3.35E-02 |
| PSMA4 | - - - - - | ENSGALG00000003046 | proteasome subunit alpha 4 | 0.26 | 7.64828 | 19.30544 | 1.11E-05 | 3.57E-04 |
| LMO2 | - - - - - | ENSGALG00000029892 | LIM domain only 2 | 0.26 | 6.03394 | 10.68087 | 1.08E-03 | 1.27E-02 |
| FKBP5 | FKBP51 | ENSGALG00000042148 | FK506 binding protein 5 | 0.26 | 5.39740 | 12.66841 | 3.72E-04 | 5.79E-03 |
| MRS2 | - - - - - | ENSGALG00000040084 | MRS2, magnesium transporter | 0.26 | 4.24320 | 8.68971 | 3.20E-03 | 2.76E-02 |
| EHBP1L1 | - - - - - | - - - - - | EH domain binding protein 1 like 1 | 0.26 | 3.96693 | 7.36309 | 6.66E-03 | 4.67E-02 |
| NKAIN4 | - - - - - | ENSGALG00000005774 | Na+/K+ transporting ATPase interacting 4 | 0.26 | 5.44718 | 7.57230 | 5.93E-03 | 4.30E-02 |
| KEAP1 | - - - - - | - - - - - | ----- | 0.26 | 5.53265 | 7.95492 | 4.80E-03 | 3.72E-02 |
| TMEM18 | - - - - - | ENSGALG00000028744 | transmembrane protein 18 | 0.26 | 4.89895 | 9.33993 | 2.24E-03 | 2.17E-02 |

| | | | | | | | | |
|---|---|---|---|---|---|---|---|---|
| FANCI | - - - - - | ENSGALG00000006658 | Fanconi anemia complementation group I | 0.26 | 5.77614 | 15.72413 | 7.33E-05 | 1.63E-03 |
| MYL9 | DTNB | ENSGALG00000028567 | myosin, light chain 9, regulatory | 0.26 | 6.79380 | 13.80296 | 2.03E-04 | 3.62E-03 |
| PASK | - - - - - | ENSGALG00000006016 | PAS domain containing serine/threonine kinase | 0.26 | 5.02959 | 10.50039 | 1.19E-03 | 1.36E-02 |
| TSPAN13 | tetraspanin-13 | ENSGALG00000027240 | tetraspanin 13 | 0.26 | 5.54985 | 13.78103 | 2.05E-04 | 3.65E-03 |
| SPDL1 | - - - - - | ENSGALG00000001942 | spindle apparatus coiled-coil protein 1 | 0.26 | 6.01354 | 22.74504 | 1.85E-06 | 7.89E-05 |
| CENPH | CENP-H | ENSGALG00000014788 | centromere protein H | 0.26 | 6.42928 | 7.87024 | 5.03E-03 | 3.83E-02 |
| TTLL4 | - - - - - | ENSGALG00000027991 | tubulin tyrosine ligase like 4 | 0.26 | 4.93779 | 10.01213 | 1.56E-03 | 1.66E-02 |
| PRMT7 | - - - - - | ENSGALG00000038865 | protein arginine methyltransferase 7 | 0.26 | 5.24857 | 9.49463 | 2.06E-03 | 2.06E-02 |
| ORC6 | ORC6L | ENSGALG00000004342 | origin recognition complex subunit 6 | 0.26 | 5.78619 | 10.90512 | 9.59E-04 | 1.17E-02 |
| CBY1 | - - - - - | ENSGALG00000012218 | chibby family member 1, beta catenin antagonist | 0.26 | 4.92294 | 7.32573 | 6.80E-03 | 4.73E-02 |
| LOC424740 | | - - - - - | uncharacterized LOC424740 | 0.26 | 7.51417 | 12.07009 | 5.12E-04 | 7.39E-03 |
| SEM1 | - - - - - | - - - - - | ----- | 0.26 | 6.98687 | 10.11063 | 1.47E-03 | 1.60E-02 |
| AUH | - - - - - | ENSGALG00000021843 | AU RNA binding methylglutaconyl-CoA hydratase | 0.26 | 5.02778 | 8.84502 | 2.94E-03 | 2.61E-02 |
| PCDH1 | - - - - - | ENSGALG00000002553 | protocadherin 1 | 0.26 | 6.08777 | 8.97526 | 2.74E-03 | 2.47E-02 |
| HSPA4L | - - - - - | ENSGALG00000010185 | heat shock protein family A (Hsp70) member 4 like | 0.26 | 7.28681 | 14.40789 | 1.47E-04 | 2.80E-03 |
| HOXB7 | - - - - - | ENSGALG00000032740 | homeobox B7 | 0.26 | 6.71775 | 13.12439 | 2.91E-04 | 4.83E-03 |
| MAPKAPK2 | - - - - - | ENSGALG00000000883 | mitogen-activated protein kinase-activated protein kinase 2 | 0.26 | 6.94108 | 23.87964 | 1.03E-06 | 4.87E-05 |
| USP16 | - - - - - | ENSGALG00000015813 | ubiquitin specific peptidase 16 | 0.26 | 6.56619 | 17.82951 | 2.42E-05 | 6.58E-04 |
| PPID | - - - - - | ENSGALG00000009451 | peptidylprolyl isomerase D | 0.26 | 6.41249 | 17.33254 | 3.14E-05 | 8.14E-04 |
| BBS9 | - - - - - | ENSGALG00000031650 | Bardet-Biedl syndrome 9 | 0.25 | 4.99322 | 10.59282 | 1.14E-03 | 1.32E-02 |
| PPOX | - - - - - | ENSGALG00000054130 | protoporphyrinogen oxidase | 0.25 | 5.17291 | 9.32970 | 2.25E-03 | 2.18E-02 |
| RGMA | - - - - - | ENSGALG00000039173 | repulsive guidance molecule family member a | 0.25 | 6.57980 | 8.27425 | 4.02E-03 | 3.26E-02 |
| ZNF704 | - - - - - | ENSGALG00000030011 | zinc finger protein 704 | 0.25 | 5.05163 | 7.55260 | 5.99E-03 | 4.33E-02 |
| DYNLT1 | - - - - - | ENSGALG00000019979 | dynein light chain Tctex-type 1 | 0.25 | 5.47765 | 15.42414 | 8.59E-05 | 1.84E-03 |
| DCK | - - - - - | ENSGALG00000028501 | deoxycytidine kinase | 0.25 | 5.79197 | 16.84257 | 4.06E-05 | 1.01E-03 |
| PHB | - - - - - | ENSGALG00000038604 | prohibitin | 0.25 | 6.39268 | 17.63993 | 2.67E-05 | 7.15E-04 |
| MTERF3 | MTERFD1 | ENSGALG00000040511 | mitochondrial transcription termination factor 3 | 0.25 | 5.49501 | 8.08314 | 4.47E-03 | 3.52E-02 |
| DLL1 | - - - - - | ENSGALG00000011182 | delta like canonical Notch ligand 1 | 0.25 | 5.62670 | 7.29027 | 6.93E-03 | 4.80E-02 |
| CDC34 | - - - - - | ENSGALG00000024332 | cell division cycle 34 | 0.25 | 5.04676 | 9.08932 | 2.57E-03 | 2.37E-02 |
| CHCHD6 | - - - - - | ENSGALG00000006056 | coiled-coil-helix-coiled-coil-helix domain containing 6 | 0.25 | 5.65915 | 11.24535 | 7.98E-04 | 1.02E-02 |
| TRMT5 | - - - - - | ENSGALG00000011904 | tRNA methyltransferase 5 | 0.25 | 4.90593 | 10.74592 | 1.05E-03 | 1.24E-02 |
| MOB3A | - - - - - | ENSGALG00000031729 | MOB kinase activator 3A | 0.25 | 5.48461 | 9.66688 | 1.88E-03 | 1.92E-02 |
| CNN3 | calponin-3 | ENSGALG00000005597 | calponin 3 | 0.25 | 8.54193 | 23.12355 | 1.52E-06 | 6.74E-05 |
| LOC107049885 | | - - - - - | M-phase inducer phosphatase 2-like | 0.25 | 7.43478 | 13.83340 | 2.00E-04 | 3.57E-03 |
| LOC101748569 | | - - - - - | caseinolytic peptidase B protein homolog | 0.25 | 4.15230 | 9.15219 | 2.48E-03 | 2.31E-02 |
| SMC6 | SMC6L1 | ENSGALG00000016467 | structural maintenance of chromosomes 6 | 0.25 | 5.95176 | 14.54530 | 1.37E-04 | 2.63E-03 |
| STK38L | - - - - - | ENSGALG00000014094 | serine/threonine kinase 38 like | 0.25 | 5.06041 | 9.18272 | 2.44E-03 | 2.29E-02 |
| ALG12 | 6-mannosyltransferase) | ENSGALG00000008539 | ALG12, alpha-1,6-mannosyltransferase | 0.25 | 7.07042 | 17.79264 | 2.46E-05 | 6.68E-04 |
| NSMAF | - - - - - | ENSGALG00000038838 | neutral sphingomyelinase activation associated factor | 0.25 | 5.60782 | 13.26079 | 2.71E-04 | 4.58E-03 |
| TMEM62 | - - - - - | ENSGALG00000009260 | transmembrane protein 62 | 0.25 | 4.50505 | 7.26516 | 7.03E-03 | 4.84E-02 |
| CCT4 | - - - - - | ENSGALG00000008812 | chaperonin containing TCP1 subunit 4 | 0.25 | 8.99825 | 15.70785 | 7.39E-05 | 1.64E-03 |
| H2A2BL | - - - - - | - - - - - | ----- | 0.25 | 6.51745 | 10.06324 | 1.51E-03 | 1.63E-02 |
| NUP188 | - - - - - | ENSGALG00000043394 | nucleoporin 188 | 0.25 | 7.51489 | 15.46722 | 8.39E-05 | 1.81E-03 |
| PAWR | - - - - - | ENSGALG00000010320 | pro-apoptotic WT1 regulator | 0.25 | 6.39826 | 8.71200 | 3.16E-03 | 2.74E-02 |

| | | | | | | | | |
|---|---|---|---|---|---|---|---|---|
| SEPSECS | - - - - - | ENSGALG00000014386 | Sep (O-phosphoserine) tRNA:Sec (selenocysteine) tRNA synthase | 0.25 | 5.42836 | 11.08038 | 8.72E-04 | 1.09E-02 |
| TAB2 | - - - - - | ENSGALG00000012356 | TGF-beta activated kinase 1/MAP3K7 binding protein 2 | 0.25 | 6.91616 | 15.18081 | 9.77E-05 | 2.03E-03 |
| PIGH | - - - - - | ENSGALG00000009510 | phosphatidylinositol glycan anchor biosynthesis class H | 0.25 | 4.98004 | 9.63057 | 1.91E-03 | 1.95E-02 |
| ACTN4 | - - - - - | ENSGALG00000036496 | actinin, alpha 4 | 0.25 | 8.20983 | 13.57042 | 2.30E-04 | 4.01E-03 |
| PXN | - - - - - | - - - - - | ----- | 0.25 | 6.26703 | 11.42452 | 7.25E-04 | 9.52E-03 |
| MIS12 | - - - - - | - - - - - | MIS12, kinetochore complex component | 0.25 | 4.24411 | 7.60044 | 5.84E-03 | 4.25E-02 |
| ATP5G1 | - - - - - | ENSGALG00000001330 | ATP synthase, H+ transporting, mitochondrial Fo complex subunit C1 (subunit 9) | 0.25 | 7.32672 | 14.29752 | 1.56E-04 | 2.92E-03 |
| WDR55 | - - - - - | ENSGALG00000029199 | WD repeat domain 55 | 0.25 | 6.17020 | 10.47577 | 1.21E-03 | 1.38E-02 |
| FAM126A | - - - - - | ENSGALG00000010925 | family with sequence similarity 126 member A | 0.25 | 6.85737 | 19.64713 | 9.31E-06 | 3.06E-04 |
| LOXL3 | - - - - - | ENSGALG00000036142 | lysyl oxidase like 3 | 0.25 | 5.36065 | 7.40966 | 6.49E-03 | 4.60E-02 |
| RTFDC1 | C20H20ORF43 | ENSGALG00000007709 | replication termination factor 2 domain containing 1 | 0.24 | 6.42555 | 17.37972 | 3.06E-05 | 7.97E-04 |
| TLE1 | - - - - - | ENSGALG00000012575 | transducin like enhancer of split 1 | 0.24 | 5.92589 | 8.54319 | 3.47E-03 | 2.93E-02 |
| GLRX3 | TXNL2 | ENSGALG00000010464 | glutaredoxin 3 | 0.24 | 7.12783 | 15.57941 | 7.91E-05 | 1.74E-03 |
| LRRC28 | - - - - - | ENSGALG00000007057 | leucine rich repeat containing 28 | 0.24 | 5.20778 | 13.39970 | 2.52E-04 | 4.32E-03 |
| LARS | - - - - - | ENSGALG00000007494 | leucyl-tRNA synthetase | 0.24 | 7.51089 | 22.93560 | 1.68E-06 | 7.27E-05 |
| PLAA | - - - - - | ENSGALG00000001782 | phospholipase A2 activating protein | 0.24 | 6.31391 | 9.22432 | 2.39E-03 | 2.26E-02 |
| MRPS10 | - - - - - | ENSGALG00000009890 | mitochondrial ribosomal protein S10 | 0.24 | 4.95445 | 8.41252 | 3.73E-03 | 3.10E-02 |
| NTMT1 | AD-003 | ENSGALG00000017611 | N-terminal Xaa-Pro-Lys N-methyltransferase 1 | 0.24 | 5.96174 | 9.90844 | 1.65E-03 | 1.73E-02 |
| RPA3 | - - - - - | ENSGALG00000010700 | replication protein A3 | 0.24 | 5.88040 | 12.04083 | 5.20E-04 | 7.47E-03 |
| PHF14 | - - - - - | ENSGALG00000038041 | PHD finger protein 14 | 0.24 | 6.84283 | 13.25160 | 2.72E-04 | 4.59E-03 |
| GREB1L | - - - - - | ENSGALG00000014935 | GREB1 like retinoic acid receptor coactivator | 0.24 | 7.00002 | 8.01831 | 4.63E-03 | 3.62E-02 |
| MRPS17 | - - - - - | ENSGALG00000002379 | mitochondrial ribosomal protein S17 | 0.24 | 6.23440 | 11.94654 | 5.47E-04 | 7.77E-03 |
| DRAM2 | - - - - - | ENSGALG00000036491 | DNA damage regulated autophagy modulator 2 | 0.24 | 4.34734 | 9.14914 | 2.49E-03 | 2.32E-02 |
| RECQL5 | - - - - - | ENSGALG00000002424 | RecQ like helicase 5 | 0.24 | 5.19822 | 7.49279 | 6.19E-03 | 4.45E-02 |
| GPATCH11 | CCDC75 | ENSGALG00000010561 | G-patch domain containing 11 | 0.24 | 5.23341 | 10.15944 | 1.44E-03 | 1.57E-02 |
| EOGT | AER61 | ENSGALG00000013406 | EGF domain specific O-linked N-acetylglucosamine transferase | 0.24 | 5.03937 | 8.82784 | 2.97E-03 | 2.62E-02 |
| ACO2 | - - - - - | ENSGALG00000011950 | aconitase 2 | 0.24 | 7.98686 | 15.40480 | 8.68E-05 | 1.85E-03 |
| KNTC1 | - - - - - | - - - - - | kinetochore associated 1 | 0.24 | 6.71099 | 11.41802 | 7.27E-04 | 9.52E-03 |
| NFYB | - - - - - | ENSGALG00000041193 | Nuclear transcription factor Y subunit beta D346 | 0.24 | 5.32316 | 7.24613 | 7.11E-03 | 4.87E-02 |
| FAM120B | - - - - - | ENSGALG00000011180 | family with sequence similarity 120B | 0.24 | 4.69085 | 7.55859 | 5.97E-03 | 4.33E-02 |
| NAT10 | - - - - - | ENSGALG00000014573 | N-acetyltransferase 10 | 0.24 | 7.13831 | 12.75044 | 3.56E-04 | 5.59E-03 |
| CSRNP3 | - - - - - | ENSGALG00000010971 | cysteine and serine rich nuclear protein 3 | 0.24 | 4.10734 | 7.92963 | 4.86E-03 | 3.75E-02 |
| UBB | polyubiquitin-C | ENSGALG00000041277 | ubiquitin B | 0.24 | 9.35011 | 7.30022 | 6.89E-03 | 4.78E-02 |
| CAPN10 | - - - - - | ENSGALG00000034562 | calpain 10 | 0.24 | 5.86666 | 11.73247 | 6.14E-04 | 8.44E-03 |
| EHD4 | - - - - - | ENSGALG00000008950 | EH domain containing 4 | 0.24 | 4.76549 | 9.24150 | 2.37E-03 | 2.25E-02 |
| LARP1B | - - - - - | ENSGALG00000010207 | La ribonucleoprotein domain family member 1B | 0.24 | 4.92840 | 7.36102 | 6.67E-03 | 4.67E-02 |
| FAAH | - - - - - | ENSGALG00000010433 | fatty acid amide hydrolase | 0.24 | 4.70069 | 8.18925 | 4.21E-03 | 3.38E-02 |
| LOC107050659 | | - - - - - | valine--tRNA ligase-like | 0.24 | 5.38592 | 8.51512 | 3.52E-03 | 2.97E-02 |
| ANKRD46 | - - - - - | ENSGALG00000041445 | ankyrin repeat domain 46 | 0.24 | 6.31290 | 17.56495 | 2.78E-05 | 7.34E-04 |
| DIRC2 | - - - - - | - - - - - | disrupted in renal carcinoma 2 | 0.24 | 4.64376 | 8.85346 | 2.93E-03 | 2.60E-02 |
| SDF2L1 | - - - - - | ENSGALG00000031982 | stromal cell derived factor 2 like 1 | 0.24 | 5.79707 | 10.11129 | 1.47E-03 | 1.60E-02 |
| FAM63A | - - - - - | ENSGALG00000028499 | family with sequence similarity 63 member A | 0.24 | 6.50941 | 13.60696 | 2.25E-04 | 3.94E-03 |
| TMEM14C | - - - - - | ENSGALG00000034591 | transmembrane protein 14C | 0.24 | 5.47265 | 7.35203 | 6.70E-03 | 4.69E-02 |
| ACTR10 | - - - - - | ENSGALG00000012041 | actin-related protein 10 homolog | 0.24 | 6.07869 | 18.53481 | 1.67E-05 | 4.88E-04 |

| | | | | | | | | | |
|---|---|---|---|---|---|---|---|---|---|
| NDE1 | ----- | ENSGALG00000032590 | nudE neurodevelopment protein 1 | 0.24 | 6.06873 | 11.89479 | 5.63E-04 | 7.90E-03 |
| PRKG1 | ----- | ----- | protein kinase, cGMP-dependent, type I | 0.24 | 6.42627 | 8.40007 | 3.75E-03 | 3.11E-02 |
| C2CD2 | ----- | ENSGALG00000016148 | C2 calcium dependent domain containing 2 | 0.24 | 6.08293 | 16.19396 | 5.72E-05 | 1.33E-03 |
| SPICE1 | ----- | ENSGALG00000045606 | spindle and centriole associated protein 1 | 0.24 | 5.90207 | 15.46307 | 8.41E-05 | 1.81E-03 |
| PACSIN2 | FAP52 | ENSGALG00000014159 | protein kinase C and casein kinase substrate in neurons 2 | 0.24 | 5.72071 | 15.68334 | 7.49E-05 | 1.66E-03 |
| SMUG1 | ----- | ENSGALG00000053068 | single-strand-selective monofunctional uracil-DNA glycosylase 1 | 0.24 | 6.06992 | 9.18621 | 2.44E-03 | 2.29E-02 |
| TRMT6 | ----- | ENSGALG00000009169 | tRNA methyltransferase 6 | 0.24 | 5.53981 | 11.46227 | 7.10E-04 | 9.37E-03 |
| ALDH18A1 | ----- | ENSGALG00000034655 | aldehyde dehydrogenase 18 family member A1 | 0.23 | 6.44183 | 10.80583 | 1.01E-03 | 1.21E-02 |
| PPWD1 | ----- | ENSGALG00000014756 | peptidylprolyl isomerase domain and WD repeat containing 1 | 0.23 | 6.19289 | 7.41180 | 6.48E-03 | 4.60E-02 |
| NEPRO | ----- | ENSGALG00000015182 | nucleolus and neural progenitor protein | 0.23 | 4.74453 | 8.44806 | 3.65E-03 | 3.06E-02 |
| LAMTOR1 | ----- | ENSGALG00000050119 | late endosomal/lysosomal adaptor, MAPK and MTOR activator 1 | 0.23 | 5.60330 | 9.31964 | 2.27E-03 | 2.19E-02 |
| ADAR | ----- | ENSGALG00000021475 | adenosine deaminase, RNA specific | 0.23 | 6.68016 | 21.26386 | 4.00E-06 | 1.53E-04 |
| CENPE | ----- | ENSGALG00000013208 | centromere protein E | 0.23 | 7.40760 | 17.81938 | 2.43E-05 | 6.60E-04 |
| DTD1 | ----- | ENSGALG00000008808 | D-tyrosyl-tRNA deacylase 1 | 0.23 | 5.64118 | 12.40037 | 4.29E-04 | 6.46E-03 |
| HEXIM1 | ----- | ENSGALG00000041926 | HEXIM P-TEFb complex subunit 1 | 0.23 | 6.85336 | 11.14049 | 8.45E-04 | 1.06E-02 |
| TIPIN | ----- | ENSGALG00000007649 | TIMELESS interacting protein | 0.23 | 5.85431 | 10.20105 | 1.40E-03 | 1.54E-02 |
| C18H17orf62 | CYBC1 | ENSGALG00000001607 | chromosome 18 C17orf62 homolog | 0.23 | 6.15960 | 11.51891 | 6.89E-04 | 9.17E-03 |
| CDKL5 | ----- | ENSGALG00000016529 | cyclin dependent kinase like 5 | 0.23 | 4.61079 | 7.96454 | 4.77E-03 | 3.70E-02 |
| POT1 | ----- | ENSGALG00000008740 | protection of telomeres 1 | 0.23 | 5.16395 | 9.28783 | 2.31E-03 | 2.21E-02 |
| FBXO39 | ----- | ENSGALG00000029322 | F-box protein 39 | 0.23 | 5.00193 | 9.93794 | 1.62E-03 | 1.71E-02 |
| CLCC1 | ----- | ENSGALG00000002106 | chloride channel CLIC like 1 | 0.23 | 5.74986 | 9.61659 | 1.93E-03 | 1.96E-02 |
| CDK1 | CDC2 | ENSGALG00000003085 | cyclin dependent kinase 1 | 0.23 | 7.94959 | 10.85260 | 9.87E-04 | 1.19E-02 |
| ECHDC3 | ----- | ENSGALG00000006690 | enoyl-CoA hydratase domain containing 3 | 0.23 | 5.03755 | 9.85825 | 1.69E-03 | 1.77E-02 |
| BIN3 | ----- | ENSGALG00000052825 | bridging integrator 3 | 0.23 | 6.36570 | 11.69517 | 6.27E-04 | 8.54E-03 |
| RAD18 | ----- | ENSGALG00000008368 | RAD18, E3 ubiquitin protein ligase | 0.23 | 5.73937 | 10.82532 | 1.00E-03 | 1.20E-02 |
| CCND1 | ----- | ENSGALG00000007555 | cyclin D1 | 0.23 | 6.05195 | 7.55117 | 6.00E-03 | 4.33E-02 |
| NAPA | ----- | ENSGALG00000034747 | NSF attachment protein alpha | 0.23 | 6.75884 | 12.01171 | 5.29E-04 | 7.56E-03 |
| RTN4IP1 | ----- | ENSGALG00000015343 | reticulon 4 interacting protein 1 | 0.23 | 4.70930 | 7.90832 | 4.92E-03 | 3.79E-02 |
| TYRO3 | ----- | ----- | ----- | 0.23 | 6.28525 | 8.61790 | 3.33E-03 | 2.85E-02 |
| C14H16orf91 | ----- | ----- | chromosome 14 C16orf91 homolog | 0.23 | 4.40611 | 8.11083 | 4.40E-03 | 3.48E-02 |
| FAM149B1 | ----- | ENSGALG00000002693 | family with sequence similarity 149 member B1 | 0.23 | 6.32258 | 13.66306 | 2.19E-04 | 3.85E-03 |
| STRA6 | ----- | ENSGALG00000001449 | stimulated by retinoic acid 6 | 0.23 | 7.35311 | 7.85197 | 5.08E-03 | 3.86E-02 |
| COPB2 | ----- | ENSGALG00000005357 | coatomer protein complex subunit beta 2 | 0.23 | 7.98743 | 20.01227 | 7.69E-06 | 2.64E-04 |
| ATM | ----- | ENSGALG00000017159 | ATM serine/threonine kinase | 0.23 | 6.05522 | 11.37772 | 7.43E-04 | 9.68E-03 |
| HYPK | ----- | ENSGALG00000013916 | huntingtin interacting protein K | 0.23 | 5.76116 | 11.80249 | 5.92E-04 | 8.19E-03 |
| CTNND2 | ----- | ----- | catenin delta 2 | 0.23 | 6.08532 | 11.06677 | 8.79E-04 | 1.09E-02 |
| CEP83 | ----- | ENSGALG00000019336 | centrosomal protein 83 | 0.23 | 4.82223 | 8.40399 | 3.74E-03 | 3.10E-02 |
| C2H18ORF8 | ----- | ----- | ----- | 0.23 | 4.82235 | 8.18510 | 4.22E-03 | 3.38E-02 |
| IPO13 | importin-13 | ENSGALG00000010087 | importin 13 | 0.23 | 6.29662 | 9.73664 | 1.81E-03 | 1.86E-02 |
| BUB1B | BUBR1 | ENSGALG00000036892 | BUB1 mitotic checkpoint serine/threonine kinase B | 0.23 | 7.79571 | 9.99985 | 1.57E-03 | 1.67E-02 |
| FUNDC2 | ----- | ENSGALG00000029585 | FUN14 domain containing 2 | 0.23 | 6.48786 | 21.21341 | 4.11E-06 | 1.56E-04 |
| FKBP4 | ----- | ENSGALG00000014331 | FK506 binding protein 4 | 0.23 | 8.64115 | 13.92515 | 1.90E-04 | 3.44E-03 |
| BAG3 | ----- | ENSGALG00000009433 | BCL2 associated athanogene 3 | 0.23 | 6.13865 | 11.17045 | 8.31E-04 | 1.05E-02 |
| TSFM | ----- | ----- | ----- | 0.23 | 4.53471 | 8.77979 | 3.05E-03 | 2.67E-02 |

| | | | | | | | | |
|---|---|---|---|---|---|---|---|---|
| WWC1 | - - - - - | ENSGALG00000001826 | WW and C2 domain containing 1 | 0.23 | 6.27476 | 14.34219 | 1.52E-04 | 2.86E-03 |
| VAPA | - - - - - | ENSGALG00000039455 | VAMP associated protein A | 0.22 | 7.27048 | 18.00004 | 2.21E-05 | 6.12E-04 |
| ESD | - - - - - | ENSGALG00000016991 | esterase D | 0.22 | 6.36058 | 12.13661 | 4.94E-04 | 7.21E-03 |
| CPSF3 | - - - - - | ENSGALG00000016424 | cleavage and polyadenylation specific factor 3 | 0.22 | 6.56983 | 14.39730 | 1.48E-04 | 2.81E-03 |
| ALG8 | - - - - - | ENSGALG00000019042 | ALG8, alpha-1,3-glucosyltransferase | 0.22 | 5.55924 | 10.06046 | 1.51E-03 | 1.63E-02 |
| PAPSS1 | - - - - - | ENSGALG00000010542 | 3'-phosphoadenosine 5'-phosphosulfate synthase 1 | 0.22 | 6.98528 | 11.03428 | 8.94E-04 | 1.10E-02 |
| INTS11 | CPSF3L | ENSGALG00000001691 | integrator complex subunit 11 | 0.22 | 6.11686 | 12.77871 | 3.51E-04 | 5.53E-03 |
| MBD4 | - - - - - | ENSGALG00000020747 | methyl-CpG binding domain 4, DNA glycosylase | 0.22 | 4.65172 | 7.87089 | 5.02E-03 | 3.83E-02 |
| NRP2 | neuropilin-2 | ENSGALG00000008621 | neuropilin 2 | 0.22 | 8.71159 | 8.38615 | 3.78E-03 | 3.12E-02 |
| TMEM43 | - - - - - | ENSGALG00000006345 | transmembrane protein 43 | 0.22 | 5.70904 | 10.41066 | 1.25E-03 | 1.41E-02 |
| GUCY1B3 | - - - - - | ENSGALG00000009361 | guanylate cyclase 1 soluble subunit beta | 0.22 | 5.06100 | 9.03299 | 2.65E-03 | 2.42E-02 |
| DIAPH3 | - - - - - | ENSGALG00000016937 | diaphanous related formin 3 | 0.22 | 4.92897 | 7.73639 | 5.41E-03 | 4.03E-02 |
| TRIP13 | - - - - - | ENSGALG00000033834 | thyroid hormone receptor interactor 13 | 0.22 | 5.70814 | 8.69989 | 3.18E-03 | 2.75E-02 |
| CRCP | RCP9 | ENSGALG00000002587 | CGRP receptor component | 0.22 | 5.69892 | 9.65500 | 1.89E-03 | 1.93E-02 |
| MPV17L2 | - - - - - | ENSGALG00000041429 | MPV17 mitochondrial inner membrane protein like 2 | 0.22 | 4.89425 | 7.70618 | 5.50E-03 | 4.08E-02 |
| CDYL | - - - - - | - - - - - | chromodomain Y-like | 0.22 | 5.31184 | 7.64459 | 5.69E-03 | 4.18E-02 |
| UACA | - - - - - | ENSGALG00000034100 | uveal autoantigen with coiled-coil domains and ankyrin repeats | 0.22 | 6.85444 | 13.84665 | 1.98E-04 | 3.55E-03 |
| DACH2 | - - - - - | ENSGALG00000006886 | dachshund family transcription factor 2 | 0.22 | 6.11926 | 7.38523 | 6.58E-03 | 4.64E-02 |
| SNN | - - - - - | ENSGALG00000028876 | stannin | 0.22 | 4.77734 | 9.23022 | 2.38E-03 | 2.26E-02 |
| SIVA1 | - - - - - | ENSGALG00000011619 | SIVA1 apoptosis inducing factor | 0.22 | 4.79301 | 8.55840 | 3.44E-03 | 2.91E-02 |
| TTC37 | - - - - - | ENSGALG00000014670 | tetratricopeptide repeat domain 37 | 0.22 | 6.65755 | 7.33943 | 6.75E-03 | 4.71E-02 |
| KNSTRN | C15ORF23 | ENSGALG00000039576 | kinetochore-localized astrin/SPAG5 binding protein | 0.22 | 6.34051 | 12.05803 | 5.16E-04 | 7.41E-03 |
| WDHD1 | - - - - - | ENSGALG00000012199 | WD repeat and HMG-box DNA binding protein 1 | 0.22 | 6.42327 | 12.15840 | 4.89E-04 | 7.15E-03 |
| POLR1E | - - - - - | ENSGALG00000005395 | RNA polymerase I subunit E | 0.22 | 6.23700 | 9.32158 | 2.26E-03 | 2.18E-02 |
| C3H6ORF203 | C6orf203 | ENSGALG00000015316 | chromosome 3 open reading frame, human C6orf203 | 0.22 | 4.83158 | 7.40192 | 6.52E-03 | 4.61E-02 |
| COMMD10 | - - - - - | ENSGALG00000002308 | COMM domain containing 10 | 0.22 | 5.80969 | 7.61772 | 5.78E-03 | 4.22E-02 |
| RNGTT | - - - - - | ENSGALG00000015787 | RNA guanylyltransferase and 5'-phosphatase | 0.21 | 5.99327 | 8.74673 | 3.10E-03 | 2.70E-02 |
| HMCES | C12H3ORF37 | ENSGALG00000005897 | 5-hydroxymethylcytosine (hmC) binding, ES cell-specific | 0.21 | 5.65425 | 8.14280 | 4.32E-03 | 3.43E-02 |
| ECE1 | ECE-1 | ENSGALG00000031343 | endothelin converting enzyme 1 | 0.21 | 6.79756 | 11.90609 | 5.60E-04 | 7.88E-03 |
| STT3A | - - - - - | ENSGALG00000042730 | STT3A, catalytic subunit of the oligosaccharyltransferase complex | 0.21 | 7.61485 | 19.15268 | 1.21E-05 | 3.76E-04 |
| FIP1L1 | - - - - - | ENSGALG00000013943 | factor interacting with PAPOLA and CPSF1 | 0.21 | 6.20385 | 11.32778 | 7.64E-04 | 9.85E-03 |
| GPHN | - - - - - | ENSGALG00000009587 | gephyrin | 0.21 | 5.89127 | 10.43515 | 1.24E-03 | 1.40E-02 |
| RBM12B | - - - - - | ENSGALG00000035129 | RNA binding motif protein 12B | 0.21 | 6.57951 | 15.60808 | 7.79E-05 | 1.72E-03 |
| CCT3 | - - - - - | ENSGALG00000048034 | chaperonin containing TCP1 subunit 3 | 0.21 | 7.46963 | 8.26706 | 4.04E-03 | 3.27E-02 |
| STK16 | - - - - - | ENSGALG00000011326 | serine/threonine kinase 16 | 0.21 | 4.99177 | 7.33773 | 6.75E-03 | 4.71E-02 |
| GNL3 | - - - - - | ENSGALG00000001613 | G protein nucleolar 3 | 0.21 | 7.79089 | 11.24519 | 7.98E-04 | 1.02E-02 |
| PSIP1 | PSIP2 | ENSGALG00000015105 | PC4 and SFRS1 interacting protein 1 | 0.21 | 7.46882 | 8.79522 | 3.02E-03 | 2.65E-02 |
| MAP9 | - - - - - | ENSGALG00000020253 | microtubule associated protein 9 | 0.21 | 5.37132 | 8.82685 | 2.97E-03 | 2.62E-02 |
| SLITRK2 | - - - - - | ENSGALG00000009192 | SLIT and NTRK like family member 2 | 0.21 | 5.53252 | 12.57824 | 3.90E-04 | 6.01E-03 |
| STIP1 | - - - - - | ENSGALG00000046811 | stress induced phosphoprotein 1 | 0.21 | 7.99707 | 9.24821 | 2.36E-03 | 2.25E-02 |
| MYEF2 | - - - - - | ENSGALG00000032546 | myelin expression factor 2 | 0.21 | 7.46448 | 17.50186 | 2.87E-05 | 7.53E-04 |
| KIRREL | - - - - - | ENSGALG00000029528 | kin of IRRE like (Drosophila) | 0.21 | 8.45365 | 13.00736 | 3.10E-04 | 5.06E-03 |
| RRN3 | - - - - - | ENSGALG00000041989 | RRN3 homolog, RNA polymerase I transcription factor | 0.21 | 5.82923 | 10.63808 | 1.11E-03 | 1.29E-02 |
| GARS | - - - - - | ENSGALG00000005694 | glycyl-tRNA synthetase | 0.21 | 7.86267 | 15.21601 | 9.59E-05 | 2.00E-03 |

| | | | | | | | | |
|---|---|---|---|---|---|---|---|---|
| PPP4R2 | ----- | ENSGALG00000007810 | protein phosphatase 4 regulatory subunit 2 | 0.21 | 7.49860 | 17.85314 | 2.39E-05 | 6.52E-04 |
| WDCP | ----- | ENSGALG00000019867 | WD repeat and coiled coil containing | 0.21 | 5.36850 | 10.38836 | 1.27E-03 | 1.42E-02 |
| ZNF706 | ----- | ENSGALG00000041200 | zinc finger protein 706 | 0.21 | 7.41346 | 11.10054 | 8.63E-04 | 1.08E-02 |
| COG6 | ----- | ENSGALG00000017035 | component of oligomeric golgi complex 6 | 0.21 | 5.84731 | 10.82088 | 1.00E-03 | 1.21E-02 |
| HPF1 | ----- | ENSGALG00000009668 | histone PARylation factor 1 | 0.21 | 6.44703 | 14.37191 | 1.50E-04 | 2.83E-03 |
| HNRNPLL | HNRPLL | ENSGALG00000008504 | heterogeneous nuclear ribonucleoprotein L like | 0.21 | 7.12424 | 15.59177 | 7.86E-05 | 1.73E-03 |
| AHSA1 | ----- | ENSGALG00000010463 | activator of Hsp90 ATPase activity 1 | 0.21 | 6.93121 | 8.59232 | 3.38E-03 | 2.88E-02 |
| KPNA2 | ----- | ENSGALG00000003584 | karyopherin subunit alpha 2 | 0.21 | 8.96834 | 11.52233 | 6.88E-04 | 9.16E-03 |
| REV1 | REV1L | ENSGALG00000016766 | REV1, DNA directed polymerase | 0.21 | 5.54681 | 7.29082 | 6.93E-03 | 4.80E-02 |
| SRM | ----- | ENSGALG00000003039 | spermidine synthase | 0.21 | 6.49134 | 14.51409 | 1.39E-04 | 2.67E-03 |
| RCN1L | ----- | ----- | ----- | 0.21 | 7.09829 | 18.19934 | 1.99E-05 | 5.62E-04 |
| ATP2C1 | ----- | ENSGALG00000034340 | ATPase secretory pathway Ca2+ transporting 1 | 0.21 | 6.85174 | 16.53784 | 4.77E-05 | 1.15E-03 |
| TWSG1 | ----- | ENSGALG00000052629 | twisted gastrulation BMP signaling modulator 1 | 0.21 | 6.60112 | 10.92534 | 9.49E-04 | 1.16E-02 |
| MKL1 | ----- | ENSGALG00000012024 | megakaryoblastic leukemia (translocation) 1 | 0.21 | 6.70139 | 10.72175 | 1.06E-03 | 1.25E-02 |
| FCHSD2 | ----- | ENSGALG00000017329 | FCH and double SH3 domains 2 | 0.21 | 5.35022 | 8.23257 | 4.11E-03 | 3.31E-02 |
| EVI5 | ----- | ENSGALG00000029582 | ecotropic viral integration site 5 | 0.21 | 6.23157 | 14.61247 | 1.32E-04 | 2.56E-03 |
| DDX17 | ----- | ENSGALG00000037360 | DEAD-box helicase 17 | 0.21 | 8.99892 | 8.60174 | 3.36E-03 | 2.87E-02 |
| G6PD | ----- | ----- | ----- | 0.21 | 4.77391 | 8.16433 | 4.27E-03 | 3.40E-02 |
| CKAP2 | ----- | ENSGALG00000017025 | cytoskeleton associated protein 2 | 0.21 | 8.48512 | 10.27348 | 1.35E-03 | 1.49E-02 |
| IGSF9 | ----- | ENSGALG00000031193 | immunoglobulin superfamily member 9 | 0.21 | 7.38102 | 12.24810 | 4.66E-04 | 6.87E-03 |
| KIF1BP | kbp | ENSGALG00000004156 | KIF1 binding protein | 0.21 | 5.69318 | 12.03104 | 5.23E-04 | 7.50E-03 |
| GEMIN5 | ----- | ENSGALG00000003937 | gem nuclear organelle associated protein 5 | 0.20 | 7.16899 | 11.89963 | 5.61E-04 | 7.89E-03 |
| PSMB1 | ----- | ENSGALG00000011174 | proteasome subunit beta 1 | 0.20 | 7.49922 | 12.27478 | 4.59E-04 | 6.79E-03 |
| CD2AP | ----- | ENSGALG00000016720 | CD2 associated protein | 0.20 | 6.03195 | 7.74440 | 5.39E-03 | 4.02E-02 |
| SNU13 | ----- | ENSGALG00000011931 | small nuclear ribonucleoprotein 13 | 0.20 | 6.76330 | 11.54212 | 6.80E-04 | 9.09E-03 |
| CIR1 | CIR | ENSGALG00000014276 | corepressor interacting with RBPJ, 1 | 0.20 | 6.17022 | 10.96727 | 9.27E-04 | 1.14E-02 |
| POLR2E | ----- | ENSGALG00000002591 | RNA polymerase II subunit E | 0.20 | 6.71610 | 12.38725 | 4.32E-04 | 6.50E-03 |
| VASH2 | ----- | ENSGALG00000020067 | vasohibin 2 | 0.20 | 6.77793 | 8.64241 | 3.28E-03 | 2.82E-02 |
| PRR11 | ----- | ENSGALG00000054015 | proline rich 11 | 0.20 | 5.09606 | 7.69986 | 5.52E-03 | 4.09E-02 |
| PKM | ----- | ----- | ----- | 0.20 | 10.80797 | 8.17438 | 4.25E-03 | 3.39E-02 |
| NUP205 | ----- | ENSGALG00000011662 | nucleoporin 205 | 0.20 | 7.98813 | 12.22984 | 4.70E-04 | 6.92E-03 |
| GNG5 | ----- | ENSGALG00000027561 | G protein subunit gamma 5 | 0.20 | 6.02537 | 9.28527 | 2.31E-03 | 2.21E-02 |
| KIF18B | ----- | ENSGALG00000031553 | kinesin family member 18B | 0.20 | 5.91159 | 11.84270 | 5.79E-04 | 8.09E-03 |
| NUP160 | ----- | ENSGALG00000043074 | nucleoporin 160 | 0.20 | 8.08976 | 12.12431 | 4.98E-04 | 7.23E-03 |
| DCPS | ----- | ENSGALG00000031143 | decapping enzyme, scavenger | 0.20 | 5.99075 | 10.32512 | 1.31E-03 | 1.46E-02 |
| GTF3C2 | ----- | ----- | general transcription factor IIIC subunit 2 | 0.20 | 5.89233 | 10.72955 | 1.05E-03 | 1.25E-02 |
| TARSL2 | ----- | ENSGALG00000003985 | threonyl-tRNA synthetase like 2 | 0.20 | 6.02396 | 12.42364 | 4.24E-04 | 6.39E-03 |
| TMTC2 | ----- | ENSGALG00000010975 | transmembrane and tetratricopeptide repeat containing 2 | 0.20 | 5.86305 | 9.23215 | 2.38E-03 | 2.26E-02 |
| MTMR14 | ----- | ENSGALG00000006601 | myotubularin related protein 14 | 0.20 | 5.60525 | 8.65914 | 3.25E-03 | 2.80E-02 |
| IARS | ----- | ENSGALG00000004813 | isoleucyl-tRNA synthetase | 0.20 | 7.66079 | 10.50864 | 1.19E-03 | 1.36E-02 |
| CBFB | ----- | ENSGALG00000003169 | core-binding factor beta subunit | 0.20 | 7.05393 | 11.53065 | 6.85E-04 | 9.14E-03 |
| SRGAP2 | ----- | ENSGALG00000000816 | SLIT-ROBO Rho GTPase activating protein 2 | 0.20 | 7.20917 | 9.64269 | 1.90E-03 | 1.94E-02 |
| CDC16 | ----- | ENSGALG00000016816 | cell division cycle 16 | 0.20 | 5.76570 | 10.29548 | 1.33E-03 | 1.48E-02 |
| DTNBP1 | ----- | ENSGALG00000012703 | dystrobrevin binding protein 1 | 0.20 | 5.39249 | 12.14618 | 4.92E-04 | 7.19E-03 |

| | | | | | | | | |
|---|---|---|---|---|---|---|---|---|
| PWP1 | - - - - - | ENSGALG00000012631 | PWP1 homolog, endonuclein | 0.20 | 6.65138 | 11.06322 | 8.81E-04 | 1.09E-02 |
| SCHIP1 | - - - - - | ENSGALG00000039468 | schwannomin interacting protein 1 | 0.20 | 5.95743 | 8.75046 | 3.10E-03 | 2.70E-02 |
| LOC107057078 | | - - - - - | acyl-CoA dehydrogenase family member 10-like | 0.20 | 7.07544 | 9.56873 | 1.98E-03 | 2.00E-02 |
| FAM110B | - - - - - | ENSGALG00000035065 | family with sequence similarity 110 member B | 0.20 | 6.16831 | 7.41275 | 6.48E-03 | 4.60E-02 |
| CHM | CHML | ENSGALG00000006906 | CHM, Rab escort protein 1 | 0.20 | 6.22184 | 9.19862 | 2.42E-03 | 2.28E-02 |
| CEP295 | - - - - - | ENSGALG00000040433 | centrosomal protein 295 | 0.20 | 6.50438 | 9.36703 | 2.21E-03 | 2.15E-02 |
| MUM1 | - - - - - | ENSGALG00010028034 | ----- | 0.20 | 5.79930 | 9.25057 | 2.35E-03 | 2.25E-02 |
| UQCC1 | UQCC | ENSGALG00000040254 | ubiquinol-cytochrome c reductase complex assembly factor 1 | 0.20 | 5.77838 | 9.76263 | 1.78E-03 | 1.84E-02 |
| TPM1 | alpha-fTM | ENSGALG00000003521 | tropomyosin 1 (alpha) | 0.20 | 9.01033 | 12.66974 | 3.72E-04 | 5.79E-03 |
| MXI1 | - - - - - | ENSGALG00000008555 | MAX interactor 1, dimerization protein | 0.20 | 5.29509 | 8.43498 | 3.68E-03 | 3.07E-02 |
| OAZ2 | - - - - - | ENSGALG00000036288 | ornithine decarboxylase antizyme 2 | 0.20 | 6.45986 | 13.09922 | 2.95E-04 | 4.87E-03 |
| SELENOT | - - - - - | ENSGALG00000010399 | selenoprotein T | 0.20 | 6.18247 | 9.93755 | 1.62E-03 | 1.71E-02 |
| LOC426821 | | - - - - - | zinc finger protein 624-like | 0.20 | 5.36857 | 7.31065 | 6.85E-03 | 4.76E-02 |
| POLR1A | - - - - - | ENSGALG00000038160 | RNA polymerase I subunit A | 0.20 | 6.59943 | 9.84366 | 1.70E-03 | 1.78E-02 |
| XPNPEP1 | - - - - - | ENSGALG00000008523 | X-prolyl aminopeptidase (aminopeptidase P) 1, soluble | 0.20 | 6.96913 | 13.55910 | 2.31E-04 | 4.02E-03 |
| RCOR3 | - - - - - | ENSGALG00000009871 | REST corepressor 3 | 0.20 | 6.98026 | 17.70703 | 2.58E-05 | 6.95E-04 |
| PCBP4 | - - - - - | ENSGALG00000027695 | poly(rC)-binding protein 4-like | 0.20 | 6.81729 | 11.48998 | 7.00E-04 | 9.26E-03 |
| SMC4 | SMC4L1 | ENSGALG00000009553 | structural maintenance of chromosomes 4 | 0.20 | 7.76906 | 18.71707 | 1.52E-05 | 4.54E-04 |
| UBE2S | - - - - - | ENSGALG00000052573 | ubiquitin conjugating enzyme E2 S | 0.19 | 7.06969 | 10.15179 | 1.44E-03 | 1.57E-02 |
| LOC422214 | | - - - - - | nuclear cap binding protein subunit 2, 20kDa | 0.19 | 6.16354 | 8.03811 | 4.58E-03 | 3.59E-02 |
| TTLL12 | - - - - - | ENSGALG00000014167 | tubulin tyrosine ligase like 12 | 0.19 | 7.15043 | 11.57741 | 6.68E-04 | 9.00E-03 |
| ECT2 | - - - - - | ENSGALG00000009157 | epithelial cell transforming 2 | 0.19 | 6.75610 | 11.03109 | 8.96E-04 | 1.11E-02 |
| HMBS | - - - - - | ENSGALG00000042939 | hydroxymethylbilane synthase | 0.19 | 6.10410 | 9.10010 | 2.56E-03 | 2.36E-02 |
| HOXA6 | HOX1 | ENSGALG00000028908 | homeobox A6 | 0.19 | 5.68488 | 7.72668 | 5.44E-03 | 4.05E-02 |
| ACTN1 | - - - - - | ENSGALG00000042458 | actinin, alpha 1 | 0.19 | 7.48797 | 14.05935 | 1.77E-04 | 3.24E-03 |
| FUS | FUS/TLS | ENSGALG00000032219 | FUS RNA binding protein | 0.19 | 9.39909 | 9.70129 | 1.84E-03 | 1.89E-02 |
| KCTD3 | - - - - - | ENSGALG00000009678 | SH3KBP1 binding protein 1 | 0.19 | 6.89329 | 10.42640 | 1.24E-03 | 1.40E-02 |
| RPF1 | - - - - - | ENSGALG00000008796 | ribosome production factor 1 homolog | 0.19 | 5.90904 | 7.44631 | 6.36E-03 | 4.54E-02 |
| ACTR6 | GARPX | ENSGALG00000040976 | ARP6 actin-related protein 6 homolog | 0.19 | 7.02980 | 7.88233 | 4.99E-03 | 3.82E-02 |
| RAD54L | GdRAD54 | ENSGALG00000010356 | RAD54-like (S. cerevisiae) | 0.19 | 5.68809 | 8.12667 | 4.36E-03 | 3.45E-02 |
| SLC39A14 | - - - - - | ENSGALG00000033989 | solute carrier family 39 member 14 | 0.19 | 6.17039 | 8.05392 | 4.54E-03 | 3.56E-02 |
| DPP7 | - - - - - | ENSGALG00000038145 | dipeptidyl peptidase 7 | 0.19 | 5.58289 | 7.74626 | 5.38E-03 | 4.02E-02 |
| KAT2A | - - - - - | ENSGALG00000003369 | lysine acetyltransferase 2A | 0.19 | 6.87670 | 11.84411 | 5.78E-04 | 8.09E-03 |
| SLC25A15 | - - - - - | ENSGALG00000017032 | solute carrier family 25 member 15 | 0.19 | 5.65290 | 8.13786 | 4.34E-03 | 3.44E-02 |
| NSF | - - - - - | ENSGALG00000001055 | N-ethylmaleimide sensitive factor, vesicle fusing ATPase | 0.19 | 6.39086 | 7.84850 | 5.09E-03 | 3.86E-02 |
| LOC107057310 | | - - - - - | uncharacterized LOC107057310 | 0.19 | 5.28197 | 8.56078 | 3.43E-03 | 2.91E-02 |
| SELENON | - - - - - | - - - - - | selenoprotein N | 0.19 | 6.92180 | 11.84913 | 5.77E-04 | 8.07E-03 |
| HJURP | - - - - - | ENSGALG00000015363 | Holliday junction recognition protein | 0.19 | 5.67883 | 7.63136 | 5.74E-03 | 4.20E-02 |
| AKR1A1 | - - - - - | ENSGALG00000010244 | aldo-keto reductase family 1 member A1 | 0.19 | 6.53082 | 10.51714 | 1.18E-03 | 1.35E-02 |
| PTP4A2 | - - - - - | ENSGALG00000003265 | protein tyrosine phosphatase type IVA, member 2 | 0.19 | 6.78516 | 9.69398 | 1.85E-03 | 1.90E-02 |
| MSH6 | - - - - - | ENSGALG00000008957 | mutS homolog 6 | 0.19 | 7.59165 | 10.53939 | 1.17E-03 | 1.34E-02 |
| NVL | - - - - - | ENSGALG00000009322 | nuclear VCP-like | 0.19 | 6.01991 | 11.11297 | 8.57E-04 | 1.07E-02 |
| EIF3D | - - - - - | ENSGALG00000012529 | eukaryotic translation initiation factor 3 subunit D | 0.19 | 8.67997 | 9.62119 | 1.92E-03 | 1.96E-02 |
| EPB41L2 | - - - - - | - - - - - | erythrocyte membrane protein band 4.1 like 2 | 0.19 | 6.53254 | 8.45534 | 3.64E-03 | 3.05E-02 |

| | | | | | | | | |
|---|---|---|---|---|---|---|---|---|
| TUBB4B | TUBB2C | ENSGALG00000008586 | tubulin beta 4B class IVb | 0.19 | 7.53232 | 12.90643 | 3.27E-04 | 5.25E-03 |
| RASAL3 | - - - - - | ENSGALG00000039156 | RAS protein activator like 3 | 0.19 | 7.66323 | 9.44451 | 2.12E-03 | 2.09E-02 |
| RARS | - - - - - | ENSGALG00000001850 | arginyl-tRNA synthetase | 0.19 | 7.33114 | 8.36214 | 3.83E-03 | 3.15E-02 |
| TIMMDC1 | - - - - - | ENSGALG00000024480 | translocase of inner mitochondrial membrane domain containing 1 | 0.19 | 6.15640 | 9.19503 | 2.43E-03 | 2.28E-02 |
| SCOC | BERT | ENSGALG00000009813 | short coiled-coil protein | 0.19 | 6.42050 | 8.48365 | 3.58E-03 | 3.01E-02 |
| PKNOX1 | - - - - - | ENSGALG00000016191 | PBX/knotted 1 homeobox 1 | 0.19 | 6.30135 | 7.72906 | 5.43E-03 | 4.05E-02 |
| TBC1D13 | - - - - - | ENSGALG00000004645 | TBC1 domain family member 13 | 0.19 | 6.86690 | 10.27746 | 1.35E-03 | 1.49E-02 |
| TSPAN4 | - - - - - | ENSGALG00000006837 | tetraspanin 4 | 0.18 | 6.37562 | 10.69402 | 1.07E-03 | 1.26E-02 |
| RIMKLB | - - - - - | ENSGALG00000040184 | ribosomal modification protein rimK like family member B | 0.18 | 6.27990 | 8.24394 | 4.09E-03 | 3.30E-02 |
| CASP3 | caspase-3 | ENSGALG00000010638 | caspase 3 | 0.18 | 6.97980 | 12.41387 | 4.26E-04 | 6.42E-03 |
| FARSA | FARSLA | ENSGALG00000040258 | phenylalanyl-tRNA synthetase alpha subunit | 0.18 | 6.57360 | 8.35168 | 3.85E-03 | 3.16E-02 |
| RNF14 | - - - - - | ENSGALG00000043085 | ring finger protein 14 | 0.18 | 6.45820 | 8.09331 | 4.44E-03 | 3.51E-02 |
| HSPA4 | - - - - - | ENSGALG00000040882 | heat shock protein family A (Hsp70) member 4 | 0.18 | 7.41320 | 8.07521 | 4.49E-03 | 3.53E-02 |
| LRWD1 | - - - - - | ENSGALG00000001852 | leucine rich repeats and WD repeat domain containing 1 | 0.18 | 6.71920 | 8.70804 | 3.17E-03 | 2.74E-02 |
| CAD | - - - - - | ENSGALG00000016524 | carbamoyl-phosphate synthetase 2, aspartate transcarbamylase, and dihydroorotase | 0.18 | 8.80548 | 8.15286 | 4.30E-03 | 3.42E-02 |
| MRE11 | MRE11A | ENSGALG00000017211 | MRE11 homolog, double strand break repair nuclease | 0.18 | 6.66016 | 9.89294 | 1.66E-03 | 1.74E-02 |
| LUC7L2 | - - - - - | ENSGALG00000036238 | LUC7 like 2, pre-mRNA splicing factor | 0.18 | 7.78479 | 11.19821 | 8.19E-04 | 1.04E-02 |
| RAB8B | - - - - - | ENSGALG00000003487 | RAB8B, member RAS oncogene family | 0.18 | 6.95609 | 14.89990 | 1.13E-04 | 2.26E-03 |
| LTBP1 | - - - - - | ENSGALG00000010448 | latent transforming growth factor beta binding protein 1 | 0.18 | 8.50873 | 7.88666 | 4.98E-03 | 3.82E-02 |
| KLHL2 | - - - - - | ENSGALG00000009554 | kelch like family member 2 | 0.18 | 6.29348 | 9.04413 | 2.64E-03 | 2.41E-02 |
| PI4KB | - - - - - | ENSGALG00000024034 | phosphatidylinositol 4-kinase beta | 0.18 | 6.57973 | 8.96595 | 2.75E-03 | 2.48E-02 |
| KANSL1L | - - - - - | ENSGALG00000002866 | KAT8 regulatory NSL complex subunit 1-like | 0.18 | 5.51502 | 8.07294 | 4.49E-03 | 3.54E-02 |
| CCT6A | CCT6 | ENSGALG00000002448 | chaperonin containing TCP1 subunit 6A | 0.18 | 8.88016 | 8.07870 | 4.48E-03 | 3.53E-02 |
| CCT8 | - - - - - | ENSGALG00000015821 | chaperonin containing TCP1 subunit 8 | 0.18 | 8.80978 | 7.64928 | 5.68E-03 | 4.18E-02 |
| GTF2E2 | - - - - - | ENSGALG00000010283 | general transcription factor IIE subunit 2 | 0.18 | 5.63125 | 8.82348 | 2.97E-03 | 2.63E-02 |
| NDC1 | - - - - - | ENSGALG00000031606 | NDC1 transmembrane nucleoporin | 0.18 | 6.99153 | 8.01652 | 4.64E-03 | 3.62E-02 |
| C1QBP | - - - - - | ENSGALG00000001654 | complement C1q binding protein | 0.18 | 8.46853 | 7.96579 | 4.77E-03 | 3.70E-02 |
| LAS1L | - - - - - | ENSGALG00000043447 | LAS1 like, ribosome biogenesis factor | 0.18 | 6.80656 | 13.69747 | 2.15E-04 | 3.79E-03 |
| SUGT1 | - - - - - | ENSGALG00000016946 | SGT1 homolog, MIS12 kinetochore complex assembly cochaperone | 0.18 | 6.59409 | 11.15275 | 8.39E-04 | 1.06E-02 |
| AIFM1 | PDCD8 | ENSGALG00000004003 | apoptosis inducing factor, mitochondria associated 1 | 0.18 | 7.11476 | 11.31443 | 7.69E-04 | 9.90E-03 |
| SEC31A | SEC31L1 | ENSGALG00000040484 | SEC31 homolog A, COPII coat complex component | 0.18 | 8.94655 | 7.53139 | 6.06E-03 | 4.37E-02 |
| USPL1 | - - - - - | ENSGALG00000017079 | ubiquitin specific peptidase like 1 | 0.18 | 7.18107 | 8.08569 | 4.46E-03 | 3.52E-02 |
| EXOSC9 | - - - - - | ENSGALG00000011884 | exosome component 9 | 0.18 | 6.16976 | 8.36805 | 3.82E-03 | 3.14E-02 |
| RHOT1 | - - - - - | ENSGALG00000003397 | ras homolog family member T1 | 0.18 | 5.93000 | 10.34641 | 1.30E-03 | 1.45E-02 |
| ZCCHC24 | - - - - - | ENSGALG00000026864 | zinc finger CCHC-type containing 24 | 0.18 | 6.79975 | 8.63039 | 3.31E-03 | 2.84E-02 |
| CBR1 | - - - - - | - - - - - | ----- | 0.18 | 5.87704 | 7.41616 | 6.46E-03 | 4.60E-02 |
| ALDH9A1 | - - - - - | ENSGALG00000003495 | aldehyde dehydrogenase 9 family member A1 | 0.18 | 7.73656 | 12.89524 | 3.29E-04 | 5.27E-03 |
| NID2 | - - - - - | ENSGALG00000012394 | nidogen 2 | 0.18 | 7.77914 | 8.71029 | 3.16E-03 | 2.74E-02 |
| ANGEL2 | - - - - - | ENSGALG00000009802 | angel homolog 2 | 0.18 | 5.17148 | 7.18505 | 7.35E-03 | 5.00E-02 |
| USP51 | - - - - - | - - - - - | ----- | 0.17 | 6.97992 | 10.76146 | 1.04E-03 | 1.23E-02 |
| WDR76 | - - - - - | ENSGALG00000035465 | WD repeat domain 76 | 0.17 | 6.25639 | 7.79504 | 5.24E-03 | 3.95E-02 |
| THOC5 | - - - - - | ENSGALG00000029223 | THO complex 5 | 0.17 | 7.59716 | 10.11834 | 1.47E-03 | 1.59E-02 |
| NCBP2 | - - - - - | ENSGALG00000006843 | nuclear cap binding protein subunit 2, 20kDa | 0.17 | 7.75306 | 12.93923 | 3.22E-04 | 5.19E-03 |
| ITSN1 | - - - - - | ENSGALG00000036677 | intersectin 1 | 0.17 | 7.30274 | 10.49524 | 1.20E-03 | 1.36E-02 |

| | | | | | | | | |
|---|---|---|---|---|---|---|---|---|
| DDR1 | - - - - - | ENSGALG00000008616 | discoidin domain receptor tyrosine kinase 1 | 0.17 | 7.36127 | 8.15690 | 4.29E-03 | 3.41E-02 |
| SUFU | - - - - - | ENSGALG00000008096 | SUFU negative regulator of hedgehog signaling | 0.17 | 5.92754 | 7.37487 | 6.61E-03 | 4.65E-02 |
| FAR1 | MLSTD2 | ENSGALG00000005353 | fatty acyl-CoA reductase 1 | 0.17 | 6.65960 | 10.92832 | 9.47E-04 | 1.16E-02 |
| SKA2 | - - - - - | ENSGALG00000005090 | spindle and kinetochore associated complex subunit 2 pseudogene 1 | 0.17 | 6.61783 | 8.00305 | 4.67E-03 | 3.64E-02 |
| CCDC30 | - - - - - | - - - - - | coiled-coil domain containing 30 | 0.17 | 5.71402 | 7.84732 | 5.09E-03 | 3.86E-02 |
| NT5C2 | - - - - - | ENSGALG00000008179 | 5'-nucleotidase, cytosolic II | 0.17 | 6.59796 | 9.96518 | 1.60E-03 | 1.70E-02 |
| LOC112530429 | | - - - - - | uncharacterized LOC112530429 | 0.17 | 7.87196 | 7.31137 | 6.85E-03 | 4.76E-02 |
| NDUFB4 | - - - - - | ENSGALG00000027688 | NADH dehydrogenase (ubiquinone) 1 beta subcomplex, 4, 15kDa | 0.17 | 6.47359 | 7.40063 | 6.52E-03 | 4.61E-02 |
| PITPNB | - - - - - | ENSGALG00000005654 | phosphatidylinositol transfer protein beta | 0.17 | 7.09819 | 13.01104 | 3.10E-04 | 5.06E-03 |
| TIAL1 | - - - - - | ENSGALG00000009427 | TIA1 cytotoxic granule associated RNA binding protein like 1 | 0.17 | 5.74865 | 8.26084 | 4.05E-03 | 3.27E-02 |
| PTK2 | FAK | ENSGALG00000031741 | protein tyrosine kinase 2 | 0.16 | 7.56296 | 8.71820 | 3.15E-03 | 2.74E-02 |
| CSE1L | - - - - - | ENSGALG00000004707 | chromosome segregation 1 like | 0.16 | 8.31603 | 8.83647 | 2.95E-03 | 2.62E-02 |
| ZBTB33 | - - - - - | ENSGALG00000008611 | zinc finger and BTB domain containing 33 | 0.16 | 6.10804 | 10.13543 | 1.45E-03 | 1.58E-02 |
| ELMO2 | - - - - - | ENSGALG00000007246 | engulfment and cell motility 2 | 0.16 | 7.12961 | 9.18384 | 2.44E-03 | 2.29E-02 |
| NAP1L1 | - - - - - | ENSGALG00000010238 | nucleosome assembly protein 1 like 1 | 0.16 | 9.37804 | 7.25787 | 7.06E-03 | 4.85E-02 |
| IGSF3 | - - - - - | ENSGALG00000036738 | immunoglobulin superfamily member 3 | 0.16 | 7.33400 | 7.62534 | 5.76E-03 | 4.21E-02 |
| NF2 | - - - - - | ENSGALG00000008073 | neurofibromin 2 | 0.16 | 7.64640 | 9.38993 | 2.18E-03 | 2.14E-02 |
| UBA3 | - - - - - | ENSGALG00000013410 | ubiquitin like modifier activating enzyme 3 | 0.16 | 6.40388 | 7.53157 | 6.06E-03 | 4.37E-02 |
| MCMBP | C10orf119 | ENSGALG00000009455 | minichromosome maintenance complex binding protein | 0.16 | 6.84461 | 9.41677 | 2.15E-03 | 2.11E-02 |
| UBL3 | - - - - - | ENSGALG00000017084 | ubiquitin like 3 | 0.16 | 5.71265 | 8.30922 | 3.94E-03 | 3.22E-02 |
| GALNT1 | - - - - - | ENSGALG00000013135 | polypeptide N-acetylgalactosaminyltransferase 1 | 0.16 | 7.23504 | 9.64190 | 1.90E-03 | 1.94E-02 |
| RBM4B | - - - - - | - - - - - | ----- | 0.16 | 6.43610 | 7.93616 | 4.85E-03 | 3.74E-02 |
| GLT8D1 | - - - - - | ENSGALG00000001642 | glycosyltransferase 8 domain containing 1 | 0.16 | 5.87780 | 8.06235 | 4.52E-03 | 3.55E-02 |
| ESPL1 | - - - - - | ENSGALG00000041923 | extra spindle pole bodies like 1, separase | 0.16 | 7.48066 | 7.29187 | 6.93E-03 | 4.80E-02 |
| SAE1 | - - - - - | ENSGALG00000048152 | SUMO1 activating enzyme subunit 1 | 0.16 | 7.12681 | 8.40559 | 3.74E-03 | 3.10E-02 |
| TOP1 | - - - - - | ENSGALG00000003681 | DNA topoisomerase I | 0.16 | 8.39688 | 8.88458 | 2.88E-03 | 2.57E-02 |
| RALGAPB | - - - - - | ENSGALG00000001420 | Ral GTPase activating protein non-catalytic beta subunit | 0.16 | 7.00473 | 8.40260 | 3.75E-03 | 3.10E-02 |
| UBE2N | - - - - - | ENSGALG00000030515 | ubiquitin conjugating enzyme E2 N | 0.16 | 7.45731 | 9.13228 | 2.51E-03 | 2.33E-02 |
| ACTR1A | - - - - - | ENSGALG00000008086 | ARP1 actin-related protein 1 homolog A, centractin alpha | 0.16 | 7.43269 | 9.99464 | 1.57E-03 | 1.67E-02 |
| FAM171A1 | - - - - - | ENSGALG00000008764 | family with sequence similarity 171, member A1 | 0.16 | 6.73748 | 9.15734 | 2.48E-03 | 2.31E-02 |
| USP48 | - - - - - | ENSGALG00000030857 | ubiquitin specific peptidase 48 | 0.16 | 6.34104 | 7.81376 | 5.18E-03 | 3.92E-02 |
| BCL7A | - - - - - | ENSGALG00000004379 | BAF chromatin remodeling complex subunit BCL7A | 0.16 | 6.56648 | 8.70153 | 3.18E-03 | 2.75E-02 |
| PSMC3 | - - - - - | ENSGALG00000008108 | proteasome 26S subunit, ATPase 3 | 0.16 | 7.90511 | 9.45255 | 2.11E-03 | 2.09E-02 |
| PSME4 | - - - - - | ENSGALG00000008163 | proteasome activator subunit 4 | 0.16 | 6.94939 | 8.03178 | 4.60E-03 | 3.60E-02 |
| PTGES3 | CPGES | ENSGALG00000036837 | prostaglandin E synthase 3 | 0.15 | 8.54975 | 7.38560 | 6.57E-03 | 4.64E-02 |
| COPS4 | - - - - - | ENSGALG00000011130 | COP9 signalosome subunit 4 | 0.15 | 6.85243 | 9.09810 | 2.56E-03 | 2.37E-02 |
| MAP2K1 | - - - - - | ENSGALG00000007687 | mitogen-activated protein kinase kinase 1 | 0.15 | 6.50021 | 7.44726 | 6.35E-03 | 4.54E-02 |
| DCTN1 | Chip150 | ENSGALG00000035156 | dynactin subunit 1 | 0.15 | 8.06230 | 7.58112 | 5.90E-03 | 4.29E-02 |
| FAM98A | - - - - - | ENSGALG00000010428 | family with sequence similarity 98 member A | 0.15 | 6.50419 | 8.56445 | 3.43E-03 | 2.91E-02 |
| TMEM184C | TMEM34 | ENSGALG00000010019 | transmembrane protein 184C | 0.15 | 6.35126 | 8.37205 | 3.81E-03 | 3.14E-02 |
| FAM168A | - - - - - | ENSGALG00000017323 | family with sequence similarity 168 member A | 0.15 | 6.12791 | 8.13110 | 4.35E-03 | 3.45E-02 |
| PLK1 | - - - - - | ENSGALG00000006110 | polo like kinase 1 | 0.15 | 8.60063 | 7.37373 | 6.62E-03 | 4.65E-02 |
| TXNRD3 | - - - - - | ENSGALG00000039363 | thioredoxin reductase 3 | 0.15 | 7.19262 | 8.16335 | 4.27E-03 | 3.40E-02 |
| CTNNBL1 | - - - - - | ENSGALG00000006817 | catenin beta like 1 | 0.15 | 6.48073 | 7.62565 | 5.75E-03 | 4.21E-02 |

| | | | | | | | | |
|---|---|---|---|---|---|---|---|---|
| TRAF7 | - - - - - | ENSGALG00000005767 | TNF receptor associated factor 7 | 0.15 | 6.12917 | 7.98610 | 4.71E-03 | 3.67E-02 |
| BUB1 | - - - - - | ENSGALG00000008233 | BUB1 mitotic checkpoint serine/threonine kinase | 0.15 | 7.50334 | 8.19880 | 4.19E-03 | 3.36E-02 |
| NOMO3 | - - - - - | - - - - - | ----- | 0.15 | 7.31348 | 7.44394 | 6.37E-03 | 4.54E-02 |
| SMAD5 | - - - - - | ENSGALG00000006309 | SMAD family member 5 | 0.15 | 7.43835 | 7.47020 | 6.27E-03 | 4.49E-02 |
| DERL1 | derlin-1 | ENSGALG00000033950 | derlin 1 | 0.15 | 6.87055 | 10.15857 | 1.44E-03 | 1.57E-02 |
| MRPL20 | - - - - - | ENSGALG00000001542 | mitochondrial ribosomal protein L20 | 0.15 | 6.41045 | 8.27522 | 4.02E-03 | 3.26E-02 |
| AKIRIN2 | akirin-2 | ENSGALG00000015791 | akirin 2 | 0.14 | 7.35705 | 8.30505 | 3.95E-03 | 3.22E-02 |
| CYFIP1 | - - - - - | ENSGALG00000016736 | cytoplasmic FMR1 interacting protein 1 | 0.14 | 7.49180 | 10.56257 | 1.15E-03 | 1.33E-02 |
| DNAJC10 | - - - - - | ENSGALG00000008862 | DnaJ heat shock protein family (Hsp40) member C10 | 0.14 | 6.95216 | 8.12550 | 4.36E-03 | 3.45E-02 |
| TARDBP | - - - - - | ENSGALG00000002906 | TAR DNA binding protein | 0.14 | 6.95887 | 7.26923 | 7.01E-03 | 4.83E-02 |
| EZH2 | - - - - - | ENSGALG00000043399 | enhancer of zeste 2 polycomb repressive complex 2 subunit | 0.14 | 7.23331 | 7.40500 | 6.50E-03 | 4.60E-02 |
| RYK | - - - - - | ENSGALG00000006488 | receptor-like tyrosine kinase | 0.14 | 6.41822 | 9.00065 | 2.70E-03 | 2.45E-02 |
| FAM109B | - - - - - | - - - - - | family with sequence similarity 109, member B | 0.14 | 6.98127 | 8.28774 | 3.99E-03 | 3.24E-02 |
| GNB4 | - - - - - | ENSGALG00000008983 | G protein subunit beta 4 | 0.14 | 8.41167 | 7.64491 | 5.69E-03 | 4.18E-02 |
| MEMO1 | - - - - - | ENSGALG00000010624 | mediator of cell motility 1 | 0.14 | 6.62089 | 7.41002 | 6.49E-03 | 4.60E-02 |
| DARS | - - - - - | ENSGALG00000012355 | aspartyl-tRNA synthetase | 0.14 | 7.51766 | 7.83355 | 5.13E-03 | 3.89E-02 |
| COPG2 | - - - - - | ENSGALG00000008144 | coatomer protein complex subunit gamma 2 | 0.14 | 7.02805 | 7.23220 | 7.16E-03 | 4.90E-02 |
| UBE2G1 | - - - - - | ENSGALG00000001441 | ubiquitin conjugating enzyme E2 G1 | 0.13 | 7.35639 | 7.21169 | 7.24E-03 | 4.94E-02 |
| TAX1BP3 | - - - - - | ENSGALG00000004618 | Tax1 binding protein 3 | 0.13 | 7.70103 | 7.73536 | 5.41E-03 | 4.03E-02 |
| SEC13 | SEC13L1 | ENSGALG00000008419 | SEC13 homolog, nuclear pore and COPII coat complex component | 0.13 | 7.52425 | 7.24027 | 7.13E-03 | 4.89E-02 |
| MSI2 | - - - - - | ENSGALG00000005567 | musashi RNA binding protein 2 | 0.12 | 7.30733 | 7.69426 | 5.54E-03 | 4.10E-02 |

| Gene name | Gene stable ID | Gene description | log2FC | logCPM | LR | PValue | FDR |
|---|---|---|---|---|---|---|---|
| LOC770705 | - - - - - | envelope glycoprotein gp95-like | -3.83 | 3.5830 | 39.9227 | 2.64E-10 | 3.51E-08 |
| LOC107049175 | - - - - - | withdrawn | -3.57 | -0.2832 | 51.8086 | 6.12E-13 | 1.55E-10 |
| LOC107057413 | - - - - - | collagen alpha-1(I) chain-like | -3.43 | 1.4229 | 58.4631 | 2.07E-14 | 7.41E-12 |
| ERNI | ENSGALG00010002022 | early response to neural induction ERNI | -3.38 | 0.5707 | 40.1275 | 2.38E-10 | 3.21E-08 |
| LOC107049890 | - - - - - | pre-mRNA-splicing factor CWC22-like | -3.36 | 0.2618 | 109.2566 | 1.43E-25 | 1.68E-22 |
| LOC107049106 | - - - - - | withdrawn | -3.36 | 1.2159 | 83.2042 | 7.40E-20 | 6.09E-17 |
| LOC776874 | - - - - - | uncharacterized LOC776874 | -3.09 | 0.1795 | 63.5687 | 1.55E-15 | 7.28E-13 |
| LOC107049408 | - - - - - | uncharacterized LOC107049408 | -2.96 | 0.5184 | 34.4442 | 4.39E-09 | 4.17E-07 |
| LOC112531874 | - - - - - | uncharacterized LOC112531874 | -2.96 | 1.3292 | 32.2181 | 1.38E-08 | 1.17E-06 |
| LOC107051278 | - - - - - | withdrawn | -2.79 | 1.4534 | 113.1360 | 2.01E-26 | 2.55E-23 |
| HOXD11 | ENSGALG00000039629 | homeobox D11 | -2.78 | 2.0347 | 7.3249 | 6.80E-03 | 4.73E-02 |
| NPHS2 | ENSGALG00000004163 | NPHS2, podocin | -2.71 | 5.9236 | 19.7944 | 8.62E-06 | 2.91E-04 |
| HOXA11 | ENSGALG00000040021 | homeobox A11 | -2.60 | 0.8886 | 13.1208 | 2.92E-04 | 4.83E-03 |
| LOC112531841 | - - - - - | uncharacterized LOC112531841 | -2.47 | -0.1148 | 24.9291 | 5.95E-07 | 3.12E-05 |
| LOC112530433 | - - - - - | withdrawn | -2.45 | 1.4217 | 38.4635 | 5.58E-10 | 6.60E-08 |
| LOC107051196 | - - - - - | withdrawn | -2.34 | 0.8007 | 19.1453 | 1.21E-05 | 3.77E-04 |
| LOC112531766 | - - - - - | uncharacterized LOC112531766 | -2.33 | 1.9104 | 61.4955 | 4.44E-15 | 1.74E-12 |
| LOC112533579 | - - - - - | withdrawn | -2.33 | 1.0453 | 38.5655 | 5.29E-10 | 6.31E-08 |
| LOC112531027 | - - - - - | withdrawn | -2.30 | 1.1980 | 38.8920 | 4.48E-10 | 5.42E-08 |
| LOC112532782 | - - - - - | uncharacterized LOC112532782 | -2.25 | 1.5293 | 84.1345 | 4.62E-20 | 4.00E-17 |
| LOC107053233 | - - - - - | uncharacterized LOC107053233 | -2.23 | 0.1156 | 47.2946 | 6.11E-12 | 1.29E-09 |
| GSC | ENSGALG00000010974 | goosecoid homeobox | -2.21 | 2.0263 | 15.0176 | 1.07E-04 | 2.16E-03 |
| LOC101747821 | - - - - - | uncharacterized LOC101747821 | -2.14 | 4.9115 | 39.7066 | 2.95E-10 | 3.82E-08 |
| LOC112532134 | - - - - - | uncharacterized LOC112532134 | -2.05 | 0.1194 | 29.1734 | 6.62E-08 | 4.76E-06 |
| LOC112531014 | - - - - - | withdrawn | -2.04 | 0.2655 | 22.4415 | 2.17E-06 | 9.02E-05 |
| LOC107052944 | - - - - - | withdrawn | -2.03 | -0.2492 | 52.7948 | 3.70E-13 | 1.03E-10 |
| LOC112531457 | - - - - - | withdrawn | -2.03 | -0.3387 | 27.6143 | 1.48E-07 | 9.63E-06 |
| MYH1F | ENSGALG00000042257 | myosin, heavy chain 1F, skeletal muscle | -2.02 | 1.3999 | 20.2047 | 6.96E-06 | 2.42E-04 |
| LOC107054528 | - - - - - | uncharacterized LOC107054528 | -2.00 | 0.8390 | 51.8370 | 6.03E-13 | 1.55E-10 |
| LOC107054238 | - - - - - | uncharacterized LOC107054238 | -2.00 | 1.1706 | 14.8267 | 1.18E-04 | 2.34E-03 |
| CD69L | - - - - - | CD69 molecule like | -1.99 | 6.3941 | 36.3386 | 1.66E-09 | 1.77E-07 |
| OPN4-1 | ENSGALG00000041662 | photopigment melanopsin-like | -1.98 | 1.2905 | 45.6812 | 1.39E-11 | 2.63E-09 |
| CLC2BL1 | - - - - - | c-type lectin like 13, MHCY region | -1.97 | 2.3077 | 62.8385 | 2.24E-15 | 9.72E-13 |
| POMC | ENSGALG00000016600 | proopiomelanocortin | -1.95 | -0.1350 | 18.6478 | 1.57E-05 | 4.67E-04 |
| LOC107054230 | - - - - - | uncharacterized LOC107054230 | -1.94 | 0.1815 | 47.1049 | 6.73E-12 | 1.37E-09 |
| GIMAP1 | - - - - - | GIMAP1-GIMAP5 readthrough | -1.93 | 2.5829 | 149.4346 | 2.30E-34 | 7.58E-31 |
| HOXA10 | ENSGALG00000026631 | homeobox A10 | -1.93 | 0.9354 | 9.5798 | 1.97E-03 | 1.99E-02 |
| LOC107049467 | - - - - - | uncharacterized LOC107049467 | -1.87 | 3.1810 | 28.8176 | 7.95E-08 | 5.54E-06 |
| LOC107049564 | - - - - - | uncharacterized LOC107049564 | -1.86 | 1.9839 | 58.8550 | 1.70E-14 | 6.21E-12 |
| LOC107053000 | - - - - - | uncharacterized LOC107053000 | -1.84 | 0.9609 | 71.7903 | 2.39E-17 | 1.27E-14 |
| SLC9A2 | ENSGALG00000016791 | solute carrier family 9 member A2 | -1.82 | 0.0793 | 42.3935 | 7.46E-11 | 1.17E-08 |
| LOC107057189 | - - - - - | withdrawn | -1.82 | 2.7086 | 42.3222 | 7.74E-11 | 1.19E-08 |

| | | | | | | | |
|---|---|---|---|---|---|---|---|
| SST | | somatostatin | -1.81 | 3.1571 | 25.7276 | 3.93E-07 | 2.20E-05 |
| LOC107052779 | - - - - - | withdrawn | -1.80 | 0.7403 | 76.9193 | 1.78E-18 | 1.13E-15 |
| LOC107052419 | - - - - - | withdrawn | -1.79 | -0.1847 | 24.4238 | 7.73E-07 | 3.84E-05 |
| LOC107055075 | - - - - - | withdrawn | -1.79 | -0.1392 | 30.5064 | 3.33E-08 | 2.56E-06 |
| TRBV19 | ENSGALG00000014750 | T cell receptor beta variable 19 | -1.79 | -0.4286 | 19.5641 | 9.73E-06 | 3.18E-04 |
| LOC107056991 | - - - - - | withdrawn | -1.78 | 0.7548 | 20.8835 | 4.88E-06 | 1.81E-04 |
| CCR4 | ENSGALG00000039146 | C-C motif chemokine receptor 4 | -1.77 | -0.1060 | 26.8226 | 2.23E-07 | 1.33E-05 |
| MYH1D | ENSGALG00000027323 | myosin, heavy chain 1D, skeletal muscle | -1.76 | 4.8111 | 19.5131 | 9.99E-06 | 3.24E-04 |
| LOC112530773 | - - - - - | withdrawn | -1.76 | 2.7706 | 117.8886 | 1.83E-27 | 2.74E-24 |
| YF5 | ENSGALG00000047314 | MHC class I antigen YF5 | -1.74 | 0.7700 | 55.9557 | 7.41E-14 | 2.54E-11 |
| LOC112531946 | - - - - - | withdrawn | -1.74 | -0.9087 | 17.5967 | 2.73E-05 | 7.27E-04 |
| LOC107052216 | - - - - - | uncharacterized LOC107052216 | -1.72 | 0.8875 | 33.0242 | 9.10E-09 | 7.97E-07 |
| LOC107049103 | - - - - - | proline-rich protein 36-like | -1.71 | -0.4585 | 23.4500 | 1.28E-06 | 5.84E-05 |
| LOC101748467 | - - - - - | G2/M phase-specific E3 ubiquitin-protein ligase-like | -1.69 | -0.7904 | 15.1993 | 9.67E-05 | 2.02E-03 |
| LOC107055072 | - - - - - | uncharacterized LOC107055072 | -1.68 | -0.6072 | 22.5898 | 2.01E-06 | 8.48E-05 |
| LOC112531094 | - - - - - | withdrawn | -1.67 | 0.0306 | 39.2369 | 3.75E-10 | 4.68E-08 |
| LOC112533488 | - - - - - | uncharacterized LOC112533488 | -1.66 | 0.1181 | 36.1457 | 1.83E-09 | 1.91E-07 |
| LOC107051988 | - - - - - | withdrawn | -1.66 | -0.5014 | 18.1208 | 2.07E-05 | 5.78E-04 |
| LOC107053477 | - - - - - | withdrawn | -1.64 | 4.1892 | 22.4785 | 2.13E-06 | 8.90E-05 |
| LOC107052164 | - - - - - | uncharacterized LOC107052164 | -1.64 | -0.6131 | 22.0547 | 2.65E-06 | 1.07E-04 |
| LOC107053870 | - - - - - | translation initiation factor IF-2-like | -1.63 | 2.4895 | 10.5706 | 1.15E-03 | 1.33E-02 |
| LOC101748511 | - - - - - | uncharacterized LOC101748511 | -1.62 | 0.5462 | 28.3316 | 1.02E-07 | 6.89E-06 |
| HIST1H2A4L3 | ENSGALG00000032645 | histone cluster 1, H2A-IV-like 3 | -1.61 | -0.4712 | 28.8464 | 7.84E-08 | 5.49E-06 |
| LOC101750546 | - - - - - | circumsporozoite protein-like | -1.61 | 0.3854 | 33.1317 | 8.61E-09 | 7.58E-07 |
| LOC112531945 | - - - - - | uncharacterized LOC112531945 | -1.61 | 0.2160 | 13.5177 | 2.36E-04 | 4.10E-03 |
| CDKN1A | ENSGALG00000028318 | cyclin dependent kinase inhibitor 1A | -1.60 | 0.9699 | 12.4588 | 4.16E-04 | 6.30E-03 |
| C1QC | ENSGALG00000029669 | complement C1q C chain | -1.60 | 0.5115 | 16.6195 | 4.57E-05 | 1.11E-03 |
| LOC101750035 | - - - - - | G2/M phase-specific E3 ubiquitin-protein ligase-like | -1.59 | 1.2593 | 63.2869 | 1.79E-15 | 8.17E-13 |
| MYH13 | - - - - - | myosin, heavy chain 13, skeletal muscle | -1.59 | 0.2335 | 11.6420 | 6.45E-04 | 8.74E-03 |
| LOC107053886 | - - - - - | withdrawn | -1.57 | 2.3576 | 49.7580 | 1.74E-12 | 4.09E-10 |
| LOC107053584 | - - - - - | uncharacterized LOC107053584 | -1.56 | -0.4039 | 21.1381 | 4.27E-06 | 1.62E-04 |
| LOC101749515 | - - - - - | withdrawn | -1.56 | 0.3308 | 41.6956 | 1.07E-10 | 1.60E-08 |
| LOC101749127 | - - - - - | uncharacterized LOC101749127 | -1.55 | -0.4074 | 20.7914 | 5.12E-06 | 1.89E-04 |
| LOC107055074 | - - - - - | withdrawn | -1.54 | 0.2841 | 20.6338 | 5.56E-06 | 2.03E-04 |
| LOC101752000 | - - - - - | uncharacterized LOC101752000 | -1.52 | 0.1583 | 34.8831 | 3.50E-09 | 3.47E-07 |
| FADS1L1 | - - - - - | fatty acid desaturase 1-like | -1.52 | 0.7620 | 19.1620 | 1.20E-05 | 3.75E-04 |
| LOC112531034 | - - - - - | withdrawn | -1.50 | 1.7884 | 21.4520 | 3.63E-06 | 1.40E-04 |
| C1QA | ENSGALG00000021569 | complement C1q A chain | -1.50 | 0.8949 | 20.4098 | 6.25E-06 | 2.22E-04 |
| UROC1 | ENSGALG00000006306 | urocanate hydratase 1 | -1.49 | 2.3029 | 39.7228 | 2.93E-10 | 3.82E-08 |
| LOC107055024 | - - - - - | uncharacterized LOC107055024 | -1.49 | 0.8652 | 46.2974 | 1.02E-11 | 1.94E-09 |
| CD44 | ENSGALG00000039080 | CD44 molecule (Indian blood group) | -1.49 | 1.7337 | 14.0232 | 1.81E-04 | 3.29E-03 |
| ACKR4 | ENSGALG00000041491 | atypical chemokine receptor 4 | -1.49 | -0.1858 | 27.9939 | 1.22E-07 | 8.07E-06 |
| LOC112533411 | - - - - - | protocadherin beta-15-like | -1.48 | -0.6070 | 15.2921 | 9.21E-05 | 1.94E-03 |
| LOC107054516 | - - - - - | uncharacterized LOC107054516 | -1.47 | 0.1094 | 19.7021 | 9.05E-06 | 2.99E-04 |

| | | | | | | | |
|---|---|---|---|---|---|---|---|
| LOC107053122 | - - - - - | endogenous retrovirus group K member 18 Pol protein-like | -1.46 | 1.3590 | 15.4499 | 8.47E-05 | 1.82E-03 |
| VCAM1 | ENSGALG00000005257 | vascular cell adhesion molecule 1 | -1.46 | -0.1018 | 15.6032 | 7.81E-05 | 1.72E-03 |
| LOC112532661 | - - - - - | uncharacterized LOC112532661 | -1.45 | 0.4106 | 37.6133 | 8.63E-10 | 9.79E-08 |
| C14orf180 | ENSGALG00000053336 | chromosome 14 open reading frame 180 | -1.45 | -0.4925 | 9.2377 | 2.37E-03 | 2.25E-02 |
| C1QB | ENSGALG00000004771 | complement C1q B chain | -1.44 | 0.6999 | 14.5875 | 1.34E-04 | 2.58E-03 |
| LOC112532651 | - - - - - | uncharacterized LOC112532651 | -1.43 | -0.9547 | 14.4186 | 1.46E-04 | 2.78E-03 |
| LOC100859625 | - - - - - | uncharacterized LOC100859625 | -1.43 | 1.3269 | 26.1392 | 3.18E-07 | 1.83E-05 |
| LOC112532141 | - - - - - | withdrawn | -1.43 | 2.1305 | 10.7123 | 1.06E-03 | 1.26E-02 |
| LOC112530090 | - - - - - | small nucleolar RNA SNORD49 | -1.42 | 0.0995 | 17.2964 | 3.20E-05 | 8.22E-04 |
| LOC107050560 | - - - - - | protocadherin beta-15-like | -1.41 | -0.1443 | 8.7364 | 3.12E-03 | 2.71E-02 |
| LOC112531208 | - - - - - | withdrawn | -1.41 | 3.4992 | 22.1792 | 2.48E-06 | 1.02E-04 |
| CERS4L | - - - - - | ceramide synthase 4-like | -1.40 | -0.9479 | 15.1877 | 9.73E-05 | 2.02E-03 |
| LOC107051926 | - - - - - | uncharacterized LOC107051926 | -1.39 | 2.3194 | 43.5600 | 4.11E-11 | 6.82E-09 |
| LOC107051856 | - - - - - | withdrawn | -1.38 | -0.8604 | 11.1092 | 8.59E-04 | 1.07E-02 |
| GUCA1C | ENSGALG00000015364 | guanylate cyclase activator 1C | -1.37 | 1.9929 | 11.9129 | 5.57E-04 | 7.88E-03 |
| LOC107050024 | - - - - - | withdrawn | -1.37 | 2.1710 | 31.1484 | 2.39E-08 | 1.90E-06 |
| LOC107054094 | - - - - - | withdrawn | -1.35 | -0.6452 | 10.8485 | 9.89E-04 | 1.19E-02 |
| LOC112531155 | - - - - - | withdrawn | -1.35 | 5.4080 | 124.2262 | 7.52E-29 | 1.37E-25 |
| LOC107049730 | - - - - - | protocadherin beta-15-like | -1.34 | 1.3557 | 20.3810 | 6.35E-06 | 2.25E-04 |
| LOC107051715 | - - - - - | uncharacterized LOC107051715 | -1.34 | -0.5309 | 16.2199 | 5.64E-05 | 1.32E-03 |
| LOC112532459 | - - - - - | small nucleolar RNA SNORA26 | -1.34 | 0.2309 | 14.6360 | 1.30E-04 | 2.55E-03 |
| NAT8 | ENSGALG00000050294 | N-acetyltransferase 8 (GCN5-related, putative) | -1.33 | 1.8156 | 40.2256 | 2.26E-10 | 3.10E-08 |
| LOC112530111 | - - - - - | uncharacterized LOC112530111 | -1.33 | 0.7827 | 23.8761 | 1.03E-06 | 4.87E-05 |
| LOC101751866 | - - - - - | uncharacterized LOC101751866 | -1.33 | -0.0816 | 23.5966 | 1.19E-06 | 5.48E-05 |
| ACHE | ENSGALG00000047182 | acetylcholinesterase (Cartwright blood group) | -1.33 | 4.7552 | 82.3940 | 1.11E-19 | 8.74E-17 |
| LOC107054189 | - - - - - | uncharacterized LOC107054189 | -1.33 | -0.5886 | 12.9260 | 3.24E-04 | 5.21E-03 |
| LOC107054055 | - - - - - | withdrawn | -1.32 | 1.2740 | 28.6882 | 8.50E-08 | 5.83E-06 |
| LOC112533200 | - - - - - | withdrawn | -1.32 | 0.8788 | 22.0806 | 2.61E-06 | 1.06E-04 |
| LOC107057188 | - - - - - | borealin-like | -1.32 | 5.8994 | 48.9107 | 2.68E-12 | 6.12E-10 |
| TIMD4 | ENSGALG00000003876 | T-cell immunoglobulin and mucin domain containing 4 | -1.32 | 0.6782 | 21.0526 | 4.47E-06 | 1.68E-04 |
| LOC107054707 | - - - - - | withdrawn | -1.32 | 1.4918 | 31.7419 | 1.76E-08 | 1.47E-06 |
| PFKFB4 | ENSGALG00000032385 | 6-phosphofructo-2-kinase/fructose-2,6-biphosphatase 4 | -1.31 | 1.7097 | 36.5918 | 1.46E-09 | 1.59E-07 |
| LOC107049676 | - - - - - | uncharacterized LOC107049676 | -1.31 | -0.3008 | 7.3558 | 6.68E-03 | 4.68E-02 |
| LOC101748554 | - - - - - | uncharacterized LOC101748554 | -1.31 | -0.6596 | 15.0139 | 1.07E-04 | 2.16E-03 |
| TOR1BL | ENSGALG00000023709 | torsin family 1, member B-like | -1.31 | -0.7463 | 14.1319 | 1.70E-04 | 3.15E-03 |
| LOC107052166 | - - - - - | uncharacterized LOC107052166 | -1.31 | -0.6254 | 15.5180 | 8.17E-05 | 1.78E-03 |
| LOC101748762 | - - - - - | uncharacterized LOC101748762 | -1.30 | -0.1021 | 20.4451 | 6.14E-06 | 2.20E-04 |
| CCL26 | ENSGALG00000014585 | C-C motif chemokine ligand 26 | -1.30 | 0.0391 | 17.7663 | 2.50E-05 | 6.75E-04 |
| LOC112532055 | - - - - - | uncharacterized LOC112532055 | -1.29 | 0.3688 | 22.3064 | 2.32E-06 | 9.66E-05 |
| LOC112532631 | - - - - - | uncharacterized LOC112532631 | -1.29 | -0.0652 | 9.3475 | 2.23E-03 | 2.17E-02 |
| KRT9L | - - - - - | keratin, type I cytoskeletal 9-like | -1.29 | 0.1826 | 29.6588 | 5.15E-08 | 3.80E-06 |
| TLX1 | ENSGALG00000007831 | T-cell leukemia homeobox 1 | -1.28 | -0.1156 | 10.0592 | 1.52E-03 | 1.63E-02 |
| LOC101747910 | - - - - - | uncharacterized LOC101747910 | -1.26 | 1.0686 | 51.8017 | 6.14E-13 | 1.55E-10 |
| LOC112531200 | - - - - - | withdrawn | -1.26 | 0.0957 | 18.8667 | 1.40E-05 | 4.26E-04 |

| | | | | | | | |
|---|---|---|---|---|---|---|---|
| OGN | ENSGALG00000026736 | osteoglycin | -1.26 | 3.3039 | 18.0957 | 2.10E-05 | 5.84E-04 |
| IP6K3 | ENSGALG00000026536 | inositol hexakisphosphate kinase 3 | -1.26 | 0.2558 | 21.8882 | 2.89E-06 | 1.15E-04 |
| GLRA4 | ENSGALG00000004936 | glycine receptor alpha 4 | -1.26 | 1.8450 | 30.5518 | 3.25E-08 | 2.51E-06 |
| ZP3L1 | - - - - - | zona pellucida sperm-binding protein 3-like | -1.26 | -0.1198 | 20.1940 | 7.00E-06 | 2.43E-04 |
| LOC107053858 | - - - - - | uncharacterized LOC107053858 | -1.26 | -0.8405 | 11.0724 | 8.76E-04 | 1.09E-02 |
| PHF24 | ENSGALG00000001900 | PHD finger protein 24 | -1.25 | 0.8339 | 17.4179 | 3.00E-05 | 7.85E-04 |
| ECM2 | ENSGALG00000004695 | extracellular matrix protein 2 | -1.25 | 3.0014 | 44.0048 | 3.28E-11 | 5.61E-09 |
| NRXN3 | ENSGALG00000027255 | neurexin 3 | -1.25 | 2.7336 | 35.5530 | 2.48E-09 | 2.52E-07 |
| HAL | ENSGALG00000011421 | histidine ammonia-lyase | -1.25 | 2.0860 | 27.2704 | 1.77E-07 | 1.11E-05 |
| DRD2 | ENSGALG00000007794 | dopamine receptor D2 | -1.25 | -0.0675 | 17.0046 | 3.73E-05 | 9.40E-04 |
| LOC112533486 | - - - - - | withdrawn | -1.25 | 1.2086 | 30.7156 | 2.99E-08 | 2.32E-06 |
| SHOX | ENSGALG00000016698 | short stature homeobox | -1.24 | 1.0476 | 7.1853 | 7.35E-03 | 5.00E-02 |
| LOC107049937 | - - - - - | solute carrier family 2, facilitated glucose transporter member 4-like | -1.24 | 0.5272 | 23.2300 | 1.44E-06 | 6.42E-05 |
| MROH2BL2 | - - - - - | maestro heat-like repeat-containing protein family member 2B | -1.23 | -0.8546 | 9.9854 | 1.58E-03 | 1.68E-02 |
| LOC112530427 | - - - - - | withdrawn | -1.23 | 3.1434 | 10.5478 | 1.16E-03 | 1.34E-02 |
| LOC112532894 | - - - - - | uncharacterized LOC112532894 | -1.23 | 2.0193 | 36.6773 | 1.39E-09 | 1.54E-07 |
| TMEM61 | ENSGALG00000010801 | transmembrane protein 61 | -1.23 | 2.2488 | 14.6135 | 1.32E-04 | 2.56E-03 |
| LOC112533473 | - - - - - | withdrawn | -1.23 | -0.5365 | 14.6277 | 1.31E-04 | 2.55E-03 |
| LOC107049565 | - - - - - | uncharacterized LOC107049565 | -1.21 | 1.1272 | 22.8171 | 1.78E-06 | 7.72E-05 |
| TDRD5 | ENSGALG00000033634 | tudor domain containing 5 | -1.21 | 0.3269 | 16.3951 | 5.14E-05 | 1.22E-03 |
| LOC112531252 | - - - - - | withdrawn | -1.21 | 1.3395 | 11.8205 | 5.86E-04 | 8.12E-03 |
| FFAR4 | ENSGALG00000026733 | free fatty acid receptor 4 | -1.21 | -0.7963 | 12.6217 | 3.81E-04 | 5.90E-03 |
| LOC112533409 | - - - - - | withdrawn | -1.21 | 1.0258 | 19.4386 | 1.04E-05 | 3.35E-04 |
| LOC107054197 | - - - - - | withdrawn | -1.20 | 0.0800 | 18.6578 | 1.56E-05 | 4.65E-04 |
| LOC101748439 | - - - - - | uncharacterized LOC101748439 | -1.20 | 2.4470 | 13.4069 | 2.51E-04 | 4.31E-03 |
| LOC107057093 | - - - - - | c-type lectin like 17, MHCY region | -1.20 | -0.3664 | 17.3489 | 3.11E-05 | 8.09E-04 |
| LOC101748259 | - - - - - | uncharacterized LOC101748259 | -1.20 | 2.2407 | 14.4520 | 1.44E-04 | 2.75E-03 |
| SLC6A19 | ENSGALG00000019527 | solute carrier family 6 member 19 | -1.20 | 0.0204 | 19.1867 | 1.19E-05 | 3.74E-04 |
| LOC101751481 | - - - - - | withdrawn | -1.20 | -0.6135 | 11.3453 | 7.56E-04 | 9.80E-03 |
| LOC112531018 | - - - - - | withdrawn | -1.20 | 1.0949 | 20.3118 | 6.58E-06 | 2.31E-04 |
| CAPN13 | ENSGALG00000020084 | calpain 13 | -1.19 | -0.6607 | 10.5451 | 1.16E-03 | 1.34E-02 |
| LOC425214 | - - - - - | withdrawn | -1.19 | 2.1665 | 29.4373 | 5.78E-08 | 4.21E-06 |
| LRIT2 | ENSGALG00000002307 | leucine rich repeat, Ig-like and transmembrane domains 2 | -1.19 | -0.0662 | 7.8774 | 5.01E-03 | 3.83E-02 |
| LOC100859202 | - - - - - | c-type lectin like 17, MHCY region | -1.19 | 0.0656 | 15.4128 | 8.64E-05 | 1.85E-03 |
| LOC100857473 | - - - - - | alpha-1,2-mannosyltransferase ALG9-like | -1.18 | 2.0710 | 49.1459 | 2.38E-12 | 5.51E-10 |
| TLR7 | ENSGALG00000016590 | toll like receptor 7 | -1.18 | 0.1917 | 9.6154 | 1.93E-03 | 1.96E-02 |
| LOC769128 | - - - - - | withdrawn | -1.18 | -0.3311 | 16.2212 | 5.64E-05 | 1.32E-03 |
| LOC107054803 | - - - - - | withdrawn | -1.17 | -0.5491 | 8.9354 | 2.80E-03 | 2.51E-02 |
| WISP1 | ENSGALG00000030115 | WNT1 inducible signaling pathway protein 1 | -1.17 | 0.6096 | 15.1615 | 9.87E-05 | 2.04E-03 |
| CNGA3 | ENSGALG00000039826 | cyclic nucleotide gated channel alpha 3 | -1.17 | 3.7080 | 24.6941 | 6.72E-07 | 3.47E-05 |
| LOC107051325 | - - - - - | uncharacterized LOC107051325 | -1.17 | -0.1867 | 12.9022 | 3.28E-04 | 5.26E-03 |
| LOC107054433 | - - - - - | uncharacterized LOC107054433 | -1.17 | 1.2119 | 18.5614 | 1.65E-05 | 4.84E-04 |
| YF6 | - - - - - | major histocompatibility complex Y, class I heavy chain 4 | -1.16 | 2.1438 | 36.4422 | 1.57E-09 | 1.70E-07 |
| LOC101748035 | - - - - - | uncharacterized LOC101748035 | -1.16 | 0.1557 | 20.5678 | 5.76E-06 | 2.08E-04 |

| | | | | | | | |
|---|---|---|---|---|---|---|---|
| LOC107052408 | - - - - - | uncharacterized LOC107052408 | -1.16 | 1.4291 | 15.8253 | 6.95E-05 | 1.57E-03 |
| FAM135B | ENSGALG00000039831 | family with sequence similarity 135 member B | -1.15 | -0.5442 | 8.4234 | 3.70E-03 | 3.09E-02 |
| LOC107055022 | - - - - - | uncharacterized LOC107055022 | -1.15 | -0.5479 | 9.4654 | 2.09E-03 | 2.08E-02 |
| LOC101750579 | - - - - - | uncharacterized LOC101750579 | -1.14 | 1.3095 | 18.5637 | 1.64E-05 | 4.84E-04 |
| LOC100859665 | - - - - - | uncharacterized LOC100859665 | -1.14 | -0.4256 | 11.1401 | 8.45E-04 | 1.06E-02 |
| ANXA13 | ENSGALG00000036418 | annexin A13 | -1.14 | -0.4609 | 10.5486 | 1.16E-03 | 1.34E-02 |
| LOC112531893 | - - - - - | withdrawn | -1.13 | 4.8767 | 67.9387 | 1.69E-16 | 8.41E-14 |
| TSHR | ENSGALG00000010572 | thyroid stimulating hormone receptor | -1.13 | -0.3684 | 11.4443 | 7.17E-04 | 9.44E-03 |
| BG8 | ENSGALG00000049461 | MHC B-G antigen | -1.13 | 3.8175 | 66.9738 | 2.75E-16 | 1.33E-13 |
| DIRAS2 | ENSGALG00000005323 | DIRAS family GTPase 2 | -1.13 | 0.6415 | 10.1380 | 1.45E-03 | 1.58E-02 |
| FRMPD3 | ENSGALG00000028975 | FERM and PDZ domain containing 3 | -1.12 | 2.5916 | 12.0915 | 5.07E-04 | 7.33E-03 |
| LOC101751606 | - - - - - | withdrawn | -1.12 | -0.6816 | 9.8348 | 1.71E-03 | 1.78E-02 |
| LOC107054464 | - - - - - | withdrawn | -1.12 | 0.4291 | 9.1950 | 2.43E-03 | 2.28E-02 |
| COR8 | - - - - - | olfactory receptor family 5 subfamily J member 2 | -1.12 | 0.4188 | 11.5562 | 6.75E-04 | 9.05E-03 |
| LOC112530987 | - - - - - | trichohyalin-like | -1.12 | 1.4065 | 33.4620 | 7.27E-09 | 6.53E-07 |
| LOC100857439 | - - - - - | histone H3.2-like | -1.11 | -0.7538 | 11.3204 | 7.67E-04 | 9.88E-03 |
| SH3RF2 | ENSGALG00000043904 | SH3 domain containing ring finger 2 | -1.11 | -0.2390 | 17.0165 | 3.71E-05 | 9.35E-04 |
| LOC101747844 | - - - - - | carbohydrate sulfotransferase 9-like | -1.11 | 1.8658 | 16.9590 | 3.82E-05 | 9.60E-04 |
| THRB | ENSGALG00000011294 | thyroid hormone receptor beta | -1.10 | 1.8904 | 8.4576 | 3.64E-03 | 3.05E-02 |
| TDRD15 | ENSGALG00000016492 | tudor domain containing 15 | -1.09 | 0.7286 | 11.1719 | 8.30E-04 | 1.05E-02 |
| LOC101750063 | - - - - - | uncharacterized LOC101750063 | -1.09 | 0.8986 | 14.2687 | 1.58E-04 | 2.96E-03 |
| MOG | ENSGALG00000022875 | myelin oligodendrocyte glycoprotein | -1.09 | 0.9298 | 15.0505 | 1.05E-04 | 2.14E-03 |
| LDHD | ENSGALG00000048399 | lactate dehydrogenase D | -1.09 | 2.1942 | 16.1696 | 5.79E-05 | 1.35E-03 |
| PTER | ENSGALG00000008728 | phosphotriesterase related | -1.09 | 4.7433 | 77.1523 | 1.58E-18 | 1.04E-15 |
| LOC101747727 | - - - - - | uncharacterized LOC101747727 | -1.08 | 2.1762 | 27.4037 | 1.65E-07 | 1.05E-05 |
| LOC112533302 | - - - - - | withdrawn | -1.08 | -0.1280 | 12.1028 | 5.03E-04 | 7.30E-03 |
| LOC416951 | - - - - - | uncharacterized LOC416951 | -1.08 | -0.6478 | 8.4652 | 3.62E-03 | 3.03E-02 |
| LOC107051082 | - - - - - | withdrawn | -1.08 | 6.6021 | 54.2742 | 1.74E-13 | 5.74E-11 |
| LOC101751655 | - - - - - | uncharacterized LOC101751655 | -1.07 | -0.0644 | 13.4674 | 2.43E-04 | 4.20E-03 |
| TRPM2 | ENSGALG00000005631 | transient receptor potential cation channel subfamily M member 2 | -1.07 | 2.5581 | 25.8265 | 3.74E-07 | 2.12E-05 |
| LOC112532477 | - - - - - | fibrinogen-like protein 1-like protein | -1.07 | 1.0951 | 27.0347 | 2.00E-07 | 1.22E-05 |
| LOC107053147 | - - - - - | uncharacterized LOC107053147 | -1.07 | 0.2723 | 8.3908 | 3.77E-03 | 3.12E-02 |
| LOC112533187 | - - - - - | withdrawn | -1.07 | 1.5061 | 16.1902 | 5.73E-05 | 1.34E-03 |
| LRIT1 | ENSGALG00000002303 | leucine rich repeat, Ig-like and transmembrane domains 1 | -1.07 | 2.3944 | 7.4108 | 6.48E-03 | 4.60E-02 |
| LOC107054716 | - - - - - | uncharacterized LOC107054716 | -1.07 | -0.9416 | 9.2358 | 2.37E-03 | 2.25E-02 |
| TMC3 | ENSGALG00000006376 | transmembrane channel like 3 | -1.06 | 2.2190 | 17.3176 | 3.16E-05 | 8.17E-04 |
| LOC101748916 | - - - - - | extensin-like | -1.06 | 6.2215 | 88.3271 | 5.55E-21 | 5.07E-18 |
| HIST1H2A3 | ENSGALG00000049268 | histone cluster 1, H2A, III | -1.06 | 0.0473 | 11.3444 | 7.57E-04 | 9.80E-03 |
| PROX2 | - - - - - | prospero homeobox 2 | -1.06 | 0.5407 | 18.9468 | 1.34E-05 | 4.11E-04 |
| TBX15 | ENSGALG00000014776 | T-box 15 | -1.05 | 4.9235 | 9.9786 | 1.58E-03 | 1.69E-02 |
| LOC107053136 | - - - - - | uncharacterized LOC107053136 | -1.05 | -0.7293 | 7.8612 | 5.05E-03 | 3.84E-02 |
| CMYA5 | ENSGALG00000026553 | cardiomyopathy associated 5 | -1.05 | 1.1028 | 11.3136 | 7.69E-04 | 9.90E-03 |
| MSX2 | ENSGALG00000038848 | msh homeobox 2 | -1.05 | 4.2172 | 9.6171 | 1.93E-03 | 1.96E-02 |
| MR1 | - - - - - | major histocompatibility complex Y, class I heavy chain like | -1.05 | 2.0465 | 24.6020 | 7.05E-07 | 3.61E-05 |

| | | | | | | | |
|---|---|---|---|---|---|---|---|
| CD300LG | ENSGALG00000007713 | CD300 molecule like family member g | -1.04 | 1.7064 | 41.2373 | 1.35E-10 | 1.98E-08 |
| LOC107052834 | - - - - - | withdrawn | -1.04 | 2.3910 | 14.5560 | 1.36E-04 | 2.62E-03 |
| LOC112531637 | - - - - - | small nucleolar RNA SNORA40 | -1.03 | 0.7744 | 13.3099 | 2.64E-04 | 4.49E-03 |
| TMEM8C | ENSGALG00000021520 | transmembrane protein 8C | -1.03 | -0.0793 | 9.8864 | 1.67E-03 | 1.75E-02 |
| LOC107052588 | - - - - - | withdrawn | -1.03 | 0.5329 | 11.7805 | 5.99E-04 | 8.27E-03 |
| TNFRSF10B | ENSGALG00000000452 | tumor necrosis factor receptor superfamily, member 10b | -1.03 | 3.0715 | 21.7413 | 3.12E-06 | 1.24E-04 |
| LOC112530989 | - - - - - | periodic tryptophan protein 2 homolog | -1.03 | -0.1656 | 7.4942 | 6.19E-03 | 4.45E-02 |
| UBXN11 | ENSGALG00000054685 | UBX domain protein 11 | -1.03 | 0.4344 | 18.5650 | 1.64E-05 | 4.84E-04 |
| FMO3 | ENSGALG00000040573 | flavin containing monooxygenase 3 | -1.02 | 0.3124 | 14.0714 | 1.76E-04 | 3.24E-03 |
| LOC112530944 | - - - - - | withdrawn | -1.02 | 1.1667 | 15.3056 | 9.14E-05 | 1.93E-03 |
| CX3CR1 | ENSGALG00000038000 | C-X3-C motif chemokine receptor 1 | -1.02 | -0.3079 | 9.0877 | 2.57E-03 | 2.37E-02 |
| SYTL2L | ENSGALG00000008326 | synaptotagmin-like protein 2-like | -1.01 | 0.8700 | 18.8265 | 1.43E-05 | 4.34E-04 |
| TMEM163 | ENSGALG00000012190 | transmembrane protein 163 | -1.01 | 2.5935 | 24.3814 | 7.90E-07 | 3.92E-05 |
| LOC112533550 | - - - - - | withdrawn | -1.01 | -0.5442 | 8.3024 | 3.96E-03 | 3.22E-02 |
| LOC112531154 | - - - - - | withdrawn | -1.01 | 3.6098 | 31.3933 | 2.11E-08 | 1.72E-06 |
| TGM3 | ENSGALG00000004804 | transglutaminase 3 | -1.01 | 0.0482 | 11.1602 | 8.36E-04 | 1.06E-02 |
| CDH10 | ENSGALG00000032022 | cadherin 10 | -1.01 | 1.3337 | 9.8263 | 1.72E-03 | 1.79E-02 |
| LOC101748470 | - - - - - | uncharacterized LOC101748470 | -1.01 | 0.2654 | 15.5571 | 8.01E-05 | 1.75E-03 |
| NDST4 | ENSGALG00000012015 | N-deacetylase and N-sulfotransferase 4 | -1.01 | 0.9871 | 23.3553 | 1.35E-06 | 6.09E-05 |
| CHRNG | ENSGALG00000007906 | cholinergic receptor nicotinic gamma subunit | -1.00 | 1.9579 | 19.2824 | 1.13E-05 | 3.60E-04 |
| ANKK1 | ENSGALG00000007815 | ankyrin repeat and kinase domain containing 1 | -1.00 | 0.9434 | 10.7645 | 1.03E-03 | 1.23E-02 |
| LOC107051247 | - - - - - | uncharacterized LOC107051247 | -0.99 | 0.5967 | 14.2528 | 1.60E-04 | 2.98E-03 |
| CHRNA10 | ENSGALG00000022702 | cholinergic receptor, nicotinic, alpha 10 (neuronal) | -0.99 | 0.9824 | 10.9502 | 9.36E-04 | 1.15E-02 |
| IGSF5 | ENSGALG00000016132 | immunoglobulin superfamily member 5 | -0.99 | -0.4849 | 10.4920 | 1.20E-03 | 1.37E-02 |
| MYL2 | ENSGALG00000004582 | myosin, light chain 2, regulatory, cardiac, slow | -0.99 | 2.3645 | 10.5889 | 1.14E-03 | 1.32E-02 |
| LOC771200 | - - - - - | uncharacterized LOC771200 | -0.99 | 2.4641 | 23.9572 | 9.85E-07 | 4.76E-05 |
| LOC107054020 | - - - - - | uncharacterized LOC107054020 | -0.99 | -0.1544 | 7.3347 | 6.76E-03 | 4.72E-02 |
| C5H14orf159 | ENSGALG00000010703 | chromosome 5 C14orf159 homolog | -0.99 | 3.1955 | 62.7268 | 2.37E-15 | 1.00E-12 |
| USH1G | ENSGALG00000038769 | USH1 protein network component sans | -0.99 | -0.8892 | 7.7667 | 5.32E-03 | 3.99E-02 |
| NCAM2 | ENSGALG00000015737 | neural cell adhesion molecule 2 | -0.98 | 2.0462 | 11.5257 | 6.86E-04 | 9.15E-03 |
| FSHR | ENSGALG00000009100 | follicle stimulating hormone receptor | -0.98 | 0.5245 | 8.8798 | 2.88E-03 | 2.58E-02 |
| LOC107049782 | - - - - - | uncharacterized LOC107049782 | -0.98 | 0.5337 | 10.4243 | 1.24E-03 | 1.40E-02 |
| ADMP | ENSGALG00000037408 | anti-dorsalizing morphogenetic protein | -0.97 | -0.8098 | 7.8708 | 5.02E-03 | 3.83E-02 |
| LOC112531854 | - - - - - | uncharacterized LOC112531854 | -0.97 | 1.4673 | 19.6333 | 9.38E-06 | 3.08E-04 |
| PLSCR5 | ENSGALG00000006807 | phospholipid scramblase family member 5 | -0.97 | 1.7322 | 24.2189 | 8.60E-07 | 4.25E-05 |
| LOC416695 | - - - - - | tubulin alpha-3 chain-like | -0.97 | 2.4335 | 35.2768 | 2.86E-09 | 2.87E-07 |
| SIRPA | ENSGALG00000025778 | signal-regulatory protein alpha | -0.97 | 2.0011 | 20.1774 | 7.06E-06 | 2.45E-04 |
| LOC101747373 | - - - - - | uncharacterized LOC101747373 | -0.96 | 4.7909 | 72.9961 | 1.30E-17 | 7.13E-15 |
| NRSN2 | - - - - - | neurensin 2 | -0.96 | -0.6441 | 8.0680 | 4.51E-03 | 3.54E-02 |
| C31H17orf49 | - - - - - | chromosome unknown C17orf49 homolog | -0.96 | 5.4093 | 127.5499 | 1.41E-29 | 2.90E-26 |
| DLX3 | ENSGALG00000040529 | distal-less homeobox 3 | -0.96 | 1.4655 | 7.9152 | 4.90E-03 | 3.77E-02 |
| ABCA13 | - - - - - | ATP-binding cassette, sub-family A (ABC1), member 13 | -0.95 | -0.6732 | 8.9546 | 2.77E-03 | 2.49E-02 |
| LOC107055338 | - - - - - | uncharacterized LOC107055338 | -0.95 | -0.3273 | 9.0576 | 2.62E-03 | 2.40E-02 |
| AOAH | ENSGALG00000035505 | acyloxyacyl hydrolase | -0.95 | 1.9665 | 13.3487 | 2.59E-04 | 4.42E-03 |

| | | | | | | | |
|---|---|---|---|---|---|---|---|
| ADAM5P | ENSGALG00000009606 | ADAM metallopeptidase domain 5, pseudogene | -0.95 | 1.6585 | 33.5602 | 6.91E-09 | 6.25E-07 |
| LOC101749363 | - - - - - | uncharacterized LOC101749363 | -0.95 | 4.3702 | 34.1263 | 5.16E-09 | 4.86E-07 |
| LOC101750312 | - - - - - | uncharacterized LOC101750312 | -0.95 | 1.2905 | 12.7718 | 3.52E-04 | 5.54E-03 |
| LOC112532770 | - - - - - | putative methyltransferase DDB_G0268948 | -0.95 | 4.8362 | 44.4035 | 2.67E-11 | 4.68E-09 |
| MB | ENSGALG00000012541 | myoglobin | -0.95 | 0.8093 | 7.4822 | 6.23E-03 | 4.47E-02 |
| AKR1D1 | ENSGALG00000012834 | aldo-keto reductase family 1 member D1 | -0.95 | 5.8071 | 47.1968 | 6.42E-12 | 1.34E-09 |
| LOC112531894 | - - - - - | VPS28 subunit of ESCRT-I | -0.95 | 6.0543 | 167.4991 | 2.60E-38 | 1.07E-34 |
| LOC107051491 | - - - - - | withdrawn | -0.94 | 4.3215 | 25.8916 | 3.61E-07 | 2.06E-05 |
| LOC112530177 | - - - - - | uncharacterized LOC112530177 | -0.94 | -0.5097 | 8.7506 | 3.09E-03 | 2.70E-02 |
| PXDNL | ENSGALG00000036838 | peroxidasin like | -0.94 | 2.7196 | 10.5981 | 1.13E-03 | 1.32E-02 |
| NPBWR2 | ENSGALG00000029170 | neuropeptides B and W receptor 2 | -0.94 | -0.2248 | 9.2074 | 2.41E-03 | 2.27E-02 |
| LOC112529916 | - - - - - | small nucleolar SNORD12/SNORD106 | -0.94 | 1.1979 | 19.4765 | 1.02E-05 | 3.29E-04 |
| LOC112532269 | - - - - - | uncharacterized LOC112532269 | -0.94 | 0.2725 | 16.0060 | 6.31E-05 | 1.45E-03 |
| CRB1 | ENSGALG00000002287 | crumbs cell polarity complex component 1 | -0.93 | 2.1877 | 14.3448 | 1.52E-04 | 2.86E-03 |
| LOC112532647 | - - - - - | uncharacterized LOC112532647 | -0.93 | 0.7096 | 14.3633 | 1.51E-04 | 2.84E-03 |
| LDLRAD1 | ENSGALG00000020647 | low density lipoprotein receptor class A domain containing 1 | -0.93 | 3.0225 | 32.6531 | 1.10E-08 | 9.54E-07 |
| LOC100859819 | - - - - - | cocaine- and amphetamine-regulated transcript protein-like | -0.93 | 0.6455 | 15.5078 | 8.22E-05 | 1.78E-03 |
| LOC101748820 | - - - - - | uncharacterized LOC101748820 | -0.93 | 1.9421 | 8.8674 | 2.90E-03 | 2.58E-02 |
| DLGAP1L | - - - - - | ----- | -0.93 | -0.3793 | 9.9291 | 1.63E-03 | 1.72E-02 |
| LOC101747354 | - - - - - | alanine and glycine-rich protein-like | -0.93 | -0.6566 | 8.3417 | 3.87E-03 | 3.17E-02 |
| LOC107054704 | - - - - - | major histocompatibility complex Y, class I heavy chain 36 | -0.93 | 1.6416 | 16.1938 | 5.72E-05 | 1.33E-03 |
| CPXM2 | - - - - - | carboxypeptidase X, M14 family member 2 | -0.93 | 0.6144 | 8.6400 | 3.29E-03 | 2.82E-02 |
| PTPRQ | ENSGALG00000010935 | protein tyrosine phosphatase, receptor type Q | -0.92 | 4.6318 | 25.5327 | 4.35E-07 | 2.40E-05 |
| LOC101747644 | - - - - - | uncharacterized LOC101747644 | -0.92 | -0.7038 | 8.8103 | 3.00E-03 | 2.64E-02 |
| TACR1 | ENSGALG00000036638 | tachykinin receptor 1 | -0.92 | -0.4400 | 10.0426 | 1.53E-03 | 1.64E-02 |
| LOC112533261 | - - - - - | uncharacterized LOC112533261 | -0.92 | -0.5360 | 7.9129 | 4.91E-03 | 3.78E-02 |
| LOC421892 | - - - - - | transcription elongation factor B polypeptide 3-like | -0.92 | 0.3373 | 10.2842 | 1.34E-03 | 1.48E-02 |
| LOC112530519 | - - - - - | translation initiation factor IF-2 | -0.92 | -0.6255 | 8.3797 | 3.79E-03 | 3.13E-02 |
| TLR2A | ENSGALG00000034722 | toll-like receptor 2 family member A | -0.92 | 1.7878 | 12.0189 | 5.27E-04 | 7.54E-03 |
| HIST1H4I | ENSGALG00000048097 | histone cluster 1, H4i | -0.92 | 2.6999 | 21.4858 | 3.56E-06 | 1.38E-04 |
| AQP3 | ENSGALG00000002452 | aquaporin 3 (Gill blood group) | -0.91 | 1.9235 | 9.5944 | 1.95E-03 | 1.98E-02 |
| TUBA4B | - - - - - | ----- | -0.91 | 1.1991 | 18.3617 | 1.83E-05 | 5.23E-04 |
| CYSLTR1 | ENSGALG00000004128 | cysteinyl leukotriene receptor 1 | -0.91 | 3.1795 | 47.6275 | 5.15E-12 | 1.13E-09 |
| AWAT1 | ENSGALG00000004367 | acyl-CoA wax alcohol acyltransferase 1 | -0.91 | 1.4629 | 21.6427 | 3.28E-06 | 1.29E-04 |
| NPR1 | ENSGALG00000015753 | natriuretic peptide receptor A/guanylate cyclase A (atrionatriuretic peptide receptor A) | -0.91 | 0.3596 | 11.3495 | 7.55E-04 | 9.79E-03 |
| TMEM68L | - - - - - | ----- | -0.91 | 0.6544 | 12.9185 | 3.25E-04 | 5.22E-03 |
| LOC112532643 | - - - - - | uncharacterized LOC112532643 | -0.90 | -0.6656 | 7.3664 | 6.65E-03 | 4.67E-02 |
| DYNLRB2 | ENSGALG00000005402 | dynein light chain roadblock-type 2 | -0.89 | 1.7377 | 10.0742 | 1.50E-03 | 1.62E-02 |
| F7 | ENSGALG00000016833 | coagulation factor VII | -0.89 | -0.4675 | 8.9886 | 2.72E-03 | 2.46E-02 |
| LOC112531865 | - - - - - | uncharacterized LOC112531865 | -0.89 | -0.4476 | 7.9000 | 4.94E-03 | 3.80E-02 |
| RASGRF2 | ENSGALG00000015598 | Ras protein specific guanine nucleotide releasing factor 2 | -0.89 | 2.3171 | 17.5854 | 2.75E-05 | 7.30E-04 |
| LOC112533090 | - - - - - | small Cajal body-specific RNA 6 | -0.89 | -0.1418 | 7.6745 | 5.60E-03 | 4.13E-02 |
| SPTSSBL | ENSGALG00000048373 | serine palmitoyltransferase small subunit B-like | -0.89 | 1.6928 | 20.2231 | 6.89E-06 | 2.41E-04 |
| LOC112530210 | - - - - - | uncharacterized LOC112530210 | -0.88 | 0.5528 | 9.0453 | 2.63E-03 | 2.41E-02 |

| | | | | | | | |
|---|---|---|---|---|---|---|---|
| LOC112531846 | - - - - - | uncharacterized LOC112531846 | -0.88 | 1.7800 | 11.3695 | 7.47E-04 | 9.70E-03 |
| SP9 | ENSGALG00000043079 | Sp9 transcription factor | -0.88 | -0.5359 | 7.3397 | 6.74E-03 | 4.71E-02 |
| LOC107049660 | - - - - - | glycogen phosphorylase, muscle form-like | -0.88 | 1.1245 | 14.4569 | 1.43E-04 | 2.75E-03 |
| LOC107054255 | - - - - - | uncharacterized LOC107054255 | -0.88 | 0.1284 | 10.2935 | 1.34E-03 | 1.48E-02 |
| KCNK18 | ENSGALG00000009265 | potassium two pore domain channel subfamily K member 18 | -0.88 | 0.7770 | 10.3650 | 1.28E-03 | 1.44E-02 |
| THEMIS2 | ENSGALG00000000731 | thymocyte selection associated family member 2 | -0.88 | 1.1781 | 10.2958 | 1.33E-03 | 1.48E-02 |
| TNFSF13B | ENSGALG00000016852 | tumor necrosis factor superfamily member 13b | -0.88 | 0.2918 | 8.2654 | 4.04E-03 | 3.27E-02 |
| ITGBL1 | ENSGALG00000016867 | integrin subunit beta like 1 | -0.87 | 1.1139 | 15.8228 | 6.96E-05 | 1.57E-03 |
| RNASEK | ENSGALG00000050001 | ribonuclease K | -0.87 | 7.1111 | 139.7589 | 3.01E-32 | 8.24E-29 |
| CLDN9 | - - - - - | ----- | -0.87 | -0.0349 | 12.4765 | 4.12E-04 | 6.26E-03 |
| ACTR10L | - - - - - | actin related protein 10-like | -0.87 | 3.6453 | 24.5135 | 7.38E-07 | 3.73E-05 |
| MYH1G | ENSGALG00000028612 | myosin, heavy chain 1G, skeletal muscle | -0.87 | 0.8824 | 7.5569 | 5.98E-03 | 4.33E-02 |
| SCML4 | - - - - - | Scm polycomb group protein like 4 | -0.87 | 3.9183 | 17.5725 | 2.77E-05 | 7.33E-04 |
| BEST4 | ENSGALG00000043605 | bestrophin 4 | -0.87 | 1.3020 | 9.5522 | 2.00E-03 | 2.01E-02 |
| PCSK9 | ENSGALG00000047448 | proprotein convertase subtilisin/kexin type 9 | -0.87 | 0.1239 | 8.0626 | 4.52E-03 | 3.55E-02 |
| LOC107050768 | - - - - - | histone-lysine N-methyltransferase SETD1A-like | -0.86 | 5.9723 | 17.0174 | 3.70E-05 | 9.35E-04 |
| TRPA1 | ENSGALG00000034751 | transient receptor potential cation channel subfamily A member 1 | -0.86 | 1.1498 | 9.0953 | 2.56E-03 | 2.37E-02 |
| SLC5A7 | ENSGALG00000016804 | solute carrier family 5 member 7 | -0.86 | 3.8045 | 8.3261 | 3.91E-03 | 3.20E-02 |
| ST8SIA3Z | ENSGALG00000039151 | ST8 alpha-N-acetyl-neuraminide alpha-2,8-sialyltransferase 3-Z | -0.86 | 0.9577 | 9.3143 | 2.27E-03 | 2.19E-02 |
| LOC107052234 | - - - - - | uncharacterized LOC107052234 | -0.86 | 1.7663 | 18.9072 | 1.37E-05 | 4.18E-04 |
| PAK6 | - - - - - | p21 (RAC1) activated kinase 6 | -0.86 | 1.2269 | 14.0701 | 1.76E-04 | 3.24E-03 |
| TNR | ENSGALG00000004526 | tenascin R | -0.86 | 0.7556 | 8.2194 | 4.14E-03 | 3.33E-02 |
| LOC112531887 | - - - - - | uncharacterized LOC112531887 | -0.86 | 1.8382 | 9.3653 | 2.21E-03 | 2.16E-02 |
| ASIP | ENSGALG00000039101 | agouti signaling protein | -0.85 | 1.3824 | 7.3685 | 6.64E-03 | 4.66E-02 |
| CRYBA2 | ENSGALG00000011354 | crystallin beta A2 | -0.85 | 0.5738 | 9.4605 | 2.10E-03 | 2.08E-02 |
| TRPM8 | ENSGALG00000004116 | transient receptor potential cation channel subfamily M member 8 | -0.85 | 0.1614 | 10.9820 | 9.20E-04 | 1.13E-02 |
| LOC107054371 | - - - - - | uncharacterized LOC107054371 | -0.85 | -0.3310 | 9.5429 | 2.01E-03 | 2.02E-02 |
| TRIM39.2 | ENSGALG00000037170 | tripartite motif containing 39.2 | -0.85 | -0.2506 | 8.9352 | 2.80E-03 | 2.51E-02 |
| LOC112531323 | - - - - - | nephrin-like | -0.85 | 0.3236 | 8.7506 | 3.10E-03 | 2.70E-02 |
| TRIM65 | - - - - - | tripartite motif containing 65 | -0.84 | 1.9285 | 15.0642 | 1.04E-04 | 2.13E-03 |
| LOC101749220 | - - - - - | uncharacterized LOC101749220 | -0.84 | 1.3203 | 12.9234 | 3.25E-04 | 5.21E-03 |
| LEFTY2 | ENSGALG00010017549 | left-right determination factor 1 | -0.84 | 1.6610 | 13.0996 | 2.95E-04 | 4.87E-03 |
| ITGB4 | ENSGALG00000002389 | integrin subunit beta 4 | -0.84 | 2.5900 | 29.7481 | 4.92E-08 | 3.66E-06 |
| LOC112532409 | - - - - - | uncharacterized LOC112532409 | -0.84 | 1.7056 | 13.9499 | 1.88E-04 | 3.40E-03 |
| IL16 | ENSGALG00000006388 | interleukin 16 | -0.84 | 1.8450 | 13.1206 | 2.92E-04 | 4.83E-03 |
| LOC107049615 | - - - - - | withdrawn | -0.84 | 3.4636 | 19.7546 | 8.80E-06 | 2.96E-04 |
| NTN4 | ENSGALG00000011406 | netrin 4 | -0.83 | 2.2899 | 18.5214 | 1.68E-05 | 4.89E-04 |
| LOC112532757 | - - - - - | uncharacterized LOC112532757 | -0.83 | 0.1231 | 7.9419 | 4.83E-03 | 3.74E-02 |
| LOC112531521 | - - - - - | uncharacterized LOC112531521 | -0.83 | -0.2687 | 11.5134 | 6.91E-04 | 9.19E-03 |
| ITIH6 | - - - - - | inter-alpha-trypsin inhibitor heavy chain family member 6 | -0.83 | 1.5468 | 12.6418 | 3.77E-04 | 5.86E-03 |
| HOXA9 | ENSGALG00000028983 | homeobox A9 | -0.83 | 4.4537 | 10.3578 | 1.29E-03 | 1.44E-02 |
| TNNI3K | ENSGALG00000011353 | TNNI3 interacting kinase | -0.83 | 0.0924 | 7.4181 | 6.46E-03 | 4.60E-02 |
| LOC112532925 | - - - - - | uncharacterized LOC112532925 | -0.83 | -0.4197 | 7.2729 | 7.00E-03 | 4.83E-02 |
| CXCR1 | ENSGALG00000036627 | C-X-C motif chemokine receptor 1 | -0.82 | 0.0681 | 10.5414 | 1.17E-03 | 1.34E-02 |

| | | | | | | | |
|---|---|---|---|---|---|---|---|
| F13A1 | ENSGALG00000012802 | coagulation factor XIII A chain | -0.82 | 0.5466 | 8.9228 | 2.82E-03 | 2.53E-02 |
| EDA2R | ENSGALG00000031094 | ectodysplasin A2 receptor | -0.82 | 2.9892 | 9.4860 | 2.07E-03 | 2.07E-02 |
| NEK10 | ENSGALG00000011322 | NIMA related kinase 10 | -0.82 | 0.3430 | 10.9420 | 9.40E-04 | 1.15E-02 |
| SLC5A1 | ENSGALG00000006728 | solute carrier family 5 member 1 | -0.82 | 1.6325 | 20.5742 | 5.74E-06 | 2.08E-04 |
| LOC107053028 | - - - - - | uncharacterized LOC107053028 | -0.82 | 1.1374 | 9.4789 | 2.08E-03 | 2.07E-02 |
| ABLIM3 | ENSGALG00000002790 | actin binding LIM protein family member 3 | -0.82 | 2.6373 | 33.3213 | 7.81E-09 | 6.95E-07 |
| GAL | ENSGALG00000007047 | galanin and GMAP prepropeptide | -0.82 | 0.9198 | 9.0468 | 2.63E-03 | 2.41E-02 |
| LOC112532982 | - - - - - | uncharacterized LOC112532982 | -0.81 | 3.0178 | 11.5669 | 6.71E-04 | 9.03E-03 |
| LOC107053889 | - - - - - | uncharacterized LOC107053889 | -0.81 | 1.1145 | 10.9805 | 9.21E-04 | 1.13E-02 |
| LOC107052229 | - - - - - | uncharacterized LOC107052229 | -0.81 | 2.5227 | 19.5318 | 9.89E-06 | 3.22E-04 |
| LOC101751319 | - - - - - | uncharacterized LOC101751319 | -0.81 | 1.2655 | 8.7658 | 3.07E-03 | 2.69E-02 |
| LOC107053148 | - - - - - | uncharacterized LOC107053148 | -0.81 | -0.2788 | 9.2333 | 2.38E-03 | 2.26E-02 |
| KCNG4 | ENSGALG00000005502 | potassium voltage-gated channel modifier subfamily G member 4 | -0.81 | 0.0922 | 7.3257 | 6.80E-03 | 4.73E-02 |
| LOC112530028 | - - - - - | uncharacterized LOC112530028 | -0.81 | 0.1278 | 7.4684 | 6.28E-03 | 4.50E-02 |
| GRM3 | ENSGALG00000006576 | glutamate metabotropic receptor 3 | -0.81 | 4.5135 | 25.4180 | 4.62E-07 | 2.51E-05 |
| SLC26A6 | ENSGALG00000026089 | solute carrier family 26 member 6 | -0.81 | 0.9376 | 7.8413 | 5.11E-03 | 3.87E-02 |
| LOC112532504 | - - - - - | uncharacterized LOC112532504 | -0.80 | 2.8152 | 38.2433 | 6.25E-10 | 7.29E-08 |
| LOC112533555 | - - - - - | olfactory receptor 14C36-like | -0.80 | 2.1750 | 11.7142 | 6.20E-04 | 8.50E-03 |
| LOC112532781 | - - - - - | uncharacterized LOC112532781 | -0.80 | 0.6585 | 12.5769 | 3.91E-04 | 6.01E-03 |
| CD109 | ENSGALG00000015910 | CD109 molecule | -0.80 | 3.0583 | 15.9946 | 6.35E-05 | 1.46E-03 |
| LOC100858131 | - - - - - | trichohyalin-like | -0.80 | 2.0516 | 23.2280 | 1.44E-06 | 6.42E-05 |
| LOC101749506 | - - - - - | uncharacterized LOC101749506 | -0.80 | 1.2322 | 17.6977 | 2.59E-05 | 6.97E-04 |
| VNN1 | ENSGALG00000013993 | vanin 1 | -0.80 | -0.0144 | 10.7674 | 1.03E-03 | 1.23E-02 |
| TCEA3 | ENSGALG00000050173 | transcription elongation factor A3 | -0.80 | 1.8113 | 18.1158 | 2.08E-05 | 5.79E-04 |
| LOC422150 | - - - - - | c-X-C chemokine receptor type 3-like | -0.79 | -0.2648 | 9.0512 | 2.63E-03 | 2.41E-02 |
| LOC112532109 | - - - - - | uncharacterized LOC112532109 | -0.79 | 0.8221 | 9.3371 | 2.25E-03 | 2.17E-02 |
| LOC107053787 | - - - - - | uncharacterized LOC107053787 | -0.79 | 0.1913 | 8.2128 | 4.16E-03 | 3.34E-02 |
| CCR7 | ENSGALG00000035733 | C-C motif chemokine receptor 7 | -0.79 | 1.4940 | 9.2958 | 2.30E-03 | 2.21E-02 |
| SNX20 | ENSGALG00000038374 | sorting nexin 20 | -0.79 | 2.4642 | 23.4923 | 1.25E-06 | 5.75E-05 |
| LOC107053086 | - - - - - | uncharacterized LOC107053086 | -0.79 | 1.8956 | 16.5378 | 4.77E-05 | 1.15E-03 |
| LOC107051875 | - - - - - | uncharacterized LOC107051875 | -0.79 | 3.3660 | 17.4025 | 3.02E-05 | 7.89E-04 |
| CACNG3 | ENSGALG00000038087 | calcium voltage-gated channel auxiliary subunit gamma 3 | -0.79 | 3.2388 | 25.1016 | 5.44E-07 | 2.89E-05 |
| LRRC55 | - - - - - | leucine rich repeat containing 55 | -0.79 | 0.4716 | 9.2168 | 2.40E-03 | 2.27E-02 |
| LOC112533354 | - - - - - | maestro heat-like repeat-containing protein family member 7 | -0.79 | 3.5521 | 36.1750 | 1.80E-09 | 1.89E-07 |
| SEMA5A | ENSGALG00000028685 | semaphorin 5A | -0.78 | 5.8966 | 17.7692 | 2.49E-05 | 6.75E-04 |
| HIST1H46L2 | ENSGALG00000053680 | histone cluster 1, H4-IV, germinal H4-like 2 | -0.78 | 0.1069 | 9.6075 | 1.94E-03 | 1.97E-02 |
| C3 | ENSGALG00000030038 | complement component 3 | -0.78 | 1.9876 | 11.0908 | 8.68E-04 | 1.08E-02 |
| MYOCD | ENSGALG00000001021 | myocardin | -0.78 | 2.2193 | 7.7694 | 5.31E-03 | 3.99E-02 |
| LOC107052021 | - - - - - | uncharacterized LOC107052021 | -0.78 | 1.1790 | 7.3718 | 6.63E-03 | 4.66E-02 |
| AIPL1 | - - - - - | aryl hydrocarbon receptor interacting protein like 1 | -0.78 | 0.7967 | 11.4503 | 7.15E-04 | 9.42E-03 |
| UPK1B | ENSGALG00000015079 | uroplakin 1B | -0.78 | 3.3279 | 11.8997 | 5.61E-04 | 7.89E-03 |
| AGTR2 | ENSGALG00000005936 | angiotensin II receptor type 2 | -0.78 | 0.7899 | 8.0834 | 4.47E-03 | 3.52E-02 |
| LOC107048987 | - - - - - | putative methyltransferase DDB_G0268948 | -0.78 | 5.1298 | 32.1065 | 1.46E-08 | 1.23E-06 |
| LOC107053414 | - - - - - | mucin 5 like | -0.77 | 1.2801 | 8.1672 | 4.27E-03 | 3.40E-02 |

| | | | | | | | |
|---|---|---|---|---|---|---|---|
| LOC107053617 | - - - - - | uncharacterized LOC107053617 | -0.77 | -0.1445 | 7.6584 | 5.65E-03 | 4.16E-02 |
| TRMU | ENSGALG00000031922 | tRNA 5-methylaminomethyl-2-thiouridylate methyltransferase | -0.77 | 4.8616 | 79.3074 | 5.32E-19 | 3.80E-16 |
| ARL2 | - - - - - | ADP-ribosylation factor-like 2 | -0.77 | -0.1459 | 11.0303 | 8.96E-04 | 1.11E-02 |
| COL17A1 | ENSGALG00000029617 | collagen type XVII alpha 1 chain | -0.77 | 3.2570 | 12.9637 | 3.18E-04 | 5.14E-03 |
| PDGFD | ENSGALG00000017179 | platelet derived growth factor D | -0.77 | 5.0233 | 53.5912 | 2.47E-13 | 7.81E-11 |
| C16orf96 | - - - - - | chromosome 16 open reading frame 96 | -0.77 | 1.6082 | 12.4714 | 4.13E-04 | 6.27E-03 |
| LOC112532562 | - - - - - | translation initiation factor IF-2-like | -0.76 | 1.3958 | 12.9662 | 3.17E-04 | 5.14E-03 |
| MYLK2 | ENSGALG00000006273 | myosin light chain kinase 2 | -0.76 | 0.0385 | 11.5677 | 6.71E-04 | 9.03E-03 |
| LOC107055181 | - - - - - | uncharacterized LOC107055181 | -0.76 | -0.1918 | 9.1107 | 2.54E-03 | 2.35E-02 |
| FBP1 | ENSGALG00000012613 | fructose-bisphosphatase 1 | -0.76 | 1.7639 | 15.0109 | 1.07E-04 | 2.16E-03 |
| LOC112531456 | - - - - - | uncharacterized LOC112531456 | -0.76 | 3.7788 | 26.6167 | 2.48E-07 | 1.46E-05 |
| BAMBI | ENSGALG00000040993 | BMP and activin membrane bound inhibitor | -0.76 | 5.4853 | 9.5464 | 2.00E-03 | 2.02E-02 |
| LOC101747954 | - - - - - | uncharacterized LOC101747954 | -0.76 | 1.3608 | 10.2534 | 1.36E-03 | 1.50E-02 |
| WDYHV1 | ENSGALG00000030059 | WDYHV motif containing 1 | -0.76 | 5.5439 | 41.4030 | 1.24E-10 | 1.84E-08 |
| LOC112530195 | - - - - - | uncharacterized LOC112530195 | -0.76 | 0.4629 | 9.0948 | 2.56E-03 | 2.37E-02 |
| DSCAM | ENSGALG00000016138 | DS cell adhesion molecule | -0.75 | 1.5117 | 8.4660 | 3.62E-03 | 3.03E-02 |
| JAKMIP1 | ENSGALG00000015528 | janus kinase and microtubule interacting protein 1 | -0.75 | -0.0988 | 7.2389 | 7.13E-03 | 4.89E-02 |
| LOC112532500 | - - - - - | uncharacterized LOC112532500 | -0.75 | 0.4271 | 7.9277 | 4.87E-03 | 3.75E-02 |
| LOC768418 | - - - - - | zinc finger FYVE domain-containing protein 16-like | -0.75 | 1.4173 | 10.1718 | 1.43E-03 | 1.56E-02 |
| NPAS2 | ENSGALG00000016774 | neuronal PAS domain protein 2 | -0.75 | 1.9196 | 16.6953 | 4.39E-05 | 1.07E-03 |
| ABI3 | ENSGALG00000044071 | ABI family member 3 | -0.75 | 1.9854 | 21.4543 | 3.62E-06 | 1.40E-04 |
| GALNTL6 | ENSGALG00000030588 | polypeptide N-acetylgalactosaminyltransferase-like 6 | -0.75 | 1.0325 | 13.3254 | 2.62E-04 | 4.46E-03 |
| STOX1 | - - - - - | storkhead box 1 | -0.75 | 4.4966 | 23.7998 | 1.07E-06 | 5.01E-05 |
| LOC112532293 | - - - - - | translation initiation factor IF-2-like | -0.74 | 1.4276 | 15.8989 | 6.68E-05 | 1.51E-03 |
| SEZ6 | ENSGALG00000026431 | seizure related 6 homolog | -0.74 | 3.3221 | 8.5883 | 3.38E-03 | 2.88E-02 |
| MLPH | ENSGALG00000003904 | melanophilin | -0.74 | 0.9784 | 14.6313 | 1.31E-04 | 2.55E-03 |
| SLC22A7 | ENSGALG00000001661 | solute carrier family 22 member 7 | -0.74 | 0.0138 | 7.3613 | 6.66E-03 | 4.67E-02 |
| HHLA2 | ENSGALG00000022871 | HERV-H LTR-associating 2 | -0.74 | 2.4883 | 17.6465 | 2.66E-05 | 7.15E-04 |
| LOC101750376 | - - - - - | kallikrein-15-like | -0.74 | 1.0311 | 14.9426 | 1.11E-04 | 2.22E-03 |
| CSMD1 | ENSGALG00000035230 | CUB and Sushi multiple domains 1 | -0.74 | 2.2912 | 8.7970 | 3.02E-03 | 2.65E-02 |
| DLGAP2 | ENSGALG00000032937 | DLG associated protein 2 | -0.74 | 1.4084 | 12.5772 | 3.90E-04 | 6.01E-03 |
| CALCR | ENSGALG00000009509 | calcitonin receptor | -0.73 | 1.3389 | 14.5301 | 1.38E-04 | 2.65E-03 |
| C5H11orf16 | - - - - - | ----- | -0.73 | 0.8950 | 8.5988 | 3.36E-03 | 2.87E-02 |
| LOC107056111 | - - - - - | uncharacterized LOC107056111 | -0.73 | 0.6635 | 9.9350 | 1.62E-03 | 1.71E-02 |
| LOC112530469 | - - - - - | cytochrome P450 2G1-like | -0.73 | 2.5844 | 8.9001 | 2.85E-03 | 2.55E-02 |
| A2ML4 | ENSGALG00000000293 | alpha-2-macroglobulin-like 4 | -0.73 | 4.3589 | 13.6936 | 2.15E-04 | 3.80E-03 |
| NLRC3 | ENSGALG00000033278 | NLR family CARD domain containing 3 | -0.73 | 1.0725 | 12.9447 | 3.21E-04 | 5.19E-03 |
| ATP2C2 | - - - - - | ATPase secretory pathway Ca2+ transporting 2 | -0.73 | 2.7038 | 11.8243 | 5.85E-04 | 8.12E-03 |
| LOC112531849 | - - - - - | paired mesoderm homeobox protein 2A-like | -0.73 | 1.9789 | 7.9011 | 4.94E-03 | 3.80E-02 |
| LRRC34 | ENSGALG00000009402 | leucine rich repeat containing 34 | -0.73 | 1.9383 | 14.0928 | 1.74E-04 | 3.21E-03 |
| RGS4 | ENSGALG00000002564 | regulator of G-protein signaling 4 | -0.73 | 2.4443 | 8.2858 | 4.00E-03 | 3.24E-02 |
| SYT15 | ENSGALG00000002046 | synaptotagmin 15 | -0.73 | 1.8851 | 11.9119 | 5.58E-04 | 7.88E-03 |
| MSLN | ENSGALG00000005349 | mesothelin | -0.73 | 1.8040 | 11.7130 | 6.21E-04 | 8.50E-03 |
| BTN3A3L1 | - - - - - | butyrophilin subfamily 3 member A3-like 1 | -0.73 | 1.7781 | 8.5328 | 3.49E-03 | 2.94E-02 |

| | | | | | | | |
|---|---|---|---|---|---|---|---|
| SNAP91 | ENSGALG00000015846 | synaptosome associated protein 91 | -0.72 | 3.6163 | 19.0341 | 1.28E-05 | 3.95E-04 |
| DUSP18 | - - - - - | ----- | -0.72 | 1.5410 | 11.1533 | 8.39E-04 | 1.06E-02 |
| STK32B | ENSGALG00000029510 | serine/threonine kinase 32B | -0.72 | 1.3437 | 8.3146 | 3.93E-03 | 3.21E-02 |
| KCNJ15 | ENSGALG00000016055 | potassium inwardly rectifying channel subfamily J member 15 | -0.72 | 1.6621 | 18.6585 | 1.56E-05 | 4.65E-04 |
| ALKBH8 | ENSGALG00000017170 | alkB homolog 8, tRNA methyltransferase | -0.72 | 4.2026 | 19.9052 | 8.14E-06 | 2.79E-04 |
| LOC107057099 | - - - - - | MHCY region zinc finger protein 2 | -0.72 | 2.4613 | 16.6385 | 4.52E-05 | 1.10E-03 |
| STMN2 | ENSGALG00000039690 | stathmin 2 | -0.72 | 5.1126 | 9.5454 | 2.00E-03 | 2.02E-02 |
| SNCA | ENSGALG00000010379 | synuclein alpha | -0.71 | 0.7653 | 12.9829 | 3.14E-04 | 5.11E-03 |
| LOC112532090 | - - - - - | uncharacterized LOC112532090 | -0.71 | 0.7088 | 8.0034 | 4.67E-03 | 3.64E-02 |
| LOC112532494 | - - - - - | uncharacterized LOC112532494 | -0.71 | 0.3321 | 7.9243 | 4.88E-03 | 3.76E-02 |
| ACACB | - - - - - | acetyl-CoA carboxylase beta | -0.71 | 1.0666 | 9.4787 | 2.08E-03 | 2.07E-02 |
| DGKK | ENSGALG00000003841 | diacylglycerol kinase eta | -0.71 | 1.6248 | 15.9042 | 6.66E-05 | 1.51E-03 |
| CAPG | ENSGALG00000036175 | capping actin protein, gelsolin like | -0.70 | 2.9246 | 11.6439 | 6.44E-04 | 8.74E-03 |
| C3H1orf95 | ENSGALG00000009219 | chromosome 3 C1orf95 homolog | -0.70 | 1.7966 | 7.5088 | 6.14E-03 | 4.42E-02 |
| LOC107051601 | - - - - - | uncharacterized LOC107051601 | -0.70 | 0.3026 | 8.9693 | 2.75E-03 | 2.48E-02 |
| LOC101751026 | - - - - - | uncharacterized LOC101751026 | -0.70 | 1.9480 | 17.1901 | 3.38E-05 | 8.63E-04 |
| CLRN1 | ENSGALG00000010384 | clarin 1 | -0.69 | 0.1588 | 7.6916 | 5.55E-03 | 4.10E-02 |
| RAMP1 | ENSGALG00000003821 | receptor activity modifying protein 1 | -0.69 | 2.4873 | 13.3670 | 2.56E-04 | 4.39E-03 |
| BATF | ENSGALG00000010323 | basic leucine zipper ATF-like transcription factor | -0.69 | 0.3015 | 9.0188 | 2.67E-03 | 2.43E-02 |
| RBFOX1 | ENSGALG00000034325 | RNA binding protein, fox-1 homolog 1 | -0.69 | 4.6657 | 30.7869 | 2.88E-08 | 2.26E-06 |
| LOC771422 | - - - - - | transmembrane protein 151B-like | -0.68 | 1.6909 | 8.7163 | 3.15E-03 | 2.74E-02 |
| KLF2 | ENSGALG00000003939 | Kruppel like factor 2 | -0.68 | 2.2220 | 9.2247 | 2.39E-03 | 2.26E-02 |
| ADRA1D | ENSGALG00000038787 | adrenoceptor alpha 1D | -0.68 | 0.2885 | 7.7091 | 5.49E-03 | 4.07E-02 |
| TUBA4A | - - - - - | ----- | -0.68 | 2.5652 | 17.5444 | 2.81E-05 | 7.40E-04 |
| GCNT4 | ENSGALG00000014944 | glucosaminyl (N-acetyl) transferase 4, core 2 | -0.68 | 0.9834 | 7.3806 | 6.59E-03 | 4.65E-02 |
| LOC101748220 | - - - - - | uncharacterized LOC101748220 | -0.68 | 0.8233 | 7.7172 | 5.47E-03 | 4.06E-02 |
| LOC112531072 | - - - - - | zinc finger protein 135-like | -0.68 | 1.9019 | 7.2849 | 6.95E-03 | 4.80E-02 |
| LOC112532340 | - - - - - | uncharacterized LOC112532340 | -0.68 | 0.7885 | 9.4087 | 2.16E-03 | 2.12E-02 |
| LOC112532555 | - - - - - | uncharacterized LOC112532555 | -0.67 | 1.2094 | 12.6497 | 3.76E-04 | 5.84E-03 |
| MEI1 | - - - - - | meiotic double-stranded break formation protein 1 | -0.67 | 2.3256 | 15.0111 | 1.07E-04 | 2.16E-03 |
| LOC107053805 | - - - - - | uncharacterized LOC107053805 | -0.67 | 2.8661 | 23.9827 | 9.72E-07 | 4.72E-05 |
| SFRP4 | ENSGALG00000031997 | secreted frizzled related protein 4 | -0.67 | 0.9313 | 9.1743 | 2.45E-03 | 2.30E-02 |
| LOC101748673 | - - - - - | uncharacterized LOC101748673 | -0.67 | 2.5692 | 18.3257 | 1.86E-05 | 5.32E-04 |
| LOC770617 | - - - - - | transmembrane protein 100-like | -0.67 | 4.6940 | 40.5001 | 1.97E-10 | 2.77E-08 |
| FBLN5 | ENSGALG00000010747 | fibulin 5 | -0.67 | 2.6138 | 13.2465 | 2.73E-04 | 4.59E-03 |
| CA3A | ENSGALG00000033700 | carbonic anhydrase 3A | -0.66 | 1.2402 | 9.8495 | 1.70E-03 | 1.78E-02 |
| LOC107053811 | - - - - - | uncharacterized LOC107053811 | -0.66 | 2.0932 | 8.9050 | 2.84E-03 | 2.55E-02 |
| LOC112530132 | - - - - - | uncharacterized LOC112530132 | -0.66 | 0.4762 | 9.1434 | 2.50E-03 | 2.32E-02 |
| CD3E | ENSGALG00000007416 | CD3e molecule | -0.66 | 2.5889 | 20.4130 | 6.24E-06 | 2.22E-04 |
| MREG | ENSGALG00000003758 | melanoregulin | -0.66 | 2.1988 | 11.6271 | 6.50E-04 | 8.80E-03 |
| CYP26C1 | ENSGALG00000043213 | cytochrome P450 family 26 subfamily C member 1 | -0.66 | 2.5367 | 11.3762 | 7.44E-04 | 9.68E-03 |
| LOC100859492 | - - - - - | uncharacterized LOC100859492 | -0.66 | 0.4901 | 7.3751 | 6.61E-03 | 4.65E-02 |
| MTFP1 | ENSGALG00000038952 | mitochondrial fission process 1 | -0.65 | 3.8482 | 26.5864 | 2.52E-07 | 1.48E-05 |
| RXRA | ENSGALG00000042055 | retinoid X receptor alpha | -0.65 | 4.6947 | 23.1304 | 1.51E-06 | 6.73E-05 |

| | | | | | | | |
|---|---|---|---|---|---|---|---|
| LOC101747725 | - - - - - | uncharacterized LOC101747725 | -0.65 | 0.8168 | 12.1261 | 4.97E-04 | 7.23E-03 |
| LOC107052937 | - - - - - | uncharacterized LOC107052937 | -0.65 | -0.0025 | 8.5973 | 3.37E-03 | 2.87E-02 |
| LCP1 | ENSGALG00000016986 | lymphocyte cytosolic protein 1 | -0.65 | 4.1589 | 18.8988 | 1.38E-05 | 4.19E-04 |
| CLCN4 | ENSGALG00000016607 | chloride voltage-gated channel 4 | -0.65 | 4.2664 | 21.7876 | 3.05E-06 | 1.21E-04 |
| LOC107053523 | - - - - - | uncharacterized LOC107053523 | -0.65 | 1.8325 | 13.9731 | 1.85E-04 | 3.36E-03 |
| SOUL | ENSGALG00000012464 | SOUL protein | -0.65 | 0.6092 | 7.8810 | 5.00E-03 | 3.83E-02 |
| FAM46C | ENSGALG00000014453 | family with sequence similarity 46 member C | -0.65 | 0.9583 | 8.8533 | 2.93E-03 | 2.60E-02 |
| SAT1 | ENSGALG00000016348 | spermidine/spermine N1-acetyltransferase 1 | -0.64 | 5.7613 | 76.7383 | 1.95E-18 | 1.19E-15 |
| LOC101747455 | - - - - - | uncharacterized LOC101747455 | -0.64 | 2.4163 | 8.5819 | 3.40E-03 | 2.88E-02 |
| TYW3 | ENSGALG00000032549 | tRNA-yW synthesizing protein 3 homolog | -0.64 | 4.1539 | 23.6861 | 1.13E-06 | 5.27E-05 |
| PPFIA2 | ENSGALG00000010958 | PTPRF interacting protein alpha 2 | -0.64 | 2.9270 | 12.9502 | 3.20E-04 | 5.18E-03 |
| GJB5 | ENSGALG00000054289 | gap junction protein beta 5 | -0.64 | 1.0311 | 7.7958 | 5.24E-03 | 3.95E-02 |
| LOC107054593 | - - - - - | uncharacterized LOC107054593 | -0.63 | 1.2465 | 14.5217 | 1.39E-04 | 2.66E-03 |
| FAM43B | ENSGALG00000026367 | family with sequence similarity 43 member B | -0.63 | 2.3047 | 11.7913 | 5.95E-04 | 8.23E-03 |
| GHDC | ENSGALG00000003333 | GH3 domain containing | -0.63 | 3.5499 | 19.0313 | 1.29E-05 | 3.95E-04 |
| LOC107053391 | - - - - - | uncharacterized LOC107053391 | -0.63 | 2.0366 | 15.7536 | 7.22E-05 | 1.62E-03 |
| LOC112533362 | - - - - - | uncharacterized LOC112533362 | -0.63 | 1.5042 | 8.8284 | 2.97E-03 | 2.62E-02 |
| LOC112530592 | - - - - - | uncharacterized LOC112530592 | -0.63 | 1.3093 | 11.2445 | 7.99E-04 | 1.02E-02 |
| ABCA12 | ENSGALG00000003553 | ATP binding cassette subfamily A member 12 | -0.63 | 2.3211 | 11.4941 | 6.98E-04 | 9.24E-03 |
| LOC107051864 | - - - - - | uncharacterized LOC107051864 | -0.63 | 1.2503 | 8.8953 | 2.86E-03 | 2.56E-02 |
| LOC101749170 | - - - - - | uncharacterized LOC101749170 | -0.63 | 2.5927 | 9.5499 | 2.00E-03 | 2.02E-02 |
| SCEL | ENSGALG00000027514 | sciellin | -0.63 | 2.1434 | 14.0312 | 1.80E-04 | 3.28E-03 |
| RANBP17 | - - - - - | RAN binding protein 17 | -0.63 | 4.0731 | 10.0388 | 1.53E-03 | 1.64E-02 |
| MIR1683 | ENSGALG00000025224 | microRNA 1683 | -0.63 | 1.0486 | 9.9201 | 1.63E-03 | 1.72E-02 |
| GABRA1 | ENSGALG00000001698 | gamma-aminobutyric acid type A receptor alpha1 subunit | -0.62 | 0.7749 | 7.7692 | 5.31E-03 | 3.99E-02 |
| APCDD1 | ENSGALG00000050840 | APC down-regulated 1 | -0.62 | 7.5974 | 8.2240 | 4.13E-03 | 3.33E-02 |
| LOC101749495 | - - - - - | uncharacterized LOC101749495 | -0.62 | 3.8348 | 26.9476 | 2.09E-07 | 1.27E-05 |
| MYO3A | ENSGALG00000007647 | myosin IIIA | -0.62 | 2.3665 | 11.5516 | 6.77E-04 | 9.06E-03 |
| C1H11ORF87 | - - - - - | ----- | -0.62 | 4.4501 | 22.7893 | 1.81E-06 | 7.75E-05 |
| LOC107051956 | - - - - - | uncharacterized LOC107051956 | -0.62 | 0.2756 | 7.6153 | 5.79E-03 | 4.23E-02 |
| C10orf10 | - - - - - | withdrawn | -0.62 | 1.0263 | 10.4182 | 1.25E-03 | 1.40E-02 |
| LOC107052369 | - - - - - | uncharacterized LOC107052369 | -0.62 | 2.8834 | 13.9957 | 1.83E-04 | 3.33E-03 |
| ANKRD34B | ENSGALG00000044727 | ankyrin repeat domain 34B | -0.62 | 2.4974 | 15.4610 | 8.42E-05 | 1.81E-03 |
| AVD | ENSGALG00000025945 | avidin | -0.62 | 0.8939 | 7.3441 | 6.73E-03 | 4.71E-02 |
| ZNF408 | - - - - - | ----- | -0.62 | 3.8465 | 34.5453 | 4.16E-09 | 4.01E-07 |
| SERPINB6L | - - - - - | ----- | -0.62 | 3.7655 | 36.5862 | 1.46E-09 | 1.59E-07 |
| LOC101749148 | - - - - - | uncharacterized LOC101749148 | -0.62 | 1.3384 | 10.0186 | 1.55E-03 | 1.66E-02 |
| KIAA1257 | ENSGALG00000044299 | KIAA1257 | -0.62 | 2.3959 | 8.2706 | 4.03E-03 | 3.26E-02 |
| TMEM240 | ENSGALG00000035387 | transmembrane protein 240 | -0.61 | 2.8689 | 15.3099 | 9.12E-05 | 1.93E-03 |
| LOC107052653 | - - - - - | uncharacterized LOC107052653 | -0.61 | 2.2899 | 12.0689 | 5.13E-04 | 7.39E-03 |
| LOC100858386 | ENSGALG00000050166 | uncharacterized LOC100858386 | -0.61 | 4.6717 | 52.1753 | 5.08E-13 | 1.35E-10 |
| LOC101750048 | - - - - - | uncharacterized LOC101750048 | -0.61 | 1.0595 | 9.2951 | 2.30E-03 | 2.21E-02 |
| IQUB | ENSGALG00000008823 | IQ motif and ubiquitin domain containing | -0.61 | 2.0614 | 13.5577 | 2.31E-04 | 4.02E-03 |
| LOC107055206 | - - - - - | uncharacterized LOC107055206 | -0.61 | 2.3012 | 13.0304 | 3.06E-04 | 5.02E-03 |

| | | | | | | | |
|---|---|---|---|---|---|---|---|
| CES1L1 | - - - - - | ----- | -0.61 | 2.0585 | 9.2384 | 2.37E-03 | 2.25E-02 |
| RPE65 | ENSGALG00000011259 | RPE65, retinoid isomerohydrolase | -0.61 | 1.9700 | 7.9619 | 4.78E-03 | 3.71E-02 |
| SLC9A5 | ENSGALG00000007867 | solute carrier family 9 member A5 | -0.61 | 1.1952 | 10.9982 | 9.12E-04 | 1.12E-02 |
| PNOC | ENSGALG00000016620 | prepronociceptin | -0.61 | 1.4567 | 9.3255 | 2.26E-03 | 2.18E-02 |
| LOC101751097 | - - - - - | uncharacterized LOC101751097 | -0.61 | 4.0127 | 9.3322 | 2.25E-03 | 2.18E-02 |
| NINJ2 | ENSGALG00000012989 | ninjurin 2 | -0.61 | 3.4420 | 16.7892 | 4.18E-05 | 1.03E-03 |
| LOC101748935 | - - - - - | uncharacterized LOC101748935 | -0.61 | 1.2335 | 9.5425 | 2.01E-03 | 2.02E-02 |
| EVA1B | ENSGALG00000022440 | eva-1 homolog B | -0.61 | 4.5409 | 19.5898 | 9.60E-06 | 3.14E-04 |
| CFAP57 | ENSGALG00000009902 | cilia and flagella associated protein 57 | -0.61 | 0.8498 | 10.4506 | 1.23E-03 | 1.39E-02 |
| PDK4 | ENSGALG00000009700 | pyruvate dehydrogenase kinase 4 | -0.61 | 1.5851 | 10.2414 | 1.37E-03 | 1.51E-02 |
| RGS9 | ENSGALG00000029610 | regulator of G-protein signaling 9 | -0.60 | 4.4991 | 13.7774 | 2.06E-04 | 3.65E-03 |
| PLEKHD1 | ENSGALG00000009431 | pleckstrin homology and coiled-coil domain containing D1 | -0.60 | 3.2272 | 20.8565 | 4.95E-06 | 1.83E-04 |
| GRID1 | ENSGALG00000037366 | glutamate ionotropic receptor delta type subunit 1 | -0.60 | 1.8335 | 10.8658 | 9.80E-04 | 1.19E-02 |
| LOC112531640 | - - - - - | small nucleolar RNA SNORD89 | -0.60 | 0.8284 | 8.5160 | 3.52E-03 | 2.97E-02 |
| LXN | ENSGALG00000009651 | latexin | -0.60 | 0.8134 | 9.5271 | 2.02E-03 | 2.03E-02 |
| ENTPD8L | - - - - - | ----- | -0.59 | 2.6038 | 14.2452 | 1.60E-04 | 2.99E-03 |
| TTC9 | ENSGALG00000017652 | tetratricopeptide repeat domain 9 | -0.59 | 2.5747 | 13.9138 | 1.91E-04 | 3.45E-03 |
| FCN2 | ENSGALG00000040771 | ficolin 2 | -0.59 | 2.1294 | 14.9104 | 1.13E-04 | 2.25E-03 |
| LOC107054619 | - - - - - | uncharacterized LOC107054619 | -0.59 | 0.6093 | 9.6675 | 1.88E-03 | 1.92E-02 |
| FAM129A | ENSGALG00000004812 | family with sequence similarity 129 member A | -0.59 | 4.0733 | 25.7122 | 3.96E-07 | 2.21E-05 |
| RAB39A | ENSGALG00000017166 | RAB39A, member RAS oncogene family | -0.59 | 2.0707 | 16.4637 | 4.96E-05 | 1.19E-03 |
| NOX3 | ENSGALG00000013659 | NADPH oxidase 3 | -0.59 | 1.8679 | 7.9441 | 4.82E-03 | 3.74E-02 |
| LOC107051605 | - - - - - | uncharacterized LOC107051605 | -0.59 | 0.4401 | 8.5813 | 3.40E-03 | 2.88E-02 |
| ALKBH1 | ENSGALG00000010485 | alkB homolog 1, histone H2A dioxygenase | -0.59 | 4.8547 | 73.7406 | 8.91E-18 | 5.05E-15 |
| IGFN1 | ENSGALG00000000295 | immunoglobulin-like and fibronectin type III domain containing 1 | -0.59 | 2.4379 | 10.4591 | 1.22E-03 | 1.38E-02 |
| LOC107054771 | - - - - - | N-alpha-acetyltransferase 38, NatC auxiliary subunit-like | -0.59 | 2.5409 | 13.3528 | 2.58E-04 | 4.41E-03 |
| CDH23 | ENSGALG00000036824 | cadherin related 23 | -0.59 | 3.1835 | 11.4216 | 7.26E-04 | 9.52E-03 |
| ZNF764L | - - - - - | ----- | -0.59 | 3.7304 | 12.4949 | 4.08E-04 | 6.22E-03 |
| LOC101748755 | - - - - - | copine-5-like | -0.58 | 0.9298 | 8.4062 | 3.74E-03 | 3.10E-02 |
| PTPRO | ENSGALG00000013085 | protein tyrosine phosphatase, receptor type O | -0.58 | 5.1930 | 16.7045 | 4.37E-05 | 1.07E-03 |
| DTX3L | ENSGALG00000012075 | deltex E3 ubiquitin ligase 3L | -0.58 | 2.8566 | 16.7682 | 4.22E-05 | 1.04E-03 |
| LOC107049475 | - - - - - | uncharacterized LOC107049475 | -0.58 | 4.9430 | 11.0678 | 8.78E-04 | 1.09E-02 |
| CLMN | ENSGALG00000030719 | calmin | -0.58 | 5.7188 | 12.5238 | 4.02E-04 | 6.16E-03 |
| TUBAL3 | ENSGALG00000008444 | tubulin alpha like 3 | -0.58 | 4.9124 | 20.3189 | 6.55E-06 | 2.31E-04 |
| SULT1B1 | ENSGALG00000023120 | sulfotransferase family cytosolic 1B member 1 | -0.58 | 3.1448 | 16.0277 | 6.24E-05 | 1.44E-03 |
| XKR8 | ENSGALG00000030305 | XK related 8 | -0.58 | 3.1437 | 17.6120 | 2.71E-05 | 7.24E-04 |
| LOC414835 | - - - - - | cHz-cadherin | -0.58 | 1.9729 | 8.7731 | 3.06E-03 | 2.68E-02 |
| OCA2 | ENSGALG00000016740 | OCA2 melanosomal transmembrane protein | -0.57 | 2.3083 | 9.3240 | 2.26E-03 | 2.18E-02 |
| TESC | ENSGALG00000008206 | tescalcin | -0.57 | 3.7337 | 17.5115 | 2.86E-05 | 7.51E-04 |
| LOC112531294 | - - - - - | withdrawn | -0.57 | 5.4016 | 13.2972 | 2.66E-04 | 4.51E-03 |
| GLRB | ENSGALG00000009395 | glycine receptor beta | -0.57 | 2.4441 | 7.8516 | 5.08E-03 | 3.86E-02 |
| TMCC3 | ENSGALG00000011320 | transmembrane and coiled-coil domain family 3 | -0.57 | 3.2348 | 7.7967 | 5.23E-03 | 3.95E-02 |
| LOC107054482 | - - - - - | protocadherin gamma-B1 pseudogene | -0.57 | 0.6980 | 7.9322 | 4.86E-03 | 3.75E-02 |
| SCIN | ENSGALG00000010770 | scinderin | -0.57 | 3.1764 | 11.4200 | 7.27E-04 | 9.52E-03 |

| | | | | | | | |
|---|---|---|---|---|---|---|---|
| LOC107055055 | - - - - - | uncharacterized LOC107055055 | -0.57 | 2.6447 | 7.8587 | 5.06E-03 | 3.85E-02 |
| MKRN3 | - - - - - | makorin ring finger protein 3 | -0.57 | 1.8523 | 8.7334 | 3.12E-03 | 2.72E-02 |
| PROKR2 | - - - - - | ----- | -0.57 | 1.7994 | 7.6012 | 5.83E-03 | 4.25E-02 |
| TPD52L1 | ENSGALG00000014843 | tumor protein D52-like 1 | -0.57 | 2.2362 | 11.5760 | 6.68E-04 | 9.00E-03 |
| LOC107052050 | - - - - - | uncharacterized LOC107052050 | -0.57 | 1.7602 | 9.5070 | 2.05E-03 | 2.05E-02 |
| LOC112531576 | - - - - - | translation initiation factor IF-2-like | -0.57 | 1.8913 | 10.3649 | 1.28E-03 | 1.44E-02 |
| ACTN2 | ENSGALG00000014463 | actinin alpha 2 | -0.57 | 4.1916 | 8.0277 | 4.61E-03 | 3.60E-02 |
| BAALC | ENSGALG00000051919 | brain and acute leukemia, cytoplasmic | -0.57 | 2.2481 | 8.0826 | 4.47E-03 | 3.52E-02 |
| FAM184B | ENSGALG00000041854 | family with sequence similarity 184 member B | -0.56 | 2.3435 | 10.1434 | 1.45E-03 | 1.57E-02 |
| MCF2 | ENSGALG00000006562 | MCF.2 cell line derived transforming sequence | -0.56 | 5.1185 | 31.2599 | 2.26E-08 | 1.83E-06 |
| LOC107050638 | - - - - - | vinexin-like | -0.56 | 1.5712 | 7.8827 | 4.99E-03 | 3.82E-02 |
| GPR17 | - - - - - | G protein-coupled receptor 17 | -0.56 | 3.3991 | 9.6987 | 1.84E-03 | 1.90E-02 |
| KCNQ5 | ENSGALG00000037249 | potassium voltage-gated channel subfamily Q member 5 | -0.56 | 3.0778 | 13.6604 | 2.19E-04 | 3.85E-03 |
| TEX14 | - - - - - | testis expressed 14, intercellular bridge forming factor | -0.56 | 3.1851 | 13.2464 | 2.73E-04 | 4.59E-03 |
| ACBD7 | ENSGALG00000019663 | acyl-CoA binding domain containing 7 | -0.56 | 3.1633 | 19.0320 | 1.29E-05 | 3.95E-04 |
| PIGP | - - - - - | phosphatidylinositol glycan anchor biosynthesis class P | -0.55 | 4.0440 | 14.9808 | 1.09E-04 | 2.18E-03 |
| SELENOM | ENSGALG00000047792 | selenoprotein M | -0.55 | 6.0708 | 46.8719 | 7.58E-12 | 1.50E-09 |
| TAPBP | ENSGALG00000008022 | TAP binding protein | -0.55 | 3.1231 | 9.8378 | 1.71E-03 | 1.78E-02 |
| SUSD5 | ENSGALG00000042289 | sushi domain containing 5 | -0.55 | 4.6856 | 15.8503 | 6.86E-05 | 1.55E-03 |
| VEGFD | ENSGALG00000016558 | vascular endothelial growth factor D | -0.55 | 6.8709 | 12.2425 | 4.67E-04 | 6.88E-03 |
| EFHC1 | ENSGALG00000016674 | EF-hand domain containing 1 | -0.55 | 1.9693 | 8.2215 | 4.14E-03 | 3.33E-02 |
| PHACTR1 | ENSGALG00000012732 | phosphatase and actin regulator 1 | -0.55 | 2.3840 | 9.0308 | 2.65E-03 | 2.42E-02 |
| CYP26A1 | ENSGALG00000033695 | cytochrome P450 family 26 subfamily A member 1 | -0.55 | 4.8935 | 9.3085 | 2.28E-03 | 2.19E-02 |
| EML5 | ENSGALG00000010639 | echinoderm microtubule associated protein like 5 | -0.55 | 2.4259 | 8.2025 | 4.18E-03 | 3.35E-02 |
| CNNM1 | ENSGALG00000007489 | cyclin and CBS domain divalent metal cation transport mediator 1 | -0.55 | 3.1578 | 9.0929 | 2.57E-03 | 2.37E-02 |
| EHHADH | ENSGALG00000053254 | enoyl-CoA hydratase and 3-hydroxyacyl CoA dehydrogenase | -0.55 | 3.9621 | 20.7643 | 5.19E-06 | 1.91E-04 |
| LOC107054545 | - - - - - | uncharacterized LOC107054545 | -0.54 | 1.6631 | 8.3006 | 3.96E-03 | 3.22E-02 |
| LOC101749129 | - - - - - | uncharacterized LOC101749129 | -0.54 | 2.7393 | 15.5227 | 8.15E-05 | 1.78E-03 |
| LOC112530163 | - - - - - | SH2B adapter protein 1-like | -0.54 | 5.4226 | 21.6289 | 3.31E-06 | 1.30E-04 |
| LOC107050463 | - - - - - | serine-aspartate repeat-containing protein F-like | -0.54 | 6.7541 | 27.8473 | 1.31E-07 | 8.64E-06 |
| ABCG1 | ENSGALG00000016164 | ATP binding cassette subfamily G member 1 | -0.54 | 3.1991 | 12.7739 | 3.51E-04 | 5.53E-03 |
| PLEKHS1 | ENSGALG00000020679 | pleckstrin homology domain containing S1 | -0.54 | 1.6665 | 9.6810 | 1.86E-03 | 1.91E-02 |
| ENPP3 | ENSGALG00000037587 | ectonucleotide pyrophosphatase/phosphodiesterase 3 | -0.54 | 2.6275 | 10.7029 | 1.07E-03 | 1.26E-02 |
| TMF1 | ENSGALG00000013408 | TATA element modulatory factor 1 | -0.54 | 6.1897 | 82.0966 | 1.30E-19 | 9.69E-17 |
| PIK3IP1 | ENSGALG00000006942 | phosphoinositide-3-kinase interacting protein 1 | -0.53 | 6.9640 | 50.4403 | 1.23E-12 | 3.02E-10 |
| FRRS1L | ENSGALG00000025792 | ferric chelate reductase 1 like | -0.53 | 5.7762 | 39.3639 | 3.52E-10 | 4.45E-08 |
| XKR6 | - - - - - | XK related 6 | -0.53 | 5.7456 | 14.6684 | 1.28E-04 | 2.51E-03 |
| SLCO4A1 | ENSGALG00000005460 | solute carrier organic anion transporter family member 4A1 | -0.53 | 3.3229 | 9.1598 | 2.47E-03 | 2.31E-02 |
| LOC769704 | - - - - - | fatty acyl-CoA hydrolase precursor, medium chain-like | -0.53 | 2.0424 | 10.4554 | 1.22E-03 | 1.39E-02 |
| CLIC6 | ENSGALG00000016020 | chloride intracellular channel 6 | -0.53 | 2.5335 | 11.5601 | 6.74E-04 | 9.05E-03 |
| KIAA1024 | ENSGALG00000006519 | KIAA1024 | -0.53 | 3.5259 | 10.0064 | 1.56E-03 | 1.66E-02 |
| ZNF226L | ENSGALG00000032405 | zinc finger protein 226-like | -0.53 | 3.5949 | 16.2047 | 5.69E-05 | 1.33E-03 |
| CAPNS2 | - - - - - | calpain small subunit 2 | -0.53 | 1.9215 | 10.8837 | 9.70E-04 | 1.18E-02 |
| LOC101748706 | - - - - - | ATP-dependent DNA helicase Q4-like | -0.53 | 6.3234 | 26.5524 | 2.56E-07 | 1.49E-05 |

| | | | | | | | |
|---|---|---|---|---|---|---|---|
| TMEM79 | ENSGALG00000039643 | transmembrane protein 79-like | -0.53 | 1.8675 | 9.3379 | 2.24E-03 | 2.17E-02 |
| MANBA | ENSGALG00000012308 | mannosidase beta | -0.53 | 4.1394 | 19.8360 | 8.44E-06 | 2.86E-04 |
| HIST1H110 | ENSGALG00000031159 | histone cluster 1, H1.10 | -0.52 | 1.6294 | 7.7551 | 5.36E-03 | 4.00E-02 |
| LOC107051764 | - - - - - | uncharacterized LOC107051764 | -0.52 | 3.5693 | 25.0307 | 5.64E-07 | 2.98E-05 |
| C1HXORF59 | ENSGALG00000016268 | chromosome 1 open reading frame, human CXORF59 | -0.52 | 3.0858 | 7.8287 | 5.14E-03 | 3.89E-02 |
| LOC112532476 | - - - - - | uncharacterized LOC112532476 | -0.52 | 1.4191 | 9.4910 | 2.06E-03 | 2.06E-02 |
| SLC9B2 | ENSGALG00000012319 | solute carrier family 9 member B2 | -0.52 | 3.8477 | 20.8257 | 5.03E-06 | 1.86E-04 |
| MCF2L2 | ENSGALG00000008744 | MCF.2 cell line derived transforming sequence-like 2 | -0.52 | 3.5266 | 10.7167 | 1.06E-03 | 1.26E-02 |
| GABRG2 | ENSGALG00000038042 | gamma-aminobutyric acid type A receptor gamma2 subunit | -0.52 | 2.3560 | 8.6895 | 3.20E-03 | 2.76E-02 |
| RPS6KA2 | ENSGALG00000011473 | ribosomal protein S6 kinase A2 | -0.52 | 2.4304 | 10.8793 | 9.72E-04 | 1.18E-02 |
| EPHA10 | ENSGALG00000001912 | EPH receptor A10 | -0.52 | 2.5897 | 9.2098 | 2.41E-03 | 2.27E-02 |
| SMAD6 | ENSGALG00000025898 | SMAD family member 6 | -0.52 | 4.5607 | 8.6904 | 3.20E-03 | 2.76E-02 |
| GPC5 | ENSGALG00000016902 | glypican 5 | -0.52 | 3.8805 | 9.9594 | 1.60E-03 | 1.70E-02 |
| LOC101749699 | - - - - - | uncharacterized LOC101749699 | -0.52 | 2.2077 | 7.3471 | 6.72E-03 | 4.70E-02 |
| LOC112530196 | - - - - - | transmembrane protein 79-like | -0.52 | 2.6789 | 9.3725 | 2.20E-03 | 2.15E-02 |
| HIST2H3D | ENSGALG00000035244 | histone cluster 2 H3 family member d | -0.52 | 2.6356 | 8.5256 | 3.50E-03 | 2.95E-02 |
| JADE2 | ENSGALG00000006424 | jade family PHD finger 2 | -0.51 | 2.8534 | 12.4744 | 4.13E-04 | 6.26E-03 |
| CCNG1 | ENSGALG00000001718 | cyclin G1 | -0.51 | 6.8396 | 13.7974 | 2.04E-04 | 3.63E-03 |
| GAS6 | ENSGALG00000016820 | growth arrest specific 6 | -0.51 | 3.4361 | 10.9215 | 9.51E-04 | 1.16E-02 |
| USH2A | ENSGALG00000009652 | usherin | -0.51 | 1.1066 | 8.2376 | 4.10E-03 | 3.31E-02 |
| ARAP2 | ENSGALG00000041373 | ArfGAP with RhoGAP domain, ankyrin repeat and PH domain 2 | -0.51 | 2.8914 | 13.6591 | 2.19E-04 | 3.85E-03 |
| FBLN1 | ENSGALG00000014233 | fibulin 1 | -0.51 | 6.8284 | 29.3448 | 6.06E-08 | 4.37E-06 |
| DGKG | ENSGALG00000005721 | diacylglycerol kinase gamma | -0.51 | 1.7581 | 7.7876 | 5.26E-03 | 3.96E-02 |
| PPP1R9A | ENSGALG00000009686 | protein phosphatase 1 regulatory subunit 9A | -0.51 | 3.5129 | 12.4679 | 4.14E-04 | 6.27E-03 |
| ADAMTS8 | ENSGALG00000001370 | ADAM metallopeptidase with thrombospondin type 1 motif, 8 | -0.51 | 4.3106 | 11.9475 | 5.47E-04 | 7.77E-03 |
| MAP10 | ENSGALG00000049034 | microtubule associated protein 10 | -0.50 | 3.1927 | 15.9142 | 6.63E-05 | 1.51E-03 |
| OGFOD3 | ENSGALG00000001618 | 2-oxoglutarate and iron dependent oxygenase domain containing 3 | -0.50 | 5.2003 | 29.4927 | 5.61E-08 | 4.12E-06 |
| RERGL | ENSGALG00000013112 | RERG like | -0.50 | 4.7617 | 12.8108 | 3.45E-04 | 5.46E-03 |
| JARID2 | ENSGALG00000012702 | jumonji and AT-rich interaction domain containing 2 | -0.50 | 7.0104 | 13.3239 | 2.62E-04 | 4.46E-03 |
| APLN | ENSGALG00000054286 | apelin | -0.50 | 2.0328 | 7.7075 | 5.50E-03 | 4.07E-02 |
| LOC107052651 | - - - - - | uncharacterized LOC107052651 | -0.50 | 3.5272 | 11.3268 | 7.64E-04 | 9.85E-03 |
| MNR2 | ENSGALG00000011349 | homeodomain protein | -0.50 | 5.6768 | 7.4525 | 6.33E-03 | 4.53E-02 |
| LOC112530211 | - - - - - | uncharacterized LOC112530211 | -0.50 | 1.7194 | 8.3054 | 3.95E-03 | 3.22E-02 |
| TEC | ENSGALG00000014124 | tec protein tyrosine kinase | -0.50 | 1.8933 | 9.1109 | 2.54E-03 | 2.35E-02 |
| NT5C1A | ENSGALG00000003816 | 5'-nucleotidase, cytosolic IA | -0.49 | 2.4410 | 11.6818 | 6.31E-04 | 8.58E-03 |
| AIFM2 | ENSGALG00000004777 | apoptosis inducing factor, mitochondria associated 2 | -0.49 | 2.9145 | 8.3284 | 3.90E-03 | 3.19E-02 |
| LOC112532097 | - - - - - | uncharacterized LOC112532097 | -0.49 | 2.6873 | 10.9920 | 9.15E-04 | 1.13E-02 |
| SLC36A4 | ENSGALG00000017227 | solute carrier family 36 member 4 | -0.49 | 3.1699 | 17.2797 | 3.23E-05 | 8.27E-04 |
| STK11IP | ENSGALG00000033515 | serine/threonine kinase 11 interacting protein | -0.49 | 4.2130 | 22.7902 | 1.81E-06 | 7.75E-05 |
| TRIM7.2 | ENSGALG00000038278 | Gallus gallus tripartite motif containing 7.2 (TRIM7.2), mRNA. | -0.49 | 5.5297 | 25.1390 | 5.33E-07 | 2.84E-05 |
| NOL7 | ENSGALG00000012695 | nucleolar protein 7 | -0.48 | 6.0179 | 45.5231 | 1.51E-11 | 2.82E-09 |
| NACAD | ENSGALG00000050931 | NAC alpha domain containing | -0.48 | 4.7965 | 12.8265 | 3.42E-04 | 5.44E-03 |
| COLGALT2 | ENSGALG00000038320 | collagen beta(1-O)galactosyltransferase 1 | -0.48 | 4.4140 | 10.5099 | 1.19E-03 | 1.36E-02 |
| UNC80 | ENSGALG00000041563 | unc-80 homolog, NALCN activator | -0.48 | 3.6643 | 9.0244 | 2.66E-03 | 2.43E-02 |

| | | | | | | | |
|---|---|---|---|---|---|---|---|
| LOC101751423 | - - - - - | uncharacterized LOC101751423 | -0.48 | 6.7619 | 23.9539 | 9.87E-07 | 4.76E-05 |
| LPIN1 | ENSGALG00000016456 | lipin 1 | -0.48 | 4.6155 | 17.6077 | 2.71E-05 | 7.24E-04 |
| FAM20C | ENSGALG00000038768 | FAM20C, golgi associated secretory pathway kinase | -0.48 | 4.1433 | 8.2914 | 3.98E-03 | 3.24E-02 |
| TFCP2L1 | ENSGALG00000011635 | transcription factor CP2 like 1 | -0.48 | 4.4176 | 9.7177 | 1.82E-03 | 1.88E-02 |
| SORBS2 | ENSGALG00000013427 | sorbin and SH3 domain containing 2 | -0.48 | 4.8485 | 18.5248 | 1.68E-05 | 4.89E-04 |
| LOC107054413 | - - - - - | uncharacterized LOC107054413 | -0.47 | 3.7496 | 12.0633 | 5.14E-04 | 7.40E-03 |
| LOC107051951 | - - - - - | uncharacterized LOC107051951 | -0.47 | 2.3732 | 7.7675 | 5.32E-03 | 3.99E-02 |
| DGKI | ENSGALG00000012890 | diacylglycerol kinase iota | -0.47 | 2.0171 | 7.4340 | 6.40E-03 | 4.56E-02 |
| PCDH15 | ENSGALG00000002744 | protocadherin related 15 | -0.47 | 4.3080 | 8.2888 | 3.99E-03 | 3.24E-02 |
| GDAP1L1 | ENSGALG00000004309 | ganglioside induced differentiation associated protein 1 like 1 | -0.47 | 4.8989 | 33.5834 | 6.83E-09 | 6.21E-07 |
| LOC422757 | - - - - - | uncharacterized LOC422757 | -0.47 | 4.8054 | 14.3827 | 1.49E-04 | 2.82E-03 |
| SULT6B1 | ENSGALG00000046375 | sulfotransferase family, cytosolic, 6B, member 1 | -0.47 | 2.6071 | 10.6230 | 1.12E-03 | 1.30E-02 |
| LOC107049262 | - - - - - | uncharacterized LOC107049262 | -0.47 | 3.0852 | 10.7002 | 1.07E-03 | 1.26E-02 |
| TRHDE | ENSGALG00000010200 | thyrotropin releasing hormone degrading enzyme | -0.47 | 2.9299 | 11.6313 | 6.48E-04 | 8.78E-03 |
| IFNGR1 | ENSGALG00000013865 | interferon gamma receptor 1 | -0.47 | 5.1815 | 21.7961 | 3.03E-06 | 1.21E-04 |
| COBL | ENSGALG00000013102 | cordon-bleu WH2 repeat protein | -0.47 | 3.2638 | 7.3740 | 6.62E-03 | 4.65E-02 |
| ITM2C | ENSGALG00000036111 | integral membrane protein 2C | -0.47 | 3.4699 | 18.6850 | 1.54E-05 | 4.61E-04 |
| NAV3 | ENSGALG00000010311 | neuron navigator 3 | -0.47 | 3.5275 | 13.1817 | 2.83E-04 | 4.72E-03 |
| MFSD9 | - - - - - | major facilitator superfamily domain containing 9 | -0.46 | 4.5647 | 33.7425 | 6.29E-09 | 5.78E-07 |
| GABRD | ENSGALG00000001282 | gamma-aminobutyric acid type A receptor delta subunit | -0.46 | 1.8535 | 7.1934 | 7.32E-03 | 4.98E-02 |
| TCTN1 | ENSGALG00000041861 | tectonic family member 1 | -0.46 | 2.1608 | 11.6150 | 6.54E-04 | 8.83E-03 |
| TAL2 | ENSGALG00000054131 | T-cell acute lymphocytic leukemia 2 | -0.46 | 3.2324 | 7.5729 | 5.93E-03 | 4.30E-02 |
| ARSI | ENSGALG00000005616 | arylsulfatase family member I | -0.46 | 6.2115 | 13.1760 | 2.84E-04 | 4.72E-03 |
| GPR1 | ENSGALG00000008589 | G protein-coupled receptor 1 | -0.46 | 5.2440 | 39.8393 | 2.76E-10 | 3.63E-08 |
| MSH4 | ENSGALG00000011392 | mutS homolog 4 | -0.46 | 5.5881 | 21.4923 | 3.55E-06 | 1.38E-04 |
| ARRDC1 | ENSGALG00000008475 | arrestin domain containing 1 | -0.46 | 3.9230 | 15.7021 | 7.41E-05 | 1.65E-03 |
| C1QTNF5 | ENSGALG00000006758 | C1q and tumor necrosis factor related protein 5 | -0.46 | 2.3963 | 7.2264 | 7.18E-03 | 4.91E-02 |
| TMEM173 | ENSGALG00000041129 | transmembrane protein 173 | -0.46 | 2.5911 | 8.9860 | 2.72E-03 | 2.46E-02 |
| SCG5 | ENSGALG00000009725 | secretogranin V | -0.46 | 2.6498 | 9.3771 | 2.20E-03 | 2.15E-02 |
| DUSP27 | ENSGALG00000015453 | dual specificity phosphatase 27 (putative) | -0.46 | 3.0917 | 7.3207 | 6.82E-03 | 4.74E-02 |
| RWDD2A | ENSGALG00000015851 | RWD domain containing 2A | -0.46 | 3.2433 | 10.9203 | 9.51E-04 | 1.16E-02 |
| ASCC2 | ENSGALG00000008059 | activating signal cointegrator 1 complex subunit 2 | -0.46 | 5.8472 | 53.1190 | 3.14E-13 | 9.39E-11 |
| RFT1 | ENSGALG00000001945 | RFT1 homolog | -0.45 | 5.5045 | 23.8357 | 1.05E-06 | 4.96E-05 |
| ACAD6L | ENSGALG00000035994 | probable acyl coa dehydrogenase 6-like | -0.45 | 4.1806 | 23.9311 | 9.98E-07 | 4.79E-05 |
| LIMCH1 | - - - - - | LIM and calponin homology domains 1 | -0.45 | 5.3642 | 18.7906 | 1.46E-05 | 4.40E-04 |
| COL9A1 | ENSGALG00000015970 | collagen type IX alpha 1 chain | -0.45 | 4.7149 | 11.8294 | 5.83E-04 | 8.11E-03 |
| OCLN | ENSGALG00000037316 | occludin | -0.45 | 4.1083 | 23.7723 | 1.08E-06 | 5.07E-05 |
| ADGRB2 | ENSGALG00000043209 | adhesion G protein-coupled receptor B2 | -0.45 | 3.1762 | 7.7129 | 5.48E-03 | 4.07E-02 |
| MAGI2 | ENSGALG00000038834 | membrane associated guanylate kinase, WW and PDZ domain containing 2 | -0.44 | 4.1653 | 12.2622 | 4.62E-04 | 6.83E-03 |
| MOB3C | ENSGALG00000025946 | MOB kinase activator 3C | -0.44 | 2.4430 | 7.7181 | 5.47E-03 | 4.06E-02 |
| STAMBPL1 | ENSGALG00000006331 | STAM binding protein like 1 | -0.44 | 2.8035 | 10.6393 | 1.11E-03 | 1.29E-02 |
| LOC112533257 | - - - - - | uncharacterized LOC112533257 | -0.44 | 2.9611 | 8.6359 | 3.30E-03 | 2.83E-02 |
| PSEN1 | ENSGALG00000009320 | presenilin 1 | -0.44 | 5.9815 | 41.9252 | 9.48E-11 | 1.44E-08 |
| AR | ENSGALG00000030879 | androgen receptor | -0.44 | 4.6892 | 8.5818 | 3.40E-03 | 2.88E-02 |

| | | | | | | | |
|---|---|---|---|---|---|---|---|
| CA10 | ENSGALG00000002993 | carbonic anhydrase 10 | -0.44 | 3.6282 | 7.8180 | 5.17E-03 | 3.91E-02 |
| SELENBP1 | ENSGALG00000000866 | selenium binding protein 1 | -0.44 | 3.9044 | 11.8281 | 5.83E-04 | 8.11E-03 |
| PQLC3 | - - - - - | PQ loop repeat containing 3 | -0.44 | 4.4517 | 19.1756 | 1.19E-05 | 3.74E-04 |
| GUCY1B4 | ENSGALG00000005076 | guanylate cyclase 1, soluble, beta 4 | -0.44 | 3.0206 | 11.3608 | 7.50E-04 | 9.74E-03 |
| ADRA2B | ENSGALG00000052016 | adrenoceptor alpha 2B | -0.44 | 2.6648 | 7.3044 | 6.88E-03 | 4.77E-02 |
| PASD1 | ENSGALG00000009063 | PAS domain containing 1 | -0.44 | 3.3350 | 10.0901 | 1.49E-03 | 1.61E-02 |
| POSTN | ENSGALG00000017046 | periostin | -0.44 | 7.8263 | 21.7268 | 3.14E-06 | 1.24E-04 |
| NQO2 | ENSGALG00000025747 | NAD(P)H quinone dehydrogenase 2 | -0.44 | 4.1038 | 20.3135 | 6.57E-06 | 2.31E-04 |
| LOC396224 | - - - - - | lamin A/C | -0.43 | 4.6344 | 19.3934 | 1.06E-05 | 3.42E-04 |
| LOC107052740 | - - - - - | uncharacterized LOC107052740 | -0.43 | 4.0481 | 25.3320 | 4.83E-07 | 2.60E-05 |
| KIAA1143 | ENSGALG00000035952 | KIAA1143 | -0.43 | 5.7346 | 22.2879 | 2.35E-06 | 9.73E-05 |
| MEGF11 | ENSGALG00000007631 | multiple EGF like domains 11 | -0.43 | 3.0974 | 8.6029 | 3.36E-03 | 2.87E-02 |
| CPQ | ENSGALG00000030866 | carboxypeptidase Q | -0.43 | 3.8750 | 8.3087 | 3.95E-03 | 3.22E-02 |
| NTL | ENSGALG00000005156 | neurotrypsin-like | -0.43 | 4.5426 | 22.2556 | 2.39E-06 | 9.87E-05 |
| PPP1R17 | ENSGALG00000036390 | protein phosphatase 1 regulatory subunit 17 | -0.43 | 4.7114 | 7.8748 | 5.01E-03 | 3.83E-02 |
| DNAH1 | ENSGALG00000004297 | dynein axonemal heavy chain 1 | -0.43 | 3.2262 | 8.4658 | 3.62E-03 | 3.03E-02 |
| HS3ST3B1L | - - - - - | heparan sulfate glucosamine 3-O-sulfotransferase 3B1-like | -0.43 | 4.9266 | 7.5576 | 5.98E-03 | 4.33E-02 |
| LOC101747846 | - - - - - | uncharacterized LOC101747846 | -0.43 | 2.6770 | 8.7903 | 3.03E-03 | 2.66E-02 |
| LOC112529987 | - - - - - | uncharacterized LOC112529987 | -0.43 | 2.9333 | 8.6517 | 3.27E-03 | 2.81E-02 |
| SCUBE3 | ENSGALG00000002675 | signal peptide, CUB domain and EGF like domain containing 3 | -0.43 | 6.8607 | 12.0043 | 5.31E-04 | 7.58E-03 |
| PGGHG | ENSGALG00000004234 | protein-glucosylgalactosylhydroxylysine glucosidase | -0.43 | 3.6153 | 16.5974 | 4.62E-05 | 1.12E-03 |
| MYBL1 | ENSGALG00000033304 | MYB proto-oncogene like 1 | -0.43 | 4.2112 | 13.2031 | 2.79E-04 | 4.67E-03 |
| HELB | ENSGALG00000009885 | DNA helicase B | -0.43 | 4.8840 | 33.7861 | 6.15E-09 | 5.69E-07 |
| LOC107050995 | - - - - - | neuralized-like protein 4 | -0.43 | 5.4411 | 33.9857 | 5.55E-09 | 5.19E-07 |
| SYTL4 | ENSGALG00000006786 | synaptotagmin like 4 | -0.43 | 4.4535 | 20.2812 | 6.69E-06 | 2.34E-04 |
| CTBS | ENSGALG00000008780 | chitobiase | -0.43 | 3.7617 | 15.0148 | 1.07E-04 | 2.16E-03 |
| CACNA1D | ENSGALG00000005332 | calcium voltage-gated channel subunit alpha1 D | -0.42 | 4.4649 | 10.9867 | 9.18E-04 | 1.13E-02 |
| MAT2B | ENSGALG00000001750 | methionine adenosyltransferase 2B | -0.42 | 5.5876 | 40.2989 | 2.18E-10 | 3.03E-08 |
| RNF144B | ENSGALG00000012683 | ring finger protein 144B | -0.42 | 1.8774 | 7.5805 | 5.90E-03 | 4.29E-02 |
| TBC1D8 | ENSGALG00000016776 | TBC1 domain family member 8 | -0.42 | 4.4025 | 17.2547 | 3.27E-05 | 8.37E-04 |
| LOC112533441 | - - - - - | uncharacterized LOC112533441 | -0.42 | 3.4200 | 13.4126 | 2.50E-04 | 4.30E-03 |
| THBS4 | ENSGALG00000014804 | thrombospondin 4 | -0.42 | 5.5646 | 20.1294 | 7.24E-06 | 2.50E-04 |
| KLB | ENSGALG00000041663 | klotho beta | -0.42 | 3.6054 | 13.5262 | 2.35E-04 | 4.09E-03 |
| TATDN3 | ENSGALG00000009811 | TatD DNase domain containing 3 | -0.42 | 4.1375 | 19.0457 | 1.28E-05 | 3.94E-04 |
| MAP7D2 | ENSGALG00000016415 | MAP7 domain containing 2 | -0.42 | 5.1146 | 19.7254 | 8.94E-06 | 2.98E-04 |
| SLC7A2 | ENSGALG00000013627 | solute carrier family 7 member 2 | -0.42 | 4.1304 | 14.7372 | 1.24E-04 | 2.43E-03 |
| AKR1B10L3 | ENSGALG00000019284 | aldo-keto reductase family 1 member B10-like | -0.42 | 5.4831 | 13.0561 | 3.02E-04 | 4.95E-03 |
| PROCR | ENSGALG00000006547 | protein C receptor | -0.42 | 2.2834 | 9.3125 | 2.28E-03 | 2.19E-02 |
| UVRAG | ENSGALG00000000845 | UV radiation resistance associated | -0.42 | 4.2202 | 15.6901 | 7.46E-05 | 1.65E-03 |
| MYH11 | ENSGALG00000006520 | myosin, heavy chain 11, smooth muscle | -0.42 | 5.5091 | 8.4461 | 3.66E-03 | 3.06E-02 |
| LOC112531627 | - - - - - | small nucleolar RNA SNORA31 | -0.42 | 2.7444 | 8.3634 | 3.83E-03 | 3.15E-02 |
| ARHGAP40 | ENSGALG00000003550 | Rho GTPase activating protein 40 | -0.42 | 4.4838 | 10.4207 | 1.25E-03 | 1.40E-02 |
| ADAMTS3 | ENSGALG00000011623 | ADAM metallopeptidase with thrombospondin type 1 motif 3 | -0.42 | 5.8559 | 24.5727 | 7.16E-07 | 3.65E-05 |
| GPC6 | ENSGALG00000016900 | glypican 6 | -0.42 | 2.6936 | 7.1938 | 7.32E-03 | 4.98E-02 |

| | | | | | | | |
|---|---|---|---|---|---|---|---|
| LOC112532514 | - - - - - | golgin subfamily B member 1-like | -0.42 | 3.4478 | 12.1005 | 5.04E-04 | 7.30E-03 |
| GRTP1 | ENSGALG00000016828 | growth hormone regulated TBC protein 1 | -0.42 | 3.1535 | 10.7293 | 1.05E-03 | 1.25E-02 |
| MICU2 | ENSGALG00000017125 | mitochondrial calcium uptake 2 | -0.42 | 6.0676 | 59.8667 | 1.02E-14 | 3.88E-12 |
| ABCG4 | ENSGALG00000006795 | ATP binding cassette subfamily G member 4 | -0.42 | 3.3358 | 9.1394 | 2.50E-03 | 2.32E-02 |
| SFI1 | ENSGALG00000051488 | SFI1 centrin binding protein | -0.41 | 5.1941 | 34.1279 | 5.16E-09 | 4.86E-07 |
| UNC79 | ENSGALG00000010874 | unc-79 homolog (C. elegans) | -0.41 | 4.3139 | 7.7980 | 5.23E-03 | 3.95E-02 |
| JMJD4 | ENSGALG00000005417 | jumonji domain containing 4 | -0.41 | 6.4313 | 43.8155 | 3.61E-11 | 6.12E-09 |
| TMEM106B | ENSGALG00000035140 | transmembrane protein 106B | -0.41 | 5.5629 | 21.1296 | 4.29E-06 | 1.62E-04 |
| PMLL | - - - - - | promyelocytic leukemia-like | -0.41 | 2.7921 | 9.1806 | 2.45E-03 | 2.29E-02 |
| SLX4 | ENSGALG00000031841 | SLX4 structure-specific endonuclease subunit homolog | -0.41 | 6.4257 | 23.9060 | 1.01E-06 | 4.82E-05 |
| TBC1D19 | ENSGALG00000029673 | TBC1 domain family member 19 | -0.41 | 4.4972 | 26.5718 | 2.54E-07 | 1.48E-05 |
| GON4L | - - - - - | gon-4 like | -0.41 | 6.3058 | 23.0314 | 1.59E-06 | 6.97E-05 |
| ACADSB | ENSGALG00000041417 | acyl-CoA dehydrogenase, short/branched chain | -0.41 | 5.7961 | 20.2059 | 6.95E-06 | 2.42E-04 |
| ISPD | ENSGALG00000010795 | isoprenoid synthase domain containing | -0.41 | 4.7572 | 19.1678 | 1.20E-05 | 3.75E-04 |
| ZFAND2A | ENSGALG00000003122 | zinc finger AN1-type containing 2A | -0.41 | 4.0705 | 9.4883 | 2.07E-03 | 2.07E-02 |
| SLC16A14 | ENSGALG00000002897 | solute carrier family 16 member 14 | -0.41 | 4.8796 | 9.7929 | 1.75E-03 | 1.82E-02 |
| INF2 | ENSGALG00000011608 | inverted formin, FH2 and WH2 domain containing | -0.41 | 3.4949 | 10.2232 | 1.39E-03 | 1.52E-02 |
| FAM84B | - - - - - | family with sequence similarity 84 member B | -0.41 | 4.7994 | 9.7564 | 1.79E-03 | 1.85E-02 |
| MPP3 | ENSGALG00000042521 | membrane palmitoylated protein 3 | -0.40 | 4.4300 | 14.1825 | 1.66E-04 | 3.08E-03 |
| SLC9A7 | ENSGALG00000016734 | solute carrier family 9 member A7 | -0.40 | 3.3638 | 10.8592 | 9.83E-04 | 1.19E-02 |
| DNASE2B | ENSGALG00000028790 | deoxyribonuclease 2 beta | -0.40 | 3.3624 | 13.0947 | 2.96E-04 | 4.88E-03 |
| MANBAL15 | - - - - - | ----- | -0.40 | 4.2546 | 10.9417 | 9.40E-04 | 1.15E-02 |
| DGAT2 | ENSGALG00000040418 | diacylglycerol O-acyltransferase 2 | -0.40 | 2.5207 | 7.7861 | 5.26E-03 | 3.96E-02 |
| PHKA2 | ENSGALG00000016518 | phosphorylase kinase regulatory subunit alpha 2 | -0.40 | 4.9122 | 27.1375 | 1.89E-07 | 1.17E-05 |
| HEMK1 | ENSGALG00000002253 | HemK methyltransferase family member 1 | -0.40 | 4.8224 | 14.2328 | 1.62E-04 | 3.00E-03 |
| DERA | ENSGALG00000013095 | deoxyribose-phosphate aldolase | -0.40 | 4.5685 | 11.6157 | 6.54E-04 | 8.83E-03 |
| TMEM130 | ENSGALG00000003569 | transmembrane protein 130 | -0.40 | 2.9258 | 12.0331 | 5.23E-04 | 7.50E-03 |
| ACBD5 | ENSGALG00000007519 | acyl-CoA binding domain containing 5 | -0.40 | 4.8245 | 19.4947 | 1.01E-05 | 3.27E-04 |
| LOC107050437 | - - - - - | plectin-like | -0.40 | 3.0119 | 7.2476 | 7.10E-03 | 4.87E-02 |
| AFMID | ENSGALG00000000109 | arylformamidase | -0.40 | 5.5823 | 22.0910 | 2.60E-06 | 1.06E-04 |
| NRDE2 | ENSGALG00000010677 | NRDE-2, necessary for RNA interference, domain containing | -0.39 | 5.5148 | 27.7599 | 1.37E-07 | 8.97E-06 |
| TCP11L2 | - - - - - | ----- | -0.39 | 5.1269 | 20.0174 | 7.67E-06 | 2.64E-04 |
| NDRG4 | ENSGALG00000043662 | NDRG family member 4 | -0.39 | 5.3637 | 18.7851 | 1.46E-05 | 4.40E-04 |
| LHFPL3 | ENSGALG00000043367 | LHFPL tetraspan subfamily member 3 | -0.39 | 3.2799 | 10.0652 | 1.51E-03 | 1.63E-02 |
| SPRTN | ENSGALG00000011065 | SprT-like N-terminal domain | -0.39 | 5.3259 | 23.3563 | 1.35E-06 | 6.09E-05 |
| MT | - - - - - | ----- | -0.39 | 4.5123 | 12.2969 | 4.54E-04 | 6.73E-03 |
| IL17RA | ENSGALG00000033887 | interleukin 17 receptor A | -0.39 | 5.3280 | 18.2858 | 1.90E-05 | 5.41E-04 |
| LOC107053353 | - - - - - | dispanin subfamily A member 2b-like | -0.39 | 3.3824 | 9.0777 | 2.59E-03 | 2.38E-02 |
| CILP | ENSGALG00000007358 | cartilage intermediate layer protein | -0.39 | 3.0687 | 9.3896 | 2.18E-03 | 2.14E-02 |
| RAB4A | ENSGALG00000036218 | RAB4A, member RAS oncogene family | -0.39 | 5.2281 | 23.3002 | 1.39E-06 | 6.21E-05 |
| MMRN1 | ENSGALG00000010391 | multimerin 1 | -0.38 | 3.7898 | 10.3267 | 1.31E-03 | 1.46E-02 |
| IRF5 | ENSGALG00000039982 | interferon regulatory factor 5 | -0.38 | 3.1202 | 9.8334 | 1.71E-03 | 1.78E-02 |
| ANKRD52 | ENSGALG00000031973 | ankyrin repeat domain 52 | -0.38 | 5.9130 | 18.5311 | 1.67E-05 | 4.89E-04 |
| ZNF692 | ENSGALG00000041845 | zinc finger protein 692 | -0.38 | 4.4944 | 7.8317 | 5.13E-03 | 3.89E-02 |

| | | | | | | | |
|---|---|---|---|---|---|---|---|
| BAIAP2 | ENSGALG00000042538 | BAI1 associated protein 2 | -0.38 | 4.6891 | 13.0654 | 3.01E-04 | 4.94E-03 |
| IFT57 | ENSGALG00000015356 | intraflagellar transport 57 | -0.38 | 4.8745 | 12.8621 | 3.35E-04 | 5.35E-03 |
| MSRB3 | ENSGALG00000026661 | methionine sulfoxide reductase B3 | -0.38 | 3.8680 | 9.3214 | 2.26E-03 | 2.18E-02 |
| RIN3 | ENSGALG00000010806 | Ras and Rab interactor 3 | -0.38 | 3.3086 | 10.1836 | 1.42E-03 | 1.55E-02 |
| GALNT13 | ENSGALG00000012536 | polypeptide N-acetylgalactosaminyltransferase 13 | -0.38 | 4.2972 | 9.7756 | 1.77E-03 | 1.83E-02 |
| COG3 | ENSGALG00000016980 | component of oligomeric golgi complex 3 | -0.38 | 5.5091 | 28.2393 | 1.07E-07 | 7.17E-06 |
| BTN1 | ENSGALG00000052240 | B-butyrophilin 1 | -0.38 | 4.5159 | 15.1194 | 1.01E-04 | 2.08E-03 |
| CAPN2 | ENSGALG00000009360 | calpain 2 | -0.38 | 3.9266 | 11.0946 | 8.66E-04 | 1.08E-02 |
| LOC107057318 | - - - - - | uncharacterized LOC107057318 | -0.38 | 4.9907 | 23.9444 | 9.92E-07 | 4.77E-05 |
| LOC100858295 | - - - - - | uncharacterized LOC100858295 | -0.37 | 4.0369 | 8.2615 | 4.05E-03 | 3.27E-02 |
| AZIN2 | ENSGALG00000003614 | arginine decarboxylase | -0.37 | 3.6679 | 9.3427 | 2.24E-03 | 2.17E-02 |
| TNFRSF11A | ENSGALG00000045642 | TNF receptor superfamily member 11a | -0.37 | 3.4423 | 9.2078 | 2.41E-03 | 2.27E-02 |
| NPNT | ENSGALG00000043555 | nephronectin | -0.37 | 4.8912 | 9.5526 | 2.00E-03 | 2.01E-02 |
| CHRD | ENSGALG00000026983 | chordin | -0.37 | 3.3149 | 9.5537 | 2.00E-03 | 2.01E-02 |
| BTBD11 | ENSGALG00000039595 | BTB domain containing 11 | -0.37 | 4.9477 | 14.1235 | 1.71E-04 | 3.16E-03 |
| CKLF | ENSGALG00000005254 | chemokine like factor | -0.37 | 3.2512 | 9.4065 | 2.16E-03 | 2.12E-02 |
| MED12L | ENSGALG00000010370 | mediator complex subunit 12 like | -0.37 | 5.5303 | 22.4807 | 2.12E-06 | 8.90E-05 |
| KIF21A | ENSGALG00000007098 | kinesin family member 21A | -0.37 | 6.6450 | 25.4434 | 4.56E-07 | 2.49E-05 |
| RMDN2 | ENSGALG00000010554 | regulator of microtubule dynamics 2 | -0.37 | 2.8907 | 8.1792 | 4.24E-03 | 3.38E-02 |
| LOC107052931 | - - - - - | uncharacterized LOC107052931 | -0.37 | 3.0329 | 7.9462 | 4.82E-03 | 3.73E-02 |
| RPS6KC1 | ENSGALG00000009796 | ribosomal protein S6 kinase C1 | -0.37 | 5.5215 | 22.5318 | 2.07E-06 | 8.70E-05 |
| KLHL42 | ENSGALG00000017296 | kelch like family member 42 | -0.36 | 2.6081 | 7.7460 | 5.38E-03 | 4.02E-02 |
| LYRM2 | ENSGALG00000015762 | LYR motif containing 2 | -0.36 | 3.7619 | 12.5834 | 3.89E-04 | 6.00E-03 |
| TTBK1 | ENSGALG00000008627 | tau tubulin kinase 1 | -0.36 | 5.7080 | 19.3713 | 1.08E-05 | 3.45E-04 |
| LAMA2 | - - - - - | laminin subunit alpha 2 | -0.36 | 4.6300 | 12.2627 | 4.62E-04 | 6.83E-03 |
| TTLL7 | ENSGALG00000008850 | tubulin tyrosine ligase like 7 | -0.36 | 3.8677 | 11.6968 | 6.26E-04 | 8.54E-03 |
| CNST | ENSGALG00000010649 | consortin, connexin sorting protein | -0.36 | 4.4966 | 17.1610 | 3.43E-05 | 8.73E-04 |
| FRRS1 | ENSGALG00000005418 | ferric chelate reductase 1 | -0.36 | 4.4469 | 9.8776 | 1.67E-03 | 1.76E-02 |
| KLHL14 | ENSGALG00000036969 | kelch like family member 14 | -0.36 | 3.5778 | 9.4449 | 2.12E-03 | 2.09E-02 |
| CGN | ENSGALG00000000920 | cingulin | -0.36 | 4.1249 | 9.3477 | 2.23E-03 | 2.17E-02 |
| EDNRB | ENSGALG00000016912 | endothelin receptor type B | -0.36 | 4.8768 | 10.5301 | 1.17E-03 | 1.35E-02 |
| CNTN3 | ENSGALG00000007862 | contactin 3 | -0.36 | 3.4652 | 11.4529 | 7.14E-04 | 9.41E-03 |
| PDLIM1 | ENSGALG00000005422 | PDZ and LIM domain 1 | -0.36 | 4.0361 | 10.7630 | 1.04E-03 | 1.23E-02 |
| ACSS3 | ENSGALG00000010940 | acyl-CoA synthetase short-chain family member 3 | -0.36 | 4.1770 | 9.1520 | 2.48E-03 | 2.31E-02 |
| C4HCXorf57 | - - - - - | ----- | -0.36 | 4.1318 | 11.4956 | 6.98E-04 | 9.24E-03 |
| TESK2 | ENSGALG00000010230 | testis-specific kinase 2 | -0.36 | 3.9473 | 9.0911 | 2.57E-03 | 2.37E-02 |
| LOC107051594 | - - - - - | uncharacterized LOC107051594 | -0.36 | 3.3726 | 12.5571 | 3.95E-04 | 6.06E-03 |
| MOGAT1 | ENSGALG00000005217 | monoacylglycerol O-acyltransferase 1 | -0.36 | 2.8271 | 8.4457 | 3.66E-03 | 3.06E-02 |
| FAM198A | - - - - - | family with sequence similarity 198 member A | -0.36 | 4.5853 | 9.8718 | 1.68E-03 | 1.76E-02 |
| DPH2 | ENSGALG00000010089 | DPH2 homolog | -0.36 | 4.2432 | 15.3306 | 9.02E-05 | 1.91E-03 |
| LOC100858426 | - - - - - | uncharacterized LOC100858426 | -0.36 | 3.2703 | 9.4362 | 2.13E-03 | 2.09E-02 |
| FZD6 | ENSGALG00000030626 | frizzled class receptor 6 | -0.36 | 5.5087 | 21.3737 | 3.78E-06 | 1.45E-04 |
| PATJ | ENSGALG00000010934 | PATJ, crumbs cell polarity complex component | -0.35 | 4.4341 | 11.2925 | 7.78E-04 | 9.99E-03 |
| ZYG11B | - - - - - | ----- | -0.35 | 5.3889 | 24.5104 | 7.39E-07 | 3.73E-05 |

| | | | | | | | |
|---|---|---|---|---|---|---|---|
| PLEKHA6 | ENSGALG00000038399 | pleckstrin homology domain containing A6 | -0.35 | 5.3491 | 13.7630 | 2.07E-04 | 3.68E-03 |
| ODF3L15 | - - - - - | ----- | -0.35 | 6.3048 | 9.5987 | 1.95E-03 | 1.97E-02 |
| ST3GAL2L | - - - - - | ----- | -0.35 | 3.3701 | 10.0253 | 1.54E-03 | 1.65E-02 |
| PCK2 | ENSGALG00000052014 | phosphoenolpyruvate carboxykinase 2, mitochondrial | -0.35 | 5.6270 | 15.4048 | 8.68E-05 | 1.85E-03 |
| TMOD2 | ENSGALG00000004740 | tropomodulin 2 | -0.35 | 4.2813 | 9.4395 | 2.12E-03 | 2.09E-02 |
| WWP1 | ENSGALG00000041795 | WW domain containing E3 ubiquitin protein ligase 1 | -0.35 | 5.3182 | 14.5968 | 1.33E-04 | 2.57E-03 |
| ZFYVE26 | ENSGALG00000028762 | zinc finger FYVE-type containing 26 | -0.35 | 5.1024 | 16.0677 | 6.11E-05 | 1.41E-03 |
| SMYD3 | ENSGALG00000010653 | SET and MYND domain containing 3 | -0.35 | 3.4200 | 8.3774 | 3.80E-03 | 3.13E-02 |
| CHRM4 | ENSGALG00000008365 | Gallus gallus cholinergic receptor muscarinic 4 (CHRM4), mRNA. | -0.35 | 4.6567 | 8.4300 | 3.69E-03 | 3.08E-02 |
| LRP8 | ENSGALG00000010692 | LDL receptor related protein 8 | -0.35 | 5.1100 | 14.3019 | 1.56E-04 | 2.92E-03 |
| GNG11 | - - - - - | G protein subunit gamma 11 | -0.35 | 3.3980 | 7.3316 | 6.78E-03 | 4.72E-02 |
| RMND1 | ENSGALG00000012968 | required for meiotic nuclear division 1 homolog | -0.35 | 5.2107 | 33.3091 | 7.86E-09 | 6.95E-07 |
| MBTPS2 | ENSGALG00000016382 | membrane bound transcription factor peptidase, site 2 | -0.35 | 4.2655 | 14.6642 | 1.28E-04 | 2.51E-03 |
| ZFYVE19 | ENSGALG00000008460 | zinc finger FYVE-type containing 19 | -0.35 | 6.3979 | 15.0390 | 1.05E-04 | 2.15E-03 |
| CSGALNACT1 | ENSGALG00000010125 | chondroitin sulfate N-acetylgalactosaminyltransferase 1 | -0.35 | 3.1728 | 8.6192 | 3.33E-03 | 2.85E-02 |
| PLTP | ENSGALG00000036686 | phospholipid transfer protein | -0.35 | 4.5260 | 7.3004 | 6.89E-03 | 4.78E-02 |
| ABCC3 | ENSGALG00000007522 | ATP binding cassette subfamily C member 3 | -0.35 | 6.2814 | 13.1582 | 2.86E-04 | 4.76E-03 |
| CUL9 | - - - - - | cullin 9 | -0.35 | 5.2247 | 18.7738 | 1.47E-05 | 4.42E-04 |
| PECR | ENSGALG00000011497 | peroxisomal trans-2-enoyl-CoA reductase | -0.35 | 4.7793 | 20.5443 | 5.83E-06 | 2.10E-04 |
| TFB1M | ENSGALG00000013647 | transcription factor B1, mitochondrial | -0.35 | 4.3925 | 10.7359 | 1.05E-03 | 1.25E-02 |
| MORC2 | ENSGALG00000007512 | MORC family CW-type zinc finger 1 | -0.34 | 6.5408 | 20.8936 | 4.86E-06 | 1.81E-04 |
| PIK3CD | ENSGALG00000002583 | phosphatidylinositol-4,5-bisphosphate 3-kinase catalytic subunit delta | -0.34 | 4.2282 | 11.9685 | 5.41E-04 | 7.70E-03 |
| MYL12B | ENSGALG00000038635 | myosin light chain 12B | -0.34 | 4.3564 | 11.1930 | 8.21E-04 | 1.04E-02 |
| SIL1 | ENSGALG00000002457 | SIL1 nucleotide exchange factor | -0.34 | 3.5648 | 7.6233 | 5.76E-03 | 4.21E-02 |
| ELMO3 | ENSGALG00000030220 | engulfment and cell motility 3 | -0.34 | 3.4918 | 10.1332 | 1.46E-03 | 1.58E-02 |
| ELP6 | ENSGALG00000031227 | elongator acetyltransferase complex subunit 6 | -0.34 | 4.0837 | 7.2052 | 7.27E-03 | 4.96E-02 |
| SOX11 | ENSGALG00000016397 | SRY-box 11 | -0.34 | 6.8148 | 19.2698 | 1.13E-05 | 3.61E-04 |
| ALCAM | ENSGALG00000015348 | activated leukocyte cell adhesion molecule | -0.34 | 6.5977 | 8.5638 | 3.43E-03 | 2.91E-02 |
| COL25A1 | ENSGALG00000039411 | collagen type XXV alpha 1 chain | -0.34 | 4.6909 | 10.3398 | 1.30E-03 | 1.45E-02 |
| ISM2 | - - - - - | isthmin 2 | -0.34 | 4.8959 | 11.1339 | 8.48E-04 | 1.06E-02 |
| TBCEL | ENSGALG00000018819 | tubulin folding cofactor E like | -0.34 | 4.6710 | 15.4800 | 8.34E-05 | 1.80E-03 |
| LOC107057415 | - - - - - | uncharacterized LOC107057415 | -0.34 | 5.0325 | 16.2245 | 5.63E-05 | 1.32E-03 |
| DOCK10 | ENSGALG00000005030 | dedicator of cytokinesis 10 | -0.33 | 4.5215 | 7.7672 | 5.32E-03 | 3.99E-02 |
| RNF169 | ENSGALG00000017309 | ring finger protein 169 | -0.33 | 4.0521 | 8.6539 | 3.26E-03 | 2.81E-02 |
| STAGL | - - - - - | ----- | -0.33 | 5.8986 | 20.4456 | 6.14E-06 | 2.20E-04 |
| SARAF | ENSGALG00000011395 | store-operated calcium entry associated regulatory factor | -0.33 | 4.5391 | 12.7465 | 3.57E-04 | 5.60E-03 |
| TUB | ENSGALG00000005986 | tubby bipartite transcription factor | -0.33 | 5.4768 | 11.9080 | 5.59E-04 | 7.88E-03 |
| MAN2B2 | ENSGALG00000015537 | mannosidase alpha class 2B member 2 | -0.33 | 5.1245 | 12.5122 | 4.04E-04 | 6.19E-03 |
| DHCR24 | ENSGALG00000010798 | 24-dehydrocholesterol reductase | -0.33 | 6.1292 | 18.4770 | 1.72E-05 | 4.97E-04 |
| TRAF4 | ENSGALG00000003987 | TNF receptor associated factor 4 | -0.33 | 4.6865 | 17.6067 | 2.72E-05 | 7.24E-04 |
| LOC107050300 | - - - - - | withdrawn | -0.33 | 3.2994 | 7.3372 | 6.75E-03 | 4.71E-02 |
| CHST12 | ENSGALG00000004276 | carbohydrate sulfotransferase 12 | -0.33 | 4.0865 | 10.6960 | 1.07E-03 | 1.26E-02 |
| NUDT1 | ENSGALG00000004245 | nudix hydrolase 1 | -0.33 | 6.0313 | 18.4377 | 1.76E-05 | 5.06E-04 |
| C5H15orf57 | ENSGALG00000029332 | chromosome 5 C15orf57 homolog | -0.33 | 5.4589 | 17.2461 | 3.28E-05 | 8.39E-04 |

| | | | | | | | |
|---|---|---|---|---|---|---|---|
| TMEM169 | ENSGALG00000011493 | transmembrane protein 169 | -0.33 | 6.3634 | 16.0795 | 6.07E-05 | 1.41E-03 |
| PRICKLE4 | ENSGALG00000028262 | prickle planar cell polarity protein 4 | -0.33 | 4.0108 | 12.7415 | 3.58E-04 | 5.60E-03 |
| PIEZO2 | ENSGALG00000042134 | piezo type mechanosensitive ion channel component 2 | -0.33 | 4.4422 | 9.4298 | 2.13E-03 | 2.10E-02 |
| NUDT9 | ENSGALG00000010932 | nudix hydrolase 9 | -0.33 | 4.4305 | 10.7485 | 1.04E-03 | 1.24E-02 |
| OGFRL1 | ENSGALG00000015951 | opioid growth factor receptor like 1 | -0.32 | 4.4471 | 11.1239 | 8.52E-04 | 1.07E-02 |
| RGN | ENSGALG00000016724 | regucalcin | -0.32 | 4.6717 | 9.4450 | 2.12E-03 | 2.09E-02 |
| ATR | ENSGALG00000002663 | ATR serine/threonine kinase | -0.32 | 6.5894 | 15.4467 | 8.49E-05 | 1.82E-03 |
| MRPL43 | ENSGALG00000034352 | mitochondrial ribosomal protein L43 | -0.32 | 4.7641 | 7.9381 | 4.84E-03 | 3.74E-02 |
| NUMBL | - - - - - | ----- | -0.32 | 4.4197 | 11.2697 | 7.88E-04 | 1.01E-02 |
| JMJD7 | ENSGALG00000026846 | jumonji domain containing 7 | -0.32 | 3.5424 | 10.5474 | 1.16E-03 | 1.34E-02 |
| COBLL1 | ENSGALG00000011068 | cordon-bleu WH2 repeat protein like 1 | -0.32 | 4.2411 | 9.8469 | 1.70E-03 | 1.78E-02 |
| LOC107051290 | - - - - - | withdrawn | -0.32 | 7.2330 | 9.7300 | 1.81E-03 | 1.87E-02 |
| CADM2 | ENSGALG00000043393 | cell adhesion molecule 2 | -0.32 | 3.7571 | 7.5434 | 6.02E-03 | 4.35E-02 |
| PDPR | ENSGALG00000002690 | pyruvate dehydrogenase phosphatase regulatory subunit | -0.32 | 5.9581 | 19.8494 | 8.38E-06 | 2.84E-04 |
| PCTP | ENSGALG00000003099 | phosphatidylcholine transfer protein | -0.32 | 5.0152 | 8.1833 | 4.23E-03 | 3.38E-02 |
| DNAJC27 | ENSGALG00000028967 | DnaJ heat shock protein family (Hsp40) member C27 | -0.32 | 4.1258 | 11.1299 | 8.49E-04 | 1.07E-02 |
| ANAPC5 | ENSGALG00000004179 | anaphase promoting complex subunit 5 | -0.32 | 5.7588 | 14.8582 | 1.16E-04 | 2.30E-03 |
| TOR3A | ENSGALG00000004216 | torsin family 3 member A | -0.32 | 3.9954 | 11.3830 | 7.41E-04 | 9.66E-03 |
| CECR1 | ENSGALG00000013031 | cat eye syndrome chromosome region, candidate 1 | -0.32 | 3.2515 | 7.5977 | 5.84E-03 | 4.26E-02 |
| GSTO1 | - - - - - | ----- | -0.32 | 5.5020 | 16.7873 | 4.18E-05 | 1.03E-03 |
| MFSD4B | ENSGALG00000049668 | major facilitator superfamily domain containing 4B | -0.32 | 5.5123 | 19.7487 | 8.83E-06 | 2.97E-04 |
| LOC101750715 | - - - - - | uncharacterized LOC101750715 | -0.32 | 4.7197 | 13.2928 | 2.66E-04 | 4.52E-03 |
| TOP1MT | ENSGALG00000029572 | DNA topoisomerase I mitochondrial | -0.31 | 5.5375 | 23.0606 | 1.57E-06 | 6.91E-05 |
| DYNC1I1 | ENSGALG00000009722 | dynein cytoplasmic 1 intermediate chain 1 | -0.31 | 4.6788 | 7.4814 | 6.23E-03 | 4.47E-02 |
| IMPA1 | ENSGALG00000034487 | inositol monophosphatase 1 | -0.31 | 5.5301 | 12.5498 | 3.96E-04 | 6.08E-03 |
| LOC107057416 | - - - - - | uncharacterized LOC107057416 | -0.31 | 4.7135 | 8.5812 | 3.40E-03 | 2.88E-02 |
| PRKAA2 | ENSGALG00000010826 | protein kinase AMP-activated catalytic subunit alpha 2 | -0.31 | 3.6220 | 7.5043 | 6.16E-03 | 4.43E-02 |
| NEXN | ENSGALG00000008945 | nexilin F-actin binding protein | -0.31 | 5.2389 | 9.3525 | 2.23E-03 | 2.17E-02 |
| GLYCTK | ENSGALG00000054430 | glycerate kinase | -0.31 | 5.3033 | 10.8459 | 9.90E-04 | 1.19E-02 |
| LOC101751615 | - - - - - | uncharacterized LOC101751615 | -0.31 | 3.0496 | 7.2759 | 6.99E-03 | 4.82E-02 |
| SLC25A30 | ENSGALG00000016979 | solute carrier family 25 member 30 | -0.31 | 4.4331 | 8.3764 | 3.80E-03 | 3.13E-02 |
| CMTM6 | ENSGALG00000011490 | CKLF like MARVEL transmembrane domain containing 6 | -0.31 | 5.5229 | 17.3119 | 3.17E-05 | 8.18E-04 |
| DMXL2 | ENSGALG00000039924 | Dmx like 2 | -0.31 | 4.6979 | 9.1730 | 2.46E-03 | 2.30E-02 |
| NFKBIZ | ENSGALG00000015346 | NFKB inhibitor zeta | -0.31 | 3.8525 | 8.9906 | 2.71E-03 | 2.46E-02 |
| CCDC80 | ENSGALG00000015193 | coiled-coil domain containing 80 | -0.31 | 6.7096 | 15.0940 | 1.02E-04 | 2.10E-03 |
| VASN | ENSGALG00000047356 | vasorin | -0.31 | 6.1328 | 17.8565 | 2.38E-05 | 6.52E-04 |
| FKBP9 | ENSGALG00000037669 | FK506 binding protein 9 | -0.31 | 8.2219 | 16.3297 | 5.32E-05 | 1.26E-03 |
| PEX26 | ENSGALG00000019286 | peroxisomal biogenesis factor 26 | -0.31 | 4.9790 | 19.7337 | 8.90E-06 | 2.98E-04 |
| LMINA | - - - - - | ----- | -0.30 | 4.0567 | 8.2559 | 4.06E-03 | 3.28E-02 |
| KIF5C | ENSGALG00000012462 | kinesin family member 5C | -0.30 | 6.5978 | 13.5054 | 2.38E-04 | 4.12E-03 |
| TECPR1 | ENSGALG00000003507 | tectonin beta-propeller repeat containing 1 | -0.30 | 3.9104 | 7.9427 | 4.83E-03 | 3.74E-02 |
| CTSB | ENSGALG00000030016 | cathepsin B | -0.30 | 5.8936 | 14.0589 | 1.77E-04 | 3.24E-03 |
| SNAPIN | ENSGALG00000026796 | SNAP associated protein | -0.30 | 5.0482 | 23.0055 | 1.62E-06 | 7.03E-05 |
| EVC2 | ENSGALG00000015029 | EvC ciliary complex subunit 2 | -0.30 | 4.4756 | 13.2557 | 2.72E-04 | 4.59E-03 |

| | | | | | | | |
|---|---|---|---|---|---|---|---|
| MIOS | ENSGALG00000010699 | meiosis regulator for oocyte development | -0.30 | 5.9045 | 13.1562 | 2.87E-04 | 4.76E-03 |
| TCP11L1 | ENSGALG00000011688 | t-complex 11 like 1 | -0.30 | 5.3672 | 12.4812 | 4.11E-04 | 6.25E-03 |
| TMEM60 | ENSGALG00000008330 | transmembrane protein 60 | -0.30 | 4.3556 | 9.0775 | 2.59E-03 | 2.38E-02 |
| C24H11orf57 | - - - - - | ----- | -0.30 | 5.1565 | 18.8050 | 1.45E-05 | 4.37E-04 |
| TTPAL | ENSGALG00000004244 | alpha tocopherol transfer protein like | -0.30 | 5.1976 | 17.9440 | 2.27E-05 | 6.27E-04 |
| GALNT5 | - - - - - | polypeptide N-acetylgalactosaminyltransferase 5 | -0.30 | 3.8254 | 8.4217 | 3.71E-03 | 3.09E-02 |
| CDKL2 | ENSGALG00000010119 | cyclin dependent kinase like 2 | -0.30 | 3.8495 | 7.6630 | 5.64E-03 | 4.15E-02 |
| OSGIN2 | ENSGALG00000042840 | oxidative stress induced growth inhibitor family member 2 | -0.30 | 4.4740 | 8.5422 | 3.47E-03 | 2.93E-02 |
| LOC420294 | - - - - - | cylindromatosis-like | -0.30 | 4.6614 | 13.6099 | 2.25E-04 | 3.93E-03 |
| DNAJC12 | ENSGALG00000002769 | DnaJ heat shock protein family (Hsp40) member C12 | -0.30 | 4.8539 | 15.0186 | 1.06E-04 | 2.16E-03 |
| TRADD | ENSGALG00000003195 | TNFRSF1A associated via death domain | -0.30 | 4.5141 | 12.3736 | 4.35E-04 | 6.53E-03 |
| DNAJC5G | - - - - - | DnaJ heat shock protein family (Hsp40) member C5 gamma | -0.30 | 3.4616 | 7.3468 | 6.72E-03 | 4.70E-02 |
| AATK | ENSGALG00000006901 | apoptosis associated tyrosine kinase | -0.30 | 5.0542 | 10.5799 | 1.14E-03 | 1.32E-02 |
| PMVK | ENSGALG00000046656 | phosphomevalonate kinase | -0.30 | 4.8774 | 11.9800 | 5.38E-04 | 7.66E-03 |
| CTPS2 | ENSGALG00000016549 | CTP synthase 2 | -0.30 | 3.8226 | 10.6587 | 1.10E-03 | 1.28E-02 |
| ARMC1 | ENSGALG00000036567 | armadillo repeat containing 1 | -0.30 | 5.2941 | 9.7777 | 1.77E-03 | 1.83E-02 |
| PCMTD2 | ENSGALG00000005901 | protein-L-isoaspartate (D-aspartate) O-methyltransferase domain containing 2 | -0.29 | 5.2991 | 15.7821 | 7.11E-05 | 1.60E-03 |
| PLCD1 | ENSGALG00000005805 | phospholipase C delta 1 | -0.29 | 5.9543 | 9.1407 | 2.50E-03 | 2.32E-02 |
| KIAA1147 | - - - - - | ----- | -0.29 | 4.4351 | 10.6631 | 1.09E-03 | 1.28E-02 |
| IFNAR2 | ENSGALG00000041867 | interferon alpha and beta receptor subunit 2 | -0.29 | 5.4003 | 19.1793 | 1.19E-05 | 3.74E-04 |
| PLEKHA7 | ENSGALG00000029679 | pleckstrin homology domain containing A7 | -0.29 | 5.6426 | 8.6286 | 3.31E-03 | 2.84E-02 |
| FBXO25 | ENSGALG00000016359 | F-box protein 25 | -0.29 | 3.8647 | 8.4802 | 3.59E-03 | 3.01E-02 |
| FBXL8 | ENSGALG00000003201 | F-box and leucine rich repeat protein 8 | -0.29 | 3.6948 | 7.6510 | 5.67E-03 | 4.17E-02 |
| DAP | ENSGALG00000013002 | death associated protein | -0.29 | 5.8910 | 18.8222 | 1.43E-05 | 4.34E-04 |
| AFG1L | ENSGALG00000015303 | lactation elevated 1 | -0.29 | 4.1142 | 10.2978 | 1.33E-03 | 1.48E-02 |
| IRAK4 | ENSGALG00000009586 | interleukin 1 receptor associated kinase 4 | -0.29 | 3.6889 | 8.8778 | 2.89E-03 | 2.58E-02 |
| TRPC1 | ENSGALG00000002639 | transient receptor potential cation channel subfamily C member 1 | -0.29 | 5.7582 | 20.6940 | 5.39E-06 | 1.97E-04 |
| ITPR2 | ENSGALG00000014071 | inositol 1,4,5-trisphosphate receptor type 2 | -0.29 | 6.1582 | 7.2860 | 6.95E-03 | 4.80E-02 |
| APPL1 | ENSGALG00000005527 | adaptor protein, phosphotyrosine interacting with PH domain and leucine zipper 1 | -0.29 | 5.7678 | 11.5593 | 6.74E-04 | 9.05E-03 |
| TTC12 | ENSGALG00000007833 | tetratricopeptide repeat domain 12 | -0.29 | 4.5829 | 11.4304 | 7.23E-04 | 9.50E-03 |
| RECQL | ENSGALG00000013174 | RecQ like helicase | -0.29 | 5.1476 | 15.9709 | 6.43E-05 | 1.47E-03 |
| ELMO1 | ENSGALG00000035492 | engulfment and cell motility 1 | -0.29 | 4.3862 | 9.8680 | 1.68E-03 | 1.76E-02 |
| UNC5B | ENSGALG00000004569 | unc-5 netrin receptor B | -0.28 | 5.5500 | 7.9794 | 4.73E-03 | 3.68E-02 |
| NOX5 | ENSGALG00000008063 | NADPH oxidase 5 | -0.28 | 6.2767 | 8.2626 | 4.05E-03 | 3.27E-02 |
| PTGES3L | ENSGALG00000038129 | prostaglandin E synthase 3 (cytosolic)-like | -0.28 | 4.1798 | 9.8403 | 1.71E-03 | 1.78E-02 |
| LOC425117 | - - - - - | uncharacterized LOC425117 | -0.28 | 6.4418 | 15.5016 | 8.24E-05 | 1.79E-03 |
| PLK3 | ENSGALG00000010129 | polo like kinase 3 | -0.28 | 4.5311 | 10.0570 | 1.52E-03 | 1.63E-02 |
| EPG5 | - - - - - | ectopic P-granules autophagy protein 5 homolog | -0.28 | 5.2362 | 9.3471 | 2.23E-03 | 2.17E-02 |
| SLC7A6 | - - - - - | solute carrier family 7 member 6 | -0.28 | 5.1823 | 11.3300 | 7.63E-04 | 9.85E-03 |
| TMEM170A | ENSGALG00000002861 | transmembrane protein 170A | -0.28 | 4.7436 | 10.7258 | 1.06E-03 | 1.25E-02 |
| BBS5 | ENSGALG00000009846 | Bardet-Biedl syndrome 5 | -0.28 | 5.0702 | 10.4139 | 1.25E-03 | 1.41E-02 |
| TMX4 | ENSGALG00000028587 | thioredoxin related transmembrane protein 4 | -0.28 | 5.9879 | 12.8237 | 3.42E-04 | 5.44E-03 |
| CEP350 | ENSGALG00000003955 | centrosomal protein 350 | -0.28 | 6.2836 | 13.2324 | 2.75E-04 | 4.61E-03 |
| FANCG | ENSGALG00000002009 | Fanconi anemia complementation group G | -0.28 | 5.2517 | 7.8378 | 5.12E-03 | 3.88E-02 |

| | | | | | | | |
|---|---|---|---|---|---|---|---|
| RCAN1 | ENSGALG00000016014 | regulator of calcineurin 1 | -0.28 | 4.3664 | 7.6844 | 5.57E-03 | 4.11E-02 |
| C10H15orf40 | - - - - - | C15ORF40 homolog | -0.28 | 4.3805 | 8.1766 | 4.24E-03 | 3.39E-02 |
| TIAM1 | ENSGALG00000015842 | T-cell lymphoma invasion and metastasis 1 | -0.28 | 6.4095 | 9.7036 | 1.84E-03 | 1.89E-02 |
| BLOC1S3 | - - - - - | iogenesis of lysosomal organelles complex-1, subunit 3 | -0.28 | 5.9283 | 8.5920 | 3.38E-03 | 2.88E-02 |
| HIST1H2B5L | ENSGALG00000054287 | histone cluster 1,  H2B-V-like | -0.28 | 6.3293 | 17.4625 | 2.93E-05 | 7.68E-04 |
| DFNA5 | ENSGALG00000010997 | DFNA5, deafness associated tumor suppressor | -0.28 | 5.7902 | 8.6033 | 3.36E-03 | 2.87E-02 |
| ZBTB38 | ENSGALG00000036543 | zinc finger and BTB domain containing 38 | -0.28 | 4.5363 | 7.7620 | 5.34E-03 | 3.99E-02 |
| VPS37A | ENSGALG00000013646 | VPS37A, ESCRT-I subunit | -0.27 | 4.1444 | 9.3753 | 2.20E-03 | 2.15E-02 |
| NAT9 | ENSGALG00000007723 | N-acetyltransferase 9 (putative) | -0.27 | 4.8714 | 14.0590 | 1.77E-04 | 3.24E-03 |
| AVL9 | ENSGALG00000041568 | AVL9 cell migration associated | -0.27 | 4.8185 | 8.8723 | 2.90E-03 | 2.58E-02 |
| SCARF2 | ENSGALG00000034560 | scavenger receptor class F, member 2 | -0.27 | 7.0539 | 15.0618 | 1.04E-04 | 2.13E-03 |
| YAE1D1 | ENSGALG00000043636 | Yae1 domain containing 1 | -0.27 | 3.9226 | 9.3467 | 2.23E-03 | 2.17E-02 |
| TCEA2 | ENSGALG00000005976 | transcription elongation factor A2 | -0.27 | 6.0340 | 18.1206 | 2.07E-05 | 5.78E-04 |
| BAZ2B | ENSGALG00000012579 | bromodomain adjacent to zinc finger domain 2B | -0.27 | 7.1472 | 12.3155 | 4.49E-04 | 6.69E-03 |
| LOC107049545 | - - - - - | cytochrome c oxidase subunit 5B | -0.27 | 6.0338 | 10.4897 | 1.20E-03 | 1.37E-02 |
| INTS7 | ENSGALG00000009843 | integrator complex subunit 7 | -0.27 | 6.0375 | 11.8978 | 5.62E-04 | 7.89E-03 |
| PRKAB1 | ENSGALG00000007373 | protein kinase AMP-activated non-catalytic subunit beta 1 | -0.27 | 6.2090 | 18.2138 | 1.97E-05 | 5.58E-04 |
| ALAS1 | ENSGALG00000003948 | 5'-aminolevulinate synthase 1 | -0.27 | 5.6723 | 7.4433 | 6.37E-03 | 4.54E-02 |
| SPOCK3 | ENSGALG00000009568 | SPARC/osteonectin, cwcv and kazal like domains proteoglycan 3 | -0.27 | 6.4278 | 12.8123 | 3.44E-04 | 5.46E-03 |
| KRIT1 | ENSGALG00000042651 | KRIT1, ankyrin repeat containing | -0.27 | 5.7797 | 22.7633 | 1.83E-06 | 7.83E-05 |
| MERTK | ENSGALG00000008257 | MER proto-oncogene, tyrosine kinase | -0.27 | 5.1761 | 13.2459 | 2.73E-04 | 4.59E-03 |
| ROBO1 | ENSGALG00000015511 | roundabout guidance receptor 1 | -0.27 | 6.8791 | 14.0632 | 1.77E-04 | 3.24E-03 |
| TK2 | ENSGALG00000034216 | thymidine kinase 2, mitochondrial | -0.27 | 7.0559 | 10.3189 | 1.32E-03 | 1.46E-02 |
| PRR5L | ENSGALG00000007920 | proline rich 5 like | -0.27 | 5.2611 | 10.5825 | 1.14E-03 | 1.32E-02 |
| USP30 | ENSGALG00000040265 | ubiquitin specific peptidase 30 | -0.27 | 5.3636 | 14.4442 | 1.44E-04 | 2.76E-03 |
| MIEN1 | ENSGALG00000026328 | migration and invasion enhancer 1 | -0.27 | 5.1399 | 14.4551 | 1.44E-04 | 2.75E-03 |
| HARS2 | ENSGALG00000029346 | histidyl-tRNA synthetase 2, mitochondrial | -0.27 | 4.2768 | 7.6377 | 5.72E-03 | 4.19E-02 |
| ABCG2 | ENSGALG00000030677 | ATP binding cassette subfamily G member 2 (Junior blood group) | -0.27 | 3.5482 | 7.2874 | 6.94E-03 | 4.80E-02 |
| CCDC173 | ENSGALG00000031069 | coiled-coil domain containing 173 | -0.27 | 6.4063 | 12.4868 | 4.10E-04 | 6.24E-03 |
| GLUD1 | - - - - - | ----- | -0.27 | 5.7915 | 21.7123 | 3.17E-06 | 1.25E-04 |
| LOC112530288 | - - - - - | proline-rich receptor-like protein kinase PERK2 | -0.27 | 4.4495 | 7.2854 | 6.95E-03 | 4.80E-02 |
| FRMPD4 | ENSGALG00000033541 | FERM and PDZ domain containing 4 | -0.26 | 3.6611 | 7.7232 | 5.45E-03 | 4.05E-02 |
| SPPL2B | - - - - - | ----- | -0.26 | 6.6749 | 8.0868 | 4.46E-03 | 3.52E-02 |
| GPATCH1 | ENSGALG00000004839 | G-patch domain containing 1 | -0.26 | 5.5999 | 13.9799 | 1.85E-04 | 3.35E-03 |
| RHPN2 | ENSGALG00000004814 | rhophilin Rho GTPase binding protein 2 | -0.26 | 4.5231 | 8.8653 | 2.91E-03 | 2.58E-02 |
| RFTN2 | ENSGALG00000036471 | raftlin family member 2 | -0.26 | 6.2042 | 9.4118 | 2.16E-03 | 2.12E-02 |
| MFN1 | ENSGALG00000041873 | mitofusin 1 | -0.26 | 7.0836 | 18.1637 | 2.03E-05 | 5.69E-04 |
| CELSR3 | ENSGALG00000036763 | cadherin EGF LAG seven-pass G-type receptor 3 | -0.26 | 6.5574 | 12.2927 | 4.55E-04 | 6.74E-03 |
| TRAF3IP2 | ENSGALG00000015026 | TRAF3 interacting protein 2 | -0.26 | 4.9824 | 9.2310 | 2.38E-03 | 2.26E-02 |
| RNF217 | ENSGALG00000013651 | ring finger protein 217 | -0.26 | 6.3671 | 12.0575 | 5.16E-04 | 7.41E-03 |
| NANP | ENSGALG00000047977 | N-acetylneuraminic acid phosphatase | -0.26 | 5.2581 | 12.6148 | 3.83E-04 | 5.91E-03 |
| SELENOS | ENSGALG00000007162 | selenoprotein S | -0.26 | 5.8177 | 10.8588 | 9.83E-04 | 1.19E-02 |
| PLA2G10 | ENSGALG00000021314 | phospholipase A2 group X | -0.26 | 4.8347 | 9.6913 | 1.85E-03 | 1.90E-02 |
| SYNE1 | ENSGALG00000013505 | spectrin repeat containing, nuclear envelope 1 | -0.26 | 5.7772 | 9.5404 | 2.01E-03 | 2.02E-02 |

| | | | | | | | |
|---|---|---|---|---|---|---|---|
| XPO1p | ENSGALG00000038549 | exportin 1,  transcribed pseudogene | -0.26 | 3.7298 | 7.3952 | 6.54E-03 | 4.62E-02 |
| RREB1 | ENSGALG00000012799 | ras responsive element binding protein 1 | -0.26 | 5.3452 | 7.7213 | 5.46E-03 | 4.05E-02 |
| KIAA0930 | ENSGALG00000014226 | KIAA0930 | -0.26 | 5.3241 | 11.7579 | 6.06E-04 | 8.34E-03 |
| RNF149 | ENSGALG00000016779 | ring finger protein 149 | -0.26 | 5.8404 | 15.7769 | 7.13E-05 | 1.60E-03 |
| TBL3 | ENSGALG00000005558 | transducin beta like 3 | -0.26 | 7.2349 | 18.5439 | 1.66E-05 | 4.88E-04 |
| FAM83H | ENSGALG00000033118 | family with sequence similarity 83 member H | -0.26 | 4.1850 | 7.7599 | 5.34E-03 | 4.00E-02 |
| CDIP1 | ENSGALG00000036502 | cell death-inducing p53 target 1 | -0.26 | 5.0583 | 10.3051 | 1.33E-03 | 1.47E-02 |
| MSRB1 | ENSGALG00000005414 | methionine sulfoxide reductase B1 | -0.26 | 4.6937 | 7.4292 | 6.42E-03 | 4.57E-02 |
| ZNF512B | ENSGALG00000006013 | zinc finger protein 512B | -0.25 | 6.5257 | 25.4157 | 4.62E-07 | 2.51E-05 |
| VPS37B | ENSGALG00000003743 | VPS37B, ESCRT-I subunit | -0.25 | 6.3651 | 18.2178 | 1.97E-05 | 5.58E-04 |
| NOCT | ENSGALG00000032957 | nocturnin | -0.25 | 4.3777 | 9.4509 | 2.11E-03 | 2.09E-02 |
| ICK | ENSGALG00000016321 | intestinal cell kinase | -0.25 | 6.0281 | 15.1545 | 9.91E-05 | 2.05E-03 |
| MAML1 | - - - - - | mastermind like transcriptional coactivator 1 | -0.25 | 5.8221 | 10.0953 | 1.49E-03 | 1.61E-02 |
| LOC101748694 | - - - - - | dentin sialophosphoprotein-like | -0.25 | 5.6915 | 11.7003 | 6.25E-04 | 8.54E-03 |
| SEMA5B | ENSGALG00000011686 | semaphorin 5B | -0.25 | 8.0429 | 14.9920 | 1.08E-04 | 2.17E-03 |
| AGA | ENSGALG00000010716 | aspartylglucosaminidase | -0.25 | 5.7214 | 12.8759 | 3.33E-04 | 5.32E-03 |
| L2HGDH | ENSGALG00000012282 | L-2-hydroxyglutarate dehydrogenase | -0.25 | 4.8378 | 9.4606 | 2.10E-03 | 2.08E-02 |
| MCF2L | ENSGALG00000016834 | MCF.2 cell line derived transforming sequence like | -0.25 | 5.1371 | 8.7998 | 3.01E-03 | 2.65E-02 |
| ISCU | ENSGALG00000004869 | iron-sulfur cluster assembly enzyme | -0.25 | 6.8065 | 15.7488 | 7.23E-05 | 1.62E-03 |
| TMEM38A | ENSGALG00000003778 | transmembrane protein 38A | -0.25 | 5.3101 | 12.3612 | 4.38E-04 | 6.56E-03 |
| LETM2 | ENSGALG00000036049 | leucine zipper and EF-hand containing transmembrane protein 2 | -0.25 | 4.5601 | 11.0503 | 8.87E-04 | 1.10E-02 |
| VCPKMT | ENSGALG00000012269 | valosin containing protein lysine methyltransferase | -0.25 | 4.3761 | 7.8801 | 5.00E-03 | 3.83E-02 |
| ALMS1 | ENSGALG00000044620 | ALMS1, centrosome and basal body associated protein | -0.25 | 6.8736 | 12.1267 | 4.97E-04 | 7.23E-03 |
| TMEM150C | ENSGALG00000011175 | transmembrane protein 150C | -0.25 | 4.9603 | 8.1614 | 4.28E-03 | 3.40E-02 |
| PRMT8 | ENSGALG00000013272 | protein arginine methyltransferase 1 | -0.25 | 4.8158 | 7.7737 | 5.30E-03 | 3.98E-02 |
| NCKIPSD | ENSGALG00000005713 | NCK interacting protein with SH3 domain | -0.25 | 5.5631 | 8.3025 | 3.96E-03 | 3.22E-02 |
| AXIN2 | ENSGALG00000039200 | axin 2 | -0.25 | 5.3737 | 7.2716 | 7.01E-03 | 4.83E-02 |
| FAAH2 | ENSGALG00000028084 | fatty acid amide hydrolase 2 | -0.24 | 5.5503 | 14.7205 | 1.25E-04 | 2.45E-03 |
| C4HXorf56 | - - - - - | ----- | -0.24 | 6.1445 | 12.9782 | 3.15E-04 | 5.11E-03 |
| LDLRAD3 | ENSGALG00000038913 | low density lipoprotein receptor class A domain containing 3 | -0.24 | 7.1575 | 7.2098 | 7.25E-03 | 4.94E-02 |
| SDC2 | ENSGALG00000031424 | syndecan 2 | -0.24 | 6.4457 | 10.7110 | 1.07E-03 | 1.26E-02 |
| INTS6L | ENSGALG00000006157 | integrator complex subunit 6 like | -0.24 | 6.1823 | 13.4956 | 2.39E-04 | 4.14E-03 |
| PTDSS1 | ENSGALG00000030185 | phosphatidylserine synthase 1 | -0.24 | 6.1719 | 16.0357 | 6.22E-05 | 1.43E-03 |
| ZDHHC20 | ENSGALG00000017126 | zinc finger DHHC-type containing 20 | -0.24 | 6.6535 | 19.0978 | 1.24E-05 | 3.84E-04 |
| CCBL1 | - - - - - | ----- | -0.24 | 4.4544 | 9.3677 | 2.21E-03 | 2.15E-02 |
| SLC39A11 | ENSGALG00000004413 | solute carrier family 39 member 11 | -0.24 | 5.0172 | 11.1726 | 8.30E-04 | 1.05E-02 |
| PLEKHG3 | - - - - - | pleckstrin homology and RhoGEF domain containing G3 | -0.24 | 5.4747 | 7.2357 | 7.15E-03 | 4.89E-02 |
| SIN3A | ENSGALG00000001644 | SIN3 transcription regulator family member A | -0.24 | 7.2455 | 16.3272 | 5.33E-05 | 1.26E-03 |
| TMEM181 | ENSGALG00000013737 | transmembrane protein 181 | -0.24 | 5.1592 | 7.6604 | 5.64E-03 | 4.16E-02 |
| NCLN | ENSGALG00000013369 | nicalin | -0.24 | 6.7790 | 16.7502 | 4.26E-05 | 1.05E-03 |
| RLIM | ENSGALG00000007750 | ring finger protein, LIM domain interacting | -0.24 | 6.9172 | 16.9535 | 3.83E-05 | 9.61E-04 |
| MAN1B1 | ENSGALG00000037375 | mannosidase alpha class 1B member 1 | -0.24 | 5.8033 | 8.6848 | 3.21E-03 | 2.77E-02 |
| MVK | ENSGALG00000013848 | mevalonate kinase | -0.24 | 5.8181 | 12.4298 | 4.23E-04 | 6.38E-03 |
| ATF7IP | ENSGALG00000011814 | activating transcription factor 7 interacting protein | -0.24 | 6.6857 | 11.9591 | 5.44E-04 | 7.73E-03 |

| | | | | | | | |
|---|---|---|---|---|---|---|---|
| KIF13B | ENSGALG00000016639 | kinesin family member 13B | -0.24 | 5.4384 | 9.1078 | 2.55E-03 | 2.36E-02 |
| FAM20B | ENSGALG00000004227 | FAM20B, glycosaminoglycan xylosylkinase | -0.24 | 5.5172 | 13.9024 | 1.93E-04 | 3.47E-03 |
| GTF2F2 | ENSGALG00000016974 | general transcription factor IIF subunit 2 | -0.24 | 7.2035 | 15.2815 | 9.26E-05 | 1.95E-03 |
| MPHOSPH9 | - - - - - | M-phase phosphoprotein 9 | -0.24 | 5.2130 | 9.9175 | 1.64E-03 | 1.73E-02 |
| CNOT6L | ENSGALG00000010333 | CCR4-NOT transcription complex subunit 6 like | -0.24 | 5.3242 | 9.7023 | 1.84E-03 | 1.89E-02 |
| PIKFYVE | ENSGALG00000008842 | phosphoinositide kinase, FYVE-type zinc finger containing | -0.24 | 6.3719 | 9.8933 | 1.66E-03 | 1.74E-02 |
| TTC17 | ENSGALG00000039558 | tetratricopeptide repeat domain 17 | -0.23 | 5.8890 | 12.0218 | 5.26E-04 | 7.53E-03 |
| RAB11FIP5 | ENSGALG00000047393 | RAB11 family interacting protein 5 | -0.23 | 4.9828 | 8.0539 | 4.54E-03 | 3.56E-02 |
| TNFRSF1A | ENSGALG00000039461 | TNF receptor superfamily member 1A | -0.23 | 4.5355 | 7.5509 | 6.00E-03 | 4.33E-02 |
| PARP12 | ENSGALG00000046187 | poly(ADP-ribose) polymerase family member 12 | -0.23 | 4.7084 | 8.6752 | 3.23E-03 | 2.78E-02 |
| C1GALT1C1 | ENSGALG00000008513 | C1GALT1 specific chaperone 1 | -0.23 | 4.3078 | 7.2174 | 7.22E-03 | 4.93E-02 |
| PROS1 | ENSGALG00000015409 | protein S (alpha) | -0.23 | 6.8792 | 16.1589 | 5.82E-05 | 1.35E-03 |
| CBL | ENSGALG00000029077 | Cbl proto-oncogene | -0.23 | 6.4465 | 14.9845 | 1.08E-04 | 2.18E-03 |
| RBM15 | ENSGALG00000000401 | RNA binding motif protein 15 | -0.23 | 6.6983 | 8.8649 | 2.91E-03 | 2.58E-02 |
| B4GALT4 | ENSGALG00000015069 | beta-1,4-galactosyltransferase 4 | -0.23 | 5.1586 | 10.4591 | 1.22E-03 | 1.38E-02 |
| GPATCH2 | ENSGALG00000009627 | G-patch domain containing 2 | -0.23 | 5.5154 | 11.5097 | 6.92E-04 | 9.20E-03 |
| SPINT1 | ENSGALG00000008469 | serine peptidase inhibitor, Kunitz type 1 | -0.23 | 5.8626 | 9.3622 | 2.22E-03 | 2.16E-02 |
| LNPK | ENSGALG00000037944 | lunapark, ER junction formation factor | -0.23 | 5.5839 | 13.2438 | 2.73E-04 | 4.59E-03 |
| DHX35 | ENSGALG00000003658 | DEAH-box helicase 35 | -0.23 | 4.6799 | 9.8605 | 1.69E-03 | 1.77E-02 |
| GCLC | ENSGALG00000016313 | glutamate-cysteine ligase catalytic subunit | -0.23 | 5.3399 | 7.3141 | 6.84E-03 | 4.75E-02 |
| NBEAL2 | ENSGALG00000039120 | neurobeachin like 2 | -0.23 | 5.7322 | 11.6181 | 6.53E-04 | 8.83E-03 |
| LOC101749469 | - - - - - | uncharacterized LOC101749469 | -0.23 | 7.5329 | 9.2440 | 2.36E-03 | 2.25E-02 |
| TRMT61A | ENSGALG00000011522 | tRNA methyltransferase 61A | -0.23 | 5.7684 | 9.5196 | 2.03E-03 | 2.04E-02 |
| BMP7 | ENSGALG00000007668 | bone morphogenetic protein 7 | -0.23 | 5.8962 | 10.0461 | 1.53E-03 | 1.64E-02 |
| IDI1 | - - - - - | ----- | -0.23 | 6.1165 | 7.4677 | 6.28E-03 | 4.50E-02 |
| MDM2 | ENSGALG00000009942 | MDM2 proto-oncogene | -0.23 | 6.4703 | 8.8718 | 2.90E-03 | 2.58E-02 |
| USP20 | ENSGALG00000041104 | ubiquitin specific peptidase 20 | -0.22 | 6.0375 | 11.4865 | 7.01E-04 | 9.27E-03 |
| DOCK9 | ENSGALG00000016883 | dedicator of cytokinesis 9 | -0.22 | 5.0869 | 8.0500 | 4.55E-03 | 3.57E-02 |
| MGARP | ENSGALG00000046580 | mitochondria localized glutamic acid rich protein | -0.22 | 5.5878 | 8.1872 | 4.22E-03 | 3.38E-02 |
| ZBTB26 | ENSGALG00000042762 | zinc finger and BTB domain containing 26 | -0.22 | 5.7410 | 8.7884 | 3.03E-03 | 2.66E-02 |
| CDC42EP1 | ENSGALG00000038556 | CDC42 effector protein 1 | -0.22 | 7.0204 | 13.1934 | 2.81E-04 | 4.69E-03 |
| CAMSAP1 | ENSGALG00000001692 | calmodulin regulated spectrin associated protein 1 | -0.22 | 6.4631 | 10.3679 | 1.28E-03 | 1.44E-02 |
| RD3 | ENSGALG00000009861 | retinal degeneration 3 | -0.22 | 4.8471 | 9.1693 | 2.46E-03 | 2.30E-02 |
| GCC1 | ENSGALG00000033149 | GRIP and coiled-coil domain containing 1 | -0.22 | 4.9203 | 9.7960 | 1.75E-03 | 1.82E-02 |
| PSKH1 | ENSGALG00000001647 | protein serine kinase H1 | -0.22 | 5.6429 | 9.6597 | 1.88E-03 | 1.93E-02 |
| FOXN2 | ENSGALG00000008942 | forkhead box N2 | -0.22 | 5.7749 | 7.9854 | 4.72E-03 | 3.67E-02 |
| N6AMT1 | ENSGALG00000015802 | N-6 adenine-specific DNA methyltransferase 1 (putative) | -0.22 | 4.9812 | 7.8615 | 5.05E-03 | 3.84E-02 |
| ZBTB47 | ENSGALG00000005287 | zinc finger and BTB domain containing 47 | -0.22 | 5.6995 | 8.8203 | 2.98E-03 | 2.63E-02 |
| BCS1L | ENSGALG00000011386 | BCS1 homolog, ubiquinol-cytochrome c reductase complex chaperone | -0.22 | 4.8420 | 7.4329 | 6.40E-03 | 4.56E-02 |
| ANO5 | ENSGALG00000003748 | anoctamin 5 | -0.22 | 6.5501 | 14.1707 | 1.67E-04 | 3.09E-03 |
| YOD1 | ENSGALG00000037597 | YOD1 deubiquitinase | -0.22 | 6.1802 | 10.7439 | 1.05E-03 | 1.24E-02 |
| MAPKAPK5 | ENSGALG00000004743 | mitogen-activated protein kinase-activated protein kinase 5 | -0.22 | 6.2773 | 12.9882 | 3.13E-04 | 5.11E-03 |
| LOC420992 | - - - - - | uncharacterized LOC420992 | -0.22 | 5.9674 | 11.2504 | 7.96E-04 | 1.02E-02 |
| ZC2HC1A | ENSGALG00000035932 | zinc finger, C2HC-type containing 1A | -0.22 | 5.7628 | 12.3527 | 4.40E-04 | 6.59E-03 |

| | | | | | | | |
|---|---|---|---|---|---|---|---|
| WDR6 | ENSGALG00000006936 | WD repeat domain 6 | -0.22 | 5.0309 | 8.1713 | 4.26E-03 | 3.39E-02 |
| TANC1 | ENSGALG00000012570 | tetratricopeptide repeat, ankyrin repeat and coiled-coil containing 1 | -0.22 | 6.2596 | 9.6706 | 1.87E-03 | 1.92E-02 |
| CTTNBP2 | ENSGALG00000009134 | cortactin binding protein 2 | -0.22 | 5.0996 | 7.3334 | 6.77E-03 | 4.72E-02 |
| INO80C | ENSGALG00000051335 | INO80 complex subunit C | -0.22 | 6.0060 | 10.2133 | 1.39E-03 | 1.53E-02 |
| RAB11FIP1 | ENSGALG00000003129 | RAB11 family interacting protein 1 | -0.22 | 5.5137 | 9.9277 | 1.63E-03 | 1.72E-02 |
| PDIA5 | ENSGALG00000011689 | protein disulfide isomerase family A member 5 | -0.22 | 6.3500 | 12.6882 | 3.68E-04 | 5.74E-03 |
| PGM3 | ENSGALG00000015854 | phosphoglucomutase 3 | -0.22 | 5.3264 | 8.5087 | 3.53E-03 | 2.97E-02 |
| UBE3B | ENSGALG00000013851 | ubiquitin protein ligase E3B | -0.21 | 6.0024 | 10.1643 | 1.43E-03 | 1.56E-02 |
| UGGT1 | ENSGALG00000002141 | UDP-glucose glycoprotein glucosyltransferase 1 | -0.21 | 6.9309 | 8.2680 | 4.03E-03 | 3.27E-02 |
| SYNE3 | ENSGALG00000011076 | spectrin repeat containing nuclear envelope family member 3 | -0.21 | 6.2863 | 9.3443 | 2.24E-03 | 2.17E-02 |
| GNS | ENSGALG00000009856 | glucosamine (N-acetyl)-6-sulfatase | -0.21 | 6.6696 | 15.4242 | 8.59E-05 | 1.84E-03 |
| NPLOC4 | ENSGALG00000004583 | NPL4 homolog, ubiquitin recognition factor | -0.21 | 6.7924 | 9.0485 | 2.63E-03 | 2.41E-02 |
| GOLGA3 | ENSGALG00000002158 | golgin A3 | -0.21 | 5.6928 | 9.2127 | 2.40E-03 | 2.27E-02 |
| CAMK1 | ENSGALG00000029912 | calcium/calmodulin dependent protein kinase I | -0.21 | 5.2561 | 9.0422 | 2.64E-03 | 2.41E-02 |
| RFC2 | ENSGALG00000029449 | replication factor C subunit 2 | -0.21 | 6.2614 | 9.4749 | 2.08E-03 | 2.07E-02 |
| CCDC92 | ENSGALG00000003146 | coiled-coil domain containing 92 | -0.21 | 5.5542 | 7.5934 | 5.86E-03 | 4.27E-02 |
| PANX2 | ENSGALG00000000003 | pannexin 2 | -0.21 | 5.6368 | 7.7268 | 5.44E-03 | 4.05E-02 |
| MDM4 | ENSGALG00000000636 | MDM4, p53 regulator | -0.21 | 7.4226 | 10.7888 | 1.02E-03 | 1.22E-02 |
| LGALSL | ENSGALG00000027096 | galectin like | -0.21 | 4.6639 | 8.4149 | 3.72E-03 | 3.10E-02 |
| TTYH3 | ENSGALG00000004310 | tweety family member 3 | -0.21 | 8.4824 | 11.8335 | 5.82E-04 | 8.11E-03 |
| TMEM248 | ENSGALG00000033954 | transmembrane protein 248 | -0.21 | 6.5879 | 11.9961 | 5.33E-04 | 7.61E-03 |
| OSBP2 | ENSGALG00000007758 | oxysterol binding protein 2 | -0.21 | 5.1127 | 8.3006 | 3.96E-03 | 3.22E-02 |
| CLIP2 | ENSGALG00000030450 | CAP-GLY domain containing linker protein 2 | -0.21 | 7.2871 | 9.3694 | 2.21E-03 | 2.15E-02 |
| LOC776273 | ENSGALG00000036085 | 1-phosphatidylinositol-4,5-bisphosphate phosphodiesterase gamma-1-like | -0.21 | 7.3000 | 15.3073 | 9.14E-05 | 1.93E-03 |
| AFG3L2 | ENSGALG00000013853 | AFG3 like matrix AAA peptidase subunit 2 | -0.21 | 6.8375 | 17.6423 | 2.67E-05 | 7.15E-04 |
| SMYD5 | ENSGALG00000022920 | SMYD family member 5 | -0.21 | 5.0026 | 7.7674 | 5.32E-03 | 3.99E-02 |
| CORO7 | ENSGALG00000038325 | coronin 7 | -0.21 | 5.1163 | 7.3497 | 6.71E-03 | 4.70E-02 |
| ARL6IP4 | ENSGALG00000003682 | ADP ribosylation factor like GTPase 6 interacting protein 4 | -0.21 | 5.8407 | 13.8573 | 1.97E-04 | 3.54E-03 |
| COG2 | ENSGALG00000011114 | component of oligomeric golgi complex 2 | -0.21 | 5.2859 | 11.5397 | 6.81E-04 | 9.10E-03 |
| P3H3 | ENSGALG00000014490 | prolyl 3-hydroxylase 3 | -0.21 | 5.4666 | 7.4145 | 6.47E-03 | 4.60E-02 |
| TUBGCP6 | ENSGALG00000032156 | tubulin gamma complex associated protein 6 | -0.21 | 5.0536 | 7.8279 | 5.14E-03 | 3.89E-02 |
| GZF1 | ENSGALG00000053640 | GDNF inducible zinc finger protein 1 | -0.20 | 5.6733 | 7.8766 | 5.01E-03 | 3.83E-02 |
| SIN3B | ENSGALG00000003771 | SIN3 transcription regulator family member B | -0.20 | 6.1070 | 8.8556 | 2.92E-03 | 2.59E-02 |
| SUCLG2 | ENSGALG00000007652 | succinate-CoA ligase GDP-forming beta subunit | -0.20 | 6.9411 | 8.3994 | 3.75E-03 | 3.11E-02 |
| YIPF3 | ENSGALG00000041037 | Yip1 domain family member 3 | -0.20 | 5.9614 | 13.1601 | 2.86E-04 | 4.76E-03 |
| CHCHD5 | ENSGALG00000030457 | coiled-coil-helix-coiled-coil-helix domain containing 5 | -0.20 | 6.4152 | 10.4930 | 1.20E-03 | 1.37E-02 |
| ASB6 | ENSGALG00000004378 | ankyrin repeat and SOCS box containing 6 | -0.20 | 5.5897 | 7.6804 | 5.58E-03 | 4.12E-02 |
| SLC25A24 | ENSGALG00000001938 | solute carrier family 25 member 24 | -0.20 | 5.3139 | 8.7493 | 3.10E-03 | 2.70E-02 |
| FKBP15 | ENSGALG00000008838 | FK506 binding protein 15 | -0.20 | 6.2928 | 13.0632 | 3.01E-04 | 4.94E-03 |
| PISD | ENSGALG00000006872 | phosphatidylserine decarboxylase | -0.20 | 5.9242 | 7.8356 | 5.12E-03 | 3.88E-02 |
| POLH | ENSGALG00000010317 | DNA polymerase eta | -0.20 | 5.3014 | 8.8707 | 2.90E-03 | 2.58E-02 |
| WDR26 | ENSGALG00000043081 | WD repeat domain 26 | -0.20 | 6.7943 | 15.2557 | 9.39E-05 | 1.97E-03 |
| HEY1 | ENSGALG00000035031 | hes related family bHLH transcription factor with YRPW motif 1 | -0.20 | 6.1767 | 7.7795 | 5.28E-03 | 3.97E-02 |
| CENPI | ENSGALG00000005038 | centromere protein I | -0.20 | 5.9279 | 10.9143 | 9.54E-04 | 1.16E-02 |

| | | | | | | | |
|---|---|---|---|---|---|---|---|
| ZBTB17 | ENSGALG00000035352 | zinc finger and BTB domain containing 17 | -0.20 | 5.5238 | 9.9429 | 1.61E-03 | 1.71E-02 |
| SYT11 | ENSGALG00000014646 | synaptotagmin 11 | -0.20 | 7.7367 | 11.1444 | 8.43E-04 | 1.06E-02 |
| E2F4 | ENSGALG00000001722 | E2F transcription factor 4 | -0.20 | 6.4078 | 11.4987 | 6.96E-04 | 9.23E-03 |
| ACSL1 | ENSGALG00000010628 | acyl-CoA synthetase long-chain family member 1 | -0.20 | 5.9203 | 7.6696 | 5.62E-03 | 4.14E-02 |
| DENND4A | ENSGALG00000041403 | DENN domain containing 4A | -0.20 | 6.5107 | 8.1386 | 4.33E-03 | 3.44E-02 |
| PLOD2 | ENSGALG00000006783 | procollagen-lysine,2-oxoglutarate 5-dioxygenase 2 | -0.20 | 7.5922 | 11.0846 | 8.70E-04 | 1.08E-02 |
| BOD1L1 | ENSGALG00000026470 | biorientation of chromosomes in cell division 1 like 1 | -0.20 | 6.8387 | 7.8098 | 5.20E-03 | 3.92E-02 |
| PLEKHO1 | ENSGALG00000037540 | pleckstrin homology domain containing O1 | -0.20 | 6.1886 | 8.5705 | 3.42E-03 | 2.90E-02 |
| DIS3L | ENSGALG00000007643 | DIS3 like exosome 3'-5' exoribonuclease | -0.20 | 4.9449 | 7.2225 | 7.20E-03 | 4.92E-02 |
| FAM160B1 | ENSGALG00000031404 | family with sequence similarity 160 member B1 | -0.20 | 6.0890 | 11.9071 | 5.59E-04 | 7.88E-03 |
| EMC3 | ENSGALG00000003738 | ER membrane protein complex subunit 3 | -0.20 | 5.4547 | 10.5854 | 1.14E-03 | 1.32E-02 |
| CTSD | ENSGALG00000006613 | cathepsin D | -0.20 | 6.3516 | 8.9783 | 2.73E-03 | 2.47E-02 |
| C3H1ORF198 | - - - - - | ----- | -0.20 | 7.0214 | 13.9237 | 1.90E-04 | 3.44E-03 |
| IQCE | - - - - - | IQ motif containing E | -0.19 | 6.7900 | 10.2704 | 1.35E-03 | 1.49E-02 |
| VPS4B | ENSGALG00000012879 | vacuolar protein sorting 4 homolog B | -0.19 | 5.9254 | 8.6233 | 3.32E-03 | 2.84E-02 |
| ZBED1 | ENSGALG00000016681 | dehydrogenase/reductase X-linked | -0.19 | 4.7626 | 7.2235 | 7.20E-03 | 4.92E-02 |
| SNAPC4 | ENSGALG00000044726 | small nuclear RNA activating complex polypeptide 4 | -0.19 | 5.5412 | 7.3927 | 6.55E-03 | 4.62E-02 |
| PNN | ENSGALG00000010167 | pinin, desmosome associated protein | -0.19 | 8.0080 | 10.1819 | 1.42E-03 | 1.55E-02 |
| PHACTR2 | ENSGALG00000013768 | phosphatase and actin regulator 2 | -0.19 | 6.3553 | 8.9431 | 2.79E-03 | 2.51E-02 |
| RIT1 | ENSGALG00000014647 | Ras like without CAAX 1 | -0.19 | 7.7333 | 8.7888 | 3.03E-03 | 2.66E-02 |
| SCFD2 | ENSGALG00000039168 | sec1 family domain containing 2 | -0.19 | 5.9459 | 7.9246 | 4.88E-03 | 3.76E-02 |
| GPATCH4 | ENSGALG00000048365 | G-patch domain containing 4 | -0.19 | 5.9615 | 7.2029 | 7.28E-03 | 4.96E-02 |
| RNF25 | ENSGALG00000011375 | ring finger protein 25 | -0.19 | 5.6819 | 9.4637 | 2.10E-03 | 2.08E-02 |
| CASD1 | ENSGALG00000009653 | CAS1 domain containing 1 | -0.19 | 5.6799 | 11.0571 | 8.83E-04 | 1.09E-02 |
| WASHC4 | ENSGALG00000028254 | WASH complex subunit 4 | -0.19 | 6.0403 | 11.6834 | 6.31E-04 | 8.58E-03 |
| SSH1 | - - - - - | ----- | -0.19 | 5.4368 | 7.4100 | 6.49E-03 | 4.60E-02 |
| HGSNAT | ENSGALG00000010136 | heparan-alpha-glucosaminide N-acetyltransferase | -0.19 | 5.5631 | 10.8259 | 1.00E-03 | 1.20E-02 |
| SGF29 | ENSGALG00000020590 | SAGA complex associated factor 29 | -0.19 | 6.5443 | 10.4453 | 1.23E-03 | 1.39E-02 |
| RB1CC1 | ENSGALG00000015259 | RB1 inducible coiled-coil 1 | -0.19 | 5.7344 | 8.4153 | 3.72E-03 | 3.10E-02 |
| TELO2 | ENSGALG00000009297 | telomere maintenance 2 | -0.18 | 5.3252 | 9.6243 | 1.92E-03 | 1.96E-02 |
| CLCN7 | ENSGALG00000030826 | chloride voltage-gated channel 7 | -0.18 | 5.5771 | 8.8143 | 2.99E-03 | 2.64E-02 |
| IGDCC4 | ENSGALG00000007410 | immunoglobulin superfamily DCC subclass member 4 | -0.18 | 7.0035 | 7.2602 | 7.05E-03 | 4.85E-02 |
| ARFIP2 | ENSGALG00000037839 | ADP ribosylation factor interacting protein 2 | -0.18 | 6.0981 | 10.3729 | 1.28E-03 | 1.43E-02 |
| SLC22A5 | ENSGALG00000036920 | solute carrier family 22 member 5 | -0.18 | 6.7246 | 11.4229 | 7.25E-04 | 9.52E-03 |
| PCF11 | ENSGALG00000017249 | PCF11 cleavage and polyadenylation factor subunit | -0.18 | 7.2874 | 8.3178 | 3.93E-03 | 3.21E-02 |
| SMIM14 | ENSGALG00000014321 | small integral membrane protein 14 | -0.18 | 5.6410 | 7.5799 | 5.90E-03 | 4.29E-02 |
| ATPAF1 | ENSGALG00000010444 | ATP synthase mitochondrial F1 complex assembly factor 1 | -0.18 | 5.2283 | 7.5906 | 5.87E-03 | 4.27E-02 |
| IREB2 | ENSGALG00000035462 | iron responsive element binding protein 2 | -0.18 | 7.6632 | 11.4149 | 7.29E-04 | 9.53E-03 |
| SEC61A1 | ENSGALG00000005966 | Sec61 translocon alpha 1 subunit | -0.18 | 8.1082 | 9.6172 | 1.93E-03 | 1.96E-02 |
| LIPA | ENSGALG00000006378 | lipase A, lysosomal acid type | -0.18 | 5.7719 | 8.8151 | 2.99E-03 | 2.64E-02 |
| TMEM209 | ENSGALG00000008036 | transmembrane protein 209 | -0.18 | 5.8948 | 8.4334 | 3.68E-03 | 3.07E-02 |
| BTG1 | ENSGALG00000011276 | BTG anti-proliferation factor 1 | -0.18 | 7.2913 | 11.2515 | 7.96E-04 | 1.02E-02 |
| MIA3 | ENSGALG00000041034 | MIA family member 3, ER export factor | -0.18 | 6.7623 | 11.9298 | 5.52E-04 | 7.84E-03 |
| SLC25A20 | ENSGALG00000028227 | solute carrier family 25 member 20 | -0.18 | 7.1363 | 10.6443 | 1.10E-03 | 1.29E-02 |

| | | | | | | | |
|---|---|---|---|---|---|---|---|
| LOC107050717 | - - - - - | calumenin-like | -0.18 | 6.0546 | 7.7250 | 5.45E-03 | 4.05E-02 |
| USP44 | ENSGALG00000011400 | ubiquitin specific peptidase 44 | -0.17 | 6.7124 | 7.2634 | 7.04E-03 | 4.84E-02 |
| LIMK2 | ENSGALG00000006950 | LIM domain kinase 2 | -0.17 | 5.7644 | 7.3332 | 6.77E-03 | 4.72E-02 |
| STRADA | ENSGALG00000000550 | STE20-related kinase adaptor alpha | -0.17 | 6.7915 | 8.6211 | 3.32E-03 | 2.85E-02 |
| FAM98B | ENSGALG00000009744 | family with sequence similarity 98 member B | -0.17 | 7.0277 | 7.9021 | 4.94E-03 | 3.80E-02 |
| ATP6V1E1 | ENSGALG00000013036 | ATPase H+ transporting V1 subunit E1 | -0.17 | 5.8730 | 7.5095 | 6.14E-03 | 4.42E-02 |
| FMNL2 | ENSGALG00000012525 | formin like 2 | -0.17 | 6.9978 | 8.6995 | 3.18E-03 | 2.75E-02 |
| EPB41L1 | ENSGALG00000034171 | erythrocyte membrane protein band 4.1 like 1 | -0.17 | 6.6353 | 9.5191 | 2.03E-03 | 2.04E-02 |
| GATAD2A | ENSGALG00000042900 | GATA zinc finger domain containing 2A | -0.17 | 7.6874 | 10.9402 | 9.41E-04 | 1.15E-02 |
| CLCN3 | ENSGALG00000009674 | chloride voltage-gated channel 3 | -0.17 | 6.5591 | 10.2295 | 1.38E-03 | 1.52E-02 |
| TWF2 | ENSGALG00000003977 | twinfilin actin binding protein 2 | -0.17 | 7.2063 | 7.6669 | 5.62E-03 | 4.15E-02 |
| PRPF4 | ENSGALG00000008857 | pre-mRNA processing factor 4 | -0.17 | 6.1693 | 9.1313 | 2.51E-03 | 2.33E-02 |
| NRAS | ENSGALG00000026692 | neuroblastoma RAS viral oncogene homolog | -0.17 | 6.3362 | 9.4535 | 2.11E-03 | 2.09E-02 |
| TCEB3 | ENSGALG00000034625 | transcription elongation factor B subunit 3 | -0.16 | 5.7617 | 8.3751 | 3.80E-03 | 3.13E-02 |
| FZD1 | ENSGALG00000009064 | frizzled class receptor 1 | -0.16 | 7.4481 | 7.7681 | 5.32E-03 | 3.99E-02 |
| GMPPA | ENSGALG00000002417 | GDP-mannose pyrophosphorylase A | -0.16 | 6.1277 | 8.7730 | 3.06E-03 | 2.68E-02 |
| MIER2 | ENSGALG00000001108 | MIER family member 2 | -0.16 | 5.7777 | 8.9512 | 2.77E-03 | 2.50E-02 |
| ST7L | ENSGALG00000001538 | suppression of tumorigenicity 7 like | -0.16 | 5.5877 | 7.7772 | 5.29E-03 | 3.98E-02 |
| PDSS1 | ENSGALG00000007559 | prenyl (decaprenyl) diphosphate synthase, subunit 1 | -0.16 | 5.3148 | 7.5474 | 6.01E-03 | 4.34E-02 |
| MVB12B | ENSGALG00000000950 | multivesicular body subunit 12B | -0.16 | 7.0264 | 11.9003 | 5.61E-04 | 7.89E-03 |
| SLC39A9 | ENSGALG00000009438 | solute carrier family 39 member 9 | -0.16 | 6.8457 | 8.4361 | 3.68E-03 | 3.07E-02 |
| GTF2H5 | ENSGALG00000013733 | general transcription factor IIH subunit 5 | -0.16 | 6.9059 | 7.7408 | 5.40E-03 | 4.03E-02 |
| NUP133 | ENSGALG00000011091 | nucleoporin 133 | -0.16 | 6.9068 | 7.4123 | 6.48E-03 | 4.60E-02 |
| BAZ1A | - - - - - | bromodomain adjacent to zinc finger domain 1A | -0.15 | 6.4957 | 9.2488 | 2.36E-03 | 2.25E-02 |
| SCAP | ENSGALG00000035768 | SREBF chaperone | -0.15 | 7.1686 | 7.6460 | 5.69E-03 | 4.18E-02 |
| PRCC | ENSGALG00000040696 | papillary renal cell carcinoma (translocation-associated) | -0.15 | 6.8223 | 7.4012 | 6.52E-03 | 4.61E-02 |
| TMEM57 | ENSGALG00000001067 | transmembrane protein 57 | -0.15 | 6.3874 | 10.3007 | 1.33E-03 | 1.48E-02 |
| GNA11 | ENSGALG00000003238 | G protein subunit alpha 11 | -0.15 | 6.3483 | 7.4479 | 6.35E-03 | 4.54E-02 |
| RGL1 | ENSGALG00000004728 | ral guanine nucleotide dissociation stimulator like 1 | -0.15 | 7.2066 | 8.8781 | 2.89E-03 | 2.58E-02 |
| SGPL1 | ENSGALG00000004553 | sphingosine-1-phosphate lyase 1 | -0.14 | 6.6678 | 7.3730 | 6.62E-03 | 4.65E-02 |
| LOC428335 | - - - - - | hyperpolarization activated cyclic nucleotide-gated potassium channel 2 | -0.14 | 7.1072 | 8.7309 | 3.13E-03 | 2.72E-02 |
| TSN | ENSGALG00000011652 | translin | -0.14 | 7.2902 | 7.3414 | 6.74E-03 | 4.71E-02 |
| NDST1 | ENSGALG00000004581 | N-deacetylase and N-sulfotransferase 1 | -0.14 | 6.8812 | 7.2614 | 7.05E-03 | 4.84E-02 |
| SMAP2 | ENSGALG00000029244 | small ArfGAP2 | -0.14 | 6.5502 | 7.2516 | 7.08E-03 | 4.86E-02 |
| KCTD10 | ENSGALG00000005138 | potassium channel tetramerization domain containing 10 | -0.13 | 6.8974 | 8.0583 | 4.53E-03 | 3.56E-02 |
