# Supplementary material for: Phenotypic divergence between broiler and layer chicken lines is regulated at the molecular level during development
Source: BMC Genomics. 2024 Feb 12;25:168. doi: 10.1186/s12864-024-10083-x (PMC10863267; doi:10.1186/s12864-024-10083-x)
Supplement: Supplementary file 3 — Supplementary Material 3 [file 12864_2024_10083_MOESM3_ESM.pdf]

Table S3 - Metacore report for enrichment analysis of up-regulated DEGs in broilers (FDR<0.05).

Enrichment by GO Processes

				MetaCore_up-regulated DEGs_TT vs CC		
**#**	**Processes**	**GO terms**	**Total**	**p-value**	**FDR**	**In Data**
1	cellular metabolic process	0044237	9803	2.888E-34	2.527E-30	630
2	metabolic process	0008152	10984	1.234E-30	5.399E-27	672
3	organic substance metabolic process	0071704	10355	1.435E-28	4.185E-25	637
4	cellular process	0009987	20611	7.243E-28	1.584E-24	1033
5	nitrogen compound metabolic process	0006807	8991	2.356E-27	4.123E-24	570
6	primary metabolic process	0044238	9606	2.638E-26	3.847E-23	595
7	cellular nitrogen compound metabolic process	0034641	4558	9.655E-25	1.207E-21	339
8	cellular component organization or biogenesis	0071840	7982	1.422E-22	1.555E-19	505
9	cellular component organization	0016043	7724	2.022E-22	1.965E-19	492
10	organelle organization	0006996	4772	7.313E-22	6.398E-19	341
11	biosynthetic process	0009058	3874	1.802E-20	1.433E-17	288
12	organic cyclic compound metabolic process	1901360	4272	2.668E-20	1.933E-17	309
13	heterocycle metabolic process	0046483	3868	2.873E-20	1.933E-17	287
14	cellular macromolecule metabolic process	0044260	6517	4.343E-20	2.714E-17	424
15	cellular biosynthetic process	0044249	3674	7.358E-20	4.291E-17	275
16	organic substance biosynthetic process	1901576	3788	8.811E-20	4.818E-17	281
17	organonitrogen compound metabolic process	1901564	6893	6.435E-19	3.312E-16	438
18	cellular aromatic compound metabolic process	0006725	3936	5.033E-18	2.446E-15	283
19	cellular component biogenesis	0044085	3826	1.003E-17	4.618E-15	276
20	cellular response to stress	0033554	2525	1.192E-17	5.211E-15	203
21	cellular component assembly	0022607	3520	1.251E-17	5.211E-15	259
22	nucleobase-containing compound metabolic process	0006139	3605	1.922E-17	7.645E-15	263
23	developmental process	0044767	8604	3.250E-17	1.236E-14	513
24	macromolecule metabolic process	0044259	8228	1.810E-16	6.598E-14	492
25	cellular nitrogen compound biosynthetic process	0044271	2188	6.154E-16	2.154E-13	178
26	multicellular organism development	0007275	7455	9.186E-16	3.091E-13	452
27	anatomical structure development	0048856	7980	1.599E-15	5.180E-13	476
28	phosphate-containing compound metabolic process	0006796	2384	7.089E-15	2.215E-12	186
29	phosphorus metabolic process	0006793	2418	1.369E-14	4.130E-12	187

30	response to stress	0006950	5453	2.888E-14	8.421E-12	347
31	nucleic acid metabolic process	0090304	2937	1.036E-13	2.925E-11	213
32	cellular protein metabolic process	0044267	4694	1.075E-13	2.938E-11	306
33	protein-containing complex subunit organization	0071822	2233	1.418E-13	3.759E-11	173
34	protein-containing complex assembly	0006461	1924	1.474E-13	3.794E-11	155
35	positive regulation of molecular function	0044093	2397	1.800E-13	4.499E-11	182
36	cellular macromolecule biosynthetic process	0034645	2314	4.242E-13	1.031E-10	176
37	DNA metabolic process	0006259	1071	5.309E-13	1.255E-10	101
38	organic cyclic compound biosynthetic process	1901362	1719	5.760E-13	1.326E-10	141
39	regulation of cellular protein metabolic process	0032268	3517	7.707E-13	1.729E-10	241
40	heterocycle biosynthetic process	0018130	1510	7.957E-13	1.740E-10	128
41	positive regulation of transferase activity	0051347	1003	8.800E-13	1.878E-10	96
42	aromatic compound biosynthetic process	0019438	1531	9.811E-13	2.044E-10	129
43	system development	0048731	6600	1.288E-12	2.621E-10	396
44	macromolecule biosynthetic process	0009059	2368	1.626E-12	3.234E-10	177
45	intracellular signal transduction	0035556	2354	1.864E-12	3.624E-10	176
46	mitotic cell cycle	0000278	1101	2.796E-12	5.317E-10	101
47	positive regulation of cellular metabolic process	0031325	4586	3.203E-12	5.962E-10	294
48	organonitrogen compound biosynthetic process	1901566	1832	3.873E-12	7.060E-10	145
49	response to hormone	0009725	1700	4.622E-12	8.253E-10	137
50	mitotic cell cycle process	1903047	934	7.996E-12	1.399E-09	89

Table S3 - Metacore report for enrichment analysis of down-regulated DEGs in broilers (FDR<0.05).

Enrichment by GO Processes

#	Processes	GO terms	Total	p-value	FDR	In Data
				MetaCore_down-regulated DEGs_TT vs CC		
1	cellular process	0009987	20611	1.31E-27	1.12E-23	1005
2	localization	0051179	7692	2.20E-24	9.41E-21	486
3	regulation of biological quality	0065008	5771	3.49E-24	9.97E-21	392
4	transport	0006810	5914	1.03E-20	2.20E-17	387
5	establishment of localization	0051649	6110	1.54E-20	2.64E-17	396
6	cell development	0048468	2652	2.46E-20	3.19E-17	214
7	system development	0048731	6600	2.60E-20	3.19E-17	419
8	cell projection organization	0030030	1798	8.67E-20	9.03E-17	162
9	anatomical structure morphogenesis	0009653	3274	9.47E-20	9.03E-17	247
10	nervous system development	0007399	3418	3.57E-19	3.06E-16	253
11	plasma membrane bounded cell projection organization	0120036	1734	5.42E-19	4.22E-16	156
12	anatomical structure development	0048856	7980	1.08E-18	7.69E-16	478
13	multicellular organism development	0007275	7455	1.40E-18	9.22E-16	453
14	neuron differentiation	0030182	1676	4.62E-18	2.83E-15	150
15	neuron projection development	0031175	1099	5.18E-18	2.96E-15	113
16	positive regulation of transport	0051050	1592	9.53E-18	5.11E-15	144
17	neurogenesis	0022008	2236	1.40E-17	6.75E-15	182
18	developmental process	0044767	8604	1.50E-17	6.75E-15	502
19	generation of neurons	0048699	2049	1.50E-17	6.75E-15	171
20	regulation of signaling	0023051	4883	2.40E-17	1.03E-14	323
21	neuron development	0048666	1375	3.42E-17	1.40E-14	129
22	regulation of cell communication	0010646	4844	4.48E-17	1.75E-14	320
23	adenylate cyclase-modulating G protein-coupled receptor signaling pat	0007188	323	1.85E-16	6.89E-14	52
24	adenylate cyclase-activating G protein-coupled receptor signaling path	0010579	210	3.13E-16	1.12E-13	41
25	regulation of multicellular organismal process	0051239	4059	4.81E-16	1.65E-13	276
26	regulation of lyase activity	0051339	86	1.36E-15	4.48E-13	26
27	regulation of localization	0032879	4020	2.97E-15	9.42E-13	271
28	animal organ development	0048513	5239	1.19E-14	3.63E-12	330

29	regulation of transport	0051049	2764	1.45E-14	4.29E-12	202
30	regulation of phosphorus metabolic process	0051174	2298	1.70E-14	4.77E-12	176
31	regulation of cellular component organization	0051128	3480	1.73E-14	4.77E-12	240
32	regulation of cyclase activity	0031279	87	1.78E-14	4.77E-12	25
33	secretion	0046903	1469	3.09E-14	8.04E-12	127
34	regulation of phosphate metabolic process	0019220	2297	3.29E-14	8.31E-12	175
35	regulation of anatomical structure morphogenesis	0022603	1477	4.60E-14	1.13E-11	127
36	positive regulation of transmembrane transport	0034764	423	7.72E-14	1.84E-11	56
37	cell morphogenesis	0000902	1097	1.66E-13	3.85E-11	102
38	positive regulation of renal sodium excretion	0035815	25	1.96E-13	4.43E-11	14
39	cell differentiation	0030154	5389	2.06E-13	4.53E-11	332
40	cellular developmental process	0048869	5455	2.31E-13	4.94E-11	335
41	regulation of developmental process	0050793	3724	2.83E-13	5.91E-11	248
42	cell-cell signaling	0007267	1687	3.21E-13	6.55E-11	137
43	cellular component morphogenesis	0030154	939	3.61E-13	7.20E-11	91
44	actin cytoskeleton organization	0030036	745	3.92E-13	7.63E-11	78
45	regulation of signal transduction	0009966	4318	5.68E-13	1.08E-10	277
46	export from cell	0140352	1351	7.19E-13	1.34E-10	116
47	positive regulation of muscle contraction	0045933	78	1.01E-12	1.78E-10	22
48	cell communication	0007154	8259	1.01E-12	1.78E-10	464
49	plasma membrane bounded cell projection morphogenesis	0120039	759	1.02E-12	1.78E-10	78
50	secretion by cell	0032940	1279	1.20E-12	2.06E-10	111
