# Supplementary material for: Phenotypic divergence between broiler and layer chicken lines is regulated at the molecular level during development
Source: BMC Genomics. 2024 Feb 12;25:168. doi: 10.1186/s12864-024-10083-x (PMC10863267; doi:10.1186/s12864-024-10083-x)
Supplement: Supplementary file 4 — Supplementary Material 4 [file 12864_2024_10083_MOESM4_ESM.pdf]

Table S4. Mechanisms and interaction efects (activation and inhibition) among transcription factors and other molecules which participate in the GO Mitotic Cell Cycle network, using the analyze network (TF) algorithm by Metacore™ (Clarivate Analytics) [https://portal.genego.com/] from upregulated DEGs based on FDR<0.05 list between broilers (TT) and layers (CC) breeds.

Interactions Report

**From**		**To**						**From**	**DEGs Upregulated FDR<0.05**	**To**	**DEGs Upregulated FDR<0.05**
**Network Object "FROM"**	**Object Type**	**Network Object "TO"**	**Object Type**	**Interaction Effects**	**Mechanism**	**Link Info**	**References (PMID)**	**Input IDs**	**Fold Change**	**Input IDs**	**Fold Change**
**NF-AT1(NFATC2)**	Transcription factor	**PLK1**	Protein kinase	Inhibition	Transcription regulation	NF-AT1(NFATC2) inhibits transcription of PLK1.	34491912			**PLK1**	0.1533479
**RelA (p65 NF-kB subunit)**	Transcription factor	**PLK1**	Protein kinase	Activation	Transcription regulation	NF-kB subunit RelA transcriptionally activates PLK1.	21610149;25159142			**PLK1**	0.1533479
**STAT3**	Transcription factor	**PLK1**	Protein kinase	Activation	Transcription regulation	STAT3 probably binds to gene PLK1 promoter.	17498291;22108192;26405196;29339720;32962858;33126079			**PLK1**	0.1533479
**NF-AT1(NFATC2)**	Transcription factor	**Alpha-centractin**	Generic binding protein	Activation	Transcription regulation	NF-AT1(NFATC2) activates transcription of Alpha-centractin.	34491912			**ACTR1A**	0.1594823
**NF-AT1(NFATC2)**	Transcription factor	**Separase**	Generic protease	Activation	Transcription regulation	NF-AT1(NFATC2) activates transcription of	27935966;34491912			**ESPL1**	0.1598843
**NF-AT1(NFATC2)**	Transcription factor	**C10orf119**	Protein	Inhibition	Transcription regulation	NF-AT1(NFATC2) inhibits transcription of C10orf119.	34491912			**MCMBP**	0.1625428
**NF-AT1(NFATC2)**	Transcription factor	**FAM33A**	Protein	Activation	Transcription regulation	NF-AT1(NFATC2) activates transcription of	34491912			**SKA2**	0.1698637
**p63**	Transcription factor	**MRE11**	Generic enzyme	Activation	Transcription regulation	p63 and p73 bind to the promoter regions of Rad51, BRCA2, and mre11. Moreover, deltaNp63 and deltaNp73 transactivate Rad51, BRCA2, and mre11	19390658;19816568			**MRE11**	0.1825779
**FOXP3**	Transcription factor	**Tubulin beta 2C**	Generic binding protein	Activation	Transcription regulation	Tubulin beta 2C has binding sites in promoter region for FOXP3 identificated by chromatin immunoprecipitation.	17237761			**TUBB4B**	0.1873019
**NF-AT1(NFATC2)**	Transcription factor	**Tubulin beta 2C**	Generic binding protein	Inhibition	Transcription regulation	NF-AT1(NFATC2) inhibits transcription of Tubulin beta 2C.	34491912			**TUBB4B**	0.1873019
**RelA (p65 NF-kB subunit)**	Transcription factor	**ECT2**	Regulators (GDI, GAP, GEF)	Inhibition	Transcription regulation	RelA (p65 NF-kB subunit) inhibits transcription of ECT2.	28579529			**ECT2**	0.1936667
**STAT3**	Transcription factor	**ECT2**	Regulators (GDI, GAP, GEF)	Inhibition	Transcription regulation	STAT3 binds to gene ECT2 promoter and suppresses ECT2	18065416;22328012			**ECT2**	0.1936667
**HIF1A**	Transcription factor	**UBE2S**	Generic enzyme	Activation	Transcription regulation	E2-EPF is a hypoxia-inducible gene directly regulated via HIF1.	21281817			**UBE2S**	0.1945732

**c-Rel (NF-kB subunit)**	Transcription factor	**IRF4**	Transcription factor	Activation	Transcription regulation	C-Rel (NF-kB subunit) can bind to gene IRF4 promoter and activates IRF4 expression.	10770796;16272311;17513759;30850343			**MUM1**	0.1989764
**FOXP3**	Transcription factor	**IRF4**	Transcription factor	Activation	Transcription regulation	FOXP3 binds to IRF4 promoter and stimulates IRF4 mRNA expression.	17220892;17237761;17440451;19182775;19766086;21439160;22579475;28903735			**MUM1**	0.1989764
**STAT3**	Transcription factor	**IRF4**	Transcription factor	Activation	Transcription regulation	STAT3 binds to the Rora, as well as the Batf, Irf4, Ahr, and Maf genes.	20493732;21215659;21436836;21442426;28910419;30413785			**MUM1**	0.1989764
**RelA (p65 NF-kB subunit)**	Transcription factor	**ASAP**	Protein	Activation	Transcription regulation	RelA (p65 NF-kB subunit) activates transcription of ASAP.	28579529			**MAP9**	0.2126209
**RelA (p65 NF-kB subunit)**	Transcription factor	**WDHD1**	Generic binding protein	Inhibition	Transcription regulation	RelA (p65 NF-kB subunit) inhibits transcription of WDHD1.	28579529			**WDHD1**	0.216176
**c-Rel (NF-kB subunit)**	Transcription factor	**ATM**	Protein kinase	Activation	Transcription regulation	c-Rel directly transactivates ATM, EP300 (p300), SFN (14-3-3sigma), TGF-beta1, MMP1 (matrix metalloproteinase 1) and [illegible]	19502793			**ATM**	0.2279364
**p63**	Transcription factor	**ATM**	Protein kinase	Activation	Transcription regulation	ATM is a direct DeltaNp63 alpha transcriptional target.	20663147;29362488			**ATM**	0.2279364
**c-Rel (NF-kB subunit)**	Transcription factor	**Cyclin D1**	Generic binding protein	Activation	Transcription regulation	c-Rel (NF-kB subunit) can bind to gene Cyclin D1 promoter.	18226221;19847166;23552605			**CCND1**	0.2298516
**HIF1A**	Transcription factor	**Cyclin D1**	Generic binding protein	Inhibition	Transcription regulation	HIF-1 was able to directly bind to the promoter region of cyclin D1.	20179204;23289374;23300831;23555163;23839513;25313256;25681586;26151812;26364616;26497365;26702149;26920732;27708244;30979776;31884068;33931028			**CCND1**	0.2298516
**p63**	Transcription factor	**Cyclin D1**	Generic binding protein	Activation	Transcription regulation	p63 activates transcription of Cyclin D1.	23837456;24718831;27341130;27447744;31826234;33194023;33833226			**CCND1**	0.2298516
**RelA (p65 NF-kB subunit)**	Transcription factor	**Cyclin D1**	Generic binding protein	Activation	Transcription regulation	RELA (NF-kappaB p65 subunit) interacts with the CCND1 (Cyclin D1) promoter.	15652748;15691880;15755876;15870875;16298738;16331275;16498412;16518410;16682409;17008396;17404108;18174238;19016262;19444329;19800042;19880242;20462455;20509140;21056029;21131967;21901538;23037503;23172667;23552605;23563178;23591394;23981302;24508135;24531845;24760979;25331947;25412312;25661319;26874278;27166260;27443256;27811358;28696292;31757943;32627006;32816854;34764323			**CCND1**	0.2298516

**p63**	Transcription factor	**CDK1 (p34)**	Protein kinase	Activation	Transcription regulation	p63 and p73 bind to and stimulate activity of ADA, POLD2, Cyclin D3, Cdk1 and CDC25c promoters.	19861536;23229819;31826234			**CDK1**	0.2307478
**RelA (p65 NF-kB subunit)**	Transcription factor	**CENP-E**	Generic binding protein	Inhibition	Transcription regulation	RelA (p65 NF-kB subunit) inhibits transcription of CENP-E.	28579529			**CENPE**	0.2331247
**RelA (p65 NF-kB subunit)**	Transcription factor	**Rod**	Generic binding protein	Inhibition	Transcription regulation	RelA (p65 NF-kB subunit) inhibits transcription of	28579529			**KNTC1**	0.2402103
**NF-AT1(NFATC2)**	Transcription factor	**MIS12**	Generic binding protein	Inhibition	Transcription regulation	NF-AT1(NFATC2) inhibits transcription of MIS12.	34491912			**MIS12**	0.2468404
**NF-AT1(NFATC2)**	Transcription factor	**CENP-H**	Generic binding protein	Activation	Transcription regulation	NF-AT1(NFATC2) activates transcription of	34491912			**CENPH**	0.2581865
**p63**	Transcription factor	**NFBD1**	Generic binding protein	Inhibition	Transcription regulation	NFBD1 was identificated as p63 target gene by using chromatin immunoprecipitation analysis and RT-PCR.	17036050;19390658;20023394			**MDC1**	0.2734507
**STAT3**	Transcription factor	**NFBD1**	Generic binding protein	Activation	Transcription regulation	Stat3 probably regulates transcription of NFBD1.	18555785;20064451;26261078			**MDC1**	0.2734507
**NF-AT1(NFATC2)**	Transcription factor	**Aladin**	Generic binding protein	Activation	Transcription regulation	NF-AT1(NFATC2) activates transcription of	34491912			**AAAS**	0.30629
**RelA (p65 NF-kB subunit)**	Transcription factor	**CDC20**	Generic binding protein	Activation	Transcription regulation	RelA (p65 NF-kB subunit) activates transcription of CDC20.	25362179			**CDC20**	0.314848
**RelA (p65 NF-kB subunit)**	Transcription factor	**HKLP2**	Generic binding protein	Inhibition	Transcription regulation	RelA (p65 NF-kB subunit) inhibits transcription of HKLP2.	28579529			**KIF15**	0.37926
**HIF1A**	Transcription factor	**BCAT1**	Generic enzyme	Activation	Transcription regulation	HIF1A activates transcription of BCAT1.	32088728			**BCAT1**	
**NF-AT1(NFATC2)**	Transcription factor	**CAP-E**	Generic binding protein	Activation	Transcription regulation	NF-AT1(NFATC2) activates transcription of	34491912			**SMC2**	
**STAT3**	Transcription factor	**HSP70**	Generic binding protein	Activation	Transcription regulation	STAT3 binds to the sequence of the HSP70 promoter and stimulates HSP70 expression.	19185844;19754877;22069317			**HSPA4;HSPA8;HSPA5**	
**NF-AT1(NFATC2)**	Transcription factor	**HSC70**	Generic enzyme	Inhibition	Transcription regulation	NF-AT1(NFATC2) inhibits transcription of HSC70.	34491912			**HSPA8**	
**STAT3**	Transcription factor	**HSC70**	Generic enzyme	Activation	Transcription regulation	STAT3 activates transcription of HSC70.	18555785;22069317			**HSPA8**	
**NF-AT1(NFATC2)**	Transcription factor	**NAA50**	Generic enzyme	Inhibition	Transcription regulation	NF-AT1(NFATC2) inhibits transcription of NAA50.	34491912			**MAK**	
**RelA (p65 NF-kB subunit)**	Transcription factor	**NAA50**	Generic enzyme	Activation	Transcription regulation	RelA (p65 NF-kB subunit) activates transcription of NAA50.	23603904			**MAK**	
**c-Rel (NF-kB subunit)**	Transcription factor	**INSC**	Generic binding protein	Activation	Transcription regulation	c-Rel (NF-kB subunit) activates transcription of INSC.	26694615			**INSC**	

**RelA (p65 NF-kB subunit)**	Transcription factor	**HSP90 alpha**	Generic binding protein	Activation	Transcription regulation	Interaction of purified RelA (p65 NF-kB subunit) with HSP90 alpha was shown by liquid-chromatography mass spectrometry and directed functional perturbation studies using RNA interference.	14743216;17724475;24523406			**HSP90AA1**	
**STAT3**	Transcription factor	**HSP90 alpha**	Generic binding protein	Activation	Transcription regulation	STAT3 binds to gene HSP90 alpha promoter and activates HSP90 alpha expression.	12559950;17427945;21079652;23228483			**HSP90AA1**	
**c-Rel (NF-kB subunit)**	Transcription factor	**NFKBIA**	Generic binding protein	Activation	Transcription regulation	Interaction of purified c-Rel (NF-kB subunit) with NFKBIA was shown by liquid-chromatography mass spectrometry and directed functional perturbation studies using RNA interference.	7937093;8262046;8887627;10706725;14743216;14961554;17513759;17579043;17675583;18508759;19797428;20740013;22011580;25159142;32817348				
**NF-AT1(NFATC2)**	Transcription factor	**NFKBIA**	Generic binding protein	Activation	Transcription regulation	NF-AT1(NFATC2) activates transcription of	34491912				
**NOTCH1 (NICD)**	Transcription factor	**NFKBIA**	Generic binding protein	Inhibition	Transcription regulation	NOTCH1 (NICD) inhibits transcription of NFKBIA.	16838279;18560356				

**RelA (p65 NF-kB subunit)**	Transcription factor	**NFKBIA**	Generic binding protein	Activation	Transcription regulation	NFkappaBp65 binds to IkappaB promoter and activates expression of NFKBIA	8246997;8319912;9150141;9566883;9694722;9738011;9751059;9865693;9990853;10364173;10498867;11413190;11533489;11981037;12162804;12419806;12718890;12967348;12972430;14685242;14743216;15102471;15226358;15308667;15464842;15657065;15799966;15892871;15975999;16163708;16408291;16467852;16675465;16792530;16817975;16928772;16951195;17254973;17362989;17409387;17537731;17579043;17612295;17622249;17675583;17707233;18029440;18045535;18204072;18276832;18334673;18401342;18408078;18455150;18508759;18583959;18987305;18988733;19338389;19342686;19556516;19668231;19747262;19797428;19854828;19874202;20007970;20368414;20534538;20634424;20685965;20713516;20740013;21098220;21108432;21173233;21464397;21483817;21862580;21945668;22189654;22623725;22696686;22723832;23188828;23247759;23297421;23392668;23422506;23527155;23715268;23898399;24019758;24129565;24303801;24459141;24500711;24634218;2467				
**STAT3**	Transcription factor	**NFKBIA**	Generic binding protein	Inhibition	Transcription regulation	STAT3 inhibits transcription of NFKBIA.	15958548;27668411;33805945				
**STAT3**	Transcription factor	**Pin1**	Generic enzyme	Activation	Transcription regulation	STAT3 activates transcription of Pin1.	22394374;29269260				
**HIF1A**	Transcription factor	**CD40(TNFRSF5)**	Generic receptor	Activation	Transcription regulation	HIF1A activates transcription of CD40(TNFRSF5).	34103524				
**NF-AT1(NFATC2)**	Transcription factor	**CD40(TNFRSF5)**	Generic receptor	Activation	Transcription regulation	NF-AT1(NFATC2) activates transcription of CD40(TNFRSF5).	34491912				
**RelA (p65 NF-kB subunit)**	Transcription factor	**CD40(TNFRSF5)**	Generic receptor	Activation	Transcription regulation	RelA (p65 NF-kB subunit) activates transcription of CD40(TNFRSF5).	11830590;12193701;16020513;17114447;27916733;28214900				
**STAT3**	Transcription factor	**CD40(TNFRSF5)**	Generic receptor	Activation	Transcription regulation	STAT3 probably regulates transcription of CD40(TNFRSF5) in human B-cell lymphoma.	18277385;34103524				
**c-Rel (NF-kB subunit)**	Transcription factor	**NF-AT1(NFATC2)**	Transcription factor	Activation	Transcription regulation	c-Rel (NF-kB subunit) activates transcription of NF-AT1(NFATC2).	25299780				

**c-Rel (NF-kB subunit)**	Transcription factor	**NF-kB2 (p100)**	Transcription factor	Activation	Transcription regulation	Interaction of purified c-Rel (NF-kB subunit) with NF-kB2 (p100) was shown by liquid-chromatography mass spectrometry and directed functional perturbation studies using RNA interference.	8152812;8413211;14743216;16191192;16595631;16973832;19270711;19494297;25159142;26186194;26324762;28514442;28767691				
**c-Rel (NF-kB subunit)**	Transcription factor	**FOXP3**	Transcription factor	Activation	Transcription regulation	JunB and c-Rel cooperatively enhance Foxp3 expression during induced regulatory T cell differentiation.	20064449;20064450;20072126;21371435;21472440;21490927;23200824;23297791;23405894;27217485				
**c-Rel (NF-kB subunit)**	Transcription factor	**HIF1A**	Transcription factor	Activation	Transcription regulation	c-Rel (NF-kB subunit) activates transcription of HIF1A.	10425220;18393939;31332228				
**FOXP3**	Transcription factor	**c-Rel (NF-kB**	Transcription factor	Inhibition	Transcription regulation	FOXP3 inhibits transcription of c-Rel (NF-	17237765;21490927;28903735				
**HIF1A**	Transcription factor	**RelA (p65 NF-kB subunit)**	Transcription factor	Activation	Transcription regulation	HIF1A activates transcription of RelA (p65 NF-kB subunit).	19454749;21642357;23123196;24535079;25368386;25703326;25823824;26172294;26696754;27191981;31432144				
**HIF1A**	Transcription factor	**c-Rel (NF-kB subunit)**	Transcription factor	Activation	Transcription regulation	HIF1A activates transcription of c-Rel (NF-kB subunit).	25823824				
**HIF1A**	Transcription factor	**p63**	Transcription factor	Activation	Transcription regulation	HIF1A activates transcription of p63.	30655535;33920782				
**HIF1A**	Transcription factor	**FOXP3**	Transcription factor	Activation	Transcription regulation	HIF-1A interacts with Foxp3 in iTreg cells and HIF-1 mediates the degradation of Foxp3.	21871655;22579475;22988108;27557492;30185770;33438204				
**HIF1A**	Transcription factor	**STAT3**	Transcription factor	Activation	Transcription regulation	HIF1A can form an active complex with the transcriptional co-activator p300 and phosphorylated-STAT3 at the VEGF promoter.	15735682;18985005;19336759;21960021;24718784;26404487;29666476;34014492				
**HIF1A**	Transcription factor	**HIF1A**	Transcription factor	Activation	Transcription regulation	HIF1A activates transcription of HIF1A .	12764143;16010420;18003758;21042279				
**IRF4**	Transcription factor	**FOXP3**	Transcription factor	Inhibition	Transcription regulation	IRF4 inhibits transcription of FOXP3.	19088203;19182775;28824171	**MUM1**	0.1989764		
**NF-AT1(NFATC2)**	Transcription factor	**c-Rel (NF-kB subunit)**	Transcription factor	Activation	Transcription regulation	NF-AT1(NFATC2) binds to gene c-Rel (NF-kB subunit) promoter and activates c-Rel (NF-kB subunit) expression.	17617603				
**NF-AT1(NFATC2)**	Transcription factor	**STAT3**	Transcription factor	Activation	Transcription regulation	NF-AT1(NFATC2) activates transcription of	34491912				
**NF-AT1(NFATC2)**	Transcription factor	**NF-kB2 (p100)**	Transcription factor	Activation	Transcription regulation	NF-AT1(NFATC2) activates transcription of NF-kB2 (p100).	34491912				

**NF-AT1(NFATC2)**	Transcription factor	**FOXP3**	Transcription factor	Activation	Transcription regulation	NF-ATc2 binds to FOXP3 promoter and activates it.	15790681;16517728;18326816;19564342;20064450;20108139;21226022;22991461;25583478;26777750				
**NF-AT1(NFATC2)**	Transcription factor	**NF-AT1(NFATC2)**	Transcription factor	Activation	Transcription regulation	NF-AT1(NFATC2) activates transcription of NF-AT1(NFATC2).	9794241;12949491;15546140;15928679;34491912				
**NF-kB2 (p100)**	Transcription factor	**HIF1A**	Transcription factor	Activation	Transcription regulation	NF-kB2 (p100) activates transcription of HIF1A.	18393939				
**p63**	Transcription factor	**p63**	Transcription factor	Activation	Transcription regulation	p63 activates transcription of p63.	18198175;26203771;29339502;29440247;30713093;31551362				
**RelA (p65 NF-kB subunit)**	Transcription factor	**c-Rel (NF-kB subunit)**	Transcription factor	Activation	Transcription regulation	RelA (p65 NF-kB subunit) activates transcription of c-Rel (NF-kB subunit).	7649478;8139561;11043773;12162804;14743216;15143182;15818410;17072328;17693123;18309928;18832697;19023096;19087197;19094066;19273289;19302050;19519322;19524538;19607980;20026420;20503182;20740013;21368872;21779400;23646894;23982206;24722732;25416956;26186194;26324762;26887983;28514442;29603325;32492432				
**RelA (p65 NF-kB subunit)**	Transcription factor	**HIF1A**	Transcription factor	Activation	Transcription regulation	RelA (p65 NF-kB subunit) binds to HIF1A promoter and induces HIF1A expression	17272744;17898080;18393939;18432192;20813154;21565532;23123196;23185615;23311761;23713977;24042437;24213609;24316875;24477458;24535079;25028521;25749383;25823824;25879517;26154152;26647819;26743088;27144516;27191981;28000883;28257048;28882872;29343683;29535421;30628067;31332228;31391533				
**RelA (p65 NF-kB subunit)**	Transcription factor	**p63**	Transcription factor	Activation	Transcription regulation	RelA (p65 NF-kB subunit) binds to p63 promoter and activates p63 expression.	20052674;21088498;24069219;31523027;34172737				
**RelA (p65 NF-kB subunit)**	Transcription factor	**RelA (p65 NF-kB subunit)**	Transcription factor	Activation	Transcription regulation	RelA (p65 NF-kB subunit) activates transcription of RelA (p65 NF-kB subunit).	16331275;29485902;29898962;33298918				

**RelA (p65 NF-kB subunit)**	Transcription factor	**NF-kB2 (p100)**	Transcription factor	Activation	Transcription regulation	Interaction of purified RelA (p65 NF-kB subunit) with NF-kB2 (p100) was shown by liquid-chromatography mass spectrometry and directed functional perturbation studies using RNA interference.	8413211;14743216;16009713;16191192;16595631;16817975;16973832;17254973;17855547;19524538;21102550;22388891;23016877;23247759;24722732;24825921;25159142;26324762;26588041;28276104;28579529;29476964;30366905;32078488;34155144				
**RelA (p65 NF-kB subunit)**	Transcription factor	**STAT3**	Transcription factor	Activation	Transcription regulation	RelA (p65 NF-kB subunit) activates transcription of STAT3 .	12057007;16189514;16236134;16713569;17487688;18811964;19345327;20038813;21187858;21556775;22586032;23178493;23370526;25931145;28433552;28637784				
**RelA (p65 NF-kB subunit)**	Transcription factor	**FOXP3**	Transcription factor	Activation	Transcription regulation	Human proximal FOXP3 promoter is controlled by activation through the TCR involving PKC and the NF-kappaB subunit p65 and by inhibition through a negative feedback loop [illegible]	18326816;19701891;20064450;20462637;20966256;21226022;21268019;21508258;22576743;23144749;23178569;25154413;26163261				
**STAT3**	Transcription factor	**FOXP3**	Transcription factor	Activation	Transcription regulation	STAT3 binds to gene FOXP3 promoter and activates FOXP3 expression.	16645171;17878325;18326816;19379825;20164832;20404810;22328012;25993445;26198700;28011648;28469791				
**STAT3**	Transcription factor	**NF-kB2 (p100)**	Transcription factor	Activation	Transcription regulation	STAT3 activates transcription of NF-kB2 (p100).	16651533;33184494				
**STAT3**	Transcription factor	**p63**	Transcription factor	Activation	Transcription regulation	Binding of the endogenous STAT3 to the Np63 promoter in Hep3B cells was demonstrated by ChIP assay. EMSA and ABCD assay shows direct binding of STAT3 to STAT3-RE of the Np63 promoter and such binding is stimulated by Np63.	18198175;22328012;22806179;25546438				

**STAT3**	Transcription factor	**HIF1A**	Transcription factor	Activation	Transcription regulation	Stat3 protein directly binds to the HIF-1 promoter and contributes to HIF-1 transcriptional regulation.	18644974;18985005;19020709;19265129;20924113;21102525;21827581;21871655;21960021;22328012;22402588;23486688;23604114;23612755;23991099;24435707;24525913;24658058;24718784;24820265;24981247;25193384;25492480;25655308;25833823;26154152;26324850;27011063;27220595;27325313;27633343;27806334;28257048;28345605;28499822;29343683;30036966;30454647;31068616;31286834;31332228;31481496;32142763;32369445;33332446;33753024;34363022;34702814				
**STAT3**	Transcription factor	**NF-AT1(NFATC2)**	Transcription factor	Activation	Transcription regulation	STAT3binds and activates the NFATc2 gene.	20493732;21321078;28804544				
