# Supplementary material for: Phenotypic divergence between broiler and layer chicken lines is regulated at the molecular level during development
Source: BMC Genomics. 2024 Feb 12;25:168. doi: 10.1186/s12864-024-10083-x (PMC10863267; doi:10.1186/s12864-024-10083-x)
Supplement: Supplementary file 5 — Supplementary Material 5 [file 12864_2024_10083_MOESM5_ESM.pdf]

Table S6. Mechanisms and interactions efects among transcription factors and other molecules which participate in the GO Cell Differentiation network, using the analyze network (TF) algorithm by Metacore™ (Clarivate Analytics) [https://portal.genego.com/] from downregulated DEGs based on FDR<0.05 list between broilers (TT) and layers (CC) lines.

Interactions Report

From		To						From	DEGs Downregulated FDR<0.05	To	DEGs Downregulated FDR<0.05
**Network Object "FROM"**	**Object Type**	**Network Object "TO"**	**Object Type**	**Interaction Effects**	**Mechanism**	**Link Info**	**References (PMID)**	**Input IDs**	**Fold Change**	**Input IDs**	**Fold Change**
**CREB1**	Transcription factor	**WISP1**	Generic binding protein	Activation	Transcription regulation	WISP1 promoter has a putative CREB1-binding site.	15753290;18481207;19238344;19339243			**WISP1**	-1.17
**CREB1**	Transcription factor	**TSH receptor**	GPCR	Unspecified	Transcription regulation	TSH receptor promoter has a putative CREB1-binding site.	15753290			**TSHR**	-1.13
**Androgen receptor**	Transcription factor	**Clusterin**	Generic binding protein	Activation	Transcription regulation	Androgen receptor can bind to gene Clusterin promoter and activates Clusterin expression.	7999255;17148459;17299418;22383394;23476140;23715282	**AR**	-0.44198	**TRPM2**	-1.07
**CREB1**	Transcription factor	**TRPM2**	Generic channel	Unspecified	Transcription regulation	TRPM2 promoter has a putative CREB1-binding site.	15708008;15753290			**TRPM2**	-1.07
**Androgen receptor**	Transcription factor	**TRPM2**	Generic channel	Unspecified	Transcription regulation	Androgen receptor can bind to gene TRPM2 promoter.	17679089;33640491	**AR**	-0.44198	**TRPM2**	-1.07
**SMAD3**	Transcription factor	**MSX-2**	Transcription factor	Unspecified	Transcription regulation	SMAD3 regulates transcription of MSX-2.	30042129			**MSX2**	-1.05
**CREB1**	Transcription factor	**MSX-2**	Transcription factor	Unspecified	Transcription regulation	MSX-2 promoter has a putative CREB1-binding site.	15459107;15753290			**MSX2**	-1.05
**Androgen receptor**	Transcription factor	**MSX-2**	Transcription factor	Unspecified	Transcription regulation	Androgen receptor probably regulates gene MSX2 transcription.	20634343;33640491	**AR**	-0.44198	**MSX2**	-1.05
**SMAD3**	Transcription factor	**CX3CR1**	GPCR	Activation	Transcription regulation	SMAD3 activates transcription of CX3CR1.	29234059			**CX3CR1**	-1.02
**Androgen receptor**	Transcription factor	**TGM3**	Generic enzyme	Unspecified	Transcription regulation	Using the MNI algorithm, we identified TGM3 as AR downstream target gene in the AR pathway.	17299418	**AR**	-0.44198	**TGM3**	-1.01
**Androgen receptor**	Transcription factor	**NCAM2**	Generic binding protein	Unspecified	Transcription regulation	Androgen receptor regulates transcription of NCAM2.	27221037	**AR**	-0.44198	**NCAM2**	-0.98
**SMAD3**	Transcription factor	**FSHR**	GPCR	Activation	Transcription regulation	SMAD3 binds to the FSHR promoter and activates it.	19535790;23690627;24979474			**FSHR**	-0.98
**Androgen receptor**	Transcription factor	**FSHR**	GPCR	Activation	Transcription regulation	Androgen receptor activates transcription of FSHR.	14551232;15761038;25941928;33662476	**AR**	-0.44198	**FSHR**	-0.98
**CREB1**	Transcription factor	**DLX3**	Transcription factor	Unspecified	Transcription regulation	DLX3 promoter has a putative CREB1-binding site.	15753290;33846571			**DLX3**	-0.96
**Androgen receptor**	Transcription factor	**PTPRR**	Protein phosphatase	Inhibition	Transcription regulation	Using the MNI algorithm, we identified PTPRR as AR downstream target gene in the AR pathway.	17299418;25592066	**AR**	-0.44198	**PTPRQ**	-0.92
**ATF-2**	Transcription factor	**Histone H4**	Generic binding protein	Activation	Covalent modification	ATF-2 covalently modifies Histone H4 and activates it.	10821277			**HIST1H4I**	-0.92

**HDAC2**	Generic enzyme	**Histone H4**	Generic binding protein	Unspecified	Deacetylation	HDAC2 deacetylates Histone H4.	10958685;22441348;22579787;24102378;27568559;27568567			**HIST1H4I**	-0.92
**p300**	Generic enzyme	**Histone H4**	Generic binding protein	Unspecified	Covalent modification	p300 acetylates Histone H4	8945521;9726987;10025405;10497301;10921904;11163245;11511360;11559745;11940655;12052856;12609999;15289617;15834423;15932940;16325578;17267393;17300219;17320507;18397880;21315607;21789216;23303459			**HIST1H4I**	-0.92
**CBP**	Generic enzyme	**Histone H4**	Generic binding protein	Unspecified	Acetylation	CBP acetylates Histone H4.	8945521;9823900;10497301;11134336;11163245;11559745;11607014;12609999;12665567;12748280;17267393;17320507;21315607;21789216;23831576;24748101;27934968;31801086			**HIST1H4I**	-0.92
**CREB1**	Transcription factor	**Histone H4**	Generic binding protein	Activation	Transcription regulation	CREB1 can bind to gene Histone H4 promoter and activates Histone H4 expression.	9398163;15753290			**HIST1H4I**	-0.92
**SMAD3**	Transcription factor	**BAFF(TNFSF13B)**	Receptor ligand	Activation	Transcription regulation	SMAD3 binds to gene BAFF(TNFSF13B) promoter and promotes BAFF(TNFSF13B) expression.	18334541			**TNFSF13B**	-0.88
**CREB1**	Transcription factor	**PCSK9**	Generic protease	Unspecified	Transcription regulation	PCSK9 promoter has a putative CREB1-binding site.	15753290;33846571			**PCSK9**	-0.87
**CREB1**	Transcription factor	**Tenascin-R**	Generic binding protein	Unspecified	Transcription regulation	Tenascin-R promoter has a putative CREB1-binding site.	9925948;12927810;15753290			**TNR**	-0.86
**Androgen receptor**	Transcription factor	**PARD3**	Generic binding protein	Unspecified	Transcription regulation	PARD3 is a possible transcriptional target of Androgen receptor.	19668381	**AR**	-0.44198	**ASIP**	-0.85
**CREB1**	Transcription factor	**PARD3**	Generic binding protein	Unspecified	Transcription regulation	PARD3 promoter has a putative CREB1-binding site.	15753290			**ASIP**	-0.85
**ATF/CREB**	Transcription factor	**NTN4**	Generic binding protein	Unspecified	Transcription regulation	ATF/CREB regulates transcription of NTN4.	32871102	**BATF**	-0.686649	**NTN4**	-0.83
**CREB1**	Transcription factor	**HOXA7**	Transcription factor	Unspecified	Transcription regulation	HOXA7 promoter has a putative CREB1-binding site.	7890170;15753290			**HOXA9**	-0.83

**ATF-2**	Transcription factor	**Galectin-1**	Receptor ligand	Unspecified	Transcription regulation	ATF-2 regulates transcription of Galectin-1.	33599733			**GAL**	-0.82
**Androgen receptor**	Transcription factor	**CD109**	Generic binding protein	Unspecified	Transcription regulation	Androgen receptor regulates transcription of CD109.	33687952	**AR**	-0.44198	**CD109**	-0.80
**SMAD3**	Transcription factor	**CCR7**	GPCR	Unspecified	Transcription regulation	SMAD3 probably regulates transcription of CCR7 in human B-cell lymphoma.	18277385;25961925			**CCR7**	-0.79
**Androgen receptor**	Transcription factor	**C3**	Generic binding protein	Inhibition	Transcription regulation	Androgen receptor inhibits transcription of C3.	10395065;26468082	**AR**	-0.44198	**C3**	-0.78
**SMAD3**	Transcription factor	**Myocardin**	Transcription factor	Activation	Transcription regulation	Smad2/3 and Smad4 bind and activate the gene Myocardin promoter.	16224064;21673106;32029901			**MYOCD**	-0.78
**Androgen receptor**	Transcription factor	**DSCAM**	Generic binding protein	Unspecified	Transcription regulation	Androgen receptor binds to gene DSCAM promoter.	19668381	**AR**	-0.44198	**DSCAM**	-0.75
**CREB1**	Transcription factor	**AP180**	Generic binding protein	Unspecified	Transcription regulation	AP180 promoter has a putative CREB1-binding site.	15753290			**SNAP91**	-0.72
**Androgen receptor**	Transcription factor	**STMN2**	Generic binding protein	Unspecified	Transcription regulation	Using the MNI algorithm, we identified STMN2 as AR downstream gene in the AR pathway.	17299418	**AR**	-0.44198	**STMN2**	-0.72
**HDAC2**	Generic enzyme	**BATF**	Transcription factor	Unspecified	Transcription regulation	HDAC2 co-regulates transcription of BATF.	32796851			**BATF**	-0.69
**p300**	Generic enzyme	**BATF**	Transcription factor	Unspecified	Transcription regulation	p300 co-regulates transcription of BATF.	32871225			**BATF**	-0.69
**CREB1**	Transcription factor	**BATF**	Transcription factor	Unspecified	Transcription regulation	BATF promoter has a putative CREB1-binding site.	15753290;18077348			**BATF**	-0.69
**CREB1**	Transcription factor	**SFRP4**	Generic binding protein	Unspecified	Transcription regulation	SFRP4 promoter has a putative CREB1-binding site.	12974383;15753290			**SFRP4**	-0.67
**CREB1**	Transcription factor	**CYP26C1**	Generic enzyme	Unspecified	Transcription regulation	CYP26C1 promoter has a putative CREB1-binding site.	15753290			**CYP26C1**	-0.66
**CREB1**	Transcription factor	**RXRA**	Transcription factor	Unspecified	Transcription regulation	RXRA promoter has a putative CREB1-binding site.	15753290			**RXRA**	-0.65
**Androgen receptor**	Transcription factor	**ABCA12**	Transporter	Unspecified	Transcription regulation	Androgen receptor regulates transcription of ABCA12.	33640491	**AR**	-0.44198	**ABCA12**	-0.63
**CREB1**	Transcription factor	**ABCA12**	Transporter	Unspecified	Transcription regulation	ABCA12 promoter has a putative CREB1-binding site.	15753290			**ABCA12**	-0.63
**CREB1**	Transcription factor	**Sciellin**	Generic binding protein	Unspecified	Transcription regulation	Sciellin promoter has a putative CREB1-binding site.	15753290;33846571			**SCEL**	-0.63
**CREB1**	Transcription factor	**MYO3A**	Generic kinase	Unspecified	Transcription regulation	MYO3A promoter has a putative CREB1-binding site.	15753290			**MYO3A**	-0.62
**CREB1**	Transcription factor	**ALKBH1**	Generic enzyme	Unspecified	Transcription regulation	ALKBH1 promoter has a putative CREB1-binding site.	15753290			**ALKBH1**	-0.59
**Androgen receptor**	Transcription factor	**ALKBH1**	Generic enzyme	Unspecified	Transcription regulation	Androgen receptor regulates transcription of ALKBH1.	24027196	**AR**	-0.44198	**ALKBH1**	-0.59
**CREB1**	Transcription factor	**PTP-2**	Generic receptor	Unspecified	Transcription regulation	PTP-2 promoter has a putative CREB1-binding site.	15753290;33846571			**PTPRO**	-0.58
**CREB1**	Transcription factor	**TESC**	Generic binding protein	Unspecified	Transcription regulation	TESC promoter has a putative CREB1-binding site.	15753290			**TESC**	-0.57
**Androgen receptor**	Transcription factor	**Alpha-actinin 2**	Generic binding protein	Unspecified	Transcription regulation	Using the MNI algorithm, we identified Alpha-actinin 2 as AR downstream gene in the AR pathway.	15072553;17299418	**AR**	-0.44198	**ACTN2**	-0.57

**Androgen receptor**	Transcription factor	**EPHA10**	Receptor with enzyme activity	Unspecified	Transcription regulation	Androgen receptor binds to gene EPHA10 promoter.	19668381	**AR**	-0.44198	**EPHA10**	-0.52
**Androgen receptor**	Transcription factor	**SMAD6**	Transcription factor	Unspecified	Transcription regulation	Androgen receptor regulates transcription of SMAD6.	33640491	**AR**	-0.44198	**SMAD6**	-0.52
**CREB1**	Transcription factor	**SMAD6**	Transcription factor	Activation	Transcription regulation	CREB1 binds to gene SMAD6 promoter and activates SMAD6 expression.	14755548			**SMAD6**	-0.52
**p300**	Generic enzyme	**Histone H3.2**	Generic binding protein	Unspecified	Acetylation	Histone H3.2 is acetylated by p300.	16096645;21669532			**HIST2H3D**	-0.52
**HDAC2**	Generic enzyme	**Histone H3**	Generic binding protein	Unspecified	Deacetylation	HDAC2 deacetylates Histone H3 tails.	[illegible]7819;22579787;24102378;24935000;26934447;26980768;30979734;32315286;33082288;34242623			**HIST2H3D**	-0.52
**CBP**	Generic enzyme	**Histone H3**	Generic binding protein	Unspecified	Acetylation	CBP/P300 acetylates Histone H3.	8945521;10497301;11134336;11559745;11607014;12609999;12665567;15141169;15616580;16122695;17320507;19270680;19631660;21315607;23434580;24748101;27934968;28953875;31182547;31801086			**HIST2H3D**	-0.52

**p300**	Generic enzyme	**Histone H3**	Generic binding protein	Unspecified	Covalent modification	p300 acetylates Histone H3.	8945521;9726987;10025405;10497301;10921904;11511360;11559745;11940655;12609999;15141169;15289617;15834423;15932940;16109717;16987993;17300219;17320507;18697823;19270680;19652528;20065107;20587414;20676058;21315607;21378166;22893703;23390536;26082460;26229107;28953875;30150647;30858153;31150057;31152160;31659122;33164305;33172955;33361394;33578969			**HIST2H3D**	-0.52
**CREB1**	Transcription factor	**PHF15**	Generic binding protein	Unspecified	Transcription regulation	PHF15 promoter has a putative CREB1-binding site.	15753290;17192397			**JADE2**	-0.51
**Androgen receptor**	Transcription factor	**Gas6**	Receptor ligand	Activation	Transcription regulation	Androgen-dependent Gas6 Promoter Activity is Mediated by Binding of AR to ARE.	20048160	**AR**	-0.44198	**GAS6**	-0.51
**CREB1**	Transcription factor	**DGK-gamma**	Lipid kinase	Unspecified	Transcription regulation	Diacylglycerol kinase, gamma promoter has a putative CREB1-binding site.	15753290			**DGKG**	-0.51
**Androgen receptor**	Transcription factor	**Neurabin-1**	Generic binding protein	Unspecified	Transcription regulation	Androgen receptor binds to gene Neurabin-1 promoter.	19668381	**AR**	-0.44198	**PPP1R9A**	-0.51
**CREB1**	Transcription factor	**Neurabin-1**	Generic binding protein	Unspecified	Transcription regulation	Neurabin-1 promoter has a putative CREB1-binding site.	15753290			**PPP1R9A**	-0.51
**CREB1**	Transcription factor	**JARID2**	Transcription factor	Unspecified	Transcription regulation	JARID2 promoter has a putative CREB1-binding site.	15753290			**JARID2**	-0.50
**Androgen receptor**	Transcription factor	**LIPIN1**	Generic binding protein	Unspecified	Transcription regulation	Using the MNI algorithm, we identified LIPIN1 as AR downstream target gene in the AR pathway.	17299418	**AR**	-0.44198	**LPIN1**	-0.48
**CREB1**	Transcription factor	**IFNGR1**	Generic receptor	Unspecified	Transcription regulation	IFNGR1 promoter has a putative CREB1-binding site.	7890167;9089099;15753290;22182699			**IFNGR1**	-0.47
**Androgen receptor**	Transcription factor	**IFNGR1**	Generic receptor	Unspecified	Transcription regulation	Androgen receptor regulates transcription of IFNGR1.	33640491	**AR**	-0.44198	**IFNGR1**	-0.47
**CREB1**	Transcription factor	**ITM2C**	Generic binding protein	Unspecified	Transcription regulation	ITM2C promoter has a putative CREB1-binding site.	15753290			**ITM2C**	-0.47
**CREB1**	Transcription factor	**TCTN1**	Voltage-gated ion channel	Unspecified	Transcription regulation	TCTN1 promoter has a putative CREB1-binding site.	15753290			**TCTN1**	-0.46

**CREB1**	Transcription factor	**COL9A1**	Generic binding protein	Unspecified	Transcription regulation	CREB1 regulates transcription of COL9A1.	23762244			**COL9A1**	-0.45
**CREB1**	Transcription factor	**Presenilin 1**	Generic protease	Unspecified	Transcription regulation	Presenilin 1 promoter has a putative CREB1-binding site.	11116137;12444985;15753290;29269871			**PSEN1**	-0.44
**CREB1**	Transcription factor	**Androgen receptor**	Transcription factor	Activation	Transcription regulation	Androgen receptor is a CREB1 target gene	21403841			**AR**	-0.44
**p300**	Generic enzyme	**Androgen receptor**	Transcription factor	Activation	Covalent modification	p300 acetylated the AR at a highly conserved lysine-rich motif carboxyl-terminal to the zinc finger DNA-binding domain. AR activity is induced by p300.	10529898;10779504;11971970;14612401;16289629;16434977;16598769;16713569;17163421;18612376;21572438;22518120;23172223;23518348;24480624;28473532;28549433;30317630;32668201;34158377			**AR**	-0.44
**HDAC2**	Generic enzyme	**Androgen receptor**	Transcription factor	Unspecified	Transcription regulation	HDAC2 co-regulates transcription of Androgen receptor.	22531786;24292680;30124873;31843555			**AR**	-0.44
**Androgen receptor**	Transcription factor	**Androgen receptor**	Transcription factor	Unspecified	Transcription regulation	Androgen receptor regulates transcription of Androgen receptor.	15634333;21701558;22014572;24722067;24895212;25296973;25552417;26336819;28925401;30217192;31135075;32278794;32745318;33277366;34127806;34256096	**AR**	-0.44198	**AR**	-0.44
**Androgen receptor**	Transcription factor	**SELENBP1**	Generic binding protein	Unspecified	Transcription regulation	Androgen receptor regulates transcription of SELENBP1.	1540595;17721441;33640491	**AR**	-0.44198	**SELENBP1**	-0.44
**SMAD3**	Transcription factor	**OSF-2**	Receptor ligand	Unspecified	Transcription regulation	SMAD3 regulates transcription of OSF-2 in response to TGF-beta stimulation.	17540359;18277385;27872089;32451392			**POSTN**	-0.44
**CREB1**	Transcription factor	**OSF-2**	Receptor ligand	Activation	Transcription regulation	CREB1 activates transcription of OSF-2.	18277385;21367774;30946695			**POSTN**	-0.44
**Androgen receptor**	Transcription factor	**OSF-2**	Receptor ligand	Inhibition	Transcription regulation	Androgen receptor inhibits transcription of OSF-2.	17299418;30395538	**AR**	-0.44198	**POSTN**	-0.44
**CREB1**	Transcription factor	**A-Myb**	Transcription factor	Unspecified	Transcription regulation	CREB1 regulates transcription of A-Myb.	23762244;33846571			**MYBL1**	-0.43
**ATF/CREB**	Transcription factor	**MYH11**	Generic binding protein	Activation	Transcription regulation	ATF/CREB activates transcription of MYH11.	21680899	**BATF**	-0.686649	**MYH11**	-0.42
**Androgen receptor**	Transcription factor	**MYH11**	Generic binding protein	Unspecified	Transcription regulation	Using the MNI algorithm, we identified MYH11 as AR downstream target gene in the AR pathway.	17299418	**AR**	-0.44198	**MYH11**	-0.42

**Androgen receptor**	Transcription factor	**BAIAP2**	Generic binding protein	Unspecified	Transcription regulation	Androgen receptor binds to gene BAIAP2 promoter.	19668381	**AR**	-0.44198	**BAIAP2**	-0.38
**CREB1**	Transcription factor	**BAIAP2**	Generic binding protein	Unspecified	Transcription regulation	BAIAP2 promoter has a putative CREB1-binding site.	15753290;24760838			**BAIAP2**	-0.38
**Androgen receptor**	Transcription factor	**NPNT**	Generic binding protein	Unspecified	Transcription regulation	Androgen receptor regulates transcription of NPNT.	33640491	**AR**	-0.44198	**NPNT**	-0.37
**CREB1**	Transcription factor	**Chordin**	Generic binding protein	Unspecified	Transcription regulation	Chordin promoter has a putative CREB1-binding site.	15753290			**CHRD**	-0.37
**Androgen receptor**	Transcription factor	**Cingulin**	Generic binding protein	Unspecified	Transcription regulation	Androgen receptor regulates transcription of Cingulin.	33640491	**AR**	-0.44198	**CGN**	-0.36
**CREB1**	Transcription factor	**Cingulin**	Generic binding protein	Unspecified	Transcription regulation	Cingulin promoter has a putative CREB1-binding site.	15753290			**CGN**	-0.36
**CREB1**	Transcription factor	**Tropomodulin-2**	Generic binding protein	Unspecified	Transcription regulation	Tropomodulin-2 promoter has a putative CREB1-binding site.	15753290			**TMOD2**	-0.35
**CREB1**	Transcription factor	**APOER2**	Generic receptor	Unspecified	Transcription regulation	APOER2 promoter has a putative CREB1-binding site.	15753290			**LRP8**	-0.35
**Androgen receptor**	Transcription factor	**PI3K cat class IA (p110-delta)**	Lipid kinase	Unspecified	Transcription regulation	Androgen receptor regulates transcription of PI3K cat class IA (p110-delta).	21909140	**AR**	-0.44198	**PIK3CD**	-0.34
**Androgen receptor**	Transcription factor	**CD166**	Generic binding protein	Unspecified	Transcription regulation	Using the MNI algorithm, we identified CD166 as AR downstream target gene in the AR pathway.	17299418;21701558;33640491	**AR**	-0.44198	**ALCAM**	-0.34
**CREB1**	Transcription factor	**KIF5C**	Generic binding protein	Unspecified	Transcription regulation	KIF5C promoter has a putative CREB1-binding site.	15753290			**KIF5C**	-0.30
**CREB1**	Transcription factor	**Cathepsin B**	Generic protease	Unspecified	Transcription regulation	CREB1 probably regulates transcription of Cathepsin B in human B-cell lymphoma.	10190277;17724614;18277385			**CTSB**	-0.30
**Androgen receptor**	Transcription factor	**AATK**	Protein kinase	Unspecified	Transcription regulation	Androgen receptor regulates transcription of AATK.	25781993	**AR**	-0.44198	**AATK**	-0.30
**CREB1**	Transcription factor	**Calcipressin 1**	Generic binding protein	Activation	Transcription regulation	Calcipressin 1 promoter has a putative CREB1-binding site.	15753290;21216952;26157140			**RCAN1**	-0.28
**Androgen receptor**	Transcription factor	**Tiam1**	Regulators (GDI, GAP, GEF)	Unspecified	Transcription regulation	Androgen receptor binds to gene Tiam1 promoter.	19668381	**AR**	-0.44198	**TIAM1**	-0.28
**SMAD3**	Transcription factor	**Axin2**	Generic binding protein	Activation	Transcription regulation	SMAD3 activates transcription of Axin2.	20097766;29269485;30042129			**AXIN2**	-0.25
**CREB1**	Transcription factor	**Sin3A**	Generic binding protein	Unspecified	Transcription regulation	Sin3A promoter has a putative CREB1-binding site.	15753290			**SIN3A**	-0.24
**CREB1**	Transcription factor	**Cosmc**	Generic enzyme	Unspecified	Transcription regulation	Cosmc promoter has a putative CREB1-binding site.	15753290			**C1GALT1C1**	-0.23
**Histone H4**	Generic binding protein	**BMP7**	Receptor ligand	Unspecified	Transcription regulation	Histone H4 co-regulates transcription of BMP7.	17135397	**HIST1H4I**	-0.915472	**BMP7**	-0.23
**HDAC2**	Generic enzyme	**BMP7**	Receptor ligand	Inhibition	Transcription regulation	HDAC2 co-inhibits transcription of BMP7.	29072686			**BMP7**	-0.23
**p300**	Generic enzyme	**BMP7**	Receptor ligand	Activation	Transcription regulation	p300 occupies the gene BMP7 enhancer region.	22981823			**BMP7**	-0.23
**CREB1**	Transcription factor	**FOXN2**	Transcription factor	Unspecified	Transcription regulation	FOXN2 promoter has a putative CREB1-binding site.	15753290			**FOXN2**	-0.22
**CREB1**	Transcription factor	**CaMK II delta**	Protein kinase	Unspecified	Transcription regulation	CaMK II delta promoter has a putative CREB1-binding site.	15753290			**CAMK1**	-0.21

**ATF-2**	Transcription factor	**CaMK II delta**	Protein kinase	Unspecified	Transcription regulation	ATF-2 regulates transcription of CaMK II delta.	29360953			**CAMK1**	-0.21
**CREB1**	Transcription factor	**OSBP2**	Protein	Unspecified	Transcription regulation	OSBP2 was identificated as CREB1 target gene by chromatin immunoprecipitation and RT-PCR.	15082775			**OSBP2**	-0.21
**CREB1**	Transcription factor	**HEY1**	Transcription factor	Unspecified	Transcription regulation	HEY1 promoter has a putative CREB1-binding site.	15753290			**HEY1**	-0.20
**SMAD3**	Transcription factor	**HEY1**	Transcription factor	Activation	Transcription regulation	TGF-beta induces rapid in vivo binding of endogenous Smad3/Smad4 protein complexes with SCR elements in the HEY1 promoter and increase its mRNA.	14976548;15231748;16189514;19473993			**HEY1**	-0.20
**CREB1**	Transcription factor	**E2F4**	Transcription factor	Activation	Transcription regulation	CREB1 activates transcription of E2F4.	15753290;27211267			**E2F4**	-0.20
**CREB1**	Transcription factor	**BTG1**	Generic binding protein	Activation	Transcription regulation	CREB1 binds to gene BTG1 promoter and activates BTG1 expression.	15753290;17353275;27188441			**BTG1**	-0.18
**Androgen receptor**	Transcription factor	**LIMK2**	Protein kinase	Unspecified	Transcription regulation	Androgen receptor regulates transcription of LIMK2.	17721441;33640491	**AR**	-0.44198	**LIMK2**	-0.17
**Androgen receptor**	Transcription factor	**PTK9L**	Generic binding protein	Unspecified	Transcription regulation	Androgen receptor binds to gene PTK9L promoter.	19668381	**AR**	-0.44198	**TWF2**	-0.17
**CREB1**	Transcription factor	**NUP133**	Transporter	Unspecified	Transcription regulation	NUP133 promoter has a putative CREB1-binding site.	15753290;20920259			**NUP133**	-0.16
**CREB1**	Transcription factor	**HOXD11**	Transcription factor	Unspecified	Transcription regulation	HOXD11 promoter has a putative CREB1-binding site.	15753290			**HOXD11**	-2.78
**COUP-TFII**	Transcription factor	**HOXD11**	Transcription factor	Unspecified	Transcription regulation	ARP-1/COUP-TFII binds to Hoxd11 region IX.	8824591	**COUP-TFII**		**HOXD11**	-2.78
**SMAD3**	Transcription factor	**HOXD11**	Transcription factor	Activation	Transcription regulation	SMAD3 activates transcription of HOXD11.	24016758			**HOXD11**	-2.78
**Androgen receptor**	Transcription factor	**HOXD11**	Transcription factor	Unspecified	Transcription regulation	Androgen receptor binds to gene HOXD11 promoter.	19668381	**AR**	-0.44198	**HOXD11**	-2.78
**CREB1**	Transcription factor	**HOXA11**	Transcription factor	Unspecified	Transcription regulation	HOXA11 promoter has a putative CREB1-binding site.	15753290			**HOXA11;HOXA10**	-2.60
**Androgen receptor**	Transcription factor	**HOXA11**	Transcription factor	Unspecified	Transcription regulation	Androgen receptor binds to gene HOXA11 promoter.	19668381	**AR**	-0.44198	**HOXA11;HOXA10**	-2.60
**CREB1**	Transcription factor	**HOXA2**	Transcription factor	Unspecified	Transcription regulation	HOXA2 promoter has a putative CREB1-binding site.	15753290			**HOXA11**	-2.60
**CREB1**	Transcription factor	**GSC**	Transcription factor	Unspecified	Transcription regulation	GSC promoter has a putative CREB1-binding site.	15753290			**GSC**	-2.21
**SMAD3**	Transcription factor	**GSC**	Transcription factor	Activation	Transcription regulation	SMAD3 activates transcription of GSC.	9702197;9865691;10660041;10712925;10775259;15761153;21245162;21741376;22196728;25805847;31582430;31915377			**GSC**	-2.21
**CREB1**	Transcription factor	**HOXA10**	Transcription factor	Inhibition	Transcription regulation	CREB1 inhibits transcription of HOXA10.	15753290;26840046			**HOXA10**	-1.93

**p300**	Generic enzyme	**CD44**	Generic receptor	Activation	Transcription regulation	p300 binds to CD44 promoter after LPS treatment.	20206554			**CD44**	-1.49
**Androgen receptor**	Transcription factor	**CD44**	Generic receptor	Unspecified	Transcription regulation	Androgen receptor regulates transcription of CD44.	17721441;20200161;21865353;24981513;31719098;32587378;33323967;33687952	**AR**	-0.44198	**CD44**	-1.49
**CREB1**	Transcription factor	**HDAC2**	Generic enzyme	Activation	Transcription regulation	CREB1 activates transcription of HDAC2.	33725236;33846571				
**p300**	Generic enzyme	**CBP**	Generic enzyme	Inhibition	Transcription regulation	The adenoviral protein E1A disrupts interaction of CBP and SRCAP.	9267036;10347196;19698979;24607903;30220457;33602823				
**HDAC2**	Generic enzyme	**BDNF**	Receptor ligand	Inhibition	Transcription regulation	HDAC2 coregulates and suppresses BDNF promoter	19424149;22388814;25209292;25242807;27534825;27626660;30504275;31641124				
**CREB1**	Transcription factor	**XIAP**	Generic binding protein	Activation	Transcription regulation	XIAP promoter has a putative CREB1-binding site.	15753290;22740515				
**CREB1**	Transcription factor	**H-Ras**	RAS - superfamily	Unspecified	Transcription regulation	H-Ras promoter has a putative CREB1-binding site.	15753290				
**CBP**	Generic enzyme	**SMAD3**	Transcription factor	Activation	Covalent modification	CBP covalently modifies SMAD3 and activates it.	9679060;10497242;15467747;15750622;15849193;15990875;16862174;16959941;17074756;17283070;18003620;19589780;20110770;25416030;26029993;29489750				
**p300**	Generic enzyme	**NGFR(TNFRSF16)**	Generic receptor	Unspecified	Transcription regulation	p300 co-regulates transcription of NGFR(TNFRSF16).	29431744				
**BATF**	Transcription factor	**TGF-beta receptor type III (betaglycan)**	Receptor with enzyme activity	Unspecified	Transcription regulation	BATF regulates transcription of TGF-beta receptor type III (betaglycan).	24584090	**BATF**	-0.686649		
**Androgen receptor**	Transcription factor	**TAB1**	Generic binding protein	Unspecified	Transcription regulation	TAB1 was identificated as Androgen receptor novel target gene by chromatin immunoprecipitation analysis and RT-PCR.	17553165	**AR**	-0.44198		
**HDAC2**	Generic enzyme	**Histone H3.1**	Generic binding protein	Unspecified	Deacetylation	HDAC2 deacetylates Histone H3.1.	29317660				

**HDAC2**	Generic enzyme	**EGFR**	Receptor with enzyme activity	Inhibition	Transcription regulation	ZIP and the NuRD components (RbAp46/48, HDAC1, HDAC2, Mi-2, MTA2) occupied the promoter of EGFR gene spanning the putative ZIP-binding element.	19644445					
**Androgen receptor**	Transcription factor	**Shc**	Generic binding protein	Activation	Transcription regulation	Androgen receptor activates transcription of Shc .	17699749;17721441	**AR**		-0.44198		
**HDAC2**	Generic enzyme	**CREB1**	Transcription factor	Unspecified	Transcription regulation	HDAC2 co-regulates transcription of CREB1.	12567184;19424149;20473547;24223142;28402861					
**Androgen receptor**	Transcription factor	**CREB1**	Transcription factor	Activation	Transcription regulation	Androgen receptor activates transcription of CREB1.	27967242;33640491	**AR**		-0.44198		
**p300**	Generic enzyme	**EGFR**	Receptor with enzyme activity	Activation	Transcription regulation	p300 co-activates transcription of EGFR.	15601870;24722339;30914776;32424275;32759223					
**Androgen receptor**	Transcription factor	**TGF-beta receptor type III (betaglycan)**	Receptor with enzyme activity	Inhibition	Transcription regulation	Androgen receptor inhibits TGFBR3 gene transcription.	10706107;21602788	**AR**		-0.44198		
**CREB1**	Transcription factor	**Histone H3.1**	Generic binding protein	Unspecified	Transcription regulation	CREB1 regulates transcription of Histone H3.1.	15753290					
**CREB1**	Transcription factor	**MEK3(MAP2K3)**	Protein kinase	Unspecified	Transcription regulation	MEK3(MAP2K3) promoter has a putative CREB1-binding site.	15753290					
**CREB1**	Transcription factor	**MEK1(MAP2K1)**	Protein kinase	Unspecified	Transcription regulation	MEK1(MAP2K1) promoter has a putative CREB1-binding site.	15753290					
**CREB1**	Transcription factor	**MEK2(MAP2K2)**	Protein kinase	Unspecified	Transcription regulation	MEK2(MAP2K2) promoter has a putative CREB1-binding site.	15753290					
**HDAC2**	Generic enzyme	**SMAD3**	Transcription factor	Unspecified	Transcription regulation	HDAC2 co-regulates transcription of SMAD3.	26354229					
**CREB1**	Transcription factor	**ActRIIB**	Receptor with enzyme activity	Unspecified	Transcription regulation	CREB1 regulates transcription of ActRIIB .	15753290;17936724					
**CREB1**	Transcription factor	**BMP receptor 2**	Receptor with enzyme activity	Unspecified	Transcription regulation	BMP receptor2 promoter has a putative CREB1-binding site.	15753290					
**Histone H3**	Generic binding protein	**BDNF**	Receptor ligand	Unspecified	Transcription regulation	Histone H3 co-regulates transcription of BDNF.	19038219					
**SMAD3**	Transcription factor	**TGF-beta receptor type III (betaglycan)**	Receptor with enzyme activity	Inhibition	Transcription regulation	SMAD3 is proposed to downregulate transcription of Betaglycan.	12399463					
**CBP**	Generic enzyme	**XIAP**	Generic binding protein	Activation	Transcription regulation	CBP co-activates transcription of XIAP.	24566868					
**p300**	Generic enzyme	**BDNF**	Receptor ligand	Activation	Transcription regulation	p300 co-activates transcription of BDNF.	24603592;27739595;32444594					
**CREB1**	Transcription factor	**Histone H3.3**	Generic binding protein	Unspecified	Transcription regulation	CREB1 regulates transcription of Histone H3.3.	15753290					

**CREB1**	Transcription factor	**BDNF**	Receptor ligand	Activation	Transcription regulation	CREB1 binds to gene BDNF promoter and promotes BDNF expression	9149093;12114522;14593184;15342915;15753290;16337694;16337876;17360587;17456785;18234890;18584881;19038219;19476549;19489105;19712055;20226774;20810894;21606928;22267118;22607375;22848609;23035088;23055509;23726845;23770418;23946409;24358339;24367698;25041363;25209292;25643298;26842955;27611779;30591585;31359606;31409756;31974426;33256199				
**Androgen receptor**	Transcription factor	**MEK1(MAP2K1)**	Protein kinase	Unspecified	Transcription regulation	Androgen receptor binds to gene MEK1(MAP2K1)	19668381;32934023	**AR**	-0.44198		
**SMAD3**	Transcription factor	**SMAD3**	Transcription factor	Unspecified	Transcription regulation	SMAD3 regulates transcription of SMAD3.	15761153;28747678;31744885;33462242;34580281				
**p300**	Generic enzyme	**MEK6(MAP2K6)**	Protein kinase	Unspecified	Transcription regulation	p300 co-regulates transcription of MEK6(MAP2K6).	29313809				
**CREB1**	Transcription factor	**Inhibin alpha subunit**	Receptor ligand	Activation	Transcription regulation	Creb1 binds and activates the promoter of inhibin-alpha gene.	15650079;22734036				
**p300**	Generic enzyme	**ATF-2**	Transcription factor	Activation	Acetylation	ATF-2 b-ZIP could serve as an acetyltransferase substrate for p300 HAT. Using mass spectrometry, two p300 HAT lysine acetylation sites were mapped in ATF-2 b-ZIP. Immunoprecipitation-western blot analysis with anti-acetyl-lysine antibody revealed that ATF-2 can undergo reversible acetylation in vivo. Mutational analysis of the two ATF-2 b-ZIP acetylation sites revealed their potential contributions to ATF-2-mediated transcriptional activation.	10327051;17590016;23153039;23800081;24069158				

**CBP**	Generic enzyme	**BDNF**	Receptor ligand	Activation	Transcription regulation	CBP coregulates and activates BDNF promoter	[illegible]6549;19712055;20811339;22043863;24603592;27010597;27626660;30850733;31641124;32444594				
**HDAC2**	Generic enzyme	**TrkB**	Receptor with enzyme activity	Unspecified	Transcription regulation	HDAC2 co-regulates transcription of TrkB.	29567811				
**p300**	Generic enzyme	**TGF-beta receptor type III (betaglycan)**	Receptor with enzyme activity	Unspecified	Transcription regulation	p300 co-regulates transcription of TGF-beta receptor type III (betaglycan).	30367089				
**p300**	Generic enzyme	**HDAC2**	Generic enzyme	Activation	Transcription regulation	p300 probably binds to HDAC2 in human B-cell lymphoma.	18277385;24948597;30737378				
**Androgen receptor**	Transcription factor	**Histone H3.3**	Generic binding protein	Unspecified	Transcription regulation	Androgen receptor regulates transcription of Histone H3.3.	33640491	**AR**	-0.44198		
**CBP**	Generic enzyme	**EGFR**	Receptor with enzyme activity	Activation	Transcription regulation	CBP binds EGFR promoter and coactivates transcription.	21080969;21464950;26820293				
**CBP**	Generic enzyme	**HDAC2**	Generic enzyme	Activation	Acetylation	CBP acetylates HDAC2 activating it.	10958685;20388487				
**CREB1**	Transcription factor	**ActRIIA**	Receptor with enzyme activity	Unspecified	Transcription regulation	ActRIIA promoter has a putative CREB1-binding site.	15753290				
**CREB1**	Transcription factor	**GRB2**	Generic binding protein	Unspecified	Transcription regulation	GRB2 promoter has a putative CREB1-binding site.	15753290				
**CREB1**	Transcription factor	**TAK1(MAP3K7)**	Protein kinase	Unspecified	Transcription regulation	TAK1(MAP3K7) promoter has a putative CREB1-binding site.	15753290				
**ATF/CREB**	Transcription factor	**BDNF**	Receptor ligand	Activation	Transcription regulation	ATF/CREB activates transcription of BDNF.	20814019;25392083;27010597;31915257	**BATF**	-0.686649		
**CREB1**	Transcription factor	**TrkB**	Receptor with enzyme activity	Activation	Transcription regulation	CREB1 can bind to gene TrkB promoter and activates TrkB expression.	10395916;15234351;15753290;24760838				
**Androgen receptor**	Transcription factor	**TrkB**	Receptor with enzyme activity	Unspecified	Transcription regulation	Androgen receptor regulates transcription of TrkB.	29567811	**AR**	-0.44198		
**p300**	Generic enzyme	**p300**	Generic enzyme	Unspecified	Transcription regulation	p300 co-regulates transcription of p300.	20081228;30349051				
**CREB1**	Transcription factor	**CREB1**	Transcription factor	Activation	Transcription regulation	CREB1 can bind to gene CREB1 promoter and activates CREB1 expression.	8381074;17937658;25541153;32298238				
**ATF-2**	Transcription factor	**ATF-2**	Transcription factor	Unspecified	Transcription regulation	ATF-2 can bind to gene ATF-2 promoter.	2145272				

**p300**	Generic enzyme	**SMAD3**	Transcription factor	Activation	Covalent modification	p300 interacts with Smad3 and activate it by acetylation.	9679056;9722503;9813111;9843571;9865691;10497242;10575014;10712925;10775259;11058129;11134049;11359933;12023901;14534577;15133024;15345715;15352157;15470497;15623506;15688032;15824515;15907489;16109717;16319104;16862174;16876108;17074756;17340614;17469184;18055455;18077182;18486259;19003557;19266485;19395477;20016942;20110770;20564330;20651248;21567395;23235480;24324267;25375657;25526531;25707573;26138247;26966274;28803990;29331689;29489750;29520103;30060				
**Androgen receptor**	Transcription factor	**ATF-2**	Transcription factor	Unspecified	Transcription regulation	Androgen receptor regulates transcription of ATF-2.	9920921;11518798;26052614	**AR**	-0.44198		
