# Supplementary material for: Phenotypic divergence between broiler and layer chicken lines is regulated at the molecular level during development
Source: BMC Genomics. 2024 Feb 12;25:168. doi: 10.1186/s12864-024-10083-x (PMC10863267; doi:10.1186/s12864-024-10083-x)
Supplement: Supplementary file 6 — Supplementary Material 6 [file 12864_2024_10083_MOESM6_ESM.pdf]

#clustering method	cluster number	cluster color	gene count	protein name	protein identifier	protein description
MCL	1	Red	31	ACTN2	9031.ENSGALP00000023309	Actinin alpha 2/3; Alpha-actinin-2; F-actin cross-linking protein which is thought to anchor actin to a variety of intracellular structures. This is a bundling protein (By similarity)
MCL	1	Red	31	AR	9031.ENSGALP00000007301	Gallus gallus androgen receptor (AR), mRNA
MCL	1	Red	31	ATF2	9031.ENSGALP00000032623	Cyclic AMP-dependent transcription factor ATF-2; Transcriptional activator which regulates the transcription of various genes, including those involved in anti- apoptosis, cell growth, and DNA damage response. Dependent on its binding partner, binds to CRE (cAMP response element) consensus sequences (5'-TGACGTCA-3') or to AP-1 (activator protein 1) consensus sequences (5'-TGACTCA-3') (By similarity); Belongs to the bZIP family. ATF subfamily
MCL	1	Red	31	BATF	9031.ENSGALP00000016785	Uncharacterized protein; Basic leucine zipper transcription factor, ATF-like
MCL	1	Red	31	BMP7	9031.ENSGALP00000012416	Bone morphogenetic protein 7
MCL	1	Red	31	CAMK1	9031.ENSGALP00000010784	Calcium/calmodulin-dependent protein kinase type 1 isoform x1; Calcium/calmodulin-dependent protein kinase I; Belongs to the protein kinase superfamily
MCL	1	Red	31	CHRD	9031.ENSGALP00000041768	Chordin; Gallus gallus chordin (CHRD), mRNA; Belongs to the chordin family
MCL	1	Red	31	CREB1	9031.ENSGALP00000013824	Cyclic amp-responsive element-binding protein 1; Gallus gallus cAMP responsive element binding protein 1 (CREB1), mRNA
MCL	1	Red	31	DLX3	9031.ENSGALP00000043061	Homeobox protein dlx-3; Gallus gallus distal-less homeobox 3 (DLX3), mRNA
MCL	1	Red	31	E2F4	9031.ENSGALP00000002638	Transcription factor e2f4/5; Gallus gallus E2F transcription factor 4, p107/p130-binding (E2F4), mRNA
MCL	1	Red	31	EP300	9031.ENSGALP00000019537	Histone acetyltransferase p300; E1A binding protein p300
MCL	1	Red	31	FOXN2	9031.ENSGALP00000014522	Forkhead box protein n2; Gallus gallus forkhead box N2 (FOXN2), mRNA
MCL	1	Red	31	FSHR	9031.ENSGALP00000014797	Follicle-stimulating hormone receptor precursor; G protein-coupled receptor for follitropin, the follicle- stimulating hormone. Through cAMP production activates the downstream PI3K-AKT and ERK1/ERK2 signaling pathways
MCL	1	Red	31	GSC	9031.ENSGALP00000017857	Homeobox protein goosecoid; Involved in the development of the organizer region in the gastrula (Hensen node in chicken)
MCL	1	Red	31	HDAC2	9031.ENSGALP00000024133	Histone deacetylase 1/2; Responsible for the deacetylation of lysine residues on the N-terminal part of the core histones (H2A, H2B, H3 and H4). Histone deacetylation gives a tag for epigenetic repression and plays an important role in transcriptional regulation, cell cycle progression and developmental events. Histone deacetylases act via the formation of large multiprotein complexes (By similarity)
MCL	1	Red	31	HEY1	9031.ENSGALP00000041743	Hairy/enhancer-of-split related with yrpw motif protein 1 isoform x2; Hes-related family bHLH transcription factor with YRPW motif 1
MCL	1	Red	31	HIST1H111[illegible]	9031.ENSGALP00000019205	Histone h1.11r; Histones H1 are necessary for the condensation of nucleosome chains into higher-order structures
MCL	1	Red	31	HIST1H2A3	9031.ENSGALP00000040820	Gallus gallus histone cluster 1, H2A, IV (similar to human histone cluster 2, class H2A, member C) (HIST1H2A4), mRNA
MCL	1	Red	31	IFNGR1	9031.ENSGALP00000031140	Uncharacterized protein; Gallus gallus interferon gamma receptor 1 (IFNGR1), mRNA
MCL	1	Red	31	JARID2	9031.ENSGALP00000020711	Protein jumonji; Regulator of histone methyltransferase complexes that plays an essential role in embryonic development. Acts by modulating histone methyltransferase activity and promoting the recruitment of histone methyltransferase complexes to their target genes. Binds DNA and mediates the recruitment of the PRC2 complex to target genes in embryonic stem cells. Does not have histone demethylase activity but regulates activity of various histone methyltransferase complexes. In embryonic stem cells, it associates with the PRC2 complex and inhibits trimethylation of 'Lys-27' of histone [...]
MCL	1	Red	31	MSX2	9031.ENSGALP00000040969	Homeobox protein MSX-2; Acts as a transcriptional regulator in bone development. Binds to DNA (By similarity). Morphogenetic role
MCL	1	Red	31	MYBL1	9031.ENSGALP00000024981	Myb-related protein a; Strong transcriptional activator; DNA-binding protein that specifically recognize the sequence 5'-YAAC[GT]G-3'. Could have a role in the proliferation and/or differentiation of neurogenic, spermatogenic and B-lymphoid cells
MCL	1	Red	31	MYH11	9031.ENSGALP00000010520	Myosin heavy chain 9/10/11/14; Muscle contraction
MCL	1	Red	31	MYOCD	9031.ENSGALP00000040936	Myocardin; Gallus gallus myocardin (MYOCD), mRNA
MCL	1	Red	31	NPNT	9031.ENSGALP00000017173	Nephronectin isoform x5; Nephronectin
MCL	1	Red	31	NR2F2	9031.ENSGALP00000011318	COUP transcription factor 2; Ligand-activated transcription factor. Activated by high concentrations of 9-cis-retinoic acid and all-trans-retinoic acid, but not by dexamethasone, cortisol or progesterone (in vitro) (By similarity). May be involved in motor neuron development; Belongs to the nuclear hormone receptor family. NR2 subfamily

MCL	1	Red	31	PSEN1	9031.ENSGALP00000031992	Presenilin-1; Catalytic subunit of the gamma-secretase complex, an endoprotease complex that catalyzes the intramembrane cleavage of integral membrane proteins such as Notch receptors and APP (amyloid-beta precursor protein). Requires the presence of the other members of the gamma-secretase complex for protease activity. Plays a role in Notch and Wnt signaling cascades and regulation of downstream processes via its role in processing key regulatory proteins
MCL	1	Red	31	RXRA	9031.ENSGALP00000004130	Retinoic acid receptor rxr-alpha isoform x1; Uncharacterized protein; Retinoid X receptor, alpha
MCL	1	Red	31	SIN3A	9031.ENSGALP00000002508	SIN3 transcription regulator family member A
MCL	1	Red	31	SMAD3	9031.ENSGALP00000012765	Mothers against decapentaplegic homolog 3; Transcriptional modulator activated by TGF-beta (transforming growth factor) and activin type 1 receptor kinase. SMAD3 is a receptor-regulated SMAD (R-SMAD) (By similarity)
MCL	1	Red	31	SMAD6	9031.ENSGALP00000041470	Mothers against decapentaplegic homolog 6; Acts as a mediator of TGF-beta and BMP antiflammatory activity. Suppresses IL1R-TLR signaling, preventing NF-kappa-B activation, and BMP-SMAD1 signaling pathway. Binds to regulatory elements in target promoter regions (By similarity)
MCL	2	Brown	12	ALCAM	9031.ENSGALP00000024723	CD166 antigen; Cell adhesion molecule that mediates both heterotypic cell-cell contacts via its interaction with CD6, as well as homotypic cell-cell contacts. Promotes T-cell activation and proliferation via its interactions with CD6 (By similarity). Contributes to the formation and maturation of the immunological synapse via its interactions with CD6 (By similarity). Mediates homotypic interactions with cells that express ALCAM. Mediates attachment of dendritic cells onto endothelial cells via homotypic interaction. Inhibits endothelial cell migration and promotes endothelial tube for [...]
MCL	2	Brown	12	BAIAP2	9031.ENSGALP00000011192	BAI1-associated protein 2
MCL	2	Brown	12	CCR7	9031.ENSGALP00000042198	C-c chemokine receptor type 7 precursor; Gallus gallus chemokine (C-C motif) receptor 7 (CCR7), mRNA; Belongs to the G-protein coupled receptor 1 family
MCL	2	Brown	12	CD44	9031.ENSGALP00000012731	CD44 antigen precursor; Uncharacterized protein; Gallus gallus CD44 molecule (Indian blood group) (CD44), mRNA
MCL	2	Brown	12	COL9A1	9031.ENSGALP00000036264	Collagen alpha-1(ix) chain precursor; Structural component of hyaline cartilage and vitreous of the eye
MCL	2	Brown	12	CX3CR1	9031.ENSGALP00000019474	CX3C chemokine receptor 1; Chemokine (C-X3-C motif) receptor 1; Belongs to the G-protein coupled receptor 1 family
MCL	2	Brown	12	DSCAM	9031.ENSGALP00000025946	Down syndrome cell adhesion molecule homolog isoform x1; Down syndrome cell adhesion molecule homolog; Cell adhesion molecule that plays a role in neuronal self-avoidance. Promotes repulsion between specific neuronal processes of either the same cell or the same subtype of cells. Mediates within retinal amacrine and ganglion cell subtypes both isoneuronal self-avoidance for creating an orderly dendritic arborization and heteroneuronal self-avoidance to maintain the mosaic spacing between amacrine and ganglion cell bodies (By similarity). Receptor for netrin required for axon guidance i [...]
MCL	2	Brown	12	NTN4	9031.ENSGALP00000018590	Netrin-4 isoform X1; Netrin 4
MCL	2	Brown	12	PIK3CD	9031.ENSGALP00000004061	Phosphatidylinositol-4,5-bisphosphate 3-kinase catalytic subunit alpha/beta/delta; Gallus gallus phosphoinositide-3-kinase, catalytic, delta polypeptide (PIK3CD), mRNA; Belongs to the PI3/PI4-kinase family
MCL	2	Brown	12	PPP1R9A	9031.ENSGALP00000015748	Uncharacterized protein; Protein phosphatase 1, regulatory subunit 9A
MCL	2	Brown	12	TIAM1	9031.ENSGALP00000025495	T-lymphoma invasion and metastasis-inducing protein 1 isoform x1; T-cell lymphoma invasion and metastasis 1
MCL	2	Brown	12	TNFSF13B	9031.ENSGALP00000027177	Gallus gallus tumor necrosis factor (ligand) superfamily, member 13b (TNFSF13B), mRNA
MCL	3	Olive	8	ALKBH1	9031.ENSGALP00000017055	Alkylated dna repair protein alkb homolog 1; AlkB homolog 1, histone H2A dioxygenase; Gallus gallus alkB, alkylation repair homolog 1 (E. coli) (ALKBH1), mRNA
MCL	3	Olive	8	LIMK2	9031.ENSGALP00000011249	LIM domain kinase 2; Displays serine/threonine-specific phosphorylation of myelin basic protein and histone (MBP) in vitro; Belongs to the protein kinase superfamily. TKL Ser/Thr protein kinase family
MCL	3	Olive	8	MYO3A	9031.ENSGALP00000012361	Myosin-iiia isoform x1; Myosin IIIA; Belongs to the TRAFAC class myosin-kinesin ATPase superfamily. Myosin family
MCL	3	Olive	8	PAG1	9031.ENSGALP00000030225	Phosphoprotein associated with glycosphingolipid-enriched microdomains 1 isoform x2; Phosphoprotein membrane anchor with glycosphingolipid microdomains 1
MCL	3	Olive	8	PTPRO	9031.ENSGALP00000036664	Gallus gallus protein tyrosine phosphatase, receptor type, O (PTPRO), mRNA
MCL	3	Olive	8	PTPRQ	9031.ENSGALP00000017773	Phosphatidylinositol phosphatase ptprq isoform x1; Protein tyrosine phosphatase, receptor type, Q
MCL	3	Olive	8	SNAP91	9031.ENSGALP00000025502	Synaptosome associated protein 91; Gallus gallus synaptosomal-associated protein, 91kDa homolog (mouse) (SNAP91), mRNA
MCL	3	Olive	8	TWF2	9031.ENSGALP00000006326	Twinfilin-2; Actin-binding protein involved in motile and morphological processes. Inhibits actin polymerization, likely by sequestering G- actin (By similarity)
MCL	4	Green	4	AXIN2	9031.ENSGALP00000006394	Axin 2; Gallus gallus axin 2 (AXIN2), mRNA
MCL	4	Green	4	CD109	9031.ENSGALP00000025597	CD109 molecule

MCL	4	Green	4	LRP8	9031.ENSGALP00000017370	Low-density lipoprotein receptor-related protein 8 precursor; Cell surface receptor for Reelin (RELN) and apolipoprotein E (apoE)-containing ligands. Also binds alpha2-macroglobulin. LRP8 participates in transmitting the extracellular Reelin signal to intracellular signaling processes, by binding to DAB1 on its cytoplasmic tail. Reelin acts via both the VLDL receptor (VLDLR) and LRP8 to regulate DAB1 tyrosine phosphorylation and microtubule function in neurons. LRP8 has higher affinity for Reelin than VLDLR. LRP8 is thus a key component of the Reelin pathway which governs neuronal laye [...]
MCL	4	Green	4	PCSK9	9031.ENSGALP00000028013	Proprotein convertase subtilisin/kexin type 9
MCL	5	Lime Gree	2	SCEL	9031.ENSGALP00000041023	Uncharacterized protein; Sciellin
MCL	5	Lime Gree	2	TGM3	9031.ENSGALP00000007658	Protein-glutamine gamma-glutamyltransferase e isoform x2; Transglutaminase 3
MCL	6	Cyan	2	AATK	9031.ENSGALP00000011162	Serine/threonine-protein kinase lmtk1 isoform x3; Apoptosis-associated tyrosine kinase
MCL	6	Cyan	2	TNR	9031.ENSGALP00000042383	Tenascin-r precursor; Neural extracellular matrix (ECM) protein involved in interactions with different cells and matrix components. Involved in cell attachment and neurite formation. Interaction with CNTN1 enhances the neurite outgrowth
MCL	7	Blue	2	RCAN1	9031.ENSGALP00000025753	Gallus gallus regulator of calcineurin 1 (RCAN1), mRNA
MCL	7	Blue	2	TESC	9031.ENSGALP00000013348	Calcineurin b homologous protein 3; Functions as an integral cofactor in cell pH regulation by controlling plasma membrane-type Na(+)/H(+) exchange activity. Promotes the induction of hematopoietic stem cell differentiation toward megakaryocytic lineage. Essential for the coupling of ERK cascade activation with the expression of ETS family genes in megakaryocytic differentiation. Also involved in granulocytic differentiation in a ERK-dependent manner. Inhibits the phosphatase activity of calcineurin (By similarity)
MCL	8	Purple	2	HOXA10	9031.ENSGALP00000018009	Homeobox a10; Uncharacterized protein
MCL	8	Purple	2	HOXA9	9031.ENSGALP00000018013	Homeobox protein hox-a9; Sequence-specific transcription factor which is part of a developmental regulatory system that provides cells with specific positional identities on the anterior-posterior axis
