# Supplementary figures and images for: Phenotypic divergence between broiler and layer chicken lines is regulated at the molecular level during development
Source: BMC Genomics. 2024 Feb 12;25:168. doi: 10.1186/s12864-024-10083-x (PMC10863267; doi:10.1186/s12864-024-10083-x)

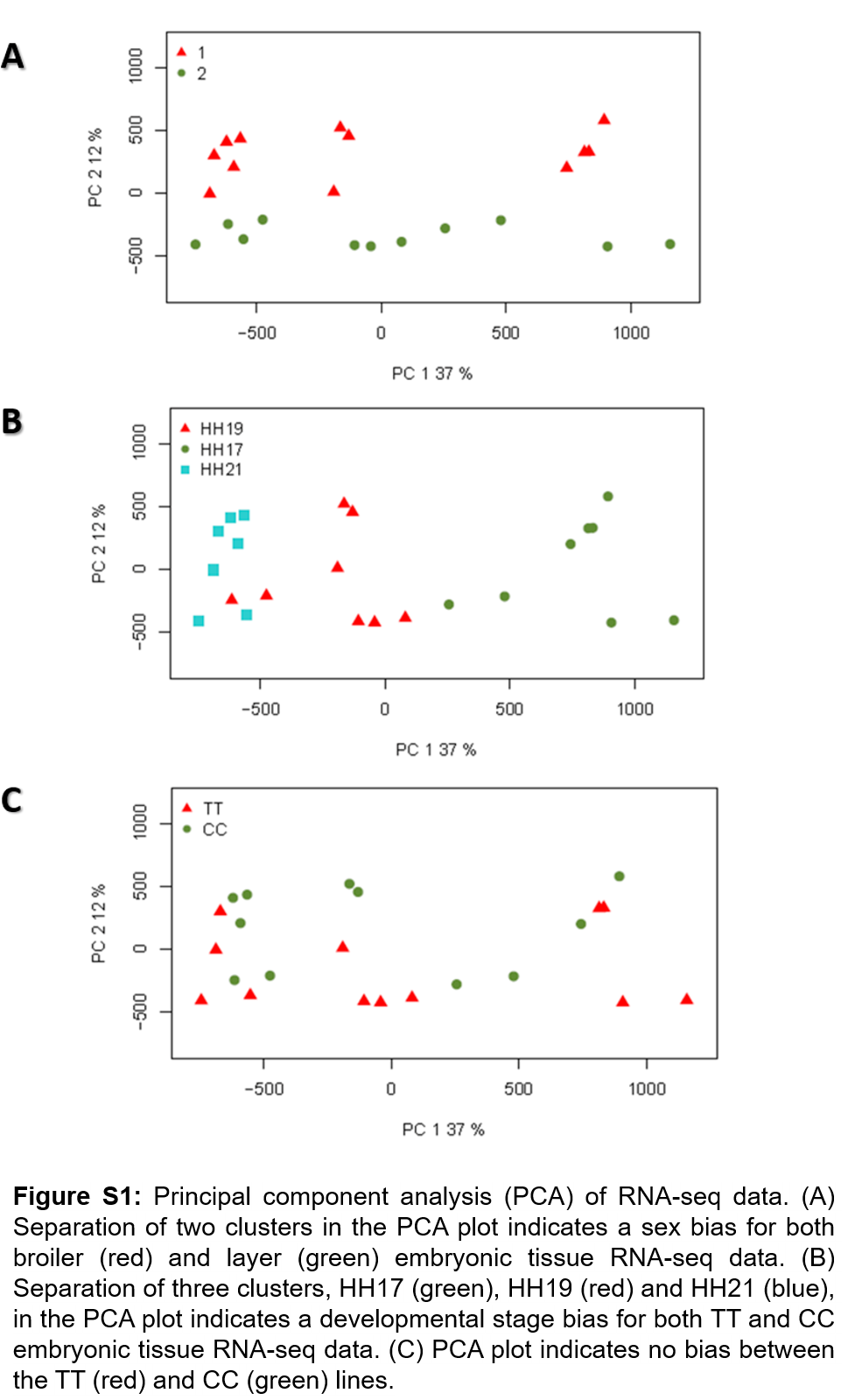

Supplement: Supplementary file 7 — Supplementary Material 7 [file 12864_2024_10083_MOESM7_ESM.png]
